# Analysis of Protein–Protein Functional Associations by Using Gene Ontology and KEGG Pathway

**DOI:** 10.1155/2019/4963289

**Published:** 2019-07-18

**Authors:** Fei Yuan, Xiaoyong Pan, Lei Chen, Yu-Hang Zhang, Tao Huang, Yu-Dong Cai

**Affiliations:** ^1^Department of Science & Technology, Binzhou Medical University Hospital, Binzhou 256603, Shandong, China; ^2^BASF & IDLab, Ghent University, Ghent, Belgium; ^3^College of Information Engineering, Shanghai Maritime University, Shanghai 201306, China; ^4^Shanghai Key Laboratory of PMMP, East China Normal University, Shanghai 200241, China; ^5^Institute of Health Sciences, Shanghai Institutes for Biological Sciences, Chinese Academy of Sciences, Shanghai 200031, China; ^6^School of Life Sciences, Shanghai University, Shanghai 200444, China

## Abstract

Protein–protein interaction (PPI) plays an extremely remarkable role in the growth, reproduction, and metabolism of all lives. A thorough investigation of PPI can uncover the mechanism of how proteins express their functions. In this study, we used gene ontology (GO) terms and biological pathways to study an extended version of PPI (protein–protein functional associations) and subsequently identify some essential GO terms and pathways that can indicate the difference between two proteins with and without functional associations. The protein–protein functional associations validated by experiments were retrieved from STRING, a well-known database on collected associations between proteins from multiple sources, and they were termed as positive samples. The negative samples were constructed by randomly pairing two proteins. Each sample was represented by several features based on GO and KEGG pathway information of two proteins. Then, the mutual information was adopted to evaluate the importance of all features and some important ones could be accessed, from which a number of essential GO terms or KEGG pathways were identified. The final analysis of some important GO terms and one KEGG pathway can partly uncover the difference between proteins with and without functional associations.

## 1. Introduction

Protein is the material foundation of all living things [1]. Protein–protein interaction (PPI) plays an extremely significant role in the growth, reproduction, and metabolism of any life, even in a single cell [2, 3]. Proteins can be easily clustered in three methods: (I) homology of protein subunits, (II) stability of interactions, and (III) combination mode of subunits [4–6]. By connecting related proteins, PPI initiates the action of various functional or structural proteins in every single cell [4]. Given that proteins influence different biological processes, even in single cells, conducting a study on PPI to further determine protein functions and life activities is a relevant endeavor.

PPI has been thoroughly studied both in experimental and computing scenarios. To study PPI via experiments, coimmunoprecipitation, Western blot, and yeast two-hybrid systems are generally adopted [7, 8]. As for computational methods, several algorithms have been developed to identify PPI, and the two main ones are the topology-free approaches and the graph-based approaches, which are based on distances between proteins and specialized clustering techniques, respectively [9, 10]. Some other computational methods predict PPIs from protein sequences using machine learning. Jansen et al. developed a Bayesian network to integrate multiple genomic features to predict PPIs [11]. Shen et al. trained a support vector machine classifier using conjoint triad features derived from sequences [12]. Pan et al. first used latent Dirichlet allocation model to extract latent topic features from the conjoint triad features, then the learned topic features were fed into a random forest classifier to predict PPIs [13]. Hashemifar et al. trained a deep learning model to predict PPIs using evolutionary information with random projection and data augmentation [14]. In addition, with the development and innovation of computational technologies, the use of updated algorithms has allowed researchers to predict and study PPIs conveniently and accurately alongside the utilization of different databases and methods.

Gene ontology (GO) is a bioinformatic concept that was originally proposed to unify the representation of genes and gene products of many species [15, 16]. The ontology covers three main domains, namely, (I) cellular component, (II) molecular function, and (III) biological process, which can easily cluster all genes and gene products with a directed acyclic graph (DAG) [16]. For convenience, the ontological domains are widely used in computational biology to avoid redundancy of different annotations of a single functional or structural gene [17, 18]. GO terms, which have been updated given the development of biological science, can summarize the specific role of genes and their products in living cells, and they are regarded as powerful tools in computational biology science [16]. Different kinds of PPIs are also included in the various terms of GO annotations. The specific locations or functions of PPIs in cells have been investigated to easily describe and distinguish the several kinds of GO terms. The GO annotations contain informative signals for PPIs. For example, Patil and Nakamura trained a machine learning classifier to infer PPIs using features derived from sequence similarity, shared GO terms and domains [19]. Ben-Hur et al. used a kernel method to integrate sequences, GO annotations, local network properties and homologous interactions for predicting PPIs [20]. Stefan et al. generated features for proteins from GO DAG; then, the extracted features were fed into a random forest classifier to predict PPIs [21]. However, these studies only adopted the GO annotations to construct the model for predicting PPIs. They did not analyze which GO annotations were highly related to the determination of PPIs. In addition, genes can be clustered into several biological pathways. Some essential pathways may be highly related to PPIs.

In this study, we investigated an extended version of PPI (protein–protein function associations) by using GO terms and KEGG pathways. Considering the fact that few PPI studies with computational methods investigated which GO terms were highly related to the determination of PPIs, the purpose of this study was to identify key GO terms or KEGG pathways that can indicate the difference between two proteins with and without functional associations. We first extracted protein–protein functional associations with experiment validations reported in Search Tool for the Retrieval of Interacting Genes/Proteins (STRING) [22, 23], a well-known database on collected associations between proteins, as the positive samples, and then we randomly selected proteins to constitute the negative samples. Considering that the random selection of negative samples may influence the results, 10 sets of negative samples were constructed, thereby constituting 10 datasets, each of which contained the same positive samples. Each protein–protein functional association was encoded into a vector by using the GO terms and KEGG pathways. Then, mutual information was adopted to evaluate the importance of all features in each dataset. From the feature lists, in which features were ranked in the decreasing order of their importance, some important features were identified, and their corresponding GO terms or KEGG pathways were obtainable. Finally, we analyzed some most important GO terms and one KEGG pathway to partly uncover the difference between proteins with and without functional associations.

## 2. Materials and Methods

### 2.1. Materials

All human protein–protein functional associations used in this study were retrieved from STRING (http://www.string-db.org/, version 9.1) [22, 23], a well-known public database on several collected associations between proteins from various organisms. These associations have been derived from the following four sources: (I) genomic context, (II) high-throughput experiments, (III) (conserved) coexpression, and (IV) previous knowledge. To obtain the human protein–protein functional associations in this database, we downloaded a file named “protein.links.detailed.v9.1.txt.gz” and then extracted lines starting with “9606” (i.e., the code of human in STRING). A total of 2,425,314 human protein–protein functional associations involving 20,770 proteins were accessed. The purpose of this study is to identify some important GO terms or KEGG pathways that can indicate the difference between two proteins with and without functional associations. Thus, we refined the 20,770 proteins as follows: (1) utilize CD-HIT [24] to discard similar proteins such that the similarity between any two remaining proteins was less than 0.25 and (2) exclude proteins whose GO term or KEGG pathway information was not available, from which we obtained 8,916 proteins. The derived proteins can comprise 588,154 human protein–protein functional associations. Furthermore, we selected 70,392 human protein–protein functional associations among the above-mentioned associations. The “Experimental” scores of these associations are larger than zero, meaning that they are validated by solid experiments. These associations involved 6,623 human proteins. For convenience, these associations were termed positive associations in this study and are provided in Supplementary Material S1.

**Figure 1:**
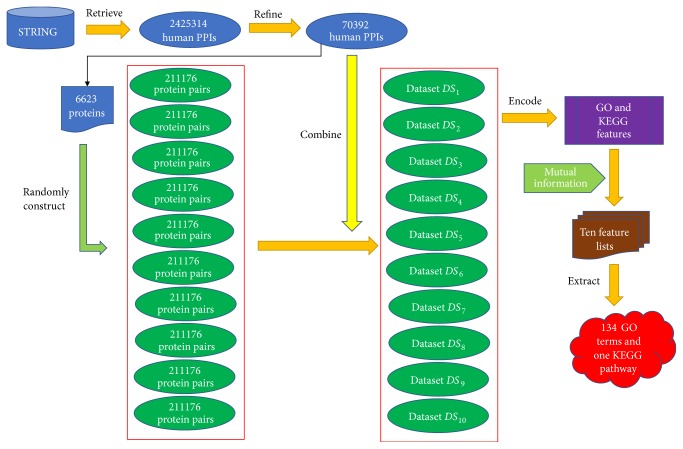
The whole procedures for analyzing protein–protein functional associations based on gene ontology (GO) and KEGG pathways. The raw 2,425,314 human PPIs were retrieved from STRING and refined by excluding similar proteins and selecting those validated by experiments, resulting in 70,392 PPIs. 6,623 proteins were involved in investigated PPIs and used to construct ten sets of protein pairs, each of which combined with 70,392 PPIs to constitute ten datasets. Each sample was represented by GO and KEGG features, which were evaluated by mutual information, producing ten feature lists, from which we extracted most important features, corresponding to 134 GO terms and one KEGG pathway.

To extract the difference between positive associations and any two proteins without functional associations, some negative associations are necessary. Given that negative associations are substantially more than the positive ones, we constructed 211,176 differing pairs of proteins, which were thrice as many as positive associations, and each of them was produced as follows: (1) random selection of two different proteins from 6,623 proteins, and (II) these two proteins cannot comprise an association reported in STRING. The obtained negative and positive associations constituted a dataset. Considering that the produced negative associations may influence the results, we randomly produced 10 sets of negative associations. Each of the sets, together with the positive associations, constituted a dataset, thus producing 10 datasets, which were denoted as *DS*_1_, *DS*_2_,…, *DS*_10_. By analyzing these datasets, some essential information for protein–protein functional associations can be discovered. The whole procedures are illustrated in Figure 1.

### 2.2. Representation of Protein–Protein Function Associations

GO terms [16] and KEGG pathways [25] are always used to elucidate and describe molecular functions, cellular components, and biological and signal processes of genes. From Gene Ontology Consortium [16], 17,916 GO terms were retrieved. Accordingly, a protein *p* can be encoded as

(1) $$\begin{array}{c} v_{GO}\left(\;p\;\right)={\left[\;g_{1}^{p},g_{2}^{p},\ldots ,g_{17916}^{p}\;\right]}^{T}, \end{array}$$

where

(2) $$\begin{array}{c} g_{i}^{p}=\left\{\begin{array}{cc} 1 & If\;\;p\;\;is\;\;annotated\;\;by\;\;the\;\;i\text{-}th\;\;GO\;\;term\\ 0 & Otherwise. \end{array}\right. \end{array}$$

For two proteins *p*_1_ and *p*_2_ that comprised either a positive association or a negative association *P* = (*p*_1_, *p*_2_), because there was no order information in *P*, i.e., (*p*_1_, *p*_2_) was identical to (*p*_2_, *p*_1_), it was not appropriate to simply combine the features of* p*_1_ and* p*_2_. To exclude the order information of *P*, we adopted the following scheme that has been used in some studies [26, 27]. For *P* = (*p*_1_, *p*_2_), it was encoded into a vector by using *v*_GO_(*p*_1_) and *v*_GO_(*p*_2_) as follows:

(3) VGOP=vGOp1⊗vGOp2=g1p1+g1p2,g1p1−g1p2,…,g17916p1+g17916p2,g17916p1−g17916p2T.

Moreover, according to KEGG [25], there were 279 pathways, based on which the protein *p* can be represented by

(4) $$\begin{array}{c} v_{pathway}\left(\;p\;\right)={\left[\;k_{1}^{p},k_{2}^{p},\ldots ,k_{279}^{p}\;\right]}^{T}, \end{array}$$

where

(5) kip=1If  p  is  annotated  by  the  i-th  KEGG  pathway0Otherwise.

Similarly, *P* = (*p*_1_, *p*_2_) can be encoded into

(6) VpathwayP=vpathwayp1⊗vpathwayp2=k1p1+k1p2,k1p1−k1p2,…,k279p1+k279p2,k279p1−k279p2T.

By integrating the GO term and KEGG pathway information of proteins into *P* = (*p*_1_, *p*_2_), each association can be finally encoded as

(7) $$\begin{array}{c} V\left(\;P\;\right)=V_{GO}\left(\;P\;\right)⊕V_{pathway}\left(\;P\;\right)=\left[\begin{array}{c} V_{GO}\left(P\right)\\ V_{pathway}\left(P\right) \end{array}\right]. \end{array}$$

A total of 36,390 features were used to represent each positive association or negative association. The information of each GO term or KEGG pathway was contained by these two features.

### 2.3. Feature Evaluation with Mutual Information

As mentioned in Section 2.2, several features were used to represent each protein–protein functional association. However, not all are highly related to sufficiently determine the differences between positive and negative associations, i.e., not all GO terms and KEGG pathways can be used to mark the associations. Here, we adopted the mutual information (MI) of each feature and target (class labels of samples) to evaluate the importance of each feature. The evaluations use the following equation to access the relationship between the two variables of *x* and *y*:

(8) $$\begin{array}{c} I\left(\;x,y\;\right)=\iint p\left(\;x,y\;\right)\log\frac{p\left(x,y\right)}{p\left(x\right)p\left(y\right)}dxdy, \end{array}$$

where *p*(*x*) and *p*(*y*) are the marginal probabilistic density of variables *x* and *y*, while *p*(*x*, *y*) is their joint probabilistic density.

Given a dataset in which each sample is represented by *N* features, after the MI values of all features were calculated, features were sorted by their MI values in decreasing order, thereby producing a feature list named MaxRel feature list, which is formulated as

(9) $$\begin{array}{c} L=\left[\;f_{1},f_{2},\ldots ,f_{N}\;\right], \end{array}$$

where *f*_*i*_ represents a feature in the dataset.

To quickly implement the program of MI, we adopted the program of minimum redundancy maximum relevance (mRMR) method [28], which integrates the MI program. This program has been applied in solving several complicated biological problems [26, 29–45].

## 3. Results

### 3.1. Results of the Feature Evaluation

As mentioned in Section 2.2, each association in the 10 datasets was represented by 36,390 features. We calculated the MI value of each feature in each of the datasets *DS*_1_, *DS*_2_,…, *DS*_10_. Subsequently, ten MaxRel feature lists could be accessed. A part of these 10 lists is provided in Supplementary Material S2.

### 3.2. Extracting Important GO Terms and KEGG Pathways

**Figure 2:**
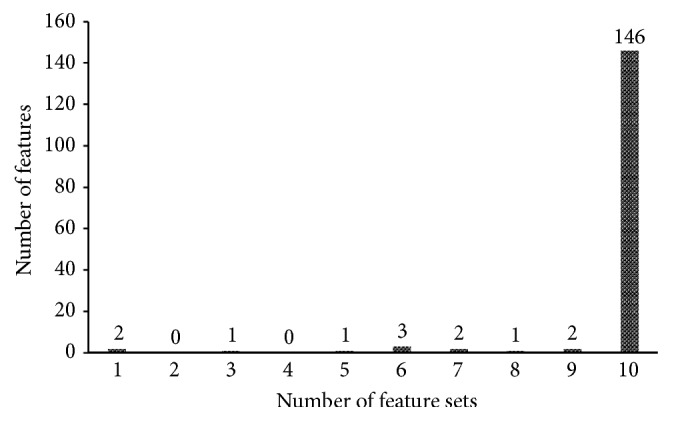
Distribution of 158 selected features: 146, 2, 1, 2, 3, and 4 feature/s in 10, 9, 8, 7, 6, and less than 6 feature sets derived from 10 datasets, respectively.

**Figure 3:**
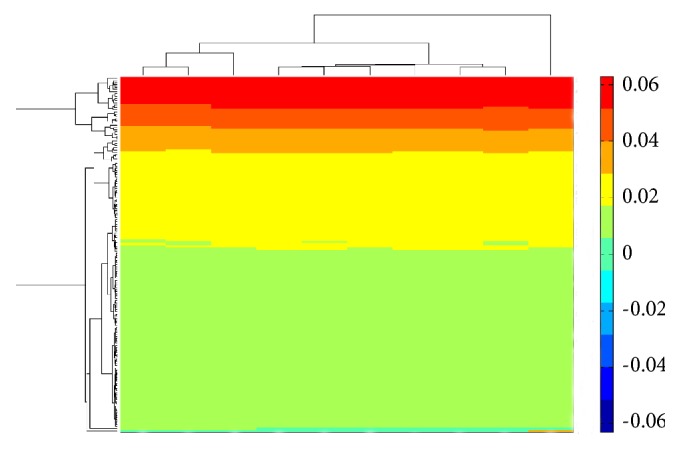
Heat map of MI values of 158 features in the 10 datasets. X-axis represents ten datasets; Y-axis represents 158 features.

Features with high ranks (large MI values) in the MaxRel feature list are more important than those with low ranks (small MI values). For the MI value, we set 0.01 as the threshold to select important features in each MaxRel feature list, thus producing 10 feature sets denoted as *F*_1_, *F*_2_,…, *F*_10_. The numbers of selected features in these sets are listed in Table 1. In the tabulation, the sizes of the 10 feature sets are nearly the same. After the features in these 10 sets were combined, 158 features were obtained (Supplementary Material S3). The obtained number (i.e., 158) did not differ much from the size of each feature set, which indicates that the majority of the 158 features were included in each set. In particular, among the 158 features, 146 features were included in all 10 feature sets, while 2, 1, 2, 3, and 4 feature/s were included in nine, eight, seven, six, and less than six feature sets (Figure 2), respectively. Considering that the negative associations in each of the 10 datasets somewhat differed, we predicted that the random selection of negative PPIs will not have a strong influence on the selection of the 158 features; i.e., the features can effectively determine the difference between positive and negative associations.  Figure 3 shows a heat map of MI values of the 158 features in the 10 datasets. In the figure, the MI values of each of the 158 features in the 10 datasets are nearly the same. Similarly, the distributions of the MI values of the 158 features in the 10 datasets are nearly the same, which validates the above-mentioned results. Subsequently, an extensive investigation to further uncover the mechanism of proteins with function associations was conducted.

**Figure 4:**
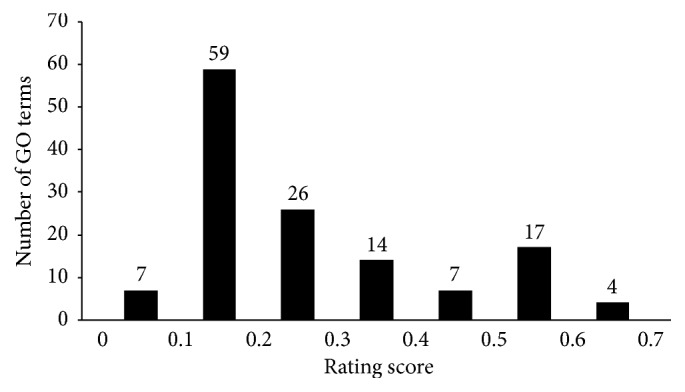
The distribution of the rating scores of 134 selected GO terms.

A careful checking showed that the important 158 features were derived from 134 GO terms and one KEGG pathway (Supplementary Material S4). To further evaluate their importance, we adopted a calculation technique called rating score measurement for each GO term. In this paper, the rating score is expressed as the sum of MI values of the related features in the 10 MaxRel feature lists. The scores are also provided in Supplementary Material S4. The rating score for the KEGG pathway (hsa03010) was 0.107, while the distribution of rating scores for 134 GO terms is illustrated in Figure 4.

### 3.3. Analysis of the Importance of Selected Features

**Figure 5:**
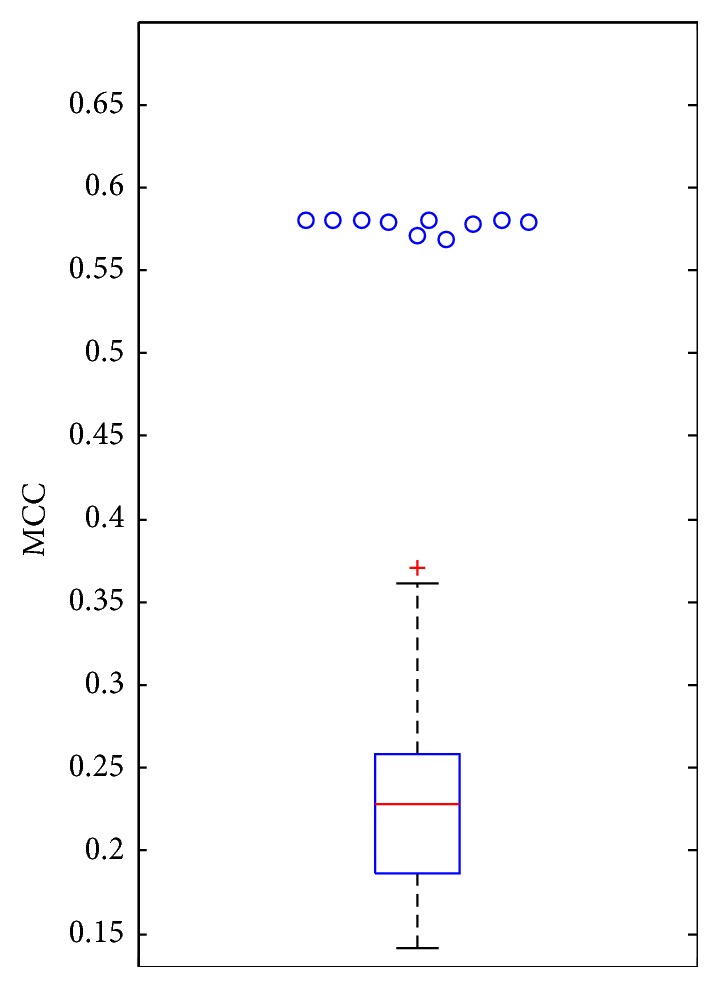
The performance of the random forest (RF) on ten datasets, in which samples were represented by selected 158 features or randomly selected 158 features, evaluated by tenfold cross-validation. The box plot indicates the distribution of MCCs yielded by RF with randomly selected 158 features and the circles represent the MCCs yielded by RF with selected 158 features on ten datasets. It is clear that based on selected 158 selected features, RF produced much better performance, implying the strong associations between these features and PPIs.

As mentioned in Section 3.2, we finally selected 158 features that were deemed to be highly related to PPIs. To confirm such conclusion, we did the following test. For each of ten datasets mentioned in Section 2.1, each sample in the dataset was represented by these 158 features. And we also randomly constructed 100 feature sets, each of which consisted of 158 features. Samples in *DS*_1_ were represented by features in each of these feature sets to comprise 100 datasets. The classic classification algorithm, random forest (RF) [44, 46–50], was performed on all above-mentioned datasets, evaluated by tenfold cross-validation. The predicted results were counted as Matthews correlation coefficient (MCC) [40, 44, 47, 51–53], which are shown in Figure 5. It can be observed that the RF with selected 158 features yielded the MCCs between 0.55 and 0.60, while the RF with randomly selected 158 features generated the MCCs around 0.23. Clearly, the selected 158 features can capture the essential properties of PPIs, thereby providing more powerful distinguishing ability. Investigation on these features can help uncover the mechanisms of PPIs.

## 4. Discussion

As mentioned in Section 3.2, 134 GO terms and one KEGG pathway were regarded important in determining the difference between positive and negative associations. This section gave a detailed analysis on them.

### 4.1. Analysis of Key GO Terms

**Figure 6:**
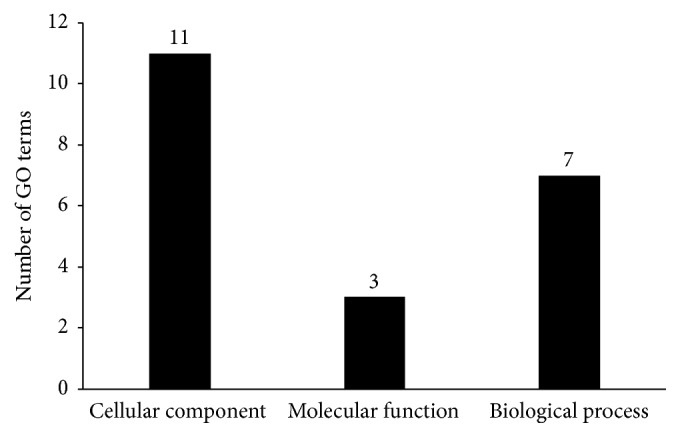
Distribution of 21 GO terms on three groups: cellular component, molecular function, and biological process.

Analyzing above-mentioned 134 GO terms one by one is difficult. Here, we selected the most important 21 GO terms with rating scores larger than 0.5 for detailed analysis, which are listed in Table 2. 21 GO terms can be clustered into three groups: cellular component, molecular function, and biological process [15]. The distribution of the aforementioned 21 GO terms on these three groups is shown in Figure 6. Eleven GO terms are clustered into cellular component, three terms into molecular function, and seven terms into biological process. All these GO terms can be proven or inferred as associated with PPIs in published literature, as to be discussed below.

*Cellular Component GO Terms*. As described above, eleven of the 21 GO terms clustered as cellular components refer to the part of a single cell and its specific extracellular environment, taking account for more than 52% of selected GO terms [16]. Comparing with molecular function and biological process as other two GO categories, which mostly reflect the indirect and functional relationships between different proteins, cellular component reflects the direct interactive relationships. Thus, the enrichment of functional clustered GO terms in such GO category indicated that subcellular localization and regional protein distribution may contribute more to the distinction of positive and negative associations. Direct PPIs which take the majority of all PPIs relied on the direct molecular interactions between proteins. The participants of most positive associations must share similar physical subcellular localizations, while those of the negative ones do not have to. Therefore, comparing to molecular function and biological process, it is quite reasonable for the cellular component category of GO terms to take the majority of all the enriched biological processes contributing to the recognition of positive PPIs.

The cellular component GO term with the highest rating score was GO: 0044428, describing the nuclear part of the eukaryotic cells, involving in chromosomes housing and replicating. Such processes involve multiple effective PPIs, like Esc2 and Rad51 [54, 55]. Therefore, the functional enrichment of genes involved in such cellular component may be more probable to participate in an actual PPI, contributing to the recognition of positive PPIs. Similarly, GO: 0031981, describing the nuclear lumen region, and GO: 0005634, describing a more general region of the cell, nucleus, may also involve in multiple PPIs. It has been widely reported that the nucleus region involves multiple subgroup of PPIs, regulating the expression and replication of genes [56–58]. Therefore, having nucleus as one of the busiest regions in cells, genes identified in such region may actually tend to be participating in certain PPIs.

Apart from the nucleus region of the cell, according to our results, we also identified that cellular regions associated with functional organelles may also be related to PPIs. GO: 0044422, describing the organelle part of cells, GO: 0070013, describing intracellular organelle lumen, GO:0044446, describing the intracellular organelle part, and GO:0043233, describing organelle lumen, have all been screened out as the potential cellular components that may be associated with positive PPIs [59]. Similar with the nucleus regions, comparing to extracellular matrix and other intracellular regions, the organelles and its related biochemical reactions space involve in more actually interacting PPIs [59–61]. Therefore, PPIs that locate in such region tend out to actually happen, indicating that these GO terms contribute to describing an effective gene cellular component features of genes that actually participate in PPIs.

Apart from such specific GO terms, we also identified some more general ones, like GO: 0032991 (protein-containing complex), GO: 0044424 (intracellular part), GO: 0005622 (intracellular), and GO: 0031974 (membrane-enclosed lumen). They all describe the regions that enrich significant biological processes of the cells. Therefore, actual PPIs tend to enrich in such region, revealing the specific PPI distribution pattern in the eukaryotic cells.

*Molecular Function GO Terms*. Three molecular function associated GO terms were extracted. The top GO term was GO: 1901363, describing heterocyclic compound binding. According to recent publications, various PPIs can actually be functional enriched in the heterocyclic compound binding, like the interactions between PDK1 and AKT in the eukaryotic cells [62, 63]. Therefore, genes that participate in such molecular function may tend to be more probable to actually contribute to PPIs. Similarly, the other molecular function GO term, named GO: 0097159, which describes organic cyclic compound binding, also involves various PPIs, like interactions among TBK1, PDPK1, and AURKA [64]. As for the last term, GO: 0003676, it describes the nucleic acid binding. As analyzed above, the nucleus region, where nucleic acid binding processes mostly occur, can distinguish the positive and negative PPIs due to its relative high interaction frequency [56–58]. Therefore, it is quite reasonable to speculate that such molecular function GO term may also be related to PPIs.

*Biological Process GO Terms*. Apart from above-mentioned cellular component and molecular function associated GO terms, we also identified a group of functional enrichment results that can be clustered into the biological processes cluster. All these GO terms describe effective metabolic processes in the cells. GO: 0044260 and GO: 0043170 describe the macromolecule metabolic processes. According to recent publications, such metabolic processes involve various PPIs, like the interactions in mTOR signaling pathways [65]. Apart from that, GO: 0044238, describing the primary metabolic process, has also been confirmed to contribute to PPIs. Considering the normal anabolic and catabolic processes, all involving functional PPIs [66–68], it is quite reasonable for genes participating in such biological processes to also participate in effective PPIs. The following three GO terms, GO: 0090304 (nucleic acid metabolic process), GO: 0006139 (nucleobase-containing compound metabolic process), and GO: 0071704 (organic substance metabolic process), may also contribute to PPIs, considering that the nucleus region has been discussed to be quite significant for PPIs [56–58] and proteins turn out to be one of the major subgroups of organic substance in eukaryotic cells; these three GO terms may also actually contribute to the identification of PPIs. As for the remaining GO term, GO: 0044237, it describes a general concept of all the cellular metabolic processes. Considering the analyses listed above, metabolic processes in the cells enrich various actual PPIs and are reasonable to be predicted and screened out as a potential identifier for positive PPIs.

On the basis of the analyses, all 21 GO terms are involved in different aspects of PPI, and they can be used to mark proteins with functional associations. For the remaining GO terms shown in Supplementary Material S4, it is anticipated that they also have associations with PPIs.

### 4.2. Analysis of Other GO Terms and KEGG Pathways

As for other GO terms extracted in this study, although not so relevant with PPIs as such GO terms described in Section 4.1, some of them have also been reported to be functionally related to certain PPIs. For instance, GO: 0006807, describing nitrogen compound metabolism, has been widely reported to be functionally related to compound-protein interactions but not protein–protein interactions [69, 70]. However, when extensively studying biological processes of such GO term, we found out that various specific PPIs are just like the interactions between the protein products of* TIMP1* and* MMP2* [71]. Therefore, in this study, some identified GO terms have not been directly reported to contribute to the PPIs. However, by digging deep into the actual biological processes, molecular functions and cellular components of them, we actually found that various novel identified PPIs are associated with these GO terms.

Furthermore, one KEGG pathway hsa03010 was obtained in our study. It describes the ribosome associated pathway. Considering that genes/proteins that participate in such pathway may interact with each other, forming the complex of ribosome, such KEGG pathway, may also contribute to the distinction of positive and negative PPIs.

## 5. Conclusions

This study investigated protein–protein functional associations based on GO terms and KEGG pathways. By using mutual information, we identified important GO terms and KEGG pathways that can describe the difference between actual associations and pairs of proteins without associations and help understand the mechanisms of protein interactions. A possible future research direction is to further use these GO terms and KEGG pathways to build a computational method for inferring novel associations between proteins, enriching the biological functional annotation of proteins.

## Figures and Tables

**Table 1:** Number of selected features in each MaxRel feature list.

Dataset	Number of selected features
*DS*_1_	154
*DS*_2_	154
*DS*_3_	153
*DS*_4_	155
*DS*_5_	149
*DS*_6_	150
*DS*_7_	155
*DS*_8_	152
*DS*_9_	153
*DS*_10_	153

**Table 2:** Information of most important 21 GO terms.

GO term ID	GO term	Rating score	Group
GO:0044260	cellular macromolecule metabolic process	0.688	Biological process
GO:0043170	macromolecule metabolic process	0.640	Biological process
GO:0044428	nuclear part	0.618	Cellular component
GO:1901363	heterocyclic compound binding	0.600	Molecular function
GO:0032991	protein-containing complex	0.593	Cellular component
GO:0097159	organic cyclic compound binding	0.591	Molecular function
GO:0031981	nuclear lumen	0.590	Cellular component
GO:0044238	primary metabolic process	0.589	Biological process
GO:0003676	nucleic acid binding	0.583	Molecular function
GO:0090304	nucleic acid metabolic process	0.569	Biological process
GO:0071704	organic substance metabolic process	0.556	Biological process
GO:0044237	cellular metabolic process	0.552	Biological process
GO:0005634	nucleus	0.549	Cellular component
GO:0044446	intracellular organelle part	0.547	Cellular component
GO:0044424	intracellular part	0.537	Cellular component
GO:0044422	organelle part	0.536	Cellular component
GO:0070013	intracellular organelle lumen	0.529	Cellular component
GO:0005622	intracellular	0.523	Cellular component
GO:0043233	organelle lumen	0.521	Cellular component
GO:0031974	membrane-enclosed lumen	0.514	Cellular component
GO:0006139	nucleobase-containing compound metabolic process	0.506	Biological process

## Data Availability

The original data used to support the findings of this study are available at STRING dataset and in supplementary information files.

## Supplementary Materials

Supplementary 1 70392 protein–protein functional associations.

Supplementary 2 A part of MaxRel feature list on ten datasets obtained by mutual information of each feature.

Supplementary 3 Selected 158 features and their occurrences in the 10 feature sets of the 10 datasets (√: feature is in the feature set; ☓: feature is not included in the feature set).

Supplementary 4 Extracted important GO terms and their rating scores.
