# Supplementary material for: Analysis of Protein–Protein Functional Associations by Using Gene Ontology and KEGG Pathway
Source: Biomed Res Int. 2019 Jul 18;2019:4963289. doi: 10.1155/2019/4963289 (PMC6668538; doi:10.1155/2019/4963289)
Supplement: Supplementary 2 — A part of MaxRel feature list on ten datasets obtained by mutual information of each feature. [file 4963289.f2.docx]

**Supplementary Material S2.** A part of MaxRel feature list on ten datasets obtained by mutual information of each feature

1. Part of MaxRel feature list on *DS*_1_

| **Feature** | **Score** |
| --- | --- |
| GO:0044428_1+GO:0044428_2 | 0.061773 |
| GO:1901363_1+GO:1901363_2 | 0.060524 |
| GO:0097159_1+GO:0097159_2 | 0.059689 |
| GO:0032991_1+GO:0032991_2 | 0.059345 |
| GO:0031981_1+GO:0031981_2 | 0.058817 |
| GO:0003676_1+GO:0003676_2 | 0.058778 |
| GO:0090304_1+GO:0090304_2 | 0.057685 |
| GO:0044260_1+GO:0044260_2 | 0.054618 |
| GO:0044446_1+GO:0044446_2 | 0.054513 |
| GO:0005634_1+GO:0005634_2 | 0.053704 |
| GO:0044422_1+GO:0044422_2 | 0.053406 |
| GO:0070013_1+GO:0070013_2 | 0.052513 |
| GO:0043233_1+GO:0043233_2 | 0.051704 |
| GO:0006139_1+GO:0006139_2 | 0.051383 |
| GO:0031974_1+GO:0031974_2 | 0.050987 |
| GO:0046483_1+GO:0046483_2 | 0.048816 |
| GO:0043170_1+GO:0043170_2 | 0.048588 |
| GO:0006725_1+GO:0006725_2 | 0.048386 |
| GO:1901360_1+GO:1901360_2 | 0.046439 |
| GO:0034641_1+GO:0034641_2 | 0.046024 |
| GO:0010467_1+GO:0010467_2 | 0.044285 |
| GO:0006807_1+GO:0006807_2 | 0.042318 |
| GO:0044238_1+GO:0044238_2 | 0.040622 |
| GO:0016070_1+GO:0016070_2 | 0.040386 |
| GO:0044237_1+GO:0044237_2 | 0.038111 |
| GO:0071704_1+GO:0071704_2 | 0.037628 |
| GO:0043228_1+GO:0043228_2 | 0.036247 |
| GO:0043232_1+GO:0043232_2 | 0.036247 |
| GO:0034645_1+GO:0034645_2 | 0.033346 |
| GO:0009059_1+GO:0009059_2 | 0.032216 |
| GO:0005654_1+GO:0005654_2 | 0.031401 |
| GO:0044424_1+GO:0044424_2 | 0.029869 |
| GO:0005622_1+GO:0005622_2 | 0.028978 |
| GO:0071840_1+GO:0071840_2 | 0.028662 |
| GO:1901576_1+GO:1901576_2 | 0.027578 |
| GO:0044249_1+GO:0044249_2 | 0.027339 |
| GO:0009058_1+GO:0009058_2 | 0.026521 |
| GO:0043234_1+GO:0043234_2 | 0.025051 |
| GO:0032774_1+GO:0032774_2 | 0.024754 |
| GO:0044822_1+GO:0044822_2 | 0.024349 |
| abs(GO:0005488_1-GO:0005488_2) | 0.023395 |
| GO:0034654_1+GO:0034654_2 | 0.023278 |
| GO:0003723_1+GO:0003723_2 | 0.023236 |
| abs(GO:0044424_1-GO:0044424_2) | 0.02312 |
| abs(GO:0005622_1-GO:0005622_2) | 0.022599 |
| GO:0018130_1+GO:0018130_2 | 0.022369 |
| GO:0019438_1+GO:0019438_2 | 0.022327 |
| GO:0044271_1+GO:0044271_2 | 0.021907 |
| GO:0016043_1+GO:0016043_2 | 0.021901 |
| abs(GO:0043229_1-GO:0043229_2) | 0.021653 |
| GO:0016071_1+GO:0016071_2 | 0.021618 |
| GO:1901362_1+GO:1901362_2 | 0.021614 |
| abs(GO:0044425_1-GO:0044425_2) | 0.021412 |
| abs(GO:0043226_1-GO:0043226_2) | 0.021399 |
| abs(GO:0031224_1-GO:0031224_2) | 0.021126 |
| GO:0005515_1+GO:0005515_2 | 0.02109 |
| GO:0044764_1+GO:0044764_2 | 0.020685 |
| GO:0016032_1+GO:0016032_2 | 0.020517 |
| abs(GO:0016021_1-GO:0016021_2) | 0.020454 |
| GO:0044267_1+GO:0044267_2 | 0.02001 |
| GO:0044403_1+GO:0044403_2 | 0.019874 |
| GO:0044419_1+GO:0044419_2 | 0.019874 |
| GO:0031224_1+GO:0031224_2 | 0.019762 |
| GO:0060255_1+GO:0060255_2 | 0.019269 |
| GO:0030529_1+GO:0030529_2 | 0.019106 |
| GO:0016021_1+GO:0016021_2 | 0.01894 |
| GO:0044451_1+GO:0044451_2 | 0.018807 |
| abs(GO:0044238_1-GO:0044238_2) | 0.018649 |
| GO:0005730_1+GO:0005730_2 | 0.018508 |
| abs(GO:0071704_1-GO:0071704_2) | 0.018309 |
| GO:0006351_1+GO:0006351_2 | 0.018195 |
| abs(GO:0008152_1-GO:0008152_2) | 0.017855 |
| abs(GO:0043227_1-GO:0043227_2) | 0.017608 |
| GO:0010556_1+GO:0010556_2 | 0.017544 |
| abs(GO:0044237_1-GO:0044237_2) | 0.017476 |
| GO:0044265_1+GO:0044265_2 | 0.017063 |
| GO:0019538_1+GO:0019538_2 | 0.017032 |
| GO:0080090_1+GO:0080090_2 | 0.016978 |
| GO:0010468_1+GO:0010468_2 | 0.016872 |
| GO:2000112_1+GO:2000112_2 | 0.016704 |
| abs(GO:0016020_1-GO:0016020_2) | 0.016588 |
| GO:0031323_1+GO:0031323_2 | 0.016517 |
| GO:0031326_1+GO:0031326_2 | 0.016438 |
| GO:0019083_1+GO:0019083_2 | 0.016436 |
| GO:0051171_1+GO:0051171_2 | 0.016364 |
| GO:0009889_1+GO:0009889_2 | 0.016279 |
| GO:0044464_1+GO:0044464_2 | 0.016041 |
| GO:0005623_1+GO:0005623_2 | 0.016014 |
| GO:0003674_1+GO:0003674_2 | 0.01591 |
| GO:0022613_1+GO:0022613_2 | 0.015864 |
| GO:0019219_1+GO:0019219_2 | 0.015801 |
| GO:0019222_1+GO:0019222_2 | 0.015788 |
| abs(GO:0043170_1-GO:0043170_2) | 0.015323 |
| GO:0009057_1+GO:0009057_2 | 0.015165 |
| GO:0005829_1+GO:0005829_2 | 0.015127 |
| GO:0000956_1+GO:0000956_2 | 0.014985 |
| GO:0051276_1+GO:0051276_2 | 0.014915 |
| GO:0006996_1+GO:0006996_2 | 0.014842 |
| abs(GO:0043231_1-GO:0043231_2) | 0.014841 |
| GO:0006402_1+GO:0006402_2 | 0.014628 |
| GO:0006401_1+GO:0006401_2 | 0.014401 |
| GO:0006396_1+GO:0006396_2 | 0.014288 |
| GO:0009987_1+GO:0009987_2 | 0.014239 |
| abs(GO:0044260_1-GO:0044260_2) | 0.014154 |
| abs(GO:0003674_1-GO:0003674_2) | 0.014023 |
| GO:0043933_1+GO:0043933_2 | 0.01358 |
| abs(GO:0044464_1-GO:0044464_2) | 0.013457 |
| abs(GO:0005623_1-GO:0005623_2) | 0.01344 |
| GO:0006413_1+GO:0006413_2 | 0.013356 |
| GO:0000184_1+GO:0000184_2 | 0.013307 |
| GO:0006415_1+GO:0006415_2 | 0.013165 |
| GO:0032984_1+GO:0032984_2 | 0.012696 |
| GO:0043241_1+GO:0043241_2 | 0.012671 |
| GO:0034655_1+GO:0034655_2 | 0.012568 |
| abs(GO:0044764_1-GO:0044764_2) | 0.012376 |
| GO:0006974_1+GO:0006974_2 | 0.012354 |
| abs(GO:0016032_1-GO:0016032_2) | 0.012251 |
| GO:0022626_1+GO:0022626_2 | 0.012199 |
| GO:0005694_1+GO:0005694_2 | 0.012076 |
| GO:0043624_1+GO:0043624_2 | 0.012045 |
| GO:0042254_1+GO:0042254_2 | 0.011911 |
| GO:0019080_1+GO:0019080_2 | 0.011878 |
| GO:0044033_1+GO:0044033_2 | 0.011835 |
| GO:0044270_1+GO:0044270_2 | 0.011707 |
| abs(GO:0044419_1-GO:0044419_2) | 0.01168 |
| abs(GO:0044403_1-GO:0044403_2) | 0.01168 |
| GO:0046700_1+GO:0046700_2 | 0.011553 |
| GO:0019439_1+GO:0019439_2 | 0.01138 |
| GO:0006414_1+GO:0006414_2 | 0.011318 |
| GO:0006614_1+GO:0006614_2 | 0.011104 |
| abs(GO:0009987_1-GO:0009987_2) | 0.011039 |
| GO:0045047_1+GO:0045047_2 | 0.011009 |
| GO:0072599_1+GO:0072599_2 | 0.011009 |
| GO:0022411_1+GO:0022411_2 | 0.011004 |
| GO:0044445_1+GO:0044445_2 | 0.010984 |
| GO:0006613_1+GO:0006613_2 | 0.010837 |
| GO:0044427_1+GO:0044427_2 | 0.010799 |
| GO:1901361_1+GO:1901361_2 | 0.010775 |
| GO:0006281_1+GO:0006281_2 | 0.010775 |
| abs(GO:0005730_1-GO:0005730_2) | 0.010714 |
| GO:0019058_1+GO:0019058_2 | 0.010704 |
| hsa03010_1+hsa03010_2 | 0.010688 |
| GO:1990234_1+GO:1990234_2 | 0.010466 |
| GO:0016604_1+GO:0016604_2 | 0.010457 |
| GO:0070972_1+GO:0070972_2 | 0.010416 |
| GO:0006412_1+GO:0006412_2 | 0.010293 |
| GO:0008150_1+GO:0008150_2 | 0.010281 |
| GO:0044710_1+GO:0044710_2 | 0.010265 |
| GO:0044391_1+GO:0044391_2 | 0.010196 |
| GO:0006259_1+GO:0006259_2 | 0.010176 |
| abs(GO:0019083_1-GO:0019083_2) | 0.010123 |
| GO:0000375_1+GO:0000375_2 | 0.0101 |
| GO:0033554_1+GO:0033554_2 | 0.010011 |

1. Part of MaxRel feature list on *DS*_2_

| **Feature** | **Score** |
| --- | --- |
| GO:0044428_1+GO:0044428_2 | 0.062563 |
| GO:1901363_1+GO:1901363_2 | 0.060441 |
| GO:0031981_1+GO:0031981_2 | 0.059966 |
| GO:0032991_1+GO:0032991_2 | 0.059618 |
| GO:0097159_1+GO:0097159_2 | 0.059585 |
| GO:0003676_1+GO:0003676_2 | 0.058332 |
| GO:0090304_1+GO:0090304_2 | 0.056878 |
| GO:0044446_1+GO:0044446_2 | 0.055506 |
| GO:0044260_1+GO:0044260_2 | 0.05464 |
| GO:0005634_1+GO:0005634_2 | 0.054445 |
| GO:0044422_1+GO:0044422_2 | 0.054378 |
| GO:0070013_1+GO:0070013_2 | 0.053773 |
| GO:0043233_1+GO:0043233_2 | 0.052892 |
| GO:0031974_1+GO:0031974_2 | 0.052258 |
| GO:0006139_1+GO:0006139_2 | 0.050703 |
| GO:0043170_1+GO:0043170_2 | 0.048484 |
| GO:0046483_1+GO:0046483_2 | 0.048339 |
| GO:0006725_1+GO:0006725_2 | 0.04788 |
| GO:1901360_1+GO:1901360_2 | 0.045953 |
| GO:0034641_1+GO:0034641_2 | 0.045544 |
| GO:0010467_1+GO:0010467_2 | 0.044386 |
| GO:0006807_1+GO:0006807_2 | 0.042068 |
| GO:0044238_1+GO:0044238_2 | 0.040588 |
| GO:0016070_1+GO:0016070_2 | 0.039578 |
| GO:0044237_1+GO:0044237_2 | 0.038006 |
| GO:0071704_1+GO:0071704_2 | 0.037559 |
| GO:0043232_1+GO:0043232_2 | 0.036491 |
| GO:0043228_1+GO:0043228_2 | 0.036491 |
| GO:0034645_1+GO:0034645_2 | 0.033246 |
| GO:0009059_1+GO:0009059_2 | 0.032223 |
| GO:0005654_1+GO:0005654_2 | 0.031952 |
| GO:0044424_1+GO:0044424_2 | 0.030808 |
| GO:0005622_1+GO:0005622_2 | 0.029825 |
| GO:0071840_1+GO:0071840_2 | 0.029337 |
| GO:1901576_1+GO:1901576_2 | 0.027534 |
| GO:0044249_1+GO:0044249_2 | 0.027259 |
| GO:0009058_1+GO:0009058_2 | 0.026623 |
| GO:0043234_1+GO:0043234_2 | 0.025431 |
| GO:0032774_1+GO:0032774_2 | 0.024352 |
| GO:0044822_1+GO:0044822_2 | 0.023905 |
| abs(GO:0044424_1-GO:0044424_2) | 0.02382 |
| abs(GO:0005488_1-GO:0005488_2) | 0.023781 |
| abs(GO:0005622_1-GO:0005622_2) | 0.023238 |
| GO:0034654_1+GO:0034654_2 | 0.022958 |
| GO:0003723_1+GO:0003723_2 | 0.022702 |
| GO:0016043_1+GO:0016043_2 | 0.022405 |
| GO:0018130_1+GO:0018130_2 | 0.022067 |
| abs(GO:0043229_1-GO:0043229_2) | 0.022016 |
| GO:0019438_1+GO:0019438_2 | 0.021907 |
| abs(GO:0043226_1-GO:0043226_2) | 0.021685 |
| GO:0044271_1+GO:0044271_2 | 0.021502 |
| GO:0016071_1+GO:0016071_2 | 0.021326 |
| GO:1901362_1+GO:1901362_2 | 0.0213 |
| GO:0005515_1+GO:0005515_2 | 0.021255 |
| GO:0044764_1+GO:0044764_2 | 0.020961 |
| GO:0044267_1+GO:0044267_2 | 0.020887 |
| GO:0016032_1+GO:0016032_2 | 0.020802 |
| abs(GO:0031224_1-GO:0031224_2) | 0.020627 |
| abs(GO:0044425_1-GO:0044425_2) | 0.020512 |
| GO:0044403_1+GO:0044403_2 | 0.020079 |
| GO:0044419_1+GO:0044419_2 | 0.020079 |
| abs(GO:0016021_1-GO:0016021_2) | 0.019957 |
| GO:0031224_1+GO:0031224_2 | 0.019496 |
| GO:0044451_1+GO:0044451_2 | 0.019383 |
| GO:0005730_1+GO:0005730_2 | 0.019098 |
| GO:0030529_1+GO:0030529_2 | 0.019092 |
| GO:0060255_1+GO:0060255_2 | 0.018772 |
| GO:0016021_1+GO:0016021_2 | 0.018684 |
| abs(GO:0044238_1-GO:0044238_2) | 0.018533 |
| abs(GO:0071704_1-GO:0071704_2) | 0.018189 |
| GO:0006351_1+GO:0006351_2 | 0.017975 |
| abs(GO:0043227_1-GO:0043227_2) | 0.017816 |
| abs(GO:0008152_1-GO:0008152_2) | 0.017763 |
| GO:0019538_1+GO:0019538_2 | 0.017598 |
| abs(GO:0044237_1-GO:0044237_2) | 0.017389 |
| GO:0010556_1+GO:0010556_2 | 0.017322 |
| GO:0044265_1+GO:0044265_2 | 0.017118 |
| GO:0044464_1+GO:0044464_2 | 0.016812 |
| GO:0005623_1+GO:0005623_2 | 0.016796 |
| GO:0080090_1+GO:0080090_2 | 0.016788 |
| GO:0022613_1+GO:0022613_2 | 0.016738 |
| GO:2000112_1+GO:2000112_2 | 0.016632 |
| GO:0010468_1+GO:0010468_2 | 0.016495 |
| GO:0031326_1+GO:0031326_2 | 0.01642 |
| GO:0031323_1+GO:0031323_2 | 0.016414 |
| GO:0019083_1+GO:0019083_2 | 0.016335 |
| GO:0051171_1+GO:0051171_2 | 0.016308 |
| GO:0003674_1+GO:0003674_2 | 0.016293 |
| GO:0009889_1+GO:0009889_2 | 0.016211 |
| abs(GO:0016020_1-GO:0016020_2) | 0.015928 |
| GO:0019219_1+GO:0019219_2 | 0.015697 |
| abs(GO:0043170_1-GO:0043170_2) | 0.015512 |
| GO:0006996_1+GO:0006996_2 | 0.015332 |
| GO:0019222_1+GO:0019222_2 | 0.015282 |
| GO:0009057_1+GO:0009057_2 | 0.01519 |
| abs(GO:0043231_1-GO:0043231_2) | 0.015048 |
| GO:0005829_1+GO:0005829_2 | 0.014923 |
| GO:0051276_1+GO:0051276_2 | 0.014803 |
| GO:0000956_1+GO:0000956_2 | 0.014643 |
| abs(GO:0003674_1-GO:0003674_2) | 0.014413 |
| GO:0006402_1+GO:0006402_2 | 0.014359 |
| abs(GO:0044260_1-GO:0044260_2) | 0.014344 |
| GO:0006396_1+GO:0006396_2 | 0.014324 |
| abs(GO:0044464_1-GO:0044464_2) | 0.014142 |
| abs(GO:0005623_1-GO:0005623_2) | 0.014134 |
| GO:0009987_1+GO:0009987_2 | 0.014129 |
| GO:0006401_1+GO:0006401_2 | 0.014045 |
| GO:0043933_1+GO:0043933_2 | 0.013571 |
| GO:0006413_1+GO:0006413_2 | 0.013267 |
| GO:0000184_1+GO:0000184_2 | 0.013199 |
| GO:0006415_1+GO:0006415_2 | 0.013031 |
| GO:0032984_1+GO:0032984_2 | 0.012579 |
| GO:0034655_1+GO:0034655_2 | 0.012542 |
| abs(GO:0044764_1-GO:0044764_2) | 0.012482 |
| GO:0043241_1+GO:0043241_2 | 0.012475 |
| GO:0005694_1+GO:0005694_2 | 0.012468 |
| GO:0042254_1+GO:0042254_2 | 0.012456 |
| abs(GO:0016032_1-GO:0016032_2) | 0.012359 |
| GO:0006974_1+GO:0006974_2 | 0.012357 |
| GO:0022626_1+GO:0022626_2 | 0.012178 |
| GO:0043624_1+GO:0043624_2 | 0.011866 |
| GO:0019080_1+GO:0019080_2 | 0.011756 |
| GO:0044033_1+GO:0044033_2 | 0.011744 |
| abs(GO:0044403_1-GO:0044403_2) | 0.011728 |
| abs(GO:0044419_1-GO:0044419_2) | 0.011728 |
| GO:0044270_1+GO:0044270_2 | 0.011696 |
| GO:0046700_1+GO:0046700_2 | 0.011534 |
| GO:0019439_1+GO:0019439_2 | 0.011388 |
| GO:0006414_1+GO:0006414_2 | 0.011245 |
| abs(GO:0005730_1-GO:0005730_2) | 0.011088 |
| GO:0044427_1+GO:0044427_2 | 0.011066 |
| GO:0016604_1+GO:0016604_2 | 0.011065 |
| abs(GO:0009987_1-GO:0009987_2) | 0.01098 |
| GO:0006614_1+GO:0006614_2 | 0.010965 |
| GO:0022411_1+GO:0022411_2 | 0.01089 |
| GO:0072599_1+GO:0072599_2 | 0.010888 |
| GO:0045047_1+GO:0045047_2 | 0.010888 |
| GO:1990234_1+GO:1990234_2 | 0.010862 |
| GO:1901361_1+GO:1901361_2 | 0.010769 |
| GO:0006613_1+GO:0006613_2 | 0.010752 |
| abs(GO:0022613_1-GO:0022613_2) | 0.010695 |
| GO:0044445_1+GO:0044445_2 | 0.01069 |
| hsa03010_1+hsa03010_2 | 0.01069 |
| GO:0006281_1+GO:0006281_2 | 0.010661 |
| GO:0019058_1+GO:0019058_2 | 0.010495 |
| abs(GO:0044451_1-GO:0044451_2) | 0.010414 |
| GO:0070972_1+GO:0070972_2 | 0.010333 |
| GO:0044710_1+GO:0044710_2 | 0.010318 |
| GO:0044391_1+GO:0044391_2 | 0.010214 |
| GO:0033554_1+GO:0033554_2 | 0.010206 |
| GO:0006259_1+GO:0006259_2 | 0.010163 |
| GO:0000375_1+GO:0000375_2 | 0.01014 |
| GO:0008150_1+GO:0008150_2 | 0.010128 |
| abs(GO:0019083_1-GO:0019083_2) | 0.010058 |

1. Part of MaxRel feature list on *DS*_3_

| **Feature** | **Score** |
| --- | --- |
| GO:0044428_1+GO:0044428_2 | 0.062018 |
| GO:1901363_1+GO:1901363_2 | 0.060434 |
| GO:0097159_1+GO:0097159_2 | 0.059663 |
| GO:0032991_1+GO:0032991_2 | 0.059246 |
| GO:0031981_1+GO:0031981_2 | 0.059165 |
| GO:0003676_1+GO:0003676_2 | 0.058421 |
| GO:0090304_1+GO:0090304_2 | 0.056526 |
| GO:0044446_1+GO:0044446_2 | 0.055268 |
| GO:0044260_1+GO:0044260_2 | 0.054699 |
| GO:0005634_1+GO:0005634_2 | 0.054192 |
| GO:0044422_1+GO:0044422_2 | 0.053982 |
| GO:0070013_1+GO:0070013_2 | 0.053365 |
| GO:0043233_1+GO:0043233_2 | 0.052635 |
| GO:0031974_1+GO:0031974_2 | 0.051951 |
| GO:0006139_1+GO:0006139_2 | 0.050278 |
| GO:0043170_1+GO:0043170_2 | 0.048763 |
| GO:0046483_1+GO:0046483_2 | 0.047833 |
| GO:0006725_1+GO:0006725_2 | 0.047471 |
| GO:1901360_1+GO:1901360_2 | 0.045629 |
| GO:0034641_1+GO:0034641_2 | 0.045354 |
| GO:0010467_1+GO:0010467_2 | 0.044135 |
| GO:0006807_1+GO:0006807_2 | 0.041933 |
| GO:0044238_1+GO:0044238_2 | 0.040686 |
| GO:0016070_1+GO:0016070_2 | 0.038976 |
| GO:0044237_1+GO:0044237_2 | 0.038284 |
| GO:0071704_1+GO:0071704_2 | 0.03765 |
| GO:0043228_1+GO:0043228_2 | 0.036134 |
| GO:0043232_1+GO:0043232_2 | 0.036134 |
| GO:0034645_1+GO:0034645_2 | 0.032742 |
| GO:0009059_1+GO:0009059_2 | 0.031722 |
| GO:0005654_1+GO:0005654_2 | 0.031486 |
| GO:0044424_1+GO:0044424_2 | 0.030793 |
| GO:0005622_1+GO:0005622_2 | 0.029992 |
| GO:0071840_1+GO:0071840_2 | 0.028635 |
| GO:1901576_1+GO:1901576_2 | 0.027237 |
| GO:0044249_1+GO:0044249_2 | 0.027003 |
| GO:0009058_1+GO:0009058_2 | 0.026314 |
| GO:0043234_1+GO:0043234_2 | 0.025044 |
| GO:0044822_1+GO:0044822_2 | 0.024084 |
| abs(GO:0044424_1-GO:0044424_2) | 0.023905 |
| GO:0032774_1+GO:0032774_2 | 0.023899 |
| abs(GO:0005488_1-GO:0005488_2) | 0.023603 |
| abs(GO:0005622_1-GO:0005622_2) | 0.023445 |
| GO:0003723_1+GO:0003723_2 | 0.022934 |
| GO:0034654_1+GO:0034654_2 | 0.022577 |
| GO:0016043_1+GO:0016043_2 | 0.021858 |
| abs(GO:0043229_1-GO:0043229_2) | 0.021826 |
| GO:0018130_1+GO:0018130_2 | 0.021649 |
| GO:0019438_1+GO:0019438_2 | 0.021635 |
| GO:0005515_1+GO:0005515_2 | 0.021581 |
| abs(GO:0043226_1-GO:0043226_2) | 0.021453 |
| GO:0044271_1+GO:0044271_2 | 0.02121 |
| GO:1901362_1+GO:1901362_2 | 0.021074 |
| GO:0016071_1+GO:0016071_2 | 0.021037 |
| GO:0044764_1+GO:0044764_2 | 0.020895 |
| abs(GO:0044425_1-GO:0044425_2) | 0.020886 |
| abs(GO:0031224_1-GO:0031224_2) | 0.020753 |
| GO:0016032_1+GO:0016032_2 | 0.020716 |
| GO:0044267_1+GO:0044267_2 | 0.020517 |
| abs(GO:0016021_1-GO:0016021_2) | 0.020046 |
| GO:0044419_1+GO:0044419_2 | 0.02002 |
| GO:0044403_1+GO:0044403_2 | 0.02002 |
| GO:0031224_1+GO:0031224_2 | 0.019639 |
| GO:0060255_1+GO:0060255_2 | 0.018953 |
| GO:0016021_1+GO:0016021_2 | 0.0188 |
| GO:0044451_1+GO:0044451_2 | 0.018792 |
| GO:0005730_1+GO:0005730_2 | 0.018771 |
| abs(GO:0044238_1-GO:0044238_2) | 0.018608 |
| GO:0030529_1+GO:0030529_2 | 0.018504 |
| abs(GO:0071704_1-GO:0071704_2) | 0.018271 |
| abs(GO:0008152_1-GO:0008152_2) | 0.018096 |
| abs(GO:0043227_1-GO:0043227_2) | 0.017625 |
| GO:0006351_1+GO:0006351_2 | 0.017551 |
| abs(GO:0044237_1-GO:0044237_2) | 0.017467 |
| GO:0019538_1+GO:0019538_2 | 0.017466 |
| GO:0044265_1+GO:0044265_2 | 0.017442 |
| GO:0010556_1+GO:0010556_2 | 0.017129 |
| GO:0044464_1+GO:0044464_2 | 0.016803 |
| GO:0080090_1+GO:0080090_2 | 0.016803 |
| GO:0005623_1+GO:0005623_2 | 0.01677 |
| GO:0003674_1+GO:0003674_2 | 0.016588 |
| GO:0031323_1+GO:0031323_2 | 0.016522 |
| GO:2000112_1+GO:2000112_2 | 0.016469 |
| GO:0010468_1+GO:0010468_2 | 0.016453 |
| abs(GO:0016020_1-GO:0016020_2) | 0.016299 |
| GO:0019083_1+GO:0019083_2 | 0.016252 |
| GO:0031326_1+GO:0031326_2 | 0.016181 |
| GO:0022613_1+GO:0022613_2 | 0.0161 |
| GO:0009889_1+GO:0009889_2 | 0.016028 |
| GO:0051171_1+GO:0051171_2 | 0.015878 |
| GO:0019222_1+GO:0019222_2 | 0.015575 |
| GO:0009057_1+GO:0009057_2 | 0.015447 |
| abs(GO:0043170_1-GO:0043170_2) | 0.015344 |
| GO:0005829_1+GO:0005829_2 | 0.015299 |
| GO:0019219_1+GO:0019219_2 | 0.015269 |
| GO:0006996_1+GO:0006996_2 | 0.015245 |
| GO:0051276_1+GO:0051276_2 | 0.014962 |
| abs(GO:0043231_1-GO:0043231_2) | 0.014871 |
| GO:0000956_1+GO:0000956_2 | 0.014779 |
| abs(GO:0003674_1-GO:0003674_2) | 0.014643 |
| GO:0006402_1+GO:0006402_2 | 0.014527 |
| GO:0009987_1+GO:0009987_2 | 0.014353 |
| GO:0006401_1+GO:0006401_2 | 0.014265 |
| abs(GO:0044464_1-GO:0044464_2) | 0.01414 |
| abs(GO:0005623_1-GO:0005623_2) | 0.014118 |
| abs(GO:0044260_1-GO:0044260_2) | 0.01407 |
| GO:0006396_1+GO:0006396_2 | 0.013847 |
| GO:0043933_1+GO:0043933_2 | 0.013835 |
| GO:0000184_1+GO:0000184_2 | 0.013501 |
| GO:0006413_1+GO:0006413_2 | 0.013413 |
| GO:0006415_1+GO:0006415_2 | 0.013153 |
| GO:0032984_1+GO:0032984_2 | 0.012708 |
| GO:0043241_1+GO:0043241_2 | 0.012671 |
| GO:0005694_1+GO:0005694_2 | 0.012482 |
| GO:0022626_1+GO:0022626_2 | 0.012454 |
| abs(GO:0044764_1-GO:0044764_2) | 0.012432 |
| GO:0034655_1+GO:0034655_2 | 0.012372 |
| abs(GO:0016032_1-GO:0016032_2) | 0.012301 |
| GO:0042254_1+GO:0042254_2 | 0.01221 |
| GO:0006974_1+GO:0006974_2 | 0.01215 |
| GO:0043624_1+GO:0043624_2 | 0.012008 |
| GO:0044033_1+GO:0044033_2 | 0.011929 |
| GO:0019080_1+GO:0019080_2 | 0.011889 |
| abs(GO:0044419_1-GO:0044419_2) | 0.011681 |
| abs(GO:0044403_1-GO:0044403_2) | 0.011681 |
| GO:0044270_1+GO:0044270_2 | 0.011426 |
| GO:0006414_1+GO:0006414_2 | 0.011313 |
| GO:0046700_1+GO:0046700_2 | 0.011261 |
| GO:0022411_1+GO:0022411_2 | 0.011134 |
| abs(GO:0009987_1-GO:0009987_2) | 0.011127 |
| GO:0019439_1+GO:0019439_2 | 0.011123 |
| GO:0044427_1+GO:0044427_2 | 0.011091 |
| GO:0006614_1+GO:0006614_2 | 0.011046 |
| GO:0044445_1+GO:0044445_2 | 0.011019 |
| GO:0072599_1+GO:0072599_2 | 0.010989 |
| GO:0045047_1+GO:0045047_2 | 0.010989 |
| abs(GO:0005730_1-GO:0005730_2) | 0.010855 |
| hsa03010_1+hsa03010_2 | 0.010811 |
| GO:0006613_1+GO:0006613_2 | 0.01079 |
| GO:0006281_1+GO:0006281_2 | 0.01072 |
| GO:0019058_1+GO:0019058_2 | 0.010718 |
| GO:1990234_1+GO:1990234_2 | 0.010603 |
| GO:1901361_1+GO:1901361_2 | 0.010514 |
| GO:0016604_1+GO:0016604_2 | 0.010452 |
| GO:0044391_1+GO:0044391_2 | 0.0104 |
| GO:0070972_1+GO:0070972_2 | 0.010352 |
| GO:0008150_1+GO:0008150_2 | 0.010334 |
| GO:0006412_1+GO:0006412_2 | 0.010246 |
| abs(GO:0022613_1-GO:0022613_2) | 0.010185 |
| GO:0044710_1+GO:0044710_2 | 0.010154 |
| GO:0033554_1+GO:0033554_2 | 0.010088 |
| abs(GO:0044451_1-GO:0044451_2) | 0.010032 |
| GO:0006259_1+GO:0006259_2 | 0.010003 |

1. Part of MaxRel feature list on *DS*_4_

| **Feature** | **Score** |
| --- | --- |
| GO:0044428_1+GO:0044428_2 | 0.062818 |
| GO:0031981_1+GO:0031981_2 | 0.060133 |
| GO:1901363_1+GO:1901363_2 | 0.060045 |
| GO:0032991_1+GO:0032991_2 | 0.059798 |
| GO:0097159_1+GO:0097159_2 | 0.059165 |
| GO:0003676_1+GO:0003676_2 | 0.058628 |
| GO:0090304_1+GO:0090304_2 | 0.057036 |
| GO:0044446_1+GO:0044446_2 | 0.0555 |
| GO:0044260_1+GO:0044260_2 | 0.054919 |
| GO:0005634_1+GO:0005634_2 | 0.054281 |
| GO:0044422_1+GO:0044422_2 | 0.054181 |
| GO:0070013_1+GO:0070013_2 | 0.053569 |
| GO:0043233_1+GO:0043233_2 | 0.05275 |
| GO:0031974_1+GO:0031974_2 | 0.052093 |
| GO:0006139_1+GO:0006139_2 | 0.050846 |
| GO:0043170_1+GO:0043170_2 | 0.048856 |
| GO:0046483_1+GO:0046483_2 | 0.048379 |
| GO:0006725_1+GO:0006725_2 | 0.048024 |
| GO:1901360_1+GO:1901360_2 | 0.046085 |
| GO:0034641_1+GO:0034641_2 | 0.045622 |
| GO:0010467_1+GO:0010467_2 | 0.044175 |
| GO:0006807_1+GO:0006807_2 | 0.042125 |
| GO:0044238_1+GO:0044238_2 | 0.040562 |
| GO:0016070_1+GO:0016070_2 | 0.039323 |
| GO:0044237_1+GO:0044237_2 | 0.038156 |
| GO:0071704_1+GO:0071704_2 | 0.037669 |
| GO:0043228_1+GO:0043228_2 | 0.0367 |
| GO:0043232_1+GO:0043232_2 | 0.0367 |
| GO:0034645_1+GO:0034645_2 | 0.033018 |
| GO:0005654_1+GO:0005654_2 | 0.031929 |
| GO:0009059_1+GO:0009059_2 | 0.031798 |
| GO:0044424_1+GO:0044424_2 | 0.030566 |
| GO:0005622_1+GO:0005622_2 | 0.029698 |
| GO:0071840_1+GO:0071840_2 | 0.028654 |
| GO:1901576_1+GO:1901576_2 | 0.027293 |
| GO:0044249_1+GO:0044249_2 | 0.027029 |
| GO:0009058_1+GO:0009058_2 | 0.026245 |
| GO:0043234_1+GO:0043234_2 | 0.025652 |
| GO:0044822_1+GO:0044822_2 | 0.024607 |
| GO:0032774_1+GO:0032774_2 | 0.02436 |
| abs(GO:0044424_1-GO:0044424_2) | 0.02378 |
| abs(GO:0005488_1-GO:0005488_2) | 0.023486 |
| GO:0003723_1+GO:0003723_2 | 0.023347 |
| abs(GO:0005622_1-GO:0005622_2) | 0.023277 |
| GO:0034654_1+GO:0034654_2 | 0.022827 |
| abs(GO:0043229_1-GO:0043229_2) | 0.022297 |
| GO:0016043_1+GO:0016043_2 | 0.021982 |
| GO:0018130_1+GO:0018130_2 | 0.02198 |
| GO:0019438_1+GO:0019438_2 | 0.021899 |
| GO:0016071_1+GO:0016071_2 | 0.021748 |
| abs(GO:0043226_1-GO:0043226_2) | 0.021728 |
| GO:0044271_1+GO:0044271_2 | 0.021409 |
| GO:0005515_1+GO:0005515_2 | 0.021308 |
| GO:1901362_1+GO:1901362_2 | 0.02128 |
| GO:0044764_1+GO:0044764_2 | 0.021134 |
| GO:0016032_1+GO:0016032_2 | 0.020957 |
| abs(GO:0031224_1-GO:0031224_2) | 0.020882 |
| abs(GO:0044425_1-GO:0044425_2) | 0.020619 |
| GO:0044267_1+GO:0044267_2 | 0.020366 |
| GO:0044419_1+GO:0044419_2 | 0.020302 |
| GO:0044403_1+GO:0044403_2 | 0.020302 |
| abs(GO:0016021_1-GO:0016021_2) | 0.020263 |
| GO:0031224_1+GO:0031224_2 | 0.019879 |
| GO:0044451_1+GO:0044451_2 | 0.019238 |
| GO:0016021_1+GO:0016021_2 | 0.019068 |
| GO:0060255_1+GO:0060255_2 | 0.01883 |
| GO:0030529_1+GO:0030529_2 | 0.018718 |
| GO:0005730_1+GO:0005730_2 | 0.018701 |
| abs(GO:0044238_1-GO:0044238_2) | 0.018238 |
| abs(GO:0071704_1-GO:0071704_2) | 0.01802 |
| abs(GO:0008152_1-GO:0008152_2) | 0.017897 |
| GO:0006351_1+GO:0006351_2 | 0.017864 |
| abs(GO:0043227_1-GO:0043227_2) | 0.017846 |
| GO:0044265_1+GO:0044265_2 | 0.017782 |
| abs(GO:0044237_1-GO:0044237_2) | 0.017324 |
| GO:0019538_1+GO:0019538_2 | 0.017189 |
| GO:0010556_1+GO:0010556_2 | 0.017131 |
| GO:0080090_1+GO:0080090_2 | 0.016775 |
| GO:0044464_1+GO:0044464_2 | 0.016707 |
| GO:0005623_1+GO:0005623_2 | 0.016668 |
| GO:0019083_1+GO:0019083_2 | 0.016485 |
| GO:2000112_1+GO:2000112_2 | 0.016483 |
| GO:0003674_1+GO:0003674_2 | 0.016406 |
| GO:0010468_1+GO:0010468_2 | 0.016398 |
| GO:0031323_1+GO:0031323_2 | 0.016325 |
| GO:0031326_1+GO:0031326_2 | 0.016156 |
| abs(GO:0016020_1-GO:0016020_2) | 0.016122 |
| GO:0051171_1+GO:0051171_2 | 0.016058 |
| GO:0009889_1+GO:0009889_2 | 0.015979 |
| GO:0009057_1+GO:0009057_2 | 0.015832 |
| GO:0051252_1+GO:0051252_2 | 0.015828 |
| GO:0022613_1+GO:0022613_2 | 0.01573 |
| GO:0019219_1+GO:0019219_2 | 0.01546 |
| GO:0019222_1+GO:0019222_2 | 0.015354 |
| abs(GO:0043231_1-GO:0043231_2) | 0.015286 |
| GO:0005829_1+GO:0005829_2 | 0.01526 |
| GO:0000956_1+GO:0000956_2 | 0.01518 |
| abs(GO:0043170_1-GO:0043170_2) | 0.015171 |
| GO:0006996_1+GO:0006996_2 | 0.015056 |
| GO:0051276_1+GO:0051276_2 | 0.014898 |
| GO:0006402_1+GO:0006402_2 | 0.014888 |
| GO:0006401_1+GO:0006401_2 | 0.01465 |
| abs(GO:0003674_1-GO:0003674_2) | 0.014554 |
| GO:0009987_1+GO:0009987_2 | 0.014386 |
| GO:0006396_1+GO:0006396_2 | 0.014141 |
| abs(GO:0044464_1-GO:0044464_2) | 0.014054 |
| abs(GO:0005623_1-GO:0005623_2) | 0.014021 |
| abs(GO:0044260_1-GO:0044260_2) | 0.013959 |
| GO:0000184_1+GO:0000184_2 | 0.013523 |
| GO:0006413_1+GO:0006413_2 | 0.013368 |
| GO:0043933_1+GO:0043933_2 | 0.013231 |
| GO:0006415_1+GO:0006415_2 | 0.013086 |
| GO:0032984_1+GO:0032984_2 | 0.012737 |
| GO:0034655_1+GO:0034655_2 | 0.012733 |
| GO:0043241_1+GO:0043241_2 | 0.012671 |
| abs(GO:0044764_1-GO:0044764_2) | 0.012651 |
| GO:0006974_1+GO:0006974_2 | 0.012552 |
| abs(GO:0016032_1-GO:0016032_2) | 0.012518 |
| GO:0005694_1+GO:0005694_2 | 0.012444 |
| GO:0022626_1+GO:0022626_2 | 0.012358 |
| GO:0043624_1+GO:0043624_2 | 0.012053 |
| abs(GO:0044419_1-GO:0044419_2) | 0.01196 |
| abs(GO:0044403_1-GO:0044403_2) | 0.01196 |
| GO:0044270_1+GO:0044270_2 | 0.011906 |
| GO:0019080_1+GO:0019080_2 | 0.011895 |
| GO:0044033_1+GO:0044033_2 | 0.011846 |
| GO:0042254_1+GO:0042254_2 | 0.011788 |
| GO:0046700_1+GO:0046700_2 | 0.011722 |
| GO:0019439_1+GO:0019439_2 | 0.011572 |
| GO:0006414_1+GO:0006414_2 | 0.011532 |
| abs(GO:0009987_1-GO:0009987_2) | 0.011174 |
| GO:0044427_1+GO:0044427_2 | 0.011144 |
| GO:0006614_1+GO:0006614_2 | 0.011117 |
| GO:0022411_1+GO:0022411_2 | 0.011062 |
| GO:0044445_1+GO:0044445_2 | 0.01106 |
| GO:0072599_1+GO:0072599_2 | 0.011057 |
| GO:0045047_1+GO:0045047_2 | 0.011057 |
| GO:0006281_1+GO:0006281_2 | 0.011042 |
| GO:1901361_1+GO:1901361_2 | 0.010952 |
| GO:0006613_1+GO:0006613_2 | 0.010947 |
| abs(GO:0005730_1-GO:0005730_2) | 0.010825 |
| hsa03010_1+hsa03010_2 | 0.010759 |
| GO:0016604_1+GO:0016604_2 | 0.010695 |
| GO:0019058_1+GO:0019058_2 | 0.01059 |
| GO:1990234_1+GO:1990234_2 | 0.010428 |
| GO:0044391_1+GO:0044391_2 | 0.010414 |
| GO:0070972_1+GO:0070972_2 | 0.010392 |
| abs(GO:0044451_1-GO:0044451_2) | 0.010355 |
| GO:0006259_1+GO:0006259_2 | 0.010323 |
| GO:0008150_1+GO:0008150_2 | 0.010292 |
| GO:0006412_1+GO:0006412_2 | 0.010216 |
| GO:0044710_1+GO:0044710_2 | 0.010176 |
| abs(GO:0019083_1-GO:0019083_2) | 0.010166 |
| GO:0000375_1+GO:0000375_2 | 0.01016 |
| GO:0033554_1+GO:0033554_2 | 0.010076 |

1. Part of MaxRel feature list on *DS*_5_

| Feature | Score |
| --- | --- |
| GO:0044428_1+GO:0044428_2 | 0.060343 |
| GO:1901363_1+GO:1901363_2 | 0.059525 |
| GO:0097159_1+GO:0097159_2 | 0.058685 |
| GO:0031981_1+GO:0031981_2 | 0.057691 |
| GO:0032991_1+GO:0032991_2 | 0.057679 |
| GO:0003676_1+GO:0003676_2 | 0.057565 |
| GO:0090304_1+GO:0090304_2 | 0.055971 |
| GO:0044260_1+GO:0044260_2 | 0.053991 |
| GO:0044446_1+GO:0044446_2 | 0.053093 |
| GO:0005634_1+GO:0005634_2 | 0.05309 |
| GO:0044422_1+GO:0044422_2 | 0.051954 |
| GO:0070013_1+GO:0070013_2 | 0.051409 |
| GO:0043233_1+GO:0043233_2 | 0.050633 |
| GO:0031974_1+GO:0031974_2 | 0.050029 |
| GO:0006139_1+GO:0006139_2 | 0.049983 |
| GO:0043170_1+GO:0043170_2 | 0.048125 |
| GO:0046483_1+GO:0046483_2 | 0.047467 |
| GO:0006725_1+GO:0006725_2 | 0.047026 |
| GO:1901360_1+GO:1901360_2 | 0.045213 |
| GO:0034641_1+GO:0034641_2 | 0.044807 |
| GO:0010467_1+GO:0010467_2 | 0.043715 |
| GO:0006807_1+GO:0006807_2 | 0.041276 |
| GO:0044238_1+GO:0044238_2 | 0.039948 |
| GO:0016070_1+GO:0016070_2 | 0.038758 |
| GO:0044237_1+GO:0044237_2 | 0.037296 |
| GO:0071704_1+GO:0071704_2 | 0.036976 |
| GO:0043228_1+GO:0043228_2 | 0.03514 |
| GO:0043232_1+GO:0043232_2 | 0.03514 |
| GO:0034645_1+GO:0034645_2 | 0.032193 |
| GO:0009059_1+GO:0009059_2 | 0.031225 |
| GO:0005654_1+GO:0005654_2 | 0.030802 |
| GO:0044424_1+GO:0044424_2 | 0.029696 |
| GO:0005622_1+GO:0005622_2 | 0.028842 |
| GO:0071840_1+GO:0071840_2 | 0.027649 |
| GO:1901576_1+GO:1901576_2 | 0.026856 |
| GO:0044249_1+GO:0044249_2 | 0.026437 |
| GO:0009058_1+GO:0009058_2 | 0.025822 |
| GO:0043234_1+GO:0043234_2 | 0.024223 |
| GO:0032774_1+GO:0032774_2 | 0.023834 |
| GO:0044822_1+GO:0044822_2 | 0.023723 |
| abs(GO:0005488_1-GO:0005488_2) | 0.023067 |
| abs(GO:0044424_1-GO:0044424_2) | 0.022841 |
| GO:0003723_1+GO:0003723_2 | 0.022481 |
| abs(GO:0005622_1-GO:0005622_2) | 0.022339 |
| GO:0034654_1+GO:0034654_2 | 0.022305 |
| GO:0018130_1+GO:0018130_2 | 0.021442 |
| GO:0016071_1+GO:0016071_2 | 0.021389 |
| GO:0019438_1+GO:0019438_2 | 0.021378 |
| GO:0016043_1+GO:0016043_2 | 0.021019 |
| abs(GO:0043229_1-GO:0043229_2) | 0.020914 |
| GO:0044271_1+GO:0044271_2 | 0.020907 |
| GO:1901362_1+GO:1901362_2 | 0.020825 |
| abs(GO:0031224_1-GO:0031224_2) | 0.020665 |
| abs(GO:0044425_1-GO:0044425_2) | 0.020634 |
| GO:0005515_1+GO:0005515_2 | 0.020527 |
| abs(GO:0043226_1-GO:0043226_2) | 0.020463 |
| GO:0044267_1+GO:0044267_2 | 0.020288 |
| abs(GO:0016021_1-GO:0016021_2) | 0.020025 |
| GO:0044764_1+GO:0044764_2 | 0.019991 |
| GO:0016032_1+GO:0016032_2 | 0.019811 |
| GO:0031224_1+GO:0031224_2 | 0.01955 |
| GO:0044419_1+GO:0044419_2 | 0.019212 |
| GO:0044403_1+GO:0044403_2 | 0.019212 |
| GO:0060255_1+GO:0060255_2 | 0.018828 |
| GO:0016021_1+GO:0016021_2 | 0.018732 |
| GO:0044451_1+GO:0044451_2 | 0.018673 |
| GO:0030529_1+GO:0030529_2 | 0.018623 |
| abs(GO:0044238_1-GO:0044238_2) | 0.018452 |
| GO:0005730_1+GO:0005730_2 | 0.01823 |
| abs(GO:0071704_1-GO:0071704_2) | 0.018198 |
| abs(GO:0008152_1-GO:0008152_2) | 0.017787 |
| GO:0006351_1+GO:0006351_2 | 0.017368 |
| GO:0019538_1+GO:0019538_2 | 0.017193 |
| abs(GO:0044237_1-GO:0044237_2) | 0.01701 |
| abs(GO:0043227_1-GO:0043227_2) | 0.016968 |
| GO:0044265_1+GO:0044265_2 | 0.016912 |
| GO:0010556_1+GO:0010556_2 | 0.016846 |
| GO:0080090_1+GO:0080090_2 | 0.016739 |
| GO:0010468_1+GO:0010468_2 | 0.016284 |
| GO:0031323_1+GO:0031323_2 | 0.016183 |
| GO:0044464_1+GO:0044464_2 | 0.016128 |
| GO:2000112_1+GO:2000112_2 | 0.016108 |
| GO:0005623_1+GO:0005623_2 | 0.016104 |
| abs(GO:0016020_1-GO:0016020_2) | 0.016027 |
| GO:0003674_1+GO:0003674_2 | 0.015962 |
| GO:0019083_1+GO:0019083_2 | 0.015941 |
| GO:0031326_1+GO:0031326_2 | 0.015889 |
| GO:0051171_1+GO:0051171_2 | 0.015882 |
| GO:0009889_1+GO:0009889_2 | 0.015714 |
| GO:0022613_1+GO:0022613_2 | 0.015636 |
| GO:0051252_1+GO:0051252_2 | 0.015512 |
| abs(GO:0043170_1-GO:0043170_2) | 0.015421 |
| GO:0019219_1+GO:0019219_2 | 0.015229 |
| GO:0019222_1+GO:0019222_2 | 0.01521 |
| GO:0009057_1+GO:0009057_2 | 0.014977 |
| GO:0051276_1+GO:0051276_2 | 0.014877 |
| GO:0000956_1+GO:0000956_2 | 0.014767 |
| GO:0005829_1+GO:0005829_2 | 0.014613 |
| GO:0006402_1+GO:0006402_2 | 0.014459 |
| GO:0006996_1+GO:0006996_2 | 0.014349 |
| abs(GO:0043231_1-GO:0043231_2) | 0.014238 |
| abs(GO:0003674_1-GO:0003674_2) | 0.014146 |
| GO:0006401_1+GO:0006401_2 | 0.014134 |
| abs(GO:0044260_1-GO:0044260_2) | 0.013929 |
| GO:0006396_1+GO:0006396_2 | 0.01391 |
| GO:0009987_1+GO:0009987_2 | 0.013835 |
| abs(GO:0044464_1-GO:0044464_2) | 0.013473 |
| abs(GO:0005623_1-GO:0005623_2) | 0.013456 |
| GO:0006413_1+GO:0006413_2 | 0.013131 |
| GO:0000184_1+GO:0000184_2 | 0.013121 |
| GO:0043933_1+GO:0043933_2 | 0.013004 |
| GO:0006415_1+GO:0006415_2 | 0.01288 |
| GO:0005694_1+GO:0005694_2 | 0.012464 |
| GO:0034655_1+GO:0034655_2 | 0.01233 |
| GO:0032984_1+GO:0032984_2 | 0.012299 |
| GO:0043241_1+GO:0043241_2 | 0.012257 |
| GO:0006974_1+GO:0006974_2 | 0.012198 |
| GO:0022626_1+GO:0022626_2 | 0.01202 |
| abs(GO:0044764_1-GO:0044764_2) | 0.011827 |
| abs(GO:0016032_1-GO:0016032_2) | 0.011694 |
| GO:0043624_1+GO:0043624_2 | 0.011638 |
| GO:0042254_1+GO:0042254_2 | 0.011572 |
| GO:0019080_1+GO:0019080_2 | 0.011554 |
| GO:0044033_1+GO:0044033_2 | 0.011513 |
| GO:0044270_1+GO:0044270_2 | 0.011463 |
| GO:0046700_1+GO:0046700_2 | 0.011308 |
| GO:0006414_1+GO:0006414_2 | 0.011194 |
| abs(GO:0044419_1-GO:0044419_2) | 0.011162 |
| abs(GO:0044403_1-GO:0044403_2) | 0.011162 |
| GO:0019439_1+GO:0019439_2 | 0.0111 |
| GO:0044427_1+GO:0044427_2 | 0.011045 |
| GO:0006614_1+GO:0006614_2 | 0.010957 |
| GO:0045047_1+GO:0045047_2 | 0.010885 |
| GO:0072599_1+GO:0072599_2 | 0.010885 |
| GO:0006613_1+GO:0006613_2 | 0.010735 |
| GO:0006281_1+GO:0006281_2 | 0.010721 |
| GO:0044445_1+GO:0044445_2 | 0.01071 |
| abs(GO:0009987_1-GO:0009987_2) | 0.010658 |
| GO:0022411_1+GO:0022411_2 | 0.010582 |
| GO:1901361_1+GO:1901361_2 | 0.010558 |
| abs(GO:0005730_1-GO:0005730_2) | 0.010527 |
| hsa03010_1+hsa03010_2 | 0.010497 |
| GO:0016604_1+GO:0016604_2 | 0.010487 |
| GO:0070972_1+GO:0070972_2 | 0.01039 |
| GO:1990234_1+GO:1990234_2 | 0.010262 |
| GO:0006259_1+GO:0006259_2 | 0.010202 |
| GO:0019058_1+GO:0019058_2 | 0.010178 |
| GO:0044391_1+GO:0044391_2 | 0.010172 |
| GO:0008150_1+GO:0008150_2 | 0.010021 |

1. Part of MaxRel feature list on *DS*_6_

| **Feature** | **Score** |
| --- | --- |
| GO:0044428_1+GO:0044428_2 | 0.060678 |
| GO:1901363_1+GO:1901363_2 | 0.058768 |
| GO:0032991_1+GO:0032991_2 | 0.058531 |
| GO:0097159_1+GO:0097159_2 | 0.057905 |
| GO:0031981_1+GO:0031981_2 | 0.057613 |
| GO:0003676_1+GO:0003676_2 | 0.056813 |
| GO:0090304_1+GO:0090304_2 | 0.056013 |
| GO:0044446_1+GO:0044446_2 | 0.053961 |
| GO:0044260_1+GO:0044260_2 | 0.053744 |
| GO:0005634_1+GO:0005634_2 | 0.053466 |
| GO:0044422_1+GO:0044422_2 | 0.052736 |
| GO:0070013_1+GO:0070013_2 | 0.051646 |
| GO:0043233_1+GO:0043233_2 | 0.050794 |
| GO:0031974_1+GO:0031974_2 | 0.050168 |
| GO:0006139_1+GO:0006139_2 | 0.04954 |
| GO:0043170_1+GO:0043170_2 | 0.047826 |
| GO:0046483_1+GO:0046483_2 | 0.047132 |
| GO:0006725_1+GO:0006725_2 | 0.046787 |
| GO:1901360_1+GO:1901360_2 | 0.044887 |
| GO:0034641_1+GO:0034641_2 | 0.044463 |
| GO:0010467_1+GO:0010467_2 | 0.043381 |
| GO:0006807_1+GO:0006807_2 | 0.041137 |
| GO:0044238_1+GO:0044238_2 | 0.039693 |
| GO:0016070_1+GO:0016070_2 | 0.038491 |
| GO:0044237_1+GO:0044237_2 | 0.037011 |
| GO:0071704_1+GO:0071704_2 | 0.036749 |
| GO:0043232_1+GO:0043232_2 | 0.035478 |
| GO:0043228_1+GO:0043228_2 | 0.035478 |
| GO:0034645_1+GO:0034645_2 | 0.032495 |
| GO:0009059_1+GO:0009059_2 | 0.03147 |
| GO:0005654_1+GO:0005654_2 | 0.031336 |
| GO:0044424_1+GO:0044424_2 | 0.029338 |
| GO:0005622_1+GO:0005622_2 | 0.02855 |
| GO:0071840_1+GO:0071840_2 | 0.027442 |
| GO:1901576_1+GO:1901576_2 | 0.026877 |
| GO:0044249_1+GO:0044249_2 | 0.026712 |
| GO:0009058_1+GO:0009058_2 | 0.025901 |
| GO:0043234_1+GO:0043234_2 | 0.025077 |
| GO:0032774_1+GO:0032774_2 | 0.02375 |
| GO:0044822_1+GO:0044822_2 | 0.023418 |
| abs(GO:0005488_1-GO:0005488_2) | 0.023035 |
| abs(GO:0044424_1-GO:0044424_2) | 0.022664 |
| GO:0034654_1+GO:0034654_2 | 0.022283 |
| abs(GO:0005622_1-GO:0005622_2) | 0.022241 |
| GO:0003723_1+GO:0003723_2 | 0.022201 |
| GO:0018130_1+GO:0018130_2 | 0.021529 |
| GO:0019438_1+GO:0019438_2 | 0.021472 |
| GO:0016071_1+GO:0016071_2 | 0.02138 |
| abs(GO:0043229_1-GO:0043229_2) | 0.021263 |
| GO:0044271_1+GO:0044271_2 | 0.020975 |
| GO:1901362_1+GO:1901362_2 | 0.020911 |
| GO:0016043_1+GO:0016043_2 | 0.020888 |
| abs(GO:0043226_1-GO:0043226_2) | 0.020815 |
| abs(GO:0031224_1-GO:0031224_2) | 0.020634 |
| abs(GO:0044425_1-GO:0044425_2) | 0.020401 |
| GO:0005515_1+GO:0005515_2 | 0.020257 |
| GO:0044764_1+GO:0044764_2 | 0.02011 |
| abs(GO:0016021_1-GO:0016021_2) | 0.019988 |
| GO:0016032_1+GO:0016032_2 | 0.019957 |
| GO:0044267_1+GO:0044267_2 | 0.019881 |
| GO:0031224_1+GO:0031224_2 | 0.019668 |
| GO:0044419_1+GO:0044419_2 | 0.019336 |
| GO:0044403_1+GO:0044403_2 | 0.019336 |
| GO:0044451_1+GO:0044451_2 | 0.019018 |
| GO:0016021_1+GO:0016021_2 | 0.018853 |
| GO:0060255_1+GO:0060255_2 | 0.018585 |
| abs(GO:0044238_1-GO:0044238_2) | 0.018543 |
| GO:0005730_1+GO:0005730_2 | 0.0184 |
| GO:0030529_1+GO:0030529_2 | 0.018333 |
| abs(GO:0071704_1-GO:0071704_2) | 0.018225 |
| abs(GO:0008152_1-GO:0008152_2) | 0.017769 |
| abs(GO:0043227_1-GO:0043227_2) | 0.017331 |
| GO:0006351_1+GO:0006351_2 | 0.017273 |
| abs(GO:0044237_1-GO:0044237_2) | 0.017189 |
| GO:0044265_1+GO:0044265_2 | 0.017091 |
| GO:0010556_1+GO:0010556_2 | 0.016704 |
| GO:0019538_1+GO:0019538_2 | 0.016665 |
| GO:0080090_1+GO:0080090_2 | 0.016512 |
| GO:0010468_1+GO:0010468_2 | 0.016234 |
| GO:0031323_1+GO:0031323_2 | 0.016114 |
| abs(GO:0016020_1-GO:0016020_2) | 0.016102 |
| GO:2000112_1+GO:2000112_2 | 0.016041 |
| GO:0019083_1+GO:0019083_2 | 0.015954 |
| GO:0044464_1+GO:0044464_2 | 0.015783 |
| GO:0003674_1+GO:0003674_2 | 0.015778 |
| GO:0005623_1+GO:0005623_2 | 0.015742 |
| GO:0031326_1+GO:0031326_2 | 0.015721 |
| GO:0051171_1+GO:0051171_2 | 0.015549 |
| GO:0009889_1+GO:0009889_2 | 0.015546 |
| abs(GO:0043170_1-GO:0043170_2) | 0.015523 |
| GO:0051252_1+GO:0051252_2 | 0.015445 |
| GO:0022613_1+GO:0022613_2 | 0.015442 |
| GO:0019222_1+GO:0019222_2 | 0.015227 |
| GO:0009057_1+GO:0009057_2 | 0.015102 |
| GO:0019219_1+GO:0019219_2 | 0.014951 |
| GO:0005829_1+GO:0005829_2 | 0.014918 |
| GO:0000956_1+GO:0000956_2 | 0.014786 |
| abs(GO:0043231_1-GO:0043231_2) | 0.014706 |
| GO:0006402_1+GO:0006402_2 | 0.014482 |
| GO:0051276_1+GO:0051276_2 | 0.014466 |
| GO:0006996_1+GO:0006996_2 | 0.014443 |
| abs(GO:0044260_1-GO:0044260_2) | 0.014222 |
| GO:0006401_1+GO:0006401_2 | 0.014205 |
| abs(GO:0003674_1-GO:0003674_2) | 0.013959 |
| GO:0009987_1+GO:0009987_2 | 0.013785 |
| GO:0006396_1+GO:0006396_2 | 0.013758 |
| GO:0000184_1+GO:0000184_2 | 0.013269 |
| abs(GO:0044464_1-GO:0044464_2) | 0.013246 |
| abs(GO:0005623_1-GO:0005623_2) | 0.013221 |
| GO:0006413_1+GO:0006413_2 | 0.013059 |
| GO:0006415_1+GO:0006415_2 | 0.013046 |
| GO:0043933_1+GO:0043933_2 | 0.01303 |
| GO:0032984_1+GO:0032984_2 | 0.012358 |
| GO:0043241_1+GO:0043241_2 | 0.012343 |
| GO:0006974_1+GO:0006974_2 | 0.012336 |
| GO:0022626_1+GO:0022626_2 | 0.01209 |
| GO:0005694_1+GO:0005694_2 | 0.012084 |
| abs(GO:0044764_1-GO:0044764_2) | 0.01194 |
| GO:0034655_1+GO:0034655_2 | 0.011885 |
| GO:0043624_1+GO:0043624_2 | 0.011846 |
| abs(GO:0016032_1-GO:0016032_2) | 0.011828 |
| GO:0019080_1+GO:0019080_2 | 0.011745 |
| GO:0044033_1+GO:0044033_2 | 0.011699 |
| GO:0042254_1+GO:0042254_2 | 0.011574 |
| GO:0006414_1+GO:0006414_2 | 0.011324 |
| abs(GO:0044419_1-GO:0044419_2) | 0.011271 |
| abs(GO:0044403_1-GO:0044403_2) | 0.011271 |
| GO:0044270_1+GO:0044270_2 | 0.010997 |
| GO:0006614_1+GO:0006614_2 | 0.010942 |
| GO:0006281_1+GO:0006281_2 | 0.010896 |
| GO:0046700_1+GO:0046700_2 | 0.010865 |
| GO:0045047_1+GO:0045047_2 | 0.010838 |
| GO:0072599_1+GO:0072599_2 | 0.010838 |
| GO:0022411_1+GO:0022411_2 | 0.010835 |
| abs(GO:0009987_1-GO:0009987_2) | 0.0108 |
| GO:0044427_1+GO:0044427_2 | 0.010778 |
| GO:0019439_1+GO:0019439_2 | 0.010726 |
| abs(GO:0005730_1-GO:0005730_2) | 0.010691 |
| GO:0006613_1+GO:0006613_2 | 0.010674 |
| GO:0016604_1+GO:0016604_2 | 0.01067 |
| GO:1990234_1+GO:1990234_2 | 0.010636 |
| GO:0019058_1+GO:0019058_2 | 0.010625 |
| GO:0044445_1+GO:0044445_2 | 0.010579 |
| hsa03010_1+hsa03010_2 | 0.010492 |
| GO:0070972_1+GO:0070972_2 | 0.010235 |
| abs(GO:0044451_1-GO:0044451_2) | 0.010179 |
| GO:1901361_1+GO:1901361_2 | 0.010125 |
| GO:0006259_1+GO:0006259_2 | 0.010056 |
| GO:0000375_1+GO:0000375_2 | 0.010043 |
| GO:0044391_1+GO:0044391_2 | 0.010002 |

1. Part of MaxRel feature list on *DS*_7_

| **Feature** | **Score** |
| --- | --- |
| GO:0044428_1+GO:0044428_2 | 0.061804 |
| GO:1901363_1+GO:1901363_2 | 0.059953 |
| GO:0031981_1+GO:0031981_2 | 0.059511 |
| GO:0032991_1+GO:0032991_2 | 0.059452 |
| GO:0097159_1+GO:0097159_2 | 0.059178 |
| GO:0003676_1+GO:0003676_2 | 0.058528 |
| GO:0090304_1+GO:0090304_2 | 0.057529 |
| GO:0044260_1+GO:0044260_2 | 0.054847 |
| GO:0005634_1+GO:0005634_2 | 0.05462 |
| GO:0044446_1+GO:0044446_2 | 0.054223 |
| GO:0044422_1+GO:0044422_2 | 0.053251 |
| GO:0070013_1+GO:0070013_2 | 0.053151 |
| GO:0043233_1+GO:0043233_2 | 0.052368 |
| GO:0031974_1+GO:0031974_2 | 0.051517 |
| GO:0006139_1+GO:0006139_2 | 0.050882 |
| GO:0043170_1+GO:0043170_2 | 0.048765 |
| GO:0046483_1+GO:0046483_2 | 0.048397 |
| GO:0006725_1+GO:0006725_2 | 0.04788 |
| GO:1901360_1+GO:1901360_2 | 0.045871 |
| GO:0034641_1+GO:0034641_2 | 0.045614 |
| GO:0010467_1+GO:0010467_2 | 0.044573 |
| GO:0006807_1+GO:0006807_2 | 0.041896 |
| GO:0044238_1+GO:0044238_2 | 0.040287 |
| GO:0016070_1+GO:0016070_2 | 0.039927 |
| GO:0044237_1+GO:0044237_2 | 0.037592 |
| GO:0071704_1+GO:0071704_2 | 0.037209 |
| GO:0043232_1+GO:0043232_2 | 0.036031 |
| GO:0043228_1+GO:0043228_2 | 0.036031 |
| GO:0034645_1+GO:0034645_2 | 0.033473 |
| GO:0009059_1+GO:0009059_2 | 0.032166 |
| GO:0005654_1+GO:0005654_2 | 0.03144 |
| GO:0044424_1+GO:0044424_2 | 0.030026 |
| GO:0005622_1+GO:0005622_2 | 0.029215 |
| GO:0071840_1+GO:0071840_2 | 0.028378 |
| GO:1901576_1+GO:1901576_2 | 0.027071 |
| GO:0044249_1+GO:0044249_2 | 0.026924 |
| GO:0009058_1+GO:0009058_2 | 0.026031 |
| GO:0043234_1+GO:0043234_2 | 0.025243 |
| GO:0032774_1+GO:0032774_2 | 0.024606 |
| GO:0044822_1+GO:0044822_2 | 0.024265 |
| abs(GO:0005488_1-GO:0005488_2) | 0.023425 |
| abs(GO:0044424_1-GO:0044424_2) | 0.023241 |
| GO:0034654_1+GO:0034654_2 | 0.02314 |
| GO:0003723_1+GO:0003723_2 | 0.022964 |
| abs(GO:0005622_1-GO:0005622_2) | 0.02284 |
| GO:0018130_1+GO:0018130_2 | 0.022272 |
| GO:0019438_1+GO:0019438_2 | 0.022155 |
| GO:0016071_1+GO:0016071_2 | 0.02168 |
| abs(GO:0043229_1-GO:0043229_2) | 0.02166 |
| GO:0044271_1+GO:0044271_2 | 0.021631 |
| GO:0016043_1+GO:0016043_2 | 0.021567 |
| GO:1901362_1+GO:1901362_2 | 0.021454 |
| abs(GO:0043226_1-GO:0043226_2) | 0.021311 |
| abs(GO:0031224_1-GO:0031224_2) | 0.021219 |
| GO:0005515_1+GO:0005515_2 | 0.021202 |
| abs(GO:0044425_1-GO:0044425_2) | 0.020969 |
| GO:0044764_1+GO:0044764_2 | 0.020799 |
| GO:0016032_1+GO:0016032_2 | 0.020608 |
| abs(GO:0016021_1-GO:0016021_2) | 0.020532 |
| GO:0044267_1+GO:0044267_2 | 0.020341 |
| GO:0031224_1+GO:0031224_2 | 0.019995 |
| GO:0044403_1+GO:0044403_2 | 0.019984 |
| GO:0044419_1+GO:0044419_2 | 0.019984 |
| GO:0060255_1+GO:0060255_2 | 0.019173 |
| GO:0044451_1+GO:0044451_2 | 0.019155 |
| GO:0016021_1+GO:0016021_2 | 0.019151 |
| GO:0030529_1+GO:0030529_2 | 0.018884 |
| GO:0005730_1+GO:0005730_2 | 0.01871 |
| abs(GO:0044238_1-GO:0044238_2) | 0.018661 |
| abs(GO:0071704_1-GO:0071704_2) | 0.01833 |
| GO:0006351_1+GO:0006351_2 | 0.018124 |
| abs(GO:0008152_1-GO:0008152_2) | 0.017971 |
| abs(GO:0043227_1-GO:0043227_2) | 0.017556 |
| GO:0044265_1+GO:0044265_2 | 0.017535 |
| GO:0010556_1+GO:0010556_2 | 0.017375 |
| abs(GO:0044237_1-GO:0044237_2) | 0.017292 |
| GO:0019538_1+GO:0019538_2 | 0.017211 |
| GO:0080090_1+GO:0080090_2 | 0.017051 |
| GO:2000112_1+GO:2000112_2 | 0.016824 |
| GO:0010468_1+GO:0010468_2 | 0.016737 |
| GO:0031323_1+GO:0031323_2 | 0.01667 |
| GO:0019083_1+GO:0019083_2 | 0.016624 |
| GO:0051171_1+GO:0051171_2 | 0.016372 |
| GO:0044464_1+GO:0044464_2 | 0.01632 |
| GO:0031326_1+GO:0031326_2 | 0.016315 |
| GO:0005623_1+GO:0005623_2 | 0.016283 |
| GO:0003674_1+GO:0003674_2 | 0.016256 |
| GO:0022613_1+GO:0022613_2 | 0.016208 |
| abs(GO:0016020_1-GO:0016020_2) | 0.016175 |
| GO:0009889_1+GO:0009889_2 | 0.016075 |
| GO:0019219_1+GO:0019219_2 | 0.015837 |
| abs(GO:0043170_1-GO:0043170_2) | 0.015607 |
| GO:0019222_1+GO:0019222_2 | 0.01556 |
| GO:0009057_1+GO:0009057_2 | 0.015388 |
| GO:0005829_1+GO:0005829_2 | 0.015099 |
| GO:0000956_1+GO:0000956_2 | 0.015055 |
| GO:0051276_1+GO:0051276_2 | 0.014929 |
| abs(GO:0043231_1-GO:0043231_2) | 0.014822 |
| GO:0006996_1+GO:0006996_2 | 0.014792 |
| GO:0006402_1+GO:0006402_2 | 0.014714 |
| GO:0006401_1+GO:0006401_2 | 0.014501 |
| abs(GO:0044260_1-GO:0044260_2) | 0.014451 |
| abs(GO:0003674_1-GO:0003674_2) | 0.014438 |
| GO:0006396_1+GO:0006396_2 | 0.014143 |
| GO:0009987_1+GO:0009987_2 | 0.013765 |
| abs(GO:0044464_1-GO:0044464_2) | 0.013735 |
| GO:0000184_1+GO:0000184_2 | 0.013718 |
| abs(GO:0005623_1-GO:0005623_2) | 0.01371 |
| GO:0043933_1+GO:0043933_2 | 0.013586 |
| GO:0006413_1+GO:0006413_2 | 0.013523 |
| GO:0006415_1+GO:0006415_2 | 0.013351 |
| GO:0032984_1+GO:0032984_2 | 0.01277 |
| GO:0043241_1+GO:0043241_2 | 0.012681 |
| GO:0005694_1+GO:0005694_2 | 0.012545 |
| GO:0006974_1+GO:0006974_2 | 0.012406 |
| GO:0034655_1+GO:0034655_2 | 0.012378 |
| abs(GO:0044764_1-GO:0044764_2) | 0.012336 |
| GO:0022626_1+GO:0022626_2 | 0.012311 |
| abs(GO:0016032_1-GO:0016032_2) | 0.012193 |
| GO:0043624_1+GO:0043624_2 | 0.012125 |
| GO:0042254_1+GO:0042254_2 | 0.012025 |
| GO:0019080_1+GO:0019080_2 | 0.011971 |
| GO:0044033_1+GO:0044033_2 | 0.011952 |
| abs(GO:0044419_1-GO:0044419_2) | 0.011626 |
| abs(GO:0044403_1-GO:0044403_2) | 0.011626 |
| GO:0044270_1+GO:0044270_2 | 0.011457 |
| GO:0006414_1+GO:0006414_2 | 0.011447 |
| GO:0046700_1+GO:0046700_2 | 0.011323 |
| GO:0044427_1+GO:0044427_2 | 0.011316 |
| GO:0006614_1+GO:0006614_2 | 0.011179 |
| GO:0019439_1+GO:0019439_2 | 0.011124 |
| GO:0045047_1+GO:0045047_2 | 0.01108 |
| GO:0072599_1+GO:0072599_2 | 0.01108 |
| GO:0044445_1+GO:0044445_2 | 0.010966 |
| GO:0022411_1+GO:0022411_2 | 0.010966 |
| GO:0006281_1+GO:0006281_2 | 0.010957 |
| GO:0006613_1+GO:0006613_2 | 0.010924 |
| hsa03010_1+hsa03010_2 | 0.010879 |
| abs(GO:0005730_1-GO:0005730_2) | 0.010869 |
| abs(GO:0009987_1-GO:0009987_2) | 0.010748 |
| GO:0019058_1+GO:0019058_2 | 0.010674 |
| GO:0016604_1+GO:0016604_2 | 0.010636 |
| GO:1990234_1+GO:1990234_2 | 0.010569 |
| GO:1901361_1+GO:1901361_2 | 0.010546 |
| GO:0044391_1+GO:0044391_2 | 0.010445 |
| GO:0070972_1+GO:0070972_2 | 0.010435 |
| GO:0006259_1+GO:0006259_2 | 0.010333 |
| abs(GO:0019083_1-GO:0019083_2) | 0.010264 |
| abs(GO:0022613_1-GO:0022613_2) | 0.010263 |
| GO:0033554_1+GO:0033554_2 | 0.010231 |
| abs(GO:0044451_1-GO:0044451_2) | 0.010206 |
| abs(GO:0005634_1-GO:0005634_2) | 0.010105 |
| GO:0000375_1+GO:0000375_2 | 0.010095 |
| GO:0006412_1+GO:0006412_2 | 0.010081 |
| GO:0008150_1+GO:0008150_2 | 0.010023 |

1. Part of MaxRel feature list on *DS*_8_

| **Feature** | **Score** |
| --- | --- |
| GO:0044428_1+GO:0044428_2 | 0.062018 |
| GO:0032991_1+GO:0032991_2 | 0.059717 |
| GO:1901363_1+GO:1901363_2 | 0.059688 |
| GO:0031981_1+GO:0031981_2 | 0.059125 |
| GO:0097159_1+GO:0097159_2 | 0.058828 |
| GO:0003676_1+GO:0003676_2 | 0.058332 |
| GO:0090304_1+GO:0090304_2 | 0.057143 |
| GO:0044260_1+GO:0044260_2 | 0.05492 |
| GO:0044446_1+GO:0044446_2 | 0.054886 |
| GO:0005634_1+GO:0005634_2 | 0.053697 |
| GO:0044422_1+GO:0044422_2 | 0.053652 |
| GO:0070013_1+GO:0070013_2 | 0.053217 |
| GO:0043233_1+GO:0043233_2 | 0.052437 |
| GO:0031974_1+GO:0031974_2 | 0.051724 |
| GO:0006139_1+GO:0006139_2 | 0.051165 |
| GO:0043170_1+GO:0043170_2 | 0.048825 |
| GO:0046483_1+GO:0046483_2 | 0.048573 |
| GO:0006725_1+GO:0006725_2 | 0.048149 |
| GO:1901360_1+GO:1901360_2 | 0.046075 |
| GO:0034641_1+GO:0034641_2 | 0.045882 |
| GO:0010467_1+GO:0010467_2 | 0.044506 |
| GO:0006807_1+GO:0006807_2 | 0.042475 |
| GO:0044238_1+GO:0044238_2 | 0.040604 |
| GO:0016070_1+GO:0016070_2 | 0.039921 |
| GO:0044237_1+GO:0044237_2 | 0.038246 |
| GO:0071704_1+GO:0071704_2 | 0.037599 |
| GO:0043232_1+GO:0043232_2 | 0.03643 |
| GO:0043228_1+GO:0043228_2 | 0.03643 |
| GO:0034645_1+GO:0034645_2 | 0.033178 |
| GO:0009059_1+GO:0009059_2 | 0.032033 |
| GO:0005654_1+GO:0005654_2 | 0.031501 |
| GO:0044424_1+GO:0044424_2 | 0.030613 |
| GO:0005622_1+GO:0005622_2 | 0.029754 |
| GO:0071840_1+GO:0071840_2 | 0.028251 |
| GO:1901576_1+GO:1901576_2 | 0.027139 |
| GO:0044249_1+GO:0044249_2 | 0.026905 |
| GO:0009058_1+GO:0009058_2 | 0.026144 |
| GO:0043234_1+GO:0043234_2 | 0.025649 |
| GO:0032774_1+GO:0032774_2 | 0.024329 |
| abs(GO:0044424_1-GO:0044424_2) | 0.023792 |
| abs(GO:0005488_1-GO:0005488_2) | 0.023771 |
| GO:0044822_1+GO:0044822_2 | 0.023693 |
| abs(GO:0005622_1-GO:0005622_2) | 0.023308 |
| GO:0034654_1+GO:0034654_2 | 0.022844 |
| GO:0003723_1+GO:0003723_2 | 0.02276 |
| GO:0018130_1+GO:0018130_2 | 0.021937 |
| GO:0019438_1+GO:0019438_2 | 0.021897 |
| abs(GO:0043229_1-GO:0043229_2) | 0.021694 |
| GO:0005515_1+GO:0005515_2 | 0.021512 |
| abs(GO:0043226_1-GO:0043226_2) | 0.021429 |
| GO:0016043_1+GO:0016043_2 | 0.021412 |
| GO:0044271_1+GO:0044271_2 | 0.021376 |
| GO:1901362_1+GO:1901362_2 | 0.021273 |
| GO:0016071_1+GO:0016071_2 | 0.021147 |
| abs(GO:0031224_1-GO:0031224_2) | 0.021041 |
| GO:0044267_1+GO:0044267_2 | 0.021003 |
| abs(GO:0044425_1-GO:0044425_2) | 0.020943 |
| GO:0044764_1+GO:0044764_2 | 0.020759 |
| GO:0016032_1+GO:0016032_2 | 0.020593 |
| abs(GO:0016021_1-GO:0016021_2) | 0.020317 |
| GO:0044403_1+GO:0044403_2 | 0.019984 |
| GO:0044419_1+GO:0044419_2 | 0.019984 |
| GO:0031224_1+GO:0031224_2 | 0.019698 |
| GO:0060255_1+GO:0060255_2 | 0.019236 |
| GO:0044451_1+GO:0044451_2 | 0.019093 |
| GO:0005730_1+GO:0005730_2 | 0.018828 |
| GO:0016021_1+GO:0016021_2 | 0.018824 |
| GO:0030529_1+GO:0030529_2 | 0.018812 |
| abs(GO:0044238_1-GO:0044238_2) | 0.01869 |
| abs(GO:0071704_1-GO:0071704_2) | 0.0183 |
| abs(GO:0008152_1-GO:0008152_2) | 0.018048 |
| GO:0006351_1+GO:0006351_2 | 0.017942 |
| GO:0019538_1+GO:0019538_2 | 0.017702 |
| abs(GO:0043227_1-GO:0043227_2) | 0.017669 |
| abs(GO:0044237_1-GO:0044237_2) | 0.017525 |
| GO:0044265_1+GO:0044265_2 | 0.017446 |
| GO:0010556_1+GO:0010556_2 | 0.017358 |
| GO:0080090_1+GO:0080090_2 | 0.017056 |
| GO:0010468_1+GO:0010468_2 | 0.016807 |
| GO:0044464_1+GO:0044464_2 | 0.0168 |
| GO:0005623_1+GO:0005623_2 | 0.016773 |
| GO:2000112_1+GO:2000112_2 | 0.016766 |
| GO:0031323_1+GO:0031323_2 | 0.016692 |
| GO:0051171_1+GO:0051171_2 | 0.016422 |
| GO:0003674_1+GO:0003674_2 | 0.016414 |
| abs(GO:0016020_1-GO:0016020_2) | 0.01639 |
| GO:0031326_1+GO:0031326_2 | 0.016269 |
| GO:0009889_1+GO:0009889_2 | 0.016083 |
| GO:0022613_1+GO:0022613_2 | 0.016008 |
| GO:0019222_1+GO:0019222_2 | 0.015832 |
| GO:0019219_1+GO:0019219_2 | 0.015801 |
| GO:0019083_1+GO:0019083_2 | 0.01575 |
| GO:0009057_1+GO:0009057_2 | 0.015675 |
| abs(GO:0043170_1-GO:0043170_2) | 0.015568 |
| GO:0005829_1+GO:0005829_2 | 0.015413 |
| abs(GO:0043231_1-GO:0043231_2) | 0.014847 |
| GO:0006996_1+GO:0006996_2 | 0.014785 |
| GO:0051276_1+GO:0051276_2 | 0.014642 |
| abs(GO:0003674_1-GO:0003674_2) | 0.014593 |
| GO:0000956_1+GO:0000956_2 | 0.014589 |
| abs(GO:0044260_1-GO:0044260_2) | 0.014429 |
| GO:0009987_1+GO:0009987_2 | 0.014391 |
| GO:0006402_1+GO:0006402_2 | 0.014219 |
| abs(GO:0044464_1-GO:0044464_2) | 0.014215 |
| abs(GO:0005623_1-GO:0005623_2) | 0.0142 |
| GO:0006396_1+GO:0006396_2 | 0.014133 |
| GO:0006401_1+GO:0006401_2 | 0.013837 |
| GO:0043933_1+GO:0043933_2 | 0.013246 |
| GO:0006413_1+GO:0006413_2 | 0.013143 |
| GO:0000184_1+GO:0000184_2 | 0.013046 |
| GO:0006415_1+GO:0006415_2 | 0.01274 |
| GO:0032984_1+GO:0032984_2 | 0.012504 |
| GO:0043241_1+GO:0043241_2 | 0.012416 |
| GO:0005694_1+GO:0005694_2 | 0.01241 |
| abs(GO:0044764_1-GO:0044764_2) | 0.012385 |
| GO:0006974_1+GO:0006974_2 | 0.012356 |
| GO:0034655_1+GO:0034655_2 | 0.012293 |
| abs(GO:0016032_1-GO:0016032_2) | 0.012257 |
| GO:0043624_1+GO:0043624_2 | 0.011893 |
| GO:0022626_1+GO:0022626_2 | 0.011828 |
| GO:0042254_1+GO:0042254_2 | 0.011788 |
| abs(GO:0044403_1-GO:0044403_2) | 0.011714 |
| abs(GO:0044419_1-GO:0044419_2) | 0.011714 |
| GO:0044270_1+GO:0044270_2 | 0.011459 |
| GO:0019080_1+GO:0019080_2 | 0.011363 |
| GO:0044033_1+GO:0044033_2 | 0.011356 |
| GO:0046700_1+GO:0046700_2 | 0.011331 |
| abs(GO:0009987_1-GO:0009987_2) | 0.011321 |
| GO:0019439_1+GO:0019439_2 | 0.011123 |
| GO:0044427_1+GO:0044427_2 | 0.011052 |
| GO:0016604_1+GO:0016604_2 | 0.010976 |
| GO:0006414_1+GO:0006414_2 | 0.010975 |
| GO:0006281_1+GO:0006281_2 | 0.010936 |
| GO:0022411_1+GO:0022411_2 | 0.010925 |
| abs(GO:0005730_1-GO:0005730_2) | 0.010866 |
| GO:1901361_1+GO:1901361_2 | 0.010584 |
| GO:0006614_1+GO:0006614_2 | 0.010569 |
| GO:0044445_1+GO:0044445_2 | 0.010541 |
| GO:0045047_1+GO:0045047_2 | 0.010491 |
| GO:0072599_1+GO:0072599_2 | 0.010491 |
| hsa03010_1+hsa03010_2 | 0.010442 |
| GO:0006613_1+GO:0006613_2 | 0.010361 |
| GO:0008150_1+GO:0008150_2 | 0.010343 |
| GO:0033554_1+GO:0033554_2 | 0.010298 |
| GO:1990234_1+GO:1990234_2 | 0.010272 |
| GO:0044710_1+GO:0044710_2 | 0.010266 |
| GO:0019058_1+GO:0019058_2 | 0.010248 |
| abs(GO:0044451_1-GO:0044451_2) | 0.010223 |
| GO:0006259_1+GO:0006259_2 | 0.010209 |
| abs(GO:0022613_1-GO:0022613_2) | 0.010072 |
| GO:0044391_1+GO:0044391_2 | 0.010058 |
| GO:0000375_1+GO:0000375_2 | 0.010003 |

1. Part of MaxRel feature list on *DS*_9_

| **Feature** | **Score** |
| --- | --- |
| GO:0044428_1+GO:0044428_2 | 0.061457 |
| GO:1901363_1+GO:1901363_2 | 0.060153 |
| GO:0032991_1+GO:0032991_2 | 0.059642 |
| GO:0097159_1+GO:0097159_2 | 0.059207 |
| GO:0031981_1+GO:0031981_2 | 0.059065 |
| GO:0003676_1+GO:0003676_2 | 0.058151 |
| GO:0090304_1+GO:0090304_2 | 0.057232 |
| GO:0044446_1+GO:0044446_2 | 0.055008 |
| GO:0044260_1+GO:0044260_2 | 0.054891 |
| GO:0044422_1+GO:0044422_2 | 0.053771 |
| GO:0005634_1+GO:0005634_2 | 0.05372 |
| GO:0070013_1+GO:0070013_2 | 0.052644 |
| GO:0043233_1+GO:0043233_2 | 0.05193 |
| GO:0031974_1+GO:0031974_2 | 0.05141 |
| GO:0006139_1+GO:0006139_2 | 0.050697 |
| GO:0043170_1+GO:0043170_2 | 0.048565 |
| GO:0046483_1+GO:0046483_2 | 0.048193 |
| GO:0006725_1+GO:0006725_2 | 0.047863 |
| GO:1901360_1+GO:1901360_2 | 0.045762 |
| GO:0034641_1+GO:0034641_2 | 0.045493 |
| GO:0010467_1+GO:0010467_2 | 0.044522 |
| GO:0006807_1+GO:0006807_2 | 0.042117 |
| GO:0044238_1+GO:0044238_2 | 0.040279 |
| GO:0016070_1+GO:0016070_2 | 0.039567 |
| GO:0044237_1+GO:0044237_2 | 0.037913 |
| GO:0071704_1+GO:0071704_2 | 0.037219 |
| GO:0043228_1+GO:0043228_2 | 0.036565 |
| GO:0043232_1+GO:0043232_2 | 0.036565 |
| GO:0034645_1+GO:0034645_2 | 0.032941 |
| GO:0009059_1+GO:0009059_2 | 0.031887 |
| GO:0005654_1+GO:0005654_2 | 0.03136 |
| GO:0005488_1+GO:0005488_2 | 0.031178 |
| GO:0044424_1+GO:0044424_2 | 0.030517 |
| GO:0005622_1+GO:0005622_2 | 0.029608 |
| GO:0071840_1+GO:0071840_2 | 0.028131 |
| GO:1901576_1+GO:1901576_2 | 0.027243 |
| GO:0044249_1+GO:0044249_2 | 0.026919 |
| GO:0009058_1+GO:0009058_2 | 0.026218 |
| GO:0043234_1+GO:0043234_2 | 0.025642 |
| GO:0044822_1+GO:0044822_2 | 0.024318 |
| GO:0032774_1+GO:0032774_2 | 0.024092 |
| abs(GO:0044424_1-GO:0044424_2) | 0.023449 |
| abs(GO:0005488_1-GO:0005488_2) | 0.023103 |
| GO:0003723_1+GO:0003723_2 | 0.022982 |
| abs(GO:0005622_1-GO:0005622_2) | 0.022946 |
| GO:0034654_1+GO:0034654_2 | 0.022752 |
| GO:0018130_1+GO:0018130_2 | 0.021875 |
| GO:0019438_1+GO:0019438_2 | 0.021806 |
| GO:0016071_1+GO:0016071_2 | 0.021718 |
| abs(GO:0043229_1-GO:0043229_2) | 0.021557 |
| GO:0016043_1+GO:0016043_2 | 0.021381 |
| GO:0044271_1+GO:0044271_2 | 0.021321 |
| abs(GO:0043226_1-GO:0043226_2) | 0.02119 |
| GO:1901362_1+GO:1901362_2 | 0.021124 |
| GO:0044764_1+GO:0044764_2 | 0.021117 |
| abs(GO:0031224_1-GO:0031224_2) | 0.021073 |
| GO:0016032_1+GO:0016032_2 | 0.020915 |
| abs(GO:0044425_1-GO:0044425_2) | 0.020828 |
| GO:0005515_1+GO:0005515_2 | 0.020731 |
| GO:0044267_1+GO:0044267_2 | 0.020646 |
| abs(GO:0016021_1-GO:0016021_2) | 0.020363 |
| GO:0044419_1+GO:0044419_2 | 0.02026 |
| GO:0044403_1+GO:0044403_2 | 0.02026 |
| GO:0031224_1+GO:0031224_2 | 0.019919 |
| GO:0016021_1+GO:0016021_2 | 0.019045 |
| GO:0005730_1+GO:0005730_2 | 0.018804 |
| GO:0030529_1+GO:0030529_2 | 0.018778 |
| GO:0044451_1+GO:0044451_2 | 0.018678 |
| GO:0060255_1+GO:0060255_2 | 0.018662 |
| abs(GO:0044238_1-GO:0044238_2) | 0.018357 |
| abs(GO:0071704_1-GO:0071704_2) | 0.017986 |
| GO:0006351_1+GO:0006351_2 | 0.017637 |
| GO:0044265_1+GO:0044265_2 | 0.017624 |
| abs(GO:0008152_1-GO:0008152_2) | 0.017581 |
| GO:0019538_1+GO:0019538_2 | 0.017497 |
| abs(GO:0044237_1-GO:0044237_2) | 0.017282 |
| abs(GO:0043227_1-GO:0043227_2) | 0.017222 |
| GO:0010556_1+GO:0010556_2 | 0.017055 |
| GO:0080090_1+GO:0080090_2 | 0.016689 |
| GO:0019083_1+GO:0019083_2 | 0.016627 |
| abs(GO:0016020_1-GO:0016020_2) | 0.016425 |
| GO:2000112_1+GO:2000112_2 | 0.016334 |
| GO:0010468_1+GO:0010468_2 | 0.016293 |
| GO:0044464_1+GO:0044464_2 | 0.016259 |
| GO:0005623_1+GO:0005623_2 | 0.016233 |
| GO:0031323_1+GO:0031323_2 | 0.01622 |
| GO:0022613_1+GO:0022613_2 | 0.016196 |
| GO:0031326_1+GO:0031326_2 | 0.016071 |
| GO:0003674_1+GO:0003674_2 | 0.01606 |
| GO:0051171_1+GO:0051171_2 | 0.016058 |
| GO:0009889_1+GO:0009889_2 | 0.015874 |
| GO:0009057_1+GO:0009057_2 | 0.015603 |
| GO:0019219_1+GO:0019219_2 | 0.015414 |
| GO:0005829_1+GO:0005829_2 | 0.015278 |
| abs(GO:0043170_1-GO:0043170_2) | 0.015191 |
| GO:0019222_1+GO:0019222_2 | 0.015174 |
| GO:0000956_1+GO:0000956_2 | 0.015093 |
| GO:0006996_1+GO:0006996_2 | 0.014966 |
| GO:0006402_1+GO:0006402_2 | 0.01482 |
| GO:0051276_1+GO:0051276_2 | 0.014691 |
| abs(GO:0043231_1-GO:0043231_2) | 0.014642 |
| GO:0006401_1+GO:0006401_2 | 0.014468 |
| abs(GO:0003674_1-GO:0003674_2) | 0.014273 |
| GO:0006396_1+GO:0006396_2 | 0.014195 |
| abs(GO:0044260_1-GO:0044260_2) | 0.014058 |
| GO:0009987_1+GO:0009987_2 | 0.014026 |
| GO:0000184_1+GO:0000184_2 | 0.01357 |
| abs(GO:0044464_1-GO:0044464_2) | 0.013545 |
| abs(GO:0005623_1-GO:0005623_2) | 0.01353 |
| GO:0043933_1+GO:0043933_2 | 0.013474 |
| GO:0006413_1+GO:0006413_2 | 0.013447 |
| GO:0006415_1+GO:0006415_2 | 0.013298 |
| GO:0032984_1+GO:0032984_2 | 0.012877 |
| GO:0043241_1+GO:0043241_2 | 0.01278 |
| abs(GO:0044764_1-GO:0044764_2) | 0.012611 |
| abs(GO:0016032_1-GO:0016032_2) | 0.012458 |
| GO:0022626_1+GO:0022626_2 | 0.012439 |
| GO:0006974_1+GO:0006974_2 | 0.012402 |
| GO:0005694_1+GO:0005694_2 | 0.01232 |
| GO:0034655_1+GO:0034655_2 | 0.012148 |
| GO:0043624_1+GO:0043624_2 | 0.012137 |
| GO:0019080_1+GO:0019080_2 | 0.012086 |
| GO:0042254_1+GO:0042254_2 | 0.012071 |
| GO:0044033_1+GO:0044033_2 | 0.012058 |
| abs(GO:0044403_1-GO:0044403_2) | 0.01187 |
| abs(GO:0044419_1-GO:0044419_2) | 0.01187 |
| GO:0006414_1+GO:0006414_2 | 0.011433 |
| GO:0044270_1+GO:0044270_2 | 0.011321 |
| GO:0006614_1+GO:0006614_2 | 0.011256 |
| GO:0072599_1+GO:0072599_2 | 0.0112 |
| GO:0045047_1+GO:0045047_2 | 0.0112 |
| GO:0046700_1+GO:0046700_2 | 0.011172 |
| GO:0022411_1+GO:0022411_2 | 0.011094 |
| GO:0019439_1+GO:0019439_2 | 0.011048 |
| GO:0006613_1+GO:0006613_2 | 0.01103 |
| GO:0044445_1+GO:0044445_2 | 0.011007 |
| GO:0044427_1+GO:0044427_2 | 0.01098 |
| abs(GO:0005730_1-GO:0005730_2) | 0.010929 |
| hsa03010_1+hsa03010_2 | 0.0109 |
| abs(GO:0009987_1-GO:0009987_2) | 0.010869 |
| GO:0006281_1+GO:0006281_2 | 0.010761 |
| GO:0019058_1+GO:0019058_2 | 0.010711 |
| GO:1990234_1+GO:1990234_2 | 0.01054 |
| GO:0070972_1+GO:0070972_2 | 0.010504 |
| GO:1901361_1+GO:1901361_2 | 0.010461 |
| GO:0044391_1+GO:0044391_2 | 0.010398 |
| GO:0016604_1+GO:0016604_2 | 0.010383 |
| GO:0006412_1+GO:0006412_2 | 0.010307 |
| abs(GO:0019083_1-GO:0019083_2) | 0.010266 |
| abs(GO:0022613_1-GO:0022613_2) | 0.010248 |
| GO:0006259_1+GO:0006259_2 | 0.010107 |
| GO:0033554_1+GO:0033554_2 | 0.010061 |
| GO:0008150_1+GO:0008150_2 | 0.010017 |

1. Part of MaxRel feature list on *DS*_10_

| **Feature** | **Score** |
| --- | --- |
| GO:0044428_1+GO:0044428_2 | 0.061773 |
| GO:1901363_1+GO:1901363_2 | 0.060524 |
| GO:0097159_1+GO:0097159_2 | 0.059689 |
| GO:0032991_1+GO:0032991_2 | 0.059345 |
| GO:0031981_1+GO:0031981_2 | 0.058817 |
| GO:0003676_1+GO:0003676_2 | 0.058778 |
| GO:0090304_1+GO:0090304_2 | 0.057685 |
| GO:0044260_1+GO:0044260_2 | 0.054618 |
| GO:0044446_1+GO:0044446_2 | 0.054513 |
| GO:0005634_1+GO:0005634_2 | 0.053704 |
| GO:0044422_1+GO:0044422_2 | 0.053406 |
| GO:0070013_1+GO:0070013_2 | 0.052513 |
| GO:0043233_1+GO:0043233_2 | 0.051704 |
| GO:0006139_1+GO:0006139_2 | 0.051383 |
| GO:0031974_1+GO:0031974_2 | 0.050987 |
| GO:0046483_1+GO:0046483_2 | 0.048816 |
| GO:0043170_1+GO:0043170_2 | 0.048588 |
| GO:0006725_1+GO:0006725_2 | 0.048386 |
| GO:1901360_1+GO:1901360_2 | 0.046439 |
| GO:0034641_1+GO:0034641_2 | 0.046024 |
| GO:0010467_1+GO:0010467_2 | 0.044285 |
| GO:0006807_1+GO:0006807_2 | 0.042318 |
| GO:0044238_1+GO:0044238_2 | 0.040622 |
| GO:0016070_1+GO:0016070_2 | 0.040386 |
| GO:0044237_1+GO:0044237_2 | 0.038111 |
| GO:0071704_1+GO:0071704_2 | 0.037628 |
| GO:0043228_1+GO:0043228_2 | 0.036247 |
| GO:0043232_1+GO:0043232_2 | 0.036247 |
| GO:0034645_1+GO:0034645_2 | 0.033346 |
| GO:0009059_1+GO:0009059_2 | 0.032216 |
| GO:0005654_1+GO:0005654_2 | 0.031401 |
| GO:0044424_1+GO:0044424_2 | 0.029869 |
| GO:0005622_1+GO:0005622_2 | 0.028978 |
| GO:0071840_1+GO:0071840_2 | 0.028662 |
| GO:1901576_1+GO:1901576_2 | 0.027578 |
| GO:0044249_1+GO:0044249_2 | 0.027339 |
| GO:0009058_1+GO:0009058_2 | 0.026521 |
| GO:0043234_1+GO:0043234_2 | 0.025051 |
| GO:0032774_1+GO:0032774_2 | 0.024754 |
| GO:0044822_1+GO:0044822_2 | 0.024349 |
| abs(GO:0005488_1-GO:0005488_2) | 0.023395 |
| GO:0034654_1+GO:0034654_2 | 0.023278 |
| GO:0003723_1+GO:0003723_2 | 0.023236 |
| abs(GO:0044424_1-GO:0044424_2) | 0.02312 |
| abs(GO:0005622_1-GO:0005622_2) | 0.022599 |
| GO:0018130_1+GO:0018130_2 | 0.022369 |
| GO:0019438_1+GO:0019438_2 | 0.022327 |
| GO:0044271_1+GO:0044271_2 | 0.021907 |
| GO:0016043_1+GO:0016043_2 | 0.021901 |
| abs(GO:0043229_1-GO:0043229_2) | 0.021653 |
| GO:0016071_1+GO:0016071_2 | 0.021618 |
| GO:1901362_1+GO:1901362_2 | 0.021614 |
| abs(GO:0044425_1-GO:0044425_2) | 0.021412 |
| abs(GO:0043226_1-GO:0043226_2) | 0.021399 |
| abs(GO:0031224_1-GO:0031224_2) | 0.021126 |
| GO:0005515_1+GO:0005515_2 | 0.02109 |
| GO:0044764_1+GO:0044764_2 | 0.020685 |
| GO:0016032_1+GO:0016032_2 | 0.020517 |
| abs(GO:0016021_1-GO:0016021_2) | 0.020454 |
| GO:0044267_1+GO:0044267_2 | 0.02001 |
| GO:0044403_1+GO:0044403_2 | 0.019874 |
| GO:0044419_1+GO:0044419_2 | 0.019874 |
| GO:0031224_1+GO:0031224_2 | 0.019762 |
| GO:0060255_1+GO:0060255_2 | 0.019269 |
| GO:0030529_1+GO:0030529_2 | 0.019106 |
| GO:0016021_1+GO:0016021_2 | 0.01894 |
| GO:0044451_1+GO:0044451_2 | 0.018807 |
| abs(GO:0044238_1-GO:0044238_2) | 0.018649 |
| GO:0005730_1+GO:0005730_2 | 0.018508 |
| abs(GO:0071704_1-GO:0071704_2) | 0.018309 |
| GO:0006351_1+GO:0006351_2 | 0.018195 |
| abs(GO:0008152_1-GO:0008152_2) | 0.017855 |
| abs(GO:0043227_1-GO:0043227_2) | 0.017608 |
| GO:0010556_1+GO:0010556_2 | 0.017544 |
| abs(GO:0044237_1-GO:0044237_2) | 0.017476 |
| GO:0044265_1+GO:0044265_2 | 0.017063 |
| GO:0019538_1+GO:0019538_2 | 0.017032 |
| GO:0080090_1+GO:0080090_2 | 0.016978 |
| GO:0010468_1+GO:0010468_2 | 0.016872 |
| GO:2000112_1+GO:2000112_2 | 0.016704 |
| abs(GO:0016020_1-GO:0016020_2) | 0.016588 |
| GO:0031323_1+GO:0031323_2 | 0.016517 |
| GO:0031326_1+GO:0031326_2 | 0.016438 |
| GO:0019083_1+GO:0019083_2 | 0.016436 |
| GO:0051171_1+GO:0051171_2 | 0.016364 |
| GO:0009889_1+GO:0009889_2 | 0.016279 |
| GO:0044464_1+GO:0044464_2 | 0.016041 |
| GO:0005623_1+GO:0005623_2 | 0.016014 |
| GO:0003674_1+GO:0003674_2 | 0.01591 |
| GO:0022613_1+GO:0022613_2 | 0.015864 |
| GO:0019219_1+GO:0019219_2 | 0.015801 |
| GO:0019222_1+GO:0019222_2 | 0.015788 |
| abs(GO:0043170_1-GO:0043170_2) | 0.015323 |
| GO:0009057_1+GO:0009057_2 | 0.015165 |
| GO:0005829_1+GO:0005829_2 | 0.015127 |
| GO:0000956_1+GO:0000956_2 | 0.014985 |
| GO:0051276_1+GO:0051276_2 | 0.014915 |
| GO:0006996_1+GO:0006996_2 | 0.014842 |
| abs(GO:0043231_1-GO:0043231_2) | 0.014841 |
| GO:0006402_1+GO:0006402_2 | 0.014628 |
| GO:0006401_1+GO:0006401_2 | 0.014401 |
| GO:0006396_1+GO:0006396_2 | 0.014288 |
| GO:0009987_1+GO:0009987_2 | 0.014239 |
| abs(GO:0044260_1-GO:0044260_2) | 0.014154 |
| abs(GO:0003674_1-GO:0003674_2) | 0.014023 |
| GO:0043933_1+GO:0043933_2 | 0.01358 |
| abs(GO:0044464_1-GO:0044464_2) | 0.013457 |
| abs(GO:0005623_1-GO:0005623_2) | 0.01344 |
| GO:0006413_1+GO:0006413_2 | 0.013356 |
| GO:0000184_1+GO:0000184_2 | 0.013307 |
| GO:0006415_1+GO:0006415_2 | 0.013165 |
| GO:0032984_1+GO:0032984_2 | 0.012696 |
| GO:0043241_1+GO:0043241_2 | 0.012671 |
| GO:0034655_1+GO:0034655_2 | 0.012568 |
| abs(GO:0044764_1-GO:0044764_2) | 0.012376 |
| GO:0006974_1+GO:0006974_2 | 0.012354 |
| abs(GO:0016032_1-GO:0016032_2) | 0.012251 |
| GO:0022626_1+GO:0022626_2 | 0.012199 |
| GO:0005694_1+GO:0005694_2 | 0.012076 |
| GO:0043624_1+GO:0043624_2 | 0.012045 |
| GO:0042254_1+GO:0042254_2 | 0.011911 |
| GO:0019080_1+GO:0019080_2 | 0.011878 |
| GO:0044033_1+GO:0044033_2 | 0.011835 |
| GO:0044270_1+GO:0044270_2 | 0.011707 |
| abs(GO:0044419_1-GO:0044419_2) | 0.01168 |
| abs(GO:0044403_1-GO:0044403_2) | 0.01168 |
| GO:0046700_1+GO:0046700_2 | 0.011553 |
| GO:0019439_1+GO:0019439_2 | 0.01138 |
| GO:0006414_1+GO:0006414_2 | 0.011318 |
| GO:0006614_1+GO:0006614_2 | 0.011104 |
| abs(GO:0009987_1-GO:0009987_2) | 0.011039 |
| GO:0045047_1+GO:0045047_2 | 0.011009 |
| GO:0072599_1+GO:0072599_2 | 0.011009 |
| GO:0022411_1+GO:0022411_2 | 0.011004 |
| GO:0044445_1+GO:0044445_2 | 0.010984 |
| GO:0006613_1+GO:0006613_2 | 0.010837 |
| GO:0044427_1+GO:0044427_2 | 0.010799 |
| GO:1901361_1+GO:1901361_2 | 0.010775 |
| GO:0006281_1+GO:0006281_2 | 0.010775 |
| abs(GO:0005730_1-GO:0005730_2) | 0.010714 |
| GO:0019058_1+GO:0019058_2 | 0.010704 |
| hsa03010_1+hsa03010_2 | 0.010688 |
| GO:1990234_1+GO:1990234_2 | 0.010466 |
| GO:0016604_1+GO:0016604_2 | 0.010457 |
| GO:0070972_1+GO:0070972_2 | 0.010416 |
| GO:0006412_1+GO:0006412_2 | 0.010293 |
| GO:0008150_1+GO:0008150_2 | 0.010281 |
| GO:0044710_1+GO:0044710_2 | 0.010265 |
| GO:0044391_1+GO:0044391_2 | 0.010196 |
| GO:0006259_1+GO:0006259_2 | 0.010176 |
| abs(GO:0019083_1-GO:0019083_2) | 0.010123 |
| GO:0000375_1+GO:0000375_2 | 0.0101 |
| GO:0033554_1+GO:0033554_2 | 0.010011 |
