# Supplementary material for: Analysis of Protein–Protein Functional Associations by Using Gene Ontology and KEGG Pathway
Source: Biomed Res Int. 2019 Jul 18;2019:4963289. doi: 10.1155/2019/4963289 (PMC6668538; doi:10.1155/2019/4963289)
Supplement: Supplementary 3 — Selected 158 features and their occurrences in the 10 feature sets of the 10 datasets (√: feature is in the feature set; ☓: feature is not included in the feature set). [file 4963289.f3.docx]

**Supplementary Material S3.** Selected 158 features and their occurrences in the 10 feature sets of the 10 datasets (√: feature is in the feature set; ☓: feature is not included in the feature set)

| **Feature name** | **Frequency** | **Feature set on** | | | | | | | | | |
| --- | --- | --- | --- | --- | --- | --- | --- | --- | --- | --- | --- |
|  |  | ***DS*_1_** | ***DS*_2_** | ***DS*_3_** | ***DS*_4_** | ***DS*_5_** | ***DS*_6_** | ***DS*_7_** | ***DS*_8_** | ***DS*_9_** | ***DS*_10_** |
| hsa03010_1+hsa03010_2 | 10 | √ | √ | √ | √ | √ | √ | √ | √ | √ | √ |
| GO:0000184_1+GO:0000184_2 | 10 | √ | √ | √ | √ | √ | √ | √ | √ | √ | √ |
| GO:0000375_1+GO:0000375_2 | 7 | √ | √ | ☓ | √ | ☓ | √ | √ | √ | ☓ | √ |
| GO:0000956_1+GO:0000956_2 | 10 | √ | √ | √ | √ | √ | √ | √ | √ | √ | √ |
| GO:0003674_1+GO:0003674_2 | 10 | √ | √ | √ | √ | √ | √ | √ | √ | √ | √ |
| GO:0003676_1+GO:0003676_2 | 10 | √ | √ | √ | √ | √ | √ | √ | √ | √ | √ |
| GO:0003723_1+GO:0003723_2 | 10 | √ | √ | √ | √ | √ | √ | √ | √ | √ | √ |
| GO:0005488_1+GO:0005488_2 | 1 | ☓ | ☓ | ☓ | ☓ | ☓ | ☓ | ☓ | ☓ | √ | ☓ |
| GO:0005515_1+GO:0005515_2 | 10 | √ | √ | √ | √ | √ | √ | √ | √ | √ | √ |
| GO:0005622_1+GO:0005622_2 | 10 | √ | √ | √ | √ | √ | √ | √ | √ | √ | √ |
| GO:0005623_1+GO:0005623_2 | 10 | √ | √ | √ | √ | √ | √ | √ | √ | √ | √ |
| GO:0005634_1+GO:0005634_2 | 10 | √ | √ | √ | √ | √ | √ | √ | √ | √ | √ |
| GO:0005654_1+GO:0005654_2 | 10 | √ | √ | √ | √ | √ | √ | √ | √ | √ | √ |
| GO:0005694_1+GO:0005694_2 | 10 | √ | √ | √ | √ | √ | √ | √ | √ | √ | √ |
| GO:0005730_1+GO:0005730_2 | 10 | √ | √ | √ | √ | √ | √ | √ | √ | √ | √ |
| GO:0005829_1+GO:0005829_2 | 10 | √ | √ | √ | √ | √ | √ | √ | √ | √ | √ |
| GO:0006139_1+GO:0006139_2 | 10 | √ | √ | √ | √ | √ | √ | √ | √ | √ | √ |
| GO:0006259_1+GO:0006259_2 | 10 | √ | √ | √ | √ | √ | √ | √ | √ | √ | √ |
| GO:0006281_1+GO:0006281_2 | 10 | √ | √ | √ | √ | √ | √ | √ | √ | √ | √ |
| GO:0006351_1+GO:0006351_2 | 10 | √ | √ | √ | √ | √ | √ | √ | √ | √ | √ |
| GO:0006396_1+GO:0006396_2 | 10 | √ | √ | √ | √ | √ | √ | √ | √ | √ | √ |
| GO:0006401_1+GO:0006401_2 | 10 | √ | √ | √ | √ | √ | √ | √ | √ | √ | √ |
| GO:0006402_1+GO:0006402_2 | 10 | √ | √ | √ | √ | √ | √ | √ | √ | √ | √ |
| GO:0006412_1+GO:0006412_2 | 6 | √ | ☓ | √ | √ | ☓ | ☓ | √ | ☓ | √ | √ |
| GO:0006413_1+GO:0006413_2 | 10 | √ | √ | √ | √ | √ | √ | √ | √ | √ | √ |
| GO:0006414_1+GO:0006414_2 | 10 | √ | √ | √ | √ | √ | √ | √ | √ | √ | √ |
| GO:0006415_1+GO:0006415_2 | 10 | √ | √ | √ | √ | √ | √ | √ | √ | √ | √ |
| GO:0006613_1+GO:0006613_2 | 10 | √ | √ | √ | √ | √ | √ | √ | √ | √ | √ |
| GO:0006614_1+GO:0006614_2 | 10 | √ | √ | √ | √ | √ | √ | √ | √ | √ | √ |
| GO:0006725_1+GO:0006725_2 | 10 | √ | √ | √ | √ | √ | √ | √ | √ | √ | √ |
| GO:0006807_1+GO:0006807_2 | 10 | √ | √ | √ | √ | √ | √ | √ | √ | √ | √ |
| GO:0006974_1+GO:0006974_2 | 10 | √ | √ | √ | √ | √ | √ | √ | √ | √ | √ |
| GO:0006996_1+GO:0006996_2 | 10 | √ | √ | √ | √ | √ | √ | √ | √ | √ | √ |
| GO:0008150_1+GO:0008150_2 | 9 | √ | √ | √ | √ | √ | ☓ | √ | √ | √ | √ |
| GO:0009057_1+GO:0009057_2 | 10 | √ | √ | √ | √ | √ | √ | √ | √ | √ | √ |
| GO:0009058_1+GO:0009058_2 | 10 | √ | √ | √ | √ | √ | √ | √ | √ | √ | √ |
| GO:0009059_1+GO:0009059_2 | 10 | √ | √ | √ | √ | √ | √ | √ | √ | √ | √ |
| GO:0009889_1+GO:0009889_2 | 10 | √ | √ | √ | √ | √ | √ | √ | √ | √ | √ |
| GO:0009987_1+GO:0009987_2 | 10 | √ | √ | √ | √ | √ | √ | √ | √ | √ | √ |
| GO:0010467_1+GO:0010467_2 | 10 | √ | √ | √ | √ | √ | √ | √ | √ | √ | √ |
| GO:0010468_1+GO:0010468_2 | 10 | √ | √ | √ | √ | √ | √ | √ | √ | √ | √ |
| GO:0010556_1+GO:0010556_2 | 10 | √ | √ | √ | √ | √ | √ | √ | √ | √ | √ |
| GO:0016021_1+GO:0016021_2 | 10 | √ | √ | √ | √ | √ | √ | √ | √ | √ | √ |
| GO:0016032_1+GO:0016032_2 | 10 | √ | √ | √ | √ | √ | √ | √ | √ | √ | √ |
| GO:0016043_1+GO:0016043_2 | 10 | √ | √ | √ | √ | √ | √ | √ | √ | √ | √ |
| GO:0016070_1+GO:0016070_2 | 10 | √ | √ | √ | √ | √ | √ | √ | √ | √ | √ |
| GO:0016071_1+GO:0016071_2 | 10 | √ | √ | √ | √ | √ | √ | √ | √ | √ | √ |
| GO:0016604_1+GO:0016604_2 | 10 | √ | √ | √ | √ | √ | √ | √ | √ | √ | √ |
| GO:0018130_1+GO:0018130_2 | 10 | √ | √ | √ | √ | √ | √ | √ | √ | √ | √ |
| GO:0019058_1+GO:0019058_2 | 10 | √ | √ | √ | √ | √ | √ | √ | √ | √ | √ |
| GO:0019080_1+GO:0019080_2 | 10 | √ | √ | √ | √ | √ | √ | √ | √ | √ | √ |
| GO:0019083_1+GO:0019083_2 | 10 | √ | √ | √ | √ | √ | √ | √ | √ | √ | √ |
| GO:0019219_1+GO:0019219_2 | 10 | √ | √ | √ | √ | √ | √ | √ | √ | √ | √ |
| GO:0019222_1+GO:0019222_2 | 10 | √ | √ | √ | √ | √ | √ | √ | √ | √ | √ |
| GO:0019438_1+GO:0019438_2 | 10 | √ | √ | √ | √ | √ | √ | √ | √ | √ | √ |
| GO:0019439_1+GO:0019439_2 | 10 | √ | √ | √ | √ | √ | √ | √ | √ | √ | √ |
| GO:0019538_1+GO:0019538_2 | 10 | √ | √ | √ | √ | √ | √ | √ | √ | √ | √ |
| GO:0022411_1+GO:0022411_2 | 10 | √ | √ | √ | √ | √ | √ | √ | √ | √ | √ |
| GO:0022613_1+GO:0022613_2 | 10 | √ | √ | √ | √ | √ | √ | √ | √ | √ | √ |
| GO:0022626_1+GO:0022626_2 | 10 | √ | √ | √ | √ | √ | √ | √ | √ | √ | √ |
| GO:0030529_1+GO:0030529_2 | 10 | √ | √ | √ | √ | √ | √ | √ | √ | √ | √ |
| GO:0031224_1+GO:0031224_2 | 10 | √ | √ | √ | √ | √ | √ | √ | √ | √ | √ |
| GO:0031323_1+GO:0031323_2 | 10 | √ | √ | √ | √ | √ | √ | √ | √ | √ | √ |
| GO:0031326_1+GO:0031326_2 | 10 | √ | √ | √ | √ | √ | √ | √ | √ | √ | √ |
| GO:0031974_1+GO:0031974_2 | 10 | √ | √ | √ | √ | √ | √ | √ | √ | √ | √ |
| GO:0031981_1+GO:0031981_2 | 10 | √ | √ | √ | √ | √ | √ | √ | √ | √ | √ |
| GO:0032774_1+GO:0032774_2 | 10 | √ | √ | √ | √ | √ | √ | √ | √ | √ | √ |
| GO:0032984_1+GO:0032984_2 | 10 | √ | √ | √ | √ | √ | √ | √ | √ | √ | √ |
| GO:0032991_1+GO:0032991_2 | 10 | √ | √ | √ | √ | √ | √ | √ | √ | √ | √ |
| GO:0033554_1+GO:0033554_2 | 8 | √ | √ | √ | √ | ☓ | ☓ | √ | √ | √ | √ |
| GO:0034641_1+GO:0034641_2 | 10 | √ | √ | √ | √ | √ | √ | √ | √ | √ | √ |
| GO:0034645_1+GO:0034645_2 | 10 | √ | √ | √ | √ | √ | √ | √ | √ | √ | √ |
| GO:0034654_1+GO:0034654_2 | 10 | √ | √ | √ | √ | √ | √ | √ | √ | √ | √ |
| GO:0034655_1+GO:0034655_2 | 10 | √ | √ | √ | √ | √ | √ | √ | √ | √ | √ |
| GO:0042254_1+GO:0042254_2 | 10 | √ | √ | √ | √ | √ | √ | √ | √ | √ | √ |
| GO:0043170_1+GO:0043170_2 | 10 | √ | √ | √ | √ | √ | √ | √ | √ | √ | √ |
| GO:0043228_1+GO:0043228_2 | 10 | √ | √ | √ | √ | √ | √ | √ | √ | √ | √ |
| GO:0043232_1+GO:0043232_2 | 10 | √ | √ | √ | √ | √ | √ | √ | √ | √ | √ |
| GO:0043233_1+GO:0043233_2 | 10 | √ | √ | √ | √ | √ | √ | √ | √ | √ | √ |
| GO:0043234_1+GO:0043234_2 | 10 | √ | √ | √ | √ | √ | √ | √ | √ | √ | √ |
| GO:0043241_1+GO:0043241_2 | 10 | √ | √ | √ | √ | √ | √ | √ | √ | √ | √ |
| GO:0043624_1+GO:0043624_2 | 10 | √ | √ | √ | √ | √ | √ | √ | √ | √ | √ |
| GO:0043933_1+GO:0043933_2 | 10 | √ | √ | √ | √ | √ | √ | √ | √ | √ | √ |
| GO:0044033_1+GO:0044033_2 | 10 | √ | √ | √ | √ | √ | √ | √ | √ | √ | √ |
| GO:0044237_1+GO:0044237_2 | 10 | √ | √ | √ | √ | √ | √ | √ | √ | √ | √ |
| GO:0044238_1+GO:0044238_2 | 10 | √ | √ | √ | √ | √ | √ | √ | √ | √ | √ |
| GO:0044249_1+GO:0044249_2 | 10 | √ | √ | √ | √ | √ | √ | √ | √ | √ | √ |
| GO:0044260_1+GO:0044260_2 | 10 | √ | √ | √ | √ | √ | √ | √ | √ | √ | √ |
| GO:0044265_1+GO:0044265_2 | 10 | √ | √ | √ | √ | √ | √ | √ | √ | √ | √ |
| GO:0044267_1+GO:0044267_2 | 10 | √ | √ | √ | √ | √ | √ | √ | √ | √ | √ |
| GO:0044270_1+GO:0044270_2 | 10 | √ | √ | √ | √ | √ | √ | √ | √ | √ | √ |
| GO:0044271_1+GO:0044271_2 | 10 | √ | √ | √ | √ | √ | √ | √ | √ | √ | √ |
| GO:0044391_1+GO:0044391_2 | 10 | √ | √ | √ | √ | √ | √ | √ | √ | √ | √ |
| GO:0044403_1+GO:0044403_2 | 10 | √ | √ | √ | √ | √ | √ | √ | √ | √ | √ |
| GO:0044419_1+GO:0044419_2 | 10 | √ | √ | √ | √ | √ | √ | √ | √ | √ | √ |
| GO:0044422_1+GO:0044422_2 | 10 | √ | √ | √ | √ | √ | √ | √ | √ | √ | √ |
| GO:0044424_1+GO:0044424_2 | 10 | √ | √ | √ | √ | √ | √ | √ | √ | √ | √ |
| GO:0044427_1+GO:0044427_2 | 10 | √ | √ | √ | √ | √ | √ | √ | √ | √ | √ |
| GO:0044428_1+GO:0044428_2 | 10 | √ | √ | √ | √ | √ | √ | √ | √ | √ | √ |
| GO:0044445_1+GO:0044445_2 | 10 | √ | √ | √ | √ | √ | √ | √ | √ | √ | √ |
| GO:0044446_1+GO:0044446_2 | 10 | √ | √ | √ | √ | √ | √ | √ | √ | √ | √ |
| GO:0044451_1+GO:0044451_2 | 10 | √ | √ | √ | √ | √ | √ | √ | √ | √ | √ |
| GO:0044464_1+GO:0044464_2 | 10 | √ | √ | √ | √ | √ | √ | √ | √ | √ | √ |
| GO:0044710_1+GO:0044710_2 | 5 | ☓ | √ | √ | √ | ☓ | ☓ | ☓ | √ | ☓ | √ |
| GO:0044764_1+GO:0044764_2 | 10 | √ | √ | √ | √ | √ | √ | √ | √ | √ | √ |
| GO:0044822_1+GO:0044822_2 | 10 | √ | √ | √ | √ | √ | √ | √ | √ | √ | √ |
| GO:0045047_1+GO:0045047_2 | 10 | √ | √ | √ | √ | √ | √ | √ | √ | √ | √ |
| GO:0046483_1+GO:0046483_2 | 10 | √ | √ | √ | √ | √ | √ | √ | √ | √ | √ |
| GO:0046700_1+GO:0046700_2 | 10 | √ | √ | √ | √ | √ | √ | √ | √ | √ | √ |
| GO:0051171_1+GO:0051171_2 | 10 | √ | √ | √ | √ | √ | √ | √ | √ | √ | √ |
| GO:0051252_1+GO:0051252_2 | 3 | ☓ | ☓ | ☓ | √ | √ | √ | ☓ | ☓ | ☓ | ☓ |
| GO:0051276_1+GO:0051276_2 | 10 | √ | √ | √ | √ | √ | √ | √ | √ | √ | √ |
| GO:0060255_1+GO:0060255_2 | 10 | √ | √ | √ | √ | √ | √ | √ | √ | √ | √ |
| GO:0070013_1+GO:0070013_2 | 10 | √ | √ | √ | √ | √ | √ | √ | √ | √ | √ |
| GO:0070972_1+GO:0070972_2 | 9 | √ | √ | √ | √ | √ | √ | √ | ☓ | √ | √ |
| GO:0071704_1+GO:0071704_2 | 10 | √ | √ | √ | √ | √ | √ | √ | √ | √ | √ |
| GO:0071840_1+GO:0071840_2 | 10 | √ | √ | √ | √ | √ | √ | √ | √ | √ | √ |
| GO:0072599_1+GO:0072599_2 | 10 | √ | √ | √ | √ | √ | √ | √ | √ | √ | √ |
| GO:0080090_1+GO:0080090_2 | 10 | √ | √ | √ | √ | √ | √ | √ | √ | √ | √ |
| GO:0090304_1+GO:0090304_2 | 10 | √ | √ | √ | √ | √ | √ | √ | √ | √ | √ |
| GO:0097159_1+GO:0097159_2 | 10 | √ | √ | √ | √ | √ | √ | √ | √ | √ | √ |
| GO:1901360_1+GO:1901360_2 | 10 | √ | √ | √ | √ | √ | √ | √ | √ | √ | √ |
| GO:1901361_1+GO:1901361_2 | 10 | √ | √ | √ | √ | √ | √ | √ | √ | √ | √ |
| GO:1901362_1+GO:1901362_2 | 10 | √ | √ | √ | √ | √ | √ | √ | √ | √ | √ |
| GO:1901363_1+GO:1901363_2 | 10 | √ | √ | √ | √ | √ | √ | √ | √ | √ | √ |
| GO:1901576_1+GO:1901576_2 | 10 | √ | √ | √ | √ | √ | √ | √ | √ | √ | √ |
| GO:1990234_1+GO:1990234_2 | 10 | √ | √ | √ | √ | √ | √ | √ | √ | √ | √ |
| GO:2000112_1+GO:2000112_2 | 10 | √ | √ | √ | √ | √ | √ | √ | √ | √ | √ |
| abs(GO:0003674_1-GO:0003674_2) | 10 | √ | √ | √ | √ | √ | √ | √ | √ | √ | √ |
| abs(GO:0005488_1-GO:0005488_2) | 10 | √ | √ | √ | √ | √ | √ | √ | √ | √ | √ |
| abs(GO:0005622_1-GO:0005622_2) | 10 | √ | √ | √ | √ | √ | √ | √ | √ | √ | √ |
| abs(GO:0005623_1-GO:0005623_2) | 10 | √ | √ | √ | √ | √ | √ | √ | √ | √ | √ |
| abs(GO:0005634_1-GO:0005634_2) | 1 | ☓ | ☓ | ☓ | ☓ | ☓ | ☓ | √ | ☓ | ☓ | ☓ |
| abs(GO:0005730_1-GO:0005730_2) | 10 | √ | √ | √ | √ | √ | √ | √ | √ | √ | √ |
| abs(GO:0008152_1-GO:0008152_2) | 10 | √ | √ | √ | √ | √ | √ | √ | √ | √ | √ |
| abs(GO:0009987_1-GO:0009987_2) | 10 | √ | √ | √ | √ | √ | √ | √ | √ | √ | √ |
| abs(GO:0016020_1-GO:0016020_2) | 10 | √ | √ | √ | √ | √ | √ | √ | √ | √ | √ |
| abs(GO:0016021_1-GO:0016021_2) | 10 | √ | √ | √ | √ | √ | √ | √ | √ | √ | √ |
| abs(GO:0016032_1-GO:0016032_2) | 10 | √ | √ | √ | √ | √ | √ | √ | √ | √ | √ |
| abs(GO:0019083_1-GO:0019083_2) | 6 | √ | √ | ☓ | √ | ☓ | ☓ | √ | ☓ | √ | √ |
| abs(GO:0022613_1-GO:0022613_2) | 6 | √ | √ | √ | ☓ | ☓ | ☓ | √ | √ | √ | ☓ |
| abs(GO:0031224_1-GO:0031224_2) | 10 | √ | √ | √ | √ | √ | √ | √ | √ | √ | √ |
| abs(GO:0043170_1-GO:0043170_2) | 10 | √ | √ | √ | √ | √ | √ | √ | √ | √ | √ |
| abs(GO:0043226_1-GO:0043226_2) | 10 | √ | √ | √ | √ | √ | √ | √ | √ | √ | √ |
| abs(GO:0043227_1-GO:0043227_2) | 10 | √ | √ | √ | √ | √ | √ | √ | √ | √ | √ |
| abs(GO:0043229_1-GO:0043229_2) | 10 | √ | √ | √ | √ | √ | √ | √ | √ | √ | √ |
| abs(GO:0043231_1-GO:0043231_2) | 10 | √ | √ | √ | √ | √ | √ | √ | √ | √ | √ |
| abs(GO:0044237_1-GO:0044237_2) | 10 | √ | √ | √ | √ | √ | √ | √ | √ | √ | √ |
| abs(GO:0044238_1-GO:0044238_2) | 10 | √ | √ | √ | √ | √ | √ | √ | √ | √ | √ |
| abs(GO:0044260_1-GO:0044260_2) | 10 | √ | √ | √ | √ | √ | √ | √ | √ | √ | √ |
| abs(GO:0044403_1-GO:0044403_2) | 10 | √ | √ | √ | √ | √ | √ | √ | √ | √ | √ |
| abs(GO:0044419_1-GO:0044419_2) | 10 | √ | √ | √ | √ | √ | √ | √ | √ | √ | √ |
| abs(GO:0044424_1-GO:0044424_2) | 10 | √ | √ | √ | √ | √ | √ | √ | √ | √ | √ |
| abs(GO:0044425_1-GO:0044425_2) | 10 | √ | √ | √ | √ | √ | √ | √ | √ | √ | √ |
| abs(GO:0044451_1-GO:0044451_2) | 7 | √ | √ | √ | √ | ☓ | √ | √ | √ | ☓ | ☓ |
| abs(GO:0044464_1-GO:0044464_2) | 10 | √ | √ | √ | √ | √ | √ | √ | √ | √ | √ |
| abs(GO:0044764_1-GO:0044764_2) | 10 | √ | √ | √ | √ | √ | √ | √ | √ | √ | √ |
| abs(GO:0071704_1-GO:0071704_2) | 10 | √ | √ | √ | √ | √ | √ | √ | √ | √ | √ |
