# Supplementary material for: Analysis of Protein–Protein Functional Associations by Using Gene Ontology and KEGG Pathway
Source: Biomed Res Int. 2019 Jul 18;2019:4963289. doi: 10.1155/2019/4963289 (PMC6668538; doi:10.1155/2019/4963289)
Supplement: Supplementary 4 — Extracted important GO terms and their rating scores. [file 4963289.f4.docx]

**Supplementary Material S4.** Extracted important GO terms and their rating scores

| **GO term/KEGG pathway ID** | **GO term/KEGG pathway** | **Rating score** |
| --- | --- | --- |
| GO:0044260 | cellular macromolecule metabolic process | 0.688 |
| GO:0043170 | macromolecule metabolic process | 0.64 |
| GO:0044428 | nuclear part | 0.618 |
| GO:1901363 | heterocyclic compound binding | 0.6 |
| GO:0032991 | macromolecular complex | 0.593 |
| GO:0097159 | organic cyclic compound binding | 0.591 |
| GO:0031981 | nuclear lumen | 0.59 |
| GO:0044238 | primary metabolic process | 0.589 |
| GO:0003676 | nucleic acid binding | 0.583 |
| GO:0090304 | nucleic acid metabolic process | 0.569 |
| GO:0071704 | organic substance metabolic process | 0.556 |
| GO:0044237 | cellular metabolic process | 0.552 |
| GO:0005634 | nucleus | 0.549 |
| GO:0044446 | intracellular organelle part | 0.547 |
| GO:0044424 | intracellular part | 0.537 |
| GO:0044422 | organelle part | 0.536 |
| GO:0070013 | intracellular organelle lumen | 0.529 |
| GO:0005622 | intracellular | 0.523 |
| GO:0043233 | organelle lumen | 0.521 |
| GO:0031974 | membrane-enclosed lumen | 0.514 |
| GO:0006139 | nucleobase-containing compound metabolic process | 0.506 |
| GO:0046483 | heterocycle metabolic process | 0.482 |
| GO:0006725 | cellular aromatic compound metabolic process | 0.477 |
| GO:1901360 | organic cyclic compound metabolic process | 0.458 |
| GO:0034641 | cellular nitrogen compound metabolic process | 0.455 |
| GO:0010467 | gene expression | 0.442 |
| GO:0006807 | nitrogen compound metabolic process | 0.42 |
| GO:0031224 | intrinsic component of membrane | 0.406 |
| GO:0016070 | RNA metabolic process | 0.395 |
| GO:0016021 | integral component of membrane | 0.391 |
| GO:0043228 | non-membrane-bounded organelle | 0.362 |
| GO:0043232 | intracellular non-membrane-bounded organelle | 0.362 |
| GO:0044764 | multi-organism cellular process | 0.331 |
| GO:0034645 | cellular macromolecule biosynthetic process | 0.33 |
| GO:0016032 | viral process | 0.328 |
| GO:0009059 | macromolecule biosynthetic process | 0.319 |
| GO:0044403 | symbiosis, encompassing mutualism through parasitism | 0.316 |
| GO:0044419 | interspecies interaction between organisms | 0.316 |
| GO:0005654 | nucleoplasm | 0.315 |
| GO:0003674 | molecular_function | 0.305 |
| GO:0044464 | cell part | 0.302 |
| GO:0005623 | cell | 0.301 |
| GO:0005730 | nucleolus | 0.295 |
| GO:0071840 | cellular component organization or biogenesis | 0.284 |
| GO:1901576 | organic substance biosynthetic process | 0.273 |
| GO:0044249 | cellular biosynthetic process | 0.27 |
| GO:0005488 | binding | 0.266 |
| GO:0009058 | biosynthetic process | 0.263 |
| GO:0044451 | nucleoplasm part | 0.262 |
| GO:0043234 | protein complex | 0.253 |
| GO:0009987 | cellular process | 0.251 |
| GO:0032774 | RNA biosynthetic process | 0.242 |
| GO:0044822 | poly(A) RNA binding | 0.241 |
| GO:0003723 | RNA binding | 0.229 |
| GO:0034654 | nucleobase-containing compound biosynthetic process | 0.228 |
| GO:0019083 | viral transcription | 0.224 |
| GO:0022613 | ribonucleoprotein complex biogenesis | 0.222 |
| GO:0018130 | heterocycle biosynthetic process | 0.219 |
| GO:0019438 | aromatic compound biosynthetic process | 0.219 |
| GO:0043229 | intracellular organelle | 0.217 |
| GO:0016043 | cellular component organization | 0.216 |
| GO:0016071 | mRNA metabolic process | 0.215 |
| GO:0044271 | cellular nitrogen compound biosynthetic process | 0.214 |
| GO:0043226 | organelle | 0.213 |
| GO:1901362 | organic cyclic compound biosynthetic process | 0.212 |
| GO:0005515 | protein binding | 0.211 |
| GO:0044425 | membrane part | 0.208 |
| GO:0044267 | cellular protein metabolic process | 0.204 |
| GO:0060255 | regulation of macromolecule metabolic process | 0.189 |
| GO:0030529 | ribonucleoprotein complex | 0.188 |
| GO:0008152 | metabolic process | 0.179 |
| GO:0006351 | transcription, DNA-templated | 0.178 |
| GO:0043227 | membrane-bounded organelle | 0.175 |
| GO:0044265 | cellular macromolecule catabolic process | 0.173 |
| GO:0019538 | protein metabolic process | 0.173 |
| GO:0010556 | regulation of macromolecule biosynthetic process | 0.172 |
| GO:0080090 | regulation of primary metabolic process | 0.168 |
| GO:0010468 | regulation of gene expression | 0.165 |
| GO:2000112 | regulation of cellular macromolecule biosynthetic process | 0.165 |
| GO:0031323 | regulation of cellular metabolic process | 0.164 |
| GO:0016020 | membrane | 0.162 |
| GO:0031326 | regulation of cellular biosynthetic process | 0.162 |
| GO:0051171 | regulation of nitrogen compound metabolic process | 0.161 |
| GO:0009889 | regulation of biosynthetic process | 0.16 |
| GO:0019219 | regulation of nucleobase-containing compound metabolic process | 0.155 |
| GO:0019222 | regulation of metabolic process | 0.154 |
| GO:0009057 | macromolecule catabolic process | 0.154 |
| GO:0005829 | cytosol | 0.151 |
| GO:0000956 | nuclear-transcribed mRNA catabolic process | 0.149 |
| GO:0006996 | organelle organization | 0.149 |
| GO:0043231 | intracellular membrane-bounded organelle | 0.148 |
| GO:0051276 | chromosome organization | 0.148 |
| GO:0006402 | mRNA catabolic process | 0.146 |
| GO:0006401 | RNA catabolic process | 0.143 |
| GO:0006396 | RNA processing | 0.141 |
| GO:0000184 | nuclear-transcribed mRNA catabolic process, nonsense-mediated decay | 0.134 |
| GO:0043933 | macromolecular complex subunit organization | 0.134 |
| GO:0006413 | translational initiation | 0.133 |
| GO:0006415 | translational termination | 0.131 |
| GO:0032984 | macromolecular complex disassembly | 0.126 |
| GO:0043241 | protein complex disassembly | 0.126 |
| GO:0005694 | chromosome | 0.124 |
| GO:0034655 | nucleobase-containing compound catabolic process | 0.124 |
| GO:0006974 | cellular response to DNA damage stimulus | 0.123 |
| GO:0022626 | cytosolic ribosome | 0.122 |
| GO:0043624 | cellular protein complex disassembly | 0.12 |
| GO:0042254 | ribosome biogenesis | 0.119 |
| GO:0019080 | viral gene expression | 0.118 |
| GO:0044033 | multi-organism metabolic process | 0.118 |
| GO:0044270 | cellular nitrogen compound catabolic process | 0.115 |
| GO:0046700 | heterocycle catabolic process | 0.114 |
| GO:0006414 | translational elongation | 0.113 |
| GO:0019439 | aromatic compound catabolic process | 0.112 |
| GO:0044427 | chromosomal part | 0.111 |
| GO:0006614 | SRP-dependent cotranslational protein targeting to membrane | 0.11 |
| GO:0022411 | cellular component disassembly | 0.11 |
| GO:0045047 | protein targeting to ER | 0.109 |
| GO:0072599 | establishment of protein localization to endoplasmic reticulum | 0.109 |
| GO:0044445 | cytosolic part | 0.108 |
| GO:0006281 | DNA repair | 0.108 |
| GO:0006613 | cotranslational protein targeting to membrane | 0.108 |
| hsa03010 | Ribosome | 0.107 |
| GO:0016604 | nuclear body | 0.107 |
| GO:1901361 | organic cyclic compound catabolic process | 0.106 |
| GO:0019058 | viral life cycle | 0.106 |
| GO:1990234 | transferase complex | 0.105 |
| GO:0044391 | ribosomal subunit | 0.103 |
| GO:0006259 | DNA metabolic process | 0.102 |
| GO:0070972 | protein localization to endoplasmic reticulum | 0.094 |
| GO:0008150 | biological_process | 0.092 |
| GO:0033554 | cellular response to stress | 0.081 |
| GO:0000375 | RNA splicing, via transesterification reactions | 0.071 |
| GO:0006412 | translation | 0.061 |
| GO:0044710 | single-organism metabolic process | 0.051 |
| GO:0051252 | regulation of RNA metabolic process | 0.047 |
