# Supplementary material for: Analysis of Protein–Protein Functional Associations by Using Gene Ontology and KEGG Pathway
Source: Biomed Res Int. 2019 Jul 18;2019:4963289. doi: 10.1155/2019/4963289 (PMC6668538; doi:10.1155/2019/4963289)
Supplement: Supplementary 1 — 70392 protein–protein functional associations. [file 4963289.f1.pdf]

Supplementary Matertal S1. 70392 protein-protein functional associations

Ensembl ID of protein 1	Ensembl ID of protein 2
ENSP00000001008	ENSP00000206249
ENSP00000001008	ENSP00000216274
ENSP00000001008	ENSP00000217244
ENSP00000001008	ENSP00000222247
ENSP00000001008	ENSP00000222567
ENSP00000001008	ENSP00000234590
ENSP00000001008	ENSP00000242057
ENSP00000001008	ENSP00000246802
ENSP00000001008	ENSP00000247970
ENSP00000001008	ENSP00000252487
ENSP00000001008	ENSP00000254940
ENSP00000001008	ENSP00000263038
ENSP00000001008	ENSP00000265963
ENSP00000001008	ENSP00000265970
ENSP00000001008	ENSP00000270225
ENSP00000001008	ENSP00000287878
ENSP00000001008	ENSP00000300591
ENSP00000001008	ENSP00000305958
ENSP00000001008	ENSP00000306999
ENSP00000001008	ENSP00000313050
ENSP00000001008	ENSP00000316854
ENSP00000001008	ENSP00000317992
ENSP00000001008	ENSP00000322396
ENSP00000001008	ENSP00000322570
ENSP00000001008	ENSP00000329466
ENSP00000001008	ENSP00000332513
ENSP00000001008	ENSP00000338862
ENSP00000001008	ENSP00000338990
ENSP00000001008	ENSP00000339527
ENSP00000001008	ENSP00000340237
ENSP00000001008	ENSP00000340820
ENSP00000001008	ENSP00000348565
ENSP00000001008	ENSP00000351141
ENSP00000001008	ENSP00000351697
ENSP00000001008	ENSP00000352928
ENSP00000001008	ENSP00000354040
ENSP00000001008	ENSP00000354812
ENSP00000001008	ENSP00000359345
ENSP00000001008	ENSP00000359385
ENSP00000001008	ENSP00000359688
ENSP00000001008	ENSP00000360412
ENSP00000001008	ENSP00000361640

ENSP00000001008	ENSP00000363384
ENSP00000001008	ENSP00000368349
ENSP00000001008	ENSP00000370376
ENSP00000001008	ENSP00000380982
ENSP00000001008	ENSP00000381657
ENSP00000001008	ENSP00000382392
ENSP00000001008	ENSP00000402338
ENSP00000003084	ENSP00000053867
ENSP00000003084	ENSP00000216455
ENSP00000003084	ENSP00000216479
ENSP00000003084	ENSP00000217244
ENSP00000003084	ENSP00000219473
ENSP00000003084	ENSP00000219548
ENSP00000003084	ENSP00000234160
ENSP00000003084	ENSP00000241052
ENSP00000003084	ENSP00000243253
ENSP00000003084	ENSP00000249647
ENSP00000003084	ENSP00000254301
ENSP00000003084	ENSP00000259512
ENSP00000003084	ENSP00000261247
ENSP00000003084	ENSP00000261514
ENSP00000003084	ENSP00000262394
ENSP00000003084	ENSP00000264380
ENSP00000003084	ENSP00000265175
ENSP00000003084	ENSP00000265316
ENSP00000003084	ENSP00000265717
ENSP00000003084	ENSP00000281537
ENSP00000003084	ENSP00000287295
ENSP00000003084	ENSP00000290649
ENSP00000003084	ENSP00000296084
ENSP00000003084	ENSP00000303507
ENSP00000003084	ENSP00000305958
ENSP00000003084	ENSP00000306124
ENSP00000003084	ENSP00000308236
ENSP00000003084	ENSP00000311344
ENSP00000003084	ENSP00000311873
ENSP00000003084	ENSP00000312235
ENSP00000003084	ENSP00000312506
ENSP00000003084	ENSP00000313391
ENSP00000003084	ENSP00000327107
ENSP00000003084	ENSP00000334061
ENSP00000003084	ENSP00000340019
ENSP00000003084	ENSP00000340210
ENSP00000003084	ENSP00000345530
ENSP00000003084	ENSP00000347778
ENSP00000003084	ENSP00000356969

ENSP00000003084	ENSP00000357283
ENSP00000003084	ENSP00000357484
ENSP00000003084	ENSP00000358857
ENSP00000003084	ENSP00000381045
ENSP00000003084	ENSP00000381863
ENSP00000003084	ENSP00000392330
ENSP00000003100	ENSP00000219368
ENSP00000003100	ENSP00000220584
ENSP00000003100	ENSP00000227524
ENSP00000003100	ENSP00000243253
ENSP00000003100	ENSP00000254521
ENSP00000003100	ENSP00000255389
ENSP00000003100	ENSP00000256319
ENSP00000003100	ENSP00000261507
ENSP00000003100	ENSP00000261942
ENSP00000003100	ENSP00000264027
ENSP00000003100	ENSP00000265896
ENSP00000003100	ENSP00000272163
ENSP00000003100	ENSP00000276014
ENSP00000003100	ENSP00000277010
ENSP00000003100	ENSP00000312458
ENSP00000003100	ENSP00000320604
ENSP00000003100	ENSP00000325425
ENSP00000003100	ENSP00000325677
ENSP00000003100	ENSP00000327716
ENSP00000003100	ENSP00000330812
ENSP00000003100	ENSP00000336724
ENSP00000003100	ENSP00000340896
ENSP00000003100	ENSP00000341662
ENSP00000003100	ENSP00000344460
ENSP00000003100	ENSP00000348762
ENSP00000003100	ENSP00000349877
ENSP00000003100	ENSP00000355955
ENSP00000003100	ENSP00000360918
ENSP00000003100	ENSP00000373918
ENSP00000003302	ENSP00000007516
ENSP00000003302	ENSP00000156471
ENSP00000003302	ENSP00000255194
ENSP00000003302	ENSP00000261601
ENSP00000003302	ENSP00000261881
ENSP00000003302	ENSP00000281708
ENSP00000003302	ENSP00000290158
ENSP00000003302	ENSP00000312995
ENSP00000003302	ENSP00000313490
ENSP00000003302	ENSP00000330343
ENSP00000003302	ENSP00000345530

ENSP00000003302	ENSP00000356405
ENSP00000003302	ENSP00000365588
ENSP00000003302	ENSP00000366746
ENSP00000003302	ENSP00000371475
ENSP00000003302	ENSP00000373060
ENSP00000003302	ENSP00000378529
ENSP00000003302	ENSP00000379228
ENSP00000003302	ENSP00000413520
ENSP00000005226	ENSP00000252597
ENSP00000005226	ENSP00000320076
ENSP00000005226	ENSP00000354951
ENSP00000005340	ENSP00000212015
ENSP00000005340	ENSP00000216044
ENSP00000005340	ENSP00000217244
ENSP00000005340	ENSP00000217964
ENSP00000005340	ENSP00000219473
ENSP00000005340	ENSP00000230419
ENSP00000005340	ENSP00000243878
ENSP00000005340	ENSP00000251871
ENSP00000005340	ENSP00000252137
ENSP00000005340	ENSP00000254250
ENSP00000005340	ENSP00000255764
ENSP00000005340	ENSP00000256379
ENSP00000005340	ENSP00000256474
ENSP00000005340	ENSP00000257430
ENSP00000005340	ENSP00000258648
ENSP00000005340	ENSP00000261531
ENSP00000005340	ENSP00000262320
ENSP00000005340	ENSP00000262457
ENSP00000005340	ENSP00000262643
ENSP00000005340	ENSP00000263121
ENSP00000005340	ENSP00000263205
ENSP00000005340	ENSP00000264160
ENSP00000005340	ENSP00000265026
ENSP00000005340	ENSP00000265175
ENSP00000005340	ENSP00000265350
ENSP00000005340	ENSP00000266126
ENSP00000005340	ENSP00000267064
ENSP00000005340	ENSP00000268459
ENSP00000005340	ENSP00000277541
ENSP00000005340	ENSP00000281928
ENSP00000005340	ENSP00000286317
ENSP00000005340	ENSP00000290330
ENSP00000005340	ENSP00000290663
ENSP00000005340	ENSP00000292035
ENSP00000005340	ENSP00000293777

ENSP00000005340	ENSP00000296084
ENSP00000005340	ENSP00000296215
ENSP00000005340	ENSP00000296550
ENSP00000005340	ENSP00000297347
ENSP00000005340	ENSP00000300574
ENSP00000005340	ENSP00000301599
ENSP00000005340	ENSP00000303315
ENSP00000005340	ENSP00000307863
ENSP00000005340	ENSP00000308339
ENSP00000005340	ENSP00000309117
ENSP00000005340	ENSP00000310800
ENSP00000005340	ENSP00000311005
ENSP00000005340	ENSP00000311825
ENSP00000005340	ENSP00000313391
ENSP00000005340	ENSP00000315379
ENSP00000005340	ENSP00000315859
ENSP00000005340	ENSP00000317985
ENSP00000005340	ENSP00000319501
ENSP00000005340	ENSP00000322885
ENSP00000005340	ENSP00000323720
ENSP00000005340	ENSP00000325612
ENSP00000005340	ENSP00000326128
ENSP00000005340	ENSP00000326767
ENSP00000005340	ENSP00000334003
ENSP00000005340	ENSP00000337439
ENSP00000005340	ENSP00000343129
ENSP00000005340	ENSP00000344456
ENSP00000005340	ENSP00000345464
ENSP00000005340	ENSP00000348550
ENSP00000005340	ENSP00000349640
ENSP00000005340	ENSP00000352514
ENSP00000005340	ENSP00000354911
ENSP00000005340	ENSP00000355296
ENSP00000005340	ENSP00000355760
ENSP00000005340	ENSP00000357047
ENSP00000005340	ENSP00000357283
ENSP00000005340	ENSP00000357484
ENSP00000005340	ENSP00000357907
ENSP00000005340	ENSP00000358081
ENSP00000005340	ENSP00000360371
ENSP00000005340	ENSP00000362948
ENSP00000005340	ENSP00000363571
ENSP00000005340	ENSP00000364860
ENSP00000005340	ENSP00000365530
ENSP00000005340	ENSP00000367069
ENSP00000005340	ENSP00000367638

ENSP00000005340	ENSP00000367841
ENSP00000005340	ENSP00000368678
ENSP00000005340	ENSP00000368976
ENSP00000005340	ENSP00000374455
ENSP00000005340	ENSP00000378180
ENSP00000005340	ENSP00000381216
ENSP00000005340	ENSP00000381795
ENSP00000005340	ENSP00000382423
ENSP00000005340	ENSP00000383623
ENSP00000005340	ENSP00000384040
ENSP00000005340	ENSP00000384048
ENSP00000005340	ENSP00000392617
ENSP00000005340	ENSP00000396771
ENSP00000005340	ENSP00000396976
ENSP00000005340	ENSP00000399511
ENSP00000005340	ENSP00000402240
ENSP00000005386	ENSP00000202017
ENSP00000005386	ENSP00000215587
ENSP00000005386	ENSP00000216297
ENSP00000005386	ENSP00000251038
ENSP00000005386	ENSP00000262265
ENSP00000005386	ENSP00000262319
ENSP00000005386	ENSP00000263857
ENSP00000005386	ENSP00000264718
ENSP00000005386	ENSP00000276603
ENSP00000005386	ENSP00000295121
ENSP00000005386	ENSP00000312735
ENSP00000005386	ENSP00000314949
ENSP00000005386	ENSP00000335321
ENSP00000005386	ENSP00000337393
ENSP00000005386	ENSP00000337797
ENSP00000005386	ENSP00000339109
ENSP00000005386	ENSP00000345530
ENSP00000005386	ENSP00000353971
ENSP00000005386	ENSP00000354558
ENSP00000005386	ENSP00000356989
ENSP00000005386	ENSP00000362546
ENSP00000005386	ENSP00000363734
ENSP00000005386	ENSP00000364289
ENSP00000005386	ENSP00000370258
ENSP00000005386	ENSP00000370589
ENSP00000005386	ENSP00000376097
ENSP00000005386	ENSP00000382779
ENSP00000005386	ENSP00000384048
ENSP00000005386	ENSP00000385722
ENSP00000006101	ENSP00000222547

ENSP00000006101	ENSP00000225567
ENSP00000006101	ENSP00000262812
ENSP00000006101	ENSP00000264028
ENSP00000006101	ENSP00000310521
ENSP00000006101	ENSP00000312735
ENSP00000006101	ENSP00000325002
ENSP00000006101	ENSP00000328079
ENSP00000006101	ENSP00000329419
ENSP00000006101	ENSP00000344055
ENSP00000006101	ENSP00000347408
ENSP00000006101	ENSP00000356530
ENSP00000006101	ENSP00000357048
ENSP00000006101	ENSP00000401445
ENSP00000006275	ENSP00000222008
ENSP00000006275	ENSP00000283351
ENSP00000006275	ENSP00000291574
ENSP00000006275	ENSP00000302783
ENSP00000006275	ENSP00000305459
ENSP00000006275	ENSP00000308496
ENSP00000006275	ENSP00000312318
ENSP00000006275	ENSP00000314615
ENSP00000006275	ENSP00000316990
ENSP00000006275	ENSP00000336606
ENSP00000006275	ENSP00000343246
ENSP00000006275	ENSP00000352708
ENSP00000006275	ENSP00000359000
ENSP00000006275	ENSP00000360183
ENSP00000006275	ENSP00000373979
ENSP00000006275	ENSP00000376871
ENSP00000006275	ENSP00000397441
ENSP00000006275	ENSP00000420405
ENSP00000006658	ENSP00000225916
ENSP00000006658	ENSP00000264951
ENSP00000006658	ENSP00000347733
ENSP00000007264	ENSP00000229395
ENSP00000007264	ENSP00000230056
ENSP00000007264	ENSP00000237283
ENSP00000007264	ENSP00000267807
ENSP00000007264	ENSP00000271715
ENSP00000007264	ENSP00000295951
ENSP00000007264	ENSP00000307567
ENSP00000007264	ENSP00000323880
ENSP00000007264	ENSP00000336587
ENSP00000007264	ENSP00000355890
ENSP00000007264	ENSP00000362578
ENSP00000007390	ENSP00000229214

ENSP00000007390	ENSP00000234626
ENSP00000007390	ENSP00000251038
ENSP00000007390	ENSP00000260129
ENSP00000007390	ENSP00000263657
ENSP00000007390	ENSP00000325448
ENSP00000007390	ENSP00000391944
ENSP00000007516	ENSP00000184266
ENSP00000007516	ENSP00000215565
ENSP00000007516	ENSP00000216194
ENSP00000007516	ENSP00000233190
ENSP00000007516	ENSP00000233627
ENSP00000007516	ENSP00000237889
ENSP00000007516	ENSP00000242210
ENSP00000007516	ENSP00000247866
ENSP00000007516	ENSP00000251038
ENSP00000007516	ENSP00000252102
ENSP00000007516	ENSP00000252711
ENSP00000007516	ENSP00000259037
ENSP00000007516	ENSP00000260956
ENSP00000007516	ENSP00000261643
ENSP00000007516	ENSP00000262430
ENSP00000007516	ENSP00000262764
ENSP00000007516	ENSP00000263774
ENSP00000007516	ENSP00000265276
ENSP00000007516	ENSP00000266544
ENSP00000007516	ENSP00000268668
ENSP00000007516	ENSP00000274137
ENSP00000007516	ENSP00000276689
ENSP00000007516	ENSP00000278618
ENSP00000007516	ENSP00000280701
ENSP00000007516	ENSP00000281031
ENSP00000007516	ENSP00000283429
ENSP00000007516	ENSP00000290429
ENSP00000007516	ENSP00000296684
ENSP00000007516	ENSP00000299166
ENSP00000007516	ENSP00000301457
ENSP00000007516	ENSP00000304783
ENSP00000007516	ENSP00000309463
ENSP00000007516	ENSP00000311740
ENSP00000007516	ENSP00000312370
ENSP00000007516	ENSP00000315774
ENSP00000007516	ENSP00000317177
ENSP00000007516	ENSP00000318646
ENSP00000007516	ENSP00000322450
ENSP00000007516	ENSP00000327070
ENSP00000007516	ENSP00000327268

ENSP00000007516	ENSP00000330737
ENSP00000007516	ENSP00000330787
ENSP00000007516	ENSP00000330945
ENSP00000007516	ENSP00000341243
ENSP00000007516	ENSP00000342793
ENSP00000007516	ENSP00000346196
ENSP00000007516	ENSP00000347778
ENSP00000007516	ENSP00000347988
ENSP00000007516	ENSP00000354687
ENSP00000007516	ENSP00000354813
ENSP00000007516	ENSP00000354876
ENSP00000007516	ENSP00000354961
ENSP00000007516	ENSP00000355046
ENSP00000007516	ENSP00000355206
ENSP00000007516	ENSP00000355384
ENSP00000007516	ENSP00000355607
ENSP00000007516	ENSP00000356972
ENSP00000007516	ENSP00000360492
ENSP00000007516	ENSP00000362058
ENSP00000007516	ENSP00000362873
ENSP00000007516	ENSP00000363205
ENSP00000007516	ENSP00000369176
ENSP00000007516	ENSP00000370930
ENSP00000007516	ENSP00000377770
ENSP00000007516	ENSP00000379607
ENSP00000007516	ENSP00000387262
ENSP00000007516	ENSP00000392985
ENSP00000007516	ENSP00000398290
ENSP00000007516	ENSP00000409315
ENSP00000009530	ENSP00000310832
ENSP00000009530	ENSP00000363976
ENSP00000009530	ENSP00000370023
ENSP00000009530	ENSP00000378723
ENSP00000009530	ENSP00000393566
ENSP00000009589	ENSP00000196551
ENSP00000009589	ENSP00000199320
ENSP00000009589	ENSP00000202773
ENSP00000009589	ENSP00000206249
ENSP00000009589	ENSP00000211372
ENSP00000009589	ENSP00000216554
ENSP00000009589	ENSP00000222008
ENSP00000009589	ENSP00000222247
ENSP00000009589	ENSP00000225430
ENSP00000009589	ENSP00000227524
ENSP00000009589	ENSP00000230050
ENSP00000009589	ENSP00000230340

ENSP00000009589	ENSP00000232905
ENSP00000009589	ENSP00000241600
ENSP00000009589	ENSP00000245539
ENSP00000009589	ENSP00000248450
ENSP00000009589	ENSP00000251038
ENSP00000009589	ENSP00000253108
ENSP00000009589	ENSP00000253233
ENSP00000009589	ENSP00000253788
ENSP00000009589	ENSP00000254037
ENSP00000009589	ENSP00000258169
ENSP00000009589	ENSP00000258455
ENSP00000009589	ENSP00000260102
ENSP00000009589	ENSP00000268206
ENSP00000009589	ENSP00000270625
ENSP00000009589	ENSP00000272418
ENSP00000009589	ENSP00000274606
ENSP00000009589	ENSP00000278572
ENSP00000009589	ENSP00000280557
ENSP00000009589	ENSP00000283109
ENSP00000009589	ENSP00000287878
ENSP00000009589	ENSP00000295930
ENSP00000009589	ENSP00000296674
ENSP00000009589	ENSP00000296802
ENSP00000009589	ENSP00000296805
ENSP00000009589	ENSP00000297338
ENSP00000009589	ENSP00000297908
ENSP00000009589	ENSP00000301585
ENSP00000009589	ENSP00000301729
ENSP00000009589	ENSP00000302896
ENSP00000009589	ENSP00000311028
ENSP00000009589	ENSP00000317376
ENSP00000009589	ENSP00000318646
ENSP00000009589	ENSP00000319977
ENSP00000009589	ENSP00000320658
ENSP00000009589	ENSP00000323687
ENSP00000009589	ENSP00000323856
ENSP00000009589	ENSP00000326981
ENSP00000009589	ENSP00000329715
ENSP00000009589	ENSP00000331019
ENSP00000009589	ENSP00000336702
ENSP00000009589	ENSP00000337127
ENSP00000009589	ENSP00000339095
ENSP00000009589	ENSP00000340278
ENSP00000009589	ENSP00000341885
ENSP00000009589	ENSP00000345096
ENSP00000009589	ENSP00000345156

ENSP00000009589	ENSP00000346015
ENSP00000009589	ENSP00000346022
ENSP00000009589	ENSP00000346050
ENSP00000009589	ENSP00000346067
ENSP00000009589	ENSP00000346725
ENSP00000009589	ENSP00000352308
ENSP00000009589	ENSP00000352706
ENSP00000009589	ENSP00000353059
ENSP00000009589	ENSP00000355471
ENSP00000009589	ENSP00000356134
ENSP00000009589	ENSP00000358120
ENSP00000009589	ENSP00000358812
ENSP00000009589	ENSP00000361076
ENSP00000009589	ENSP00000362036
ENSP00000009589	ENSP00000362576
ENSP00000009589	ENSP00000362688
ENSP00000009589	ENSP00000362744
ENSP00000009589	ENSP00000363308
ENSP00000009589	ENSP00000363676
ENSP00000009589	ENSP00000364119
ENSP00000009589	ENSP00000364320
ENSP00000009589	ENSP00000364883
ENSP00000009589	ENSP00000365588
ENSP00000009589	ENSP00000368790
ENSP00000009589	ENSP00000369757
ENSP00000009589	ENSP00000370023
ENSP00000009589	ENSP00000372199
ENSP00000009589	ENSP00000372445
ENSP00000009589	ENSP00000373060
ENSP00000009589	ENSP00000379339
ENSP00000009589	ENSP00000379607
ENSP00000009589	ENSP00000379888
ENSP00000009589	ENSP00000380718
ENSP00000009589	ENSP00000384048
ENSP00000009589	ENSP00000387429
ENSP00000009589	ENSP00000393241
ENSP00000009589	ENSP00000396484
ENSP00000009589	ENSP00000402338
ENSP00000009589	ENSP00000403175
ENSP00000009589	ENSP00000404318
ENSP00000009589	ENSP00000409167
ENSP00000009589	ENSP00000412583
ENSP00000009589	ENSP00000414321
ENSP00000009589	ENSP00000415212
ENSP00000009589	ENSP00000416110
ENSP00000009589	ENSP00000416511

ENSP00000009589	ENSP00000417281
ENSP00000011619	ENSP00000257430
ENSP00000011619	ENSP00000266069
ENSP00000011619	ENSP00000268719
ENSP00000011619	ENSP00000278715
ENSP00000011619	ENSP00000294008
ENSP00000011619	ENSP00000295408
ENSP00000011619	ENSP00000301962
ENSP00000011619	ENSP00000302830
ENSP00000011619	ENSP00000304226
ENSP00000011619	ENSP00000313059
ENSP00000011619	ENSP00000315859
ENSP00000011619	ENSP00000320623
ENSP00000011619	ENSP00000323527
ENSP00000011619	ENSP00000347087
ENSP00000011619	ENSP00000351221
ENSP00000011619	ENSP00000354201
ENSP00000011619	ENSP00000355388
ENSP00000011619	ENSP00000355596
ENSP00000011619	ENSP00000356405
ENSP00000011619	ENSP00000357711
ENSP00000011619	ENSP00000364486
ENSP00000011619	ENSP00000377865
ENSP00000011619	ENSP00000384675
ENSP00000011619	ENSP00000406490
ENSP00000011653	ENSP00000204566
ENSP00000011653	ENSP00000219548
ENSP00000011653	ENSP00000221132
ENSP00000011653	ENSP00000234590
ENSP00000011653	ENSP00000273067
ENSP00000011653	ENSP00000283635
ENSP00000011653	ENSP00000290158
ENSP00000011653	ENSP00000291009
ENSP00000011653	ENSP00000296930
ENSP00000011653	ENSP00000298229
ENSP00000011653	ENSP00000302896
ENSP00000011653	ENSP00000302935
ENSP00000011653	ENSP00000318195
ENSP00000011653	ENSP00000325589
ENSP00000011653	ENSP00000331201
ENSP00000011653	ENSP00000339795
ENSP00000011653	ENSP00000340019
ENSP00000011653	ENSP00000342681
ENSP00000011653	ENSP00000346067
ENSP00000011653	ENSP00000346550
ENSP00000011653	ENSP00000354119

ENSP00000011653	ENSP00000354883
ENSP00000011653	ENSP00000356346
ENSP00000011653	ENSP00000358490
ENSP00000011653	ENSP00000359206
ENSP00000011653	ENSP00000360798
ENSP00000011653	ENSP00000363548
ENSP00000011653	ENSP00000364898
ENSP00000011653	ENSP00000369293
ENSP00000011653	ENSP00000393566
ENSP00000012049	ENSP00000362306
ENSP00000012049	ENSP00000367029
ENSP00000012049	ENSP00000386394
ENSP00000012134	ENSP00000254325
ENSP00000012134	ENSP00000262643
ENSP00000012134	ENSP00000264335
ENSP00000012134	ENSP00000313059
ENSP00000012134	ENSP00000341243
ENSP00000012134	ENSP00000359206
ENSP00000012134	ENSP00000364912
ENSP00000013070	ENSP00000263805
ENSP00000013807	ENSP00000055682
ENSP00000013807	ENSP00000217173
ENSP00000013807	ENSP00000219066
ENSP00000013807	ENSP00000219548
ENSP00000013807	ENSP00000225504
ENSP00000013807	ENSP00000231790
ENSP00000013807	ENSP00000233146
ENSP00000013807	ENSP00000234420
ENSP00000013807	ENSP00000247815
ENSP00000013807	ENSP00000251303
ENSP00000013807	ENSP00000252108
ENSP00000013807	ENSP00000254719
ENSP00000013807	ENSP00000256854
ENSP00000013807	ENSP00000261424
ENSP00000013807	ENSP00000263187
ENSP00000013807	ENSP00000263974
ENSP00000013807	ENSP00000264161
ENSP00000013807	ENSP00000264926
ENSP00000013807	ENSP00000265081
ENSP00000013807	ENSP00000265849
ENSP00000013807	ENSP00000265963
ENSP00000013807	ENSP00000265970
ENSP00000013807	ENSP00000268043
ENSP00000013807	ENSP00000278412
ENSP00000013807	ENSP00000278616
ENSP00000013807	ENSP00000294008

ENSP00000013807	ENSP00000296273
ENSP00000013807	ENSP00000299424
ENSP00000013807	ENSP00000301764
ENSP00000013807	ENSP00000302111
ENSP00000013807	ENSP00000305790
ENSP00000013807	ENSP00000307853
ENSP00000013807	ENSP00000308944
ENSP00000013807	ENSP00000310520
ENSP00000013807	ENSP00000311873
ENSP00000013807	ENSP00000313171
ENSP00000013807	ENSP00000313420
ENSP00000013807	ENSP00000314949
ENSP00000013807	ENSP00000315925
ENSP00000013807	ENSP00000318977
ENSP00000013807	ENSP00000322570
ENSP00000013807	ENSP00000323439
ENSP00000013807	ENSP00000325863
ENSP00000013807	ENSP00000328940
ENSP00000013807	ENSP00000330343
ENSP00000013807	ENSP00000336606
ENSP00000013807	ENSP00000336856
ENSP00000013807	ENSP00000337127
ENSP00000013807	ENSP00000337353
ENSP00000013807	ENSP00000343001
ENSP00000013807	ENSP00000343741
ENSP00000013807	ENSP00000344537
ENSP00000013807	ENSP00000347232
ENSP00000013807	ENSP00000347978
ENSP00000013807	ENSP00000348020
ENSP00000013807	ENSP00000348089
ENSP00000013807	ENSP00000351284
ENSP00000013807	ENSP00000351697
ENSP00000013807	ENSP00000352257
ENSP00000013807	ENSP00000353575
ENSP00000013807	ENSP00000354522
ENSP00000013807	ENSP00000354558
ENSP00000013807	ENSP00000354883
ENSP00000013807	ENSP00000354957
ENSP00000013807	ENSP00000355518
ENSP00000013807	ENSP00000356989
ENSP00000013807	ENSP00000357402
ENSP00000013807	ENSP00000358478
ENSP00000013807	ENSP00000360532
ENSP00000013807	ENSP00000362441
ENSP00000013807	ENSP00000364126
ENSP00000013807	ENSP00000364270

ENSP00000013807	ENSP00000367910
ENSP00000013807	ENSP00000368349
ENSP00000013807	ENSP00000369411
ENSP00000013807	ENSP00000370151
ENSP00000013807	ENSP00000380504
ENSP00000013807	ENSP00000394649
ENSP00000013807	ENSP00000406490
ENSP00000013807	ENSP00000406868
ENSP00000013807	ENSP00000407047
ENSP00000016171	ENSP00000251038
ENSP00000016171	ENSP00000255390
ENSP00000016171	ENSP00000261643
ENSP00000016171	ENSP00000268379
ENSP00000016171	ENSP00000299335
ENSP00000016171	ENSP00000326579
ENSP00000016171	ENSP00000346015
ENSP00000016171	ENSP00000353073
ENSP00000016171	ENSP00000360782
ENSP00000016171	ENSP00000361042
ENSP00000016171	ENSP00000369335
ENSP00000016171	ENSP00000387262
ENSP00000019317	ENSP00000162749
ENSP00000019317	ENSP00000222792
ENSP00000019317	ENSP00000232458
ENSP00000019317	ENSP00000242719
ENSP00000019317	ENSP00000249923
ENSP00000019317	ENSP00000255194
ENSP00000019317	ENSP00000256442
ENSP00000019317	ENSP00000256658
ENSP00000019317	ENSP00000258062
ENSP00000019317	ENSP00000263795
ENSP00000019317	ENSP00000301838
ENSP00000019317	ENSP00000311005
ENSP00000019317	ENSP00000314499
ENSP00000019317	ENSP00000316845
ENSP00000019317	ENSP00000322957
ENSP00000019317	ENSP00000334836
ENSP00000019317	ENSP00000344055
ENSP00000019317	ENSP00000347169
ENSP00000019317	ENSP00000348349
ENSP00000019317	ENSP00000348550
ENSP00000019317	ENSP00000349824
ENSP00000019317	ENSP00000349959
ENSP00000019317	ENSP00000350199
ENSP00000019317	ENSP00000351926
ENSP00000019317	ENSP00000352603

ENSP00000019317	ENSP00000354927
ENSP00000019317	ENSP00000359067
ENSP00000019317	ENSP00000365005
ENSP00000019317	ENSP00000366121
ENSP00000019317	ENSP00000379612
ENSP00000019317	ENSP00000379709
ENSP00000019317	ENSP00000386456
ENSP00000019317	ENSP00000391249
ENSP00000019317	ENSP00000394794
ENSP00000020926	ENSP00000216492
ENSP00000020926	ENSP00000368244
ENSP00000023939	ENSP00000270708
ENSP00000023939	ENSP00000368438
ENSP00000025008	ENSP00000240079
ENSP00000025008	ENSP00000254101
ENSP00000025008	ENSP00000254719
ENSP00000025008	ENSP00000256474
ENSP00000025008	ENSP00000258947
ENSP00000025008	ENSP00000263121
ENSP00000025008	ENSP00000288199
ENSP00000025008	ENSP00000288828
ENSP00000025008	ENSP00000296679
ENSP00000025008	ENSP00000315702
ENSP00000025008	ENSP00000324560
ENSP00000025008	ENSP00000329967
ENSP00000025008	ENSP00000334675
ENSP00000025008	ENSP00000338990
ENSP00000025008	ENSP00000341189
ENSP00000025008	ENSP00000349785
ENSP00000025008	ENSP00000351250
ENSP00000025008	ENSP00000351908
ENSP00000025008	ENSP00000353375
ENSP00000025008	ENSP00000354777
ENSP00000025008	ENSP00000357907
ENSP00000025008	ENSP00000363205
ENSP00000025008	ENSP00000367029
ENSP00000025008	ENSP00000367797
ENSP00000025008	ENSP00000369339
ENSP00000025008	ENSP00000385025
ENSP00000025008	ENSP00000392896
ENSP00000025301	ENSP00000265717
ENSP00000025301	ENSP00000294008
ENSP00000026218	ENSP00000216452
ENSP00000026218	ENSP00000251968
ENSP00000026218	ENSP00000278616
ENSP00000026218	ENSP00000310842

ENSP00000026218	ENSP00000312458
ENSP00000026218	ENSP00000354560
ENSP00000026218	ENSP00000369820
ENSP00000026218	ENSP00000420037
ENSP00000035383	ENSP00000252699
ENSP00000035383	ENSP00000258418
ENSP00000035383	ENSP00000273666
ENSP00000035383	ENSP00000307134
ENSP00000035383	ENSP00000312318
ENSP00000035383	ENSP00000339952
ENSP00000035383	ENSP00000368824
ENSP00000037502	ENSP00000162749
ENSP00000037502	ENSP00000234590
ENSP00000037502	ENSP00000252999
ENSP00000037502	ENSP00000254301
ENSP00000037502	ENSP00000257879
ENSP00000037502	ENSP00000261424
ENSP00000037502	ENSP00000302830
ENSP00000037502	ENSP00000322273
ENSP00000037502	ENSP00000336927
ENSP00000037502	ENSP00000339933
ENSP00000037502	ENSP00000346839
ENSP00000037869	ENSP00000310596
ENSP00000039007	ENSP00000225916
ENSP00000039007	ENSP00000231706
ENSP00000039007	ENSP00000261783
ENSP00000039007	ENSP00000318318
ENSP00000039007	ENSP00000318351
ENSP00000039007	ENSP00000332444
ENSP00000039007	ENSP00000358423
ENSP00000039007	ENSP00000364986
ENSP00000040663	ENSP00000256897
ENSP00000040663	ENSP00000304668
ENSP00000040663	ENSP00000319531
ENSP00000040663	ENSP00000378350
ENSP00000040738	ENSP00000360916
ENSP00000040738	ENSP00000379607
ENSP00000040877	ENSP00000220669
ENSP00000040877	ENSP00000254803
ENSP00000040877	ENSP00000256216
ENSP00000040877	ENSP00000260102
ENSP00000040877	ENSP00000263629
ENSP00000040877	ENSP00000290429
ENSP00000040877	ENSP00000300648
ENSP00000040877	ENSP00000307491
ENSP00000040877	ENSP00000311984

ENSP00000040877	ENSP00000314441
ENSP00000040877	ENSP00000321711
ENSP00000040877	ENSP00000324956
ENSP00000040877	ENSP00000326981
ENSP00000040877	ENSP00000334538
ENSP00000040877	ENSP00000339532
ENSP00000040877	ENSP00000341082
ENSP00000040877	ENSP00000344460
ENSP00000040877	ENSP00000346015
ENSP00000040877	ENSP00000348877
ENSP00000040877	ENSP00000358812
ENSP00000040877	ENSP00000360286
ENSP00000040877	ENSP00000371155
ENSP00000040877	ENSP00000388275
ENSP00000043402	ENSP00000172229
ENSP00000043402	ENSP00000247271
ENSP00000043402	ENSP00000258418
ENSP00000043402	ENSP00000267484
ENSP00000043402	ENSP00000337838
ENSP00000043402	ENSP00000347451
ENSP00000043402	ENSP00000358059
ENSP00000046794	ENSP00000085219
ENSP00000046794	ENSP00000220597
ENSP00000046794	ENSP00000226951
ENSP00000046794	ENSP00000233154
ENSP00000046794	ENSP00000244007
ENSP00000046794	ENSP00000263881
ENSP00000046794	ENSP00000264122
ENSP00000046794	ENSP00000295756
ENSP00000046794	ENSP00000308774
ENSP00000046794	ENSP00000313059
ENSP00000046794	ENSP00000316460
ENSP00000046794	ENSP00000339186
ENSP00000046794	ENSP00000354119
ENSP00000046794	ENSP00000356346
ENSP00000046794	ENSP00000360916
ENSP00000046794	ENSP00000364898
ENSP00000046794	ENSP00000365891
ENSP00000046794	ENSP00000366746
ENSP00000046794	ENSP00000366936
ENSP00000053243	ENSP00000365048
ENSP00000053468	ENSP00000081029
ENSP00000053468	ENSP00000196551
ENSP00000053468	ENSP00000241600
ENSP00000053468	ENSP00000245539
ENSP00000053468	ENSP00000251038

ENSP00000053468	ENSP00000254037
ENSP00000053468	ENSP00000258455
ENSP00000053468	ENSP00000272418
ENSP00000053468	ENSP00000295491
ENSP00000053468	ENSP00000301585
ENSP00000053468	ENSP00000337127
ENSP00000053468	ENSP00000341692
ENSP00000053468	ENSP00000347087
ENSP00000053468	ENSP00000352706
ENSP00000053468	ENSP00000361206
ENSP00000053468	ENSP00000362036
ENSP00000053468	ENSP00000362208
ENSP00000053468	ENSP00000362576
ENSP00000053468	ENSP00000372445
ENSP00000053867	ENSP00000222254
ENSP00000053867	ENSP00000254101
ENSP00000053867	ENSP00000262238
ENSP00000053867	ENSP00000292432
ENSP00000053867	ENSP00000343246
ENSP00000053867	ENSP00000348234
ENSP00000053867	ENSP00000350249
ENSP00000053867	ENSP00000360060
ENSP00000053867	ENSP00000363827
ENSP00000053867	ENSP00000364814
ENSP00000053867	ENSP00000379607
ENSP00000054668	ENSP00000323516
ENSP00000054668	ENSP00000379607
ENSP00000054950	ENSP00000221566
ENSP00000054950	ENSP00000244769
ENSP00000054950	ENSP00000336702
ENSP00000054950	ENSP00000348163
ENSP00000054950	ENSP00000357998
ENSP00000054950	ENSP00000363055
ENSP00000054950	ENSP00000370557
ENSP00000054950	ENSP00000391944
ENSP00000055335	ENSP00000216392
ENSP00000055335	ENSP00000317904
ENSP00000055335	ENSP00000396452
ENSP00000055682	ENSP00000216367
ENSP00000055682	ENSP00000219066
ENSP00000055682	ENSP00000221486
ENSP00000055682	ENSP00000222673
ENSP00000055682	ENSP00000234626
ENSP00000055682	ENSP00000241436
ENSP00000055682	ENSP00000254942
ENSP00000055682	ENSP00000263642

ENSP00000055682	ENSP00000264926
ENSP00000055682	ENSP00000267430
ENSP00000055682	ENSP00000268124
ENSP00000055682	ENSP00000269214
ENSP00000055682	ENSP00000276072
ENSP00000055682	ENSP00000276603
ENSP00000055682	ENSP00000285021
ENSP00000055682	ENSP00000290949
ENSP00000055682	ENSP00000294053
ENSP00000055682	ENSP00000306999
ENSP00000055682	ENSP00000308944
ENSP00000055682	ENSP00000310520
ENSP00000055682	ENSP00000311873
ENSP00000055682	ENSP00000313350
ENSP00000055682	ENSP00000315700
ENSP00000055682	ENSP00000322570
ENSP00000055682	ENSP00000336606
ENSP00000055682	ENSP00000340305
ENSP00000055682	ENSP00000340879
ENSP00000055682	ENSP00000343392
ENSP00000055682	ENSP00000349393
ENSP00000055682	ENSP00000351284
ENSP00000055682	ENSP00000352257
ENSP00000055682	ENSP00000355185
ENSP00000055682	ENSP00000358576
ENSP00000055682	ENSP00000360116
ENSP00000055682	ENSP00000361310
ENSP00000055682	ENSP00000364126
ENSP00000055682	ENSP00000364270
ENSP00000055682	ENSP00000367527
ENSP00000055682	ENSP00000368349
ENSP00000055682	ENSP00000368438
ENSP00000055682	ENSP00000369335
ENSP00000055682	ENSP00000370150
ENSP00000055682	ENSP00000370151
ENSP00000055682	ENSP00000370222
ENSP00000055682	ENSP00000370376
ENSP00000055682	ENSP00000371475
ENSP00000055682	ENSP00000375809
ENSP00000055682	ENSP00000381657
ENSP00000056217	ENSP00000222308
ENSP00000056217	ENSP00000237380
ENSP00000056217	ENSP00000243776
ENSP00000056217	ENSP00000244007
ENSP00000056217	ENSP00000262643
ENSP00000056217	ENSP00000342112

ENSP00000056217	ENSP00000349877
ENSP00000062104	ENSP00000345464
ENSP00000064780	ENSP00000348278
ENSP00000070846	ENSP00000261590
ENSP00000070846	ENSP00000312356
ENSP00000070846	ENSP00000344456
ENSP00000070846	ENSP00000359531
ENSP00000072869	ENSP00000251038
ENSP00000072869	ENSP00000254759
ENSP00000072869	ENSP00000262507
ENSP00000072869	ENSP00000264951
ENSP00000072869	ENSP00000268124
ENSP00000072869	ENSP00000288532
ENSP00000072869	ENSP00000300452
ENSP00000072869	ENSP00000312587
ENSP00000072869	ENSP00000322316
ENSP00000072869	ENSP00000333946
ENSP00000072869	ENSP00000348877
ENSP00000072869	ENSP00000358033
ENSP00000072869	ENSP00000371607
ENSP00000078527	ENSP00000251038
ENSP00000081029	ENSP00000196551
ENSP00000081029	ENSP00000206249
ENSP00000081029	ENSP00000211372
ENSP00000081029	ENSP00000241600
ENSP00000081029	ENSP00000245539
ENSP00000081029	ENSP00000254037
ENSP00000081029	ENSP00000258455
ENSP00000081029	ENSP00000272418
ENSP00000081029	ENSP00000288937
ENSP00000081029	ENSP00000295491
ENSP00000081029	ENSP00000296509
ENSP00000081029	ENSP00000301585
ENSP00000081029	ENSP00000301962
ENSP00000081029	ENSP00000324560
ENSP00000081029	ENSP00000337127
ENSP00000081029	ENSP00000341692
ENSP00000081029	ENSP00000352706
ENSP00000081029	ENSP00000361206
ENSP00000081029	ENSP00000362036
ENSP00000081029	ENSP00000362208
ENSP00000081029	ENSP00000372445
ENSP00000081029	ENSP00000393241
ENSP00000081029	ENSP00000400312
ENSP00000081029	ENSP00000403175
ENSP00000081029	ENSP00000412583

ENSP00000081029	ENSP00000416110
ENSP00000083182	ENSP00000223500
ENSP00000083182	ENSP00000242338
ENSP00000083182	ENSP00000262306
ENSP00000083182	ENSP00000264198
ENSP00000083182	ENSP00000284811
ENSP00000083182	ENSP00000284981
ENSP00000083182	ENSP00000287152
ENSP00000083182	ENSP00000293883
ENSP00000083182	ENSP00000295633
ENSP00000083182	ENSP00000301327
ENSP00000083182	ENSP00000322396
ENSP00000083182	ENSP00000342136
ENSP00000083182	ENSP00000345702
ENSP00000083182	ENSP00000365943
ENSP00000083182	ENSP00000369695
ENSP00000083182	ENSP00000377520
ENSP00000083182	ENSP00000390783
ENSP00000085219	ENSP00000244007
ENSP00000085219	ENSP00000313419
ENSP00000085219	ENSP00000351926
ENSP00000085219	ENSP00000352603
ENSP00000085219	ENSP00000356346
ENSP00000085219	ENSP00000364898
ENSP00000085219	ENSP00000384675
ENSP00000086933	ENSP00000372023
ENSP00000156084	ENSP00000254770
ENSP00000156084	ENSP00000317904
ENSP00000156084	ENSP00000322016
ENSP00000156084	ENSP00000339933
ENSP00000156084	ENSP00000340684
ENSP00000156084	ENSP00000356652
ENSP00000156084	ENSP00000401121
ENSP00000156084	ENSP00000403359
ENSP00000156109	ENSP00000235090
ENSP00000156109	ENSP00000253329
ENSP00000156109	ENSP00000260210
ENSP00000156109	ENSP00000261674
ENSP00000156109	ENSP00000263642
ENSP00000156109	ENSP00000268482
ENSP00000156109	ENSP00000278886
ENSP00000156109	ENSP00000298532
ENSP00000156109	ENSP00000300086
ENSP00000156109	ENSP00000304350
ENSP00000156109	ENSP00000326261
ENSP00000156109	ENSP00000330188

ENSP00000156109	ENSP00000360532
ENSP00000156109	ENSP00000363313
ENSP00000156109	ENSP00000366829
ENSP00000156109	ENSP00000374507
ENSP00000156471	ENSP00000199814
ENSP00000156471	ENSP00000216297
ENSP00000156471	ENSP00000227524
ENSP00000156471	ENSP00000235090
ENSP00000156471	ENSP00000247815
ENSP00000156471	ENSP00000254719
ENSP00000156471	ENSP00000261531
ENSP00000156471	ENSP00000264705
ENSP00000156471	ENSP00000268043
ENSP00000156471	ENSP00000286835
ENSP00000156471	ENSP00000296702
ENSP00000156471	ENSP00000300648
ENSP00000156471	ENSP00000304370
ENSP00000156471	ENSP00000307525
ENSP00000156471	ENSP00000309166
ENSP00000156471	ENSP00000325863
ENSP00000156471	ENSP00000329466
ENSP00000156471	ENSP00000334148
ENSP00000156471	ENSP00000354518
ENSP00000156471	ENSP00000356146
ENSP00000156471	ENSP00000357631
ENSP00000156471	ENSP00000358554
ENSP00000156471	ENSP00000360532
ENSP00000156471	ENSP00000363021
ENSP00000156471	ENSP00000365747
ENSP00000156471	ENSP00000367835
ENSP00000156471	ENSP00000369335
ENSP00000156471	ENSP00000370150
ENSP00000156471	ENSP00000377262
ENSP00000159087	ENSP00000262507
ENSP00000159087	ENSP00000263817
ENSP00000159087	ENSP00000281141
ENSP00000159087	ENSP00000344460
ENSP00000159087	ENSP00000353073
ENSP00000159087	ENSP00000359531
ENSP00000159087	ENSP00000362688
ENSP00000159087	ENSP00000372689
ENSP00000159111	ENSP00000206249
ENSP00000159111	ENSP00000209875
ENSP00000159111	ENSP00000255764
ENSP00000159111	ENSP00000264515
ENSP00000159111	ENSP00000295066

ENSP00000159111	ENSP00000307208
ENSP00000159111	ENSP00000320485
ENSP00000159111	ENSP00000320566
ENSP00000159111	ENSP00000337127
ENSP00000159111	ENSP00000340896
ENSP00000159111	ENSP00000349049
ENSP00000159111	ENSP00000349213
ENSP00000159111	ENSP00000358810
ENSP00000159111	ENSP00000366974
ENSP00000159111	ENSP00000373340
ENSP00000159111	ENSP00000382688
ENSP00000159111	ENSP00000384313
ENSP00000159111	ENSP00000384675
ENSP00000160373	ENSP00000295951
ENSP00000160373	ENSP00000301843
ENSP00000160373	ENSP00000311344
ENSP00000160373	ENSP00000315702
ENSP00000160373	ENSP00000317334
ENSP00000160373	ENSP00000350071
ENSP00000160373	ENSP00000352676
ENSP00000160373	ENSP00000358810
ENSP00000161863	ENSP00000199814
ENSP00000161863	ENSP00000215793
ENSP00000161863	ENSP00000216252
ENSP00000161863	ENSP00000216277
ENSP00000161863	ENSP00000216727
ENSP00000161863	ENSP00000217109
ENSP00000161863	ENSP00000221494
ENSP00000161863	ENSP00000222969
ENSP00000161863	ENSP00000227524
ENSP00000161863	ENSP00000229214
ENSP00000161863	ENSP00000230340
ENSP00000161863	ENSP00000230859
ENSP00000161863	ENSP00000236273
ENSP00000161863	ENSP00000243563
ENSP00000161863	ENSP00000245544
ENSP00000161863	ENSP00000254193
ENSP00000161863	ENSP00000257829
ENSP00000161863	ENSP00000260210
ENSP00000161863	ENSP00000261531
ENSP00000161863	ENSP00000261708
ENSP00000161863	ENSP00000261973
ENSP00000161863	ENSP00000262320
ENSP00000161863	ENSP00000264951
ENSP00000161863	ENSP00000266079
ENSP00000161863	ENSP00000266735

ENSP00000161863	ENSP00000269385
ENSP00000161863	ENSP00000271628
ENSP00000161863	ENSP00000296215
ENSP00000161863	ENSP00000297990
ENSP00000161863	ENSP00000298281
ENSP00000161863	ENSP00000298532
ENSP00000161863	ENSP00000298937
ENSP00000161863	ENSP00000300291
ENSP00000161863	ENSP00000301962
ENSP00000161863	ENSP00000303191
ENSP00000161863	ENSP00000304350
ENSP00000161863	ENSP00000304370
ENSP00000161863	ENSP00000304704
ENSP00000161863	ENSP00000305790
ENSP00000161863	ENSP00000315035
ENSP00000161863	ENSP00000315379
ENSP00000161863	ENSP00000315674
ENSP00000161863	ENSP00000315791
ENSP00000161863	ENSP00000323302
ENSP00000161863	ENSP00000324122
ENSP00000161863	ENSP00000326477
ENSP00000161863	ENSP00000326806
ENSP00000161863	ENSP00000328854
ENSP00000161863	ENSP00000329466
ENSP00000161863	ENSP00000334148
ENSP00000161863	ENSP00000335321
ENSP00000161863	ENSP00000336752
ENSP00000161863	ENSP00000339090
ENSP00000161863	ENSP00000339353
ENSP00000161863	ENSP00000345917
ENSP00000161863	ENSP00000348010
ENSP00000161863	ENSP00000350967
ENSP00000161863	ENSP00000351492
ENSP00000161863	ENSP00000352738
ENSP00000161863	ENSP00000355541
ENSP00000161863	ENSP00000357965
ENSP00000161863	ENSP00000358554
ENSP00000161863	ENSP00000358812
ENSP00000161863	ENSP00000358997
ENSP00000161863	ENSP00000360031
ENSP00000161863	ENSP00000360497
ENSP00000161863	ENSP00000360532
ENSP00000161863	ENSP00000361557
ENSP00000161863	ENSP00000361577
ENSP00000161863	ENSP00000362110
ENSP00000161863	ENSP00000363313

ENSP00000161863	ENSP00000364289
ENSP00000161863	ENSP00000366135
ENSP00000161863	ENSP00000366557
ENSP00000161863	ENSP00000367835
ENSP00000161863	ENSP00000368017
ENSP00000161863	ENSP00000370460
ENSP00000161863	ENSP00000376973
ENSP00000161863	ENSP00000383059
ENSP00000161863	ENSP00000387006
ENSP00000161863	ENSP00000391432
ENSP00000161863	ENSP00000400591
ENSP00000161863	ENSP00000404151
ENSP00000162330	ENSP00000171887
ENSP00000162330	ENSP00000206249
ENSP00000162330	ENSP00000211998
ENSP00000162330	ENSP00000228307
ENSP00000162330	ENSP00000233154
ENSP00000162330	ENSP00000262304
ENSP00000162330	ENSP00000276893
ENSP00000162330	ENSP00000298229
ENSP00000162330	ENSP00000300574
ENSP00000162330	ENSP00000309230
ENSP00000162330	ENSP00000310561
ENSP00000162330	ENSP00000312506
ENSP00000162330	ENSP00000313169
ENSP00000162330	ENSP00000316460
ENSP00000162330	ENSP00000317817
ENSP00000162330	ENSP00000324422
ENSP00000162330	ENSP00000340944
ENSP00000162330	ENSP00000341189
ENSP00000162330	ENSP00000361264
ENSP00000162330	ENSP00000371341
ENSP00000162330	ENSP00000380921
ENSP00000162749	ENSP00000185150
ENSP00000162749	ENSP00000216274
ENSP00000162749	ENSP00000219789
ENSP00000162749	ENSP00000220751
ENSP00000162749	ENSP00000221566
ENSP00000162749	ENSP00000225972
ENSP00000162749	ENSP00000227758
ENSP00000162749	ENSP00000231572
ENSP00000162749	ENSP00000232003
ENSP00000162749	ENSP00000234111
ENSP00000162749	ENSP00000237289
ENSP00000162749	ENSP00000238341
ENSP00000162749	ENSP00000252015

ENSP00000162749	ENSP00000259808
ENSP00000162749	ENSP00000260187
ENSP00000162749	ENSP00000265717
ENSP00000162749	ENSP00000268605
ENSP00000162749	ENSP00000269143
ENSP00000162749	ENSP00000286186
ENSP00000162749	ENSP00000287322
ENSP00000162749	ENSP00000301838
ENSP00000162749	ENSP00000310129
ENSP00000162749	ENSP00000310933
ENSP00000162749	ENSP00000312664
ENSP00000162749	ENSP00000313420
ENSP00000162749	ENSP00000318822
ENSP00000162749	ENSP00000319713
ENSP00000162749	ENSP00000325448
ENSP00000162749	ENSP00000326699
ENSP00000162749	ENSP00000327647
ENSP00000162749	ENSP00000337053
ENSP00000162749	ENSP00000338983
ENSP00000162749	ENSP00000339191
ENSP00000162749	ENSP00000340409
ENSP00000162749	ENSP00000340944
ENSP00000162749	ENSP00000341189
ENSP00000162749	ENSP00000341268
ENSP00000162749	ENSP00000343412
ENSP00000162749	ENSP00000347979
ENSP00000162749	ENSP00000350785
ENSP00000162749	ENSP00000356694
ENSP00000162749	ENSP00000358335
ENSP00000162749	ENSP00000358997
ENSP00000162749	ENSP00000359727
ENSP00000162749	ENSP00000364898
ENSP00000162749	ENSP00000380921
ENSP00000162749	ENSP00000381891
ENSP00000162749	ENSP00000395102
ENSP00000162749	ENSP00000401445
ENSP00000162749	ENSP00000410481
ENSP00000163416	ENSP00000292535
ENSP00000163416	ENSP00000370719
ENSP00000163416	ENSP00000382327
ENSP00000164024	ENSP00000283943
ENSP00000164024	ENSP00000296084
ENSP00000164024	ENSP00000310800
ENSP00000164024	ENSP00000313309
ENSP00000164024	ENSP00000314556
ENSP00000164024	ENSP00000319831

ENSP00000164024	ENSP00000326924
ENSP00000164024	ENSP00000334003
ENSP00000164024	ENSP00000351605
ENSP00000164024	ENSP00000354895
ENSP00000164024	ENSP00000388872
ENSP00000164227	ENSP00000222330
ENSP00000164227	ENSP00000226574
ENSP00000164227	ENSP00000260947
ENSP00000164227	ENSP00000261435
ENSP00000164227	ENSP00000262193
ENSP00000164227	ENSP00000262367
ENSP00000164227	ENSP00000295025
ENSP00000164227	ENSP00000306245
ENSP00000164227	ENSP00000311825
ENSP00000164227	ENSP00000334061
ENSP00000164227	ENSP00000340879
ENSP00000164227	ENSP00000344456
ENSP00000164227	ENSP00000349049
ENSP00000164227	ENSP00000349748
ENSP00000164227	ENSP00000350512
ENSP00000164227	ENSP00000351894
ENSP00000164227	ENSP00000359531
ENSP00000164227	ENSP00000359727
ENSP00000164227	ENSP00000364893
ENSP00000164227	ENSP00000369785
ENSP00000164305	ENSP00000251038
ENSP00000164305	ENSP00000304370
ENSP00000164305	ENSP00000330945
ENSP00000164305	ENSP00000334148
ENSP00000166139	ENSP00000242208
ENSP00000166139	ENSP00000341551
ENSP00000166139	ENSP00000346839
ENSP00000166345	ENSP00000209728
ENSP00000166345	ENSP00000212015
ENSP00000166345	ENSP00000216297
ENSP00000166345	ENSP00000219473
ENSP00000166345	ENSP00000219479
ENSP00000166345	ENSP00000220669
ENSP00000166345	ENSP00000229595
ENSP00000166345	ENSP00000234170
ENSP00000166345	ENSP00000235310
ENSP00000166345	ENSP00000238379
ENSP00000166345	ENSP00000251864
ENSP00000166345	ENSP00000251968
ENSP00000166345	ENSP00000254719
ENSP00000166345	ENSP00000254940

ENSP00000166345	ENSP00000257934
ENSP00000166345	ENSP00000260643
ENSP00000166345	ENSP00000261173
ENSP00000166345	ENSP00000261531
ENSP00000166345	ENSP00000263187
ENSP00000166345	ENSP00000263795
ENSP00000166345	ENSP00000263925
ENSP00000166345	ENSP00000264705
ENSP00000166345	ENSP00000267430
ENSP00000166345	ENSP00000276326
ENSP00000166345	ENSP00000278412
ENSP00000166345	ENSP00000296509
ENSP00000166345	ENSP00000300896
ENSP00000166345	ENSP00000307142
ENSP00000166345	ENSP00000308466
ENSP00000166345	ENSP00000308546
ENSP00000166345	ENSP00000308944
ENSP00000166345	ENSP00000310129
ENSP00000166345	ENSP00000310520
ENSP00000166345	ENSP00000317468
ENSP00000166345	ENSP00000322885
ENSP00000166345	ENSP00000325448
ENSP00000166345	ENSP00000331209
ENSP00000166345	ENSP00000336606
ENSP00000166345	ENSP00000338606
ENSP00000166345	ENSP00000338862
ENSP00000166345	ENSP00000339047
ENSP00000166345	ENSP00000340879
ENSP00000166345	ENSP00000341848
ENSP00000166345	ENSP00000343635
ENSP00000166345	ENSP00000343741
ENSP00000166345	ENSP00000347038
ENSP00000166345	ENSP00000348573
ENSP00000166345	ENSP00000352572
ENSP00000166345	ENSP00000353073
ENSP00000166345	ENSP00000354486
ENSP00000166345	ENSP00000355865
ENSP00000166345	ENSP00000355922
ENSP00000166345	ENSP00000357861
ENSP00000166345	ENSP00000358478
ENSP00000166345	ENSP00000359403
ENSP00000166345	ENSP00000360310
ENSP00000166345	ENSP00000360412
ENSP00000166345	ENSP00000360532
ENSP00000166345	ENSP00000360621
ENSP00000166345	ENSP00000360992

ENSP00000166345	ENSP00000362936
ENSP00000166345	ENSP00000363433
ENSP00000166345	ENSP00000368119
ENSP00000166345	ENSP00000368801
ENSP00000166345	ENSP00000370410
ENSP00000166345	ENSP00000370588
ENSP00000166345	ENSP00000386394
ENSP00000166345	ENSP00000389175
ENSP00000166345	ENSP00000394649
ENSP00000166345	ENSP00000396704
ENSP00000166345	ENSP00000406868
ENSP00000166345	ENSP00000407047
ENSP00000166345	ENSP00000410818
ENSP00000167106	ENSP00000361599
ENSP00000168977	ENSP00000215071
ENSP00000168977	ENSP00000222553
ENSP00000168977	ENSP00000244741
ENSP00000168977	ENSP00000258418
ENSP00000168977	ENSP00000265437
ENSP00000168977	ENSP00000276654
ENSP00000168977	ENSP00000346155
ENSP00000168977	ENSP00000348577
ENSP00000168977	ENSP00000351113
ENSP00000168977	ENSP00000354532
ENSP00000168977	ENSP00000369519
ENSP00000168977	ENSP00000369862
ENSP00000168977	ENSP00000401508
ENSP00000169551	ENSP00000216034
ENSP00000169551	ENSP00000250615
ENSP00000169551	ENSP00000252487
ENSP00000169551	ENSP00000258424
ENSP00000169551	ENSP00000259667
ENSP00000169551	ENSP00000270538
ENSP00000169551	ENSP00000276416
ENSP00000169551	ENSP00000295767
ENSP00000169551	ENSP00000296805
ENSP00000169551	ENSP00000300151
ENSP00000169551	ENSP00000301585
ENSP00000169551	ENSP00000310723
ENSP00000169551	ENSP00000315693
ENSP00000169551	ENSP00000318115
ENSP00000169551	ENSP00000320236
ENSP00000169551	ENSP00000320758
ENSP00000169551	ENSP00000338293
ENSP00000169551	ENSP00000344220
ENSP00000169551	ENSP00000347919

ENSP00000169551	ENSP00000349205
ENSP00000169551	ENSP00000353698
ENSP00000169551	ENSP00000354499
ENSP00000169551	ENSP00000354876
ENSP00000169551	ENSP00000355572
ENSP00000169551	ENSP00000365766
ENSP00000169551	ENSP00000366863
ENSP00000169551	ENSP00000367934
ENSP00000169551	ENSP00000368066
ENSP00000169551	ENSP00000368523
ENSP00000169551	ENSP00000387262
ENSP00000170168	ENSP00000203001
ENSP00000170168	ENSP00000253107
ENSP00000170168	ENSP00000265881
ENSP00000170168	ENSP00000327821
ENSP00000170168	ENSP00000339529
ENSP00000170168	ENSP00000348107
ENSP00000170168	ENSP00000366135
ENSP00000170168	ENSP00000368438
ENSP00000170168	ENSP00000376973
ENSP00000170168	ENSP00000395574
ENSP00000170564	ENSP00000227524
ENSP00000170564	ENSP00000247219
ENSP00000170564	ENSP00000304370
ENSP00000170564	ENSP00000304704
ENSP00000170564	ENSP00000334148
ENSP00000170564	ENSP00000339723
ENSP00000170564	ENSP00000358854
ENSP00000170630	ENSP00000231449
ENSP00000170630	ENSP00000304915
ENSP00000170630	ENSP00000305133
ENSP00000170630	ENSP00000331201
ENSP00000170630	ENSP00000340944
ENSP00000170630	ENSP00000360730
ENSP00000170630	ENSP00000365016
ENSP00000171887	ENSP00000216194
ENSP00000171887	ENSP00000219476
ENSP00000171887	ENSP00000228307
ENSP00000171887	ENSP00000252699
ENSP00000171887	ENSP00000262039
ENSP00000171887	ENSP00000298552
ENSP00000171887	ENSP00000306245
ENSP00000171887	ENSP00000313059
ENSP00000171887	ENSP00000313169
ENSP00000171887	ENSP00000313420
ENSP00000171887	ENSP00000327145

ENSP00000171887	ENSP00000341189
ENSP00000171887	ENSP00000348020
ENSP00000171887	ENSP00000352121
ENSP00000171887	ENSP00000354558
ENSP00000171887	ENSP00000355296
ENSP00000171887	ENSP00000363680
ENSP00000171887	ENSP00000365016
ENSP00000171887	ENSP00000379607
ENSP00000171887	ENSP00000402515
ENSP00000172229	ENSP00000220751
ENSP00000172229	ENSP00000226730
ENSP00000172229	ENSP00000256637
ENSP00000172229	ENSP00000256707
ENSP00000172229	ENSP00000262294
ENSP00000172229	ENSP00000264246
ENSP00000172229	ENSP00000281043
ENSP00000172229	ENSP00000284981
ENSP00000172229	ENSP00000298552
ENSP00000172229	ENSP00000322457
ENSP00000172229	ENSP00000326366
ENSP00000172229	ENSP00000329357
ENSP00000172229	ENSP00000333537
ENSP00000172229	ENSP00000339191
ENSP00000172229	ENSP00000341268
ENSP00000172229	ENSP00000347451
ENSP00000172229	ENSP00000354207
ENSP00000172229	ENSP00000364847
ENSP00000172229	ENSP00000394794
ENSP00000172229	ENSP00000416959
ENSP00000173527	ENSP00000251038
ENSP00000173527	ENSP00000294973
ENSP00000173527	ENSP00000355518
ENSP00000173527	ENSP00000410758
ENSP00000173898	ENSP00000230340
ENSP00000173898	ENSP00000257909
ENSP00000173898	ENSP00000306245
ENSP00000173898	ENSP00000343535
ENSP00000174618	ENSP00000219476
ENSP00000174618	ENSP00000246912
ENSP00000174618	ENSP00000351490
ENSP00000174618	ENSP00000353622
ENSP00000174618	ENSP00000382562
ENSP00000174618	ENSP00000414187
ENSP00000175506	ENSP00000287878
ENSP00000175506	ENSP00000329466
ENSP00000175506	ENSP00000361699

ENSP00000176183	ENSP00000219548
ENSP00000176183	ENSP00000233154
ENSP00000176183	ENSP00000290332
ENSP00000176183	ENSP00000295101
ENSP00000176183	ENSP00000360916
ENSP00000176195	ENSP00000230588
ENSP00000176195	ENSP00000326366
ENSP00000176195	ENSP00000358120
ENSP00000176195	ENSP00000365678
ENSP00000178640	ENSP00000181839
ENSP00000178640	ENSP00000216160
ENSP00000178640	ENSP00000219473
ENSP00000178640	ENSP00000224073
ENSP00000178640	ENSP00000228307
ENSP00000178640	ENSP00000233078
ENSP00000178640	ENSP00000234590
ENSP00000178640	ENSP00000245960
ENSP00000178640	ENSP00000254654
ENSP00000178640	ENSP00000261211
ENSP00000178640	ENSP00000263043
ENSP00000178640	ENSP00000264380
ENSP00000178640	ENSP00000265562
ENSP00000178640	ENSP00000282388
ENSP00000178640	ENSP00000282541
ENSP00000178640	ENSP00000283228
ENSP00000178640	ENSP00000306682
ENSP00000178640	ENSP00000309103
ENSP00000178640	ENSP00000311005
ENSP00000178640	ENSP00000311343
ENSP00000178640	ENSP00000312411
ENSP00000178640	ENSP00000320754
ENSP00000178640	ENSP00000334188
ENSP00000178640	ENSP00000340944
ENSP00000178640	ENSP00000352257
ENSP00000178640	ENSP00000354927
ENSP00000178640	ENSP00000355296
ENSP00000178640	ENSP00000356379
ENSP00000178640	ENSP00000356595
ENSP00000178640	ENSP00000357494
ENSP00000178640	ENSP00000357907
ENSP00000178640	ENSP00000358293
ENSP00000178640	ENSP00000362656
ENSP00000178640	ENSP00000373702
ENSP00000178640	ENSP00000374455
ENSP00000178640	ENSP00000377047
ENSP00000178640	ENSP00000378306

ENSP00000179259	ENSP00000216392
ENSP00000179259	ENSP00000232607
ENSP00000179259	ENSP00000246166
ENSP00000179259	ENSP00000253237
ENSP00000179259	ENSP00000261531
ENSP00000179259	ENSP00000263956
ENSP00000179259	ENSP00000304350
ENSP00000179259	ENSP00000321259
ENSP00000179259	ENSP00000359531
ENSP00000179259	ENSP00000361180
ENSP00000179259	ENSP00000362413
ENSP00000179259	ENSP00000363676
ENSP00000181839	ENSP00000209728
ENSP00000181839	ENSP00000216297
ENSP00000181839	ENSP00000221265
ENSP00000181839	ENSP00000224140
ENSP00000181839	ENSP00000225504
ENSP00000181839	ENSP00000225728
ENSP00000181839	ENSP00000229595
ENSP00000181839	ENSP00000236051
ENSP00000181839	ENSP00000246802
ENSP00000181839	ENSP00000254719
ENSP00000181839	ENSP00000254803
ENSP00000181839	ENSP00000254942
ENSP00000181839	ENSP00000256398
ENSP00000181839	ENSP00000256897
ENSP00000181839	ENSP00000261211
ENSP00000181839	ENSP00000261601
ENSP00000181839	ENSP00000261845
ENSP00000181839	ENSP00000262173
ENSP00000181839	ENSP00000262518
ENSP00000181839	ENSP00000264157
ENSP00000181839	ENSP00000264515
ENSP00000181839	ENSP00000264951
ENSP00000181839	ENSP00000265073
ENSP00000181839	ENSP00000265857
ENSP00000181839	ENSP00000268043
ENSP00000181839	ENSP00000271764
ENSP00000181839	ENSP00000273064
ENSP00000181839	ENSP00000276603
ENSP00000181839	ENSP00000282077
ENSP00000181839	ENSP00000286835
ENSP00000181839	ENSP00000291386
ENSP00000181839	ENSP00000293406
ENSP00000181839	ENSP00000295066
ENSP00000181839	ENSP00000295240

ENSP00000181839	ENSP00000296786
ENSP00000181839	ENSP00000297185
ENSP00000181839	ENSP00000298283
ENSP00000181839	ENSP00000299543
ENSP00000181839	ENSP00000299601
ENSP00000181839	ENSP00000300584
ENSP00000181839	ENSP00000301364
ENSP00000181839	ENSP00000302478
ENSP00000181839	ENSP00000302896
ENSP00000181839	ENSP00000304593
ENSP00000181839	ENSP00000311005
ENSP00000181839	ENSP00000312735
ENSP00000181839	ENSP00000314348
ENSP00000181839	ENSP00000315674
ENSP00000181839	ENSP00000315693
ENSP00000181839	ENSP00000315859
ENSP00000181839	ENSP00000319104
ENSP00000181839	ENSP00000320566
ENSP00000181839	ENSP00000320917
ENSP00000181839	ENSP00000321449
ENSP00000181839	ENSP00000322396
ENSP00000181839	ENSP00000325376
ENSP00000181839	ENSP00000325677
ENSP00000181839	ENSP00000331310
ENSP00000181839	ENSP00000331817
ENSP00000181839	ENSP00000336842
ENSP00000181839	ENSP00000338788
ENSP00000181839	ENSP00000338862
ENSP00000181839	ENSP00000340896
ENSP00000181839	ENSP00000341885
ENSP00000181839	ENSP00000343741
ENSP00000181839	ENSP00000351284
ENSP00000181839	ENSP00000353622
ENSP00000181839	ENSP00000354673
ENSP00000181839	ENSP00000354812
ENSP00000181839	ENSP00000355013
ENSP00000181839	ENSP00000355205
ENSP00000181839	ENSP00000356146
ENSP00000181839	ENSP00000356405
ENSP00000181839	ENSP00000356989
ENSP00000181839	ENSP00000357887
ENSP00000181839	ENSP00000357907
ENSP00000181839	ENSP00000358812
ENSP00000181839	ENSP00000359518
ENSP00000181839	ENSP00000360116
ENSP00000181839	ENSP00000362900

ENSP00000181839	ENSP00000363779
ENSP00000181839	ENSP00000364289
ENSP00000181839	ENSP00000364912
ENSP00000181839	ENSP00000366135
ENSP00000181839	ENSP00000367029
ENSP00000181839	ENSP00000368349
ENSP00000181839	ENSP00000373700
ENSP00000181839	ENSP00000374213
ENSP00000181839	ENSP00000374280
ENSP00000181839	ENSP00000374529
ENSP00000181839	ENSP00000379217
ENSP00000181839	ENSP00000380504
ENSP00000181839	ENSP00000381657
ENSP00000181839	ENSP00000386759
ENSP00000181839	ENSP00000397259
ENSP00000181839	ENSP00000402338
ENSP00000185150	ENSP00000238789
ENSP00000185150	ENSP00000248633
ENSP00000185150	ENSP00000253719
ENSP00000185150	ENSP00000258654
ENSP00000185150	ENSP00000259512
ENSP00000185150	ENSP00000260643
ENSP00000185150	ENSP00000261534
ENSP00000185150	ENSP00000261942
ENSP00000185150	ENSP00000265070
ENSP00000185150	ENSP00000298852
ENSP00000185150	ENSP00000299767
ENSP00000185150	ENSP00000303511
ENSP00000185150	ENSP00000318147
ENSP00000185150	ENSP00000320885
ENSP00000185150	ENSP00000332979
ENSP00000185150	ENSP00000333836
ENSP00000185150	ENSP00000337053
ENSP00000185150	ENSP00000347140
ENSP00000185150	ENSP00000366395
ENSP00000185150	ENSP00000379457
ENSP00000185150	ENSP00000381891
ENSP00000185150	ENSP00000387347
ENSP00000193322	ENSP00000297313
ENSP00000193322	ENSP00000333298
ENSP00000194118	ENSP00000222115
ENSP00000194118	ENSP00000236067
ENSP00000194118	ENSP00000243253
ENSP00000194118	ENSP00000331487
ENSP00000194118	ENSP00000343885
ENSP00000194214	ENSP00000238789

ENSP00000194214	ENSP00000256474
ENSP00000194214	ENSP00000257934
ENSP00000194214	ENSP00000261211
ENSP00000194214	ENSP00000261819
ENSP00000194214	ENSP00000261881
ENSP00000194214	ENSP00000265148
ENSP00000194214	ENSP00000287598
ENSP00000194214	ENSP00000291539
ENSP00000194214	ENSP00000296577
ENSP00000194214	ENSP00000300291
ENSP00000194214	ENSP00000302530
ENSP00000194214	ENSP00000314004
ENSP00000194214	ENSP00000344741
ENSP00000194214	ENSP00000346566
ENSP00000194214	ENSP00000355013
ENSP00000194214	ENSP00000386156
ENSP00000194214	ENSP00000403852
ENSP00000195654	ENSP00000228027
ENSP00000195654	ENSP00000262854
ENSP00000195654	ENSP00000262903
ENSP00000195654	ENSP00000283943
ENSP00000195654	ENSP00000304500
ENSP00000195654	ENSP00000308496
ENSP00000195654	ENSP00000348714
ENSP00000195654	ENSP00000361245
ENSP00000195654	ENSP00000382269
ENSP00000195654	ENSP00000414786
ENSP00000196169	ENSP00000222005
ENSP00000196169	ENSP00000229201
ENSP00000196169	ENSP00000240159
ENSP00000196169	ENSP00000250113
ENSP00000196169	ENSP00000251303
ENSP00000196169	ENSP00000261881
ENSP00000196169	ENSP00000262794
ENSP00000196169	ENSP00000268043
ENSP00000196169	ENSP00000307853
ENSP00000196169	ENSP00000313829
ENSP00000196169	ENSP00000314311
ENSP00000196169	ENSP00000321636
ENSP00000196169	ENSP00000322242
ENSP00000196169	ENSP00000325690
ENSP00000196169	ENSP00000326128
ENSP00000196169	ENSP00000328940
ENSP00000196169	ENSP00000331879
ENSP00000196169	ENSP00000343103
ENSP00000196169	ENSP00000351284

ENSP00000196169	ENSP00000354522
ENSP00000196169	ENSP00000366843
ENSP00000196169	ENSP00000369335
ENSP00000196169	ENSP00000369757
ENSP00000196169	ENSP00000370151
ENSP00000196169	ENSP00000372023
ENSP00000196169	ENSP00000381031
ENSP00000196169	ENSP00000387303
ENSP00000196371	ENSP00000311505
ENSP00000196371	ENSP00000335060
ENSP00000196551	ENSP00000199320
ENSP00000196551	ENSP00000202773
ENSP00000196551	ENSP00000211372
ENSP00000196551	ENSP00000219252
ENSP00000196551	ENSP00000221859
ENSP00000196551	ENSP00000222247
ENSP00000196551	ENSP00000224140
ENSP00000196551	ENSP00000225430
ENSP00000196551	ENSP00000227524
ENSP00000196551	ENSP00000229214
ENSP00000196551	ENSP00000230050
ENSP00000196551	ENSP00000230859
ENSP00000196551	ENSP00000232888
ENSP00000196551	ENSP00000232905
ENSP00000196551	ENSP00000241600
ENSP00000196551	ENSP00000244230
ENSP00000196551	ENSP00000251038
ENSP00000196551	ENSP00000252543
ENSP00000196551	ENSP00000253099
ENSP00000196551	ENSP00000253788
ENSP00000196551	ENSP00000257829
ENSP00000196551	ENSP00000258455
ENSP00000196551	ENSP00000259469
ENSP00000196551	ENSP00000259569
ENSP00000196551	ENSP00000260102
ENSP00000196551	ENSP00000260443
ENSP00000196551	ENSP00000262544
ENSP00000196551	ENSP00000262584
ENSP00000196551	ENSP00000262803
ENSP00000196551	ENSP00000263629
ENSP00000196551	ENSP00000263657
ENSP00000196551	ENSP00000264515
ENSP00000196551	ENSP00000264995
ENSP00000196551	ENSP00000265100
ENSP00000196551	ENSP00000268206
ENSP00000196551	ENSP00000268661

ENSP00000196551	ENSP00000268802
ENSP00000196551	ENSP00000270625
ENSP00000196551	ENSP00000272418
ENSP00000196551	ENSP00000274606
ENSP00000196551	ENSP00000277541
ENSP00000196551	ENSP00000278572
ENSP00000196551	ENSP00000280557
ENSP00000196551	ENSP00000283109
ENSP00000196551	ENSP00000287038
ENSP00000196551	ENSP00000288937
ENSP00000196551	ENSP00000289371
ENSP00000196551	ENSP00000295491
ENSP00000196551	ENSP00000295930
ENSP00000196551	ENSP00000296674
ENSP00000196551	ENSP00000296792
ENSP00000196551	ENSP00000296802
ENSP00000196551	ENSP00000296805
ENSP00000196551	ENSP00000297908
ENSP00000196551	ENSP00000297990
ENSP00000196551	ENSP00000298283
ENSP00000196551	ENSP00000300151
ENSP00000196551	ENSP00000301729
ENSP00000196551	ENSP00000301740
ENSP00000196551	ENSP00000302896
ENSP00000196551	ENSP00000306548
ENSP00000196551	ENSP00000307567
ENSP00000196551	ENSP00000307889
ENSP00000196551	ENSP00000308897
ENSP00000196551	ENSP00000309334
ENSP00000196551	ENSP00000309433
ENSP00000196551	ENSP00000311028
ENSP00000196551	ENSP00000311430
ENSP00000196551	ENSP00000312042
ENSP00000196551	ENSP00000312735
ENSP00000196551	ENSP00000317376
ENSP00000196551	ENSP00000318195
ENSP00000196551	ENSP00000318646
ENSP00000196551	ENSP00000322419
ENSP00000196551	ENSP00000323856
ENSP00000196551	ENSP00000324122
ENSP00000196551	ENSP00000326261
ENSP00000196551	ENSP00000326699
ENSP00000196551	ENSP00000326981
ENSP00000196551	ENSP00000329466
ENSP00000196551	ENSP00000329967
ENSP00000196551	ENSP00000331019

ENSP00000196551	ENSP00000336702
ENSP00000196551	ENSP00000337127
ENSP00000196551	ENSP00000339095
ENSP00000196551	ENSP00000339795
ENSP00000196551	ENSP00000340896
ENSP00000196551	ENSP00000341243
ENSP00000196551	ENSP00000341692
ENSP00000196551	ENSP00000341885
ENSP00000196551	ENSP00000342889
ENSP00000196551	ENSP00000345156
ENSP00000196551	ENSP00000346015
ENSP00000196551	ENSP00000346022
ENSP00000196551	ENSP00000346027
ENSP00000196551	ENSP00000346037
ENSP00000196551	ENSP00000346050
ENSP00000196551	ENSP00000346067
ENSP00000196551	ENSP00000346453
ENSP00000196551	ENSP00000347087
ENSP00000196551	ENSP00000349959
ENSP00000196551	ENSP00000351596
ENSP00000196551	ENSP00000352706
ENSP00000196551	ENSP00000354525
ENSP00000196551	ENSP00000354739
ENSP00000196551	ENSP00000355471
ENSP00000196551	ENSP00000355890
ENSP00000196551	ENSP00000356134
ENSP00000196551	ENSP00000357480
ENSP00000196551	ENSP00000358854
ENSP00000196551	ENSP00000359345
ENSP00000196551	ENSP00000361076
ENSP00000196551	ENSP00000361206
ENSP00000196551	ENSP00000361465
ENSP00000196551	ENSP00000362036
ENSP00000196551	ENSP00000362208
ENSP00000196551	ENSP00000362306
ENSP00000196551	ENSP00000362688
ENSP00000196551	ENSP00000362744
ENSP00000196551	ENSP00000362993
ENSP00000196551	ENSP00000363018
ENSP00000196551	ENSP00000363250
ENSP00000196551	ENSP00000363308
ENSP00000196551	ENSP00000363676
ENSP00000196551	ENSP00000363944
ENSP00000196551	ENSP00000364320
ENSP00000196551	ENSP00000367923
ENSP00000196551	ENSP00000368790

ENSP00000196551	ENSP00000368927
ENSP00000196551	ENSP00000369757
ENSP00000196551	ENSP00000370589
ENSP00000196551	ENSP00000372445
ENSP00000196551	ENSP00000373404
ENSP00000196551	ENSP00000374566
ENSP00000196551	ENSP00000375730
ENSP00000196551	ENSP00000377640
ENSP00000196551	ENSP00000379339
ENSP00000196551	ENSP00000379888
ENSP00000196551	ENSP00000380156
ENSP00000196551	ENSP00000386717
ENSP00000196551	ENSP00000389103
ENSP00000196551	ENSP00000392270
ENSP00000196551	ENSP00000393241
ENSP00000196551	ENSP00000402338
ENSP00000196551	ENSP00000403175
ENSP00000196551	ENSP00000412583
ENSP00000196551	ENSP00000414321
ENSP00000196551	ENSP00000416110
ENSP00000196551	ENSP00000418661
ENSP00000196551	ENSP00000420714
ENSP00000198536	ENSP00000230431
ENSP00000198536	ENSP00000340944
ENSP00000198536	ENSP00000355136
ENSP00000198536	ENSP00000359403
ENSP00000198767	ENSP00000222567
ENSP00000198767	ENSP00000246802
ENSP00000198767	ENSP00000262643
ENSP00000198767	ENSP00000263663
ENSP00000198767	ENSP00000263857
ENSP00000198767	ENSP00000263974
ENSP00000198767	ENSP00000298746
ENSP00000198767	ENSP00000302640
ENSP00000198767	ENSP00000312735
ENSP00000198767	ENSP00000314971
ENSP00000198767	ENSP00000322570
ENSP00000198767	ENSP00000322706
ENSP00000198767	ENSP00000327072
ENSP00000198767	ENSP00000351697
ENSP00000198767	ENSP00000358563
ENSP00000198767	ENSP00000361982
ENSP00000198767	ENSP00000367029
ENSP00000198767	ENSP00000368349
ENSP00000198767	ENSP00000416892
ENSP00000199320	ENSP00000211372

ENSP00000199320	ENSP00000229214
ENSP00000199320	ENSP00000230340
ENSP00000199320	ENSP00000232888
ENSP00000199320	ENSP00000241600
ENSP00000199320	ENSP00000244230
ENSP00000199320	ENSP00000245539
ENSP00000199320	ENSP00000247219
ENSP00000199320	ENSP00000253083
ENSP00000199320	ENSP00000254037
ENSP00000199320	ENSP00000254928
ENSP00000199320	ENSP00000256031
ENSP00000199320	ENSP00000258455
ENSP00000199320	ENSP00000259750
ENSP00000199320	ENSP00000261637
ENSP00000199320	ENSP00000261708
ENSP00000199320	ENSP00000263657
ENSP00000199320	ENSP00000265537
ENSP00000199320	ENSP00000267425
ENSP00000199320	ENSP00000268802
ENSP00000199320	ENSP00000269385
ENSP00000199320	ENSP00000270625
ENSP00000199320	ENSP00000272418
ENSP00000199320	ENSP00000272521
ENSP00000199320	ENSP00000278572
ENSP00000199320	ENSP00000283109
ENSP00000199320	ENSP00000291576
ENSP00000199320	ENSP00000296674
ENSP00000199320	ENSP00000296792
ENSP00000199320	ENSP00000297990
ENSP00000199320	ENSP00000301364
ENSP00000199320	ENSP00000301962
ENSP00000199320	ENSP00000302896
ENSP00000199320	ENSP00000308179
ENSP00000199320	ENSP00000311028
ENSP00000199320	ENSP00000314193
ENSP00000199320	ENSP00000317376
ENSP00000199320	ENSP00000318646
ENSP00000199320	ENSP00000326981
ENSP00000199320	ENSP00000327179
ENSP00000199320	ENSP00000328854
ENSP00000199320	ENSP00000336712
ENSP00000199320	ENSP00000341885
ENSP00000199320	ENSP00000342381
ENSP00000199320	ENSP00000346067
ENSP00000199320	ENSP00000355541
ENSP00000199320	ENSP00000356548

ENSP00000199320	ENSP00000360031
ENSP00000199320	ENSP00000362036
ENSP00000199320	ENSP00000363746
ENSP00000199320	ENSP00000364320
ENSP00000199320	ENSP00000364847
ENSP00000199320	ENSP00000370968
ENSP00000199320	ENSP00000372695
ENSP00000199320	ENSP00000377954
ENSP00000199320	ENSP00000379339
ENSP00000199320	ENSP00000379607
ENSP00000199320	ENSP00000384302
ENSP00000199320	ENSP00000393241
ENSP00000199320	ENSP00000399454
ENSP00000199320	ENSP00000402869
ENSP00000199320	ENSP00000403175
ENSP00000199320	ENSP00000405614
ENSP00000199320	ENSP00000412583
ENSP00000199320	ENSP00000416110
ENSP00000199320	ENSP00000420714
ENSP00000199389	ENSP00000203001
ENSP00000199389	ENSP00000216554
ENSP00000199389	ENSP00000222005
ENSP00000199389	ENSP00000229595
ENSP00000199389	ENSP00000233057
ENSP00000199389	ENSP00000251038
ENSP00000199389	ENSP00000256383
ENSP00000199389	ENSP00000259750
ENSP00000199389	ENSP00000261845
ENSP00000199389	ENSP00000266079
ENSP00000199389	ENSP00000266126
ENSP00000199389	ENSP00000273783
ENSP00000199389	ENSP00000284202
ENSP00000199389	ENSP00000289371
ENSP00000199389	ENSP00000298532
ENSP00000199389	ENSP00000298746
ENSP00000199389	ENSP00000300648
ENSP00000199389	ENSP00000302801
ENSP00000199389	ENSP00000303315
ENSP00000199389	ENSP00000304350
ENSP00000199389	ENSP00000310448
ENSP00000199389	ENSP00000310649
ENSP00000199389	ENSP00000311005
ENSP00000199389	ENSP00000313603
ENSP00000199389	ENSP00000313674
ENSP00000199389	ENSP00000323302
ENSP00000199389	ENSP00000324122

ENSP00000199389	ENSP00000325448
ENSP00000199389	ENSP00000331310
ENSP00000199389	ENSP00000331817
ENSP00000199389	ENSP00000336702
ENSP00000199389	ENSP00000342381
ENSP00000199389	ENSP00000344220
ENSP00000199389	ENSP00000352738
ENSP00000199389	ENSP00000353575
ENSP00000199389	ENSP00000355884
ENSP00000199389	ENSP00000356548
ENSP00000199389	ENSP00000363308
ENSP00000199389	ENSP00000363313
ENSP00000199389	ENSP00000369042
ENSP00000199389	ENSP00000370258
ENSP00000199389	ENSP00000373082
ENSP00000199389	ENSP00000374399
ENSP00000199389	ENSP00000389065
ENSP00000199389	ENSP00000394869
ENSP00000199389	ENSP00000411327
ENSP00000199389	ENSP00000412553
ENSP00000199389	ENSP00000413319
ENSP00000199389	ENSP00000414373
ENSP00000199389	ENSP00000417229
ENSP00000199447	ENSP00000216185
ENSP00000199447	ENSP00000217515
ENSP00000199447	ENSP00000241052
ENSP00000199447	ENSP00000242576
ENSP00000199447	ENSP00000252487
ENSP00000199447	ENSP00000264065
ENSP00000199447	ENSP00000265462
ENSP00000199447	ENSP00000304668
ENSP00000199447	ENSP00000304908
ENSP00000199447	ENSP00000313921
ENSP00000199447	ENSP00000342026
ENSP00000199447	ENSP00000369757
ENSP00000199447	ENSP00000381293
ENSP00000199448	ENSP00000374455
ENSP00000199706	ENSP00000261908
ENSP00000199706	ENSP00000301585
ENSP00000199706	ENSP00000376204
ENSP00000199706	ENSP00000396622
ENSP00000199814	ENSP00000215829
ENSP00000199814	ENSP00000221232
ENSP00000199814	ENSP00000222969
ENSP00000199814	ENSP00000227524
ENSP00000199814	ENSP00000236273

ENSP00000199814	ENSP00000253108
ENSP00000199814	ENSP00000254193
ENSP00000199814	ENSP00000261531
ENSP00000199814	ENSP00000263657
ENSP00000199814	ENSP00000264233
ENSP00000199814	ENSP00000266735
ENSP00000199814	ENSP00000267812
ENSP00000199814	ENSP00000268482
ENSP00000199814	ENSP00000294053
ENSP00000199814	ENSP00000295373
ENSP00000199814	ENSP00000295488
ENSP00000199814	ENSP00000296215
ENSP00000199814	ENSP00000296702
ENSP00000199814	ENSP00000297151
ENSP00000199814	ENSP00000298532
ENSP00000199814	ENSP00000301740
ENSP00000199814	ENSP00000302160
ENSP00000199814	ENSP00000303191
ENSP00000199814	ENSP00000304350
ENSP00000199814	ENSP00000304370
ENSP00000199814	ENSP00000309166
ENSP00000199814	ENSP00000310448
ENSP00000199814	ENSP00000313674
ENSP00000199814	ENSP00000326531
ENSP00000199814	ENSP00000326806
ENSP00000199814	ENSP00000328992
ENSP00000199814	ENSP00000329419
ENSP00000199814	ENSP00000331907
ENSP00000199814	ENSP00000332103
ENSP00000199814	ENSP00000334148
ENSP00000199814	ENSP00000339090
ENSP00000199814	ENSP00000342374
ENSP00000199814	ENSP00000343557
ENSP00000199814	ENSP00000344758
ENSP00000199814	ENSP00000351137
ENSP00000199814	ENSP00000351492
ENSP00000199814	ENSP00000357048
ENSP00000199814	ENSP00000357631
ENSP00000199814	ENSP00000358554
ENSP00000199814	ENSP00000359454
ENSP00000199814	ENSP00000360532
ENSP00000199814	ENSP00000361577
ENSP00000199814	ENSP00000362592
ENSP00000199814	ENSP00000362688
ENSP00000199814	ENSP00000366557
ENSP00000199814	ENSP00000370258

ENSP00000199814	ENSP00000375863
ENSP00000199814	ENSP00000376973
ENSP00000199814	ENSP00000379607
ENSP00000199814	ENSP00000380336
ENSP00000199814	ENSP00000387006
ENSP00000199814	ENSP00000393953
ENSP00000199814	ENSP00000404151
ENSP00000199814	ENSP00000412566
ENSP00000199814	ENSP00000416959
ENSP00000200135	ENSP00000220659
ENSP00000200135	ENSP00000223369
ENSP00000200135	ENSP00000257700
ENSP00000200135	ENSP00000261597
ENSP00000200135	ENSP00000262812
ENSP00000200135	ENSP00000264028
ENSP00000200135	ENSP00000281513
ENSP00000200135	ENSP00000287598
ENSP00000200135	ENSP00000302530
ENSP00000200135	ENSP00000305810
ENSP00000200135	ENSP00000311429
ENSP00000200135	ENSP00000320130
ENSP00000200135	ENSP00000325002
ENSP00000200135	ENSP00000325863
ENSP00000200135	ENSP00000328236
ENSP00000200135	ENSP00000357048
ENSP00000200135	ENSP00000363055
ENSP00000200135	ENSP00000382562
ENSP00000200135	ENSP00000390783
ENSP00000200135	ENSP00000397394
ENSP00000200135	ENSP00000397435
ENSP00000200135	ENSP00000408910
ENSP00000200181	ENSP00000244769
ENSP00000200181	ENSP00000265354
ENSP00000200181	ENSP00000314879
ENSP00000200181	ENSP00000323856
ENSP00000200181	ENSP00000329797
ENSP00000200181	ENSP00000340937
ENSP00000200181	ENSP00000341189
ENSP00000200181	ENSP00000359596
ENSP00000200181	ENSP00000363559
ENSP00000200453	ENSP00000256383
ENSP00000200453	ENSP00000263121
ENSP00000200453	ENSP00000311825
ENSP00000200453	ENSP00000355001
ENSP00000200453	ENSP00000358953
ENSP00000200453	ENSP00000364398

ENSP00000200676	ENSP00000381031
ENSP00000200691	ENSP00000237014
ENSP00000200691	ENSP00000300086
ENSP00000201586	ENSP00000252699
ENSP00000201586	ENSP00000286190
ENSP00000202773	ENSP00000206249
ENSP00000202773	ENSP00000216554
ENSP00000202773	ENSP00000222247
ENSP00000202773	ENSP00000225430
ENSP00000202773	ENSP00000226796
ENSP00000202773	ENSP00000230050
ENSP00000202773	ENSP00000230859
ENSP00000202773	ENSP00000238112
ENSP00000202773	ENSP00000245539
ENSP00000202773	ENSP00000252543
ENSP00000202773	ENSP00000253099
ENSP00000202773	ENSP00000253788
ENSP00000202773	ENSP00000254037
ENSP00000202773	ENSP00000254940
ENSP00000202773	ENSP00000257829
ENSP00000202773	ENSP00000258455
ENSP00000202773	ENSP00000259469
ENSP00000202773	ENSP00000260102
ENSP00000202773	ENSP00000260443
ENSP00000202773	ENSP00000262584
ENSP00000202773	ENSP00000264995
ENSP00000202773	ENSP00000265100
ENSP00000202773	ENSP00000268616
ENSP00000202773	ENSP00000268661
ENSP00000202773	ENSP00000269499
ENSP00000202773	ENSP00000270625
ENSP00000202773	ENSP00000272418
ENSP00000202773	ENSP00000274242
ENSP00000202773	ENSP00000274606
ENSP00000202773	ENSP00000278572
ENSP00000202773	ENSP00000287038
ENSP00000202773	ENSP00000294189
ENSP00000202773	ENSP00000296490
ENSP00000202773	ENSP00000296674
ENSP00000202773	ENSP00000296802
ENSP00000202773	ENSP00000298283
ENSP00000202773	ENSP00000299767
ENSP00000202773	ENSP00000300151
ENSP00000202773	ENSP00000302896
ENSP00000202773	ENSP00000303315
ENSP00000202773	ENSP00000304353

ENSP00000202773	ENSP00000305702
ENSP00000202773	ENSP00000306548
ENSP00000202773	ENSP00000307889
ENSP00000202773	ENSP00000308897
ENSP00000202773	ENSP00000309334
ENSP00000202773	ENSP00000309830
ENSP00000202773	ENSP00000311028
ENSP00000202773	ENSP00000311430
ENSP00000202773	ENSP00000314348
ENSP00000202773	ENSP00000315791
ENSP00000202773	ENSP00000317376
ENSP00000202773	ENSP00000318646
ENSP00000202773	ENSP00000322419
ENSP00000202773	ENSP00000326981
ENSP00000202773	ENSP00000328690
ENSP00000202773	ENSP00000329466
ENSP00000202773	ENSP00000332444
ENSP00000202773	ENSP00000336702
ENSP00000202773	ENSP00000337053
ENSP00000202773	ENSP00000337839
ENSP00000202773	ENSP00000338862
ENSP00000202773	ENSP00000339095
ENSP00000202773	ENSP00000339795
ENSP00000202773	ENSP00000340019
ENSP00000202773	ENSP00000341885
ENSP00000202773	ENSP00000345096
ENSP00000202773	ENSP00000345156
ENSP00000202773	ENSP00000346015
ENSP00000202773	ENSP00000346022
ENSP00000202773	ENSP00000346027
ENSP00000202773	ENSP00000346037
ENSP00000202773	ENSP00000346050
ENSP00000202773	ENSP00000346088
ENSP00000202773	ENSP00000346120
ENSP00000202773	ENSP00000346725
ENSP00000202773	ENSP00000347087
ENSP00000202773	ENSP00000348010
ENSP00000202773	ENSP00000350708
ENSP00000202773	ENSP00000354040
ENSP00000202773	ENSP00000354525
ENSP00000202773	ENSP00000354687
ENSP00000202773	ENSP00000354739
ENSP00000202773	ENSP00000355899
ENSP00000202773	ENSP00000357748
ENSP00000202773	ENSP00000359127
ENSP00000202773	ENSP00000359345

ENSP00000202773	ENSP00000360286
ENSP00000202773	ENSP00000360922
ENSP00000202773	ENSP00000361076
ENSP00000202773	ENSP00000362744
ENSP00000202773	ENSP00000363018
ENSP00000202773	ENSP00000363559
ENSP00000202773	ENSP00000363676
ENSP00000202773	ENSP00000364320
ENSP00000202773	ENSP00000366519
ENSP00000202773	ENSP00000369757
ENSP00000202773	ENSP00000370083
ENSP00000202773	ENSP00000370589
ENSP00000202773	ENSP00000373404
ENSP00000202773	ENSP00000374390
ENSP00000202773	ENSP00000375730
ENSP00000202773	ENSP00000377640
ENSP00000202773	ENSP00000377865
ENSP00000202773	ENSP00000378160
ENSP00000202773	ENSP00000379607
ENSP00000202773	ENSP00000379888
ENSP00000202773	ENSP00000380156
ENSP00000202773	ENSP00000380982
ENSP00000202773	ENSP00000382392
ENSP00000202773	ENSP00000386717
ENSP00000202773	ENSP00000389103
ENSP00000202773	ENSP00000391723
ENSP00000202773	ENSP00000392859
ENSP00000202773	ENSP00000393393
ENSP00000202773	ENSP00000402338
ENSP00000202773	ENSP00000414321
ENSP00000202816	ENSP00000226796
ENSP00000202816	ENSP00000229214
ENSP00000202816	ENSP00000246802
ENSP00000202816	ENSP00000250615
ENSP00000202816	ENSP00000252115
ENSP00000202816	ENSP00000257527
ENSP00000202816	ENSP00000263657
ENSP00000202816	ENSP00000264995
ENSP00000202816	ENSP00000267425
ENSP00000202816	ENSP00000268661
ENSP00000202816	ENSP00000270625
ENSP00000202816	ENSP00000274606
ENSP00000202816	ENSP00000274849
ENSP00000202816	ENSP00000278572
ENSP00000202816	ENSP00000282999
ENSP00000202816	ENSP00000291576

ENSP00000202816	ENSP00000297990
ENSP00000202816	ENSP00000298283
ENSP00000202816	ENSP00000298746
ENSP00000202816	ENSP00000314311
ENSP00000202816	ENSP00000314348
ENSP00000202816	ENSP00000318195
ENSP00000202816	ENSP00000322396
ENSP00000202816	ENSP00000324122
ENSP00000202816	ENSP00000327179
ENSP00000202816	ENSP00000329466
ENSP00000202816	ENSP00000331817
ENSP00000202816	ENSP00000332340
ENSP00000202816	ENSP00000339795
ENSP00000202816	ENSP00000340823
ENSP00000202816	ENSP00000341885
ENSP00000202816	ENSP00000355541
ENSP00000202816	ENSP00000356290
ENSP00000202816	ENSP00000358563
ENSP00000202816	ENSP00000358812
ENSP00000202816	ENSP00000361982
ENSP00000202816	ENSP00000364320
ENSP00000202816	ENSP00000370589
ENSP00000202816	ENSP00000371101
ENSP00000202816	ENSP00000380982
ENSP00000202816	ENSP00000384490
ENSP00000202816	ENSP00000388920
ENSP00000202816	ENSP00000420357
ENSP00000203001	ENSP00000220496
ENSP00000203001	ENSP00000232905
ENSP00000203001	ENSP00000248553
ENSP00000203001	ENSP00000260956
ENSP00000203001	ENSP00000263791
ENSP00000203001	ENSP00000266126
ENSP00000203001	ENSP00000280557
ENSP00000203001	ENSP00000282728
ENSP00000203001	ENSP00000304668
ENSP00000203001	ENSP00000314311
ENSP00000203001	ENSP00000323424
ENSP00000203001	ENSP00000323880
ENSP00000203001	ENSP00000354125
ENSP00000203001	ENSP00000361010
ENSP00000203001	ENSP00000362688
ENSP00000203001	ENSP00000364543
ENSP00000203001	ENSP00000372827
ENSP00000203001	ENSP00000374399
ENSP00000203001	ENSP00000377344

ENSP00000203001	ENSP00000389182
ENSP00000203001	ENSP00000394400
ENSP00000203001	ENSP00000394869
ENSP00000203001	ENSP00000399530
ENSP00000203001	ENSP00000400626
ENSP00000203001	ENSP00000412310
ENSP00000203001	ENSP00000417229
ENSP00000203629	ENSP00000234170
ENSP00000203629	ENSP00000371308
ENSP00000204517	ENSP00000417281
ENSP00000204549	ENSP00000278919
ENSP00000204549	ENSP00000358605
ENSP00000204566	ENSP00000222008
ENSP00000204566	ENSP00000222330
ENSP00000204566	ENSP00000257787
ENSP00000204566	ENSP00000267484
ENSP00000204566	ENSP00000337838
ENSP00000204566	ENSP00000359507
ENSP00000204566	ENSP00000363985
ENSP00000204604	ENSP00000219551
ENSP00000204604	ENSP00000226574
ENSP00000204604	ENSP00000232003
ENSP00000204604	ENSP00000262120
ENSP00000204604	ENSP00000283147
ENSP00000204604	ENSP00000288840
ENSP00000204604	ENSP00000295025
ENSP00000204604	ENSP00000321326
ENSP00000204604	ENSP00000341551
ENSP00000204615	ENSP00000358105
ENSP00000204615	ENSP00000361548
ENSP00000204637	ENSP00000225430
ENSP00000204637	ENSP00000228280
ENSP00000204637	ENSP00000230050
ENSP00000204637	ENSP00000241453
ENSP00000204637	ENSP00000253099
ENSP00000204637	ENSP00000259469
ENSP00000204637	ENSP00000260102
ENSP00000204637	ENSP00000260443
ENSP00000204637	ENSP00000262584
ENSP00000204637	ENSP00000264995
ENSP00000204637	ENSP00000265100
ENSP00000204637	ENSP00000268661
ENSP00000204637	ENSP00000274242
ENSP00000204637	ENSP00000274606
ENSP00000204637	ENSP00000287038
ENSP00000204637	ENSP00000298283

ENSP00000204637	ENSP00000300151
ENSP00000204637	ENSP00000308897
ENSP00000204637	ENSP00000309334
ENSP00000204637	ENSP00000311430
ENSP00000204637	ENSP00000346015
ENSP00000204637	ENSP00000346022
ENSP00000204637	ENSP00000354525
ENSP00000204637	ENSP00000354739
ENSP00000204637	ENSP00000359345
ENSP00000204637	ENSP00000361076
ENSP00000204637	ENSP00000363676
ENSP00000204637	ENSP00000364320
ENSP00000204637	ENSP00000373404
ENSP00000204637	ENSP00000377640
ENSP00000204637	ENSP00000380156
ENSP00000204637	ENSP00000386717
ENSP00000204679	ENSP00000262803
ENSP00000204679	ENSP00000332887
ENSP00000204679	ENSP00000364188
ENSP00000204679	ENSP00000369335
ENSP00000204679	ENSP00000369965
ENSP00000204679	ENSP00000403817
ENSP00000204726	ENSP00000299424
ENSP00000204726	ENSP00000312664
ENSP00000204726	ENSP00000339435
ENSP00000204726	ENSP00000355599
ENSP00000204726	ENSP00000355777
ENSP00000204726	ENSP00000357484
ENSP00000204726	ENSP00000381045
ENSP00000204961	ENSP00000233154
ENSP00000204961	ENSP00000259406
ENSP00000204961	ENSP00000260130
ENSP00000204961	ENSP00000311005
ENSP00000204961	ENSP00000326366
ENSP00000204961	ENSP00000348550
ENSP00000204961	ENSP00000349465
ENSP00000204961	ENSP00000355136
ENSP00000204961	ENSP00000363763
ENSP00000204961	ENSP00000370410
ENSP00000204961	ENSP00000381098
ENSP00000204961	ENSP00000392423
ENSP00000204961	ENSP00000394794
ENSP00000205194	ENSP00000222008
ENSP00000205194	ENSP00000223273
ENSP00000205214	ENSP00000206595
ENSP00000205214	ENSP00000209875

ENSP00000205214	ENSP00000216133
ENSP00000205214	ENSP00000229595
ENSP00000205214	ENSP00000240055
ENSP00000205214	ENSP00000246112
ENSP00000205214	ENSP00000252029
ENSP00000205214	ENSP00000254900
ENSP00000205214	ENSP00000255631
ENSP00000205214	ENSP00000262144
ENSP00000205214	ENSP00000262518
ENSP00000205214	ENSP00000262803
ENSP00000205214	ENSP00000263360
ENSP00000205214	ENSP00000264705
ENSP00000205214	ENSP00000265195
ENSP00000205214	ENSP00000266744
ENSP00000205214	ENSP00000269385
ENSP00000205214	ENSP00000269397
ENSP00000205214	ENSP00000274031
ENSP00000205214	ENSP00000278618
ENSP00000205214	ENSP00000282030
ENSP00000205214	ENSP00000302216
ENSP00000205214	ENSP00000308750
ENSP00000205214	ENSP00000309457
ENSP00000205214	ENSP00000311825
ENSP00000205214	ENSP00000316578
ENSP00000205214	ENSP00000318646
ENSP00000205214	ENSP00000326477
ENSP00000205214	ENSP00000331057
ENSP00000205214	ENSP00000335044
ENSP00000205214	ENSP00000341280
ENSP00000205214	ENSP00000341805
ENSP00000205214	ENSP00000341992
ENSP00000205214	ENSP00000343885
ENSP00000205214	ENSP00000345096
ENSP00000205214	ENSP00000345445
ENSP00000205214	ENSP00000347169
ENSP00000205214	ENSP00000358417
ENSP00000205214	ENSP00000359321
ENSP00000205214	ENSP00000360515
ENSP00000205214	ENSP00000362344
ENSP00000205214	ENSP00000362592
ENSP00000205214	ENSP00000364289
ENSP00000205214	ENSP00000365735
ENSP00000205214	ENSP00000366135
ENSP00000205214	ENSP00000366953
ENSP00000205214	ENSP00000369042
ENSP00000205214	ENSP00000369335

ENSP00000205214	ENSP00000376204
ENSP00000205214	ENSP00000381436
ENSP00000205214	ENSP00000382688
ENSP00000205214	ENSP00000383295
ENSP00000205214	ENSP00000384490
ENSP00000205214	ENSP00000387982
ENSP00000205214	ENSP00000393275
ENSP00000205214	ENSP00000404658
ENSP00000205890	ENSP00000281172
ENSP00000205890	ENSP00000354623
ENSP00000205890	ENSP00000356694
ENSP00000205890	ENSP00000410833
ENSP00000205948	ENSP00000263816
ENSP00000205948	ENSP00000317872
ENSP00000205948	ENSP00000321334
ENSP00000205948	ENSP00000364709
ENSP00000206020	ENSP00000379282
ENSP00000206020	ENSP00000390746
ENSP00000206249	ENSP00000212015
ENSP00000206249	ENSP00000216037
ENSP00000206249	ENSP00000216297
ENSP00000206249	ENSP00000217130
ENSP00000206249	ENSP00000219431
ENSP00000206249	ENSP00000219476
ENSP00000206249	ENSP00000219548
ENSP00000206249	ENSP00000220764
ENSP00000206249	ENSP00000222247
ENSP00000206249	ENSP00000222254
ENSP00000206249	ENSP00000223073
ENSP00000206249	ENSP00000223641
ENSP00000206249	ENSP00000224073
ENSP00000206249	ENSP00000225430
ENSP00000206249	ENSP00000225916
ENSP00000206249	ENSP00000225983
ENSP00000206249	ENSP00000226574
ENSP00000206249	ENSP00000227507
ENSP00000206249	ENSP00000228251
ENSP00000206249	ENSP00000228534
ENSP00000206249	ENSP00000229595
ENSP00000206249	ENSP00000230050
ENSP00000206249	ENSP00000233146
ENSP00000206249	ENSP00000234420
ENSP00000206249	ENSP00000235372
ENSP00000206249	ENSP00000237530
ENSP00000206249	ENSP00000240304
ENSP00000206249	ENSP00000241600

ENSP00000206249	ENSP00000242057
ENSP00000206249	ENSP00000242351
ENSP00000206249	ENSP00000244741
ENSP00000206249	ENSP00000245479
ENSP00000206249	ENSP00000245812
ENSP00000206249	ENSP00000245960
ENSP00000206249	ENSP00000248114
ENSP00000206249	ENSP00000249636
ENSP00000206249	ENSP00000250003
ENSP00000206249	ENSP00000251289
ENSP00000206249	ENSP00000251871
ENSP00000206249	ENSP00000252543
ENSP00000206249	ENSP00000252699
ENSP00000206249	ENSP00000253363
ENSP00000206249	ENSP00000254630
ENSP00000206249	ENSP00000254803
ENSP00000206249	ENSP00000256379
ENSP00000206249	ENSP00000256429
ENSP00000206249	ENSP00000256897
ENSP00000206249	ENSP00000257430
ENSP00000206249	ENSP00000257829
ENSP00000206249	ENSP00000258455
ENSP00000206249	ENSP00000258648
ENSP00000206249	ENSP00000259406
ENSP00000206249	ENSP00000260210
ENSP00000206249	ENSP00000260947
ENSP00000206249	ENSP00000261245
ENSP00000206249	ENSP00000261413
ENSP00000206249	ENSP00000261531
ENSP00000206249	ENSP00000261875
ENSP00000206249	ENSP00000261884
ENSP00000206249	ENSP00000261893
ENSP00000206249	ENSP00000262030
ENSP00000206249	ENSP00000262077
ENSP00000206249	ENSP00000262213
ENSP00000206249	ENSP00000262367
ENSP00000206249	ENSP00000262584
ENSP00000206249	ENSP00000262753
ENSP00000206249	ENSP00000262803
ENSP00000206249	ENSP00000263121
ENSP00000206249	ENSP00000263663
ENSP00000206249	ENSP00000264451
ENSP00000206249	ENSP00000264515
ENSP00000206249	ENSP00000264637
ENSP00000206249	ENSP00000265350
ENSP00000206249	ENSP00000265963

ENSP00000206249	ENSP00000266079
ENSP00000206249	ENSP00000267064
ENSP00000206249	ENSP00000268182
ENSP00000206249	ENSP00000269122
ENSP00000206249	ENSP00000269701
ENSP00000206249	ENSP00000270625
ENSP00000206249	ENSP00000271002
ENSP00000206249	ENSP00000274031
ENSP00000206249	ENSP00000274793
ENSP00000206249	ENSP00000278572
ENSP00000206249	ENSP00000282892
ENSP00000206249	ENSP00000283943
ENSP00000206249	ENSP00000284957
ENSP00000206249	ENSP00000285298
ENSP00000206249	ENSP00000286317
ENSP00000206249	ENSP00000286523
ENSP00000206249	ENSP00000287295
ENSP00000206249	ENSP00000287820
ENSP00000206249	ENSP00000290231
ENSP00000206249	ENSP00000292385
ENSP00000206249	ENSP00000294189
ENSP00000206249	ENSP00000296255
ENSP00000206249	ENSP00000296509
ENSP00000206249	ENSP00000296674
ENSP00000206249	ENSP00000296930
ENSP00000206249	ENSP00000297185
ENSP00000206249	ENSP00000297347
ENSP00000206249	ENSP00000299424
ENSP00000206249	ENSP00000300413
ENSP00000206249	ENSP00000300651
ENSP00000206249	ENSP00000301740
ENSP00000206249	ENSP00000302111
ENSP00000206249	ENSP00000302640
ENSP00000206249	ENSP00000302896
ENSP00000206249	ENSP00000303315
ENSP00000206249	ENSP00000305422
ENSP00000206249	ENSP00000306245
ENSP00000206249	ENSP00000307260
ENSP00000206249	ENSP00000307684
ENSP00000206249	ENSP00000307863
ENSP00000206249	ENSP00000307889
ENSP00000206249	ENSP00000309334
ENSP00000206249	ENSP00000309474
ENSP00000206249	ENSP00000310785
ENSP00000206249	ENSP00000311028
ENSP00000206249	ENSP00000311430

ENSP00000206249	ENSP00000311747
ENSP00000206249	ENSP00000312649
ENSP00000206249	ENSP00000313158
ENSP00000206249	ENSP00000313420
ENSP00000206249	ENSP00000313829
ENSP00000206249	ENSP00000314949
ENSP00000206249	ENSP00000315397
ENSP00000206249	ENSP00000316879
ENSP00000206249	ENSP00000317872
ENSP00000206249	ENSP00000318195
ENSP00000206249	ENSP00000318646
ENSP00000206249	ENSP00000321503
ENSP00000206249	ENSP00000322716
ENSP00000206249	ENSP00000323720
ENSP00000206249	ENSP00000323856
ENSP00000206249	ENSP00000323858
ENSP00000206249	ENSP00000323967
ENSP00000206249	ENSP00000323978
ENSP00000206249	ENSP00000324573
ENSP00000206249	ENSP00000324956
ENSP00000206249	ENSP00000325120
ENSP00000206249	ENSP00000325376
ENSP00000206249	ENSP00000325612
ENSP00000206249	ENSP00000326261
ENSP00000206249	ENSP00000326519
ENSP00000206249	ENSP00000326767
ENSP00000206249	ENSP00000327072
ENSP00000206249	ENSP00000327197
ENSP00000206249	ENSP00000327213
ENSP00000206249	ENSP00000328111
ENSP00000206249	ENSP00000328773
ENSP00000206249	ENSP00000329002
ENSP00000206249	ENSP00000329357
ENSP00000206249	ENSP00000333188
ENSP00000206249	ENSP00000333275
ENSP00000206249	ENSP00000333633
ENSP00000206249	ENSP00000335321
ENSP00000206249	ENSP00000336817
ENSP00000206249	ENSP00000337088
ENSP00000206249	ENSP00000337194
ENSP00000206249	ENSP00000337513
ENSP00000206249	ENSP00000338157
ENSP00000206249	ENSP00000338235
ENSP00000206249	ENSP00000338607
ENSP00000206249	ENSP00000338983
ENSP00000206249	ENSP00000339145

ENSP00000206249	ENSP00000339191
ENSP00000206249	ENSP00000339495
ENSP00000206249	ENSP00000339527
ENSP00000206249	ENSP00000339529
ENSP00000206249	ENSP00000339795
ENSP00000206249	ENSP00000340019
ENSP00000206249	ENSP00000340761
ENSP00000206249	ENSP00000340896
ENSP00000206249	ENSP00000341208
ENSP00000206249	ENSP00000341551
ENSP00000206249	ENSP00000341692
ENSP00000206249	ENSP00000341885
ENSP00000206249	ENSP00000342137
ENSP00000206249	ENSP00000342307
ENSP00000206249	ENSP00000342434
ENSP00000206249	ENSP00000344456
ENSP00000206249	ENSP00000344547
ENSP00000206249	ENSP00000345156
ENSP00000206249	ENSP00000345179
ENSP00000206249	ENSP00000345917
ENSP00000206249	ENSP00000346015
ENSP00000206249	ENSP00000346022
ENSP00000206249	ENSP00000346027
ENSP00000206249	ENSP00000346050
ENSP00000206249	ENSP00000346067
ENSP00000206249	ENSP00000346088
ENSP00000206249	ENSP00000346120
ENSP00000206249	ENSP00000346206
ENSP00000206249	ENSP00000346634
ENSP00000206249	ENSP00000347733
ENSP00000206249	ENSP00000347948
ENSP00000206249	ENSP00000348069
ENSP00000206249	ENSP00000348128
ENSP00000206249	ENSP00000348551
ENSP00000206249	ENSP00000348610
ENSP00000206249	ENSP00000349049
ENSP00000206249	ENSP00000349977
ENSP00000206249	ENSP00000350283
ENSP00000206249	ENSP00000350512
ENSP00000206249	ENSP00000350854
ENSP00000206249	ENSP00000350894
ENSP00000206249	ENSP00000351407
ENSP00000206249	ENSP00000351894
ENSP00000206249	ENSP00000352257
ENSP00000206249	ENSP00000352463
ENSP00000206249	ENSP00000352738

ENSP00000206249	ENSP00000353622
ENSP00000206249	ENSP00000354040
ENSP00000206249	ENSP00000354061
ENSP00000206249	ENSP00000354522
ENSP00000206249	ENSP00000354718
ENSP00000206249	ENSP00000354739
ENSP00000206249	ENSP00000355011
ENSP00000206249	ENSP00000355759
ENSP00000206249	ENSP00000356520
ENSP00000206249	ENSP00000357047
ENSP00000206249	ENSP00000357341
ENSP00000206249	ENSP00000357448
ENSP00000206249	ENSP00000357643
ENSP00000206249	ENSP00000357711
ENSP00000206249	ENSP00000358404
ENSP00000206249	ENSP00000358554
ENSP00000206249	ENSP00000358563
ENSP00000206249	ENSP00000358778
ENSP00000206249	ENSP00000358857
ENSP00000206249	ENSP00000358953
ENSP00000206249	ENSP00000358994
ENSP00000206249	ENSP00000359345
ENSP00000206249	ENSP00000359531
ENSP00000206249	ENSP00000359818
ENSP00000206249	ENSP00000360025
ENSP00000206249	ENSP00000360031
ENSP00000206249	ENSP00000360828
ENSP00000206249	ENSP00000360922
ENSP00000206249	ENSP00000361010
ENSP00000206249	ENSP00000361076
ENSP00000206249	ENSP00000361824
ENSP00000206249	ENSP00000362153
ENSP00000206249	ENSP00000362208
ENSP00000206249	ENSP00000362592
ENSP00000206249	ENSP00000362637
ENSP00000206249	ENSP00000362688
ENSP00000206249	ENSP00000362744
ENSP00000206249	ENSP00000362820
ENSP00000206249	ENSP00000363018
ENSP00000206249	ENSP00000363193
ENSP00000206249	ENSP00000363676
ENSP00000206249	ENSP00000363745
ENSP00000206249	ENSP00000364839
ENSP00000206249	ENSP00000364895
ENSP00000206249	ENSP00000364929
ENSP00000206249	ENSP00000365016

ENSP00000206249	ENSP00000365025
ENSP00000206249	ENSP00000365439
ENSP00000206249	ENSP00000366135
ENSP00000206249	ENSP00000366413
ENSP00000206249	ENSP00000366434
ENSP00000206249	ENSP00000366607
ENSP00000206249	ENSP00000366915
ENSP00000206249	ENSP00000367263
ENSP00000206249	ENSP00000367406
ENSP00000206249	ENSP00000367797
ENSP00000206249	ENSP00000368253
ENSP00000206249	ENSP00000369716
ENSP00000206249	ENSP00000369757
ENSP00000206249	ENSP00000369965
ENSP00000206249	ENSP00000370589
ENSP00000206249	ENSP00000370968
ENSP00000206249	ENSP00000374213
ENSP00000206249	ENSP00000375730
ENSP00000206249	ENSP00000375863
ENSP00000206249	ENSP00000376309
ENSP00000206249	ENSP00000376611
ENSP00000206249	ENSP00000377262
ENSP00000206249	ENSP00000377640
ENSP00000206249	ENSP00000377944
ENSP00000206249	ENSP00000379888
ENSP00000206249	ENSP00000380414
ENSP00000206249	ENSP00000381031
ENSP00000206249	ENSP00000381045
ENSP00000206249	ENSP00000381098
ENSP00000206249	ENSP00000382250
ENSP00000206249	ENSP00000382392
ENSP00000206249	ENSP00000382423
ENSP00000206249	ENSP00000382688
ENSP00000206249	ENSP00000384109
ENSP00000206249	ENSP00000384675
ENSP00000206249	ENSP00000385722
ENSP00000206249	ENSP00000389103
ENSP00000206249	ENSP00000392466
ENSP00000206249	ENSP00000392601
ENSP00000206249	ENSP00000396704
ENSP00000206249	ENSP00000399968
ENSP00000206249	ENSP00000401277
ENSP00000206249	ENSP00000404121
ENSP00000206249	ENSP00000406888
ENSP00000206249	ENSP00000407487
ENSP00000206249	ENSP00000407879

ENSP00000206249	ENSP00000414187
ENSP00000206249	ENSP00000414321
ENSP00000206249	ENSP00000416097
ENSP00000206249	ENSP00000416110
ENSP00000206249	ENSP00000417281
ENSP00000206423	ENSP00000379607
ENSP00000206474	ENSP00000243706
ENSP00000206474	ENSP00000253669
ENSP00000206474	ENSP00000260372
ENSP00000206474	ENSP00000282058
ENSP00000206474	ENSP00000369871
ENSP00000206595	ENSP00000263360
ENSP00000206595	ENSP00000269197
ENSP00000206595	ENSP00000282030
ENSP00000206595	ENSP00000283351
ENSP00000206595	ENSP00000376204
ENSP00000206765	ENSP00000233190
ENSP00000206765	ENSP00000248553
ENSP00000206765	ENSP00000338345
ENSP00000206765	ENSP00000361855
ENSP00000207870	ENSP00000251038
ENSP00000207870	ENSP00000264029
ENSP00000207870	ENSP00000264705
ENSP00000207870	ENSP00000330408
ENSP00000207870	ENSP00000360268
ENSP00000209665	ENSP00000317992
ENSP00000209665	ENSP00000358812
ENSP00000209665	ENSP00000360412
ENSP00000209665	ENSP00000366519
ENSP00000209665	ENSP00000366557
ENSP00000209665	ENSP00000379865
ENSP00000209665	ENSP00000382392
ENSP00000209728	ENSP00000209875
ENSP00000209728	ENSP00000212015
ENSP00000209728	ENSP00000216367
ENSP00000209728	ENSP00000219097
ENSP00000209728	ENSP00000225983
ENSP00000209728	ENSP00000229201
ENSP00000209728	ENSP00000229595
ENSP00000209728	ENSP00000234296
ENSP00000209728	ENSP00000237654
ENSP00000209728	ENSP00000238789
ENSP00000209728	ENSP00000241071
ENSP00000209728	ENSP00000244741
ENSP00000209728	ENSP00000245960
ENSP00000209728	ENSP00000247843

ENSP00000209728	ENSP00000251038
ENSP00000209728	ENSP00000251074
ENSP00000209728	ENSP00000251289
ENSP00000209728	ENSP00000256442
ENSP00000209728	ENSP00000257789
ENSP00000209728	ENSP00000257934
ENSP00000209728	ENSP00000259008
ENSP00000209728	ENSP00000261424
ENSP00000209728	ENSP00000261881
ENSP00000209728	ENSP00000262315
ENSP00000209728	ENSP00000262519
ENSP00000209728	ENSP00000262854
ENSP00000209728	ENSP00000263043
ENSP00000209728	ENSP00000263121
ENSP00000209728	ENSP00000263201
ENSP00000209728	ENSP00000263228
ENSP00000209728	ENSP00000264108
ENSP00000209728	ENSP00000264169
ENSP00000209728	ENSP00000264335
ENSP00000209728	ENSP00000264515
ENSP00000209728	ENSP00000264977
ENSP00000209728	ENSP00000265056
ENSP00000209728	ENSP00000265465
ENSP00000209728	ENSP00000267197
ENSP00000209728	ENSP00000269127
ENSP00000209728	ENSP00000269397
ENSP00000209728	ENSP00000276014
ENSP00000209728	ENSP00000276603
ENSP00000209728	ENSP00000278412
ENSP00000209728	ENSP00000278616
ENSP00000209728	ENSP00000281708
ENSP00000209728	ENSP00000282572
ENSP00000209728	ENSP00000287598
ENSP00000209728	ENSP00000288976
ENSP00000209728	ENSP00000295066
ENSP00000209728	ENSP00000295926
ENSP00000209728	ENSP00000295989
ENSP00000209728	ENSP00000296273
ENSP00000209728	ENSP00000296292
ENSP00000209728	ENSP00000296509
ENSP00000209728	ENSP00000296755
ENSP00000209728	ENSP00000297431
ENSP00000209728	ENSP00000298852
ENSP00000209728	ENSP00000300231
ENSP00000209728	ENSP00000301019
ENSP00000209728	ENSP00000302530

ENSP00000209728	ENSP00000306983
ENSP00000209728	ENSP00000307853
ENSP00000209728	ENSP00000310520
ENSP00000209728	ENSP00000310596
ENSP00000209728	ENSP00000311083
ENSP00000209728	ENSP00000312042
ENSP00000209728	ENSP00000313171
ENSP00000209728	ENSP00000318944
ENSP00000209728	ENSP00000323099
ENSP00000209728	ENSP00000323439
ENSP00000209728	ENSP00000324122
ENSP00000209728	ENSP00000325677
ENSP00000209728	ENSP00000325863
ENSP00000209728	ENSP00000326813
ENSP00000209728	ENSP00000329419
ENSP00000209728	ENSP00000333836
ENSP00000209728	ENSP00000334061
ENSP00000209728	ENSP00000335357
ENSP00000209728	ENSP00000336868
ENSP00000209728	ENSP00000340879
ENSP00000209728	ENSP00000340896
ENSP00000209728	ENSP00000341382
ENSP00000209728	ENSP00000343701
ENSP00000209728	ENSP00000343741
ENSP00000209728	ENSP00000344504
ENSP00000209728	ENSP00000345917
ENSP00000209728	ENSP00000346725
ENSP00000209728	ENSP00000347087
ENSP00000209728	ENSP00000347168
ENSP00000209728	ENSP00000348577
ENSP00000209728	ENSP00000349887
ENSP00000209728	ENSP00000350767
ENSP00000209728	ENSP00000351284
ENSP00000209728	ENSP00000353332
ENSP00000209728	ENSP00000353793
ENSP00000209728	ENSP00000354040
ENSP00000209728	ENSP00000354720
ENSP00000209728	ENSP00000354916
ENSP00000209728	ENSP00000354927
ENSP00000209728	ENSP00000355153
ENSP00000209728	ENSP00000355388
ENSP00000209728	ENSP00000356405
ENSP00000209728	ENSP00000356655
ENSP00000209728	ENSP00000360116
ENSP00000209728	ENSP00000360412
ENSP00000209728	ENSP00000360621

ENSP00000209728	ENSP00000364119
ENSP00000209728	ENSP00000365005
ENSP00000209728	ENSP00000365811
ENSP00000209728	ENSP00000366974
ENSP00000209728	ENSP00000367034
ENSP00000209728	ENSP00000368438
ENSP00000209728	ENSP00000368976
ENSP00000209728	ENSP00000369411
ENSP00000209728	ENSP00000369716
ENSP00000209728	ENSP00000370151
ENSP00000209728	ENSP00000370589
ENSP00000209728	ENSP00000371475
ENSP00000209728	ENSP00000372023
ENSP00000209728	ENSP00000376945
ENSP00000209728	ENSP00000377747
ENSP00000209728	ENSP00000378350
ENSP00000209728	ENSP00000378529
ENSP00000209728	ENSP00000381657
ENSP00000209728	ENSP00000382423
ENSP00000209728	ENSP00000383986
ENSP00000209728	ENSP00000384048
ENSP00000209728	ENSP00000384211
ENSP00000209728	ENSP00000392028
ENSP00000209728	ENSP00000402338
ENSP00000209873	ENSP00000295025
ENSP00000209873	ENSP00000360286
ENSP00000209875	ENSP00000212015
ENSP00000209875	ENSP00000216297
ENSP00000209875	ENSP00000225983
ENSP00000209875	ENSP00000229201
ENSP00000209875	ENSP00000234296
ENSP00000209875	ENSP00000243326
ENSP00000209875	ENSP00000245919
ENSP00000209875	ENSP00000247087
ENSP00000209875	ENSP00000252799
ENSP00000209875	ENSP00000252996
ENSP00000209875	ENSP00000256474
ENSP00000209875	ENSP00000257789
ENSP00000209875	ENSP00000259456
ENSP00000209875	ENSP00000260403
ENSP00000209875	ENSP00000260731
ENSP00000209875	ENSP00000262133
ENSP00000209875	ENSP00000262198
ENSP00000209875	ENSP00000263208
ENSP00000209875	ENSP00000263381
ENSP00000209875	ENSP00000263753

ENSP00000209875	ENSP00000263816
ENSP00000209875	ENSP00000264709
ENSP00000209875	ENSP00000265073
ENSP00000209875	ENSP00000265100
ENSP00000209875	ENSP00000267163
ENSP00000209875	ENSP00000267807
ENSP00000209875	ENSP00000269468
ENSP00000209875	ENSP00000271715
ENSP00000209875	ENSP00000272163
ENSP00000209875	ENSP00000277225
ENSP00000209875	ENSP00000278412
ENSP00000209875	ENSP00000282516
ENSP00000209875	ENSP00000284811
ENSP00000209875	ENSP00000287437
ENSP00000209875	ENSP00000287598
ENSP00000209875	ENSP00000296509
ENSP00000209875	ENSP00000299550
ENSP00000209875	ENSP00000300835
ENSP00000209875	ENSP00000301280
ENSP00000209875	ENSP00000301764
ENSP00000209875	ENSP00000307023
ENSP00000209875	ENSP00000309457
ENSP00000209875	ENSP00000312042
ENSP00000209875	ENSP00000315700
ENSP00000209875	ENSP00000316121
ENSP00000209875	ENSP00000316578
ENSP00000209875	ENSP00000318086
ENSP00000209875	ENSP00000318115
ENSP00000209875	ENSP00000319104
ENSP00000209875	ENSP00000319169
ENSP00000209875	ENSP00000327077
ENSP00000209875	ENSP00000329357
ENSP00000209875	ENSP00000330074
ENSP00000209875	ENSP00000336817
ENSP00000209875	ENSP00000342905
ENSP00000209875	ENSP00000343023
ENSP00000209875	ENSP00000343745
ENSP00000209875	ENSP00000346067
ENSP00000209875	ENSP00000347168
ENSP00000209875	ENSP00000349049
ENSP00000209875	ENSP00000349723
ENSP00000209875	ENSP00000350767
ENSP00000209875	ENSP00000352257
ENSP00000209875	ENSP00000352516
ENSP00000209875	ENSP00000354730
ENSP00000209875	ENSP00000354995

ENSP00000209875	ENSP00000355944
ENSP00000209875	ENSP00000356263
ENSP00000209875	ENSP00000357260
ENSP00000209875	ENSP00000357643
ENSP00000209875	ENSP00000357965
ENSP00000209875	ENSP00000358778
ENSP00000209875	ENSP00000359345
ENSP00000209875	ENSP00000360621
ENSP00000209875	ENSP00000360660
ENSP00000209875	ENSP00000362306
ENSP00000209875	ENSP00000362441
ENSP00000209875	ENSP00000362592
ENSP00000209875	ENSP00000362650
ENSP00000209875	ENSP00000364839
ENSP00000209875	ENSP00000365877
ENSP00000209875	ENSP00000367034
ENSP00000209875	ENSP00000368349
ENSP00000209875	ENSP00000370557
ENSP00000209875	ENSP00000376127
ENSP00000209875	ENSP00000377655
ENSP00000209875	ENSP00000378295
ENSP00000209875	ENSP00000379607
ENSP00000209875	ENSP00000381565
ENSP00000209875	ENSP00000386227
ENSP00000209875	ENSP00000395535
ENSP00000209875	ENSP00000417980
ENSP00000210060	ENSP00000249923
ENSP00000210060	ENSP00000255194
ENSP00000210060	ENSP00000256658
ENSP00000210060	ENSP00000301740
ENSP00000210060	ENSP00000336702
ENSP00000210060	ENSP00000344055
ENSP00000210060	ENSP00000346022
ENSP00000210060	ENSP00000347836
ENSP00000210060	ENSP00000350199
ENSP00000210060	ENSP00000357907
ENSP00000210060	ENSP00000366156
ENSP00000210060	ENSP00000386394
ENSP00000210313	ENSP00000215071
ENSP00000210313	ENSP00000219313
ENSP00000210313	ENSP00000253003
ENSP00000210313	ENSP00000261208
ENSP00000210313	ENSP00000264639
ENSP00000210313	ENSP00000265849
ENSP00000210313	ENSP00000295901
ENSP00000210313	ENSP00000298852

ENSP00000210313	ENSP00000299259
ENSP00000210313	ENSP00000305682
ENSP00000210313	ENSP00000309474
ENSP00000210313	ENSP00000310129
ENSP00000210313	ENSP00000348442
ENSP00000210313	ENSP00000396937
ENSP00000210444	ENSP00000352928
ENSP00000210444	ENSP00000399511
ENSP00000211372	ENSP00000222247
ENSP00000211372	ENSP00000226796
ENSP00000211372	ENSP00000230050
ENSP00000211372	ENSP00000232905
ENSP00000211372	ENSP00000241600
ENSP00000211372	ENSP00000245539
ENSP00000211372	ENSP00000252543
ENSP00000211372	ENSP00000253233
ENSP00000211372	ENSP00000258455
ENSP00000211372	ENSP00000262584
ENSP00000211372	ENSP00000265100
ENSP00000211372	ENSP00000268206
ENSP00000211372	ENSP00000270625
ENSP00000211372	ENSP00000272418
ENSP00000211372	ENSP00000274606
ENSP00000211372	ENSP00000278572
ENSP00000211372	ENSP00000280557
ENSP00000211372	ENSP00000282050
ENSP00000211372	ENSP00000295930
ENSP00000211372	ENSP00000296674
ENSP00000211372	ENSP00000296802
ENSP00000211372	ENSP00000296805
ENSP00000211372	ENSP00000297908
ENSP00000211372	ENSP00000298283
ENSP00000211372	ENSP00000300151
ENSP00000211372	ENSP00000301585
ENSP00000211372	ENSP00000302896
ENSP00000211372	ENSP00000306548
ENSP00000211372	ENSP00000307889
ENSP00000211372	ENSP00000309334
ENSP00000211372	ENSP00000311028
ENSP00000211372	ENSP00000317376
ENSP00000211372	ENSP00000318646
ENSP00000211372	ENSP00000323856
ENSP00000211372	ENSP00000326981
ENSP00000211372	ENSP00000331019
ENSP00000211372	ENSP00000336702
ENSP00000211372	ENSP00000337127

ENSP00000211372	ENSP00000339095
ENSP00000211372	ENSP00000341885
ENSP00000211372	ENSP00000346022
ENSP00000211372	ENSP00000346050
ENSP00000211372	ENSP00000346067
ENSP00000211372	ENSP00000346725
ENSP00000211372	ENSP00000354525
ENSP00000211372	ENSP00000355471
ENSP00000211372	ENSP00000356134
ENSP00000211372	ENSP00000361076
ENSP00000211372	ENSP00000362036
ENSP00000211372	ENSP00000362744
ENSP00000211372	ENSP00000363308
ENSP00000211372	ENSP00000363676
ENSP00000211372	ENSP00000364320
ENSP00000211372	ENSP00000368790
ENSP00000211372	ENSP00000369757
ENSP00000211372	ENSP00000373404
ENSP00000211372	ENSP00000375730
ENSP00000211372	ENSP00000378160
ENSP00000211372	ENSP00000379339
ENSP00000211372	ENSP00000379888
ENSP00000211372	ENSP00000389103
ENSP00000211372	ENSP00000393393
ENSP00000211372	ENSP00000414321
ENSP00000211372	ENSP00000420714
ENSP00000211402	ENSP00000225916
ENSP00000211402	ENSP00000264193
ENSP00000211402	ENSP00000284719
ENSP00000211402	ENSP00000294053
ENSP00000211402	ENSP00000307567
ENSP00000211402	ENSP00000313350
ENSP00000211402	ENSP00000344537
ENSP00000211402	ENSP00000353741
ENSP00000211402	ENSP00000355890
ENSP00000211402	ENSP00000362576
ENSP00000211402	ENSP00000364794
ENSP00000211998	ENSP00000228307
ENSP00000211998	ENSP00000254336
ENSP00000211998	ENSP00000261017
ENSP00000211998	ENSP00000262304
ENSP00000211998	ENSP00000269122
ENSP00000211998	ENSP00000284776
ENSP00000211998	ENSP00000286627
ENSP00000211998	ENSP00000312435
ENSP00000211998	ENSP00000329466

ENSP00000211998	ENSP00000341189
ENSP00000211998	ENSP00000344456
ENSP00000211998	ENSP00000348128
ENSP00000211998	ENSP00000355136
ENSP00000211998	ENSP00000357907
ENSP00000211998	ENSP00000379607
ENSP00000212015	ENSP00000215587
ENSP00000212015	ENSP00000215793
ENSP00000212015	ENSP00000216277
ENSP00000212015	ENSP00000216294
ENSP00000212015	ENSP00000217244
ENSP00000212015	ENSP00000217901
ENSP00000212015	ENSP00000217964
ENSP00000212015	ENSP00000219476
ENSP00000212015	ENSP00000222122
ENSP00000212015	ENSP00000222553
ENSP00000212015	ENSP00000223129
ENSP00000212015	ENSP00000225916
ENSP00000212015	ENSP00000226574
ENSP00000212015	ENSP00000228280
ENSP00000212015	ENSP00000229201
ENSP00000212015	ENSP00000229595
ENSP00000212015	ENSP00000230124
ENSP00000212015	ENSP00000230859
ENSP00000212015	ENSP00000232424
ENSP00000212015	ENSP00000234296
ENSP00000212015	ENSP00000234626
ENSP00000212015	ENSP00000235521
ENSP00000212015	ENSP00000242728
ENSP00000212015	ENSP00000245206
ENSP00000212015	ENSP00000247843
ENSP00000212015	ENSP00000248673
ENSP00000212015	ENSP00000250003
ENSP00000212015	ENSP00000251038
ENSP00000212015	ENSP00000251363
ENSP00000212015	ENSP00000254101
ENSP00000212015	ENSP00000254605
ENSP00000212015	ENSP00000254719
ENSP00000212015	ENSP00000256442
ENSP00000212015	ENSP00000257829
ENSP00000212015	ENSP00000260526
ENSP00000212015	ENSP00000261531
ENSP00000212015	ENSP00000261881
ENSP00000212015	ENSP00000261944
ENSP00000212015	ENSP00000262519
ENSP00000212015	ENSP00000262803

ENSP00000212015	ENSP00000262982
ENSP00000212015	ENSP00000263201
ENSP00000212015	ENSP00000263208
ENSP00000212015	ENSP00000263377
ENSP00000212015	ENSP00000263857
ENSP00000212015	ENSP00000263881
ENSP00000212015	ENSP00000264093
ENSP00000212015	ENSP00000264108
ENSP00000212015	ENSP00000264183
ENSP00000212015	ENSP00000264335
ENSP00000212015	ENSP00000264705
ENSP00000212015	ENSP00000264951
ENSP00000212015	ENSP00000265056
ENSP00000212015	ENSP00000265433
ENSP00000212015	ENSP00000267163
ENSP00000212015	ENSP00000267197
ENSP00000212015	ENSP00000268043
ENSP00000212015	ENSP00000268058
ENSP00000212015	ENSP00000269143
ENSP00000212015	ENSP00000274031
ENSP00000212015	ENSP00000274242
ENSP00000212015	ENSP00000280190
ENSP00000212015	ENSP00000281043
ENSP00000212015	ENSP00000281708
ENSP00000212015	ENSP00000282388
ENSP00000212015	ENSP00000282541
ENSP00000212015	ENSP00000282884
ENSP00000212015	ENSP00000283195
ENSP00000212015	ENSP00000287820
ENSP00000212015	ENSP00000287957
ENSP00000212015	ENSP00000288976
ENSP00000212015	ENSP00000290158
ENSP00000212015	ENSP00000292432
ENSP00000212015	ENSP00000294053
ENSP00000212015	ENSP00000296328
ENSP00000212015	ENSP00000296504
ENSP00000212015	ENSP00000296674
ENSP00000212015	ENSP00000296755
ENSP00000212015	ENSP00000296930
ENSP00000212015	ENSP00000297185
ENSP00000212015	ENSP00000297338
ENSP00000212015	ENSP00000297431
ENSP00000212015	ENSP00000297792
ENSP00000212015	ENSP00000298139
ENSP00000212015	ENSP00000298937
ENSP00000212015	ENSP00000299626

ENSP00000212015	ENSP00000300231
ENSP00000212015	ENSP00000300291
ENSP00000212015	ENSP00000301788
ENSP00000212015	ENSP00000301843
ENSP00000212015	ENSP00000302478
ENSP00000212015	ENSP00000304802
ENSP00000212015	ENSP00000306561
ENSP00000212015	ENSP00000307208
ENSP00000212015	ENSP00000307272
ENSP00000212015	ENSP00000307889
ENSP00000212015	ENSP00000308741
ENSP00000212015	ENSP00000309474
ENSP00000212015	ENSP00000309555
ENSP00000212015	ENSP00000309572
ENSP00000212015	ENSP00000310042
ENSP00000212015	ENSP00000310448
ENSP00000212015	ENSP00000310459
ENSP00000212015	ENSP00000311344
ENSP00000212015	ENSP00000311873
ENSP00000212015	ENSP00000312735
ENSP00000212015	ENSP00000312778
ENSP00000212015	ENSP00000314080
ENSP00000212015	ENSP00000314420
ENSP00000212015	ENSP00000314949
ENSP00000212015	ENSP00000315644
ENSP00000212015	ENSP00000315700
ENSP00000212015	ENSP00000316328
ENSP00000212015	ENSP00000316578
ENSP00000212015	ENSP00000317333
ENSP00000212015	ENSP00000318977
ENSP00000212015	ENSP00000319501
ENSP00000212015	ENSP00000320346
ENSP00000212015	ENSP00000320566
ENSP00000212015	ENSP00000321636
ENSP00000212015	ENSP00000321711
ENSP00000212015	ENSP00000322419
ENSP00000212015	ENSP00000322570
ENSP00000212015	ENSP00000322579
ENSP00000212015	ENSP00000324124
ENSP00000212015	ENSP00000325863
ENSP00000212015	ENSP00000325941
ENSP00000212015	ENSP00000327070
ENSP00000212015	ENSP00000328854
ENSP00000212015	ENSP00000330074
ENSP00000212015	ENSP00000332171
ENSP00000212015	ENSP00000333813

ENSP00000212015	ENSP00000333920
ENSP00000212015	ENSP00000333948
ENSP00000212015	ENSP00000334675
ENSP00000212015	ENSP00000334836
ENSP00000212015	ENSP00000335321
ENSP00000212015	ENSP00000335357
ENSP00000212015	ENSP00000337034
ENSP00000212015	ENSP00000337127
ENSP00000212015	ENSP00000339095
ENSP00000212015	ENSP00000339527
ENSP00000212015	ENSP00000340019
ENSP00000212015	ENSP00000340684
ENSP00000212015	ENSP00000340820
ENSP00000212015	ENSP00000341024
ENSP00000212015	ENSP00000341957
ENSP00000212015	ENSP00000342056
ENSP00000212015	ENSP00000343313
ENSP00000212015	ENSP00000343392
ENSP00000212015	ENSP00000343741
ENSP00000212015	ENSP00000344456
ENSP00000212015	ENSP00000345656
ENSP00000212015	ENSP00000346088
ENSP00000212015	ENSP00000346227
ENSP00000212015	ENSP00000346437
ENSP00000212015	ENSP00000346566
ENSP00000212015	ENSP00000346987
ENSP00000212015	ENSP00000347232
ENSP00000212015	ENSP00000347733
ENSP00000212015	ENSP00000347978
ENSP00000212015	ENSP00000348069
ENSP00000212015	ENSP00000348089
ENSP00000212015	ENSP00000348107
ENSP00000212015	ENSP00000348965
ENSP00000212015	ENSP00000349049
ENSP00000212015	ENSP00000349213
ENSP00000212015	ENSP00000349723
ENSP00000212015	ENSP00000349892
ENSP00000212015	ENSP00000350283
ENSP00000212015	ENSP00000351284
ENSP00000212015	ENSP00000352257
ENSP00000212015	ENSP00000352516
ENSP00000212015	ENSP00000353622
ENSP00000212015	ENSP00000354347
ENSP00000212015	ENSP00000354522
ENSP00000212015	ENSP00000354558
ENSP00000212015	ENSP00000354720

ENSP00000212015	ENSP00000354916
ENSP00000212015	ENSP00000355185
ENSP00000212015	ENSP00000355731
ENSP00000212015	ENSP00000356146
ENSP00000212015	ENSP00000356379
ENSP00000212015	ENSP00000356694
ENSP00000212015	ENSP00000357348
ENSP00000212015	ENSP00000357748
ENSP00000212015	ENSP00000357881
ENSP00000212015	ENSP00000357907
ENSP00000212015	ENSP00000358576
ENSP00000212015	ENSP00000360025
ENSP00000212015	ENSP00000360532
ENSP00000212015	ENSP00000360621
ENSP00000212015	ENSP00000360798
ENSP00000212015	ENSP00000361287
ENSP00000212015	ENSP00000361433
ENSP00000212015	ENSP00000364260
ENSP00000212015	ENSP00000364270
ENSP00000212015	ENSP00000364524
ENSP00000212015	ENSP00000364929
ENSP00000212015	ENSP00000365016
ENSP00000212015	ENSP00000365439
ENSP00000212015	ENSP00000365588
ENSP00000212015	ENSP00000365877
ENSP00000212015	ENSP00000366974
ENSP00000212015	ENSP00000367797
ENSP00000212015	ENSP00000368349
ENSP00000212015	ENSP00000368438
ENSP00000212015	ENSP00000369042
ENSP00000212015	ENSP00000369335
ENSP00000212015	ENSP00000369497
ENSP00000212015	ENSP00000369695
ENSP00000212015	ENSP00000369716
ENSP00000212015	ENSP00000371475
ENSP00000212015	ENSP00000372023
ENSP00000212015	ENSP00000373060
ENSP00000212015	ENSP00000374213
ENSP00000212015	ENSP00000377655
ENSP00000212015	ENSP00000377865
ENSP00000212015	ENSP00000378812
ENSP00000212015	ENSP00000380504
ENSP00000212015	ENSP00000381198
ENSP00000212015	ENSP00000381657
ENSP00000212015	ENSP00000381932
ENSP00000212015	ENSP00000386456

ENSP00000212015	ENSP00000386759
ENSP00000212015	ENSP00000387429
ENSP00000212015	ENSP00000391723
ENSP00000212015	ENSP00000394791
ENSP00000212015	ENSP00000395535
ENSP00000212015	ENSP00000398930
ENSP00000212015	ENSP00000399968
ENSP00000212015	ENSP00000401508
ENSP00000212015	ENSP00000402527
ENSP00000212015	ENSP00000404318
ENSP00000212015	ENSP00000409167
ENSP00000212015	ENSP00000418111
ENSP00000212355	ENSP00000242208
ENSP00000212355	ENSP00000243786
ENSP00000212355	ENSP00000307046
ENSP00000212355	ENSP00000340698
ENSP00000212355	ENSP00000341803
ENSP00000212355	ENSP00000355896
ENSP00000212355	ENSP00000362299
ENSP00000215071	ENSP00000216455
ENSP00000215071	ENSP00000219313
ENSP00000215071	ENSP00000220849
ENSP00000215071	ENSP00000241071
ENSP00000215071	ENSP00000245960
ENSP00000215071	ENSP00000248566
ENSP00000215071	ENSP00000253003
ENSP00000215071	ENSP00000259457
ENSP00000215071	ENSP00000261211
ENSP00000215071	ENSP00000261601
ENSP00000215071	ENSP00000261712
ENSP00000215071	ENSP00000261758
ENSP00000215071	ENSP00000261881
ENSP00000215071	ENSP00000262193
ENSP00000215071	ENSP00000264639
ENSP00000215071	ENSP00000265990
ENSP00000215071	ENSP00000268802
ENSP00000215071	ENSP00000269143
ENSP00000215071	ENSP00000281708
ENSP00000215071	ENSP00000290541
ENSP00000215071	ENSP00000295901
ENSP00000215071	ENSP00000298852
ENSP00000215071	ENSP00000299206
ENSP00000215071	ENSP00000299259
ENSP00000215071	ENSP00000300738
ENSP00000215071	ENSP00000303279
ENSP00000215071	ENSP00000309474

ENSP00000215071	ENSP00000310129
ENSP00000215071	ENSP00000311665
ENSP00000215071	ENSP00000313350
ENSP00000215071	ENSP00000315674
ENSP00000215071	ENSP00000318944
ENSP00000215071	ENSP00000320508
ENSP00000215071	ENSP00000321711
ENSP00000215071	ENSP00000333122
ENSP00000215071	ENSP00000333950
ENSP00000215071	ENSP00000334188
ENSP00000215071	ENSP00000337501
ENSP00000215071	ENSP00000339109
ENSP00000215071	ENSP00000340836
ENSP00000215071	ENSP00000347997
ENSP00000215071	ENSP00000348442
ENSP00000215071	ENSP00000349785
ENSP00000215071	ENSP00000349970
ENSP00000215071	ENSP00000350708
ENSP00000215071	ENSP00000351947
ENSP00000215071	ENSP00000352219
ENSP00000215071	ENSP00000352257
ENSP00000215071	ENSP00000354032
ENSP00000215071	ENSP00000354558
ENSP00000215071	ENSP00000354883
ENSP00000215071	ENSP00000356655
ENSP00000215071	ENSP00000360216
ENSP00000215071	ENSP00000362110
ENSP00000215071	ENSP00000362334
ENSP00000215071	ENSP00000364794
ENSP00000215071	ENSP00000369531
ENSP00000215071	ENSP00000370408
ENSP00000215071	ENSP00000376945
ENSP00000215071	ENSP00000384211
ENSP00000215071	ENSP00000386212
ENSP00000215071	ENSP00000391723
ENSP00000215071	ENSP00000396937
ENSP00000215071	ENSP00000417748
ENSP00000215115	ENSP00000261531
ENSP00000215115	ENSP00000263121
ENSP00000215115	ENSP00000320485
ENSP00000215115	ENSP00000323967
ENSP00000215115	ENSP00000335044
ENSP00000215115	ENSP00000378180
ENSP00000215375	ENSP00000216194
ENSP00000215375	ENSP00000220751
ENSP00000215375	ENSP00000236067

ENSP00000215375	ENSP00000251038
ENSP00000215375	ENSP00000262030
ENSP00000215375	ENSP00000265537
ENSP00000215375	ENSP00000273258
ENSP00000215375	ENSP00000277865
ENSP00000215375	ENSP00000282050
ENSP00000215375	ENSP00000284727
ENSP00000215375	ENSP00000290299
ENSP00000215375	ENSP00000292475
ENSP00000215375	ENSP00000295901
ENSP00000215375	ENSP00000300688
ENSP00000215375	ENSP00000301587
ENSP00000215375	ENSP00000306003
ENSP00000215375	ENSP00000335203
ENSP00000215375	ENSP00000343313
ENSP00000215375	ENSP00000349142
ENSP00000215375	ENSP00000349325
ENSP00000215375	ENSP00000349877
ENSP00000215375	ENSP00000354632
ENSP00000215375	ENSP00000354676
ENSP00000215375	ENSP00000358737
ENSP00000215375	ENSP00000361686
ENSP00000215375	ENSP00000364188
ENSP00000215375	ENSP00000370414
ENSP00000215375	ENSP00000377954
ENSP00000215375	ENSP00000387262
ENSP00000215375	ENSP00000389649
ENSP00000215479	ENSP00000267426
ENSP00000215479	ENSP00000333697
ENSP00000215479	ENSP00000365131
ENSP00000215479	ENSP00000393887
ENSP00000215565	ENSP00000252102
ENSP00000215565	ENSP00000308351
ENSP00000215565	ENSP00000348965
ENSP00000215565	ENSP00000367265
ENSP00000215570	ENSP00000223129
ENSP00000215570	ENSP00000253003
ENSP00000215570	ENSP00000257245
ENSP00000215570	ENSP00000258418
ENSP00000215570	ENSP00000295767
ENSP00000215570	ENSP00000307023
ENSP00000215570	ENSP00000315702
ENSP00000215570	ENSP00000320236
ENSP00000215570	ENSP00000321239
ENSP00000215570	ENSP00000335493
ENSP00000215570	ENSP00000338293

ENSP00000215570	ENSP00000353622
ENSP00000215570	ENSP00000356405
ENSP00000215570	ENSP00000357360
ENSP00000215570	ENSP00000360157
ENSP00000215570	ENSP00000361993
ENSP00000215570	ENSP00000365766
ENSP00000215570	ENSP00000368332
ENSP00000215570	ENSP00000389934
ENSP00000215587	ENSP00000215739
ENSP00000215587	ENSP00000216297
ENSP00000215587	ENSP00000219252
ENSP00000215587	ENSP00000221859
ENSP00000215587	ENSP00000222567
ENSP00000215587	ENSP00000225504
ENSP00000215587	ENSP00000237380
ENSP00000215587	ENSP00000237455
ENSP00000215587	ENSP00000238789
ENSP00000215587	ENSP00000242719
ENSP00000215587	ENSP00000247843
ENSP00000215587	ENSP00000247970
ENSP00000215587	ENSP00000251038
ENSP00000215587	ENSP00000253107
ENSP00000215587	ENSP00000254719
ENSP00000215587	ENSP00000255764
ENSP00000215587	ENSP00000256257
ENSP00000215587	ENSP00000259605
ENSP00000215587	ENSP00000260956
ENSP00000215587	ENSP00000261531
ENSP00000215587	ENSP00000262608
ENSP00000215587	ENSP00000263390
ENSP00000215587	ENSP00000263795
ENSP00000215587	ENSP00000263857
ENSP00000215587	ENSP00000263997
ENSP00000215587	ENSP00000264718
ENSP00000215587	ENSP00000268058
ENSP00000215587	ENSP00000268711
ENSP00000215587	ENSP00000270225
ENSP00000215587	ENSP00000272645
ENSP00000215587	ENSP00000282326
ENSP00000215587	ENSP00000283875
ENSP00000215587	ENSP00000286317
ENSP00000215587	ENSP00000286835
ENSP00000215587	ENSP00000288199
ENSP00000215587	ENSP00000288976
ENSP00000215587	ENSP00000292363
ENSP00000215587	ENSP00000293860

ENSP00000215587	ENSP00000295131
ENSP00000215587	ENSP00000295321
ENSP00000215587	ENSP00000299424
ENSP00000215587	ENSP00000299543
ENSP00000215587	ENSP00000299601
ENSP00000215587	ENSP00000299767
ENSP00000215587	ENSP00000299853
ENSP00000215587	ENSP00000300651
ENSP00000215587	ENSP00000301788
ENSP00000215587	ENSP00000302478
ENSP00000215587	ENSP00000303088
ENSP00000215587	ENSP00000303515
ENSP00000215587	ENSP00000304321
ENSP00000215587	ENSP00000306245
ENSP00000215587	ENSP00000306906
ENSP00000215587	ENSP00000306983
ENSP00000215587	ENSP00000307208
ENSP00000215587	ENSP00000310260
ENSP00000215587	ENSP00000311977
ENSP00000215587	ENSP00000312735
ENSP00000215587	ENSP00000314311
ENSP00000215587	ENSP00000314949
ENSP00000215587	ENSP00000315950
ENSP00000215587	ENSP00000317636
ENSP00000215587	ENSP00000318115
ENSP00000215587	ENSP00000318641
ENSP00000215587	ENSP00000323720
ENSP00000215587	ENSP00000324124
ENSP00000215587	ENSP00000326767
ENSP00000215587	ENSP00000327889
ENSP00000215587	ENSP00000331111
ENSP00000215587	ENSP00000332340
ENSP00000215587	ENSP00000334564
ENSP00000215587	ENSP00000335357
ENSP00000215587	ENSP00000337340
ENSP00000215587	ENSP00000340823
ENSP00000215587	ENSP00000342121
ENSP00000215587	ENSP00000342889
ENSP00000215587	ENSP00000343129
ENSP00000215587	ENSP00000345530
ENSP00000215587	ENSP00000347345
ENSP00000215587	ENSP00000348168
ENSP00000215587	ENSP00000349213
ENSP00000215587	ENSP00000350967
ENSP00000215587	ENSP00000354775
ENSP00000215587	ENSP00000354916

ENSP00000215587	ENSP00000356146
ENSP00000215587	ENSP00000359531
ENSP00000215587	ENSP00000360473
ENSP00000215587	ENSP00000361465
ENSP00000215587	ENSP00000362546
ENSP00000215587	ENSP00000362900
ENSP00000215587	ENSP00000363193
ENSP00000215587	ENSP00000363734
ENSP00000215587	ENSP00000363779
ENSP00000215587	ENSP00000366828
ENSP00000215587	ENSP00000367029
ENSP00000215587	ENSP00000369695
ENSP00000215587	ENSP00000371546
ENSP00000215587	ENSP00000371800
ENSP00000215587	ENSP00000372694
ENSP00000215587	ENSP00000376097
ENSP00000215587	ENSP00000377969
ENSP00000215587	ENSP00000378736
ENSP00000215587	ENSP00000379607
ENSP00000215587	ENSP00000381064
ENSP00000215587	ENSP00000383503
ENSP00000215587	ENSP00000386759
ENSP00000215587	ENSP00000397636
ENSP00000215587	ENSP00000402515
ENSP00000215587	ENSP00000403852
ENSP00000215587	ENSP00000405636
ENSP00000215587	ENSP00000406293
ENSP00000215587	ENSP00000410530
ENSP00000215587	ENSP00000410954
ENSP00000215587	ENSP00000412319
ENSP00000215587	ENSP00000414110
ENSP00000215587	ENSP00000415678
ENSP00000215587	ENSP00000416599
ENSP00000215730	ENSP00000222547
ENSP00000215730	ENSP00000223369
ENSP00000215730	ENSP00000225567
ENSP00000215730	ENSP00000225724
ENSP00000215730	ENSP00000230449
ENSP00000215730	ENSP00000231668
ENSP00000215730	ENSP00000236192
ENSP00000215730	ENSP00000249647
ENSP00000215730	ENSP00000253861
ENSP00000215730	ENSP00000256897
ENSP00000215730	ENSP00000258301
ENSP00000215730	ENSP00000259400
ENSP00000215730	ENSP00000260818

ENSP00000215730	ENSP00000263277
ENSP00000215730	ENSP00000263897
ENSP00000215730	ENSP00000264157
ENSP00000215730	ENSP00000272427
ENSP00000215730	ENSP00000286448
ENSP00000215730	ENSP00000294179
ENSP00000215730	ENSP00000295926
ENSP00000215730	ENSP00000305255
ENSP00000215730	ENSP00000305810
ENSP00000215730	ENSP00000310521
ENSP00000215730	ENSP00000312027
ENSP00000215730	ENSP00000317714
ENSP00000215730	ENSP00000323377
ENSP00000215730	ENSP00000324628
ENSP00000215730	ENSP00000327650
ENSP00000215730	ENSP00000332326
ENSP00000215730	ENSP00000334100
ENSP00000215730	ENSP00000336790
ENSP00000215730	ENSP00000337501
ENSP00000215730	ENSP00000339191
ENSP00000215730	ENSP00000342793
ENSP00000215730	ENSP00000351926
ENSP00000215730	ENSP00000353564
ENSP00000215730	ENSP00000360183
ENSP00000215730	ENSP00000361048
ENSP00000215730	ENSP00000362399
ENSP00000215730	ENSP00000363055
ENSP00000215730	ENSP00000366225
ENSP00000215730	ENSP00000368976
ENSP00000215730	ENSP00000369409
ENSP00000215730	ENSP00000370557
ENSP00000215730	ENSP00000374529
ENSP00000215730	ENSP00000381293
ENSP00000215730	ENSP00000389934
ENSP00000215730	ENSP00000390783
ENSP00000215730	ENSP00000417748
ENSP00000215739	ENSP00000234170
ENSP00000215739	ENSP00000245960
ENSP00000215739	ENSP00000256398
ENSP00000215739	ENSP00000256442
ENSP00000215739	ENSP00000260383
ENSP00000215739	ENSP00000260731
ENSP00000215739	ENSP00000261597
ENSP00000215739	ENSP00000262300
ENSP00000215739	ENSP00000271452
ENSP00000215739	ENSP00000274680

ENSP00000215739	ENSP00000286317
ENSP00000215739	ENSP00000296509
ENSP00000215739	ENSP00000300231
ENSP00000215739	ENSP00000301280
ENSP00000215739	ENSP00000301332
ENSP00000215739	ENSP00000311344
ENSP00000215739	ENSP00000312735
ENSP00000215739	ENSP00000320309
ENSP00000215739	ENSP00000322957
ENSP00000215739	ENSP00000323074
ENSP00000215739	ENSP00000325612
ENSP00000215739	ENSP00000337926
ENSP00000215739	ENSP00000338629
ENSP00000215739	ENSP00000339299
ENSP00000215739	ENSP00000343023
ENSP00000215739	ENSP00000346762
ENSP00000215739	ENSP00000348215
ENSP00000215739	ENSP00000348577
ENSP00000215739	ENSP00000353796
ENSP00000215739	ENSP00000354158
ENSP00000215739	ENSP00000354526
ENSP00000215739	ENSP00000354633
ENSP00000215739	ENSP00000355672
ENSP00000215739	ENSP00000355759
ENSP00000215739	ENSP00000355922
ENSP00000215739	ENSP00000357643
ENSP00000215739	ENSP00000357682
ENSP00000215739	ENSP00000359740
ENSP00000215739	ENSP00000359837
ENSP00000215739	ENSP00000366271
ENSP00000215739	ENSP00000377265
ENSP00000215739	ENSP00000380129
ENSP00000215739	ENSP00000384675
ENSP00000215739	ENSP00000417980
ENSP00000215742	ENSP00000263663
ENSP00000215742	ENSP00000304689
ENSP00000215742	ENSP00000309555
ENSP00000215742	ENSP00000361048
ENSP00000215780	ENSP00000219409
ENSP00000215780	ENSP00000284719
ENSP00000215780	ENSP00000294053
ENSP00000215780	ENSP00000307567
ENSP00000215780	ENSP00000353741
ENSP00000215780	ENSP00000355890
ENSP00000215781	ENSP00000258743
ENSP00000215781	ENSP00000263409

ENSP00000215781	ENSP00000338799
ENSP00000215793	ENSP00000215829
ENSP00000215793	ENSP00000215957
ENSP00000215793	ENSP00000221494
ENSP00000215793	ENSP00000221566
ENSP00000215793	ENSP00000225983
ENSP00000215793	ENSP00000227524
ENSP00000215793	ENSP00000228284
ENSP00000215793	ENSP00000235090
ENSP00000215793	ENSP00000252699
ENSP00000215793	ENSP00000254193
ENSP00000215793	ENSP00000257548
ENSP00000215793	ENSP00000261531
ENSP00000215793	ENSP00000262056
ENSP00000215793	ENSP00000264233
ENSP00000215793	ENSP00000266079
ENSP00000215793	ENSP00000266735
ENSP00000215793	ENSP00000267812
ENSP00000215793	ENSP00000268206
ENSP00000215793	ENSP00000268482
ENSP00000215793	ENSP00000268483
ENSP00000215793	ENSP00000271628
ENSP00000215793	ENSP00000274712
ENSP00000215793	ENSP00000295488
ENSP00000215793	ENSP00000296702
ENSP00000215793	ENSP00000296805
ENSP00000215793	ENSP00000300413
ENSP00000215793	ENSP00000301740
ENSP00000215793	ENSP00000301764
ENSP00000215793	ENSP00000302160
ENSP00000215793	ENSP00000303191
ENSP00000215793	ENSP00000304350
ENSP00000215793	ENSP00000304370
ENSP00000215793	ENSP00000305790
ENSP00000215793	ENSP00000310448
ENSP00000215793	ENSP00000312411
ENSP00000215793	ENSP00000312671
ENSP00000215793	ENSP00000315035
ENSP00000215793	ENSP00000315379
ENSP00000215793	ENSP00000317790
ENSP00000215793	ENSP00000318861
ENSP00000215793	ENSP00000319341
ENSP00000215793	ENSP00000322608
ENSP00000215793	ENSP00000323302
ENSP00000215793	ENSP00000324122
ENSP00000215793	ENSP00000326806

ENSP00000215793	ENSP00000329357
ENSP00000215793	ENSP00000329466
ENSP00000215793	ENSP00000332103
ENSP00000215793	ENSP00000334148
ENSP00000215793	ENSP00000335321
ENSP00000215793	ENSP00000337405
ENSP00000215793	ENSP00000342374
ENSP00000215793	ENSP00000349785
ENSP00000215793	ENSP00000352308
ENSP00000215793	ENSP00000356520
ENSP00000215793	ENSP00000357631
ENSP00000215793	ENSP00000359454
ENSP00000215793	ENSP00000360286
ENSP00000215793	ENSP00000360532
ENSP00000215793	ENSP00000361577
ENSP00000215793	ENSP00000361824
ENSP00000215793	ENSP00000362110
ENSP00000215793	ENSP00000363313
ENSP00000215793	ENSP00000364813
ENSP00000215793	ENSP00000366557
ENSP00000215793	ENSP00000366607
ENSP00000215793	ENSP00000366829
ENSP00000215793	ENSP00000368801
ENSP00000215793	ENSP00000370589
ENSP00000215793	ENSP00000378350
ENSP00000215793	ENSP00000379394
ENSP00000215793	ENSP00000379607
ENSP00000215793	ENSP00000386161
ENSP00000215793	ENSP00000400591
ENSP00000215793	ENSP00000410758
ENSP00000215793	ENSP00000412566
ENSP00000215793	ENSP00000414006
ENSP00000215793	ENSP00000414634
ENSP00000215829	ENSP00000221448
ENSP00000215829	ENSP00000221494
ENSP00000215829	ENSP00000227524
ENSP00000215829	ENSP00000235090
ENSP00000215829	ENSP00000240304
ENSP00000215829	ENSP00000243563
ENSP00000215829	ENSP00000244520
ENSP00000215829	ENSP00000251038
ENSP00000215829	ENSP00000252622
ENSP00000215829	ENSP00000254193
ENSP00000215829	ENSP00000261531
ENSP00000215829	ENSP00000261973
ENSP00000215829	ENSP00000262962

ENSP00000215829	ENSP00000266079
ENSP00000215829	ENSP00000266735
ENSP00000215829	ENSP00000267812
ENSP00000215829	ENSP00000268483
ENSP00000215829	ENSP00000270257
ENSP00000215829	ENSP00000272645
ENSP00000215829	ENSP00000274712
ENSP00000215829	ENSP00000281950
ENSP00000215829	ENSP00000285873
ENSP00000215829	ENSP00000290158
ENSP00000215829	ENSP00000293404
ENSP00000215829	ENSP00000293406
ENSP00000215829	ENSP00000296702
ENSP00000215829	ENSP00000300146
ENSP00000215829	ENSP00000300413
ENSP00000215829	ENSP00000301740
ENSP00000215829	ENSP00000301764
ENSP00000215829	ENSP00000302160
ENSP00000215829	ENSP00000303191
ENSP00000215829	ENSP00000304350
ENSP00000215829	ENSP00000304370
ENSP00000215829	ENSP00000305790
ENSP00000215829	ENSP00000308533
ENSP00000215829	ENSP00000308546
ENSP00000215829	ENSP00000309830
ENSP00000215829	ENSP00000310448
ENSP00000215829	ENSP00000310596
ENSP00000215829	ENSP00000313674
ENSP00000215829	ENSP00000314425
ENSP00000215829	ENSP00000315035
ENSP00000215829	ENSP00000315379
ENSP00000215829	ENSP00000318861
ENSP00000215829	ENSP00000319169
ENSP00000215829	ENSP00000319341
ENSP00000215829	ENSP00000323302
ENSP00000215829	ENSP00000324122
ENSP00000215829	ENSP00000325448
ENSP00000215829	ENSP00000326261
ENSP00000215829	ENSP00000326806
ENSP00000215829	ENSP00000334148
ENSP00000215829	ENSP00000334538
ENSP00000215829	ENSP00000335321
ENSP00000215829	ENSP00000337405
ENSP00000215829	ENSP00000342374
ENSP00000215829	ENSP00000346986
ENSP00000215829	ENSP00000348010

ENSP00000215829	ENSP00000351137
ENSP00000215829	ENSP00000357631
ENSP00000215829	ENSP00000358716
ENSP00000215829	ENSP00000360532
ENSP00000215829	ENSP00000360876
ENSP00000215829	ENSP00000361577
ENSP00000215829	ENSP00000361699
ENSP00000215829	ENSP00000362110
ENSP00000215829	ENSP00000362936
ENSP00000215829	ENSP00000363313
ENSP00000215829	ENSP00000364289
ENSP00000215829	ENSP00000364813
ENSP00000215829	ENSP00000366557
ENSP00000215829	ENSP00000366607
ENSP00000215829	ENSP00000367030
ENSP00000215829	ENSP00000367439
ENSP00000215829	ENSP00000370083
ENSP00000215829	ENSP00000370119
ENSP00000215829	ENSP00000372883
ENSP00000215829	ENSP00000376758
ENSP00000215829	ENSP00000376973
ENSP00000215829	ENSP00000379607
ENSP00000215829	ENSP00000392270
ENSP00000215829	ENSP00000400591
ENSP00000215829	ENSP00000401371
ENSP00000215829	ENSP00000403345
ENSP00000215829	ENSP00000406280
ENSP00000215829	ENSP00000410758
ENSP00000215829	ENSP00000412566
ENSP00000215829	ENSP00000414006
ENSP00000215829	ENSP00000414634
ENSP00000215917	ENSP00000251038
ENSP00000215957	ENSP00000263246
ENSP00000215957	ENSP00000268668
ENSP00000215957	ENSP00000359345
ENSP00000215957	ENSP00000378326
ENSP00000215957	ENSP00000380921
ENSP00000215957	ENSP00000382064
ENSP00000215957	ENSP00000384792
ENSP00000215957	ENSP00000386520
ENSP00000215980	ENSP00000283006
ENSP00000215980	ENSP00000336868
ENSP00000216027	ENSP00000245157
ENSP00000216027	ENSP00000251038
ENSP00000216027	ENSP00000268057
ENSP00000216027	ENSP00000294053

ENSP00000216027	ENSP00000297185
ENSP00000216027	ENSP00000300107
ENSP00000216027	ENSP00000310623
ENSP00000216027	ENSP00000318115
ENSP00000216027	ENSP00000353701
ENSP00000216027	ENSP00000363205
ENSP00000216034	ENSP00000217244
ENSP00000216034	ENSP00000251038
ENSP00000216034	ENSP00000252487
ENSP00000216034	ENSP00000259750
ENSP00000216034	ENSP00000260257
ENSP00000216034	ENSP00000262030
ENSP00000216034	ENSP00000270538
ENSP00000216034	ENSP00000274680
ENSP00000216034	ENSP00000284320
ENSP00000216034	ENSP00000293288
ENSP00000216034	ENSP00000295030
ENSP00000216034	ENSP00000296255
ENSP00000216034	ENSP00000315693
ENSP00000216034	ENSP00000317331
ENSP00000216034	ENSP00000318115
ENSP00000216034	ENSP00000342381
ENSP00000216034	ENSP00000351214
ENSP00000216034	ENSP00000352219
ENSP00000216034	ENSP00000361686
ENSP00000216034	ENSP00000361993
ENSP00000216034	ENSP00000364188
ENSP00000216034	ENSP00000365766
ENSP00000216034	ENSP00000368523
ENSP00000216034	ENSP00000370607
ENSP00000216034	ENSP00000387262
ENSP00000216036	ENSP00000341551
ENSP00000216037	ENSP00000222307
ENSP00000216037	ENSP00000231487
ENSP00000216037	ENSP00000254908
ENSP00000216037	ENSP00000261884
ENSP00000216037	ENSP00000262133
ENSP00000216037	ENSP00000306245
ENSP00000216037	ENSP00000329662
ENSP00000216037	ENSP00000333298
ENSP00000216037	ENSP00000344504
ENSP00000216039	ENSP00000344668
ENSP00000216039	ENSP00000361993
ENSP00000216044	ENSP00000217964
ENSP00000216044	ENSP00000219473
ENSP00000216044	ENSP00000238647

ENSP00000216044	ENSP00000251871
ENSP00000216044	ENSP00000252137
ENSP00000216044	ENSP00000255764
ENSP00000216044	ENSP00000258648
ENSP00000216044	ENSP00000260363
ENSP00000216044	ENSP00000260753
ENSP00000216044	ENSP00000261758
ENSP00000216044	ENSP00000263121
ENSP00000216044	ENSP00000263205
ENSP00000216044	ENSP00000266126
ENSP00000216044	ENSP00000267064
ENSP00000216044	ENSP00000268668
ENSP00000216044	ENSP00000286317
ENSP00000216044	ENSP00000290663
ENSP00000216044	ENSP00000292035
ENSP00000216044	ENSP00000293777
ENSP00000216044	ENSP00000297347
ENSP00000216044	ENSP00000297689
ENSP00000216044	ENSP00000306100
ENSP00000216044	ENSP00000323720
ENSP00000216044	ENSP00000323858
ENSP00000216044	ENSP00000325612
ENSP00000216044	ENSP00000326128
ENSP00000216044	ENSP00000342181
ENSP00000216044	ENSP00000357047
ENSP00000216044	ENSP00000357907
ENSP00000216044	ENSP00000360532
ENSP00000216044	ENSP00000361467
ENSP00000216044	ENSP00000362948
ENSP00000216044	ENSP00000373272
ENSP00000216044	ENSP00000374455
ENSP00000216044	ENSP00000377262
ENSP00000216044	ENSP00000382064
ENSP00000216044	ENSP00000384391
ENSP00000216044	ENSP00000391774
ENSP00000216044	ENSP00000392617
ENSP00000216044	ENSP00000394869
ENSP00000216068	ENSP00000254579
ENSP00000216068	ENSP00000265104
ENSP00000216068	ENSP00000305503
ENSP00000216068	ENSP00000321029
ENSP00000216068	ENSP00000348965
ENSP00000216068	ENSP00000349016
ENSP00000216068	ENSP00000352400
ENSP00000216068	ENSP00000356989
ENSP00000216068	ENSP00000360473

ENSP00000216068	ENSP00000367841
ENSP00000216068	ENSP00000414402
ENSP00000216099	ENSP00000369816
ENSP00000216106	ENSP00000246337
ENSP00000216106	ENSP00000364398
ENSP00000216106	ENSP00000366221
ENSP00000216115	ENSP00000259075
ENSP00000216115	ENSP00000267953
ENSP00000216115	ENSP00000329623
ENSP00000216115	ENSP00000358022
ENSP00000216129	ENSP00000276692
ENSP00000216129	ENSP00000294008
ENSP00000216129	ENSP00000329466
ENSP00000216133	ENSP00000217244
ENSP00000216133	ENSP00000234396
ENSP00000216133	ENSP00000252108
ENSP00000216133	ENSP00000263736
ENSP00000216133	ENSP00000267064
ENSP00000216133	ENSP00000269197
ENSP00000216133	ENSP00000269385
ENSP00000216133	ENSP00000316173
ENSP00000216133	ENSP00000343535
ENSP00000216133	ENSP00000356448
ENSP00000216133	ENSP00000358862
ENSP00000216133	ENSP00000359321
ENSP00000216133	ENSP00000361287
ENSP00000216133	ENSP00000363787
ENSP00000216133	ENSP00000370151
ENSP00000216133	ENSP00000396439
ENSP00000216133	ENSP00000418734
ENSP00000216133	ENSP00000419494
ENSP00000216139	ENSP00000368686
ENSP00000216139	ENSP00000378326
ENSP00000216160	ENSP00000220751
ENSP00000216160	ENSP00000250615
ENSP00000216160	ENSP00000262039
ENSP00000216160	ENSP00000262177
ENSP00000216160	ENSP00000288422
ENSP00000216160	ENSP00000288840
ENSP00000216160	ENSP00000297185
ENSP00000216160	ENSP00000299424
ENSP00000216160	ENSP00000301364
ENSP00000216160	ENSP00000301838
ENSP00000216160	ENSP00000325958
ENSP00000216160	ENSP00000331310
ENSP00000216160	ENSP00000344668

ENSP00000216160	ENSP00000344683
ENSP00000216160	ENSP00000346839
ENSP00000216160	ENSP00000349205
ENSP00000216160	ENSP00000351141
ENSP00000216160	ENSP00000356530
ENSP00000216160	ENSP00000358335
ENSP00000216160	ENSP00000358997
ENSP00000216160	ENSP00000360268
ENSP00000216160	ENSP00000365439
ENSP00000216160	ENSP00000372023
ENSP00000216160	ENSP00000375986
ENSP00000216160	ENSP00000380150
ENSP00000216160	ENSP00000401445
ENSP00000216185	ENSP00000216455
ENSP00000216185	ENSP00000219169
ENSP00000216185	ENSP00000220959
ENSP00000216185	ENSP00000222008
ENSP00000216185	ENSP00000229416
ENSP00000216185	ENSP00000233893
ENSP00000216185	ENSP00000241052
ENSP00000216185	ENSP00000252622
ENSP00000216185	ENSP00000254250
ENSP00000216185	ENSP00000259667
ENSP00000216185	ENSP00000262030
ENSP00000216185	ENSP00000265462
ENSP00000216185	ENSP00000286428
ENSP00000216185	ENSP00000292180
ENSP00000216185	ENSP00000304908
ENSP00000216185	ENSP00000307900
ENSP00000216185	ENSP00000313921
ENSP00000216185	ENSP00000321259
ENSP00000216185	ENSP00000327070
ENSP00000216185	ENSP00000330343
ENSP00000216185	ENSP00000330836
ENSP00000216185	ENSP00000331897
ENSP00000216185	ENSP00000332513
ENSP00000216185	ENSP00000340278
ENSP00000216185	ENSP00000342026
ENSP00000216185	ENSP00000345023
ENSP00000216185	ENSP00000360286
ENSP00000216185	ENSP00000363135
ENSP00000216185	ENSP00000364212
ENSP00000216185	ENSP00000366620
ENSP00000216185	ENSP00000379933
ENSP00000216185	ENSP00000381698
ENSP00000216187	ENSP00000253413

ENSP00000216187	ENSP00000264065
ENSP00000216187	ENSP00000296255
ENSP00000216187	ENSP00000333946
ENSP00000216187	ENSP00000337053
ENSP00000216190	ENSP00000216554
ENSP00000216190	ENSP00000220062
ENSP00000216190	ENSP00000220849
ENSP00000216190	ENSP00000225688
ENSP00000216190	ENSP00000248706
ENSP00000216190	ENSP00000253108
ENSP00000216190	ENSP00000261868
ENSP00000216190	ENSP00000266671
ENSP00000216190	ENSP00000267396
ENSP00000216190	ENSP00000281172
ENSP00000216190	ENSP00000291582
ENSP00000216190	ENSP00000300249
ENSP00000216190	ENSP00000310040
ENSP00000216190	ENSP00000315659
ENSP00000216190	ENSP00000316845
ENSP00000216190	ENSP00000327315
ENSP00000216190	ENSP00000329002
ENSP00000216190	ENSP00000329466
ENSP00000216190	ENSP00000329933
ENSP00000216190	ENSP00000339136
ENSP00000216190	ENSP00000339299
ENSP00000216190	ENSP00000348215
ENSP00000216190	ENSP00000348442
ENSP00000216190	ENSP00000354125
ENSP00000216190	ENSP00000355296
ENSP00000216190	ENSP00000358140
ENSP00000216190	ENSP00000360020
ENSP00000216190	ENSP00000361009
ENSP00000216190	ENSP00000362688
ENSP00000216190	ENSP00000364749
ENSP00000216190	ENSP00000369716
ENSP00000216190	ENSP00000370258
ENSP00000216190	ENSP00000374213
ENSP00000216190	ENSP00000377747
ENSP00000216190	ENSP00000379607
ENSP00000216190	ENSP00000380450
ENSP00000216190	ENSP00000381891
ENSP00000216190	ENSP00000384675
ENSP00000216190	ENSP00000416892
ENSP00000216194	ENSP00000251038
ENSP00000216194	ENSP00000264220
ENSP00000216194	ENSP00000286621

ENSP00000216194	ENSP00000298532
ENSP00000216194	ENSP00000304169
ENSP00000216194	ENSP00000306817
ENSP00000216194	ENSP00000314440
ENSP00000216194	ENSP00000335357
ENSP00000216194	ENSP00000352928
ENSP00000216194	ENSP00000354125
ENSP00000216194	ENSP00000355518
ENSP00000216194	ENSP00000360532
ENSP00000216194	ENSP00000362717
ENSP00000216194	ENSP00000367615
ENSP00000216194	ENSP00000379568
ENSP00000216194	ENSP00000382595
ENSP00000216194	ENSP00000382779
ENSP00000216194	ENSP00000417229
ENSP00000216211	ENSP00000264234
ENSP00000216218	ENSP00000229595
ENSP00000216218	ENSP00000237014
ENSP00000216218	ENSP00000262844
ENSP00000216218	ENSP00000263246
ENSP00000216218	ENSP00000264029
ENSP00000216218	ENSP00000284981
ENSP00000216218	ENSP00000294053
ENSP00000216218	ENSP00000295121
ENSP00000216218	ENSP00000295897
ENSP00000216218	ENSP00000296084
ENSP00000216218	ENSP00000301329
ENSP00000216218	ENSP00000307900
ENSP00000216218	ENSP00000312664
ENSP00000216218	ENSP00000326813
ENSP00000216218	ENSP00000347548
ENSP00000216218	ENSP00000347948
ENSP00000216218	ENSP00000350785
ENSP00000216218	ENSP00000352706
ENSP00000216218	ENSP00000357341
ENSP00000216218	ENSP00000358404
ENSP00000216218	ENSP00000360916
ENSP00000216218	ENSP00000368605
ENSP00000216218	ENSP00000401566
ENSP00000216223	ENSP00000226730
ENSP00000216223	ENSP00000296545
ENSP00000216223	ENSP00000331201
ENSP00000216223	ENSP00000341208
ENSP00000216223	ENSP00000345530
ENSP00000216223	ENSP00000354125
ENSP00000216223	ENSP00000358043

ENSP00000216223	ENSP00000364898
ENSP00000216223	ENSP00000366936
ENSP00000216223	ENSP00000369293
ENSP00000216223	ENSP00000369312
ENSP00000216223	ENSP00000409346
ENSP00000216237	ENSP00000251900
ENSP00000216237	ENSP00000262133
ENSP00000216237	ENSP00000262238
ENSP00000216237	ENSP00000267163
ENSP00000216237	ENSP00000325634
ENSP00000216237	ENSP00000341805
ENSP00000216237	ENSP00000350767
ENSP00000216237	ENSP00000351490
ENSP00000216237	ENSP00000351947
ENSP00000216237	ENSP00000358862
ENSP00000216237	ENSP00000362166
ENSP00000216237	ENSP00000363787
ENSP00000216237	ENSP00000372699
ENSP00000216237	ENSP00000396271
ENSP00000216237	ENSP00000396439
ENSP00000216237	ENSP00000400572
ENSP00000216237	ENSP00000412190
ENSP00000216237	ENSP00000419494
ENSP00000216252	ENSP00000221494
ENSP00000216252	ENSP00000222969
ENSP00000216252	ENSP00000227524
ENSP00000216252	ENSP00000251038
ENSP00000216252	ENSP00000252115
ENSP00000216252	ENSP00000262300
ENSP00000216252	ENSP00000268482
ENSP00000216252	ENSP00000271628
ENSP00000216252	ENSP00000301764
ENSP00000216252	ENSP00000304370
ENSP00000216252	ENSP00000305790
ENSP00000216252	ENSP00000310448
ENSP00000216252	ENSP00000318861
ENSP00000216252	ENSP00000324122
ENSP00000216252	ENSP00000331817
ENSP00000216252	ENSP00000332103
ENSP00000216252	ENSP00000334148
ENSP00000216252	ENSP00000335321
ENSP00000216252	ENSP00000339090
ENSP00000216252	ENSP00000356520
ENSP00000216252	ENSP00000366557
ENSP00000216252	ENSP00000402084
ENSP00000216252	ENSP00000412566

ENSP00000216254	ENSP00000216484
ENSP00000216254	ENSP00000219551
ENSP00000216254	ENSP00000222673
ENSP00000216254	ENSP00000228318
ENSP00000216254	ENSP00000234590
ENSP00000216254	ENSP00000251038
ENSP00000216254	ENSP00000252699
ENSP00000216254	ENSP00000253792
ENSP00000216254	ENSP00000254035
ENSP00000216254	ENSP00000256379
ENSP00000216254	ENSP00000262554
ENSP00000216254	ENSP00000264029
ENSP00000216254	ENSP00000264161
ENSP00000216254	ENSP00000268668
ENSP00000216254	ENSP00000277865
ENSP00000216254	ENSP00000282541
ENSP00000216254	ENSP00000283943
ENSP00000216254	ENSP00000290299
ENSP00000216254	ENSP00000292475
ENSP00000216254	ENSP00000294053
ENSP00000216254	ENSP00000296684
ENSP00000216254	ENSP00000297598
ENSP00000216254	ENSP00000300151
ENSP00000216254	ENSP00000300648
ENSP00000216254	ENSP00000301587
ENSP00000216254	ENSP00000301740
ENSP00000216254	ENSP00000307786
ENSP00000216254	ENSP00000308610
ENSP00000216254	ENSP00000317159
ENSP00000216254	ENSP00000327070
ENSP00000216254	ENSP00000331897
ENSP00000216254	ENSP00000333667
ENSP00000216254	ENSP00000336927
ENSP00000216254	ENSP00000337127
ENSP00000216254	ENSP00000339826
ENSP00000216254	ENSP00000340019
ENSP00000216254	ENSP00000341838
ENSP00000216254	ENSP00000342056
ENSP00000216254	ENSP00000358737
ENSP00000216254	ENSP00000359454
ENSP00000216254	ENSP00000361287
ENSP00000216254	ENSP00000366482
ENSP00000216254	ENSP00000367923
ENSP00000216254	ENSP00000368565
ENSP00000216254	ENSP00000382177
ENSP00000216254	ENSP00000382269

ENSP00000216254	ENSP00000387262
ENSP00000216254	ENSP00000389649
ENSP00000216254	ENSP00000397598
ENSP00000216254	ENSP00000409275
ENSP00000216254	ENSP00000420588
ENSP00000216259	ENSP00000266095
ENSP00000216259	ENSP00000296255
ENSP00000216259	ENSP00000300648
ENSP00000216259	ENSP00000303279
ENSP00000216259	ENSP00000321239
ENSP00000216259	ENSP00000324792
ENSP00000216259	ENSP00000330343
ENSP00000216259	ENSP00000341679
ENSP00000216259	ENSP00000344456
ENSP00000216259	ENSP00000346155
ENSP00000216259	ENSP00000364794
ENSP00000216274	ENSP00000216392
ENSP00000216274	ENSP00000222005
ENSP00000216274	ENSP00000227758
ENSP00000216274	ENSP00000248244
ENSP00000216274	ENSP00000259808
ENSP00000216274	ENSP00000301838
ENSP00000216274	ENSP00000305958
ENSP00000216274	ENSP00000308351
ENSP00000216274	ENSP00000321503
ENSP00000216274	ENSP00000332208
ENSP00000216274	ENSP00000360215
ENSP00000216277	ENSP00000216727
ENSP00000216277	ENSP00000230050
ENSP00000216277	ENSP00000230859
ENSP00000216277	ENSP00000235090
ENSP00000216277	ENSP00000238112
ENSP00000216277	ENSP00000243563
ENSP00000216277	ENSP00000245838
ENSP00000216277	ENSP00000245934
ENSP00000216277	ENSP00000246548
ENSP00000216277	ENSP00000251038
ENSP00000216277	ENSP00000253048
ENSP00000216277	ENSP00000257177
ENSP00000216277	ENSP00000261211
ENSP00000216277	ENSP00000262367
ENSP00000216277	ENSP00000262577
ENSP00000216277	ENSP00000263202
ENSP00000216277	ENSP00000264335
ENSP00000216277	ENSP00000264951
ENSP00000216277	ENSP00000265073

ENSP00000216277	ENSP00000274606
ENSP00000216277	ENSP00000277541
ENSP00000216277	ENSP00000282007
ENSP00000216277	ENSP00000290158
ENSP00000216277	ENSP00000290810
ENSP00000216277	ENSP00000291386
ENSP00000216277	ENSP00000292476
ENSP00000216277	ENSP00000295025
ENSP00000216277	ENSP00000296161
ENSP00000216277	ENSP00000296490
ENSP00000216277	ENSP00000296783
ENSP00000216277	ENSP00000296930
ENSP00000216277	ENSP00000298281
ENSP00000216277	ENSP00000299767
ENSP00000216277	ENSP00000300291
ENSP00000216277	ENSP00000312735
ENSP00000216277	ENSP00000314348
ENSP00000216277	ENSP00000315791
ENSP00000216277	ENSP00000317872
ENSP00000216277	ENSP00000321711
ENSP00000216277	ENSP00000322885
ENSP00000216277	ENSP00000325377
ENSP00000216277	ENSP00000326699
ENSP00000216277	ENSP00000328169
ENSP00000216277	ENSP00000332444
ENSP00000216277	ENSP00000334061
ENSP00000216277	ENSP00000336752
ENSP00000216277	ENSP00000338413
ENSP00000216277	ENSP00000339245
ENSP00000216277	ENSP00000339353
ENSP00000216277	ENSP00000348010
ENSP00000216277	ENSP00000348565
ENSP00000216277	ENSP00000354340
ENSP00000216277	ENSP00000359531
ENSP00000216277	ENSP00000361076
ENSP00000216277	ENSP00000361949
ENSP00000216277	ENSP00000366135
ENSP00000216277	ENSP00000367406
ENSP00000216277	ENSP00000368017
ENSP00000216277	ENSP00000386134
ENSP00000216277	ENSP00000391774
ENSP00000216277	ENSP00000401435
ENSP00000216277	ENSP00000414187
ENSP00000216286	ENSP00000252034
ENSP00000216286	ENSP00000363827
ENSP00000216286	ENSP00000384169

ENSP00000216294	ENSP00000267163
ENSP00000216294	ENSP00000283921
ENSP00000216294	ENSP00000298532
ENSP00000216294	ENSP00000319597
ENSP00000216294	ENSP00000343129
ENSP00000216297	ENSP00000217244
ENSP00000216297	ENSP00000219252
ENSP00000216297	ENSP00000219473
ENSP00000216297	ENSP00000220659
ENSP00000216297	ENSP00000220669
ENSP00000216297	ENSP00000221265
ENSP00000216297	ENSP00000224140
ENSP00000216297	ENSP00000225504
ENSP00000216297	ENSP00000225688
ENSP00000216297	ENSP00000225916
ENSP00000216297	ENSP00000229595
ENSP00000216297	ENSP00000238616
ENSP00000216297	ENSP00000238789
ENSP00000216297	ENSP00000247219
ENSP00000216297	ENSP00000251127
ENSP00000216297	ENSP00000252445
ENSP00000216297	ENSP00000253107
ENSP00000216297	ENSP00000254719
ENSP00000216297	ENSP00000254900
ENSP00000216297	ENSP00000255078
ENSP00000216297	ENSP00000256398
ENSP00000216297	ENSP00000257575
ENSP00000216297	ENSP00000261424
ENSP00000216297	ENSP00000261951
ENSP00000216297	ENSP00000262315
ENSP00000216297	ENSP00000262519
ENSP00000216297	ENSP00000262544
ENSP00000216297	ENSP00000262608
ENSP00000216297	ENSP00000262794
ENSP00000216297	ENSP00000262803
ENSP00000216297	ENSP00000263201
ENSP00000216297	ENSP00000263208
ENSP00000216297	ENSP00000263274
ENSP00000216297	ENSP00000263736
ENSP00000216297	ENSP00000263795
ENSP00000216297	ENSP00000263857
ENSP00000216297	ENSP00000265304
ENSP00000216297	ENSP00000265990
ENSP00000216297	ENSP00000267197
ENSP00000216297	ENSP00000269127
ENSP00000216297	ENSP00000272444

ENSP00000216297	ENSP00000275053
ENSP00000216297	ENSP00000276533
ENSP00000216297	ENSP00000278412
ENSP00000216297	ENSP00000280614
ENSP00000216297	ENSP00000283351
ENSP00000216297	ENSP00000283875
ENSP00000216297	ENSP00000287239
ENSP00000216297	ENSP00000290649
ENSP00000216297	ENSP00000294339
ENSP00000216297	ENSP00000296273
ENSP00000216297	ENSP00000296328
ENSP00000216297	ENSP00000298852
ENSP00000216297	ENSP00000299601
ENSP00000216297	ENSP00000302640
ENSP00000216297	ENSP00000303511
ENSP00000216297	ENSP00000304051
ENSP00000216297	ENSP00000305958
ENSP00000216297	ENSP00000306124
ENSP00000216297	ENSP00000307208
ENSP00000216297	ENSP00000309142
ENSP00000216297	ENSP00000310966
ENSP00000216297	ENSP00000312625
ENSP00000216297	ENSP00000313420
ENSP00000216297	ENSP00000314949
ENSP00000216297	ENSP00000319104
ENSP00000216297	ENSP00000319799
ENSP00000216297	ENSP00000322570
ENSP00000216297	ENSP00000326477
ENSP00000216297	ENSP00000329029
ENSP00000216297	ENSP00000329662
ENSP00000216297	ENSP00000329933
ENSP00000216297	ENSP00000330074
ENSP00000216297	ENSP00000331211
ENSP00000216297	ENSP00000334538
ENSP00000216297	ENSP00000336856
ENSP00000216297	ENSP00000342434
ENSP00000216297	ENSP00000343190
ENSP00000216297	ENSP00000343741
ENSP00000216297	ENSP00000344504
ENSP00000216297	ENSP00000347168
ENSP00000216297	ENSP00000348168
ENSP00000216297	ENSP00000349213
ENSP00000216297	ENSP00000349393
ENSP00000216297	ENSP00000350708
ENSP00000216297	ENSP00000350767
ENSP00000216297	ENSP00000351250

ENSP00000216297	ENSP00000351524
ENSP00000216297	ENSP00000351697
ENSP00000216297	ENSP00000351947
ENSP00000216297	ENSP00000353622
ENSP00000216297	ENSP00000353698
ENSP00000216297	ENSP00000353793
ENSP00000216297	ENSP00000354522
ENSP00000216297	ENSP00000354977
ENSP00000216297	ENSP00000355013
ENSP00000216297	ENSP00000355759
ENSP00000216297	ENSP00000356290
ENSP00000216297	ENSP00000356405
ENSP00000216297	ENSP00000357602
ENSP00000216297	ENSP00000357965
ENSP00000216297	ENSP00000358147
ENSP00000216297	ENSP00000358423
ENSP00000216297	ENSP00000358497
ENSP00000216297	ENSP00000359531
ENSP00000216297	ENSP00000359552
ENSP00000216297	ENSP00000360515
ENSP00000216297	ENSP00000360532
ENSP00000216297	ENSP00000360817
ENSP00000216297	ENSP00000361465
ENSP00000216297	ENSP00000361642
ENSP00000216297	ENSP00000362900
ENSP00000216297	ENSP00000364289
ENSP00000216297	ENSP00000365025
ENSP00000216297	ENSP00000366395
ENSP00000216297	ENSP00000366974
ENSP00000216297	ENSP00000367029
ENSP00000216297	ENSP00000367034
ENSP00000216297	ENSP00000367787
ENSP00000216297	ENSP00000368349
ENSP00000216297	ENSP00000369411
ENSP00000216297	ENSP00000369716
ENSP00000216297	ENSP00000370151
ENSP00000216297	ENSP00000372023
ENSP00000216297	ENSP00000374280
ENSP00000216297	ENSP00000377747
ENSP00000216297	ENSP00000378350
ENSP00000216297	ENSP00000379372
ENSP00000216297	ENSP00000379607
ENSP00000216297	ENSP00000380414
ENSP00000216297	ENSP00000384597
ENSP00000216297	ENSP00000386239
ENSP00000216297	ENSP00000386759

ENSP00000216297	ENSP00000392028
ENSP00000216297	ENSP00000396704
ENSP00000216297	ENSP00000420588
ENSP00000216330	ENSP00000217244
ENSP00000216330	ENSP00000222247
ENSP00000216330	ENSP00000222567
ENSP00000216330	ENSP00000227322
ENSP00000216330	ENSP00000227474
ENSP00000216330	ENSP00000233893
ENSP00000216330	ENSP00000246802
ENSP00000216330	ENSP00000247970
ENSP00000216330	ENSP00000248566
ENSP00000216330	ENSP00000251038
ENSP00000216330	ENSP00000254193
ENSP00000216330	ENSP00000254605
ENSP00000216330	ENSP00000254940
ENSP00000216330	ENSP00000257934
ENSP00000216330	ENSP00000261868
ENSP00000216330	ENSP00000262238
ENSP00000216330	ENSP00000263657
ENSP00000216330	ENSP00000264995
ENSP00000216330	ENSP00000265073
ENSP00000216330	ENSP00000265963
ENSP00000216330	ENSP00000272065
ENSP00000216330	ENSP00000296802
ENSP00000216330	ENSP00000302801
ENSP00000216330	ENSP00000305682
ENSP00000216330	ENSP00000317992
ENSP00000216330	ENSP00000322396
ENSP00000216330	ENSP00000330343
ENSP00000216330	ENSP00000338862
ENSP00000216330	ENSP00000351141
ENSP00000216330	ENSP00000354040
ENSP00000216330	ENSP00000354558
ENSP00000216330	ENSP00000356548
ENSP00000216330	ENSP00000356989
ENSP00000216330	ENSP00000358812
ENSP00000216330	ENSP00000359345
ENSP00000216330	ENSP00000359688
ENSP00000216330	ENSP00000360412
ENSP00000216330	ENSP00000363384
ENSP00000216330	ENSP00000365025
ENSP00000216330	ENSP00000368438
ENSP00000216330	ENSP00000368856
ENSP00000216330	ENSP00000368927
ENSP00000216330	ENSP00000369396

ENSP00000216330	ENSP00000370105
ENSP00000216330	ENSP00000371888
ENSP00000216330	ENSP00000373509
ENSP00000216330	ENSP00000374399
ENSP00000216330	ENSP00000379888
ENSP00000216330	ENSP00000380982
ENSP00000216330	ENSP00000381657
ENSP00000216330	ENSP00000399511
ENSP00000216330	ENSP00000402338
ENSP00000216330	ENSP00000417281
ENSP00000216330	ENSP00000420714
ENSP00000216367	ENSP00000230859
ENSP00000216367	ENSP00000233146
ENSP00000216367	ENSP00000234420
ENSP00000216367	ENSP00000246802
ENSP00000216367	ENSP00000253462
ENSP00000216367	ENSP00000254719
ENSP00000216367	ENSP00000257177
ENSP00000216367	ENSP00000260810
ENSP00000216367	ENSP00000261211
ENSP00000216367	ENSP00000262315
ENSP00000216367	ENSP00000262460
ENSP00000216367	ENSP00000263201
ENSP00000216367	ENSP00000264169
ENSP00000216367	ENSP00000265081
ENSP00000216367	ENSP00000265140
ENSP00000216367	ENSP00000269159
ENSP00000216367	ENSP00000278412
ENSP00000216367	ENSP00000288976
ENSP00000216367	ENSP00000293406
ENSP00000216367	ENSP00000296783
ENSP00000216367	ENSP00000297990
ENSP00000216367	ENSP00000307850
ENSP00000216367	ENSP00000311368
ENSP00000216367	ENSP00000314348
ENSP00000216367	ENSP00000316879
ENSP00000216367	ENSP00000322570
ENSP00000216367	ENSP00000335357
ENSP00000216367	ENSP00000338573
ENSP00000216367	ENSP00000338788
ENSP00000216367	ENSP00000340281
ENSP00000216367	ENSP00000345096
ENSP00000216367	ENSP00000351697
ENSP00000216367	ENSP00000354040
ENSP00000216367	ENSP00000354125
ENSP00000216367	ENSP00000354723

ENSP00000216367	ENSP00000354916
ENSP00000216367	ENSP00000358563
ENSP00000216367	ENSP00000360031
ENSP00000216367	ENSP00000360621
ENSP00000216367	ENSP00000368438
ENSP00000216367	ENSP00000369757
ENSP00000216367	ENSP00000370151
ENSP00000216367	ENSP00000380460
ENSP00000216367	ENSP00000380504
ENSP00000216367	ENSP00000401018
ENSP00000216367	ENSP00000417229
ENSP00000216367	ENSP00000417281
ENSP00000216392	ENSP00000222286
ENSP00000216392	ENSP00000234040
ENSP00000216392	ENSP00000238994
ENSP00000216392	ENSP00000254035
ENSP00000216392	ENSP00000263056
ENSP00000216392	ENSP00000264233
ENSP00000216392	ENSP00000267484
ENSP00000216392	ENSP00000268124
ENSP00000216392	ENSP00000282541
ENSP00000216392	ENSP00000284601
ENSP00000216392	ENSP00000314949
ENSP00000216392	ENSP00000321636
ENSP00000216392	ENSP00000329466
ENSP00000216392	ENSP00000330484
ENSP00000216392	ENSP00000331897
ENSP00000216392	ENSP00000336927
ENSP00000216392	ENSP00000337127
ENSP00000216392	ENSP00000337838
ENSP00000216392	ENSP00000342032
ENSP00000216392	ENSP00000354558
ENSP00000216392	ENSP00000359096
ENSP00000216392	ENSP00000359100
ENSP00000216392	ENSP00000359818
ENSP00000216392	ENSP00000362413
ENSP00000216392	ENSP00000363873
ENSP00000216392	ENSP00000372316
ENSP00000216392	ENSP00000381863
ENSP00000216392	ENSP00000393832
ENSP00000216410	ENSP00000216455
ENSP00000216410	ENSP00000240922
ENSP00000216410	ENSP00000244204
ENSP00000216410	ENSP00000253108
ENSP00000216410	ENSP00000264995
ENSP00000216410	ENSP00000282541

ENSP00000216410	ENSP00000300146
ENSP00000216410	ENSP00000302160
ENSP00000216410	ENSP00000360621
ENSP00000216410	ENSP00000379678
ENSP00000216410	ENSP00000379888
ENSP00000216410	ENSP00000387170
ENSP00000216420	ENSP00000288774
ENSP00000216420	ENSP00000360116
ENSP00000216442	ENSP00000231487
ENSP00000216442	ENSP00000234396
ENSP00000216442	ENSP00000249289
ENSP00000216442	ENSP00000251038
ENSP00000216442	ENSP00000251076
ENSP00000216442	ENSP00000253413
ENSP00000216442	ENSP00000272238
ENSP00000216442	ENSP00000273398
ENSP00000216442	ENSP00000290949
ENSP00000216442	ENSP00000299138
ENSP00000216442	ENSP00000332247
ENSP00000216442	ENSP00000352522
ENSP00000216442	ENSP00000362085
ENSP00000216442	ENSP00000363162
ENSP00000216452	ENSP00000369820
ENSP00000216455	ENSP00000216832
ENSP00000216455	ENSP00000217244
ENSP00000216455	ENSP00000219313
ENSP00000216455	ENSP00000219548
ENSP00000216455	ENSP00000222308
ENSP00000216455	ENSP00000232905
ENSP00000216455	ENSP00000233084
ENSP00000216455	ENSP00000234160
ENSP00000216455	ENSP00000239938
ENSP00000216455	ENSP00000244741
ENSP00000216455	ENSP00000251038
ENSP00000216455	ENSP00000251303
ENSP00000216455	ENSP00000252329
ENSP00000216455	ENSP00000253003
ENSP00000216455	ENSP00000253693
ENSP00000216455	ENSP00000259457
ENSP00000216455	ENSP00000259469
ENSP00000216455	ENSP00000259569
ENSP00000216455	ENSP00000261601
ENSP00000216455	ENSP00000261712
ENSP00000216455	ENSP00000262030
ENSP00000216455	ENSP00000262193
ENSP00000216455	ENSP00000263413

ENSP00000216455	ENSP00000264639
ENSP00000216455	ENSP00000265148
ENSP00000216455	ENSP00000266735
ENSP00000216455	ENSP00000268058
ENSP00000216455	ENSP00000277865
ENSP00000216455	ENSP00000280346
ENSP00000216455	ENSP00000282050
ENSP00000216455	ENSP00000282728
ENSP00000216455	ENSP00000290541
ENSP00000216455	ENSP00000293362
ENSP00000216455	ENSP00000295901
ENSP00000216455	ENSP00000297185
ENSP00000216455	ENSP00000298852
ENSP00000216455	ENSP00000299767
ENSP00000216455	ENSP00000307143
ENSP00000216455	ENSP00000309474
ENSP00000216455	ENSP00000310129
ENSP00000216455	ENSP00000311083
ENSP00000216455	ENSP00000311505
ENSP00000216455	ENSP00000314129
ENSP00000216455	ENSP00000318195
ENSP00000216455	ENSP00000325448
ENSP00000216455	ENSP00000325919
ENSP00000216455	ENSP00000328940
ENSP00000216455	ENSP00000329915
ENSP00000216455	ENSP00000333122
ENSP00000216455	ENSP00000338629
ENSP00000216455	ENSP00000340019
ENSP00000216455	ENSP00000341243
ENSP00000216455	ENSP00000343313
ENSP00000216455	ENSP00000343535
ENSP00000216455	ENSP00000346437
ENSP00000216455	ENSP00000346453
ENSP00000216455	ENSP00000347948
ENSP00000216455	ENSP00000348442
ENSP00000216455	ENSP00000350708
ENSP00000216455	ENSP00000354673
ENSP00000216455	ENSP00000355922
ENSP00000216455	ENSP00000358404
ENSP00000216455	ENSP00000359206
ENSP00000216455	ENSP00000359531
ENSP00000216455	ENSP00000360060
ENSP00000216455	ENSP00000362334
ENSP00000216455	ENSP00000362413
ENSP00000216455	ENSP00000365175
ENSP00000216455	ENSP00000370222

ENSP00000216455	ENSP00000370376
ENSP00000216455	ENSP00000370611
ENSP00000216455	ENSP00000370741
ENSP00000216455	ENSP00000371546
ENSP00000216455	ENSP00000373918
ENSP00000216455	ENSP00000379607
ENSP00000216455	ENSP00000379933
ENSP00000216455	ENSP00000380349
ENSP00000216455	ENSP00000381036
ENSP00000216455	ENSP00000384211
ENSP00000216455	ENSP00000387477
ENSP00000216455	ENSP00000396937
ENSP00000216455	ENSP00000412566
ENSP00000216455	ENSP00000416583
ENSP00000216455	ENSP00000417281
ENSP00000216465	ENSP00000216951
ENSP00000216465	ENSP00000284719
ENSP00000216465	ENSP00000339435
ENSP00000216465	ENSP00000353622
ENSP00000216465	ENSP00000356257
ENSP00000216465	ENSP00000414670
ENSP00000216479	ENSP00000216774
ENSP00000216479	ENSP00000219066
ENSP00000216479	ENSP00000221232
ENSP00000216479	ENSP00000222005
ENSP00000216479	ENSP00000227322
ENSP00000216479	ENSP00000232978
ENSP00000216479	ENSP00000233623
ENSP00000216479	ENSP00000236959
ENSP00000216479	ENSP00000244763
ENSP00000216479	ENSP00000246062
ENSP00000216479	ENSP00000251038
ENSP00000216479	ENSP00000253792
ENSP00000216479	ENSP00000256854
ENSP00000216479	ENSP00000260956
ENSP00000216479	ENSP00000260970
ENSP00000216479	ENSP00000262030
ENSP00000216479	ENSP00000262544
ENSP00000216479	ENSP00000264161
ENSP00000216479	ENSP00000265351
ENSP00000216479	ENSP00000267884
ENSP00000216479	ENSP00000282999
ENSP00000216479	ENSP00000283943
ENSP00000216479	ENSP00000284719
ENSP00000216479	ENSP00000295702
ENSP00000216479	ENSP00000296255

ENSP00000216479	ENSP00000296273
ENSP00000216479	ENSP00000296509
ENSP00000216479	ENSP00000298852
ENSP00000216479	ENSP00000299767
ENSP00000216479	ENSP00000300648
ENSP00000216479	ENSP00000302478
ENSP00000216479	ENSP00000305958
ENSP00000216479	ENSP00000309474
ENSP00000216479	ENSP00000310129
ENSP00000216479	ENSP00000312066
ENSP00000216479	ENSP00000317331
ENSP00000216479	ENSP00000317334
ENSP00000216479	ENSP00000332887
ENSP00000216479	ENSP00000340019
ENSP00000216479	ENSP00000341213
ENSP00000216479	ENSP00000342181
ENSP00000216479	ENSP00000346791
ENSP00000216479	ENSP00000349142
ENSP00000216479	ENSP00000354876
ENSP00000216479	ENSP00000355890
ENSP00000216479	ENSP00000361949
ENSP00000216479	ENSP00000363559
ENSP00000216479	ENSP00000367208
ENSP00000216479	ENSP00000367923
ENSP00000216479	ENSP00000373918
ENSP00000216479	ENSP00000378890
ENSP00000216479	ENSP00000381003
ENSP00000216479	ENSP00000382269
ENSP00000216479	ENSP00000386918
ENSP00000216479	ENSP00000391944
ENSP00000216479	ENSP00000396452
ENSP00000216484	ENSP00000217901
ENSP00000216484	ENSP00000219368
ENSP00000216484	ENSP00000228318
ENSP00000216484	ENSP00000232014
ENSP00000216484	ENSP00000233616
ENSP00000216484	ENSP00000245222
ENSP00000216484	ENSP00000247225
ENSP00000216484	ENSP00000258052
ENSP00000216484	ENSP00000262554
ENSP00000216484	ENSP00000264335
ENSP00000216484	ENSP00000265351
ENSP00000216484	ENSP00000302790
ENSP00000216484	ENSP00000326869
ENSP00000216484	ENSP00000336712
ENSP00000216484	ENSP00000346453

ENSP00000216484	ENSP00000355890
ENSP00000216484	ENSP00000359000
ENSP00000216484	ENSP00000361287
ENSP00000216484	ENSP00000362298
ENSP00000216487	ENSP00000222008
ENSP00000216487	ENSP00000244007
ENSP00000216487	ENSP00000300574
ENSP00000216489	ENSP00000262177
ENSP00000216492	ENSP00000220478
ENSP00000216492	ENSP00000317714
ENSP00000216492	ENSP00000340914
ENSP00000216492	ENSP00000368244
ENSP00000216500	ENSP00000216554
ENSP00000216500	ENSP00000256383
ENSP00000216500	ENSP00000261875
ENSP00000216500	ENSP00000266126
ENSP00000216500	ENSP00000273783
ENSP00000216500	ENSP00000306894
ENSP00000216500	ENSP00000315925
ENSP00000216500	ENSP00000330408
ENSP00000216500	ENSP00000353575
ENSP00000216500	ENSP00000355308
ENSP00000216500	ENSP00000359067
ENSP00000216500	ENSP00000361057
ENSP00000216500	ENSP00000364119
ENSP00000216500	ENSP00000370607
ENSP00000216500	ENSP00000394869
ENSP00000216513	ENSP00000297261
ENSP00000216513	ENSP00000347294
ENSP00000216554	ENSP00000217244
ENSP00000216554	ENSP00000219313
ENSP00000216554	ENSP00000219439
ENSP00000216554	ENSP00000220659
ENSP00000216554	ENSP00000232905
ENSP00000216554	ENSP00000246337
ENSP00000216554	ENSP00000253108
ENSP00000216554	ENSP00000256383
ENSP00000216554	ENSP00000261708
ENSP00000216554	ENSP00000261868
ENSP00000216554	ENSP00000263629
ENSP00000216554	ENSP00000263791
ENSP00000216554	ENSP00000264705
ENSP00000216554	ENSP00000265245
ENSP00000216554	ENSP00000266126
ENSP00000216554	ENSP00000270625
ENSP00000216554	ENSP00000273783

ENSP00000216554	ENSP00000280557
ENSP00000216554	ENSP00000284292
ENSP00000216554	ENSP00000289371
ENSP00000216554	ENSP00000296577
ENSP00000216554	ENSP00000302896
ENSP00000216554	ENSP00000307889
ENSP00000216554	ENSP00000309334
ENSP00000216554	ENSP00000310040
ENSP00000216554	ENSP00000312042
ENSP00000216554	ENSP00000316879
ENSP00000216554	ENSP00000318646
ENSP00000216554	ENSP00000326806
ENSP00000216554	ENSP00000329029
ENSP00000216554	ENSP00000330343
ENSP00000216554	ENSP00000330408
ENSP00000216554	ENSP00000336702
ENSP00000216554	ENSP00000339095
ENSP00000216554	ENSP00000340281
ENSP00000216554	ENSP00000346067
ENSP00000216554	ENSP00000346725
ENSP00000216554	ENSP00000350512
ENSP00000216554	ENSP00000352928
ENSP00000216554	ENSP00000353741
ENSP00000216554	ENSP00000354040
ENSP00000216554	ENSP00000354125
ENSP00000216554	ENSP00000356146
ENSP00000216554	ENSP00000358140
ENSP00000216554	ENSP00000359531
ENSP00000216554	ENSP00000362688
ENSP00000216554	ENSP00000364119
ENSP00000216554	ENSP00000365025
ENSP00000216554	ENSP00000365806
ENSP00000216554	ENSP00000368927
ENSP00000216554	ENSP00000369757
ENSP00000216554	ENSP00000370258
ENSP00000216554	ENSP00000373090
ENSP00000216554	ENSP00000391723
ENSP00000216554	ENSP00000394290
ENSP00000216554	ENSP00000403576
ENSP00000216554	ENSP00000409074
ENSP00000216554	ENSP00000409367
ENSP00000216554	ENSP00000411162
ENSP00000216554	ENSP00000417229
ENSP00000216727	ENSP00000229922
ENSP00000216727	ENSP00000230859
ENSP00000216727	ENSP00000240185

ENSP00000216727	ENSP00000250003
ENSP00000216727	ENSP00000261531
ENSP00000216727	ENSP00000263265
ENSP00000216727	ENSP00000265271
ENSP00000216727	ENSP00000272203
ENSP00000216727	ENSP00000296930
ENSP00000216727	ENSP00000302768
ENSP00000216727	ENSP00000302886
ENSP00000216727	ENSP00000310649
ENSP00000216727	ENSP00000316879
ENSP00000216727	ENSP00000318195
ENSP00000216727	ENSP00000318318
ENSP00000216727	ENSP00000321711
ENSP00000216727	ENSP00000325690
ENSP00000216727	ENSP00000331817
ENSP00000216727	ENSP00000331879
ENSP00000216727	ENSP00000332444
ENSP00000216727	ENSP00000336712
ENSP00000216727	ENSP00000336752
ENSP00000216727	ENSP00000340281
ENSP00000216727	ENSP00000360922
ENSP00000216727	ENSP00000361949
ENSP00000216727	ENSP00000362820
ENSP00000216727	ENSP00000365877
ENSP00000216727	ENSP00000366135
ENSP00000216727	ENSP00000368017
ENSP00000216727	ENSP00000368824
ENSP00000216727	ENSP00000379607
ENSP00000216727	ENSP00000394400
ENSP00000216756	ENSP00000323099
ENSP00000216756	ENSP00000343741
ENSP00000216774	ENSP00000223641
ENSP00000216774	ENSP00000239944
ENSP00000216774	ENSP00000248673
ENSP00000216774	ENSP00000253099
ENSP00000216774	ENSP00000259469
ENSP00000216774	ENSP00000260102
ENSP00000216774	ENSP00000260956
ENSP00000216774	ENSP00000261531
ENSP00000216774	ENSP00000262027
ENSP00000216774	ENSP00000262584
ENSP00000216774	ENSP00000264995
ENSP00000216774	ENSP00000265100
ENSP00000216774	ENSP00000267884
ENSP00000216774	ENSP00000268661
ENSP00000216774	ENSP00000282050

ENSP00000216774	ENSP00000282276
ENSP00000216774	ENSP00000282388
ENSP00000216774	ENSP00000282999
ENSP00000216774	ENSP00000288937
ENSP00000216774	ENSP00000294053
ENSP00000216774	ENSP00000295890
ENSP00000216774	ENSP00000298283
ENSP00000216774	ENSP00000300151
ENSP00000216774	ENSP00000302896
ENSP00000216774	ENSP00000306548
ENSP00000216774	ENSP00000308897
ENSP00000216774	ENSP00000311028
ENSP00000216774	ENSP00000311430
ENSP00000216774	ENSP00000312066
ENSP00000216774	ENSP00000314311
ENSP00000216774	ENSP00000315859
ENSP00000216774	ENSP00000317376
ENSP00000216774	ENSP00000320508
ENSP00000216774	ENSP00000326981
ENSP00000216774	ENSP00000328023
ENSP00000216774	ENSP00000339795
ENSP00000216774	ENSP00000341082
ENSP00000216774	ENSP00000342181
ENSP00000216774	ENSP00000346015
ENSP00000216774	ENSP00000346022
ENSP00000216774	ENSP00000353826
ENSP00000216774	ENSP00000354525
ENSP00000216774	ENSP00000354739
ENSP00000216774	ENSP00000359345
ENSP00000216774	ENSP00000360532
ENSP00000216774	ENSP00000361287
ENSP00000216774	ENSP00000363676
ENSP00000216774	ENSP00000366121
ENSP00000216774	ENSP00000373404
ENSP00000216774	ENSP00000375730
ENSP00000216774	ENSP00000377486
ENSP00000216774	ENSP00000377865
ENSP00000216774	ENSP00000379865
ENSP00000216774	ENSP00000382595
ENSP00000216774	ENSP00000389103
ENSP00000216774	ENSP00000396704
ENSP00000216774	ENSP00000403817
ENSP00000216774	ENSP00000416959
ENSP00000216832	ENSP00000221494
ENSP00000216832	ENSP00000251102
ENSP00000216832	ENSP00000253363

ENSP00000216832	ENSP00000260970
ENSP00000216832	ENSP00000261531
ENSP00000216832	ENSP00000301740
ENSP00000216832	ENSP00000311825
ENSP00000216832	ENSP00000315859
ENSP00000216832	ENSP00000326261
ENSP00000216832	ENSP00000329466
ENSP00000216832	ENSP00000337194
ENSP00000216832	ENSP00000337340
ENSP00000216832	ENSP00000343557
ENSP00000216832	ENSP00000358242
ENSP00000216832	ENSP00000358554
ENSP00000216832	ENSP00000360532
ENSP00000216832	ENSP00000361162
ENSP00000216832	ENSP00000362900
ENSP00000216832	ENSP00000365318
ENSP00000216832	ENSP00000366557
ENSP00000216832	ENSP00000379607
ENSP00000216832	ENSP00000383142
ENSP00000216840	ENSP00000219476
ENSP00000216840	ENSP00000246166
ENSP00000216840	ENSP00000251038
ENSP00000216840	ENSP00000286317
ENSP00000216840	ENSP00000355511
ENSP00000216840	ENSP00000363708
ENSP00000216840	ENSP00000366557
ENSP00000216840	ENSP00000366607
ENSP00000216840	ENSP00000394071
ENSP00000216879	ENSP00000217173
ENSP00000216879	ENSP00000219548
ENSP00000216879	ENSP00000220669
ENSP00000216879	ENSP00000240185
ENSP00000216879	ENSP00000252108
ENSP00000216879	ENSP00000252115
ENSP00000216879	ENSP00000255764
ENSP00000216879	ENSP00000259512
ENSP00000216879	ENSP00000263202
ENSP00000216879	ENSP00000267884
ENSP00000216879	ENSP00000276416
ENSP00000216879	ENSP00000280700
ENSP00000216879	ENSP00000289528
ENSP00000216879	ENSP00000294179
ENSP00000216879	ENSP00000297540
ENSP00000216879	ENSP00000309031
ENSP00000216879	ENSP00000309474
ENSP00000216879	ENSP00000310129

ENSP00000216879	ENSP00000321617
ENSP00000216879	ENSP00000326813
ENSP00000216879	ENSP00000331487
ENSP00000216879	ENSP00000331817
ENSP00000216879	ENSP00000341947
ENSP00000216879	ENSP00000343001
ENSP00000216879	ENSP00000346088
ENSP00000216879	ENSP00000348602
ENSP00000216879	ENSP00000364486
ENSP00000216879	ENSP00000375881
ENSP00000216879	ENSP00000380460
ENSP00000216879	ENSP00000382562
ENSP00000216923	ENSP00000312649
ENSP00000216923	ENSP00000318373
ENSP00000216923	ENSP00000347301
ENSP00000216923	ENSP00000378503
ENSP00000216951	ENSP00000229416
ENSP00000216951	ENSP00000241052
ENSP00000216951	ENSP00000251038
ENSP00000216951	ENSP00000263228
ENSP00000216951	ENSP00000272286
ENSP00000216951	ENSP00000276603
ENSP00000216951	ENSP00000354995
ENSP00000216951	ENSP00000365943
ENSP00000216968	ENSP00000257430
ENSP00000216968	ENSP00000321326
ENSP00000216968	ENSP00000339191
ENSP00000217109	ENSP00000243563
ENSP00000217109	ENSP00000260947
ENSP00000217109	ENSP00000261531
ENSP00000217109	ENSP00000262077
ENSP00000217109	ENSP00000263857
ENSP00000217109	ENSP00000292476
ENSP00000217109	ENSP00000314733
ENSP00000217109	ENSP00000314949
ENSP00000217109	ENSP00000315791
ENSP00000217109	ENSP00000318861
ENSP00000217109	ENSP00000336752
ENSP00000217109	ENSP00000350283
ENSP00000217109	ENSP00000366557
ENSP00000217109	ENSP00000368438
ENSP00000217109	ENSP00000379339
ENSP00000217130	ENSP00000269468
ENSP00000217130	ENSP00000285021
ENSP00000217130	ENSP00000339845
ENSP00000217173	ENSP00000231524

ENSP00000217173	ENSP00000231790
ENSP00000217173	ENSP00000263202
ENSP00000217173	ENSP00000283943
ENSP00000217173	ENSP00000321617
ENSP00000217173	ENSP00000326813
ENSP00000217173	ENSP00000326921
ENSP00000217173	ENSP00000328203
ENSP00000217173	ENSP00000331201
ENSP00000217173	ENSP00000331487
ENSP00000217173	ENSP00000340305
ENSP00000217173	ENSP00000347498
ENSP00000217173	ENSP00000347836
ENSP00000217173	ENSP00000347997
ENSP00000217173	ENSP00000350708
ENSP00000217173	ENSP00000354883
ENSP00000217173	ENSP00000363338
ENSP00000217173	ENSP00000369531
ENSP00000217173	ENSP00000380460
ENSP00000217195	ENSP00000254942
ENSP00000217244	ENSP00000217515
ENSP00000217244	ENSP00000219700
ENSP00000217244	ENSP00000220058
ENSP00000217244	ENSP00000221265
ENSP00000217244	ENSP00000222005
ENSP00000217244	ENSP00000222330
ENSP00000217244	ENSP00000225504
ENSP00000217244	ENSP00000225983
ENSP00000217244	ENSP00000226218
ENSP00000217244	ENSP00000227163
ENSP00000217244	ENSP00000228916
ENSP00000217244	ENSP00000229251
ENSP00000217244	ENSP00000229595
ENSP00000217244	ENSP00000230056
ENSP00000217244	ENSP00000236192
ENSP00000217244	ENSP00000236979
ENSP00000217244	ENSP00000237596
ENSP00000217244	ENSP00000238789
ENSP00000217244	ENSP00000239938
ENSP00000217244	ENSP00000244496
ENSP00000217244	ENSP00000244741
ENSP00000217244	ENSP00000245960
ENSP00000217244	ENSP00000246891
ENSP00000217244	ENSP00000247970
ENSP00000217244	ENSP00000252445
ENSP00000217244	ENSP00000252622
ENSP00000217244	ENSP00000253107

ENSP00000217244	ENSP00000253693
ENSP00000217244	ENSP00000254079
ENSP00000217244	ENSP00000254301
ENSP00000217244	ENSP00000256474
ENSP00000217244	ENSP00000256897
ENSP00000217244	ENSP00000257181
ENSP00000217244	ENSP00000257430
ENSP00000217244	ENSP00000260803
ENSP00000217244	ENSP00000261245
ENSP00000217244	ENSP00000261448
ENSP00000217244	ENSP00000262367
ENSP00000217244	ENSP00000263228
ENSP00000217244	ENSP00000263274
ENSP00000217244	ENSP00000264670
ENSP00000217244	ENSP00000264834
ENSP00000217244	ENSP00000265073
ENSP00000217244	ENSP00000265354
ENSP00000217244	ENSP00000265372
ENSP00000217244	ENSP00000265462
ENSP00000217244	ENSP00000267812
ENSP00000217244	ENSP00000268058
ENSP00000217244	ENSP00000268605
ENSP00000217244	ENSP00000268717
ENSP00000217244	ENSP00000269127
ENSP00000217244	ENSP00000269385
ENSP00000217244	ENSP00000269397
ENSP00000217244	ENSP00000272937
ENSP00000217244	ENSP00000273480
ENSP00000217244	ENSP00000273783
ENSP00000217244	ENSP00000276072
ENSP00000217244	ENSP00000278412
ENSP00000217244	ENSP00000279392
ENSP00000217244	ENSP00000281043
ENSP00000217244	ENSP00000283882
ENSP00000217244	ENSP00000283928
ENSP00000217244	ENSP00000285243
ENSP00000217244	ENSP00000287878
ENSP00000217244	ENSP00000294008
ENSP00000217244	ENSP00000295685
ENSP00000217244	ENSP00000296028
ENSP00000217244	ENSP00000296509
ENSP00000217244	ENSP00000297338
ENSP00000217244	ENSP00000297990
ENSP00000217244	ENSP00000298281
ENSP00000217244	ENSP00000299259
ENSP00000217244	ENSP00000299543

ENSP00000217244	ENSP00000299601
ENSP00000217244	ENSP00000299767
ENSP00000217244	ENSP00000302111
ENSP00000217244	ENSP00000302640
ENSP00000217244	ENSP00000302935
ENSP00000217244	ENSP00000305790
ENSP00000217244	ENSP00000305958
ENSP00000217244	ENSP00000306245
ENSP00000217244	ENSP00000307491
ENSP00000217244	ENSP00000308750
ENSP00000217244	ENSP00000310040
ENSP00000217244	ENSP00000310042
ENSP00000217244	ENSP00000311677
ENSP00000217244	ENSP00000312042
ENSP00000217244	ENSP00000312664
ENSP00000217244	ENSP00000312735
ENSP00000217244	ENSP00000313420
ENSP00000217244	ENSP00000314620
ENSP00000217244	ENSP00000314949
ENSP00000217244	ENSP00000314971
ENSP00000217244	ENSP00000315859
ENSP00000217244	ENSP00000316454
ENSP00000217244	ENSP00000317904
ENSP00000217244	ENSP00000318195
ENSP00000217244	ENSP00000318604
ENSP00000217244	ENSP00000318822
ENSP00000217244	ENSP00000319104
ENSP00000217244	ENSP00000321449
ENSP00000217244	ENSP00000322419
ENSP00000217244	ENSP00000325120
ENSP00000217244	ENSP00000327179
ENSP00000217244	ENSP00000329029
ENSP00000217244	ENSP00000329357
ENSP00000217244	ENSP00000329419
ENSP00000217244	ENSP00000329466
ENSP00000217244	ENSP00000330753
ENSP00000217244	ENSP00000334538
ENSP00000217244	ENSP00000336752
ENSP00000217244	ENSP00000338345
ENSP00000217244	ENSP00000339191
ENSP00000217244	ENSP00000340671
ENSP00000217244	ENSP00000342011
ENSP00000217244	ENSP00000342681
ENSP00000217244	ENSP00000343190
ENSP00000217244	ENSP00000344456
ENSP00000217244	ENSP00000344572

ENSP00000217244	ENSP00000346566
ENSP00000217244	ENSP00000347168
ENSP00000217244	ENSP00000347329
ENSP00000217244	ENSP00000347548
ENSP00000217244	ENSP00000348577
ENSP00000217244	ENSP00000348877
ENSP00000217244	ENSP00000349437
ENSP00000217244	ENSP00000349640
ENSP00000217244	ENSP00000350283
ENSP00000217244	ENSP00000350704
ENSP00000217244	ENSP00000350757
ENSP00000217244	ENSP00000350767
ENSP00000217244	ENSP00000351284
ENSP00000217244	ENSP00000351407
ENSP00000217244	ENSP00000351490
ENSP00000217244	ENSP00000351492
ENSP00000217244	ENSP00000351575
ENSP00000217244	ENSP00000351885
ENSP00000217244	ENSP00000351947
ENSP00000217244	ENSP00000352257
ENSP00000217244	ENSP00000353622
ENSP00000217244	ENSP00000353698
ENSP00000217244	ENSP00000353741
ENSP00000217244	ENSP00000354125
ENSP00000217244	ENSP00000354522
ENSP00000217244	ENSP00000354558
ENSP00000217244	ENSP00000355013
ENSP00000217244	ENSP00000355051
ENSP00000217244	ENSP00000355899
ENSP00000217244	ENSP00000356346
ENSP00000217244	ENSP00000356405
ENSP00000217244	ENSP00000357113
ENSP00000217244	ENSP00000357789
ENSP00000217244	ENSP00000357998
ENSP00000217244	ENSP00000358120
ENSP00000217244	ENSP00000358140
ENSP00000217244	ENSP00000358404
ENSP00000217244	ENSP00000359345
ENSP00000217244	ENSP00000360497
ENSP00000217244	ENSP00000360821
ENSP00000217244	ENSP00000362824
ENSP00000217244	ENSP00000364119
ENSP00000217244	ENSP00000365025
ENSP00000217244	ENSP00000365891
ENSP00000217244	ENSP00000366271
ENSP00000217244	ENSP00000366974

ENSP00000217244	ENSP00000367034
ENSP00000217244	ENSP00000367208
ENSP00000217244	ENSP00000368438
ENSP00000217244	ENSP00000369162
ENSP00000217244	ENSP00000369716
ENSP00000217244	ENSP00000370571
ENSP00000217244	ENSP00000370589
ENSP00000217244	ENSP00000372654
ENSP00000217244	ENSP00000374280
ENSP00000217244	ENSP00000374455
ENSP00000217244	ENSP00000377218
ENSP00000217244	ENSP00000377262
ENSP00000217244	ENSP00000377372
ENSP00000217244	ENSP00000377747
ENSP00000217244	ENSP00000377969
ENSP00000217244	ENSP00000378517
ENSP00000217244	ENSP00000379457
ENSP00000217244	ENSP00000380414
ENSP00000217244	ENSP00000380982
ENSP00000217244	ENSP00000382779
ENSP00000217244	ENSP00000384026
ENSP00000217244	ENSP00000384313
ENSP00000217244	ENSP00000384597
ENSP00000217244	ENSP00000386621
ENSP00000217244	ENSP00000386759
ENSP00000217244	ENSP00000391944
ENSP00000217244	ENSP00000392028
ENSP00000217244	ENSP00000402084
ENSP00000217244	ENSP00000402935
ENSP00000217244	ENSP00000409167
ENSP00000217244	ENSP00000419494
ENSP00000217246	ENSP00000254942
ENSP00000217246	ENSP00000276603
ENSP00000217372	ENSP00000265295
ENSP00000217372	ENSP00000323046
ENSP00000217446	ENSP00000251038
ENSP00000217446	ENSP00000256031
ENSP00000217446	ENSP00000261534
ENSP00000217446	ENSP00000278243
ENSP00000217446	ENSP00000299626
ENSP00000217446	ENSP00000300658
ENSP00000217446	ENSP00000309430
ENSP00000217446	ENSP00000326699
ENSP00000217446	ENSP00000334052
ENSP00000217446	ENSP00000340466
ENSP00000217446	ENSP00000347206

ENSP00000217446	ENSP00000350263
ENSP00000217446	ENSP00000359848
ENSP00000217446	ENSP00000360644
ENSP00000217446	ENSP00000415203
ENSP00000217455	ENSP00000334044
ENSP00000217455	ENSP00000339484
ENSP00000217455	ENSP00000366249
ENSP00000217455	ENSP00000368699
ENSP00000217455	ENSP00000373039
ENSP00000217455	ENSP00000387262
ENSP00000217455	ENSP00000396152
ENSP00000217455	ENSP00000410416
ENSP00000217515	ENSP00000219313
ENSP00000217515	ENSP00000233893
ENSP00000217515	ENSP00000234590
ENSP00000217515	ENSP00000261601
ENSP00000217515	ENSP00000265462
ENSP00000217515	ENSP00000286428
ENSP00000217515	ENSP00000292180
ENSP00000217515	ENSP00000295901
ENSP00000217515	ENSP00000296577
ENSP00000217515	ENSP00000298852
ENSP00000217515	ENSP00000302160
ENSP00000217515	ENSP00000310129
ENSP00000217515	ENSP00000313921
ENSP00000217515	ENSP00000330836
ENSP00000217515	ENSP00000340237
ENSP00000217515	ENSP00000362110
ENSP00000217515	ENSP00000375881
ENSP00000217893	ENSP00000225916
ENSP00000217893	ENSP00000233242
ENSP00000217893	ENSP00000240055
ENSP00000217893	ENSP00000240316
ENSP00000217893	ENSP00000246548
ENSP00000217893	ENSP00000247843
ENSP00000217893	ENSP00000251038
ENSP00000217893	ENSP00000252996
ENSP00000217893	ENSP00000254037
ENSP00000217893	ENSP00000256383
ENSP00000217893	ENSP00000256398
ENSP00000217893	ENSP00000256854
ENSP00000217893	ENSP00000256996
ENSP00000217893	ENSP00000259469
ENSP00000217893	ENSP00000260956
ENSP00000217893	ENSP00000261531
ENSP00000217893	ENSP00000261593

ENSP00000217893	ENSP00000262039
ENSP00000217893	ENSP00000263390
ENSP00000217893	ENSP00000263974
ENSP00000217893	ENSP00000264193
ENSP00000217893	ENSP00000264335
ENSP00000217893	ENSP00000264515
ENSP00000217893	ENSP00000264834
ENSP00000217893	ENSP00000264883
ENSP00000217893	ENSP00000265970
ENSP00000217893	ENSP00000265990
ENSP00000217893	ENSP00000272286
ENSP00000217893	ENSP00000276072
ENSP00000217893	ENSP00000278616
ENSP00000217893	ENSP00000283882
ENSP00000217893	ENSP00000294168
ENSP00000217893	ENSP00000298937
ENSP00000217893	ENSP00000299424
ENSP00000217893	ENSP00000303315
ENSP00000217893	ENSP00000305422
ENSP00000217893	ENSP00000305790
ENSP00000217893	ENSP00000306983
ENSP00000217893	ENSP00000307684
ENSP00000217893	ENSP00000307850
ENSP00000217893	ENSP00000309555
ENSP00000217893	ENSP00000311028
ENSP00000217893	ENSP00000311505
ENSP00000217893	ENSP00000313420
ENSP00000217893	ENSP00000314311
ENSP00000217893	ENSP00000316114
ENSP00000217893	ENSP00000331310
ENSP00000217893	ENSP00000338413
ENSP00000217893	ENSP00000339109
ENSP00000217893	ENSP00000339529
ENSP00000217893	ENSP00000341805
ENSP00000217893	ENSP00000343701
ENSP00000217893	ENSP00000343741
ENSP00000217893	ENSP00000344537
ENSP00000217893	ENSP00000345702
ENSP00000217893	ENSP00000347733
ENSP00000217893	ENSP00000350283
ENSP00000217893	ENSP00000350967
ENSP00000217893	ENSP00000352121
ENSP00000217893	ENSP00000352673
ENSP00000217893	ENSP00000353575
ENSP00000217893	ENSP00000354558
ENSP00000217893	ENSP00000354633

ENSP00000217893	ENSP00000354951
ENSP00000217893	ENSP00000354995
ENSP00000217893	ENSP00000358854
ENSP00000217893	ENSP00000358886
ENSP00000217893	ENSP00000359531
ENSP00000217893	ENSP00000360515
ENSP00000217893	ENSP00000362068
ENSP00000217893	ENSP00000362688
ENSP00000217893	ENSP00000363779
ENSP00000217893	ENSP00000364895
ENSP00000217893	ENSP00000367406
ENSP00000217893	ENSP00000369695
ENSP00000217893	ENSP00000369736
ENSP00000217893	ENSP00000369871
ENSP00000217893	ENSP00000389182
ENSP00000217893	ENSP00000397259
ENSP00000217893	ENSP00000402515
ENSP00000217901	ENSP00000222673
ENSP00000217901	ENSP00000253792
ENSP00000217901	ENSP00000259569
ENSP00000217901	ENSP00000263201
ENSP00000217901	ENSP00000264705
ENSP00000217901	ENSP00000265195
ENSP00000217901	ENSP00000269122
ENSP00000217901	ENSP00000273398
ENSP00000217901	ENSP00000280346
ENSP00000217901	ENSP00000300648
ENSP00000217901	ENSP00000310623
ENSP00000217901	ENSP00000313953
ENSP00000217901	ENSP00000317159
ENSP00000217901	ENSP00000327070
ENSP00000217901	ENSP00000331897
ENSP00000217901	ENSP00000333946
ENSP00000217901	ENSP00000335261
ENSP00000217901	ENSP00000335304
ENSP00000217901	ENSP00000342056
ENSP00000217901	ENSP00000346173
ENSP00000217901	ENSP00000346453
ENSP00000217901	ENSP00000350230
ENSP00000217901	ENSP00000353826
ENSP00000217901	ENSP00000355013
ENSP00000217901	ENSP00000361699
ENSP00000217901	ENSP00000365534
ENSP00000217901	ENSP00000367923
ENSP00000217901	ENSP00000368439
ENSP00000217901	ENSP00000369757

ENSP00000217901	ENSP00000377446
ENSP00000217901	ENSP00000377527
ENSP00000217901	ENSP00000378782
ENSP00000217901	ENSP00000387262
ENSP00000217939	ENSP00000232905
ENSP00000217939	ENSP00000258418
ENSP00000217964	ENSP00000219473
ENSP00000217964	ENSP00000225983
ENSP00000217964	ENSP00000231487
ENSP00000217964	ENSP00000232978
ENSP00000217964	ENSP00000247843
ENSP00000217964	ENSP00000251038
ENSP00000217964	ENSP00000251871
ENSP00000217964	ENSP00000255764
ENSP00000217964	ENSP00000256031
ENSP00000217964	ENSP00000256379
ENSP00000217964	ENSP00000258648
ENSP00000217964	ENSP00000260970
ENSP00000217964	ENSP00000262518
ENSP00000217964	ENSP00000262519
ENSP00000217964	ENSP00000262904
ENSP00000217964	ENSP00000263121
ENSP00000217964	ENSP00000263205
ENSP00000217964	ENSP00000264160
ENSP00000217964	ENSP00000264637
ENSP00000217964	ENSP00000265175
ENSP00000217964	ENSP00000265350
ENSP00000217964	ENSP00000267064
ENSP00000217964	ENSP00000267101
ENSP00000217964	ENSP00000267176
ENSP00000217964	ENSP00000267197
ENSP00000217964	ENSP00000267415
ENSP00000217964	ENSP00000276603
ENSP00000217964	ENSP00000277541
ENSP00000217964	ENSP00000281928
ENSP00000217964	ENSP00000283195
ENSP00000217964	ENSP00000286317
ENSP00000217964	ENSP00000290158
ENSP00000217964	ENSP00000290663
ENSP00000217964	ENSP00000292035
ENSP00000217964	ENSP00000293777
ENSP00000217964	ENSP00000296755
ENSP00000217964	ENSP00000297338
ENSP00000217964	ENSP00000297347
ENSP00000217964	ENSP00000300231
ENSP00000217964	ENSP00000304593

ENSP00000217964	ENSP00000308699
ENSP00000217964	ENSP00000311028
ENSP00000217964	ENSP00000312262
ENSP00000217964	ENSP00000313829
ENSP00000217964	ENSP00000315791
ENSP00000217964	ENSP00000320566
ENSP00000217964	ENSP00000323302
ENSP00000217964	ENSP00000323720
ENSP00000217964	ENSP00000325612
ENSP00000217964	ENSP00000326128
ENSP00000217964	ENSP00000326767
ENSP00000217964	ENSP00000328169
ENSP00000217964	ENSP00000334188
ENSP00000217964	ENSP00000336842
ENSP00000217964	ENSP00000344456
ENSP00000217964	ENSP00000348551
ENSP00000217964	ENSP00000353622
ENSP00000217964	ENSP00000355205
ENSP00000217964	ENSP00000356405
ENSP00000217964	ENSP00000356448
ENSP00000217964	ENSP00000356652
ENSP00000217964	ENSP00000356989
ENSP00000217964	ENSP00000357047
ENSP00000217964	ENSP00000357494
ENSP00000217964	ENSP00000357887
ENSP00000217964	ENSP00000357907
ENSP00000217964	ENSP00000360371
ENSP00000217964	ENSP00000360621
ENSP00000217964	ENSP00000362948
ENSP00000217964	ENSP00000363313
ENSP00000217964	ENSP00000363734
ENSP00000217964	ENSP00000364929
ENSP00000217964	ENSP00000370826
ENSP00000217964	ENSP00000372694
ENSP00000217964	ENSP00000374213
ENSP00000217964	ENSP00000381216
ENSP00000217964	ENSP00000384048
ENSP00000217964	ENSP00000386759
ENSP00000217964	ENSP00000391774
ENSP00000217964	ENSP00000392617
ENSP00000217964	ENSP00000412319
ENSP00000217964	ENSP00000415678
ENSP00000217999	ENSP00000226218
ENSP00000217999	ENSP00000372023
ENSP00000218004	ENSP00000283195
ENSP00000218004	ENSP00000290158

ENSP00000218004	ENSP00000300128
ENSP00000218004	ENSP00000336712
ENSP00000218004	ENSP00000348577
ENSP00000218006	ENSP00000229922
ENSP00000218006	ENSP00000251102
ENSP00000218006	ENSP00000262460
ENSP00000218006	ENSP00000264917
ENSP00000218006	ENSP00000337127
ENSP00000218006	ENSP00000351015
ENSP00000218006	ENSP00000367208
ENSP00000218056	ENSP00000253003
ENSP00000218056	ENSP00000256398
ENSP00000218056	ENSP00000282892
ENSP00000218056	ENSP00000298937
ENSP00000218056	ENSP00000304593
ENSP00000218056	ENSP00000334061
ENSP00000218056	ENSP00000357907
ENSP00000218056	ENSP00000360473
ENSP00000218056	ENSP00000386759
ENSP00000218056	ENSP00000396937
ENSP00000218104	ENSP00000254942
ENSP00000218104	ENSP00000256216
ENSP00000218104	ENSP00000258873
ENSP00000218104	ENSP00000264951
ENSP00000218104	ENSP00000300146
ENSP00000218104	ENSP00000302160
ENSP00000218104	ENSP00000346067
ENSP00000218104	ENSP00000349016
ENSP00000218104	ENSP00000350249
ENSP00000218104	ENSP00000357051
ENSP00000218104	ENSP00000360798
ENSP00000218104	ENSP00000370719
ENSP00000218104	ENSP00000410758
ENSP00000218147	ENSP00000225983
ENSP00000218147	ENSP00000419494
ENSP00000218364	ENSP00000221448
ENSP00000218364	ENSP00000221494
ENSP00000218364	ENSP00000225504
ENSP00000218364	ENSP00000227524
ENSP00000218364	ENSP00000233084
ENSP00000218364	ENSP00000251038
ENSP00000218364	ENSP00000254193
ENSP00000218364	ENSP00000261531
ENSP00000218364	ENSP00000301764
ENSP00000218364	ENSP00000303015
ENSP00000218364	ENSP00000305790

ENSP00000218364	ENSP00000307863
ENSP00000218364	ENSP00000310723
ENSP00000218364	ENSP00000314949
ENSP00000218364	ENSP00000335321
ENSP00000218364	ENSP00000340823
ENSP00000218364	ENSP00000346236
ENSP00000218364	ENSP00000354522
ENSP00000218364	ENSP00000361577
ENSP00000218364	ENSP00000362110
ENSP00000218364	ENSP00000362900
ENSP00000218364	ENSP00000366557
ENSP00000218364	ENSP00000368801
ENSP00000218364	ENSP00000380982
ENSP00000218364	ENSP00000412566
ENSP00000218516	ENSP00000285599
ENSP00000218516	ENSP00000353655
ENSP00000218516	ENSP00000409279
ENSP00000218652	ENSP00000220763
ENSP00000218652	ENSP00000223051
ENSP00000218652	ENSP00000251968
ENSP00000218652	ENSP00000262052
ENSP00000218652	ENSP00000297265
ENSP00000218652	ENSP00000297290
ENSP00000218652	ENSP00000300658
ENSP00000218652	ENSP00000311200
ENSP00000218652	ENSP00000324560
ENSP00000218652	ENSP00000326921
ENSP00000218652	ENSP00000331201
ENSP00000218652	ENSP00000337127
ENSP00000218652	ENSP00000345530
ENSP00000218652	ENSP00000347408
ENSP00000218652	ENSP00000347498
ENSP00000218652	ENSP00000367299
ENSP00000218652	ENSP00000374328
ENSP00000218652	ENSP00000379500
ENSP00000218652	ENSP00000400312
ENSP00000218652	ENSP00000404705
ENSP00000218758	ENSP00000272322
ENSP00000218758	ENSP00000273398
ENSP00000218758	ENSP00000314499
ENSP00000218758	ENSP00000378517
ENSP00000219022	ENSP00000300589
ENSP00000219066	ENSP00000222286
ENSP00000219066	ENSP00000224862
ENSP00000219066	ENSP00000230859
ENSP00000219066	ENSP00000231790

ENSP00000219066	ENSP00000233146
ENSP00000219066	ENSP00000234420
ENSP00000219066	ENSP00000242576
ENSP00000219066	ENSP00000258428
ENSP00000219066	ENSP00000263187
ENSP00000219066	ENSP00000264926
ENSP00000219066	ENSP00000265081
ENSP00000219066	ENSP00000265849
ENSP00000219066	ENSP00000267430
ENSP00000219066	ENSP00000282999
ENSP00000219066	ENSP00000288985
ENSP00000219066	ENSP00000296273
ENSP00000219066	ENSP00000306080
ENSP00000219066	ENSP00000306561
ENSP00000219066	ENSP00000307853
ENSP00000219066	ENSP00000308944
ENSP00000219066	ENSP00000310520
ENSP00000219066	ENSP00000318977
ENSP00000219066	ENSP00000322570
ENSP00000219066	ENSP00000323511
ENSP00000219066	ENSP00000324792
ENSP00000219066	ENSP00000330343
ENSP00000219066	ENSP00000334675
ENSP00000219066	ENSP00000337353
ENSP00000219066	ENSP00000339503
ENSP00000219066	ENSP00000343392
ENSP00000219066	ENSP00000343741
ENSP00000219066	ENSP00000344504
ENSP00000219066	ENSP00000347978
ENSP00000219066	ENSP00000348020
ENSP00000219066	ENSP00000348089
ENSP00000219066	ENSP00000349016
ENSP00000219066	ENSP00000351284
ENSP00000219066	ENSP00000351697
ENSP00000219066	ENSP00000352121
ENSP00000219066	ENSP00000352257
ENSP00000219066	ENSP00000354522
ENSP00000219066	ENSP00000354558
ENSP00000219066	ENSP00000358478
ENSP00000219066	ENSP00000361310
ENSP00000219066	ENSP00000364126
ENSP00000219066	ENSP00000364270
ENSP00000219066	ENSP00000368349
ENSP00000219066	ENSP00000374213
ENSP00000219066	ENSP00000400806
ENSP00000219066	ENSP00000406490

ENSP00000219066	ENSP00000406868
ENSP00000219097	ENSP00000223129
ENSP00000219097	ENSP00000234296
ENSP00000219097	ENSP00000234626
ENSP00000219097	ENSP00000254719
ENSP00000219097	ENSP00000254942
ENSP00000219097	ENSP00000257789
ENSP00000219097	ENSP00000263201
ENSP00000219097	ENSP00000264169
ENSP00000219097	ENSP00000265056
ENSP00000219097	ENSP00000265728
ENSP00000219097	ENSP00000297431
ENSP00000219097	ENSP00000352257
ENSP00000219097	ENSP00000360621
ENSP00000219139	ENSP00000247986
ENSP00000219139	ENSP00000284981
ENSP00000219139	ENSP00000367408
ENSP00000219168	ENSP00000369519
ENSP00000219169	ENSP00000219409
ENSP00000219169	ENSP00000219700
ENSP00000219169	ENSP00000226105
ENSP00000219169	ENSP00000228872
ENSP00000219169	ENSP00000231498
ENSP00000219169	ENSP00000251038
ENSP00000219169	ENSP00000259667
ENSP00000219169	ENSP00000262077
ENSP00000219169	ENSP00000264883
ENSP00000219169	ENSP00000265304
ENSP00000219169	ENSP00000278412
ENSP00000219169	ENSP00000283195
ENSP00000219169	ENSP00000290158
ENSP00000219169	ENSP00000290231
ENSP00000219169	ENSP00000296509
ENSP00000219169	ENSP00000304593
ENSP00000219169	ENSP00000305503
ENSP00000219169	ENSP00000310668
ENSP00000219169	ENSP00000311436
ENSP00000219169	ENSP00000316032
ENSP00000219169	ENSP00000330343
ENSP00000219169	ENSP00000332721
ENSP00000219169	ENSP00000343701
ENSP00000219169	ENSP00000344547
ENSP00000219169	ENSP00000351926
ENSP00000219169	ENSP00000362463
ENSP00000219169	ENSP00000365811
ENSP00000219169	ENSP00000371155

ENSP00000219235	ENSP00000229416
ENSP00000219235	ENSP00000292199
ENSP00000219235	ENSP00000294728
ENSP00000219240	ENSP00000251038
ENSP00000219240	ENSP00000264718
ENSP00000219240	ENSP00000264932
ENSP00000219240	ENSP00000282050
ENSP00000219240	ENSP00000293195
ENSP00000219240	ENSP00000354499
ENSP00000219240	ENSP00000355518
ENSP00000219240	ENSP00000359211
ENSP00000219252	ENSP00000221249
ENSP00000219252	ENSP00000221265
ENSP00000219252	ENSP00000221859
ENSP00000219252	ENSP00000222286
ENSP00000219252	ENSP00000222567
ENSP00000219252	ENSP00000225504
ENSP00000219252	ENSP00000228136
ENSP00000219252	ENSP00000230050
ENSP00000219252	ENSP00000235090
ENSP00000219252	ENSP00000237380
ENSP00000219252	ENSP00000237455
ENSP00000219252	ENSP00000238789
ENSP00000219252	ENSP00000241600
ENSP00000219252	ENSP00000242719
ENSP00000219252	ENSP00000245539
ENSP00000219252	ENSP00000247219
ENSP00000219252	ENSP00000247843
ENSP00000219252	ENSP00000251871
ENSP00000219252	ENSP00000252115
ENSP00000219252	ENSP00000252445
ENSP00000219252	ENSP00000253107
ENSP00000219252	ENSP00000255764
ENSP00000219252	ENSP00000256257
ENSP00000219252	ENSP00000256379
ENSP00000219252	ENSP00000259605
ENSP00000219252	ENSP00000260956
ENSP00000219252	ENSP00000261168
ENSP00000219252	ENSP00000261917
ENSP00000219252	ENSP00000263390
ENSP00000219252	ENSP00000263997
ENSP00000219252	ENSP00000264399
ENSP00000219252	ENSP00000264515
ENSP00000219252	ENSP00000264718
ENSP00000219252	ENSP00000264995
ENSP00000219252	ENSP00000265073

ENSP00000219252	ENSP00000265304
ENSP00000219252	ENSP00000265717
ENSP00000219252	ENSP00000265963
ENSP00000219252	ENSP00000267064
ENSP00000219252	ENSP00000267978
ENSP00000219252	ENSP00000268661
ENSP00000219252	ENSP00000268711
ENSP00000219252	ENSP00000270625
ENSP00000219252	ENSP00000272444
ENSP00000219252	ENSP00000272645
ENSP00000219252	ENSP00000278572
ENSP00000219252	ENSP00000282884
ENSP00000219252	ENSP00000283875
ENSP00000219252	ENSP00000285398
ENSP00000219252	ENSP00000286317
ENSP00000219252	ENSP00000286835
ENSP00000219252	ENSP00000287878
ENSP00000219252	ENSP00000287936
ENSP00000219252	ENSP00000288199
ENSP00000219252	ENSP00000288985
ENSP00000219252	ENSP00000292035
ENSP00000219252	ENSP00000292363
ENSP00000219252	ENSP00000295131
ENSP00000219252	ENSP00000295321
ENSP00000219252	ENSP00000296674
ENSP00000219252	ENSP00000298746
ENSP00000219252	ENSP00000299543
ENSP00000219252	ENSP00000299767
ENSP00000219252	ENSP00000300291
ENSP00000219252	ENSP00000300648
ENSP00000219252	ENSP00000301788
ENSP00000219252	ENSP00000302478
ENSP00000219252	ENSP00000304321
ENSP00000219252	ENSP00000304593
ENSP00000219252	ENSP00000305459
ENSP00000219252	ENSP00000306123
ENSP00000219252	ENSP00000306906
ENSP00000219252	ENSP00000306983
ENSP00000219252	ENSP00000310260
ENSP00000219252	ENSP00000310723
ENSP00000219252	ENSP00000312735
ENSP00000219252	ENSP00000314311
ENSP00000219252	ENSP00000314949
ENSP00000219252	ENSP00000315950
ENSP00000219252	ENSP00000317636
ENSP00000219252	ENSP00000318604

ENSP00000219252	ENSP00000318641
ENSP00000219252	ENSP00000319104
ENSP00000219252	ENSP00000319169
ENSP00000219252	ENSP00000321636
ENSP00000219252	ENSP00000323720
ENSP00000219252	ENSP00000324122
ENSP00000219252	ENSP00000324124
ENSP00000219252	ENSP00000325677
ENSP00000219252	ENSP00000326238
ENSP00000219252	ENSP00000326767
ENSP00000219252	ENSP00000326806
ENSP00000219252	ENSP00000327889
ENSP00000219252	ENSP00000329933
ENSP00000219252	ENSP00000331111
ENSP00000219252	ENSP00000331817
ENSP00000219252	ENSP00000334675
ENSP00000219252	ENSP00000336606
ENSP00000219252	ENSP00000336790
ENSP00000219252	ENSP00000336842
ENSP00000219252	ENSP00000337340
ENSP00000219252	ENSP00000339495
ENSP00000219252	ENSP00000339933
ENSP00000219252	ENSP00000340796
ENSP00000219252	ENSP00000340823
ENSP00000219252	ENSP00000342121
ENSP00000219252	ENSP00000342300
ENSP00000219252	ENSP00000342889
ENSP00000219252	ENSP00000345096
ENSP00000219252	ENSP00000345530
ENSP00000219252	ENSP00000346022
ENSP00000219252	ENSP00000346067
ENSP00000219252	ENSP00000347978
ENSP00000219252	ENSP00000348089
ENSP00000219252	ENSP00000348168
ENSP00000219252	ENSP00000350967
ENSP00000219252	ENSP00000353655
ENSP00000219252	ENSP00000354775
ENSP00000219252	ENSP00000355013
ENSP00000219252	ENSP00000356146
ENSP00000219252	ENSP00000358497
ENSP00000219252	ENSP00000358563
ENSP00000219252	ENSP00000359531
ENSP00000219252	ENSP00000361084
ENSP00000219252	ENSP00000361982
ENSP00000219252	ENSP00000362900
ENSP00000219252	ENSP00000363018

ENSP00000219252	ENSP00000363676
ENSP00000219252	ENSP00000363779
ENSP00000219252	ENSP00000366135
ENSP00000219252	ENSP00000366828
ENSP00000219252	ENSP00000368927
ENSP00000219252	ENSP00000369695
ENSP00000219252	ENSP00000370589
ENSP00000219252	ENSP00000371800
ENSP00000219252	ENSP00000372335
ENSP00000219252	ENSP00000377969
ENSP00000219252	ENSP00000378529
ENSP00000219252	ENSP00000379566
ENSP00000219252	ENSP00000379933
ENSP00000219252	ENSP00000380271
ENSP00000219252	ENSP00000381064
ENSP00000219252	ENSP00000383503
ENSP00000219252	ENSP00000385722
ENSP00000219252	ENSP00000386759
ENSP00000219252	ENSP00000389103
ENSP00000219252	ENSP00000396452
ENSP00000219252	ENSP00000397598
ENSP00000219252	ENSP00000397636
ENSP00000219252	ENSP00000403852
ENSP00000219252	ENSP00000405636
ENSP00000219252	ENSP00000410530
ENSP00000219252	ENSP00000410954
ENSP00000219252	ENSP00000414110
ENSP00000219252	ENSP00000414237
ENSP00000219252	ENSP00000416599
ENSP00000219281	ENSP00000341551
ENSP00000219313	ENSP00000219431
ENSP00000219313	ENSP00000222308
ENSP00000219313	ENSP00000222553
ENSP00000219313	ENSP00000225430
ENSP00000219313	ENSP00000231449
ENSP00000219313	ENSP00000231487
ENSP00000219313	ENSP00000237596
ENSP00000219313	ENSP00000248450
ENSP00000219313	ENSP00000248566
ENSP00000219313	ENSP00000250092
ENSP00000219313	ENSP00000251038
ENSP00000219313	ENSP00000253003
ENSP00000219313	ENSP00000256216
ENSP00000219313	ENSP00000256442
ENSP00000219313	ENSP00000259457
ENSP00000219313	ENSP00000261601

ENSP00000219313	ENSP00000261712
ENSP00000219313	ENSP00000262193
ENSP00000219313	ENSP00000262850
ENSP00000219313	ENSP00000264639
ENSP00000219313	ENSP00000265388
ENSP00000219313	ENSP00000265849
ENSP00000219313	ENSP00000280701
ENSP00000219313	ENSP00000284202
ENSP00000219313	ENSP00000289528
ENSP00000219313	ENSP00000290541
ENSP00000219313	ENSP00000295901
ENSP00000219313	ENSP00000296509
ENSP00000219313	ENSP00000296543
ENSP00000219313	ENSP00000298852
ENSP00000219313	ENSP00000309474
ENSP00000219313	ENSP00000310129
ENSP00000219313	ENSP00000311665
ENSP00000219313	ENSP00000317334
ENSP00000219313	ENSP00000325448
ENSP00000219313	ENSP00000331310
ENSP00000219313	ENSP00000336606
ENSP00000219313	ENSP00000336702
ENSP00000219313	ENSP00000337405
ENSP00000219313	ENSP00000339095
ENSP00000219313	ENSP00000341243
ENSP00000219313	ENSP00000347184
ENSP00000219313	ENSP00000348442
ENSP00000219313	ENSP00000350708
ENSP00000219313	ENSP00000353098
ENSP00000219313	ENSP00000354673
ENSP00000219313	ENSP00000359518
ENSP00000219313	ENSP00000360821
ENSP00000219313	ENSP00000361699
ENSP00000219313	ENSP00000362166
ENSP00000219313	ENSP00000362334
ENSP00000219313	ENSP00000364119
ENSP00000219313	ENSP00000379607
ENSP00000219313	ENSP00000381891
ENSP00000219313	ENSP00000384211
ENSP00000219313	ENSP00000386212
ENSP00000219313	ENSP00000396937
ENSP00000219313	ENSP00000401508
ENSP00000219313	ENSP00000402084
ENSP00000219313	ENSP00000406490
ENSP00000219313	ENSP00000417748
ENSP00000219368	ENSP00000220584

ENSP00000219368	ENSP00000220853
ENSP00000219368	ENSP00000228027
ENSP00000219368	ENSP00000244051
ENSP00000219368	ENSP00000245222
ENSP00000219368	ENSP00000247225
ENSP00000219368	ENSP00000251363
ENSP00000219368	ENSP00000256319
ENSP00000219368	ENSP00000258052
ENSP00000219368	ENSP00000261507
ENSP00000219368	ENSP00000262265
ENSP00000219368	ENSP00000262315
ENSP00000219368	ENSP00000262519
ENSP00000219368	ENSP00000263702
ENSP00000219368	ENSP00000264027
ENSP00000219368	ENSP00000265351
ENSP00000219368	ENSP00000265896
ENSP00000219368	ENSP00000267197
ENSP00000219368	ENSP00000270538
ENSP00000219368	ENSP00000271375
ENSP00000219368	ENSP00000272163
ENSP00000219368	ENSP00000277010
ENSP00000219368	ENSP00000280350
ENSP00000219368	ENSP00000285949
ENSP00000219368	ENSP00000286317
ENSP00000219368	ENSP00000287936
ENSP00000219368	ENSP00000288266
ENSP00000219368	ENSP00000292535
ENSP00000219368	ENSP00000298292
ENSP00000219368	ENSP00000302790
ENSP00000219368	ENSP00000310721
ENSP00000219368	ENSP00000312458
ENSP00000219368	ENSP00000315693
ENSP00000219368	ENSP00000317334
ENSP00000219368	ENSP00000318115
ENSP00000219368	ENSP00000327716
ENSP00000219368	ENSP00000331479
ENSP00000219368	ENSP00000331487
ENSP00000219368	ENSP00000332258
ENSP00000219368	ENSP00000333946
ENSP00000219368	ENSP00000336724
ENSP00000219368	ENSP00000341159
ENSP00000219368	ENSP00000341551
ENSP00000219368	ENSP00000341662
ENSP00000219368	ENSP00000342609
ENSP00000219368	ENSP00000346733
ENSP00000219368	ENSP00000348762

ENSP00000219368	ENSP00000350012
ENSP00000219368	ENSP00000350297
ENSP00000219368	ENSP00000351520
ENSP00000219368	ENSP00000354519
ENSP00000219368	ENSP00000355517
ENSP00000219368	ENSP00000356591
ENSP00000219368	ENSP00000358695
ENSP00000219368	ENSP00000358831
ENSP00000219368	ENSP00000359000
ENSP00000219368	ENSP00000359380
ENSP00000219368	ENSP00000365766
ENSP00000219368	ENSP00000371236
ENSP00000219368	ENSP00000372720
ENSP00000219368	ENSP00000379430
ENSP00000219368	ENSP00000381293
ENSP00000219368	ENSP00000381567
ENSP00000219368	ENSP00000386284
ENSP00000219368	ENSP00000389277
ENSP00000219368	ENSP00000397441
ENSP00000219368	ENSP00000402240
ENSP00000219368	ENSP00000412922
ENSP00000219400	ENSP00000251038
ENSP00000219400	ENSP00000297151
ENSP00000219400	ENSP00000418348
ENSP00000219409	ENSP00000280605
ENSP00000219409	ENSP00000368438
ENSP00000219431	ENSP00000233893
ENSP00000219431	ENSP00000245206
ENSP00000219431	ENSP00000256594
ENSP00000219431	ENSP00000265881
ENSP00000219431	ENSP00000290299
ENSP00000219431	ENSP00000300086
ENSP00000219431	ENSP00000300291
ENSP00000219431	ENSP00000337127
ENSP00000219431	ENSP00000350708
ENSP00000219431	ENSP00000356652
ENSP00000219431	ENSP00000379339
ENSP00000219431	ENSP00000393393
ENSP00000219439	ENSP00000256383
ENSP00000219439	ENSP00000261875
ENSP00000219439	ENSP00000263208
ENSP00000219439	ENSP00000266126
ENSP00000219439	ENSP00000273783
ENSP00000219439	ENSP00000315925
ENSP00000219439	ENSP00000330408
ENSP00000219439	ENSP00000353575

ENSP00000219439	ENSP00000355308
ENSP00000219439	ENSP00000362869
ENSP00000219439	ENSP00000364119
ENSP00000219439	ENSP00000370607
ENSP00000219439	ENSP00000394869
ENSP00000219454	ENSP00000352271
ENSP00000219473	ENSP00000224073
ENSP00000219473	ENSP00000225504
ENSP00000219473	ENSP00000225728
ENSP00000219473	ENSP00000226796
ENSP00000219473	ENSP00000228916
ENSP00000219473	ENSP00000234170
ENSP00000219473	ENSP00000234827
ENSP00000219473	ENSP00000236051
ENSP00000219473	ENSP00000240922
ENSP00000219473	ENSP00000243563
ENSP00000219473	ENSP00000245838
ENSP00000219473	ENSP00000247219
ENSP00000219473	ENSP00000247843
ENSP00000219473	ENSP00000248566
ENSP00000219473	ENSP00000251871
ENSP00000219473	ENSP00000254193
ENSP00000219473	ENSP00000254940
ENSP00000219473	ENSP00000256379
ENSP00000219473	ENSP00000256442
ENSP00000219473	ENSP00000257829
ENSP00000219473	ENSP00000261758
ENSP00000219473	ENSP00000261845
ENSP00000219473	ENSP00000262455
ENSP00000219473	ENSP00000262518
ENSP00000219473	ENSP00000262544
ENSP00000219473	ENSP00000262803
ENSP00000219473	ENSP00000262812
ENSP00000219473	ENSP00000262966
ENSP00000219473	ENSP00000263205
ENSP00000219473	ENSP00000263390
ENSP00000219473	ENSP00000263657
ENSP00000219473	ENSP00000263710
ENSP00000219473	ENSP00000264028
ENSP00000219473	ENSP00000264160
ENSP00000219473	ENSP00000264515
ENSP00000219473	ENSP00000264951
ENSP00000219473	ENSP00000265073
ENSP00000219473	ENSP00000265175
ENSP00000219473	ENSP00000265857
ENSP00000219473	ENSP00000267064

ENSP00000219473	ENSP00000268482
ENSP00000219473	ENSP00000273398
ENSP00000219473	ENSP00000278572
ENSP00000219473	ENSP00000281928
ENSP00000219473	ENSP00000282007
ENSP00000219473	ENSP00000282441
ENSP00000219473	ENSP00000283882
ENSP00000219473	ENSP00000284719
ENSP00000219473	ENSP00000285021
ENSP00000219473	ENSP00000286317
ENSP00000219473	ENSP00000287878
ENSP00000219473	ENSP00000288985
ENSP00000219473	ENSP00000290663
ENSP00000219473	ENSP00000293406
ENSP00000219473	ENSP00000293777
ENSP00000219473	ENSP00000294119
ENSP00000219473	ENSP00000296328
ENSP00000219473	ENSP00000296577
ENSP00000219473	ENSP00000296755
ENSP00000219473	ENSP00000298532
ENSP00000219473	ENSP00000298746
ENSP00000219473	ENSP00000299138
ENSP00000219473	ENSP00000300231
ENSP00000219473	ENSP00000304593
ENSP00000219473	ENSP00000305682
ENSP00000219473	ENSP00000306124
ENSP00000219473	ENSP00000307684
ENSP00000219473	ENSP00000307863
ENSP00000219473	ENSP00000308944
ENSP00000219473	ENSP00000310129
ENSP00000219473	ENSP00000311005
ENSP00000219473	ENSP00000312066
ENSP00000219473	ENSP00000312735
ENSP00000219473	ENSP00000314615
ENSP00000219473	ENSP00000314949
ENSP00000219473	ENSP00000316879
ENSP00000219473	ENSP00000317992
ENSP00000219473	ENSP00000320309
ENSP00000219473	ENSP00000320949
ENSP00000219473	ENSP00000322608
ENSP00000219473	ENSP00000323720
ENSP00000219473	ENSP00000324560
ENSP00000219473	ENSP00000325448
ENSP00000219473	ENSP00000325612
ENSP00000219473	ENSP00000326128
ENSP00000219473	ENSP00000326767

ENSP00000219473	ENSP00000326806
ENSP00000219473	ENSP00000327179
ENSP00000219473	ENSP00000327716
ENSP00000219473	ENSP00000329419
ENSP00000219473	ENSP00000331487
ENSP00000219473	ENSP00000331897
ENSP00000219473	ENSP00000331907
ENSP00000219473	ENSP00000332103
ENSP00000219473	ENSP00000334675
ENSP00000219473	ENSP00000336606
ENSP00000219473	ENSP00000336842
ENSP00000219473	ENSP00000338788
ENSP00000219473	ENSP00000338862
ENSP00000219473	ENSP00000343535
ENSP00000219473	ENSP00000343557
ENSP00000219473	ENSP00000345530
ENSP00000219473	ENSP00000347883
ENSP00000219473	ENSP00000348089
ENSP00000219473	ENSP00000348442
ENSP00000219473	ENSP00000348577
ENSP00000219473	ENSP00000350708
ENSP00000219473	ENSP00000352738
ENSP00000219473	ENSP00000353741
ENSP00000219473	ENSP00000353998
ENSP00000219473	ENSP00000354061
ENSP00000219473	ENSP00000354125
ENSP00000219473	ENSP00000354558
ENSP00000219473	ENSP00000354927
ENSP00000219473	ENSP00000355051
ENSP00000219473	ENSP00000355205
ENSP00000219473	ENSP00000355231
ENSP00000219473	ENSP00000355890
ENSP00000219473	ENSP00000356520
ENSP00000219473	ENSP00000357047
ENSP00000219473	ENSP00000357887
ENSP00000219473	ENSP00000357907
ENSP00000219473	ENSP00000358081
ENSP00000219473	ENSP00000358140
ENSP00000219473	ENSP00000358478
ENSP00000219473	ENSP00000358563
ENSP00000219473	ENSP00000359345
ENSP00000219473	ENSP00000359552
ENSP00000219473	ENSP00000360515
ENSP00000219473	ENSP00000360532
ENSP00000219473	ENSP00000361465
ENSP00000219473	ENSP00000361577

ENSP00000219473	ENSP00000361699
ENSP00000219473	ENSP00000362306
ENSP00000219473	ENSP00000362936
ENSP00000219473	ENSP00000362948
ENSP00000219473	ENSP00000365005
ENSP00000219473	ENSP00000366607
ENSP00000219473	ENSP00000366974
ENSP00000219473	ENSP00000367029
ENSP00000219473	ENSP00000367498
ENSP00000219473	ENSP00000374213
ENSP00000219473	ENSP00000376268
ENSP00000219473	ENSP00000377655
ENSP00000219473	ENSP00000377969
ENSP00000219473	ENSP00000379228
ENSP00000219473	ENSP00000380129
ENSP00000219473	ENSP00000380460
ENSP00000219473	ENSP00000380982
ENSP00000219473	ENSP00000382423
ENSP00000219473	ENSP00000384048
ENSP00000219473	ENSP00000384211
ENSP00000219473	ENSP00000388872
ENSP00000219473	ENSP00000391774
ENSP00000219473	ENSP00000392617
ENSP00000219473	ENSP00000396937
ENSP00000219473	ENSP00000402527
ENSP00000219473	ENSP00000406293
ENSP00000219473	ENSP00000412566
ENSP00000219473	ENSP00000417281
ENSP00000219473	ENSP00000418734
ENSP00000219476	ENSP00000220849
ENSP00000219476	ENSP00000223369
ENSP00000219476	ENSP00000227507
ENSP00000219476	ENSP00000228872
ENSP00000219476	ENSP00000246166
ENSP00000219476	ENSP00000247970
ENSP00000219476	ENSP00000248673
ENSP00000219476	ENSP00000255465
ENSP00000219476	ENSP00000262039
ENSP00000219476	ENSP00000262320
ENSP00000219476	ENSP00000262643
ENSP00000219476	ENSP00000264335
ENSP00000219476	ENSP00000265970
ENSP00000219476	ENSP00000274255
ENSP00000219476	ENSP00000278616
ENSP00000219476	ENSP00000281043
ENSP00000219476	ENSP00000282388

ENSP00000219476	ENSP00000297316
ENSP00000219476	ENSP00000298552
ENSP00000219476	ENSP00000298630
ENSP00000219476	ENSP00000303423
ENSP00000219476	ENSP00000306100
ENSP00000219476	ENSP00000313034
ENSP00000219476	ENSP00000313420
ENSP00000219476	ENSP00000330098
ENSP00000219476	ENSP00000331152
ENSP00000219476	ENSP00000339191
ENSP00000219476	ENSP00000341189
ENSP00000219476	ENSP00000343741
ENSP00000219476	ENSP00000347733
ENSP00000219476	ENSP00000348813
ENSP00000219476	ENSP00000349078
ENSP00000219476	ENSP00000350785
ENSP00000219476	ENSP00000351490
ENSP00000219476	ENSP00000352121
ENSP00000219476	ENSP00000353698
ENSP00000219476	ENSP00000354558
ENSP00000219476	ENSP00000359567
ENSP00000219476	ENSP00000381045
ENSP00000219476	ENSP00000402515
ENSP00000219476	ENSP00000408020
ENSP00000219479	ENSP00000241052
ENSP00000219479	ENSP00000242576
ENSP00000219479	ENSP00000252487
ENSP00000219479	ENSP00000254942
ENSP00000219479	ENSP00000301585
ENSP00000219479	ENSP00000304908
ENSP00000219479	ENSP00000312735
ENSP00000219479	ENSP00000368438
ENSP00000219479	ENSP00000369456
ENSP00000219479	ENSP00000369757
ENSP00000219548	ENSP00000223029
ENSP00000219548	ENSP00000230882
ENSP00000219548	ENSP00000242057
ENSP00000219548	ENSP00000244769
ENSP00000219548	ENSP00000245816
ENSP00000219548	ENSP00000246062
ENSP00000219548	ENSP00000254942
ENSP00000219548	ENSP00000255631
ENSP00000219548	ENSP00000260818
ENSP00000219548	ENSP00000262375
ENSP00000219548	ENSP00000263202
ENSP00000219548	ENSP00000263734

ENSP00000219548	ENSP00000266000
ENSP00000219548	ENSP00000267415
ENSP00000219548	ENSP00000272638
ENSP00000219548	ENSP00000274255
ENSP00000219548	ENSP00000276603
ENSP00000219548	ENSP00000284981
ENSP00000219548	ENSP00000292211
ENSP00000219548	ENSP00000294339
ENSP00000219548	ENSP00000296474
ENSP00000219548	ENSP00000296509
ENSP00000219548	ENSP00000298910
ENSP00000219548	ENSP00000303507
ENSP00000219548	ENSP00000306449
ENSP00000219548	ENSP00000309474
ENSP00000219548	ENSP00000309572
ENSP00000219548	ENSP00000319788
ENSP00000219548	ENSP00000320758
ENSP00000219548	ENSP00000321617
ENSP00000219548	ENSP00000323275
ENSP00000219548	ENSP00000326391
ENSP00000219548	ENSP00000326813
ENSP00000219548	ENSP00000331057
ENSP00000219548	ENSP00000331201
ENSP00000219548	ENSP00000331487
ENSP00000219548	ENSP00000334061
ENSP00000219548	ENSP00000337088
ENSP00000219548	ENSP00000338345
ENSP00000219548	ENSP00000338814
ENSP00000219548	ENSP00000340019
ENSP00000219548	ENSP00000340278
ENSP00000219548	ENSP00000340305
ENSP00000219548	ENSP00000340820
ENSP00000219548	ENSP00000341551
ENSP00000219548	ENSP00000344874
ENSP00000219548	ENSP00000345464
ENSP00000219548	ENSP00000346240
ENSP00000219548	ENSP00000350249
ENSP00000219548	ENSP00000351908
ENSP00000219548	ENSP00000352514
ENSP00000219548	ENSP00000355865
ENSP00000219548	ENSP00000356489
ENSP00000219548	ENSP00000359727
ENSP00000219548	ENSP00000360644
ENSP00000219548	ENSP00000363089
ENSP00000219548	ENSP00000377496
ENSP00000219548	ENSP00000380019

ENSP00000219548	ENSP00000380460
ENSP00000219551	ENSP00000265354
ENSP00000219551	ENSP00000267101
ENSP00000219551	ENSP00000271555
ENSP00000219551	ENSP00000277541
ENSP00000219551	ENSP00000302216
ENSP00000219551	ENSP00000304676
ENSP00000219551	ENSP00000309871
ENSP00000219551	ENSP00000343785
ENSP00000219551	ENSP00000363055
ENSP00000219551	ENSP00000367972
ENSP00000219551	ENSP00000369042
ENSP00000219596	ENSP00000297135
ENSP00000219599	ENSP00000223127
ENSP00000219599	ENSP00000276603
ENSP00000219599	ENSP00000351575
ENSP00000219599	ENSP00000376758
ENSP00000219689	ENSP00000291688
ENSP00000219689	ENSP00000321449
ENSP00000219689	ENSP00000368438
ENSP00000219689	ENSP00000380460
ENSP00000219700	ENSP00000229416
ENSP00000219700	ENSP00000241052
ENSP00000219700	ENSP00000251038
ENSP00000219700	ENSP00000265304
ENSP00000219700	ENSP00000284981
ENSP00000219700	ENSP00000286627
ENSP00000219700	ENSP00000313921
ENSP00000219700	ENSP00000333433
ENSP00000219700	ENSP00000381863
ENSP00000219789	ENSP00000227471
ENSP00000219789	ENSP00000251038
ENSP00000219789	ENSP00000256854
ENSP00000219789	ENSP00000260257
ENSP00000219789	ENSP00000261386
ENSP00000219789	ENSP00000264161
ENSP00000219789	ENSP00000264167
ENSP00000219789	ENSP00000266095
ENSP00000219789	ENSP00000274680
ENSP00000219789	ENSP00000281828
ENSP00000219789	ENSP00000288937
ENSP00000219789	ENSP00000292199
ENSP00000219789	ENSP00000311505
ENSP00000219789	ENSP00000314440
ENSP00000219789	ENSP00000315693
ENSP00000219789	ENSP00000320309

ENSP00000219789	ENSP00000322316
ENSP00000219789	ENSP00000340093
ENSP00000219789	ENSP00000345530
ENSP00000219789	ENSP00000346037
ENSP00000219789	ENSP00000353826
ENSP00000219789	ENSP00000357998
ENSP00000219789	ENSP00000362306
ENSP00000219789	ENSP00000367498
ENSP00000219789	ENSP00000370372
ENSP00000219789	ENSP00000379228
ENSP00000219789	ENSP00000379568
ENSP00000219789	ENSP00000381891
ENSP00000219905	ENSP00000254029
ENSP00000219905	ENSP00000262904
ENSP00000219905	ENSP00000269397
ENSP00000219905	ENSP00000308750
ENSP00000219905	ENSP00000351490
ENSP00000219905	ENSP00000358862
ENSP00000219905	ENSP00000368686
ENSP00000219905	ENSP00000419494
ENSP00000220058	ENSP00000222286
ENSP00000220058	ENSP00000229214
ENSP00000220058	ENSP00000261211
ENSP00000220058	ENSP00000263629
ENSP00000220058	ENSP00000334188
ENSP00000220058	ENSP00000388872
ENSP00000220062	ENSP00000340954
ENSP00000220062	ENSP00000341551
ENSP00000220478	ENSP00000327145
ENSP00000220496	ENSP00000222969
ENSP00000220496	ENSP00000227524
ENSP00000220496	ENSP00000254193
ENSP00000220496	ENSP00000258415
ENSP00000220496	ENSP00000261531
ENSP00000220496	ENSP00000270538
ENSP00000220496	ENSP00000286621
ENSP00000220496	ENSP00000294053
ENSP00000220496	ENSP00000301764
ENSP00000220496	ENSP00000304350
ENSP00000220496	ENSP00000305790
ENSP00000220496	ENSP00000307525
ENSP00000220496	ENSP00000314499
ENSP00000220496	ENSP00000335321
ENSP00000220496	ENSP00000340879
ENSP00000220496	ENSP00000342374
ENSP00000220496	ENSP00000351137

ENSP00000220496	ENSP00000357998
ENSP00000220496	ENSP00000360532
ENSP00000220496	ENSP00000362110
ENSP00000220496	ENSP00000366557
ENSP00000220496	ENSP00000368565
ENSP00000220509	ENSP00000231368
ENSP00000220509	ENSP00000249923
ENSP00000220509	ENSP00000251038
ENSP00000220509	ENSP00000253083
ENSP00000220509	ENSP00000253699
ENSP00000220509	ENSP00000255465
ENSP00000220509	ENSP00000258301
ENSP00000220509	ENSP00000262215
ENSP00000220509	ENSP00000263688
ENSP00000220509	ENSP00000265056
ENSP00000220509	ENSP00000273398
ENSP00000220509	ENSP00000300245
ENSP00000220509	ENSP00000305255
ENSP00000220509	ENSP00000309457
ENSP00000220509	ENSP00000311200
ENSP00000220509	ENSP00000312870
ENSP00000220509	ENSP00000313420
ENSP00000220509	ENSP00000326534
ENSP00000220509	ENSP00000344055
ENSP00000220509	ENSP00000348455
ENSP00000220509	ENSP00000354558
ENSP00000220509	ENSP00000360252
ENSP00000220509	ENSP00000360268
ENSP00000220509	ENSP00000367265
ENSP00000220509	ENSP00000367406
ENSP00000220509	ENSP00000369810
ENSP00000220509	ENSP00000390783
ENSP00000220509	ENSP00000402515
ENSP00000220509	ENSP00000418661
ENSP00000220514	ENSP00000266732
ENSP00000220514	ENSP00000286186
ENSP00000220514	ENSP00000288602
ENSP00000220514	ENSP00000323880
ENSP00000220514	ENSP00000356595
ENSP00000220514	ENSP00000357051
ENSP00000220531	ENSP00000240079
ENSP00000220531	ENSP00000261597
ENSP00000220531	ENSP00000281172
ENSP00000220531	ENSP00000285116
ENSP00000220531	ENSP00000316109
ENSP00000220531	ENSP00000318128

ENSP00000220531	ENSP00000336856
ENSP00000220531	ENSP00000341680
ENSP00000220531	ENSP00000353564
ENSP00000220531	ENSP00000357674
ENSP00000220531	ENSP00000361048
ENSP00000220531	ENSP00000365401
ENSP00000220531	ENSP00000380598
ENSP00000220562	ENSP00000244007
ENSP00000220562	ENSP00000300574
ENSP00000220584	ENSP00000231487
ENSP00000220584	ENSP00000254521
ENSP00000220584	ENSP00000256319
ENSP00000220584	ENSP00000258415
ENSP00000220584	ENSP00000260129
ENSP00000220584	ENSP00000261507
ENSP00000220584	ENSP00000264027
ENSP00000220584	ENSP00000272163
ENSP00000220584	ENSP00000285949
ENSP00000220584	ENSP00000303992
ENSP00000220584	ENSP00000310721
ENSP00000220584	ENSP00000315626
ENSP00000220584	ENSP00000320604
ENSP00000220584	ENSP00000325425
ENSP00000220584	ENSP00000325690
ENSP00000220584	ENSP00000330812
ENSP00000220584	ENSP00000341662
ENSP00000220584	ENSP00000343657
ENSP00000220584	ENSP00000345972
ENSP00000220584	ENSP00000348380
ENSP00000220584	ENSP00000358605
ENSP00000220584	ENSP00000360918
ENSP00000220584	ENSP00000367079
ENSP00000220584	ENSP00000387019
ENSP00000220597	ENSP00000274376
ENSP00000220597	ENSP00000340944
ENSP00000220597	ENSP00000364898
ENSP00000220616	ENSP00000263816
ENSP00000220616	ENSP00000286091
ENSP00000220616	ENSP00000299767
ENSP00000220659	ENSP00000221232
ENSP00000220659	ENSP00000222284
ENSP00000220659	ENSP00000225728
ENSP00000220659	ENSP00000228955
ENSP00000220659	ENSP00000244763
ENSP00000220659	ENSP00000247970
ENSP00000220659	ENSP00000248553

ENSP00000220659	ENSP00000253003
ENSP00000220659	ENSP00000255305
ENSP00000220659	ENSP00000262133
ENSP00000220659	ENSP00000262982
ENSP00000220659	ENSP00000263974
ENSP00000220659	ENSP00000265073
ENSP00000220659	ENSP00000265388
ENSP00000220659	ENSP00000266263
ENSP00000220659	ENSP00000272645
ENSP00000220659	ENSP00000276072
ENSP00000220659	ENSP00000282003
ENSP00000220659	ENSP00000285398
ENSP00000220659	ENSP00000287295
ENSP00000220659	ENSP00000291386
ENSP00000220659	ENSP00000293195
ENSP00000220659	ENSP00000298281
ENSP00000220659	ENSP00000298532
ENSP00000220659	ENSP00000299424
ENSP00000220659	ENSP00000301788
ENSP00000220659	ENSP00000302790
ENSP00000220659	ENSP00000304704
ENSP00000220659	ENSP00000308944
ENSP00000220659	ENSP00000310842
ENSP00000220659	ENSP00000312735
ENSP00000220659	ENSP00000313953
ENSP00000220659	ENSP00000315791
ENSP00000220659	ENSP00000317992
ENSP00000220659	ENSP00000318604
ENSP00000220659	ENSP00000323720
ENSP00000220659	ENSP00000324124
ENSP00000220659	ENSP00000325002
ENSP00000220659	ENSP00000337405
ENSP00000220659	ENSP00000342889
ENSP00000220659	ENSP00000346453
ENSP00000220659	ENSP00000347345
ENSP00000220659	ENSP00000348010
ENSP00000220659	ENSP00000351575
ENSP00000220659	ENSP00000354125
ENSP00000220659	ENSP00000354742
ENSP00000220659	ENSP00000356405
ENSP00000220659	ENSP00000358140
ENSP00000220659	ENSP00000358716
ENSP00000220659	ENSP00000359211
ENSP00000220659	ENSP00000359290
ENSP00000220659	ENSP00000359484
ENSP00000220659	ENSP00000360644

ENSP00000220659	ENSP00000364119
ENSP00000220659	ENSP00000364289
ENSP00000220659	ENSP00000366748
ENSP00000220659	ENSP00000370258
ENSP00000220659	ENSP00000378736
ENSP00000220659	ENSP00000379372
ENSP00000220659	ENSP00000384597
ENSP00000220659	ENSP00000386992
ENSP00000220659	ENSP00000403852
ENSP00000220659	ENSP00000417229
ENSP00000220669	ENSP00000238789
ENSP00000220669	ENSP00000248633
ENSP00000220669	ENSP00000251038
ENSP00000220669	ENSP00000253107
ENSP00000220669	ENSP00000276692
ENSP00000220669	ENSP00000306080
ENSP00000220669	ENSP00000320885
ENSP00000220669	ENSP00000331487
ENSP00000220669	ENSP00000358140
ENSP00000220669	ENSP00000363338
ENSP00000220669	ENSP00000370258
ENSP00000220669	ENSP00000384048
ENSP00000220676	ENSP00000222115
ENSP00000220676	ENSP00000227758
ENSP00000220676	ENSP00000242285
ENSP00000220676	ENSP00000256658
ENSP00000220676	ENSP00000257430
ENSP00000220676	ENSP00000271628
ENSP00000220676	ENSP00000300249
ENSP00000220676	ENSP00000301788
ENSP00000220676	ENSP00000349298
ENSP00000220676	ENSP00000350199
ENSP00000220676	ENSP00000386394
ENSP00000220676	ENSP00000393596
ENSP00000220676	ENSP00000402240
ENSP00000220751	ENSP00000227758
ENSP00000220751	ENSP00000234590
ENSP00000220751	ENSP00000252543
ENSP00000220751	ENSP00000254691
ENSP00000220751	ENSP00000259808
ENSP00000220751	ENSP00000300303
ENSP00000220751	ENSP00000300589
ENSP00000220751	ENSP00000309830
ENSP00000220751	ENSP00000311028
ENSP00000220751	ENSP00000315112
ENSP00000220751	ENSP00000335657

ENSP00000220751	ENSP00000337127
ENSP00000220751	ENSP00000341551
ENSP00000220751	ENSP00000356319
ENSP00000220751	ENSP00000358335
ENSP00000220751	ENSP00000358997
ENSP00000220751	ENSP00000363089
ENSP00000220751	ENSP00000364858
ENSP00000220751	ENSP00000368698
ENSP00000220751	ENSP00000376309
ENSP00000220751	ENSP00000401980
ENSP00000220763	ENSP00000338413
ENSP00000220763	ENSP00000367835
ENSP00000220764	ENSP00000238647
ENSP00000220764	ENSP00000264029
ENSP00000220764	ENSP00000331201
ENSP00000220764	ENSP00000344936
ENSP00000220849	ENSP00000228825
ENSP00000220849	ENSP00000229251
ENSP00000220849	ENSP00000237281
ENSP00000220849	ENSP00000248342
ENSP00000220849	ENSP00000253108
ENSP00000220849	ENSP00000253363
ENSP00000220849	ENSP00000256442
ENSP00000220849	ENSP00000259569
ENSP00000220849	ENSP00000261531
ENSP00000220849	ENSP00000261908
ENSP00000220849	ENSP00000263734
ENSP00000220849	ENSP00000265753
ENSP00000220849	ENSP00000268058
ENSP00000220849	ENSP00000271628
ENSP00000220849	ENSP00000278616
ENSP00000220849	ENSP00000281708
ENSP00000220849	ENSP00000283195
ENSP00000220849	ENSP00000290795
ENSP00000220849	ENSP00000291576
ENSP00000220849	ENSP00000296930
ENSP00000220849	ENSP00000297185
ENSP00000220849	ENSP00000299259
ENSP00000220849	ENSP00000302768
ENSP00000220849	ENSP00000304102
ENSP00000220849	ENSP00000304822
ENSP00000220849	ENSP00000310040
ENSP00000220849	ENSP00000310129
ENSP00000220849	ENSP00000316476
ENSP00000220849	ENSP00000316879
ENSP00000220849	ENSP00000318195

ENSP00000220849	ENSP00000319977
ENSP00000220849	ENSP00000326806
ENSP00000220849	ENSP00000328103
ENSP00000220849	ENSP00000329466
ENSP00000220849	ENSP00000329715
ENSP00000220849	ENSP00000332444
ENSP00000220849	ENSP00000340281
ENSP00000220849	ENSP00000346340
ENSP00000220849	ENSP00000346453
ENSP00000220849	ENSP00000347206
ENSP00000220849	ENSP00000348442
ENSP00000220849	ENSP00000354125
ENSP00000220849	ENSP00000355370
ENSP00000220849	ENSP00000358140
ENSP00000220849	ENSP00000361949
ENSP00000220849	ENSP00000362688
ENSP00000220849	ENSP00000362900
ENSP00000220849	ENSP00000364847
ENSP00000220849	ENSP00000365175
ENSP00000220849	ENSP00000376309
ENSP00000220849	ENSP00000381293
ENSP00000220849	ENSP00000381891
ENSP00000220849	ENSP00000387477
ENSP00000220849	ENSP00000401371
ENSP00000220849	ENSP00000415363
ENSP00000220849	ENSP00000416892
ENSP00000220853	ENSP00000243253
ENSP00000220853	ENSP00000245046
ENSP00000220853	ENSP00000253457
ENSP00000220853	ENSP00000267750
ENSP00000220853	ENSP00000277010
ENSP00000220853	ENSP00000283351
ENSP00000220853	ENSP00000301281
ENSP00000220853	ENSP00000302176
ENSP00000220853	ENSP00000327716
ENSP00000220853	ENSP00000354522
ENSP00000220853	ENSP00000360645
ENSP00000220853	ENSP00000361845
ENSP00000220853	ENSP00000420608
ENSP00000220913	ENSP00000265717
ENSP00000220913	ENSP00000269216
ENSP00000220913	ENSP00000291688
ENSP00000220913	ENSP00000307684
ENSP00000220913	ENSP00000316114
ENSP00000220913	ENSP00000324560
ENSP00000220913	ENSP00000340954

ENSP00000220913	ENSP00000345681
ENSP00000220913	ENSP00000353458
ENSP00000220913	ENSP00000379065
ENSP00000220913	ENSP00000379607
ENSP00000220913	ENSP00000384434
ENSP00000220913	ENSP00000396749
ENSP00000220913	ENSP00000400312
ENSP00000220913	ENSP00000417281
ENSP00000220959	ENSP00000248553
ENSP00000220959	ENSP00000260810
ENSP00000220959	ENSP00000261531
ENSP00000220959	ENSP00000261942
ENSP00000220959	ENSP00000262643
ENSP00000220959	ENSP00000263390
ENSP00000220959	ENSP00000265192
ENSP00000220959	ENSP00000265849
ENSP00000220959	ENSP00000268957
ENSP00000220959	ENSP00000290158
ENSP00000220959	ENSP00000294008
ENSP00000220959	ENSP00000294119
ENSP00000220959	ENSP00000296328
ENSP00000220959	ENSP00000297185
ENSP00000220959	ENSP00000297261
ENSP00000220959	ENSP00000301764
ENSP00000220959	ENSP00000302530
ENSP00000220959	ENSP00000302768
ENSP00000220959	ENSP00000307491
ENSP00000220959	ENSP00000317636
ENSP00000220959	ENSP00000325120
ENSP00000220959	ENSP00000326767
ENSP00000220959	ENSP00000329466
ENSP00000220959	ENSP00000331057
ENSP00000220959	ENSP00000333873
ENSP00000220959	ENSP00000337340
ENSP00000220959	ENSP00000341024
ENSP00000220959	ENSP00000343701
ENSP00000220959	ENSP00000348168
ENSP00000220959	ENSP00000348442
ENSP00000220959	ENSP00000348784
ENSP00000220959	ENSP00000348877
ENSP00000220959	ENSP00000349640
ENSP00000220959	ENSP00000361990
ENSP00000220959	ENSP00000364847
ENSP00000220959	ENSP00000379144
ENSP00000220959	ENSP00000386165
ENSP00000220959	ENSP00000396068

ENSP00000220959	ENSP00000399968
ENSP00000220959	ENSP00000406490
ENSP00000221114	ENSP00000259632
ENSP00000221114	ENSP00000300087
ENSP00000221114	ENSP00000346067
ENSP00000221114	ENSP00000408910
ENSP00000221114	ENSP00000414906
ENSP00000221132	ENSP00000239849
ENSP00000221132	ENSP00000259808
ENSP00000221132	ENSP00000286186
ENSP00000221132	ENSP00000301838
ENSP00000221132	ENSP00000310933
ENSP00000221132	ENSP00000340278
ENSP00000221132	ENSP00000341268
ENSP00000221132	ENSP00000341692
ENSP00000221132	ENSP00000374455
ENSP00000221232	ENSP00000225916
ENSP00000221232	ENSP00000229195
ENSP00000221232	ENSP00000233078
ENSP00000221232	ENSP00000240159
ENSP00000221232	ENSP00000247219
ENSP00000221232	ENSP00000251038
ENSP00000221232	ENSP00000252115
ENSP00000221232	ENSP00000254900
ENSP00000221232	ENSP00000256897
ENSP00000221232	ENSP00000260210
ENSP00000221232	ENSP00000261741
ENSP00000221232	ENSP00000261951
ENSP00000221232	ENSP00000262367
ENSP00000221232	ENSP00000263702
ENSP00000221232	ENSP00000263857
ENSP00000221232	ENSP00000263974
ENSP00000221232	ENSP00000264157
ENSP00000221232	ENSP00000264389
ENSP00000221232	ENSP00000268957
ENSP00000221232	ENSP00000273064
ENSP00000221232	ENSP00000276072
ENSP00000221232	ENSP00000280701
ENSP00000221232	ENSP00000285896
ENSP00000221232	ENSP00000288774
ENSP00000221232	ENSP00000290429
ENSP00000221232	ENSP00000291688
ENSP00000221232	ENSP00000294168
ENSP00000221232	ENSP00000295926
ENSP00000221232	ENSP00000295989
ENSP00000221232	ENSP00000296328

ENSP00000221232	ENSP00000296509
ENSP00000221232	ENSP00000302160
ENSP00000221232	ENSP00000302640
ENSP00000221232	ENSP00000313350
ENSP00000221232	ENSP00000314949
ENSP00000221232	ENSP00000318374
ENSP00000221232	ENSP00000320566
ENSP00000221232	ENSP00000320949
ENSP00000221232	ENSP00000322242
ENSP00000221232	ENSP00000325376
ENSP00000221232	ENSP00000326261
ENSP00000221232	ENSP00000329029
ENSP00000221232	ENSP00000331817
ENSP00000221232	ENSP00000337839
ENSP00000221232	ENSP00000339095
ENSP00000221232	ENSP00000339191
ENSP00000221232	ENSP00000340271
ENSP00000221232	ENSP00000344537
ENSP00000221232	ENSP00000354673
ENSP00000221232	ENSP00000355051
ENSP00000221232	ENSP00000358854
ENSP00000221232	ENSP00000359531
ENSP00000221232	ENSP00000359939
ENSP00000221232	ENSP00000360473
ENSP00000221232	ENSP00000360532
ENSP00000221232	ENSP00000361287
ENSP00000221232	ENSP00000366135
ENSP00000221232	ENSP00000369965
ENSP00000221232	ENSP00000371475
ENSP00000221232	ENSP00000372023
ENSP00000221232	ENSP00000374529
ENSP00000221232	ENSP00000380460
ENSP00000221232	ENSP00000387170
ENSP00000221232	ENSP00000388872
ENSP00000221232	ENSP00000391944
ENSP00000221249	ENSP00000255389
ENSP00000221249	ENSP00000259569
ENSP00000221249	ENSP00000261015
ENSP00000221249	ENSP00000318351
ENSP00000221249	ENSP00000346453
ENSP00000221249	ENSP00000346725
ENSP00000221249	ENSP00000353622
ENSP00000221249	ENSP00000361465
ENSP00000221249	ENSP00000368565
ENSP00000221249	ENSP00000403852
ENSP00000221265	ENSP00000221859

ENSP00000221265	ENSP00000224140
ENSP00000221265	ENSP00000225504
ENSP00000221265	ENSP00000240079
ENSP00000221265	ENSP00000248566
ENSP00000221265	ENSP00000251038
ENSP00000221265	ENSP00000253107
ENSP00000221265	ENSP00000256398
ENSP00000221265	ENSP00000261601
ENSP00000221265	ENSP00000261951
ENSP00000221265	ENSP00000262803
ENSP00000221265	ENSP00000265354
ENSP00000221265	ENSP00000265562
ENSP00000221265	ENSP00000273077
ENSP00000221265	ENSP00000276590
ENSP00000221265	ENSP00000278412
ENSP00000221265	ENSP00000280614
ENSP00000221265	ENSP00000286835
ENSP00000221265	ENSP00000289013
ENSP00000221265	ENSP00000296292
ENSP00000221265	ENSP00000296786
ENSP00000221265	ENSP00000298937
ENSP00000221265	ENSP00000299424
ENSP00000221265	ENSP00000299543
ENSP00000221265	ENSP00000299601
ENSP00000221265	ENSP00000300648
ENSP00000221265	ENSP00000300738
ENSP00000221265	ENSP00000301280
ENSP00000221265	ENSP00000303315
ENSP00000221265	ENSP00000307387
ENSP00000221265	ENSP00000309142
ENSP00000221265	ENSP00000314776
ENSP00000221265	ENSP00000314949
ENSP00000221265	ENSP00000319104
ENSP00000221265	ENSP00000329715
ENSP00000221265	ENSP00000329933
ENSP00000221265	ENSP00000339933
ENSP00000221265	ENSP00000340823
ENSP00000221265	ENSP00000340944
ENSP00000221265	ENSP00000343190
ENSP00000221265	ENSP00000343742
ENSP00000221265	ENSP00000350967
ENSP00000221265	ENSP00000351596
ENSP00000221265	ENSP00000354977
ENSP00000221265	ENSP00000355013
ENSP00000221265	ENSP00000356146
ENSP00000221265	ENSP00000356405

ENSP00000221265	ENSP00000357602
ENSP00000221265	ENSP00000360116
ENSP00000221265	ENSP00000360515
ENSP00000221265	ENSP00000362334
ENSP00000221265	ENSP00000363779
ENSP00000221265	ENSP00000365025
ENSP00000221265	ENSP00000366135
ENSP00000221265	ENSP00000366746
ENSP00000221265	ENSP00000367029
ENSP00000221265	ENSP00000369695
ENSP00000221265	ENSP00000374280
ENSP00000221265	ENSP00000376097
ENSP00000221265	ENSP00000379217
ENSP00000221265	ENSP00000386759
ENSP00000221265	ENSP00000403852
ENSP00000221403	ENSP00000222330
ENSP00000221403	ENSP00000231487
ENSP00000221403	ENSP00000265073
ENSP00000221403	ENSP00000280871
ENSP00000221403	ENSP00000282892
ENSP00000221403	ENSP00000287878
ENSP00000221403	ENSP00000298746
ENSP00000221403	ENSP00000301364
ENSP00000221403	ENSP00000304350
ENSP00000221403	ENSP00000307684
ENSP00000221403	ENSP00000318318
ENSP00000221403	ENSP00000332444
ENSP00000221403	ENSP00000336842
ENSP00000221403	ENSP00000349577
ENSP00000221403	ENSP00000357907
ENSP00000221403	ENSP00000358563
ENSP00000221403	ENSP00000361982
ENSP00000221403	ENSP00000361990
ENSP00000221403	ENSP00000366620
ENSP00000221403	ENSP00000368119
ENSP00000221403	ENSP00000420608
ENSP00000221419	ENSP00000225983
ENSP00000221419	ENSP00000256474
ENSP00000221419	ENSP00000262982
ENSP00000221419	ENSP00000264731
ENSP00000221419	ENSP00000282486
ENSP00000221419	ENSP00000298852
ENSP00000221419	ENSP00000301740
ENSP00000221419	ENSP00000312318
ENSP00000221419	ENSP00000322016
ENSP00000221419	ENSP00000345530

ENSP00000221419	ENSP00000351947
ENSP00000221419	ENSP00000353735
ENSP00000221419	ENSP00000353920
ENSP00000221419	ENSP00000354522
ENSP00000221419	ENSP00000360876
ENSP00000221419	ENSP00000363373
ENSP00000221419	ENSP00000365439
ENSP00000221419	ENSP00000366237
ENSP00000221419	ENSP00000379607
ENSP00000221419	ENSP00000379847
ENSP00000221419	ENSP00000381293
ENSP00000221431	ENSP00000225298
ENSP00000221431	ENSP00000231487
ENSP00000221431	ENSP00000233078
ENSP00000221431	ENSP00000235521
ENSP00000221431	ENSP00000240185
ENSP00000221431	ENSP00000256854
ENSP00000221431	ENSP00000258499
ENSP00000221431	ENSP00000264161
ENSP00000221431	ENSP00000264705
ENSP00000221431	ENSP00000270223
ENSP00000221431	ENSP00000297338
ENSP00000221431	ENSP00000298746
ENSP00000221431	ENSP00000301585
ENSP00000221431	ENSP00000304102
ENSP00000221431	ENSP00000304592
ENSP00000221431	ENSP00000311766
ENSP00000221431	ENSP00000312356
ENSP00000221431	ENSP00000322016
ENSP00000221431	ENSP00000325448
ENSP00000221431	ENSP00000340019
ENSP00000221431	ENSP00000341243
ENSP00000221431	ENSP00000347495
ENSP00000221431	ENSP00000350512
ENSP00000221431	ENSP00000354347
ENSP00000221431	ENSP00000355890
ENSP00000221431	ENSP00000365775
ENSP00000221431	ENSP00000369335
ENSP00000221431	ENSP00000371888
ENSP00000221431	ENSP00000377914
ENSP00000221448	ENSP00000221494
ENSP00000221448	ENSP00000226796
ENSP00000221448	ENSP00000227524
ENSP00000221448	ENSP00000231487
ENSP00000221448	ENSP00000240304
ENSP00000221448	ENSP00000242719

ENSP00000221448	ENSP00000243563
ENSP00000221448	ENSP00000244204
ENSP00000221448	ENSP00000244520
ENSP00000221448	ENSP00000254193
ENSP00000221448	ENSP00000254942
ENSP00000221448	ENSP00000258428
ENSP00000221448	ENSP00000261531
ENSP00000221448	ENSP00000261776
ENSP00000221448	ENSP00000261973
ENSP00000221448	ENSP00000262803
ENSP00000221448	ENSP00000265073
ENSP00000221448	ENSP00000265334
ENSP00000221448	ENSP00000266735
ENSP00000221448	ENSP00000299543
ENSP00000221448	ENSP00000300291
ENSP00000221448	ENSP00000300413
ENSP00000221448	ENSP00000301740
ENSP00000221448	ENSP00000301764
ENSP00000221448	ENSP00000302768
ENSP00000221448	ENSP00000304350
ENSP00000221448	ENSP00000305790
ENSP00000221448	ENSP00000307863
ENSP00000221448	ENSP00000308533
ENSP00000221448	ENSP00000310448
ENSP00000221448	ENSP00000313674
ENSP00000221448	ENSP00000313829
ENSP00000221448	ENSP00000315859
ENSP00000221448	ENSP00000316879
ENSP00000221448	ENSP00000319169
ENSP00000221448	ENSP00000322016
ENSP00000221448	ENSP00000326806
ENSP00000221448	ENSP00000329466
ENSP00000221448	ENSP00000331879
ENSP00000221448	ENSP00000333666
ENSP00000221448	ENSP00000336524
ENSP00000221448	ENSP00000336712
ENSP00000221448	ENSP00000337405
ENSP00000221448	ENSP00000340281
ENSP00000221448	ENSP00000341551
ENSP00000221448	ENSP00000342374
ENSP00000221448	ENSP00000348010
ENSP00000221448	ENSP00000349748
ENSP00000221448	ENSP00000353564
ENSP00000221448	ENSP00000358374
ENSP00000221448	ENSP00000359206
ENSP00000221448	ENSP00000359958

ENSP00000221448	ENSP00000360147
ENSP00000221448	ENSP00000360464
ENSP00000221448	ENSP00000360532
ENSP00000221448	ENSP00000360876
ENSP00000221448	ENSP00000361577
ENSP00000221448	ENSP00000362110
ENSP00000221448	ENSP00000362820
ENSP00000221448	ENSP00000362900
ENSP00000221448	ENSP00000362936
ENSP00000221448	ENSP00000364289
ENSP00000221448	ENSP00000366607
ENSP00000221448	ENSP00000368801
ENSP00000221448	ENSP00000370083
ENSP00000221448	ENSP00000370119
ENSP00000221448	ENSP00000370377
ENSP00000221448	ENSP00000377262
ENSP00000221448	ENSP00000378350
ENSP00000221448	ENSP00000379093
ENSP00000221448	ENSP00000379607
ENSP00000221448	ENSP00000381216
ENSP00000221448	ENSP00000383059
ENSP00000221448	ENSP00000400591
ENSP00000221448	ENSP00000405963
ENSP00000221448	ENSP00000412566
ENSP00000221448	ENSP00000420195
ENSP00000221455	ENSP00000244769
ENSP00000221455	ENSP00000254942
ENSP00000221455	ENSP00000381932
ENSP00000221466	ENSP00000295897
ENSP00000221466	ENSP00000378394
ENSP00000221480	ENSP00000223136
ENSP00000221480	ENSP00000225873
ENSP00000221480	ENSP00000241052
ENSP00000221480	ENSP00000256216
ENSP00000221480	ENSP00000288774
ENSP00000221480	ENSP00000295030
ENSP00000221480	ENSP00000320646
ENSP00000221480	ENSP00000349016
ENSP00000221480	ENSP00000349543
ENSP00000221480	ENSP00000356563
ENSP00000221480	ENSP00000358312
ENSP00000221480	ENSP00000407401
ENSP00000221486	ENSP00000233146
ENSP00000221486	ENSP00000234420
ENSP00000221486	ENSP00000241436
ENSP00000221486	ENSP00000251038

ENSP00000221486	ENSP00000258428
ENSP00000221486	ENSP00000264951
ENSP00000221486	ENSP00000265081
ENSP00000221486	ENSP00000288774
ENSP00000221486	ENSP00000298139
ENSP00000221486	ENSP00000302801
ENSP00000221486	ENSP00000308193
ENSP00000221486	ENSP00000311873
ENSP00000221486	ENSP00000313350
ENSP00000221486	ENSP00000317039
ENSP00000221486	ENSP00000317636
ENSP00000221486	ENSP00000318977
ENSP00000221486	ENSP00000322570
ENSP00000221486	ENSP00000323439
ENSP00000221486	ENSP00000325863
ENSP00000221486	ENSP00000337405
ENSP00000221486	ENSP00000337623
ENSP00000221486	ENSP00000340305
ENSP00000221486	ENSP00000343392
ENSP00000221486	ENSP00000347232
ENSP00000221486	ENSP00000347978
ENSP00000221486	ENSP00000348107
ENSP00000221486	ENSP00000351284
ENSP00000221486	ENSP00000351697
ENSP00000221486	ENSP00000353622
ENSP00000221486	ENSP00000354522
ENSP00000221486	ENSP00000361310
ENSP00000221486	ENSP00000364126
ENSP00000221486	ENSP00000368349
ENSP00000221486	ENSP00000368438
ENSP00000221486	ENSP00000374399
ENSP00000221486	ENSP00000394869
ENSP00000221494	ENSP00000222969
ENSP00000221494	ENSP00000227524
ENSP00000221494	ENSP00000236273
ENSP00000221494	ENSP00000242784
ENSP00000221494	ENSP00000243563
ENSP00000221494	ENSP00000244520
ENSP00000221494	ENSP00000246190
ENSP00000221494	ENSP00000247001
ENSP00000221494	ENSP00000248378
ENSP00000221494	ENSP00000252115
ENSP00000221494	ENSP00000253363
ENSP00000221494	ENSP00000254193
ENSP00000221494	ENSP00000259235
ENSP00000221494	ENSP00000260210

ENSP00000221494	ENSP00000261167
ENSP00000221494	ENSP00000261531
ENSP00000221494	ENSP00000261973
ENSP00000221494	ENSP00000262498
ENSP00000221494	ENSP00000262518
ENSP00000221494	ENSP00000262710
ENSP00000221494	ENSP00000263697
ENSP00000221494	ENSP00000265872
ENSP00000221494	ENSP00000266079
ENSP00000221494	ENSP00000266735
ENSP00000221494	ENSP00000267812
ENSP00000221494	ENSP00000268482
ENSP00000221494	ENSP00000271526
ENSP00000221494	ENSP00000271628
ENSP00000221494	ENSP00000276201
ENSP00000221494	ENSP00000291688
ENSP00000221494	ENSP00000296702
ENSP00000221494	ENSP00000300413
ENSP00000221494	ENSP00000301011
ENSP00000221494	ENSP00000301740
ENSP00000221494	ENSP00000302160
ENSP00000221494	ENSP00000302768
ENSP00000221494	ENSP00000303191
ENSP00000221494	ENSP00000304350
ENSP00000221494	ENSP00000304370
ENSP00000221494	ENSP00000304903
ENSP00000221494	ENSP00000305790
ENSP00000221494	ENSP00000307863
ENSP00000221494	ENSP00000310448
ENSP00000221494	ENSP00000310723
ENSP00000221494	ENSP00000311677
ENSP00000221494	ENSP00000313674
ENSP00000221494	ENSP00000314491
ENSP00000221494	ENSP00000315379
ENSP00000221494	ENSP00000315859
ENSP00000221494	ENSP00000316109
ENSP00000221494	ENSP00000316879
ENSP00000221494	ENSP00000318861
ENSP00000221494	ENSP00000322016
ENSP00000221494	ENSP00000323302
ENSP00000221494	ENSP00000326261
ENSP00000221494	ENSP00000326806
ENSP00000221494	ENSP00000331817
ENSP00000221494	ENSP00000333376
ENSP00000221494	ENSP00000334148
ENSP00000221494	ENSP00000335321

ENSP00000221494	ENSP00000337405
ENSP00000221494	ENSP00000337501
ENSP00000221494	ENSP00000337632
ENSP00000221494	ENSP00000339090
ENSP00000221494	ENSP00000340019
ENSP00000221494	ENSP00000340281
ENSP00000221494	ENSP00000342374
ENSP00000221494	ENSP00000346236
ENSP00000221494	ENSP00000347592
ENSP00000221494	ENSP00000352308
ENSP00000221494	ENSP00000355011
ENSP00000221494	ENSP00000355050
ENSP00000221494	ENSP00000355205
ENSP00000221494	ENSP00000356520
ENSP00000221494	ENSP00000356541
ENSP00000221494	ENSP00000357631
ENSP00000221494	ENSP00000358554
ENSP00000221494	ENSP00000358605
ENSP00000221494	ENSP00000360286
ENSP00000221494	ENSP00000360532
ENSP00000221494	ENSP00000362110
ENSP00000221494	ENSP00000362820
ENSP00000221494	ENSP00000362900
ENSP00000221494	ENSP00000362936
ENSP00000221494	ENSP00000363313
ENSP00000221494	ENSP00000363745
ENSP00000221494	ENSP00000364289
ENSP00000221494	ENSP00000365439
ENSP00000221494	ENSP00000365747
ENSP00000221494	ENSP00000366607
ENSP00000221494	ENSP00000366829
ENSP00000221494	ENSP00000368801
ENSP00000221494	ENSP00000368856
ENSP00000221494	ENSP00000369218
ENSP00000221494	ENSP00000374213
ENSP00000221494	ENSP00000375863
ENSP00000221494	ENSP00000376309
ENSP00000221494	ENSP00000378165
ENSP00000221494	ENSP00000383059
ENSP00000221494	ENSP00000400591
ENSP00000221494	ENSP00000404121
ENSP00000221494	ENSP00000412566
ENSP00000221494	ENSP00000416959
ENSP00000221496	ENSP00000257863
ENSP00000221496	ENSP00000274712
ENSP00000221496	ENSP00000297261

ENSP00000221496	ENSP00000306894
ENSP00000221496	ENSP00000332353
ENSP00000221496	ENSP00000350512
ENSP00000221496	ENSP00000365943
ENSP00000221498	ENSP00000300574
ENSP00000221498	ENSP00000325508
ENSP00000221498	ENSP00000390783
ENSP00000221498	ENSP00000400223
ENSP00000221554	ENSP00000227524
ENSP00000221554	ENSP00000252137
ENSP00000221554	ENSP00000261531
ENSP00000221554	ENSP00000261745
ENSP00000221554	ENSP00000264233
ENSP00000221554	ENSP00000268206
ENSP00000221554	ENSP00000278886
ENSP00000221554	ENSP00000291688
ENSP00000221554	ENSP00000295488
ENSP00000221554	ENSP00000296805
ENSP00000221554	ENSP00000298288
ENSP00000221554	ENSP00000298532
ENSP00000221554	ENSP00000304350
ENSP00000221554	ENSP00000304370
ENSP00000221554	ENSP00000307491
ENSP00000221554	ENSP00000326531
ENSP00000221554	ENSP00000334148
ENSP00000221554	ENSP00000351137
ENSP00000221554	ENSP00000355296
ENSP00000221554	ENSP00000359454
ENSP00000221554	ENSP00000360532
ENSP00000221554	ENSP00000364212
ENSP00000221554	ENSP00000364289
ENSP00000221554	ENSP00000366557
ENSP00000221554	ENSP00000376973
ENSP00000221554	ENSP00000387006
ENSP00000221554	ENSP00000416583
ENSP00000221566	ENSP00000223136
ENSP00000221566	ENSP00000223641
ENSP00000221566	ENSP00000225726
ENSP00000221566	ENSP00000230882
ENSP00000221566	ENSP00000234590
ENSP00000221566	ENSP00000238994
ENSP00000221566	ENSP00000239944
ENSP00000221566	ENSP00000240159
ENSP00000221566	ENSP00000242465
ENSP00000221566	ENSP00000249636
ENSP00000221566	ENSP00000252487

ENSP00000221566	ENSP00000254878
ENSP00000221566	ENSP00000265857
ENSP00000221566	ENSP00000266732
ENSP00000221566	ENSP00000276570
ENSP00000221566	ENSP00000277541
ENSP00000221566	ENSP00000287143
ENSP00000221566	ENSP00000294053
ENSP00000221566	ENSP00000299767
ENSP00000221566	ENSP00000300175
ENSP00000221566	ENSP00000307674
ENSP00000221566	ENSP00000310521
ENSP00000221566	ENSP00000317331
ENSP00000221566	ENSP00000320508
ENSP00000221566	ENSP00000326981
ENSP00000221566	ENSP00000327716
ENSP00000221566	ENSP00000332171
ENSP00000221566	ENSP00000333920
ENSP00000221566	ENSP00000340864
ENSP00000221566	ENSP00000341170
ENSP00000221566	ENSP00000341551
ENSP00000221566	ENSP00000349887
ENSP00000221566	ENSP00000350005
ENSP00000221566	ENSP00000350708
ENSP00000221566	ENSP00000351665
ENSP00000221566	ENSP00000355896
ENSP00000221566	ENSP00000357360
ENSP00000221566	ENSP00000360660
ENSP00000221566	ENSP00000365131
ENSP00000221566	ENSP00000370588
ENSP00000221566	ENSP00000378517
ENSP00000221566	ENSP00000381293
ENSP00000221566	ENSP00000383115
ENSP00000221566	ENSP00000388841
ENSP00000221566	ENSP00000397956
ENSP00000221573	ENSP00000298532
ENSP00000221671	ENSP00000222005
ENSP00000221855	ENSP00000255465
ENSP00000221855	ENSP00000256031
ENSP00000221855	ENSP00000299601
ENSP00000221855	ENSP00000327009
ENSP00000221855	ENSP00000343535
ENSP00000221855	ENSP00000347719
ENSP00000221855	ENSP00000351665
ENSP00000221855	ENSP00000355560
ENSP00000221855	ENSP00000356579
ENSP00000221855	ENSP00000356989

ENSP00000221855	ENSP00000357651
ENSP00000221855	ENSP00000363734
ENSP00000221855	ENSP00000369736
ENSP00000221855	ENSP00000372694
ENSP00000221855	ENSP00000376946
ENSP00000221855	ENSP00000386621
ENSP00000221855	ENSP00000408910
ENSP00000221855	ENSP00000412319
ENSP00000221855	ENSP00000414906
ENSP00000221855	ENSP00000415678
ENSP00000221859	ENSP00000222567
ENSP00000221859	ENSP00000224862
ENSP00000221859	ENSP00000225504
ENSP00000221859	ENSP00000225728
ENSP00000221859	ENSP00000225916
ENSP00000221859	ENSP00000229971
ENSP00000221859	ENSP00000237455
ENSP00000221859	ENSP00000242719
ENSP00000221859	ENSP00000245539
ENSP00000221859	ENSP00000247843
ENSP00000221859	ENSP00000247970
ENSP00000221859	ENSP00000247977
ENSP00000221859	ENSP00000251038
ENSP00000221859	ENSP00000252445
ENSP00000221859	ENSP00000252602
ENSP00000221859	ENSP00000254719
ENSP00000221859	ENSP00000254900
ENSP00000221859	ENSP00000255764
ENSP00000221859	ENSP00000256257
ENSP00000221859	ENSP00000256379
ENSP00000221859	ENSP00000256398
ENSP00000221859	ENSP00000256897
ENSP00000221859	ENSP00000259605
ENSP00000221859	ENSP00000262498
ENSP00000221859	ENSP00000262608
ENSP00000221859	ENSP00000263390
ENSP00000221859	ENSP00000263997
ENSP00000221859	ENSP00000265073
ENSP00000221859	ENSP00000272645
ENSP00000221859	ENSP00000277865
ENSP00000221859	ENSP00000283875
ENSP00000221859	ENSP00000283882
ENSP00000221859	ENSP00000286317
ENSP00000221859	ENSP00000288199
ENSP00000221859	ENSP00000288985
ENSP00000221859	ENSP00000292363

ENSP00000221859	ENSP00000295131
ENSP00000221859	ENSP00000296509
ENSP00000221859	ENSP00000299543
ENSP00000221859	ENSP00000301788
ENSP00000221859	ENSP00000302160
ENSP00000221859	ENSP00000304321
ENSP00000221859	ENSP00000304593
ENSP00000221859	ENSP00000306906
ENSP00000221859	ENSP00000306983
ENSP00000221859	ENSP00000307491
ENSP00000221859	ENSP00000307684
ENSP00000221859	ENSP00000308944
ENSP00000221859	ENSP00000312735
ENSP00000221859	ENSP00000314949
ENSP00000221859	ENSP00000315791
ENSP00000221859	ENSP00000315950
ENSP00000221859	ENSP00000317636
ENSP00000221859	ENSP00000318604
ENSP00000221859	ENSP00000318674
ENSP00000221859	ENSP00000321386
ENSP00000221859	ENSP00000321927
ENSP00000221859	ENSP00000324124
ENSP00000221859	ENSP00000325677
ENSP00000221859	ENSP00000330098
ENSP00000221859	ENSP00000334188
ENSP00000221859	ENSP00000334675
ENSP00000221859	ENSP00000336868
ENSP00000221859	ENSP00000337340
ENSP00000221859	ENSP00000339845
ENSP00000221859	ENSP00000340823
ENSP00000221859	ENSP00000342121
ENSP00000221859	ENSP00000342889
ENSP00000221859	ENSP00000345096
ENSP00000221859	ENSP00000347345
ENSP00000221859	ENSP00000347719
ENSP00000221859	ENSP00000348089
ENSP00000221859	ENSP00000348708
ENSP00000221859	ENSP00000351284
ENSP00000221859	ENSP00000351575
ENSP00000221859	ENSP00000353098
ENSP00000221859	ENSP00000354775
ENSP00000221859	ENSP00000357494
ENSP00000221859	ENSP00000357887
ENSP00000221859	ENSP00000358478
ENSP00000221859	ENSP00000359531
ENSP00000221859	ENSP00000360515

ENSP00000221859	ENSP00000364289
ENSP00000221859	ENSP00000366271
ENSP00000221859	ENSP00000366828
ENSP00000221859	ENSP00000367029
ENSP00000221859	ENSP00000369695
ENSP00000221859	ENSP00000370968
ENSP00000221859	ENSP00000371475
ENSP00000221859	ENSP00000371800
ENSP00000221859	ENSP00000377969
ENSP00000221859	ENSP00000378736
ENSP00000221859	ENSP00000380793
ENSP00000221859	ENSP00000386134
ENSP00000221859	ENSP00000386759
ENSP00000221859	ENSP00000391723
ENSP00000221859	ENSP00000403852
ENSP00000221859	ENSP00000418111
ENSP00000221922	ENSP00000267415
ENSP00000221922	ENSP00000276603
ENSP00000221922	ENSP00000350249
ENSP00000221972	ENSP00000254801
ENSP00000221972	ENSP00000265132
ENSP00000221972	ENSP00000313419
ENSP00000221972	ENSP00000342681
ENSP00000221972	ENSP00000346839
ENSP00000221972	ENSP00000348888
ENSP00000221972	ENSP00000356346
ENSP00000221972	ENSP00000364898
ENSP00000221972	ENSP00000373952
ENSP00000221972	ENSP00000376544
ENSP00000221978	ENSP00000356737
ENSP00000221980	ENSP00000326366
ENSP00000221980	ENSP00000327513
ENSP00000222005	ENSP00000230510
ENSP00000222005	ENSP00000234626
ENSP00000222005	ENSP00000254942
ENSP00000222005	ENSP00000262315
ENSP00000222005	ENSP00000263043
ENSP00000222005	ENSP00000270223
ENSP00000222005	ENSP00000276603
ENSP00000222005	ENSP00000277010
ENSP00000222005	ENSP00000288602
ENSP00000222005	ENSP00000296084
ENSP00000222005	ENSP00000298139
ENSP00000222005	ENSP00000300146
ENSP00000222005	ENSP00000304593
ENSP00000222005	ENSP00000305958

ENSP00000222005	ENSP00000311005
ENSP00000222005	ENSP00000311083
ENSP00000222005	ENSP00000312244
ENSP00000222005	ENSP00000313601
ENSP00000222005	ENSP00000317039
ENSP00000222005	ENSP00000318646
ENSP00000222005	ENSP00000321636
ENSP00000222005	ENSP00000322180
ENSP00000222005	ENSP00000324560
ENSP00000222005	ENSP00000325677
ENSP00000222005	ENSP00000329933
ENSP00000222005	ENSP00000329967
ENSP00000222005	ENSP00000338990
ENSP00000222005	ENSP00000347232
ENSP00000222005	ENSP00000351596
ENSP00000222005	ENSP00000353059
ENSP00000222005	ENSP00000353826
ENSP00000222005	ENSP00000354558
ENSP00000222005	ENSP00000354927
ENSP00000222005	ENSP00000356989
ENSP00000222005	ENSP00000357494
ENSP00000222005	ENSP00000358335
ENSP00000222005	ENSP00000364204
ENSP00000222005	ENSP00000364543
ENSP00000222005	ENSP00000365005
ENSP00000222005	ENSP00000367029
ENSP00000222005	ENSP00000370741
ENSP00000222005	ENSP00000372827
ENSP00000222005	ENSP00000373700
ENSP00000222005	ENSP00000374455
ENSP00000222005	ENSP00000378350
ENSP00000222005	ENSP00000382423
ENSP00000222005	ENSP00000386621
ENSP00000222005	ENSP00000391249
ENSP00000222005	ENSP00000394400
ENSP00000222005	ENSP00000399530
ENSP00000222005	ENSP00000400626
ENSP00000222005	ENSP00000412310
ENSP00000222008	ENSP00000223136
ENSP00000222008	ENSP00000232003
ENSP00000222008	ENSP00000238831
ENSP00000222008	ENSP00000243918
ENSP00000222008	ENSP00000245923
ENSP00000222008	ENSP00000248139
ENSP00000222008	ENSP00000250113
ENSP00000222008	ENSP00000258301

ENSP00000222008	ENSP00000263233
ENSP00000222008	ENSP00000263274
ENSP00000222008	ENSP00000263925
ENSP00000222008	ENSP00000266126
ENSP00000222008	ENSP00000267484
ENSP00000222008	ENSP00000283351
ENSP00000222008	ENSP00000284957
ENSP00000222008	ENSP00000296509
ENSP00000222008	ENSP00000304292
ENSP00000222008	ENSP00000305255
ENSP00000222008	ENSP00000307900
ENSP00000222008	ENSP00000316854
ENSP00000222008	ENSP00000317891
ENSP00000222008	ENSP00000328070
ENSP00000222008	ENSP00000329715
ENSP00000222008	ENSP00000330945
ENSP00000222008	ENSP00000336724
ENSP00000222008	ENSP00000337838
ENSP00000222008	ENSP00000338345
ENSP00000222008	ENSP00000340836
ENSP00000222008	ENSP00000342136
ENSP00000222008	ENSP00000343435
ENSP00000222008	ENSP00000344106
ENSP00000222008	ENSP00000348459
ENSP00000222008	ENSP00000349016
ENSP00000222008	ENSP00000355739
ENSP00000222008	ENSP00000356602
ENSP00000222008	ENSP00000356920
ENSP00000222008	ENSP00000359691
ENSP00000222008	ENSP00000362588
ENSP00000222008	ENSP00000365630
ENSP00000222008	ENSP00000368959
ENSP00000222008	ENSP00000370410
ENSP00000222008	ENSP00000376871
ENSP00000222008	ENSP00000377665
ENSP00000222008	ENSP00000386134
ENSP00000222008	ENSP00000386394
ENSP00000222008	ENSP00000394071
ENSP00000222008	ENSP00000402240
ENSP00000222115	ENSP00000226284
ENSP00000222115	ENSP00000243563
ENSP00000222115	ENSP00000256474
ENSP00000222115	ENSP00000281282
ENSP00000222115	ENSP00000299601
ENSP00000222115	ENSP00000332681
ENSP00000222115	ENSP00000334100

ENSP00000222115	ENSP00000337053
ENSP00000222115	ENSP00000340935
ENSP00000222115	ENSP00000348708
ENSP00000222115	ENSP00000358413
ENSP00000222115	ENSP00000361845
ENSP00000222115	ENSP00000363559
ENSP00000222115	ENSP00000382767
ENSP00000222115	ENSP00000391432
ENSP00000222115	ENSP00000397598
ENSP00000222115	ENSP00000407879
ENSP00000222122	ENSP00000273951
ENSP00000222122	ENSP00000308741
ENSP00000222122	ENSP00000319977
ENSP00000222122	ENSP00000340671
ENSP00000222122	ENSP00000348565
ENSP00000222122	ENSP00000351407
ENSP00000222139	ENSP00000222254
ENSP00000222139	ENSP00000228280
ENSP00000222139	ENSP00000244007
ENSP00000222139	ENSP00000252723
ENSP00000222139	ENSP00000262995
ENSP00000222139	ENSP00000263233
ENSP00000222139	ENSP00000281171
ENSP00000222139	ENSP00000296474
ENSP00000222139	ENSP00000340944
ENSP00000222139	ENSP00000341208
ENSP00000222139	ENSP00000342755
ENSP00000222139	ENSP00000356346
ENSP00000222139	ENSP00000359206
ENSP00000222139	ENSP00000364898
ENSP00000222139	ENSP00000365016
ENSP00000222139	ENSP00000384053
ENSP00000222139	ENSP00000409346
ENSP00000222157	ENSP00000311005
ENSP00000222157	ENSP00000339826
ENSP00000222247	ENSP00000225430
ENSP00000222247	ENSP00000230050
ENSP00000222247	ENSP00000230859
ENSP00000222247	ENSP00000238256
ENSP00000222247	ENSP00000245539
ENSP00000222247	ENSP00000252037
ENSP00000222247	ENSP00000252543
ENSP00000222247	ENSP00000253099
ENSP00000222247	ENSP00000253788
ENSP00000222247	ENSP00000254037
ENSP00000222247	ENSP00000257829

ENSP00000222247	ENSP00000258455
ENSP00000222247	ENSP00000259469
ENSP00000222247	ENSP00000260102
ENSP00000222247	ENSP00000260443
ENSP00000222247	ENSP00000262584
ENSP00000222247	ENSP00000264995
ENSP00000222247	ENSP00000265100
ENSP00000222247	ENSP00000268616
ENSP00000222247	ENSP00000268661
ENSP00000222247	ENSP00000269499
ENSP00000222247	ENSP00000270625
ENSP00000222247	ENSP00000272418
ENSP00000222247	ENSP00000274242
ENSP00000222247	ENSP00000274606
ENSP00000222247	ENSP00000274849
ENSP00000222247	ENSP00000278412
ENSP00000222247	ENSP00000278572
ENSP00000222247	ENSP00000287038
ENSP00000222247	ENSP00000294189
ENSP00000222247	ENSP00000296411
ENSP00000222247	ENSP00000296802
ENSP00000222247	ENSP00000297990
ENSP00000222247	ENSP00000298283
ENSP00000222247	ENSP00000298310
ENSP00000222247	ENSP00000300151
ENSP00000222247	ENSP00000302886
ENSP00000222247	ENSP00000302896
ENSP00000222247	ENSP00000305702
ENSP00000222247	ENSP00000306548
ENSP00000222247	ENSP00000307889
ENSP00000222247	ENSP00000308897
ENSP00000222247	ENSP00000309334
ENSP00000222247	ENSP00000309830
ENSP00000222247	ENSP00000310042
ENSP00000222247	ENSP00000311028
ENSP00000222247	ENSP00000311430
ENSP00000222247	ENSP00000312042
ENSP00000222247	ENSP00000314348
ENSP00000222247	ENSP00000317232
ENSP00000222247	ENSP00000317376
ENSP00000222247	ENSP00000317992
ENSP00000222247	ENSP00000318604
ENSP00000222247	ENSP00000318646
ENSP00000222247	ENSP00000322419
ENSP00000222247	ENSP00000325312
ENSP00000222247	ENSP00000326981

ENSP00000222247	ENSP00000329466
ENSP00000222247	ENSP00000336856
ENSP00000222247	ENSP00000337839
ENSP00000222247	ENSP00000338862
ENSP00000222247	ENSP00000339095
ENSP00000222247	ENSP00000339795
ENSP00000222247	ENSP00000341885
ENSP00000222247	ENSP00000345156
ENSP00000222247	ENSP00000346015
ENSP00000222247	ENSP00000346022
ENSP00000222247	ENSP00000346027
ENSP00000222247	ENSP00000346037
ENSP00000222247	ENSP00000346050
ENSP00000222247	ENSP00000346088
ENSP00000222247	ENSP00000346120
ENSP00000222247	ENSP00000347087
ENSP00000222247	ENSP00000352021
ENSP00000222247	ENSP00000354040
ENSP00000222247	ENSP00000354525
ENSP00000222247	ENSP00000354739
ENSP00000222247	ENSP00000355759
ENSP00000222247	ENSP00000356811
ENSP00000222247	ENSP00000359345
ENSP00000222247	ENSP00000359688
ENSP00000222247	ENSP00000361076
ENSP00000222247	ENSP00000362744
ENSP00000222247	ENSP00000363018
ENSP00000222247	ENSP00000363559
ENSP00000222247	ENSP00000363676
ENSP00000222247	ENSP00000364320
ENSP00000222247	ENSP00000369757
ENSP00000222247	ENSP00000370589
ENSP00000222247	ENSP00000373404
ENSP00000222247	ENSP00000375730
ENSP00000222247	ENSP00000377640
ENSP00000222247	ENSP00000377865
ENSP00000222247	ENSP00000378160
ENSP00000222247	ENSP00000379607
ENSP00000222247	ENSP00000379888
ENSP00000222247	ENSP00000380156
ENSP00000222247	ENSP00000380982
ENSP00000222247	ENSP00000386717
ENSP00000222247	ENSP00000389103
ENSP00000222247	ENSP00000391723
ENSP00000222247	ENSP00000393241
ENSP00000222247	ENSP00000393393

ENSP00000222247	ENSP00000402338
ENSP00000222247	ENSP00000403175
ENSP00000222247	ENSP00000412583
ENSP00000222247	ENSP00000413152
ENSP00000222247	ENSP00000414321
ENSP00000222247	ENSP00000416110
ENSP00000222254	ENSP00000226574
ENSP00000222254	ENSP00000231487
ENSP00000222254	ENSP00000237380
ENSP00000222254	ENSP00000264637
ENSP00000222254	ENSP00000265171
ENSP00000222254	ENSP00000267101
ENSP00000222254	ENSP00000269485
ENSP00000222254	ENSP00000273317
ENSP00000222254	ENSP00000295408
ENSP00000222254	ENSP00000298229
ENSP00000222254	ENSP00000300574
ENSP00000222254	ENSP00000306124
ENSP00000222254	ENSP00000312946
ENSP00000222254	ENSP00000313829
ENSP00000222254	ENSP00000327513
ENSP00000222254	ENSP00000331044
ENSP00000222254	ENSP00000340944
ENSP00000222254	ENSP00000341189
ENSP00000222254	ENSP00000347532
ENSP00000222254	ENSP00000352121
ENSP00000222254	ENSP00000355827
ENSP00000222254	ENSP00000361855
ENSP00000222254	ENSP00000364898
ENSP00000222254	ENSP00000365016
ENSP00000222254	ENSP00000365439
ENSP00000222254	ENSP00000366936
ENSP00000222254	ENSP00000381793
ENSP00000222254	ENSP00000384675
ENSP00000222266	ENSP00000257626
ENSP00000222266	ENSP00000262133
ENSP00000222266	ENSP00000294785
ENSP00000222266	ENSP00000326366
ENSP00000222266	ENSP00000329102
ENSP00000222266	ENSP00000340995
ENSP00000222266	ENSP00000341947
ENSP00000222266	ENSP00000358105
ENSP00000222266	ENSP00000362592
ENSP00000222266	ENSP00000368686
ENSP00000222284	ENSP00000379282
ENSP00000222284	ENSP00000390746

ENSP00000222286	ENSP00000233242
ENSP00000222286	ENSP00000234590
ENSP00000222286	ENSP00000245206
ENSP00000222286	ENSP00000254035
ENSP00000222286	ENSP00000258494
ENSP00000222286	ENSP00000265354
ENSP00000222286	ENSP00000282541
ENSP00000222286	ENSP00000322570
ENSP00000222286	ENSP00000327070
ENSP00000222286	ENSP00000329189
ENSP00000222286	ENSP00000336927
ENSP00000222286	ENSP00000339933
ENSP00000222286	ENSP00000340237
ENSP00000222286	ENSP00000351697
ENSP00000222286	ENSP00000361465
ENSP00000222286	ENSP00000363873
ENSP00000222286	ENSP00000367408
ENSP00000222286	ENSP00000368349
ENSP00000222286	ENSP00000373477
ENSP00000222286	ENSP00000379607
ENSP00000222286	ENSP00000417281
ENSP00000222286	ENSP00000417963
ENSP00000222304	ENSP00000261024
ENSP00000222304	ENSP00000367265
ENSP00000222305	ENSP00000241600
ENSP00000222305	ENSP00000264731
ENSP00000222305	ENSP00000278070
ENSP00000222305	ENSP00000283882
ENSP00000222305	ENSP00000296930
ENSP00000222305	ENSP00000302530
ENSP00000222305	ENSP00000306245
ENSP00000222305	ENSP00000327048
ENSP00000222305	ENSP00000336724
ENSP00000222305	ENSP00000342056
ENSP00000222305	ENSP00000345702
ENSP00000222305	ENSP00000350283
ENSP00000222305	ENSP00000374280
ENSP00000222305	ENSP00000397441
ENSP00000222305	ENSP00000417281
ENSP00000222307	ENSP00000250113
ENSP00000222307	ENSP00000258424
ENSP00000222307	ENSP00000261708
ENSP00000222307	ENSP00000263805
ENSP00000222307	ENSP00000263925
ENSP00000222307	ENSP00000279281
ENSP00000222307	ENSP00000284957

ENSP00000222307	ENSP00000288266
ENSP00000222307	ENSP00000288422
ENSP00000222307	ENSP00000326514
ENSP00000222307	ENSP00000326767
ENSP00000222307	ENSP00000334100
ENSP00000222307	ENSP00000336524
ENSP00000222307	ENSP00000343479
ENSP00000222307	ENSP00000344547
ENSP00000222307	ENSP00000349640
ENSP00000222307	ENSP00000355840
ENSP00000222307	ENSP00000363135
ENSP00000222307	ENSP00000363972
ENSP00000222307	ENSP00000366249
ENSP00000222307	ENSP00000368699
ENSP00000222307	ENSP00000370410
ENSP00000222307	ENSP00000373039
ENSP00000222307	ENSP00000373669
ENSP00000222307	ENSP00000374455
ENSP00000222307	ENSP00000381031
ENSP00000222307	ENSP00000381795
ENSP00000222307	ENSP00000386394
ENSP00000222307	ENSP00000387209
ENSP00000222307	ENSP00000396152
ENSP00000222307	ENSP00000410416
ENSP00000222307	ENSP00000417281
ENSP00000222308	ENSP00000252037
ENSP00000222308	ENSP00000253003
ENSP00000222308	ENSP00000256474
ENSP00000222308	ENSP00000259457
ENSP00000222308	ENSP00000261712
ENSP00000222308	ENSP00000262193
ENSP00000222308	ENSP00000264639
ENSP00000222308	ENSP00000276603
ENSP00000222308	ENSP00000295901
ENSP00000222308	ENSP00000298852
ENSP00000222308	ENSP00000309474
ENSP00000222308	ENSP00000310129
ENSP00000222308	ENSP00000329623
ENSP00000222308	ENSP00000332513
ENSP00000222308	ENSP00000343412
ENSP00000222308	ENSP00000348442
ENSP00000222308	ENSP00000355601
ENSP00000222308	ENSP00000359385
ENSP00000222308	ENSP00000362334
ENSP00000222308	ENSP00000370376
ENSP00000222308	ENSP00000381863

ENSP00000222308	ENSP00000396937
ENSP00000222329	ENSP00000358716
ENSP00000222329	ENSP00000363763
ENSP00000222330	ENSP00000228307
ENSP00000222330	ENSP00000229595
ENSP00000222330	ENSP00000232607
ENSP00000222330	ENSP00000236051
ENSP00000222330	ENSP00000240922
ENSP00000222330	ENSP00000245960
ENSP00000222330	ENSP00000246166
ENSP00000222330	ENSP00000249373
ENSP00000222330	ENSP00000253693
ENSP00000222330	ENSP00000256442
ENSP00000222330	ENSP00000258052
ENSP00000222330	ENSP00000262320
ENSP00000222330	ENSP00000262367
ENSP00000222330	ENSP00000262764
ENSP00000222330	ENSP00000263246
ENSP00000222330	ENSP00000264883
ENSP00000222330	ENSP00000265073
ENSP00000222330	ENSP00000265372
ENSP00000222330	ENSP00000266126
ENSP00000222330	ENSP00000267064
ENSP00000222330	ENSP00000268459
ENSP00000222330	ENSP00000273783
ENSP00000222330	ENSP00000284981
ENSP00000222330	ENSP00000295872
ENSP00000222330	ENSP00000298746
ENSP00000222330	ENSP00000300417
ENSP00000222330	ENSP00000303088
ENSP00000222330	ENSP00000307235
ENSP00000222330	ENSP00000310042
ENSP00000222330	ENSP00000310649
ENSP00000222330	ENSP00000315693
ENSP00000222330	ENSP00000316879
ENSP00000222330	ENSP00000320485
ENSP00000222330	ENSP00000320488
ENSP00000222330	ENSP00000320646
ENSP00000222330	ENSP00000323858
ENSP00000222330	ENSP00000334061
ENSP00000222330	ENSP00000339933
ENSP00000222330	ENSP00000340820
ENSP00000222330	ENSP00000343479
ENSP00000222330	ENSP00000346236
ENSP00000222330	ENSP00000351284
ENSP00000222330	ENSP00000354927

ENSP00000222330	ENSP00000355596
ENSP00000222330	ENSP00000356602
ENSP00000222330	ENSP00000356920
ENSP00000222330	ENSP00000357051
ENSP00000222330	ENSP00000358022
ENSP00000222330	ENSP00000358404
ENSP00000222330	ENSP00000358563
ENSP00000222330	ENSP00000358918
ENSP00000222330	ENSP00000362171
ENSP00000222330	ENSP00000365005
ENSP00000222330	ENSP00000369757
ENSP00000222330	ENSP00000371846
ENSP00000222330	ENSP00000372857
ENSP00000222330	ENSP00000382423
ENSP00000222330	ENSP00000385903
ENSP00000222381	ENSP00000295897
ENSP00000222547	ENSP00000223369
ENSP00000222547	ENSP00000225567
ENSP00000222547	ENSP00000225724
ENSP00000222547	ENSP00000231668
ENSP00000222547	ENSP00000236192
ENSP00000222547	ENSP00000248633
ENSP00000222547	ENSP00000249647
ENSP00000222547	ENSP00000249923
ENSP00000222547	ENSP00000252597
ENSP00000222547	ENSP00000256897
ENSP00000222547	ENSP00000258301
ENSP00000222547	ENSP00000258654
ENSP00000222547	ENSP00000259400
ENSP00000222547	ENSP00000262544
ENSP00000222547	ENSP00000262812
ENSP00000222547	ENSP00000263897
ENSP00000222547	ENSP00000264028
ENSP00000222547	ENSP00000264157
ENSP00000222547	ENSP00000265175
ENSP00000222547	ENSP00000285243
ENSP00000222547	ENSP00000286448
ENSP00000222547	ENSP00000294179
ENSP00000222547	ENSP00000295119
ENSP00000222547	ENSP00000302783
ENSP00000222547	ENSP00000305255
ENSP00000222547	ENSP00000305647
ENSP00000222547	ENSP00000305810
ENSP00000222547	ENSP00000310521
ENSP00000222547	ENSP00000311134
ENSP00000222547	ENSP00000317714

ENSP00000222547	ENSP00000320885
ENSP00000222547	ENSP00000324628
ENSP00000222547	ENSP00000325002
ENSP00000222547	ENSP00000327309
ENSP00000222547	ENSP00000327650
ENSP00000222547	ENSP00000328079
ENSP00000222547	ENSP00000329419
ENSP00000222547	ENSP00000333950
ENSP00000222547	ENSP00000340836
ENSP00000222547	ENSP00000345656
ENSP00000222547	ENSP00000347329
ENSP00000222547	ENSP00000349785
ENSP00000222547	ENSP00000355721
ENSP00000222547	ENSP00000362399
ENSP00000222547	ENSP00000366225
ENSP00000222547	ENSP00000374529
ENSP00000222547	ENSP00000381293
ENSP00000222547	ENSP00000390783
ENSP00000222553	ENSP00000264170
ENSP00000222553	ENSP00000265896
ENSP00000222553	ENSP00000294973
ENSP00000222553	ENSP00000304102
ENSP00000222553	ENSP00000310040
ENSP00000222553	ENSP00000313050
ENSP00000222553	ENSP00000320949
ENSP00000222553	ENSP00000321711
ENSP00000222553	ENSP00000329466
ENSP00000222553	ENSP00000333946
ENSP00000222553	ENSP00000346155
ENSP00000222553	ENSP00000348442
ENSP00000222553	ENSP00000350512
ENSP00000222553	ENSP00000352928
ENSP00000222553	ENSP00000354522
ENSP00000222553	ENSP00000354687
ENSP00000222553	ENSP00000355517
ENSP00000222553	ENSP00000360992
ENSP00000222553	ENSP00000373952
ENSP00000222553	ENSP00000378782
ENSP00000222553	ENSP00000396937
ENSP00000222567	ENSP00000226796
ENSP00000222567	ENSP00000228862
ENSP00000222567	ENSP00000230050
ENSP00000222567	ENSP00000238256
ENSP00000222567	ENSP00000238789
ENSP00000222567	ENSP00000247219
ENSP00000222567	ENSP00000252037

ENSP00000222567	ENSP00000254719
ENSP00000222567	ENSP00000261881
ENSP00000222567	ENSP00000263736
ENSP00000222567	ENSP00000263857
ENSP00000222567	ENSP00000274606
ENSP00000222567	ENSP00000288976
ENSP00000222567	ENSP00000293860
ENSP00000222567	ENSP00000298746
ENSP00000222567	ENSP00000302478
ENSP00000222567	ENSP00000312735
ENSP00000222567	ENSP00000314949
ENSP00000222567	ENSP00000314971
ENSP00000222567	ENSP00000317232
ENSP00000222567	ENSP00000319104
ENSP00000222567	ENSP00000321636
ENSP00000222567	ENSP00000323856
ENSP00000222567	ENSP00000324124
ENSP00000222567	ENSP00000324792
ENSP00000222567	ENSP00000330343
ENSP00000222567	ENSP00000331111
ENSP00000222567	ENSP00000332171
ENSP00000222567	ENSP00000333920
ENSP00000222567	ENSP00000335357
ENSP00000222567	ENSP00000339095
ENSP00000222567	ENSP00000342121
ENSP00000222567	ENSP00000342889
ENSP00000222567	ENSP00000351596
ENSP00000222567	ENSP00000354916
ENSP00000222567	ENSP00000358563
ENSP00000222567	ENSP00000358812
ENSP00000222567	ENSP00000361076
ENSP00000222567	ENSP00000361465
ENSP00000222567	ENSP00000361982
ENSP00000222567	ENSP00000366557
ENSP00000222567	ENSP00000367029
ENSP00000222567	ENSP00000380504
ENSP00000222567	ENSP00000383503
ENSP00000222567	ENSP00000397636
ENSP00000222567	ENSP00000403852
ENSP00000222567	ENSP00000405636
ENSP00000222567	ENSP00000406293
ENSP00000222567	ENSP00000410530
ENSP00000222567	ENSP00000410954
ENSP00000222567	ENSP00000413152
ENSP00000222567	ENSP00000414110
ENSP00000222567	ENSP00000416599

ENSP00000222574	ENSP00000246166
ENSP00000222574	ENSP00000253003
ENSP00000222574	ENSP00000261845
ENSP00000222574	ENSP00000262133
ENSP00000222574	ENSP00000262367
ENSP00000222574	ENSP00000264157
ENSP00000222574	ENSP00000267163
ENSP00000222574	ENSP00000296328
ENSP00000222574	ENSP00000296504
ENSP00000222574	ENSP00000303148
ENSP00000222574	ENSP00000306163
ENSP00000222574	ENSP00000307272
ENSP00000222574	ENSP00000311005
ENSP00000222574	ENSP00000314949
ENSP00000222574	ENSP00000353622
ENSP00000222574	ENSP00000354848
ENSP00000222574	ENSP00000365550
ENSP00000222574	ENSP00000381031
ENSP00000222584	ENSP00000263121
ENSP00000222584	ENSP00000265351
ENSP00000222584	ENSP00000265717
ENSP00000222584	ENSP00000281043
ENSP00000222584	ENSP00000329357
ENSP00000222584	ENSP00000354850
ENSP00000222584	ENSP00000369042
ENSP00000222584	ENSP00000378306
ENSP00000222673	ENSP00000228318
ENSP00000222673	ENSP00000252699
ENSP00000222673	ENSP00000254193
ENSP00000222673	ENSP00000258886
ENSP00000222673	ENSP00000263966
ENSP00000222673	ENSP00000268668
ENSP00000222673	ENSP00000277865
ENSP00000222673	ENSP00000280346
ENSP00000222673	ENSP00000282050
ENSP00000222673	ENSP00000295951
ENSP00000222673	ENSP00000317159
ENSP00000222673	ENSP00000322570
ENSP00000222673	ENSP00000330343
ENSP00000222673	ENSP00000334188
ENSP00000222673	ENSP00000335304
ENSP00000222673	ENSP00000341838
ENSP00000222673	ENSP00000351697
ENSP00000222673	ENSP00000360569
ENSP00000222673	ENSP00000361577
ENSP00000222673	ENSP00000363944

ENSP00000222673	ENSP00000368349
ENSP00000222673	ENSP00000379607
ENSP00000222673	ENSP00000387262
ENSP00000222718	ENSP00000251871
ENSP00000222718	ENSP00000267064
ENSP00000222718	ENSP00000269397
ENSP00000222718	ENSP00000276072
ENSP00000222718	ENSP00000280098
ENSP00000222718	ENSP00000281928
ENSP00000222718	ENSP00000286364
ENSP00000222718	ENSP00000340896
ENSP00000222718	ENSP00000341805
ENSP00000222718	ENSP00000343246
ENSP00000222718	ENSP00000358854
ENSP00000222718	ENSP00000364524
ENSP00000222725	ENSP00000231357
ENSP00000222725	ENSP00000257879
ENSP00000222725	ENSP00000277541
ENSP00000222725	ENSP00000331608
ENSP00000222725	ENSP00000358532
ENSP00000222725	ENSP00000362717
ENSP00000222725	ENSP00000363373
ENSP00000222725	ENSP00000378132
ENSP00000222725	ENSP00000381293
ENSP00000222747	ENSP00000367301
ENSP00000222753	ENSP00000240423
ENSP00000222753	ENSP00000318423
ENSP00000222753	ENSP00000326296
ENSP00000222792	ENSP00000236051
ENSP00000222792	ENSP00000253339
ENSP00000222792	ENSP00000256216
ENSP00000222792	ENSP00000260008
ENSP00000222792	ENSP00000262300
ENSP00000222792	ENSP00000264639
ENSP00000222792	ENSP00000267101
ENSP00000222792	ENSP00000309871
ENSP00000222792	ENSP00000311005
ENSP00000222792	ENSP00000315659
ENSP00000222792	ENSP00000316845
ENSP00000222792	ENSP00000318884
ENSP00000222792	ENSP00000322957
ENSP00000222792	ENSP00000327315
ENSP00000222792	ENSP00000333950
ENSP00000222792	ENSP00000339299
ENSP00000222792	ENSP00000348215
ENSP00000222792	ENSP00000349145

ENSP00000222792	ENSP00000354718
ENSP00000222792	ENSP00000361009
ENSP00000222792	ENSP00000366121
ENSP00000222792	ENSP00000376410
ENSP00000222792	ENSP00000382177
ENSP00000222792	ENSP00000391249
ENSP00000222792	ENSP00000397351
ENSP00000222800	ENSP00000248553
ENSP00000222968	ENSP00000330382
ENSP00000222968	ENSP00000341947
ENSP00000222969	ENSP00000225171
ENSP00000222969	ENSP00000227524
ENSP00000222969	ENSP00000232888
ENSP00000222969	ENSP00000258383
ENSP00000222969	ENSP00000261531
ENSP00000222969	ENSP00000262584
ENSP00000222969	ENSP00000264065
ENSP00000222969	ENSP00000264233
ENSP00000222969	ENSP00000268206
ENSP00000222969	ENSP00000268482
ENSP00000222969	ENSP00000276570
ENSP00000222969	ENSP00000282050
ENSP00000222969	ENSP00000286317
ENSP00000222969	ENSP00000295373
ENSP00000222969	ENSP00000295488
ENSP00000222969	ENSP00000296805
ENSP00000222969	ENSP00000298532
ENSP00000222969	ENSP00000301740
ENSP00000222969	ENSP00000301764
ENSP00000222969	ENSP00000303191
ENSP00000222969	ENSP00000304350
ENSP00000222969	ENSP00000304370
ENSP00000222969	ENSP00000305790
ENSP00000222969	ENSP00000312311
ENSP00000222969	ENSP00000313674
ENSP00000222969	ENSP00000315700
ENSP00000222969	ENSP00000316240
ENSP00000222969	ENSP00000318861
ENSP00000222969	ENSP00000326531
ENSP00000222969	ENSP00000327070
ENSP00000222969	ENSP00000328992
ENSP00000222969	ENSP00000331907
ENSP00000222969	ENSP00000334148
ENSP00000222969	ENSP00000339845
ENSP00000222969	ENSP00000343557
ENSP00000222969	ENSP00000343745

ENSP00000222969	ENSP00000346986
ENSP00000222969	ENSP00000351137
ENSP00000222969	ENSP00000354522
ENSP00000222969	ENSP00000355050
ENSP00000222969	ENSP00000356520
ENSP00000222969	ENSP00000359454
ENSP00000222969	ENSP00000360532
ENSP00000222969	ENSP00000362041
ENSP00000222969	ENSP00000362110
ENSP00000222969	ENSP00000362744
ENSP00000222969	ENSP00000366179
ENSP00000222969	ENSP00000366557
ENSP00000222969	ENSP00000368523
ENSP00000222969	ENSP00000371236
ENSP00000222969	ENSP00000371451
ENSP00000222969	ENSP00000373404
ENSP00000222969	ENSP00000376973
ENSP00000222969	ENSP00000379607
ENSP00000222969	ENSP00000387303
ENSP00000222969	ENSP00000392601
ENSP00000222969	ENSP00000393953
ENSP00000222969	ENSP00000416959
ENSP00000222990	ENSP00000233055
ENSP00000222990	ENSP00000243918
ENSP00000222990	ENSP00000245923
ENSP00000222990	ENSP00000251038
ENSP00000222990	ENSP00000265990
ENSP00000222990	ENSP00000267484
ENSP00000222990	ENSP00000269122
ENSP00000222990	ENSP00000281187
ENSP00000222990	ENSP00000299138
ENSP00000222990	ENSP00000307850
ENSP00000222990	ENSP00000314949
ENSP00000222990	ENSP00000337838
ENSP00000222990	ENSP00000344106
ENSP00000222990	ENSP00000358497
ENSP00000222990	ENSP00000359607
ENSP00000222990	ENSP00000365567
ENSP00000223023	ENSP00000225655
ENSP00000223023	ENSP00000234396
ENSP00000223023	ENSP00000242285
ENSP00000223023	ENSP00000252699
ENSP00000223023	ENSP00000252725
ENSP00000223023	ENSP00000253083
ENSP00000223023	ENSP00000259477
ENSP00000223023	ENSP00000261017

ENSP00000223023	ENSP00000261558
ENSP00000223023	ENSP00000262375
ENSP00000223023	ENSP00000263246
ENSP00000223023	ENSP00000264864
ENSP00000223023	ENSP00000269122
ENSP00000223023	ENSP00000276416
ENSP00000223023	ENSP00000277541
ENSP00000223023	ENSP00000284637
ENSP00000223023	ENSP00000294129
ENSP00000223023	ENSP00000295685
ENSP00000223023	ENSP00000300574
ENSP00000223023	ENSP00000301843
ENSP00000223023	ENSP00000303434
ENSP00000223023	ENSP00000307272
ENSP00000223023	ENSP00000309610
ENSP00000223023	ENSP00000309629
ENSP00000223023	ENSP00000314499
ENSP00000223023	ENSP00000315659
ENSP00000223023	ENSP00000320924
ENSP00000223023	ENSP00000326238
ENSP00000223023	ENSP00000333873
ENSP00000223023	ENSP00000333950
ENSP00000223023	ENSP00000334836
ENSP00000223023	ENSP00000340494
ENSP00000223023	ENSP00000343526
ENSP00000223023	ENSP00000343741
ENSP00000223023	ENSP00000344741
ENSP00000223023	ENSP00000345530
ENSP00000223023	ENSP00000348163
ENSP00000223023	ENSP00000348215
ENSP00000223023	ENSP00000348602
ENSP00000223023	ENSP00000349959
ENSP00000223023	ENSP00000352121
ENSP00000223023	ENSP00000352603
ENSP00000223023	ENSP00000352802
ENSP00000223023	ENSP00000358994
ENSP00000223023	ENSP00000360798
ENSP00000223023	ENSP00000366121
ENSP00000223023	ENSP00000366746
ENSP00000223023	ENSP00000368818
ENSP00000223023	ENSP00000370719
ENSP00000223023	ENSP00000378338
ENSP00000223023	ENSP00000379228
ENSP00000223023	ENSP00000379709
ENSP00000223023	ENSP00000380450
ENSP00000223023	ENSP00000380921

ENSP00000223023	ENSP00000382177
ENSP00000223023	ENSP00000386456
ENSP00000223023	ENSP00000386722
ENSP00000223023	ENSP00000407879
ENSP00000223023	ENSP00000408910
ENSP00000223023	ENSP00000413625
ENSP00000223029	ENSP00000227758
ENSP00000223029	ENSP00000231572
ENSP00000223029	ENSP00000232905
ENSP00000223029	ENSP00000254336
ENSP00000223029	ENSP00000259075
ENSP00000223029	ENSP00000262027
ENSP00000223029	ENSP00000263925
ENSP00000223029	ENSP00000264161
ENSP00000223029	ENSP00000268058
ENSP00000223029	ENSP00000281172
ENSP00000223029	ENSP00000282276
ENSP00000223029	ENSP00000296564
ENSP00000223029	ENSP00000300086
ENSP00000223029	ENSP00000307567
ENSP00000223029	ENSP00000318128
ENSP00000223029	ENSP00000325448
ENSP00000223029	ENSP00000355086
ENSP00000223029	ENSP00000355865
ENSP00000223029	ENSP00000355890
ENSP00000223029	ENSP00000357283
ENSP00000223029	ENSP00000364794
ENSP00000223029	ENSP00000364815
ENSP00000223029	ENSP00000369038
ENSP00000223029	ENSP00000370410
ENSP00000223029	ENSP00000379607
ENSP00000223029	ENSP00000401121
ENSP00000223029	ENSP00000403359
ENSP00000223029	ENSP00000415316
ENSP00000223051	ENSP00000296509
ENSP00000223051	ENSP00000338413
ENSP00000223054	ENSP00000223136
ENSP00000223054	ENSP00000262812
ENSP00000223054	ENSP00000324792
ENSP00000223054	ENSP00000330343
ENSP00000223054	ENSP00000336994
ENSP00000223054	ENSP00000353741
ENSP00000223054	ENSP00000368439
ENSP00000223054	ENSP00000392330
ENSP00000223073	ENSP00000226796
ENSP00000223073	ENSP00000230859

ENSP00000223073	ENSP00000234170
ENSP00000223073	ENSP00000236051
ENSP00000223073	ENSP00000252115
ENSP00000223073	ENSP00000253107
ENSP00000223073	ENSP00000254940
ENSP00000223073	ENSP00000257829
ENSP00000223073	ENSP00000259569
ENSP00000223073	ENSP00000261015
ENSP00000223073	ENSP00000261637
ENSP00000223073	ENSP00000262056
ENSP00000223073	ENSP00000267425
ENSP00000223073	ENSP00000268661
ENSP00000223073	ENSP00000278856
ENSP00000223073	ENSP00000298746
ENSP00000223073	ENSP00000302886
ENSP00000223073	ENSP00000305702
ENSP00000223073	ENSP00000310668
ENSP00000223073	ENSP00000314311
ENSP00000223073	ENSP00000314348
ENSP00000223073	ENSP00000317992
ENSP00000223073	ENSP00000320917
ENSP00000223073	ENSP00000321711
ENSP00000223073	ENSP00000322396
ENSP00000223073	ENSP00000323858
ENSP00000223073	ENSP00000328690
ENSP00000223073	ENSP00000329466
ENSP00000223073	ENSP00000331817
ENSP00000223073	ENSP00000338573
ENSP00000223073	ENSP00000338862
ENSP00000223073	ENSP00000339145
ENSP00000223073	ENSP00000339795
ENSP00000223073	ENSP00000346453
ENSP00000223073	ENSP00000346725
ENSP00000223073	ENSP00000350491
ENSP00000223073	ENSP00000353741
ENSP00000223073	ENSP00000354040
ENSP00000223073	ENSP00000354522
ENSP00000223073	ENSP00000355541
ENSP00000223073	ENSP00000358400
ENSP00000223073	ENSP00000358563
ENSP00000223073	ENSP00000358812
ENSP00000223073	ENSP00000359345
ENSP00000223073	ENSP00000359688
ENSP00000223073	ENSP00000360031
ENSP00000223073	ENSP00000360828
ENSP00000223073	ENSP00000361076

ENSP00000223073	ENSP00000361232
ENSP00000223073	ENSP00000366519
ENSP00000223073	ENSP00000368612
ENSP00000223073	ENSP00000370589
ENSP00000223073	ENSP00000372199
ENSP00000223073	ENSP00000379607
ENSP00000223073	ENSP00000380982
ENSP00000223073	ENSP00000382392
ENSP00000223073	ENSP00000389103
ENSP00000223073	ENSP00000402338
ENSP00000223122	ENSP00000304364
ENSP00000223127	ENSP00000253490
ENSP00000223127	ENSP00000272902
ENSP00000223127	ENSP00000365147
ENSP00000223127	ENSP00000379607
ENSP00000223127	ENSP00000410833
ENSP00000223129	ENSP00000234296
ENSP00000223129	ENSP00000243326
ENSP00000223129	ENSP00000254719
ENSP00000223129	ENSP00000265056
ENSP00000223129	ENSP00000296137
ENSP00000223129	ENSP00000314129
ENSP00000223129	ENSP00000318352
ENSP00000223129	ENSP00000322957
ENSP00000223129	ENSP00000334675
ENSP00000223129	ENSP00000337088
ENSP00000223129	ENSP00000338067
ENSP00000223129	ENSP00000347232
ENSP00000223129	ENSP00000350491
ENSP00000223129	ENSP00000351284
ENSP00000223129	ENSP00000354361
ENSP00000223129	ENSP00000363021
ENSP00000223129	ENSP00000368438
ENSP00000223129	ENSP00000379607
ENSP00000223136	ENSP00000235329
ENSP00000223136	ENSP00000254442
ENSP00000223136	ENSP00000262715
ENSP00000223136	ENSP00000263150
ENSP00000223136	ENSP00000285873
ENSP00000223136	ENSP00000300589
ENSP00000223136	ENSP00000300688
ENSP00000223136	ENSP00000311344
ENSP00000223136	ENSP00000338533
ENSP00000223136	ENSP00000345656
ENSP00000223136	ENSP00000349887
ENSP00000223136	ENSP00000351813

ENSP00000223136	ENSP00000352820
ENSP00000223136	ENSP00000353059
ENSP00000223136	ENSP00000358312
ENSP00000223136	ENSP00000362688
ENSP00000223136	ENSP00000366953
ENSP00000223136	ENSP00000371888
ENSP00000223136	ENSP00000387262
ENSP00000223136	ENSP00000392330
ENSP00000223167	ENSP00000295685
ENSP00000223167	ENSP00000341838
ENSP00000223190	ENSP00000278070
ENSP00000223190	ENSP00000285599
ENSP00000223190	ENSP00000305422
ENSP00000223190	ENSP00000355759
ENSP00000223210	ENSP00000381003
ENSP00000223215	ENSP00000278937
ENSP00000223215	ENSP00000331201
ENSP00000223215	ENSP00000348762
ENSP00000223215	ENSP00000354560
ENSP00000223273	ENSP00000274030
ENSP00000223273	ENSP00000279147
ENSP00000223273	ENSP00000296577
ENSP00000223273	ENSP00000307077
ENSP00000223273	ENSP00000376946
ENSP00000223324	ENSP00000225969
ENSP00000223324	ENSP00000251038
ENSP00000223324	ENSP00000256404
ENSP00000223324	ENSP00000269143
ENSP00000223324	ENSP00000301585
ENSP00000223324	ENSP00000308275
ENSP00000223324	ENSP00000333401
ENSP00000223336	ENSP00000261070
ENSP00000223336	ENSP00000355572
ENSP00000223369	ENSP00000225567
ENSP00000223369	ENSP00000225724
ENSP00000223369	ENSP00000229314
ENSP00000223369	ENSP00000231668
ENSP00000223369	ENSP00000236192
ENSP00000223369	ENSP00000246166
ENSP00000223369	ENSP00000249647
ENSP00000223369	ENSP00000251038
ENSP00000223369	ENSP00000257700
ENSP00000223369	ENSP00000258301
ENSP00000223369	ENSP00000258654
ENSP00000223369	ENSP00000259400
ENSP00000223369	ENSP00000260818

ENSP00000223369	ENSP00000261776
ENSP00000223369	ENSP00000263116
ENSP00000223369	ENSP00000263897
ENSP00000223369	ENSP00000264157
ENSP00000223369	ENSP00000265857
ENSP00000223369	ENSP00000272322
ENSP00000223369	ENSP00000286448
ENSP00000223369	ENSP00000294179
ENSP00000223369	ENSP00000305255
ENSP00000223369	ENSP00000305647
ENSP00000223369	ENSP00000305810
ENSP00000223369	ENSP00000309163
ENSP00000223369	ENSP00000309457
ENSP00000223369	ENSP00000310521
ENSP00000223369	ENSP00000317714
ENSP00000223369	ENSP00000323377
ENSP00000223369	ENSP00000324628
ENSP00000223369	ENSP00000325002
ENSP00000223369	ENSP00000326534
ENSP00000223369	ENSP00000327650
ENSP00000223369	ENSP00000334044
ENSP00000223369	ENSP00000339484
ENSP00000223369	ENSP00000344456
ENSP00000223369	ENSP00000348215
ENSP00000223369	ENSP00000349887
ENSP00000223369	ENSP00000353073
ENSP00000223369	ENSP00000355721
ENSP00000223369	ENSP00000357887
ENSP00000223369	ENSP00000360183
ENSP00000223369	ENSP00000361699
ENSP00000223369	ENSP00000366225
ENSP00000223369	ENSP00000366249
ENSP00000223369	ENSP00000368699
ENSP00000223369	ENSP00000369810
ENSP00000223369	ENSP00000373039
ENSP00000223369	ENSP00000374529
ENSP00000223369	ENSP00000380450
ENSP00000223369	ENSP00000381293
ENSP00000223369	ENSP00000390783
ENSP00000223369	ENSP00000396152
ENSP00000223369	ENSP00000397441
ENSP00000223369	ENSP00000402240
ENSP00000223369	ENSP00000403397
ENSP00000223369	ENSP00000410416
ENSP00000223369	ENSP00000417748
ENSP00000223369	ENSP00000418661

ENSP00000223500	ENSP00000242285
ENSP00000223500	ENSP00000251038
ENSP00000223500	ENSP00000260956
ENSP00000223500	ENSP00000262995
ENSP00000223500	ENSP00000263795
ENSP00000223500	ENSP00000263856
ENSP00000223500	ENSP00000272444
ENSP00000223500	ENSP00000273398
ENSP00000223500	ENSP00000283351
ENSP00000223500	ENSP00000302239
ENSP00000223500	ENSP00000309830
ENSP00000223500	ENSP00000310440
ENSP00000223500	ENSP00000310596
ENSP00000223500	ENSP00000327077
ENSP00000223500	ENSP00000330408
ENSP00000223500	ENSP00000343742
ENSP00000223500	ENSP00000345216
ENSP00000223500	ENSP00000348081
ENSP00000223500	ENSP00000352738
ENSP00000223500	ENSP00000354522
ENSP00000223500	ENSP00000356602
ENSP00000223500	ENSP00000361202
ENSP00000223500	ENSP00000365016
ENSP00000223500	ENSP00000368699
ENSP00000223500	ENSP00000378368
ENSP00000223500	ENSP00000383431
ENSP00000223641	ENSP00000225430
ENSP00000223641	ENSP00000230449
ENSP00000223641	ENSP00000233242
ENSP00000223641	ENSP00000237530
ENSP00000223641	ENSP00000239944
ENSP00000223641	ENSP00000243253
ENSP00000223641	ENSP00000244763
ENSP00000223641	ENSP00000245923
ENSP00000223641	ENSP00000248139
ENSP00000223641	ENSP00000248566
ENSP00000223641	ENSP00000250498
ENSP00000223641	ENSP00000253861
ENSP00000223641	ENSP00000259469
ENSP00000223641	ENSP00000262213
ENSP00000223641	ENSP00000265100
ENSP00000223641	ENSP00000267484
ENSP00000223641	ENSP00000272427
ENSP00000223641	ENSP00000282074
ENSP00000223641	ENSP00000290649
ENSP00000223641	ENSP00000295770

ENSP00000223641	ENSP00000296255
ENSP00000223641	ENSP00000305892
ENSP00000223641	ENSP00000311430
ENSP00000223641	ENSP00000323377
ENSP00000223641	ENSP00000327716
ENSP00000223641	ENSP00000332326
ENSP00000223641	ENSP00000337688
ENSP00000223641	ENSP00000337838
ENSP00000223641	ENSP00000341538
ENSP00000223641	ENSP00000342181
ENSP00000223641	ENSP00000344106
ENSP00000223641	ENSP00000349887
ENSP00000223641	ENSP00000357998
ENSP00000223641	ENSP00000359000
ENSP00000223641	ENSP00000364188
ENSP00000223641	ENSP00000365131
ENSP00000223641	ENSP00000365630
ENSP00000223641	ENSP00000368959
ENSP00000223641	ENSP00000369409
ENSP00000223641	ENSP00000386717
ENSP00000223641	ENSP00000389103
ENSP00000223641	ENSP00000389934
ENSP00000223641	ENSP00000392330
ENSP00000223795	ENSP00000336762
ENSP00000223864	ENSP00000339328
ENSP00000224073	ENSP00000248566
ENSP00000224073	ENSP00000251038
ENSP00000224073	ENSP00000264335
ENSP00000224073	ENSP00000276072
ENSP00000224073	ENSP00000287598
ENSP00000224073	ENSP00000287820
ENSP00000224073	ENSP00000291688
ENSP00000224073	ENSP00000302530
ENSP00000224073	ENSP00000306245
ENSP00000224073	ENSP00000311005
ENSP00000224073	ENSP00000356331
ENSP00000224073	ENSP00000366607
ENSP00000224073	ENSP00000402338
ENSP00000224073	ENSP00000414187
ENSP00000224140	ENSP00000230050
ENSP00000224140	ENSP00000247815
ENSP00000224140	ENSP00000251038
ENSP00000224140	ENSP00000252115
ENSP00000224140	ENSP00000254037
ENSP00000224140	ENSP00000254719
ENSP00000224140	ENSP00000261531

ENSP00000224140	ENSP00000263274
ENSP00000224140	ENSP00000263360
ENSP00000224140	ENSP00000264883
ENSP00000224140	ENSP00000264951
ENSP00000224140	ENSP00000265462
ENSP00000224140	ENSP00000268043
ENSP00000224140	ENSP00000268616
ENSP00000224140	ENSP00000269499
ENSP00000224140	ENSP00000286835
ENSP00000224140	ENSP00000287038
ENSP00000224140	ENSP00000295897
ENSP00000224140	ENSP00000296328
ENSP00000224140	ENSP00000296490
ENSP00000224140	ENSP00000298139
ENSP00000224140	ENSP00000299601
ENSP00000224140	ENSP00000300648
ENSP00000224140	ENSP00000307525
ENSP00000224140	ENSP00000307786
ENSP00000224140	ENSP00000307853
ENSP00000224140	ENSP00000311873
ENSP00000224140	ENSP00000314348
ENSP00000224140	ENSP00000314949
ENSP00000224140	ENSP00000317636
ENSP00000224140	ENSP00000318195
ENSP00000224140	ENSP00000325448
ENSP00000224140	ENSP00000325863
ENSP00000224140	ENSP00000326806
ENSP00000224140	ENSP00000331310
ENSP00000224140	ENSP00000331817
ENSP00000224140	ENSP00000337839
ENSP00000224140	ENSP00000339845
ENSP00000224140	ENSP00000341243
ENSP00000224140	ENSP00000343745
ENSP00000224140	ENSP00000346015
ENSP00000224140	ENSP00000347232
ENSP00000224140	ENSP00000347978
ENSP00000224140	ENSP00000349393
ENSP00000224140	ENSP00000351284
ENSP00000224140	ENSP00000356146
ENSP00000224140	ENSP00000360532
ENSP00000224140	ENSP00000363021
ENSP00000224140	ENSP00000364289
ENSP00000224140	ENSP00000366135
ENSP00000224140	ENSP00000366843
ENSP00000224140	ENSP00000369335
ENSP00000224140	ENSP00000370150

ENSP00000224140	ENSP00000377527
ENSP00000224140	ENSP00000379933
ENSP00000224652	ENSP00000256474
ENSP00000224652	ENSP00000321636
ENSP00000224652	ENSP00000341743
ENSP00000224652	ENSP00000356405
ENSP00000224652	ENSP00000360286
ENSP00000224652	ENSP00000369757
ENSP00000224652	ENSP00000396704
ENSP00000224756	ENSP00000259895
ENSP00000224756	ENSP00000265963
ENSP00000224756	ENSP00000326514
ENSP00000224756	ENSP00000362441
ENSP00000224862	ENSP00000225504
ENSP00000224862	ENSP00000231487
ENSP00000224862	ENSP00000232501
ENSP00000224862	ENSP00000284811
ENSP00000224862	ENSP00000285021
ENSP00000224862	ENSP00000288985
ENSP00000224862	ENSP00000334675
ENSP00000224862	ENSP00000341243
ENSP00000224862	ENSP00000345530
ENSP00000224862	ENSP00000348089
ENSP00000224862	ENSP00000350708
ENSP00000224862	ENSP00000360621
ENSP00000224862	ENSP00000364270
ENSP00000224862	ENSP00000379228
ENSP00000224950	ENSP00000234420
ENSP00000224950	ENSP00000251871
ENSP00000224950	ENSP00000256379
ENSP00000224950	ENSP00000256996
ENSP00000224950	ENSP00000258648
ENSP00000224950	ENSP00000260383
ENSP00000224950	ENSP00000261424
ENSP00000224950	ENSP00000261881
ENSP00000224950	ENSP00000263043
ENSP00000224950	ENSP00000263795
ENSP00000224950	ENSP00000264926
ENSP00000224950	ENSP00000268711
ENSP00000224950	ENSP00000290663
ENSP00000224950	ENSP00000292035
ENSP00000224950	ENSP00000297347
ENSP00000224950	ENSP00000300651
ENSP00000224950	ENSP00000304370
ENSP00000224950	ENSP00000309572
ENSP00000224950	ENSP00000313171

ENSP00000224950	ENSP00000313759
ENSP00000224950	ENSP00000314949
ENSP00000224950	ENSP00000323720
ENSP00000224950	ENSP00000325612
ENSP00000224950	ENSP00000326767
ENSP00000224950	ENSP00000334148
ENSP00000224950	ENSP00000348610
ENSP00000224950	ENSP00000350249
ENSP00000224950	ENSP00000355759
ENSP00000224950	ENSP00000357047
ENSP00000224950	ENSP00000363193
ENSP00000224950	ENSP00000371419
ENSP00000225171	ENSP00000261531
ENSP00000225171	ENSP00000270538
ENSP00000225171	ENSP00000294053
ENSP00000225171	ENSP00000304350
ENSP00000225171	ENSP00000307525
ENSP00000225171	ENSP00000314499
ENSP00000225171	ENSP00000335321
ENSP00000225171	ENSP00000351137
ENSP00000225171	ENSP00000357998
ENSP00000225171	ENSP00000360532
ENSP00000225171	ENSP00000362110
ENSP00000225171	ENSP00000368565
ENSP00000225298	ENSP00000226796
ENSP00000225298	ENSP00000234677
ENSP00000225298	ENSP00000244230
ENSP00000225298	ENSP00000245539
ENSP00000225298	ENSP00000256398
ENSP00000225298	ENSP00000258455
ENSP00000225298	ENSP00000260257
ENSP00000225298	ENSP00000261531
ENSP00000225298	ENSP00000261601
ENSP00000225298	ENSP00000261708
ENSP00000225298	ENSP00000263657
ENSP00000225298	ENSP00000267425
ENSP00000225298	ENSP00000291576
ENSP00000225298	ENSP00000296792
ENSP00000225298	ENSP00000297990
ENSP00000225298	ENSP00000298292
ENSP00000225298	ENSP00000308179
ENSP00000225298	ENSP00000310590
ENSP00000225298	ENSP00000314193
ENSP00000225298	ENSP00000326981
ENSP00000225298	ENSP00000327179
ENSP00000225298	ENSP00000328854

ENSP00000225298	ENSP00000329466
ENSP00000225298	ENSP00000339095
ENSP00000225298	ENSP00000339145
ENSP00000225298	ENSP00000353246
ENSP00000225298	ENSP00000354040
ENSP00000225298	ENSP00000355541
ENSP00000225298	ENSP00000358812
ENSP00000225298	ENSP00000362105
ENSP00000225298	ENSP00000370589
ENSP00000225298	ENSP00000377944
ENSP00000225298	ENSP00000379607
ENSP00000225298	ENSP00000386134
ENSP00000225388	ENSP00000251968
ENSP00000225388	ENSP00000329466
ENSP00000225388	ENSP00000330343
ENSP00000225388	ENSP00000352738
ENSP00000225430	ENSP00000226796
ENSP00000225430	ENSP00000230050
ENSP00000225430	ENSP00000243253
ENSP00000225430	ENSP00000245539
ENSP00000225430	ENSP00000252543
ENSP00000225430	ENSP00000253099
ENSP00000225430	ENSP00000253788
ENSP00000225430	ENSP00000257879
ENSP00000225430	ENSP00000258455
ENSP00000225430	ENSP00000259469
ENSP00000225430	ENSP00000260102
ENSP00000225430	ENSP00000260443
ENSP00000225430	ENSP00000261531
ENSP00000225430	ENSP00000262584
ENSP00000225430	ENSP00000262966
ENSP00000225430	ENSP00000263360
ENSP00000225430	ENSP00000264995
ENSP00000225430	ENSP00000265100
ENSP00000225430	ENSP00000268661
ENSP00000225430	ENSP00000270625
ENSP00000225430	ENSP00000272418
ENSP00000225430	ENSP00000274242
ENSP00000225430	ENSP00000274606
ENSP00000225430	ENSP00000276603
ENSP00000225430	ENSP00000277541
ENSP00000225430	ENSP00000278572
ENSP00000225430	ENSP00000287038
ENSP00000225430	ENSP00000294189
ENSP00000225430	ENSP00000297338
ENSP00000225430	ENSP00000298283

ENSP00000225430	ENSP00000299335
ENSP00000225430	ENSP00000300151
ENSP00000225430	ENSP00000306548
ENSP00000225430	ENSP00000307889
ENSP00000225430	ENSP00000308897
ENSP00000225430	ENSP00000309334
ENSP00000225430	ENSP00000309830
ENSP00000225430	ENSP00000310040
ENSP00000225430	ENSP00000311028
ENSP00000225430	ENSP00000311430
ENSP00000225430	ENSP00000313691
ENSP00000225430	ENSP00000315693
ENSP00000225430	ENSP00000317376
ENSP00000225430	ENSP00000322419
ENSP00000225430	ENSP00000329466
ENSP00000225430	ENSP00000339095
ENSP00000225430	ENSP00000339795
ENSP00000225430	ENSP00000341538
ENSP00000225430	ENSP00000341885
ENSP00000225430	ENSP00000343392
ENSP00000225430	ENSP00000345156
ENSP00000225430	ENSP00000346015
ENSP00000225430	ENSP00000346022
ENSP00000225430	ENSP00000346027
ENSP00000225430	ENSP00000346037
ENSP00000225430	ENSP00000346050
ENSP00000225430	ENSP00000346088
ENSP00000225430	ENSP00000346725
ENSP00000225430	ENSP00000347978
ENSP00000225430	ENSP00000354525
ENSP00000225430	ENSP00000354739
ENSP00000225430	ENSP00000358831
ENSP00000225430	ENSP00000359345
ENSP00000225430	ENSP00000360286
ENSP00000225430	ENSP00000361076
ENSP00000225430	ENSP00000361800
ENSP00000225430	ENSP00000362576
ENSP00000225430	ENSP00000363018
ENSP00000225430	ENSP00000363559
ENSP00000225430	ENSP00000363676
ENSP00000225430	ENSP00000364320
ENSP00000225430	ENSP00000369757
ENSP00000225430	ENSP00000373404
ENSP00000225430	ENSP00000375730
ENSP00000225430	ENSP00000377640
ENSP00000225430	ENSP00000377865

ENSP00000225430	ENSP00000378160
ENSP00000225430	ENSP00000379607
ENSP00000225430	ENSP00000380156
ENSP00000225430	ENSP00000386717
ENSP00000225430	ENSP00000389103
ENSP00000225430	ENSP00000391723
ENSP00000225430	ENSP00000393393
ENSP00000225430	ENSP00000402338
ENSP00000225430	ENSP00000413625
ENSP00000225430	ENSP00000414321
ENSP00000225430	ENSP00000418734
ENSP00000225504	ENSP00000229179
ENSP00000225504	ENSP00000229971
ENSP00000225504	ENSP00000234827
ENSP00000225504	ENSP00000247219
ENSP00000225504	ENSP00000247970
ENSP00000225504	ENSP00000247977
ENSP00000225504	ENSP00000251038
ENSP00000225504	ENSP00000252445
ENSP00000225504	ENSP00000262173
ENSP00000225504	ENSP00000263208
ENSP00000225504	ENSP00000263390
ENSP00000225504	ENSP00000263736
ENSP00000225504	ENSP00000263857
ENSP00000225504	ENSP00000264515
ENSP00000225504	ENSP00000265990
ENSP00000225504	ENSP00000272252
ENSP00000225504	ENSP00000272444
ENSP00000225504	ENSP00000272645
ENSP00000225504	ENSP00000278412
ENSP00000225504	ENSP00000288985
ENSP00000225504	ENSP00000291386
ENSP00000225504	ENSP00000293860
ENSP00000225504	ENSP00000295321
ENSP00000225504	ENSP00000296328
ENSP00000225504	ENSP00000299543
ENSP00000225504	ENSP00000299601
ENSP00000225504	ENSP00000301788
ENSP00000225504	ENSP00000302478
ENSP00000225504	ENSP00000304593
ENSP00000225504	ENSP00000312735
ENSP00000225504	ENSP00000314949
ENSP00000225504	ENSP00000318674
ENSP00000225504	ENSP00000319104
ENSP00000225504	ENSP00000321386
ENSP00000225504	ENSP00000321927

ENSP00000225504	ENSP00000324124
ENSP00000225504	ENSP00000325677
ENSP00000225504	ENSP00000330098
ENSP00000225504	ENSP00000331111
ENSP00000225504	ENSP00000334675
ENSP00000225504	ENSP00000336868
ENSP00000225504	ENSP00000340823
ENSP00000225504	ENSP00000342889
ENSP00000225504	ENSP00000347168
ENSP00000225504	ENSP00000347345
ENSP00000225504	ENSP00000347836
ENSP00000225504	ENSP00000348089
ENSP00000225504	ENSP00000350767
ENSP00000225504	ENSP00000352738
ENSP00000225504	ENSP00000354977
ENSP00000225504	ENSP00000356405
ENSP00000225504	ENSP00000357887
ENSP00000225504	ENSP00000357907
ENSP00000225504	ENSP00000358478
ENSP00000225504	ENSP00000358497
ENSP00000225504	ENSP00000360116
ENSP00000225504	ENSP00000361465
ENSP00000225504	ENSP00000362352
ENSP00000225504	ENSP00000364270
ENSP00000225504	ENSP00000366135
ENSP00000225504	ENSP00000366271
ENSP00000225504	ENSP00000366974
ENSP00000225504	ENSP00000367029
ENSP00000225504	ENSP00000367034
ENSP00000225504	ENSP00000371475
ENSP00000225504	ENSP00000374280
ENSP00000225504	ENSP00000377969
ENSP00000225504	ENSP00000381031
ENSP00000225504	ENSP00000383503
ENSP00000225504	ENSP00000397259
ENSP00000225504	ENSP00000397636
ENSP00000225504	ENSP00000403852
ENSP00000225504	ENSP00000405636
ENSP00000225504	ENSP00000406293
ENSP00000225504	ENSP00000410530
ENSP00000225504	ENSP00000410954
ENSP00000225504	ENSP00000414110
ENSP00000225504	ENSP00000416599
ENSP00000225504	ENSP00000418111
ENSP00000225519	ENSP00000264029
ENSP00000225519	ENSP00000264705

ENSP00000225519	ENSP00000330408
ENSP00000225519	ENSP00000360268
ENSP00000225519	ENSP00000417281
ENSP00000225567	ENSP00000225724
ENSP00000225567	ENSP00000231668
ENSP00000225567	ENSP00000236192
ENSP00000225567	ENSP00000238831
ENSP00000225567	ENSP00000249647
ENSP00000225567	ENSP00000249923
ENSP00000225567	ENSP00000251038
ENSP00000225567	ENSP00000258301
ENSP00000225567	ENSP00000259400
ENSP00000225567	ENSP00000262544
ENSP00000225567	ENSP00000262812
ENSP00000225567	ENSP00000263897
ENSP00000225567	ENSP00000264028
ENSP00000225567	ENSP00000264157
ENSP00000225567	ENSP00000265175
ENSP00000225567	ENSP00000265857
ENSP00000225567	ENSP00000286448
ENSP00000225567	ENSP00000294179
ENSP00000225567	ENSP00000302783
ENSP00000225567	ENSP00000305255
ENSP00000225567	ENSP00000305647
ENSP00000225567	ENSP00000305810
ENSP00000225567	ENSP00000310521
ENSP00000225567	ENSP00000317714
ENSP00000225567	ENSP00000324628
ENSP00000225567	ENSP00000325002
ENSP00000225567	ENSP00000327309
ENSP00000225567	ENSP00000327650
ENSP00000225567	ENSP00000328079
ENSP00000225567	ENSP00000329419
ENSP00000225567	ENSP00000353564
ENSP00000225567	ENSP00000355721
ENSP00000225567	ENSP00000357048
ENSP00000225567	ENSP00000360183
ENSP00000225567	ENSP00000362399
ENSP00000225567	ENSP00000374529
ENSP00000225567	ENSP00000381293
ENSP00000225567	ENSP00000390783
ENSP00000225567	ENSP00000397441
ENSP00000225573	ENSP00000251038
ENSP00000225573	ENSP00000267064
ENSP00000225576	ENSP00000253031
ENSP00000225576	ENSP00000263116

ENSP00000225576	ENSP00000342148
ENSP00000225576	ENSP00000367615
ENSP00000225655	ENSP00000229922
ENSP00000225655	ENSP00000252699
ENSP00000225655	ENSP00000252725
ENSP00000225655	ENSP00000253861
ENSP00000225655	ENSP00000260008
ENSP00000225655	ENSP00000262300
ENSP00000225655	ENSP00000269122
ENSP00000225655	ENSP00000289373
ENSP00000225655	ENSP00000292385
ENSP00000225655	ENSP00000302790
ENSP00000225655	ENSP00000305958
ENSP00000225655	ENSP00000309629
ENSP00000225655	ENSP00000318884
ENSP00000225655	ENSP00000320924
ENSP00000225655	ENSP00000325402
ENSP00000225655	ENSP00000326238
ENSP00000225655	ENSP00000329219
ENSP00000225655	ENSP00000333950
ENSP00000225655	ENSP00000336702
ENSP00000225655	ENSP00000346791
ENSP00000225655	ENSP00000351284
ENSP00000225655	ENSP00000352425
ENSP00000225655	ENSP00000355809
ENSP00000225655	ENSP00000361467
ENSP00000225655	ENSP00000362413
ENSP00000225655	ENSP00000376410
ENSP00000225655	ENSP00000380129
ENSP00000225655	ENSP00000383623
ENSP00000225655	ENSP00000397351
ENSP00000225655	ENSP00000402084
ENSP00000225688	ENSP00000320758
ENSP00000225688	ENSP00000341551
ENSP00000225688	ENSP00000341957
ENSP00000225688	ENSP00000355133
ENSP00000225688	ENSP00000369075
ENSP00000225698	ENSP00000225983
ENSP00000225698	ENSP00000240055
ENSP00000225698	ENSP00000240316
ENSP00000225698	ENSP00000257572
ENSP00000225698	ENSP00000261168
ENSP00000225698	ENSP00000262995
ENSP00000225698	ENSP00000275015
ENSP00000225698	ENSP00000295025
ENSP00000225698	ENSP00000298097

ENSP00000225698	ENSP00000301329
ENSP00000225698	ENSP00000301585
ENSP00000225698	ENSP00000325562
ENSP00000225698	ENSP00000333568
ENSP00000225698	ENSP00000334538
ENSP00000225698	ENSP00000355865
ENSP00000225698	ENSP00000364398
ENSP00000225698	ENSP00000365175
ENSP00000225698	ENSP00000365435
ENSP00000225698	ENSP00000379607
ENSP00000225698	ENSP00000379888
ENSP00000225698	ENSP00000387477
ENSP00000225698	ENSP00000398597
ENSP00000225698	ENSP00000415363
ENSP00000225719	ENSP00000276893
ENSP00000225724	ENSP00000231668
ENSP00000225724	ENSP00000236192
ENSP00000225724	ENSP00000243918
ENSP00000225724	ENSP00000253339
ENSP00000225724	ENSP00000258301
ENSP00000225724	ENSP00000258418
ENSP00000225724	ENSP00000258654
ENSP00000225724	ENSP00000259400
ENSP00000225724	ENSP00000263116
ENSP00000225724	ENSP00000263379
ENSP00000225724	ENSP00000263897
ENSP00000225724	ENSP00000264157
ENSP00000225724	ENSP00000265175
ENSP00000225724	ENSP00000271375
ENSP00000225724	ENSP00000286448
ENSP00000225724	ENSP00000292535
ENSP00000225724	ENSP00000294179
ENSP00000225724	ENSP00000305255
ENSP00000225724	ENSP00000305459
ENSP00000225724	ENSP00000305647
ENSP00000225724	ENSP00000305810
ENSP00000225724	ENSP00000308496
ENSP00000225724	ENSP00000311825
ENSP00000225724	ENSP00000317714
ENSP00000225724	ENSP00000324628
ENSP00000225724	ENSP00000325369
ENSP00000225724	ENSP00000326869
ENSP00000225724	ENSP00000336724
ENSP00000225724	ENSP00000344055
ENSP00000225724	ENSP00000344223
ENSP00000225724	ENSP00000346139

ENSP00000225724	ENSP00000356405
ENSP00000225724	ENSP00000356437
ENSP00000225724	ENSP00000358417
ENSP00000225724	ENSP00000360183
ENSP00000225724	ENSP00000361057
ENSP00000225724	ENSP00000363349
ENSP00000225724	ENSP00000363779
ENSP00000225724	ENSP00000374529
ENSP00000225724	ENSP00000376268
ENSP00000225724	ENSP00000377769
ENSP00000225724	ENSP00000381293
ENSP00000225724	ENSP00000390783
ENSP00000225724	ENSP00000397441
ENSP00000225724	ENSP00000402240
ENSP00000225726	ENSP00000227471
ENSP00000225726	ENSP00000230582
ENSP00000225726	ENSP00000251038
ENSP00000225726	ENSP00000255389
ENSP00000225726	ENSP00000262265
ENSP00000225726	ENSP00000280350
ENSP00000225726	ENSP00000290649
ENSP00000225726	ENSP00000298292
ENSP00000225726	ENSP00000299767
ENSP00000225726	ENSP00000329411
ENSP00000225726	ENSP00000331288
ENSP00000225726	ENSP00000349887
ENSP00000225726	ENSP00000353073
ENSP00000225726	ENSP00000356989
ENSP00000225726	ENSP00000358997
ENSP00000225726	ENSP00000361076
ENSP00000225726	ENSP00000376445
ENSP00000225728	ENSP00000225916
ENSP00000225728	ENSP00000227474
ENSP00000225728	ENSP00000229595
ENSP00000225728	ENSP00000230859
ENSP00000225728	ENSP00000237380
ENSP00000225728	ENSP00000238112
ENSP00000225728	ENSP00000242480
ENSP00000225728	ENSP00000245206
ENSP00000225728	ENSP00000248566
ENSP00000225728	ENSP00000251871
ENSP00000225728	ENSP00000252622
ENSP00000225728	ENSP00000254900
ENSP00000225728	ENSP00000255764
ENSP00000225728	ENSP00000256379
ENSP00000225728	ENSP00000256398

ENSP00000225728	ENSP00000256854
ENSP00000225728	ENSP00000260129
ENSP00000225728	ENSP00000260956
ENSP00000225728	ENSP00000261245
ENSP00000225728	ENSP00000261601
ENSP00000225728	ENSP00000262367
ENSP00000225728	ENSP00000262518
ENSP00000225728	ENSP00000262608
ENSP00000225728	ENSP00000262982
ENSP00000225728	ENSP00000263390
ENSP00000225728	ENSP00000263857
ENSP00000225728	ENSP00000264501
ENSP00000225728	ENSP00000264515
ENSP00000225728	ENSP00000265073
ENSP00000225728	ENSP00000265350
ENSP00000225728	ENSP00000265351
ENSP00000225728	ENSP00000268711
ENSP00000225728	ENSP00000272645
ENSP00000225728	ENSP00000276072
ENSP00000225728	ENSP00000281928
ENSP00000225728	ENSP00000282326
ENSP00000225728	ENSP00000282884
ENSP00000225728	ENSP00000282892
ENSP00000225728	ENSP00000283882
ENSP00000225728	ENSP00000285896
ENSP00000225728	ENSP00000286317
ENSP00000225728	ENSP00000290663
ENSP00000225728	ENSP00000291688
ENSP00000225728	ENSP00000293777
ENSP00000225728	ENSP00000293860
ENSP00000225728	ENSP00000295066
ENSP00000225728	ENSP00000296328
ENSP00000225728	ENSP00000296786
ENSP00000225728	ENSP00000298937
ENSP00000225728	ENSP00000299601
ENSP00000225728	ENSP00000300651
ENSP00000225728	ENSP00000304593
ENSP00000225728	ENSP00000307850
ENSP00000225728	ENSP00000307863
ENSP00000225728	ENSP00000308944
ENSP00000225728	ENSP00000310596
ENSP00000225728	ENSP00000312735
ENSP00000225728	ENSP00000314311
ENSP00000225728	ENSP00000314949
ENSP00000225728	ENSP00000318604
ENSP00000225728	ENSP00000320566

ENSP00000225728	ENSP00000321711
ENSP00000225728	ENSP00000323720
ENSP00000225728	ENSP00000325677
ENSP00000225728	ENSP00000326767
ENSP00000225728	ENSP00000327716
ENSP00000225728	ENSP00000329029
ENSP00000225728	ENSP00000329933
ENSP00000225728	ENSP00000331111
ENSP00000225728	ENSP00000331310
ENSP00000225728	ENSP00000334188
ENSP00000225728	ENSP00000337340
ENSP00000225728	ENSP00000337405
ENSP00000225728	ENSP00000347184
ENSP00000225728	ENSP00000350967
ENSP00000225728	ENSP00000352738
ENSP00000225728	ENSP00000353622
ENSP00000225728	ENSP00000354522
ENSP00000225728	ENSP00000354742
ENSP00000225728	ENSP00000354951
ENSP00000225728	ENSP00000355205
ENSP00000225728	ENSP00000357861
ENSP00000225728	ENSP00000357887
ENSP00000225728	ENSP00000357907
ENSP00000225728	ENSP00000358064
ENSP00000225728	ENSP00000358478
ENSP00000225728	ENSP00000359290
ENSP00000225728	ENSP00000359474
ENSP00000225728	ENSP00000359518
ENSP00000225728	ENSP00000359531
ENSP00000225728	ENSP00000360025
ENSP00000225728	ENSP00000360473
ENSP00000225728	ENSP00000360515
ENSP00000225728	ENSP00000361949
ENSP00000225728	ENSP00000362532
ENSP00000225728	ENSP00000362948
ENSP00000225728	ENSP00000363193
ENSP00000225728	ENSP00000363734
ENSP00000225728	ENSP00000363779
ENSP00000225728	ENSP00000364929
ENSP00000225728	ENSP00000365025
ENSP00000225728	ENSP00000365837
ENSP00000225728	ENSP00000366135
ENSP00000225728	ENSP00000370589
ENSP00000225728	ENSP00000371236
ENSP00000225728	ENSP00000372694
ENSP00000225728	ENSP00000373715

ENSP00000225728	ENSP00000374213
ENSP00000225728	ENSP00000376204
ENSP00000225728	ENSP00000379217
ENSP00000225728	ENSP00000383503
ENSP00000225728	ENSP00000386759
ENSP00000225728	ENSP00000386992
ENSP00000225728	ENSP00000397636
ENSP00000225728	ENSP00000405636
ENSP00000225728	ENSP00000406293
ENSP00000225728	ENSP00000410530
ENSP00000225728	ENSP00000410954
ENSP00000225728	ENSP00000412319
ENSP00000225728	ENSP00000414110
ENSP00000225728	ENSP00000415678
ENSP00000225728	ENSP00000416599
ENSP00000225737	ENSP00000265717
ENSP00000225737	ENSP00000329002
ENSP00000225842	ENSP00000262854
ENSP00000225873	ENSP00000251038
ENSP00000225873	ENSP00000256216
ENSP00000225873	ENSP00000288774
ENSP00000225873	ENSP00000295030
ENSP00000225873	ENSP00000299138
ENSP00000225873	ENSP00000313432
ENSP00000225873	ENSP00000347836
ENSP00000225873	ENSP00000349016
ENSP00000225873	ENSP00000349543
ENSP00000225873	ENSP00000357051
ENSP00000225873	ENSP00000358312
ENSP00000225873	ENSP00000360116
ENSP00000225873	ENSP00000407401
ENSP00000225916	ENSP00000225983
ENSP00000225916	ENSP00000229595
ENSP00000225916	ENSP00000231487
ENSP00000225916	ENSP00000233146
ENSP00000225916	ENSP00000234040
ENSP00000225916	ENSP00000234111
ENSP00000225916	ENSP00000234296
ENSP00000225916	ENSP00000237380
ENSP00000225916	ENSP00000247219
ENSP00000225916	ENSP00000247843
ENSP00000225916	ENSP00000247970
ENSP00000225916	ENSP00000250615
ENSP00000225916	ENSP00000250894
ENSP00000225916	ENSP00000251871
ENSP00000225916	ENSP00000252996

ENSP00000225916	ENSP00000254193
ENSP00000225916	ENSP00000256379
ENSP00000225916	ENSP00000256398
ENSP00000225916	ENSP00000256442
ENSP00000225916	ENSP00000256474
ENSP00000225916	ENSP00000256897
ENSP00000225916	ENSP00000258648
ENSP00000225916	ENSP00000261819
ENSP00000225916	ENSP00000261951
ENSP00000225916	ENSP00000262300
ENSP00000225916	ENSP00000262367
ENSP00000225916	ENSP00000263205
ENSP00000225916	ENSP00000263390
ENSP00000225916	ENSP00000263620
ENSP00000225916	ENSP00000263974
ENSP00000225916	ENSP00000264183
ENSP00000225916	ENSP00000264193
ENSP00000225916	ENSP00000265354
ENSP00000225916	ENSP00000265537
ENSP00000225916	ENSP00000265990
ENSP00000225916	ENSP00000269127
ENSP00000225916	ENSP00000272645
ENSP00000225916	ENSP00000276072
ENSP00000225916	ENSP00000277541
ENSP00000225916	ENSP00000278412
ENSP00000225916	ENSP00000279873
ENSP00000225916	ENSP00000282892
ENSP00000225916	ENSP00000286317
ENSP00000225916	ENSP00000287239
ENSP00000225916	ENSP00000290663
ENSP00000225916	ENSP00000292035
ENSP00000225916	ENSP00000294168
ENSP00000225916	ENSP00000297431
ENSP00000225916	ENSP00000298937
ENSP00000225916	ENSP00000299424
ENSP00000225916	ENSP00000300648
ENSP00000225916	ENSP00000300651
ENSP00000225916	ENSP00000301764
ENSP00000225916	ENSP00000301887
ENSP00000225916	ENSP00000303315
ENSP00000225916	ENSP00000305422
ENSP00000225916	ENSP00000305790
ENSP00000225916	ENSP00000306983
ENSP00000225916	ENSP00000307208
ENSP00000225916	ENSP00000307684
ENSP00000225916	ENSP00000308236

ENSP00000225916	ENSP00000309555
ENSP00000225916	ENSP00000310649
ENSP00000225916	ENSP00000312735
ENSP00000225916	ENSP00000313420
ENSP00000225916	ENSP00000314949
ENSP00000225916	ENSP00000316114
ENSP00000225916	ENSP00000316786
ENSP00000225916	ENSP00000319104
ENSP00000225916	ENSP00000319169
ENSP00000225916	ENSP00000322579
ENSP00000225916	ENSP00000323720
ENSP00000225916	ENSP00000324444
ENSP00000225916	ENSP00000325612
ENSP00000225916	ENSP00000326550
ENSP00000225916	ENSP00000329411
ENSP00000225916	ENSP00000331310
ENSP00000225916	ENSP00000331879
ENSP00000225916	ENSP00000334061
ENSP00000225916	ENSP00000334280
ENSP00000225916	ENSP00000335044
ENSP00000225916	ENSP00000336750
ENSP00000225916	ENSP00000339353
ENSP00000225916	ENSP00000340271
ENSP00000225916	ENSP00000343392
ENSP00000225916	ENSP00000344456
ENSP00000225916	ENSP00000344504
ENSP00000225916	ENSP00000344537
ENSP00000225916	ENSP00000345681
ENSP00000225916	ENSP00000347602
ENSP00000225916	ENSP00000347733
ENSP00000225916	ENSP00000348069
ENSP00000225916	ENSP00000348610
ENSP00000225916	ENSP00000349213
ENSP00000225916	ENSP00000350078
ENSP00000225916	ENSP00000350283
ENSP00000225916	ENSP00000350967
ENSP00000225916	ENSP00000351284
ENSP00000225916	ENSP00000351490
ENSP00000225916	ENSP00000352257
ENSP00000225916	ENSP00000353622
ENSP00000225916	ENSP00000353793
ENSP00000225916	ENSP00000353804
ENSP00000225916	ENSP00000354633
ENSP00000225916	ENSP00000354673
ENSP00000225916	ENSP00000355205
ENSP00000225916	ENSP00000355884

ENSP00000225916	ENSP00000355958
ENSP00000225916	ENSP00000356405
ENSP00000225916	ENSP00000356448
ENSP00000225916	ENSP00000356530
ENSP00000225916	ENSP00000356848
ENSP00000225916	ENSP00000357047
ENSP00000225916	ENSP00000357442
ENSP00000225916	ENSP00000358854
ENSP00000225916	ENSP00000358886
ENSP00000225916	ENSP00000359290
ENSP00000225916	ENSP00000359837
ENSP00000225916	ENSP00000360286
ENSP00000225916	ENSP00000360515
ENSP00000225916	ENSP00000360621
ENSP00000225916	ENSP00000360676
ENSP00000225916	ENSP00000361998
ENSP00000225916	ENSP00000362068
ENSP00000225916	ENSP00000362948
ENSP00000225916	ENSP00000363193
ENSP00000225916	ENSP00000363779
ENSP00000225916	ENSP00000364815
ENSP00000225916	ENSP00000366974
ENSP00000225916	ENSP00000367406
ENSP00000225916	ENSP00000368686
ENSP00000225916	ENSP00000369695
ENSP00000225916	ENSP00000369716
ENSP00000225916	ENSP00000373340
ENSP00000225916	ENSP00000377218
ENSP00000225916	ENSP00000377747
ENSP00000225916	ENSP00000379372
ENSP00000225916	ENSP00000381098
ENSP00000225916	ENSP00000381657
ENSP00000225916	ENSP00000388320
ENSP00000225916	ENSP00000392028
ENSP00000225916	ENSP00000396749
ENSP00000225916	ENSP00000397259
ENSP00000225916	ENSP00000401121
ENSP00000225916	ENSP00000401445
ENSP00000225916	ENSP00000403359
ENSP00000225916	ENSP00000415316
ENSP00000225927	ENSP00000259253
ENSP00000225927	ENSP00000272227
ENSP00000225927	ENSP00000273308
ENSP00000225927	ENSP00000323313
ENSP00000225927	ENSP00000326699
ENSP00000225927	ENSP00000350102

ENSP00000225927	ENSP00000356599
ENSP00000225969	ENSP00000226796
ENSP00000225969	ENSP00000241600
ENSP00000225969	ENSP00000253099
ENSP00000225969	ENSP00000256404
ENSP00000225969	ENSP00000258383
ENSP00000225969	ENSP00000260102
ENSP00000225969	ENSP00000261819
ENSP00000225969	ENSP00000264995
ENSP00000225969	ENSP00000279242
ENSP00000225969	ENSP00000288937
ENSP00000225969	ENSP00000296102
ENSP00000225969	ENSP00000300151
ENSP00000225969	ENSP00000301585
ENSP00000225969	ENSP00000302768
ENSP00000225969	ENSP00000306548
ENSP00000225969	ENSP00000308275
ENSP00000225969	ENSP00000308897
ENSP00000225969	ENSP00000312311
ENSP00000225969	ENSP00000315017
ENSP00000225969	ENSP00000316879
ENSP00000225969	ENSP00000324122
ENSP00000225969	ENSP00000333401
ENSP00000225969	ENSP00000340281
ENSP00000225969	ENSP00000342026
ENSP00000225969	ENSP00000347087
ENSP00000225969	ENSP00000354525
ENSP00000225969	ENSP00000360498
ENSP00000225969	ENSP00000370930
ENSP00000225969	ENSP00000373404
ENSP00000225969	ENSP00000377486
ENSP00000225969	ENSP00000417602
ENSP00000225972	ENSP00000255465
ENSP00000225972	ENSP00000263753
ENSP00000225972	ENSP00000381891
ENSP00000225983	ENSP00000226574
ENSP00000225983	ENSP00000227524
ENSP00000225983	ENSP00000228318
ENSP00000225983	ENSP00000229595
ENSP00000225983	ENSP00000231449
ENSP00000225983	ENSP00000231572
ENSP00000225983	ENSP00000232014
ENSP00000225983	ENSP00000233084
ENSP00000225983	ENSP00000233146
ENSP00000225983	ENSP00000237530
ENSP00000225983	ENSP00000245206

ENSP00000225983	ENSP00000249923
ENSP00000225983	ENSP00000252699
ENSP00000225983	ENSP00000253363
ENSP00000225983	ENSP00000253792
ENSP00000225983	ENSP00000253794
ENSP00000225983	ENSP00000254900
ENSP00000225983	ENSP00000256398
ENSP00000225983	ENSP00000257566
ENSP00000225983	ENSP00000259803
ENSP00000225983	ENSP00000260665
ENSP00000225983	ENSP00000261712
ENSP00000225983	ENSP00000261819
ENSP00000225983	ENSP00000261973
ENSP00000225983	ENSP00000262027
ENSP00000225983	ENSP00000262030
ENSP00000225983	ENSP00000262032
ENSP00000225983	ENSP00000262238
ENSP00000225983	ENSP00000262518
ENSP00000225983	ENSP00000262608
ENSP00000225983	ENSP00000262776
ENSP00000225983	ENSP00000262803
ENSP00000225983	ENSP00000262850
ENSP00000225983	ENSP00000262982
ENSP00000225983	ENSP00000263201
ENSP00000225983	ENSP00000263974
ENSP00000225983	ENSP00000264161
ENSP00000225983	ENSP00000264335
ENSP00000225983	ENSP00000264639
ENSP00000225983	ENSP00000265056
ENSP00000225983	ENSP00000268379
ENSP00000225983	ENSP00000269122
ENSP00000225983	ENSP00000270625
ENSP00000225983	ENSP00000271555
ENSP00000225983	ENSP00000272227
ENSP00000225983	ENSP00000275015
ENSP00000225983	ENSP00000277865
ENSP00000225983	ENSP00000278412
ENSP00000225983	ENSP00000278572
ENSP00000225983	ENSP00000282050
ENSP00000225983	ENSP00000282516
ENSP00000225983	ENSP00000287598
ENSP00000225983	ENSP00000290158
ENSP00000225983	ENSP00000292055
ENSP00000225983	ENSP00000294168
ENSP00000225983	ENSP00000295901
ENSP00000225983	ENSP00000296255

ENSP00000225983	ENSP00000296755
ENSP00000225983	ENSP00000296785
ENSP00000225983	ENSP00000296930
ENSP00000225983	ENSP00000297185
ENSP00000225983	ENSP00000298532
ENSP00000225983	ENSP00000299767
ENSP00000225983	ENSP00000300648
ENSP00000225983	ENSP00000301030
ENSP00000225983	ENSP00000301764
ENSP00000225983	ENSP00000302886
ENSP00000225983	ENSP00000304350
ENSP00000225983	ENSP00000304592
ENSP00000225983	ENSP00000305790
ENSP00000225983	ENSP00000305976
ENSP00000225983	ENSP00000307208
ENSP00000225983	ENSP00000307567
ENSP00000225983	ENSP00000307889
ENSP00000225983	ENSP00000309474
ENSP00000225983	ENSP00000309555
ENSP00000225983	ENSP00000310040
ENSP00000225983	ENSP00000310129
ENSP00000225983	ENSP00000310668
ENSP00000225983	ENSP00000311430
ENSP00000225983	ENSP00000311747
ENSP00000225983	ENSP00000311816
ENSP00000225983	ENSP00000313420
ENSP00000225983	ENSP00000316328
ENSP00000225983	ENSP00000317334
ENSP00000225983	ENSP00000317468
ENSP00000225983	ENSP00000318861
ENSP00000225983	ENSP00000319169
ENSP00000225983	ENSP00000320566
ENSP00000225983	ENSP00000321503
ENSP00000225983	ENSP00000324560
ENSP00000225983	ENSP00000327070
ENSP00000225983	ENSP00000327213
ENSP00000225983	ENSP00000327821
ENSP00000225983	ENSP00000329419
ENSP00000225983	ENSP00000329933
ENSP00000225983	ENSP00000333568
ENSP00000225983	ENSP00000333667
ENSP00000225983	ENSP00000335321
ENSP00000225983	ENSP00000336712
ENSP00000225983	ENSP00000336842
ENSP00000225983	ENSP00000336927
ENSP00000225983	ENSP00000338157

ENSP00000225983	ENSP00000338413
ENSP00000225983	ENSP00000339527
ENSP00000225983	ENSP00000339795
ENSP00000225983	ENSP00000340019
ENSP00000225983	ENSP00000340466
ENSP00000225983	ENSP00000342026
ENSP00000225983	ENSP00000342056
ENSP00000225983	ENSP00000343535
ENSP00000225983	ENSP00000343745
ENSP00000225983	ENSP00000345681
ENSP00000225983	ENSP00000346022
ENSP00000225983	ENSP00000346050
ENSP00000225983	ENSP00000346067
ENSP00000225983	ENSP00000347733
ENSP00000225983	ENSP00000348010
ENSP00000225983	ENSP00000348551
ENSP00000225983	ENSP00000349213
ENSP00000225983	ENSP00000349748
ENSP00000225983	ENSP00000350767
ENSP00000225983	ENSP00000351284
ENSP00000225983	ENSP00000352257
ENSP00000225983	ENSP00000352400
ENSP00000225983	ENSP00000352514
ENSP00000225983	ENSP00000352516
ENSP00000225983	ENSP00000353622
ENSP00000225983	ENSP00000354125
ENSP00000225983	ENSP00000354522
ENSP00000225983	ENSP00000354586
ENSP00000225983	ENSP00000354720
ENSP00000225983	ENSP00000355011
ENSP00000225983	ENSP00000355205
ENSP00000225983	ENSP00000355759
ENSP00000225983	ENSP00000355890
ENSP00000225983	ENSP00000356520
ENSP00000225983	ENSP00000357459
ENSP00000225983	ENSP00000357887
ENSP00000225983	ENSP00000358417
ENSP00000225983	ENSP00000358716
ENSP00000225983	ENSP00000358857
ENSP00000225983	ENSP00000359345
ENSP00000225983	ENSP00000359552
ENSP00000225983	ENSP00000359727
ENSP00000225983	ENSP00000360268
ENSP00000225983	ENSP00000360532
ENSP00000225983	ENSP00000361076
ENSP00000225983	ENSP00000361202

ENSP00000225983	ENSP00000361686
ENSP00000225983	ENSP00000362413
ENSP00000225983	ENSP00000362592
ENSP00000225983	ENSP00000362744
ENSP00000225983	ENSP00000362824
ENSP00000225983	ENSP00000363018
ENSP00000225983	ENSP00000363205
ENSP00000225983	ENSP00000363745
ENSP00000225983	ENSP00000363804
ENSP00000225983	ENSP00000364188
ENSP00000225983	ENSP00000364794
ENSP00000225983	ENSP00000365877
ENSP00000225983	ENSP00000366607
ENSP00000225983	ENSP00000367299
ENSP00000225983	ENSP00000367705
ENSP00000225983	ENSP00000368438
ENSP00000225983	ENSP00000368646
ENSP00000225983	ENSP00000370023
ENSP00000225983	ENSP00000370826
ENSP00000225983	ENSP00000371607
ENSP00000225983	ENSP00000374213
ENSP00000225983	ENSP00000375986
ENSP00000225983	ENSP00000376309
ENSP00000225983	ENSP00000377954
ENSP00000225983	ENSP00000379888
ENSP00000225983	ENSP00000379933
ENSP00000225983	ENSP00000382392
ENSP00000225983	ENSP00000386759
ENSP00000225983	ENSP00000392270
ENSP00000225983	ENSP00000396976
ENSP00000225983	ENSP00000404121
ENSP00000225983	ENSP00000408957
ENSP00000225983	ENSP00000416892
ENSP00000226091	ENSP00000260130
ENSP00000226091	ENSP00000284811
ENSP00000226091	ENSP00000311005
ENSP00000226091	ENSP00000348550
ENSP00000226091	ENSP00000363921
ENSP00000226091	ENSP00000381098
ENSP00000226091	ENSP00000392423
ENSP00000226091	ENSP00000394794
ENSP00000226105	ENSP00000227474
ENSP00000226105	ENSP00000238616
ENSP00000226105	ENSP00000251038
ENSP00000226105	ENSP00000252622
ENSP00000226105	ENSP00000254605

ENSP00000226105	ENSP00000255389
ENSP00000226105	ENSP00000256031
ENSP00000226105	ENSP00000257177
ENSP00000226105	ENSP00000261396
ENSP00000226105	ENSP00000261609
ENSP00000226105	ENSP00000262077
ENSP00000226105	ENSP00000263710
ENSP00000226105	ENSP00000264276
ENSP00000226105	ENSP00000264883
ENSP00000226105	ENSP00000264951
ENSP00000226105	ENSP00000285896
ENSP00000226105	ENSP00000296577
ENSP00000226105	ENSP00000305721
ENSP00000226105	ENSP00000316032
ENSP00000226105	ENSP00000317334
ENSP00000226105	ENSP00000343701
ENSP00000226105	ENSP00000345144
ENSP00000226105	ENSP00000346453
ENSP00000226105	ENSP00000348107
ENSP00000226105	ENSP00000360286
ENSP00000226105	ENSP00000364293
ENSP00000226105	ENSP00000364582
ENSP00000226105	ENSP00000365811
ENSP00000226105	ENSP00000366607
ENSP00000226105	ENSP00000367766
ENSP00000226105	ENSP00000373715
ENSP00000226105	ENSP00000378624
ENSP00000226105	ENSP00000390158
ENSP00000226218	ENSP00000234040
ENSP00000226218	ENSP00000264708
ENSP00000226218	ENSP00000265023
ENSP00000226218	ENSP00000265171
ENSP00000226218	ENSP00000277865
ENSP00000226218	ENSP00000288828
ENSP00000226218	ENSP00000297350
ENSP00000226218	ENSP00000327647
ENSP00000226218	ENSP00000339328
ENSP00000226218	ENSP00000348602
ENSP00000226218	ENSP00000350512
ENSP00000226218	ENSP00000354782
ENSP00000226218	ENSP00000355896
ENSP00000226218	ENSP00000368646
ENSP00000226218	ENSP00000375730
ENSP00000226230	ENSP00000261507
ENSP00000226230	ENSP00000352708
ENSP00000226247	ENSP00000266744

ENSP00000226247	ENSP00000363708
ENSP00000226284	ENSP00000227758
ENSP00000226284	ENSP00000242285
ENSP00000226284	ENSP00000256658
ENSP00000226284	ENSP00000349298
ENSP00000226284	ENSP00000350199
ENSP00000226284	ENSP00000393596
ENSP00000226299	ENSP00000251968
ENSP00000226299	ENSP00000263985
ENSP00000226299	ENSP00000264029
ENSP00000226299	ENSP00000280362
ENSP00000226299	ENSP00000301021
ENSP00000226299	ENSP00000340019
ENSP00000226299	ENSP00000348565
ENSP00000226299	ENSP00000348877
ENSP00000226299	ENSP00000352928
ENSP00000226299	ENSP00000379933
ENSP00000226382	ENSP00000341679
ENSP00000226382	ENSP00000390783
ENSP00000226432	ENSP00000233616
ENSP00000226432	ENSP00000251038
ENSP00000226432	ENSP00000306124
ENSP00000226432	ENSP00000359552
ENSP00000226522	ENSP00000234170
ENSP00000226522	ENSP00000256429
ENSP00000226522	ENSP00000262850
ENSP00000226522	ENSP00000274353
ENSP00000226522	ENSP00000278671
ENSP00000226522	ENSP00000305721
ENSP00000226522	ENSP00000355153
ENSP00000226522	ENSP00000357288
ENSP00000226522	ENSP00000358423
ENSP00000226522	ENSP00000358918
ENSP00000226574	ENSP00000227163
ENSP00000226574	ENSP00000228284
ENSP00000226574	ENSP00000239849
ENSP00000226574	ENSP00000244565
ENSP00000226574	ENSP00000248673
ENSP00000226574	ENSP00000251871
ENSP00000226574	ENSP00000259808
ENSP00000226574	ENSP00000261884
ENSP00000226574	ENSP00000262238
ENSP00000226574	ENSP00000263056
ENSP00000226574	ENSP00000263401
ENSP00000226574	ENSP00000264639
ENSP00000226574	ENSP00000264705

ENSP00000226574	ENSP00000265960
ENSP00000226574	ENSP00000267935
ENSP00000226574	ENSP00000268058
ENSP00000226574	ENSP00000268638
ENSP00000226574	ENSP00000269122
ENSP00000226574	ENSP00000271002
ENSP00000226574	ENSP00000274458
ENSP00000226574	ENSP00000275015
ENSP00000226574	ENSP00000277541
ENSP00000226574	ENSP00000278572
ENSP00000226574	ENSP00000278980
ENSP00000226574	ENSP00000289431
ENSP00000226574	ENSP00000295025
ENSP00000226574	ENSP00000295897
ENSP00000226574	ENSP00000297185
ENSP00000226574	ENSP00000304133
ENSP00000226574	ENSP00000304544
ENSP00000226574	ENSP00000304803
ENSP00000226574	ENSP00000305422
ENSP00000226574	ENSP00000306245
ENSP00000226574	ENSP00000307900
ENSP00000226574	ENSP00000308236
ENSP00000226574	ENSP00000311513
ENSP00000226574	ENSP00000313420
ENSP00000226574	ENSP00000314949
ENSP00000226574	ENSP00000317891
ENSP00000226574	ENSP00000318115
ENSP00000226574	ENSP00000321203
ENSP00000226574	ENSP00000325376
ENSP00000226574	ENSP00000325663
ENSP00000226574	ENSP00000325690
ENSP00000226574	ENSP00000329357
ENSP00000226574	ENSP00000329419
ENSP00000226574	ENSP00000329967
ENSP00000226574	ENSP00000333122
ENSP00000226574	ENSP00000334061
ENSP00000226574	ENSP00000337088
ENSP00000226574	ENSP00000341805
ENSP00000226574	ENSP00000343701
ENSP00000226574	ENSP00000344352
ENSP00000226574	ENSP00000344456
ENSP00000226574	ENSP00000348054
ENSP00000226574	ENSP00000348551
ENSP00000226574	ENSP00000349748
ENSP00000226574	ENSP00000349959
ENSP00000226574	ENSP00000350283

ENSP00000226574	ENSP00000350512
ENSP00000226574	ENSP00000351894
ENSP00000226574	ENSP00000352673
ENSP00000226574	ENSP00000352738
ENSP00000226574	ENSP00000353622
ENSP00000226574	ENSP00000354346
ENSP00000226574	ENSP00000355759
ENSP00000226574	ENSP00000356623
ENSP00000226574	ENSP00000356713
ENSP00000226574	ENSP00000358812
ENSP00000226574	ENSP00000358997
ENSP00000226574	ENSP00000359206
ENSP00000226574	ENSP00000359727
ENSP00000226574	ENSP00000364893
ENSP00000226574	ENSP00000366032
ENSP00000226574	ENSP00000366607
ENSP00000226574	ENSP00000366915
ENSP00000226574	ENSP00000367454
ENSP00000226574	ENSP00000369293
ENSP00000226574	ENSP00000370984
ENSP00000226574	ENSP00000371475
ENSP00000226574	ENSP00000376903
ENSP00000226574	ENSP00000377218
ENSP00000226574	ENSP00000378503
ENSP00000226574	ENSP00000379888
ENSP00000226574	ENSP00000381461
ENSP00000226574	ENSP00000384026
ENSP00000226574	ENSP00000400476
ENSP00000226574	ENSP00000415836
ENSP00000226574	ENSP00000419475
ENSP00000226730	ENSP00000278572
ENSP00000226730	ENSP00000338010
ENSP00000226730	ENSP00000369293
ENSP00000226760	ENSP00000276603
ENSP00000226760	ENSP00000366395
ENSP00000226796	ENSP00000227524
ENSP00000226796	ENSP00000230050
ENSP00000226796	ENSP00000230859
ENSP00000226796	ENSP00000232888
ENSP00000226796	ENSP00000233078
ENSP00000226796	ENSP00000235090
ENSP00000226796	ENSP00000248450
ENSP00000226796	ENSP00000251289
ENSP00000226796	ENSP00000252115
ENSP00000226796	ENSP00000253108
ENSP00000226796	ENSP00000253237

ENSP00000226796	ENSP00000253363
ENSP00000226796	ENSP00000254037
ENSP00000226796	ENSP00000254605
ENSP00000226796	ENSP00000260956
ENSP00000226796	ENSP00000261015
ENSP00000226796	ENSP00000261637
ENSP00000226796	ENSP00000261741
ENSP00000226796	ENSP00000262056
ENSP00000226796	ENSP00000263857
ENSP00000226796	ENSP00000265753
ENSP00000226796	ENSP00000265758
ENSP00000226796	ENSP00000267425
ENSP00000226796	ENSP00000271628
ENSP00000226796	ENSP00000274606
ENSP00000226796	ENSP00000285873
ENSP00000226796	ENSP00000291576
ENSP00000226796	ENSP00000296792
ENSP00000226796	ENSP00000296802
ENSP00000226796	ENSP00000297579
ENSP00000226796	ENSP00000298746
ENSP00000226796	ENSP00000301364
ENSP00000226796	ENSP00000301788
ENSP00000226796	ENSP00000302768
ENSP00000226796	ENSP00000302896
ENSP00000226796	ENSP00000304370
ENSP00000226796	ENSP00000305702
ENSP00000226796	ENSP00000308179
ENSP00000226796	ENSP00000308332
ENSP00000226796	ENSP00000308533
ENSP00000226796	ENSP00000309334
ENSP00000226796	ENSP00000311083
ENSP00000226796	ENSP00000312981
ENSP00000226796	ENSP00000314193
ENSP00000226796	ENSP00000314311
ENSP00000226796	ENSP00000314348
ENSP00000226796	ENSP00000315182
ENSP00000226796	ENSP00000316879
ENSP00000226796	ENSP00000317992
ENSP00000226796	ENSP00000318195
ENSP00000226796	ENSP00000320917
ENSP00000226796	ENSP00000321326
ENSP00000226796	ENSP00000321706
ENSP00000226796	ENSP00000322016
ENSP00000226796	ENSP00000322570
ENSP00000226796	ENSP00000323302
ENSP00000226796	ENSP00000324122

ENSP00000226796	ENSP00000325690
ENSP00000226796	ENSP00000326806
ENSP00000226796	ENSP00000326981
ENSP00000226796	ENSP00000327179
ENSP00000226796	ENSP00000331817
ENSP00000226796	ENSP00000331879
ENSP00000226796	ENSP00000332198
ENSP00000226796	ENSP00000334148
ENSP00000226796	ENSP00000336868
ENSP00000226796	ENSP00000338862
ENSP00000226796	ENSP00000339845
ENSP00000226796	ENSP00000340281
ENSP00000226796	ENSP00000343103
ENSP00000226796	ENSP00000343745
ENSP00000226796	ENSP00000344393
ENSP00000226796	ENSP00000345917
ENSP00000226796	ENSP00000346120
ENSP00000226796	ENSP00000347087
ENSP00000226796	ENSP00000347345
ENSP00000226796	ENSP00000350071
ENSP00000226796	ENSP00000351100
ENSP00000226796	ENSP00000351697
ENSP00000226796	ENSP00000352738
ENSP00000226796	ENSP00000354812
ENSP00000226796	ENSP00000356290
ENSP00000226796	ENSP00000356811
ENSP00000226796	ENSP00000358563
ENSP00000226796	ENSP00000358716
ENSP00000226796	ENSP00000358812
ENSP00000226796	ENSP00000360031
ENSP00000226796	ENSP00000361076
ENSP00000226796	ENSP00000361949
ENSP00000226796	ENSP00000361982
ENSP00000226796	ENSP00000362688
ENSP00000226796	ENSP00000362900
ENSP00000226796	ENSP00000363308
ENSP00000226796	ENSP00000363313
ENSP00000226796	ENSP00000366974
ENSP00000226796	ENSP00000368349
ENSP00000226796	ENSP00000368887
ENSP00000226796	ENSP00000369162
ENSP00000226796	ENSP00000370083
ENSP00000226796	ENSP00000370119
ENSP00000226796	ENSP00000370589
ENSP00000226796	ENSP00000371169
ENSP00000226796	ENSP00000372199

ENSP00000226796	ENSP00000374390
ENSP00000226796	ENSP00000376309
ENSP00000226796	ENSP00000377617
ENSP00000226796	ENSP00000378160
ENSP00000226796	ENSP00000379607
ENSP00000226796	ENSP00000379888
ENSP00000226796	ENSP00000380982
ENSP00000226796	ENSP00000382392
ENSP00000226796	ENSP00000382779
ENSP00000226796	ENSP00000384302
ENSP00000226796	ENSP00000393241
ENSP00000226796	ENSP00000401371
ENSP00000226796	ENSP00000403175
ENSP00000226796	ENSP00000412583
ENSP00000226796	ENSP00000416110
ENSP00000226796	ENSP00000416959
ENSP00000226798	ENSP00000227524
ENSP00000226798	ENSP00000228284
ENSP00000226798	ENSP00000292535
ENSP00000226798	ENSP00000304370
ENSP00000226798	ENSP00000334148
ENSP00000226798	ENSP00000387006
ENSP00000226840	ENSP00000232607
ENSP00000226840	ENSP00000251038
ENSP00000226840	ENSP00000263881
ENSP00000226840	ENSP00000270776
ENSP00000226951	ENSP00000316460
ENSP00000227163	ENSP00000232014
ENSP00000227163	ENSP00000262367
ENSP00000227163	ENSP00000263620
ENSP00000227163	ENSP00000264709
ENSP00000227163	ENSP00000265372
ENSP00000227163	ENSP00000267163
ENSP00000227163	ENSP00000268058
ENSP00000227163	ENSP00000268638
ENSP00000227163	ENSP00000268679
ENSP00000227163	ENSP00000278412
ENSP00000227163	ENSP00000283921
ENSP00000227163	ENSP00000291009
ENSP00000227163	ENSP00000294702
ENSP00000227163	ENSP00000305422
ENSP00000227163	ENSP00000306245
ENSP00000227163	ENSP00000328207
ENSP00000227163	ENSP00000345681
ENSP00000227163	ENSP00000345702
ENSP00000227163	ENSP00000353344

ENSP00000227163	ENSP00000353622
ENSP00000227163	ENSP00000367797
ENSP00000227163	ENSP00000367972
ENSP00000227163	ENSP00000381891
ENSP00000227163	ENSP00000385361
ENSP00000227163	ENSP00000386165
ENSP00000227163	ENSP00000395535
ENSP00000227163	ENSP00000414187
ENSP00000227266	ENSP00000357484
ENSP00000227266	ENSP00000380349
ENSP00000227322	ENSP00000298852
ENSP00000227322	ENSP00000309831
ENSP00000227348	ENSP00000345731
ENSP00000227348	ENSP00000349486
ENSP00000227471	ENSP00000229314
ENSP00000227471	ENSP00000245615
ENSP00000227471	ENSP00000295770
ENSP00000227471	ENSP00000307275
ENSP00000227471	ENSP00000320309
ENSP00000227471	ENSP00000336994
ENSP00000227471	ENSP00000337053
ENSP00000227471	ENSP00000350263
ENSP00000227471	ENSP00000355963
ENSP00000227471	ENSP00000358997
ENSP00000227471	ENSP00000361057
ENSP00000227471	ENSP00000364188
ENSP00000227471	ENSP00000365567
ENSP00000227471	ENSP00000369618
ENSP00000227471	ENSP00000381237
ENSP00000227474	ENSP00000229214
ENSP00000227474	ENSP00000244051
ENSP00000227474	ENSP00000254803
ENSP00000227474	ENSP00000256383
ENSP00000227474	ENSP00000256398
ENSP00000227474	ENSP00000256854
ENSP00000227474	ENSP00000260129
ENSP00000227474	ENSP00000260210
ENSP00000227474	ENSP00000260956
ENSP00000227474	ENSP00000261758
ENSP00000227474	ENSP00000261868
ENSP00000227474	ENSP00000263576
ENSP00000227474	ENSP00000263657
ENSP00000227474	ENSP00000264670
ENSP00000227474	ENSP00000264951
ENSP00000227474	ENSP00000273962
ENSP00000227474	ENSP00000275605

ENSP00000227474	ENSP00000282276
ENSP00000227474	ENSP00000286317
ENSP00000227474	ENSP00000296577
ENSP00000227474	ENSP00000297990
ENSP00000227474	ENSP00000298310
ENSP00000227474	ENSP00000298746
ENSP00000227474	ENSP00000298937
ENSP00000227474	ENSP00000300648
ENSP00000227474	ENSP00000302896
ENSP00000227474	ENSP00000303515
ENSP00000227474	ENSP00000304593
ENSP00000227474	ENSP00000307863
ENSP00000227474	ENSP00000310129
ENSP00000227474	ENSP00000310596
ENSP00000227474	ENSP00000311977
ENSP00000227474	ENSP00000312356
ENSP00000227474	ENSP00000314311
ENSP00000227474	ENSP00000314441
ENSP00000227474	ENSP00000318604
ENSP00000227474	ENSP00000324122
ENSP00000227474	ENSP00000324956
ENSP00000227474	ENSP00000326981
ENSP00000227474	ENSP00000327821
ENSP00000227474	ENSP00000328671
ENSP00000227474	ENSP00000334188
ENSP00000227474	ENSP00000338862
ENSP00000227474	ENSP00000339881
ENSP00000227474	ENSP00000345530
ENSP00000227474	ENSP00000346453
ENSP00000227474	ENSP00000348107
ENSP00000227474	ENSP00000350967
ENSP00000227474	ENSP00000352678
ENSP00000227474	ENSP00000354812
ENSP00000227474	ENSP00000356476
ENSP00000227474	ENSP00000358563
ENSP00000227474	ENSP00000358812
ENSP00000227474	ENSP00000361982
ENSP00000227474	ENSP00000363779
ENSP00000227474	ENSP00000364293
ENSP00000227474	ENSP00000364320
ENSP00000227474	ENSP00000370589
ENSP00000227474	ENSP00000379228
ENSP00000227474	ENSP00000382595
ENSP00000227474	ENSP00000412922
ENSP00000227507	ENSP00000228872
ENSP00000227507	ENSP00000229758

ENSP00000227507	ENSP00000231487
ENSP00000227507	ENSP00000235090
ENSP00000227507	ENSP00000244741
ENSP00000227507	ENSP00000248553
ENSP00000227507	ENSP00000256398
ENSP00000227507	ENSP00000256897
ENSP00000227507	ENSP00000257430
ENSP00000227507	ENSP00000257934
ENSP00000227507	ENSP00000260187
ENSP00000227507	ENSP00000261424
ENSP00000227507	ENSP00000261712
ENSP00000227507	ENSP00000262133
ENSP00000227507	ENSP00000262367
ENSP00000227507	ENSP00000262904
ENSP00000227507	ENSP00000264169
ENSP00000227507	ENSP00000264335
ENSP00000227507	ENSP00000264637
ENSP00000227507	ENSP00000264639
ENSP00000227507	ENSP00000265056
ENSP00000227507	ENSP00000265351
ENSP00000227507	ENSP00000267163
ENSP00000227507	ENSP00000269701
ENSP00000227507	ENSP00000271883
ENSP00000227507	ENSP00000276072
ENSP00000227507	ENSP00000276893
ENSP00000227507	ENSP00000278616
ENSP00000227507	ENSP00000281623
ENSP00000227507	ENSP00000287598
ENSP00000227507	ENSP00000295901
ENSP00000227507	ENSP00000296509
ENSP00000227507	ENSP00000298139
ENSP00000227507	ENSP00000298552
ENSP00000227507	ENSP00000300213
ENSP00000227507	ENSP00000302530
ENSP00000227507	ENSP00000304226
ENSP00000227507	ENSP00000306999
ENSP00000227507	ENSP00000307272
ENSP00000227507	ENSP00000309474
ENSP00000227507	ENSP00000309871
ENSP00000227507	ENSP00000310841
ENSP00000227507	ENSP00000311083
ENSP00000227507	ENSP00000313420
ENSP00000227507	ENSP00000314949
ENSP00000227507	ENSP00000319169
ENSP00000227507	ENSP00000321636
ENSP00000227507	ENSP00000323246

ENSP00000227507	ENSP00000323377
ENSP00000227507	ENSP00000325120
ENSP00000227507	ENSP00000329357
ENSP00000227507	ENSP00000332171
ENSP00000227507	ENSP00000332353
ENSP00000227507	ENSP00000343741
ENSP00000227507	ENSP00000344456
ENSP00000227507	ENSP00000344460
ENSP00000227507	ENSP00000346566
ENSP00000227507	ENSP00000347169
ENSP00000227507	ENSP00000347602
ENSP00000227507	ENSP00000347733
ENSP00000227507	ENSP00000348550
ENSP00000227507	ENSP00000350283
ENSP00000227507	ENSP00000350491
ENSP00000227507	ENSP00000351284
ENSP00000227507	ENSP00000352121
ENSP00000227507	ENSP00000353622
ENSP00000227507	ENSP00000354558
ENSP00000227507	ENSP00000355013
ENSP00000227507	ENSP00000355153
ENSP00000227507	ENSP00000357907
ENSP00000227507	ENSP00000358404
ENSP00000227507	ENSP00000360660
ENSP00000227507	ENSP00000361465
ENSP00000227507	ENSP00000362532
ENSP00000227507	ENSP00000363827
ENSP00000227507	ENSP00000365775
ENSP00000227507	ENSP00000368438
ENSP00000227507	ENSP00000368686
ENSP00000227507	ENSP00000369757
ENSP00000227507	ENSP00000377793
ENSP00000227507	ENSP00000381932
ENSP00000227507	ENSP00000384109
ENSP00000227507	ENSP00000384675
ENSP00000227507	ENSP00000390098
ENSP00000227507	ENSP00000413720
ENSP00000227507	ENSP00000414259
ENSP00000227520	ENSP00000329466
ENSP00000227524	ENSP00000230050
ENSP00000227524	ENSP00000236273
ENSP00000227524	ENSP00000240304
ENSP00000227524	ENSP00000240922
ENSP00000227524	ENSP00000243563
ENSP00000227524	ENSP00000244520
ENSP00000227524	ENSP00000245539

ENSP00000227524	ENSP00000248553
ENSP00000227524	ENSP00000252137
ENSP00000227524	ENSP00000252622
ENSP00000227524	ENSP00000253108
ENSP00000227524	ENSP00000253363
ENSP00000227524	ENSP00000254193
ENSP00000227524	ENSP00000260210
ENSP00000227524	ENSP00000261531
ENSP00000227524	ENSP00000261973
ENSP00000227524	ENSP00000262962
ENSP00000227524	ENSP00000264951
ENSP00000227524	ENSP00000265107
ENSP00000227524	ENSP00000266079
ENSP00000227524	ENSP00000266735
ENSP00000227524	ENSP00000267812
ENSP00000227524	ENSP00000268482
ENSP00000227524	ENSP00000271526
ENSP00000227524	ENSP00000271628
ENSP00000227524	ENSP00000272091
ENSP00000227524	ENSP00000272139
ENSP00000227524	ENSP00000274712
ENSP00000227524	ENSP00000276570
ENSP00000227524	ENSP00000278572
ENSP00000227524	ENSP00000282251
ENSP00000227524	ENSP00000283943
ENSP00000227524	ENSP00000290541
ENSP00000227524	ENSP00000296215
ENSP00000227524	ENSP00000296702
ENSP00000227524	ENSP00000297151
ENSP00000227524	ENSP00000298229
ENSP00000227524	ENSP00000300146
ENSP00000227524	ENSP00000300413
ENSP00000227524	ENSP00000301740
ENSP00000227524	ENSP00000301764
ENSP00000227524	ENSP00000302160
ENSP00000227524	ENSP00000302896
ENSP00000227524	ENSP00000303191
ENSP00000227524	ENSP00000304350
ENSP00000227524	ENSP00000304370
ENSP00000227524	ENSP00000305790
ENSP00000227524	ENSP00000307863
ENSP00000227524	ENSP00000308369
ENSP00000227524	ENSP00000310448
ENSP00000227524	ENSP00000310596
ENSP00000227524	ENSP00000310721
ENSP00000227524	ENSP00000311028

ENSP00000227524	ENSP00000312981
ENSP00000227524	ENSP00000313674
ENSP00000227524	ENSP00000315379
ENSP00000227524	ENSP00000318861
ENSP00000227524	ENSP00000319341
ENSP00000227524	ENSP00000323302
ENSP00000227524	ENSP00000324122
ENSP00000227524	ENSP00000325448
ENSP00000227524	ENSP00000326261
ENSP00000227524	ENSP00000326411
ENSP00000227524	ENSP00000326514
ENSP00000227524	ENSP00000326806
ENSP00000227524	ENSP00000331907
ENSP00000227524	ENSP00000332103
ENSP00000227524	ENSP00000334100
ENSP00000227524	ENSP00000335321
ENSP00000227524	ENSP00000339090
ENSP00000227524	ENSP00000339095
ENSP00000227524	ENSP00000339145
ENSP00000227524	ENSP00000342374
ENSP00000227524	ENSP00000343557
ENSP00000227524	ENSP00000343701
ENSP00000227524	ENSP00000344241
ENSP00000227524	ENSP00000344741
ENSP00000227524	ENSP00000345917
ENSP00000227524	ENSP00000346236
ENSP00000227524	ENSP00000346986
ENSP00000227524	ENSP00000348010
ENSP00000227524	ENSP00000351137
ENSP00000227524	ENSP00000351284
ENSP00000227524	ENSP00000351947
ENSP00000227524	ENSP00000352308
ENSP00000227524	ENSP00000354340
ENSP00000227524	ENSP00000355050
ENSP00000227524	ENSP00000356541
ENSP00000227524	ENSP00000357631
ENSP00000227524	ENSP00000358554
ENSP00000227524	ENSP00000360216
ENSP00000227524	ENSP00000360497
ENSP00000227524	ENSP00000360532
ENSP00000227524	ENSP00000360777
ENSP00000227524	ENSP00000361557
ENSP00000227524	ENSP00000361577
ENSP00000227524	ENSP00000361616
ENSP00000227524	ENSP00000362110
ENSP00000227524	ENSP00000362115

ENSP00000227524	ENSP00000362900
ENSP00000227524	ENSP00000362936
ENSP00000227524	ENSP00000363308
ENSP00000227524	ENSP00000363313
ENSP00000227524	ENSP00000363734
ENSP00000227524	ENSP00000364270
ENSP00000227524	ENSP00000364289
ENSP00000227524	ENSP00000364813
ENSP00000227524	ENSP00000366557
ENSP00000227524	ENSP00000366607
ENSP00000227524	ENSP00000366829
ENSP00000227524	ENSP00000367439
ENSP00000227524	ENSP00000370460
ENSP00000227524	ENSP00000373354
ENSP00000227524	ENSP00000376973
ENSP00000227524	ENSP00000377262
ENSP00000227524	ENSP00000379607
ENSP00000227524	ENSP00000384534
ENSP00000227524	ENSP00000385903
ENSP00000227524	ENSP00000387006
ENSP00000227524	ENSP00000391432
ENSP00000227524	ENSP00000396937
ENSP00000227524	ENSP00000397435
ENSP00000227524	ENSP00000400591
ENSP00000227524	ENSP00000404151
ENSP00000227524	ENSP00000412566
ENSP00000227524	ENSP00000414006
ENSP00000227524	ENSP00000414634
ENSP00000227618	ENSP00000318775
ENSP00000227638	ENSP00000419952
ENSP00000227667	ENSP00000295897
ENSP00000227667	ENSP00000314615
ENSP00000227752	ENSP00000315768
ENSP00000227752	ENSP00000341208
ENSP00000227752	ENSP00000359206
ENSP00000227752	ENSP00000412237
ENSP00000227758	ENSP00000228318
ENSP00000227758	ENSP00000245912
ENSP00000227758	ENSP00000250113
ENSP00000227758	ENSP00000251968
ENSP00000227758	ENSP00000253003
ENSP00000227758	ENSP00000253462
ENSP00000227758	ENSP00000256442
ENSP00000227758	ENSP00000259075
ENSP00000227758	ENSP00000259808
ENSP00000227758	ENSP00000264193

ENSP00000227758	ENSP00000264552
ENSP00000227758	ENSP00000266087
ENSP00000227758	ENSP00000267169
ENSP00000227758	ENSP00000294119
ENSP00000227758	ENSP00000296328
ENSP00000227758	ENSP00000298743
ENSP00000227758	ENSP00000299601
ENSP00000227758	ENSP00000299767
ENSP00000227758	ENSP00000300589
ENSP00000227758	ENSP00000306906
ENSP00000227758	ENSP00000309595
ENSP00000227758	ENSP00000314132
ENSP00000227758	ENSP00000314733
ENSP00000227758	ENSP00000319673
ENSP00000227758	ENSP00000326366
ENSP00000227758	ENSP00000326579
ENSP00000227758	ENSP00000326737
ENSP00000227758	ENSP00000332454
ENSP00000227758	ENSP00000335657
ENSP00000227758	ENSP00000340935
ENSP00000227758	ENSP00000341268
ENSP00000227758	ENSP00000345341
ENSP00000227758	ENSP00000353059
ENSP00000227758	ENSP00000354822
ENSP00000227758	ENSP00000355028
ENSP00000227758	ENSP00000358335
ENSP00000227758	ENSP00000358997
ENSP00000227758	ENSP00000363089
ENSP00000227758	ENSP00000363095
ENSP00000227758	ENSP00000363680
ENSP00000227758	ENSP00000368646
ENSP00000227758	ENSP00000380921
ENSP00000227758	ENSP00000381863
ENSP00000227758	ENSP00000381891
ENSP00000227758	ENSP00000382423
ENSP00000227758	ENSP00000401980
ENSP00000227880	ENSP00000344648
ENSP00000227880	ENSP00000361990
ENSP00000227880	ENSP00000396068
ENSP00000228027	ENSP00000244051
ENSP00000228027	ENSP00000261596
ENSP00000228027	ENSP00000264005
ENSP00000228027	ENSP00000265052
ENSP00000228027	ENSP00000290949
ENSP00000228027	ENSP00000292566
ENSP00000228027	ENSP00000325518

ENSP00000228027	ENSP00000332258
ENSP00000228027	ENSP00000356591
ENSP00000228027	ENSP00000358777
ENSP00000228027	ENSP00000359380
ENSP00000228027	ENSP00000361667
ENSP00000228136	ENSP00000261368
ENSP00000228136	ENSP00000294008
ENSP00000228136	ENSP00000307491
ENSP00000228136	ENSP00000335321
ENSP00000228251	ENSP00000240055
ENSP00000228251	ENSP00000261531
ENSP00000228251	ENSP00000269385
ENSP00000228251	ENSP00000281537
ENSP00000228251	ENSP00000296930
ENSP00000228251	ENSP00000308546
ENSP00000228251	ENSP00000326261
ENSP00000228251	ENSP00000339145
ENSP00000228251	ENSP00000355675
ENSP00000228251	ENSP00000360876
ENSP00000228251	ENSP00000369871
ENSP00000228251	ENSP00000379607
ENSP00000228251	ENSP00000381657
ENSP00000228280	ENSP00000250340
ENSP00000228280	ENSP00000296871
ENSP00000228280	ENSP00000340305
ENSP00000228284	ENSP00000240316
ENSP00000228284	ENSP00000248114
ENSP00000228284	ENSP00000252622
ENSP00000228284	ENSP00000256857
ENSP00000228284	ENSP00000261531
ENSP00000228284	ENSP00000263413
ENSP00000228284	ENSP00000263736
ENSP00000228284	ENSP00000264951
ENSP00000228284	ENSP00000266079
ENSP00000228284	ENSP00000267163
ENSP00000228284	ENSP00000268483
ENSP00000228284	ENSP00000274473
ENSP00000228284	ENSP00000274712
ENSP00000228284	ENSP00000284719
ENSP00000228284	ENSP00000300146
ENSP00000228284	ENSP00000300413
ENSP00000228284	ENSP00000301740
ENSP00000228284	ENSP00000302160
ENSP00000228284	ENSP00000304370
ENSP00000228284	ENSP00000308546
ENSP00000228284	ENSP00000310596

ENSP00000228284	ENSP00000310723
ENSP00000228284	ENSP00000312981
ENSP00000228284	ENSP00000313674
ENSP00000228284	ENSP00000315379
ENSP00000228284	ENSP00000315859
ENSP00000228284	ENSP00000319341
ENSP00000228284	ENSP00000320251
ENSP00000228284	ENSP00000324122
ENSP00000228284	ENSP00000329466
ENSP00000228284	ENSP00000334148
ENSP00000228284	ENSP00000344220
ENSP00000228284	ENSP00000348565
ENSP00000228284	ENSP00000351490
ENSP00000228284	ENSP00000355899
ENSP00000228284	ENSP00000363313
ENSP00000228284	ENSP00000367265
ENSP00000228284	ENSP00000379457
ENSP00000228284	ENSP00000402338
ENSP00000228284	ENSP00000410396
ENSP00000228284	ENSP00000410758
ENSP00000228284	ENSP00000412566
ENSP00000228284	ENSP00000414006
ENSP00000228284	ENSP00000414634
ENSP00000228284	ENSP00000417229
ENSP00000228307	ENSP00000230449
ENSP00000228307	ENSP00000232458
ENSP00000228307	ENSP00000247226
ENSP00000228307	ENSP00000253861
ENSP00000228307	ENSP00000254051
ENSP00000228307	ENSP00000257430
ENSP00000228307	ENSP00000262304
ENSP00000228307	ENSP00000262995
ENSP00000228307	ENSP00000264093
ENSP00000228307	ENSP00000268182
ENSP00000228307	ENSP00000269122
ENSP00000228307	ENSP00000274376
ENSP00000228307	ENSP00000276416
ENSP00000228307	ENSP00000281171
ENSP00000228307	ENSP00000281172
ENSP00000228307	ENSP00000288602
ENSP00000228307	ENSP00000300574
ENSP00000228307	ENSP00000302478
ENSP00000228307	ENSP00000303507
ENSP00000228307	ENSP00000306124
ENSP00000228307	ENSP00000312506
ENSP00000228307	ENSP00000329140

ENSP00000228307	ENSP00000329623
ENSP00000228307	ENSP00000336740
ENSP00000228307	ENSP00000339933
ENSP00000228307	ENSP00000340944
ENSP00000228307	ENSP00000341189
ENSP00000228307	ENSP00000344220
ENSP00000228307	ENSP00000344666
ENSP00000228307	ENSP00000346839
ENSP00000228307	ENSP00000347134
ENSP00000228307	ENSP00000348602
ENSP00000228307	ENSP00000349824
ENSP00000228307	ENSP00000349959
ENSP00000228307	ENSP00000350297
ENSP00000228307	ENSP00000351015
ENSP00000228307	ENSP00000351318
ENSP00000228307	ENSP00000354718
ENSP00000228307	ENSP00000355136
ENSP00000228307	ENSP00000355296
ENSP00000228307	ENSP00000356346
ENSP00000228307	ENSP00000363763
ENSP00000228307	ENSP00000364893
ENSP00000228307	ENSP00000364898
ENSP00000228307	ENSP00000370719
ENSP00000228307	ENSP00000371236
ENSP00000228307	ENSP00000371897
ENSP00000228307	ENSP00000377047
ENSP00000228307	ENSP00000378338
ENSP00000228307	ENSP00000380942
ENSP00000228307	ENSP00000383623
ENSP00000228307	ENSP00000402240
ENSP00000228318	ENSP00000229379
ENSP00000228318	ENSP00000231706
ENSP00000228318	ENSP00000237530
ENSP00000228318	ENSP00000245796
ENSP00000228318	ENSP00000251864
ENSP00000228318	ENSP00000252487
ENSP00000228318	ENSP00000256216
ENSP00000228318	ENSP00000258886
ENSP00000228318	ENSP00000259569
ENSP00000228318	ENSP00000260102
ENSP00000228318	ENSP00000260443
ENSP00000228318	ENSP00000262215
ENSP00000228318	ENSP00000262982
ENSP00000228318	ENSP00000264705
ENSP00000228318	ENSP00000265351
ENSP00000228318	ENSP00000268379

ENSP00000228318	ENSP00000268668
ENSP00000228318	ENSP00000277865
ENSP00000228318	ENSP00000282541
ENSP00000228318	ENSP00000290429
ENSP00000228318	ENSP00000294724
ENSP00000228318	ENSP00000301585
ENSP00000228318	ENSP00000302790
ENSP00000228318	ENSP00000302896
ENSP00000228318	ENSP00000304592
ENSP00000228318	ENSP00000305653
ENSP00000228318	ENSP00000308944
ENSP00000228318	ENSP00000309595
ENSP00000228318	ENSP00000311505
ENSP00000228318	ENSP00000311766
ENSP00000228318	ENSP00000314036
ENSP00000228318	ENSP00000314380
ENSP00000228318	ENSP00000317159
ENSP00000228318	ENSP00000324127
ENSP00000228318	ENSP00000336712
ENSP00000228318	ENSP00000341838
ENSP00000228318	ENSP00000342026
ENSP00000228318	ENSP00000346453
ENSP00000228318	ENSP00000349142
ENSP00000228318	ENSP00000350012
ENSP00000228318	ENSP00000351885
ENSP00000228318	ENSP00000355518
ENSP00000228318	ENSP00000355890
ENSP00000228318	ENSP00000357880
ENSP00000228318	ENSP00000357998
ENSP00000228318	ENSP00000358478
ENSP00000228318	ENSP00000359000
ENSP00000228318	ENSP00000359526
ENSP00000228318	ENSP00000360327
ENSP00000228318	ENSP00000361207
ENSP00000228318	ENSP00000362306
ENSP00000228318	ENSP00000364649
ENSP00000228318	ENSP00000366482
ENSP00000228318	ENSP00000368646
ENSP00000228318	ENSP00000369042
ENSP00000228318	ENSP00000377280
ENSP00000228318	ENSP00000377527
ENSP00000228318	ENSP00000377640
ENSP00000228318	ENSP00000379712
ENSP00000228318	ENSP00000381237
ENSP00000228318	ENSP00000383719
ENSP00000228318	ENSP00000386239

ENSP00000228318	ENSP00000387262
ENSP00000228318	ENSP00000394382
ENSP00000228318	ENSP00000408236
ENSP00000228425	ENSP00000380921
ENSP00000228425	ENSP00000388373
ENSP00000228437	ENSP00000258106
ENSP00000228437	ENSP00000350633
ENSP00000228476	ENSP00000254101
ENSP00000228476	ENSP00000365103
ENSP00000228476	ENSP00000374280
ENSP00000228510	ENSP00000251038
ENSP00000228510	ENSP00000276603
ENSP00000228510	ENSP00000282841
ENSP00000228510	ENSP00000318604
ENSP00000228510	ENSP00000339933
ENSP00000228510	ENSP00000350249
ENSP00000228510	ENSP00000356591
ENSP00000228510	ENSP00000381031
ENSP00000228534	ENSP00000231228
ENSP00000228534	ENSP00000314425
ENSP00000228534	ENSP00000321345
ENSP00000228705	ENSP00000393596
ENSP00000228799	ENSP00000337850
ENSP00000228799	ENSP00000371341
ENSP00000228811	ENSP00000302021
ENSP00000228825	ENSP00000251038
ENSP00000228825	ENSP00000252725
ENSP00000228825	ENSP00000253083
ENSP00000228825	ENSP00000259477
ENSP00000228825	ENSP00000260956
ENSP00000228825	ENSP00000261017
ENSP00000228825	ENSP00000261534
ENSP00000228825	ENSP00000273398
ENSP00000228825	ENSP00000286091
ENSP00000228825	ENSP00000287598
ENSP00000228825	ENSP00000295685
ENSP00000228825	ENSP00000298852
ENSP00000228825	ENSP00000299626
ENSP00000228825	ENSP00000301843
ENSP00000228825	ENSP00000302530
ENSP00000228825	ENSP00000310129
ENSP00000228825	ENSP00000317334
ENSP00000228825	ENSP00000320924
ENSP00000228825	ENSP00000326238
ENSP00000228825	ENSP00000338727
ENSP00000228825	ENSP00000352802

ENSP00000228825	ENSP00000354125
ENSP00000228825	ENSP00000358994
ENSP00000228825	ENSP00000359067
ENSP00000228825	ENSP00000360149
ENSP00000228825	ENSP00000362744
ENSP00000228825	ENSP00000365147
ENSP00000228825	ENSP00000365891
ENSP00000228825	ENSP00000367841
ENSP00000228825	ENSP00000370517
ENSP00000228825	ENSP00000375857
ENSP00000228825	ENSP00000380431
ENSP00000228825	ENSP00000382177
ENSP00000228825	ENSP00000386878
ENSP00000228825	ENSP00000391723
ENSP00000228825	ENSP00000417229
ENSP00000228843	ENSP00000261584
ENSP00000228843	ENSP00000307491
ENSP00000228862	ENSP00000306245
ENSP00000228862	ENSP00000364320
ENSP00000228862	ENSP00000365439
ENSP00000228862	ENSP00000367029
ENSP00000228865	ENSP00000265372
ENSP00000228865	ENSP00000295899
ENSP00000228865	ENSP00000304185
ENSP00000228865	ENSP00000362094
ENSP00000228872	ENSP00000231487
ENSP00000228872	ENSP00000234160
ENSP00000228872	ENSP00000244741
ENSP00000228872	ENSP00000247970
ENSP00000228872	ENSP00000255465
ENSP00000228872	ENSP00000256442
ENSP00000228872	ENSP00000257430
ENSP00000228872	ENSP00000262643
ENSP00000228872	ENSP00000264335
ENSP00000228872	ENSP00000267163
ENSP00000228872	ENSP00000274255
ENSP00000228872	ENSP00000298552
ENSP00000228872	ENSP00000301764
ENSP00000228872	ENSP00000311083
ENSP00000228872	ENSP00000321239
ENSP00000228872	ENSP00000328287
ENSP00000228872	ENSP00000330074
ENSP00000228872	ENSP00000335628
ENSP00000228872	ENSP00000338413
ENSP00000228872	ENSP00000343701
ENSP00000228872	ENSP00000345895

ENSP00000228872	ENSP00000350512
ENSP00000228872	ENSP00000360821
ENSP00000228872	ENSP00000381045
ENSP00000228916	ENSP00000251968
ENSP00000228916	ENSP00000260187
ENSP00000228916	ENSP00000331201
ENSP00000228916	ENSP00000345530
ENSP00000228916	ENSP00000359688
ENSP00000228916	ENSP00000379228
ENSP00000228928	ENSP00000310042
ENSP00000228928	ENSP00000345917
ENSP00000228928	ENSP00000354040
ENSP00000228928	ENSP00000354522
ENSP00000228928	ENSP00000361949
ENSP00000228928	ENSP00000363559
ENSP00000228928	ENSP00000392601
ENSP00000228928	ENSP00000401980
ENSP00000228955	ENSP00000247219
ENSP00000228955	ENSP00000247843
ENSP00000228955	ENSP00000251038
ENSP00000228955	ENSP00000256897
ENSP00000228955	ENSP00000259895
ENSP00000228955	ENSP00000261245
ENSP00000228955	ENSP00000263857
ENSP00000228955	ENSP00000264157
ENSP00000228955	ENSP00000265963
ENSP00000228955	ENSP00000278412
ENSP00000228955	ENSP00000283875
ENSP00000228955	ENSP00000285398
ENSP00000228955	ENSP00000295926
ENSP00000228955	ENSP00000296328
ENSP00000228955	ENSP00000306983
ENSP00000228955	ENSP00000314949
ENSP00000228955	ENSP00000325448
ENSP00000228955	ENSP00000329029
ENSP00000228955	ENSP00000340823
ENSP00000228955	ENSP00000348168
ENSP00000228955	ENSP00000359531
ENSP00000228955	ENSP00000367406
ENSP00000228955	ENSP00000369695
ENSP00000228955	ENSP00000374529
ENSP00000228955	ENSP00000375809
ENSP00000228955	ENSP00000377969
ENSP00000229003	ENSP00000304903
ENSP00000229135	ENSP00000290158
ENSP00000229135	ENSP00000290219

ENSP00000229135	ENSP00000343701
ENSP00000229135	ENSP00000356713
ENSP00000229179	ENSP00000229595
ENSP00000229179	ENSP00000231498
ENSP00000229179	ENSP00000233146
ENSP00000229179	ENSP00000234420
ENSP00000229179	ENSP00000234827
ENSP00000229179	ENSP00000240159
ENSP00000229179	ENSP00000245544
ENSP00000229179	ENSP00000247815
ENSP00000229179	ENSP00000247970
ENSP00000229179	ENSP00000248566
ENSP00000229179	ENSP00000251074
ENSP00000229179	ENSP00000260327
ENSP00000229179	ENSP00000261396
ENSP00000229179	ENSP00000261531
ENSP00000229179	ENSP00000261596
ENSP00000229179	ENSP00000261881
ENSP00000229179	ENSP00000262077
ENSP00000229179	ENSP00000262315
ENSP00000229179	ENSP00000262765
ENSP00000229179	ENSP00000263390
ENSP00000229179	ENSP00000264883
ENSP00000229179	ENSP00000265081
ENSP00000229179	ENSP00000265304
ENSP00000229179	ENSP00000265351
ENSP00000229179	ENSP00000268043
ENSP00000229179	ENSP00000283195
ENSP00000229179	ENSP00000285243
ENSP00000229179	ENSP00000285968
ENSP00000229179	ENSP00000288774
ENSP00000229179	ENSP00000290158
ENSP00000229179	ENSP00000291688
ENSP00000229179	ENSP00000296411
ENSP00000229179	ENSP00000298139
ENSP00000229179	ENSP00000302790
ENSP00000229179	ENSP00000302886
ENSP00000229179	ENSP00000305503
ENSP00000229179	ENSP00000308622
ENSP00000229179	ENSP00000310668
ENSP00000229179	ENSP00000311873
ENSP00000229179	ENSP00000313171
ENSP00000229179	ENSP00000315700
ENSP00000229179	ENSP00000316032
ENSP00000229179	ENSP00000318977
ENSP00000229179	ENSP00000322180

ENSP00000229179	ENSP00000322218
ENSP00000229179	ENSP00000322242
ENSP00000229179	ENSP00000322396
ENSP00000229179	ENSP00000325312
ENSP00000229179	ENSP00000325863
ENSP00000229179	ENSP00000329466
ENSP00000229179	ENSP00000331310
ENSP00000229179	ENSP00000342262
ENSP00000229179	ENSP00000343701
ENSP00000229179	ENSP00000344460
ENSP00000229179	ENSP00000347232
ENSP00000229179	ENSP00000347329
ENSP00000229179	ENSP00000351284
ENSP00000229179	ENSP00000352021
ENSP00000229179	ENSP00000352400
ENSP00000229179	ENSP00000354522
ENSP00000229179	ENSP00000355185
ENSP00000229179	ENSP00000355560
ENSP00000229179	ENSP00000355922
ENSP00000229179	ENSP00000356448
ENSP00000229179	ENSP00000360268
ENSP00000229179	ENSP00000360286
ENSP00000229179	ENSP00000360532
ENSP00000229179	ENSP00000365811
ENSP00000229179	ENSP00000367721
ENSP00000229179	ENSP00000368959
ENSP00000229179	ENSP00000370150
ENSP00000229179	ENSP00000370151
ENSP00000229179	ENSP00000373715
ENSP00000229179	ENSP00000377793
ENSP00000229179	ENSP00000382779
ENSP00000229179	ENSP00000406293
ENSP00000229179	ENSP00000414321
ENSP00000229195	ENSP00000233078
ENSP00000229195	ENSP00000240185
ENSP00000229195	ENSP00000247219
ENSP00000229195	ENSP00000251038
ENSP00000229195	ENSP00000252115
ENSP00000229195	ENSP00000253363
ENSP00000229195	ENSP00000256897
ENSP00000229195	ENSP00000261741
ENSP00000229195	ENSP00000261951
ENSP00000229195	ENSP00000262077
ENSP00000229195	ENSP00000263576
ENSP00000229195	ENSP00000263857
ENSP00000229195	ENSP00000264157

ENSP00000229195	ENSP00000268957
ENSP00000229195	ENSP00000273064
ENSP00000229195	ENSP00000276072
ENSP00000229195	ENSP00000285896
ENSP00000229195	ENSP00000295926
ENSP00000229195	ENSP00000296328
ENSP00000229195	ENSP00000296509
ENSP00000229195	ENSP00000300146
ENSP00000229195	ENSP00000301764
ENSP00000229195	ENSP00000311343
ENSP00000229195	ENSP00000314949
ENSP00000229195	ENSP00000320949
ENSP00000229195	ENSP00000325376
ENSP00000229195	ENSP00000330060
ENSP00000229195	ENSP00000331817
ENSP00000229195	ENSP00000331879
ENSP00000229195	ENSP00000332444
ENSP00000229195	ENSP00000346120
ENSP00000229195	ENSP00000348551
ENSP00000229195	ENSP00000354673
ENSP00000229195	ENSP00000354995
ENSP00000229195	ENSP00000355051
ENSP00000229195	ENSP00000356448
ENSP00000229195	ENSP00000357907
ENSP00000229195	ENSP00000361005
ENSP00000229195	ENSP00000361287
ENSP00000229195	ENSP00000361949
ENSP00000229195	ENSP00000362532
ENSP00000229195	ENSP00000362900
ENSP00000229195	ENSP00000366135
ENSP00000229195	ENSP00000368984
ENSP00000229195	ENSP00000369965
ENSP00000229195	ENSP00000374529
ENSP00000229201	ENSP00000234296
ENSP00000229201	ENSP00000234626
ENSP00000229201	ENSP00000247815
ENSP00000229201	ENSP00000248114
ENSP00000229201	ENSP00000249636
ENSP00000229201	ENSP00000251038
ENSP00000229201	ENSP00000253462
ENSP00000229201	ENSP00000254719
ENSP00000229201	ENSP00000258052
ENSP00000229201	ENSP00000260731
ENSP00000229201	ENSP00000261396
ENSP00000229201	ENSP00000261424
ENSP00000229201	ENSP00000261881

ENSP00000229201	ENSP00000262315
ENSP00000229201	ENSP00000262367
ENSP00000229201	ENSP00000262518
ENSP00000229201	ENSP00000263201
ENSP00000229201	ENSP00000264552
ENSP00000229201	ENSP00000264926
ENSP00000229201	ENSP00000265056
ENSP00000229201	ENSP00000265728
ENSP00000229201	ENSP00000267430
ENSP00000229201	ENSP00000268043
ENSP00000229201	ENSP00000276533
ENSP00000229201	ENSP00000282326
ENSP00000229201	ENSP00000287239
ENSP00000229201	ENSP00000287598
ENSP00000229201	ENSP00000290231
ENSP00000229201	ENSP00000296273
ENSP00000229201	ENSP00000297338
ENSP00000229201	ENSP00000298139
ENSP00000229201	ENSP00000302530
ENSP00000229201	ENSP00000302955
ENSP00000229201	ENSP00000307853
ENSP00000229201	ENSP00000308699
ENSP00000229201	ENSP00000310520
ENSP00000229201	ENSP00000312870
ENSP00000229201	ENSP00000312995
ENSP00000229201	ENSP00000313171
ENSP00000229201	ENSP00000314420
ENSP00000229201	ENSP00000316032
ENSP00000229201	ENSP00000317039
ENSP00000229201	ENSP00000317636
ENSP00000229201	ENSP00000318977
ENSP00000229201	ENSP00000321636
ENSP00000229201	ENSP00000322180
ENSP00000229201	ENSP00000322570
ENSP00000229201	ENSP00000323099
ENSP00000229201	ENSP00000323663
ENSP00000229201	ENSP00000325863
ENSP00000229201	ENSP00000334474
ENSP00000229201	ENSP00000336606
ENSP00000229201	ENSP00000336868
ENSP00000229201	ENSP00000340879
ENSP00000229201	ENSP00000341382
ENSP00000229201	ENSP00000343741
ENSP00000229201	ENSP00000347232
ENSP00000229201	ENSP00000351284
ENSP00000229201	ENSP00000351697

ENSP00000229201	ENSP00000353793
ENSP00000229201	ENSP00000354522
ENSP00000229201	ENSP00000354720
ENSP00000229201	ENSP00000354957
ENSP00000229201	ENSP00000355388
ENSP00000229201	ENSP00000355922
ENSP00000229201	ENSP00000360116
ENSP00000229201	ENSP00000360621
ENSP00000229201	ENSP00000365588
ENSP00000229201	ENSP00000366974
ENSP00000229201	ENSP00000368349
ENSP00000229201	ENSP00000368438
ENSP00000229201	ENSP00000369335
ENSP00000229201	ENSP00000369411
ENSP00000229201	ENSP00000370151
ENSP00000229201	ENSP00000371155
ENSP00000229201	ENSP00000371475
ENSP00000229201	ENSP00000372023
ENSP00000229201	ENSP00000373060
ENSP00000229201	ENSP00000374213
ENSP00000229201	ENSP00000380504
ENSP00000229201	ENSP00000384048
ENSP00000229201	ENSP00000387429
ENSP00000229201	ENSP00000396484
ENSP00000229201	ENSP00000404318
ENSP00000229201	ENSP00000409167
ENSP00000229201	ENSP00000413632
ENSP00000229201	ENSP00000415212
ENSP00000229201	ENSP00000416511
ENSP00000229214	ENSP00000230050
ENSP00000229214	ENSP00000230340
ENSP00000229214	ENSP00000232888
ENSP00000229214	ENSP00000237289
ENSP00000229214	ENSP00000238256
ENSP00000229214	ENSP00000241600
ENSP00000229214	ENSP00000244230
ENSP00000229214	ENSP00000244496
ENSP00000229214	ENSP00000245539
ENSP00000229214	ENSP00000245960
ENSP00000229214	ENSP00000253099
ENSP00000229214	ENSP00000253237
ENSP00000229214	ENSP00000254037
ENSP00000229214	ENSP00000256383
ENSP00000229214	ENSP00000256442
ENSP00000229214	ENSP00000257829
ENSP00000229214	ENSP00000258499

ENSP00000229214	ENSP00000261637
ENSP00000229214	ENSP00000261708
ENSP00000229214	ENSP00000261868
ENSP00000229214	ENSP00000262056
ENSP00000229214	ENSP00000262300
ENSP00000229214	ENSP00000262854
ENSP00000229214	ENSP00000262903
ENSP00000229214	ENSP00000263629
ENSP00000229214	ENSP00000268616
ENSP00000229214	ENSP00000268802
ENSP00000229214	ENSP00000269385
ENSP00000229214	ENSP00000269499
ENSP00000229214	ENSP00000270583
ENSP00000229214	ENSP00000270625
ENSP00000229214	ENSP00000272521
ENSP00000229214	ENSP00000274606
ENSP00000229214	ENSP00000274849
ENSP00000229214	ENSP00000276201
ENSP00000229214	ENSP00000278572
ENSP00000229214	ENSP00000283109
ENSP00000229214	ENSP00000286719
ENSP00000229214	ENSP00000291576
ENSP00000229214	ENSP00000296792
ENSP00000229214	ENSP00000296802
ENSP00000229214	ENSP00000297579
ENSP00000229214	ENSP00000297990
ENSP00000229214	ENSP00000298746
ENSP00000229214	ENSP00000301364
ENSP00000229214	ENSP00000302896
ENSP00000229214	ENSP00000305702
ENSP00000229214	ENSP00000308179
ENSP00000229214	ENSP00000311028
ENSP00000229214	ENSP00000311135
ENSP00000229214	ENSP00000311430
ENSP00000229214	ENSP00000314193
ENSP00000229214	ENSP00000314311
ENSP00000229214	ENSP00000314348
ENSP00000229214	ENSP00000315674
ENSP00000229214	ENSP00000317334
ENSP00000229214	ENSP00000318604
ENSP00000229214	ENSP00000318646
ENSP00000229214	ENSP00000320566
ENSP00000229214	ENSP00000320917
ENSP00000229214	ENSP00000324122
ENSP00000229214	ENSP00000325448
ENSP00000229214	ENSP00000326806

ENSP00000229214	ENSP00000326981
ENSP00000229214	ENSP00000327179
ENSP00000229214	ENSP00000328854
ENSP00000229214	ENSP00000329466
ENSP00000229214	ENSP00000337839
ENSP00000229214	ENSP00000339095
ENSP00000229214	ENSP00000339795
ENSP00000229214	ENSP00000340019
ENSP00000229214	ENSP00000346015
ENSP00000229214	ENSP00000346050
ENSP00000229214	ENSP00000346067
ENSP00000229214	ENSP00000346120
ENSP00000229214	ENSP00000348714
ENSP00000229214	ENSP00000350009
ENSP00000229214	ENSP00000355471
ENSP00000229214	ENSP00000355541
ENSP00000229214	ENSP00000356134
ENSP00000229214	ENSP00000356520
ENSP00000229214	ENSP00000358563
ENSP00000229214	ENSP00000358812
ENSP00000229214	ENSP00000360031
ENSP00000229214	ENSP00000360215
ENSP00000229214	ENSP00000361076
ENSP00000229214	ENSP00000361245
ENSP00000229214	ENSP00000361982
ENSP00000229214	ENSP00000362744
ENSP00000229214	ENSP00000363308
ENSP00000229214	ENSP00000363642
ENSP00000229214	ENSP00000363676
ENSP00000229214	ENSP00000363746
ENSP00000229214	ENSP00000364293
ENSP00000229214	ENSP00000364320
ENSP00000229214	ENSP00000365837
ENSP00000229214	ENSP00000366135
ENSP00000229214	ENSP00000366557
ENSP00000229214	ENSP00000366974
ENSP00000229214	ENSP00000368984
ENSP00000229214	ENSP00000370589
ENSP00000229214	ENSP00000371101
ENSP00000229214	ENSP00000371155
ENSP00000229214	ENSP00000372023
ENSP00000229214	ENSP00000372695
ENSP00000229214	ENSP00000374390
ENSP00000229214	ENSP00000376309
ENSP00000229214	ENSP00000376946
ENSP00000229214	ENSP00000377865

ENSP00000229214	ENSP00000379607
ENSP00000229214	ENSP00000379888
ENSP00000229214	ENSP00000380982
ENSP00000229214	ENSP00000381031
ENSP00000229214	ENSP00000384474
ENSP00000229214	ENSP00000386134
ENSP00000229214	ENSP00000389103
ENSP00000229214	ENSP00000391723
ENSP00000229214	ENSP00000399454
ENSP00000229214	ENSP00000401980
ENSP00000229214	ENSP00000402084
ENSP00000229214	ENSP00000402338
ENSP00000229214	ENSP00000402869
ENSP00000229214	ENSP00000405614
ENSP00000229214	ENSP00000416959
ENSP00000229214	ENSP00000417229
ENSP00000229214	ENSP00000420195
ENSP00000229238	ENSP00000301585
ENSP00000229251	ENSP00000261712
ENSP00000229251	ENSP00000264389
ENSP00000229251	ENSP00000268717
ENSP00000229251	ENSP00000299259
ENSP00000229251	ENSP00000301764
ENSP00000229251	ENSP00000302955
ENSP00000229251	ENSP00000304102
ENSP00000229251	ENSP00000304822
ENSP00000229251	ENSP00000333568
ENSP00000229251	ENSP00000335557
ENSP00000229251	ENSP00000346340
ENSP00000229251	ENSP00000350512
ENSP00000229251	ENSP00000354125
ENSP00000229251	ENSP00000356438
ENSP00000229251	ENSP00000357260
ENSP00000229251	ENSP00000417229
ENSP00000229304	ENSP00000257430
ENSP00000229304	ENSP00000287322
ENSP00000229307	ENSP00000233146
ENSP00000229307	ENSP00000243326
ENSP00000229307	ENSP00000247005
ENSP00000229307	ENSP00000247970
ENSP00000229307	ENSP00000251020
ENSP00000229307	ENSP00000251289
ENSP00000229307	ENSP00000256429
ENSP00000229307	ENSP00000257566
ENSP00000229307	ENSP00000263360
ENSP00000229307	ENSP00000264709

ENSP00000229307	ENSP00000268058
ENSP00000229307	ENSP00000272134
ENSP00000229307	ENSP00000294740
ENSP00000229307	ENSP00000295901
ENSP00000229307	ENSP00000296273
ENSP00000229307	ENSP00000297792
ENSP00000229307	ENSP00000298767
ENSP00000229307	ENSP00000311816
ENSP00000229307	ENSP00000319169
ENSP00000229307	ENSP00000322915
ENSP00000229307	ENSP00000333633
ENSP00000229307	ENSP00000342307
ENSP00000229307	ENSP00000349049
ENSP00000229307	ENSP00000349213
ENSP00000229307	ENSP00000350094
ENSP00000229307	ENSP00000350708
ENSP00000229307	ENSP00000351250
ENSP00000229307	ENSP00000351490
ENSP00000229307	ENSP00000351552
ENSP00000229307	ENSP00000353622
ENSP00000229307	ENSP00000359567
ENSP00000229307	ENSP00000360621
ENSP00000229307	ENSP00000362592
ENSP00000229307	ENSP00000363089
ENSP00000229307	ENSP00000363193
ENSP00000229307	ENSP00000368253
ENSP00000229307	ENSP00000381657
ENSP00000229314	ENSP00000236192
ENSP00000229314	ENSP00000238831
ENSP00000229314	ENSP00000251038
ENSP00000229314	ENSP00000262812
ENSP00000229314	ENSP00000265175
ENSP00000229314	ENSP00000271375
ENSP00000229314	ENSP00000286448
ENSP00000229314	ENSP00000295119
ENSP00000229314	ENSP00000305459
ENSP00000229314	ENSP00000310003
ENSP00000229314	ENSP00000310521
ENSP00000229314	ENSP00000327716
ENSP00000229314	ENSP00000328079
ENSP00000229314	ENSP00000329419
ENSP00000229314	ENSP00000336724
ENSP00000229314	ENSP00000349887
ENSP00000229314	ENSP00000355308
ENSP00000229314	ENSP00000359305
ENSP00000229314	ENSP00000366225

ENSP00000229314	ENSP00000381293
ENSP00000229314	ENSP00000390783
ENSP00000229314	ENSP00000397441
ENSP00000229379	ENSP00000247655
ENSP00000229379	ENSP00000258424
ENSP00000229379	ENSP00000262077
ENSP00000229379	ENSP00000268379
ENSP00000229379	ENSP00000274787
ENSP00000229379	ENSP00000287022
ENSP00000229379	ENSP00000297564
ENSP00000229379	ENSP00000307786
ENSP00000229379	ENSP00000309565
ENSP00000229379	ENSP00000317159
ENSP00000229379	ENSP00000317780
ENSP00000229379	ENSP00000332887
ENSP00000229379	ENSP00000354499
ENSP00000229379	ENSP00000354554
ENSP00000229379	ENSP00000354876
ENSP00000229379	ENSP00000354982
ENSP00000229379	ENSP00000355572
ENSP00000229379	ENSP00000360782
ENSP00000229379	ENSP00000367934
ENSP00000229395	ENSP00000230056
ENSP00000229395	ENSP00000265148
ENSP00000229395	ENSP00000267807
ENSP00000229395	ENSP00000271715
ENSP00000229395	ENSP00000275780
ENSP00000229395	ENSP00000278572
ENSP00000229395	ENSP00000295951
ENSP00000229395	ENSP00000298317
ENSP00000229395	ENSP00000301019
ENSP00000229395	ENSP00000315702
ENSP00000229395	ENSP00000322915
ENSP00000229395	ENSP00000323288
ENSP00000229395	ENSP00000324020
ENSP00000229395	ENSP00000336587
ENSP00000229395	ENSP00000350071
ENSP00000229395	ENSP00000350094
ENSP00000229395	ENSP00000352676
ENSP00000229395	ENSP00000358810
ENSP00000229395	ENSP00000362424
ENSP00000229395	ENSP00000362578
ENSP00000229395	ENSP00000373331
ENSP00000229416	ENSP00000234396
ENSP00000229416	ENSP00000241052
ENSP00000229416	ENSP00000251038

ENSP00000229416	ENSP00000261601
ENSP00000229416	ENSP00000265316
ENSP00000229416	ENSP00000272227
ENSP00000229416	ENSP00000272286
ENSP00000229416	ENSP00000287936
ENSP00000229416	ENSP00000290597
ENSP00000229416	ENSP00000296786
ENSP00000229416	ENSP00000300648
ENSP00000229416	ENSP00000323313
ENSP00000229416	ENSP00000330836
ENSP00000229416	ENSP00000351284
ENSP00000229416	ENSP00000354995
ENSP00000229416	ENSP00000358140
ENSP00000229416	ENSP00000359258
ENSP00000229416	ENSP00000360183
ENSP00000229416	ENSP00000360268
ENSP00000229416	ENSP00000379217
ENSP00000229416	ENSP00000417963
ENSP00000229595	ENSP00000230859
ENSP00000229595	ENSP00000233146
ENSP00000229595	ENSP00000234296
ENSP00000229595	ENSP00000234420
ENSP00000229595	ENSP00000238789
ENSP00000229595	ENSP00000247219
ENSP00000229595	ENSP00000247815
ENSP00000229595	ENSP00000247843
ENSP00000229595	ENSP00000248633
ENSP00000229595	ENSP00000249636
ENSP00000229595	ENSP00000252622
ENSP00000229595	ENSP00000254037
ENSP00000229595	ENSP00000254719
ENSP00000229595	ENSP00000254900
ENSP00000229595	ENSP00000257177
ENSP00000229595	ENSP00000257789
ENSP00000229595	ENSP00000259008
ENSP00000229595	ENSP00000259050
ENSP00000229595	ENSP00000260810
ENSP00000229595	ENSP00000261211
ENSP00000229595	ENSP00000261424
ENSP00000229595	ENSP00000261601
ENSP00000229595	ENSP00000261819
ENSP00000229595	ENSP00000262300
ENSP00000229595	ENSP00000262315
ENSP00000229595	ENSP00000262608
ENSP00000229595	ENSP00000263043
ENSP00000229595	ENSP00000263121

ENSP00000229595	ENSP00000263201
ENSP00000229595	ENSP00000263208
ENSP00000229595	ENSP00000263377
ENSP00000229595	ENSP00000263390
ENSP00000229595	ENSP00000263805
ENSP00000229595	ENSP00000263956
ENSP00000229595	ENSP00000264169
ENSP00000229595	ENSP00000264183
ENSP00000229595	ENSP00000264926
ENSP00000229595	ENSP00000265056
ENSP00000229595	ENSP00000265073
ENSP00000229595	ENSP00000265081
ENSP00000229595	ENSP00000265140
ENSP00000229595	ENSP00000265970
ENSP00000229595	ENSP00000265990
ENSP00000229595	ENSP00000267064
ENSP00000229595	ENSP00000267430
ENSP00000229595	ENSP00000268043
ENSP00000229595	ENSP00000270176
ENSP00000229595	ENSP00000274140
ENSP00000229595	ENSP00000275780
ENSP00000229595	ENSP00000276072
ENSP00000229595	ENSP00000278412
ENSP00000229595	ENSP00000278616
ENSP00000229595	ENSP00000283882
ENSP00000229595	ENSP00000283928
ENSP00000229595	ENSP00000287239
ENSP00000229595	ENSP00000287598
ENSP00000229595	ENSP00000290649
ENSP00000229595	ENSP00000296273
ENSP00000229595	ENSP00000296783
ENSP00000229595	ENSP00000298139
ENSP00000229595	ENSP00000301280
ENSP00000229595	ENSP00000302478
ENSP00000229595	ENSP00000302530
ENSP00000229595	ENSP00000302955
ENSP00000229595	ENSP00000304593
ENSP00000229595	ENSP00000306983
ENSP00000229595	ENSP00000307143
ENSP00000229595	ENSP00000307208
ENSP00000229595	ENSP00000308944
ENSP00000229595	ENSP00000309141
ENSP00000229595	ENSP00000312244
ENSP00000229595	ENSP00000312981
ENSP00000229595	ENSP00000313171
ENSP00000229595	ENSP00000313243

ENSP00000229595	ENSP00000313420
ENSP00000229595	ENSP00000315700
ENSP00000229595	ENSP00000315791
ENSP00000229595	ENSP00000317087
ENSP00000229595	ENSP00000317636
ENSP00000229595	ENSP00000318977
ENSP00000229595	ENSP00000320485
ENSP00000229595	ENSP00000320885
ENSP00000229595	ENSP00000322570
ENSP00000229595	ENSP00000322957
ENSP00000229595	ENSP00000325677
ENSP00000229595	ENSP00000329466
ENSP00000229595	ENSP00000329933
ENSP00000229595	ENSP00000331310
ENSP00000229595	ENSP00000333667
ENSP00000229595	ENSP00000333836
ENSP00000229595	ENSP00000334061
ENSP00000229595	ENSP00000334675
ENSP00000229595	ENSP00000336606
ENSP00000229595	ENSP00000336842
ENSP00000229595	ENSP00000343392
ENSP00000229595	ENSP00000343741
ENSP00000229595	ENSP00000343745
ENSP00000229595	ENSP00000344741
ENSP00000229595	ENSP00000346453
ENSP00000229595	ENSP00000347232
ENSP00000229595	ENSP00000347602
ENSP00000229595	ENSP00000348010
ENSP00000229595	ENSP00000348089
ENSP00000229595	ENSP00000348564
ENSP00000229595	ENSP00000349205
ENSP00000229595	ENSP00000349213
ENSP00000229595	ENSP00000351284
ENSP00000229595	ENSP00000351697
ENSP00000229595	ENSP00000351947
ENSP00000229595	ENSP00000353332
ENSP00000229595	ENSP00000353622
ENSP00000229595	ENSP00000353793
ENSP00000229595	ENSP00000353904
ENSP00000229595	ENSP00000354522
ENSP00000229595	ENSP00000354916
ENSP00000229595	ENSP00000355051
ENSP00000229595	ENSP00000355866
ENSP00000229595	ENSP00000356530
ENSP00000229595	ENSP00000356744
ENSP00000229595	ENSP00000357597

ENSP00000229595	ENSP00000357887
ENSP00000229595	ENSP00000358478
ENSP00000229595	ENSP00000358854
ENSP00000229595	ENSP00000359531
ENSP00000229595	ENSP00000360116
ENSP00000229595	ENSP00000360621
ENSP00000229595	ENSP00000361998
ENSP00000229595	ENSP00000362352
ENSP00000229595	ENSP00000362441
ENSP00000229595	ENSP00000362592
ENSP00000229595	ENSP00000364591
ENSP00000229595	ENSP00000365025
ENSP00000229595	ENSP00000366395
ENSP00000229595	ENSP00000366565
ENSP00000229595	ENSP00000366974
ENSP00000229595	ENSP00000367406
ENSP00000229595	ENSP00000368349
ENSP00000229595	ENSP00000368438
ENSP00000229595	ENSP00000369411
ENSP00000229595	ENSP00000369695
ENSP00000229595	ENSP00000369716
ENSP00000229595	ENSP00000370151
ENSP00000229595	ENSP00000371475
ENSP00000229595	ENSP00000372023
ENSP00000229595	ENSP00000374213
ENSP00000229595	ENSP00000375809
ENSP00000229595	ENSP00000375986
ENSP00000229595	ENSP00000377262
ENSP00000229595	ENSP00000377747
ENSP00000229595	ENSP00000379372
ENSP00000229595	ENSP00000380129
ENSP00000229595	ENSP00000380504
ENSP00000229595	ENSP00000381657
ENSP00000229595	ENSP00000382688
ENSP00000229595	ENSP00000386239
ENSP00000229595	ENSP00000386759
ENSP00000229595	ENSP00000392028
ENSP00000229595	ENSP00000392985
ENSP00000229595	ENSP00000394791
ENSP00000229595	ENSP00000398824
ENSP00000229595	ENSP00000401445
ENSP00000229595	ENSP00000402084
ENSP00000229595	ENSP00000402515
ENSP00000229595	ENSP00000418734
ENSP00000229758	ENSP00000231487
ENSP00000229758	ENSP00000256442

ENSP00000229758	ENSP00000348554
ENSP00000229758	ENSP00000359206
ENSP00000229758	ENSP00000359356
ENSP00000229758	ENSP00000363322
ENSP00000229758	ENSP00000378529
ENSP00000229769	ENSP00000232424
ENSP00000229769	ENSP00000261755
ENSP00000229769	ENSP00000267430
ENSP00000229769	ENSP00000287647
ENSP00000229769	ENSP00000289081
ENSP00000229769	ENSP00000330875
ENSP00000229769	ENSP00000367808
ENSP00000229769	ENSP00000367910
ENSP00000229769	ENSP00000368438
ENSP00000229769	ENSP00000369497
ENSP00000229769	ENSP00000373952
ENSP00000229771	ENSP00000402935
ENSP00000229922	ENSP00000253083
ENSP00000229922	ENSP00000261170
ENSP00000229922	ENSP00000261249
ENSP00000229922	ENSP00000262428
ENSP00000229922	ENSP00000263265
ENSP00000229922	ENSP00000264335
ENSP00000229922	ENSP00000264995
ENSP00000229922	ENSP00000266058
ENSP00000229922	ENSP00000268661
ENSP00000229922	ENSP00000272203
ENSP00000229922	ENSP00000275605
ENSP00000229922	ENSP00000276416
ENSP00000229922	ENSP00000292385
ENSP00000229922	ENSP00000294016
ENSP00000229922	ENSP00000295685
ENSP00000229922	ENSP00000298288
ENSP00000229922	ENSP00000301843
ENSP00000229922	ENSP00000315859
ENSP00000229922	ENSP00000322170
ENSP00000229922	ENSP00000326238
ENSP00000229922	ENSP00000326806
ENSP00000229922	ENSP00000329858
ENSP00000229922	ENSP00000333950
ENSP00000229922	ENSP00000337463
ENSP00000229922	ENSP00000339845
ENSP00000229922	ENSP00000344874
ENSP00000229922	ENSP00000348163
ENSP00000229922	ENSP00000348602
ENSP00000229922	ENSP00000356825

ENSP00000229922	ENSP00000359380
ENSP00000229922	ENSP00000364289
ENSP00000229922	ENSP00000368305
ENSP00000229922	ENSP00000368824
ENSP00000229922	ENSP00000376410
ENSP00000229922	ENSP00000380431
ENSP00000229922	ENSP00000382723
ENSP00000229922	ENSP00000389516
ENSP00000229922	ENSP00000389934
ENSP00000229922	ENSP00000395863
ENSP00000229922	ENSP00000401287
ENSP00000229922	ENSP00000405864
ENSP00000229922	ENSP00000412805
ENSP00000229922	ENSP00000413625
ENSP00000229922	ENSP00000419361
ENSP00000229971	ENSP00000231487
ENSP00000229971	ENSP00000232501
ENSP00000229971	ENSP00000284811
ENSP00000229971	ENSP00000285021
ENSP00000229971	ENSP00000288985
ENSP00000229971	ENSP00000334675
ENSP00000229971	ENSP00000348089
ENSP00000229971	ENSP00000350708
ENSP00000229971	ENSP00000360621
ENSP00000229971	ENSP00000364270
ENSP00000230036	ENSP00000251038
ENSP00000230036	ENSP00000316333
ENSP00000230036	ENSP00000349687
ENSP00000230036	ENSP00000350425
ENSP00000230036	ENSP00000362334
ENSP00000230036	ENSP00000375881
ENSP00000230048	ENSP00000293406
ENSP00000230048	ENSP00000317872
ENSP00000230048	ENSP00000386227
ENSP00000230050	ENSP00000232905
ENSP00000230050	ENSP00000234170
ENSP00000230050	ENSP00000236051
ENSP00000230050	ENSP00000241600
ENSP00000230050	ENSP00000245539
ENSP00000230050	ENSP00000251038
ENSP00000230050	ENSP00000252543
ENSP00000230050	ENSP00000253099
ENSP00000230050	ENSP00000253788
ENSP00000230050	ENSP00000254037
ENSP00000230050	ENSP00000254940
ENSP00000230050	ENSP00000257829

ENSP00000230050	ENSP00000258455
ENSP00000230050	ENSP00000258654
ENSP00000230050	ENSP00000259469
ENSP00000230050	ENSP00000260102
ENSP00000230050	ENSP00000260443
ENSP00000230050	ENSP00000262584
ENSP00000230050	ENSP00000263629
ENSP00000230050	ENSP00000263657
ENSP00000230050	ENSP00000264995
ENSP00000230050	ENSP00000265100
ENSP00000230050	ENSP00000268661
ENSP00000230050	ENSP00000270625
ENSP00000230050	ENSP00000272418
ENSP00000230050	ENSP00000274242
ENSP00000230050	ENSP00000278572
ENSP00000230050	ENSP00000283109
ENSP00000230050	ENSP00000287038
ENSP00000230050	ENSP00000289371
ENSP00000230050	ENSP00000294189
ENSP00000230050	ENSP00000296411
ENSP00000230050	ENSP00000296577
ENSP00000230050	ENSP00000296674
ENSP00000230050	ENSP00000296802
ENSP00000230050	ENSP00000298283
ENSP00000230050	ENSP00000298746
ENSP00000230050	ENSP00000300151
ENSP00000230050	ENSP00000302886
ENSP00000230050	ENSP00000302896
ENSP00000230050	ENSP00000303434
ENSP00000230050	ENSP00000306548
ENSP00000230050	ENSP00000307525
ENSP00000230050	ENSP00000307889
ENSP00000230050	ENSP00000308897
ENSP00000230050	ENSP00000309334
ENSP00000230050	ENSP00000309830
ENSP00000230050	ENSP00000310448
ENSP00000230050	ENSP00000311028
ENSP00000230050	ENSP00000311430
ENSP00000230050	ENSP00000312735
ENSP00000230050	ENSP00000317376
ENSP00000230050	ENSP00000317992
ENSP00000230050	ENSP00000318646
ENSP00000230050	ENSP00000320917
ENSP00000230050	ENSP00000322396
ENSP00000230050	ENSP00000322419
ENSP00000230050	ENSP00000323856

ENSP00000230050	ENSP00000325312
ENSP00000230050	ENSP00000326981
ENSP00000230050	ENSP00000328854
ENSP00000230050	ENSP00000329466
ENSP00000230050	ENSP00000331019
ENSP00000230050	ENSP00000332198
ENSP00000230050	ENSP00000336702
ENSP00000230050	ENSP00000338862
ENSP00000230050	ENSP00000339095
ENSP00000230050	ENSP00000339795
ENSP00000230050	ENSP00000341885
ENSP00000230050	ENSP00000345096
ENSP00000230050	ENSP00000345156
ENSP00000230050	ENSP00000346015
ENSP00000230050	ENSP00000346022
ENSP00000230050	ENSP00000346027
ENSP00000230050	ENSP00000346037
ENSP00000230050	ENSP00000346050
ENSP00000230050	ENSP00000346067
ENSP00000230050	ENSP00000346088
ENSP00000230050	ENSP00000346725
ENSP00000230050	ENSP00000354040
ENSP00000230050	ENSP00000354525
ENSP00000230050	ENSP00000354739
ENSP00000230050	ENSP00000354812
ENSP00000230050	ENSP00000355541
ENSP00000230050	ENSP00000358563
ENSP00000230050	ENSP00000359345
ENSP00000230050	ENSP00000359688
ENSP00000230050	ENSP00000359939
ENSP00000230050	ENSP00000360031
ENSP00000230050	ENSP00000360412
ENSP00000230050	ENSP00000361982
ENSP00000230050	ENSP00000362306
ENSP00000230050	ENSP00000362744
ENSP00000230050	ENSP00000363018
ENSP00000230050	ENSP00000363308
ENSP00000230050	ENSP00000363384
ENSP00000230050	ENSP00000363559
ENSP00000230050	ENSP00000363676
ENSP00000230050	ENSP00000364320
ENSP00000230050	ENSP00000369757
ENSP00000230050	ENSP00000373404
ENSP00000230050	ENSP00000375730
ENSP00000230050	ENSP00000377640
ENSP00000230050	ENSP00000377865

ENSP00000230050	ENSP00000378160
ENSP00000230050	ENSP00000379339
ENSP00000230050	ENSP00000379607
ENSP00000230050	ENSP00000379888
ENSP00000230050	ENSP00000380156
ENSP00000230050	ENSP00000380982
ENSP00000230050	ENSP00000386717
ENSP00000230050	ENSP00000389103
ENSP00000230050	ENSP00000392859
ENSP00000230050	ENSP00000393241
ENSP00000230050	ENSP00000393393
ENSP00000230050	ENSP00000402338
ENSP00000230050	ENSP00000403175
ENSP00000230050	ENSP00000412583
ENSP00000230050	ENSP00000414321
ENSP00000230050	ENSP00000416110
ENSP00000230056	ENSP00000244741
ENSP00000230056	ENSP00000249499
ENSP00000230056	ENSP00000249504
ENSP00000230056	ENSP00000267807
ENSP00000230056	ENSP00000271715
ENSP00000230056	ENSP00000283921
ENSP00000230056	ENSP00000295951
ENSP00000230056	ENSP00000298317
ENSP00000230056	ENSP00000301019
ENSP00000230056	ENSP00000315949
ENSP00000230056	ENSP00000316109
ENSP00000230056	ENSP00000322915
ENSP00000230056	ENSP00000323288
ENSP00000230056	ENSP00000336587
ENSP00000230056	ENSP00000345096
ENSP00000230056	ENSP00000350094
ENSP00000230056	ENSP00000351407
ENSP00000230056	ENSP00000355153
ENSP00000230056	ENSP00000359531
ENSP00000230056	ENSP00000372170
ENSP00000230056	ENSP00000373331
ENSP00000230056	ENSP00000414920
ENSP00000230124	ENSP00000251038
ENSP00000230124	ENSP00000259253
ENSP00000230124	ENSP00000261776
ENSP00000230124	ENSP00000261842
ENSP00000230124	ENSP00000264380
ENSP00000230124	ENSP00000269122
ENSP00000230124	ENSP00000288828
ENSP00000230124	ENSP00000290974

ENSP00000230124	ENSP00000331201
ENSP00000230124	ENSP00000351926
ENSP00000230124	ENSP00000353098
ENSP00000230124	ENSP00000354519
ENSP00000230124	ENSP00000362115
ENSP00000230124	ENSP00000373399
ENSP00000230124	ENSP00000376268
ENSP00000230124	ENSP00000377148
ENSP00000230124	ENSP00000384651
ENSP00000230340	ENSP00000232888
ENSP00000230340	ENSP00000240316
ENSP00000230340	ENSP00000244230
ENSP00000230340	ENSP00000253099
ENSP00000230340	ENSP00000254037
ENSP00000230340	ENSP00000254605
ENSP00000230340	ENSP00000257829
ENSP00000230340	ENSP00000257909
ENSP00000230340	ENSP00000259750
ENSP00000230340	ENSP00000261637
ENSP00000230340	ENSP00000261708
ENSP00000230340	ENSP00000261741
ENSP00000230340	ENSP00000262294
ENSP00000230340	ENSP00000263657
ENSP00000230340	ENSP00000265758
ENSP00000230340	ENSP00000267425
ENSP00000230340	ENSP00000268482
ENSP00000230340	ENSP00000268802
ENSP00000230340	ENSP00000270625
ENSP00000230340	ENSP00000272521
ENSP00000230340	ENSP00000278412
ENSP00000230340	ENSP00000278572
ENSP00000230340	ENSP00000282412
ENSP00000230340	ENSP00000283109
ENSP00000230340	ENSP00000283875
ENSP00000230340	ENSP00000285873
ENSP00000230340	ENSP00000291576
ENSP00000230340	ENSP00000293883
ENSP00000230340	ENSP00000295373
ENSP00000230340	ENSP00000296733
ENSP00000230340	ENSP00000296792
ENSP00000230340	ENSP00000296802
ENSP00000230340	ENSP00000297579
ENSP00000230340	ENSP00000297873
ENSP00000230340	ENSP00000297990
ENSP00000230340	ENSP00000298746
ENSP00000230340	ENSP00000300151

ENSP00000230340	ENSP00000301364
ENSP00000230340	ENSP00000302896
ENSP00000230340	ENSP00000308179
ENSP00000230340	ENSP00000311028
ENSP00000230340	ENSP00000311135
ENSP00000230340	ENSP00000311430
ENSP00000230340	ENSP00000314193
ENSP00000230340	ENSP00000315674
ENSP00000230340	ENSP00000315859
ENSP00000230340	ENSP00000318646
ENSP00000230340	ENSP00000320768
ENSP00000230340	ENSP00000320917
ENSP00000230340	ENSP00000324122
ENSP00000230340	ENSP00000326981
ENSP00000230340	ENSP00000327179
ENSP00000230340	ENSP00000328854
ENSP00000230340	ENSP00000329466
ENSP00000230340	ENSP00000331879
ENSP00000230340	ENSP00000331907
ENSP00000230340	ENSP00000336712
ENSP00000230340	ENSP00000339095
ENSP00000230340	ENSP00000342381
ENSP00000230340	ENSP00000347087
ENSP00000230340	ENSP00000354125
ENSP00000230340	ENSP00000355471
ENSP00000230340	ENSP00000355541
ENSP00000230340	ENSP00000356134
ENSP00000230340	ENSP00000356520
ENSP00000230340	ENSP00000356548
ENSP00000230340	ENSP00000358563
ENSP00000230340	ENSP00000358812
ENSP00000230340	ENSP00000359688
ENSP00000230340	ENSP00000360031
ENSP00000230340	ENSP00000361287
ENSP00000230340	ENSP00000361686
ENSP00000230340	ENSP00000361982
ENSP00000230340	ENSP00000362105
ENSP00000230340	ENSP00000362744
ENSP00000230340	ENSP00000363308
ENSP00000230340	ENSP00000363642
ENSP00000230340	ENSP00000363676
ENSP00000230340	ENSP00000363746
ENSP00000230340	ENSP00000366557
ENSP00000230340	ENSP00000369162
ENSP00000230340	ENSP00000369757
ENSP00000230340	ENSP00000370589

ENSP00000230340	ENSP00000371101
ENSP00000230340	ENSP00000371169
ENSP00000230340	ENSP00000372695
ENSP00000230340	ENSP00000374219
ENSP00000230340	ENSP00000375986
ENSP00000230340	ENSP00000377040
ENSP00000230340	ENSP00000378529
ENSP00000230340	ENSP00000379888
ENSP00000230340	ENSP00000384302
ENSP00000230340	ENSP00000387303
ENSP00000230340	ENSP00000392601
ENSP00000230340	ENSP00000399454
ENSP00000230340	ENSP00000402338
ENSP00000230340	ENSP00000402869
ENSP00000230340	ENSP00000405614
ENSP00000230340	ENSP00000417229
ENSP00000230419	ENSP00000296084
ENSP00000230419	ENSP00000324857
ENSP00000230419	ENSP00000344456
ENSP00000230431	ENSP00000276603
ENSP00000230431	ENSP00000350249
ENSP00000230449	ENSP00000243253
ENSP00000230449	ENSP00000248139
ENSP00000230449	ENSP00000249647
ENSP00000230449	ENSP00000251038
ENSP00000230449	ENSP00000253861
ENSP00000230449	ENSP00000261531
ENSP00000230449	ENSP00000267484
ENSP00000230449	ENSP00000272427
ENSP00000230449	ENSP00000272972
ENSP00000230449	ENSP00000273666
ENSP00000230449	ENSP00000311505
ENSP00000230449	ENSP00000317714
ENSP00000230449	ENSP00000323377
ENSP00000230449	ENSP00000326514
ENSP00000230449	ENSP00000328182
ENSP00000230449	ENSP00000334100
ENSP00000230449	ENSP00000341538
ENSP00000230449	ENSP00000348215
ENSP00000230449	ENSP00000348602
ENSP00000230449	ENSP00000353564
ENSP00000230449	ENSP00000355721
ENSP00000230449	ENSP00000359222
ENSP00000230449	ENSP00000360532
ENSP00000230449	ENSP00000361669
ENSP00000230449	ENSP00000362399

ENSP00000230449	ENSP00000365630
ENSP00000230449	ENSP00000369863
ENSP00000230449	ENSP00000373399
ENSP00000230449	ENSP00000380450
ENSP00000230449	ENSP00000381293
ENSP00000230449	ENSP00000382177
ENSP00000230449	ENSP00000382492
ENSP00000230449	ENSP00000389934
ENSP00000230449	ENSP00000411409
ENSP00000230510	ENSP00000233623
ENSP00000230510	ENSP00000251038
ENSP00000230510	ENSP00000252999
ENSP00000230510	ENSP00000256442
ENSP00000230510	ENSP00000260731
ENSP00000230510	ENSP00000261211
ENSP00000230510	ENSP00000261531
ENSP00000230510	ENSP00000261597
ENSP00000230510	ENSP00000270861
ENSP00000230510	ENSP00000271452
ENSP00000230510	ENSP00000274289
ENSP00000230510	ENSP00000276014
ENSP00000230510	ENSP00000287598
ENSP00000230510	ENSP00000296509
ENSP00000230510	ENSP00000300249
ENSP00000230510	ENSP00000300584
ENSP00000230510	ENSP00000302530
ENSP00000230510	ENSP00000305941
ENSP00000230510	ENSP00000305958
ENSP00000230510	ENSP00000311083
ENSP00000230510	ENSP00000316121
ENSP00000230510	ENSP00000322180
ENSP00000230510	ENSP00000331831
ENSP00000230510	ENSP00000334280
ENSP00000230510	ENSP00000335463
ENSP00000230510	ENSP00000346791
ENSP00000230510	ENSP00000348554
ENSP00000230510	ENSP00000356448
ENSP00000230510	ENSP00000367208
ENSP00000230510	ENSP00000378350
ENSP00000230510	ENSP00000381003
ENSP00000230510	ENSP00000382562
ENSP00000230510	ENSP00000383145
ENSP00000230568	ENSP00000266058
ENSP00000230568	ENSP00000370034
ENSP00000230588	ENSP00000242152
ENSP00000230588	ENSP00000252999

ENSP00000230588	ENSP00000256010
ENSP00000230588	ENSP00000256857
ENSP00000230588	ENSP00000264708
ENSP00000230588	ENSP00000265023
ENSP00000230588	ENSP00000276414
ENSP00000230588	ENSP00000282091
ENSP00000230588	ENSP00000301740
ENSP00000230588	ENSP00000321106
ENSP00000230588	ENSP00000335657
ENSP00000230588	ENSP00000346839
ENSP00000230588	ENSP00000355627
ENSP00000230588	ENSP00000356213
ENSP00000230588	ENSP00000387662
ENSP00000230792	ENSP00000293406
ENSP00000230792	ENSP00000402338
ENSP00000230859	ENSP00000236051
ENSP00000230859	ENSP00000244769
ENSP00000230859	ENSP00000246802
ENSP00000230859	ENSP00000247843
ENSP00000230859	ENSP00000251038
ENSP00000230859	ENSP00000251607
ENSP00000230859	ENSP00000252115
ENSP00000230859	ENSP00000254037
ENSP00000230859	ENSP00000256383
ENSP00000230859	ENSP00000259469
ENSP00000230859	ENSP00000260129
ENSP00000230859	ENSP00000260403
ENSP00000230859	ENSP00000260810
ENSP00000230859	ENSP00000260956
ENSP00000230859	ENSP00000261015
ENSP00000230859	ENSP00000262056
ENSP00000230859	ENSP00000262077
ENSP00000230859	ENSP00000262584
ENSP00000230859	ENSP00000263201
ENSP00000230859	ENSP00000263208
ENSP00000230859	ENSP00000264515
ENSP00000230859	ENSP00000264670
ENSP00000230859	ENSP00000264951
ENSP00000230859	ENSP00000268482
ENSP00000230859	ENSP00000268802
ENSP00000230859	ENSP00000272418
ENSP00000230859	ENSP00000273064
ENSP00000230859	ENSP00000274242
ENSP00000230859	ENSP00000278572
ENSP00000230859	ENSP00000282999
ENSP00000230859	ENSP00000286835

ENSP00000230859	ENSP00000287038
ENSP00000230859	ENSP00000287239
ENSP00000230859	ENSP00000295951
ENSP00000230859	ENSP00000296792
ENSP00000230859	ENSP00000296802
ENSP00000230859	ENSP00000301764
ENSP00000230859	ENSP00000302896
ENSP00000230859	ENSP00000306561
ENSP00000230859	ENSP00000306983
ENSP00000230859	ENSP00000307889
ENSP00000230859	ENSP00000311028
ENSP00000230859	ENSP00000311430
ENSP00000230859	ENSP00000311873
ENSP00000230859	ENSP00000311977
ENSP00000230859	ENSP00000313350
ENSP00000230859	ENSP00000314311
ENSP00000230859	ENSP00000314441
ENSP00000230859	ENSP00000314792
ENSP00000230859	ENSP00000315859
ENSP00000230859	ENSP00000316879
ENSP00000230859	ENSP00000318195
ENSP00000230859	ENSP00000318977
ENSP00000230859	ENSP00000322396
ENSP00000230859	ENSP00000322457
ENSP00000230859	ENSP00000322570
ENSP00000230859	ENSP00000325518
ENSP00000230859	ENSP00000331817
ENSP00000230859	ENSP00000337839
ENSP00000230859	ENSP00000338862
ENSP00000230859	ENSP00000339845
ENSP00000230859	ENSP00000342374
ENSP00000230859	ENSP00000343745
ENSP00000230859	ENSP00000346015
ENSP00000230859	ENSP00000346120
ENSP00000230859	ENSP00000347087
ENSP00000230859	ENSP00000351284
ENSP00000230859	ENSP00000351575
ENSP00000230859	ENSP00000351697
ENSP00000230859	ENSP00000354040
ENSP00000230859	ENSP00000354522
ENSP00000230859	ENSP00000355185
ENSP00000230859	ENSP00000355899
ENSP00000230859	ENSP00000356146
ENSP00000230859	ENSP00000358140
ENSP00000230859	ENSP00000358576
ENSP00000230859	ENSP00000358812

ENSP00000230859	ENSP00000360817
ENSP00000230859	ENSP00000361076
ENSP00000230859	ENSP00000362153
ENSP00000230859	ENSP00000362306
ENSP00000230859	ENSP00000362688
ENSP00000230859	ENSP00000362900
ENSP00000230859	ENSP00000363559
ENSP00000230859	ENSP00000363676
ENSP00000230859	ENSP00000363944
ENSP00000230859	ENSP00000364119
ENSP00000230859	ENSP00000364543
ENSP00000230859	ENSP00000366135
ENSP00000230859	ENSP00000366519
ENSP00000230859	ENSP00000366974
ENSP00000230859	ENSP00000367527
ENSP00000230859	ENSP00000368349
ENSP00000230859	ENSP00000369757
ENSP00000230859	ENSP00000370741
ENSP00000230859	ENSP00000372023
ENSP00000230859	ENSP00000372827
ENSP00000230859	ENSP00000373404
ENSP00000230859	ENSP00000374213
ENSP00000230859	ENSP00000377640
ENSP00000230859	ENSP00000377865
ENSP00000230859	ENSP00000380982
ENSP00000230859	ENSP00000381045
ENSP00000230859	ENSP00000382392
ENSP00000230859	ENSP00000394400
ENSP00000230859	ENSP00000399530
ENSP00000230859	ENSP00000400626
ENSP00000230859	ENSP00000402338
ENSP00000230859	ENSP00000412310
ENSP00000230859	ENSP00000414321
ENSP00000230882	ENSP00000244007
ENSP00000230882	ENSP00000264818
ENSP00000230882	ENSP00000300574
ENSP00000230882	ENSP00000302150
ENSP00000230882	ENSP00000303554
ENSP00000230882	ENSP00000309524
ENSP00000230882	ENSP00000340944
ENSP00000230882	ENSP00000341208
ENSP00000230882	ENSP00000351894
ENSP00000230882	ENSP00000351926
ENSP00000230882	ENSP00000356346
ENSP00000230882	ENSP00000359206
ENSP00000230882	ENSP00000378394

ENSP00000230882	ENSP00000381793
ENSP00000230882	ENSP00000394794
ENSP00000230882	ENSP00000409346
ENSP00000230895	ENSP00000271643
ENSP00000230990	ENSP00000232014
ENSP00000230990	ENSP00000338157
ENSP00000231198	ENSP00000234296
ENSP00000231198	ENSP00000263791
ENSP00000231198	ENSP00000265840
ENSP00000231198	ENSP00000276603
ENSP00000231198	ENSP00000304668
ENSP00000231198	ENSP00000356562
ENSP00000231198	ENSP00000359976
ENSP00000231198	ENSP00000368305
ENSP00000231228	ENSP00000262345
ENSP00000231228	ENSP00000303231
ENSP00000231228	ENSP00000314425
ENSP00000231238	ENSP00000340237
ENSP00000231238	ENSP00000349016
ENSP00000231238	ENSP00000360141
ENSP00000231357	ENSP00000240587
ENSP00000231357	ENSP00000249442
ENSP00000231357	ENSP00000265354
ENSP00000231357	ENSP00000277541
ENSP00000231357	ENSP00000361990
ENSP00000231368	ENSP00000234296
ENSP00000231368	ENSP00000258774
ENSP00000231368	ENSP00000298852
ENSP00000231368	ENSP00000311579
ENSP00000231368	ENSP00000340879
ENSP00000231368	ENSP00000360621
ENSP00000231368	ENSP00000362399
ENSP00000231368	ENSP00000363873
ENSP00000231420	ENSP00000245206
ENSP00000231420	ENSP00000267845
ENSP00000231420	ENSP00000350928
ENSP00000231420	ENSP00000359518
ENSP00000231420	ENSP00000362298
ENSP00000231420	ENSP00000370611
ENSP00000231449	ENSP00000234396
ENSP00000231449	ENSP00000270776
ENSP00000231449	ENSP00000285046
ENSP00000231449	ENSP00000296137
ENSP00000231449	ENSP00000296328
ENSP00000231449	ENSP00000302478
ENSP00000231449	ENSP00000311430

ENSP00000231449	ENSP00000313420
ENSP00000231449	ENSP00000314949
ENSP00000231449	ENSP00000317955
ENSP00000231449	ENSP00000326534
ENSP00000231449	ENSP00000327821
ENSP00000231449	ENSP00000344446
ENSP00000231449	ENSP00000348577
ENSP00000231449	ENSP00000353393
ENSP00000231449	ENSP00000354558
ENSP00000231449	ENSP00000355205
ENSP00000231449	ENSP00000356548
ENSP00000231449	ENSP00000360730
ENSP00000231449	ENSP00000366135
ENSP00000231449	ENSP00000366843
ENSP00000231449	ENSP00000377954
ENSP00000231449	ENSP00000415203
ENSP00000231454	ENSP00000256452
ENSP00000231454	ENSP00000384053
ENSP00000231487	ENSP00000232014
ENSP00000231487	ENSP00000234396
ENSP00000231487	ENSP00000237281
ENSP00000231487	ENSP00000244426
ENSP00000231487	ENSP00000244741
ENSP00000231487	ENSP00000246548
ENSP00000231487	ENSP00000247977
ENSP00000231487	ENSP00000250003
ENSP00000231487	ENSP00000250615
ENSP00000231487	ENSP00000251038
ENSP00000231487	ENSP00000251076
ENSP00000231487	ENSP00000252602
ENSP00000231487	ENSP00000253003
ENSP00000231487	ENSP00000253413
ENSP00000231487	ENSP00000256379
ENSP00000231487	ENSP00000256897
ENSP00000231487	ENSP00000258200
ENSP00000231487	ENSP00000258873
ENSP00000231487	ENSP00000259803
ENSP00000231487	ENSP00000260766
ENSP00000231487	ENSP00000261245
ENSP00000231487	ENSP00000261712
ENSP00000231487	ENSP00000262133
ENSP00000231487	ENSP00000262306
ENSP00000231487	ENSP00000262367
ENSP00000231487	ENSP00000262643
ENSP00000231487	ENSP00000262644
ENSP00000231487	ENSP00000263026

ENSP00000231487	ENSP00000263201
ENSP00000231487	ENSP00000263228
ENSP00000231487	ENSP00000264198
ENSP00000231487	ENSP00000264389
ENSP00000231487	ENSP00000264552
ENSP00000231487	ENSP00000265056
ENSP00000231487	ENSP00000265148
ENSP00000231487	ENSP00000265651
ENSP00000231487	ENSP00000265960
ENSP00000231487	ENSP00000266087
ENSP00000231487	ENSP00000266126
ENSP00000231487	ENSP00000270225
ENSP00000231487	ENSP00000270861
ENSP00000231487	ENSP00000273398
ENSP00000231487	ENSP00000274255
ENSP00000231487	ENSP00000274289
ENSP00000231487	ENSP00000275015
ENSP00000231487	ENSP00000276326
ENSP00000231487	ENSP00000276603
ENSP00000231487	ENSP00000276893
ENSP00000231487	ENSP00000277865
ENSP00000231487	ENSP00000278616
ENSP00000231487	ENSP00000279230
ENSP00000231487	ENSP00000281623
ENSP00000231487	ENSP00000281708
ENSP00000231487	ENSP00000282892
ENSP00000231487	ENSP00000284202
ENSP00000231487	ENSP00000287598
ENSP00000231487	ENSP00000290810
ENSP00000231487	ENSP00000293860
ENSP00000231487	ENSP00000295066
ENSP00000231487	ENSP00000296328
ENSP00000231487	ENSP00000296438
ENSP00000231487	ENSP00000296509
ENSP00000231487	ENSP00000297185
ENSP00000231487	ENSP00000298097
ENSP00000231487	ENSP00000298852
ENSP00000231487	ENSP00000298937
ENSP00000231487	ENSP00000299259
ENSP00000231487	ENSP00000299543
ENSP00000231487	ENSP00000302530
ENSP00000231487	ENSP00000303208
ENSP00000231487	ENSP00000304102
ENSP00000231487	ENSP00000307786
ENSP00000231487	ENSP00000310040
ENSP00000231487	ENSP00000310332

ENSP00000231487	ENSP00000310841
ENSP00000231487	ENSP00000311083
ENSP00000231487	ENSP00000311505
ENSP00000231487	ENSP00000312870
ENSP00000231487	ENSP00000313034
ENSP00000231487	ENSP00000313159
ENSP00000231487	ENSP00000314004
ENSP00000231487	ENSP00000319482
ENSP00000231487	ENSP00000323687
ENSP00000231487	ENSP00000323822
ENSP00000231487	ENSP00000329967
ENSP00000231487	ENSP00000330098
ENSP00000231487	ENSP00000330484
ENSP00000231487	ENSP00000336842
ENSP00000231487	ENSP00000338413
ENSP00000231487	ENSP00000343741
ENSP00000231487	ENSP00000344456
ENSP00000231487	ENSP00000344866
ENSP00000231487	ENSP00000345988
ENSP00000231487	ENSP00000346240
ENSP00000231487	ENSP00000347733
ENSP00000231487	ENSP00000347836
ENSP00000231487	ENSP00000350009
ENSP00000231487	ENSP00000350012
ENSP00000231487	ENSP00000350512
ENSP00000231487	ENSP00000351052
ENSP00000231487	ENSP00000352121
ENSP00000231487	ENSP00000352522
ENSP00000231487	ENSP00000354201
ENSP00000231487	ENSP00000354340
ENSP00000231487	ENSP00000354558
ENSP00000231487	ENSP00000354720
ENSP00000231487	ENSP00000355384
ENSP00000231487	ENSP00000355511
ENSP00000231487	ENSP00000355827
ENSP00000231487	ENSP00000356211
ENSP00000231487	ENSP00000356652
ENSP00000231487	ENSP00000358022
ENSP00000231487	ENSP00000359096
ENSP00000231487	ENSP00000359100
ENSP00000231487	ENSP00000359206
ENSP00000231487	ENSP00000360116
ENSP00000231487	ENSP00000360992
ENSP00000231487	ENSP00000361965
ENSP00000231487	ENSP00000362110
ENSP00000231487	ENSP00000362334

ENSP00000231487	ENSP00000362413
ENSP00000231487	ENSP00000363779
ENSP00000231487	ENSP00000364742
ENSP00000231487	ENSP00000365534
ENSP00000231487	ENSP00000366271
ENSP00000231487	ENSP00000366620
ENSP00000231487	ENSP00000367208
ENSP00000231487	ENSP00000367319
ENSP00000231487	ENSP00000367705
ENSP00000231487	ENSP00000368438
ENSP00000231487	ENSP00000368589
ENSP00000231487	ENSP00000369335
ENSP00000231487	ENSP00000375082
ENSP00000231487	ENSP00000376945
ENSP00000231487	ENSP00000377280
ENSP00000231487	ENSP00000379430
ENSP00000231487	ENSP00000379457
ENSP00000231487	ENSP00000379607
ENSP00000231487	ENSP00000380256
ENSP00000231487	ENSP00000381863
ENSP00000231487	ENSP00000383719
ENSP00000231487	ENSP00000384109
ENSP00000231487	ENSP00000389934
ENSP00000231487	ENSP00000393154
ENSP00000231487	ENSP00000393596
ENSP00000231487	ENSP00000394071
ENSP00000231487	ENSP00000394869
ENSP00000231487	ENSP00000395574
ENSP00000231487	ENSP00000401566
ENSP00000231487	ENSP00000413720
ENSP00000231487	ENSP00000419494
ENSP00000231498	ENSP00000238112
ENSP00000231498	ENSP00000245544
ENSP00000231498	ENSP00000247970
ENSP00000231498	ENSP00000251038
ENSP00000231498	ENSP00000252115
ENSP00000231498	ENSP00000255465
ENSP00000231498	ENSP00000256442
ENSP00000231498	ENSP00000261396
ENSP00000231498	ENSP00000261531
ENSP00000231498	ENSP00000262077
ENSP00000231498	ENSP00000262193
ENSP00000231498	ENSP00000262643
ENSP00000231498	ENSP00000262765
ENSP00000231498	ENSP00000262982
ENSP00000231498	ENSP00000264883

ENSP00000231498	ENSP00000265056
ENSP00000231498	ENSP00000270223
ENSP00000231498	ENSP00000283195
ENSP00000231498	ENSP00000290158
ENSP00000231498	ENSP00000290231
ENSP00000231498	ENSP00000290541
ENSP00000231498	ENSP00000291688
ENSP00000231498	ENSP00000294008
ENSP00000231498	ENSP00000296411
ENSP00000231498	ENSP00000296509
ENSP00000231498	ENSP00000296577
ENSP00000231498	ENSP00000296755
ENSP00000231498	ENSP00000298139
ENSP00000231498	ENSP00000300231
ENSP00000231498	ENSP00000301364
ENSP00000231498	ENSP00000302886
ENSP00000231498	ENSP00000307939
ENSP00000231498	ENSP00000308622
ENSP00000231498	ENSP00000310668
ENSP00000231498	ENSP00000314129
ENSP00000231498	ENSP00000314440
ENSP00000231498	ENSP00000315791
ENSP00000231498	ENSP00000316032
ENSP00000231498	ENSP00000317334
ENSP00000231498	ENSP00000325312
ENSP00000231498	ENSP00000329466
ENSP00000231498	ENSP00000331817
ENSP00000231498	ENSP00000335357
ENSP00000231498	ENSP00000336712
ENSP00000231498	ENSP00000343701
ENSP00000231498	ENSP00000347232
ENSP00000231498	ENSP00000348577
ENSP00000231498	ENSP00000351284
ENSP00000231498	ENSP00000352021
ENSP00000231498	ENSP00000352400
ENSP00000231498	ENSP00000354742
ENSP00000231498	ENSP00000356548
ENSP00000231498	ENSP00000360286
ENSP00000231498	ENSP00000360532
ENSP00000231498	ENSP00000362900
ENSP00000231498	ENSP00000368797
ENSP00000231498	ENSP00000369042
ENSP00000231498	ENSP00000371155
ENSP00000231498	ENSP00000372857
ENSP00000231498	ENSP00000373918
ENSP00000231498	ENSP00000379568

ENSP00000231498	ENSP00000379607
ENSP00000231498	ENSP00000382779
ENSP00000231498	ENSP00000386992
ENSP00000231498	ENSP00000413632
ENSP00000231524	ENSP00000250113
ENSP00000231524	ENSP00000251968
ENSP00000231524	ENSP00000254336
ENSP00000231524	ENSP00000257909
ENSP00000231524	ENSP00000263925
ENSP00000231524	ENSP00000264926
ENSP00000231524	ENSP00000269122
ENSP00000231524	ENSP00000279036
ENSP00000231524	ENSP00000292180
ENSP00000231524	ENSP00000292363
ENSP00000231524	ENSP00000298295
ENSP00000231524	ENSP00000299853
ENSP00000231524	ENSP00000300245
ENSP00000231524	ENSP00000313881
ENSP00000231524	ENSP00000315950
ENSP00000231524	ENSP00000323913
ENSP00000231524	ENSP00000328203
ENSP00000231524	ENSP00000342087
ENSP00000231524	ENSP00000343129
ENSP00000231524	ENSP00000347836
ENSP00000231524	ENSP00000359000
ENSP00000231524	ENSP00000360116
ENSP00000231524	ENSP00000360183
ENSP00000231524	ENSP00000363095
ENSP00000231524	ENSP00000365924
ENSP00000231524	ENSP00000366607
ENSP00000231524	ENSP00000370410
ENSP00000231524	ENSP00000376871
ENSP00000231524	ENSP00000398677
ENSP00000231524	ENSP00000408236
ENSP00000231524	ENSP00000417281
ENSP00000231572	ENSP00000256854
ENSP00000231572	ENSP00000259075
ENSP00000231572	ENSP00000262027
ENSP00000231572	ENSP00000264161
ENSP00000231572	ENSP00000268835
ENSP00000231572	ENSP00000275015
ENSP00000231572	ENSP00000282276
ENSP00000231572	ENSP00000296509
ENSP00000231572	ENSP00000303279
ENSP00000231572	ENSP00000307567
ENSP00000231572	ENSP00000315859

ENSP00000231572	ENSP00000348442
ENSP00000231572	ENSP00000355890
ENSP00000231572	ENSP00000364794
ENSP00000231572	ENSP00000364815
ENSP00000231572	ENSP00000368790
ENSP00000231572	ENSP00000369038
ENSP00000231572	ENSP00000370408
ENSP00000231572	ENSP00000377954
ENSP00000231572	ENSP00000379607
ENSP00000231572	ENSP00000401121
ENSP00000231572	ENSP00000403359
ENSP00000231572	ENSP00000415316
ENSP00000231668	ENSP00000236192
ENSP00000231668	ENSP00000263897
ENSP00000231668	ENSP00000286448
ENSP00000231668	ENSP00000294179
ENSP00000231668	ENSP00000305255
ENSP00000231668	ENSP00000305810
ENSP00000231668	ENSP00000320508
ENSP00000231668	ENSP00000324628
ENSP00000231668	ENSP00000329623
ENSP00000231668	ENSP00000364289
ENSP00000231668	ENSP00000374455
ENSP00000231668	ENSP00000381293
ENSP00000231668	ENSP00000390783
ENSP00000231706	ENSP00000353073
ENSP00000231749	ENSP00000300181
ENSP00000231749	ENSP00000306906
ENSP00000231790	ENSP00000233146
ENSP00000231790	ENSP00000234420
ENSP00000231790	ENSP00000234626
ENSP00000231790	ENSP00000249910
ENSP00000231790	ENSP00000258428
ENSP00000231790	ENSP00000259008
ENSP00000231790	ENSP00000260947
ENSP00000231790	ENSP00000261424
ENSP00000231790	ENSP00000261531
ENSP00000231790	ENSP00000261881
ENSP00000231790	ENSP00000263187
ENSP00000231790	ENSP00000264169
ENSP00000231790	ENSP00000264951
ENSP00000231790	ENSP00000265081
ENSP00000231790	ENSP00000265433
ENSP00000231790	ENSP00000265849
ENSP00000231790	ENSP00000265970
ENSP00000231790	ENSP00000267430

ENSP00000231790	ENSP00000278616
ENSP00000231790	ENSP00000282050
ENSP00000231790	ENSP00000285021
ENSP00000231790	ENSP00000286800
ENSP00000231790	ENSP00000287295
ENSP00000231790	ENSP00000287647
ENSP00000231790	ENSP00000290158
ENSP00000231790	ENSP00000307853
ENSP00000231790	ENSP00000310520
ENSP00000231790	ENSP00000311766
ENSP00000231790	ENSP00000311873
ENSP00000231790	ENSP00000313420
ENSP00000231790	ENSP00000318977
ENSP00000231790	ENSP00000322570
ENSP00000231790	ENSP00000324127
ENSP00000231790	ENSP00000324463
ENSP00000231790	ENSP00000325863
ENSP00000231790	ENSP00000330343
ENSP00000231790	ENSP00000333253
ENSP00000231790	ENSP00000343129
ENSP00000231790	ENSP00000343190
ENSP00000231790	ENSP00000346236
ENSP00000231790	ENSP00000347232
ENSP00000231790	ENSP00000348020
ENSP00000231790	ENSP00000348550
ENSP00000231790	ENSP00000350283
ENSP00000231790	ENSP00000351284
ENSP00000231790	ENSP00000351697
ENSP00000231790	ENSP00000354497
ENSP00000231790	ENSP00000355185
ENSP00000231790	ENSP00000357283
ENSP00000231790	ENSP00000358576
ENSP00000231790	ENSP00000359652
ENSP00000231790	ENSP00000361027
ENSP00000231790	ENSP00000361310
ENSP00000231790	ENSP00000364270
ENSP00000231790	ENSP00000367527
ENSP00000231790	ENSP00000368349
ENSP00000231790	ENSP00000368438
ENSP00000231790	ENSP00000369335
ENSP00000231790	ENSP00000369618
ENSP00000231790	ENSP00000377865
ENSP00000231790	ENSP00000378306
ENSP00000231790	ENSP00000406490
ENSP00000231948	ENSP00000301764
ENSP00000231948	ENSP00000304102

ENSP00000231948	ENSP00000339867
ENSP00000232003	ENSP00000267101
ENSP00000232003	ENSP00000306361
ENSP00000232003	ENSP00000321239
ENSP00000232003	ENSP00000343785
ENSP00000232003	ENSP00000346839
ENSP00000232014	ENSP00000247970
ENSP00000232014	ENSP00000249373
ENSP00000232014	ENSP00000250894
ENSP00000232014	ENSP00000251871
ENSP00000232014	ENSP00000256379
ENSP00000232014	ENSP00000257749
ENSP00000232014	ENSP00000261708
ENSP00000232014	ENSP00000262238
ENSP00000232014	ENSP00000262367
ENSP00000232014	ENSP00000262904
ENSP00000232014	ENSP00000264380
ENSP00000232014	ENSP00000268058
ENSP00000232014	ENSP00000270225
ENSP00000232014	ENSP00000284719
ENSP00000232014	ENSP00000300101
ENSP00000232014	ENSP00000303315
ENSP00000232014	ENSP00000304803
ENSP00000232014	ENSP00000307235
ENSP00000232014	ENSP00000310596
ENSP00000232014	ENSP00000314444
ENSP00000232014	ENSP00000320291
ENSP00000232014	ENSP00000320485
ENSP00000232014	ENSP00000323822
ENSP00000232014	ENSP00000328103
ENSP00000232014	ENSP00000329357
ENSP00000232014	ENSP00000338157
ENSP00000232014	ENSP00000339845
ENSP00000232014	ENSP00000342307
ENSP00000232014	ENSP00000347978
ENSP00000232014	ENSP00000348551
ENSP00000232014	ENSP00000353362
ENSP00000232014	ENSP00000353622
ENSP00000232014	ENSP00000357047
ENSP00000232014	ENSP00000357907
ENSP00000232014	ENSP00000359988
ENSP00000232014	ENSP00000364912
ENSP00000232014	ENSP00000367705
ENSP00000232014	ENSP00000369716
ENSP00000232014	ENSP00000377655
ENSP00000232014	ENSP00000384434

ENSP00000232014	ENSP00000417980
ENSP00000232014	ENSP00000418210
ENSP00000232424	ENSP00000262367
ENSP00000232424	ENSP00000266744
ENSP00000232424	ENSP00000272937
ENSP00000232424	ENSP00000277541
ENSP00000232424	ENSP00000289081
ENSP00000232424	ENSP00000302216
ENSP00000232424	ENSP00000311825
ENSP00000232424	ENSP00000314949
ENSP00000232424	ENSP00000330875
ENSP00000232424	ENSP00000331057
ENSP00000232424	ENSP00000339004
ENSP00000232424	ENSP00000347140
ENSP00000232424	ENSP00000352514
ENSP00000232424	ENSP00000353735
ENSP00000232424	ENSP00000355759
ENSP00000232424	ENSP00000357348
ENSP00000232424	ENSP00000358105
ENSP00000232424	ENSP00000358417
ENSP00000232424	ENSP00000361824
ENSP00000232424	ENSP00000365735
ENSP00000232424	ENSP00000367910
ENSP00000232424	ENSP00000367972
ENSP00000232424	ENSP00000373952
ENSP00000232424	ENSP00000385021
ENSP00000232458	ENSP00000256442
ENSP00000232458	ENSP00000260363
ENSP00000232458	ENSP00000261833
ENSP00000232458	ENSP00000262643
ENSP00000232458	ENSP00000270861
ENSP00000232458	ENSP00000276014
ENSP00000232458	ENSP00000309871
ENSP00000232458	ENSP00000317985
ENSP00000232458	ENSP00000333950
ENSP00000232458	ENSP00000348349
ENSP00000232458	ENSP00000355731
ENSP00000232458	ENSP00000360532
ENSP00000232458	ENSP00000363921
ENSP00000232458	ENSP00000378624
ENSP00000232458	ENSP00000381045
ENSP00000232458	ENSP00000381565
ENSP00000232458	ENSP00000381577
ENSP00000232496	ENSP00000350447
ENSP00000232501	ENSP00000247977
ENSP00000232501	ENSP00000262144

ENSP00000232501	ENSP00000293883
ENSP00000232501	ENSP00000318674
ENSP00000232501	ENSP00000321386
ENSP00000232501	ENSP00000321927
ENSP00000232501	ENSP00000330098
ENSP00000232501	ENSP00000339881
ENSP00000232501	ENSP00000366135
ENSP00000232501	ENSP00000366271
ENSP00000232501	ENSP00000371546
ENSP00000232501	ENSP00000382779
ENSP00000232501	ENSP00000382834
ENSP00000232501	ENSP00000398028
ENSP00000232501	ENSP00000418111
ENSP00000232607	ENSP00000251038
ENSP00000232607	ENSP00000255465
ENSP00000232607	ENSP00000256442
ENSP00000232607	ENSP00000263246
ENSP00000232607	ENSP00000267103
ENSP00000232607	ENSP00000278060
ENSP00000232607	ENSP00000278412
ENSP00000232607	ENSP00000290332
ENSP00000232607	ENSP00000300231
ENSP00000232607	ENSP00000300648
ENSP00000232607	ENSP00000302640
ENSP00000232607	ENSP00000302955
ENSP00000232607	ENSP00000320646
ENSP00000232607	ENSP00000331719
ENSP00000232607	ENSP00000336856
ENSP00000232607	ENSP00000343190
ENSP00000232607	ENSP00000345681
ENSP00000232607	ENSP00000346050
ENSP00000232607	ENSP00000346155
ENSP00000232607	ENSP00000352706
ENSP00000232607	ENSP00000354522
ENSP00000232607	ENSP00000355922
ENSP00000232607	ENSP00000360532
ENSP00000232607	ENSP00000361642
ENSP00000232607	ENSP00000365534
ENSP00000232607	ENSP00000366030
ENSP00000232607	ENSP00000367029
ENSP00000232607	ENSP00000368439
ENSP00000232607	ENSP00000369100
ENSP00000232607	ENSP00000379339
ENSP00000232607	ENSP00000420588
ENSP00000232744	ENSP00000251038
ENSP00000232744	ENSP00000273480

ENSP00000232744	ENSP00000358147
ENSP00000232744	ENSP00000361705
ENSP00000232744	ENSP00000384109
ENSP00000232854	ENSP00000253233
ENSP00000232854	ENSP00000265857
ENSP00000232854	ENSP00000353741
ENSP00000232854	ENSP00000359904
ENSP00000232854	ENSP00000368790
ENSP00000232888	ENSP00000243563
ENSP00000232888	ENSP00000244230
ENSP00000232888	ENSP00000257829
ENSP00000232888	ENSP00000261637
ENSP00000232888	ENSP00000261708
ENSP00000232888	ENSP00000262265
ENSP00000232888	ENSP00000267425
ENSP00000232888	ENSP00000275820
ENSP00000232888	ENSP00000280350
ENSP00000232888	ENSP00000285873
ENSP00000232888	ENSP00000291576
ENSP00000232888	ENSP00000296792
ENSP00000232888	ENSP00000297579
ENSP00000232888	ENSP00000297990
ENSP00000232888	ENSP00000298292
ENSP00000232888	ENSP00000308179
ENSP00000232888	ENSP00000311028
ENSP00000232888	ENSP00000311135
ENSP00000232888	ENSP00000314193
ENSP00000232888	ENSP00000315674
ENSP00000232888	ENSP00000321449
ENSP00000232888	ENSP00000321810
ENSP00000232888	ENSP00000323302
ENSP00000232888	ENSP00000326981
ENSP00000232888	ENSP00000327179
ENSP00000232888	ENSP00000328854
ENSP00000232888	ENSP00000329466
ENSP00000232888	ENSP00000339145
ENSP00000232888	ENSP00000353246
ENSP00000232888	ENSP00000354040
ENSP00000232888	ENSP00000355471
ENSP00000232888	ENSP00000355541
ENSP00000232888	ENSP00000358812
ENSP00000232888	ENSP00000360327
ENSP00000232888	ENSP00000363313
ENSP00000232888	ENSP00000363642
ENSP00000232888	ENSP00000363746
ENSP00000232888	ENSP00000370258

ENSP00000232888	ENSP00000370589
ENSP00000232888	ENSP00000371169
ENSP00000232888	ENSP00000372695
ENSP00000232888	ENSP00000378529
ENSP00000232888	ENSP00000384302
ENSP00000232888	ENSP00000399454
ENSP00000232888	ENSP00000402869
ENSP00000232888	ENSP00000405614
ENSP00000232905	ENSP00000240922
ENSP00000232905	ENSP00000241600
ENSP00000232905	ENSP00000245539
ENSP00000232905	ENSP00000246337
ENSP00000232905	ENSP00000253108
ENSP00000232905	ENSP00000256383
ENSP00000232905	ENSP00000258455
ENSP00000232905	ENSP00000260129
ENSP00000232905	ENSP00000261868
ENSP00000232905	ENSP00000262803
ENSP00000232905	ENSP00000265753
ENSP00000232905	ENSP00000266126
ENSP00000232905	ENSP00000270625
ENSP00000232905	ENSP00000271764
ENSP00000232905	ENSP00000272418
ENSP00000232905	ENSP00000274606
ENSP00000232905	ENSP00000276201
ENSP00000232905	ENSP00000278572
ENSP00000232905	ENSP00000278618
ENSP00000232905	ENSP00000283109
ENSP00000232905	ENSP00000283875
ENSP00000232905	ENSP00000295930
ENSP00000232905	ENSP00000296674
ENSP00000232905	ENSP00000296802
ENSP00000232905	ENSP00000301364
ENSP00000232905	ENSP00000302768
ENSP00000232905	ENSP00000302896
ENSP00000232905	ENSP00000304854
ENSP00000232905	ENSP00000310129
ENSP00000232905	ENSP00000311028
ENSP00000232905	ENSP00000316879
ENSP00000232905	ENSP00000317376
ENSP00000232905	ENSP00000318646
ENSP00000232905	ENSP00000323856
ENSP00000232905	ENSP00000326981
ENSP00000232905	ENSP00000331019
ENSP00000232905	ENSP00000336702
ENSP00000232905	ENSP00000338235

ENSP00000232905	ENSP00000339095
ENSP00000232905	ENSP00000339844
ENSP00000232905	ENSP00000340281
ENSP00000232905	ENSP00000341885
ENSP00000232905	ENSP00000346050
ENSP00000232905	ENSP00000346067
ENSP00000232905	ENSP00000352738
ENSP00000232905	ENSP00000353393
ENSP00000232905	ENSP00000354125
ENSP00000232905	ENSP00000354919
ENSP00000232905	ENSP00000357051
ENSP00000232905	ENSP00000358140
ENSP00000232905	ENSP00000360473
ENSP00000232905	ENSP00000362576
ENSP00000232905	ENSP00000362688
ENSP00000232905	ENSP00000362744
ENSP00000232905	ENSP00000363308
ENSP00000232905	ENSP00000363921
ENSP00000232905	ENSP00000364119
ENSP00000232905	ENSP00000368927
ENSP00000232905	ENSP00000369038
ENSP00000232905	ENSP00000369757
ENSP00000232905	ENSP00000370258
ENSP00000232905	ENSP00000371475
ENSP00000232905	ENSP00000379339
ENSP00000232905	ENSP00000379888
ENSP00000232905	ENSP00000380431
ENSP00000232905	ENSP00000384474
ENSP00000232905	ENSP00000393241
ENSP00000232905	ENSP00000396937
ENSP00000232905	ENSP00000403175
ENSP00000232905	ENSP00000412583
ENSP00000232905	ENSP00000414321
ENSP00000232905	ENSP00000416110
ENSP00000232905	ENSP00000417229
ENSP00000232905	ENSP00000420714
ENSP00000232978	ENSP00000233623
ENSP00000232978	ENSP00000283351
ENSP00000232978	ENSP00000294053
ENSP00000232978	ENSP00000305958
ENSP00000232978	ENSP00000315859
ENSP00000232978	ENSP00000326424
ENSP00000232978	ENSP00000336724
ENSP00000232978	ENSP00000345793
ENSP00000232978	ENSP00000346791
ENSP00000232978	ENSP00000348551

ENSP00000232978	ENSP00000356579
ENSP00000232978	ENSP00000360183
ENSP00000232978	ENSP00000360329
ENSP00000232978	ENSP00000365439
ENSP00000232978	ENSP00000366843
ENSP00000232978	ENSP00000367208
ENSP00000232978	ENSP00000368976
ENSP00000232978	ENSP00000381003
ENSP00000232978	ENSP00000386621
ENSP00000233055	ENSP00000245923
ENSP00000233055	ENSP00000251775
ENSP00000233055	ENSP00000267484
ENSP00000233055	ENSP00000273258
ENSP00000233055	ENSP00000337838
ENSP00000233055	ENSP00000344106
ENSP00000233055	ENSP00000363349
ENSP00000233057	ENSP00000236051
ENSP00000233057	ENSP00000246802
ENSP00000233057	ENSP00000256383
ENSP00000233057	ENSP00000260045
ENSP00000233057	ENSP00000260956
ENSP00000233057	ENSP00000263666
ENSP00000233057	ENSP00000264818
ENSP00000233057	ENSP00000266987
ENSP00000233057	ENSP00000281828
ENSP00000233057	ENSP00000289081
ENSP00000233057	ENSP00000296930
ENSP00000233057	ENSP00000314733
ENSP00000233057	ENSP00000318944
ENSP00000233057	ENSP00000320309
ENSP00000233057	ENSP00000325312
ENSP00000233057	ENSP00000325794
ENSP00000233057	ENSP00000341551
ENSP00000233057	ENSP00000342755
ENSP00000233057	ENSP00000343745
ENSP00000233057	ENSP00000344352
ENSP00000233057	ENSP00000345917
ENSP00000233057	ENSP00000350869
ENSP00000233057	ENSP00000351811
ENSP00000233057	ENSP00000351908
ENSP00000233057	ENSP00000354040
ENSP00000233057	ENSP00000354522
ENSP00000233057	ENSP00000355011
ENSP00000233057	ENSP00000356520
ENSP00000233057	ENSP00000356641
ENSP00000233057	ENSP00000358335

ENSP00000233057	ENSP00000358854
ENSP00000233057	ENSP00000360412
ENSP00000233057	ENSP00000360922
ENSP00000233057	ENSP00000361202
ENSP00000233057	ENSP00000361949
ENSP00000233057	ENSP00000363559
ENSP00000233057	ENSP00000365016
ENSP00000233057	ENSP00000376445
ENSP00000233057	ENSP00000381010
ENSP00000233057	ENSP00000382392
ENSP00000233057	ENSP00000384391
ENSP00000233057	ENSP00000392601
ENSP00000233057	ENSP00000401980
ENSP00000233057	ENSP00000404121
ENSP00000233078	ENSP00000234677
ENSP00000233078	ENSP00000251038
ENSP00000233078	ENSP00000261951
ENSP00000233078	ENSP00000262803
ENSP00000233078	ENSP00000266744
ENSP00000233078	ENSP00000285896
ENSP00000233078	ENSP00000301364
ENSP00000233078	ENSP00000304704
ENSP00000233078	ENSP00000315791
ENSP00000233078	ENSP00000316032
ENSP00000233078	ENSP00000320949
ENSP00000233078	ENSP00000325376
ENSP00000233078	ENSP00000331879
ENSP00000233078	ENSP00000332444
ENSP00000233078	ENSP00000336712
ENSP00000233078	ENSP00000346453
ENSP00000233078	ENSP00000348708
ENSP00000233078	ENSP00000351284
ENSP00000233078	ENSP00000354522
ENSP00000233078	ENSP00000354673
ENSP00000233078	ENSP00000359531
ENSP00000233078	ENSP00000362900
ENSP00000233078	ENSP00000379607
ENSP00000233078	ENSP00000384573
ENSP00000233078	ENSP00000416829
ENSP00000233084	ENSP00000234170
ENSP00000233084	ENSP00000236051
ENSP00000233084	ENSP00000238823
ENSP00000233084	ENSP00000251038
ENSP00000233084	ENSP00000251607
ENSP00000233084	ENSP00000252622
ENSP00000233084	ENSP00000254940

ENSP00000233084	ENSP00000256398
ENSP00000233084	ENSP00000257829
ENSP00000233084	ENSP00000261531
ENSP00000233084	ENSP00000261700
ENSP00000233084	ENSP00000261708
ENSP00000233084	ENSP00000263629
ENSP00000233084	ENSP00000265433
ENSP00000233084	ENSP00000267812
ENSP00000233084	ENSP00000278616
ENSP00000233084	ENSP00000289371
ENSP00000233084	ENSP00000296802
ENSP00000233084	ENSP00000301740
ENSP00000233084	ENSP00000311430
ENSP00000233084	ENSP00000319977
ENSP00000233084	ENSP00000321711
ENSP00000233084	ENSP00000322396
ENSP00000233084	ENSP00000325863
ENSP00000233084	ENSP00000329715
ENSP00000233084	ENSP00000338862
ENSP00000233084	ENSP00000343557
ENSP00000233084	ENSP00000346022
ENSP00000233084	ENSP00000353246
ENSP00000233084	ENSP00000354040
ENSP00000233084	ENSP00000356785
ENSP00000233084	ENSP00000358812
ENSP00000233084	ENSP00000359345
ENSP00000233084	ENSP00000360412
ENSP00000233084	ENSP00000361465
ENSP00000233084	ENSP00000363676
ENSP00000233084	ENSP00000365439
ENSP00000233084	ENSP00000379607
ENSP00000233084	ENSP00000380982
ENSP00000233084	ENSP00000393953
ENSP00000233084	ENSP00000402338
ENSP00000233099	ENSP00000256658
ENSP00000233099	ENSP00000261842
ENSP00000233099	ENSP00000263121
ENSP00000233099	ENSP00000264639
ENSP00000233099	ENSP00000265742
ENSP00000233099	ENSP00000269122
ENSP00000233099	ENSP00000340944
ENSP00000233099	ENSP00000351926
ENSP00000233099	ENSP00000377148
ENSP00000233099	ENSP00000396704
ENSP00000233146	ENSP00000234420
ENSP00000233146	ENSP00000241436

ENSP00000233146	ENSP00000244745
ENSP00000233146	ENSP00000245479
ENSP00000233146	ENSP00000251038
ENSP00000233146	ENSP00000254719
ENSP00000233146	ENSP00000256996
ENSP00000233146	ENSP00000258428
ENSP00000233146	ENSP00000259008
ENSP00000233146	ENSP00000260947
ENSP00000233146	ENSP00000261424
ENSP00000233146	ENSP00000261531
ENSP00000233146	ENSP00000261637
ENSP00000233146	ENSP00000262315
ENSP00000233146	ENSP00000262367
ENSP00000233146	ENSP00000263795
ENSP00000233146	ENSP00000264233
ENSP00000233146	ENSP00000265007
ENSP00000233146	ENSP00000265081
ENSP00000233146	ENSP00000265304
ENSP00000233146	ENSP00000265433
ENSP00000233146	ENSP00000265849
ENSP00000233146	ENSP00000267064
ENSP00000233146	ENSP00000267430
ENSP00000233146	ENSP00000268124
ENSP00000233146	ENSP00000269397
ENSP00000233146	ENSP00000270708
ENSP00000233146	ENSP00000278616
ENSP00000233146	ENSP00000284719
ENSP00000233146	ENSP00000285021
ENSP00000233146	ENSP00000285398
ENSP00000233146	ENSP00000286428
ENSP00000233146	ENSP00000287647
ENSP00000233146	ENSP00000288985
ENSP00000233146	ENSP00000294008
ENSP00000233146	ENSP00000297316
ENSP00000233146	ENSP00000299767
ENSP00000233146	ENSP00000301764
ENSP00000233146	ENSP00000302111
ENSP00000233146	ENSP00000303515
ENSP00000233146	ENSP00000305790
ENSP00000233146	ENSP00000306561
ENSP00000233146	ENSP00000307853
ENSP00000233146	ENSP00000310520
ENSP00000233146	ENSP00000311873
ENSP00000233146	ENSP00000317904
ENSP00000233146	ENSP00000318977
ENSP00000233146	ENSP00000321636

ENSP00000233146	ENSP00000322570
ENSP00000233146	ENSP00000323099
ENSP00000233146	ENSP00000323858
ENSP00000233146	ENSP00000325863
ENSP00000233146	ENSP00000329466
ENSP00000233146	ENSP00000334061
ENSP00000233146	ENSP00000334675
ENSP00000233146	ENSP00000336856
ENSP00000233146	ENSP00000337127
ENSP00000233146	ENSP00000337353
ENSP00000233146	ENSP00000339495
ENSP00000233146	ENSP00000341243
ENSP00000233146	ENSP00000342889
ENSP00000233146	ENSP00000343392
ENSP00000233146	ENSP00000343741
ENSP00000233146	ENSP00000343745
ENSP00000233146	ENSP00000344741
ENSP00000233146	ENSP00000347232
ENSP00000233146	ENSP00000347978
ENSP00000233146	ENSP00000348089
ENSP00000233146	ENSP00000350283
ENSP00000233146	ENSP00000350491
ENSP00000233146	ENSP00000350767
ENSP00000233146	ENSP00000351284
ENSP00000233146	ENSP00000351490
ENSP00000233146	ENSP00000351697
ENSP00000233146	ENSP00000351947
ENSP00000233146	ENSP00000352257
ENSP00000233146	ENSP00000353332
ENSP00000233146	ENSP00000355013
ENSP00000233146	ENSP00000355185
ENSP00000233146	ENSP00000355518
ENSP00000233146	ENSP00000355958
ENSP00000233146	ENSP00000357440
ENSP00000233146	ENSP00000358576
ENSP00000233146	ENSP00000359444
ENSP00000233146	ENSP00000359567
ENSP00000233146	ENSP00000360268
ENSP00000233146	ENSP00000360310
ENSP00000233146	ENSP00000360532
ENSP00000233146	ENSP00000361310
ENSP00000233146	ENSP00000363021
ENSP00000233146	ENSP00000364270
ENSP00000233146	ENSP00000364289
ENSP00000233146	ENSP00000367527
ENSP00000233146	ENSP00000368349

ENSP00000233146	ENSP00000368438
ENSP00000233146	ENSP00000368646
ENSP00000233146	ENSP00000369335
ENSP00000233146	ENSP00000369411
ENSP00000233146	ENSP00000372023
ENSP00000233146	ENSP00000373952
ENSP00000233146	ENSP00000375809
ENSP00000233146	ENSP00000380504
ENSP00000233146	ENSP00000383986
ENSP00000233146	ENSP00000406490
ENSP00000233146	ENSP00000418734
ENSP00000233146	ENSP00000420588
ENSP00000233154	ENSP00000233668
ENSP00000233154	ENSP00000250113
ENSP00000233154	ENSP00000258682
ENSP00000233154	ENSP00000261017
ENSP00000233154	ENSP00000261167
ENSP00000233154	ENSP00000262320
ENSP00000233154	ENSP00000264122
ENSP00000233154	ENSP00000267101
ENSP00000233154	ENSP00000271628
ENSP00000233154	ENSP00000274376
ENSP00000233154	ENSP00000294129
ENSP00000233154	ENSP00000302079
ENSP00000233154	ENSP00000310561
ENSP00000233154	ENSP00000313829
ENSP00000233154	ENSP00000315465
ENSP00000233154	ENSP00000316460
ENSP00000233154	ENSP00000317332
ENSP00000233154	ENSP00000320924
ENSP00000233154	ENSP00000337838
ENSP00000233154	ENSP00000339299
ENSP00000233154	ENSP00000341189
ENSP00000233154	ENSP00000352802
ENSP00000233154	ENSP00000354313
ENSP00000233154	ENSP00000354566
ENSP00000233154	ENSP00000356694
ENSP00000233154	ENSP00000359552
ENSP00000233154	ENSP00000365439
ENSP00000233154	ENSP00000365637
ENSP00000233154	ENSP00000365891
ENSP00000233154	ENSP00000368190
ENSP00000233154	ENSP00000384675
ENSP00000233154	ENSP00000391774
ENSP00000233154	ENSP00000394794
ENSP00000233154	ENSP00000399511

ENSP00000233154	ENSP00000401397
ENSP00000233154	ENSP00000406091
ENSP00000233156	ENSP00000361405
ENSP00000233156	ENSP00000364709
ENSP00000233190	ENSP00000233627
ENSP00000233190	ENSP00000237289
ENSP00000233190	ENSP00000251864
ENSP00000233190	ENSP00000252102
ENSP00000233190	ENSP00000263774
ENSP00000233190	ENSP00000264230
ENSP00000233190	ENSP00000268668
ENSP00000233190	ENSP00000292180
ENSP00000233190	ENSP00000301585
ENSP00000233190	ENSP00000313490
ENSP00000233190	ENSP00000315774
ENSP00000233190	ENSP00000322450
ENSP00000233190	ENSP00000322706
ENSP00000233190	ENSP00000327070
ENSP00000233190	ENSP00000327268
ENSP00000233190	ENSP00000329466
ENSP00000233190	ENSP00000356972
ENSP00000233190	ENSP00000371888
ENSP00000233190	ENSP00000378605
ENSP00000233190	ENSP00000386722
ENSP00000233190	ENSP00000394085
ENSP00000233242	ENSP00000243253
ENSP00000233242	ENSP00000263816
ENSP00000233242	ENSP00000265517
ENSP00000233242	ENSP00000282541
ENSP00000233242	ENSP00000286091
ENSP00000233242	ENSP00000296350
ENSP00000233242	ENSP00000297261
ENSP00000233242	ENSP00000299767
ENSP00000233242	ENSP00000314615
ENSP00000233242	ENSP00000332353
ENSP00000233242	ENSP00000336927
ENSP00000233242	ENSP00000363873
ENSP00000233242	ENSP00000367992
ENSP00000233242	ENSP00000368646
ENSP00000233331	ENSP00000233809
ENSP00000233331	ENSP00000243903
ENSP00000233331	ENSP00000262238
ENSP00000233331	ENSP00000297157
ENSP00000233331	ENSP00000336842
ENSP00000233331	ENSP00000349640
ENSP00000233331	ENSP00000355205

ENSP00000233331	ENSP00000366434
ENSP00000233331	ENSP00000384198
ENSP00000233331	ENSP00000391457
ENSP00000233331	ENSP00000400476
ENSP00000233336	ENSP00000261424
ENSP00000233336	ENSP00000280665
ENSP00000233336	ENSP00000298139
ENSP00000233336	ENSP00000313171
ENSP00000233336	ENSP00000320503
ENSP00000233336	ENSP00000347232
ENSP00000233336	ENSP00000352265
ENSP00000233557	ENSP00000262306
ENSP00000233557	ENSP00000284811
ENSP00000233557	ENSP00000300181
ENSP00000233557	ENSP00000348157
ENSP00000233557	ENSP00000397435
ENSP00000233575	ENSP00000244007
ENSP00000233575	ENSP00000263686
ENSP00000233575	ENSP00000263816
ENSP00000233575	ENSP00000300574
ENSP00000233616	ENSP00000239891
ENSP00000233616	ENSP00000250498
ENSP00000233616	ENSP00000254101
ENSP00000233616	ENSP00000259253
ENSP00000233616	ENSP00000261534
ENSP00000233616	ENSP00000262215
ENSP00000233616	ENSP00000264501
ENSP00000233616	ENSP00000264639
ENSP00000233616	ENSP00000270538
ENSP00000233616	ENSP00000278243
ENSP00000233616	ENSP00000286091
ENSP00000233616	ENSP00000299626
ENSP00000233616	ENSP00000300658
ENSP00000233616	ENSP00000310621
ENSP00000233616	ENSP00000313420
ENSP00000233616	ENSP00000319979
ENSP00000233616	ENSP00000326699
ENSP00000233616	ENSP00000331268
ENSP00000233616	ENSP00000337688
ENSP00000233616	ENSP00000340466
ENSP00000233616	ENSP00000345656
ENSP00000233616	ENSP00000346809
ENSP00000233616	ENSP00000349877
ENSP00000233616	ENSP00000352708
ENSP00000233616	ENSP00000354558
ENSP00000233616	ENSP00000356530

ENSP00000233616	ENSP00000357998
ENSP00000233616	ENSP00000371888
ENSP00000233616	ENSP00000379042
ENSP00000233616	ENSP00000401445
ENSP00000233616	ENSP00000402515
ENSP00000233616	ENSP00000415203
ENSP00000233623	ENSP00000249356
ENSP00000233623	ENSP00000260970
ENSP00000233623	ENSP00000262177
ENSP00000233623	ENSP00000285398
ENSP00000233623	ENSP00000294053
ENSP00000233623	ENSP00000344431
ENSP00000233623	ENSP00000358019
ENSP00000233623	ENSP00000371451
ENSP00000233623	ENSP00000404381
ENSP00000233627	ENSP00000252102
ENSP00000233627	ENSP00000260361
ENSP00000233627	ENSP00000263774
ENSP00000233627	ENSP00000266544
ENSP00000233627	ENSP00000283429
ENSP00000233627	ENSP00000286428
ENSP00000233627	ENSP00000315774
ENSP00000233627	ENSP00000322450
ENSP00000233627	ENSP00000327268
ENSP00000233627	ENSP00000330737
ENSP00000233627	ENSP00000345917
ENSP00000233627	ENSP00000354687
ENSP00000233627	ENSP00000356972
ENSP00000233627	ENSP00000363676
ENSP00000233627	ENSP00000392985
ENSP00000233668	ENSP00000244007
ENSP00000233668	ENSP00000274376
ENSP00000233668	ENSP00000327513
ENSP00000233668	ENSP00000340944
ENSP00000233668	ENSP00000347942
ENSP00000233668	ENSP00000348307
ENSP00000233668	ENSP00000358417
ENSP00000233668	ENSP00000381793
ENSP00000233668	ENSP00000401277
ENSP00000233809	ENSP00000302665
ENSP00000233809	ENSP00000312584
ENSP00000233809	ENSP00000355751
ENSP00000233838	ENSP00000357255
ENSP00000233838	ENSP00000364709
ENSP00000233893	ENSP00000269980
ENSP00000233893	ENSP00000272227

ENSP00000233893	ENSP00000299767
ENSP00000233893	ENSP00000304802
ENSP00000233893	ENSP00000305682
ENSP00000233893	ENSP00000305958
ENSP00000233893	ENSP00000318351
ENSP00000233893	ENSP00000323822
ENSP00000233893	ENSP00000340019
ENSP00000233893	ENSP00000340278
ENSP00000233893	ENSP00000342087
ENSP00000233893	ENSP00000351644
ENSP00000233893	ENSP00000356130
ENSP00000233893	ENSP00000367208
ENSP00000233893	ENSP00000368438
ENSP00000233893	ENSP00000379607
ENSP00000233893	ENSP00000387262
ENSP00000233893	ENSP00000394382
ENSP00000233954	ENSP00000370842
ENSP00000233954	ENSP00000376445
ENSP00000234040	ENSP00000236959
ENSP00000234040	ENSP00000246548
ENSP00000234040	ENSP00000248553
ENSP00000234040	ENSP00000249636
ENSP00000234040	ENSP00000254101
ENSP00000234040	ENSP00000254900
ENSP00000234040	ENSP00000261434
ENSP00000234040	ENSP00000262193
ENSP00000234040	ENSP00000262608
ENSP00000234040	ENSP00000264160
ENSP00000234040	ENSP00000264193
ENSP00000234040	ENSP00000264335
ENSP00000234040	ENSP00000264705
ENSP00000234040	ENSP00000294168
ENSP00000234040	ENSP00000306361
ENSP00000234040	ENSP00000307272
ENSP00000234040	ENSP00000317904
ENSP00000234040	ENSP00000328103
ENSP00000234040	ENSP00000331342
ENSP00000234040	ENSP00000338703
ENSP00000234040	ENSP00000340820
ENSP00000234040	ENSP00000344537
ENSP00000234040	ENSP00000346022
ENSP00000234040	ENSP00000347134
ENSP00000234040	ENSP00000348278
ENSP00000234040	ENSP00000348455
ENSP00000234040	ENSP00000349887
ENSP00000234040	ENSP00000354558

ENSP00000234040	ENSP00000357283
ENSP00000234040	ENSP00000361287
ENSP00000234040	ENSP00000361998
ENSP00000234040	ENSP00000366482
ENSP00000234040	ENSP00000369293
ENSP00000234040	ENSP00000369757
ENSP00000234040	ENSP00000369897
ENSP00000234040	ENSP00000370421
ENSP00000234040	ENSP00000370719
ENSP00000234040	ENSP00000379678
ENSP00000234111	ENSP00000244763
ENSP00000234111	ENSP00000262265
ENSP00000234111	ENSP00000280350
ENSP00000234111	ENSP00000298292
ENSP00000234111	ENSP00000319788
ENSP00000234111	ENSP00000325448
ENSP00000234111	ENSP00000341243
ENSP00000234111	ENSP00000357880
ENSP00000234111	ENSP00000366156
ENSP00000234111	ENSP00000377520
ENSP00000234111	ENSP00000381499
ENSP00000234111	ENSP00000385746
ENSP00000234111	ENSP00000393583
ENSP00000234111	ENSP00000401445
ENSP00000234111	ENSP00000419371
ENSP00000234160	ENSP00000251968
ENSP00000234160	ENSP00000260649
ENSP00000234160	ENSP00000262544
ENSP00000234160	ENSP00000264122
ENSP00000234160	ENSP00000265175
ENSP00000234160	ENSP00000284637
ENSP00000234160	ENSP00000290541
ENSP00000234160	ENSP00000300181
ENSP00000234160	ENSP00000305503
ENSP00000234160	ENSP00000310003
ENSP00000234160	ENSP00000327541
ENSP00000234160	ENSP00000329419
ENSP00000234160	ENSP00000330945
ENSP00000234160	ENSP00000336606
ENSP00000234160	ENSP00000341848
ENSP00000234160	ENSP00000344460
ENSP00000234160	ENSP00000357861
ENSP00000234160	ENSP00000362153
ENSP00000234160	ENSP00000379500
ENSP00000234160	ENSP00000380793
ENSP00000234160	ENSP00000387477

ENSP00000234160	ENSP00000410833
ENSP00000234160	ENSP00000416097
ENSP00000234160	ENSP00000420405
ENSP00000234170	ENSP00000236051
ENSP00000234170	ENSP00000238789
ENSP00000234170	ENSP00000240055
ENSP00000234170	ENSP00000245121
ENSP00000234170	ENSP00000248633
ENSP00000234170	ENSP00000252115
ENSP00000234170	ENSP00000253107
ENSP00000234170	ENSP00000253329
ENSP00000234170	ENSP00000253363
ENSP00000234170	ENSP00000254037
ENSP00000234170	ENSP00000254940
ENSP00000234170	ENSP00000261015
ENSP00000234170	ENSP00000261637
ENSP00000234170	ENSP00000261741
ENSP00000234170	ENSP00000262056
ENSP00000234170	ENSP00000263278
ENSP00000234170	ENSP00000263629
ENSP00000234170	ENSP00000263795
ENSP00000234170	ENSP00000264065
ENSP00000234170	ENSP00000264230
ENSP00000234170	ENSP00000264670
ENSP00000234170	ENSP00000264951
ENSP00000234170	ENSP00000264995
ENSP00000234170	ENSP00000265245
ENSP00000234170	ENSP00000266529
ENSP00000234170	ENSP00000267425
ENSP00000234170	ENSP00000268616
ENSP00000234170	ENSP00000268661
ENSP00000234170	ENSP00000269499
ENSP00000234170	ENSP00000274242
ENSP00000234170	ENSP00000274606
ENSP00000234170	ENSP00000278856
ENSP00000234170	ENSP00000284000
ENSP00000234170	ENSP00000289371
ENSP00000234170	ENSP00000298292
ENSP00000234170	ENSP00000298746
ENSP00000234170	ENSP00000302886
ENSP00000234170	ENSP00000303511
ENSP00000234170	ENSP00000305702
ENSP00000234170	ENSP00000306548
ENSP00000234170	ENSP00000309334
ENSP00000234170	ENSP00000310042
ENSP00000234170	ENSP00000314311

ENSP00000234170	ENSP00000314348
ENSP00000234170	ENSP00000317992
ENSP00000234170	ENSP00000318195
ENSP00000234170	ENSP00000318604
ENSP00000234170	ENSP00000320885
ENSP00000234170	ENSP00000322419
ENSP00000234170	ENSP00000323424
ENSP00000234170	ENSP00000323858
ENSP00000234170	ENSP00000324122
ENSP00000234170	ENSP00000326806
ENSP00000234170	ENSP00000328690
ENSP00000234170	ENSP00000328854
ENSP00000234170	ENSP00000329466
ENSP00000234170	ENSP00000331817
ENSP00000234170	ENSP00000332171
ENSP00000234170	ENSP00000332340
ENSP00000234170	ENSP00000333836
ENSP00000234170	ENSP00000337839
ENSP00000234170	ENSP00000338573
ENSP00000234170	ENSP00000338862
ENSP00000234170	ENSP00000339145
ENSP00000234170	ENSP00000339795
ENSP00000234170	ENSP00000340434
ENSP00000234170	ENSP00000346037
ENSP00000234170	ENSP00000346120
ENSP00000234170	ENSP00000346236
ENSP00000234170	ENSP00000346725
ENSP00000234170	ENSP00000351596
ENSP00000234170	ENSP00000352678
ENSP00000234170	ENSP00000352738
ENSP00000234170	ENSP00000353098
ENSP00000234170	ENSP00000354040
ENSP00000234170	ENSP00000355541
ENSP00000234170	ENSP00000358563
ENSP00000234170	ENSP00000358716
ENSP00000234170	ENSP00000358812
ENSP00000234170	ENSP00000359345
ENSP00000234170	ENSP00000359688
ENSP00000234170	ENSP00000359939
ENSP00000234170	ENSP00000360031
ENSP00000234170	ENSP00000360412
ENSP00000234170	ENSP00000360828
ENSP00000234170	ENSP00000361010
ENSP00000234170	ENSP00000361076
ENSP00000234170	ENSP00000361232
ENSP00000234170	ENSP00000361982

ENSP00000234170	ENSP00000362153
ENSP00000234170	ENSP00000363559
ENSP00000234170	ENSP00000364543
ENSP00000234170	ENSP00000365806
ENSP00000234170	ENSP00000366179
ENSP00000234170	ENSP00000366519
ENSP00000234170	ENSP00000366557
ENSP00000234170	ENSP00000367439
ENSP00000234170	ENSP00000370589
ENSP00000234170	ENSP00000371201
ENSP00000234170	ENSP00000371451
ENSP00000234170	ENSP00000372199
ENSP00000234170	ENSP00000372827
ENSP00000234170	ENSP00000373090
ENSP00000234170	ENSP00000373715
ENSP00000234170	ENSP00000374390
ENSP00000234170	ENSP00000375730
ENSP00000234170	ENSP00000376307
ENSP00000234170	ENSP00000376309
ENSP00000234170	ENSP00000377344
ENSP00000234170	ENSP00000380495
ENSP00000234170	ENSP00000380982
ENSP00000234170	ENSP00000381045
ENSP00000234170	ENSP00000381293
ENSP00000234170	ENSP00000382392
ENSP00000234170	ENSP00000387347
ENSP00000234170	ENSP00000388464
ENSP00000234170	ENSP00000394290
ENSP00000234170	ENSP00000394400
ENSP00000234170	ENSP00000398597
ENSP00000234170	ENSP00000399530
ENSP00000234170	ENSP00000400626
ENSP00000234170	ENSP00000401371
ENSP00000234170	ENSP00000402338
ENSP00000234170	ENSP00000403576
ENSP00000234170	ENSP00000409074
ENSP00000234170	ENSP00000409367
ENSP00000234170	ENSP00000411162
ENSP00000234170	ENSP00000412310
ENSP00000234170	ENSP00000416959
ENSP00000234170	ENSP00000419740
ENSP00000234198	ENSP00000269385
ENSP00000234198	ENSP00000337163
ENSP00000234198	ENSP00000352257
ENSP00000234198	ENSP00000354478
ENSP00000234198	ENSP00000364847

ENSP00000234198	ENSP00000372170
ENSP00000234198	ENSP00000399968
ENSP00000234198	ENSP00000408792
ENSP00000234296	ENSP00000234626
ENSP00000234296	ENSP00000240055
ENSP00000234296	ENSP00000245960
ENSP00000234296	ENSP00000246548
ENSP00000234296	ENSP00000251038
ENSP00000234296	ENSP00000251289
ENSP00000234296	ENSP00000254719
ENSP00000234296	ENSP00000254942
ENSP00000234296	ENSP00000255465
ENSP00000234296	ENSP00000256442
ENSP00000234296	ENSP00000257789
ENSP00000234296	ENSP00000259008
ENSP00000234296	ENSP00000261424
ENSP00000234296	ENSP00000261531
ENSP00000234296	ENSP00000261881
ENSP00000234296	ENSP00000262144
ENSP00000234296	ENSP00000262315
ENSP00000234296	ENSP00000263201
ENSP00000234296	ENSP00000264108
ENSP00000234296	ENSP00000264169
ENSP00000234296	ENSP00000264335
ENSP00000234296	ENSP00000264926
ENSP00000234296	ENSP00000265056
ENSP00000234296	ENSP00000265465
ENSP00000234296	ENSP00000265728
ENSP00000234296	ENSP00000266070
ENSP00000234296	ENSP00000269127
ENSP00000234296	ENSP00000269468
ENSP00000234296	ENSP00000270861
ENSP00000234296	ENSP00000274289
ENSP00000234296	ENSP00000276603
ENSP00000234296	ENSP00000282058
ENSP00000234296	ENSP00000288976
ENSP00000234296	ENSP00000290810
ENSP00000234296	ENSP00000295640
ENSP00000234296	ENSP00000296273
ENSP00000234296	ENSP00000296509
ENSP00000234296	ENSP00000296543
ENSP00000234296	ENSP00000296755
ENSP00000234296	ENSP00000297431
ENSP00000234296	ENSP00000298139
ENSP00000234296	ENSP00000300086
ENSP00000234296	ENSP00000300231

ENSP00000234296	ENSP00000300738
ENSP00000234296	ENSP00000301019
ENSP00000234296	ENSP00000307208
ENSP00000234296	ENSP00000308944
ENSP00000234296	ENSP00000313171
ENSP00000234296	ENSP00000313420
ENSP00000234296	ENSP00000317636
ENSP00000234296	ENSP00000322396
ENSP00000234296	ENSP00000327541
ENSP00000234296	ENSP00000328720
ENSP00000234296	ENSP00000329466
ENSP00000234296	ENSP00000335357
ENSP00000234296	ENSP00000336606
ENSP00000234296	ENSP00000336856
ENSP00000234296	ENSP00000336868
ENSP00000234296	ENSP00000338413
ENSP00000234296	ENSP00000340879
ENSP00000234296	ENSP00000343741
ENSP00000234296	ENSP00000346725
ENSP00000234296	ENSP00000347168
ENSP00000234296	ENSP00000347232
ENSP00000234296	ENSP00000348565
ENSP00000234296	ENSP00000350283
ENSP00000234296	ENSP00000350767
ENSP00000234296	ENSP00000351947
ENSP00000234296	ENSP00000352257
ENSP00000234296	ENSP00000353332
ENSP00000234296	ENSP00000353793
ENSP00000234296	ENSP00000354340
ENSP00000234296	ENSP00000354916
ENSP00000234296	ENSP00000355011
ENSP00000234296	ENSP00000355153
ENSP00000234296	ENSP00000360412
ENSP00000234296	ENSP00000360532
ENSP00000234296	ENSP00000360621
ENSP00000234296	ENSP00000362578
ENSP00000234296	ENSP00000362592
ENSP00000234296	ENSP00000363021
ENSP00000234296	ENSP00000366557
ENSP00000234296	ENSP00000366953
ENSP00000234296	ENSP00000366974
ENSP00000234296	ENSP00000367034
ENSP00000234296	ENSP00000367406
ENSP00000234296	ENSP00000369335
ENSP00000234296	ENSP00000369411
ENSP00000234296	ENSP00000369716

ENSP00000234296	ENSP00000370151
ENSP00000234296	ENSP00000371475
ENSP00000234296	ENSP00000375809
ENSP00000234296	ENSP00000377747
ENSP00000234296	ENSP00000382562
ENSP00000234296	ENSP00000387982
ENSP00000234296	ENSP00000390475
ENSP00000234296	ENSP00000392028
ENSP00000234296	ENSP00000402515
ENSP00000234396	ENSP00000236067
ENSP00000234396	ENSP00000249289
ENSP00000234396	ENSP00000251076
ENSP00000234396	ENSP00000252699
ENSP00000234396	ENSP00000253083
ENSP00000234396	ENSP00000253413
ENSP00000234396	ENSP00000261714
ENSP00000234396	ENSP00000262039
ENSP00000234396	ENSP00000262554
ENSP00000234396	ENSP00000262850
ENSP00000234396	ENSP00000263208
ENSP00000234396	ENSP00000264029
ENSP00000234396	ENSP00000269385
ENSP00000234396	ENSP00000269397
ENSP00000234396	ENSP00000272238
ENSP00000234396	ENSP00000277010
ENSP00000234396	ENSP00000282841
ENSP00000234396	ENSP00000284727
ENSP00000234396	ENSP00000290949
ENSP00000234396	ENSP00000295685
ENSP00000234396	ENSP00000303191
ENSP00000234396	ENSP00000307214
ENSP00000234396	ENSP00000308750
ENSP00000234396	ENSP00000322706
ENSP00000234396	ENSP00000329715
ENSP00000234396	ENSP00000330836
ENSP00000234396	ENSP00000333946
ENSP00000234396	ENSP00000336724
ENSP00000234396	ENSP00000337127
ENSP00000234396	ENSP00000344460
ENSP00000234396	ENSP00000349078
ENSP00000234396	ENSP00000352522
ENSP00000234396	ENSP00000356470
ENSP00000234396	ENSP00000358033
ENSP00000234396	ENSP00000360183
ENSP00000234396	ENSP00000363162
ENSP00000234396	ENSP00000365388

ENSP00000234396	ENSP00000369456
ENSP00000234396	ENSP00000371888
ENSP00000234396	ENSP00000379908
ENSP00000234396	ENSP00000384490
ENSP00000234420	ENSP00000242576
ENSP00000234420	ENSP00000251038
ENSP00000234420	ENSP00000254719
ENSP00000234420	ENSP00000256996
ENSP00000234420	ENSP00000258428
ENSP00000234420	ENSP00000259008
ENSP00000234420	ENSP00000260810
ENSP00000234420	ENSP00000260947
ENSP00000234420	ENSP00000261424
ENSP00000234420	ENSP00000261531
ENSP00000234420	ENSP00000262367
ENSP00000234420	ENSP00000263642
ENSP00000234420	ENSP00000263795
ENSP00000234420	ENSP00000264233
ENSP00000234420	ENSP00000265081
ENSP00000234420	ENSP00000265304
ENSP00000234420	ENSP00000265433
ENSP00000234420	ENSP00000265849
ENSP00000234420	ENSP00000267064
ENSP00000234420	ENSP00000267430
ENSP00000234420	ENSP00000268043
ENSP00000234420	ENSP00000268711
ENSP00000234420	ENSP00000276326
ENSP00000234420	ENSP00000278616
ENSP00000234420	ENSP00000284719
ENSP00000234420	ENSP00000285021
ENSP00000234420	ENSP00000285398
ENSP00000234420	ENSP00000301280
ENSP00000234420	ENSP00000306561
ENSP00000234420	ENSP00000307853
ENSP00000234420	ENSP00000308944
ENSP00000234420	ENSP00000310520
ENSP00000234420	ENSP00000311873
ENSP00000234420	ENSP00000312995
ENSP00000234420	ENSP00000313420
ENSP00000234420	ENSP00000321636
ENSP00000234420	ENSP00000322570
ENSP00000234420	ENSP00000325863
ENSP00000234420	ENSP00000329466
ENSP00000234420	ENSP00000331310
ENSP00000234420	ENSP00000337127
ENSP00000234420	ENSP00000341243

ENSP00000234420	ENSP00000343741
ENSP00000234420	ENSP00000343745
ENSP00000234420	ENSP00000345917
ENSP00000234420	ENSP00000346566
ENSP00000234420	ENSP00000347232
ENSP00000234420	ENSP00000348020
ENSP00000234420	ENSP00000349049
ENSP00000234420	ENSP00000350283
ENSP00000234420	ENSP00000351284
ENSP00000234420	ENSP00000351697
ENSP00000234420	ENSP00000351947
ENSP00000234420	ENSP00000352257
ENSP00000234420	ENSP00000355185
ENSP00000234420	ENSP00000355958
ENSP00000234420	ENSP00000358478
ENSP00000234420	ENSP00000358576
ENSP00000234420	ENSP00000360268
ENSP00000234420	ENSP00000360310
ENSP00000234420	ENSP00000360532
ENSP00000234420	ENSP00000361310
ENSP00000234420	ENSP00000363021
ENSP00000234420	ENSP00000363313
ENSP00000234420	ENSP00000364289
ENSP00000234420	ENSP00000367527
ENSP00000234420	ENSP00000368349
ENSP00000234420	ENSP00000368438
ENSP00000234420	ENSP00000368646
ENSP00000234420	ENSP00000369335
ENSP00000234420	ENSP00000372023
ENSP00000234420	ENSP00000375809
ENSP00000234420	ENSP00000379607
ENSP00000234420	ENSP00000380504
ENSP00000234420	ENSP00000392601
ENSP00000234420	ENSP00000406490
ENSP00000234420	ENSP00000418734
ENSP00000234454	ENSP00000264108
ENSP00000234454	ENSP00000311977
ENSP00000234454	ENSP00000359939
ENSP00000234488	ENSP00000349887
ENSP00000234590	ENSP00000240922
ENSP00000234590	ENSP00000251607
ENSP00000234590	ENSP00000251968
ENSP00000234590	ENSP00000259229
ENSP00000234590	ENSP00000262030
ENSP00000234590	ENSP00000262850
ENSP00000234590	ENSP00000264335

ENSP00000234590	ENSP00000268206
ENSP00000234590	ENSP00000270776
ENSP00000234590	ENSP00000272227
ENSP00000234590	ENSP00000276326
ENSP00000234590	ENSP00000296805
ENSP00000234590	ENSP00000297185
ENSP00000234590	ENSP00000301729
ENSP00000234590	ENSP00000305958
ENSP00000234590	ENSP00000307900
ENSP00000234590	ENSP00000318131
ENSP00000234590	ENSP00000325448
ENSP00000234590	ENSP00000327070
ENSP00000234590	ENSP00000336702
ENSP00000234590	ENSP00000337127
ENSP00000234590	ENSP00000338703
ENSP00000234590	ENSP00000343557
ENSP00000234590	ENSP00000343885
ENSP00000234590	ENSP00000344460
ENSP00000234590	ENSP00000349577
ENSP00000234590	ENSP00000351284
ENSP00000234590	ENSP00000352708
ENSP00000234590	ENSP00000356290
ENSP00000234590	ENSP00000361686
ENSP00000234590	ENSP00000362334
ENSP00000234590	ENSP00000362413
ENSP00000234590	ENSP00000362824
ENSP00000234590	ENSP00000368438
ENSP00000234590	ENSP00000368699
ENSP00000234590	ENSP00000369257
ENSP00000234590	ENSP00000370376
ENSP00000234590	ENSP00000373952
ENSP00000234590	ENSP00000379607
ENSP00000234590	ENSP00000379933
ENSP00000234590	ENSP00000384523
ENSP00000234590	ENSP00000393953
ENSP00000234590	ENSP00000394071
ENSP00000234590	ENSP00000417763
ENSP00000234626	ENSP00000244741
ENSP00000234626	ENSP00000245960
ENSP00000234626	ENSP00000246062
ENSP00000234626	ENSP00000253003
ENSP00000234626	ENSP00000253462
ENSP00000234626	ENSP00000256442
ENSP00000234626	ENSP00000257789
ENSP00000234626	ENSP00000258418
ENSP00000234626	ENSP00000259750

ENSP00000234626	ENSP00000260810
ENSP00000234626	ENSP00000261845
ENSP00000234626	ENSP00000261881
ENSP00000234626	ENSP00000262315
ENSP00000234626	ENSP00000262319
ENSP00000234626	ENSP00000262519
ENSP00000234626	ENSP00000263201
ENSP00000234626	ENSP00000264926
ENSP00000234626	ENSP00000265056
ENSP00000234626	ENSP00000265140
ENSP00000234626	ENSP00000265728
ENSP00000234626	ENSP00000267197
ENSP00000234626	ENSP00000269214
ENSP00000234626	ENSP00000270861
ENSP00000234626	ENSP00000274289
ENSP00000234626	ENSP00000276014
ENSP00000234626	ENSP00000296509
ENSP00000234626	ENSP00000297338
ENSP00000234626	ENSP00000297431
ENSP00000234626	ENSP00000298139
ENSP00000234626	ENSP00000298852
ENSP00000234626	ENSP00000301280
ENSP00000234626	ENSP00000302478
ENSP00000234626	ENSP00000306999
ENSP00000234626	ENSP00000308699
ENSP00000234626	ENSP00000308944
ENSP00000234626	ENSP00000309474
ENSP00000234626	ENSP00000310129
ENSP00000234626	ENSP00000311005
ENSP00000234626	ENSP00000312995
ENSP00000234626	ENSP00000313432
ENSP00000234626	ENSP00000317334
ENSP00000234626	ENSP00000317636
ENSP00000234626	ENSP00000321636
ENSP00000234626	ENSP00000322180
ENSP00000234626	ENSP00000323663
ENSP00000234626	ENSP00000328854
ENSP00000234626	ENSP00000329419
ENSP00000234626	ENSP00000331268
ENSP00000234626	ENSP00000336868
ENSP00000234626	ENSP00000340879
ENSP00000234626	ENSP00000342381
ENSP00000234626	ENSP00000343190
ENSP00000234626	ENSP00000343741
ENSP00000234626	ENSP00000347168
ENSP00000234626	ENSP00000347232

ENSP00000234626	ENSP00000350767
ENSP00000234626	ENSP00000351284
ENSP00000234626	ENSP00000351697
ENSP00000234626	ENSP00000353793
ENSP00000234626	ENSP00000354347
ENSP00000234626	ENSP00000355153
ENSP00000234626	ENSP00000355388
ENSP00000234626	ENSP00000358478
ENSP00000234626	ENSP00000360621
ENSP00000234626	ENSP00000361310
ENSP00000234626	ENSP00000366974
ENSP00000234626	ENSP00000367034
ENSP00000234626	ENSP00000368349
ENSP00000234626	ENSP00000368438
ENSP00000234626	ENSP00000369335
ENSP00000234626	ENSP00000370151
ENSP00000234626	ENSP00000371475
ENSP00000234626	ENSP00000372023
ENSP00000234626	ENSP00000376946
ENSP00000234626	ENSP00000381565
ENSP00000234626	ENSP00000384048
ENSP00000234626	ENSP00000402515
ENSP00000234626	ENSP00000413632
ENSP00000234677	ENSP00000235521
ENSP00000234677	ENSP00000240185
ENSP00000234677	ENSP00000249923
ENSP00000234677	ENSP00000251038
ENSP00000234677	ENSP00000256854
ENSP00000234677	ENSP00000258499
ENSP00000234677	ENSP00000259154
ENSP00000234677	ENSP00000264161
ENSP00000234677	ENSP00000264705
ENSP00000234677	ENSP00000267415
ENSP00000234677	ENSP00000270223
ENSP00000234677	ENSP00000276603
ENSP00000234677	ENSP00000301585
ENSP00000234677	ENSP00000304592
ENSP00000234677	ENSP00000311766
ENSP00000234677	ENSP00000322016
ENSP00000234677	ENSP00000325448
ENSP00000234677	ENSP00000340019
ENSP00000234677	ENSP00000341243
ENSP00000234677	ENSP00000347495
ENSP00000234677	ENSP00000348565
ENSP00000234677	ENSP00000350249
ENSP00000234677	ENSP00000354347

ENSP00000234677	ENSP00000355890
ENSP00000234677	ENSP00000358563
ENSP00000234677	ENSP00000362807
ENSP00000234677	ENSP00000363205
ENSP00000234677	ENSP00000367923
ENSP00000234677	ENSP00000369335
ENSP00000234677	ENSP00000370883
ENSP00000234677	ENSP00000377914
ENSP00000234800	ENSP00000311134
ENSP00000234827	ENSP00000247970
ENSP00000234827	ENSP00000252445
ENSP00000234827	ENSP00000256854
ENSP00000234827	ENSP00000262518
ENSP00000234827	ENSP00000264515
ENSP00000234827	ENSP00000265073
ENSP00000234827	ENSP00000283882
ENSP00000234827	ENSP00000295066
ENSP00000234827	ENSP00000302160
ENSP00000234827	ENSP00000304593
ENSP00000234827	ENSP00000325677
ENSP00000234827	ENSP00000334188
ENSP00000234827	ENSP00000354977
ENSP00000234827	ENSP00000357887
ENSP00000234827	ENSP00000360116
ENSP00000234827	ENSP00000366135
ENSP00000234827	ENSP00000370968
ENSP00000234827	ENSP00000374213
ENSP00000234827	ENSP00000386134
ENSP00000234827	ENSP00000386759
ENSP00000235090	ENSP00000238112
ENSP00000235090	ENSP00000244520
ENSP00000235090	ENSP00000245934
ENSP00000235090	ENSP00000253048
ENSP00000235090	ENSP00000256429
ENSP00000235090	ENSP00000261531
ENSP00000235090	ENSP00000261600
ENSP00000235090	ENSP00000264515
ENSP00000235090	ENSP00000266735
ENSP00000235090	ENSP00000271628
ENSP00000235090	ENSP00000286307
ENSP00000235090	ENSP00000291386
ENSP00000235090	ENSP00000292476
ENSP00000235090	ENSP00000296490
ENSP00000235090	ENSP00000298281
ENSP00000235090	ENSP00000299543
ENSP00000235090	ENSP00000300413

ENSP00000235090	ENSP00000302160
ENSP00000235090	ENSP00000302530
ENSP00000235090	ENSP00000304327
ENSP00000235090	ENSP00000304704
ENSP00000235090	ENSP00000308466
ENSP00000235090	ENSP00000311677
ENSP00000235090	ENSP00000314348
ENSP00000235090	ENSP00000315791
ENSP00000235090	ENSP00000316578
ENSP00000235090	ENSP00000317872
ENSP00000235090	ENSP00000319169
ENSP00000235090	ENSP00000321320
ENSP00000235090	ENSP00000325376
ENSP00000235090	ENSP00000331369
ENSP00000235090	ENSP00000331817
ENSP00000235090	ENSP00000332444
ENSP00000235090	ENSP00000336752
ENSP00000235090	ENSP00000339145
ENSP00000235090	ENSP00000339353
ENSP00000235090	ENSP00000342374
ENSP00000235090	ENSP00000347592
ENSP00000235090	ENSP00000353246
ENSP00000235090	ENSP00000354951
ENSP00000235090	ENSP00000355050
ENSP00000235090	ENSP00000359727
ENSP00000235090	ENSP00000361162
ENSP00000235090	ENSP00000362306
ENSP00000235090	ENSP00000365735
ENSP00000235090	ENSP00000365747
ENSP00000235090	ENSP00000366607
ENSP00000235090	ENSP00000378165
ENSP00000235090	ENSP00000379607
ENSP00000235090	ENSP00000390475
ENSP00000235090	ENSP00000393275
ENSP00000235090	ENSP00000400591
ENSP00000235090	ENSP00000412566
ENSP00000235090	ENSP00000418734
ENSP00000235310	ENSP00000258428
ENSP00000235310	ENSP00000271526
ENSP00000235310	ENSP00000296509
ENSP00000235310	ENSP00000309968
ENSP00000235310	ENSP00000311368
ENSP00000235310	ENSP00000312753
ENSP00000235310	ENSP00000317872
ENSP00000235310	ENSP00000351697
ENSP00000235310	ENSP00000354730

ENSP00000235310	ENSP00000365175
ENSP00000235310	ENSP00000378529
ENSP00000235310	ENSP00000387477
ENSP00000235310	ENSP00000415363
ENSP00000235329	ENSP00000253083
ENSP00000235329	ENSP00000254442
ENSP00000235329	ENSP00000262715
ENSP00000235329	ENSP00000262854
ENSP00000235329	ENSP00000285873
ENSP00000235329	ENSP00000290299
ENSP00000235329	ENSP00000323377
ENSP00000235329	ENSP00000324404
ENSP00000235329	ENSP00000325421
ENSP00000235329	ENSP00000340093
ENSP00000235329	ENSP00000351813
ENSP00000235329	ENSP00000352820
ENSP00000235329	ENSP00000353059
ENSP00000235329	ENSP00000355865
ENSP00000235329	ENSP00000362344
ENSP00000235329	ENSP00000363591
ENSP00000235329	ENSP00000366482
ENSP00000235329	ENSP00000366953
ENSP00000235329	ENSP00000369335
ENSP00000235329	ENSP00000418667
ENSP00000235332	ENSP00000334061
ENSP00000235332	ENSP00000337396
ENSP00000235345	ENSP00000277541
ENSP00000235345	ENSP00000336724
ENSP00000235345	ENSP00000357494
ENSP00000235372	ENSP00000267163
ENSP00000235372	ENSP00000325120
ENSP00000235521	ENSP00000243563
ENSP00000235521	ENSP00000264705
ENSP00000235521	ENSP00000340019
ENSP00000235521	ENSP00000347495
ENSP00000235933	ENSP00000347948
ENSP00000236051	ENSP00000244496
ENSP00000236051	ENSP00000246802
ENSP00000236051	ENSP00000248673
ENSP00000236051	ENSP00000249636
ENSP00000236051	ENSP00000252115
ENSP00000236051	ENSP00000252622
ENSP00000236051	ENSP00000253107
ENSP00000236051	ENSP00000253108
ENSP00000236051	ENSP00000254037
ENSP00000236051	ENSP00000254940

ENSP00000236051	ENSP00000260102
ENSP00000236051	ENSP00000261015
ENSP00000236051	ENSP00000261531
ENSP00000236051	ENSP00000261741
ENSP00000236051	ENSP00000264230
ENSP00000236051	ENSP00000264670
ENSP00000236051	ENSP00000264995
ENSP00000236051	ENSP00000265245
ENSP00000236051	ENSP00000268616
ENSP00000236051	ENSP00000268661
ENSP00000236051	ENSP00000269499
ENSP00000236051	ENSP00000274606
ENSP00000236051	ENSP00000278856
ENSP00000236051	ENSP00000281708
ENSP00000236051	ENSP00000282388
ENSP00000236051	ENSP00000296411
ENSP00000236051	ENSP00000296755
ENSP00000236051	ENSP00000296802
ENSP00000236051	ENSP00000297325
ENSP00000236051	ENSP00000297990
ENSP00000236051	ENSP00000298746
ENSP00000236051	ENSP00000300231
ENSP00000236051	ENSP00000301962
ENSP00000236051	ENSP00000302886
ENSP00000236051	ENSP00000305702
ENSP00000236051	ENSP00000307889
ENSP00000236051	ENSP00000308944
ENSP00000236051	ENSP00000309334
ENSP00000236051	ENSP00000310590
ENSP00000236051	ENSP00000314348
ENSP00000236051	ENSP00000317992
ENSP00000236051	ENSP00000318195
ENSP00000236051	ENSP00000318374
ENSP00000236051	ENSP00000322016
ENSP00000236051	ENSP00000322396
ENSP00000236051	ENSP00000323858
ENSP00000236051	ENSP00000324792
ENSP00000236051	ENSP00000325312
ENSP00000236051	ENSP00000326806
ENSP00000236051	ENSP00000328690
ENSP00000236051	ENSP00000329466
ENSP00000236051	ENSP00000330343
ENSP00000236051	ENSP00000331817
ENSP00000236051	ENSP00000332340
ENSP00000236051	ENSP00000337839
ENSP00000236051	ENSP00000338573

ENSP00000236051	ENSP00000338862
ENSP00000236051	ENSP00000339145
ENSP00000236051	ENSP00000339795
ENSP00000236051	ENSP00000345917
ENSP00000236051	ENSP00000346015
ENSP00000236051	ENSP00000346120
ENSP00000236051	ENSP00000346725
ENSP00000236051	ENSP00000354040
ENSP00000236051	ENSP00000358478
ENSP00000236051	ENSP00000358563
ENSP00000236051	ENSP00000358812
ENSP00000236051	ENSP00000359345
ENSP00000236051	ENSP00000359688
ENSP00000236051	ENSP00000360031
ENSP00000236051	ENSP00000360412
ENSP00000236051	ENSP00000360828
ENSP00000236051	ENSP00000361076
ENSP00000236051	ENSP00000361092
ENSP00000236051	ENSP00000361232
ENSP00000236051	ENSP00000361982
ENSP00000236051	ENSP00000362153
ENSP00000236051	ENSP00000363391
ENSP00000236051	ENSP00000364320
ENSP00000236051	ENSP00000365806
ENSP00000236051	ENSP00000366519
ENSP00000236051	ENSP00000366557
ENSP00000236051	ENSP00000368887
ENSP00000236051	ENSP00000370589
ENSP00000236051	ENSP00000371201
ENSP00000236051	ENSP00000372199
ENSP00000236051	ENSP00000373090
ENSP00000236051	ENSP00000374390
ENSP00000236051	ENSP00000379607
ENSP00000236051	ENSP00000379888
ENSP00000236051	ENSP00000382392
ENSP00000236051	ENSP00000384015
ENSP00000236051	ENSP00000388464
ENSP00000236051	ENSP00000389103
ENSP00000236051	ENSP00000394290
ENSP00000236051	ENSP00000402338
ENSP00000236051	ENSP00000403576
ENSP00000236051	ENSP00000409074
ENSP00000236051	ENSP00000409367
ENSP00000236051	ENSP00000411162
ENSP00000236051	ENSP00000414321
ENSP00000236051	ENSP00000419740

ENSP00000236067	ENSP00000237530
ENSP00000236067	ENSP00000255108
ENSP00000236067	ENSP00000262030
ENSP00000236067	ENSP00000263083
ENSP00000236067	ENSP00000273398
ENSP00000236067	ENSP00000282050
ENSP00000236067	ENSP00000290299
ENSP00000236067	ENSP00000290541
ENSP00000236067	ENSP00000295702
ENSP00000236067	ENSP00000295890
ENSP00000236067	ENSP00000301587
ENSP00000236067	ENSP00000311028
ENSP00000236067	ENSP00000314348
ENSP00000236067	ENSP00000332247
ENSP00000236067	ENSP00000332681
ENSP00000236067	ENSP00000333873
ENSP00000236067	ENSP00000337053
ENSP00000236067	ENSP00000343885
ENSP00000236067	ENSP00000345096
ENSP00000236067	ENSP00000347134
ENSP00000236067	ENSP00000347979
ENSP00000236067	ENSP00000349142
ENSP00000236067	ENSP00000352522
ENSP00000236067	ENSP00000354632
ENSP00000236067	ENSP00000358737
ENSP00000236067	ENSP00000361057
ENSP00000236067	ENSP00000362334
ENSP00000236067	ENSP00000363162
ENSP00000236067	ENSP00000365943
ENSP00000236067	ENSP00000370719
ENSP00000236067	ENSP00000379093
ENSP00000236067	ENSP00000382767
ENSP00000236192	ENSP00000258301
ENSP00000236192	ENSP00000258654
ENSP00000236192	ENSP00000259400
ENSP00000236192	ENSP00000260818
ENSP00000236192	ENSP00000261173
ENSP00000236192	ENSP00000263897
ENSP00000236192	ENSP00000265857
ENSP00000236192	ENSP00000272322
ENSP00000236192	ENSP00000286448
ENSP00000236192	ENSP00000294179
ENSP00000236192	ENSP00000305255
ENSP00000236192	ENSP00000309457
ENSP00000236192	ENSP00000310521
ENSP00000236192	ENSP00000314615

ENSP00000236192	ENSP00000316454
ENSP00000236192	ENSP00000317714
ENSP00000236192	ENSP00000320885
ENSP00000236192	ENSP00000323377
ENSP00000236192	ENSP00000324628
ENSP00000236192	ENSP00000325002
ENSP00000236192	ENSP00000326534
ENSP00000236192	ENSP00000327650
ENSP00000236192	ENSP00000337501
ENSP00000236192	ENSP00000344055
ENSP00000236192	ENSP00000349887
ENSP00000236192	ENSP00000353073
ENSP00000236192	ENSP00000356405
ENSP00000236192	ENSP00000357887
ENSP00000236192	ENSP00000360183
ENSP00000236192	ENSP00000363349
ENSP00000236192	ENSP00000369810
ENSP00000236192	ENSP00000373215
ENSP00000236192	ENSP00000376268
ENSP00000236192	ENSP00000378368
ENSP00000236192	ENSP00000381293
ENSP00000236192	ENSP00000389934
ENSP00000236192	ENSP00000390783
ENSP00000236192	ENSP00000397441
ENSP00000236192	ENSP00000417748
ENSP00000236192	ENSP00000418661
ENSP00000236273	ENSP00000243563
ENSP00000236273	ENSP00000251038
ENSP00000236273	ENSP00000252622
ENSP00000236273	ENSP00000254193
ENSP00000236273	ENSP00000260210
ENSP00000236273	ENSP00000262056
ENSP00000236273	ENSP00000265753
ENSP00000236273	ENSP00000266529
ENSP00000236273	ENSP00000268482
ENSP00000236273	ENSP00000297151
ENSP00000236273	ENSP00000298532
ENSP00000236273	ENSP00000300213
ENSP00000236273	ENSP00000300648
ENSP00000236273	ENSP00000301740
ENSP00000236273	ENSP00000303191
ENSP00000236273	ENSP00000303712
ENSP00000236273	ENSP00000304370
ENSP00000236273	ENSP00000314311
ENSP00000236273	ENSP00000314792
ENSP00000236273	ENSP00000326806

ENSP00000236273	ENSP00000331907
ENSP00000236273	ENSP00000332103
ENSP00000236273	ENSP00000334148
ENSP00000236273	ENSP00000334188
ENSP00000236273	ENSP00000339090
ENSP00000236273	ENSP00000343557
ENSP00000236273	ENSP00000351137
ENSP00000236273	ENSP00000351492
ENSP00000236273	ENSP00000360532
ENSP00000236273	ENSP00000361577
ENSP00000236273	ENSP00000362936
ENSP00000236273	ENSP00000366557
ENSP00000236273	ENSP00000367835
ENSP00000236273	ENSP00000376309
ENSP00000236273	ENSP00000376973
ENSP00000236273	ENSP00000384573
ENSP00000236273	ENSP00000412566
ENSP00000236273	ENSP00000416959
ENSP00000236273	ENSP00000417451
ENSP00000236273	ENSP00000420195
ENSP00000236959	ENSP00000243563
ENSP00000236959	ENSP00000248566
ENSP00000236959	ENSP00000251303
ENSP00000236959	ENSP00000253462
ENSP00000236959	ENSP00000260361
ENSP00000236959	ENSP00000260598
ENSP00000236959	ENSP00000264220
ENSP00000236959	ENSP00000264335
ENSP00000236959	ENSP00000282441
ENSP00000236959	ENSP00000286621
ENSP00000236959	ENSP00000287878
ENSP00000236959	ENSP00000288532
ENSP00000236959	ENSP00000296658
ENSP00000236959	ENSP00000300738
ENSP00000236959	ENSP00000306817
ENSP00000236959	ENSP00000311876
ENSP00000236959	ENSP00000328940
ENSP00000236959	ENSP00000329466
ENSP00000236959	ENSP00000330918
ENSP00000236959	ENSP00000337358
ENSP00000236959	ENSP00000345096
ENSP00000236959	ENSP00000352673
ENSP00000236959	ENSP00000355518
ENSP00000236959	ENSP00000360286
ENSP00000236959	ENSP00000360316
ENSP00000236959	ENSP00000361699

ENSP00000236959	ENSP00000361965
ENSP00000236959	ENSP00000363384
ENSP00000236959	ENSP00000365775
ENSP00000236959	ENSP00000365891
ENSP00000236959	ENSP00000366620
ENSP00000236959	ENSP00000369897
ENSP00000236959	ENSP00000382492
ENSP00000236959	ENSP00000382595
ENSP00000236959	ENSP00000400806
ENSP00000236980	ENSP00000301585
ENSP00000237014	ENSP00000263816
ENSP00000237014	ENSP00000265857
ENSP00000237014	ENSP00000284981
ENSP00000237014	ENSP00000295897
ENSP00000237014	ENSP00000307134
ENSP00000237014	ENSP00000336790
ENSP00000237014	ENSP00000360519
ENSP00000237014	ENSP00000363779
ENSP00000237014	ENSP00000363827
ENSP00000237014	ENSP00000364217
ENSP00000237014	ENSP00000401397
ENSP00000237014	ENSP00000416097
ENSP00000237281	ENSP00000257430
ENSP00000237281	ENSP00000281043
ENSP00000237281	ENSP00000281708
ENSP00000237281	ENSP00000304102
ENSP00000237281	ENSP00000306788
ENSP00000237281	ENSP00000320648
ENSP00000237281	ENSP00000341551
ENSP00000237283	ENSP00000251038
ENSP00000237283	ENSP00000252622
ENSP00000237283	ENSP00000273962
ENSP00000237283	ENSP00000282388
ENSP00000237283	ENSP00000294053
ENSP00000237283	ENSP00000298317
ENSP00000237283	ENSP00000300648
ENSP00000237283	ENSP00000323288
ENSP00000237283	ENSP00000342374
ENSP00000237283	ENSP00000346155
ENSP00000237283	ENSP00000359531
ENSP00000237283	ENSP00000373331
ENSP00000237289	ENSP00000242719
ENSP00000237289	ENSP00000248244
ENSP00000237289	ENSP00000259808
ENSP00000237289	ENSP00000260731
ENSP00000237289	ENSP00000264335

ENSP00000237289	ENSP00000265651
ENSP00000237289	ENSP00000287218
ENSP00000237289	ENSP00000294507
ENSP00000237289	ENSP00000301838
ENSP00000237289	ENSP00000311344
ENSP00000237289	ENSP00000313059
ENSP00000237289	ENSP00000317891
ENSP00000237289	ENSP00000321203
ENSP00000237289	ENSP00000329411
ENSP00000237289	ENSP00000329967
ENSP00000237289	ENSP00000333298
ENSP00000237289	ENSP00000351908
ENSP00000237289	ENSP00000355231
ENSP00000237289	ENSP00000358997
ENSP00000237289	ENSP00000367498
ENSP00000237289	ENSP00000368824
ENSP00000237289	ENSP00000370737
ENSP00000237289	ENSP00000376250
ENSP00000237289	ENSP00000379612
ENSP00000237380	ENSP00000251871
ENSP00000237380	ENSP00000252597
ENSP00000237380	ENSP00000255764
ENSP00000237380	ENSP00000256379
ENSP00000237380	ENSP00000258648
ENSP00000237380	ENSP00000261884
ENSP00000237380	ENSP00000263205
ENSP00000237380	ENSP00000263390
ENSP00000237380	ENSP00000265350
ENSP00000237380	ENSP00000268711
ENSP00000237380	ENSP00000272645
ENSP00000237380	ENSP00000281928
ENSP00000237380	ENSP00000282007
ENSP00000237380	ENSP00000282441
ENSP00000237380	ENSP00000282892
ENSP00000237380	ENSP00000286317
ENSP00000237380	ENSP00000290663
ENSP00000237380	ENSP00000292035
ENSP00000237380	ENSP00000293777
ENSP00000237380	ENSP00000297347
ENSP00000237380	ENSP00000300651
ENSP00000237380	ENSP00000301788
ENSP00000237380	ENSP00000304604
ENSP00000237380	ENSP00000312735
ENSP00000237380	ENSP00000313169
ENSP00000237380	ENSP00000314949
ENSP00000237380	ENSP00000323720

ENSP00000237380	ENSP00000324124
ENSP00000237380	ENSP00000325612
ENSP00000237380	ENSP00000326767
ENSP00000237380	ENSP00000337340
ENSP00000237380	ENSP00000340836
ENSP00000237380	ENSP00000342343
ENSP00000237380	ENSP00000344666
ENSP00000237380	ENSP00000348215
ENSP00000237380	ENSP00000348610
ENSP00000237380	ENSP00000356437
ENSP00000237380	ENSP00000356595
ENSP00000237380	ENSP00000357047
ENSP00000237380	ENSP00000357907
ENSP00000237380	ENSP00000362948
ENSP00000237380	ENSP00000363193
ENSP00000237380	ENSP00000366746
ENSP00000237455	ENSP00000259512
ENSP00000237455	ENSP00000263897
ENSP00000237455	ENSP00000272645
ENSP00000237455	ENSP00000312735
ENSP00000237455	ENSP00000324124
ENSP00000237455	ENSP00000347836
ENSP00000237455	ENSP00000354201
ENSP00000237455	ENSP00000383911
ENSP00000237455	ENSP00000403852
ENSP00000237530	ENSP00000239891
ENSP00000237530	ENSP00000243253
ENSP00000237530	ENSP00000250498
ENSP00000237530	ENSP00000251038
ENSP00000237530	ENSP00000253003
ENSP00000237530	ENSP00000259154
ENSP00000237530	ENSP00000259569
ENSP00000237530	ENSP00000261173
ENSP00000237530	ENSP00000262554
ENSP00000237530	ENSP00000276326
ENSP00000237530	ENSP00000295770
ENSP00000237530	ENSP00000295887
ENSP00000237530	ENSP00000295989
ENSP00000237530	ENSP00000296255
ENSP00000237530	ENSP00000296786
ENSP00000237530	ENSP00000299626
ENSP00000237530	ENSP00000299767
ENSP00000237530	ENSP00000304500
ENSP00000237530	ENSP00000304592
ENSP00000237530	ENSP00000306124
ENSP00000237530	ENSP00000307275

ENSP00000237530	ENSP00000314036
ENSP00000237530	ENSP00000317331
ENSP00000237530	ENSP00000319977
ENSP00000237530	ENSP00000319979
ENSP00000237530	ENSP00000320508
ENSP00000237530	ENSP00000321239
ENSP00000237530	ENSP00000326699
ENSP00000237530	ENSP00000329466
ENSP00000237530	ENSP00000329715
ENSP00000237530	ENSP00000340466
ENSP00000237530	ENSP00000341213
ENSP00000237530	ENSP00000341538
ENSP00000237530	ENSP00000343412
ENSP00000237530	ENSP00000345528
ENSP00000237530	ENSP00000353073
ENSP00000237530	ENSP00000355890
ENSP00000237530	ENSP00000359552
ENSP00000237530	ENSP00000360149
ENSP00000237530	ENSP00000361057
ENSP00000237530	ENSP00000362592
ENSP00000237530	ENSP00000362688
ENSP00000237530	ENSP00000364188
ENSP00000237530	ENSP00000366748
ENSP00000237530	ENSP00000370414
ENSP00000237530	ENSP00000370883
ENSP00000237530	ENSP00000377527
ENSP00000237530	ENSP00000379607
ENSP00000237530	ENSP00000380793
ENSP00000237530	ENSP00000392330
ENSP00000237596	ENSP00000261813
ENSP00000237596	ENSP00000262304
ENSP00000237596	ENSP00000316454
ENSP00000237596	ENSP00000329002
ENSP00000237596	ENSP00000341838
ENSP00000237596	ENSP00000380921
ENSP00000237596	ENSP00000381565
ENSP00000237596	ENSP00000392330
ENSP00000237654	ENSP00000256398
ENSP00000237654	ENSP00000256897
ENSP00000237654	ENSP00000257789
ENSP00000237654	ENSP00000260731
ENSP00000237654	ENSP00000269214
ENSP00000237654	ENSP00000287598
ENSP00000237654	ENSP00000296509
ENSP00000237654	ENSP00000296577
ENSP00000237654	ENSP00000302530

ENSP00000237654	ENSP00000306999
ENSP00000237654	ENSP00000307853
ENSP00000237654	ENSP00000310649
ENSP00000237654	ENSP00000336524
ENSP00000237654	ENSP00000343741
ENSP00000237654	ENSP00000353698
ENSP00000237654	ENSP00000356448
ENSP00000237654	ENSP00000360621
ENSP00000237654	ENSP00000363827
ENSP00000237654	ENSP00000372023
ENSP00000237654	ENSP00000377793
ENSP00000237654	ENSP00000391723
ENSP00000237822	ENSP00000261207
ENSP00000237822	ENSP00000272167
ENSP00000237822	ENSP00000299824
ENSP00000237822	ENSP00000349145
ENSP00000237837	ENSP00000339826
ENSP00000237853	ENSP00000255764
ENSP00000237853	ENSP00000263390
ENSP00000237853	ENSP00000264157
ENSP00000237853	ENSP00000265343
ENSP00000237853	ENSP00000277541
ENSP00000237853	ENSP00000296564
ENSP00000237853	ENSP00000305689
ENSP00000237853	ENSP00000337340
ENSP00000237853	ENSP00000357494
ENSP00000237853	ENSP00000367408
ENSP00000237853	ENSP00000369695
ENSP00000237853	ENSP00000380054
ENSP00000237889	ENSP00000252102
ENSP00000237889	ENSP00000349142
ENSP00000238112	ENSP00000245934
ENSP00000238112	ENSP00000253048
ENSP00000238112	ENSP00000256383
ENSP00000238112	ENSP00000256854
ENSP00000238112	ENSP00000261531
ENSP00000238112	ENSP00000262577
ENSP00000238112	ENSP00000263629
ENSP00000238112	ENSP00000263956
ENSP00000238112	ENSP00000264335
ENSP00000238112	ENSP00000291386
ENSP00000238112	ENSP00000291688
ENSP00000238112	ENSP00000292476
ENSP00000238112	ENSP00000293860
ENSP00000238112	ENSP00000296490
ENSP00000238112	ENSP00000296930

ENSP00000238112	ENSP00000298281
ENSP00000238112	ENSP00000298310
ENSP00000238112	ENSP00000302801
ENSP00000238112	ENSP00000304704
ENSP00000238112	ENSP00000311873
ENSP00000238112	ENSP00000314348
ENSP00000238112	ENSP00000314949
ENSP00000238112	ENSP00000315791
ENSP00000238112	ENSP00000317872
ENSP00000238112	ENSP00000322570
ENSP00000238112	ENSP00000325377
ENSP00000238112	ENSP00000325448
ENSP00000238112	ENSP00000329466
ENSP00000238112	ENSP00000330343
ENSP00000238112	ENSP00000336752
ENSP00000238112	ENSP00000339109
ENSP00000238112	ENSP00000339245
ENSP00000238112	ENSP00000339353
ENSP00000238112	ENSP00000339845
ENSP00000238112	ENSP00000346236
ENSP00000238112	ENSP00000348010
ENSP00000238112	ENSP00000351697
ENSP00000238112	ENSP00000351947
ENSP00000238112	ENSP00000356405
ENSP00000238112	ENSP00000356548
ENSP00000238112	ENSP00000360532
ENSP00000238112	ENSP00000367406
ENSP00000238112	ENSP00000368349
ENSP00000238112	ENSP00000374399
ENSP00000238112	ENSP00000380453
ENSP00000238112	ENSP00000386161
ENSP00000238112	ENSP00000402038
ENSP00000238256	ENSP00000246802
ENSP00000238256	ENSP00000254940
ENSP00000238256	ENSP00000265963
ENSP00000238256	ENSP00000317992
ENSP00000238256	ENSP00000322396
ENSP00000238256	ENSP00000338862
ENSP00000238256	ENSP00000354040
ENSP00000238256	ENSP00000359345
ENSP00000238256	ENSP00000359688
ENSP00000238256	ENSP00000360412
ENSP00000238256	ENSP00000363384
ENSP00000238256	ENSP00000380982
ENSP00000238256	ENSP00000381657
ENSP00000238256	ENSP00000402338

ENSP00000238341	ENSP00000261845
ENSP00000238341	ENSP00000341268
ENSP00000238341	ENSP00000375986
ENSP00000238379	ENSP00000266095
ENSP00000238558	ENSP00000253003
ENSP00000238558	ENSP00000341551
ENSP00000238558	ENSP00000366534
ENSP00000238558	ENSP00000372023
ENSP00000238616	ENSP00000255465
ENSP00000238616	ENSP00000256398
ENSP00000238616	ENSP00000256442
ENSP00000238616	ENSP00000262457
ENSP00000238616	ENSP00000262850
ENSP00000238616	ENSP00000276014
ENSP00000238616	ENSP00000276297
ENSP00000238616	ENSP00000278412
ENSP00000238616	ENSP00000348273
ENSP00000238616	ENSP00000348577
ENSP00000238647	ENSP00000250448
ENSP00000238647	ENSP00000251968
ENSP00000238647	ENSP00000253363
ENSP00000238647	ENSP00000257934
ENSP00000238647	ENSP00000268058
ENSP00000238647	ENSP00000292211
ENSP00000238647	ENSP00000297689
ENSP00000238647	ENSP00000323780
ENSP00000238647	ENSP00000343535
ENSP00000238647	ENSP00000344936
ENSP00000238647	ENSP00000349640
ENSP00000238647	ENSP00000351908
ENSP00000238647	ENSP00000355153
ENSP00000238647	ENSP00000357484
ENSP00000238647	ENSP00000359939
ENSP00000238647	ENSP00000361433
ENSP00000238647	ENSP00000362441
ENSP00000238647	ENSP00000364699
ENSP00000238647	ENSP00000377218
ENSP00000238647	ENSP00000380414
ENSP00000238647	ENSP00000417281
ENSP00000238651	ENSP00000354501
ENSP00000238788	ENSP00000310596
ENSP00000238789	ENSP00000247843
ENSP00000238789	ENSP00000251038
ENSP00000238789	ENSP00000251968
ENSP00000238789	ENSP00000254719
ENSP00000238789	ENSP00000254940

ENSP00000238789	ENSP00000256442
ENSP00000238789	ENSP00000256996
ENSP00000238789	ENSP00000257934
ENSP00000238789	ENSP00000259050
ENSP00000238789	ENSP00000261424
ENSP00000238789	ENSP00000262518
ENSP00000238789	ENSP00000263121
ENSP00000238789	ENSP00000263208
ENSP00000238789	ENSP00000263228
ENSP00000238789	ENSP00000263795
ENSP00000238789	ENSP00000263857
ENSP00000238789	ENSP00000263974
ENSP00000238789	ENSP00000264169
ENSP00000238789	ENSP00000264183
ENSP00000238789	ENSP00000265056
ENSP00000238789	ENSP00000265990
ENSP00000238789	ENSP00000267064
ENSP00000238789	ENSP00000269127
ENSP00000238789	ENSP00000273067
ENSP00000238789	ENSP00000274140
ENSP00000238789	ENSP00000278412
ENSP00000238789	ENSP00000286317
ENSP00000238789	ENSP00000287239
ENSP00000238789	ENSP00000287598
ENSP00000238789	ENSP00000287936
ENSP00000238789	ENSP00000289528
ENSP00000238789	ENSP00000293406
ENSP00000238789	ENSP00000294168
ENSP00000238789	ENSP00000296273
ENSP00000238789	ENSP00000296328
ENSP00000238789	ENSP00000296509
ENSP00000238789	ENSP00000302530
ENSP00000238789	ENSP00000302886
ENSP00000238789	ENSP00000304593
ENSP00000238789	ENSP00000305810
ENSP00000238789	ENSP00000307208
ENSP00000238789	ENSP00000309141
ENSP00000238789	ENSP00000310129
ENSP00000238789	ENSP00000311496
ENSP00000238789	ENSP00000312735
ENSP00000238789	ENSP00000312870
ENSP00000238789	ENSP00000313171
ENSP00000238789	ENSP00000314193
ENSP00000238789	ENSP00000314949
ENSP00000238789	ENSP00000317087
ENSP00000238789	ENSP00000317468

ENSP00000238789	ENSP00000318977
ENSP00000238789	ENSP00000324124
ENSP00000238789	ENSP00000325002
ENSP00000238789	ENSP00000329419
ENSP00000238789	ENSP00000329466
ENSP00000238789	ENSP00000329933
ENSP00000238789	ENSP00000334564
ENSP00000238789	ENSP00000336842
ENSP00000238789	ENSP00000344504
ENSP00000238789	ENSP00000344537
ENSP00000238789	ENSP00000345702
ENSP00000238789	ENSP00000348442
ENSP00000238789	ENSP00000349078
ENSP00000238789	ENSP00000349213
ENSP00000238789	ENSP00000350708
ENSP00000238789	ENSP00000353458
ENSP00000238789	ENSP00000353622
ENSP00000238789	ENSP00000355205
ENSP00000238789	ENSP00000355890
ENSP00000238789	ENSP00000356405
ENSP00000238789	ENSP00000356989
ENSP00000238789	ENSP00000357048
ENSP00000238789	ENSP00000357887
ENSP00000238789	ENSP00000360116
ENSP00000238789	ENSP00000360412
ENSP00000238789	ENSP00000360621
ENSP00000238789	ENSP00000361465
ENSP00000238789	ENSP00000362208
ENSP00000238789	ENSP00000362334
ENSP00000238789	ENSP00000362688
ENSP00000238789	ENSP00000364486
ENSP00000238789	ENSP00000365025
ENSP00000238789	ENSP00000366974
ENSP00000238789	ENSP00000367029
ENSP00000238789	ENSP00000369411
ENSP00000238789	ENSP00000369716
ENSP00000238789	ENSP00000370589
ENSP00000238789	ENSP00000372023
ENSP00000238789	ENSP00000373340
ENSP00000238789	ENSP00000374213
ENSP00000238789	ENSP00000376534
ENSP00000238789	ENSP00000377747
ENSP00000238789	ENSP00000380982
ENSP00000238789	ENSP00000392028
ENSP00000238789	ENSP00000392617
ENSP00000238789	ENSP00000396704

ENSP00000238789	ENSP00000418734
ENSP00000238823	ENSP00000261700
ENSP00000238823	ENSP00000330343
ENSP00000238823	ENSP00000379607
ENSP00000238831	ENSP00000251775
ENSP00000238831	ENSP00000256433
ENSP00000238831	ENSP00000258301
ENSP00000238831	ENSP00000260643
ENSP00000238831	ENSP00000262544
ENSP00000238831	ENSP00000263116
ENSP00000238831	ENSP00000285243
ENSP00000238831	ENSP00000300022
ENSP00000238831	ENSP00000305255
ENSP00000238831	ENSP00000311134
ENSP00000238831	ENSP00000325002
ENSP00000238831	ENSP00000347329
ENSP00000238831	ENSP00000368959
ENSP00000238831	ENSP00000376871
ENSP00000238831	ENSP00000394071
ENSP00000238855	ENSP00000377148
ENSP00000238855	ENSP00000381045
ENSP00000238892	ENSP00000345731
ENSP00000238892	ENSP00000348211
ENSP00000238994	ENSP00000285896
ENSP00000238994	ENSP00000294724
ENSP00000238994	ENSP00000317904
ENSP00000238994	ENSP00000345464
ENSP00000238994	ENSP00000356489
ENSP00000239125	ENSP00000357480
ENSP00000239587	ENSP00000261424
ENSP00000239587	ENSP00000280665
ENSP00000239587	ENSP00000313171
ENSP00000239587	ENSP00000320503
ENSP00000239614	ENSP00000285398
ENSP00000239690	ENSP00000310590
ENSP00000239690	ENSP00000322706
ENSP00000239690	ENSP00000394085
ENSP00000239849	ENSP00000257527
ENSP00000239849	ENSP00000269485
ENSP00000239849	ENSP00000297350
ENSP00000239849	ENSP00000309968
ENSP00000239849	ENSP00000313829
ENSP00000239849	ENSP00000348307
ENSP00000239849	ENSP00000357798
ENSP00000239849	ENSP00000375097
ENSP00000239878	ENSP00000267169

ENSP00000239878	ENSP00000341551
ENSP00000239878	ENSP00000344683
ENSP00000239878	ENSP00000348565
ENSP00000239878	ENSP00000356982
ENSP00000239891	ENSP00000243253
ENSP00000239891	ENSP00000250498
ENSP00000239891	ENSP00000262812
ENSP00000239891	ENSP00000264501
ENSP00000239891	ENSP00000266483
ENSP00000239891	ENSP00000277010
ENSP00000239891	ENSP00000295770
ENSP00000239891	ENSP00000296255
ENSP00000239891	ENSP00000299626
ENSP00000239891	ENSP00000300658
ENSP00000239891	ENSP00000308339
ENSP00000239891	ENSP00000325369
ENSP00000239891	ENSP00000330343
ENSP00000239891	ENSP00000331268
ENSP00000239891	ENSP00000333813
ENSP00000239891	ENSP00000334052
ENSP00000239891	ENSP00000346809
ENSP00000239891	ENSP00000353104
ENSP00000239891	ENSP00000353622
ENSP00000239891	ENSP00000356530
ENSP00000239891	ENSP00000359848
ENSP00000239891	ENSP00000360149
ENSP00000239891	ENSP00000361845
ENSP00000239891	ENSP00000364188
ENSP00000239891	ENSP00000380793
ENSP00000239891	ENSP00000386918
ENSP00000239891	ENSP00000401445
ENSP00000239891	ENSP00000402338
ENSP00000239891	ENSP00000415203
ENSP00000239906	ENSP00000313059
ENSP00000239938	ENSP00000247970
ENSP00000239938	ENSP00000262367
ENSP00000239938	ENSP00000263341
ENSP00000239938	ENSP00000267101
ENSP00000239938	ENSP00000300131
ENSP00000239938	ENSP00000305422
ENSP00000239938	ENSP00000329357
ENSP00000239938	ENSP00000334188
ENSP00000239938	ENSP00000355153
ENSP00000239938	ENSP00000357494
ENSP00000239938	ENSP00000362717
ENSP00000239938	ENSP00000366607

ENSP00000239944	ENSP00000243253
ENSP00000239944	ENSP00000349887
ENSP00000239944	ENSP00000365131
ENSP00000240055	ENSP00000256398
ENSP00000240055	ENSP00000262367
ENSP00000240055	ENSP00000263795
ENSP00000240055	ENSP00000263974
ENSP00000240055	ENSP00000264169
ENSP00000240055	ENSP00000265073
ENSP00000240055	ENSP00000286317
ENSP00000240055	ENSP00000296509
ENSP00000240055	ENSP00000298937
ENSP00000240055	ENSP00000307850
ENSP00000240055	ENSP00000316328
ENSP00000240055	ENSP00000329357
ENSP00000240055	ENSP00000329933
ENSP00000240055	ENSP00000331310
ENSP00000240055	ENSP00000344537
ENSP00000240055	ENSP00000345702
ENSP00000240055	ENSP00000350967
ENSP00000240055	ENSP00000352738
ENSP00000240055	ENSP00000354633
ENSP00000240055	ENSP00000360621
ENSP00000240055	ENSP00000363779
ENSP00000240055	ENSP00000369785
ENSP00000240055	ENSP00000386759
ENSP00000240055	ENSP00000396620
ENSP00000240079	ENSP00000261017
ENSP00000240079	ENSP00000261597
ENSP00000240079	ENSP00000263036
ENSP00000240079	ENSP00000305503
ENSP00000240079	ENSP00000317542
ENSP00000240079	ENSP00000326767
ENSP00000240079	ENSP00000327077
ENSP00000240079	ENSP00000343635
ENSP00000240079	ENSP00000349640
ENSP00000240079	ENSP00000352425
ENSP00000240079	ENSP00000357448
ENSP00000240079	ENSP00000367265
ENSP00000240079	ENSP00000392896
ENSP00000240159	ENSP00000246548
ENSP00000240159	ENSP00000249636
ENSP00000240159	ENSP00000261396
ENSP00000240159	ENSP00000261424
ENSP00000240159	ENSP00000261601
ENSP00000240159	ENSP00000263208

ENSP00000240159	ENSP00000264183
ENSP00000240159	ENSP00000264515
ENSP00000240159	ENSP00000265990
ENSP00000240159	ENSP00000270225
ENSP00000240159	ENSP00000274140
ENSP00000240159	ENSP00000282077
ENSP00000240159	ENSP00000287437
ENSP00000240159	ENSP00000295066
ENSP00000240159	ENSP00000296257
ENSP00000240159	ENSP00000296786
ENSP00000240159	ENSP00000298139
ENSP00000240159	ENSP00000302160
ENSP00000240159	ENSP00000304593
ENSP00000240159	ENSP00000310596
ENSP00000240159	ENSP00000312244
ENSP00000240159	ENSP00000313171
ENSP00000240159	ENSP00000317039
ENSP00000240159	ENSP00000321636
ENSP00000240159	ENSP00000324792
ENSP00000240159	ENSP00000325863
ENSP00000240159	ENSP00000327197
ENSP00000240159	ENSP00000330343
ENSP00000240159	ENSP00000331310
ENSP00000240159	ENSP00000334474
ENSP00000240159	ENSP00000347232
ENSP00000240159	ENSP00000347602
ENSP00000240159	ENSP00000351284
ENSP00000240159	ENSP00000356989
ENSP00000240159	ENSP00000360116
ENSP00000240159	ENSP00000360473
ENSP00000240159	ENSP00000366135
ENSP00000240159	ENSP00000377655
ENSP00000240159	ENSP00000377793
ENSP00000240159	ENSP00000379217
ENSP00000240159	ENSP00000380460
ENSP00000240159	ENSP00000384900
ENSP00000240159	ENSP00000386759
ENSP00000240159	ENSP00000394791
ENSP00000240159	ENSP00000402527
ENSP00000240185	ENSP00000251038
ENSP00000240185	ENSP00000253363
ENSP00000240185	ENSP00000255764
ENSP00000240185	ENSP00000261531
ENSP00000240185	ENSP00000262803
ENSP00000240185	ENSP00000268957
ENSP00000240185	ENSP00000285896

ENSP00000240185	ENSP00000290231
ENSP00000240185	ENSP00000301364
ENSP00000240185	ENSP00000304704
ENSP00000240185	ENSP00000315791
ENSP00000240185	ENSP00000320949
ENSP00000240185	ENSP00000325376
ENSP00000240185	ENSP00000331879
ENSP00000240185	ENSP00000332444
ENSP00000240185	ENSP00000334061
ENSP00000240185	ENSP00000336712
ENSP00000240185	ENSP00000337340
ENSP00000240185	ENSP00000338862
ENSP00000240185	ENSP00000347948
ENSP00000240185	ENSP00000348168
ENSP00000240185	ENSP00000348708
ENSP00000240185	ENSP00000349594
ENSP00000240185	ENSP00000354522
ENSP00000240185	ENSP00000354673
ENSP00000240185	ENSP00000358997
ENSP00000240185	ENSP00000359531
ENSP00000240185	ENSP00000360532
ENSP00000240185	ENSP00000362900
ENSP00000240185	ENSP00000366135
ENSP00000240185	ENSP00000366843
ENSP00000240185	ENSP00000371155
ENSP00000240185	ENSP00000374455
ENSP00000240185	ENSP00000379607
ENSP00000240185	ENSP00000391723
ENSP00000240304	ENSP00000244520
ENSP00000240304	ENSP00000254193
ENSP00000240304	ENSP00000258416
ENSP00000240304	ENSP00000261973
ENSP00000240304	ENSP00000262982
ENSP00000240304	ENSP00000266735
ENSP00000240304	ENSP00000268206
ENSP00000240304	ENSP00000283195
ENSP00000240304	ENSP00000296805
ENSP00000240304	ENSP00000300413
ENSP00000240304	ENSP00000304350
ENSP00000240304	ENSP00000304370
ENSP00000240304	ENSP00000305790
ENSP00000240304	ENSP00000307863
ENSP00000240304	ENSP00000307939
ENSP00000240304	ENSP00000308546
ENSP00000240304	ENSP00000308925
ENSP00000240304	ENSP00000309457

ENSP00000240304	ENSP00000310448
ENSP00000240304	ENSP00000314311
ENSP00000240304	ENSP00000315700
ENSP00000240304	ENSP00000315859
ENSP00000240304	ENSP00000319341
ENSP00000240304	ENSP00000326806
ENSP00000240304	ENSP00000332103
ENSP00000240304	ENSP00000334148
ENSP00000240304	ENSP00000335321
ENSP00000240304	ENSP00000337340
ENSP00000240304	ENSP00000337405
ENSP00000240304	ENSP00000339145
ENSP00000240304	ENSP00000342374
ENSP00000240304	ENSP00000344241
ENSP00000240304	ENSP00000346986
ENSP00000240304	ENSP00000348010
ENSP00000240304	ENSP00000354720
ENSP00000240304	ENSP00000357631
ENSP00000240304	ENSP00000361577
ENSP00000240304	ENSP00000364289
ENSP00000240304	ENSP00000366607
ENSP00000240304	ENSP00000376309
ENSP00000240304	ENSP00000377262
ENSP00000240304	ENSP00000379607
ENSP00000240304	ENSP00000412566
ENSP00000240304	ENSP00000416959
ENSP00000240304	ENSP00000420195
ENSP00000240316	ENSP00000242872
ENSP00000240316	ENSP00000244769
ENSP00000240316	ENSP00000250113
ENSP00000240316	ENSP00000252699
ENSP00000240316	ENSP00000260129
ENSP00000240316	ENSP00000260648
ENSP00000240316	ENSP00000262127
ENSP00000240316	ENSP00000262375
ENSP00000240316	ENSP00000262643
ENSP00000240316	ENSP00000263925
ENSP00000240316	ENSP00000264982
ENSP00000240316	ENSP00000270176
ENSP00000240316	ENSP00000293362
ENSP00000240316	ENSP00000297788
ENSP00000240316	ENSP00000314837
ENSP00000240316	ENSP00000318821
ENSP00000240316	ENSP00000324203
ENSP00000240316	ENSP00000329466
ENSP00000240316	ENSP00000342381

ENSP00000240316	ENSP00000349465
ENSP00000240316	ENSP00000352257
ENSP00000240316	ENSP00000354501
ENSP00000240316	ENSP00000363787
ENSP00000240316	ENSP00000364398
ENSP00000240316	ENSP00000364591
ENSP00000240316	ENSP00000365025
ENSP00000240316	ENSP00000365198
ENSP00000240316	ENSP00000370083
ENSP00000240316	ENSP00000370119
ENSP00000240316	ENSP00000370671
ENSP00000240316	ENSP00000370968
ENSP00000240316	ENSP00000382562
ENSP00000240316	ENSP00000386394
ENSP00000240316	ENSP00000396439
ENSP00000240316	ENSP00000412566
ENSP00000240316	ENSP00000419235
ENSP00000240335	ENSP00000250448
ENSP00000240335	ENSP00000297261
ENSP00000240423	ENSP00000243103
ENSP00000240423	ENSP00000247970
ENSP00000240423	ENSP00000249499
ENSP00000240423	ENSP00000249504
ENSP00000240423	ENSP00000251496
ENSP00000240423	ENSP00000260731
ENSP00000240423	ENSP00000260766
ENSP00000240423	ENSP00000261531
ENSP00000240423	ENSP00000263710
ENSP00000240423	ENSP00000269127
ENSP00000240423	ENSP00000283921
ENSP00000240423	ENSP00000297338
ENSP00000240423	ENSP00000302114
ENSP00000240423	ENSP00000304437
ENSP00000240423	ENSP00000308699
ENSP00000240423	ENSP00000311873
ENSP00000240423	ENSP00000318977
ENSP00000240423	ENSP00000325017
ENSP00000240423	ENSP00000334675
ENSP00000240423	ENSP00000336606
ENSP00000240423	ENSP00000339109
ENSP00000240423	ENSP00000341243
ENSP00000240423	ENSP00000341382
ENSP00000240423	ENSP00000347978
ENSP00000240423	ENSP00000348089
ENSP00000240423	ENSP00000353793
ENSP00000240423	ENSP00000354995

ENSP00000240423	ENSP00000356607
ENSP00000240423	ENSP00000358062
ENSP00000240423	ENSP00000358478
ENSP00000240423	ENSP00000360532
ENSP00000240423	ENSP00000361264
ENSP00000240423	ENSP00000362441
ENSP00000240423	ENSP00000366746
ENSP00000240423	ENSP00000369716
ENSP00000240423	ENSP00000372023
ENSP00000240423	ENSP00000377747
ENSP00000240423	ENSP00000377793
ENSP00000240423	ENSP00000384675
ENSP00000240423	ENSP00000392028
ENSP00000240423	ENSP00000396704
ENSP00000240423	ENSP00000405963
ENSP00000240488	ENSP00000256442
ENSP00000240488	ENSP00000262315
ENSP00000240488	ENSP00000263187
ENSP00000240488	ENSP00000278616
ENSP00000240488	ENSP00000296273
ENSP00000240488	ENSP00000313171
ENSP00000240488	ENSP00000313420
ENSP00000240488	ENSP00000343392
ENSP00000240488	ENSP00000343741
ENSP00000240488	ENSP00000355167
ENSP00000240488	ENSP00000360310
ENSP00000240488	ENSP00000369411
ENSP00000240488	ENSP00000370151
ENSP00000240488	ENSP00000377384
ENSP00000240587	ENSP00000326296
ENSP00000240587	ENSP00000331608
ENSP00000240587	ENSP00000344456
ENSP00000240587	ENSP00000346155
ENSP00000240587	ENSP00000347294
ENSP00000240587	ENSP00000354586
ENSP00000240587	ENSP00000358799
ENSP00000240587	ENSP00000364912
ENSP00000240618	ENSP00000246551
ENSP00000240618	ENSP00000262629
ENSP00000240618	ENSP00000356329
ENSP00000240618	ENSP00000360286
ENSP00000240618	ENSP00000402134
ENSP00000240652	ENSP00000253796
ENSP00000240652	ENSP00000254661
ENSP00000240652	ENSP00000370421
ENSP00000240851	ENSP00000247970

ENSP00000240851	ENSP00000251968
ENSP00000240851	ENSP00000282892
ENSP00000240851	ENSP00000297338
ENSP00000240851	ENSP00000347294
ENSP00000240851	ENSP00000379607
ENSP00000240922	ENSP00000247843
ENSP00000240922	ENSP00000251038
ENSP00000240922	ENSP00000261745
ENSP00000240922	ENSP00000264995
ENSP00000240922	ENSP00000268661
ENSP00000240922	ENSP00000274606
ENSP00000240922	ENSP00000278572
ENSP00000240922	ENSP00000283109
ENSP00000240922	ENSP00000286428
ENSP00000240922	ENSP00000296509
ENSP00000240922	ENSP00000296543
ENSP00000240922	ENSP00000304370
ENSP00000240922	ENSP00000307684
ENSP00000240922	ENSP00000309555
ENSP00000240922	ENSP00000317992
ENSP00000240922	ENSP00000323858
ENSP00000240922	ENSP00000323967
ENSP00000240922	ENSP00000334148
ENSP00000240922	ENSP00000350512
ENSP00000240922	ENSP00000354850
ENSP00000240922	ENSP00000354972
ENSP00000240922	ENSP00000359290
ENSP00000240922	ENSP00000360532
ENSP00000240922	ENSP00000362105
ENSP00000240922	ENSP00000377617
ENSP00000240922	ENSP00000384474
ENSP00000240922	ENSP00000392617
ENSP00000240922	ENSP00000416583
ENSP00000240922	ENSP00000417229
ENSP00000240922	ENSP00000417763
ENSP00000241041	ENSP00000349016
ENSP00000241041	ENSP00000356563
ENSP00000241041	ENSP00000357051
ENSP00000241051	ENSP00000261531
ENSP00000241051	ENSP00000294008
ENSP00000241051	ENSP00000359531
ENSP00000241051	ENSP00000360532
ENSP00000241052	ENSP00000255390
ENSP00000241052	ENSP00000260045
ENSP00000241052	ENSP00000266126
ENSP00000241052	ENSP00000270776

ENSP00000241052	ENSP00000274030
ENSP00000241052	ENSP00000284957
ENSP00000241052	ENSP00000284981
ENSP00000241052	ENSP00000285896
ENSP00000241052	ENSP00000287078
ENSP00000241052	ENSP00000299335
ENSP00000241052	ENSP00000304292
ENSP00000241052	ENSP00000308925
ENSP00000241052	ENSP00000310668
ENSP00000241052	ENSP00000340836
ENSP00000241052	ENSP00000340944
ENSP00000241052	ENSP00000344460
ENSP00000241052	ENSP00000349016
ENSP00000241052	ENSP00000356595
ENSP00000241052	ENSP00000356785
ENSP00000241052	ENSP00000357494
ENSP00000241052	ENSP00000358312
ENSP00000241052	ENSP00000360286
ENSP00000241052	ENSP00000373700
ENSP00000241052	ENSP00000374467
ENSP00000241052	ENSP00000377665
ENSP00000241052	ENSP00000379678
ENSP00000241052	ENSP00000394869
ENSP00000241052	ENSP00000407401
ENSP00000241071	ENSP00000273398
ENSP00000241071	ENSP00000310129
ENSP00000241071	ENSP00000337501
ENSP00000241071	ENSP00000379612
ENSP00000241071	ENSP00000417748
ENSP00000241436	ENSP00000249636
ENSP00000241436	ENSP00000254719
ENSP00000241436	ENSP00000258428
ENSP00000241436	ENSP00000258774
ENSP00000241436	ENSP00000264233
ENSP00000241436	ENSP00000264926
ENSP00000241436	ENSP00000268124
ENSP00000241436	ENSP00000269214
ENSP00000241436	ENSP00000306561
ENSP00000241436	ENSP00000306999
ENSP00000241436	ENSP00000308944
ENSP00000241436	ENSP00000311873
ENSP00000241436	ENSP00000313350
ENSP00000241436	ENSP00000318977
ENSP00000241436	ENSP00000323439
ENSP00000241436	ENSP00000340305
ENSP00000241436	ENSP00000340879

ENSP00000241436	ENSP00000343392
ENSP00000241436	ENSP00000347978
ENSP00000241436	ENSP00000351284
ENSP00000241436	ENSP00000351697
ENSP00000241436	ENSP00000352257
ENSP00000241436	ENSP00000354720
ENSP00000241436	ENSP00000361310
ENSP00000241436	ENSP00000364270
ENSP00000241436	ENSP00000368438
ENSP00000241436	ENSP00000369335
ENSP00000241436	ENSP00000370150
ENSP00000241436	ENSP00000372316
ENSP00000241436	ENSP00000381657
ENSP00000241453	ENSP00000264122
ENSP00000241453	ENSP00000340944
ENSP00000241453	ENSP00000348685
ENSP00000241453	ENSP00000364893
ENSP00000241453	ENSP00000415151
ENSP00000241502	ENSP00000261531
ENSP00000241502	ENSP00000369816
ENSP00000241527	ENSP00000244769
ENSP00000241527	ENSP00000301585
ENSP00000241527	ENSP00000419740
ENSP00000241600	ENSP00000242057
ENSP00000241600	ENSP00000245539
ENSP00000241600	ENSP00000254928
ENSP00000241600	ENSP00000256897
ENSP00000241600	ENSP00000258383
ENSP00000241600	ENSP00000258455
ENSP00000241600	ENSP00000260443
ENSP00000241600	ENSP00000262030
ENSP00000241600	ENSP00000263657
ENSP00000241600	ENSP00000264705
ENSP00000241600	ENSP00000268206
ENSP00000241600	ENSP00000270625
ENSP00000241600	ENSP00000272418
ENSP00000241600	ENSP00000274606
ENSP00000241600	ENSP00000278572
ENSP00000241600	ENSP00000279242
ENSP00000241600	ENSP00000280557
ENSP00000241600	ENSP00000288937
ENSP00000241600	ENSP00000295491
ENSP00000241600	ENSP00000296674
ENSP00000241600	ENSP00000296802
ENSP00000241600	ENSP00000296805
ENSP00000241600	ENSP00000297908

ENSP00000241600	ENSP00000299767
ENSP00000241600	ENSP00000301585
ENSP00000241600	ENSP00000301729
ENSP00000241600	ENSP00000302896
ENSP00000241600	ENSP00000306548
ENSP00000241600	ENSP00000308275
ENSP00000241600	ENSP00000311028
ENSP00000241600	ENSP00000312311
ENSP00000241600	ENSP00000314129
ENSP00000241600	ENSP00000315017
ENSP00000241600	ENSP00000317376
ENSP00000241600	ENSP00000318646
ENSP00000241600	ENSP00000321636
ENSP00000241600	ENSP00000323856
ENSP00000241600	ENSP00000326981
ENSP00000241600	ENSP00000331019
ENSP00000241600	ENSP00000336702
ENSP00000241600	ENSP00000337127
ENSP00000241600	ENSP00000337839
ENSP00000241600	ENSP00000339095
ENSP00000241600	ENSP00000339844
ENSP00000241600	ENSP00000341692
ENSP00000241600	ENSP00000341885
ENSP00000241600	ENSP00000343081
ENSP00000241600	ENSP00000345096
ENSP00000241600	ENSP00000346022
ENSP00000241600	ENSP00000346050
ENSP00000241600	ENSP00000346453
ENSP00000241600	ENSP00000347087
ENSP00000241600	ENSP00000348722
ENSP00000241600	ENSP00000348965
ENSP00000241600	ENSP00000352706
ENSP00000241600	ENSP00000355471
ENSP00000241600	ENSP00000356134
ENSP00000241600	ENSP00000358140
ENSP00000241600	ENSP00000358812
ENSP00000241600	ENSP00000360498
ENSP00000241600	ENSP00000360782
ENSP00000241600	ENSP00000361076
ENSP00000241600	ENSP00000361206
ENSP00000241600	ENSP00000361465
ENSP00000241600	ENSP00000362036
ENSP00000241600	ENSP00000362135
ENSP00000241600	ENSP00000362208
ENSP00000241600	ENSP00000362744
ENSP00000241600	ENSP00000363018

ENSP00000241600	ENSP00000364320
ENSP00000241600	ENSP00000368790
ENSP00000241600	ENSP00000369757
ENSP00000241600	ENSP00000370258
ENSP00000241600	ENSP00000372445
ENSP00000241600	ENSP00000377486
ENSP00000241600	ENSP00000377640
ENSP00000241600	ENSP00000377865
ENSP00000241600	ENSP00000379339
ENSP00000241600	ENSP00000379888
ENSP00000241600	ENSP00000382250
ENSP00000241600	ENSP00000392859
ENSP00000241600	ENSP00000393241
ENSP00000241600	ENSP00000403175
ENSP00000241600	ENSP00000412583
ENSP00000241600	ENSP00000414321
ENSP00000241600	ENSP00000416110
ENSP00000241600	ENSP00000420714
ENSP00000242057	ENSP00000250448
ENSP00000242057	ENSP00000252996
ENSP00000242057	ENSP00000267163
ENSP00000242057	ENSP00000269385
ENSP00000242057	ENSP00000301764
ENSP00000242057	ENSP00000327048
ENSP00000242057	ENSP00000327213
ENSP00000242057	ENSP00000329357
ENSP00000242057	ENSP00000340823
ENSP00000242057	ENSP00000341692
ENSP00000242057	ENSP00000344456
ENSP00000242057	ENSP00000346067
ENSP00000242057	ENSP00000348551
ENSP00000242057	ENSP00000351407
ENSP00000242057	ENSP00000357341
ENSP00000242057	ENSP00000377969
ENSP00000242057	ENSP00000384109
ENSP00000242057	ENSP00000399968
ENSP00000242067	ENSP00000245157
ENSP00000242067	ENSP00000264499
ENSP00000242067	ENSP00000268057
ENSP00000242067	ENSP00000295240
ENSP00000242067	ENSP00000296135
ENSP00000242067	ENSP00000319062
ENSP00000242067	ENSP00000353701
ENSP00000242067	ENSP00000370031
ENSP00000242067	ENSP00000376946
ENSP00000242109	ENSP00000345528

ENSP00000242152	ENSP00000267017
ENSP00000242152	ENSP00000307822
ENSP00000242152	ENSP00000332225
ENSP00000242152	ENSP00000335074
ENSP00000242152	ENSP00000339377
ENSP00000242152	ENSP00000368766
ENSP00000242208	ENSP00000243786
ENSP00000242208	ENSP00000295633
ENSP00000242208	ENSP00000311184
ENSP00000242208	ENSP00000355010
ENSP00000242208	ENSP00000362299
ENSP00000242210	ENSP00000310800
ENSP00000242285	ENSP00000243918
ENSP00000242285	ENSP00000253083
ENSP00000242285	ENSP00000256658
ENSP00000242285	ENSP00000260257
ENSP00000242285	ENSP00000261558
ENSP00000242285	ENSP00000261842
ENSP00000242285	ENSP00000262812
ENSP00000242285	ENSP00000269122
ENSP00000242285	ENSP00000270176
ENSP00000242285	ENSP00000299601
ENSP00000242285	ENSP00000314499
ENSP00000242285	ENSP00000317431
ENSP00000242285	ENSP00000320543
ENSP00000242285	ENSP00000326550
ENSP00000242285	ENSP00000340935
ENSP00000242285	ENSP00000346566
ENSP00000242285	ENSP00000350199
ENSP00000242285	ENSP00000352603
ENSP00000242285	ENSP00000360798
ENSP00000242285	ENSP00000365891
ENSP00000242285	ENSP00000368818
ENSP00000242285	ENSP00000371341
ENSP00000242285	ENSP00000377148
ENSP00000242285	ENSP00000379607
ENSP00000242317	ENSP00000300249
ENSP00000242317	ENSP00000327009
ENSP00000242317	ENSP00000334188
ENSP00000242317	ENSP00000351665
ENSP00000242317	ENSP00000356579
ENSP00000242317	ENSP00000377793
ENSP00000242338	ENSP00000248098
ENSP00000242338	ENSP00000263409
ENSP00000242338	ENSP00000284981
ENSP00000242338	ENSP00000293925

ENSP00000242338	ENSP00000300574
ENSP00000242338	ENSP00000309338
ENSP00000242338	ENSP00000338799
ENSP00000242338	ENSP00000355370
ENSP00000242338	ENSP00000376188
ENSP00000242351	ENSP00000329466
ENSP00000242351	ENSP00000379607
ENSP00000242465	ENSP00000295897
ENSP00000242465	ENSP00000337396
ENSP00000242465	ENSP00000358925
ENSP00000242465	ENSP00000364403
ENSP00000242480	ENSP00000248098
ENSP00000242480	ENSP00000300131
ENSP00000242480	ENSP00000309555
ENSP00000242480	ENSP00000342032
ENSP00000242480	ENSP00000356972
ENSP00000242480	ENSP00000357494
ENSP00000242480	ENSP00000362717
ENSP00000242480	ENSP00000366607
ENSP00000242576	ENSP00000248553
ENSP00000242576	ENSP00000264451
ENSP00000242576	ENSP00000265081
ENSP00000242576	ENSP00000265304
ENSP00000242576	ENSP00000267430
ENSP00000242576	ENSP00000291565
ENSP00000242576	ENSP00000301764
ENSP00000242576	ENSP00000307853
ENSP00000242576	ENSP00000310520
ENSP00000242576	ENSP00000315644
ENSP00000242576	ENSP00000340879
ENSP00000242576	ENSP00000343392
ENSP00000242576	ENSP00000350491
ENSP00000242576	ENSP00000355890
ENSP00000242576	ENSP00000356785
ENSP00000242576	ENSP00000363021
ENSP00000242576	ENSP00000364126
ENSP00000242576	ENSP00000364270
ENSP00000242576	ENSP00000365534
ENSP00000242576	ENSP00000368438
ENSP00000242576	ENSP00000370376
ENSP00000242576	ENSP00000374213
ENSP00000242576	ENSP00000414066
ENSP00000242576	ENSP00000420037
ENSP00000242719	ENSP00000259808
ENSP00000242719	ENSP00000262320
ENSP00000242719	ENSP00000262854

ENSP00000242719	ENSP00000263036
ENSP00000242719	ENSP00000272645
ENSP00000242719	ENSP00000307567
ENSP00000242719	ENSP00000312735
ENSP00000242719	ENSP00000317891
ENSP00000242719	ENSP00000324124
ENSP00000242719	ENSP00000341551
ENSP00000242719	ENSP00000345530
ENSP00000242719	ENSP00000359688
ENSP00000242719	ENSP00000360116
ENSP00000242719	ENSP00000360798
ENSP00000242719	ENSP00000360992
ENSP00000242719	ENSP00000379612
ENSP00000242719	ENSP00000403852
ENSP00000242728	ENSP00000250003
ENSP00000242728	ENSP00000305422
ENSP00000242728	ENSP00000329357
ENSP00000242728	ENSP00000331057
ENSP00000242728	ENSP00000386165
ENSP00000242784	ENSP00000243563
ENSP00000242872	ENSP00000254908
ENSP00000242872	ENSP00000256637
ENSP00000242872	ENSP00000261745
ENSP00000242872	ENSP00000262643
ENSP00000242872	ENSP00000264335
ENSP00000242872	ENSP00000267163
ENSP00000242872	ENSP00000276893
ENSP00000242872	ENSP00000283006
ENSP00000242872	ENSP00000301280
ENSP00000242872	ENSP00000319788
ENSP00000242872	ENSP00000336868
ENSP00000242872	ENSP00000353868
ENSP00000242872	ENSP00000359518
ENSP00000242872	ENSP00000360621
ENSP00000242872	ENSP00000362824
ENSP00000242872	ENSP00000368438
ENSP00000242872	ENSP00000382492
ENSP00000242872	ENSP00000387477
ENSP00000243103	ENSP00000318423
ENSP00000243103	ENSP00000326296
ENSP00000243167	ENSP00000245222
ENSP00000243167	ENSP00000251038
ENSP00000243167	ENSP00000256854
ENSP00000243167	ENSP00000263985
ENSP00000243167	ENSP00000264161
ENSP00000243167	ENSP00000318351

ENSP00000243167	ENSP00000355890
ENSP00000243167	ENSP00000360268
ENSP00000243167	ENSP00000370377
ENSP00000243253	ENSP00000245680
ENSP00000243253	ENSP00000247138
ENSP00000243253	ENSP00000248378
ENSP00000243253	ENSP00000249356
ENSP00000243253	ENSP00000250498
ENSP00000243253	ENSP00000253099
ENSP00000243253	ENSP00000257575
ENSP00000243253	ENSP00000259050
ENSP00000243253	ENSP00000259469
ENSP00000243253	ENSP00000259512
ENSP00000243253	ENSP00000262177
ENSP00000243253	ENSP00000262812
ENSP00000243253	ENSP00000263897
ENSP00000243253	ENSP00000265100
ENSP00000243253	ENSP00000265295
ENSP00000243253	ENSP00000267750
ENSP00000243253	ENSP00000273067
ENSP00000243253	ENSP00000274140
ENSP00000243253	ENSP00000287936
ENSP00000243253	ENSP00000290649
ENSP00000243253	ENSP00000295770
ENSP00000243253	ENSP00000295887
ENSP00000243253	ENSP00000295890
ENSP00000243253	ENSP00000296255
ENSP00000243253	ENSP00000297185
ENSP00000243253	ENSP00000298852
ENSP00000243253	ENSP00000299767
ENSP00000243253	ENSP00000302176
ENSP00000243253	ENSP00000304051
ENSP00000243253	ENSP00000304500
ENSP00000243253	ENSP00000306106
ENSP00000243253	ENSP00000307275
ENSP00000243253	ENSP00000309141
ENSP00000243253	ENSP00000311430
ENSP00000243253	ENSP00000311496
ENSP00000243253	ENSP00000314036
ENSP00000243253	ENSP00000317087
ENSP00000243253	ENSP00000317331
ENSP00000243253	ENSP00000323377
ENSP00000243253	ENSP00000326514
ENSP00000243253	ENSP00000327716
ENSP00000243253	ENSP00000328023
ENSP00000243253	ENSP00000330945

ENSP00000243253	ENSP00000331268
ENSP00000243253	ENSP00000331288
ENSP00000243253	ENSP00000332681
ENSP00000243253	ENSP00000337053
ENSP00000243253	ENSP00000337688
ENSP00000243253	ENSP00000341538
ENSP00000243253	ENSP00000342181
ENSP00000243253	ENSP00000345528
ENSP00000243253	ENSP00000347495
ENSP00000243253	ENSP00000349877
ENSP00000243253	ENSP00000349887
ENSP00000243253	ENSP00000354525
ENSP00000243253	ENSP00000357998
ENSP00000243253	ENSP00000360149
ENSP00000243253	ENSP00000360644
ENSP00000243253	ENSP00000361057
ENSP00000243253	ENSP00000361845
ENSP00000243253	ENSP00000364188
ENSP00000243253	ENSP00000366395
ENSP00000243253	ENSP00000370414
ENSP00000243253	ENSP00000371888
ENSP00000243253	ENSP00000377401
ENSP00000243253	ENSP00000381863
ENSP00000243253	ENSP00000381891
ENSP00000243253	ENSP00000382767
ENSP00000243253	ENSP00000383719
ENSP00000243253	ENSP00000386717
ENSP00000243253	ENSP00000386918
ENSP00000243253	ENSP00000389103
ENSP00000243253	ENSP00000392330
ENSP00000243253	ENSP00000397435
ENSP00000243253	ENSP00000420608
ENSP00000243326	ENSP00000260947
ENSP00000243326	ENSP00000261531
ENSP00000243326	ENSP00000263468
ENSP00000243326	ENSP00000263857
ENSP00000243326	ENSP00000278919
ENSP00000243326	ENSP00000294740
ENSP00000243326	ENSP00000297439
ENSP00000243326	ENSP00000300738
ENSP00000243326	ENSP00000310356
ENSP00000243326	ENSP00000317039
ENSP00000243326	ENSP00000317992
ENSP00000243326	ENSP00000321636
ENSP00000243326	ENSP00000329466
ENSP00000243326	ENSP00000334002

ENSP00000243326	ENSP00000347232
ENSP00000243326	ENSP00000350249
ENSP00000243326	ENSP00000353073
ENSP00000243326	ENSP00000357965
ENSP00000243326	ENSP00000358778
ENSP00000243326	ENSP00000360532
ENSP00000243326	ENSP00000361949
ENSP00000243326	ENSP00000368253
ENSP00000243326	ENSP00000369716
ENSP00000243326	ENSP00000378180
ENSP00000243326	ENSP00000378338
ENSP00000243326	ENSP00000379607
ENSP00000243326	ENSP00000379839
ENSP00000243346	ENSP00000245919
ENSP00000243346	ENSP00000256857
ENSP00000243346	ENSP00000262367
ENSP00000243346	ENSP00000270708
ENSP00000243346	ENSP00000281043
ENSP00000243346	ENSP00000315768
ENSP00000243346	ENSP00000319531
ENSP00000243346	ENSP00000319597
ENSP00000243346	ENSP00000330721
ENSP00000243346	ENSP00000331201
ENSP00000243346	ENSP00000340698
ENSP00000243346	ENSP00000341208
ENSP00000243346	ENSP00000350283
ENSP00000243346	ENSP00000359978
ENSP00000243563	ENSP00000244520
ENSP00000243563	ENSP00000251289
ENSP00000243563	ENSP00000253363
ENSP00000243563	ENSP00000254193
ENSP00000243563	ENSP00000256854
ENSP00000243563	ENSP00000260129
ENSP00000243563	ENSP00000261015
ENSP00000243563	ENSP00000261973
ENSP00000243563	ENSP00000262633
ENSP00000243563	ENSP00000262803
ENSP00000243563	ENSP00000264161
ENSP00000243563	ENSP00000264233
ENSP00000243563	ENSP00000264731
ENSP00000243563	ENSP00000264951
ENSP00000243563	ENSP00000266079
ENSP00000243563	ENSP00000266735
ENSP00000243563	ENSP00000267064
ENSP00000243563	ENSP00000268482
ENSP00000243563	ENSP00000276201

ENSP00000243563	ENSP00000278780
ENSP00000243563	ENSP00000290158
ENSP00000243563	ENSP00000293406
ENSP00000243563	ENSP00000295488
ENSP00000243563	ENSP00000297591
ENSP00000243563	ENSP00000300413
ENSP00000243563	ENSP00000301764
ENSP00000243563	ENSP00000303191
ENSP00000243563	ENSP00000304350
ENSP00000243563	ENSP00000304370
ENSP00000243563	ENSP00000304903
ENSP00000243563	ENSP00000305790
ENSP00000243563	ENSP00000307863
ENSP00000243563	ENSP00000310448
ENSP00000243563	ENSP00000310723
ENSP00000243563	ENSP00000311135
ENSP00000243563	ENSP00000314311
ENSP00000243563	ENSP00000315859
ENSP00000243563	ENSP00000316879
ENSP00000243563	ENSP00000318861
ENSP00000243563	ENSP00000324122
ENSP00000243563	ENSP00000324203
ENSP00000243563	ENSP00000326411
ENSP00000243563	ENSP00000326531
ENSP00000243563	ENSP00000326806
ENSP00000243563	ENSP00000331907
ENSP00000243563	ENSP00000334148
ENSP00000243563	ENSP00000335321
ENSP00000243563	ENSP00000337405
ENSP00000243563	ENSP00000337839
ENSP00000243563	ENSP00000341243
ENSP00000243563	ENSP00000342374
ENSP00000243563	ENSP00000343557
ENSP00000243563	ENSP00000343701
ENSP00000243563	ENSP00000345096
ENSP00000243563	ENSP00000347495
ENSP00000243563	ENSP00000348010
ENSP00000243563	ENSP00000348708
ENSP00000243563	ENSP00000349748
ENSP00000243563	ENSP00000352738
ENSP00000243563	ENSP00000354522
ENSP00000243563	ENSP00000355153
ENSP00000243563	ENSP00000355922
ENSP00000243563	ENSP00000358563
ENSP00000243563	ENSP00000359454
ENSP00000243563	ENSP00000360532

ENSP00000243563	ENSP00000360876
ENSP00000243563	ENSP00000361949
ENSP00000243563	ENSP00000362110
ENSP00000243563	ENSP00000362592
ENSP00000243563	ENSP00000362820
ENSP00000243563	ENSP00000362936
ENSP00000243563	ENSP00000363313
ENSP00000243563	ENSP00000363397
ENSP00000243563	ENSP00000364289
ENSP00000243563	ENSP00000365439
ENSP00000243563	ENSP00000366607
ENSP00000243563	ENSP00000368801
ENSP00000243563	ENSP00000370119
ENSP00000243563	ENSP00000370460
ENSP00000243563	ENSP00000379607
ENSP00000243563	ENSP00000380336
ENSP00000243563	ENSP00000386759
ENSP00000243563	ENSP00000387170
ENSP00000243563	ENSP00000393953
ENSP00000243563	ENSP00000400591
ENSP00000243563	ENSP00000412566
ENSP00000243706	ENSP00000260372
ENSP00000243706	ENSP00000282058
ENSP00000243706	ENSP00000369871
ENSP00000243776	ENSP00000247970
ENSP00000243776	ENSP00000359206
ENSP00000243776	ENSP00000366135
ENSP00000243776	ENSP00000398597
ENSP00000243878	ENSP00000258947
ENSP00000243878	ENSP00000264335
ENSP00000243878	ENSP00000264982
ENSP00000243878	ENSP00000300181
ENSP00000243878	ENSP00000347301
ENSP00000243896	ENSP00000359526
ENSP00000243903	ENSP00000247843
ENSP00000243903	ENSP00000251038
ENSP00000243903	ENSP00000253490
ENSP00000243903	ENSP00000262238
ENSP00000243903	ENSP00000263208
ENSP00000243903	ENSP00000265990
ENSP00000243903	ENSP00000300648
ENSP00000243903	ENSP00000301764
ENSP00000243903	ENSP00000302640
ENSP00000243903	ENSP00000306983
ENSP00000243903	ENSP00000317156
ENSP00000243903	ENSP00000332444

ENSP00000243903	ENSP00000336842
ENSP00000243903	ENSP00000336856
ENSP00000243903	ENSP00000341243
ENSP00000243903	ENSP00000349640
ENSP00000243903	ENSP00000355205
ENSP00000243903	ENSP00000361465
ENSP00000243903	ENSP00000364486
ENSP00000243903	ENSP00000369695
ENSP00000243903	ENSP00000384198
ENSP00000243903	ENSP00000391457
ENSP00000243903	ENSP00000400476
ENSP00000243903	ENSP00000420588
ENSP00000243918	ENSP00000255194
ENSP00000243918	ENSP00000255389
ENSP00000243918	ENSP00000262544
ENSP00000243918	ENSP00000263116
ENSP00000243918	ENSP00000265175
ENSP00000243918	ENSP00000273398
ENSP00000243918	ENSP00000283351
ENSP00000243918	ENSP00000286835
ENSP00000243918	ENSP00000295887
ENSP00000243918	ENSP00000299138
ENSP00000243918	ENSP00000305459
ENSP00000243918	ENSP00000308496
ENSP00000243918	ENSP00000309163
ENSP00000243918	ENSP00000309457
ENSP00000243918	ENSP00000314499
ENSP00000243918	ENSP00000314615
ENSP00000243918	ENSP00000321449
ENSP00000243918	ENSP00000327716
ENSP00000243918	ENSP00000329419
ENSP00000243918	ENSP00000336724
ENSP00000243918	ENSP00000344055
ENSP00000243918	ENSP00000347408
ENSP00000243918	ENSP00000349877
ENSP00000243918	ENSP00000349887
ENSP00000243918	ENSP00000354519
ENSP00000243918	ENSP00000355231
ENSP00000243918	ENSP00000356146
ENSP00000243918	ENSP00000360183
ENSP00000243918	ENSP00000365840
ENSP00000243918	ENSP00000376268
ENSP00000243918	ENSP00000377769
ENSP00000243918	ENSP00000397441
ENSP00000243918	ENSP00000402240
ENSP00000243924	ENSP00000252244

ENSP00000243924	ENSP00000264474
ENSP00000243924	ENSP00000301607
ENSP00000243924	ENSP00000357731
ENSP00000244007	ENSP00000251864
ENSP00000244007	ENSP00000262995
ENSP00000244007	ENSP00000263409
ENSP00000244007	ENSP00000265175
ENSP00000244007	ENSP00000265963
ENSP00000244007	ENSP00000267101
ENSP00000244007	ENSP00000274376
ENSP00000244007	ENSP00000275635
ENSP00000244007	ENSP00000286364
ENSP00000244007	ENSP00000291582
ENSP00000244007	ENSP00000295408
ENSP00000244007	ENSP00000295987
ENSP00000244007	ENSP00000296440
ENSP00000244007	ENSP00000296474
ENSP00000244007	ENSP00000299440
ENSP00000244007	ENSP00000301280
ENSP00000244007	ENSP00000303507
ENSP00000244007	ENSP00000307305
ENSP00000244007	ENSP00000307889
ENSP00000244007	ENSP00000308774
ENSP00000244007	ENSP00000310561
ENSP00000244007	ENSP00000313829
ENSP00000244007	ENSP00000316460
ENSP00000244007	ENSP00000320340
ENSP00000244007	ENSP00000323424
ENSP00000244007	ENSP00000327145
ENSP00000244007	ENSP00000328203
ENSP00000244007	ENSP00000330393
ENSP00000244007	ENSP00000333568
ENSP00000244007	ENSP00000339495
ENSP00000244007	ENSP00000340944
ENSP00000244007	ENSP00000341189
ENSP00000244007	ENSP00000341658
ENSP00000244007	ENSP00000341680
ENSP00000244007	ENSP00000341692
ENSP00000244007	ENSP00000342793
ENSP00000244007	ENSP00000342905
ENSP00000244007	ENSP00000345731
ENSP00000244007	ENSP00000346566
ENSP00000244007	ENSP00000346839
ENSP00000244007	ENSP00000347942
ENSP00000244007	ENSP00000347979
ENSP00000244007	ENSP00000348685

ENSP00000244007	ENSP00000350297
ENSP00000244007	ENSP00000352608
ENSP00000244007	ENSP00000353375
ENSP00000244007	ENSP00000354119
ENSP00000244007	ENSP00000354207
ENSP00000244007	ENSP00000354341
ENSP00000244007	ENSP00000355865
ENSP00000244007	ENSP00000356595
ENSP00000244007	ENSP00000357182
ENSP00000244007	ENSP00000357494
ENSP00000244007	ENSP00000358033
ENSP00000244007	ENSP00000358081
ENSP00000244007	ENSP00000358301
ENSP00000244007	ENSP00000359489
ENSP00000244007	ENSP00000359552
ENSP00000244007	ENSP00000363649
ENSP00000244007	ENSP00000363763
ENSP00000244007	ENSP00000364898
ENSP00000244007	ENSP00000364912
ENSP00000244007	ENSP00000365016
ENSP00000244007	ENSP00000365891
ENSP00000244007	ENSP00000366603
ENSP00000244007	ENSP00000366936
ENSP00000244007	ENSP00000367787
ENSP00000244007	ENSP00000367972
ENSP00000244007	ENSP00000373700
ENSP00000244007	ENSP00000373952
ENSP00000244007	ENSP00000378306
ENSP00000244007	ENSP00000378338
ENSP00000244007	ENSP00000379158
ENSP00000244007	ENSP00000379946
ENSP00000244007	ENSP00000380921
ENSP00000244007	ENSP00000384675
ENSP00000244007	ENSP00000406091
ENSP00000244051	ENSP00000256398
ENSP00000244051	ENSP00000266126
ENSP00000244051	ENSP00000273064
ENSP00000244051	ENSP00000284957
ENSP00000244051	ENSP00000286428
ENSP00000244051	ENSP00000288532
ENSP00000244051	ENSP00000297338
ENSP00000244051	ENSP00000298937
ENSP00000244051	ENSP00000304292
ENSP00000244051	ENSP00000307272
ENSP00000244051	ENSP00000320566
ENSP00000244051	ENSP00000322957

ENSP00000244051	ENSP00000325677
ENSP00000244051	ENSP00000327716
ENSP00000244051	ENSP00000340836
ENSP00000244051	ENSP00000343190
ENSP00000244051	ENSP00000350967
ENSP00000244051	ENSP00000353622
ENSP00000244051	ENSP00000355890
ENSP00000244051	ENSP00000356405
ENSP00000244051	ENSP00000359939
ENSP00000244051	ENSP00000360676
ENSP00000244051	ENSP00000361642
ENSP00000244051	ENSP00000363205
ENSP00000244051	ENSP00000363779
ENSP00000244051	ENSP00000377665
ENSP00000244051	ENSP00000380982
ENSP00000244051	ENSP00000388320
ENSP00000244051	ENSP00000394869
ENSP00000244051	ENSP00000398597
ENSP00000244051	ENSP00000412922
ENSP00000244174	ENSP00000274520
ENSP00000244174	ENSP00000327647
ENSP00000244204	ENSP00000263925
ENSP00000244204	ENSP00000264029
ENSP00000244204	ENSP00000276590
ENSP00000244204	ENSP00000296490
ENSP00000244204	ENSP00000301329
ENSP00000244204	ENSP00000355812
ENSP00000244204	ENSP00000370410
ENSP00000244204	ENSP00000373566
ENSP00000244204	ENSP00000373715
ENSP00000244217	ENSP00000270776
ENSP00000244217	ENSP00000336927
ENSP00000244217	ENSP00000397009
ENSP00000244227	ENSP00000261973
ENSP00000244227	ENSP00000296702
ENSP00000244227	ENSP00000308546
ENSP00000244227	ENSP00000312981
ENSP00000244227	ENSP00000346986
ENSP00000244227	ENSP00000363313
ENSP00000244227	ENSP00000368801
ENSP00000244227	ENSP00000377262
ENSP00000244227	ENSP00000383059
ENSP00000244230	ENSP00000245539
ENSP00000244230	ENSP00000254037
ENSP00000244230	ENSP00000254803
ENSP00000244230	ENSP00000257829

ENSP00000244230	ENSP00000261637
ENSP00000244230	ENSP00000261708
ENSP00000244230	ENSP00000261741
ENSP00000244230	ENSP00000263657
ENSP00000244230	ENSP00000267425
ENSP00000244230	ENSP00000272521
ENSP00000244230	ENSP00000274606
ENSP00000244230	ENSP00000274849
ENSP00000244230	ENSP00000291576
ENSP00000244230	ENSP00000295373
ENSP00000244230	ENSP00000296792
ENSP00000244230	ENSP00000296802
ENSP00000244230	ENSP00000297579
ENSP00000244230	ENSP00000297990
ENSP00000244230	ENSP00000302896
ENSP00000244230	ENSP00000308179
ENSP00000244230	ENSP00000308332
ENSP00000244230	ENSP00000311028
ENSP00000244230	ENSP00000311135
ENSP00000244230	ENSP00000314193
ENSP00000244230	ENSP00000315674
ENSP00000244230	ENSP00000318646
ENSP00000244230	ENSP00000323302
ENSP00000244230	ENSP00000324122
ENSP00000244230	ENSP00000326981
ENSP00000244230	ENSP00000327179
ENSP00000244230	ENSP00000328854
ENSP00000244230	ENSP00000329466
ENSP00000244230	ENSP00000331907
ENSP00000244230	ENSP00000332340
ENSP00000244230	ENSP00000338703
ENSP00000244230	ENSP00000339095
ENSP00000244230	ENSP00000341885
ENSP00000244230	ENSP00000343557
ENSP00000244230	ENSP00000344393
ENSP00000244230	ENSP00000355471
ENSP00000244230	ENSP00000355541
ENSP00000244230	ENSP00000356134
ENSP00000244230	ENSP00000356520
ENSP00000244230	ENSP00000356903
ENSP00000244230	ENSP00000358812
ENSP00000244230	ENSP00000360031
ENSP00000244230	ENSP00000362105
ENSP00000244230	ENSP00000362744
ENSP00000244230	ENSP00000363313
ENSP00000244230	ENSP00000363642

ENSP00000244230	ENSP00000363746
ENSP00000244230	ENSP00000366557
ENSP00000244230	ENSP00000367439
ENSP00000244230	ENSP00000369757
ENSP00000244230	ENSP00000370589
ENSP00000244230	ENSP00000371169
ENSP00000244230	ENSP00000372695
ENSP00000244230	ENSP00000377944
ENSP00000244230	ENSP00000379888
ENSP00000244230	ENSP00000384048
ENSP00000244230	ENSP00000384302
ENSP00000244230	ENSP00000399454
ENSP00000244230	ENSP00000402869
ENSP00000244230	ENSP00000405614
ENSP00000244230	ENSP00000410396
ENSP00000244230	ENSP00000414321
ENSP00000244241	ENSP00000288167
ENSP00000244241	ENSP00000320936
ENSP00000244289	ENSP00000253339
ENSP00000244289	ENSP00000258424
ENSP00000244289	ENSP00000261813
ENSP00000244289	ENSP00000286428
ENSP00000244289	ENSP00000334188
ENSP00000244289	ENSP00000353622
ENSP00000244333	ENSP00000391490
ENSP00000244426	ENSP00000251038
ENSP00000244426	ENSP00000263228
ENSP00000244426	ENSP00000273480
ENSP00000244426	ENSP00000281543
ENSP00000244426	ENSP00000308897
ENSP00000244426	ENSP00000314004
ENSP00000244426	ENSP00000354739
ENSP00000244426	ENSP00000358147
ENSP00000244426	ENSP00000384109
ENSP00000244496	ENSP00000246802
ENSP00000244496	ENSP00000263657
ENSP00000244496	ENSP00000297579
ENSP00000244496	ENSP00000297990
ENSP00000244496	ENSP00000314348
ENSP00000244496	ENSP00000334100
ENSP00000244496	ENSP00000361092
ENSP00000244496	ENSP00000363313
ENSP00000244496	ENSP00000364320
ENSP00000244496	ENSP00000365025
ENSP00000244520	ENSP00000253108
ENSP00000244520	ENSP00000254193

ENSP00000244520	ENSP00000260129
ENSP00000244520	ENSP00000260257
ENSP00000244520	ENSP00000261973
ENSP00000244520	ENSP00000266079
ENSP00000244520	ENSP00000266735
ENSP00000244520	ENSP00000274680
ENSP00000244520	ENSP00000281828
ENSP00000244520	ENSP00000290158
ENSP00000244520	ENSP00000296702
ENSP00000244520	ENSP00000298288
ENSP00000244520	ENSP00000300413
ENSP00000244520	ENSP00000301740
ENSP00000244520	ENSP00000305958
ENSP00000244520	ENSP00000307863
ENSP00000244520	ENSP00000309166
ENSP00000244520	ENSP00000310448
ENSP00000244520	ENSP00000312753
ENSP00000244520	ENSP00000316109
ENSP00000244520	ENSP00000319169
ENSP00000244520	ENSP00000320309
ENSP00000244520	ENSP00000324203
ENSP00000244520	ENSP00000325690
ENSP00000244520	ENSP00000326261
ENSP00000244520	ENSP00000326806
ENSP00000244520	ENSP00000336712
ENSP00000244520	ENSP00000342374
ENSP00000244520	ENSP00000346986
ENSP00000244520	ENSP00000348010
ENSP00000244520	ENSP00000348107
ENSP00000244520	ENSP00000352308
ENSP00000244520	ENSP00000360876
ENSP00000244520	ENSP00000361577
ENSP00000244520	ENSP00000362936
ENSP00000244520	ENSP00000364289
ENSP00000244520	ENSP00000366607
ENSP00000244520	ENSP00000366829
ENSP00000244520	ENSP00000367498
ENSP00000244520	ENSP00000368801
ENSP00000244520	ENSP00000375863
ENSP00000244520	ENSP00000381031
ENSP00000244520	ENSP00000387170
ENSP00000244520	ENSP00000400591
ENSP00000244520	ENSP00000404121
ENSP00000244520	ENSP00000412566
ENSP00000244571	ENSP00000264705
ENSP00000244571	ENSP00000266126

ENSP00000244571	ENSP00000274473
ENSP00000244571	ENSP00000301585
ENSP00000244571	ENSP00000312042
ENSP00000244571	ENSP00000362993
ENSP00000244571	ENSP00000397598
ENSP00000244709	ENSP00000262629
ENSP00000244711	ENSP00000379607
ENSP00000244741	ENSP00000247970
ENSP00000244741	ENSP00000251968
ENSP00000244741	ENSP00000255465
ENSP00000244741	ENSP00000256442
ENSP00000244741	ENSP00000257430
ENSP00000244741	ENSP00000259469
ENSP00000244741	ENSP00000261402
ENSP00000244741	ENSP00000262320
ENSP00000244741	ENSP00000262367
ENSP00000244741	ENSP00000262643
ENSP00000244741	ENSP00000263036
ENSP00000244741	ENSP00000263201
ENSP00000244741	ENSP00000264717
ENSP00000244741	ENSP00000264731
ENSP00000244741	ENSP00000267023
ENSP00000244741	ENSP00000267163
ENSP00000244741	ENSP00000274255
ENSP00000244741	ENSP00000279281
ENSP00000244741	ENSP00000282058
ENSP00000244741	ENSP00000283033
ENSP00000244741	ENSP00000292180
ENSP00000244741	ENSP00000293362
ENSP00000244741	ENSP00000294360
ENSP00000244741	ENSP00000296930
ENSP00000244741	ENSP00000298832
ENSP00000244741	ENSP00000301634
ENSP00000244741	ENSP00000301764
ENSP00000244741	ENSP00000302111
ENSP00000244741	ENSP00000304226
ENSP00000244741	ENSP00000309103
ENSP00000244741	ENSP00000310129
ENSP00000244741	ENSP00000314949
ENSP00000244741	ENSP00000317891
ENSP00000244741	ENSP00000321203
ENSP00000244741	ENSP00000325120
ENSP00000244741	ENSP00000329357
ENSP00000244741	ENSP00000334061
ENSP00000244741	ENSP00000339527
ENSP00000244741	ENSP00000340995

ENSP00000244741	ENSP00000347733
ENSP00000244741	ENSP00000348551
ENSP00000244741	ENSP00000349543
ENSP00000244741	ENSP00000350283
ENSP00000244741	ENSP00000351908
ENSP00000244741	ENSP00000352257
ENSP00000244741	ENSP00000355759
ENSP00000244741	ENSP00000355958
ENSP00000244741	ENSP00000357748
ENSP00000244741	ENSP00000360025
ENSP00000244741	ENSP00000360532
ENSP00000244741	ENSP00000362029
ENSP00000244741	ENSP00000362608
ENSP00000244741	ENSP00000365025
ENSP00000244741	ENSP00000365439
ENSP00000244741	ENSP00000368438
ENSP00000244741	ENSP00000378180
ENSP00000244741	ENSP00000386165
ENSP00000244741	ENSP00000397530
ENSP00000244741	ENSP00000417281
ENSP00000244745	ENSP00000260130
ENSP00000244745	ENSP00000262315
ENSP00000244745	ENSP00000262367
ENSP00000244745	ENSP00000362068
ENSP00000244745	ENSP00000367406
ENSP00000244745	ENSP00000417281
ENSP00000244763	ENSP00000264883
ENSP00000244763	ENSP00000268379
ENSP00000244763	ENSP00000317331
ENSP00000244763	ENSP00000321853
ENSP00000244763	ENSP00000354876
ENSP00000244763	ENSP00000358563
ENSP00000244763	ENSP00000362255
ENSP00000244769	ENSP00000246672
ENSP00000244769	ENSP00000247087
ENSP00000244769	ENSP00000247665
ENSP00000244769	ENSP00000256854
ENSP00000244769	ENSP00000257940
ENSP00000244769	ENSP00000259632
ENSP00000244769	ENSP00000261507
ENSP00000244769	ENSP00000261523
ENSP00000244769	ENSP00000261647
ENSP00000244769	ENSP00000262375
ENSP00000244769	ENSP00000262630
ENSP00000244769	ENSP00000263205
ENSP00000244769	ENSP00000264160

ENSP00000244769	ENSP00000264335
ENSP00000244769	ENSP00000267064
ENSP00000244769	ENSP00000272367
ENSP00000244769	ENSP00000276646
ENSP00000244769	ENSP00000288098
ENSP00000244769	ENSP00000290949
ENSP00000244769	ENSP00000295888
ENSP00000244769	ENSP00000296327
ENSP00000244769	ENSP00000297788
ENSP00000244769	ENSP00000297954
ENSP00000244769	ENSP00000298852
ENSP00000244769	ENSP00000300291
ENSP00000244769	ENSP00000300417
ENSP00000244769	ENSP00000300574
ENSP00000244769	ENSP00000301887
ENSP00000244769	ENSP00000303252
ENSP00000244769	ENSP00000307023
ENSP00000244769	ENSP00000307863
ENSP00000244769	ENSP00000309117
ENSP00000244769	ENSP00000309629
ENSP00000244769	ENSP00000310561
ENSP00000244769	ENSP00000312222
ENSP00000244769	ENSP00000312624
ENSP00000244769	ENSP00000313059
ENSP00000244769	ENSP00000314733
ENSP00000244769	ENSP00000315112
ENSP00000244769	ENSP00000316842
ENSP00000244769	ENSP00000318298
ENSP00000244769	ENSP00000324422
ENSP00000244769	ENSP00000327509
ENSP00000244769	ENSP00000328103
ENSP00000244769	ENSP00000330408
ENSP00000244769	ENSP00000337926
ENSP00000244769	ENSP00000343535
ENSP00000244769	ENSP00000344609
ENSP00000244769	ENSP00000345216
ENSP00000244769	ENSP00000345793
ENSP00000244769	ENSP00000346412
ENSP00000244769	ENSP00000348069
ENSP00000244769	ENSP00000348273
ENSP00000244769	ENSP00000348551
ENSP00000244769	ENSP00000349078
ENSP00000244769	ENSP00000350078
ENSP00000244769	ENSP00000350094
ENSP00000244769	ENSP00000355028
ENSP00000244769	ENSP00000358092

ENSP00000244769	ENSP00000359444
ENSP00000244769	ENSP00000363095
ENSP00000244769	ENSP00000364912
ENSP00000244769	ENSP00000365175
ENSP00000244769	ENSP00000365747
ENSP00000244769	ENSP00000365840
ENSP00000244769	ENSP00000366843
ENSP00000244769	ENSP00000367086
ENSP00000244769	ENSP00000368698
ENSP00000244769	ENSP00000369372
ENSP00000244769	ENSP00000372445
ENSP00000244769	ENSP00000374529
ENSP00000244769	ENSP00000377862
ENSP00000244769	ENSP00000379093
ENSP00000244769	ENSP00000380271
ENSP00000244769	ENSP00000381693
ENSP00000244769	ENSP00000382815
ENSP00000244769	ENSP00000386520
ENSP00000244769	ENSP00000386759
ENSP00000244769	ENSP00000387477
ENSP00000244769	ENSP00000391723
ENSP00000244769	ENSP00000392466
ENSP00000244769	ENSP00000402935
ENSP00000244769	ENSP00000415836
ENSP00000244930	ENSP00000303070
ENSP00000245046	ENSP00000251038
ENSP00000245046	ENSP00000253457
ENSP00000245046	ENSP00000267750
ENSP00000245046	ENSP00000349887
ENSP00000245046	ENSP00000420608
ENSP00000245121	ENSP00000251968
ENSP00000245121	ENSP00000278412
ENSP00000245121	ENSP00000317468
ENSP00000245121	ENSP00000348442
ENSP00000245121	ENSP00000360412
ENSP00000245121	ENSP00000386227
ENSP00000245157	ENSP00000246062
ENSP00000245157	ENSP00000263734
ENSP00000245157	ENSP00000264499
ENSP00000245157	ENSP00000268057
ENSP00000245157	ENSP00000274353
ENSP00000245157	ENSP00000293362
ENSP00000245157	ENSP00000295240
ENSP00000245157	ENSP00000296135
ENSP00000245157	ENSP00000319062
ENSP00000245157	ENSP00000327077

ENSP00000245157	ENSP00000334100
ENSP00000245157	ENSP00000344456
ENSP00000245157	ENSP00000353701
ENSP00000245157	ENSP00000370031
ENSP00000245206	ENSP00000256398
ENSP00000245206	ENSP00000258742
ENSP00000245206	ENSP00000259607
ENSP00000245206	ENSP00000264029
ENSP00000245206	ENSP00000264705
ENSP00000245206	ENSP00000268251
ENSP00000245206	ENSP00000272902
ENSP00000245206	ENSP00000277865
ENSP00000245206	ENSP00000280346
ENSP00000245206	ENSP00000295899
ENSP00000245206	ENSP00000296490
ENSP00000245206	ENSP00000301329
ENSP00000245206	ENSP00000307900
ENSP00000245206	ENSP00000308610
ENSP00000245206	ENSP00000317159
ENSP00000245206	ENSP00000322180
ENSP00000245206	ENSP00000327070
ENSP00000245206	ENSP00000335304
ENSP00000245206	ENSP00000340019
ENSP00000245206	ENSP00000349022
ENSP00000245206	ENSP00000351947
ENSP00000245206	ENSP00000354558
ENSP00000245206	ENSP00000358413
ENSP00000245206	ENSP00000360286
ENSP00000245206	ENSP00000362463
ENSP00000245206	ENSP00000363779
ENSP00000245206	ENSP00000365775
ENSP00000245206	ENSP00000373509
ENSP00000245206	ENSP00000377527
ENSP00000245206	ENSP00000384675
ENSP00000245206	ENSP00000417190
ENSP00000245222	ENSP00000247225
ENSP00000245222	ENSP00000261596
ENSP00000245222	ENSP00000262518
ENSP00000245222	ENSP00000262554
ENSP00000245222	ENSP00000287295
ENSP00000245222	ENSP00000304226
ENSP00000245222	ENSP00000322218
ENSP00000245222	ENSP00000328079
ENSP00000245222	ENSP00000329419
ENSP00000245222	ENSP00000346155
ENSP00000245222	ENSP00000348577

ENSP00000245222	ENSP00000349959
ENSP00000245222	ENSP00000351908
ENSP00000245222	ENSP00000352219
ENSP00000245222	ENSP00000355205
ENSP00000245222	ENSP00000362298
ENSP00000245222	ENSP00000362751
ENSP00000245222	ENSP00000369860
ENSP00000245222	ENSP00000369862
ENSP00000245222	ENSP00000374213
ENSP00000245222	ENSP00000375986
ENSP00000245222	ENSP00000381932
ENSP00000245222	ENSP00000386759
ENSP00000245222	ENSP00000403397
ENSP00000245441	ENSP00000249636
ENSP00000245441	ENSP00000261700
ENSP00000245441	ENSP00000262320
ENSP00000245441	ENSP00000361027
ENSP00000245441	ENSP00000379607
ENSP00000245479	ENSP00000262315
ENSP00000245479	ENSP00000262367
ENSP00000245479	ENSP00000290158
ENSP00000245479	ENSP00000327048
ENSP00000245479	ENSP00000333188
ENSP00000245479	ENSP00000352514
ENSP00000245479	ENSP00000352522
ENSP00000245479	ENSP00000356331
ENSP00000245479	ENSP00000362068
ENSP00000245479	ENSP00000363193
ENSP00000245479	ENSP00000364912
ENSP00000245479	ENSP00000367406
ENSP00000245479	ENSP00000371711
ENSP00000245539	ENSP00000252543
ENSP00000245539	ENSP00000253099
ENSP00000245539	ENSP00000253788
ENSP00000245539	ENSP00000257829
ENSP00000245539	ENSP00000258455
ENSP00000245539	ENSP00000259469
ENSP00000245539	ENSP00000259569
ENSP00000245539	ENSP00000260102
ENSP00000245539	ENSP00000260443
ENSP00000245539	ENSP00000261015
ENSP00000245539	ENSP00000262584
ENSP00000245539	ENSP00000263629
ENSP00000245539	ENSP00000263657
ENSP00000245539	ENSP00000264515
ENSP00000245539	ENSP00000264995

ENSP00000245539	ENSP00000265100
ENSP00000245539	ENSP00000268206
ENSP00000245539	ENSP00000268661
ENSP00000245539	ENSP00000268802
ENSP00000245539	ENSP00000270625
ENSP00000245539	ENSP00000272418
ENSP00000245539	ENSP00000274606
ENSP00000245539	ENSP00000278572
ENSP00000245539	ENSP00000280557
ENSP00000245539	ENSP00000283109
ENSP00000245539	ENSP00000287038
ENSP00000245539	ENSP00000288937
ENSP00000245539	ENSP00000289371
ENSP00000245539	ENSP00000293860
ENSP00000245539	ENSP00000295491
ENSP00000245539	ENSP00000295930
ENSP00000245539	ENSP00000296674
ENSP00000245539	ENSP00000296792
ENSP00000245539	ENSP00000296802
ENSP00000245539	ENSP00000296805
ENSP00000245539	ENSP00000297908
ENSP00000245539	ENSP00000298283
ENSP00000245539	ENSP00000300151
ENSP00000245539	ENSP00000301585
ENSP00000245539	ENSP00000301729
ENSP00000245539	ENSP00000302896
ENSP00000245539	ENSP00000306548
ENSP00000245539	ENSP00000307567
ENSP00000245539	ENSP00000307889
ENSP00000245539	ENSP00000308897
ENSP00000245539	ENSP00000309334
ENSP00000245539	ENSP00000309433
ENSP00000245539	ENSP00000311028
ENSP00000245539	ENSP00000311430
ENSP00000245539	ENSP00000312042
ENSP00000245539	ENSP00000312735
ENSP00000245539	ENSP00000317376
ENSP00000245539	ENSP00000318646
ENSP00000245539	ENSP00000322419
ENSP00000245539	ENSP00000323856
ENSP00000245539	ENSP00000324122
ENSP00000245539	ENSP00000326981
ENSP00000245539	ENSP00000331019
ENSP00000245539	ENSP00000336702
ENSP00000245539	ENSP00000337127
ENSP00000245539	ENSP00000339095

ENSP00000245539	ENSP00000339795
ENSP00000245539	ENSP00000340896
ENSP00000245539	ENSP00000341692
ENSP00000245539	ENSP00000341885
ENSP00000245539	ENSP00000342889
ENSP00000245539	ENSP00000345156
ENSP00000245539	ENSP00000346015
ENSP00000245539	ENSP00000346022
ENSP00000245539	ENSP00000346027
ENSP00000245539	ENSP00000346037
ENSP00000245539	ENSP00000346050
ENSP00000245539	ENSP00000346067
ENSP00000245539	ENSP00000346453
ENSP00000245539	ENSP00000347087
ENSP00000245539	ENSP00000351596
ENSP00000245539	ENSP00000352706
ENSP00000245539	ENSP00000353575
ENSP00000245539	ENSP00000354522
ENSP00000245539	ENSP00000354525
ENSP00000245539	ENSP00000354739
ENSP00000245539	ENSP00000355471
ENSP00000245539	ENSP00000355890
ENSP00000245539	ENSP00000356134
ENSP00000245539	ENSP00000357480
ENSP00000245539	ENSP00000357748
ENSP00000245539	ENSP00000359345
ENSP00000245539	ENSP00000361076
ENSP00000245539	ENSP00000361206
ENSP00000245539	ENSP00000361465
ENSP00000245539	ENSP00000362036
ENSP00000245539	ENSP00000362208
ENSP00000245539	ENSP00000362688
ENSP00000245539	ENSP00000362744
ENSP00000245539	ENSP00000362993
ENSP00000245539	ENSP00000363018
ENSP00000245539	ENSP00000363250
ENSP00000245539	ENSP00000363308
ENSP00000245539	ENSP00000363676
ENSP00000245539	ENSP00000363944
ENSP00000245539	ENSP00000364320
ENSP00000245539	ENSP00000368790
ENSP00000245539	ENSP00000368927
ENSP00000245539	ENSP00000369757
ENSP00000245539	ENSP00000370589
ENSP00000245539	ENSP00000371236
ENSP00000245539	ENSP00000372445

ENSP00000245539	ENSP00000373404
ENSP00000245539	ENSP00000375730
ENSP00000245539	ENSP00000377640
ENSP00000245539	ENSP00000379339
ENSP00000245539	ENSP00000379888
ENSP00000245539	ENSP00000380156
ENSP00000245539	ENSP00000386717
ENSP00000245539	ENSP00000389103
ENSP00000245539	ENSP00000393241
ENSP00000245539	ENSP00000402338
ENSP00000245539	ENSP00000403175
ENSP00000245539	ENSP00000412583
ENSP00000245539	ENSP00000414321
ENSP00000245539	ENSP00000416110
ENSP00000245539	ENSP00000418661
ENSP00000245539	ENSP00000420714
ENSP00000245543	ENSP00000311202
ENSP00000245543	ENSP00000345107
ENSP00000245543	ENSP00000391774
ENSP00000245544	ENSP00000245923
ENSP00000245544	ENSP00000247970
ENSP00000245544	ENSP00000251038
ENSP00000245544	ENSP00000251074
ENSP00000245544	ENSP00000252115
ENSP00000245544	ENSP00000260327
ENSP00000245544	ENSP00000261396
ENSP00000245544	ENSP00000262077
ENSP00000245544	ENSP00000263576
ENSP00000245544	ENSP00000264883
ENSP00000245544	ENSP00000265388
ENSP00000245544	ENSP00000266079
ENSP00000245544	ENSP00000267484
ENSP00000245544	ENSP00000268482
ENSP00000245544	ENSP00000273130
ENSP00000245544	ENSP00000283195
ENSP00000245544	ENSP00000290231
ENSP00000245544	ENSP00000291688
ENSP00000245544	ENSP00000296786
ENSP00000245544	ENSP00000307525
ENSP00000245544	ENSP00000307863
ENSP00000245544	ENSP00000308622
ENSP00000245544	ENSP00000308944
ENSP00000245544	ENSP00000310590
ENSP00000245544	ENSP00000315035
ENSP00000245544	ENSP00000315379
ENSP00000245544	ENSP00000316032

ENSP00000245544	ENSP00000331817
ENSP00000245544	ENSP00000334675
ENSP00000245544	ENSP00000335321
ENSP00000245544	ENSP00000337838
ENSP00000245544	ENSP00000342262
ENSP00000245544	ENSP00000344106
ENSP00000245544	ENSP00000344460
ENSP00000245544	ENSP00000352400
ENSP00000245544	ENSP00000358478
ENSP00000245544	ENSP00000360286
ENSP00000245544	ENSP00000367721
ENSP00000245544	ENSP00000368959
ENSP00000245544	ENSP00000371155
ENSP00000245544	ENSP00000382779
ENSP00000245544	ENSP00000392601
ENSP00000245564	ENSP00000317334
ENSP00000245615	ENSP00000255389
ENSP00000245615	ENSP00000262134
ENSP00000245615	ENSP00000266095
ENSP00000245615	ENSP00000310551
ENSP00000245615	ENSP00000331288
ENSP00000245615	ENSP00000337463
ENSP00000245615	ENSP00000355963
ENSP00000245615	ENSP00000380184
ENSP00000245615	ENSP00000395863
ENSP00000245615	ENSP00000401287
ENSP00000245680	ENSP00000271375
ENSP00000245680	ENSP00000280346
ENSP00000245680	ENSP00000306106
ENSP00000245680	ENSP00000348877
ENSP00000245680	ENSP00000361057
ENSP00000245680	ENSP00000370414
ENSP00000245680	ENSP00000374467
ENSP00000245796	ENSP00000350409
ENSP00000245796	ENSP00000388872
ENSP00000245816	ENSP00000294053
ENSP00000245816	ENSP00000300107
ENSP00000245816	ENSP00000341213
ENSP00000245816	ENSP00000343392
ENSP00000245816	ENSP00000353826
ENSP00000245817	ENSP00000366729
ENSP00000245838	ENSP00000251038
ENSP00000245838	ENSP00000252115
ENSP00000245838	ENSP00000254442
ENSP00000245838	ENSP00000261531
ENSP00000245838	ENSP00000261600

ENSP00000245838	ENSP00000262030
ENSP00000245838	ENSP00000262715
ENSP00000245838	ENSP00000265990
ENSP00000245838	ENSP00000285398
ENSP00000245838	ENSP00000285873
ENSP00000245838	ENSP00000286428
ENSP00000245838	ENSP00000290158
ENSP00000245838	ENSP00000291688
ENSP00000245838	ENSP00000298281
ENSP00000245838	ENSP00000300574
ENSP00000245838	ENSP00000308944
ENSP00000245838	ENSP00000315791
ENSP00000245838	ENSP00000323858
ENSP00000245838	ENSP00000325376
ENSP00000245838	ENSP00000326531
ENSP00000245838	ENSP00000329466
ENSP00000245838	ENSP00000331817
ENSP00000245838	ENSP00000332444
ENSP00000245838	ENSP00000334675
ENSP00000245838	ENSP00000336712
ENSP00000245838	ENSP00000336752
ENSP00000245838	ENSP00000337839
ENSP00000245838	ENSP00000343701
ENSP00000245838	ENSP00000343741
ENSP00000245838	ENSP00000348010
ENSP00000245838	ENSP00000352738
ENSP00000245838	ENSP00000352820
ENSP00000245838	ENSP00000353059
ENSP00000245838	ENSP00000354522
ENSP00000245838	ENSP00000356470
ENSP00000245838	ENSP00000358478
ENSP00000245838	ENSP00000362441
ENSP00000245838	ENSP00000362900
ENSP00000245838	ENSP00000364289
ENSP00000245838	ENSP00000365811
ENSP00000245838	ENSP00000366953
ENSP00000245838	ENSP00000372023
ENSP00000245838	ENSP00000379607
ENSP00000245838	ENSP00000380969
ENSP00000245838	ENSP00000386520
ENSP00000245838	ENSP00000402515
ENSP00000245903	ENSP00000266557
ENSP00000245912	ENSP00000267169
ENSP00000245912	ENSP00000347948
ENSP00000245912	ENSP00000353332
ENSP00000245912	ENSP00000393693

ENSP00000245912	ENSP00000410481
ENSP00000245919	ENSP00000262367
ENSP00000245919	ENSP00000263036
ENSP00000245919	ENSP00000265372
ENSP00000245919	ENSP00000267101
ENSP00000245919	ENSP00000281437
ENSP00000245919	ENSP00000303315
ENSP00000245919	ENSP00000307235
ENSP00000245919	ENSP00000311005
ENSP00000245919	ENSP00000333633
ENSP00000245919	ENSP00000339449
ENSP00000245919	ENSP00000343745
ENSP00000245919	ENSP00000350359
ENSP00000245919	ENSP00000350708
ENSP00000245919	ENSP00000355866
ENSP00000245919	ENSP00000357494
ENSP00000245919	ENSP00000358854
ENSP00000245919	ENSP00000391944
ENSP00000245923	ENSP00000251775
ENSP00000245923	ENSP00000332979
ENSP00000245923	ENSP00000360644
ENSP00000245923	ENSP00000363349
ENSP00000245923	ENSP00000367910
ENSP00000245923	ENSP00000368959
ENSP00000245934	ENSP00000247970
ENSP00000245934	ENSP00000251038
ENSP00000245934	ENSP00000253048
ENSP00000245934	ENSP00000261531
ENSP00000245934	ENSP00000263956
ENSP00000245934	ENSP00000265073
ENSP00000245934	ENSP00000291386
ENSP00000245934	ENSP00000292476
ENSP00000245934	ENSP00000296328
ENSP00000245934	ENSP00000296490
ENSP00000245934	ENSP00000298281
ENSP00000245934	ENSP00000304704
ENSP00000245934	ENSP00000313953
ENSP00000245934	ENSP00000314348
ENSP00000245934	ENSP00000314949
ENSP00000245934	ENSP00000315791
ENSP00000245934	ENSP00000317872
ENSP00000245934	ENSP00000325377
ENSP00000245934	ENSP00000329466
ENSP00000245934	ENSP00000331369
ENSP00000245934	ENSP00000336752
ENSP00000245934	ENSP00000339353

ENSP00000245934	ENSP00000347184
ENSP00000245934	ENSP00000351947
ENSP00000245934	ENSP00000356405
ENSP00000245934	ENSP00000359976
ENSP00000245934	ENSP00000360532
ENSP00000245934	ENSP00000364794
ENSP00000245934	ENSP00000367406
ENSP00000245934	ENSP00000386161
ENSP00000245960	ENSP00000247970
ENSP00000245960	ENSP00000256398
ENSP00000245960	ENSP00000257789
ENSP00000245960	ENSP00000257934
ENSP00000245960	ENSP00000258418
ENSP00000245960	ENSP00000259569
ENSP00000245960	ENSP00000260363
ENSP00000245960	ENSP00000260731
ENSP00000245960	ENSP00000260810
ENSP00000245960	ENSP00000262300
ENSP00000245960	ENSP00000262367
ENSP00000245960	ENSP00000263181
ENSP00000245960	ENSP00000263208
ENSP00000245960	ENSP00000263657
ENSP00000245960	ENSP00000263708
ENSP00000245960	ENSP00000264335
ENSP00000245960	ENSP00000265148
ENSP00000245960	ENSP00000269216
ENSP00000245960	ENSP00000272065
ENSP00000245960	ENSP00000274289
ENSP00000245960	ENSP00000276014
ENSP00000245960	ENSP00000277541
ENSP00000245960	ENSP00000278616
ENSP00000245960	ENSP00000281171
ENSP00000245960	ENSP00000282549
ENSP00000245960	ENSP00000295951
ENSP00000245960	ENSP00000297338
ENSP00000245960	ENSP00000297431
ENSP00000245960	ENSP00000298048
ENSP00000245960	ENSP00000298532
ENSP00000245960	ENSP00000301364
ENSP00000245960	ENSP00000301764
ENSP00000245960	ENSP00000305790
ENSP00000245960	ENSP00000310649
ENSP00000245960	ENSP00000311005
ENSP00000245960	ENSP00000312150
ENSP00000245960	ENSP00000313420
ENSP00000245960	ENSP00000314792

ENSP00000245960	ENSP00000318884
ENSP00000245960	ENSP00000319169
ENSP00000245960	ENSP00000322957
ENSP00000245960	ENSP00000325120
ENSP00000245960	ENSP00000340944
ENSP00000245960	ENSP00000342307
ENSP00000245960	ENSP00000342381
ENSP00000245960	ENSP00000343741
ENSP00000245960	ENSP00000344456
ENSP00000245960	ENSP00000346453
ENSP00000245960	ENSP00000347087
ENSP00000245960	ENSP00000351113
ENSP00000245960	ENSP00000353344
ENSP00000245960	ENSP00000353904
ENSP00000245960	ENSP00000354558
ENSP00000245960	ENSP00000354916
ENSP00000245960	ENSP00000356319
ENSP00000245960	ENSP00000358423
ENSP00000245960	ENSP00000359206
ENSP00000245960	ENSP00000360532
ENSP00000245960	ENSP00000360621
ENSP00000245960	ENSP00000365025
ENSP00000245960	ENSP00000368976
ENSP00000245960	ENSP00000374566
ENSP00000245960	ENSP00000375986
ENSP00000245960	ENSP00000377047
ENSP00000245960	ENSP00000378306
ENSP00000245960	ENSP00000378350
ENSP00000245960	ENSP00000394794
ENSP00000245960	ENSP00000402084
ENSP00000246006	ENSP00000265023
ENSP00000246006	ENSP00000270176
ENSP00000246006	ENSP00000300574
ENSP00000246006	ENSP00000318298
ENSP00000246006	ENSP00000344666
ENSP00000246006	ENSP00000350512
ENSP00000246041	ENSP00000261558
ENSP00000246062	ENSP00000256854
ENSP00000246062	ENSP00000259477
ENSP00000246062	ENSP00000262519
ENSP00000246062	ENSP00000263208
ENSP00000246062	ENSP00000263657
ENSP00000246062	ENSP00000263857
ENSP00000246062	ENSP00000264161
ENSP00000246062	ENSP00000264380
ENSP00000246062	ENSP00000264499

ENSP00000246062	ENSP00000264705
ENSP00000246062	ENSP00000267197
ENSP00000246062	ENSP00000273398
ENSP00000246062	ENSP00000274242
ENSP00000246062	ENSP00000274680
ENSP00000246062	ENSP00000281828
ENSP00000246062	ENSP00000283109
ENSP00000246062	ENSP00000285021
ENSP00000246062	ENSP00000286428
ENSP00000246062	ENSP00000295066
ENSP00000246062	ENSP00000296273
ENSP00000246062	ENSP00000300648
ENSP00000246062	ENSP00000305682
ENSP00000246062	ENSP00000311200
ENSP00000246062	ENSP00000312778
ENSP00000246062	ENSP00000317334
ENSP00000246062	ENSP00000319062
ENSP00000246062	ENSP00000320309
ENSP00000246062	ENSP00000320648
ENSP00000246062	ENSP00000325448
ENSP00000246062	ENSP00000327070
ENSP00000246062	ENSP00000327959
ENSP00000246062	ENSP00000329558
ENSP00000246062	ENSP00000334188
ENSP00000246062	ENSP00000338093
ENSP00000246062	ENSP00000340896
ENSP00000246062	ENSP00000346022
ENSP00000246062	ENSP00000346566
ENSP00000246062	ENSP00000353098
ENSP00000246062	ENSP00000355890
ENSP00000246062	ENSP00000355958
ENSP00000246062	ENSP00000357051
ENSP00000246062	ENSP00000357887
ENSP00000246062	ENSP00000358140
ENSP00000246062	ENSP00000358854
ENSP00000246062	ENSP00000360286
ENSP00000246062	ENSP00000360412
ENSP00000246062	ENSP00000360473
ENSP00000246062	ENSP00000361287
ENSP00000246062	ENSP00000361465
ENSP00000246062	ENSP00000362576
ENSP00000246062	ENSP00000363676
ENSP00000246062	ENSP00000363734
ENSP00000246062	ENSP00000367498
ENSP00000246062	ENSP00000369757
ENSP00000246062	ENSP00000371888

ENSP00000246062	ENSP00000372694
ENSP00000246062	ENSP00000376946
ENSP00000246062	ENSP00000379908
ENSP00000246062	ENSP00000382595
ENSP00000246062	ENSP00000403852
ENSP00000246062	ENSP00000412319
ENSP00000246062	ENSP00000415678
ENSP00000246090	ENSP00000259750
ENSP00000246090	ENSP00000265881
ENSP00000246090	ENSP00000342381
ENSP00000246112	ENSP00000311825
ENSP00000246112	ENSP00000312385
ENSP00000246112	ENSP00000345156
ENSP00000246112	ENSP00000359384
ENSP00000246112	ENSP00000359392
ENSP00000246112	ENSP00000414750
ENSP00000246117	ENSP00000347948
ENSP00000246151	ENSP00000262966
ENSP00000246151	ENSP00000310129
ENSP00000246151	ENSP00000418734
ENSP00000246166	ENSP00000248673
ENSP00000246166	ENSP00000263997
ENSP00000246166	ENSP00000273398
ENSP00000246166	ENSP00000282388
ENSP00000246166	ENSP00000287936
ENSP00000246166	ENSP00000288602
ENSP00000246166	ENSP00000296509
ENSP00000246166	ENSP00000297316
ENSP00000246166	ENSP00000303423
ENSP00000246166	ENSP00000320488
ENSP00000246166	ENSP00000334061
ENSP00000246166	ENSP00000342032
ENSP00000246166	ENSP00000349078
ENSP00000246166	ENSP00000350633
ENSP00000246166	ENSP00000355922
ENSP00000246166	ENSP00000362171
ENSP00000246166	ENSP00000375986
ENSP00000246166	ENSP00000378503
ENSP00000246190	ENSP00000284981
ENSP00000246190	ENSP00000364289
ENSP00000246337	ENSP00000278616
ENSP00000246337	ENSP00000278715
ENSP00000246337	ENSP00000280557
ENSP00000246337	ENSP00000328079
ENSP00000246337	ENSP00000329419
ENSP00000246337	ENSP00000350967

ENSP00000246337	ENSP00000354125
ENSP00000246337	ENSP00000364119
ENSP00000246337	ENSP00000370258
ENSP00000246337	ENSP00000386284
ENSP00000246337	ENSP00000417229
ENSP00000246532	ENSP00000344860
ENSP00000246548	ENSP00000247970
ENSP00000246548	ENSP00000249636
ENSP00000246548	ENSP00000250113
ENSP00000246548	ENSP00000256398
ENSP00000246548	ENSP00000259050
ENSP00000246548	ENSP00000261584
ENSP00000246548	ENSP00000262133
ENSP00000246548	ENSP00000263202
ENSP00000246548	ENSP00000265990
ENSP00000246548	ENSP00000267163
ENSP00000246548	ENSP00000270225
ENSP00000246548	ENSP00000273067
ENSP00000246548	ENSP00000274140
ENSP00000246548	ENSP00000283195
ENSP00000246548	ENSP00000286428
ENSP00000246548	ENSP00000288532
ENSP00000246548	ENSP00000300738
ENSP00000246548	ENSP00000301788
ENSP00000246548	ENSP00000309141
ENSP00000246548	ENSP00000311496
ENSP00000246548	ENSP00000317087
ENSP00000246548	ENSP00000322242
ENSP00000246548	ENSP00000323050
ENSP00000246548	ENSP00000323439
ENSP00000246548	ENSP00000330343
ENSP00000246548	ENSP00000334474
ENSP00000246548	ENSP00000338413
ENSP00000246548	ENSP00000338862
ENSP00000246548	ENSP00000340278
ENSP00000246548	ENSP00000343023
ENSP00000246548	ENSP00000346566
ENSP00000246548	ENSP00000350283
ENSP00000246548	ENSP00000350708
ENSP00000246548	ENSP00000354742
ENSP00000246548	ENSP00000354957
ENSP00000246548	ENSP00000359939
ENSP00000246548	ENSP00000363779
ENSP00000246548	ENSP00000365773
ENSP00000246548	ENSP00000366434
ENSP00000246548	ENSP00000377655

ENSP00000246548	ENSP00000402527
ENSP00000246548	ENSP00000403817
ENSP00000246551	ENSP00000262629
ENSP00000246657	ENSP00000259607
ENSP00000246662	ENSP00000257430
ENSP00000246662	ENSP00000259075
ENSP00000246662	ENSP00000295897
ENSP00000246662	ENSP00000362424
ENSP00000246672	ENSP00000262854
ENSP00000246672	ENSP00000266744
ENSP00000246672	ENSP00000306100
ENSP00000246672	ENSP00000311825
ENSP00000246672	ENSP00000314420
ENSP00000246672	ENSP00000349892
ENSP00000246672	ENSP00000372459
ENSP00000246794	ENSP00000345530
ENSP00000246802	ENSP00000252037
ENSP00000246802	ENSP00000254037
ENSP00000246802	ENSP00000254940
ENSP00000246802	ENSP00000257177
ENSP00000246802	ENSP00000262265
ENSP00000246802	ENSP00000264230
ENSP00000246802	ENSP00000264670
ENSP00000246802	ENSP00000264995
ENSP00000246802	ENSP00000265245
ENSP00000246802	ENSP00000267425
ENSP00000246802	ENSP00000267430
ENSP00000246802	ENSP00000268661
ENSP00000246802	ENSP00000280350
ENSP00000246802	ENSP00000294008
ENSP00000246802	ENSP00000296411
ENSP00000246802	ENSP00000296783
ENSP00000246802	ENSP00000297990
ENSP00000246802	ENSP00000298292
ENSP00000246802	ENSP00000298746
ENSP00000246802	ENSP00000301364
ENSP00000246802	ENSP00000302886
ENSP00000246802	ENSP00000307853
ENSP00000246802	ENSP00000310520
ENSP00000246802	ENSP00000317232
ENSP00000246802	ENSP00000318646
ENSP00000246802	ENSP00000324122
ENSP00000246802	ENSP00000325312
ENSP00000246802	ENSP00000326806
ENSP00000246802	ENSP00000329466
ENSP00000246802	ENSP00000333376

ENSP00000246802	ENSP00000338573
ENSP00000246802	ENSP00000339095
ENSP00000246802	ENSP00000345530
ENSP00000246802	ENSP00000346566
ENSP00000246802	ENSP00000348596
ENSP00000246802	ENSP00000352021
ENSP00000246802	ENSP00000354040
ENSP00000246802	ENSP00000358019
ENSP00000246802	ENSP00000358563
ENSP00000246802	ENSP00000361982
ENSP00000246802	ENSP00000362153
ENSP00000246802	ENSP00000364293
ENSP00000246802	ENSP00000364543
ENSP00000246802	ENSP00000365806
ENSP00000246802	ENSP00000366135
ENSP00000246802	ENSP00000366434
ENSP00000246802	ENSP00000366519
ENSP00000246802	ENSP00000370589
ENSP00000246802	ENSP00000371201
ENSP00000246802	ENSP00000372827
ENSP00000246802	ENSP00000373090
ENSP00000246802	ENSP00000377793
ENSP00000246802	ENSP00000382392
ENSP00000246802	ENSP00000388464
ENSP00000246802	ENSP00000394290
ENSP00000246802	ENSP00000394400
ENSP00000246802	ENSP00000399530
ENSP00000246802	ENSP00000400626
ENSP00000246802	ENSP00000402338
ENSP00000246802	ENSP00000403576
ENSP00000246802	ENSP00000409074
ENSP00000246802	ENSP00000409367
ENSP00000246802	ENSP00000411162
ENSP00000246802	ENSP00000412310
ENSP00000246802	ENSP00000413152
ENSP00000246802	ENSP00000417281
ENSP00000246802	ENSP00000419740
ENSP00000246868	ENSP00000251038
ENSP00000246868	ENSP00000252115
ENSP00000246868	ENSP00000254940
ENSP00000246868	ENSP00000264995
ENSP00000246868	ENSP00000268206
ENSP00000246868	ENSP00000268661
ENSP00000246868	ENSP00000276603
ENSP00000246868	ENSP00000296805
ENSP00000246868	ENSP00000300086

ENSP00000246868	ENSP00000331817
ENSP00000246868	ENSP00000350011
ENSP00000246868	ENSP00000350249
ENSP00000246868	ENSP00000353073
ENSP00000246868	ENSP00000358812
ENSP00000246868	ENSP00000363308
ENSP00000246868	ENSP00000363559
ENSP00000246868	ENSP00000366557
ENSP00000246868	ENSP00000377496
ENSP00000246891	ENSP00000259235
ENSP00000246891	ENSP00000259750
ENSP00000246891	ENSP00000314733
ENSP00000246891	ENSP00000339353
ENSP00000246891	ENSP00000346340
ENSP00000246891	ENSP00000350512
ENSP00000246891	ENSP00000369257
ENSP00000246895	ENSP00000302021
ENSP00000246895	ENSP00000358747
ENSP00000246895	ENSP00000382485
ENSP00000246896	ENSP00000302021
ENSP00000246912	ENSP00000312834
ENSP00000246912	ENSP00000320886
ENSP00000246912	ENSP00000347592
ENSP00000246912	ENSP00000382562
ENSP00000247001	ENSP00000307863
ENSP00000247001	ENSP00000366829
ENSP00000247001	ENSP00000369218
ENSP00000247005	ENSP00000297261
ENSP00000247005	ENSP00000332353
ENSP00000247026	ENSP00000355050
ENSP00000247026	ENSP00000361616
ENSP00000247026	ENSP00000394085
ENSP00000247087	ENSP00000384490
ENSP00000247161	ENSP00000262367
ENSP00000247161	ENSP00000265354
ENSP00000247161	ENSP00000305422
ENSP00000247161	ENSP00000306245
ENSP00000247161	ENSP00000310094
ENSP00000247161	ENSP00000315702
ENSP00000247161	ENSP00000338345
ENSP00000247161	ENSP00000350283
ENSP00000247161	ENSP00000363804
ENSP00000247161	ENSP00000381793
ENSP00000247178	ENSP00000262039
ENSP00000247178	ENSP00000277746
ENSP00000247178	ENSP00000336927

ENSP00000247178	ENSP00000349205
ENSP00000247178	ENSP00000355231
ENSP00000247191	ENSP00000262294
ENSP00000247191	ENSP00000266087
ENSP00000247191	ENSP00000359206
ENSP00000247219	ENSP00000247843
ENSP00000247219	ENSP00000250615
ENSP00000247219	ENSP00000251089
ENSP00000247219	ENSP00000254900
ENSP00000247219	ENSP00000259569
ENSP00000247219	ENSP00000261951
ENSP00000247219	ENSP00000262608
ENSP00000247219	ENSP00000262982
ENSP00000247219	ENSP00000263121
ENSP00000247219	ENSP00000264193
ENSP00000247219	ENSP00000272645
ENSP00000247219	ENSP00000274606
ENSP00000247219	ENSP00000280614
ENSP00000247219	ENSP00000287878
ENSP00000247219	ENSP00000293406
ENSP00000247219	ENSP00000295321
ENSP00000247219	ENSP00000298852
ENSP00000247219	ENSP00000298937
ENSP00000247219	ENSP00000299767
ENSP00000247219	ENSP00000301788
ENSP00000247219	ENSP00000303088
ENSP00000247219	ENSP00000304169
ENSP00000247219	ENSP00000306983
ENSP00000247219	ENSP00000307684
ENSP00000247219	ENSP00000309142
ENSP00000247219	ENSP00000320949
ENSP00000247219	ENSP00000328690
ENSP00000247219	ENSP00000329933
ENSP00000247219	ENSP00000336702
ENSP00000247219	ENSP00000340271
ENSP00000247219	ENSP00000341805
ENSP00000247219	ENSP00000345702
ENSP00000247219	ENSP00000346022
ENSP00000247219	ENSP00000346453
ENSP00000247219	ENSP00000347345
ENSP00000247219	ENSP00000347733
ENSP00000247219	ENSP00000349213
ENSP00000247219	ENSP00000350708
ENSP00000247219	ENSP00000351575
ENSP00000247219	ENSP00000354742
ENSP00000247219	ENSP00000356405

ENSP00000247219	ENSP00000357051
ENSP00000247219	ENSP00000357602
ENSP00000247219	ENSP00000360515
ENSP00000247219	ENSP00000361465
ENSP00000247219	ENSP00000364289
ENSP00000247219	ENSP00000365025
ENSP00000247219	ENSP00000369695
ENSP00000247219	ENSP00000369716
ENSP00000247219	ENSP00000370517
ENSP00000247219	ENSP00000374280
ENSP00000247219	ENSP00000377747
ENSP00000247219	ENSP00000377865
ENSP00000247219	ENSP00000386992
ENSP00000247219	ENSP00000392617
ENSP00000247219	ENSP00000396620
ENSP00000247219	ENSP00000396937
ENSP00000247219	ENSP00000414321
ENSP00000247225	ENSP00000258052
ENSP00000247225	ENSP00000262554
ENSP00000247225	ENSP00000326869
ENSP00000247225	ENSP00000358831
ENSP00000247225	ENSP00000362298
ENSP00000247271	ENSP00000258418
ENSP00000247291	ENSP00000350854
ENSP00000247584	ENSP00000262643
ENSP00000247584	ENSP00000341243
ENSP00000247584	ENSP00000352514
ENSP00000247584	ENSP00000364912
ENSP00000247584	ENSP00000365435
ENSP00000247655	ENSP00000251038
ENSP00000247655	ENSP00000258424
ENSP00000247655	ENSP00000268379
ENSP00000247655	ENSP00000287022
ENSP00000247655	ENSP00000297564
ENSP00000247655	ENSP00000307786
ENSP00000247655	ENSP00000309565
ENSP00000247655	ENSP00000317159
ENSP00000247655	ENSP00000317780
ENSP00000247655	ENSP00000332887
ENSP00000247655	ENSP00000354499
ENSP00000247655	ENSP00000354554
ENSP00000247655	ENSP00000354876
ENSP00000247655	ENSP00000354982
ENSP00000247655	ENSP00000355572
ENSP00000247655	ENSP00000367934
ENSP00000247655	ENSP00000387262

ENSP00000247665	ENSP00000267415
ENSP00000247815	ENSP00000254719
ENSP00000247815	ENSP00000255078
ENSP00000247815	ENSP00000262794
ENSP00000247815	ENSP00000262803
ENSP00000247815	ENSP00000264926
ENSP00000247815	ENSP00000265465
ENSP00000247815	ENSP00000267430
ENSP00000247815	ENSP00000288774
ENSP00000247815	ENSP00000298139
ENSP00000247815	ENSP00000300738
ENSP00000247815	ENSP00000306561
ENSP00000247815	ENSP00000309572
ENSP00000247815	ENSP00000311873
ENSP00000247815	ENSP00000317636
ENSP00000247815	ENSP00000318977
ENSP00000247815	ENSP00000321636
ENSP00000247815	ENSP00000322180
ENSP00000247815	ENSP00000325863
ENSP00000247815	ENSP00000330343
ENSP00000247815	ENSP00000343392
ENSP00000247815	ENSP00000347232
ENSP00000247815	ENSP00000347978
ENSP00000247815	ENSP00000351284
ENSP00000247815	ENSP00000351524
ENSP00000247815	ENSP00000351885
ENSP00000247815	ENSP00000352257
ENSP00000247815	ENSP00000360817
ENSP00000247815	ENSP00000362135
ENSP00000247815	ENSP00000366135
ENSP00000247815	ENSP00000368349
ENSP00000247815	ENSP00000368438
ENSP00000247815	ENSP00000369335
ENSP00000247815	ENSP00000406490
ENSP00000247843	ENSP00000251038
ENSP00000247843	ENSP00000252996
ENSP00000247843	ENSP00000254803
ENSP00000247843	ENSP00000254900
ENSP00000247843	ENSP00000255764
ENSP00000247843	ENSP00000256379
ENSP00000247843	ENSP00000258428
ENSP00000247843	ENSP00000259569
ENSP00000247843	ENSP00000261813
ENSP00000247843	ENSP00000262518
ENSP00000247843	ENSP00000262608
ENSP00000247843	ENSP00000263062

ENSP00000247843	ENSP00000263121
ENSP00000247843	ENSP00000263228
ENSP00000247843	ENSP00000263377
ENSP00000247843	ENSP00000263795
ENSP00000247843	ENSP00000263974
ENSP00000247843	ENSP00000264183
ENSP00000247843	ENSP00000264515
ENSP00000247843	ENSP00000264926
ENSP00000247843	ENSP00000265990
ENSP00000247843	ENSP00000267064
ENSP00000247843	ENSP00000272645
ENSP00000247843	ENSP00000276072
ENSP00000247843	ENSP00000278616
ENSP00000247843	ENSP00000281043
ENSP00000247843	ENSP00000282892
ENSP00000247843	ENSP00000283882
ENSP00000247843	ENSP00000286317
ENSP00000247843	ENSP00000286428
ENSP00000247843	ENSP00000287598
ENSP00000247843	ENSP00000291688
ENSP00000247843	ENSP00000293860
ENSP00000247843	ENSP00000294168
ENSP00000247843	ENSP00000295066
ENSP00000247843	ENSP00000296509
ENSP00000247843	ENSP00000298532
ENSP00000247843	ENSP00000299424
ENSP00000247843	ENSP00000299601
ENSP00000247843	ENSP00000300107
ENSP00000247843	ENSP00000301788
ENSP00000247843	ENSP00000302478
ENSP00000247843	ENSP00000302530
ENSP00000247843	ENSP00000302955
ENSP00000247843	ENSP00000303315
ENSP00000247843	ENSP00000304370
ENSP00000247843	ENSP00000304593
ENSP00000247843	ENSP00000307684
ENSP00000247843	ENSP00000308944
ENSP00000247843	ENSP00000309555
ENSP00000247843	ENSP00000310596
ENSP00000247843	ENSP00000311083
ENSP00000247843	ENSP00000312244
ENSP00000247843	ENSP00000312697
ENSP00000247843	ENSP00000312735
ENSP00000247843	ENSP00000316114
ENSP00000247843	ENSP00000320566
ENSP00000247843	ENSP00000322180

ENSP00000247843	ENSP00000322579
ENSP00000247843	ENSP00000323720
ENSP00000247843	ENSP00000324274
ENSP00000247843	ENSP00000324444
ENSP00000247843	ENSP00000325677
ENSP00000247843	ENSP00000326806
ENSP00000247843	ENSP00000331310
ENSP00000247843	ENSP00000334148
ENSP00000247843	ENSP00000334188
ENSP00000247843	ENSP00000334280
ENSP00000247843	ENSP00000336606
ENSP00000247843	ENSP00000336842
ENSP00000247843	ENSP00000336868
ENSP00000247843	ENSP00000340271
ENSP00000247843	ENSP00000340823
ENSP00000247843	ENSP00000340896
ENSP00000247843	ENSP00000342889
ENSP00000247843	ENSP00000343741
ENSP00000247843	ENSP00000344537
ENSP00000247843	ENSP00000346453
ENSP00000247843	ENSP00000347345
ENSP00000247843	ENSP00000347602
ENSP00000247843	ENSP00000347719
ENSP00000247843	ENSP00000347733
ENSP00000247843	ENSP00000348551
ENSP00000247843	ENSP00000349049
ENSP00000247843	ENSP00000350032
ENSP00000247843	ENSP00000351250
ENSP00000247843	ENSP00000352738
ENSP00000247843	ENSP00000353622
ENSP00000247843	ENSP00000353793
ENSP00000247843	ENSP00000354633
ENSP00000247843	ENSP00000355051
ENSP00000247843	ENSP00000355205
ENSP00000247843	ENSP00000355560
ENSP00000247843	ENSP00000355720
ENSP00000247843	ENSP00000356405
ENSP00000247843	ENSP00000356989
ENSP00000247843	ENSP00000357887
ENSP00000247843	ENSP00000358478
ENSP00000247843	ENSP00000358854
ENSP00000247843	ENSP00000359290
ENSP00000247843	ENSP00000359518
ENSP00000247843	ENSP00000359531
ENSP00000247843	ENSP00000359837
ENSP00000247843	ENSP00000360473

ENSP00000247843	ENSP00000360515
ENSP00000247843	ENSP00000360532
ENSP00000247843	ENSP00000360621
ENSP00000247843	ENSP00000361310
ENSP00000247843	ENSP00000361465
ENSP00000247843	ENSP00000361998
ENSP00000247843	ENSP00000362068
ENSP00000247843	ENSP00000362166
ENSP00000247843	ENSP00000362578
ENSP00000247843	ENSP00000363779
ENSP00000247843	ENSP00000366974
ENSP00000247843	ENSP00000367406
ENSP00000247843	ENSP00000368438
ENSP00000247843	ENSP00000368565
ENSP00000247843	ENSP00000369162
ENSP00000247843	ENSP00000374213
ENSP00000247843	ENSP00000374280
ENSP00000247843	ENSP00000377265
ENSP00000247843	ENSP00000377969
ENSP00000247843	ENSP00000378350
ENSP00000247843	ENSP00000382562
ENSP00000247843	ENSP00000382688
ENSP00000247843	ENSP00000385215
ENSP00000247843	ENSP00000386759
ENSP00000247843	ENSP00000391457
ENSP00000247843	ENSP00000392617
ENSP00000247843	ENSP00000402338
ENSP00000247843	ENSP00000403852
ENSP00000247843	ENSP00000418734
ENSP00000247970	ENSP00000248673
ENSP00000247970	ENSP00000251038
ENSP00000247970	ENSP00000252037
ENSP00000247970	ENSP00000253108
ENSP00000247970	ENSP00000253329
ENSP00000247970	ENSP00000256442
ENSP00000247970	ENSP00000258062
ENSP00000247970	ENSP00000260327
ENSP00000247970	ENSP00000260753
ENSP00000247970	ENSP00000260970
ENSP00000247970	ENSP00000261308
ENSP00000247970	ENSP00000261396
ENSP00000247970	ENSP00000262043
ENSP00000247970	ENSP00000262146
ENSP00000247970	ENSP00000262300
ENSP00000247970	ENSP00000262320
ENSP00000247970	ENSP00000262519

ENSP00000247970	ENSP00000262643
ENSP00000247970	ENSP00000262765
ENSP00000247970	ENSP00000262803
ENSP00000247970	ENSP00000262854
ENSP00000247970	ENSP00000263228
ENSP00000247970	ENSP00000263390
ENSP00000247970	ENSP00000264731
ENSP00000247970	ENSP00000264883
ENSP00000247970	ENSP00000264951
ENSP00000247970	ENSP00000265354
ENSP00000247970	ENSP00000266000
ENSP00000247970	ENSP00000266070
ENSP00000247970	ENSP00000267197
ENSP00000247970	ENSP00000268058
ENSP00000247970	ENSP00000277541
ENSP00000247970	ENSP00000281708
ENSP00000247970	ENSP00000282388
ENSP00000247970	ENSP00000282441
ENSP00000247970	ENSP00000283033
ENSP00000247970	ENSP00000283195
ENSP00000247970	ENSP00000284981
ENSP00000247970	ENSP00000285968
ENSP00000247970	ENSP00000286091
ENSP00000247970	ENSP00000286835
ENSP00000247970	ENSP00000288602
ENSP00000247970	ENSP00000290130
ENSP00000247970	ENSP00000291386
ENSP00000247970	ENSP00000293288
ENSP00000247970	ENSP00000294008
ENSP00000247970	ENSP00000296666
ENSP00000247970	ENSP00000297591
ENSP00000247970	ENSP00000298281
ENSP00000247970	ENSP00000299543
ENSP00000247970	ENSP00000300181
ENSP00000247970	ENSP00000300651
ENSP00000247970	ENSP00000301740
ENSP00000247970	ENSP00000301764
ENSP00000247970	ENSP00000302530
ENSP00000247970	ENSP00000304350
ENSP00000247970	ENSP00000304593
ENSP00000247970	ENSP00000305503
ENSP00000247970	ENSP00000307863
ENSP00000247970	ENSP00000310668
ENSP00000247970	ENSP00000311430
ENSP00000247970	ENSP00000312735
ENSP00000247970	ENSP00000313391

ENSP00000247970	ENSP00000314949
ENSP00000247970	ENSP00000315859
ENSP00000247970	ENSP00000317232
ENSP00000247970	ENSP00000317891
ENSP00000247970	ENSP00000318115
ENSP00000247970	ENSP00000323050
ENSP00000247970	ENSP00000323074
ENSP00000247970	ENSP00000324274
ENSP00000247970	ENSP00000326261
ENSP00000247970	ENSP00000329029
ENSP00000247970	ENSP00000329623
ENSP00000247970	ENSP00000330343
ENSP00000247970	ENSP00000334538
ENSP00000247970	ENSP00000336868
ENSP00000247970	ENSP00000340820
ENSP00000247970	ENSP00000344456
ENSP00000247970	ENSP00000345023
ENSP00000247970	ENSP00000345530
ENSP00000247970	ENSP00000346227
ENSP00000247970	ENSP00000346634
ENSP00000247970	ENSP00000347206
ENSP00000247970	ENSP00000348551
ENSP00000247970	ENSP00000349748
ENSP00000247970	ENSP00000351926
ENSP00000247970	ENSP00000352175
ENSP00000247970	ENSP00000352257
ENSP00000247970	ENSP00000352400
ENSP00000247970	ENSP00000352738
ENSP00000247970	ENSP00000353670
ENSP00000247970	ENSP00000353920
ENSP00000247970	ENSP00000354730
ENSP00000247970	ENSP00000356146
ENSP00000247970	ENSP00000356405
ENSP00000247970	ENSP00000356694
ENSP00000247970	ENSP00000357643
ENSP00000247970	ENSP00000357907
ENSP00000247970	ENSP00000358022
ENSP00000247970	ENSP00000358997
ENSP00000247970	ENSP00000359531
ENSP00000247970	ENSP00000359988
ENSP00000247970	ENSP00000360286
ENSP00000247970	ENSP00000360944
ENSP00000247970	ENSP00000362282
ENSP00000247970	ENSP00000362608
ENSP00000247970	ENSP00000363694
ENSP00000247970	ENSP00000365025

ENSP00000247970	ENSP00000365439
ENSP00000247970	ENSP00000366135
ENSP00000247970	ENSP00000366607
ENSP00000247970	ENSP00000366863
ENSP00000247970	ENSP00000366974
ENSP00000247970	ENSP00000368927
ENSP00000247970	ENSP00000370023
ENSP00000247970	ENSP00000370150
ENSP00000247970	ENSP00000370460
ENSP00000247970	ENSP00000374529
ENSP00000247970	ENSP00000377969
ENSP00000247970	ENSP00000379228
ENSP00000247970	ENSP00000382688
ENSP00000247970	ENSP00000385746
ENSP00000247970	ENSP00000386759
ENSP00000247970	ENSP00000391774
ENSP00000247970	ENSP00000402084
ENSP00000247970	ENSP00000406293
ENSP00000247970	ENSP00000412292
ENSP00000247970	ENSP00000413152
ENSP00000247977	ENSP00000284811
ENSP00000247977	ENSP00000285021
ENSP00000247977	ENSP00000288985
ENSP00000247977	ENSP00000334675
ENSP00000247977	ENSP00000348089
ENSP00000247977	ENSP00000350708
ENSP00000247977	ENSP00000360621
ENSP00000247977	ENSP00000364270
ENSP00000247977	ENSP00000413720
ENSP00000247986	ENSP00000249373
ENSP00000247986	ENSP00000265381
ENSP00000247986	ENSP00000284981
ENSP00000247986	ENSP00000297338
ENSP00000247986	ENSP00000367408
ENSP00000247986	ENSP00000385722
ENSP00000248089	ENSP00000309555
ENSP00000248098	ENSP00000332513
ENSP00000248098	ENSP00000362306
ENSP00000248114	ENSP00000251038
ENSP00000248114	ENSP00000254616
ENSP00000248114	ENSP00000257245
ENSP00000248114	ENSP00000264515
ENSP00000248114	ENSP00000295767
ENSP00000248114	ENSP00000299259
ENSP00000248114	ENSP00000307786
ENSP00000248114	ENSP00000321239

ENSP00000248114	ENSP00000326579
ENSP00000248114	ENSP00000340896
ENSP00000248114	ENSP00000346340
ENSP00000248114	ENSP00000350512
ENSP00000248114	ENSP00000351894
ENSP00000248114	ENSP00000379042
ENSP00000248139	ENSP00000253861
ENSP00000248139	ENSP00000262306
ENSP00000248139	ENSP00000272427
ENSP00000248139	ENSP00000273480
ENSP00000248139	ENSP00000284811
ENSP00000248139	ENSP00000334100
ENSP00000248139	ENSP00000336724
ENSP00000248139	ENSP00000361162
ENSP00000248139	ENSP00000362399
ENSP00000248139	ENSP00000389934
ENSP00000248150	ENSP00000345731
ENSP00000248244	ENSP00000259075
ENSP00000248244	ENSP00000259808
ENSP00000248244	ENSP00000262213
ENSP00000248244	ENSP00000264122
ENSP00000248244	ENSP00000329411
ENSP00000248244	ENSP00000329967
ENSP00000248244	ENSP00000342755
ENSP00000248244	ENSP00000362900
ENSP00000248244	ENSP00000363089
ENSP00000248244	ENSP00000364898
ENSP00000248244	ENSP00000374552
ENSP00000248244	ENSP00000376445
ENSP00000248244	ENSP00000401980
ENSP00000248244	ENSP00000419371
ENSP00000248342	ENSP00000253108
ENSP00000248342	ENSP00000261868
ENSP00000248342	ENSP00000310040
ENSP00000248342	ENSP00000354125
ENSP00000248342	ENSP00000358140
ENSP00000248342	ENSP00000416892
ENSP00000248378	ENSP00000251038
ENSP00000248378	ENSP00000255389
ENSP00000248378	ENSP00000327716
ENSP00000248420	ENSP00000315859
ENSP00000248450	ENSP00000248566
ENSP00000248450	ENSP00000262030
ENSP00000248450	ENSP00000262966
ENSP00000248450	ENSP00000298283
ENSP00000248450	ENSP00000339145

ENSP00000248450	ENSP00000348442
ENSP00000248450	ENSP00000353246
ENSP00000248450	ENSP00000354040
ENSP00000248450	ENSP00000363559
ENSP00000248450	ENSP00000366135
ENSP00000248450	ENSP00000396937
ENSP00000248450	ENSP00000418734
ENSP00000248553	ENSP00000250237
ENSP00000248553	ENSP00000255465
ENSP00000248553	ENSP00000257940
ENSP00000248553	ENSP00000259457
ENSP00000248553	ENSP00000261531
ENSP00000248553	ENSP00000263956
ENSP00000248553	ENSP00000264689
ENSP00000248553	ENSP00000266000
ENSP00000248553	ENSP00000267812
ENSP00000248553	ENSP00000268676
ENSP00000248553	ENSP00000270223
ENSP00000248553	ENSP00000281273
ENSP00000248553	ENSP00000287022
ENSP00000248553	ENSP00000291900
ENSP00000248553	ENSP00000294008
ENSP00000248553	ENSP00000295901
ENSP00000248553	ENSP00000297185
ENSP00000248553	ENSP00000299767
ENSP00000248553	ENSP00000300022
ENSP00000248553	ENSP00000301740
ENSP00000248553	ENSP00000303434
ENSP00000248553	ENSP00000304353
ENSP00000248553	ENSP00000305790
ENSP00000248553	ENSP00000307786
ENSP00000248553	ENSP00000311505
ENSP00000248553	ENSP00000315768
ENSP00000248553	ENSP00000316879
ENSP00000248553	ENSP00000320236
ENSP00000248553	ENSP00000323635
ENSP00000248553	ENSP00000331310
ENSP00000248553	ENSP00000333568
ENSP00000248553	ENSP00000334061
ENSP00000248553	ENSP00000337340
ENSP00000248553	ENSP00000338345
ENSP00000248553	ENSP00000339479
ENSP00000248553	ENSP00000340019
ENSP00000248553	ENSP00000343526
ENSP00000248553	ENSP00000345892
ENSP00000248553	ENSP00000350633

ENSP00000248553	ENSP00000351492
ENSP00000248553	ENSP00000357980
ENSP00000248553	ENSP00000360532
ENSP00000248553	ENSP00000362869
ENSP00000248553	ENSP00000364847
ENSP00000248553	ENSP00000366800
ENSP00000248553	ENSP00000367196
ENSP00000248553	ENSP00000367319
ENSP00000248553	ENSP00000369456
ENSP00000248553	ENSP00000374455
ENSP00000248553	ENSP00000379908
ENSP00000248553	ENSP00000386165
ENSP00000248553	ENSP00000396876
ENSP00000248566	ENSP00000251074
ENSP00000248566	ENSP00000252115
ENSP00000248566	ENSP00000252622
ENSP00000248566	ENSP00000253003
ENSP00000248566	ENSP00000253861
ENSP00000248566	ENSP00000261601
ENSP00000248566	ENSP00000261712
ENSP00000248566	ENSP00000261868
ENSP00000248566	ENSP00000262193
ENSP00000248566	ENSP00000262518
ENSP00000248566	ENSP00000263576
ENSP00000248566	ENSP00000264183
ENSP00000248566	ENSP00000264515
ENSP00000248566	ENSP00000264639
ENSP00000248566	ENSP00000267425
ENSP00000248566	ENSP00000272427
ENSP00000248566	ENSP00000283351
ENSP00000248566	ENSP00000290541
ENSP00000248566	ENSP00000295901
ENSP00000248566	ENSP00000296786
ENSP00000248566	ENSP00000298852
ENSP00000248566	ENSP00000299206
ENSP00000248566	ENSP00000300291
ENSP00000248566	ENSP00000300648
ENSP00000248566	ENSP00000302160
ENSP00000248566	ENSP00000304593
ENSP00000248566	ENSP00000309474
ENSP00000248566	ENSP00000310129
ENSP00000248566	ENSP00000310260
ENSP00000248566	ENSP00000310596
ENSP00000248566	ENSP00000312244
ENSP00000248566	ENSP00000312735
ENSP00000248566	ENSP00000318374

ENSP00000248566	ENSP00000318775
ENSP00000248566	ENSP00000325677
ENSP00000248566	ENSP00000327889
ENSP00000248566	ENSP00000331310
ENSP00000248566	ENSP00000331817
ENSP00000248566	ENSP00000334188
ENSP00000248566	ENSP00000337405
ENSP00000248566	ENSP00000346022
ENSP00000248566	ENSP00000347602
ENSP00000248566	ENSP00000347997
ENSP00000248566	ENSP00000348442
ENSP00000248566	ENSP00000350283
ENSP00000248566	ENSP00000352678
ENSP00000248566	ENSP00000354883
ENSP00000248566	ENSP00000355961
ENSP00000248566	ENSP00000356405
ENSP00000248566	ENSP00000356641
ENSP00000248566	ENSP00000356989
ENSP00000248566	ENSP00000357887
ENSP00000248566	ENSP00000360116
ENSP00000248566	ENSP00000360286
ENSP00000248566	ENSP00000360473
ENSP00000248566	ENSP00000360515
ENSP00000248566	ENSP00000362110
ENSP00000248566	ENSP00000362334
ENSP00000248566	ENSP00000363018
ENSP00000248566	ENSP00000364709
ENSP00000248566	ENSP00000365837
ENSP00000248566	ENSP00000366135
ENSP00000248566	ENSP00000369497
ENSP00000248566	ENSP00000369531
ENSP00000248566	ENSP00000369897
ENSP00000248566	ENSP00000374213
ENSP00000248566	ENSP00000374280
ENSP00000248566	ENSP00000379217
ENSP00000248566	ENSP00000381064
ENSP00000248566	ENSP00000381293
ENSP00000248566	ENSP00000386212
ENSP00000248566	ENSP00000389934
ENSP00000248566	ENSP00000391944
ENSP00000248566	ENSP00000396937
ENSP00000248566	ENSP00000398208
ENSP00000248566	ENSP00000401508
ENSP00000248566	ENSP00000402240
ENSP00000248566	ENSP00000414237
ENSP00000248566	ENSP00000417132

ENSP00000248572	ENSP00000259467
ENSP00000248572	ENSP00000346127
ENSP00000248572	ENSP00000360141
ENSP00000248600	ENSP00000252934
ENSP00000248600	ENSP00000275605
ENSP00000248600	ENSP00000364188
ENSP00000248600	ENSP00000364320
ENSP00000248600	ENSP00000379339
ENSP00000248600	ENSP00000386259
ENSP00000248633	ENSP00000251968
ENSP00000248633	ENSP00000264169
ENSP00000248633	ENSP00000274140
ENSP00000248633	ENSP00000288774
ENSP00000248633	ENSP00000295030
ENSP00000248633	ENSP00000303511
ENSP00000248633	ENSP00000309141
ENSP00000248633	ENSP00000317087
ENSP00000248633	ENSP00000317468
ENSP00000248633	ENSP00000329933
ENSP00000248633	ENSP00000331106
ENSP00000248633	ENSP00000349016
ENSP00000248633	ENSP00000360412
ENSP00000248633	ENSP00000366565
ENSP00000248633	ENSP00000396704
ENSP00000248633	ENSP00000407401
ENSP00000248643	ENSP00000263857
ENSP00000248643	ENSP00000265732
ENSP00000248643	ENSP00000292478
ENSP00000248643	ENSP00000301585
ENSP00000248673	ENSP00000252108
ENSP00000248673	ENSP00000252996
ENSP00000248673	ENSP00000262300
ENSP00000248673	ENSP00000263056
ENSP00000248673	ENSP00000263121
ENSP00000248673	ENSP00000264951
ENSP00000248673	ENSP00000283882
ENSP00000248673	ENSP00000318195
ENSP00000248673	ENSP00000320503
ENSP00000248673	ENSP00000343001
ENSP00000248673	ENSP00000348708
ENSP00000248673	ENSP00000350078
ENSP00000248673	ENSP00000351596
ENSP00000248673	ENSP00000351811
ENSP00000248673	ENSP00000352400
ENSP00000248673	ENSP00000359345
ENSP00000248673	ENSP00000365756

ENSP00000248673	ENSP00000373715
ENSP00000248673	ENSP00000375986
ENSP00000248673	ENSP00000380921
ENSP00000248673	ENSP00000398597
ENSP00000248706	ENSP00000381031
ENSP00000248846	ENSP00000252936
ENSP00000248846	ENSP00000252999
ENSP00000248846	ENSP00000260383
ENSP00000248846	ENSP00000260731
ENSP00000248846	ENSP00000261435
ENSP00000248846	ENSP00000261965
ENSP00000248846	ENSP00000262300
ENSP00000248846	ENSP00000267068
ENSP00000248846	ENSP00000270861
ENSP00000248846	ENSP00000274289
ENSP00000248846	ENSP00000288976
ENSP00000248846	ENSP00000296509
ENSP00000248846	ENSP00000300584
ENSP00000248846	ENSP00000305941
ENSP00000248846	ENSP00000331831
ENSP00000248846	ENSP00000335357
ENSP00000248846	ENSP00000346566
ENSP00000248846	ENSP00000354916
ENSP00000248846	ENSP00000356405
ENSP00000248846	ENSP00000356448
ENSP00000248846	ENSP00000363734
ENSP00000248846	ENSP00000367049
ENSP00000248846	ENSP00000370517
ENSP00000248846	ENSP00000372694
ENSP00000248846	ENSP00000377470
ENSP00000248846	ENSP00000382328
ENSP00000248846	ENSP00000402084
ENSP00000248846	ENSP00000412319
ENSP00000248846	ENSP00000415678
ENSP00000248879	ENSP00000324191
ENSP00000248879	ENSP00000324444
ENSP00000248879	ENSP00000341680
ENSP00000248929	ENSP00000259469
ENSP00000248929	ENSP00000261308
ENSP00000248929	ENSP00000307387
ENSP00000248929	ENSP00000344666
ENSP00000248933	ENSP00000352516
ENSP00000248958	ENSP00000254942
ENSP00000248958	ENSP00000264065
ENSP00000248958	ENSP00000339145
ENSP00000248958	ENSP00000379500

ENSP00000248958	ENSP00000379607
ENSP00000249014	ENSP00000265960
ENSP00000249014	ENSP00000307272
ENSP00000249014	ENSP00000348455
ENSP00000249016	ENSP00000298585
ENSP00000249016	ENSP00000332225
ENSP00000249044	ENSP00000300574
ENSP00000249075	ENSP00000263409
ENSP00000249075	ENSP00000338799
ENSP00000249289	ENSP00000253413
ENSP00000249289	ENSP00000272238
ENSP00000249289	ENSP00000273398
ENSP00000249289	ENSP00000290949
ENSP00000249289	ENSP00000292114
ENSP00000249289	ENSP00000322180
ENSP00000249289	ENSP00000332247
ENSP00000249289	ENSP00000333255
ENSP00000249289	ENSP00000333873
ENSP00000249289	ENSP00000352522
ENSP00000249289	ENSP00000358777
ENSP00000249289	ENSP00000363162
ENSP00000249289	ENSP00000367697
ENSP00000249289	ENSP00000369513
ENSP00000249356	ENSP00000294053
ENSP00000249356	ENSP00000296255
ENSP00000249356	ENSP00000296702
ENSP00000249356	ENSP00000305958
ENSP00000249356	ENSP00000329558
ENSP00000249356	ENSP00000346791
ENSP00000249356	ENSP00000359305
ENSP00000249356	ENSP00000360286
ENSP00000249356	ENSP00000363162
ENSP00000249356	ENSP00000367208
ENSP00000249356	ENSP00000381003
ENSP00000249373	ENSP00000253339
ENSP00000249373	ENSP00000295709
ENSP00000249373	ENSP00000297814
ENSP00000249373	ENSP00000332353
ENSP00000249373	ENSP00000342381
ENSP00000249373	ENSP00000347294
ENSP00000249373	ENSP00000354851
ENSP00000249373	ENSP00000359206
ENSP00000249442	ENSP00000252487
ENSP00000249442	ENSP00000290913
ENSP00000249442	ENSP00000295989
ENSP00000249442	ENSP00000325562

ENSP00000249442	ENSP00000326296
ENSP00000249442	ENSP00000327959
ENSP00000249442	ENSP00000331608
ENSP00000249442	ENSP00000334538
ENSP00000249442	ENSP00000345445
ENSP00000249442	ENSP00000357360
ENSP00000249442	ENSP00000359988
ENSP00000249442	ENSP00000364946
ENSP00000249442	ENSP00000378132
ENSP00000249442	ENSP00000387262
ENSP00000249499	ENSP00000251900
ENSP00000249499	ENSP00000269385
ENSP00000249499	ENSP00000287239
ENSP00000249499	ENSP00000306894
ENSP00000249499	ENSP00000318423
ENSP00000249499	ENSP00000326296
ENSP00000249499	ENSP00000343246
ENSP00000249499	ENSP00000355013
ENSP00000249499	ENSP00000356058
ENSP00000249499	ENSP00000356737
ENSP00000249499	ENSP00000359321
ENSP00000249499	ENSP00000362588
ENSP00000249499	ENSP00000394085
ENSP00000249504	ENSP00000318423
ENSP00000249504	ENSP00000326296
ENSP00000249636	ENSP00000252137
ENSP00000249636	ENSP00000254719
ENSP00000249636	ENSP00000258428
ENSP00000249636	ENSP00000262320
ENSP00000249636	ENSP00000262367
ENSP00000249636	ENSP00000263620
ENSP00000249636	ENSP00000264709
ENSP00000249636	ENSP00000264818
ENSP00000249636	ENSP00000264926
ENSP00000249636	ENSP00000265990
ENSP00000249636	ENSP00000266971
ENSP00000249636	ENSP00000267430
ENSP00000249636	ENSP00000268058
ENSP00000249636	ENSP00000268489
ENSP00000249636	ENSP00000269468
ENSP00000249636	ENSP00000270225
ENSP00000249636	ENSP00000278616
ENSP00000249636	ENSP00000281928
ENSP00000249636	ENSP00000285021
ENSP00000249636	ENSP00000287437
ENSP00000249636	ENSP00000287820

ENSP00000249636	ENSP00000288774
ENSP00000249636	ENSP00000296273
ENSP00000249636	ENSP00000298139
ENSP00000249636	ENSP00000307900
ENSP00000249636	ENSP00000308944
ENSP00000249636	ENSP00000313420
ENSP00000249636	ENSP00000314080
ENSP00000249636	ENSP00000317636
ENSP00000249636	ENSP00000317872
ENSP00000249636	ENSP00000321636
ENSP00000249636	ENSP00000321706
ENSP00000249636	ENSP00000322242
ENSP00000249636	ENSP00000322457
ENSP00000249636	ENSP00000322570
ENSP00000249636	ENSP00000323439
ENSP00000249636	ENSP00000325120
ENSP00000249636	ENSP00000327077
ENSP00000249636	ENSP00000327197
ENSP00000249636	ENSP00000330343
ENSP00000249636	ENSP00000333275
ENSP00000249636	ENSP00000339826
ENSP00000249636	ENSP00000340305
ENSP00000249636	ENSP00000341024
ENSP00000249636	ENSP00000341189
ENSP00000249636	ENSP00000341208
ENSP00000249636	ENSP00000341382
ENSP00000249636	ENSP00000341551
ENSP00000249636	ENSP00000343392
ENSP00000249636	ENSP00000343435
ENSP00000249636	ENSP00000343741
ENSP00000249636	ENSP00000346120
ENSP00000249636	ENSP00000347232
ENSP00000249636	ENSP00000350249
ENSP00000249636	ENSP00000350283
ENSP00000249636	ENSP00000351284
ENSP00000249636	ENSP00000351697
ENSP00000249636	ENSP00000352257
ENSP00000249636	ENSP00000354340
ENSP00000249636	ENSP00000354522
ENSP00000249636	ENSP00000354558
ENSP00000249636	ENSP00000354586
ENSP00000249636	ENSP00000354742
ENSP00000249636	ENSP00000354957
ENSP00000249636	ENSP00000355185
ENSP00000249636	ENSP00000356579
ENSP00000249636	ENSP00000357965

ENSP00000249636	ENSP00000358092
ENSP00000249636	ENSP00000358478
ENSP00000249636	ENSP00000358918
ENSP00000249636	ENSP00000360147
ENSP00000249636	ENSP00000361310
ENSP00000249636	ENSP00000363095
ENSP00000249636	ENSP00000368349
ENSP00000249636	ENSP00000368438
ENSP00000249636	ENSP00000369335
ENSP00000249636	ENSP00000370150
ENSP00000249636	ENSP00000372170
ENSP00000249636	ENSP00000373060
ENSP00000249636	ENSP00000377218
ENSP00000249636	ENSP00000377655
ENSP00000249636	ENSP00000394791
ENSP00000249636	ENSP00000402515
ENSP00000249636	ENSP00000402527
ENSP00000249636	ENSP00000417281
ENSP00000249647	ENSP00000250113
ENSP00000249647	ENSP00000253861
ENSP00000249647	ENSP00000258301
ENSP00000249647	ENSP00000260818
ENSP00000249647	ENSP00000261017
ENSP00000249647	ENSP00000263233
ENSP00000249647	ENSP00000263897
ENSP00000249647	ENSP00000264157
ENSP00000249647	ENSP00000272427
ENSP00000249647	ENSP00000273398
ENSP00000249647	ENSP00000286448
ENSP00000249647	ENSP00000294179
ENSP00000249647	ENSP00000305810
ENSP00000249647	ENSP00000310521
ENSP00000249647	ENSP00000317714
ENSP00000249647	ENSP00000323377
ENSP00000249647	ENSP00000324628
ENSP00000249647	ENSP00000332326
ENSP00000249647	ENSP00000334100
ENSP00000249647	ENSP00000337501
ENSP00000249647	ENSP00000339191
ENSP00000249647	ENSP00000341681
ENSP00000249647	ENSP00000342793
ENSP00000249647	ENSP00000353564
ENSP00000249647	ENSP00000354346
ENSP00000249647	ENSP00000357674
ENSP00000249647	ENSP00000360183
ENSP00000249647	ENSP00000367197

ENSP00000249647	ENSP00000369409
ENSP00000249647	ENSP00000370719
ENSP00000249647	ENSP00000381293
ENSP00000249647	ENSP00000382177
ENSP00000249647	ENSP00000389934
ENSP00000249647	ENSP00000390783
ENSP00000249647	ENSP00000403397
ENSP00000249647	ENSP00000417748
ENSP00000249776	ENSP00000258169
ENSP00000249806	ENSP00000259512
ENSP00000249910	ENSP00000301838
ENSP00000249910	ENSP00000307684
ENSP00000249910	ENSP00000328228
ENSP00000249910	ENSP00000347979
ENSP00000249910	ENSP00000353622
ENSP00000249910	ENSP00000362441
ENSP00000249910	ENSP00000365025
ENSP00000249923	ENSP00000251038
ENSP00000249923	ENSP00000257527
ENSP00000249923	ENSP00000258062
ENSP00000249923	ENSP00000259075
ENSP00000249923	ENSP00000260102
ENSP00000249923	ENSP00000261531
ENSP00000249923	ENSP00000262215
ENSP00000249923	ENSP00000262294
ENSP00000249923	ENSP00000262812
ENSP00000249923	ENSP00000264028
ENSP00000249923	ENSP00000264705
ENSP00000249923	ENSP00000265070
ENSP00000249923	ENSP00000265849
ENSP00000249923	ENSP00000269122
ENSP00000249923	ENSP00000283195
ENSP00000249923	ENSP00000294543
ENSP00000249923	ENSP00000296255
ENSP00000249923	ENSP00000299601
ENSP00000249923	ENSP00000303279
ENSP00000249923	ENSP00000303507
ENSP00000249923	ENSP00000304592
ENSP00000249923	ENSP00000306124
ENSP00000249923	ENSP00000306275
ENSP00000249923	ENSP00000310129
ENSP00000249923	ENSP00000310521
ENSP00000249923	ENSP00000311344
ENSP00000249923	ENSP00000312735
ENSP00000249923	ENSP00000325002
ENSP00000249923	ENSP00000326699

ENSP00000249923	ENSP00000327309
ENSP00000249923	ENSP00000329419
ENSP00000249923	ENSP00000329466
ENSP00000249923	ENSP00000330945
ENSP00000249923	ENSP00000337688
ENSP00000249923	ENSP00000341243
ENSP00000249923	ENSP00000345530
ENSP00000249923	ENSP00000346206
ENSP00000249923	ENSP00000348986
ENSP00000249923	ENSP00000349970
ENSP00000249923	ENSP00000350633
ENSP00000249923	ENSP00000352257
ENSP00000249923	ENSP00000352738
ENSP00000249923	ENSP00000354347
ENSP00000249923	ENSP00000354742
ENSP00000249923	ENSP00000355890
ENSP00000249923	ENSP00000357048
ENSP00000249923	ENSP00000359000
ENSP00000249923	ENSP00000359127
ENSP00000249923	ENSP00000359380
ENSP00000249923	ENSP00000360532
ENSP00000249923	ENSP00000360798
ENSP00000249923	ENSP00000363205
ENSP00000249923	ENSP00000364794
ENSP00000249923	ENSP00000367923
ENSP00000249923	ENSP00000370607
ENSP00000249923	ENSP00000377527
ENSP00000249923	ENSP00000378529
ENSP00000249923	ENSP00000378546
ENSP00000249923	ENSP00000379228
ENSP00000249923	ENSP00000383402
ENSP00000249923	ENSP00000395701
ENSP00000249923	ENSP00000411409
ENSP00000250003	ENSP00000254035
ENSP00000250003	ENSP00000261523
ENSP00000250003	ENSP00000261531
ENSP00000250003	ENSP00000262133
ENSP00000250003	ENSP00000262367
ENSP00000250003	ENSP00000263620
ENSP00000250003	ENSP00000265354
ENSP00000250003	ENSP00000267163
ENSP00000250003	ENSP00000271555
ENSP00000250003	ENSP00000274031
ENSP00000250003	ENSP00000276326
ENSP00000250003	ENSP00000295989
ENSP00000250003	ENSP00000301788

ENSP00000250003	ENSP00000302640
ENSP00000250003	ENSP00000319169
ENSP00000250003	ENSP00000325690
ENSP00000250003	ENSP00000326391
ENSP00000250003	ENSP00000327436
ENSP00000250003	ENSP00000329357
ENSP00000250003	ENSP00000331057
ENSP00000250003	ENSP00000338983
ENSP00000250003	ENSP00000340271
ENSP00000250003	ENSP00000341551
ENSP00000250003	ENSP00000345107
ENSP00000250003	ENSP00000348551
ENSP00000250003	ENSP00000349049
ENSP00000250003	ENSP00000365877
ENSP00000250003	ENSP00000367972
ENSP00000250003	ENSP00000370968
ENSP00000250003	ENSP00000379003
ENSP00000250003	ENSP00000399968
ENSP00000250003	ENSP00000413720
ENSP00000250003	ENSP00000415151
ENSP00000250092	ENSP00000261601
ENSP00000250092	ENSP00000309474
ENSP00000250092	ENSP00000310129
ENSP00000250092	ENSP00000348163
ENSP00000250092	ENSP00000361120
ENSP00000250101	ENSP00000267415
ENSP00000250101	ENSP00000309629
ENSP00000250101	ENSP00000348986
ENSP00000250101	ENSP00000369647
ENSP00000250113	ENSP00000254250
ENSP00000250113	ENSP00000254942
ENSP00000250113	ENSP00000258947
ENSP00000250113	ENSP00000262294
ENSP00000250113	ENSP00000267484
ENSP00000250113	ENSP00000280362
ENSP00000250113	ENSP00000282326
ENSP00000250113	ENSP00000283646
ENSP00000250113	ENSP00000286317
ENSP00000250113	ENSP00000293362
ENSP00000250113	ENSP00000295685
ENSP00000250113	ENSP00000296755
ENSP00000250113	ENSP00000300181
ENSP00000250113	ENSP00000300231
ENSP00000250113	ENSP00000305785
ENSP00000250113	ENSP00000306788
ENSP00000250113	ENSP00000312318

ENSP00000250113	ENSP00000319918
ENSP00000250113	ENSP00000323050
ENSP00000250113	ENSP00000324191
ENSP00000250113	ENSP00000324444
ENSP00000250113	ENSP00000326767
ENSP00000250113	ENSP00000329466
ENSP00000250113	ENSP00000329967
ENSP00000250113	ENSP00000337838
ENSP00000250113	ENSP00000339479
ENSP00000250113	ENSP00000339845
ENSP00000250113	ENSP00000343129
ENSP00000250113	ENSP00000343745
ENSP00000250113	ENSP00000344106
ENSP00000250113	ENSP00000344460
ENSP00000250113	ENSP00000346120
ENSP00000250113	ENSP00000349640
ENSP00000250113	ENSP00000354777
ENSP00000250113	ENSP00000359345
ENSP00000250113	ENSP00000359674
ENSP00000250113	ENSP00000359740
ENSP00000250113	ENSP00000359988
ENSP00000250113	ENSP00000362166
ENSP00000250113	ENSP00000363676
ENSP00000250113	ENSP00000365550
ENSP00000250113	ENSP00000365637
ENSP00000250113	ENSP00000368402
ENSP00000250113	ENSP00000374390
ENSP00000250113	ENSP00000381031
ENSP00000250113	ENSP00000381795
ENSP00000250113	ENSP00000382595
ENSP00000250113	ENSP00000390948
ENSP00000250113	ENSP00000409279
ENSP00000250124	ENSP00000261609
ENSP00000250124	ENSP00000262765
ENSP00000250124	ENSP00000346050
ENSP00000250124	ENSP00000357452
ENSP00000250124	ENSP00000369816
ENSP00000250237	ENSP00000281273
ENSP00000250263	ENSP00000267430
ENSP00000250263	ENSP00000339845
ENSP00000250263	ENSP00000343745
ENSP00000250263	ENSP00000417686
ENSP00000250377	ENSP00000301585
ENSP00000250377	ENSP00000312356
ENSP00000250448	ENSP00000263734
ENSP00000250448	ENSP00000267101

ENSP00000250448	ENSP00000272937
ENSP00000250448	ENSP00000297689
ENSP00000250448	ENSP00000305422
ENSP00000250448	ENSP00000354478
ENSP00000250448	ENSP00000356795
ENSP00000250448	ENSP00000386165
ENSP00000250454	ENSP00000262904
ENSP00000250454	ENSP00000313674
ENSP00000250454	ENSP00000381045
ENSP00000250498	ENSP00000261845
ENSP00000250498	ENSP00000295770
ENSP00000250498	ENSP00000296255
ENSP00000250498	ENSP00000311005
ENSP00000250498	ENSP00000319979
ENSP00000250498	ENSP00000358022
ENSP00000250498	ENSP00000360149
ENSP00000250498	ENSP00000360644
ENSP00000250498	ENSP00000364188
ENSP00000250498	ENSP00000380793
ENSP00000250535	ENSP00000256474
ENSP00000250615	ENSP00000251038
ENSP00000250615	ENSP00000254654
ENSP00000250615	ENSP00000254900
ENSP00000250615	ENSP00000262608
ENSP00000250615	ENSP00000282412
ENSP00000250615	ENSP00000287936
ENSP00000250615	ENSP00000293230
ENSP00000250615	ENSP00000306682
ENSP00000250615	ENSP00000312411
ENSP00000250615	ENSP00000372023
ENSP00000250615	ENSP00000373702
ENSP00000250894	ENSP00000273130
ENSP00000250894	ENSP00000314837
ENSP00000250894	ENSP00000341189
ENSP00000250894	ENSP00000351908
ENSP00000250894	ENSP00000363089
ENSP00000250894	ENSP00000373700
ENSP00000250894	ENSP00000382423
ENSP00000250937	ENSP00000262607
ENSP00000250937	ENSP00000335675
ENSP00000250937	ENSP00000336702
ENSP00000250937	ENSP00000361965
ENSP00000250937	ENSP00000376946
ENSP00000250937	ENSP00000379678
ENSP00000250937	ENSP00000384302
ENSP00000251020	ENSP00000256362

ENSP00000251020	ENSP00000256429
ENSP00000251020	ENSP00000269385
ENSP00000251020	ENSP00000276603
ENSP00000251020	ENSP00000333537
ENSP00000251020	ENSP00000344456
ENSP00000251020	ENSP00000351552
ENSP00000251020	ENSP00000362592
ENSP00000251020	ENSP00000368253
ENSP00000251020	ENSP00000379847
ENSP00000251038	ENSP00000251089
ENSP00000251038	ENSP00000252115
ENSP00000251038	ENSP00000252445
ENSP00000251038	ENSP00000252602
ENSP00000251038	ENSP00000252622
ENSP00000251038	ENSP00000252934
ENSP00000251038	ENSP00000252996
ENSP00000251038	ENSP00000253003
ENSP00000251038	ENSP00000253099
ENSP00000251038	ENSP00000253107
ENSP00000251038	ENSP00000253108
ENSP00000251038	ENSP00000253413
ENSP00000251038	ENSP00000253462
ENSP00000251038	ENSP00000253693
ENSP00000251038	ENSP00000253699
ENSP00000251038	ENSP00000253792
ENSP00000251038	ENSP00000253794
ENSP00000251038	ENSP00000254037
ENSP00000251038	ENSP00000254193
ENSP00000251038	ENSP00000254719
ENSP00000251038	ENSP00000254940
ENSP00000251038	ENSP00000256216
ENSP00000251038	ENSP00000256319
ENSP00000251038	ENSP00000256383
ENSP00000251038	ENSP00000256433
ENSP00000251038	ENSP00000256897
ENSP00000251038	ENSP00000257177
ENSP00000251038	ENSP00000257245
ENSP00000251038	ENSP00000257548
ENSP00000251038	ENSP00000257934
ENSP00000251038	ENSP00000258383
ENSP00000251038	ENSP00000258424
ENSP00000251038	ENSP00000258428
ENSP00000251038	ENSP00000258455
ENSP00000251038	ENSP00000258874
ENSP00000251038	ENSP00000258975
ENSP00000251038	ENSP00000259477

ENSP00000251038	ENSP00000260102
ENSP00000251038	ENSP00000260443
ENSP00000251038	ENSP00000260952
ENSP00000251038	ENSP00000260956
ENSP00000251038	ENSP00000261070
ENSP00000251038	ENSP00000261396
ENSP00000251038	ENSP00000261424
ENSP00000251038	ENSP00000261434
ENSP00000251038	ENSP00000261507
ENSP00000251038	ENSP00000261558
ENSP00000251038	ENSP00000261601
ENSP00000251038	ENSP00000261637
ENSP00000251038	ENSP00000261712
ENSP00000251038	ENSP00000261778
ENSP00000251038	ENSP00000261819
ENSP00000251038	ENSP00000261868
ENSP00000251038	ENSP00000261942
ENSP00000251038	ENSP00000261951
ENSP00000251038	ENSP00000262030
ENSP00000251038	ENSP00000262077
ENSP00000251038	ENSP00000262144
ENSP00000251038	ENSP00000262173
ENSP00000251038	ENSP00000262193
ENSP00000251038	ENSP00000262319
ENSP00000251038	ENSP00000262765
ENSP00000251038	ENSP00000262812
ENSP00000251038	ENSP00000262854
ENSP00000251038	ENSP00000262982
ENSP00000251038	ENSP00000263187
ENSP00000251038	ENSP00000263346
ENSP00000251038	ENSP00000263576
ENSP00000251038	ENSP00000263629
ENSP00000251038	ENSP00000263688
ENSP00000251038	ENSP00000263857
ENSP00000251038	ENSP00000263897
ENSP00000251038	ENSP00000263974
ENSP00000251038	ENSP00000263997
ENSP00000251038	ENSP00000264005
ENSP00000251038	ENSP00000264028
ENSP00000251038	ENSP00000264151
ENSP00000251038	ENSP00000264169
ENSP00000251038	ENSP00000264170
ENSP00000251038	ENSP00000264220
ENSP00000251038	ENSP00000264255
ENSP00000251038	ENSP00000264501
ENSP00000251038	ENSP00000264639

ENSP00000251038	ENSP00000264718
ENSP00000251038	ENSP00000264926
ENSP00000251038	ENSP00000264951
ENSP00000251038	ENSP00000264995
ENSP00000251038	ENSP00000265038
ENSP00000251038	ENSP00000265056
ENSP00000251038	ENSP00000265073
ENSP00000251038	ENSP00000265175
ENSP00000251038	ENSP00000265195
ENSP00000251038	ENSP00000265304
ENSP00000251038	ENSP00000265351
ENSP00000251038	ENSP00000265462
ENSP00000251038	ENSP00000265465
ENSP00000251038	ENSP00000265960
ENSP00000251038	ENSP00000266069
ENSP00000251038	ENSP00000266735
ENSP00000251038	ENSP00000267425
ENSP00000251038	ENSP00000267522
ENSP00000251038	ENSP00000267750
ENSP00000251038	ENSP00000268206
ENSP00000251038	ENSP00000268251
ENSP00000251038	ENSP00000268379
ENSP00000251038	ENSP00000268533
ENSP00000251038	ENSP00000268616
ENSP00000251038	ENSP00000269216
ENSP00000251038	ENSP00000269499
ENSP00000251038	ENSP00000270225
ENSP00000251038	ENSP00000270583
ENSP00000251038	ENSP00000271628
ENSP00000251038	ENSP00000272065
ENSP00000251038	ENSP00000272645
ENSP00000251038	ENSP00000272748
ENSP00000251038	ENSP00000273064
ENSP00000251038	ENSP00000273398
ENSP00000251038	ENSP00000273783
ENSP00000251038	ENSP00000273920
ENSP00000251038	ENSP00000273962
ENSP00000251038	ENSP00000274606
ENSP00000251038	ENSP00000274680
ENSP00000251038	ENSP00000276201
ENSP00000251038	ENSP00000276416
ENSP00000251038	ENSP00000278243
ENSP00000251038	ENSP00000278412
ENSP00000251038	ENSP00000278483
ENSP00000251038	ENSP00000278572
ENSP00000251038	ENSP00000278618

ENSP00000251038	ENSP00000278715
ENSP00000251038	ENSP00000279147
ENSP00000251038	ENSP00000280557
ENSP00000251038	ENSP00000280614
ENSP00000251038	ENSP00000280700
ENSP00000251038	ENSP00000282007
ENSP00000251038	ENSP00000282276
ENSP00000251038	ENSP00000282412
ENSP00000251038	ENSP00000282516
ENSP00000251038	ENSP00000282841
ENSP00000251038	ENSP00000282892
ENSP00000251038	ENSP00000282999
ENSP00000251038	ENSP00000283195
ENSP00000251038	ENSP00000283290
ENSP00000251038	ENSP00000283875
ENSP00000251038	ENSP00000283943
ENSP00000251038	ENSP00000284957
ENSP00000251038	ENSP00000285021
ENSP00000251038	ENSP00000285071
ENSP00000251038	ENSP00000285398
ENSP00000251038	ENSP00000285896
ENSP00000251038	ENSP00000285968
ENSP00000251038	ENSP00000286448
ENSP00000251038	ENSP00000286835
ENSP00000251038	ENSP00000287022
ENSP00000251038	ENSP00000287038
ENSP00000251038	ENSP00000288266
ENSP00000251038	ENSP00000288532
ENSP00000251038	ENSP00000288976
ENSP00000251038	ENSP00000289371
ENSP00000251038	ENSP00000290158
ENSP00000251038	ENSP00000290231
ENSP00000251038	ENSP00000290299
ENSP00000251038	ENSP00000290429
ENSP00000251038	ENSP00000290597
ENSP00000251038	ENSP00000290810
ENSP00000251038	ENSP00000291576
ENSP00000251038	ENSP00000291688
ENSP00000251038	ENSP00000292180
ENSP00000251038	ENSP00000292476
ENSP00000251038	ENSP00000293195
ENSP00000251038	ENSP00000293350
ENSP00000251038	ENSP00000293404
ENSP00000251038	ENSP00000293406
ENSP00000251038	ENSP00000293860
ENSP00000251038	ENSP00000293883

ENSP00000251038	ENSP00000294179
ENSP00000251038	ENSP00000294189
ENSP00000251038	ENSP00000294360
ENSP00000251038	ENSP00000294724
ENSP00000251038	ENSP00000294973
ENSP00000251038	ENSP00000295065
ENSP00000251038	ENSP00000295321
ENSP00000251038	ENSP00000295685
ENSP00000251038	ENSP00000295767
ENSP00000251038	ENSP00000295890
ENSP00000251038	ENSP00000295951
ENSP00000251038	ENSP00000296102
ENSP00000251038	ENSP00000296215
ENSP00000251038	ENSP00000296273
ENSP00000251038	ENSP00000296328
ENSP00000251038	ENSP00000296411
ENSP00000251038	ENSP00000296490
ENSP00000251038	ENSP00000296509
ENSP00000251038	ENSP00000296543
ENSP00000251038	ENSP00000296702
ENSP00000251038	ENSP00000296783
ENSP00000251038	ENSP00000298281
ENSP00000251038	ENSP00000298292
ENSP00000251038	ENSP00000298310
ENSP00000251038	ENSP00000298585
ENSP00000251038	ENSP00000298746
ENSP00000251038	ENSP00000298937
ENSP00000251038	ENSP00000299138
ENSP00000251038	ENSP00000299259
ENSP00000251038	ENSP00000299424
ENSP00000251038	ENSP00000299543
ENSP00000251038	ENSP00000299601
ENSP00000251038	ENSP00000300107
ENSP00000251038	ENSP00000300151
ENSP00000251038	ENSP00000300452
ENSP00000251038	ENSP00000300648
ENSP00000251038	ENSP00000300688
ENSP00000251038	ENSP00000301587
ENSP00000251038	ENSP00000301761
ENSP00000251038	ENSP00000301788
ENSP00000251038	ENSP00000302111
ENSP00000251038	ENSP00000302114
ENSP00000251038	ENSP00000302160
ENSP00000251038	ENSP00000302478
ENSP00000251038	ENSP00000302620
ENSP00000251038	ENSP00000302768

ENSP00000251038	ENSP00000302783
ENSP00000251038	ENSP00000302790
ENSP00000251038	ENSP00000302886
ENSP00000251038	ENSP00000303279
ENSP00000251038	ENSP00000303552
ENSP00000251038	ENSP00000303584
ENSP00000251038	ENSP00000304903
ENSP00000251038	ENSP00000305503
ENSP00000251038	ENSP00000305790
ENSP00000251038	ENSP00000305958
ENSP00000251038	ENSP00000306425
ENSP00000251038	ENSP00000306548
ENSP00000251038	ENSP00000306817
ENSP00000251038	ENSP00000307208
ENSP00000251038	ENSP00000307491
ENSP00000251038	ENSP00000307850
ENSP00000251038	ENSP00000307900
ENSP00000251038	ENSP00000307939
ENSP00000251038	ENSP00000308179
ENSP00000251038	ENSP00000308332
ENSP00000251038	ENSP00000308622
ENSP00000251038	ENSP00000308897
ENSP00000251038	ENSP00000308944
ENSP00000251038	ENSP00000309142
ENSP00000251038	ENSP00000309198
ENSP00000251038	ENSP00000309463
ENSP00000251038	ENSP00000309565
ENSP00000251038	ENSP00000309830
ENSP00000251038	ENSP00000310129
ENSP00000251038	ENSP00000310440
ENSP00000251038	ENSP00000310596
ENSP00000251038	ENSP00000310668
ENSP00000251038	ENSP00000310723
ENSP00000251038	ENSP00000311200
ENSP00000251038	ENSP00000311984
ENSP00000251038	ENSP00000312066
ENSP00000251038	ENSP00000312224
ENSP00000251038	ENSP00000312311
ENSP00000251038	ENSP00000312697
ENSP00000251038	ENSP00000312735
ENSP00000251038	ENSP00000312981
ENSP00000251038	ENSP00000313432
ENSP00000251038	ENSP00000313490
ENSP00000251038	ENSP00000313921
ENSP00000251038	ENSP00000314004
ENSP00000251038	ENSP00000314073

ENSP00000251038	ENSP00000314441
ENSP00000251038	ENSP00000315017
ENSP00000251038	ENSP00000315379
ENSP00000251038	ENSP00000315644
ENSP00000251038	ENSP00000315674
ENSP00000251038	ENSP00000315693
ENSP00000251038	ENSP00000315791
ENSP00000251038	ENSP00000316032
ENSP00000251038	ENSP00000316854
ENSP00000251038	ENSP00000316879
ENSP00000251038	ENSP00000317159
ENSP00000251038	ENSP00000317780
ENSP00000251038	ENSP00000317872
ENSP00000251038	ENSP00000318195
ENSP00000251038	ENSP00000318318
ENSP00000251038	ENSP00000318374
ENSP00000251038	ENSP00000318604
ENSP00000251038	ENSP00000318775
ENSP00000251038	ENSP00000319531
ENSP00000251038	ENSP00000320885
ENSP00000251038	ENSP00000321449
ENSP00000251038	ENSP00000321617
ENSP00000251038	ENSP00000321636
ENSP00000251038	ENSP00000321810
ENSP00000251038	ENSP00000322170
ENSP00000251038	ENSP00000322242
ENSP00000251038	ENSP00000322316
ENSP00000251038	ENSP00000322570
ENSP00000251038	ENSP00000323377
ENSP00000251038	ENSP00000323439
ENSP00000251038	ENSP00000324122
ENSP00000251038	ENSP00000324124
ENSP00000251038	ENSP00000324491
ENSP00000251038	ENSP00000325002
ENSP00000251038	ENSP00000325017
ENSP00000251038	ENSP00000325421
ENSP00000251038	ENSP00000325562
ENSP00000251038	ENSP00000325655
ENSP00000251038	ENSP00000325677
ENSP00000251038	ENSP00000325919
ENSP00000251038	ENSP00000326261
ENSP00000251038	ENSP00000326514
ENSP00000251038	ENSP00000326806
ENSP00000251038	ENSP00000326813
ENSP00000251038	ENSP00000327070
ENSP00000251038	ENSP00000327179

ENSP00000251038	ENSP00000327436
ENSP00000251038	ENSP00000327716
ENSP00000251038	ENSP00000327821
ENSP00000251038	ENSP00000328023
ENSP00000251038	ENSP00000328690
ENSP00000251038	ENSP00000329029
ENSP00000251038	ENSP00000329466
ENSP00000251038	ENSP00000329553
ENSP00000251038	ENSP00000329933
ENSP00000251038	ENSP00000330408
ENSP00000251038	ENSP00000330730
ENSP00000251038	ENSP00000331310
ENSP00000251038	ENSP00000331369
ENSP00000251038	ENSP00000331487
ENSP00000251038	ENSP00000331817
ENSP00000251038	ENSP00000331849
ENSP00000251038	ENSP00000331879
ENSP00000251038	ENSP00000332444
ENSP00000251038	ENSP00000332721
ENSP00000251038	ENSP00000333401
ENSP00000251038	ENSP00000333666
ENSP00000251038	ENSP00000333813
ENSP00000251038	ENSP00000333837
ENSP00000251038	ENSP00000334100
ENSP00000251038	ENSP00000334188
ENSP00000251038	ENSP00000335041
ENSP00000251038	ENSP00000335304
ENSP00000251038	ENSP00000335357
ENSP00000251038	ENSP00000336712
ENSP00000251038	ENSP00000337127
ENSP00000251038	ENSP00000337353
ENSP00000251038	ENSP00000337358
ENSP00000251038	ENSP00000337405
ENSP00000251038	ENSP00000337839
ENSP00000251038	ENSP00000339245
ENSP00000251038	ENSP00000339435
ENSP00000251038	ENSP00000339844
ENSP00000251038	ENSP00000339881
ENSP00000251038	ENSP00000340019
ENSP00000251038	ENSP00000340093
ENSP00000251038	ENSP00000340281
ENSP00000251038	ENSP00000340427
ENSP00000251038	ENSP00000340823
ENSP00000251038	ENSP00000341382
ENSP00000251038	ENSP00000341538
ENSP00000251038	ENSP00000341692

ENSP00000251038	ENSP00000341885
ENSP00000251038	ENSP00000342026
ENSP00000251038	ENSP00000342121
ENSP00000251038	ENSP00000342181
ENSP00000251038	ENSP00000343313
ENSP00000251038	ENSP00000343392
ENSP00000251038	ENSP00000343526
ENSP00000251038	ENSP00000343535
ENSP00000251038	ENSP00000343741
ENSP00000251038	ENSP00000343943
ENSP00000251038	ENSP00000344055
ENSP00000251038	ENSP00000344393
ENSP00000251038	ENSP00000344456
ENSP00000251038	ENSP00000344460
ENSP00000251038	ENSP00000345702
ENSP00000251038	ENSP00000346015
ENSP00000251038	ENSP00000346037
ENSP00000251038	ENSP00000346155
ENSP00000251038	ENSP00000346173
ENSP00000251038	ENSP00000346236
ENSP00000251038	ENSP00000346255
ENSP00000251038	ENSP00000347324
ENSP00000251038	ENSP00000347602
ENSP00000251038	ENSP00000347719
ENSP00000251038	ENSP00000347836
ENSP00000251038	ENSP00000347919
ENSP00000251038	ENSP00000347978
ENSP00000251038	ENSP00000348010
ENSP00000251038	ENSP00000348107
ENSP00000251038	ENSP00000348168
ENSP00000251038	ENSP00000348442
ENSP00000251038	ENSP00000348762
ENSP00000251038	ENSP00000348933
ENSP00000251038	ENSP00000349142
ENSP00000251038	ENSP00000349213
ENSP00000251038	ENSP00000349393
ENSP00000251038	ENSP00000349887
ENSP00000251038	ENSP00000349959
ENSP00000251038	ENSP00000349970
ENSP00000251038	ENSP00000350011
ENSP00000251038	ENSP00000350098
ENSP00000251038	ENSP00000350263
ENSP00000251038	ENSP00000350409
ENSP00000251038	ENSP00000350512
ENSP00000251038	ENSP00000350757
ENSP00000251038	ENSP00000351100

ENSP00000251038	ENSP00000351170
ENSP00000251038	ENSP00000351214
ENSP00000251038	ENSP00000352400
ENSP00000251038	ENSP00000353010
ENSP00000251038	ENSP00000353073
ENSP00000251038	ENSP00000353246
ENSP00000251038	ENSP00000353575
ENSP00000251038	ENSP00000353622
ENSP00000251038	ENSP00000353793
ENSP00000251038	ENSP00000353904
ENSP00000251038	ENSP00000354125
ENSP00000251038	ENSP00000354340
ENSP00000251038	ENSP00000354532
ENSP00000251038	ENSP00000354673
ENSP00000251038	ENSP00000354720
ENSP00000251038	ENSP00000354742
ENSP00000251038	ENSP00000354916
ENSP00000251038	ENSP00000355013
ENSP00000251038	ENSP00000355051
ENSP00000251038	ENSP00000355205
ENSP00000251038	ENSP00000355536
ENSP00000251038	ENSP00000355541
ENSP00000251038	ENSP00000355672
ENSP00000251038	ENSP00000355884
ENSP00000251038	ENSP00000355890
ENSP00000251038	ENSP00000356146
ENSP00000251038	ENSP00000356231
ENSP00000251038	ENSP00000356263
ENSP00000251038	ENSP00000356405
ENSP00000251038	ENSP00000356448
ENSP00000251038	ENSP00000356470
ENSP00000251038	ENSP00000356476
ENSP00000251038	ENSP00000356602
ENSP00000251038	ENSP00000356811
ENSP00000251038	ENSP00000356988
ENSP00000251038	ENSP00000356989
ENSP00000251038	ENSP00000357048
ENSP00000251038	ENSP00000357602
ENSP00000251038	ENSP00000357829
ENSP00000251038	ENSP00000357973
ENSP00000251038	ENSP00000357998
ENSP00000251038	ENSP00000358140
ENSP00000251038	ENSP00000358146
ENSP00000251038	ENSP00000358400
ENSP00000251038	ENSP00000358491
ENSP00000251038	ENSP00000358497

ENSP00000251038	ENSP00000358737
ENSP00000251038	ENSP00000358812
ENSP00000251038	ENSP00000359127
ENSP00000251038	ENSP00000359211
ENSP00000251038	ENSP00000359224
ENSP00000251038	ENSP00000359290
ENSP00000251038	ENSP00000359305
ENSP00000251038	ENSP00000359518
ENSP00000251038	ENSP00000359688
ENSP00000251038	ENSP00000359837
ENSP00000251038	ENSP00000359904
ENSP00000251038	ENSP00000360183
ENSP00000251038	ENSP00000360270
ENSP00000251038	ENSP00000360286
ENSP00000251038	ENSP00000360437
ENSP00000251038	ENSP00000360473
ENSP00000251038	ENSP00000360498
ENSP00000251038	ENSP00000360645
ENSP00000251038	ENSP00000360676
ENSP00000251038	ENSP00000360782
ENSP00000251038	ENSP00000361005
ENSP00000251038	ENSP00000361047
ENSP00000251038	ENSP00000361057
ENSP00000251038	ENSP00000361064
ENSP00000251038	ENSP00000361186
ENSP00000251038	ENSP00000361642
ENSP00000251038	ENSP00000361686
ENSP00000251038	ENSP00000361949
ENSP00000251038	ENSP00000362036
ENSP00000251038	ENSP00000362166
ENSP00000251038	ENSP00000362208
ENSP00000251038	ENSP00000362334
ENSP00000251038	ENSP00000362456
ENSP00000251038	ENSP00000362463
ENSP00000251038	ENSP00000362546
ENSP00000251038	ENSP00000362688
ENSP00000251038	ENSP00000362807
ENSP00000251038	ENSP00000362900
ENSP00000251038	ENSP00000363162
ENSP00000251038	ENSP00000363384
ENSP00000251038	ENSP00000363944
ENSP00000251038	ENSP00000364037
ENSP00000251038	ENSP00000364270
ENSP00000251038	ENSP00000364289
ENSP00000251038	ENSP00000364320
ENSP00000251038	ENSP00000364649

ENSP00000251038	ENSP00000365388
ENSP00000251038	ENSP00000365773
ENSP00000251038	ENSP00000365775
ENSP00000251038	ENSP00000365830
ENSP00000251038	ENSP00000365908
ENSP00000251038	ENSP00000366030
ENSP00000251038	ENSP00000366135
ENSP00000251038	ENSP00000366156
ENSP00000251038	ENSP00000366557
ENSP00000251038	ENSP00000366565
ENSP00000251038	ENSP00000366672
ENSP00000251038	ENSP00000367835
ENSP00000251038	ENSP00000367923
ENSP00000251038	ENSP00000367934
ENSP00000251038	ENSP00000368140
ENSP00000251038	ENSP00000368165
ENSP00000251038	ENSP00000368305
ENSP00000251038	ENSP00000368468
ENSP00000251038	ENSP00000368565
ENSP00000251038	ENSP00000368801
ENSP00000251038	ENSP00000368927
ENSP00000251038	ENSP00000368959
ENSP00000251038	ENSP00000368984
ENSP00000251038	ENSP00000369042
ENSP00000251038	ENSP00000369335
ENSP00000251038	ENSP00000369519
ENSP00000251038	ENSP00000369736
ENSP00000251038	ENSP00000369860
ENSP00000251038	ENSP00000369897
ENSP00000251038	ENSP00000370150
ENSP00000251038	ENSP00000370222
ENSP00000251038	ENSP00000370377
ENSP00000251038	ENSP00000370460
ENSP00000251038	ENSP00000370718
ENSP00000251038	ENSP00000370736
ENSP00000251038	ENSP00000370741
ENSP00000251038	ENSP00000370748
ENSP00000251038	ENSP00000370930
ENSP00000251038	ENSP00000371101
ENSP00000251038	ENSP00000371155
ENSP00000251038	ENSP00000371236
ENSP00000251038	ENSP00000371388
ENSP00000251038	ENSP00000371451
ENSP00000251038	ENSP00000371475
ENSP00000251038	ENSP00000371546
ENSP00000251038	ENSP00000371888

ENSP00000251038	ENSP00000372202
ENSP00000251038	ENSP00000372326
ENSP00000251038	ENSP00000372689
ENSP00000251038	ENSP00000373474
ENSP00000251038	ENSP00000373715
ENSP00000251038	ENSP00000373979
ENSP00000251038	ENSP00000374152
ENSP00000251038	ENSP00000374213
ENSP00000251038	ENSP00000375730
ENSP00000251038	ENSP00000375809
ENSP00000251038	ENSP00000375881
ENSP00000251038	ENSP00000377040
ENSP00000251038	ENSP00000377401
ENSP00000251038	ENSP00000377446
ENSP00000251038	ENSP00000377486
ENSP00000251038	ENSP00000377969
ENSP00000251038	ENSP00000378350
ENSP00000251038	ENSP00000378782
ENSP00000251038	ENSP00000378890
ENSP00000251038	ENSP00000379372
ENSP00000251038	ENSP00000379607
ENSP00000251038	ENSP00000379933
ENSP00000251038	ENSP00000380431
ENSP00000251038	ENSP00000380982
ENSP00000251038	ENSP00000381045
ENSP00000251038	ENSP00000381293
ENSP00000251038	ENSP00000381461
ENSP00000251038	ENSP00000381657
ENSP00000251038	ENSP00000381970
ENSP00000251038	ENSP00000382250
ENSP00000251038	ENSP00000382595
ENSP00000251038	ENSP00000382779
ENSP00000251038	ENSP00000383719
ENSP00000251038	ENSP00000383911
ENSP00000251038	ENSP00000384597
ENSP00000251038	ENSP00000386394
ENSP00000251038	ENSP00000386759
ENSP00000251038	ENSP00000387219
ENSP00000251038	ENSP00000387262
ENSP00000251038	ENSP00000387471
ENSP00000251038	ENSP00000391457
ENSP00000251038	ENSP00000391723
ENSP00000251038	ENSP00000394791
ENSP00000251038	ENSP00000394869
ENSP00000251038	ENSP00000396452
ENSP00000251038	ENSP00000396704

ENSP00000251038	ENSP00000396937
ENSP00000251038	ENSP00000397441
ENSP00000251038	ENSP00000397759
ENSP00000251038	ENSP00000398789
ENSP00000251038	ENSP00000401018
ENSP00000251038	ENSP00000401371
ENSP00000251038	ENSP00000401435
ENSP00000251038	ENSP00000402240
ENSP00000251038	ENSP00000402527
ENSP00000251038	ENSP00000402935
ENSP00000251038	ENSP00000403852
ENSP00000251038	ENSP00000406490
ENSP00000251038	ENSP00000410396
ENSP00000251038	ENSP00000410833
ENSP00000251038	ENSP00000412922
ENSP00000251038	ENSP00000413625
ENSP00000251038	ENSP00000414066
ENSP00000251038	ENSP00000414786
ENSP00000251038	ENSP00000417229
ENSP00000251038	ENSP00000418348
ENSP00000251038	ENSP00000419628
ENSP00000251038	ENSP00000420357
ENSP00000251038	ENSP00000420608
ENSP00000251038	ENSP00000420714
ENSP00000251074	ENSP00000253003
ENSP00000251074	ENSP00000256398
ENSP00000251074	ENSP00000261396
ENSP00000251074	ENSP00000261531
ENSP00000251074	ENSP00000261601
ENSP00000251074	ENSP00000282892
ENSP00000251074	ENSP00000283882
ENSP00000251074	ENSP00000298937
ENSP00000251074	ENSP00000299601
ENSP00000251074	ENSP00000304593
ENSP00000251074	ENSP00000342262
ENSP00000251074	ENSP00000357907
ENSP00000251074	ENSP00000360473
ENSP00000251074	ENSP00000360515
ENSP00000251074	ENSP00000360621
ENSP00000251074	ENSP00000363779
ENSP00000251074	ENSP00000368565
ENSP00000251074	ENSP00000386759
ENSP00000251074	ENSP00000396937
ENSP00000251076	ENSP00000251363
ENSP00000251076	ENSP00000253413
ENSP00000251076	ENSP00000254442

ENSP00000251076	ENSP00000264158
ENSP00000251076	ENSP00000272238
ENSP00000251076	ENSP00000273398
ENSP00000251076	ENSP00000277865
ENSP00000251076	ENSP00000290949
ENSP00000251076	ENSP00000303208
ENSP00000251076	ENSP00000308496
ENSP00000251076	ENSP00000336724
ENSP00000251076	ENSP00000344223
ENSP00000251076	ENSP00000349131
ENSP00000251076	ENSP00000360183
ENSP00000251076	ENSP00000363162
ENSP00000251076	ENSP00000365233
ENSP00000251076	ENSP00000365943
ENSP00000251076	ENSP00000397441
ENSP00000251089	ENSP00000263629
ENSP00000251089	ENSP00000263736
ENSP00000251089	ENSP00000276072
ENSP00000251089	ENSP00000296509
ENSP00000251089	ENSP00000319104
ENSP00000251089	ENSP00000351284
ENSP00000251089	ENSP00000354673
ENSP00000251089	ENSP00000356405
ENSP00000251089	ENSP00000360515
ENSP00000251089	ENSP00000368438
ENSP00000251089	ENSP00000369965
ENSP00000251102	ENSP00000255266
ENSP00000251102	ENSP00000259953
ENSP00000251102	ENSP00000261170
ENSP00000251102	ENSP00000272602
ENSP00000251102	ENSP00000326261
ENSP00000251102	ENSP00000397755
ENSP00000251102	ENSP00000398752
ENSP00000251102	ENSP00000409285
ENSP00000251108	ENSP00000260810
ENSP00000251108	ENSP00000265140
ENSP00000251108	ENSP00000295030
ENSP00000251108	ENSP00000364270
ENSP00000251127	ENSP00000255380
ENSP00000251127	ENSP00000311200
ENSP00000251127	ENSP00000396704
ENSP00000251166	ENSP00000262306
ENSP00000251166	ENSP00000268957
ENSP00000251166	ENSP00000284811
ENSP00000251289	ENSP00000257789
ENSP00000251289	ENSP00000260810

ENSP00000251289	ENSP00000261531
ENSP00000251289	ENSP00000265056
ENSP00000251289	ENSP00000265140
ENSP00000251289	ENSP00000268661
ENSP00000251289	ENSP00000281708
ENSP00000251289	ENSP00000282388
ENSP00000251289	ENSP00000301962
ENSP00000251289	ENSP00000302886
ENSP00000251289	ENSP00000312870
ENSP00000251289	ENSP00000323858
ENSP00000251289	ENSP00000329466
ENSP00000251289	ENSP00000338573
ENSP00000251289	ENSP00000339145
ENSP00000251289	ENSP00000339795
ENSP00000251289	ENSP00000339826
ENSP00000251289	ENSP00000346725
ENSP00000251289	ENSP00000348596
ENSP00000251289	ENSP00000351947
ENSP00000251289	ENSP00000353246
ENSP00000251289	ENSP00000354040
ENSP00000251289	ENSP00000358400
ENSP00000251289	ENSP00000360412
ENSP00000251289	ENSP00000360532
ENSP00000251289	ENSP00000360621
ENSP00000251289	ENSP00000362153
ENSP00000251289	ENSP00000363384
ENSP00000251289	ENSP00000363559
ENSP00000251289	ENSP00000363944
ENSP00000251289	ENSP00000364037
ENSP00000251289	ENSP00000364320
ENSP00000251289	ENSP00000366934
ENSP00000251289	ENSP00000368612
ENSP00000251289	ENSP00000370258
ENSP00000251289	ENSP00000372023
ENSP00000251289	ENSP00000379607
ENSP00000251296	ENSP00000295959
ENSP00000251296	ENSP00000341138
ENSP00000251296	ENSP00000403383
ENSP00000251303	ENSP00000261712
ENSP00000251303	ENSP00000262193
ENSP00000251303	ENSP00000262854
ENSP00000251303	ENSP00000268379
ENSP00000251303	ENSP00000277554
ENSP00000251303	ENSP00000281950
ENSP00000251303	ENSP00000293362
ENSP00000251303	ENSP00000294008

ENSP00000251303	ENSP00000295901
ENSP00000251303	ENSP00000298139
ENSP00000251303	ENSP00000307853
ENSP00000251303	ENSP00000317039
ENSP00000251303	ENSP00000317232
ENSP00000251303	ENSP00000317636
ENSP00000251303	ENSP00000321636
ENSP00000251303	ENSP00000321706
ENSP00000251303	ENSP00000323439
ENSP00000251303	ENSP00000326519
ENSP00000251303	ENSP00000335557
ENSP00000251303	ENSP00000339897
ENSP00000251303	ENSP00000341382
ENSP00000251303	ENSP00000347232
ENSP00000251303	ENSP00000351284
ENSP00000251303	ENSP00000354720
ENSP00000251303	ENSP00000354957
ENSP00000251303	ENSP00000358716
ENSP00000251303	ENSP00000362334
ENSP00000251303	ENSP00000365588
ENSP00000251303	ENSP00000365766
ENSP00000251303	ENSP00000373060
ENSP00000251303	ENSP00000384048
ENSP00000251303	ENSP00000387429
ENSP00000251303	ENSP00000396484
ENSP00000251303	ENSP00000404318
ENSP00000251303	ENSP00000409167
ENSP00000251303	ENSP00000415212
ENSP00000251303	ENSP00000416511
ENSP00000251363	ENSP00000262982
ENSP00000251363	ENSP00000265351
ENSP00000251363	ENSP00000337688
ENSP00000251363	ENSP00000342609
ENSP00000251363	ENSP00000356989
ENSP00000251363	ENSP00000362298
ENSP00000251496	ENSP00000260731
ENSP00000251496	ENSP00000261531
ENSP00000251496	ENSP00000263795
ENSP00000251496	ENSP00000265195
ENSP00000251496	ENSP00000296930
ENSP00000251496	ENSP00000297338
ENSP00000251496	ENSP00000308699
ENSP00000251496	ENSP00000325017
ENSP00000251496	ENSP00000341243
ENSP00000251496	ENSP00000341382
ENSP00000251496	ENSP00000354720

ENSP00000251496	ENSP00000355759
ENSP00000251496	ENSP00000357440
ENSP00000251496	ENSP00000367787
ENSP00000251588	ENSP00000283027
ENSP00000251588	ENSP00000387471
ENSP00000251607	ENSP00000254037
ENSP00000251607	ENSP00000261326
ENSP00000251607	ENSP00000265963
ENSP00000251607	ENSP00000296802
ENSP00000251607	ENSP00000312224
ENSP00000251607	ENSP00000314348
ENSP00000251607	ENSP00000327821
ENSP00000251607	ENSP00000336842
ENSP00000251607	ENSP00000339532
ENSP00000251607	ENSP00000343557
ENSP00000251607	ENSP00000352678
ENSP00000251607	ENSP00000358716
ENSP00000251607	ENSP00000366672
ENSP00000251607	ENSP00000379888
ENSP00000251607	ENSP00000393953
ENSP00000251630	ENSP00000356191
ENSP00000251630	ENSP00000363571
ENSP00000251691	ENSP00000324444
ENSP00000251775	ENSP00000251968
ENSP00000251775	ENSP00000258654
ENSP00000251775	ENSP00000259512
ENSP00000251775	ENSP00000262039
ENSP00000251775	ENSP00000265990
ENSP00000251775	ENSP00000267484
ENSP00000251775	ENSP00000269122
ENSP00000251775	ENSP00000272322
ENSP00000251775	ENSP00000272444
ENSP00000251775	ENSP00000280346
ENSP00000251775	ENSP00000281187
ENSP00000251775	ENSP00000283351
ENSP00000251775	ENSP00000288828
ENSP00000251775	ENSP00000295030
ENSP00000251775	ENSP00000305459
ENSP00000251775	ENSP00000314615
ENSP00000251775	ENSP00000330393
ENSP00000251775	ENSP00000337838
ENSP00000251775	ENSP00000343741
ENSP00000251775	ENSP00000344106
ENSP00000251775	ENSP00000347581
ENSP00000251775	ENSP00000355231
ENSP00000251775	ENSP00000358497

ENSP00000251775	ENSP00000365567
ENSP00000251775	ENSP00000368698
ENSP00000251775	ENSP00000368927
ENSP00000251775	ENSP00000377665
ENSP00000251775	ENSP00000397441
ENSP00000251809	ENSP00000252487
ENSP00000251809	ENSP00000262265
ENSP00000251809	ENSP00000280350
ENSP00000251809	ENSP00000298292
ENSP00000251822	ENSP00000253339
ENSP00000251822	ENSP00000253861
ENSP00000251822	ENSP00000322957
ENSP00000251822	ENSP00000355511
ENSP00000251822	ENSP00000361120
ENSP00000251822	ENSP00000394071
ENSP00000251864	ENSP00000255324
ENSP00000251864	ENSP00000256594
ENSP00000251864	ENSP00000263116
ENSP00000251864	ENSP00000263277
ENSP00000251864	ENSP00000269122
ENSP00000251864	ENSP00000277865
ENSP00000251864	ENSP00000282050
ENSP00000251864	ENSP00000294848
ENSP00000251864	ENSP00000296218
ENSP00000251864	ENSP00000297185
ENSP00000251864	ENSP00000299767
ENSP00000251864	ENSP00000303129
ENSP00000251864	ENSP00000306099
ENSP00000251864	ENSP00000317334
ENSP00000251864	ENSP00000325136
ENSP00000251864	ENSP00000331897
ENSP00000251864	ENSP00000340019
ENSP00000251864	ENSP00000347087
ENSP00000251864	ENSP00000348886
ENSP00000251864	ENSP00000349208
ENSP00000251864	ENSP00000349594
ENSP00000251864	ENSP00000356846
ENSP00000251864	ENSP00000357812
ENSP00000251864	ENSP00000370023
ENSP00000251864	ENSP00000378368
ENSP00000251864	ENSP00000381461
ENSP00000251864	ENSP00000384900
ENSP00000251864	ENSP00000387303
ENSP00000251864	ENSP00000393275
ENSP00000251864	ENSP00000396937
ENSP00000251864	ENSP00000404232

ENSP00000251871	ENSP00000255764
ENSP00000251871	ENSP00000256379
ENSP00000251871	ENSP00000258648
ENSP00000251871	ENSP00000260947
ENSP00000251871	ENSP00000261884
ENSP00000251871	ENSP00000263121
ENSP00000251871	ENSP00000263205
ENSP00000251871	ENSP00000263377
ENSP00000251871	ENSP00000263390
ENSP00000251871	ENSP00000264160
ENSP00000251871	ENSP00000264637
ENSP00000251871	ENSP00000264718
ENSP00000251871	ENSP00000265175
ENSP00000251871	ENSP00000265350
ENSP00000251871	ENSP00000267064
ENSP00000251871	ENSP00000268058
ENSP00000251871	ENSP00000268711
ENSP00000251871	ENSP00000277541
ENSP00000251871	ENSP00000280190
ENSP00000251871	ENSP00000281928
ENSP00000251871	ENSP00000282007
ENSP00000251871	ENSP00000282892
ENSP00000251871	ENSP00000286317
ENSP00000251871	ENSP00000290663
ENSP00000251871	ENSP00000292035
ENSP00000251871	ENSP00000293777
ENSP00000251871	ENSP00000295025
ENSP00000251871	ENSP00000297347
ENSP00000251871	ENSP00000299543
ENSP00000251871	ENSP00000300651
ENSP00000251871	ENSP00000304185
ENSP00000251871	ENSP00000308759
ENSP00000251871	ENSP00000312778
ENSP00000251871	ENSP00000316578
ENSP00000251871	ENSP00000323720
ENSP00000251871	ENSP00000325612
ENSP00000251871	ENSP00000326128
ENSP00000251871	ENSP00000326767
ENSP00000251871	ENSP00000336750
ENSP00000251871	ENSP00000337340
ENSP00000251871	ENSP00000342343
ENSP00000251871	ENSP00000344456
ENSP00000251871	ENSP00000347733
ENSP00000251871	ENSP00000348069
ENSP00000251871	ENSP00000348610
ENSP00000251871	ENSP00000350283

ENSP00000251871	ENSP00000354951
ENSP00000251871	ENSP00000355759
ENSP00000251871	ENSP00000357047
ENSP00000251871	ENSP00000357440
ENSP00000251871	ENSP00000357907
ENSP00000251871	ENSP00000360371
ENSP00000251871	ENSP00000360515
ENSP00000251871	ENSP00000362948
ENSP00000251871	ENSP00000363193
ENSP00000251871	ENSP00000381216
ENSP00000251871	ENSP00000384048
ENSP00000251871	ENSP00000391774
ENSP00000251871	ENSP00000392617
ENSP00000251871	ENSP00000418734
ENSP00000251900	ENSP00000260956
ENSP00000251900	ENSP00000269197
ENSP00000251900	ENSP00000269385
ENSP00000251900	ENSP00000272286
ENSP00000251900	ENSP00000314311
ENSP00000251900	ENSP00000343535
ENSP00000251900	ENSP00000354995
ENSP00000251900	ENSP00000364839
ENSP00000251900	ENSP00000364895
ENSP00000251900	ENSP00000366974
ENSP00000251900	ENSP00000376204
ENSP00000251900	ENSP00000384490
ENSP00000251968	ENSP00000252699
ENSP00000251968	ENSP00000253794
ENSP00000251968	ENSP00000254816
ENSP00000251968	ENSP00000260187
ENSP00000251968	ENSP00000260363
ENSP00000251968	ENSP00000261531
ENSP00000251968	ENSP00000262056
ENSP00000251968	ENSP00000262305
ENSP00000251968	ENSP00000262803
ENSP00000251968	ENSP00000262812
ENSP00000251968	ENSP00000263856
ENSP00000251968	ENSP00000264380
ENSP00000251968	ENSP00000264926
ENSP00000251968	ENSP00000265175
ENSP00000251968	ENSP00000265316
ENSP00000251968	ENSP00000265562
ENSP00000251968	ENSP00000266000
ENSP00000251968	ENSP00000267064
ENSP00000251968	ENSP00000268182
ENSP00000251968	ENSP00000280346

ENSP00000251968	ENSP00000284629
ENSP00000251968	ENSP00000286428
ENSP00000251968	ENSP00000288199
ENSP00000251968	ENSP00000290330
ENSP00000251968	ENSP00000292363
ENSP00000251968	ENSP00000295030
ENSP00000251968	ENSP00000295899
ENSP00000251968	ENSP00000297661
ENSP00000251968	ENSP00000300417
ENSP00000251968	ENSP00000301765
ENSP00000251968	ENSP00000302239
ENSP00000251968	ENSP00000302955
ENSP00000251968	ENSP00000303507
ENSP00000251968	ENSP00000303511
ENSP00000251968	ENSP00000304102
ENSP00000251968	ENSP00000304592
ENSP00000251968	ENSP00000307275
ENSP00000251968	ENSP00000307387
ENSP00000251968	ENSP00000310440
ENSP00000251968	ENSP00000312697
ENSP00000251968	ENSP00000313881
ENSP00000251968	ENSP00000317468
ENSP00000251968	ENSP00000318629
ENSP00000251968	ENSP00000320885
ENSP00000251968	ENSP00000328203
ENSP00000251968	ENSP00000331201
ENSP00000251968	ENSP00000333836
ENSP00000251968	ENSP00000334657
ENSP00000251968	ENSP00000342300
ENSP00000251968	ENSP00000342755
ENSP00000251968	ENSP00000345530
ENSP00000251968	ENSP00000348573
ENSP00000251968	ENSP00000349078
ENSP00000251968	ENSP00000349486
ENSP00000251968	ENSP00000349748
ENSP00000251968	ENSP00000350990
ENSP00000251968	ENSP00000352175
ENSP00000251968	ENSP00000352516
ENSP00000251968	ENSP00000354522
ENSP00000251968	ENSP00000354772
ENSP00000251968	ENSP00000355028
ENSP00000251968	ENSP00000356104
ENSP00000251968	ENSP00000358071
ENSP00000251968	ENSP00000358737
ENSP00000251968	ENSP00000360475
ENSP00000251968	ENSP00000360532

ENSP00000251968	ENSP00000360540
ENSP00000251968	ENSP00000360621
ENSP00000251968	ENSP00000361845
ENSP00000251968	ENSP00000361949
ENSP00000251968	ENSP00000365131
ENSP00000251968	ENSP00000366565
ENSP00000251968	ENSP00000366746
ENSP00000251968	ENSP00000366843
ENSP00000251968	ENSP00000367299
ENSP00000251968	ENSP00000370719
ENSP00000251968	ENSP00000375863
ENSP00000251968	ENSP00000381031
ENSP00000251968	ENSP00000381293
ENSP00000251968	ENSP00000387262
ENSP00000251968	ENSP00000387347
ENSP00000251968	ENSP00000396876
ENSP00000251968	ENSP00000408910
ENSP00000251968	ENSP00000416892
ENSP00000251968	ENSP00000417281
ENSP00000251968	ENSP00000417764
ENSP00000252015	ENSP00000259808
ENSP00000252015	ENSP00000297265
ENSP00000252015	ENSP00000301764
ENSP00000252015	ENSP00000341268
ENSP00000252015	ENSP00000352928
ENSP00000252029	ENSP00000263629
ENSP00000252029	ENSP00000289371
ENSP00000252029	ENSP00000345445
ENSP00000252029	ENSP00000346155
ENSP00000252029	ENSP00000370737
ENSP00000252034	ENSP00000253004
ENSP00000252034	ENSP00000254035
ENSP00000252034	ENSP00000254301
ENSP00000252034	ENSP00000261921
ENSP00000252034	ENSP00000317232
ENSP00000252034	ENSP00000359652
ENSP00000252034	ENSP00000361405
ENSP00000252034	ENSP00000363827
ENSP00000252034	ENSP00000364685
ENSP00000252034	ENSP00000384169
ENSP00000252037	ENSP00000254940
ENSP00000252037	ENSP00000265963
ENSP00000252037	ENSP00000267415
ENSP00000252037	ENSP00000317992
ENSP00000252037	ENSP00000322396
ENSP00000252037	ENSP00000338862

ENSP00000252037	ENSP00000351141
ENSP00000252037	ENSP00000354040
ENSP00000252037	ENSP00000356785
ENSP00000252037	ENSP00000359345
ENSP00000252037	ENSP00000359688
ENSP00000252037	ENSP00000360310
ENSP00000252037	ENSP00000360412
ENSP00000252037	ENSP00000363384
ENSP00000252037	ENSP00000374529
ENSP00000252037	ENSP00000377496
ENSP00000252037	ENSP00000380982
ENSP00000252037	ENSP00000381657
ENSP00000252037	ENSP00000402338
ENSP00000252050	ENSP00000256398
ENSP00000252050	ENSP00000329466
ENSP00000252050	ENSP00000348577
ENSP00000252102	ENSP00000252711
ENSP00000252102	ENSP00000259037
ENSP00000252102	ENSP00000263774
ENSP00000252102	ENSP00000266544
ENSP00000252102	ENSP00000268668
ENSP00000252102	ENSP00000274137
ENSP00000252102	ENSP00000276062
ENSP00000252102	ENSP00000276689
ENSP00000252102	ENSP00000281031
ENSP00000252102	ENSP00000296684
ENSP00000252102	ENSP00000299166
ENSP00000252102	ENSP00000301457
ENSP00000252102	ENSP00000311740
ENSP00000252102	ENSP00000315774
ENSP00000252102	ENSP00000322450
ENSP00000252102	ENSP00000327268
ENSP00000252102	ENSP00000330737
ENSP00000252102	ENSP00000346196
ENSP00000252102	ENSP00000347988
ENSP00000252102	ENSP00000348965
ENSP00000252102	ENSP00000354687
ENSP00000252102	ENSP00000354961
ENSP00000252102	ENSP00000356972
ENSP00000252102	ENSP00000362058
ENSP00000252102	ENSP00000362873
ENSP00000252108	ENSP00000261942
ENSP00000252108	ENSP00000263202
ENSP00000252108	ENSP00000272638
ENSP00000252108	ENSP00000282388
ENSP00000252108	ENSP00000283943

ENSP00000252108	ENSP00000296509
ENSP00000252108	ENSP00000300574
ENSP00000252108	ENSP00000321617
ENSP00000252108	ENSP00000326813
ENSP00000252108	ENSP00000326921
ENSP00000252108	ENSP00000331201
ENSP00000252108	ENSP00000331487
ENSP00000252108	ENSP00000344936
ENSP00000252108	ENSP00000347498
ENSP00000252108	ENSP00000347997
ENSP00000252108	ENSP00000350708
ENSP00000252108	ENSP00000354883
ENSP00000252108	ENSP00000363338
ENSP00000252108	ENSP00000369531
ENSP00000252108	ENSP00000380460
ENSP00000252108	ENSP00000382269
ENSP00000252108	ENSP00000401980
ENSP00000252115	ENSP00000253107
ENSP00000252115	ENSP00000254037
ENSP00000252115	ENSP00000254193
ENSP00000252115	ENSP00000254442
ENSP00000252115	ENSP00000254942
ENSP00000252115	ENSP00000259569
ENSP00000252115	ENSP00000259750
ENSP00000252115	ENSP00000261708
ENSP00000252115	ENSP00000261776
ENSP00000252115	ENSP00000262077
ENSP00000252115	ENSP00000262715
ENSP00000252115	ENSP00000262854
ENSP00000252115	ENSP00000262903
ENSP00000252115	ENSP00000263576
ENSP00000252115	ENSP00000264029
ENSP00000252115	ENSP00000264108
ENSP00000252115	ENSP00000264157
ENSP00000252115	ENSP00000264169
ENSP00000252115	ENSP00000264951
ENSP00000252115	ENSP00000265175
ENSP00000252115	ENSP00000265245
ENSP00000252115	ENSP00000266079
ENSP00000252115	ENSP00000268482
ENSP00000252115	ENSP00000268616
ENSP00000252115	ENSP00000269499
ENSP00000252115	ENSP00000282007
ENSP00000252115	ENSP00000285873
ENSP00000252115	ENSP00000291688
ENSP00000252115	ENSP00000296257

ENSP00000252115	ENSP00000296490
ENSP00000252115	ENSP00000298746
ENSP00000252115	ENSP00000300648
ENSP00000252115	ENSP00000301364
ENSP00000252115	ENSP00000301764
ENSP00000252115	ENSP00000302768
ENSP00000252115	ENSP00000303191
ENSP00000252115	ENSP00000304704
ENSP00000252115	ENSP00000307863
ENSP00000252115	ENSP00000308332
ENSP00000252115	ENSP00000310668
ENSP00000252115	ENSP00000310723
ENSP00000252115	ENSP00000311135
ENSP00000252115	ENSP00000312311
ENSP00000252115	ENSP00000314348
ENSP00000252115	ENSP00000314441
ENSP00000252115	ENSP00000315859
ENSP00000252115	ENSP00000316879
ENSP00000252115	ENSP00000317992
ENSP00000252115	ENSP00000318195
ENSP00000252115	ENSP00000318374
ENSP00000252115	ENSP00000318861
ENSP00000252115	ENSP00000320488
ENSP00000252115	ENSP00000320917
ENSP00000252115	ENSP00000322396
ENSP00000252115	ENSP00000323302
ENSP00000252115	ENSP00000326806
ENSP00000252115	ENSP00000327197
ENSP00000252115	ENSP00000329466
ENSP00000252115	ENSP00000331907
ENSP00000252115	ENSP00000332103
ENSP00000252115	ENSP00000332444
ENSP00000252115	ENSP00000333401
ENSP00000252115	ENSP00000337405
ENSP00000252115	ENSP00000337839
ENSP00000252115	ENSP00000338862
ENSP00000252115	ENSP00000339353
ENSP00000252115	ENSP00000339845
ENSP00000252115	ENSP00000340281
ENSP00000252115	ENSP00000342381
ENSP00000252115	ENSP00000343557
ENSP00000252115	ENSP00000344055
ENSP00000252115	ENSP00000346120
ENSP00000252115	ENSP00000346453
ENSP00000252115	ENSP00000346725
ENSP00000252115	ENSP00000347087

ENSP00000252115	ENSP00000348596
ENSP00000252115	ENSP00000348714
ENSP00000252115	ENSP00000348877
ENSP00000252115	ENSP00000350249
ENSP00000252115	ENSP00000350491
ENSP00000252115	ENSP00000352400
ENSP00000252115	ENSP00000352738
ENSP00000252115	ENSP00000352820
ENSP00000252115	ENSP00000353059
ENSP00000252115	ENSP00000353246
ENSP00000252115	ENSP00000354040
ENSP00000252115	ENSP00000355541
ENSP00000252115	ENSP00000356448
ENSP00000252115	ENSP00000356470
ENSP00000252115	ENSP00000358563
ENSP00000252115	ENSP00000358812
ENSP00000252115	ENSP00000360286
ENSP00000252115	ENSP00000360412
ENSP00000252115	ENSP00000360621
ENSP00000252115	ENSP00000361232
ENSP00000252115	ENSP00000361245
ENSP00000252115	ENSP00000361577
ENSP00000252115	ENSP00000361949
ENSP00000252115	ENSP00000362171
ENSP00000252115	ENSP00000362900
ENSP00000252115	ENSP00000363313
ENSP00000252115	ENSP00000363338
ENSP00000252115	ENSP00000364289
ENSP00000252115	ENSP00000364293
ENSP00000252115	ENSP00000366135
ENSP00000252115	ENSP00000366519
ENSP00000252115	ENSP00000366607
ENSP00000252115	ENSP00000366953
ENSP00000252115	ENSP00000368984
ENSP00000252115	ENSP00000370736
ENSP00000252115	ENSP00000372445
ENSP00000252115	ENSP00000374280
ENSP00000252115	ENSP00000374390
ENSP00000252115	ENSP00000374529
ENSP00000252115	ENSP00000380982
ENSP00000252115	ENSP00000382392
ENSP00000252115	ENSP00000384302
ENSP00000252115	ENSP00000386759
ENSP00000252115	ENSP00000390475
ENSP00000252115	ENSP00000394791
ENSP00000252115	ENSP00000396704

ENSP00000252115	ENSP00000402338
ENSP00000252115	ENSP00000412566
ENSP00000252136	ENSP00000261868
ENSP00000252136	ENSP00000270223
ENSP00000252136	ENSP00000311873
ENSP00000252136	ENSP00000318977
ENSP00000252136	ENSP00000329411
ENSP00000252136	ENSP00000336606
ENSP00000252136	ENSP00000343190
ENSP00000252136	ENSP00000347978
ENSP00000252136	ENSP00000351284
ENSP00000252136	ENSP00000361642
ENSP00000252137	ENSP00000259569
ENSP00000252137	ENSP00000261712
ENSP00000252137	ENSP00000261973
ENSP00000252137	ENSP00000262962
ENSP00000252137	ENSP00000266126
ENSP00000252137	ENSP00000271452
ENSP00000252137	ENSP00000281172
ENSP00000252137	ENSP00000296015
ENSP00000252137	ENSP00000297954
ENSP00000252137	ENSP00000299259
ENSP00000252137	ENSP00000302160
ENSP00000252137	ENSP00000304370
ENSP00000252137	ENSP00000309166
ENSP00000252137	ENSP00000311005
ENSP00000252137	ENSP00000319169
ENSP00000252137	ENSP00000328854
ENSP00000252137	ENSP00000332171
ENSP00000252137	ENSP00000333920
ENSP00000252137	ENSP00000334148
ENSP00000252137	ENSP00000334474
ENSP00000252137	ENSP00000344683
ENSP00000252137	ENSP00000344741
ENSP00000252137	ENSP00000346453
ENSP00000252137	ENSP00000351137
ENSP00000252137	ENSP00000356448
ENSP00000252137	ENSP00000357907
ENSP00000252137	ENSP00000360286
ENSP00000252137	ENSP00000360488
ENSP00000252137	ENSP00000362592
ENSP00000252137	ENSP00000363349
ENSP00000252137	ENSP00000374443
ENSP00000252137	ENSP00000394869
ENSP00000252137	ENSP00000398597
ENSP00000252137	ENSP00000413632

ENSP00000252244	ENSP00000257430
ENSP00000252244	ENSP00000259075
ENSP00000252244	ENSP00000264474
ENSP00000252244	ENSP00000265023
ENSP00000252244	ENSP00000281537
ENSP00000252244	ENSP0000289081
ENSP00000252244	ENSP00000295897
ENSP00000252244	ENSP00000301607
ENSP00000252244	ENSP00000306124
ENSP00000252244	ENSP00000357731
ENSP00000252244	ENSP00000357753
ENSP00000252244	ENSP00000373952
ENSP00000252329	ENSP00000253003
ENSP00000252329	ENSP00000259457
ENSP00000252329	ENSP00000262193
ENSP00000252329	ENSP00000290541
ENSP00000252329	ENSP00000298852
ENSP00000252329	ENSP00000309474
ENSP00000252329	ENSP00000310129
ENSP00000252329	ENSP00000325919
ENSP00000252329	ENSP00000329915
ENSP00000252329	ENSP00000335808
ENSP00000252329	ENSP00000362334
ENSP00000252329	ENSP00000370222
ENSP00000252445	ENSP00000263208
ENSP00000252445	ENSP00000263390
ENSP00000252445	ENSP00000263736
ENSP00000252445	ENSP00000263857
ENSP00000252445	ENSP00000272645
ENSP00000252445	ENSP00000295066
ENSP00000252445	ENSP00000295321
ENSP00000252445	ENSP00000296328
ENSP00000252445	ENSP00000299601
ENSP00000252445	ENSP00000314949
ENSP00000252445	ENSP00000317468
ENSP00000252445	ENSP00000319104
ENSP00000252445	ENSP00000321617
ENSP00000252445	ENSP00000326879
ENSP00000252445	ENSP00000336842
ENSP00000252445	ENSP00000343190
ENSP00000252445	ENSP00000356405
ENSP00000252445	ENSP00000361465
ENSP00000252445	ENSP00000361642
ENSP00000252445	ENSP00000363559
ENSP00000252445	ENSP00000365025
ENSP00000252445	ENSP00000374280

ENSP00000252445	ENSP00000386621
ENSP00000252445	ENSP00000389813
ENSP00000252445	ENSP00000406293
ENSP00000252453	ENSP00000321239
ENSP00000252483	ENSP00000274030
ENSP00000252483	ENSP00000280200
ENSP00000252483	ENSP00000349465
ENSP00000252483	ENSP00000356991
ENSP00000252483	ENSP00000373167
ENSP00000252483	ENSP00000383623
ENSP00000252483	ENSP00000418070
ENSP00000252486	ENSP00000261693
ENSP00000252486	ENSP00000263816
ENSP00000252486	ENSP00000280154
ENSP00000252486	ENSP00000284981
ENSP00000252486	ENSP00000286627
ENSP00000252486	ENSP00000295897
ENSP00000252486	ENSP00000314615
ENSP00000252486	ENSP00000326366
ENSP00000252486	ENSP00000340820
ENSP00000252486	ENSP00000350718
ENSP00000252486	ENSP00000353508
ENSP00000252486	ENSP00000355370
ENSP00000252486	ENSP00000361508
ENSP00000252487	ENSP00000269143
ENSP00000252487	ENSP00000284320
ENSP00000252487	ENSP00000293288
ENSP00000252487	ENSP00000295767
ENSP00000252487	ENSP00000305958
ENSP00000252487	ENSP00000311766
ENSP00000252487	ENSP00000318115
ENSP00000252487	ENSP00000320236
ENSP00000252487	ENSP00000345445
ENSP00000252487	ENSP00000351214
ENSP00000252487	ENSP00000352219
ENSP00000252487	ENSP00000354204
ENSP00000252487	ENSP00000355167
ENSP00000252487	ENSP00000356785
ENSP00000252487	ENSP00000357360
ENSP00000252487	ENSP00000358042
ENSP00000252487	ENSP00000361686
ENSP00000252487	ENSP00000365766
ENSP00000252487	ENSP00000387262
ENSP00000252487	ENSP00000388872
ENSP00000252487	ENSP00000407401
ENSP00000252543	ENSP00000253099

ENSP00000252543	ENSP00000253788
ENSP00000252543	ENSP00000254037
ENSP00000252543	ENSP00000257829
ENSP00000252543	ENSP00000258455
ENSP00000252543	ENSP00000259469
ENSP00000252543	ENSP00000260102
ENSP00000252543	ENSP00000260443
ENSP00000252543	ENSP00000261531
ENSP00000252543	ENSP00000262584
ENSP00000252543	ENSP00000264995
ENSP00000252543	ENSP00000265100
ENSP00000252543	ENSP00000265245
ENSP00000252543	ENSP00000268616
ENSP00000252543	ENSP00000268661
ENSP00000252543	ENSP00000269499
ENSP00000252543	ENSP00000270625
ENSP00000252543	ENSP00000274242
ENSP00000252543	ENSP00000274606
ENSP00000252543	ENSP00000278572
ENSP00000252543	ENSP00000287038
ENSP00000252543	ENSP00000289371
ENSP00000252543	ENSP00000294189
ENSP00000252543	ENSP00000296411
ENSP00000252543	ENSP00000296674
ENSP00000252543	ENSP00000296802
ENSP00000252543	ENSP00000298283
ENSP00000252543	ENSP00000300151
ENSP00000252543	ENSP00000302886
ENSP00000252543	ENSP00000302896
ENSP00000252543	ENSP00000306548
ENSP00000252543	ENSP00000307889
ENSP00000252543	ENSP00000308897
ENSP00000252543	ENSP00000309334
ENSP00000252543	ENSP00000309830
ENSP00000252543	ENSP00000311028
ENSP00000252543	ENSP00000311430
ENSP00000252543	ENSP00000317376
ENSP00000252543	ENSP00000318646
ENSP00000252543	ENSP00000322419
ENSP00000252543	ENSP00000325312
ENSP00000252543	ENSP00000326981
ENSP00000252543	ENSP00000328690
ENSP00000252543	ENSP00000334813
ENSP00000252543	ENSP00000337839
ENSP00000252543	ENSP00000338573
ENSP00000252543	ENSP00000339095

ENSP00000252543	ENSP00000339795
ENSP00000252543	ENSP00000344683
ENSP00000252543	ENSP00000345156
ENSP00000252543	ENSP00000346015
ENSP00000252543	ENSP00000346022
ENSP00000252543	ENSP00000346027
ENSP00000252543	ENSP00000346037
ENSP00000252543	ENSP00000346050
ENSP00000252543	ENSP00000346088
ENSP00000252543	ENSP00000346725
ENSP00000252543	ENSP00000347087
ENSP00000252543	ENSP00000348965
ENSP00000252543	ENSP00000352514
ENSP00000252543	ENSP00000353741
ENSP00000252543	ENSP00000354040
ENSP00000252543	ENSP00000354525
ENSP00000252543	ENSP00000354739
ENSP00000252543	ENSP00000359345
ENSP00000252543	ENSP00000361076
ENSP00000252543	ENSP00000362153
ENSP00000252543	ENSP00000362744
ENSP00000252543	ENSP00000363018
ENSP00000252543	ENSP00000363384
ENSP00000252543	ENSP00000363559
ENSP00000252543	ENSP00000363676
ENSP00000252543	ENSP00000364320
ENSP00000252543	ENSP00000369757
ENSP00000252543	ENSP00000371101
ENSP00000252543	ENSP00000373404
ENSP00000252543	ENSP00000375730
ENSP00000252543	ENSP00000377640
ENSP00000252543	ENSP00000377865
ENSP00000252543	ENSP00000378160
ENSP00000252543	ENSP00000379607
ENSP00000252543	ENSP00000379888
ENSP00000252543	ENSP00000380156
ENSP00000252543	ENSP00000386717
ENSP00000252543	ENSP00000389103
ENSP00000252543	ENSP00000391723
ENSP00000252543	ENSP00000393241
ENSP00000252543	ENSP00000393393
ENSP00000252543	ENSP00000402338
ENSP00000252543	ENSP00000403175
ENSP00000252543	ENSP00000412583
ENSP00000252543	ENSP00000414321
ENSP00000252543	ENSP00000416110

ENSP00000252575	ENSP00000341170
ENSP00000252593	ENSP00000379383
ENSP00000252597	ENSP00000260383
ENSP00000252597	ENSP00000261597
ENSP00000252597	ENSP00000262419
ENSP00000252597	ENSP00000264389
ENSP00000252597	ENSP00000265963
ENSP00000252597	ENSP00000298295
ENSP00000252597	ENSP00000302935
ENSP00000252597	ENSP00000318821
ENSP00000252597	ENSP00000324292
ENSP00000252597	ENSP00000325414
ENSP00000252597	ENSP00000326514
ENSP00000252597	ENSP00000328854
ENSP00000252597	ENSP00000331201
ENSP00000252597	ENSP00000334100
ENSP00000252597	ENSP00000336524
ENSP00000252597	ENSP00000353564
ENSP00000252597	ENSP00000354270
ENSP00000252597	ENSP00000355944
ENSP00000252597	ENSP00000359375
ENSP00000252597	ENSP00000361276
ENSP00000252597	ENSP00000367910
ENSP00000252597	ENSP00000370671
ENSP00000252597	ENSP00000374529
ENSP00000252597	ENSP00000377793
ENSP00000252597	ENSP00000381795
ENSP00000252602	ENSP00000262584
ENSP00000252602	ENSP00000264995
ENSP00000252602	ENSP00000265100
ENSP00000252602	ENSP00000288937
ENSP00000252602	ENSP00000306548
ENSP00000252602	ENSP00000308897
ENSP00000252602	ENSP00000333837
ENSP00000252602	ENSP00000354525
ENSP00000252602	ENSP00000354739
ENSP00000252602	ENSP00000373404
ENSP00000252602	ENSP00000375730
ENSP00000252602	ENSP00000377486
ENSP00000252622	ENSP00000253107
ENSP00000252622	ENSP00000254193
ENSP00000252622	ENSP00000258400
ENSP00000252622	ENSP00000258416
ENSP00000252622	ENSP00000260210
ENSP00000252622	ENSP00000260956
ENSP00000252622	ENSP00000261951

ENSP00000252622	ENSP00000262518
ENSP00000252622	ENSP00000262982
ENSP00000252622	ENSP00000263576
ENSP00000252622	ENSP00000264233
ENSP00000252622	ENSP00000264951
ENSP00000252622	ENSP00000265304
ENSP00000252622	ENSP00000266079
ENSP00000252622	ENSP00000266735
ENSP00000252622	ENSP00000268206
ENSP00000252622	ENSP00000268483
ENSP00000252622	ENSP00000272227
ENSP00000252622	ENSP00000274712
ENSP00000252622	ENSP00000280665
ENSP00000252622	ENSP00000292535
ENSP00000252622	ENSP00000295488
ENSP00000252622	ENSP00000296215
ENSP00000252622	ENSP00000296805
ENSP00000252622	ENSP00000300146
ENSP00000252622	ENSP00000300413
ENSP00000252622	ENSP00000300441
ENSP00000252622	ENSP00000300648
ENSP00000252622	ENSP00000301740
ENSP00000252622	ENSP00000302160
ENSP00000252622	ENSP00000303515
ENSP00000252622	ENSP00000304350
ENSP00000252622	ENSP00000304903
ENSP00000252622	ENSP00000308546
ENSP00000252622	ENSP00000310448
ENSP00000252622	ENSP00000310596
ENSP00000252622	ENSP00000310723
ENSP00000252622	ENSP00000314311
ENSP00000252622	ENSP00000315035
ENSP00000252622	ENSP00000315379
ENSP00000252622	ENSP00000319341
ENSP00000252622	ENSP00000320503
ENSP00000252622	ENSP00000320566
ENSP00000252622	ENSP00000323046
ENSP00000252622	ENSP00000324122
ENSP00000252622	ENSP00000324573
ENSP00000252622	ENSP00000331310
ENSP00000252622	ENSP00000332103
ENSP00000252622	ENSP00000339090
ENSP00000252622	ENSP00000339503
ENSP00000252622	ENSP00000342374
ENSP00000252622	ENSP00000348107
ENSP00000252622	ENSP00000349465

ENSP00000252622	ENSP00000350512
ENSP00000252622	ENSP00000354522
ENSP00000252622	ENSP00000357887
ENSP00000252622	ENSP00000358716
ENSP00000252622	ENSP00000359454
ENSP00000252622	ENSP00000359939
ENSP00000252622	ENSP00000361433
ENSP00000252622	ENSP00000361577
ENSP00000252622	ENSP00000362110
ENSP00000252622	ENSP00000362413
ENSP00000252622	ENSP00000363313
ENSP00000252622	ENSP00000363734
ENSP00000252622	ENSP00000364813
ENSP00000252622	ENSP00000365837
ENSP00000252622	ENSP00000366135
ENSP00000252622	ENSP00000366557
ENSP00000252622	ENSP00000367835
ENSP00000252622	ENSP00000370083
ENSP00000252622	ENSP00000370119
ENSP00000252622	ENSP00000370589
ENSP00000252622	ENSP00000372694
ENSP00000252622	ENSP00000373715
ENSP00000252622	ENSP00000374213
ENSP00000252622	ENSP00000376973
ENSP00000252622	ENSP00000378529
ENSP00000252622	ENSP00000391723
ENSP00000252622	ENSP00000392466
ENSP00000252622	ENSP00000398597
ENSP00000252622	ENSP00000400591
ENSP00000252622	ENSP00000402240
ENSP00000252622	ENSP00000410758
ENSP00000252622	ENSP00000412319
ENSP00000252622	ENSP00000412566
ENSP00000252622	ENSP00000414006
ENSP00000252622	ENSP00000414634
ENSP00000252622	ENSP00000415678
ENSP00000252699	ENSP00000254035
ENSP00000252699	ENSP00000258682
ENSP00000252699	ENSP00000261207
ENSP00000252699	ENSP00000263666
ENSP00000252699	ENSP00000265517
ENSP00000252699	ENSP00000265810
ENSP00000252699	ENSP00000268182
ENSP00000252699	ENSP00000268668
ENSP00000252699	ENSP00000269122
ENSP00000252699	ENSP00000277575

ENSP00000252699	ENSP00000277865
ENSP00000252699	ENSP00000281537
ENSP00000252699	ENSP00000284770
ENSP00000252699	ENSP00000286091
ENSP00000252699	ENSP00000297508
ENSP00000252699	ENSP00000302640
ENSP00000252699	ENSP00000302913
ENSP00000252699	ENSP00000309629
ENSP00000252699	ENSP00000311005
ENSP00000252699	ENSP00000317159
ENSP00000252699	ENSP00000320885
ENSP00000252699	ENSP00000322170
ENSP00000252699	ENSP00000329466
ENSP00000252699	ENSP00000336927
ENSP00000252699	ENSP00000339795
ENSP00000252699	ENSP00000340937
ENSP00000252699	ENSP00000340944
ENSP00000252699	ENSP00000341838
ENSP00000252699	ENSP00000344456
ENSP00000252699	ENSP00000348128
ENSP00000252699	ENSP00000348163
ENSP00000252699	ENSP00000350249
ENSP00000252699	ENSP00000352272
ENSP00000252699	ENSP00000355153
ENSP00000252699	ENSP00000355205
ENSP00000252699	ENSP00000359345
ENSP00000252699	ENSP00000360112
ENSP00000252699	ENSP00000362424
ENSP00000252699	ENSP00000365891
ENSP00000252699	ENSP00000370083
ENSP00000252699	ENSP00000370119
ENSP00000252699	ENSP00000371729
ENSP00000252699	ENSP00000374455
ENSP00000252699	ENSP00000379607
ENSP00000252699	ENSP00000382064
ENSP00000252699	ENSP00000384792
ENSP00000252699	ENSP00000386259
ENSP00000252699	ENSP00000386520
ENSP00000252699	ENSP00000394794
ENSP00000252711	ENSP00000262878
ENSP00000252711	ENSP00000270625
ENSP00000252711	ENSP00000288828
ENSP00000252711	ENSP00000322706
ENSP00000252723	ENSP00000317636
ENSP00000252725	ENSP00000295685
ENSP00000252725	ENSP00000298910

ENSP00000252725	ENSP00000320924
ENSP00000252725	ENSP00000322170
ENSP00000252725	ENSP00000326238
ENSP00000252725	ENSP00000338727
ENSP00000252725	ENSP00000348163
ENSP00000252725	ENSP00000348784
ENSP00000252725	ENSP00000350757
ENSP00000252725	ENSP00000352802
ENSP00000252725	ENSP00000360798
ENSP00000252725	ENSP00000365147
ENSP00000252725	ENSP00000365891
ENSP00000252725	ENSP00000375857
ENSP00000252725	ENSP00000380431
ENSP00000252725	ENSP00000386878
ENSP00000252771	ENSP00000260008
ENSP00000252771	ENSP00000268182
ENSP00000252771	ENSP00000270861
ENSP00000252771	ENSP00000274289
ENSP00000252771	ENSP00000280190
ENSP00000252771	ENSP00000281282
ENSP00000252771	ENSP00000318884
ENSP00000252771	ENSP00000326699
ENSP00000252771	ENSP00000333950
ENSP00000252771	ENSP00000338157
ENSP00000252771	ENSP00000376410
ENSP00000252771	ENSP00000391249
ENSP00000252771	ENSP00000397351
ENSP00000252771	ENSP00000397598
ENSP00000252771	ENSP00000407879
ENSP00000252799	ENSP00000358918
ENSP00000252804	ENSP00000265970
ENSP00000252809	ENSP00000296137
ENSP00000252809	ENSP00000305459
ENSP00000252825	ENSP00000379607
ENSP00000252934	ENSP00000256216
ENSP00000252934	ENSP00000261712
ENSP00000252934	ENSP00000290429
ENSP00000252934	ENSP00000296577
ENSP00000252934	ENSP00000299259
ENSP00000252934	ENSP00000311505
ENSP00000252934	ENSP00000341213
ENSP00000252934	ENSP00000350512
ENSP00000252934	ENSP00000362824
ENSP00000252934	ENSP00000364847
ENSP00000252934	ENSP00000369257
ENSP00000252936	ENSP00000252999

ENSP00000252936	ENSP00000260731
ENSP00000252936	ENSP00000261531
ENSP00000252936	ENSP00000261965
ENSP00000252936	ENSP00000263791
ENSP00000252936	ENSP00000270861
ENSP00000252936	ENSP00000274289
ENSP00000252936	ENSP00000280527
ENSP00000252936	ENSP00000288976
ENSP00000252936	ENSP00000296509
ENSP00000252936	ENSP00000300584
ENSP00000252936	ENSP00000304668
ENSP00000252936	ENSP00000305941
ENSP00000252936	ENSP00000325562
ENSP00000252936	ENSP00000331831
ENSP00000252936	ENSP00000335357
ENSP00000252936	ENSP00000346566
ENSP00000252936	ENSP00000348573
ENSP00000252936	ENSP00000352572
ENSP00000252936	ENSP00000354916
ENSP00000252936	ENSP00000356448
ENSP00000252936	ENSP00000359206
ENSP00000252936	ENSP00000360532
ENSP00000252936	ENSP00000363018
ENSP00000252936	ENSP00000363734
ENSP00000252936	ENSP00000367049
ENSP00000252936	ENSP00000368976
ENSP00000252936	ENSP00000370517
ENSP00000252936	ENSP00000372694
ENSP00000252936	ENSP00000380921
ENSP00000252936	ENSP00000383145
ENSP00000252936	ENSP00000402038
ENSP00000252936	ENSP00000402240
ENSP00000252936	ENSP00000412319
ENSP00000252936	ENSP00000413625
ENSP00000252936	ENSP00000415678
ENSP00000252996	ENSP00000259235
ENSP00000252996	ENSP00000261951
ENSP00000252996	ENSP00000263208
ENSP00000252996	ENSP00000263390
ENSP00000252996	ENSP00000263974
ENSP00000252996	ENSP00000264193
ENSP00000252996	ENSP00000265372
ENSP00000252996	ENSP00000267064
ENSP00000252996	ENSP00000276072
ENSP00000252996	ENSP00000282388
ENSP00000252996	ENSP00000298937

ENSP00000252996	ENSP00000299424
ENSP00000252996	ENSP00000302327
ENSP00000252996	ENSP00000306983
ENSP00000252996	ENSP00000307208
ENSP00000252996	ENSP00000320949
ENSP00000252996	ENSP00000329357
ENSP00000252996	ENSP00000331057
ENSP00000252996	ENSP00000340271
ENSP00000252996	ENSP00000341805
ENSP00000252996	ENSP00000344537
ENSP00000252996	ENSP00000347184
ENSP00000252996	ENSP00000347733
ENSP00000252996	ENSP00000349213
ENSP00000252996	ENSP00000353622
ENSP00000252996	ENSP00000354633
ENSP00000252996	ENSP00000355051
ENSP00000252996	ENSP00000357442
ENSP00000252996	ENSP00000357494
ENSP00000252996	ENSP00000358854
ENSP00000252996	ENSP00000359958
ENSP00000252996	ENSP00000360515
ENSP00000252996	ENSP00000362068
ENSP00000252996	ENSP00000364430
ENSP00000252996	ENSP00000367406
ENSP00000252996	ENSP00000369695
ENSP00000252996	ENSP00000379372
ENSP00000252996	ENSP00000381031
ENSP00000252996	ENSP00000418734
ENSP00000252999	ENSP00000261965
ENSP00000252999	ENSP00000263816
ENSP00000252999	ENSP00000271452
ENSP00000252999	ENSP00000278174
ENSP00000252999	ENSP00000302021
ENSP00000252999	ENSP00000312435
ENSP00000252999	ENSP00000346566
ENSP00000252999	ENSP00000356448
ENSP00000252999	ENSP00000357547
ENSP00000252999	ENSP00000358142
ENSP00000252999	ENSP00000382562
ENSP00000252999	ENSP00000384169
ENSP00000253003	ENSP00000259457
ENSP00000253003	ENSP00000261601
ENSP00000253003	ENSP00000261712
ENSP00000253003	ENSP00000262193
ENSP00000253003	ENSP00000262394
ENSP00000253003	ENSP00000263205

ENSP00000253003	ENSP00000264335
ENSP00000253003	ENSP00000264639
ENSP00000253003	ENSP00000290541
ENSP00000253003	ENSP00000293879
ENSP00000253003	ENSP00000295901
ENSP00000253003	ENSP00000296255
ENSP00000253003	ENSP00000296792
ENSP00000253003	ENSP00000298852
ENSP00000253003	ENSP00000299206
ENSP00000253003	ENSP00000299259
ENSP00000253003	ENSP00000309474
ENSP00000253003	ENSP00000310129
ENSP00000253003	ENSP00000311245
ENSP00000253003	ENSP00000311665
ENSP00000253003	ENSP00000318313
ENSP00000253003	ENSP00000325448
ENSP00000253003	ENSP00000325919
ENSP00000253003	ENSP00000339449
ENSP00000253003	ENSP00000348129
ENSP00000253003	ENSP00000348442
ENSP00000253003	ENSP00000350708
ENSP00000253003	ENSP00000356641
ENSP00000253003	ENSP00000356774
ENSP00000253003	ENSP00000360216
ENSP00000253003	ENSP00000361993
ENSP00000253003	ENSP00000362334
ENSP00000253003	ENSP00000384211
ENSP00000253003	ENSP00000386212
ENSP00000253003	ENSP00000396937
ENSP00000253003	ENSP00000400476
ENSP00000253004	ENSP00000254942
ENSP00000253004	ENSP00000276603
ENSP00000253004	ENSP00000288602
ENSP00000253004	ENSP00000300245
ENSP00000253004	ENSP00000328690
ENSP00000253004	ENSP00000333667
ENSP00000253004	ENSP00000363676
ENSP00000253004	ENSP00000372199
ENSP00000253004	ENSP00000379933
ENSP00000253004	ENSP00000381654
ENSP00000253031	ENSP00000259400
ENSP00000253031	ENSP00000268129
ENSP00000253031	ENSP00000289119
ENSP00000253031	ENSP00000302657
ENSP00000253031	ENSP00000311134
ENSP00000253031	ENSP00000314615

ENSP00000253047	ENSP00000368797
ENSP00000253047	ENSP00000374455
ENSP00000253048	ENSP00000280362
ENSP00000253048	ENSP00000291386
ENSP00000253048	ENSP00000296490
ENSP00000253048	ENSP00000300291
ENSP00000253048	ENSP00000304704
ENSP00000253048	ENSP00000315791
ENSP00000253048	ENSP00000317872
ENSP00000253048	ENSP00000325377
ENSP00000253048	ENSP00000339353
ENSP00000253083	ENSP00000253699
ENSP00000253083	ENSP00000256854
ENSP00000253083	ENSP00000260008
ENSP00000253083	ENSP00000261531
ENSP00000253083	ENSP00000262039
ENSP00000253083	ENSP00000263228
ENSP00000253083	ENSP00000263277
ENSP00000253083	ENSP00000264161
ENSP00000253083	ENSP00000269122
ENSP00000253083	ENSP00000272322
ENSP00000253083	ENSP00000276416
ENSP00000253083	ENSP00000281187
ENSP00000253083	ENSP00000299138
ENSP00000253083	ENSP00000301843
ENSP00000253083	ENSP00000303434
ENSP00000253083	ENSP00000314499
ENSP00000253083	ENSP00000314615
ENSP00000253083	ENSP00000318884
ENSP00000253083	ENSP00000320543
ENSP00000253083	ENSP00000320924
ENSP00000253083	ENSP00000326534
ENSP00000253083	ENSP00000327650
ENSP00000253083	ENSP00000333657
ENSP00000253083	ENSP00000333950
ENSP00000253083	ENSP00000334836
ENSP00000253083	ENSP00000343526
ENSP00000253083	ENSP00000345530
ENSP00000253083	ENSP00000347184
ENSP00000253083	ENSP00000348163
ENSP00000253083	ENSP00000348602
ENSP00000253083	ENSP00000349205
ENSP00000253083	ENSP00000352802
ENSP00000253083	ENSP00000354522
ENSP00000253083	ENSP00000354558
ENSP00000253083	ENSP00000354681

ENSP00000253083	ENSP00000354916
ENSP00000253083	ENSP00000355471
ENSP00000253083	ENSP00000356134
ENSP00000253083	ENSP00000356811
ENSP00000253083	ENSP00000358140
ENSP00000253083	ENSP00000360183
ENSP00000253083	ENSP00000360532
ENSP00000253083	ENSP00000360798
ENSP00000253083	ENSP00000362399
ENSP00000253083	ENSP00000365891
ENSP00000253083	ENSP00000366746
ENSP00000253083	ENSP00000369810
ENSP00000253083	ENSP00000372697
ENSP00000253083	ENSP00000376410
ENSP00000253083	ENSP00000378529
ENSP00000253083	ENSP00000379228
ENSP00000253083	ENSP00000380921
ENSP00000253083	ENSP00000386456
ENSP00000253083	ENSP00000386878
ENSP00000253083	ENSP00000390831
ENSP00000253083	ENSP00000391197
ENSP00000253083	ENSP00000397351
ENSP00000253083	ENSP00000401435
ENSP00000253083	ENSP00000404016
ENSP00000253083	ENSP00000406988
ENSP00000253083	ENSP00000407487
ENSP00000253083	ENSP00000409952
ENSP00000253099	ENSP00000253788
ENSP00000253099	ENSP00000256404
ENSP00000253099	ENSP00000257829
ENSP00000253099	ENSP00000258383
ENSP00000253099	ENSP00000258455
ENSP00000253099	ENSP00000259469
ENSP00000253099	ENSP00000260102
ENSP00000253099	ENSP00000260443
ENSP00000253099	ENSP00000262077
ENSP00000253099	ENSP00000263629
ENSP00000253099	ENSP00000263657
ENSP00000253099	ENSP00000264335
ENSP00000253099	ENSP00000264995
ENSP00000253099	ENSP00000268206
ENSP00000253099	ENSP00000268661
ENSP00000253099	ENSP00000270538
ENSP00000253099	ENSP00000270625
ENSP00000253099	ENSP00000274242
ENSP00000253099	ENSP00000274606

ENSP00000253099	ENSP00000278572
ENSP00000253099	ENSP00000279242
ENSP00000253099	ENSP00000287038
ENSP00000253099	ENSP00000288937
ENSP00000253099	ENSP00000289371
ENSP00000253099	ENSP00000294189
ENSP00000253099	ENSP00000296102
ENSP00000253099	ENSP00000296577
ENSP00000253099	ENSP00000296802
ENSP00000253099	ENSP00000296805
ENSP00000253099	ENSP00000297908
ENSP00000253099	ENSP00000300151
ENSP00000253099	ENSP00000301585
ENSP00000253099	ENSP00000302160
ENSP00000253099	ENSP00000302896
ENSP00000253099	ENSP00000305503
ENSP00000253099	ENSP00000306548
ENSP00000253099	ENSP00000307889
ENSP00000253099	ENSP00000308275
ENSP00000253099	ENSP00000308897
ENSP00000253099	ENSP00000309334
ENSP00000253099	ENSP00000309830
ENSP00000253099	ENSP00000310668
ENSP00000253099	ENSP00000310723
ENSP00000253099	ENSP00000311028
ENSP00000253099	ENSP00000312311
ENSP00000253099	ENSP00000315017
ENSP00000253099	ENSP00000317376
ENSP00000253099	ENSP00000318646
ENSP00000253099	ENSP00000322419
ENSP00000253099	ENSP00000324122
ENSP00000253099	ENSP00000326981
ENSP00000253099	ENSP00000328690
ENSP00000253099	ENSP00000328854
ENSP00000253099	ENSP00000333401
ENSP00000253099	ENSP00000333837
ENSP00000253099	ENSP00000336702
ENSP00000253099	ENSP00000338862
ENSP00000253099	ENSP00000339095
ENSP00000253099	ENSP00000339795
ENSP00000253099	ENSP00000339845
ENSP00000253099	ENSP00000341082
ENSP00000253099	ENSP00000343392
ENSP00000253099	ENSP00000343745
ENSP00000253099	ENSP00000345156
ENSP00000253099	ENSP00000346015

ENSP00000253099	ENSP00000346022
ENSP00000253099	ENSP00000346027
ENSP00000253099	ENSP00000346037
ENSP00000253099	ENSP00000346050
ENSP00000253099	ENSP00000346088
ENSP00000253099	ENSP00000346725
ENSP00000253099	ENSP00000348886
ENSP00000253099	ENSP00000354040
ENSP00000253099	ENSP00000354125
ENSP00000253099	ENSP00000354525
ENSP00000253099	ENSP00000359345
ENSP00000253099	ENSP00000360031
ENSP00000253099	ENSP00000360498
ENSP00000253099	ENSP00000361076
ENSP00000253099	ENSP00000362744
ENSP00000253099	ENSP00000363018
ENSP00000253099	ENSP00000363676
ENSP00000253099	ENSP00000364320
ENSP00000253099	ENSP00000369757
ENSP00000253099	ENSP00000369965
ENSP00000253099	ENSP00000370930
ENSP00000253099	ENSP00000373404
ENSP00000253099	ENSP00000375730
ENSP00000253099	ENSP00000377486
ENSP00000253099	ENSP00000377640
ENSP00000253099	ENSP00000377865
ENSP00000253099	ENSP00000378160
ENSP00000253099	ENSP00000379888
ENSP00000253099	ENSP00000380156
ENSP00000253099	ENSP00000380982
ENSP00000253099	ENSP00000382392
ENSP00000253099	ENSP00000386717
ENSP00000253099	ENSP00000389103
ENSP00000253099	ENSP00000393393
ENSP00000253099	ENSP00000402338
ENSP00000253099	ENSP00000403817
ENSP00000253099	ENSP00000414321
ENSP00000253099	ENSP00000417229
ENSP00000253099	ENSP00000417602
ENSP00000253107	ENSP00000254037
ENSP00000253107	ENSP00000254605
ENSP00000253107	ENSP00000254940
ENSP00000253107	ENSP00000256383
ENSP00000253107	ENSP00000259469
ENSP00000253107	ENSP00000259569
ENSP00000253107	ENSP00000260210

ENSP00000253107	ENSP00000260443
ENSP00000253107	ENSP00000261015
ENSP00000253107	ENSP00000261868
ENSP00000253107	ENSP00000262294
ENSP00000253107	ENSP00000262518
ENSP00000253107	ENSP00000264670
ENSP00000253107	ENSP00000265304
ENSP00000253107	ENSP00000269127
ENSP00000253107	ENSP00000269385
ENSP00000253107	ENSP00000274606
ENSP00000253107	ENSP00000278412
ENSP00000253107	ENSP00000278856
ENSP00000253107	ENSP00000283351
ENSP00000253107	ENSP00000287038
ENSP00000253107	ENSP00000290158
ENSP00000253107	ENSP00000291634
ENSP00000253107	ENSP00000296802
ENSP00000253107	ENSP00000298310
ENSP00000253107	ENSP00000298746
ENSP00000253107	ENSP00000299601
ENSP00000253107	ENSP00000300648
ENSP00000253107	ENSP00000302768
ENSP00000253107	ENSP00000304593
ENSP00000253107	ENSP00000305702
ENSP00000253107	ENSP00000309830
ENSP00000253107	ENSP00000310596
ENSP00000253107	ENSP00000311343
ENSP00000253107	ENSP00000312735
ENSP00000253107	ENSP00000314348
ENSP00000253107	ENSP00000314971
ENSP00000253107	ENSP00000316879
ENSP00000253107	ENSP00000317992
ENSP00000253107	ENSP00000320566
ENSP00000253107	ENSP00000322396
ENSP00000253107	ENSP00000322570
ENSP00000253107	ENSP00000323424
ENSP00000253107	ENSP00000323858
ENSP00000253107	ENSP00000328690
ENSP00000253107	ENSP00000331817
ENSP00000253107	ENSP00000336712
ENSP00000253107	ENSP00000337839
ENSP00000253107	ENSP00000338862
ENSP00000253107	ENSP00000339145
ENSP00000253107	ENSP00000339795
ENSP00000253107	ENSP00000339845
ENSP00000253107	ENSP00000340281

ENSP00000253107	ENSP00000343745
ENSP00000253107	ENSP00000345917
ENSP00000253107	ENSP00000346120
ENSP00000253107	ENSP00000346453
ENSP00000253107	ENSP00000346725
ENSP00000253107	ENSP00000348107
ENSP00000253107	ENSP00000350967
ENSP00000253107	ENSP00000351697
ENSP00000253107	ENSP00000353246
ENSP00000253107	ENSP00000354040
ENSP00000253107	ENSP00000354522
ENSP00000253107	ENSP00000354673
ENSP00000253107	ENSP00000355013
ENSP00000253107	ENSP00000355899
ENSP00000253107	ENSP00000356290
ENSP00000253107	ENSP00000356405
ENSP00000253107	ENSP00000356989
ENSP00000253107	ENSP00000357048
ENSP00000253107	ENSP00000358497
ENSP00000253107	ENSP00000358563
ENSP00000253107	ENSP00000358605
ENSP00000253107	ENSP00000358812
ENSP00000253107	ENSP00000359345
ENSP00000253107	ENSP00000359518
ENSP00000253107	ENSP00000359688
ENSP00000253107	ENSP00000359939
ENSP00000253107	ENSP00000360412
ENSP00000253107	ENSP00000360828
ENSP00000253107	ENSP00000361010
ENSP00000253107	ENSP00000361092
ENSP00000253107	ENSP00000361232
ENSP00000253107	ENSP00000361465
ENSP00000253107	ENSP00000361982
ENSP00000253107	ENSP00000362576
ENSP00000253107	ENSP00000363559
ENSP00000253107	ENSP00000364320
ENSP00000253107	ENSP00000365025
ENSP00000253107	ENSP00000366135
ENSP00000253107	ENSP00000366395
ENSP00000253107	ENSP00000366519
ENSP00000253107	ENSP00000367029
ENSP00000253107	ENSP00000368349
ENSP00000253107	ENSP00000368887
ENSP00000253107	ENSP00000369716
ENSP00000253107	ENSP00000370589
ENSP00000253107	ENSP00000371201

ENSP00000253107	ENSP00000372199
ENSP00000253107	ENSP00000374213
ENSP00000253107	ENSP00000374280
ENSP00000253107	ENSP00000374390
ENSP00000253107	ENSP00000377640
ENSP00000253107	ENSP00000377747
ENSP00000253107	ENSP00000379888
ENSP00000253107	ENSP00000380156
ENSP00000253107	ENSP00000380453
ENSP00000253107	ENSP00000380982
ENSP00000253107	ENSP00000382392
ENSP00000253107	ENSP00000386134
ENSP00000253107	ENSP00000388464
ENSP00000253107	ENSP00000392028
ENSP00000253107	ENSP00000396704
ENSP00000253107	ENSP00000402338
ENSP00000253107	ENSP00000419740
ENSP00000253108	ENSP00000254037
ENSP00000253108	ENSP00000258169
ENSP00000253108	ENSP00000260129
ENSP00000253108	ENSP00000261531
ENSP00000253108	ENSP00000261868
ENSP00000253108	ENSP00000262056
ENSP00000253108	ENSP00000268616
ENSP00000253108	ENSP00000269127
ENSP00000253108	ENSP00000269499
ENSP00000253108	ENSP00000270625
ENSP00000253108	ENSP00000278572
ENSP00000253108	ENSP00000281141
ENSP00000253108	ENSP00000287295
ENSP00000253108	ENSP00000289371
ENSP00000253108	ENSP00000296577
ENSP00000253108	ENSP00000307863
ENSP00000253108	ENSP00000307889
ENSP00000253108	ENSP00000308622
ENSP00000253108	ENSP00000310040
ENSP00000253108	ENSP00000310448
ENSP00000253108	ENSP00000310668
ENSP00000253108	ENSP00000311028
ENSP00000253108	ENSP00000318195
ENSP00000253108	ENSP00000318604
ENSP00000253108	ENSP00000320917
ENSP00000253108	ENSP00000322396
ENSP00000253108	ENSP00000323856
ENSP00000253108	ENSP00000325448
ENSP00000253108	ENSP00000336702

ENSP00000253108	ENSP00000337839
ENSP00000253108	ENSP00000338862
ENSP00000253108	ENSP00000344241
ENSP00000253108	ENSP00000346453
ENSP00000253108	ENSP00000346725
ENSP00000253108	ENSP00000348157
ENSP00000253108	ENSP00000348455
ENSP00000253108	ENSP00000350249
ENSP00000253108	ENSP00000350512
ENSP00000253108	ENSP00000351492
ENSP00000253108	ENSP00000354040
ENSP00000253108	ENSP00000354125
ENSP00000253108	ENSP00000358140
ENSP00000253108	ENSP00000359345
ENSP00000253108	ENSP00000359531
ENSP00000253108	ENSP00000360532
ENSP00000253108	ENSP00000361949
ENSP00000253108	ENSP00000362110
ENSP00000253108	ENSP00000362688
ENSP00000253108	ENSP00000362936
ENSP00000253108	ENSP00000363944
ENSP00000253108	ENSP00000364289
ENSP00000253108	ENSP00000365025
ENSP00000253108	ENSP00000368887
ENSP00000253108	ENSP00000368927
ENSP00000253108	ENSP00000369716
ENSP00000253108	ENSP00000369757
ENSP00000253108	ENSP00000377496
ENSP00000253108	ENSP00000377747
ENSP00000253108	ENSP00000379607
ENSP00000253108	ENSP00000379888
ENSP00000253108	ENSP00000380982
ENSP00000253108	ENSP00000384302
ENSP00000253108	ENSP00000387170
ENSP00000253108	ENSP00000388872
ENSP00000253108	ENSP00000392028
ENSP00000253108	ENSP00000402338
ENSP00000253108	ENSP00000416892
ENSP00000253110	ENSP00000276603
ENSP00000253233	ENSP00000258975
ENSP00000253233	ENSP00000270625
ENSP00000253233	ENSP00000278572
ENSP00000253233	ENSP00000302896
ENSP00000253233	ENSP00000318646
ENSP00000253233	ENSP00000326981
ENSP00000253233	ENSP00000393241

ENSP00000253233	ENSP00000403175
ENSP00000253233	ENSP00000412583
ENSP00000253233	ENSP00000416110
ENSP00000253237	ENSP00000256578
ENSP00000253237	ENSP00000263265
ENSP00000253237	ENSP00000268661
ENSP00000253237	ENSP00000271628
ENSP00000253237	ENSP00000278572
ENSP00000253237	ENSP00000282388
ENSP00000253237	ENSP00000282412
ENSP00000253237	ENSP00000290231
ENSP00000253237	ENSP00000301019
ENSP00000253237	ENSP00000301764
ENSP00000253237	ENSP00000302955
ENSP00000253237	ENSP00000310448
ENSP00000253237	ENSP00000315700
ENSP00000253237	ENSP00000318646
ENSP00000253237	ENSP00000339145
ENSP00000253237	ENSP00000339795
ENSP00000253237	ENSP00000341885
ENSP00000253237	ENSP00000344741
ENSP00000253237	ENSP00000346725
ENSP00000253237	ENSP00000352645
ENSP00000253237	ENSP00000353246
ENSP00000253237	ENSP00000354040
ENSP00000253237	ENSP00000355958
ENSP00000253237	ENSP00000356641
ENSP00000253237	ENSP00000358400
ENSP00000253237	ENSP00000366135
ENSP00000253237	ENSP00000368468
ENSP00000253237	ENSP00000368612
ENSP00000253237	ENSP00000369757
ENSP00000253237	ENSP00000370258
ENSP00000253237	ENSP00000379607
ENSP00000253237	ENSP00000381657
ENSP00000253237	ENSP00000384109
ENSP00000253247	ENSP00000263209
ENSP00000253303	ENSP00000263934
ENSP00000253303	ENSP00000286091
ENSP00000253303	ENSP00000290332
ENSP00000253303	ENSP00000301729
ENSP00000253303	ENSP00000359699
ENSP00000253303	ENSP00000361467
ENSP00000253303	ENSP00000370376
ENSP00000253303	ENSP00000383059
ENSP00000253303	ENSP00000394071

ENSP00000253303	ENSP00000406293
ENSP00000253303	ENSP00000417686
ENSP00000253329	ENSP00000259569
ENSP00000253329	ENSP00000261531
ENSP00000253329	ENSP00000338862
ENSP00000253329	ENSP00000339723
ENSP00000253329	ENSP00000343535
ENSP00000253329	ENSP00000354040
ENSP00000253329	ENSP00000358242
ENSP00000253329	ENSP00000359042
ENSP00000253329	ENSP00000359345
ENSP00000253329	ENSP00000360532
ENSP00000253329	ENSP00000380982
ENSP00000253332	ENSP00000312262
ENSP00000253339	ENSP00000258418
ENSP00000253339	ENSP00000261402
ENSP00000253339	ENSP00000263228
ENSP00000253339	ENSP00000263360
ENSP00000253339	ENSP00000276014
ENSP00000253339	ENSP00000282441
ENSP00000253339	ENSP00000305422
ENSP00000253339	ENSP00000307023
ENSP00000253339	ENSP00000308339
ENSP00000253339	ENSP00000309103
ENSP00000253339	ENSP00000315702
ENSP00000253339	ENSP00000318089
ENSP00000253339	ENSP00000319169
ENSP00000253339	ENSP00000321711
ENSP00000253339	ENSP00000322957
ENSP00000253339	ENSP00000324422
ENSP00000253339	ENSP00000324729
ENSP00000253339	ENSP00000336740
ENSP00000253339	ENSP00000336790
ENSP00000253339	ENSP00000346809
ENSP00000253339	ENSP00000351113
ENSP00000253339	ENSP00000351327
ENSP00000253339	ENSP00000354916
ENSP00000253339	ENSP00000361009
ENSP00000253339	ENSP00000367407
ENSP00000253339	ENSP00000396704
ENSP00000253339	ENSP00000417281
ENSP00000253354	ENSP00000379607
ENSP00000253363	ENSP00000255764
ENSP00000253363	ENSP00000257181
ENSP00000253363	ENSP00000261531
ENSP00000253363	ENSP00000261973

ENSP00000253363	ENSP00000262710
ENSP00000253363	ENSP00000267812
ENSP00000253363	ENSP00000278572
ENSP00000253363	ENSP00000296015
ENSP00000253363	ENSP00000296702
ENSP00000253363	ENSP00000300413
ENSP00000253363	ENSP00000301740
ENSP00000253363	ENSP00000303191
ENSP00000253363	ENSP00000304370
ENSP00000253363	ENSP00000307863
ENSP00000253363	ENSP00000313829
ENSP00000253363	ENSP00000315379
ENSP00000253363	ENSP00000315859
ENSP00000253363	ENSP00000324274
ENSP00000253363	ENSP00000325120
ENSP00000253363	ENSP00000326261
ENSP00000253363	ENSP00000329466
ENSP00000253363	ENSP00000330188
ENSP00000253363	ENSP00000333275
ENSP00000253363	ENSP00000334148
ENSP00000253363	ENSP00000335321
ENSP00000253363	ENSP00000337340
ENSP00000253363	ENSP00000339723
ENSP00000253363	ENSP00000341885
ENSP00000253363	ENSP00000344456
ENSP00000253363	ENSP00000346015
ENSP00000253363	ENSP00000346725
ENSP00000253363	ENSP00000346762
ENSP00000253363	ENSP00000347592
ENSP00000253363	ENSP00000349594
ENSP00000253363	ENSP00000351894
ENSP00000253363	ENSP00000352257
ENSP00000253363	ENSP00000354673
ENSP00000253363	ENSP00000355050
ENSP00000253363	ENSP00000357631
ENSP00000253363	ENSP00000360286
ENSP00000253363	ENSP00000360464
ENSP00000253363	ENSP00000360497
ENSP00000253363	ENSP00000360532
ENSP00000253363	ENSP00000361162
ENSP00000253363	ENSP00000362820
ENSP00000253363	ENSP00000377262
ENSP00000253363	ENSP00000379607
ENSP00000253363	ENSP00000391432
ENSP00000253363	ENSP00000391723
ENSP00000253363	ENSP00000416959

ENSP00000253413	ENSP00000253794
ENSP00000253413	ENSP00000261758
ENSP00000253413	ENSP00000263856
ENSP00000253413	ENSP00000272238
ENSP00000253413	ENSP00000273398
ENSP00000253413	ENSP00000297265
ENSP00000253413	ENSP00000308944
ENSP00000253413	ENSP00000310440
ENSP00000253413	ENSP00000322706
ENSP00000253413	ENSP00000332247
ENSP00000253413	ENSP00000333873
ENSP00000253413	ENSP00000333946
ENSP00000253413	ENSP00000336927
ENSP00000253413	ENSP00000343313
ENSP00000253413	ENSP00000352522
ENSP00000253413	ENSP00000354673
ENSP00000253413	ENSP00000356723
ENSP00000253413	ENSP00000358478
ENSP00000253413	ENSP00000363162
ENSP00000253413	ENSP00000366513
ENSP00000253413	ENSP00000367299
ENSP00000253413	ENSP00000368439
ENSP00000253413	ENSP00000384675
ENSP00000253413	ENSP00000386722
ENSP00000253413	ENSP00000386738
ENSP00000253413	ENSP00000389934
ENSP00000253413	ENSP00000407487
ENSP00000253457	ENSP00000267750
ENSP00000253457	ENSP00000397435
ENSP00000253457	ENSP00000420608
ENSP00000253458	ENSP00000336524
ENSP00000253458	ENSP00000349049
ENSP00000253458	ENSP00000355231
ENSP00000253458	ENSP00000360532
ENSP00000253458	ENSP00000398597
ENSP00000253458	ENSP00000399511
ENSP00000253462	ENSP00000260810
ENSP00000253462	ENSP00000261881
ENSP00000253462	ENSP00000262460
ENSP00000253462	ENSP00000263201
ENSP00000253462	ENSP00000265056
ENSP00000253462	ENSP00000265140
ENSP00000253462	ENSP00000276533
ENSP00000253462	ENSP00000291386
ENSP00000253462	ENSP00000304370
ENSP00000253462	ENSP00000312870

ENSP00000253462	ENSP00000313420
ENSP00000253462	ENSP00000317872
ENSP00000253462	ENSP00000330875
ENSP00000253462	ENSP00000334148
ENSP00000253462	ENSP00000343741
ENSP00000253462	ENSP00000349298
ENSP00000253462	ENSP00000353793
ENSP00000253462	ENSP00000372023
ENSP00000253462	ENSP00000393596
ENSP00000253462	ENSP00000401018
ENSP00000253462	ENSP00000413632
ENSP00000253490	ENSP00000304593
ENSP00000253490	ENSP00000336842
ENSP00000253490	ENSP00000348163
ENSP00000253490	ENSP00000357887
ENSP00000253669	ENSP00000260372
ENSP00000253669	ENSP00000282058
ENSP00000253669	ENSP00000369871
ENSP00000253680	ENSP00000371341
ENSP00000253686	ENSP00000301585
ENSP00000253686	ENSP00000346120
ENSP00000253693	ENSP00000256474
ENSP00000253693	ENSP00000261211
ENSP00000253693	ENSP00000263228
ENSP00000253693	ENSP00000297265
ENSP00000253693	ENSP00000330408
ENSP00000253693	ENSP00000343190
ENSP00000253693	ENSP00000356405
ENSP00000253693	ENSP00000366607
ENSP00000253699	ENSP00000259400
ENSP00000253699	ENSP00000260818
ENSP00000253699	ENSP00000262039
ENSP00000253699	ENSP00000273375
ENSP00000253699	ENSP00000273398
ENSP00000253699	ENSP00000327650
ENSP00000253699	ENSP00000341680
ENSP00000253699	ENSP00000360183
ENSP00000253699	ENSP00000369810
ENSP00000253719	ENSP00000260187
ENSP00000253719	ENSP00000262215
ENSP00000253719	ENSP00000265304
ENSP00000253719	ENSP00000290649
ENSP00000253719	ENSP00000302239
ENSP00000253719	ENSP00000308944
ENSP00000253719	ENSP00000310440
ENSP00000253719	ENSP00000324560

ENSP00000253719	ENSP00000331201
ENSP00000253719	ENSP00000341551
ENSP00000253719	ENSP00000343435
ENSP00000253719	ENSP00000347495
ENSP00000253719	ENSP00000358478
ENSP00000253719	ENSP00000366395
ENSP00000253719	ENSP00000367299
ENSP00000253788	ENSP00000254037
ENSP00000253788	ENSP00000254940
ENSP00000253788	ENSP00000257829
ENSP00000253788	ENSP00000258455
ENSP00000253788	ENSP00000259469
ENSP00000253788	ENSP00000260102
ENSP00000253788	ENSP00000260443
ENSP00000253788	ENSP00000262584
ENSP00000253788	ENSP00000264995
ENSP00000253788	ENSP00000265100
ENSP00000253788	ENSP00000265245
ENSP00000253788	ENSP00000268616
ENSP00000253788	ENSP00000268661
ENSP00000253788	ENSP00000269499
ENSP00000253788	ENSP00000270625
ENSP00000253788	ENSP00000272418
ENSP00000253788	ENSP00000274242
ENSP00000253788	ENSP00000274606
ENSP00000253788	ENSP00000278572
ENSP00000253788	ENSP00000287038
ENSP00000253788	ENSP00000294189
ENSP00000253788	ENSP00000296674
ENSP00000253788	ENSP00000296802
ENSP00000253788	ENSP00000298283
ENSP00000253788	ENSP00000300151
ENSP00000253788	ENSP00000302886
ENSP00000253788	ENSP00000302896
ENSP00000253788	ENSP00000305702
ENSP00000253788	ENSP00000306548
ENSP00000253788	ENSP00000307889
ENSP00000253788	ENSP00000308897
ENSP00000253788	ENSP00000309334
ENSP00000253788	ENSP00000309830
ENSP00000253788	ENSP00000311028
ENSP00000253788	ENSP00000311430
ENSP00000253788	ENSP00000314348
ENSP00000253788	ENSP00000315791
ENSP00000253788	ENSP00000317376
ENSP00000253788	ENSP00000318646

ENSP00000253788	ENSP00000322419
ENSP00000253788	ENSP00000326981
ENSP00000253788	ENSP00000336702
ENSP00000253788	ENSP00000337839
ENSP00000253788	ENSP00000338573
ENSP00000253788	ENSP00000338862
ENSP00000253788	ENSP00000339095
ENSP00000253788	ENSP00000339795
ENSP00000253788	ENSP00000341885
ENSP00000253788	ENSP00000345156
ENSP00000253788	ENSP00000346015
ENSP00000253788	ENSP00000346022
ENSP00000253788	ENSP00000346027
ENSP00000253788	ENSP00000346037
ENSP00000253788	ENSP00000346050
ENSP00000253788	ENSP00000346088
ENSP00000253788	ENSP00000346120
ENSP00000253788	ENSP00000346725
ENSP00000253788	ENSP00000348010
ENSP00000253788	ENSP00000351596
ENSP00000253788	ENSP00000354040
ENSP00000253788	ENSP00000354525
ENSP00000253788	ENSP00000354739
ENSP00000253788	ENSP00000356290
ENSP00000253788	ENSP00000359345
ENSP00000253788	ENSP00000361076
ENSP00000253788	ENSP00000362153
ENSP00000253788	ENSP00000362306
ENSP00000253788	ENSP00000362744
ENSP00000253788	ENSP00000363018
ENSP00000253788	ENSP00000363559
ENSP00000253788	ENSP00000363676
ENSP00000253788	ENSP00000364320
ENSP00000253788	ENSP00000366519
ENSP00000253788	ENSP00000369757
ENSP00000253788	ENSP00000373404
ENSP00000253788	ENSP00000374390
ENSP00000253788	ENSP00000375730
ENSP00000253788	ENSP00000377640
ENSP00000253788	ENSP00000377865
ENSP00000253788	ENSP00000378160
ENSP00000253788	ENSP00000379607
ENSP00000253788	ENSP00000379888
ENSP00000253788	ENSP00000380156
ENSP00000253788	ENSP00000380982
ENSP00000253788	ENSP00000382392

ENSP00000253788	ENSP00000386717
ENSP00000253788	ENSP00000389103
ENSP00000253788	ENSP00000391723
ENSP00000253788	ENSP00000393393
ENSP00000253788	ENSP00000402338
ENSP00000253788	ENSP00000414321
ENSP00000253792	ENSP00000254942
ENSP00000253792	ENSP00000255465
ENSP00000253792	ENSP00000256216
ENSP00000253792	ENSP00000257829
ENSP00000253792	ENSP00000258886
ENSP00000253792	ENSP00000262193
ENSP00000253792	ENSP00000264335
ENSP00000253792	ENSP00000264705
ENSP00000253792	ENSP00000269980
ENSP00000253792	ENSP00000273075
ENSP00000253792	ENSP00000276603
ENSP00000253792	ENSP00000280346
ENSP00000253792	ENSP00000282050
ENSP00000253792	ENSP00000304592
ENSP00000253792	ENSP00000314129
ENSP00000253792	ENSP00000329466
ENSP00000253792	ENSP00000331201
ENSP00000253792	ENSP00000335304
ENSP00000253792	ENSP00000349142
ENSP00000253792	ENSP00000353741
ENSP00000253792	ENSP00000353804
ENSP00000253792	ENSP00000354347
ENSP00000253792	ENSP00000355153
ENSP00000253792	ENSP00000360268
ENSP00000253792	ENSP00000366482
ENSP00000253792	ENSP00000367615
ENSP00000253792	ENSP00000377527
ENSP00000253792	ENSP00000379607
ENSP00000253792	ENSP00000381654
ENSP00000253792	ENSP00000394382
ENSP00000253794	ENSP00000263856
ENSP00000253794	ENSP00000273398
ENSP00000253794	ENSP00000290330
ENSP00000253794	ENSP00000310440
ENSP00000253794	ENSP00000317468
ENSP00000253794	ENSP00000334061
ENSP00000253794	ENSP00000366565
ENSP00000253794	ENSP00000367299
ENSP00000253796	ENSP00000331746
ENSP00000253801	ENSP00000355944

ENSP00000253801	ENSP00000398789
ENSP00000253861	ENSP00000261531
ENSP00000253861	ENSP00000264380
ENSP00000253861	ENSP00000272427
ENSP00000253861	ENSP00000272972
ENSP00000253861	ENSP00000273666
ENSP00000253861	ENSP00000276416
ENSP00000253861	ENSP00000303554
ENSP00000253861	ENSP00000311505
ENSP00000253861	ENSP00000322170
ENSP00000253861	ENSP00000323377
ENSP00000253861	ENSP00000325506
ENSP00000253861	ENSP00000326514
ENSP00000253861	ENSP00000327650
ENSP00000253861	ENSP00000334100
ENSP00000253861	ENSP00000336524
ENSP00000253861	ENSP00000341538
ENSP00000253861	ENSP00000341680
ENSP00000253861	ENSP00000343313
ENSP00000253861	ENSP00000348168
ENSP00000253861	ENSP00000348215
ENSP00000253861	ENSP00000348602
ENSP00000253861	ENSP00000353564
ENSP00000253861	ENSP00000354519
ENSP00000253861	ENSP00000355231
ENSP00000253861	ENSP00000355721
ENSP00000253861	ENSP00000356231
ENSP00000253861	ENSP00000360532
ENSP00000253861	ENSP00000362399
ENSP00000253861	ENSP00000365630
ENSP00000253861	ENSP00000369318
ENSP00000253861	ENSP00000369860
ENSP00000253861	ENSP00000373399
ENSP00000253861	ENSP00000376268
ENSP00000253861	ENSP00000378736
ENSP00000253861	ENSP00000380450
ENSP00000253861	ENSP00000382177
ENSP00000253861	ENSP00000389934
ENSP00000253861	ENSP00000390783
ENSP00000253861	ENSP00000393596
ENSP00000253861	ENSP00000394071
ENSP00000254029	ENSP00000264515
ENSP00000254029	ENSP00000309555
ENSP00000254029	ENSP00000324122
ENSP00000254029	ENSP00000340896
ENSP00000254029	ENSP00000349640

ENSP00000254029	ENSP00000351490
ENSP00000254029	ENSP00000359976
ENSP00000254029	ENSP00000362413
ENSP00000254029	ENSP00000363944
ENSP00000254029	ENSP00000364037
ENSP00000254029	ENSP00000370150
ENSP00000254029	ENSP00000391457
ENSP00000254029	ENSP00000396439
ENSP00000254029	ENSP00000411851
ENSP00000254035	ENSP00000268668
ENSP00000254035	ENSP00000277865
ENSP00000254035	ENSP00000294053
ENSP00000254035	ENSP00000295962
ENSP00000254035	ENSP00000307786
ENSP00000254035	ENSP00000317159
ENSP00000254035	ENSP00000330945
ENSP00000254035	ENSP00000341838
ENSP00000254035	ENSP00000355587
ENSP00000254035	ENSP00000362413
ENSP00000254035	ENSP00000363873
ENSP00000254037	ENSP00000254940
ENSP00000254037	ENSP00000256897
ENSP00000254037	ENSP00000257177
ENSP00000254037	ENSP00000258455
ENSP00000254037	ENSP00000259469
ENSP00000254037	ENSP00000260102
ENSP00000254037	ENSP00000260210
ENSP00000254037	ENSP00000260443
ENSP00000254037	ENSP00000260956
ENSP00000254037	ENSP00000263629
ENSP00000254037	ENSP00000263657
ENSP00000254037	ENSP00000264995
ENSP00000254037	ENSP00000265100
ENSP00000254037	ENSP00000267750
ENSP00000254037	ENSP00000268802
ENSP00000254037	ENSP00000270625
ENSP00000254037	ENSP00000274606
ENSP00000254037	ENSP00000278572
ENSP00000254037	ENSP00000283109
ENSP00000254037	ENSP00000283875
ENSP00000254037	ENSP00000286835
ENSP00000254037	ENSP00000296577
ENSP00000254037	ENSP00000296702
ENSP00000254037	ENSP00000296783
ENSP00000254037	ENSP00000296786
ENSP00000254037	ENSP00000296802

ENSP00000254037	ENSP00000298746
ENSP00000254037	ENSP00000299601
ENSP00000254037	ENSP00000300151
ENSP00000254037	ENSP00000300413
ENSP00000254037	ENSP00000303279
ENSP00000254037	ENSP00000304350
ENSP00000254037	ENSP00000306548
ENSP00000254037	ENSP00000307525
ENSP00000254037	ENSP00000307863
ENSP00000254037	ENSP00000307889
ENSP00000254037	ENSP00000309334
ENSP00000254037	ENSP00000313350
ENSP00000254037	ENSP00000314311
ENSP00000254037	ENSP00000315674
ENSP00000254037	ENSP00000317992
ENSP00000254037	ENSP00000321449
ENSP00000254037	ENSP00000326411
ENSP00000254037	ENSP00000326806
ENSP00000254037	ENSP00000329558
ENSP00000254037	ENSP00000331310
ENSP00000254037	ENSP00000331369
ENSP00000254037	ENSP00000331817
ENSP00000254037	ENSP00000338788
ENSP00000254037	ENSP00000338862
ENSP00000254037	ENSP00000339095
ENSP00000254037	ENSP00000339795
ENSP00000254037	ENSP00000340019
ENSP00000254037	ENSP00000343081
ENSP00000254037	ENSP00000345917
ENSP00000254037	ENSP00000346015
ENSP00000254037	ENSP00000346050
ENSP00000254037	ENSP00000346088
ENSP00000254037	ENSP00000348168
ENSP00000254037	ENSP00000348596
ENSP00000254037	ENSP00000353246
ENSP00000254037	ENSP00000354525
ENSP00000254037	ENSP00000355471
ENSP00000254037	ENSP00000355572
ENSP00000254037	ENSP00000356134
ENSP00000254037	ENSP00000356146
ENSP00000254037	ENSP00000357748
ENSP00000254037	ENSP00000358563
ENSP00000254037	ENSP00000358812
ENSP00000254037	ENSP00000359606
ENSP00000254037	ENSP00000359688
ENSP00000254037	ENSP00000360412

ENSP00000254037	ENSP00000360473
ENSP00000254037	ENSP00000361076
ENSP00000254037	ENSP00000361092
ENSP00000254037	ENSP00000361982
ENSP00000254037	ENSP00000362036
ENSP00000254037	ENSP00000362105
ENSP00000254037	ENSP00000362413
ENSP00000254037	ENSP00000362900
ENSP00000254037	ENSP00000363384
ENSP00000254037	ENSP00000363559
ENSP00000254037	ENSP00000363676
ENSP00000254037	ENSP00000364119
ENSP00000254037	ENSP00000364289
ENSP00000254037	ENSP00000364293
ENSP00000254037	ENSP00000364543
ENSP00000254037	ENSP00000366135
ENSP00000254037	ENSP00000366519
ENSP00000254037	ENSP00000366557
ENSP00000254037	ENSP00000368565
ENSP00000254037	ENSP00000368801
ENSP00000254037	ENSP00000369757
ENSP00000254037	ENSP00000371169
ENSP00000254037	ENSP00000372827
ENSP00000254037	ENSP00000377344
ENSP00000254037	ENSP00000377640
ENSP00000254037	ENSP00000377865
ENSP00000254037	ENSP00000377944
ENSP00000254037	ENSP00000378160
ENSP00000254037	ENSP00000379888
ENSP00000254037	ENSP00000380982
ENSP00000254037	ENSP00000382392
ENSP00000254037	ENSP00000384302
ENSP00000254037	ENSP00000386134
ENSP00000254037	ENSP00000386717
ENSP00000254037	ENSP00000387170
ENSP00000254037	ENSP00000387219
ENSP00000254037	ENSP00000388464
ENSP00000254037	ENSP00000393393
ENSP00000254037	ENSP00000394400
ENSP00000254037	ENSP00000399530
ENSP00000254037	ENSP00000400626
ENSP00000254037	ENSP00000402338
ENSP00000254037	ENSP00000412310
ENSP00000254037	ENSP00000417229
ENSP00000254037	ENSP00000419599
ENSP00000254037	ENSP00000419851

ENSP00000254051	ENSP00000267101
ENSP00000254079	ENSP00000365025
ENSP00000254101	ENSP00000260270
ENSP00000254101	ENSP00000261712
ENSP00000254101	ENSP00000261819
ENSP00000254101	ENSP00000265857
ENSP00000254101	ENSP00000285021
ENSP00000254101	ENSP00000286070
ENSP00000254101	ENSP00000287239
ENSP00000254101	ENSP00000287878
ENSP00000254101	ENSP00000294724
ENSP00000254101	ENSP00000295685
ENSP00000254101	ENSP00000300648
ENSP00000254101	ENSP00000305958
ENSP00000254101	ENSP00000310842
ENSP00000254101	ENSP00000313420
ENSP00000254101	ENSP00000317904
ENSP00000254101	ENSP00000324560
ENSP00000254101	ENSP00000327145
ENSP00000254101	ENSP00000343745
ENSP00000254101	ENSP00000347134
ENSP00000254101	ENSP00000348215
ENSP00000254101	ENSP00000349594
ENSP00000254101	ENSP00000351908
ENSP00000254101	ENSP00000354347
ENSP00000254101	ENSP00000356489
ENSP00000254101	ENSP00000360112
ENSP00000254101	ENSP00000361310
ENSP00000254101	ENSP00000404812
ENSP00000254193	ENSP00000256854
ENSP00000254193	ENSP00000260129
ENSP00000254193	ENSP00000260210
ENSP00000254193	ENSP00000261531
ENSP00000254193	ENSP00000261973
ENSP00000254193	ENSP00000262460
ENSP00000254193	ENSP00000262962
ENSP00000254193	ENSP00000262982
ENSP00000254193	ENSP00000263073
ENSP00000254193	ENSP00000264670
ENSP00000254193	ENSP00000264883
ENSP00000254193	ENSP00000265753
ENSP00000254193	ENSP00000266079
ENSP00000254193	ENSP00000266735
ENSP00000254193	ENSP00000267812
ENSP00000254193	ENSP00000268482
ENSP00000254193	ENSP00000268483

ENSP00000254193	ENSP00000271628
ENSP00000254193	ENSP00000274712
ENSP00000254193	ENSP00000276570
ENSP00000254193	ENSP00000292476
ENSP00000254193	ENSP00000293406
ENSP00000254193	ENSP00000296702
ENSP00000254193	ENSP00000300413
ENSP00000254193	ENSP00000301740
ENSP00000254193	ENSP00000301764
ENSP00000254193	ENSP00000302160
ENSP00000254193	ENSP00000303191
ENSP00000254193	ENSP00000304350
ENSP00000254193	ENSP00000304370
ENSP00000254193	ENSP00000305790
ENSP00000254193	ENSP00000307684
ENSP00000254193	ENSP00000307863
ENSP00000254193	ENSP00000308533
ENSP00000254193	ENSP00000308546
ENSP00000254193	ENSP00000310448
ENSP00000254193	ENSP00000310723
ENSP00000254193	ENSP00000311135
ENSP00000254193	ENSP00000314311
ENSP00000254193	ENSP00000315035
ENSP00000254193	ENSP00000315379
ENSP00000254193	ENSP00000318195
ENSP00000254193	ENSP00000318861
ENSP00000254193	ENSP00000319341
ENSP00000254193	ENSP00000323302
ENSP00000254193	ENSP00000324122
ENSP00000254193	ENSP00000325677
ENSP00000254193	ENSP00000326261
ENSP00000254193	ENSP00000326806
ENSP00000254193	ENSP00000331817
ENSP00000254193	ENSP00000331907
ENSP00000254193	ENSP00000332103
ENSP00000254193	ENSP00000334148
ENSP00000254193	ENSP00000335321
ENSP00000254193	ENSP00000339090
ENSP00000254193	ENSP00000340766
ENSP00000254193	ENSP00000342374
ENSP00000254193	ENSP00000343081
ENSP00000254193	ENSP00000343557
ENSP00000254193	ENSP00000344241
ENSP00000254193	ENSP00000346986
ENSP00000254193	ENSP00000347087
ENSP00000254193	ENSP00000348010

ENSP00000254193	ENSP00000348722
ENSP00000254193	ENSP00000351137
ENSP00000254193	ENSP00000352738
ENSP00000254193	ENSP00000354522
ENSP00000254193	ENSP00000355261
ENSP00000254193	ENSP00000356405
ENSP00000254193	ENSP00000357631
ENSP00000254193	ENSP00000358605
ENSP00000254193	ENSP00000360515
ENSP00000254193	ENSP00000360532
ENSP00000254193	ENSP00000360876
ENSP00000254193	ENSP00000360992
ENSP00000254193	ENSP00000361949
ENSP00000254193	ENSP00000362110
ENSP00000254193	ENSP00000362900
ENSP00000254193	ENSP00000362936
ENSP00000254193	ENSP00000363313
ENSP00000254193	ENSP00000363734
ENSP00000254193	ENSP00000364289
ENSP00000254193	ENSP00000366557
ENSP00000254193	ENSP00000366843
ENSP00000254193	ENSP00000367030
ENSP00000254193	ENSP00000367835
ENSP00000254193	ENSP00000368438
ENSP00000254193	ENSP00000370083
ENSP00000254193	ENSP00000370119
ENSP00000254193	ENSP00000370460
ENSP00000254193	ENSP00000370589
ENSP00000254193	ENSP00000372694
ENSP00000254193	ENSP00000376309
ENSP00000254193	ENSP00000376973
ENSP00000254193	ENSP00000387006
ENSP00000254193	ENSP00000400591
ENSP00000254193	ENSP00000401371
ENSP00000254193	ENSP00000412319
ENSP00000254193	ENSP00000412566
ENSP00000254193	ENSP00000415678
ENSP00000254193	ENSP00000416959
ENSP00000254193	ENSP00000420195
ENSP00000254250	ENSP00000272139
ENSP00000254250	ENSP00000305503
ENSP00000254250	ENSP00000307863
ENSP00000254250	ENSP00000309555
ENSP00000254250	ENSP00000309606
ENSP00000254250	ENSP00000324274
ENSP00000254250	ENSP00000328088

ENSP00000254250	ENSP00000352185
ENSP00000254250	ENSP00000354951
ENSP00000254250	ENSP00000360464
ENSP00000254250	ENSP00000360532
ENSP00000254250	ENSP00000362824
ENSP00000254301	ENSP00000262776
ENSP00000254301	ENSP00000304364
ENSP00000254301	ENSP00000307387
ENSP00000254301	ENSP00000321706
ENSP00000254301	ENSP00000358918
ENSP00000254301	ENSP00000367064
ENSP00000254301	ENSP00000381031
ENSP00000254301	ENSP00000387426
ENSP00000254325	ENSP00000257934
ENSP00000254325	ENSP00000262367
ENSP00000254325	ENSP00000264758
ENSP00000254325	ENSP00000265717
ENSP00000254325	ENSP00000265990
ENSP00000254325	ENSP00000267064
ENSP00000254325	ENSP00000268057
ENSP00000254325	ENSP00000276072
ENSP00000254325	ENSP00000294168
ENSP00000254325	ENSP00000300738
ENSP00000254325	ENSP00000302955
ENSP00000254325	ENSP00000307208
ENSP00000254325	ENSP00000322570
ENSP00000254325	ENSP00000323880
ENSP00000254325	ENSP00000332171
ENSP00000254325	ENSP00000333920
ENSP00000254325	ENSP00000341243
ENSP00000254325	ENSP00000341551
ENSP00000254325	ENSP00000343741
ENSP00000254325	ENSP00000344537
ENSP00000254325	ENSP00000349213
ENSP00000254325	ENSP00000350708
ENSP00000254325	ENSP00000351284
ENSP00000254325	ENSP00000351697
ENSP00000254325	ENSP00000353458
ENSP00000254325	ENSP00000360532
ENSP00000254325	ENSP00000366974
ENSP00000254325	ENSP00000368349
ENSP00000254325	ENSP00000368438
ENSP00000254325	ENSP00000368646
ENSP00000254325	ENSP00000372023
ENSP00000254325	ENSP00000375986
ENSP00000254336	ENSP00000264389

ENSP00000254336	ENSP00000311134
ENSP00000254336	ENSP00000337289
ENSP00000254336	ENSP00000362807
ENSP00000254336	ENSP00000381031
ENSP00000254336	ENSP00000397394
ENSP00000254337	ENSP00000301764
ENSP00000254351	ENSP00000260130
ENSP00000254351	ENSP00000307046
ENSP00000254351	ENSP00000341170
ENSP00000254351	ENSP00000344468
ENSP00000254351	ENSP00000367408
ENSP00000254442	ENSP00000256398
ENSP00000254442	ENSP00000264158
ENSP00000254442	ENSP00000264515
ENSP00000254442	ENSP00000264951
ENSP00000254442	ENSP00000266126
ENSP00000254442	ENSP00000282007
ENSP00000254442	ENSP00000290541
ENSP00000254442	ENSP00000310596
ENSP00000254442	ENSP00000320949
ENSP00000254442	ENSP00000331817
ENSP00000254442	ENSP00000340896
ENSP00000254442	ENSP00000356811
ENSP00000254442	ENSP00000360310
ENSP00000254442	ENSP00000361433
ENSP00000254521	ENSP00000256319
ENSP00000254521	ENSP00000260433
ENSP00000254521	ENSP00000261507
ENSP00000254521	ENSP00000264027
ENSP00000254521	ENSP00000265896
ENSP00000254521	ENSP00000269703
ENSP00000254521	ENSP00000272163
ENSP00000254521	ENSP00000277010
ENSP00000254521	ENSP00000285949
ENSP00000254521	ENSP00000304802
ENSP00000254521	ENSP00000310649
ENSP00000254521	ENSP00000310721
ENSP00000254521	ENSP00000320604
ENSP00000254521	ENSP00000325425
ENSP00000254521	ENSP00000330812
ENSP00000254521	ENSP00000341662
ENSP00000254521	ENSP00000348380
ENSP00000254521	ENSP00000348762
ENSP00000254521	ENSP00000356737
ENSP00000254521	ENSP00000357650
ENSP00000254521	ENSP00000360918

ENSP00000254521	ENSP00000380184
ENSP00000254521	ENSP00000383901
ENSP00000254579	ENSP00000260731
ENSP00000254579	ENSP00000295951
ENSP00000254579	ENSP00000296509
ENSP00000254579	ENSP00000300249
ENSP00000254579	ENSP00000360310
ENSP00000254579	ENSP00000377793
ENSP00000254605	ENSP00000256319
ENSP00000254605	ENSP00000260129
ENSP00000254605	ENSP00000262315
ENSP00000254605	ENSP00000264670
ENSP00000254605	ENSP00000270625
ENSP00000254605	ENSP00000290158
ENSP00000254605	ENSP00000298746
ENSP00000254605	ENSP00000309433
ENSP00000254605	ENSP00000310042
ENSP00000254605	ENSP00000312981
ENSP00000254605	ENSP00000329466
ENSP00000254605	ENSP00000333934
ENSP00000254605	ENSP00000343701
ENSP00000254605	ENSP00000346120
ENSP00000254605	ENSP00000347087
ENSP00000254605	ENSP00000348107
ENSP00000254605	ENSP00000352678
ENSP00000254605	ENSP00000352738
ENSP00000254605	ENSP00000356548
ENSP00000254605	ENSP00000356989
ENSP00000254605	ENSP00000358563
ENSP00000254605	ENSP00000361433
ENSP00000254605	ENSP00000362105
ENSP00000254605	ENSP00000364289
ENSP00000254605	ENSP00000365811
ENSP00000254605	ENSP00000365877
ENSP00000254605	ENSP00000366135
ENSP00000254605	ENSP00000369042
ENSP00000254605	ENSP00000370589
ENSP00000254605	ENSP00000374390
ENSP00000254605	ENSP00000379607
ENSP00000254605	ENSP00000379888
ENSP00000254605	ENSP00000380982
ENSP00000254605	ENSP00000419740
ENSP00000254616	ENSP00000257245
ENSP00000254616	ENSP00000295767
ENSP00000254616	ENSP00000320236
ENSP00000254616	ENSP00000364699

ENSP00000254624	ENSP00000261211
ENSP00000254624	ENSP00000397435
ENSP00000254630	ENSP00000301585
ENSP00000254630	ENSP00000329466
ENSP00000254630	ENSP00000331201
ENSP00000254630	ENSP00000336790
ENSP00000254630	ENSP00000362208
ENSP00000254630	ENSP00000373570
ENSP00000254654	ENSP00000262039
ENSP00000254654	ENSP00000299424
ENSP00000254654	ENSP00000301364
ENSP00000254654	ENSP00000331201
ENSP00000254654	ENSP00000343190
ENSP00000254654	ENSP00000349205
ENSP00000254654	ENSP00000356530
ENSP00000254654	ENSP00000360268
ENSP00000254654	ENSP00000372023
ENSP00000254654	ENSP00000375986
ENSP00000254654	ENSP00000378306
ENSP00000254654	ENSP00000401445
ENSP00000254661	ENSP00000331746
ENSP00000254691	ENSP00000269280
ENSP00000254695	ENSP00000264335
ENSP00000254695	ENSP00000313059
ENSP00000254719	ENSP00000255078
ENSP00000254719	ENSP00000255465
ENSP00000254719	ENSP00000256442
ENSP00000254719	ENSP00000256996
ENSP00000254719	ENSP00000258774
ENSP00000254719	ENSP00000259008
ENSP00000254719	ENSP00000259569
ENSP00000254719	ENSP00000260810
ENSP00000254719	ENSP00000260956
ENSP00000254719	ENSP00000261424
ENSP00000254719	ENSP00000261881
ENSP00000254719	ENSP00000262315
ENSP00000254719	ENSP00000262460
ENSP00000254719	ENSP00000262518
ENSP00000254719	ENSP00000262794
ENSP00000254719	ENSP00000262803
ENSP00000254719	ENSP00000263073
ENSP00000254719	ENSP00000263187
ENSP00000254719	ENSP00000263201
ENSP00000254719	ENSP00000263208
ENSP00000254719	ENSP00000263795
ENSP00000254719	ENSP00000263857

ENSP00000254719	ENSP00000264335
ENSP00000254719	ENSP00000264926
ENSP00000254719	ENSP00000265056
ENSP00000254719	ENSP00000265073
ENSP00000254719	ENSP00000265081
ENSP00000254719	ENSP00000265304
ENSP00000254719	ENSP00000265351
ENSP00000254719	ENSP00000265465
ENSP00000254719	ENSP00000265728
ENSP00000254719	ENSP00000265963
ENSP00000254719	ENSP00000267064
ENSP00000254719	ENSP00000267430
ENSP00000254719	ENSP00000274311
ENSP00000254719	ENSP00000276533
ENSP00000254719	ENSP00000278412
ENSP00000254719	ENSP00000278616
ENSP00000254719	ENSP00000281708
ENSP00000254719	ENSP00000286428
ENSP00000254719	ENSP00000287598
ENSP00000254719	ENSP00000290158
ENSP00000254719	ENSP00000296137
ENSP00000254719	ENSP00000296273
ENSP00000254719	ENSP00000296509
ENSP00000254719	ENSP00000297185
ENSP00000254719	ENSP00000298139
ENSP00000254719	ENSP00000298532
ENSP00000254719	ENSP00000299206
ENSP00000254719	ENSP00000300648
ENSP00000254719	ENSP00000301280
ENSP00000254719	ENSP00000302530
ENSP00000254719	ENSP00000307004
ENSP00000254719	ENSP00000309572
ENSP00000254719	ENSP00000310520
ENSP00000254719	ENSP00000311873
ENSP00000254719	ENSP00000312735
ENSP00000254719	ENSP00000313171
ENSP00000254719	ENSP00000313350
ENSP00000254719	ENSP00000313420
ENSP00000254719	ENSP00000314949
ENSP00000254719	ENSP00000314971
ENSP00000254719	ENSP00000317039
ENSP00000254719	ENSP00000318822
ENSP00000254719	ENSP00000318977
ENSP00000254719	ENSP00000321636
ENSP00000254719	ENSP00000322419
ENSP00000254719	ENSP00000322570

ENSP00000254719	ENSP00000323099
ENSP00000254719	ENSP00000323439
ENSP00000254719	ENSP00000323663
ENSP00000254719	ENSP00000324124
ENSP00000254719	ENSP00000325863
ENSP00000254719	ENSP00000326819
ENSP00000254719	ENSP00000329002
ENSP00000254719	ENSP00000331111
ENSP00000254719	ENSP00000332171
ENSP00000254719	ENSP00000336606
ENSP00000254719	ENSP00000336712
ENSP00000254719	ENSP00000336868
ENSP00000254719	ENSP00000337088
ENSP00000254719	ENSP00000338990
ENSP00000254719	ENSP00000339826
ENSP00000254719	ENSP00000340766
ENSP00000254719	ENSP00000340879
ENSP00000254719	ENSP00000341243
ENSP00000254719	ENSP00000341382
ENSP00000254719	ENSP00000343392
ENSP00000254719	ENSP00000343741
ENSP00000254719	ENSP00000346453
ENSP00000254719	ENSP00000347168
ENSP00000254719	ENSP00000347232
ENSP00000254719	ENSP00000347978
ENSP00000254719	ENSP00000348089
ENSP00000254719	ENSP00000350283
ENSP00000254719	ENSP00000350491
ENSP00000254719	ENSP00000350767
ENSP00000254719	ENSP00000351284
ENSP00000254719	ENSP00000351524
ENSP00000254719	ENSP00000352257
ENSP00000254719	ENSP00000354361
ENSP00000254719	ENSP00000354720
ENSP00000254719	ENSP00000354957
ENSP00000254719	ENSP00000355205
ENSP00000254719	ENSP00000355261
ENSP00000254719	ENSP00000357283
ENSP00000254719	ENSP00000360532
ENSP00000254719	ENSP00000360817
ENSP00000254719	ENSP00000361465
ENSP00000254719	ENSP00000362413
ENSP00000254719	ENSP00000363021
ENSP00000254719	ENSP00000364270
ENSP00000254719	ENSP00000364320
ENSP00000254719	ENSP00000366974

ENSP00000254719	ENSP00000367029
ENSP00000254719	ENSP00000367034
ENSP00000254719	ENSP00000367910
ENSP00000254719	ENSP00000368349
ENSP00000254719	ENSP00000368438
ENSP00000254719	ENSP00000368881
ENSP00000254719	ENSP00000369335
ENSP00000254719	ENSP00000369411
ENSP00000254719	ENSP00000369497
ENSP00000254719	ENSP00000370150
ENSP00000254719	ENSP00000370151
ENSP00000254719	ENSP00000371475
ENSP00000254719	ENSP00000372023
ENSP00000254719	ENSP00000373952
ENSP00000254719	ENSP00000374213
ENSP00000254719	ENSP00000375863
ENSP00000254719	ENSP00000378295
ENSP00000254719	ENSP00000379607
ENSP00000254719	ENSP00000379865
ENSP00000254719	ENSP00000380460
ENSP00000254719	ENSP00000380921
ENSP00000254719	ENSP00000383503
ENSP00000254719	ENSP00000396704
ENSP00000254719	ENSP00000397636
ENSP00000254719	ENSP00000403852
ENSP00000254719	ENSP00000405636
ENSP00000254719	ENSP00000410530
ENSP00000254719	ENSP00000410954
ENSP00000254719	ENSP00000414110
ENSP00000254719	ENSP00000416599
ENSP00000254719	ENSP00000420588
ENSP00000254730	ENSP00000254942
ENSP00000254730	ENSP00000261531
ENSP00000254730	ENSP00000278572
ENSP00000254730	ENSP00000302896
ENSP00000254730	ENSP00000326981
ENSP00000254730	ENSP00000341082
ENSP00000254759	ENSP00000262030
ENSP00000254759	ENSP00000265896
ENSP00000254759	ENSP00000267430
ENSP00000254759	ENSP00000277010
ENSP00000254759	ENSP00000300452
ENSP00000254759	ENSP00000307853
ENSP00000254759	ENSP00000310520
ENSP00000254759	ENSP00000310547
ENSP00000254759	ENSP00000322316

ENSP00000254759	ENSP00000333946
ENSP00000254759	ENSP00000353698
ENSP00000254759	ENSP00000355517
ENSP00000254759	ENSP00000355739
ENSP00000254759	ENSP00000360183
ENSP00000254770	ENSP00000254942
ENSP00000254770	ENSP00000300086
ENSP00000254799	ENSP00000301585
ENSP00000254799	ENSP00000336712
ENSP00000254799	ENSP00000379607
ENSP00000254803	ENSP00000254846
ENSP00000254803	ENSP00000261708
ENSP00000254803	ENSP00000306983
ENSP00000254803	ENSP00000311513
ENSP00000254803	ENSP00000324122
ENSP00000254803	ENSP00000327179
ENSP00000254803	ENSP00000328854
ENSP00000254803	ENSP00000338703
ENSP00000254803	ENSP00000341280
ENSP00000254803	ENSP00000355629
ENSP00000254803	ENSP00000356903
ENSP00000254803	ENSP00000363313
ENSP00000254803	ENSP00000369695
ENSP00000254803	ENSP00000370589
ENSP00000254803	ENSP00000382688
ENSP00000254803	ENSP00000402338
ENSP00000254803	ENSP00000415151
ENSP00000254816	ENSP00000254942
ENSP00000254816	ENSP00000302120
ENSP00000254816	ENSP00000360116
ENSP00000254846	ENSP00000262265
ENSP00000254846	ENSP00000262519
ENSP00000254846	ENSP00000264515
ENSP00000254846	ENSP00000267064
ENSP00000254846	ENSP00000267197
ENSP00000254846	ENSP00000280350
ENSP00000254846	ENSP00000298292
ENSP00000254846	ENSP00000312697
ENSP00000254846	ENSP00000341551
ENSP00000254846	ENSP00000347168
ENSP00000254846	ENSP00000350767
ENSP00000254846	ENSP00000357887
ENSP00000254846	ENSP00000365891
ENSP00000254846	ENSP00000366974
ENSP00000254846	ENSP00000367034
ENSP00000254846	ENSP00000371546

ENSP00000254846	ENSP00000373340
ENSP00000254846	ENSP00000386759
ENSP00000254878	ENSP00000300107
ENSP00000254878	ENSP00000308339
ENSP00000254878	ENSP00000310551
ENSP00000254878	ENSP00000339245
ENSP00000254878	ENSP00000348554
ENSP00000254878	ENSP00000354876
ENSP00000254878	ENSP00000364912
ENSP00000254878	ENSP00000368698
ENSP00000254878	ENSP00000371846
ENSP00000254878	ENSP00000375986
ENSP00000254878	ENSP00000377865
ENSP00000254878	ENSP00000387262
ENSP00000254878	ENSP00000417281
ENSP00000254900	ENSP00000256398
ENSP00000254900	ENSP00000256897
ENSP00000254900	ENSP00000261531
ENSP00000254900	ENSP00000261819
ENSP00000254900	ENSP00000263620
ENSP00000254900	ENSP00000263974
ENSP00000254900	ENSP00000264183
ENSP00000254900	ENSP00000264193
ENSP00000254900	ENSP00000264731
ENSP00000254900	ENSP00000265990
ENSP00000254900	ENSP00000269127
ENSP00000254900	ENSP00000276072
ENSP00000254900	ENSP00000278412
ENSP00000254900	ENSP00000279873
ENSP00000254900	ENSP00000283882
ENSP00000254900	ENSP00000287820
ENSP00000254900	ENSP00000299424
ENSP00000254900	ENSP00000303315
ENSP00000254900	ENSP00000306983
ENSP00000254900	ENSP00000307208
ENSP00000254900	ENSP00000312697
ENSP00000254900	ENSP00000316114
ENSP00000254900	ENSP00000319169
ENSP00000254900	ENSP00000320566
ENSP00000254900	ENSP00000322579
ENSP00000254900	ENSP00000323720
ENSP00000254900	ENSP00000329933
ENSP00000254900	ENSP00000331310
ENSP00000254900	ENSP00000334061
ENSP00000254900	ENSP00000335044
ENSP00000254900	ENSP00000343741

ENSP00000254900	ENSP00000347602
ENSP00000254900	ENSP00000347733
ENSP00000254900	ENSP00000349213
ENSP00000254900	ENSP00000350078
ENSP00000254900	ENSP00000350967
ENSP00000254900	ENSP00000351490
ENSP00000254900	ENSP00000353622
ENSP00000254900	ENSP00000354633
ENSP00000254900	ENSP00000354673
ENSP00000254900	ENSP00000355205
ENSP00000254900	ENSP00000356530
ENSP00000254900	ENSP00000357887
ENSP00000254900	ENSP00000359290
ENSP00000254900	ENSP00000359518
ENSP00000254900	ENSP00000360532
ENSP00000254900	ENSP00000360621
ENSP00000254900	ENSP00000360676
ENSP00000254900	ENSP00000361998
ENSP00000254900	ENSP00000362068
ENSP00000254900	ENSP00000363779
ENSP00000254900	ENSP00000369695
ENSP00000254900	ENSP00000372023
ENSP00000254900	ENSP00000373340
ENSP00000254900	ENSP00000374213
ENSP00000254900	ENSP00000377747
ENSP00000254900	ENSP00000381657
ENSP00000254900	ENSP00000392028
ENSP00000254900	ENSP00000401445
ENSP00000254900	ENSP00000402084
ENSP00000254908	ENSP00000258648
ENSP00000254908	ENSP00000259667
ENSP00000254908	ENSP00000261531
ENSP00000254908	ENSP00000266126
ENSP00000254908	ENSP00000268058
ENSP00000254908	ENSP00000282388
ENSP00000254908	ENSP00000282892
ENSP00000254908	ENSP00000283875
ENSP00000254908	ENSP00000284957
ENSP00000254908	ENSP00000286317
ENSP00000254908	ENSP00000297347
ENSP00000254908	ENSP00000301280
ENSP00000254908	ENSP00000304292
ENSP00000254908	ENSP00000305502
ENSP00000254908	ENSP00000329029
ENSP00000254908	ENSP00000340836
ENSP00000254908	ENSP00000348107

ENSP00000254908	ENSP00000357448
ENSP00000254908	ENSP00000359837
ENSP00000254908	ENSP00000360532
ENSP00000254908	ENSP00000377665
ENSP00000254908	ENSP00000394869
ENSP00000254928	ENSP00000301585
ENSP00000254928	ENSP00000302896
ENSP00000254928	ENSP00000326981
ENSP00000254928	ENSP00000353826
ENSP00000254928	ENSP00000355471
ENSP00000254928	ENSP00000356134
ENSP00000254928	ENSP00000360286
ENSP00000254928	ENSP00000360532
ENSP00000254928	ENSP00000362576
ENSP00000254940	ENSP00000260956
ENSP00000254940	ENSP00000261015
ENSP00000254940	ENSP00000263512
ENSP00000254940	ENSP00000264230
ENSP00000254940	ENSP00000264670
ENSP00000254940	ENSP00000264995
ENSP00000254940	ENSP00000265245
ENSP00000254940	ENSP00000268616
ENSP00000254940	ENSP00000268661
ENSP00000254940	ENSP00000269499
ENSP00000254940	ENSP00000273861
ENSP00000254940	ENSP00000274606
ENSP00000254940	ENSP00000278856
ENSP00000254940	ENSP00000296411
ENSP00000254940	ENSP00000296802
ENSP00000254940	ENSP00000302886
ENSP00000254940	ENSP00000305702
ENSP00000254940	ENSP00000314311
ENSP00000254940	ENSP00000314348
ENSP00000254940	ENSP00000317232
ENSP00000254940	ENSP00000317992
ENSP00000254940	ENSP00000322396
ENSP00000254940	ENSP00000323858
ENSP00000254940	ENSP00000325312
ENSP00000254940	ENSP00000328690
ENSP00000254940	ENSP00000332340
ENSP00000254940	ENSP00000334594
ENSP00000254940	ENSP00000337839
ENSP00000254940	ENSP00000338573
ENSP00000254940	ENSP00000338862
ENSP00000254940	ENSP00000339145
ENSP00000254940	ENSP00000339795

ENSP00000254940	ENSP00000345156
ENSP00000254940	ENSP00000346120
ENSP00000254940	ENSP00000346236
ENSP00000254940	ENSP00000346725
ENSP00000254940	ENSP00000352021
ENSP00000254940	ENSP00000352308
ENSP00000254940	ENSP00000352738
ENSP00000254940	ENSP00000354040
ENSP00000254940	ENSP00000358716
ENSP00000254940	ENSP00000359305
ENSP00000254940	ENSP00000359345
ENSP00000254940	ENSP00000359688
ENSP00000254940	ENSP00000360412
ENSP00000254940	ENSP00000360828
ENSP00000254940	ENSP00000361076
ENSP00000254940	ENSP00000361232
ENSP00000254940	ENSP00000362153
ENSP00000254940	ENSP00000363018
ENSP00000254940	ENSP00000363559
ENSP00000254940	ENSP00000364320
ENSP00000254940	ENSP00000365806
ENSP00000254940	ENSP00000366519
ENSP00000254940	ENSP00000368887
ENSP00000254940	ENSP00000370616
ENSP00000254940	ENSP00000371201
ENSP00000254940	ENSP00000372199
ENSP00000254940	ENSP00000373090
ENSP00000254940	ENSP00000377865
ENSP00000254940	ENSP00000379607
ENSP00000254940	ENSP00000379888
ENSP00000254940	ENSP00000380495
ENSP00000254940	ENSP00000380982
ENSP00000254940	ENSP00000382392
ENSP00000254940	ENSP00000388464
ENSP00000254940	ENSP00000389103
ENSP00000254940	ENSP00000394290
ENSP00000254940	ENSP00000401177
ENSP00000254940	ENSP00000402338
ENSP00000254940	ENSP00000403576
ENSP00000254940	ENSP00000409074
ENSP00000254940	ENSP00000409367
ENSP00000254940	ENSP00000411162
ENSP00000254940	ENSP00000413152
ENSP00000254940	ENSP00000419740
ENSP00000254942	ENSP00000257789
ENSP00000254942	ENSP00000260283

ENSP00000254942	ENSP00000261173
ENSP00000254942	ENSP00000262238
ENSP00000254942	ENSP00000263274
ENSP00000254942	ENSP00000264157
ENSP00000254942	ENSP00000265175
ENSP00000254942	ENSP00000265433
ENSP00000254942	ENSP00000267415
ENSP00000254942	ENSP00000268058
ENSP00000254942	ENSP00000269932
ENSP00000254942	ENSP00000271628
ENSP00000254942	ENSP00000272444
ENSP00000254942	ENSP00000276603
ENSP00000254942	ENSP00000278616
ENSP00000254942	ENSP00000282007
ENSP00000254942	ENSP00000290158
ENSP00000254942	ENSP00000291688
ENSP00000254942	ENSP00000294008
ENSP00000254942	ENSP00000294053
ENSP00000254942	ENSP00000294740
ENSP00000254942	ENSP00000297591
ENSP00000254942	ENSP00000298139
ENSP00000254942	ENSP00000299427
ENSP00000254942	ENSP00000299767
ENSP00000254942	ENSP00000300086
ENSP00000254942	ENSP00000301011
ENSP00000254942	ENSP00000301740
ENSP00000254942	ENSP00000301764
ENSP00000254942	ENSP00000302647
ENSP00000254942	ENSP00000303042
ENSP00000254942	ENSP00000305631
ENSP00000254942	ENSP00000307853
ENSP00000254942	ENSP00000307863
ENSP00000254942	ENSP00000307889
ENSP00000254942	ENSP00000311135
ENSP00000254942	ENSP00000311579
ENSP00000254942	ENSP00000311816
ENSP00000254942	ENSP00000314311
ENSP00000254942	ENSP00000318195
ENSP00000254942	ENSP00000318262
ENSP00000254942	ENSP00000319169
ENSP00000254942	ENSP00000319240
ENSP00000254942	ENSP00000319501
ENSP00000254942	ENSP00000320503
ENSP00000254942	ENSP00000321259
ENSP00000254942	ENSP00000321636
ENSP00000254942	ENSP00000323435

ENSP00000254942	ENSP00000325677
ENSP00000254942	ENSP00000325690
ENSP00000254942	ENSP00000325863
ENSP00000254942	ENSP00000328187
ENSP00000254942	ENSP00000328671
ENSP00000254942	ENSP00000328690
ENSP00000254942	ENSP00000328773
ENSP00000254942	ENSP00000328992
ENSP00000254942	ENSP00000329360
ENSP00000254942	ENSP00000332013
ENSP00000254942	ENSP00000332325
ENSP00000254942	ENSP00000334040
ENSP00000254942	ENSP00000334836
ENSP00000254942	ENSP00000340434
ENSP00000254942	ENSP00000340466
ENSP00000254942	ENSP00000342502
ENSP00000254942	ENSP00000343023
ENSP00000254942	ENSP00000343313
ENSP00000254942	ENSP00000343657
ENSP00000254942	ENSP00000344547
ENSP00000254942	ENSP00000345917
ENSP00000254942	ENSP00000346065
ENSP00000254942	ENSP00000346120
ENSP00000254942	ENSP00000346634
ENSP00000254942	ENSP00000347232
ENSP00000254942	ENSP00000347592
ENSP00000254942	ENSP00000349393
ENSP00000254942	ENSP00000350249
ENSP00000254942	ENSP00000350283
ENSP00000254942	ENSP00000351697
ENSP00000254942	ENSP00000352257
ENSP00000254942	ENSP00000352738
ENSP00000254942	ENSP00000354620
ENSP00000254942	ENSP00000354720
ENSP00000254942	ENSP00000354826
ENSP00000254942	ENSP00000355086
ENSP00000254942	ENSP00000355922
ENSP00000254942	ENSP00000357711
ENSP00000254942	ENSP00000358081
ENSP00000254942	ENSP00000358563
ENSP00000254942	ENSP00000358576
ENSP00000254942	ENSP00000359988
ENSP00000254942	ENSP00000360497
ENSP00000254942	ENSP00000360621
ENSP00000254942	ENSP00000361202
ENSP00000254942	ENSP00000361894

ENSP00000254942	ENSP00000361949
ENSP00000254942	ENSP00000362170
ENSP00000254942	ENSP00000362532
ENSP00000254942	ENSP00000367406
ENSP00000254942	ENSP00000368646
ENSP00000254942	ENSP00000369757
ENSP00000254942	ENSP00000369887
ENSP00000254942	ENSP00000370125
ENSP00000254942	ENSP00000370377
ENSP00000254942	ENSP00000371475
ENSP00000254942	ENSP00000376903
ENSP00000254942	ENSP00000377040
ENSP00000254942	ENSP00000377496
ENSP00000254942	ENSP00000379372
ENSP00000254942	ENSP00000379654
ENSP00000254942	ENSP00000379888
ENSP00000254942	ENSP00000384160
ENSP00000254942	ENSP00000392028
ENSP00000254942	ENSP00000394390
ENSP00000254942	ENSP00000395535
ENSP00000254942	ENSP00000396439
ENSP00000254942	ENSP00000400476
ENSP00000254942	ENSP00000406888
ENSP00000254942	ENSP00000417281
ENSP00000254963	ENSP00000361433
ENSP00000255078	ENSP00000268043
ENSP00000255078	ENSP00000286835
ENSP00000255078	ENSP00000307525
ENSP00000255078	ENSP00000325863
ENSP00000255078	ENSP00000331310
ENSP00000255078	ENSP00000347978
ENSP00000255078	ENSP00000356146
ENSP00000255078	ENSP00000363021
ENSP00000255078	ENSP00000369335
ENSP00000255078	ENSP00000370150
ENSP00000255082	ENSP00000263056
ENSP00000255108	ENSP00000263083
ENSP00000255108	ENSP00000268206
ENSP00000255108	ENSP00000296805
ENSP00000255108	ENSP00000297185
ENSP00000255108	ENSP00000321320
ENSP00000255108	ENSP00000334564
ENSP00000255108	ENSP00000348877
ENSP00000255108	ENSP00000361465
ENSP00000255108	ENSP00000419599
ENSP00000255194	ENSP00000256658

ENSP00000255194	ENSP00000258062
ENSP00000255194	ENSP00000259400
ENSP00000255194	ENSP00000261531
ENSP00000255194	ENSP00000264705
ENSP00000255194	ENSP00000269122
ENSP00000255194	ENSP00000280772
ENSP00000255194	ENSP00000286448
ENSP00000255194	ENSP00000287598
ENSP00000255194	ENSP00000294543
ENSP00000255194	ENSP00000302530
ENSP00000255194	ENSP00000303279
ENSP00000255194	ENSP00000305459
ENSP00000255194	ENSP00000309262
ENSP00000255194	ENSP00000319979
ENSP00000255194	ENSP00000337688
ENSP00000255194	ENSP00000343535
ENSP00000255194	ENSP00000344055
ENSP00000255194	ENSP00000347408
ENSP00000255194	ENSP00000350199
ENSP00000255194	ENSP00000364794
ENSP00000255194	ENSP00000365837
ENSP00000255194	ENSP00000377769
ENSP00000255194	ENSP00000415434
ENSP00000255194	ENSP00000418661
ENSP00000255226	ENSP00000357674
ENSP00000255226	ENSP00000417281
ENSP00000255266	ENSP00000348554
ENSP00000255266	ENSP00000351015
ENSP00000255304	ENSP00000270776
ENSP00000255304	ENSP00000291009
ENSP00000255304	ENSP00000296564
ENSP00000255304	ENSP00000339933
ENSP00000255304	ENSP00000343362
ENSP00000255305	ENSP00000336702
ENSP00000255324	ENSP00000269397
ENSP00000255324	ENSP00000294848
ENSP00000255324	ENSP00000346065
ENSP00000255324	ENSP00000349208
ENSP00000255324	ENSP00000356846
ENSP00000255324	ENSP00000357812
ENSP00000255324	ENSP00000360116
ENSP00000255324	ENSP00000360532
ENSP00000255380	ENSP00000355146
ENSP00000255389	ENSP00000261407
ENSP00000255389	ENSP00000262177
ENSP00000255389	ENSP00000263228

ENSP00000255389	ENSP00000263817
ENSP00000255389	ENSP00000264005
ENSP00000255389	ENSP00000265689
ENSP00000255389	ENSP00000266095
ENSP00000255389	ENSP00000267750
ENSP00000255389	ENSP00000287936
ENSP00000255389	ENSP00000302177
ENSP00000255389	ENSP00000305459
ENSP00000255389	ENSP00000306124
ENSP00000255389	ENSP00000310721
ENSP00000255389	ENSP00000314196
ENSP00000255389	ENSP00000327716
ENSP00000255389	ENSP00000331719
ENSP00000255389	ENSP00000333873
ENSP00000255389	ENSP00000349877
ENSP00000255389	ENSP00000349970
ENSP00000255389	ENSP00000350967
ENSP00000255389	ENSP00000354519
ENSP00000255389	ENSP00000359067
ENSP00000255389	ENSP00000359380
ENSP00000255389	ENSP00000359552
ENSP00000255389	ENSP00000361287
ENSP00000255389	ENSP00000368439
ENSP00000255389	ENSP00000401445
ENSP00000255389	ENSP00000402240
ENSP00000255390	ENSP00000261070
ENSP00000255390	ENSP00000299335
ENSP00000255390	ENSP00000351644
ENSP00000255390	ENSP00000354876
ENSP00000255390	ENSP00000355572
ENSP00000255390	ENSP00000356231
ENSP00000255390	ENSP00000357973
ENSP00000255390	ENSP00000359976
ENSP00000255390	ENSP00000387262
ENSP00000255465	ENSP00000256398
ENSP00000255465	ENSP00000256442
ENSP00000255465	ENSP00000256925
ENSP00000255465	ENSP00000257934
ENSP00000255465	ENSP00000260187
ENSP00000255465	ENSP00000262133
ENSP00000255465	ENSP00000262643
ENSP00000255465	ENSP00000263246
ENSP00000255465	ENSP00000264335
ENSP00000255465	ENSP00000265056
ENSP00000255465	ENSP00000265351
ENSP00000255465	ENSP00000267163

ENSP00000255465	ENSP00000268043
ENSP00000255465	ENSP00000269214
ENSP00000255465	ENSP00000274255
ENSP00000255465	ENSP00000276014
ENSP00000255465	ENSP00000278412
ENSP00000255465	ENSP00000278612
ENSP00000255465	ENSP00000278616
ENSP00000255465	ENSP00000287598
ENSP00000255465	ENSP00000292535
ENSP00000255465	ENSP00000296509
ENSP00000255465	ENSP00000298552
ENSP00000255465	ENSP00000301019
ENSP00000255465	ENSP00000301039
ENSP00000255465	ENSP00000302530
ENSP00000255465	ENSP00000304226
ENSP00000255465	ENSP00000306999
ENSP00000255465	ENSP00000309166
ENSP00000255465	ENSP00000309871
ENSP00000255465	ENSP00000310040
ENSP00000255465	ENSP00000311083
ENSP00000255465	ENSP00000313420
ENSP00000255465	ENSP00000313490
ENSP00000255465	ENSP00000321636
ENSP00000255465	ENSP00000322180
ENSP00000255465	ENSP00000325120
ENSP00000255465	ENSP00000326128
ENSP00000255465	ENSP00000329357
ENSP00000255465	ENSP00000330074
ENSP00000255465	ENSP00000335044
ENSP00000255465	ENSP00000340466
ENSP00000255465	ENSP00000341189
ENSP00000255465	ENSP00000343741
ENSP00000255465	ENSP00000344456
ENSP00000255465	ENSP00000346566
ENSP00000255465	ENSP00000347129
ENSP00000255465	ENSP00000347169
ENSP00000255465	ENSP00000348886
ENSP00000255465	ENSP00000350283
ENSP00000255465	ENSP00000350491
ENSP00000255465	ENSP00000352121
ENSP00000255465	ENSP00000352257
ENSP00000255465	ENSP00000353098
ENSP00000255465	ENSP00000353622
ENSP00000255465	ENSP00000355865
ENSP00000255465	ENSP00000356990
ENSP00000255465	ENSP00000360621

ENSP00000255465	ENSP00000362688
ENSP00000255465	ENSP00000363827
ENSP00000255465	ENSP00000377793
ENSP00000255465	ENSP00000378529
ENSP00000255465	ENSP00000379607
ENSP00000255465	ENSP00000382595
ENSP00000255476	ENSP00000281172
ENSP00000255476	ENSP00000290524
ENSP00000255559	ENSP00000309710
ENSP00000255559	ENSP00000362376
ENSP00000255608	ENSP00000294119
ENSP00000255608	ENSP00000304102
ENSP00000255608	ENSP00000335306
ENSP00000255608	ENSP00000338157
ENSP00000255608	ENSP00000341551
ENSP00000255608	ENSP00000350512
ENSP00000255608	ENSP00000354522
ENSP00000255616	ENSP00000300086
ENSP00000255616	ENSP00000340995
ENSP00000255631	ENSP00000288828
ENSP00000255631	ENSP00000297185
ENSP00000255631	ENSP00000323858
ENSP00000255631	ENSP00000337688
ENSP00000255631	ENSP00000347948
ENSP00000255631	ENSP00000349131
ENSP00000255631	ENSP00000356530
ENSP00000255631	ENSP00000357998
ENSP00000255631	ENSP00000365943
ENSP00000255631	ENSP00000386621
ENSP00000255631	ENSP00000401445
ENSP00000255759	ENSP00000327077
ENSP00000255764	ENSP00000256379
ENSP00000255764	ENSP00000258648
ENSP00000255764	ENSP00000261758
ENSP00000255764	ENSP00000263205
ENSP00000255764	ENSP00000263390
ENSP00000255764	ENSP00000264160
ENSP00000255764	ENSP00000265343
ENSP00000255764	ENSP00000265350
ENSP00000255764	ENSP00000265872
ENSP00000255764	ENSP00000267064
ENSP00000255764	ENSP00000268711
ENSP00000255764	ENSP00000272645
ENSP00000255764	ENSP00000281928
ENSP00000255764	ENSP00000282884
ENSP00000255764	ENSP00000282892

ENSP00000255764	ENSP00000286317
ENSP00000255764	ENSP00000290663
ENSP00000255764	ENSP00000292035
ENSP00000255764	ENSP00000293406
ENSP00000255764	ENSP00000293777
ENSP00000255764	ENSP00000296930
ENSP00000255764	ENSP00000297347
ENSP00000255764	ENSP00000299424
ENSP00000255764	ENSP00000300651
ENSP00000255764	ENSP00000301788
ENSP00000255764	ENSP00000306983
ENSP00000255764	ENSP00000307850
ENSP00000255764	ENSP00000312735
ENSP00000255764	ENSP00000314311
ENSP00000255764	ENSP00000314949
ENSP00000255764	ENSP00000317636
ENSP00000255764	ENSP00000323720
ENSP00000255764	ENSP00000324124
ENSP00000255764	ENSP00000325612
ENSP00000255764	ENSP00000326128
ENSP00000255764	ENSP00000326767
ENSP00000255764	ENSP00000334061
ENSP00000255764	ENSP00000335261
ENSP00000255764	ENSP00000337340
ENSP00000255764	ENSP00000342343
ENSP00000255764	ENSP00000342889
ENSP00000255764	ENSP00000348610
ENSP00000255764	ENSP00000357047
ENSP00000255764	ENSP00000357907
ENSP00000255764	ENSP00000359290
ENSP00000255764	ENSP00000360371
ENSP00000255764	ENSP00000362948
ENSP00000255764	ENSP00000363193
ENSP00000255764	ENSP00000369162
ENSP00000255764	ENSP00000369695
ENSP00000255764	ENSP00000381216
ENSP00000255764	ENSP00000384048
ENSP00000255764	ENSP00000391774
ENSP00000255764	ENSP00000392617
ENSP00000255764	ENSP00000396749
ENSP00000255764	ENSP00000403852
ENSP00000255764	ENSP00000418734
ENSP00000256010	ENSP00000256637
ENSP00000256010	ENSP00000358106
ENSP00000256010	ENSP00000370372
ENSP00000256031	ENSP00000261819

ENSP00000256031	ENSP00000281187
ENSP00000256031	ENSP00000290299
ENSP00000256031	ENSP00000299601
ENSP00000256031	ENSP00000318977
ENSP00000256031	ENSP00000327309
ENSP00000256031	ENSP00000337475
ENSP00000256031	ENSP00000355471
ENSP00000256031	ENSP00000356562
ENSP00000256031	ENSP00000356807
ENSP00000256031	ENSP00000357341
ENSP00000256031	ENSP00000362532
ENSP00000256031	ENSP00000384211
ENSP00000256151	ENSP00000315702
ENSP00000256151	ENSP00000322957
ENSP00000256151	ENSP00000346879
ENSP00000256151	ENSP00000364847
ENSP00000256216	ENSP00000262850
ENSP00000256216	ENSP00000263228
ENSP00000256216	ENSP00000280701
ENSP00000256216	ENSP00000287078
ENSP00000256216	ENSP00000290429
ENSP00000256216	ENSP00000304102
ENSP00000256216	ENSP00000306080
ENSP00000256216	ENSP00000310040
ENSP00000256216	ENSP00000319977
ENSP00000256216	ENSP00000342056
ENSP00000256216	ENSP00000348565
ENSP00000256216	ENSP00000349016
ENSP00000256216	ENSP00000350512
ENSP00000256216	ENSP00000354720
ENSP00000256216	ENSP00000358312
ENSP00000256216	ENSP00000360992
ENSP00000256216	ENSP00000363591
ENSP00000256216	ENSP00000364320
ENSP00000256216	ENSP00000366607
ENSP00000256216	ENSP00000367407
ENSP00000256216	ENSP00000387262
ENSP00000256216	ENSP00000397598
ENSP00000256255	ENSP00000290649
ENSP00000256255	ENSP00000337053
ENSP00000256255	ENSP00000366395
ENSP00000256255	ENSP00000379383
ENSP00000256257	ENSP00000272645
ENSP00000256257	ENSP00000297156
ENSP00000256257	ENSP00000312735
ENSP00000256257	ENSP00000324124

ENSP00000256257	ENSP00000403852
ENSP00000256319	ENSP00000261507
ENSP00000256319	ENSP00000264027
ENSP00000256319	ENSP00000265896
ENSP00000256319	ENSP00000272163
ENSP00000256319	ENSP00000277010
ENSP00000256319	ENSP00000285949
ENSP00000256319	ENSP00000295119
ENSP00000256319	ENSP00000301825
ENSP00000256319	ENSP00000310721
ENSP00000256319	ENSP00000320604
ENSP00000256319	ENSP00000325425
ENSP00000256319	ENSP00000330812
ENSP00000256319	ENSP00000333946
ENSP00000256319	ENSP00000341662
ENSP00000256319	ENSP00000348762
ENSP00000256319	ENSP00000351520
ENSP00000256319	ENSP00000355517
ENSP00000256319	ENSP00000360918
ENSP00000256319	ENSP00000365840
ENSP00000256319	ENSP00000367346
ENSP00000256319	ENSP00000379430
ENSP00000256319	ENSP00000387019
ENSP00000256339	ENSP00000270225
ENSP00000256379	ENSP00000258648
ENSP00000256379	ENSP00000258886
ENSP00000256379	ENSP00000263205
ENSP00000256379	ENSP00000263390
ENSP00000256379	ENSP00000263857
ENSP00000256379	ENSP00000264160
ENSP00000256379	ENSP00000264637
ENSP00000256379	ENSP00000265350
ENSP00000256379	ENSP00000265990
ENSP00000256379	ENSP00000267064
ENSP00000256379	ENSP00000268711
ENSP00000256379	ENSP00000280190
ENSP00000256379	ENSP00000281928
ENSP00000256379	ENSP00000282007
ENSP00000256379	ENSP00000282884
ENSP00000256379	ENSP00000282892
ENSP00000256379	ENSP00000286317
ENSP00000256379	ENSP00000290663
ENSP00000256379	ENSP00000292035
ENSP00000256379	ENSP00000293777
ENSP00000256379	ENSP00000296328
ENSP00000256379	ENSP00000297347

ENSP00000256379	ENSP00000298139
ENSP00000256379	ENSP00000299543
ENSP00000256379	ENSP00000300651
ENSP00000256379	ENSP00000302478
ENSP00000256379	ENSP00000312735
ENSP00000256379	ENSP00000312778
ENSP00000256379	ENSP00000314949
ENSP00000256379	ENSP00000316578
ENSP00000256379	ENSP00000322180
ENSP00000256379	ENSP00000323720
ENSP00000256379	ENSP00000325612
ENSP00000256379	ENSP00000326128
ENSP00000256379	ENSP00000326767
ENSP00000256379	ENSP00000337340
ENSP00000256379	ENSP00000342343
ENSP00000256379	ENSP00000347232
ENSP00000256379	ENSP00000348069
ENSP00000256379	ENSP00000348610
ENSP00000256379	ENSP00000355759
ENSP00000256379	ENSP00000357047
ENSP00000256379	ENSP00000357907
ENSP00000256379	ENSP00000360371
ENSP00000256379	ENSP00000361465
ENSP00000256379	ENSP00000362948
ENSP00000256379	ENSP00000363193
ENSP00000256379	ENSP00000369162
ENSP00000256379	ENSP00000381216
ENSP00000256379	ENSP00000384048
ENSP00000256379	ENSP00000391774
ENSP00000256379	ENSP00000392617
ENSP00000256379	ENSP00000418734
ENSP00000256383	ENSP00000259569
ENSP00000256383	ENSP00000260045
ENSP00000256383	ENSP00000261531
ENSP00000256383	ENSP00000261868
ENSP00000256383	ENSP00000263360
ENSP00000256383	ENSP00000263629
ENSP00000256383	ENSP00000263657
ENSP00000256383	ENSP00000263791
ENSP00000256383	ENSP00000264705
ENSP00000256383	ENSP00000266126
ENSP00000256383	ENSP00000272418
ENSP00000256383	ENSP00000273783
ENSP00000256383	ENSP00000274680
ENSP00000256383	ENSP00000280557
ENSP00000256383	ENSP00000284202

ENSP00000256383	ENSP00000289371
ENSP00000256383	ENSP00000296792
ENSP00000256383	ENSP00000298048
ENSP00000256383	ENSP00000307235
ENSP00000256383	ENSP00000310596
ENSP00000256383	ENSP00000316879
ENSP00000256383	ENSP00000320309
ENSP00000256383	ENSP00000323424
ENSP00000256383	ENSP00000331879
ENSP00000256383	ENSP00000336702
ENSP00000256383	ENSP00000339090
ENSP00000256383	ENSP00000340281
ENSP00000256383	ENSP00000341885
ENSP00000256383	ENSP00000344220
ENSP00000256383	ENSP00000345917
ENSP00000256383	ENSP00000346453
ENSP00000256383	ENSP00000349142
ENSP00000256383	ENSP00000353575
ENSP00000256383	ENSP00000354125
ENSP00000256383	ENSP00000355086
ENSP00000256383	ENSP00000355185
ENSP00000256383	ENSP00000356548
ENSP00000256383	ENSP00000358140
ENSP00000256383	ENSP00000361010
ENSP00000256383	ENSP00000362688
ENSP00000256383	ENSP00000363779
ENSP00000256383	ENSP00000364119
ENSP00000256383	ENSP00000368927
ENSP00000256383	ENSP00000370258
ENSP00000256383	ENSP00000379607
ENSP00000256383	ENSP00000394869
ENSP00000256383	ENSP00000417229
ENSP00000256398	ENSP00000256442
ENSP00000256398	ENSP00000256897
ENSP00000256398	ENSP00000259569
ENSP00000256398	ENSP00000259750
ENSP00000256398	ENSP00000260257
ENSP00000256398	ENSP00000260643
ENSP00000256398	ENSP00000261211
ENSP00000256398	ENSP00000261609
ENSP00000256398	ENSP00000261819
ENSP00000256398	ENSP00000262027
ENSP00000256398	ENSP00000262367
ENSP00000256398	ENSP00000262394
ENSP00000256398	ENSP00000262608
ENSP00000256398	ENSP00000262643

ENSP00000256398	ENSP00000262715
ENSP00000256398	ENSP00000263377
ENSP00000256398	ENSP00000264157
ENSP00000256398	ENSP00000264276
ENSP00000256398	ENSP00000264501
ENSP00000256398	ENSP00000264515
ENSP00000256398	ENSP00000270225
ENSP00000256398	ENSP00000274680
ENSP00000256398	ENSP00000276014
ENSP00000256398	ENSP00000276416
ENSP00000256398	ENSP00000278412
ENSP00000256398	ENSP00000282572
ENSP00000256398	ENSP00000282884
ENSP00000256398	ENSP00000285873
ENSP00000256398	ENSP00000286719
ENSP00000256398	ENSP00000287239
ENSP00000256398	ENSP00000291688
ENSP00000256398	ENSP00000293879
ENSP00000256398	ENSP00000295926
ENSP00000256398	ENSP00000296755
ENSP00000256398	ENSP00000296792
ENSP00000256398	ENSP00000296802
ENSP00000256398	ENSP00000298937
ENSP00000256398	ENSP00000299543
ENSP00000256398	ENSP00000300231
ENSP00000256398	ENSP00000300249
ENSP00000256398	ENSP00000305721
ENSP00000256398	ENSP00000307684
ENSP00000256398	ENSP00000311665
ENSP00000256398	ENSP00000313603
ENSP00000256398	ENSP00000314348
ENSP00000256398	ENSP00000314949
ENSP00000256398	ENSP00000318313
ENSP00000256398	ENSP00000320309
ENSP00000256398	ENSP00000320566
ENSP00000256398	ENSP00000321617
ENSP00000256398	ENSP00000322180
ENSP00000256398	ENSP00000323858
ENSP00000256398	ENSP00000325677
ENSP00000256398	ENSP00000327716
ENSP00000256398	ENSP00000328690
ENSP00000256398	ENSP00000331111
ENSP00000256398	ENSP00000332340
ENSP00000256398	ENSP00000334061
ENSP00000256398	ENSP00000334188
ENSP00000256398	ENSP00000336856

ENSP00000256398	ENSP00000337839
ENSP00000256398	ENSP00000339449
ENSP00000256398	ENSP00000340271
ENSP00000256398	ENSP00000340896
ENSP00000256398	ENSP00000341551
ENSP00000256398	ENSP00000342381
ENSP00000256398	ENSP00000343701
ENSP00000256398	ENSP00000345144
ENSP00000256398	ENSP00000345702
ENSP00000256398	ENSP00000346120
ENSP00000256398	ENSP00000346453
ENSP00000256398	ENSP00000347168
ENSP00000256398	ENSP00000348129
ENSP00000256398	ENSP00000350767
ENSP00000256398	ENSP00000350967
ENSP00000256398	ENSP00000352678
ENSP00000256398	ENSP00000352820
ENSP00000256398	ENSP00000353059
ENSP00000256398	ENSP00000353622
ENSP00000256398	ENSP00000354673
ENSP00000256398	ENSP00000355013
ENSP00000256398	ENSP00000355866
ENSP00000256398	ENSP00000356405
ENSP00000256398	ENSP00000356548
ENSP00000256398	ENSP00000356641
ENSP00000256398	ENSP00000356774
ENSP00000256398	ENSP00000356811
ENSP00000256398	ENSP00000357882
ENSP00000256398	ENSP00000360116
ENSP00000256398	ENSP00000360473
ENSP00000256398	ENSP00000360515
ENSP00000256398	ENSP00000360676
ENSP00000256398	ENSP00000360828
ENSP00000256398	ENSP00000361232
ENSP00000256398	ENSP00000361245
ENSP00000256398	ENSP00000362352
ENSP00000256398	ENSP00000363734
ENSP00000256398	ENSP00000363779
ENSP00000256398	ENSP00000364582
ENSP00000256398	ENSP00000364929
ENSP00000256398	ENSP00000365811
ENSP00000256398	ENSP00000365837
ENSP00000256398	ENSP00000366828
ENSP00000256398	ENSP00000366953
ENSP00000256398	ENSP00000366974
ENSP00000256398	ENSP00000367029

ENSP00000256398	ENSP00000367034
ENSP00000256398	ENSP00000367299
ENSP00000256398	ENSP00000367766
ENSP00000256398	ENSP00000368438
ENSP00000256398	ENSP00000371051
ENSP00000256398	ENSP00000372694
ENSP00000256398	ENSP00000373082
ENSP00000256398	ENSP00000374280
ENSP00000256398	ENSP00000374529
ENSP00000256398	ENSP00000376268
ENSP00000256398	ENSP00000378624
ENSP00000256398	ENSP00000379888
ENSP00000256398	ENSP00000380495
ENSP00000256398	ENSP00000380982
ENSP00000256398	ENSP00000381657
ENSP00000256398	ENSP00000382779
ENSP00000256398	ENSP00000383503
ENSP00000256398	ENSP00000388320
ENSP00000256398	ENSP00000389065
ENSP00000256398	ENSP00000390158
ENSP00000256398	ENSP00000397259
ENSP00000256398	ENSP00000397636
ENSP00000256398	ENSP00000402338
ENSP00000256398	ENSP00000405636
ENSP00000256398	ENSP00000410530
ENSP00000256398	ENSP00000410954
ENSP00000256398	ENSP00000411327
ENSP00000256398	ENSP00000412319
ENSP00000256398	ENSP00000412553
ENSP00000256398	ENSP00000412922
ENSP00000256398	ENSP00000413319
ENSP00000256398	ENSP00000414110
ENSP00000256398	ENSP00000414373
ENSP00000256398	ENSP00000415678
ENSP00000256398	ENSP00000416599
ENSP00000256398	ENSP00000419599
ENSP00000256404	ENSP00000258383
ENSP00000256404	ENSP00000260102
ENSP00000256404	ENSP00000262077
ENSP00000256404	ENSP00000264995
ENSP00000256404	ENSP00000273075
ENSP00000256404	ENSP00000279242
ENSP00000256404	ENSP00000288937
ENSP00000256404	ENSP00000300151
ENSP00000256404	ENSP00000305503
ENSP00000256404	ENSP00000306548

ENSP00000256404	ENSP00000308897
ENSP00000256404	ENSP00000312311
ENSP00000256404	ENSP00000315017
ENSP00000256404	ENSP00000324122
ENSP00000256404	ENSP00000333401
ENSP00000256404	ENSP00000351015
ENSP00000256404	ENSP00000360498
ENSP00000256404	ENSP00000370930
ENSP00000256404	ENSP00000377486
ENSP00000256404	ENSP00000417602
ENSP00000256429	ENSP00000261531
ENSP00000256429	ENSP00000262367
ENSP00000256429	ENSP00000264718
ENSP00000256429	ENSP00000296504
ENSP00000256429	ENSP00000318085
ENSP00000256429	ENSP00000319169
ENSP00000256429	ENSP00000326550
ENSP00000256429	ENSP00000329933
ENSP00000256429	ENSP00000349049
ENSP00000256429	ENSP00000351552
ENSP00000256429	ENSP00000352516
ENSP00000256429	ENSP00000353622
ENSP00000256429	ENSP00000356520
ENSP00000256429	ENSP00000362441
ENSP00000256429	ENSP00000362592
ENSP00000256429	ENSP00000369716
ENSP00000256429	ENSP00000417281
ENSP00000256433	ENSP00000343435
ENSP00000256442	ENSP00000256897
ENSP00000256442	ENSP00000257789
ENSP00000256442	ENSP00000257934
ENSP00000256442	ENSP00000260731
ENSP00000256442	ENSP00000260810
ENSP00000256442	ENSP00000261531
ENSP00000256442	ENSP00000261712
ENSP00000256442	ENSP00000261819
ENSP00000256442	ENSP00000262300
ENSP00000256442	ENSP00000262315
ENSP00000256442	ENSP00000262460
ENSP00000256442	ENSP00000262519
ENSP00000256442	ENSP00000262904
ENSP00000256442	ENSP00000263026
ENSP00000256442	ENSP00000263043
ENSP00000256442	ENSP00000263181
ENSP00000256442	ENSP00000263201
ENSP00000256442	ENSP00000263208

ENSP00000256442	ENSP00000263228
ENSP00000256442	ENSP00000263657
ENSP00000256442	ENSP00000264169
ENSP00000256442	ENSP00000264639
ENSP00000256442	ENSP00000265104
ENSP00000256442	ENSP00000265140
ENSP00000256442	ENSP00000265351
ENSP00000256442	ENSP00000265728
ENSP00000256442	ENSP00000268043
ENSP00000256442	ENSP00000269214
ENSP00000256442	ENSP00000269397
ENSP00000256442	ENSP00000274255
ENSP00000256442	ENSP00000274289
ENSP00000256442	ENSP00000276014
ENSP00000256442	ENSP00000276893
ENSP00000256442	ENSP00000278412
ENSP00000256442	ENSP00000278616
ENSP00000256442	ENSP00000281708
ENSP00000256442	ENSP00000285896
ENSP00000256442	ENSP00000286428
ENSP00000256442	ENSP00000287598
ENSP00000256442	ENSP00000293406
ENSP00000256442	ENSP00000295767
ENSP00000256442	ENSP00000295901
ENSP00000256442	ENSP00000296509
ENSP00000256442	ENSP00000296577
ENSP00000256442	ENSP00000298048
ENSP00000256442	ENSP00000298552
ENSP00000256442	ENSP00000299381
ENSP00000256442	ENSP00000299543
ENSP00000256442	ENSP00000300291
ENSP00000256442	ENSP00000300738
ENSP00000256442	ENSP00000302111
ENSP00000256442	ENSP00000302530
ENSP00000256442	ENSP00000303507
ENSP00000256442	ENSP00000304226
ENSP00000256442	ENSP00000305503
ENSP00000256442	ENSP00000306999
ENSP00000256442	ENSP00000307023
ENSP00000256442	ENSP00000307272
ENSP00000256442	ENSP00000307853
ENSP00000256442	ENSP00000309474
ENSP00000256442	ENSP00000309871
ENSP00000256442	ENSP00000310649
ENSP00000256442	ENSP00000311083
ENSP00000256442	ENSP00000313420

ENSP00000256442	ENSP00000314004
ENSP00000256442	ENSP00000316032
ENSP00000256442	ENSP00000318775
ENSP00000256442	ENSP00000318977
ENSP00000256442	ENSP00000319169
ENSP00000256442	ENSP00000321636
ENSP00000256442	ENSP00000322180
ENSP00000256442	ENSP00000325863
ENSP00000256442	ENSP00000328720
ENSP00000256442	ENSP00000332353
ENSP00000256442	ENSP00000336842
ENSP00000256442	ENSP00000337396
ENSP00000256442	ENSP00000339109
ENSP00000256442	ENSP00000340879
ENSP00000256442	ENSP00000342307
ENSP00000256442	ENSP00000343701
ENSP00000256442	ENSP00000343741
ENSP00000256442	ENSP00000344547
ENSP00000256442	ENSP00000346566
ENSP00000256442	ENSP00000346987
ENSP00000256442	ENSP00000347087
ENSP00000256442	ENSP00000347168
ENSP00000256442	ENSP00000347169
ENSP00000256442	ENSP00000347232
ENSP00000256442	ENSP00000347602
ENSP00000256442	ENSP00000347978
ENSP00000256442	ENSP00000348554
ENSP00000256442	ENSP00000348965
ENSP00000256442	ENSP00000350283
ENSP00000256442	ENSP00000350491
ENSP00000256442	ENSP00000350767
ENSP00000256442	ENSP00000351052
ENSP00000256442	ENSP00000351284
ENSP00000256442	ENSP00000351416
ENSP00000256442	ENSP00000352121
ENSP00000256442	ENSP00000352257
ENSP00000256442	ENSP00000352514
ENSP00000256442	ENSP00000353375
ENSP00000256442	ENSP00000353698
ENSP00000256442	ENSP00000353793
ENSP00000256442	ENSP00000354522
ENSP00000256442	ENSP00000354558
ENSP00000256442	ENSP00000354791
ENSP00000256442	ENSP00000354916
ENSP00000256442	ENSP00000354927
ENSP00000256442	ENSP00000355884

ENSP00000256442	ENSP00000356920
ENSP00000256442	ENSP00000357907
ENSP00000256442	ENSP00000360025
ENSP00000256442	ENSP00000360268
ENSP00000256442	ENSP00000360286
ENSP00000256442	ENSP00000360532
ENSP00000256442	ENSP00000360621
ENSP00000256442	ENSP00000361310
ENSP00000256442	ENSP00000362334
ENSP00000256442	ENSP00000363021
ENSP00000256442	ENSP00000363322
ENSP00000256442	ENSP00000363827
ENSP00000256442	ENSP00000364119
ENSP00000256442	ENSP00000364591
ENSP00000256442	ENSP00000366974
ENSP00000256442	ENSP00000367034
ENSP00000256442	ENSP00000368438
ENSP00000256442	ENSP00000368565
ENSP00000256442	ENSP00000368976
ENSP00000256442	ENSP00000369757
ENSP00000256442	ENSP00000370151
ENSP00000256442	ENSP00000370589
ENSP00000256442	ENSP00000372023
ENSP00000256442	ENSP00000373060
ENSP00000256442	ENSP00000374566
ENSP00000256442	ENSP00000377793
ENSP00000256442	ENSP00000378350
ENSP00000256442	ENSP00000378529
ENSP00000256442	ENSP00000379607
ENSP00000256442	ENSP00000380256
ENSP00000256442	ENSP00000381565
ENSP00000256442	ENSP00000383986
ENSP00000256442	ENSP00000384048
ENSP00000256442	ENSP00000385995
ENSP00000256442	ENSP00000391723
ENSP00000256442	ENSP00000394394
ENSP00000256442	ENSP00000402515
ENSP00000256452	ENSP00000260130
ENSP00000256452	ENSP00000384053
ENSP00000256474	ENSP00000258886
ENSP00000256474	ENSP00000259235
ENSP00000256474	ENSP00000261600
ENSP00000256474	ENSP00000261623
ENSP00000256474	ENSP00000262306
ENSP00000256474	ENSP00000262375
ENSP00000256474	ENSP00000263734

ENSP00000256474	ENSP00000273317
ENSP00000256474	ENSP00000274255
ENSP00000256474	ENSP00000278520
ENSP00000256474	ENSP00000278616
ENSP00000256474	ENSP00000284811
ENSP00000256474	ENSP00000286428
ENSP00000256474	ENSP00000290349
ENSP00000256474	ENSP00000296328
ENSP00000256474	ENSP00000298852
ENSP00000256474	ENSP00000299163
ENSP00000256474	ENSP00000301788
ENSP00000256474	ENSP00000304051
ENSP00000256474	ENSP00000304802
ENSP00000256474	ENSP00000305926
ENSP00000256474	ENSP00000311313
ENSP00000256474	ENSP00000314949
ENSP00000256474	ENSP00000317334
ENSP00000256474	ENSP00000318089
ENSP00000256474	ENSP00000318195
ENSP00000256474	ENSP00000318646
ENSP00000256474	ENSP00000320340
ENSP00000256474	ENSP00000324422
ENSP00000256474	ENSP00000328236
ENSP00000256474	ENSP00000329357
ENSP00000256474	ENSP00000333122
ENSP00000256474	ENSP00000334188
ENSP00000256474	ENSP00000344547
ENSP00000256474	ENSP00000346027
ENSP00000256474	ENSP00000346839
ENSP00000256474	ENSP00000348984
ENSP00000256474	ENSP00000350009
ENSP00000256474	ENSP00000350512
ENSP00000256474	ENSP00000350767
ENSP00000256474	ENSP00000351492
ENSP00000256474	ENSP00000355153
ENSP00000256474	ENSP00000355601
ENSP00000256474	ENSP00000355759
ENSP00000256474	ENSP00000357789
ENSP00000256474	ENSP00000359345
ENSP00000256474	ENSP00000366843
ENSP00000256474	ENSP00000370968
ENSP00000256474	ENSP00000378529
ENSP00000256474	ENSP00000382204
ENSP00000256474	ENSP00000395574
ENSP00000256578	ENSP00000260643
ENSP00000256578	ENSP00000276603

ENSP00000256578	ENSP00000286621
ENSP00000256578	ENSP00000345096
ENSP00000256578	ENSP00000350249
ENSP00000256578	ENSP00000367615
ENSP00000256594	ENSP00000262133
ENSP00000256594	ENSP00000296684
ENSP00000256594	ENSP00000314129
ENSP00000256594	ENSP00000343313
ENSP00000256637	ENSP00000281187
ENSP00000256637	ENSP00000299138
ENSP00000256637	ENSP00000349437
ENSP00000256644	ENSP00000339245
ENSP00000256644	ENSP00000351894
ENSP00000256644	ENSP00000353622
ENSP00000256644	ENSP00000356231
ENSP00000256658	ENSP00000258062
ENSP00000256658	ENSP00000261842
ENSP00000256658	ENSP00000264705
ENSP00000256658	ENSP00000269122
ENSP00000256658	ENSP00000287598
ENSP00000256658	ENSP00000294543
ENSP00000256658	ENSP00000299601
ENSP00000256658	ENSP00000302530
ENSP00000256658	ENSP00000303279
ENSP00000256658	ENSP00000314499
ENSP00000256658	ENSP00000315568
ENSP00000256658	ENSP00000317431
ENSP00000256658	ENSP00000320543
ENSP00000256658	ENSP00000325369
ENSP00000256658	ENSP00000328773
ENSP00000256658	ENSP00000330408
ENSP00000256658	ENSP00000331201
ENSP00000256658	ENSP00000334836
ENSP00000256658	ENSP00000340935
ENSP00000256658	ENSP00000344572
ENSP00000256658	ENSP00000346022
ENSP00000256658	ENSP00000348596
ENSP00000256658	ENSP00000349824
ENSP00000256658	ENSP00000350199
ENSP00000256658	ENSP00000352603
ENSP00000256658	ENSP00000356290
ENSP00000256658	ENSP00000358423
ENSP00000256658	ENSP00000359484
ENSP00000256658	ENSP00000362115
ENSP00000256658	ENSP00000364794
ENSP00000256658	ENSP00000369757

ENSP00000256658	ENSP00000377148
ENSP00000256658	ENSP00000386456
ENSP00000256658	ENSP00000388340
ENSP00000256707	ENSP00000333568
ENSP00000256707	ENSP00000354207
ENSP00000256707	ENSP00000356405
ENSP00000256722	ENSP00000298545
ENSP00000256722	ENSP00000346577
ENSP00000256854	ENSP00000260129
ENSP00000256854	ENSP00000260210
ENSP00000256854	ENSP00000261868
ENSP00000256854	ENSP00000262027
ENSP00000256854	ENSP00000262518
ENSP00000256854	ENSP00000263390
ENSP00000256854	ENSP00000263985
ENSP00000256854	ENSP00000264670
ENSP00000256854	ENSP00000264951
ENSP00000256854	ENSP00000268379
ENSP00000256854	ENSP00000282276
ENSP00000256854	ENSP00000283943
ENSP00000256854	ENSP00000300648
ENSP00000256854	ENSP00000302160
ENSP00000256854	ENSP00000302896
ENSP00000256854	ENSP00000303279
ENSP00000256854	ENSP00000307567
ENSP00000256854	ENSP00000310596
ENSP00000256854	ENSP00000312735
ENSP00000256854	ENSP00000317334
ENSP00000256854	ENSP00000318604
ENSP00000256854	ENSP00000320566
ENSP00000256854	ENSP00000329466
ENSP00000256854	ENSP00000339090
ENSP00000256854	ENSP00000340019
ENSP00000256854	ENSP00000341243
ENSP00000256854	ENSP00000348107
ENSP00000256854	ENSP00000349887
ENSP00000256854	ENSP00000352738
ENSP00000256854	ENSP00000353622
ENSP00000256854	ENSP00000353998
ENSP00000256854	ENSP00000354125
ENSP00000256854	ENSP00000355205
ENSP00000256854	ENSP00000355890
ENSP00000256854	ENSP00000356548
ENSP00000256854	ENSP00000358042
ENSP00000256854	ENSP00000359518
ENSP00000256854	ENSP00000360327

ENSP00000256854	ENSP00000361287
ENSP00000256854	ENSP00000362576
ENSP00000256854	ENSP00000362688
ENSP00000256854	ENSP00000362900
ENSP00000256854	ENSP00000364035
ENSP00000256854	ENSP00000364794
ENSP00000256854	ENSP00000367923
ENSP00000256854	ENSP00000368140
ENSP00000256854	ENSP00000374213
ENSP00000256854	ENSP00000376946
ENSP00000256854	ENSP00000380460
ENSP00000256854	ENSP00000397598
ENSP00000256854	ENSP00000401371
ENSP00000256854	ENSP00000401508
ENSP00000256854	ENSP00000406293
ENSP00000256854	ENSP00000417229
ENSP00000256857	ENSP00000258947
ENSP00000256857	ENSP00000318195
ENSP00000256857	ENSP00000363313
ENSP00000256857	ENSP00000400591
ENSP00000256897	ENSP00000257934
ENSP00000256897	ENSP00000259667
ENSP00000256897	ENSP00000261168
ENSP00000256897	ENSP00000261211
ENSP00000256897	ENSP00000261245
ENSP00000256897	ENSP00000261951
ENSP00000256897	ENSP00000262608
ENSP00000256897	ENSP00000263579
ENSP00000256897	ENSP00000264161
ENSP00000256897	ENSP00000264926
ENSP00000256897	ENSP00000265100
ENSP00000256897	ENSP00000265963
ENSP00000256897	ENSP00000273064
ENSP00000256897	ENSP00000280614
ENSP00000256897	ENSP00000283109
ENSP00000256897	ENSP00000283875
ENSP00000256897	ENSP00000285021
ENSP00000256897	ENSP00000285398
ENSP00000256897	ENSP00000285896
ENSP00000256897	ENSP00000286317
ENSP00000256897	ENSP00000290158
ENSP00000256897	ENSP00000296328
ENSP00000256897	ENSP00000299601
ENSP00000256897	ENSP00000300146
ENSP00000256897	ENSP00000302120
ENSP00000256897	ENSP00000302160

ENSP00000256897	ENSP00000309142
ENSP00000256897	ENSP00000310596
ENSP00000256897	ENSP00000311083
ENSP00000256897	ENSP00000311825
ENSP00000256897	ENSP00000312318
ENSP00000256897	ENSP00000314949
ENSP00000256897	ENSP00000317159
ENSP00000256897	ENSP00000318195
ENSP00000256897	ENSP00000320949
ENSP00000256897	ENSP00000321259
ENSP00000256897	ENSP00000323720
ENSP00000256897	ENSP00000332444
ENSP00000256897	ENSP00000333633
ENSP00000256897	ENSP00000340279
ENSP00000256897	ENSP00000340466
ENSP00000256897	ENSP00000348168
ENSP00000256897	ENSP00000353622
ENSP00000256897	ENSP00000353660
ENSP00000256897	ENSP00000354673
ENSP00000256897	ENSP00000357602
ENSP00000256897	ENSP00000359290
ENSP00000256897	ENSP00000363533
ENSP00000256897	ENSP00000363559
ENSP00000256897	ENSP00000368646
ENSP00000256897	ENSP00000369757
ENSP00000256897	ENSP00000370589
ENSP00000256897	ENSP00000371236
ENSP00000256897	ENSP00000375809
ENSP00000256897	ENSP00000381657
ENSP00000256897	ENSP00000382492
ENSP00000256897	ENSP00000416097
ENSP00000256925	ENSP00000262643
ENSP00000256925	ENSP00000264731
ENSP00000256925	ENSP00000295897
ENSP00000256925	ENSP00000347444
ENSP00000256996	ENSP00000261424
ENSP00000256996	ENSP00000265081
ENSP00000256996	ENSP00000285021
ENSP00000256996	ENSP00000301019
ENSP00000256996	ENSP00000301764
ENSP00000256996	ENSP00000304102
ENSP00000256996	ENSP00000305790
ENSP00000256996	ENSP00000313350
ENSP00000256996	ENSP00000330343
ENSP00000256996	ENSP00000343741
ENSP00000256996	ENSP00000347168

ENSP00000256996	ENSP00000350512
ENSP00000256996	ENSP00000350708
ENSP00000256996	ENSP00000350767
ENSP00000256996	ENSP00000351284
ENSP00000256996	ENSP00000352257
ENSP00000256996	ENSP00000352928
ENSP00000256996	ENSP00000353735
ENSP00000256996	ENSP00000354522
ENSP00000256996	ENSP00000358857
ENSP00000256996	ENSP00000360515
ENSP00000256996	ENSP00000362592
ENSP00000256996	ENSP00000363021
ENSP00000256996	ENSP00000364270
ENSP00000256996	ENSP00000366953
ENSP00000256996	ENSP00000366974
ENSP00000256996	ENSP00000367034
ENSP00000256996	ENSP00000380504
ENSP00000256996	ENSP00000384109
ENSP00000256996	ENSP00000386621
ENSP00000256996	ENSP00000415434
ENSP00000257177	ENSP00000260956
ENSP00000257177	ENSP00000262584
ENSP00000257177	ENSP00000286835
ENSP00000257177	ENSP00000302530
ENSP00000257177	ENSP00000313350
ENSP00000257177	ENSP00000314441
ENSP00000257177	ENSP00000322457
ENSP00000257177	ENSP00000338862
ENSP00000257177	ENSP00000339845
ENSP00000257177	ENSP00000340879
ENSP00000257177	ENSP00000343741
ENSP00000257177	ENSP00000343745
ENSP00000257177	ENSP00000344401
ENSP00000257177	ENSP00000351697
ENSP00000257177	ENSP00000354040
ENSP00000257177	ENSP00000354522
ENSP00000257177	ENSP00000354911
ENSP00000257177	ENSP00000356146
ENSP00000257177	ENSP00000358812
ENSP00000257177	ENSP00000366135
ENSP00000257177	ENSP00000366557
ENSP00000257177	ENSP00000370151
ENSP00000257177	ENSP00000370741
ENSP00000257177	ENSP00000372023
ENSP00000257177	ENSP00000373404
ENSP00000257177	ENSP00000380982

ENSP00000257177	ENSP00000402515
ENSP00000257181	ENSP00000260970
ENSP00000257181	ENSP00000267812
ENSP00000257181	ENSP00000296702
ENSP00000257181	ENSP00000315859
ENSP00000257181	ENSP00000324274
ENSP00000257181	ENSP00000348510
ENSP00000257181	ENSP00000357631
ENSP00000257181	ENSP00000362820
ENSP00000257181	ENSP00000362900
ENSP00000257181	ENSP00000377262
ENSP00000257181	ENSP00000394085
ENSP00000257215	ENSP00000269216
ENSP00000257245	ENSP00000295767
ENSP00000257245	ENSP00000320236
ENSP00000257245	ENSP00000354957
ENSP00000257245	ENSP00000356953
ENSP00000257245	ENSP00000364699
ENSP00000257245	ENSP00000367637
ENSP00000257245	ENSP00000368066
ENSP00000257245	ENSP00000387262
ENSP00000257287	ENSP00000270708
ENSP00000257287	ENSP00000371308
ENSP00000257287	ENSP00000384490
ENSP00000257312	ENSP00000384573
ENSP00000257430	ENSP00000258742
ENSP00000257430	ENSP00000258743
ENSP00000257430	ENSP00000261170
ENSP00000257430	ENSP00000261844
ENSP00000257430	ENSP00000262077
ENSP00000257430	ENSP00000262320
ENSP00000257430	ENSP00000262367
ENSP00000257430	ENSP00000263734
ENSP00000257430	ENSP00000263805
ENSP00000257430	ENSP00000264731
ENSP00000257430	ENSP00000264883
ENSP00000257430	ENSP00000265843
ENSP00000257430	ENSP00000268182
ENSP00000257430	ENSP00000276072
ENSP00000257430	ENSP00000280020
ENSP00000257430	ENSP00000281282
ENSP00000257430	ENSP00000283195
ENSP00000257430	ENSP00000287598
ENSP00000257430	ENSP00000287820
ENSP00000257430	ENSP00000291688
ENSP00000257430	ENSP00000296509

ENSP00000257430	ENSP00000296600
ENSP00000257430	ENSP00000297135
ENSP00000257430	ENSP00000299259
ENSP00000257430	ENSP00000300249
ENSP00000257430	ENSP00000300574
ENSP00000257430	ENSP00000301764
ENSP00000257430	ENSP00000302530
ENSP00000257430	ENSP00000307134
ENSP00000257430	ENSP00000309474
ENSP00000257430	ENSP00000313829
ENSP00000257430	ENSP00000316032
ENSP00000257430	ENSP00000316845
ENSP00000257430	ENSP00000318921
ENSP00000257430	ENSP00000320758
ENSP00000257430	ENSP00000325376
ENSP00000257430	ENSP00000325785
ENSP00000257430	ENSP00000326366
ENSP00000257430	ENSP00000331201
ENSP00000257430	ENSP00000336856
ENSP00000257430	ENSP00000337998
ENSP00000257430	ENSP00000338983
ENSP00000257430	ENSP00000340688
ENSP00000257430	ENSP00000341551
ENSP00000257430	ENSP00000344456
ENSP00000257430	ENSP00000347329
ENSP00000257430	ENSP00000349486
ENSP00000257430	ENSP00000350297
ENSP00000257430	ENSP00000350512
ENSP00000257430	ENSP00000351416
ENSP00000257430	ENSP00000352400
ENSP00000257430	ENSP00000352516
ENSP00000257430	ENSP00000352676
ENSP00000257430	ENSP00000354560
ENSP00000257430	ENSP00000354720
ENSP00000257430	ENSP00000354961
ENSP00000257430	ENSP00000355536
ENSP00000257430	ENSP00000356331
ENSP00000257430	ENSP00000356438
ENSP00000257430	ENSP00000356448
ENSP00000257430	ENSP00000357965
ENSP00000257430	ENSP00000358242
ENSP00000257430	ENSP00000358335
ENSP00000257430	ENSP00000358404
ENSP00000257430	ENSP00000358497
ENSP00000257430	ENSP00000358994
ENSP00000257430	ENSP00000359206

ENSP00000257430	ENSP00000363021
ENSP00000257430	ENSP00000363804
ENSP00000257430	ENSP00000363921
ENSP00000257430	ENSP00000364893
ENSP00000257430	ENSP00000368438
ENSP00000257430	ENSP00000368767
ENSP00000257430	ENSP00000371155
ENSP00000257430	ENSP00000372815
ENSP00000257430	ENSP00000379394
ENSP00000257430	ENSP00000386259
ENSP00000257430	ENSP00000387128
ENSP00000257430	ENSP00000387170
ENSP00000257430	ENSP00000394794
ENSP00000257430	ENSP00000396688
ENSP00000257430	ENSP00000408236
ENSP00000257430	ENSP00000413720
ENSP00000257430	ENSP00000416097
ENSP00000257515	ENSP00000358081
ENSP00000257527	ENSP00000261017
ENSP00000257527	ENSP00000348215
ENSP00000257548	ENSP00000262052
ENSP00000257548	ENSP00000262193
ENSP00000257548	ENSP00000262966
ENSP00000257548	ENSP00000263202
ENSP00000257548	ENSP00000263974
ENSP00000257548	ENSP00000265990
ENSP00000257548	ENSP00000272645
ENSP00000257548	ENSP00000273398
ENSP00000257548	ENSP00000280701
ENSP00000257548	ENSP00000284320
ENSP00000257548	ENSP00000290429
ENSP00000257548	ENSP00000291688
ENSP00000257548	ENSP00000294053
ENSP00000257548	ENSP00000296328
ENSP00000257548	ENSP00000311200
ENSP00000257548	ENSP00000314949
ENSP00000257548	ENSP00000348551
ENSP00000257548	ENSP00000350009
ENSP00000257548	ENSP00000354951
ENSP00000257548	ENSP00000360473
ENSP00000257548	ENSP00000361993
ENSP00000257548	ENSP00000369335
ENSP00000257548	ENSP00000381863
ENSP00000257566	ENSP00000295709
ENSP00000257566	ENSP00000297261
ENSP00000257566	ENSP00000314733

ENSP00000257566	ENSP00000355866
ENSP00000257566	ENSP00000362717
ENSP00000257572	ENSP00000267953
ENSP00000257572	ENSP00000309103
ENSP00000257572	ENSP00000329623
ENSP00000257572	ENSP00000358022
ENSP00000257575	ENSP00000265056
ENSP00000257575	ENSP00000296273
ENSP00000257575	ENSP00000296755
ENSP00000257575	ENSP00000300231
ENSP00000257575	ENSP00000360621
ENSP00000257626	ENSP00000284981
ENSP00000257626	ENSP00000294785
ENSP00000257626	ENSP00000326366
ENSP00000257632	ENSP00000264234
ENSP00000257694	ENSP00000345656
ENSP00000257700	ENSP00000262133
ENSP00000257700	ENSP00000263897
ENSP00000257700	ENSP00000264028
ENSP00000257700	ENSP00000281513
ENSP00000257700	ENSP00000305810
ENSP00000257700	ENSP00000310521
ENSP00000257700	ENSP00000325002
ENSP00000257700	ENSP00000328079
ENSP00000257700	ENSP00000329419
ENSP00000257700	ENSP00000370003
ENSP00000257700	ENSP00000390783
ENSP00000257749	ENSP00000266269
ENSP00000257749	ENSP00000348551
ENSP00000257770	ENSP00000305689
ENSP00000257770	ENSP00000346839
ENSP00000257770	ENSP00000359652
ENSP00000257770	ENSP00000363827
ENSP00000257770	ENSP00000369335
ENSP00000257770	ENSP00000372023
ENSP00000257776	ENSP00000306697
ENSP00000257787	ENSP00000263925
ENSP00000257787	ENSP00000269216
ENSP00000257787	ENSP00000347836
ENSP00000257787	ENSP00000370410
ENSP00000257789	ENSP00000261424
ENSP00000257789	ENSP00000261531
ENSP00000257789	ENSP00000261881
ENSP00000257789	ENSP00000262144
ENSP00000257789	ENSP00000262315
ENSP00000257789	ENSP00000263201

ENSP00000257789	ENSP00000264108
ENSP00000257789	ENSP00000264169
ENSP00000257789	ENSP00000265056
ENSP00000257789	ENSP00000265728
ENSP00000257789	ENSP00000266070
ENSP00000257789	ENSP00000269468
ENSP00000257789	ENSP00000276014
ENSP00000257789	ENSP00000282572
ENSP00000257789	ENSP00000297431
ENSP00000257789	ENSP00000307208
ENSP00000257789	ENSP00000313171
ENSP00000257789	ENSP00000336868
ENSP00000257789	ENSP00000340879
ENSP00000257789	ENSP00000344741
ENSP00000257789	ENSP00000345917
ENSP00000257789	ENSP00000346725
ENSP00000257789	ENSP00000350283
ENSP00000257789	ENSP00000352257
ENSP00000257789	ENSP00000353793
ENSP00000257789	ENSP00000355011
ENSP00000257789	ENSP00000360215
ENSP00000257789	ENSP00000360412
ENSP00000257789	ENSP00000360532
ENSP00000257789	ENSP00000360621
ENSP00000257789	ENSP00000362135
ENSP00000257789	ENSP00000362592
ENSP00000257789	ENSP00000364037
ENSP00000257789	ENSP00000366953
ENSP00000257789	ENSP00000369335
ENSP00000257789	ENSP00000370151
ENSP00000257789	ENSP00000371475
ENSP00000257789	ENSP00000387982
ENSP00000257789	ENSP00000390475
ENSP00000257829	ENSP00000260363
ENSP00000257829	ENSP00000261637
ENSP00000257829	ENSP00000261708
ENSP00000257829	ENSP00000262584
ENSP00000257829	ENSP00000263657
ENSP00000257829	ENSP00000264230
ENSP00000257829	ENSP00000264995
ENSP00000257829	ENSP00000265245
ENSP00000257829	ENSP00000268482
ENSP00000257829	ENSP00000268661
ENSP00000257829	ENSP00000270625
ENSP00000257829	ENSP00000272521
ENSP00000257829	ENSP00000274606

ENSP00000257829	ENSP00000274849
ENSP00000257829	ENSP00000278572
ENSP00000257829	ENSP00000283109
ENSP00000257829	ENSP00000287038
ENSP00000257829	ENSP00000291576
ENSP00000257829	ENSP00000291582
ENSP00000257829	ENSP00000295373
ENSP00000257829	ENSP00000296792
ENSP00000257829	ENSP00000296802
ENSP00000257829	ENSP00000297579
ENSP00000257829	ENSP00000297990
ENSP00000257829	ENSP00000298283
ENSP00000257829	ENSP00000298746
ENSP00000257829	ENSP00000300151
ENSP00000257829	ENSP00000300648
ENSP00000257829	ENSP00000302896
ENSP00000257829	ENSP00000305702
ENSP00000257829	ENSP00000306548
ENSP00000257829	ENSP00000308179
ENSP00000257829	ENSP00000309334
ENSP00000257829	ENSP00000309433
ENSP00000257829	ENSP00000309572
ENSP00000257829	ENSP00000310649
ENSP00000257829	ENSP00000310723
ENSP00000257829	ENSP00000311028
ENSP00000257829	ENSP00000311135
ENSP00000257829	ENSP00000311430
ENSP00000257829	ENSP00000314193
ENSP00000257829	ENSP00000314348
ENSP00000257829	ENSP00000315674
ENSP00000257829	ENSP00000317376
ENSP00000257829	ENSP00000320917
ENSP00000257829	ENSP00000321449
ENSP00000257829	ENSP00000323858
ENSP00000257829	ENSP00000324122
ENSP00000257829	ENSP00000324403
ENSP00000257829	ENSP00000325002
ENSP00000257829	ENSP00000326806
ENSP00000257829	ENSP00000326981
ENSP00000257829	ENSP00000327179
ENSP00000257829	ENSP00000328690
ENSP00000257829	ENSP00000328854
ENSP00000257829	ENSP00000329466
ENSP00000257829	ENSP00000329933
ENSP00000257829	ENSP00000331907
ENSP00000257829	ENSP00000332258

ENSP00000257829	ENSP00000332340
ENSP00000257829	ENSP00000338573
ENSP00000257829	ENSP00000339095
ENSP00000257829	ENSP00000339795
ENSP00000257829	ENSP00000342181
ENSP00000257829	ENSP00000345156
ENSP00000257829	ENSP00000345917
ENSP00000257829	ENSP00000346050
ENSP00000257829	ENSP00000346120
ENSP00000257829	ENSP00000346725
ENSP00000257829	ENSP00000351947
ENSP00000257829	ENSP00000352678
ENSP00000257829	ENSP00000354040
ENSP00000257829	ENSP00000354045
ENSP00000257829	ENSP00000354522
ENSP00000257829	ENSP00000355541
ENSP00000257829	ENSP00000356520
ENSP00000257829	ENSP00000356591
ENSP00000257829	ENSP00000357907
ENSP00000257829	ENSP00000358563
ENSP00000257829	ENSP00000358812
ENSP00000257829	ENSP00000359688
ENSP00000257829	ENSP00000360031
ENSP00000257829	ENSP00000360412
ENSP00000257829	ENSP00000360532
ENSP00000257829	ENSP00000360828
ENSP00000257829	ENSP00000361076
ENSP00000257829	ENSP00000361186
ENSP00000257829	ENSP00000361232
ENSP00000257829	ENSP00000361982
ENSP00000257829	ENSP00000362153
ENSP00000257829	ENSP00000362744
ENSP00000257829	ENSP00000363018
ENSP00000257829	ENSP00000363642
ENSP00000257829	ENSP00000363676
ENSP00000257829	ENSP00000363746
ENSP00000257829	ENSP00000364320
ENSP00000257829	ENSP00000365806
ENSP00000257829	ENSP00000366557
ENSP00000257829	ENSP00000366974
ENSP00000257829	ENSP00000369716
ENSP00000257829	ENSP00000369757
ENSP00000257829	ENSP00000370589
ENSP00000257829	ENSP00000371101
ENSP00000257829	ENSP00000372199
ENSP00000257829	ENSP00000372695

ENSP00000257829	ENSP00000373090
ENSP00000257829	ENSP00000373404
ENSP00000257829	ENSP00000375730
ENSP00000257829	ENSP00000377747
ENSP00000257829	ENSP00000379607
ENSP00000257829	ENSP00000379888
ENSP00000257829	ENSP00000380495
ENSP00000257829	ENSP00000381654
ENSP00000257829	ENSP00000384302
ENSP00000257829	ENSP00000386134
ENSP00000257829	ENSP00000387303
ENSP00000257829	ENSP00000392601
ENSP00000257829	ENSP00000394290
ENSP00000257829	ENSP00000398824
ENSP00000257829	ENSP00000399454
ENSP00000257829	ENSP00000402869
ENSP00000257829	ENSP00000403576
ENSP00000257829	ENSP00000405614
ENSP00000257829	ENSP00000409074
ENSP00000257829	ENSP00000409367
ENSP00000257829	ENSP00000411162
ENSP00000257829	ENSP00000414321
ENSP00000257829	ENSP00000417229
ENSP00000257879	ENSP00000265354
ENSP00000257879	ENSP00000303476
ENSP00000257879	ENSP00000319831
ENSP00000257879	ENSP00000339958
ENSP00000257879	ENSP00000340944
ENSP00000257879	ENSP00000346839
ENSP00000257879	ENSP00000354951
ENSP00000257879	ENSP00000355751
ENSP00000257879	ENSP00000362334
ENSP00000257879	ENSP00000362717
ENSP00000257879	ENSP00000369042
ENSP00000257910	ENSP00000352928
ENSP00000257934	ENSP00000260363
ENSP00000257934	ENSP00000260731
ENSP00000257934	ENSP00000260753
ENSP00000257934	ENSP00000260766
ENSP00000257934	ENSP00000262643
ENSP00000257934	ENSP00000263181
ENSP00000257934	ENSP00000263736
ENSP00000257934	ENSP00000264157
ENSP00000257934	ENSP00000265056
ENSP00000257934	ENSP00000265148
ENSP00000257934	ENSP00000265963

ENSP00000257934	ENSP00000269214
ENSP00000257934	ENSP00000270861
ENSP00000257934	ENSP00000272444
ENSP00000257934	ENSP00000274289
ENSP00000257934	ENSP00000276014
ENSP00000257934	ENSP00000278616
ENSP00000257934	ENSP00000282516
ENSP00000257934	ENSP00000285873
ENSP00000257934	ENSP00000288976
ENSP00000257934	ENSP00000295321
ENSP00000257934	ENSP00000295926
ENSP00000257934	ENSP00000296509
ENSP00000257934	ENSP00000296733
ENSP00000257934	ENSP00000297338
ENSP00000257934	ENSP00000297431
ENSP00000257934	ENSP00000298048
ENSP00000257934	ENSP00000300584
ENSP00000257934	ENSP00000304437
ENSP00000257934	ENSP00000306999
ENSP00000257934	ENSP00000312870
ENSP00000257934	ENSP00000313420
ENSP00000257934	ENSP00000316032
ENSP00000257934	ENSP00000318775
ENSP00000257934	ENSP00000319104
ENSP00000257934	ENSP00000321636
ENSP00000257934	ENSP00000322570
ENSP00000257934	ENSP00000323439
ENSP00000257934	ENSP00000324020
ENSP00000257934	ENSP00000335357
ENSP00000257934	ENSP00000339109
ENSP00000257934	ENSP00000341382
ENSP00000257934	ENSP00000343741
ENSP00000257934	ENSP00000344936
ENSP00000257934	ENSP00000346566
ENSP00000257934	ENSP00000348554
ENSP00000257934	ENSP00000349959
ENSP00000257934	ENSP00000351697
ENSP00000257934	ENSP00000354558
ENSP00000257934	ENSP00000354720
ENSP00000257934	ENSP00000354916
ENSP00000257934	ENSP00000354957
ENSP00000257934	ENSP00000356319
ENSP00000257934	ENSP00000356607
ENSP00000257934	ENSP00000356920
ENSP00000257934	ENSP00000358423
ENSP00000257934	ENSP00000358497

ENSP00000257934	ENSP00000360310
ENSP00000257934	ENSP00000360621
ENSP00000257934	ENSP00000361264
ENSP00000257934	ENSP00000368349
ENSP00000257934	ENSP00000368976
ENSP00000257934	ENSP00000371475
ENSP00000257934	ENSP00000371888
ENSP00000257934	ENSP00000372689
ENSP00000257934	ENSP00000374529
ENSP00000257934	ENSP00000374566
ENSP00000257934	ENSP00000378350
ENSP00000257934	ENSP00000378529
ENSP00000257934	ENSP00000384675
ENSP00000257934	ENSP00000396704
ENSP00000257934	ENSP00000405963
ENSP00000257934	ENSP00000413632
ENSP00000257940	ENSP00000303315
ENSP00000258052	ENSP00000260433
ENSP00000258052	ENSP00000261507
ENSP00000258052	ENSP00000261881
ENSP00000258052	ENSP00000262300
ENSP00000258052	ENSP00000262554
ENSP00000258052	ENSP00000264027
ENSP00000258052	ENSP00000277010
ENSP00000258052	ENSP00000285949
ENSP00000258052	ENSP00000292114
ENSP00000258052	ENSP00000309259
ENSP00000258052	ENSP00000315693
ENSP00000258052	ENSP00000315700
ENSP00000258052	ENSP00000320604
ENSP00000258052	ENSP00000322180
ENSP00000258052	ENSP00000326699
ENSP00000258052	ENSP00000327716
ENSP00000258052	ENSP00000341662
ENSP00000258052	ENSP00000348215
ENSP00000258052	ENSP00000352121
ENSP00000258052	ENSP00000360918
ENSP00000258052	ENSP00000371475
ENSP00000258052	ENSP00000372023
ENSP00000258052	ENSP00000380450
ENSP00000258052	ENSP00000402084
ENSP00000258062	ENSP00000261842
ENSP00000258062	ENSP00000267540
ENSP00000258062	ENSP00000300574
ENSP00000258062	ENSP00000314499
ENSP00000258062	ENSP00000334836

ENSP00000258062	ENSP00000336790
ENSP00000258062	ENSP00000344055
ENSP00000258062	ENSP00000348602
ENSP00000258062	ENSP00000349824
ENSP00000258062	ENSP00000350199
ENSP00000258062	ENSP00000351926
ENSP00000258062	ENSP00000360076
ENSP00000258062	ENSP00000370719
ENSP00000258062	ENSP00000377148
ENSP00000258062	ENSP00000384222
ENSP00000258062	ENSP00000386456
ENSP00000258083	ENSP00000376776
ENSP00000258105	ENSP00000258499
ENSP00000258105	ENSP00000352408
ENSP00000258106	ENSP00000264731
ENSP00000258106	ENSP00000272645
ENSP00000258106	ENSP00000282549
ENSP00000258106	ENSP00000283921
ENSP00000258106	ENSP00000337396
ENSP00000258106	ENSP00000359215
ENSP00000258106	ENSP00000384597
ENSP00000258169	ENSP00000258455
ENSP00000258169	ENSP00000323046
ENSP00000258169	ENSP00000348107
ENSP00000258169	ENSP00000349679
ENSP00000258169	ENSP00000361433
ENSP00000258169	ENSP00000366135
ENSP00000258169	ENSP00000379607
ENSP00000258169	ENSP00000387911
ENSP00000258180	ENSP00000329002
ENSP00000258200	ENSP00000264169
ENSP00000258243	ENSP00000329466
ENSP00000258243	ENSP00000357283
ENSP00000258301	ENSP00000259400
ENSP00000258301	ENSP00000260818
ENSP00000258301	ENSP00000263116
ENSP00000258301	ENSP00000263688
ENSP00000258301	ENSP00000263897
ENSP00000258301	ENSP00000272322
ENSP00000258301	ENSP00000286448
ENSP00000258301	ENSP00000294179
ENSP00000258301	ENSP00000295119
ENSP00000258301	ENSP00000303554
ENSP00000258301	ENSP00000305255
ENSP00000258301	ENSP00000305810
ENSP00000258301	ENSP00000309457

ENSP00000258301	ENSP00000310521
ENSP00000258301	ENSP00000317955
ENSP00000258301	ENSP00000326534
ENSP00000258301	ENSP00000327650
ENSP00000258301	ENSP00000330320
ENSP00000258301	ENSP00000344055
ENSP00000258301	ENSP00000357484
ENSP00000258301	ENSP00000360183
ENSP00000258301	ENSP00000366225
ENSP00000258301	ENSP00000369810
ENSP00000258301	ENSP00000373215
ENSP00000258301	ENSP00000379933
ENSP00000258301	ENSP00000381293
ENSP00000258301	ENSP00000390783
ENSP00000258301	ENSP00000401435
ENSP00000258301	ENSP00000406988
ENSP00000258301	ENSP00000409952
ENSP00000258301	ENSP00000417748
ENSP00000258301	ENSP00000418661
ENSP00000258381	ENSP00000327268
ENSP00000258381	ENSP00000414187
ENSP00000258383	ENSP00000260102
ENSP00000258383	ENSP00000262077
ENSP00000258383	ENSP00000279242
ENSP00000258383	ENSP00000288937
ENSP00000258383	ENSP00000294008
ENSP00000258383	ENSP00000300151
ENSP00000258383	ENSP00000301585
ENSP00000258383	ENSP00000305503
ENSP00000258383	ENSP00000306548
ENSP00000258383	ENSP00000308275
ENSP00000258383	ENSP00000308897
ENSP00000258383	ENSP00000312311
ENSP00000258383	ENSP00000315017
ENSP00000258383	ENSP00000324122
ENSP00000258383	ENSP00000333401
ENSP00000258383	ENSP00000360286
ENSP00000258383	ENSP00000360498
ENSP00000258383	ENSP00000361699
ENSP00000258383	ENSP00000365318
ENSP00000258383	ENSP00000370930
ENSP00000258383	ENSP00000377486
ENSP00000258383	ENSP00000383142
ENSP00000258383	ENSP00000387262
ENSP00000258383	ENSP00000417602
ENSP00000258390	ENSP00000354251

ENSP00000258390	ENSP00000361048
ENSP00000258399	ENSP00000259457
ENSP00000258399	ENSP00000264515
ENSP00000258399	ENSP00000266126
ENSP00000258399	ENSP00000283875
ENSP00000258399	ENSP00000286835
ENSP00000258399	ENSP00000295897
ENSP00000258399	ENSP00000303279
ENSP00000258399	ENSP00000326921
ENSP00000258399	ENSP00000331201
ENSP00000258399	ENSP00000342026
ENSP00000258399	ENSP00000347498
ENSP00000258399	ENSP00000348442
ENSP00000258399	ENSP00000354673
ENSP00000258399	ENSP00000356146
ENSP00000258399	ENSP00000364486
ENSP00000258399	ENSP00000364794
ENSP00000258399	ENSP00000368349
ENSP00000258399	ENSP00000370258
ENSP00000258399	ENSP00000380460
ENSP00000258400	ENSP00000286758
ENSP00000258400	ENSP00000370410
ENSP00000258411	ENSP00000266066
ENSP00000258411	ENSP00000268459
ENSP00000258411	ENSP00000296145
ENSP00000258411	ENSP00000297261
ENSP00000258411	ENSP00000322304
ENSP00000258411	ENSP00000344456
ENSP00000258411	ENSP00000358404
ENSP00000258415	ENSP00000265896
ENSP00000258415	ENSP00000304592
ENSP00000258415	ENSP00000348762
ENSP00000258415	ENSP00000368646
ENSP00000258416	ENSP00000260956
ENSP00000258416	ENSP00000264951
ENSP00000258416	ENSP00000285896
ENSP00000258416	ENSP00000296802
ENSP00000258416	ENSP00000302160
ENSP00000258416	ENSP00000302768
ENSP00000258416	ENSP00000302955
ENSP00000258416	ENSP00000310040
ENSP00000258416	ENSP00000311876
ENSP00000258416	ENSP00000314311
ENSP00000258416	ENSP00000316879
ENSP00000258416	ENSP00000318646
ENSP00000258416	ENSP00000321617

ENSP00000258416	ENSP00000321711
ENSP00000258416	ENSP00000325677
ENSP00000258416	ENSP00000328103
ENSP00000258416	ENSP00000336702
ENSP00000258416	ENSP00000340281
ENSP00000258416	ENSP00000345096
ENSP00000258416	ENSP00000364289
ENSP00000258416	ENSP00000369965
ENSP00000258416	ENSP00000379888
ENSP00000258416	ENSP00000387170
ENSP00000258416	ENSP00000392859
ENSP00000258418	ENSP00000261951
ENSP00000258418	ENSP00000266581
ENSP00000258418	ENSP00000276282
ENSP00000258418	ENSP00000292616
ENSP00000258418	ENSP00000294818
ENSP00000258418	ENSP00000295628
ENSP00000258418	ENSP00000296144
ENSP00000258418	ENSP00000298288
ENSP00000258418	ENSP00000298910
ENSP00000258418	ENSP00000300417
ENSP00000258418	ENSP00000303248
ENSP00000258418	ENSP00000304923
ENSP00000258418	ENSP00000306864
ENSP00000258418	ENSP00000307023
ENSP00000258418	ENSP00000311505
ENSP00000258418	ENSP00000314901
ENSP00000258418	ENSP00000315702
ENSP00000258418	ENSP00000319303
ENSP00000258418	ENSP00000326324
ENSP00000258418	ENSP00000326759
ENSP00000258418	ENSP00000329380
ENSP00000258418	ENSP00000332879
ENSP00000258418	ENSP00000337797
ENSP00000258418	ENSP00000338887
ENSP00000258418	ENSP00000341944
ENSP00000258418	ENSP00000344242
ENSP00000258418	ENSP00000344758
ENSP00000258418	ENSP00000345985
ENSP00000258418	ENSP00000346155
ENSP00000258418	ENSP00000346809
ENSP00000258418	ENSP00000347451
ENSP00000258418	ENSP00000348215
ENSP00000258418	ENSP00000348278
ENSP00000258418	ENSP00000349486
ENSP00000258418	ENSP00000351113

ENSP00000258418	ENSP00000356191
ENSP00000258418	ENSP00000356247
ENSP00000258418	ENSP00000358765
ENSP00000258418	ENSP00000360560
ENSP00000258418	ENSP00000361185
ENSP00000258418	ENSP00000364700
ENSP00000258418	ENSP00000366121
ENSP00000258418	ENSP00000368135
ENSP00000258418	ENSP00000369395
ENSP00000258418	ENSP00000373600
ENSP00000258418	ENSP00000373853
ENSP00000258418	ENSP00000383382
ENSP00000258418	ENSP00000386357
ENSP00000258418	ENSP00000399441
ENSP00000258418	ENSP00000406367
ENSP00000258418	ENSP00000411568
ENSP00000258424	ENSP00000261813
ENSP00000258424	ENSP00000268379
ENSP00000258424	ENSP00000274787
ENSP00000258424	ENSP00000287022
ENSP00000258424	ENSP00000297564
ENSP00000258424	ENSP00000307786
ENSP00000258424	ENSP00000309565
ENSP00000258424	ENSP00000314380
ENSP00000258424	ENSP00000317159
ENSP00000258424	ENSP00000317780
ENSP00000258424	ENSP00000319169
ENSP00000258424	ENSP00000328938
ENSP00000258424	ENSP00000331897
ENSP00000258424	ENSP00000332887
ENSP00000258424	ENSP00000342300
ENSP00000258424	ENSP00000354499
ENSP00000258424	ENSP00000354554
ENSP00000258424	ENSP00000354876
ENSP00000258424	ENSP00000354982
ENSP00000258424	ENSP00000355572
ENSP00000258424	ENSP00000360782
ENSP00000258424	ENSP00000361042
ENSP00000258424	ENSP00000365243
ENSP00000258424	ENSP00000367934
ENSP00000258424	ENSP00000387262
ENSP00000258428	ENSP00000263642
ENSP00000258428	ENSP00000264233
ENSP00000258428	ENSP00000265081
ENSP00000258428	ENSP00000265990
ENSP00000258428	ENSP00000267430

ENSP00000258428	ENSP00000268124
ENSP00000258428	ENSP00000269214
ENSP00000258428	ENSP00000282516
ENSP00000258428	ENSP00000296509
ENSP00000258428	ENSP00000297338
ENSP00000258428	ENSP00000299206
ENSP00000258428	ENSP00000306561
ENSP00000258428	ENSP00000306999
ENSP00000258428	ENSP00000307853
ENSP00000258428	ENSP00000308944
ENSP00000258428	ENSP00000310520
ENSP00000258428	ENSP00000311368
ENSP00000258428	ENSP00000311873
ENSP00000258428	ENSP00000313350
ENSP00000258428	ENSP00000313851
ENSP00000258428	ENSP00000318977
ENSP00000258428	ENSP00000323439
ENSP00000258428	ENSP00000327145
ENSP00000258428	ENSP00000336606
ENSP00000258428	ENSP00000340305
ENSP00000258428	ENSP00000341382
ENSP00000258428	ENSP00000343392
ENSP00000258428	ENSP00000347978
ENSP00000258428	ENSP00000348089
ENSP00000258428	ENSP00000351284
ENSP00000258428	ENSP00000351697
ENSP00000258428	ENSP00000351947
ENSP00000258428	ENSP00000352257
ENSP00000258428	ENSP00000354720
ENSP00000258428	ENSP00000354957
ENSP00000258428	ENSP00000358478
ENSP00000258428	ENSP00000361310
ENSP00000258428	ENSP00000364270
ENSP00000258428	ENSP00000365588
ENSP00000258428	ENSP00000367808
ENSP00000258428	ENSP00000368438
ENSP00000258428	ENSP00000369335
ENSP00000258428	ENSP00000370150
ENSP00000258428	ENSP00000371475
ENSP00000258428	ENSP00000372316
ENSP00000258428	ENSP00000373060
ENSP00000258428	ENSP00000381657
ENSP00000258428	ENSP00000384048
ENSP00000258428	ENSP00000387429
ENSP00000258428	ENSP00000396484
ENSP00000258428	ENSP00000404318

ENSP00000258428	ENSP00000406490
ENSP00000258428	ENSP00000409167
ENSP00000258428	ENSP00000415212
ENSP00000258428	ENSP00000416511
ENSP00000258443	ENSP00000335076
ENSP00000258443	ENSP00000363680
ENSP00000258455	ENSP00000259469
ENSP00000258455	ENSP00000260102
ENSP00000258455	ENSP00000260443
ENSP00000258455	ENSP00000260956
ENSP00000258455	ENSP00000261015
ENSP00000258455	ENSP00000262584
ENSP00000258455	ENSP00000263629
ENSP00000258455	ENSP00000264995
ENSP00000258455	ENSP00000265107
ENSP00000258455	ENSP00000268206
ENSP00000258455	ENSP00000268379
ENSP00000258455	ENSP00000268661
ENSP00000258455	ENSP00000270625
ENSP00000258455	ENSP00000272418
ENSP00000258455	ENSP00000274606
ENSP00000258455	ENSP00000278412
ENSP00000258455	ENSP00000278572
ENSP00000258455	ENSP00000280557
ENSP00000258455	ENSP00000281141
ENSP00000258455	ENSP00000283109
ENSP00000258455	ENSP00000285873
ENSP00000258455	ENSP00000287038
ENSP00000258455	ENSP00000289371
ENSP00000258455	ENSP00000294189
ENSP00000258455	ENSP00000295491
ENSP00000258455	ENSP00000296577
ENSP00000258455	ENSP00000296674
ENSP00000258455	ENSP00000296802
ENSP00000258455	ENSP00000296805
ENSP00000258455	ENSP00000297579
ENSP00000258455	ENSP00000297908
ENSP00000258455	ENSP00000298283
ENSP00000258455	ENSP00000300151
ENSP00000258455	ENSP00000300213
ENSP00000258455	ENSP00000301585
ENSP00000258455	ENSP00000302896
ENSP00000258455	ENSP00000303191
ENSP00000258455	ENSP00000305653
ENSP00000258455	ENSP00000306548
ENSP00000258455	ENSP00000307889

ENSP00000258455	ENSP00000308179
ENSP00000258455	ENSP00000308897
ENSP00000258455	ENSP00000309334
ENSP00000258455	ENSP00000311028
ENSP00000258455	ENSP00000311430
ENSP00000258455	ENSP00000314193
ENSP00000258455	ENSP00000314311
ENSP00000258455	ENSP00000317376
ENSP00000258455	ENSP00000318374
ENSP00000258455	ENSP00000318604
ENSP00000258455	ENSP00000318646
ENSP00000258455	ENSP00000322419
ENSP00000258455	ENSP00000323856
ENSP00000258455	ENSP00000326981
ENSP00000258455	ENSP00000327179
ENSP00000258455	ENSP00000331019
ENSP00000258455	ENSP00000336702
ENSP00000258455	ENSP00000337127
ENSP00000258455	ENSP00000337839
ENSP00000258455	ENSP00000339095
ENSP00000258455	ENSP00000339795
ENSP00000258455	ENSP00000341692
ENSP00000258455	ENSP00000341885
ENSP00000258455	ENSP00000345156
ENSP00000258455	ENSP00000346015
ENSP00000258455	ENSP00000346022
ENSP00000258455	ENSP00000346027
ENSP00000258455	ENSP00000346037
ENSP00000258455	ENSP00000346050
ENSP00000258455	ENSP00000346067
ENSP00000258455	ENSP00000346120
ENSP00000258455	ENSP00000347087
ENSP00000258455	ENSP00000352706
ENSP00000258455	ENSP00000354522
ENSP00000258455	ENSP00000354739
ENSP00000258455	ENSP00000355471
ENSP00000258455	ENSP00000356134
ENSP00000258455	ENSP00000358812
ENSP00000258455	ENSP00000359345
ENSP00000258455	ENSP00000360782
ENSP00000258455	ENSP00000361076
ENSP00000258455	ENSP00000361206
ENSP00000258455	ENSP00000362036
ENSP00000258455	ENSP00000362208
ENSP00000258455	ENSP00000362744
ENSP00000258455	ENSP00000363018

ENSP00000258455	ENSP00000363676
ENSP00000258455	ENSP00000364320
ENSP00000258455	ENSP00000368790
ENSP00000258455	ENSP00000368887
ENSP00000258455	ENSP00000369757
ENSP00000258455	ENSP00000372445
ENSP00000258455	ENSP00000373404
ENSP00000258455	ENSP00000375730
ENSP00000258455	ENSP00000377486
ENSP00000258455	ENSP00000377640
ENSP00000258455	ENSP00000379213
ENSP00000258455	ENSP00000379888
ENSP00000258455	ENSP00000380156
ENSP00000258455	ENSP00000382250
ENSP00000258455	ENSP00000382779
ENSP00000258455	ENSP00000386717
ENSP00000258455	ENSP00000389103
ENSP00000258455	ENSP00000393241
ENSP00000258455	ENSP00000402338
ENSP00000258455	ENSP00000403175
ENSP00000258455	ENSP00000412583
ENSP00000258455	ENSP00000414321
ENSP00000258455	ENSP00000416110
ENSP00000258494	ENSP00000301585
ENSP00000258499	ENSP00000262193
ENSP00000258499	ENSP00000263974
ENSP00000258499	ENSP00000265990
ENSP00000258499	ENSP00000272645
ENSP00000258499	ENSP00000284320
ENSP00000258499	ENSP00000287598
ENSP00000258499	ENSP00000291688
ENSP00000258499	ENSP00000296328
ENSP00000258499	ENSP00000301788
ENSP00000258499	ENSP00000302530
ENSP00000258499	ENSP00000307260
ENSP00000258499	ENSP00000309572
ENSP00000258499	ENSP00000314949
ENSP00000258499	ENSP00000333401
ENSP00000258499	ENSP00000348551
ENSP00000258499	ENSP00000350009
ENSP00000258499	ENSP00000360473
ENSP00000258499	ENSP00000370930
ENSP00000258499	ENSP00000381863
ENSP00000258499	ENSP00000406888
ENSP00000258648	ENSP00000260956
ENSP00000258648	ENSP00000261531

ENSP00000258648	ENSP00000261758
ENSP00000258648	ENSP00000261884
ENSP00000258648	ENSP00000262367
ENSP00000258648	ENSP00000263205
ENSP00000258648	ENSP00000263390
ENSP00000258648	ENSP00000265350
ENSP00000258648	ENSP00000267064
ENSP00000258648	ENSP00000268711
ENSP00000258648	ENSP00000282007
ENSP00000258648	ENSP00000284981
ENSP00000258648	ENSP00000286317
ENSP00000258648	ENSP00000290663
ENSP00000258648	ENSP00000292035
ENSP00000258648	ENSP00000293777
ENSP00000258648	ENSP00000297347
ENSP00000258648	ENSP00000299543
ENSP00000258648	ENSP00000300651
ENSP00000258648	ENSP00000323720
ENSP00000258648	ENSP00000325612
ENSP00000258648	ENSP00000326128
ENSP00000258648	ENSP00000326767
ENSP00000258648	ENSP00000337340
ENSP00000258648	ENSP00000342434
ENSP00000258648	ENSP00000349640
ENSP00000258648	ENSP00000354620
ENSP00000258648	ENSP00000354951
ENSP00000258648	ENSP00000355317
ENSP00000258648	ENSP00000357047
ENSP00000258648	ENSP00000357907
ENSP00000258648	ENSP00000360371
ENSP00000258648	ENSP00000360532
ENSP00000258648	ENSP00000362948
ENSP00000258648	ENSP00000363193
ENSP00000258648	ENSP00000365588
ENSP00000258648	ENSP00000369162
ENSP00000258648	ENSP00000373060
ENSP00000258648	ENSP00000384048
ENSP00000258648	ENSP00000387429
ENSP00000258648	ENSP00000391774
ENSP00000258648	ENSP00000392617
ENSP00000258648	ENSP00000396484
ENSP00000258648	ENSP00000404318
ENSP00000258648	ENSP00000409167
ENSP00000258648	ENSP00000415212
ENSP00000258648	ENSP00000416511
ENSP00000258648	ENSP00000418734

ENSP00000258654	ENSP00000262544
ENSP00000258654	ENSP00000264028
ENSP00000258654	ENSP00000265175
ENSP00000258654	ENSP00000282884
ENSP00000258654	ENSP00000286448
ENSP00000258654	ENSP00000291574
ENSP00000258654	ENSP00000294179
ENSP00000258654	ENSP00000305442
ENSP00000258654	ENSP00000305459
ENSP00000258654	ENSP00000310521
ENSP00000258654	ENSP00000311134
ENSP00000258654	ENSP00000325002
ENSP00000258654	ENSP00000328079
ENSP00000258654	ENSP00000329419
ENSP00000258654	ENSP00000355629
ENSP00000258654	ENSP00000356411
ENSP00000258654	ENSP00000361076
ENSP00000258654	ENSP00000362576
ENSP00000258654	ENSP00000363349
ENSP00000258654	ENSP00000390783
ENSP00000258654	ENSP00000397441
ENSP00000258654	ENSP00000403397
ENSP00000258654	ENSP00000406988
ENSP00000258654	ENSP00000409952
ENSP00000258662	ENSP00000264228
ENSP00000258679	ENSP00000355153
ENSP00000258682	ENSP00000279230
ENSP00000258682	ENSP00000296238
ENSP00000258682	ENSP00000312664
ENSP00000258682	ENSP00000324573
ENSP00000258682	ENSP00000331727
ENSP00000258682	ENSP00000345731
ENSP00000258682	ENSP00000349022
ENSP00000258682	ENSP00000351593
ENSP00000258682	ENSP00000353508
ENSP00000258682	ENSP00000367408
ENSP00000258682	ENSP00000381863
ENSP00000258711	ENSP00000259253
ENSP00000258742	ENSP00000264029
ENSP00000258742	ENSP00000264932
ENSP00000258742	ENSP00000272902
ENSP00000258742	ENSP00000296490
ENSP00000258742	ENSP00000301329
ENSP00000258742	ENSP00000304185
ENSP00000258742	ENSP00000307004
ENSP00000258742	ENSP00000308622

ENSP00000258742	ENSP00000311768
ENSP00000258742	ENSP00000313243
ENSP00000258742	ENSP00000320130
ENSP00000258742	ENSP00000331879
ENSP00000258742	ENSP00000346206
ENSP00000258742	ENSP00000349022
ENSP00000258742	ENSP00000357440
ENSP00000258742	ENSP00000358854
ENSP00000258742	ENSP00000359444
ENSP00000258742	ENSP00000360286
ENSP00000258742	ENSP00000362463
ENSP00000258742	ENSP00000373509
ENSP00000258742	ENSP00000381970
ENSP00000258743	ENSP00000338157
ENSP00000258743	ENSP00000338799
ENSP00000258743	ENSP00000357470
ENSP00000258743	ENSP00000379213
ENSP00000258770	ENSP00000304311
ENSP00000258770	ENSP00000397435
ENSP00000258774	ENSP00000260810
ENSP00000258774	ENSP00000261454
ENSP00000258774	ENSP00000261881
ENSP00000258774	ENSP00000262519
ENSP00000258774	ENSP00000265140
ENSP00000258774	ENSP00000267197
ENSP00000258774	ENSP00000278616
ENSP00000258774	ENSP00000283033
ENSP00000258774	ENSP00000286091
ENSP00000258774	ENSP00000322570
ENSP00000258774	ENSP00000323439
ENSP00000258774	ENSP00000325863
ENSP00000258774	ENSP00000340879
ENSP00000258774	ENSP00000343741
ENSP00000258774	ENSP00000351697
ENSP00000258774	ENSP00000352257
ENSP00000258774	ENSP00000354957
ENSP00000258774	ENSP00000361310
ENSP00000258774	ENSP00000363021
ENSP00000258774	ENSP00000364270
ENSP00000258774	ENSP00000368349
ENSP00000258774	ENSP00000368438
ENSP00000258774	ENSP00000370151
ENSP00000258774	ENSP00000371475
ENSP00000258774	ENSP00000392270
ENSP00000258774	ENSP00000420298
ENSP00000258829	ENSP00000342828

ENSP00000258873	ENSP00000320646
ENSP00000258873	ENSP00000357650
ENSP00000258873	ENSP00000359380
ENSP00000258873	ENSP00000367407
ENSP00000258873	ENSP00000369042
ENSP00000258874	ENSP00000300181
ENSP00000258874	ENSP00000310623
ENSP00000258874	ENSP00000356290
ENSP00000258874	ENSP00000377617
ENSP00000258886	ENSP00000264029
ENSP00000258886	ENSP00000268668
ENSP00000258886	ENSP00000274030
ENSP00000258886	ENSP00000282541
ENSP00000258886	ENSP00000296684
ENSP00000258886	ENSP00000307786
ENSP00000258886	ENSP00000317159
ENSP00000258886	ENSP00000339826
ENSP00000258886	ENSP00000341838
ENSP00000258886	ENSP00000342056
ENSP00000258886	ENSP00000344866
ENSP00000258886	ENSP00000348632
ENSP00000258886	ENSP00000354251
ENSP00000258886	ENSP00000358737
ENSP00000258886	ENSP00000366482
ENSP00000258886	ENSP00000367923
ENSP00000258886	ENSP00000368565
ENSP00000258886	ENSP00000409275
ENSP00000258947	ENSP00000262419
ENSP00000258947	ENSP00000263925
ENSP00000258947	ENSP00000264447
ENSP00000258947	ENSP00000268720
ENSP00000258947	ENSP00000288422
ENSP00000258947	ENSP00000289013
ENSP00000258947	ENSP00000295878
ENSP00000258947	ENSP00000297431
ENSP00000258947	ENSP00000297532
ENSP00000258947	ENSP00000309606
ENSP00000258947	ENSP00000309629
ENSP00000258947	ENSP00000311134
ENSP00000258947	ENSP00000311505
ENSP00000258947	ENSP00000321706
ENSP00000258947	ENSP00000324292
ENSP00000258947	ENSP00000329967
ENSP00000258947	ENSP00000349822
ENSP00000258947	ENSP00000354270
ENSP00000258947	ENSP00000354777

ENSP00000258947	ENSP00000355840
ENSP00000258947	ENSP00000355944
ENSP00000258947	ENSP00000357887
ENSP00000258947	ENSP00000358994
ENSP00000258947	ENSP00000359115
ENSP00000258947	ENSP00000359740
ENSP00000258947	ENSP00000361186
ENSP00000258947	ENSP00000362807
ENSP00000258947	ENSP00000363021
ENSP00000258947	ENSP00000364126
ENSP00000258947	ENSP00000365175
ENSP00000258947	ENSP00000370410
ENSP00000258947	ENSP00000371805
ENSP00000258947	ENSP00000379612
ENSP00000258947	ENSP00000381795
ENSP00000258947	ENSP00000396876
ENSP00000258947	ENSP00000401980
ENSP00000258947	ENSP00000419371
ENSP00000258975	ENSP00000263629
ENSP00000258975	ENSP00000289371
ENSP00000258975	ENSP00000301585
ENSP00000258975	ENSP00000304350
ENSP00000258975	ENSP00000343885
ENSP00000258975	ENSP00000362576
ENSP00000258991	ENSP00000276416
ENSP00000258991	ENSP00000295030
ENSP00000258991	ENSP00000348602
ENSP00000259008	ENSP00000260810
ENSP00000259008	ENSP00000260947
ENSP00000259008	ENSP00000261245
ENSP00000259008	ENSP00000261424
ENSP00000259008	ENSP00000261584
ENSP00000259008	ENSP00000264926
ENSP00000259008	ENSP00000265056
ENSP00000259008	ENSP00000265849
ENSP00000259008	ENSP00000267430
ENSP00000259008	ENSP00000268043
ENSP00000259008	ENSP00000269214
ENSP00000259008	ENSP00000273398
ENSP00000259008	ENSP00000279281
ENSP00000259008	ENSP00000283875
ENSP00000259008	ENSP00000287598
ENSP00000259008	ENSP00000296509
ENSP00000259008	ENSP00000297338
ENSP00000259008	ENSP00000302530
ENSP00000259008	ENSP00000306999

ENSP00000259008	ENSP00000307853
ENSP00000259008	ENSP00000310520
ENSP00000259008	ENSP00000313350
ENSP00000259008	ENSP00000321636
ENSP00000259008	ENSP00000322570
ENSP00000259008	ENSP00000325290
ENSP00000259008	ENSP00000325863
ENSP00000259008	ENSP00000333837
ENSP00000259008	ENSP00000347232
ENSP00000259008	ENSP00000348168
ENSP00000259008	ENSP00000350283
ENSP00000259008	ENSP00000351697
ENSP00000259008	ENSP00000355185
ENSP00000259008	ENSP00000359818
ENSP00000259008	ENSP00000360621
ENSP00000259008	ENSP00000364270
ENSP00000259008	ENSP00000366135
ENSP00000259008	ENSP00000368349
ENSP00000259008	ENSP00000369335
ENSP00000259008	ENSP00000382562
ENSP00000259008	ENSP00000388872
ENSP00000259008	ENSP00000406490
ENSP00000259050	ENSP00000261942
ENSP00000259050	ENSP00000263202
ENSP00000259050	ENSP00000264515
ENSP00000259050	ENSP00000273064
ENSP00000259050	ENSP00000288199
ENSP00000259050	ENSP00000295066
ENSP00000259050	ENSP00000304370
ENSP00000259050	ENSP00000315791
ENSP00000259050	ENSP00000331487
ENSP00000259050	ENSP00000343535
ENSP00000259050	ENSP00000357998
ENSP00000259050	ENSP00000360916
ENSP00000259050	ENSP00000379457
ENSP00000259050	ENSP00000383719
ENSP00000259075	ENSP00000262294
ENSP00000259075	ENSP00000309871
ENSP00000259075	ENSP00000325376
ENSP00000259075	ENSP00000325448
ENSP00000259075	ENSP00000329411
ENSP00000259075	ENSP00000329967
ENSP00000259075	ENSP00000343535
ENSP00000259075	ENSP00000344668
ENSP00000259075	ENSP00000347504
ENSP00000259075	ENSP00000351813

ENSP00000259075	ENSP00000352522
ENSP00000259075	ENSP00000364794
ENSP00000259075	ENSP00000377954
ENSP00000259154	ENSP00000261758
ENSP00000259154	ENSP00000262265
ENSP00000259154	ENSP00000263966
ENSP00000259154	ENSP00000264705
ENSP00000259154	ENSP00000265970
ENSP00000259154	ENSP00000280350
ENSP00000259154	ENSP00000280772
ENSP00000259154	ENSP00000283195
ENSP00000259154	ENSP00000298292
ENSP00000259154	ENSP00000304102
ENSP00000259154	ENSP00000311766
ENSP00000259154	ENSP00000317790
ENSP00000259154	ENSP00000325690
ENSP00000259154	ENSP00000348163
ENSP00000259154	ENSP00000361824
ENSP00000259154	ENSP00000380921
ENSP00000259206	ENSP00000300086
ENSP00000259206	ENSP00000330959
ENSP00000259206	ENSP00000350249
ENSP00000259229	ENSP00000371994
ENSP00000259235	ENSP00000262306
ENSP00000259235	ENSP00000264183
ENSP00000259235	ENSP00000268058
ENSP00000259235	ENSP00000299424
ENSP00000259235	ENSP00000335321
ENSP00000259235	ENSP00000340271
ENSP00000259235	ENSP00000342755
ENSP00000259235	ENSP00000350512
ENSP00000259235	ENSP00000353622
ENSP00000259235	ENSP00000358605
ENSP00000259235	ENSP00000360515
ENSP00000259235	ENSP00000368801
ENSP00000259235	ENSP00000386161
ENSP00000259235	ENSP00000417281
ENSP00000259253	ENSP00000262764
ENSP00000259253	ENSP00000264065
ENSP00000259253	ENSP00000272227
ENSP00000259253	ENSP00000280700
ENSP00000259253	ENSP00000286091
ENSP00000259253	ENSP00000295770
ENSP00000259253	ENSP00000297625
ENSP00000259253	ENSP00000307491
ENSP00000259253	ENSP00000317177

ENSP00000259253	ENSP00000329466
ENSP00000259253	ENSP00000336702
ENSP00000259253	ENSP00000340466
ENSP00000259253	ENSP00000342793
ENSP00000259253	ENSP00000347948
ENSP00000259253	ENSP00000351075
ENSP00000259253	ENSP00000356807
ENSP00000259253	ENSP00000360149
ENSP00000259253	ENSP00000364893
ENSP00000259396	ENSP00000263368
ENSP00000259396	ENSP00000264731
ENSP00000259396	ENSP00000277865
ENSP00000259396	ENSP00000295897
ENSP00000259396	ENSP00000303148
ENSP00000259396	ENSP00000312584
ENSP00000259396	ENSP00000381891
ENSP00000259400	ENSP00000261531
ENSP00000259400	ENSP00000263897
ENSP00000259400	ENSP00000269122
ENSP00000259400	ENSP00000272322
ENSP00000259400	ENSP00000286448
ENSP00000259400	ENSP00000294179
ENSP00000259400	ENSP00000303191
ENSP00000259400	ENSP00000305255
ENSP00000259400	ENSP00000305810
ENSP00000259400	ENSP00000309457
ENSP00000259400	ENSP00000310521
ENSP00000259400	ENSP00000317714
ENSP00000259400	ENSP00000326534
ENSP00000259400	ENSP00000326921
ENSP00000259400	ENSP00000327650
ENSP00000259400	ENSP00000331201
ENSP00000259400	ENSP00000344055
ENSP00000259400	ENSP00000347408
ENSP00000259400	ENSP00000347498
ENSP00000259400	ENSP00000352425
ENSP00000259400	ENSP00000360183
ENSP00000259400	ENSP00000366225
ENSP00000259400	ENSP00000375881
ENSP00000259400	ENSP00000386759
ENSP00000259400	ENSP00000390783
ENSP00000259400	ENSP00000403397
ENSP00000259400	ENSP00000418661
ENSP00000259406	ENSP00000264335
ENSP00000259406	ENSP00000360141
ENSP00000259406	ENSP00000368699

ENSP00000259406	ENSP00000371483
ENSP00000259406	ENSP00000377496
ENSP00000259457	ENSP00000260187
ENSP00000259457	ENSP00000261712
ENSP00000259457	ENSP00000262193
ENSP00000259457	ENSP00000262966
ENSP00000259457	ENSP00000263881
ENSP00000259457	ENSP00000264639
ENSP00000259457	ENSP00000277541
ENSP00000259457	ENSP00000282050
ENSP00000259457	ENSP00000290541
ENSP00000259457	ENSP00000293362
ENSP00000259457	ENSP00000295901
ENSP00000259457	ENSP00000298852
ENSP00000259457	ENSP00000302239
ENSP00000259457	ENSP00000309474
ENSP00000259457	ENSP00000310129
ENSP00000259457	ENSP00000311505
ENSP00000259457	ENSP00000314837
ENSP00000259457	ENSP00000325919
ENSP00000259457	ENSP00000348442
ENSP00000259457	ENSP00000350708
ENSP00000259457	ENSP00000351284
ENSP00000259457	ENSP00000362334
ENSP00000259457	ENSP00000369335
ENSP00000259457	ENSP00000370222
ENSP00000259457	ENSP00000384211
ENSP00000259457	ENSP00000396937
ENSP00000259457	ENSP00000403852
ENSP00000259457	ENSP00000413632
ENSP00000259457	ENSP00000418734
ENSP00000259467	ENSP00000312262
ENSP00000259467	ENSP00000317334
ENSP00000259467	ENSP00000363021
ENSP00000259469	ENSP00000260102
ENSP00000259469	ENSP00000260443
ENSP00000259469	ENSP00000261015
ENSP00000259469	ENSP00000262584
ENSP00000259469	ENSP00000263073
ENSP00000259469	ENSP00000263208
ENSP00000259469	ENSP00000264995
ENSP00000259469	ENSP00000265100
ENSP00000259469	ENSP00000265245
ENSP00000259469	ENSP00000268206
ENSP00000259469	ENSP00000268616
ENSP00000259469	ENSP00000268661

ENSP00000259469	ENSP00000269499
ENSP00000259469	ENSP00000274242
ENSP00000259469	ENSP00000274606
ENSP00000259469	ENSP00000278856
ENSP00000259469	ENSP00000287038
ENSP00000259469	ENSP00000288937
ENSP00000259469	ENSP00000294189
ENSP00000259469	ENSP00000296411
ENSP00000259469	ENSP00000296674
ENSP00000259469	ENSP00000296802
ENSP00000259469	ENSP00000296805
ENSP00000259469	ENSP00000297908
ENSP00000259469	ENSP00000298283
ENSP00000259469	ENSP00000300151
ENSP00000259469	ENSP00000302886
ENSP00000259469	ENSP00000304903
ENSP00000259469	ENSP00000306548
ENSP00000259469	ENSP00000307889
ENSP00000259469	ENSP00000308897
ENSP00000259469	ENSP00000309334
ENSP00000259469	ENSP00000309433
ENSP00000259469	ENSP00000309830
ENSP00000259469	ENSP00000311028
ENSP00000259469	ENSP00000311430
ENSP00000259469	ENSP00000317376
ENSP00000259469	ENSP00000322419
ENSP00000259469	ENSP00000325312
ENSP00000259469	ENSP00000337839
ENSP00000259469	ENSP00000338862
ENSP00000259469	ENSP00000339095
ENSP00000259469	ENSP00000339795
ENSP00000259469	ENSP00000340766
ENSP00000259469	ENSP00000341082
ENSP00000259469	ENSP00000341538
ENSP00000259469	ENSP00000343392
ENSP00000259469	ENSP00000345156
ENSP00000259469	ENSP00000346015
ENSP00000259469	ENSP00000346022
ENSP00000259469	ENSP00000346027
ENSP00000259469	ENSP00000346037
ENSP00000259469	ENSP00000346050
ENSP00000259469	ENSP00000346088
ENSP00000259469	ENSP00000352021
ENSP00000259469	ENSP00000353998
ENSP00000259469	ENSP00000354040
ENSP00000259469	ENSP00000354525

ENSP00000259469	ENSP00000354739
ENSP00000259469	ENSP00000355261
ENSP00000259469	ENSP00000357998
ENSP00000259469	ENSP00000359345
ENSP00000259469	ENSP00000361076
ENSP00000259469	ENSP00000362306
ENSP00000259469	ENSP00000362744
ENSP00000259469	ENSP00000363018
ENSP00000259469	ENSP00000363384
ENSP00000259469	ENSP00000363676
ENSP00000259469	ENSP00000363944
ENSP00000259469	ENSP00000364320
ENSP00000259469	ENSP00000369757
ENSP00000259469	ENSP00000369965
ENSP00000259469	ENSP00000373404
ENSP00000259469	ENSP00000375730
ENSP00000259469	ENSP00000377486
ENSP00000259469	ENSP00000377640
ENSP00000259469	ENSP00000377865
ENSP00000259469	ENSP00000378160
ENSP00000259469	ENSP00000379607
ENSP00000259469	ENSP00000379888
ENSP00000259469	ENSP00000380156
ENSP00000259469	ENSP00000386717
ENSP00000259469	ENSP00000388330
ENSP00000259469	ENSP00000389103
ENSP00000259469	ENSP00000391723
ENSP00000259469	ENSP00000393393
ENSP00000259469	ENSP00000402338
ENSP00000259469	ENSP00000414321
ENSP00000259469	ENSP00000417763
ENSP00000259469	ENSP00000419370
ENSP00000259477	ENSP00000295685
ENSP00000259477	ENSP00000317334
ENSP00000259477	ENSP00000320924
ENSP00000259477	ENSP00000325448
ENSP00000259477	ENSP00000326238
ENSP00000259477	ENSP00000348163
ENSP00000259477	ENSP00000352802
ENSP00000259477	ENSP00000365147
ENSP00000259477	ENSP00000365891
ENSP00000259477	ENSP00000376946
ENSP00000259477	ENSP00000380431
ENSP00000259477	ENSP00000386878
ENSP00000259512	ENSP00000261534
ENSP00000259512	ENSP00000261942

ENSP00000259512	ENSP00000263202
ENSP00000259512	ENSP00000263228
ENSP00000259512	ENSP00000272638
ENSP00000259512	ENSP00000273067
ENSP00000259512	ENSP00000274140
ENSP00000259512	ENSP00000290158
ENSP00000259512	ENSP00000290649
ENSP00000259512	ENSP00000294119
ENSP00000259512	ENSP00000295897
ENSP00000259512	ENSP00000296350
ENSP00000259512	ENSP00000305810
ENSP00000259512	ENSP00000309141
ENSP00000259512	ENSP00000312506
ENSP00000259512	ENSP00000317087
ENSP00000259512	ENSP00000326813
ENSP00000259512	ENSP00000331487
ENSP00000259512	ENSP00000337053
ENSP00000259512	ENSP00000346130
ENSP00000259512	ENSP00000347140
ENSP00000259512	ENSP00000349970
ENSP00000259512	ENSP00000366395
ENSP00000259512	ENSP00000381282
ENSP00000259512	ENSP00000392330
ENSP00000259512	ENSP00000401445
ENSP00000259569	ENSP00000261396
ENSP00000259569	ENSP00000261741
ENSP00000259569	ENSP00000262193
ENSP00000259569	ENSP00000262554
ENSP00000259569	ENSP00000262765
ENSP00000259569	ENSP00000262982
ENSP00000259569	ENSP00000264883
ENSP00000259569	ENSP00000265351
ENSP00000259569	ENSP00000268379
ENSP00000259569	ENSP00000269980
ENSP00000259569	ENSP00000273398
ENSP00000259569	ENSP00000273962
ENSP00000259569	ENSP00000280346
ENSP00000259569	ENSP00000283195
ENSP00000259569	ENSP00000283875
ENSP00000259569	ENSP00000287675
ENSP00000259569	ENSP00000290158
ENSP00000259569	ENSP00000296257
ENSP00000259569	ENSP00000296411
ENSP00000259569	ENSP00000296802
ENSP00000259569	ENSP00000297579
ENSP00000259569	ENSP00000300648

ENSP00000259569	ENSP00000302768
ENSP00000259569	ENSP00000302886
ENSP00000259569	ENSP00000306983
ENSP00000259569	ENSP00000307272
ENSP00000259569	ENSP00000307889
ENSP00000259569	ENSP00000309433
ENSP00000259569	ENSP00000310042
ENSP00000259569	ENSP00000310668
ENSP00000259569	ENSP00000312356
ENSP00000259569	ENSP00000313953
ENSP00000259569	ENSP00000316879
ENSP00000259569	ENSP00000318195
ENSP00000259569	ENSP00000318351
ENSP00000259569	ENSP00000318977
ENSP00000259569	ENSP00000325312
ENSP00000259569	ENSP00000327197
ENSP00000259569	ENSP00000331817
ENSP00000259569	ENSP00000332444
ENSP00000259569	ENSP00000336712
ENSP00000259569	ENSP00000338573
ENSP00000259569	ENSP00000340281
ENSP00000259569	ENSP00000340823
ENSP00000259569	ENSP00000343701
ENSP00000259569	ENSP00000344460
ENSP00000259569	ENSP00000346050
ENSP00000259569	ENSP00000346566
ENSP00000259569	ENSP00000350967
ENSP00000259569	ENSP00000353741
ENSP00000259569	ENSP00000354720
ENSP00000259569	ENSP00000354742
ENSP00000259569	ENSP00000354812
ENSP00000259569	ENSP00000354957
ENSP00000259569	ENSP00000357748
ENSP00000259569	ENSP00000359000
ENSP00000259569	ENSP00000359305
ENSP00000259569	ENSP00000360031
ENSP00000259569	ENSP00000360286
ENSP00000259569	ENSP00000361949
ENSP00000259569	ENSP00000362900
ENSP00000259569	ENSP00000363779
ENSP00000259569	ENSP00000365811
ENSP00000259569	ENSP00000369042
ENSP00000259569	ENSP00000369695
ENSP00000259569	ENSP00000370589
ENSP00000259569	ENSP00000372689
ENSP00000259569	ENSP00000376309

ENSP00000259569	ENSP00000377655
ENSP00000259569	ENSP00000377865
ENSP00000259569	ENSP00000379888
ENSP00000259569	ENSP00000386992
ENSP00000259569	ENSP00000389103
ENSP00000259569	ENSP00000394382
ENSP00000259569	ENSP00000394791
ENSP00000259569	ENSP00000401371
ENSP00000259569	ENSP00000402527
ENSP00000259605	ENSP00000264731
ENSP00000259605	ENSP00000272645
ENSP00000259605	ENSP00000312735
ENSP00000259605	ENSP00000324124
ENSP00000259605	ENSP00000347836
ENSP00000259605	ENSP00000355028
ENSP00000259605	ENSP00000403852
ENSP00000259607	ENSP00000265077
ENSP00000259607	ENSP00000286758
ENSP00000259608	ENSP00000340944
ENSP00000259608	ENSP00000364898
ENSP00000259632	ENSP00000354791
ENSP00000259632	ENSP00000408910
ENSP00000259632	ENSP00000414906
ENSP00000259633	ENSP00000342681
ENSP00000259633	ENSP00000364898
ENSP00000259667	ENSP00000261245
ENSP00000259667	ENSP00000301729
ENSP00000259667	ENSP00000302886
ENSP00000259667	ENSP00000336702
ENSP00000259667	ENSP00000356652
ENSP00000259667	ENSP00000357880
ENSP00000259726	ENSP00000294008
ENSP00000259750	ENSP00000260102
ENSP00000259750	ENSP00000260643
ENSP00000259750	ENSP00000262544
ENSP00000259750	ENSP00000263657
ENSP00000259750	ENSP00000263791
ENSP00000259750	ENSP00000268802
ENSP00000259750	ENSP00000278572
ENSP00000259750	ENSP00000283109
ENSP00000259750	ENSP00000285968
ENSP00000259750	ENSP00000297338
ENSP00000259750	ENSP00000301364
ENSP00000259750	ENSP00000308699
ENSP00000259750	ENSP00000331817
ENSP00000259750	ENSP00000349016

ENSP00000259750	ENSP00000353508
ENSP00000259750	ENSP00000354720
ENSP00000259750	ENSP00000355471
ENSP00000259750	ENSP00000355541
ENSP00000259750	ENSP00000356548
ENSP00000259750	ENSP00000360676
ENSP00000259750	ENSP00000363559
ENSP00000259750	ENSP00000363779
ENSP00000259751	ENSP00000345731
ENSP00000259782	ENSP00000276603
ENSP00000259803	ENSP00000262367
ENSP00000259803	ENSP00000368805
ENSP00000259803	ENSP00000394791
ENSP00000259806	ENSP00000297689
ENSP00000259806	ENSP00000359531
ENSP00000259808	ENSP00000260187
ENSP00000259808	ENSP00000263036
ENSP00000259808	ENSP00000263056
ENSP00000259808	ENSP00000286186
ENSP00000259808	ENSP00000300589
ENSP00000259808	ENSP00000301838
ENSP00000259808	ENSP00000313829
ENSP00000259808	ENSP00000321503
ENSP00000259808	ENSP00000327647
ENSP00000259808	ENSP00000337797
ENSP00000259808	ENSP00000340305
ENSP00000259808	ENSP00000341189
ENSP00000259808	ENSP00000341268
ENSP00000259808	ENSP00000343313
ENSP00000259808	ENSP00000347979
ENSP00000259808	ENSP00000348632
ENSP00000259808	ENSP00000354927
ENSP00000259808	ENSP00000358335
ENSP00000259808	ENSP00000360215
ENSP00000259808	ENSP00000363089
ENSP00000259808	ENSP00000369100
ENSP00000259808	ENSP00000374455
ENSP00000259808	ENSP00000374552
ENSP00000259808	ENSP00000376250
ENSP00000259808	ENSP00000379612
ENSP00000259845	ENSP00000301607
ENSP00000259873	ENSP00000381031
ENSP00000259874	ENSP00000297156
ENSP00000259874	ENSP00000358022
ENSP00000259895	ENSP00000261245
ENSP00000259895	ENSP00000265963

ENSP00000259895	ENSP00000265990
ENSP00000259895	ENSP00000282892
ENSP00000259895	ENSP00000283875
ENSP00000259895	ENSP00000285021
ENSP00000259895	ENSP00000285398
ENSP00000259895	ENSP00000314949
ENSP00000259895	ENSP00000347978
ENSP00000259895	ENSP00000348168
ENSP00000259895	ENSP00000350283
ENSP00000259895	ENSP00000350708
ENSP00000259895	ENSP00000359531
ENSP00000259895	ENSP00000375809
ENSP00000259895	ENSP00000377969
ENSP00000259895	ENSP00000396251
ENSP00000259963	ENSP00000337053
ENSP00000259963	ENSP00000366395
ENSP00000260008	ENSP00000260526
ENSP00000260008	ENSP00000262300
ENSP00000260008	ENSP00000263773
ENSP00000260008	ENSP00000281282
ENSP00000260008	ENSP00000303507
ENSP00000260008	ENSP00000322170
ENSP00000260008	ENSP00000322608
ENSP00000260008	ENSP00000322957
ENSP00000260008	ENSP00000332592
ENSP00000260008	ENSP00000348349
ENSP00000260008	ENSP00000351333
ENSP00000260008	ENSP00000360076
ENSP00000260008	ENSP00000360183
ENSP00000260008	ENSP00000365891
ENSP00000260008	ENSP00000366121
ENSP00000260008	ENSP00000370589
ENSP00000260008	ENSP00000377793
ENSP00000260008	ENSP00000380450
ENSP00000260008	ENSP00000381567
ENSP00000260008	ENSP00000391249
ENSP00000260008	ENSP00000397598
ENSP00000260008	ENSP00000402084
ENSP00000260008	ENSP00000407879
ENSP00000260045	ENSP00000350512
ENSP00000260049	ENSP00000263326
ENSP00000260049	ENSP00000280357
ENSP00000260102	ENSP00000260443
ENSP00000260102	ENSP00000261637
ENSP00000260102	ENSP00000262584
ENSP00000260102	ENSP00000263657

ENSP00000260102	ENSP00000263857
ENSP00000260102	ENSP00000264028
ENSP00000260102	ENSP00000264670
ENSP00000260102	ENSP00000264995
ENSP00000260102	ENSP00000265100
ENSP00000260102	ENSP00000268206
ENSP00000260102	ENSP00000268661
ENSP00000260102	ENSP00000272418
ENSP00000260102	ENSP00000274242
ENSP00000260102	ENSP00000274606
ENSP00000260102	ENSP00000278572
ENSP00000260102	ENSP00000279242
ENSP00000260102	ENSP00000287038
ENSP00000260102	ENSP00000288937
ENSP00000260102	ENSP00000294008
ENSP00000260102	ENSP00000294189
ENSP00000260102	ENSP00000296102
ENSP00000260102	ENSP00000296411
ENSP00000260102	ENSP00000296674
ENSP00000260102	ENSP00000296802
ENSP00000260102	ENSP00000296805
ENSP00000260102	ENSP00000297908
ENSP00000260102	ENSP00000298283
ENSP00000260102	ENSP00000300151
ENSP00000260102	ENSP00000301585
ENSP00000260102	ENSP00000302886
ENSP00000260102	ENSP00000302896
ENSP00000260102	ENSP00000306080
ENSP00000260102	ENSP00000306548
ENSP00000260102	ENSP00000307889
ENSP00000260102	ENSP00000308275
ENSP00000260102	ENSP00000308897
ENSP00000260102	ENSP00000309334
ENSP00000260102	ENSP00000309830
ENSP00000260102	ENSP00000311028
ENSP00000260102	ENSP00000311430
ENSP00000260102	ENSP00000312311
ENSP00000260102	ENSP00000312735
ENSP00000260102	ENSP00000314949
ENSP00000260102	ENSP00000315017
ENSP00000260102	ENSP00000317376
ENSP00000260102	ENSP00000318646
ENSP00000260102	ENSP00000322396
ENSP00000260102	ENSP00000322419
ENSP00000260102	ENSP00000325312
ENSP00000260102	ENSP00000326981

ENSP00000260102	ENSP00000328079
ENSP00000260102	ENSP00000329419
ENSP00000260102	ENSP00000333401
ENSP00000260102	ENSP00000338573
ENSP00000260102	ENSP00000339095
ENSP00000260102	ENSP00000339145
ENSP00000260102	ENSP00000339723
ENSP00000260102	ENSP00000339795
ENSP00000260102	ENSP00000339845
ENSP00000260102	ENSP00000341082
ENSP00000260102	ENSP00000341885
ENSP00000260102	ENSP00000342381
ENSP00000260102	ENSP00000343392
ENSP00000260102	ENSP00000343745
ENSP00000260102	ENSP00000345156
ENSP00000260102	ENSP00000346022
ENSP00000260102	ENSP00000346027
ENSP00000260102	ENSP00000346037
ENSP00000260102	ENSP00000346050
ENSP00000260102	ENSP00000346088
ENSP00000260102	ENSP00000346725
ENSP00000260102	ENSP00000354040
ENSP00000260102	ENSP00000354525
ENSP00000260102	ENSP00000354739
ENSP00000260102	ENSP00000357048
ENSP00000260102	ENSP00000359345
ENSP00000260102	ENSP00000360031
ENSP00000260102	ENSP00000360498
ENSP00000260102	ENSP00000361076
ENSP00000260102	ENSP00000362153
ENSP00000260102	ENSP00000362744
ENSP00000260102	ENSP00000363018
ENSP00000260102	ENSP00000363384
ENSP00000260102	ENSP00000363676
ENSP00000260102	ENSP00000364320
ENSP00000260102	ENSP00000369757
ENSP00000260102	ENSP00000370930
ENSP00000260102	ENSP00000373404
ENSP00000260102	ENSP00000375730
ENSP00000260102	ENSP00000377486
ENSP00000260102	ENSP00000377640
ENSP00000260102	ENSP00000377865
ENSP00000260102	ENSP00000378160
ENSP00000260102	ENSP00000379607
ENSP00000260102	ENSP00000379888
ENSP00000260102	ENSP00000380156

ENSP00000260102	ENSP00000386717
ENSP00000260102	ENSP00000389103
ENSP00000260102	ENSP00000391723
ENSP00000260102	ENSP00000393393
ENSP00000260102	ENSP00000402338
ENSP00000260102	ENSP00000414321
ENSP00000260102	ENSP00000417602
ENSP00000260102	ENSP00000419740
ENSP00000260118	ENSP00000273398
ENSP00000260126	ENSP00000379607
ENSP00000260129	ENSP00000260210
ENSP00000260129	ENSP00000260803
ENSP00000260129	ENSP00000262133
ENSP00000260129	ENSP00000262367
ENSP00000260129	ENSP00000262518
ENSP00000260129	ENSP00000263360
ENSP00000260129	ENSP00000264951
ENSP00000260129	ENSP00000286317
ENSP00000260129	ENSP00000287936
ENSP00000260129	ENSP00000291688
ENSP00000260129	ENSP00000296577
ENSP00000260129	ENSP00000298746
ENSP00000260129	ENSP00000300146
ENSP00000260129	ENSP00000300648
ENSP00000260129	ENSP00000300651
ENSP00000260129	ENSP00000301740
ENSP00000260129	ENSP00000307863
ENSP00000260129	ENSP00000309433
ENSP00000260129	ENSP00000310448
ENSP00000260129	ENSP00000310596
ENSP00000260129	ENSP00000312318
ENSP00000260129	ENSP00000320566
ENSP00000260129	ENSP00000324203
ENSP00000260129	ENSP00000326806
ENSP00000260129	ENSP00000329102
ENSP00000260129	ENSP00000331879
ENSP00000260129	ENSP00000332103
ENSP00000260129	ENSP00000339090
ENSP00000260129	ENSP00000341947
ENSP00000260129	ENSP00000344241
ENSP00000260129	ENSP00000348010
ENSP00000260129	ENSP00000348107
ENSP00000260129	ENSP00000351894
ENSP00000260129	ENSP00000352257
ENSP00000260129	ENSP00000352678
ENSP00000260129	ENSP00000355205

ENSP00000260129	ENSP00000358563
ENSP00000260129	ENSP00000360515
ENSP00000260129	ENSP00000361577
ENSP00000260129	ENSP00000362592
ENSP00000260129	ENSP00000362936
ENSP00000260129	ENSP00000364289
ENSP00000260129	ENSP00000368686
ENSP00000260129	ENSP00000369042
ENSP00000260129	ENSP00000370589
ENSP00000260129	ENSP00000374213
ENSP00000260129	ENSP00000376973
ENSP00000260129	ENSP00000379888
ENSP00000260129	ENSP00000380982
ENSP00000260129	ENSP00000386134
ENSP00000260129	ENSP00000412566
ENSP00000260130	ENSP00000307046
ENSP00000260130	ENSP00000323280
ENSP00000260130	ENSP00000324560
ENSP00000260130	ENSP00000329797
ENSP00000260130	ENSP00000344666
ENSP00000260130	ENSP00000346466
ENSP00000260130	ENSP00000353154
ENSP00000260130	ENSP00000363763
ENSP00000260130	ENSP00000368698
ENSP00000260130	ENSP00000380054
ENSP00000260187	ENSP00000261601
ENSP00000260187	ENSP00000263974
ENSP00000260187	ENSP00000264515
ENSP00000260187	ENSP00000264552
ENSP00000260187	ENSP00000265562
ENSP00000260187	ENSP00000266126
ENSP00000260187	ENSP00000272645
ENSP00000260187	ENSP00000290663
ENSP00000260187	ENSP00000304592
ENSP00000260187	ENSP00000307387
ENSP00000260187	ENSP00000314420
ENSP00000260187	ENSP00000326921
ENSP00000260187	ENSP00000331201
ENSP00000260187	ENSP00000331487
ENSP00000260187	ENSP00000341268
ENSP00000260187	ENSP00000342026
ENSP00000260187	ENSP00000343535
ENSP00000260187	ENSP00000345530
ENSP00000260187	ENSP00000347301
ENSP00000260187	ENSP00000347498
ENSP00000260187	ENSP00000348442

ENSP00000260187	ENSP00000353826
ENSP00000260187	ENSP00000354673
ENSP00000260187	ENSP00000355899
ENSP00000260187	ENSP00000360899
ENSP00000260187	ENSP00000362608
ENSP00000260187	ENSP00000417281
ENSP00000260197	ENSP00000281187
ENSP00000260197	ENSP00000299138
ENSP00000260197	ENSP00000349437
ENSP00000260210	ENSP00000260363
ENSP00000260210	ENSP00000261531
ENSP00000260210	ENSP00000262518
ENSP00000260210	ENSP00000263576
ENSP00000260210	ENSP00000264233
ENSP00000260210	ENSP00000266529
ENSP00000260210	ENSP00000267812
ENSP00000260210	ENSP00000268482
ENSP00000260210	ENSP00000276201
ENSP00000260210	ENSP00000291688
ENSP00000260210	ENSP00000295488
ENSP00000260210	ENSP00000296215
ENSP00000260210	ENSP00000296702
ENSP00000260210	ENSP00000297151
ENSP00000260210	ENSP00000298532
ENSP00000260210	ENSP00000300146
ENSP00000260210	ENSP00000300413
ENSP00000260210	ENSP00000301740
ENSP00000260210	ENSP00000302160
ENSP00000260210	ENSP00000303191
ENSP00000260210	ENSP00000304370
ENSP00000260210	ENSP00000304593
ENSP00000260210	ENSP00000304903
ENSP00000260210	ENSP00000305924
ENSP00000260210	ENSP00000307260
ENSP00000260210	ENSP00000307863
ENSP00000260210	ENSP00000310448
ENSP00000260210	ENSP00000310596
ENSP00000260210	ENSP00000310723
ENSP00000260210	ENSP00000311344
ENSP00000260210	ENSP00000313829
ENSP00000260210	ENSP00000318861
ENSP00000260210	ENSP00000324203
ENSP00000260210	ENSP00000326806
ENSP00000260210	ENSP00000327716
ENSP00000260210	ENSP00000332103
ENSP00000260210	ENSP00000334148

ENSP00000260210	ENSP00000335321
ENSP00000260210	ENSP00000339090
ENSP00000260210	ENSP00000342374
ENSP00000260210	ENSP00000346986
ENSP00000260210	ENSP00000348107
ENSP00000260210	ENSP00000351492
ENSP00000260210	ENSP00000354951
ENSP00000260210	ENSP00000355205
ENSP00000260210	ENSP00000356405
ENSP00000260210	ENSP00000357631
ENSP00000260210	ENSP00000358854
ENSP00000260210	ENSP00000359454
ENSP00000260210	ENSP00000360286
ENSP00000260210	ENSP00000360532
ENSP00000260210	ENSP00000361577
ENSP00000260210	ENSP00000362532
ENSP00000260210	ENSP00000362936
ENSP00000260210	ENSP00000364813
ENSP00000260210	ENSP00000366135
ENSP00000260210	ENSP00000366557
ENSP00000260210	ENSP00000366607
ENSP00000260210	ENSP00000366843
ENSP00000260210	ENSP00000368755
ENSP00000260210	ENSP00000374213
ENSP00000260210	ENSP00000376973
ENSP00000260210	ENSP00000377262
ENSP00000260210	ENSP00000380460
ENSP00000260210	ENSP00000381198
ENSP00000260210	ENSP00000383402
ENSP00000260210	ENSP00000386759
ENSP00000260210	ENSP00000402240
ENSP00000260210	ENSP00000404151
ENSP00000260210	ENSP00000410758
ENSP00000260210	ENSP00000412566
ENSP00000260210	ENSP00000414006
ENSP00000260210	ENSP00000414634
ENSP00000260257	ENSP00000260443
ENSP00000260257	ENSP00000262027
ENSP00000260257	ENSP00000263629
ENSP00000260257	ENSP00000274353
ENSP00000260257	ENSP00000281828
ENSP00000260257	ENSP00000298569
ENSP00000260257	ENSP00000302114
ENSP00000260257	ENSP00000302801
ENSP00000260257	ENSP00000303279
ENSP00000260257	ENSP00000335675

ENSP00000260257	ENSP00000340093
ENSP00000260257	ENSP00000341543
ENSP00000260257	ENSP00000344241
ENSP00000260257	ENSP00000348107
ENSP00000260257	ENSP00000350967
ENSP00000260257	ENSP00000351380
ENSP00000260257	ENSP00000354204
ENSP00000260257	ENSP00000356329
ENSP00000260257	ENSP00000356405
ENSP00000260257	ENSP00000356548
ENSP00000260257	ENSP00000360286
ENSP00000260257	ENSP00000362576
ENSP00000260257	ENSP00000364794
ENSP00000260257	ENSP00000367029
ENSP00000260257	ENSP00000367498
ENSP00000260257	ENSP00000368140
ENSP00000260257	ENSP00000370083
ENSP00000260257	ENSP00000370119
ENSP00000260257	ENSP00000370377
ENSP00000260257	ENSP00000370607
ENSP00000260257	ENSP00000374399
ENSP00000260257	ENSP00000394085
ENSP00000260270	ENSP00000293195
ENSP00000260270	ENSP00000297185
ENSP00000260270	ENSP00000307786
ENSP00000260270	ENSP00000310623
ENSP00000260270	ENSP00000363205
ENSP00000260323	ENSP00000362399
ENSP00000260324	ENSP00000311505
ENSP00000260327	ENSP00000261596
ENSP00000260327	ENSP00000263857
ENSP00000260327	ENSP00000299601
ENSP00000260327	ENSP00000315693
ENSP00000260327	ENSP00000356405
ENSP00000260327	ENSP00000368066
ENSP00000260327	ENSP00000374280
ENSP00000260327	ENSP00000377769
ENSP00000260361	ENSP00000263774
ENSP00000260361	ENSP00000266544
ENSP00000260361	ENSP00000354687
ENSP00000260361	ENSP00000362058
ENSP00000260361	ENSP00000369176
ENSP00000260363	ENSP00000262305
ENSP00000260363	ENSP00000263208
ENSP00000260363	ENSP00000296137
ENSP00000260363	ENSP00000296509

ENSP00000260363	ENSP00000297338
ENSP00000260363	ENSP00000297814
ENSP00000260363	ENSP00000301332
ENSP00000260363	ENSP00000302239
ENSP00000260363	ENSP00000309871
ENSP00000260363	ENSP00000322180
ENSP00000260363	ENSP00000323858
ENSP00000260363	ENSP00000329466
ENSP00000260363	ENSP00000336868
ENSP00000260363	ENSP00000339933
ENSP00000260363	ENSP00000342181
ENSP00000260363	ENSP00000346566
ENSP00000260363	ENSP00000347719
ENSP00000260363	ENSP00000354851
ENSP00000260363	ENSP00000355090
ENSP00000260363	ENSP00000355388
ENSP00000260363	ENSP00000355560
ENSP00000260363	ENSP00000356530
ENSP00000260363	ENSP00000356989
ENSP00000260363	ENSP00000362592
ENSP00000260363	ENSP00000364119
ENSP00000260363	ENSP00000368976
ENSP00000260363	ENSP00000377793
ENSP00000260363	ENSP00000379607
ENSP00000260363	ENSP00000380921
ENSP00000260363	ENSP00000382562
ENSP00000260363	ENSP00000393596
ENSP00000260372	ENSP00000282058
ENSP00000260372	ENSP00000369871
ENSP00000260382	ENSP00000352265
ENSP00000260383	ENSP00000261965
ENSP00000260383	ENSP00000264335
ENSP00000260383	ENSP00000271643
ENSP00000260383	ENSP00000309189
ENSP00000260383	ENSP00000322957
ENSP00000260383	ENSP00000324292
ENSP00000260383	ENSP00000341213
ENSP00000260383	ENSP00000360532
ENSP00000260383	ENSP00000362532
ENSP00000260383	ENSP00000367049
ENSP00000260383	ENSP00000380921
ENSP00000260383	ENSP00000382562
ENSP00000260383	ENSP00000384391
ENSP00000260383	ENSP00000404232
ENSP00000260383	ENSP00000416097
ENSP00000260385	ENSP00000313059

ENSP00000260385	ENSP00000345656
ENSP00000260385	ENSP00000414996
ENSP00000260403	ENSP00000260731
ENSP00000260403	ENSP00000262982
ENSP00000260403	ENSP00000301764
ENSP00000260403	ENSP00000314491
ENSP00000260403	ENSP00000319918
ENSP00000260403	ENSP00000321711
ENSP00000260403	ENSP00000355388
ENSP00000260403	ENSP00000359818
ENSP00000260403	ENSP00000360310
ENSP00000260403	ENSP00000380718
ENSP00000260433	ENSP00000261507
ENSP00000260433	ENSP00000264027
ENSP00000260433	ENSP00000265896
ENSP00000260433	ENSP00000277010
ENSP00000260433	ENSP00000341662
ENSP00000260433	ENSP00000348762
ENSP00000260433	ENSP00000367910
ENSP00000260442	ENSP00000293288
ENSP00000260442	ENSP00000309103
ENSP00000260442	ENSP00000314132
ENSP00000260442	ENSP00000329623
ENSP00000260442	ENSP00000353059
ENSP00000260442	ENSP00000358022
ENSP00000260442	ENSP00000363591
ENSP00000260443	ENSP00000261015
ENSP00000260443	ENSP00000262584
ENSP00000260443	ENSP00000264230
ENSP00000260443	ENSP00000264670
ENSP00000260443	ENSP00000264995
ENSP00000260443	ENSP00000265100
ENSP00000260443	ENSP00000265245
ENSP00000260443	ENSP00000268661
ENSP00000260443	ENSP00000270538
ENSP00000260443	ENSP00000274242
ENSP00000260443	ENSP00000274606
ENSP00000260443	ENSP00000278856
ENSP00000260443	ENSP00000287038
ENSP00000260443	ENSP00000294189
ENSP00000260443	ENSP00000296674
ENSP00000260443	ENSP00000296802
ENSP00000260443	ENSP00000298283
ENSP00000260443	ENSP00000300151
ENSP00000260443	ENSP00000302886
ENSP00000260443	ENSP00000302896

ENSP00000260443	ENSP00000305702
ENSP00000260443	ENSP00000306548
ENSP00000260443	ENSP00000307525
ENSP00000260443	ENSP00000307889
ENSP00000260443	ENSP00000308897
ENSP00000260443	ENSP00000309334
ENSP00000260443	ENSP00000309830
ENSP00000260443	ENSP00000310042
ENSP00000260443	ENSP00000311028
ENSP00000260443	ENSP00000311430
ENSP00000260443	ENSP00000314348
ENSP00000260443	ENSP00000317376
ENSP00000260443	ENSP00000317992
ENSP00000260443	ENSP00000322419
ENSP00000260443	ENSP00000326981
ENSP00000260443	ENSP00000328690
ENSP00000260443	ENSP00000338573
ENSP00000260443	ENSP00000338862
ENSP00000260443	ENSP00000339145
ENSP00000260443	ENSP00000339795
ENSP00000260443	ENSP00000341551
ENSP00000260443	ENSP00000343392
ENSP00000260443	ENSP00000345156
ENSP00000260443	ENSP00000345917
ENSP00000260443	ENSP00000346015
ENSP00000260443	ENSP00000346022
ENSP00000260443	ENSP00000346027
ENSP00000260443	ENSP00000346037
ENSP00000260443	ENSP00000346050
ENSP00000260443	ENSP00000346067
ENSP00000260443	ENSP00000346088
ENSP00000260443	ENSP00000346725
ENSP00000260443	ENSP00000348596
ENSP00000260443	ENSP00000351380
ENSP00000260443	ENSP00000352021
ENSP00000260443	ENSP00000353246
ENSP00000260443	ENSP00000354040
ENSP00000260443	ENSP00000354522
ENSP00000260443	ENSP00000354525
ENSP00000260443	ENSP00000354739
ENSP00000260443	ENSP00000359345
ENSP00000260443	ENSP00000359688
ENSP00000260443	ENSP00000361076
ENSP00000260443	ENSP00000362153
ENSP00000260443	ENSP00000363018
ENSP00000260443	ENSP00000363384

ENSP00000260443	ENSP00000363559
ENSP00000260443	ENSP00000363676
ENSP00000260443	ENSP00000364320
ENSP00000260443	ENSP00000365806
ENSP00000260443	ENSP00000366519
ENSP00000260443	ENSP00000368565
ENSP00000260443	ENSP00000368887
ENSP00000260443	ENSP00000369757
ENSP00000260443	ENSP00000371201
ENSP00000260443	ENSP00000373090
ENSP00000260443	ENSP00000373404
ENSP00000260443	ENSP00000375730
ENSP00000260443	ENSP00000377865
ENSP00000260443	ENSP00000378160
ENSP00000260443	ENSP00000379888
ENSP00000260443	ENSP00000380156
ENSP00000260443	ENSP00000380982
ENSP00000260443	ENSP00000382392
ENSP00000260443	ENSP00000386717
ENSP00000260443	ENSP00000388464
ENSP00000260443	ENSP00000389103
ENSP00000260443	ENSP00000393393
ENSP00000260443	ENSP00000394290
ENSP00000260443	ENSP00000402338
ENSP00000260443	ENSP00000403576
ENSP00000260443	ENSP00000409074
ENSP00000260443	ENSP00000409367
ENSP00000260443	ENSP00000411162
ENSP00000260443	ENSP00000419740
ENSP00000260447	ENSP00000378890
ENSP00000260453	ENSP00000381031
ENSP00000260508	ENSP00000356231
ENSP00000260508	ENSP00000365534
ENSP00000260508	ENSP00000379607
ENSP00000260526	ENSP00000315659
ENSP00000260526	ENSP00000316845
ENSP00000260526	ENSP00000318884
ENSP00000260526	ENSP00000327315
ENSP00000260526	ENSP00000333950
ENSP00000260526	ENSP00000361009
ENSP00000260526	ENSP00000376410
ENSP00000260526	ENSP00000384675
ENSP00000260526	ENSP00000394794
ENSP00000260526	ENSP00000397351
ENSP00000260598	ENSP00000294973
ENSP00000260598	ENSP00000304903

ENSP00000260598	ENSP00000315931
ENSP00000260598	ENSP00000333019
ENSP00000260598	ENSP00000344683
ENSP00000260598	ENSP00000354532
ENSP00000260598	ENSP00000356290
ENSP00000260598	ENSP00000412566
ENSP00000260605	ENSP00000265104
ENSP00000260605	ENSP00000348965
ENSP00000260605	ENSP00000354791
ENSP00000260605	ENSP00000358081
ENSP00000260643	ENSP00000262544
ENSP00000260643	ENSP00000262607
ENSP00000260643	ENSP00000298937
ENSP00000260643	ENSP00000308339
ENSP00000260643	ENSP00000314615
ENSP00000260643	ENSP00000341551
ENSP00000260643	ENSP00000342381
ENSP00000260643	ENSP00000350967
ENSP00000260643	ENSP00000356530
ENSP00000260643	ENSP00000357887
ENSP00000260643	ENSP00000361965
ENSP00000260643	ENSP00000363779
ENSP00000260643	ENSP00000386918
ENSP00000260643	ENSP00000401445
ENSP00000260643	ENSP00000419599
ENSP00000260648	ENSP00000373477
ENSP00000260649	ENSP00000303507
ENSP00000260649	ENSP00000320378
ENSP00000260649	ENSP00000366121
ENSP00000260662	ENSP00000271002
ENSP00000260662	ENSP00000283006
ENSP00000260662	ENSP00000325677
ENSP00000260662	ENSP00000335463
ENSP00000260662	ENSP00000336868
ENSP00000260662	ENSP00000337289
ENSP00000260662	ENSP00000364737
ENSP00000260662	ENSP00000377007
ENSP00000260665	ENSP00000262608
ENSP00000260665	ENSP00000262803
ENSP00000260665	ENSP00000264645
ENSP00000260665	ENSP00000276326
ENSP00000260665	ENSP00000297338
ENSP00000260665	ENSP00000301585
ENSP00000260665	ENSP00000305790
ENSP00000260665	ENSP00000312649
ENSP00000260665	ENSP00000313829

ENSP00000260665	ENSP00000329466
ENSP00000260665	ENSP00000337393
ENSP00000260665	ENSP00000338990
ENSP00000260665	ENSP00000343412
ENSP00000260665	ENSP00000349016
ENSP00000260665	ENSP00000363745
ENSP00000260665	ENSP00000364289
ENSP00000260665	ENSP00000372445
ENSP00000260665	ENSP00000381863
ENSP00000260731	ENSP00000261531
ENSP00000260731	ENSP00000261881
ENSP00000260731	ENSP00000261965
ENSP00000260731	ENSP00000262315
ENSP00000260731	ENSP00000263181
ENSP00000260731	ENSP00000264335
ENSP00000260731	ENSP00000264926
ENSP00000260731	ENSP00000265104
ENSP00000260731	ENSP00000265148
ENSP00000260731	ENSP00000271452
ENSP00000260731	ENSP00000276014
ENSP00000260731	ENSP00000282077
ENSP00000260731	ENSP00000282572
ENSP00000260731	ENSP00000285873
ENSP00000260731	ENSP00000286428
ENSP00000260731	ENSP00000287598
ENSP00000260731	ENSP00000296509
ENSP00000260731	ENSP00000296733
ENSP00000260731	ENSP00000297338
ENSP00000260731	ENSP00000299543
ENSP00000260731	ENSP00000300249
ENSP00000260731	ENSP00000300584
ENSP00000260731	ENSP00000301332
ENSP00000260731	ENSP00000302530
ENSP00000260731	ENSP00000306123
ENSP00000260731	ENSP00000308546
ENSP00000260731	ENSP00000318884
ENSP00000260731	ENSP00000322180
ENSP00000260731	ENSP00000325017
ENSP00000260731	ENSP00000327009
ENSP00000260731	ENSP00000332592
ENSP00000260731	ENSP00000334044
ENSP00000260731	ENSP00000334188
ENSP00000260731	ENSP00000335357
ENSP00000260731	ENSP00000336868
ENSP00000260731	ENSP00000339484
ENSP00000260731	ENSP00000341243

ENSP00000260731	ENSP00000343741
ENSP00000260731	ENSP00000343745
ENSP00000260731	ENSP00000346566
ENSP00000260731	ENSP00000347719
ENSP00000260731	ENSP00000348722
ENSP00000260731	ENSP00000348965
ENSP00000260731	ENSP00000349016
ENSP00000260731	ENSP00000349238
ENSP00000260731	ENSP00000351665
ENSP00000260731	ENSP00000351885
ENSP00000260731	ENSP00000353793
ENSP00000260731	ENSP00000354791
ENSP00000260731	ENSP00000354916
ENSP00000260731	ENSP00000355560
ENSP00000260731	ENSP00000356405
ENSP00000260731	ENSP00000356579
ENSP00000260731	ENSP00000356989
ENSP00000260731	ENSP00000357651
ENSP00000260731	ENSP00000360473
ENSP00000260731	ENSP00000360532
ENSP00000260731	ENSP00000361800
ENSP00000260731	ENSP00000361990
ENSP00000260731	ENSP00000362306
ENSP00000260731	ENSP00000362334
ENSP00000260731	ENSP00000364204
ENSP00000260731	ENSP00000366249
ENSP00000260731	ENSP00000368699
ENSP00000260731	ENSP00000368976
ENSP00000260731	ENSP00000369736
ENSP00000260731	ENSP00000373039
ENSP00000260731	ENSP00000374566
ENSP00000260731	ENSP00000377793
ENSP00000260731	ENSP00000378529
ENSP00000260731	ENSP00000382562
ENSP00000260731	ENSP00000383145
ENSP00000260731	ENSP00000396152
ENSP00000260731	ENSP00000410416
ENSP00000260731	ENSP00000414402
ENSP00000260731	ENSP00000418734
ENSP00000260753	ENSP00000263208
ENSP00000260753	ENSP00000269385
ENSP00000260753	ENSP00000286428
ENSP00000260753	ENSP00000296509
ENSP00000260753	ENSP00000301332
ENSP00000260753	ENSP00000322180
ENSP00000260753	ENSP00000323858

ENSP00000260753	ENSP00000346566
ENSP00000260753	ENSP00000354851
ENSP00000260753	ENSP00000355090
ENSP00000260753	ENSP00000368976
ENSP00000260753	ENSP00000384490
ENSP00000260766	ENSP00000262039
ENSP00000260766	ENSP00000263121
ENSP00000260766	ENSP00000265970
ENSP00000260766	ENSP00000275820
ENSP00000260766	ENSP00000286364
ENSP00000260766	ENSP00000295065
ENSP00000260766	ENSP00000322218
ENSP00000260766	ENSP00000322457
ENSP00000260766	ENSP00000352121
ENSP00000260766	ENSP00000361064
ENSP00000260766	ENSP00000361141
ENSP00000260766	ENSP00000373399
ENSP00000260803	ENSP00000261868
ENSP00000260803	ENSP00000262300
ENSP00000260803	ENSP00000264951
ENSP00000260803	ENSP00000302801
ENSP00000260803	ENSP00000307863
ENSP00000260803	ENSP00000321449
ENSP00000260803	ENSP00000323424
ENSP00000260803	ENSP00000326411
ENSP00000260803	ENSP00000326806
ENSP00000260803	ENSP00000356989
ENSP00000260803	ENSP00000361010
ENSP00000260803	ENSP00000364289
ENSP00000260803	ENSP00000386134
ENSP00000260803	ENSP00000402240
ENSP00000260803	ENSP00000417748
ENSP00000260810	ENSP00000260947
ENSP00000260810	ENSP00000261424
ENSP00000260810	ENSP00000262315
ENSP00000260810	ENSP00000262460
ENSP00000260810	ENSP00000263201
ENSP00000260810	ENSP00000265056
ENSP00000260810	ENSP00000265433
ENSP00000260810	ENSP00000265728
ENSP00000260810	ENSP00000265849
ENSP00000260810	ENSP00000268058
ENSP00000260810	ENSP00000276533
ENSP00000260810	ENSP00000278616
ENSP00000260810	ENSP00000286800
ENSP00000260810	ENSP00000287598

ENSP00000260810	ENSP00000292475
ENSP00000260810	ENSP00000296273
ENSP00000260810	ENSP00000296509
ENSP00000260810	ENSP00000300738
ENSP00000260810	ENSP00000302530
ENSP00000260810	ENSP00000305899
ENSP00000260810	ENSP00000307525
ENSP00000260810	ENSP00000308944
ENSP00000260810	ENSP00000312870
ENSP00000260810	ENSP00000313171
ENSP00000260810	ENSP00000322570
ENSP00000260810	ENSP00000323663
ENSP00000260810	ENSP00000334294
ENSP00000260810	ENSP00000340879
ENSP00000260810	ENSP00000343741
ENSP00000260810	ENSP00000348775
ENSP00000260810	ENSP00000349576
ENSP00000260810	ENSP00000350283
ENSP00000260810	ENSP00000354522
ENSP00000260810	ENSP00000363708
ENSP00000260810	ENSP00000364895
ENSP00000260810	ENSP00000366513
ENSP00000260810	ENSP00000366863
ENSP00000260810	ENSP00000369411
ENSP00000260810	ENSP00000370151
ENSP00000260810	ENSP00000371475
ENSP00000260810	ENSP00000372023
ENSP00000260810	ENSP00000373060
ENSP00000260810	ENSP00000377384
ENSP00000260810	ENSP00000381657
ENSP00000260810	ENSP00000396704
ENSP00000260810	ENSP00000401018
ENSP00000260810	ENSP00000417281
ENSP00000260818	ENSP00000261531
ENSP00000260818	ENSP00000264157
ENSP00000260818	ENSP00000273398
ENSP00000260818	ENSP00000286448
ENSP00000260818	ENSP00000305255
ENSP00000260818	ENSP00000310521
ENSP00000260818	ENSP00000317714
ENSP00000260818	ENSP00000327650
ENSP00000260818	ENSP00000355721
ENSP00000260818	ENSP00000362399
ENSP00000260818	ENSP00000374529
ENSP00000260818	ENSP00000390783
ENSP00000260947	ENSP00000263468

ENSP00000260947	ENSP00000263857
ENSP00000260947	ENSP00000265081
ENSP00000260947	ENSP00000265433
ENSP00000260947	ENSP00000278919
ENSP00000260947	ENSP00000286800
ENSP00000260947	ENSP00000294119
ENSP00000260947	ENSP00000296702
ENSP00000260947	ENSP00000296930
ENSP00000260947	ENSP00000314949
ENSP00000260947	ENSP00000315791
ENSP00000260947	ENSP00000323050
ENSP00000260947	ENSP00000325863
ENSP00000260947	ENSP00000334002
ENSP00000260947	ENSP00000341170
ENSP00000260947	ENSP00000343412
ENSP00000260947	ENSP00000347184
ENSP00000260947	ENSP00000350283
ENSP00000260947	ENSP00000352257
ENSP00000260947	ENSP00000355759
ENSP00000260947	ENSP00000357965
ENSP00000260947	ENSP00000358778
ENSP00000260947	ENSP00000360116
ENSP00000260947	ENSP00000363779
ENSP00000260947	ENSP00000365588
ENSP00000260947	ENSP00000366434
ENSP00000260947	ENSP00000368438
ENSP00000260947	ENSP00000368797
ENSP00000260947	ENSP00000369497
ENSP00000260947	ENSP00000369716
ENSP00000260947	ENSP00000378180
ENSP00000260947	ENSP00000378338
ENSP00000260947	ENSP00000381031
ENSP00000260947	ENSP00000381045
ENSP00000260947	ENSP00000387911
ENSP00000260952	ENSP00000361699
ENSP00000260956	ENSP00000261531
ENSP00000260956	ENSP00000261601
ENSP00000260956	ENSP00000262215
ENSP00000260956	ENSP00000263857
ENSP00000260956	ENSP00000264670
ENSP00000260956	ENSP00000264951
ENSP00000260956	ENSP00000265304
ENSP00000260956	ENSP00000265881
ENSP00000260956	ENSP00000267064
ENSP00000260956	ENSP00000269385
ENSP00000260956	ENSP00000272645

ENSP00000260956	ENSP00000273962
ENSP00000260956	ENSP00000274311
ENSP00000260956	ENSP00000274606
ENSP00000260956	ENSP00000283109
ENSP00000260956	ENSP00000284719
ENSP00000260956	ENSP00000285896
ENSP00000260956	ENSP00000286317
ENSP00000260956	ENSP00000286428
ENSP00000260956	ENSP00000290663
ENSP00000260956	ENSP00000296509
ENSP00000260956	ENSP00000296783
ENSP00000260956	ENSP00000296802
ENSP00000260956	ENSP00000298310
ENSP00000260956	ENSP00000298746
ENSP00000260956	ENSP00000302160
ENSP00000260956	ENSP00000302801
ENSP00000260956	ENSP00000302896
ENSP00000260956	ENSP00000309433
ENSP00000260956	ENSP00000314441
ENSP00000260956	ENSP00000316879
ENSP00000260956	ENSP00000318195
ENSP00000260956	ENSP00000318646
ENSP00000260956	ENSP00000319531
ENSP00000260956	ENSP00000322016
ENSP00000260956	ENSP00000322396
ENSP00000260956	ENSP00000322570
ENSP00000260956	ENSP00000323856
ENSP00000260956	ENSP00000324122
ENSP00000260956	ENSP00000324124
ENSP00000260956	ENSP00000325369
ENSP00000260956	ENSP00000325677
ENSP00000260956	ENSP00000326981
ENSP00000260956	ENSP00000327821
ENSP00000260956	ENSP00000329466
ENSP00000260956	ENSP00000331310
ENSP00000260956	ENSP00000337405
ENSP00000260956	ENSP00000339095
ENSP00000260956	ENSP00000340281
ENSP00000260956	ENSP00000342181
ENSP00000260956	ENSP00000342889
ENSP00000260956	ENSP00000345917
ENSP00000260956	ENSP00000346022
ENSP00000260956	ENSP00000346088
ENSP00000260956	ENSP00000347948
ENSP00000260956	ENSP00000348168
ENSP00000260956	ENSP00000348722

ENSP00000260956	ENSP00000350491
ENSP00000260956	ENSP00000351697
ENSP00000260956	ENSP00000353246
ENSP00000260956	ENSP00000354125
ENSP00000260956	ENSP00000354522
ENSP00000260956	ENSP00000354558
ENSP00000260956	ENSP00000356405
ENSP00000260956	ENSP00000356476
ENSP00000260956	ENSP00000356989
ENSP00000260956	ENSP00000357452
ENSP00000260956	ENSP00000357748
ENSP00000260956	ENSP00000357880
ENSP00000260956	ENSP00000358563
ENSP00000260956	ENSP00000359127
ENSP00000260956	ENSP00000359321
ENSP00000260956	ENSP00000359345
ENSP00000260956	ENSP00000360473
ENSP00000260956	ENSP00000360876
ENSP00000260956	ENSP00000361465
ENSP00000260956	ENSP00000361982
ENSP00000260956	ENSP00000362105
ENSP00000260956	ENSP00000362456
ENSP00000260956	ENSP00000362576
ENSP00000260956	ENSP00000362744
ENSP00000260956	ENSP00000363384
ENSP00000260956	ENSP00000363559
ENSP00000260956	ENSP00000363676
ENSP00000260956	ENSP00000363779
ENSP00000260956	ENSP00000363944
ENSP00000260956	ENSP00000364289
ENSP00000260956	ENSP00000364320
ENSP00000260956	ENSP00000365837
ENSP00000260956	ENSP00000366135
ENSP00000260956	ENSP00000367407
ENSP00000260956	ENSP00000368349
ENSP00000260956	ENSP00000368438
ENSP00000260956	ENSP00000368565
ENSP00000260956	ENSP00000368927
ENSP00000260956	ENSP00000369042
ENSP00000260956	ENSP00000369757
ENSP00000260956	ENSP00000370589
ENSP00000260956	ENSP00000370741
ENSP00000260956	ENSP00000374399
ENSP00000260956	ENSP00000379607
ENSP00000260956	ENSP00000379888
ENSP00000260956	ENSP00000380460

ENSP00000260956	ENSP00000381216
ENSP00000260956	ENSP00000382392
ENSP00000260956	ENSP00000384490
ENSP00000260956	ENSP00000391774
ENSP00000260956	ENSP00000402338
ENSP00000260956	ENSP00000403852
ENSP00000260956	ENSP00000414321
ENSP00000260956	ENSP00000417229
ENSP00000260970	ENSP00000283351
ENSP00000260970	ENSP00000294053
ENSP00000260970	ENSP00000297151
ENSP00000260970	ENSP00000297338
ENSP00000260970	ENSP00000305958
ENSP00000260970	ENSP00000314949
ENSP00000260970	ENSP00000315859
ENSP00000260970	ENSP00000324274
ENSP00000260970	ENSP00000326424
ENSP00000260970	ENSP00000329357
ENSP00000260970	ENSP00000331817
ENSP00000260970	ENSP00000336724
ENSP00000260970	ENSP00000337127
ENSP00000260970	ENSP00000337340
ENSP00000260970	ENSP00000345793
ENSP00000260970	ENSP00000346791
ENSP00000260970	ENSP00000348551
ENSP00000260970	ENSP00000356579
ENSP00000260970	ENSP00000360329
ENSP00000260970	ENSP00000360464
ENSP00000260970	ENSP00000360532
ENSP00000260970	ENSP00000366843
ENSP00000260970	ENSP00000367208
ENSP00000260970	ENSP00000368976
ENSP00000260970	ENSP00000381003
ENSP00000260970	ENSP00000386621
ENSP00000261015	ENSP00000262030
ENSP00000261015	ENSP00000264926
ENSP00000261015	ENSP00000268661
ENSP00000261015	ENSP00000277541
ENSP00000261015	ENSP00000278856
ENSP00000261015	ENSP00000296802
ENSP00000261015	ENSP00000298746
ENSP00000261015	ENSP00000302886
ENSP00000261015	ENSP00000305702
ENSP00000261015	ENSP00000307889
ENSP00000261015	ENSP00000313851
ENSP00000261015	ENSP00000314348

ENSP00000261015	ENSP00000317992
ENSP00000261015	ENSP00000318604
ENSP00000261015	ENSP00000322396
ENSP00000261015	ENSP00000323858
ENSP00000261015	ENSP00000328690
ENSP00000261015	ENSP00000329466
ENSP00000261015	ENSP00000338573
ENSP00000261015	ENSP00000338862
ENSP00000261015	ENSP00000339095
ENSP00000261015	ENSP00000339145
ENSP00000261015	ENSP00000339795
ENSP00000261015	ENSP00000346725
ENSP00000261015	ENSP00000351644
ENSP00000261015	ENSP00000352021
ENSP00000261015	ENSP00000353246
ENSP00000261015	ENSP00000354040
ENSP00000261015	ENSP00000358400
ENSP00000261015	ENSP00000358563
ENSP00000261015	ENSP00000358812
ENSP00000261015	ENSP00000359345
ENSP00000261015	ENSP00000359688
ENSP00000261015	ENSP00000360412
ENSP00000261015	ENSP00000360828
ENSP00000261015	ENSP00000361076
ENSP00000261015	ENSP00000363559
ENSP00000261015	ENSP00000364320
ENSP00000261015	ENSP00000366519
ENSP00000261015	ENSP00000366557
ENSP00000261015	ENSP00000368612
ENSP00000261015	ENSP00000368887
ENSP00000261015	ENSP00000370258
ENSP00000261015	ENSP00000380982
ENSP00000261015	ENSP00000381220
ENSP00000261015	ENSP00000382392
ENSP00000261015	ENSP00000402338
ENSP00000261015	ENSP00000414321
ENSP00000261015	ENSP00000420714
ENSP00000261017	ENSP00000269221
ENSP00000261017	ENSP00000281172
ENSP00000261017	ENSP00000301765
ENSP00000261017	ENSP00000313829
ENSP00000261017	ENSP00000315465
ENSP00000261017	ENSP00000327077
ENSP00000261017	ENSP00000352425
ENSP00000261017	ENSP00000354251
ENSP00000261017	ENSP00000355296

ENSP00000261017	ENSP00000355809
ENSP00000261017	ENSP00000355922
ENSP00000261017	ENSP00000362085
ENSP00000261017	ENSP00000362424
ENSP00000261017	ENSP00000363095
ENSP00000261017	ENSP00000365401
ENSP00000261017	ENSP00000365891
ENSP00000261017	ENSP00000380921
ENSP00000261024	ENSP00000284981
ENSP00000261024	ENSP00000309710
ENSP00000261024	ENSP00000373363
ENSP00000261024	ENSP00000374455
ENSP00000261024	ENSP00000419952
ENSP00000261037	ENSP00000271643
ENSP00000261070	ENSP00000263228
ENSP00000261070	ENSP00000282050
ENSP00000261070	ENSP00000295767
ENSP00000261070	ENSP00000325136
ENSP00000261070	ENSP00000326052
ENSP00000261070	ENSP00000352219
ENSP00000261070	ENSP00000391723
ENSP00000261167	ENSP00000304903
ENSP00000261167	ENSP00000326767
ENSP00000261167	ENSP00000326879
ENSP00000261167	ENSP00000349640
ENSP00000261167	ENSP00000355050
ENSP00000261167	ENSP00000358059
ENSP00000261167	ENSP00000365747
ENSP00000261167	ENSP00000368801
ENSP00000261167	ENSP00000370719
ENSP00000261167	ENSP00000387170
ENSP00000261168	ENSP00000261880
ENSP00000261168	ENSP00000265963
ENSP00000261168	ENSP00000269468
ENSP00000261168	ENSP00000272645
ENSP00000261168	ENSP00000282032
ENSP00000261168	ENSP00000283875
ENSP00000261168	ENSP00000285398
ENSP00000261168	ENSP00000301788
ENSP00000261168	ENSP00000314949
ENSP00000261168	ENSP00000329357
ENSP00000261168	ENSP00000330343
ENSP00000261168	ENSP00000339826
ENSP00000261168	ENSP00000341551
ENSP00000261168	ENSP00000348168
ENSP00000261168	ENSP00000355596

ENSP00000261168	ENSP00000356634
ENSP00000261168	ENSP00000357965
ENSP00000261168	ENSP00000375809
ENSP00000261168	ENSP00000377969
ENSP00000261170	ENSP00000262460
ENSP00000261170	ENSP00000264917
ENSP00000261170	ENSP00000327107
ENSP00000261170	ENSP00000337127
ENSP00000261170	ENSP00000345096
ENSP00000261170	ENSP00000349493
ENSP00000261170	ENSP00000351015
ENSP00000261170	ENSP00000367208
ENSP00000261173	ENSP00000262052
ENSP00000261173	ENSP00000264335
ENSP00000261173	ENSP00000265351
ENSP00000261173	ENSP00000273398
ENSP00000261173	ENSP00000286448
ENSP00000261173	ENSP00000303507
ENSP00000261173	ENSP00000304500
ENSP00000261173	ENSP00000305647
ENSP00000261173	ENSP00000306106
ENSP00000261173	ENSP00000311200
ENSP00000261173	ENSP00000328968
ENSP00000261173	ENSP00000333873
ENSP00000261173	ENSP00000344393
ENSP00000261173	ENSP00000355968
ENSP00000261173	ENSP00000357998
ENSP00000261173	ENSP00000370607
ENSP00000261173	ENSP00000385083
ENSP00000261183	ENSP00000286448
ENSP00000261183	ENSP00000291688
ENSP00000261183	ENSP00000296786
ENSP00000261183	ENSP00000302239
ENSP00000261183	ENSP00000303554
ENSP00000261183	ENSP00000318262
ENSP00000261183	ENSP00000318723
ENSP00000261183	ENSP00000325506
ENSP00000261183	ENSP00000329466
ENSP00000261183	ENSP00000342793
ENSP00000261183	ENSP00000348577
ENSP00000261183	ENSP00000354519
ENSP00000261183	ENSP00000354558
ENSP00000261183	ENSP00000358140
ENSP00000261183	ENSP00000369860
ENSP00000261183	ENSP00000369862
ENSP00000261183	ENSP00000376268

ENSP00000261191	ENSP00000261531
ENSP00000261191	ENSP00000310260
ENSP00000261191	ENSP00000318641
ENSP00000261191	ENSP00000327889
ENSP00000261191	ENSP00000385722
ENSP00000261191	ENSP00000415151
ENSP00000261207	ENSP00000265073
ENSP00000261207	ENSP00000272167
ENSP00000261207	ENSP00000274031
ENSP00000261207	ENSP00000276326
ENSP00000261207	ENSP00000295951
ENSP00000261207	ENSP00000298910
ENSP00000261207	ENSP00000302935
ENSP00000261207	ENSP00000306245
ENSP00000261207	ENSP00000317790
ENSP00000261207	ENSP00000317985
ENSP00000261207	ENSP00000331310
ENSP00000261207	ENSP00000344666
ENSP00000261207	ENSP00000348010
ENSP00000261207	ENSP00000349049
ENSP00000261207	ENSP00000350512
ENSP00000261207	ENSP00000355296
ENSP00000261207	ENSP00000357907
ENSP00000261207	ENSP00000359206
ENSP00000261207	ENSP00000361824
ENSP00000261207	ENSP00000380942
ENSP00000261208	ENSP00000357907
ENSP00000261211	ENSP00000261596
ENSP00000261211	ENSP00000262077
ENSP00000261211	ENSP00000262173
ENSP00000261211	ENSP00000263121
ENSP00000261211	ENSP00000265717
ENSP00000261211	ENSP00000269214
ENSP00000261211	ENSP00000272322
ENSP00000261211	ENSP00000296328
ENSP00000261211	ENSP00000296509
ENSP00000261211	ENSP00000299543
ENSP00000261211	ENSP00000306999
ENSP00000261211	ENSP00000310649
ENSP00000261211	ENSP00000314949
ENSP00000261211	ENSP00000317904
ENSP00000261211	ENSP00000325863
ENSP00000261211	ENSP00000336606
ENSP00000261211	ENSP00000338703
ENSP00000261211	ENSP00000341382
ENSP00000261211	ENSP00000344220

ENSP00000261211	ENSP00000346566
ENSP00000261211	ENSP00000347444
ENSP00000261211	ENSP00000347978
ENSP00000261211	ENSP00000348554
ENSP00000261211	ENSP00000351284
ENSP00000261211	ENSP00000353564
ENSP00000261211	ENSP00000353698
ENSP00000261211	ENSP00000357048
ENSP00000261211	ENSP00000357748
ENSP00000261211	ENSP00000358140
ENSP00000261211	ENSP00000360310
ENSP00000261211	ENSP00000362334
ENSP00000261211	ENSP00000366121
ENSP00000261211	ENSP00000368305
ENSP00000261211	ENSP00000369335
ENSP00000261211	ENSP00000371475
ENSP00000261211	ENSP00000372857
ENSP00000261211	ENSP00000378350
ENSP00000261211	ENSP00000380129
ENSP00000261211	ENSP00000384313
ENSP00000261211	ENSP00000402527
ENSP00000261211	ENSP00000417963
ENSP00000261245	ENSP00000265963
ENSP00000261245	ENSP00000267163
ENSP00000261245	ENSP00000273480
ENSP00000261245	ENSP00000276072
ENSP00000261245	ENSP00000283875
ENSP00000261245	ENSP00000285398
ENSP00000261245	ENSP00000298552
ENSP00000261245	ENSP00000299238
ENSP00000261245	ENSP00000312697
ENSP00000261245	ENSP00000313881
ENSP00000261245	ENSP00000315950
ENSP00000261245	ENSP00000320646
ENSP00000261245	ENSP00000333633
ENSP00000261245	ENSP00000342087
ENSP00000261245	ENSP00000342755
ENSP00000261245	ENSP00000348168
ENSP00000261245	ENSP00000350283
ENSP00000261245	ENSP00000350708
ENSP00000261245	ENSP00000353332
ENSP00000261245	ENSP00000356811
ENSP00000261245	ENSP00000358778
ENSP00000261245	ENSP00000363384
ENSP00000261245	ENSP00000365504
ENSP00000261245	ENSP00000375809

ENSP00000261245	ENSP00000377617
ENSP00000261245	ENSP00000391685
ENSP00000261245	ENSP00000395497
ENSP00000261245	ENSP00000396251
ENSP00000261245	ENSP00000401105
ENSP00000261245	ENSP00000403156
ENSP00000261247	ENSP00000298852
ENSP00000261247	ENSP00000310129
ENSP00000261249	ENSP00000288828
ENSP00000261249	ENSP00000299138
ENSP00000261249	ENSP00000309433
ENSP00000261249	ENSP00000353741
ENSP00000261292	ENSP00000347206
ENSP00000261292	ENSP00000373952
ENSP00000261302	ENSP00000337088
ENSP00000261308	ENSP00000261531
ENSP00000261308	ENSP00000263201
ENSP00000261308	ENSP00000273783
ENSP00000261308	ENSP00000360532
ENSP00000261308	ENSP00000366557
ENSP00000261308	ENSP00000387006
ENSP00000261326	ENSP00000263774
ENSP00000261326	ENSP00000302896
ENSP00000261326	ENSP00000310623
ENSP00000261326	ENSP00000326981
ENSP00000261326	ENSP00000356972
ENSP00000261368	ENSP00000317817
ENSP00000261368	ENSP00000322457
ENSP00000261368	ENSP00000338345
ENSP00000261368	ENSP00000342667
ENSP00000261368	ENSP00000355865
ENSP00000261381	ENSP00000379383
ENSP00000261386	ENSP00000326699
ENSP00000261386	ENSP00000350012
ENSP00000261396	ENSP00000261881
ENSP00000261396	ENSP00000262077
ENSP00000261396	ENSP00000262315
ENSP00000261396	ENSP00000263208
ENSP00000261396	ENSP00000264718
ENSP00000261396	ENSP00000264883
ENSP00000261396	ENSP00000265351
ENSP00000261396	ENSP00000283195
ENSP00000261396	ENSP00000291688
ENSP00000261396	ENSP00000296257
ENSP00000261396	ENSP00000296509
ENSP00000261396	ENSP00000297185

ENSP00000261396	ENSP00000302790
ENSP00000261396	ENSP00000302886
ENSP00000261396	ENSP00000305503
ENSP00000261396	ENSP00000308622
ENSP00000261396	ENSP00000310668
ENSP00000261396	ENSP00000316032
ENSP00000261396	ENSP00000318977
ENSP00000261396	ENSP00000322180
ENSP00000261396	ENSP00000322242
ENSP00000261396	ENSP00000325863
ENSP00000261396	ENSP00000327197
ENSP00000261396	ENSP00000329466
ENSP00000261396	ENSP00000329933
ENSP00000261396	ENSP00000336606
ENSP00000261396	ENSP00000338413
ENSP00000261396	ENSP00000342262
ENSP00000261396	ENSP00000346453
ENSP00000261396	ENSP00000351284
ENSP00000261396	ENSP00000352021
ENSP00000261396	ENSP00000352400
ENSP00000261396	ENSP00000353793
ENSP00000261396	ENSP00000354522
ENSP00000261396	ENSP00000354673
ENSP00000261396	ENSP00000355922
ENSP00000261396	ENSP00000356470
ENSP00000261396	ENSP00000360286
ENSP00000261396	ENSP00000367721
ENSP00000261396	ENSP00000369335
ENSP00000261396	ENSP00000382562
ENSP00000261396	ENSP00000382779
ENSP00000261396	ENSP00000386759
ENSP00000261396	ENSP00000394791
ENSP00000261402	ENSP00000298552
ENSP00000261402	ENSP00000341551
ENSP00000261405	ENSP00000266556
ENSP00000261405	ENSP00000280527
ENSP00000261405	ENSP00000286091
ENSP00000261405	ENSP00000296550
ENSP00000261405	ENSP00000297350
ENSP00000261405	ENSP00000299767
ENSP00000261405	ENSP00000329380
ENSP00000261405	ENSP00000341637
ENSP00000261405	ENSP00000348307
ENSP00000261405	ENSP00000352455
ENSP00000261405	ENSP00000353393
ENSP00000261405	ENSP00000354927

ENSP00000261405	ENSP00000357484
ENSP00000261405	ENSP00000360997
ENSP00000261405	ENSP00000367408
ENSP00000261407	ENSP00000262134
ENSP00000261407	ENSP00000266095
ENSP00000261407	ENSP00000310551
ENSP00000261407	ENSP00000337463
ENSP00000261407	ENSP00000355963
ENSP00000261407	ENSP00000380184
ENSP00000261407	ENSP00000395863
ENSP00000261407	ENSP00000401287
ENSP00000261413	ENSP00000264230
ENSP00000261413	ENSP00000301585
ENSP00000261413	ENSP00000379607
ENSP00000261416	ENSP00000271764
ENSP00000261416	ENSP00000362314
ENSP00000261424	ENSP00000261881
ENSP00000261424	ENSP00000261951
ENSP00000261424	ENSP00000262315
ENSP00000261424	ENSP00000262518
ENSP00000261424	ENSP00000263201
ENSP00000261424	ENSP00000263202
ENSP00000261424	ENSP00000263274
ENSP00000261424	ENSP00000263377
ENSP00000261424	ENSP00000263795
ENSP00000261424	ENSP00000264926
ENSP00000261424	ENSP00000265140
ENSP00000261424	ENSP00000265433
ENSP00000261424	ENSP00000265465
ENSP00000261424	ENSP00000265849
ENSP00000261424	ENSP00000266182
ENSP00000261424	ENSP00000267163
ENSP00000261424	ENSP00000267430
ENSP00000261424	ENSP00000268043
ENSP00000261424	ENSP00000269214
ENSP00000261424	ENSP00000278412
ENSP00000261424	ENSP00000278616
ENSP00000261424	ENSP00000280614
ENSP00000261424	ENSP00000282326
ENSP00000261424	ENSP00000282516
ENSP00000261424	ENSP00000285021
ENSP00000261424	ENSP00000287598
ENSP00000261424	ENSP00000288774
ENSP00000261424	ENSP00000296273
ENSP00000261424	ENSP00000296509
ENSP00000261424	ENSP00000297338

ENSP00000261424	ENSP00000297431
ENSP00000261424	ENSP00000298832
ENSP00000261424	ENSP00000300249
ENSP00000261424	ENSP00000300738
ENSP00000261424	ENSP00000302530
ENSP00000261424	ENSP00000304903
ENSP00000261424	ENSP00000306999
ENSP00000261424	ENSP00000307853
ENSP00000261424	ENSP00000308699
ENSP00000261424	ENSP00000308759
ENSP00000261424	ENSP00000308944
ENSP00000261424	ENSP00000309142
ENSP00000261424	ENSP00000309572
ENSP00000261424	ENSP00000310520
ENSP00000261424	ENSP00000311873
ENSP00000261424	ENSP00000313171
ENSP00000261424	ENSP00000313851
ENSP00000261424	ENSP00000318977
ENSP00000261424	ENSP00000321636
ENSP00000261424	ENSP00000322180
ENSP00000261424	ENSP00000322242
ENSP00000261424	ENSP00000322570
ENSP00000261424	ENSP00000323439
ENSP00000261424	ENSP00000325677
ENSP00000261424	ENSP00000325863
ENSP00000261424	ENSP00000329466
ENSP00000261424	ENSP00000336606
ENSP00000261424	ENSP00000340879
ENSP00000261424	ENSP00000343392
ENSP00000261424	ENSP00000343741
ENSP00000261424	ENSP00000343745
ENSP00000261424	ENSP00000347168
ENSP00000261424	ENSP00000347232
ENSP00000261424	ENSP00000347719
ENSP00000261424	ENSP00000347978
ENSP00000261424	ENSP00000349393
ENSP00000261424	ENSP00000350011
ENSP00000261424	ENSP00000350283
ENSP00000261424	ENSP00000350491
ENSP00000261424	ENSP00000350767
ENSP00000261424	ENSP00000351284
ENSP00000261424	ENSP00000351697
ENSP00000261424	ENSP00000352257
ENSP00000261424	ENSP00000352738
ENSP00000261424	ENSP00000353332
ENSP00000261424	ENSP00000353793

ENSP00000261424	ENSP00000354522
ENSP00000261424	ENSP00000354720
ENSP00000261424	ENSP00000355759
ENSP00000261424	ENSP00000357602
ENSP00000261424	ENSP00000360310
ENSP00000261424	ENSP00000360371
ENSP00000261424	ENSP00000360621
ENSP00000261424	ENSP00000361707
ENSP00000261424	ENSP00000362352
ENSP00000261424	ENSP00000363021
ENSP00000261424	ENSP00000364270
ENSP00000261424	ENSP00000366974
ENSP00000261424	ENSP00000367034
ENSP00000261424	ENSP00000367787
ENSP00000261424	ENSP00000368349
ENSP00000261424	ENSP00000368438
ENSP00000261424	ENSP00000368591
ENSP00000261424	ENSP00000369335
ENSP00000261424	ENSP00000369411
ENSP00000261424	ENSP00000370150
ENSP00000261424	ENSP00000370151
ENSP00000261424	ENSP00000371419
ENSP00000261424	ENSP00000371475
ENSP00000261424	ENSP00000372023
ENSP00000261424	ENSP00000374213
ENSP00000261424	ENSP00000375809
ENSP00000261424	ENSP00000380504
ENSP00000261424	ENSP00000382562
ENSP00000261424	ENSP00000396704
ENSP00000261424	ENSP00000406490
ENSP00000261424	ENSP00000420588
ENSP00000261434	ENSP00000268379
ENSP00000261434	ENSP00000280701
ENSP00000261434	ENSP00000282999
ENSP00000261434	ENSP00000304592
ENSP00000261434	ENSP00000315693
ENSP00000261434	ENSP00000348965
ENSP00000261434	ENSP00000360782
ENSP00000261434	ENSP00000363308
ENSP00000261435	ENSP00000261965
ENSP00000261435	ENSP00000262367
ENSP00000261435	ENSP00000264731
ENSP00000261435	ENSP00000314733
ENSP00000261435	ENSP00000345530
ENSP00000261454	ENSP00000262367
ENSP00000261454	ENSP00000288422

ENSP00000261454	ENSP00000311825
ENSP00000261454	ENSP00000322457
ENSP00000261454	ENSP00000342828
ENSP00000261454	ENSP00000353344
ENSP00000261454	ENSP00000356331
ENSP00000261454	ENSP00000356595
ENSP00000261454	ENSP00000378286
ENSP00000261454	ENSP00000378733
ENSP00000261475	ENSP00000296755
ENSP00000261475	ENSP00000300231
ENSP00000261475	ENSP00000311344
ENSP00000261488	ENSP00000263925
ENSP00000261488	ENSP00000338157
ENSP00000261488	ENSP00000370410
ENSP00000261488	ENSP00000386394
ENSP00000261499	ENSP00000265993
ENSP00000261499	ENSP00000304941
ENSP00000261499	ENSP00000335094
ENSP00000261499	ENSP00000337396
ENSP00000261499	ENSP00000356774
ENSP00000261499	ENSP00000366135
ENSP00000261499	ENSP00000376827
ENSP00000261507	ENSP00000264027
ENSP00000261507	ENSP00000264451
ENSP00000261507	ENSP00000265896
ENSP00000261507	ENSP00000272163
ENSP00000261507	ENSP00000277010
ENSP00000261507	ENSP00000285949
ENSP00000261507	ENSP00000287936
ENSP00000261507	ENSP00000310721
ENSP00000261507	ENSP00000325425
ENSP00000261507	ENSP00000330812
ENSP00000261507	ENSP00000333946
ENSP00000261507	ENSP00000336712
ENSP00000261507	ENSP00000341662
ENSP00000261507	ENSP00000344741
ENSP00000261507	ENSP00000348762
ENSP00000261507	ENSP00000355517
ENSP00000261507	ENSP00000356591
ENSP00000261507	ENSP00000357775
ENSP00000261507	ENSP00000359380
ENSP00000261507	ENSP00000361845
ENSP00000261507	ENSP00000371236
ENSP00000261507	ENSP00000379430
ENSP00000261507	ENSP00000386284
ENSP00000261509	ENSP00000284776

ENSP00000261509	ENSP00000294129
ENSP00000261514	ENSP00000274376
ENSP00000261514	ENSP00000304500
ENSP00000261514	ENSP00000311842
ENSP00000261514	ENSP00000349887
ENSP00000261514	ENSP00000357484
ENSP00000261517	ENSP00000262315
ENSP00000261517	ENSP00000273398
ENSP00000261517	ENSP00000299138
ENSP00000261517	ENSP00000311200
ENSP00000261517	ENSP00000342793
ENSP00000261517	ENSP00000349131
ENSP00000261517	ENSP00000365233
ENSP00000261517	ENSP00000365943
ENSP00000261520	ENSP00000263390
ENSP00000261520	ENSP00000296564
ENSP00000261520	ENSP00000380054
ENSP00000261523	ENSP00000266744
ENSP00000261523	ENSP00000285873
ENSP00000261523	ENSP00000300651
ENSP00000261523	ENSP00000305422
ENSP00000261523	ENSP00000314420
ENSP00000261523	ENSP00000321345
ENSP00000261523	ENSP00000348877
ENSP00000261523	ENSP00000358778
ENSP00000261523	ENSP00000364847
ENSP00000261523	ENSP00000368253
ENSP00000261523	ENSP00000370826
ENSP00000261523	ENSP00000377520
ENSP00000261523	ENSP00000399968
ENSP00000261531	ENSP00000261597
ENSP00000261531	ENSP00000261600
ENSP00000261531	ENSP00000261712
ENSP00000261531	ENSP00000261965
ENSP00000261531	ENSP00000261973
ENSP00000261531	ENSP00000262077
ENSP00000261531	ENSP00000262133
ENSP00000261531	ENSP00000262238
ENSP00000261531	ENSP00000262544
ENSP00000261531	ENSP00000262584
ENSP00000261531	ENSP00000262710
ENSP00000261531	ENSP00000262803
ENSP00000261531	ENSP00000262962
ENSP00000261531	ENSP00000262982
ENSP00000261531	ENSP00000263121
ENSP00000261531	ENSP00000263208

ENSP00000261531	ENSP00000263360
ENSP00000261531	ENSP00000263381
ENSP00000261531	ENSP00000263956
ENSP00000261531	ENSP00000263974
ENSP00000261531	ENSP00000264028
ENSP00000261531	ENSP00000264065
ENSP00000261531	ENSP00000264447
ENSP00000261531	ENSP00000264515
ENSP00000261531	ENSP00000264639
ENSP00000261531	ENSP00000264720
ENSP00000261531	ENSP00000264926
ENSP00000261531	ENSP00000265433
ENSP00000261531	ENSP00000265748
ENSP00000261531	ENSP00000265753
ENSP00000261531	ENSP00000265990
ENSP00000261531	ENSP00000266000
ENSP00000261531	ENSP00000266070
ENSP00000261531	ENSP00000266079
ENSP00000261531	ENSP00000266732
ENSP00000261531	ENSP00000267064
ENSP00000261531	ENSP00000267163
ENSP00000261531	ENSP00000267812
ENSP00000261531	ENSP00000268182
ENSP00000261531	ENSP00000268482
ENSP00000261531	ENSP00000268717
ENSP00000261531	ENSP00000271452
ENSP00000261531	ENSP00000271628
ENSP00000261531	ENSP00000271715
ENSP00000261531	ENSP00000276570
ENSP00000261531	ENSP00000277541
ENSP00000261531	ENSP00000278572
ENSP00000261531	ENSP00000278616
ENSP00000261531	ENSP00000281708
ENSP00000261531	ENSP00000282326
ENSP00000261531	ENSP00000282516
ENSP00000261531	ENSP00000282999
ENSP00000261531	ENSP00000283195
ENSP00000261531	ENSP00000283943
ENSP00000261531	ENSP00000284719
ENSP00000261531	ENSP00000285873
ENSP00000261531	ENSP00000286070
ENSP00000261531	ENSP00000287598
ENSP00000261531	ENSP00000289371
ENSP00000261531	ENSP00000290158
ENSP00000261531	ENSP00000292211
ENSP00000261531	ENSP00000292476

ENSP00000261531	ENSP00000292616
ENSP00000261531	ENSP00000295373
ENSP00000261531	ENSP00000296015
ENSP00000261531	ENSP00000296043
ENSP00000261531	ENSP00000296215
ENSP00000261531	ENSP00000296273
ENSP00000261531	ENSP00000296702
ENSP00000261531	ENSP00000297151
ENSP00000261531	ENSP00000297338
ENSP00000261531	ENSP00000297814
ENSP00000261531	ENSP00000298281
ENSP00000261531	ENSP00000298746
ENSP00000261531	ENSP00000298767
ENSP00000261531	ENSP00000298852
ENSP00000261531	ENSP00000299767
ENSP00000261531	ENSP00000300648
ENSP00000261531	ENSP00000301740
ENSP00000261531	ENSP00000301764
ENSP00000261531	ENSP00000301843
ENSP00000261531	ENSP00000302530
ENSP00000261531	ENSP00000302790
ENSP00000261531	ENSP00000303191
ENSP00000261531	ENSP00000304102
ENSP00000261531	ENSP00000304350
ENSP00000261531	ENSP00000304370
ENSP00000261531	ENSP00000304592
ENSP00000261531	ENSP00000304704
ENSP00000261531	ENSP00000305790
ENSP00000261531	ENSP00000306983
ENSP00000261531	ENSP00000307387
ENSP00000261531	ENSP00000307863
ENSP00000261531	ENSP00000307889
ENSP00000261531	ENSP00000308351
ENSP00000261531	ENSP00000308944
ENSP00000261531	ENSP00000309166
ENSP00000261531	ENSP00000309262
ENSP00000261531	ENSP00000309474
ENSP00000261531	ENSP00000309831
ENSP00000261531	ENSP00000309871
ENSP00000261531	ENSP00000310040
ENSP00000261531	ENSP00000310129
ENSP00000261531	ENSP00000310448
ENSP00000261531	ENSP00000310723
ENSP00000261531	ENSP00000310842
ENSP00000261531	ENSP00000311028
ENSP00000261531	ENSP00000311135

ENSP00000261531	ENSP00000311221
ENSP00000261531	ENSP00000311344
ENSP00000261531	ENSP00000311430
ENSP00000261531	ENSP00000311747
ENSP00000261531	ENSP00000312042
ENSP00000261531	ENSP00000312066
ENSP00000261531	ENSP00000312697
ENSP00000261531	ENSP00000312981
ENSP00000261531	ENSP00000313420
ENSP00000261531	ENSP00000313829
ENSP00000261531	ENSP00000314949
ENSP00000261531	ENSP00000315791
ENSP00000261531	ENSP00000316240
ENSP00000261531	ENSP00000317334
ENSP00000261531	ENSP00000317985
ENSP00000261531	ENSP00000318195
ENSP00000261531	ENSP00000318641
ENSP00000261531	ENSP00000318861
ENSP00000261531	ENSP00000320485
ENSP00000261531	ENSP00000320949
ENSP00000261531	ENSP00000321706
ENSP00000261531	ENSP00000322016
ENSP00000261531	ENSP00000322570
ENSP00000261531	ENSP00000323377
ENSP00000261531	ENSP00000323856
ENSP00000261531	ENSP00000323967
ENSP00000261531	ENSP00000325002
ENSP00000261531	ENSP00000325017
ENSP00000261531	ENSP00000325120
ENSP00000261531	ENSP00000325376
ENSP00000261531	ENSP00000325377
ENSP00000261531	ENSP00000325863
ENSP00000261531	ENSP00000326261
ENSP00000261531	ENSP00000326514
ENSP00000261531	ENSP00000326603
ENSP00000261531	ENSP00000326806
ENSP00000261531	ENSP00000327650
ENSP00000261531	ENSP00000328992
ENSP00000261531	ENSP00000329419
ENSP00000261531	ENSP00000329466
ENSP00000261531	ENSP00000329715
ENSP00000261531	ENSP00000330343
ENSP00000261531	ENSP00000331310
ENSP00000261531	ENSP00000331678
ENSP00000261531	ENSP00000331907
ENSP00000261531	ENSP00000332103

ENSP00000261531	ENSP00000332681
ENSP00000261531	ENSP00000334100
ENSP00000261531	ENSP00000334148
ENSP00000261531	ENSP00000335321
ENSP00000261531	ENSP00000336712
ENSP00000261531	ENSP00000336752
ENSP00000261531	ENSP00000337088
ENSP00000261531	ENSP00000338862
ENSP00000261531	ENSP00000339109
ENSP00000261531	ENSP00000339353
ENSP00000261531	ENSP00000339495
ENSP00000261531	ENSP00000339587
ENSP00000261531	ENSP00000339723
ENSP00000261531	ENSP00000340019
ENSP00000261531	ENSP00000340281
ENSP00000261531	ENSP00000340796
ENSP00000261531	ENSP00000341382
ENSP00000261531	ENSP00000341551
ENSP00000261531	ENSP00000342181
ENSP00000261531	ENSP00000342300
ENSP00000261531	ENSP00000342905
ENSP00000261531	ENSP00000343557
ENSP00000261531	ENSP00000343701
ENSP00000261531	ENSP00000343741
ENSP00000261531	ENSP00000346088
ENSP00000261531	ENSP00000346120
ENSP00000261531	ENSP00000346236
ENSP00000261531	ENSP00000346566
ENSP00000261531	ENSP00000346634
ENSP00000261531	ENSP00000346762
ENSP00000261531	ENSP00000347232
ENSP00000261531	ENSP00000347379
ENSP00000261531	ENSP00000348442
ENSP00000261531	ENSP00000348510
ENSP00000261531	ENSP00000348551
ENSP00000261531	ENSP00000348554
ENSP00000261531	ENSP00000348565
ENSP00000261531	ENSP00000348577
ENSP00000261531	ENSP00000348708
ENSP00000261531	ENSP00000348965
ENSP00000261531	ENSP00000349049
ENSP00000261531	ENSP00000349205
ENSP00000261531	ENSP00000349213
ENSP00000261531	ENSP00000349748
ENSP00000261531	ENSP00000349977
ENSP00000261531	ENSP00000350199

ENSP00000261531	ENSP00000350512
ENSP00000261531	ENSP00000351137
ENSP00000261531	ENSP00000351552
ENSP00000261531	ENSP00000351947
ENSP00000261531	ENSP00000352047
ENSP00000261531	ENSP00000352257
ENSP00000261531	ENSP00000352308
ENSP00000261531	ENSP00000352408
ENSP00000261531	ENSP00000352678
ENSP00000261531	ENSP00000352738
ENSP00000261531	ENSP00000353458
ENSP00000261531	ENSP00000353622
ENSP00000261531	ENSP00000353741
ENSP00000261531	ENSP00000354040
ENSP00000261531	ENSP00000354061
ENSP00000261531	ENSP00000354346
ENSP00000261531	ENSP00000354518
ENSP00000261531	ENSP00000354720
ENSP00000261531	ENSP00000354739
ENSP00000261531	ENSP00000354742
ENSP00000261531	ENSP00000354791
ENSP00000261531	ENSP00000354848
ENSP00000261531	ENSP00000354851
ENSP00000261531	ENSP00000354920
ENSP00000261531	ENSP00000355011
ENSP00000261531	ENSP00000355050
ENSP00000261531	ENSP00000355809
ENSP00000261531	ENSP00000356405
ENSP00000261531	ENSP00000356520
ENSP00000261531	ENSP00000356811
ENSP00000261531	ENSP00000357048
ENSP00000261531	ENSP00000357283
ENSP00000261531	ENSP00000357631
ENSP00000261531	ENSP00000358400
ENSP00000261531	ENSP00000358417
ENSP00000261531	ENSP00000358554
ENSP00000261531	ENSP00000358799
ENSP00000261531	ENSP00000359552
ENSP00000261531	ENSP00000359988
ENSP00000261531	ENSP00000360183
ENSP00000261531	ENSP00000360286
ENSP00000261531	ENSP00000360532
ENSP00000261531	ENSP00000360676
ENSP00000261531	ENSP00000360916
ENSP00000261531	ENSP00000361076
ENSP00000261531	ENSP00000361180

ENSP00000261531	ENSP00000361219
ENSP00000261531	ENSP00000361465
ENSP00000261531	ENSP00000361577
ENSP00000261531	ENSP00000361824
ENSP00000261531	ENSP00000362041
ENSP00000261531	ENSP00000362110
ENSP00000261531	ENSP00000362441
ENSP00000261531	ENSP00000362688
ENSP00000261531	ENSP00000362820
ENSP00000261531	ENSP00000363313
ENSP00000261531	ENSP00000363745
ENSP00000261531	ENSP00000364119
ENSP00000261531	ENSP00000364289
ENSP00000261531	ENSP00000365439
ENSP00000261531	ENSP00000365735
ENSP00000261531	ENSP00000366135
ENSP00000261531	ENSP00000366179
ENSP00000261531	ENSP00000366557
ENSP00000261531	ENSP00000366829
ENSP00000261531	ENSP00000367208
ENSP00000261531	ENSP00000367263
ENSP00000261531	ENSP00000367498
ENSP00000261531	ENSP00000367797
ENSP00000261531	ENSP00000367835
ENSP00000261531	ENSP00000368523
ENSP00000261531	ENSP00000369100
ENSP00000261531	ENSP00000369162
ENSP00000261531	ENSP00000369218
ENSP00000261531	ENSP00000369411
ENSP00000261531	ENSP00000369716
ENSP00000261531	ENSP00000370119
ENSP00000261531	ENSP00000370517
ENSP00000261531	ENSP00000371419
ENSP00000261531	ENSP00000371451
ENSP00000261531	ENSP00000372689
ENSP00000261531	ENSP00000374455
ENSP00000261531	ENSP00000376309
ENSP00000261531	ENSP00000376973
ENSP00000261531	ENSP00000378350
ENSP00000261531	ENSP00000379607
ENSP00000261531	ENSP00000380336
ENSP00000261531	ENSP00000381293
ENSP00000261531	ENSP00000381565
ENSP00000261531	ENSP00000382281
ENSP00000261531	ENSP00000382562
ENSP00000261531	ENSP00000382779

ENSP00000261531	ENSP00000384048
ENSP00000261531	ENSP00000385722
ENSP00000261531	ENSP00000386717
ENSP00000261531	ENSP00000387006
ENSP00000261531	ENSP00000388464
ENSP00000261531	ENSP00000389103
ENSP00000261531	ENSP00000389934
ENSP00000261531	ENSP00000391249
ENSP00000261531	ENSP00000392028
ENSP00000261531	ENSP00000392270
ENSP00000261531	ENSP00000392601
ENSP00000261531	ENSP00000394316
ENSP00000261531	ENSP00000396301
ENSP00000261531	ENSP00000396439
ENSP00000261531	ENSP00000396876
ENSP00000261531	ENSP00000396937
ENSP00000261531	ENSP00000398597
ENSP00000261531	ENSP00000399968
ENSP00000261531	ENSP00000404121
ENSP00000261531	ENSP00000404623
ENSP00000261531	ENSP00000407487
ENSP00000261531	ENSP00000408910
ENSP00000261531	ENSP00000410772
ENSP00000261531	ENSP00000412553
ENSP00000261531	ENSP00000412566
ENSP00000261531	ENSP00000416110
ENSP00000261531	ENSP00000416892
ENSP00000261531	ENSP00000416959
ENSP00000261534	ENSP00000278243
ENSP00000261534	ENSP00000286091
ENSP00000261534	ENSP00000299626
ENSP00000261534	ENSP00000300658
ENSP00000261534	ENSP00000314615
ENSP00000261534	ENSP00000346809
ENSP00000261534	ENSP00000349877
ENSP00000261534	ENSP00000355963
ENSP00000261534	ENSP00000359305
ENSP00000261534	ENSP00000360149
ENSP00000261534	ENSP00000360473
ENSP00000261534	ENSP00000360644
ENSP00000261534	ENSP00000364188
ENSP00000261534	ENSP00000366395
ENSP00000261534	ENSP00000415203
ENSP00000261534	ENSP00000417748
ENSP00000261558	ENSP00000269122
ENSP00000261558	ENSP00000294543

ENSP00000261558	ENSP00000325369
ENSP00000261558	ENSP00000347408
ENSP00000261558	ENSP00000351926
ENSP00000261558	ENSP00000356146
ENSP00000261558	ENSP00000360798
ENSP00000261558	ENSP00000365891
ENSP00000261584	ENSP00000286800
ENSP00000261584	ENSP00000295897
ENSP00000261584	ENSP00000323050
ENSP00000261584	ENSP00000326819
ENSP00000261584	ENSP00000331310
ENSP00000261584	ENSP00000350283
ENSP00000261584	ENSP00000369497
ENSP00000261590	ENSP00000294785
ENSP00000261590	ENSP00000296084
ENSP00000261590	ENSP00000326366
ENSP00000261590	ENSP00000331678
ENSP00000261592	ENSP00000310440
ENSP00000261592	ENSP00000311825
ENSP00000261592	ENSP00000319597
ENSP00000261592	ENSP00000379607
ENSP00000261593	ENSP00000294008
ENSP00000261593	ENSP00000302120
ENSP00000261593	ENSP00000354951
ENSP00000261596	ENSP00000264005
ENSP00000261596	ENSP00000266095
ENSP00000261596	ENSP00000269143
ENSP00000261596	ENSP00000285518
ENSP00000261596	ENSP00000290231
ENSP00000261596	ENSP00000296786
ENSP00000261596	ENSP00000310551
ENSP00000261596	ENSP00000314036
ENSP00000261596	ENSP00000316032
ENSP00000261596	ENSP00000318115
ENSP00000261596	ENSP00000321711
ENSP00000261596	ENSP00000332258
ENSP00000261596	ENSP00000337463
ENSP00000261596	ENSP00000348577
ENSP00000261596	ENSP00000356591
ENSP00000261596	ENSP00000361667
ENSP00000261596	ENSP00000369862
ENSP00000261596	ENSP00000371155
ENSP00000261596	ENSP00000380184
ENSP00000261596	ENSP00000395863
ENSP00000261596	ENSP00000401287
ENSP00000261596	ENSP00000413405

ENSP00000261597	ENSP00000262056
ENSP00000261597	ENSP00000262059
ENSP00000261597	ENSP00000263202
ENSP00000261597	ENSP00000263710
ENSP00000261597	ENSP00000265056
ENSP00000261597	ENSP00000265148
ENSP00000261597	ENSP00000265465
ENSP00000261597	ENSP00000267163
ENSP00000261597	ENSP00000270861
ENSP00000261597	ENSP00000271452
ENSP00000261597	ENSP00000273398
ENSP00000261597	ENSP00000274289
ENSP00000261597	ENSP00000282074
ENSP00000261597	ENSP00000283006
ENSP00000261597	ENSP00000285116
ENSP00000261597	ENSP00000295030
ENSP00000261597	ENSP00000295685
ENSP00000261597	ENSP00000296509
ENSP00000261597	ENSP00000302530
ENSP00000261597	ENSP00000305459
ENSP00000261597	ENSP00000312870
ENSP00000261597	ENSP00000336868
ENSP00000261597	ENSP00000339503
ENSP00000261597	ENSP00000345793
ENSP00000261597	ENSP00000346566
ENSP00000261597	ENSP00000351665
ENSP00000261597	ENSP00000354791
ENSP00000261597	ENSP00000355944
ENSP00000261597	ENSP00000360329
ENSP00000261597	ENSP00000360532
ENSP00000261597	ENSP00000362110
ENSP00000261597	ENSP00000363055
ENSP00000261597	ENSP00000367034
ENSP00000261597	ENSP00000369075
ENSP00000261597	ENSP00000370408
ENSP00000261597	ENSP00000370557
ENSP00000261597	ENSP00000382562
ENSP00000261597	ENSP00000397441
ENSP00000261597	ENSP00000413632
ENSP00000261597	ENSP00000419628
ENSP00000261600	ENSP00000267163
ENSP00000261600	ENSP00000295899
ENSP00000261600	ENSP00000299608
ENSP00000261600	ENSP00000318861
ENSP00000261600	ENSP00000326531
ENSP00000261600	ENSP00000341805

ENSP00000261600	ENSP00000347592
ENSP00000261600	ENSP00000360488
ENSP00000261600	ENSP00000380969
ENSP00000261600	ENSP00000396301
ENSP00000261601	ENSP00000261609
ENSP00000261601	ENSP00000261712
ENSP00000261601	ENSP00000262193
ENSP00000261601	ENSP00000262315
ENSP00000261601	ENSP00000262518
ENSP00000261601	ENSP00000262854
ENSP00000261601	ENSP00000263208
ENSP00000261601	ENSP00000264515
ENSP00000261601	ENSP00000264552
ENSP00000261601	ENSP00000264639
ENSP00000261601	ENSP00000265073
ENSP00000261601	ENSP00000265351
ENSP00000261601	ENSP00000265990
ENSP00000261601	ENSP00000268616
ENSP00000261601	ENSP00000272645
ENSP00000261601	ENSP00000273064
ENSP00000261601	ENSP00000283351
ENSP00000261601	ENSP00000283943
ENSP00000261601	ENSP00000288774
ENSP00000261601	ENSP00000289081
ENSP00000261601	ENSP00000290810
ENSP00000261601	ENSP00000295066
ENSP00000261601	ENSP00000295901
ENSP00000261601	ENSP00000296273
ENSP00000261601	ENSP00000296792
ENSP00000261601	ENSP00000298852
ENSP00000261601	ENSP00000300648
ENSP00000261601	ENSP00000301962
ENSP00000261601	ENSP00000302239
ENSP00000261601	ENSP00000302530
ENSP00000261601	ENSP00000304593
ENSP00000261601	ENSP00000309198
ENSP00000261601	ENSP00000309474
ENSP00000261601	ENSP00000310129
ENSP00000261601	ENSP00000310590
ENSP00000261601	ENSP00000311665
ENSP00000261601	ENSP00000314311
ENSP00000261601	ENSP00000320566
ENSP00000261601	ENSP00000321711
ENSP00000261601	ENSP00000322242
ENSP00000261601	ENSP00000323687
ENSP00000261601	ENSP00000325677

ENSP00000261601	ENSP00000331310
ENSP00000261601	ENSP00000333376
ENSP00000261601	ENSP00000334808
ENSP00000261601	ENSP00000337405
ENSP00000261601	ENSP00000337839
ENSP00000261601	ENSP00000340596
ENSP00000261601	ENSP00000343535
ENSP00000261601	ENSP00000345530
ENSP00000261601	ENSP00000347836
ENSP00000261601	ENSP00000348442
ENSP00000261601	ENSP00000350009
ENSP00000261601	ENSP00000350512
ENSP00000261601	ENSP00000350708
ENSP00000261601	ENSP00000350967
ENSP00000261601	ENSP00000351596
ENSP00000261601	ENSP00000351947
ENSP00000261601	ENSP00000353622
ENSP00000261601	ENSP00000353793
ENSP00000261601	ENSP00000354558
ENSP00000261601	ENSP00000354673
ENSP00000261601	ENSP00000355205
ENSP00000261601	ENSP00000356405
ENSP00000261601	ENSP00000356989
ENSP00000261601	ENSP00000358400
ENSP00000261601	ENSP00000360116
ENSP00000261601	ENSP00000362334
ENSP00000261601	ENSP00000363779
ENSP00000261601	ENSP00000364929
ENSP00000261601	ENSP00000366135
ENSP00000261601	ENSP00000374213
ENSP00000261601	ENSP00000374280
ENSP00000261601	ENSP00000380460
ENSP00000261601	ENSP00000381045
ENSP00000261601	ENSP00000381863
ENSP00000261601	ENSP00000381891
ENSP00000261601	ENSP00000382269
ENSP00000261601	ENSP00000382779
ENSP00000261601	ENSP00000384211
ENSP00000261601	ENSP00000396937
ENSP00000261601	ENSP00000401445
ENSP00000261609	ENSP00000262850
ENSP00000261609	ENSP00000301962
ENSP00000261609	ENSP00000312995
ENSP00000261609	ENSP00000320898
ENSP00000261609	ENSP00000323099
ENSP00000261609	ENSP00000329466

ENSP00000261609	ENSP00000334808
ENSP00000261609	ENSP00000348577
ENSP00000261609	ENSP00000350283
ENSP00000261609	ENSP00000352676
ENSP00000261609	ENSP00000355596
ENSP00000261609	ENSP00000362578
ENSP00000261609	ENSP00000364270
ENSP00000261609	ENSP00000373060
ENSP00000261609	ENSP00000381045
ENSP00000261609	ENSP00000381863
ENSP00000261609	ENSP00000406490
ENSP00000261623	ENSP00000321239
ENSP00000261623	ENSP00000356505
ENSP00000261623	ENSP00000380450
ENSP00000261637	ENSP00000261708
ENSP00000261637	ENSP00000263187
ENSP00000261637	ENSP00000263657
ENSP00000261637	ENSP00000263795
ENSP00000261637	ENSP00000264995
ENSP00000261637	ENSP00000270625
ENSP00000261637	ENSP00000272418
ENSP00000261637	ENSP00000272521
ENSP00000261637	ENSP00000291576
ENSP00000261637	ENSP00000296792
ENSP00000261637	ENSP00000296802
ENSP00000261637	ENSP00000297579
ENSP00000261637	ENSP00000297990
ENSP00000261637	ENSP00000300151
ENSP00000261637	ENSP00000302896
ENSP00000261637	ENSP00000308179
ENSP00000261637	ENSP00000310596
ENSP00000261637	ENSP00000311135
ENSP00000261637	ENSP00000314193
ENSP00000261637	ENSP00000315674
ENSP00000261637	ENSP00000318646
ENSP00000261637	ENSP00000324122
ENSP00000261637	ENSP00000326981
ENSP00000261637	ENSP00000327179
ENSP00000261637	ENSP00000328854
ENSP00000261637	ENSP00000329466
ENSP00000261637	ENSP00000331907
ENSP00000261637	ENSP00000339095
ENSP00000261637	ENSP00000341885
ENSP00000261637	ENSP00000346015
ENSP00000261637	ENSP00000349274
ENSP00000261637	ENSP00000353741

ENSP00000261637	ENSP00000355471
ENSP00000261637	ENSP00000355541
ENSP00000261637	ENSP00000356134
ENSP00000261637	ENSP00000356520
ENSP00000261637	ENSP00000358812
ENSP00000261637	ENSP00000359688
ENSP00000261637	ENSP00000360031
ENSP00000261637	ENSP00000363642
ENSP00000261637	ENSP00000363746
ENSP00000261637	ENSP00000366557
ENSP00000261637	ENSP00000366974
ENSP00000261637	ENSP00000368984
ENSP00000261637	ENSP00000370589
ENSP00000261637	ENSP00000371101
ENSP00000261637	ENSP00000371169
ENSP00000261637	ENSP00000372695
ENSP00000261637	ENSP00000379888
ENSP00000261637	ENSP00000384302
ENSP00000261637	ENSP00000399454
ENSP00000261637	ENSP00000402869
ENSP00000261637	ENSP00000405614
ENSP00000261637	ENSP00000410396
ENSP00000261637	ENSP00000414006
ENSP00000261643	ENSP00000293195
ENSP00000261643	ENSP00000299335
ENSP00000261643	ENSP00000354499
ENSP00000261643	ENSP00000361042
ENSP00000261647	ENSP00000276201
ENSP00000261647	ENSP00000299084
ENSP00000261647	ENSP00000299238
ENSP00000261647	ENSP00000338413
ENSP00000261652	ENSP00000297156
ENSP00000261652	ENSP00000355958
ENSP00000261652	ENSP00000358997
ENSP00000261652	ENSP00000365048
ENSP00000261658	ENSP00000286186
ENSP00000261658	ENSP00000329623
ENSP00000261658	ENSP00000360116
ENSP00000261658	ENSP00000389277
ENSP00000261660	ENSP00000272322
ENSP00000261660	ENSP00000273398
ENSP00000261660	ENSP00000314499
ENSP00000261660	ENSP00000350249
ENSP00000261674	ENSP00000304704
ENSP00000261674	ENSP00000315859
ENSP00000261674	ENSP00000326261

ENSP00000261674	ENSP00000339723
ENSP00000261674	ENSP00000358242
ENSP00000261674	ENSP00000394085
ENSP00000261693	ENSP00000314508
ENSP00000261693	ENSP00000356969
ENSP00000261693	ENSP00000407401
ENSP00000261700	ENSP00000308546
ENSP00000261700	ENSP00000314949
ENSP00000261700	ENSP00000346120
ENSP00000261700	ENSP00000350708
ENSP00000261700	ENSP00000355596
ENSP00000261700	ENSP00000379607
ENSP00000261708	ENSP00000263576
ENSP00000261708	ENSP00000263657
ENSP00000261708	ENSP00000267425
ENSP00000261708	ENSP00000268482
ENSP00000261708	ENSP00000272521
ENSP00000261708	ENSP00000288828
ENSP00000261708	ENSP00000291576
ENSP00000261708	ENSP00000295373
ENSP00000261708	ENSP00000296792
ENSP00000261708	ENSP00000296802
ENSP00000261708	ENSP00000297579
ENSP00000261708	ENSP00000297990
ENSP00000261708	ENSP00000302896
ENSP00000261708	ENSP00000308179
ENSP00000261708	ENSP00000311135
ENSP00000261708	ENSP00000314193
ENSP00000261708	ENSP00000314348
ENSP00000261708	ENSP00000315674
ENSP00000261708	ENSP00000323858
ENSP00000261708	ENSP00000324122
ENSP00000261708	ENSP00000326981
ENSP00000261708	ENSP00000327179
ENSP00000261708	ENSP00000328690
ENSP00000261708	ENSP00000328854
ENSP00000261708	ENSP00000329466
ENSP00000261708	ENSP00000331817
ENSP00000261708	ENSP00000331907
ENSP00000261708	ENSP00000332340
ENSP00000261708	ENSP00000339095
ENSP00000261708	ENSP00000346120
ENSP00000261708	ENSP00000348848
ENSP00000261708	ENSP00000355471
ENSP00000261708	ENSP00000355541
ENSP00000261708	ENSP00000356134

ENSP00000261708	ENSP00000356520
ENSP00000261708	ENSP00000358716
ENSP00000261708	ENSP00000358812
ENSP00000261708	ENSP00000359688
ENSP00000261708	ENSP00000360828
ENSP00000261708	ENSP00000361232
ENSP00000261708	ENSP00000362744
ENSP00000261708	ENSP00000363642
ENSP00000261708	ENSP00000363746
ENSP00000261708	ENSP00000364119
ENSP00000261708	ENSP00000364320
ENSP00000261708	ENSP00000366557
ENSP00000261708	ENSP00000370589
ENSP00000261708	ENSP00000371101
ENSP00000261708	ENSP00000372695
ENSP00000261708	ENSP00000379888
ENSP00000261708	ENSP00000380495
ENSP00000261708	ENSP00000384302
ENSP00000261708	ENSP00000387303
ENSP00000261708	ENSP00000392601
ENSP00000261708	ENSP00000399454
ENSP00000261708	ENSP00000402338
ENSP00000261708	ENSP00000402869
ENSP00000261708	ENSP00000405614
ENSP00000261708	ENSP00000410396
ENSP00000261712	ENSP00000262193
ENSP00000261712	ENSP00000262320
ENSP00000261712	ENSP00000264515
ENSP00000261712	ENSP00000264639
ENSP00000261712	ENSP00000265990
ENSP00000261712	ENSP00000276326
ENSP00000261712	ENSP00000282516
ENSP00000261712	ENSP00000290541
ENSP00000261712	ENSP00000295066
ENSP00000261712	ENSP00000295901
ENSP00000261712	ENSP00000296543
ENSP00000261712	ENSP00000296786
ENSP00000261712	ENSP00000298852
ENSP00000261712	ENSP00000298910
ENSP00000261712	ENSP00000299259
ENSP00000261712	ENSP00000300413
ENSP00000261712	ENSP00000309474
ENSP00000261712	ENSP00000310129
ENSP00000261712	ENSP00000311665
ENSP00000261712	ENSP00000319977
ENSP00000261712	ENSP00000321711

ENSP00000261712	ENSP00000328940
ENSP00000261712	ENSP00000341243
ENSP00000261712	ENSP00000341551
ENSP00000261712	ENSP00000348442
ENSP00000261712	ENSP00000349970
ENSP00000261712	ENSP00000350708
ENSP00000261712	ENSP00000351947
ENSP00000261712	ENSP00000356130
ENSP00000261712	ENSP00000357980
ENSP00000261712	ENSP00000360532
ENSP00000261712	ENSP00000362306
ENSP00000261712	ENSP00000362334
ENSP00000261712	ENSP00000362413
ENSP00000261712	ENSP00000384211
ENSP00000261712	ENSP00000386212
ENSP00000261712	ENSP00000396937
ENSP00000261714	ENSP00000272252
ENSP00000261714	ENSP00000274030
ENSP00000261714	ENSP00000276326
ENSP00000261714	ENSP00000284981
ENSP00000261714	ENSP00000294189
ENSP00000261714	ENSP00000339299
ENSP00000261714	ENSP00000356591
ENSP00000261714	ENSP00000363162
ENSP00000261714	ENSP00000363676
ENSP00000261714	ENSP00000366746
ENSP00000261714	ENSP00000368119
ENSP00000261714	ENSP00000368797
ENSP00000261714	ENSP00000379607
ENSP00000261714	ENSP00000390783
ENSP00000261714	ENSP00000408058
ENSP00000261735	ENSP00000379607
ENSP00000261740	ENSP00000263246
ENSP00000261740	ENSP00000299163
ENSP00000261740	ENSP00000344668
ENSP00000261740	ENSP00000379457
ENSP00000261741	ENSP00000267425
ENSP00000261741	ENSP00000291576
ENSP00000261741	ENSP00000297990
ENSP00000261741	ENSP00000308179
ENSP00000261741	ENSP00000308332
ENSP00000261741	ENSP00000314441
ENSP00000261741	ENSP00000317992
ENSP00000261741	ENSP00000320917
ENSP00000261741	ENSP00000321449
ENSP00000261741	ENSP00000322396

ENSP00000261741	ENSP00000327179
ENSP00000261741	ENSP00000338862
ENSP00000261741	ENSP00000344393
ENSP00000261741	ENSP00000346453
ENSP00000261741	ENSP00000346725
ENSP00000261741	ENSP00000352021
ENSP00000261741	ENSP00000354040
ENSP00000261741	ENSP00000359688
ENSP00000261741	ENSP00000363642
ENSP00000261741	ENSP00000363746
ENSP00000261741	ENSP00000370736
ENSP00000261741	ENSP00000372695
ENSP00000261741	ENSP00000380982
ENSP00000261741	ENSP00000384302
ENSP00000261741	ENSP00000399454
ENSP00000261741	ENSP00000402338
ENSP00000261741	ENSP00000402869
ENSP00000261741	ENSP00000405614
ENSP00000261741	ENSP00000410396
ENSP00000261745	ENSP00000262962
ENSP00000261745	ENSP00000266095
ENSP00000261745	ENSP00000268661
ENSP00000261745	ENSP00000278572
ENSP00000261745	ENSP00000382177
ENSP00000261745	ENSP00000416583
ENSP00000261745	ENSP00000417763
ENSP00000261755	ENSP00000267430
ENSP00000261755	ENSP00000286070
ENSP00000261755	ENSP00000289081
ENSP00000261755	ENSP00000321636
ENSP00000261755	ENSP00000326819
ENSP00000261755	ENSP00000330875
ENSP00000261755	ENSP00000333283
ENSP00000261755	ENSP00000350283
ENSP00000261755	ENSP00000361824
ENSP00000261755	ENSP00000367910
ENSP00000261755	ENSP00000369274
ENSP00000261755	ENSP00000373952
ENSP00000261755	ENSP00000385021
ENSP00000261758	ENSP00000261868
ENSP00000261758	ENSP00000263377
ENSP00000261758	ENSP00000264389
ENSP00000261758	ENSP00000264705
ENSP00000261758	ENSP00000265350
ENSP00000261758	ENSP00000283195
ENSP00000261758	ENSP00000290158

ENSP00000261758	ENSP00000292035
ENSP00000261758	ENSP00000296411
ENSP00000261758	ENSP00000297145
ENSP00000261758	ENSP00000300291
ENSP00000261758	ENSP00000311766
ENSP00000261758	ENSP00000317790
ENSP00000261758	ENSP00000325612
ENSP00000261758	ENSP00000325690
ENSP00000261758	ENSP00000357047
ENSP00000261758	ENSP00000361824
ENSP00000261758	ENSP00000364320
ENSP00000261758	ENSP00000365439
ENSP00000261758	ENSP00000368226
ENSP00000261758	ENSP00000369411
ENSP00000261758	ENSP00000381216
ENSP00000261758	ENSP00000384048
ENSP00000261758	ENSP00000389277
ENSP00000261776	ENSP00000261842
ENSP00000261776	ENSP00000262039
ENSP00000261776	ENSP00000264380
ENSP00000261776	ENSP00000273398
ENSP00000261776	ENSP00000281141
ENSP00000261776	ENSP00000286448
ENSP00000261776	ENSP00000288828
ENSP00000261776	ENSP00000299138
ENSP00000261776	ENSP00000310521
ENSP00000261776	ENSP00000313490
ENSP00000261776	ENSP00000320758
ENSP00000261776	ENSP00000322957
ENSP00000261776	ENSP00000331817
ENSP00000261776	ENSP00000343741
ENSP00000261776	ENSP00000344220
ENSP00000261776	ENSP00000351926
ENSP00000261776	ENSP00000353741
ENSP00000261776	ENSP00000355231
ENSP00000261776	ENSP00000367079
ENSP00000261776	ENSP00000377148
ENSP00000261776	ENSP00000382177
ENSP00000261776	ENSP00000386759
ENSP00000261776	ENSP00000396937
ENSP00000261783	ENSP00000265537
ENSP00000261783	ENSP00000284637
ENSP00000261783	ENSP00000327509
ENSP00000261783	ENSP00000339186
ENSP00000261783	ENSP00000354347
ENSP00000261783	ENSP00000365811

ENSP00000261783	ENSP00000377954
ENSP00000261796	ENSP00000288167
ENSP00000261813	ENSP00000270708
ENSP00000261813	ENSP00000274255
ENSP00000261813	ENSP00000286428
ENSP00000261813	ENSP00000315859
ENSP00000261813	ENSP00000328938
ENSP00000261813	ENSP00000334188
ENSP00000261813	ENSP00000346037
ENSP00000261813	ENSP00000356989
ENSP00000261813	ENSP00000360473
ENSP00000261813	ENSP00000363313
ENSP00000261813	ENSP00000363734
ENSP00000261813	ENSP00000372694
ENSP00000261813	ENSP00000412319
ENSP00000261813	ENSP00000415678
ENSP00000261819	ENSP00000262030
ENSP00000261819	ENSP00000262144
ENSP00000261819	ENSP00000262367
ENSP00000261819	ENSP00000262608
ENSP00000261819	ENSP00000263208
ENSP00000261819	ENSP00000263228
ENSP00000261819	ENSP00000265351
ENSP00000261819	ENSP00000286800
ENSP00000261819	ENSP00000287598
ENSP00000261819	ENSP00000287878
ENSP00000261819	ENSP00000299381
ENSP00000261819	ENSP00000300648
ENSP00000261819	ENSP00000301019
ENSP00000261819	ENSP00000302790
ENSP00000261819	ENSP00000314004
ENSP00000261819	ENSP00000318775
ENSP00000261819	ENSP00000328720
ENSP00000261819	ENSP00000334061
ENSP00000261819	ENSP00000337340
ENSP00000261819	ENSP00000338157
ENSP00000261819	ENSP00000339109
ENSP00000261819	ENSP00000344741
ENSP00000261819	ENSP00000344936
ENSP00000261819	ENSP00000345530
ENSP00000261819	ENSP00000346987
ENSP00000261819	ENSP00000348554
ENSP00000261819	ENSP00000351947
ENSP00000261819	ENSP00000362592
ENSP00000261819	ENSP00000363322
ENSP00000261819	ENSP00000366953

ENSP00000261819	ENSP00000373060
ENSP00000261819	ENSP00000378350
ENSP00000261819	ENSP00000378529
ENSP00000261819	ENSP00000379228
ENSP00000261819	ENSP00000380921
ENSP00000261819	ENSP00000387982
ENSP00000261819	ENSP00000392985
ENSP00000261819	ENSP00000394394
ENSP00000261833	ENSP00000297338
ENSP00000261833	ENSP00000317985
ENSP00000261833	ENSP00000355596
ENSP00000261833	ENSP00000355731
ENSP00000261833	ENSP00000366603
ENSP00000261842	ENSP00000294543
ENSP00000261842	ENSP00000295897
ENSP00000261842	ENSP00000298229
ENSP00000261842	ENSP00000317431
ENSP00000261842	ENSP00000320543
ENSP00000261842	ENSP00000328773
ENSP00000261842	ENSP00000352603
ENSP00000261842	ENSP00000354519
ENSP00000261842	ENSP00000360777
ENSP00000261842	ENSP00000362115
ENSP00000261842	ENSP00000384534
ENSP00000261844	ENSP00000280098
ENSP00000261845	ENSP00000262982
ENSP00000261845	ENSP00000263688
ENSP00000261845	ENSP00000263791
ENSP00000261845	ENSP00000265000
ENSP00000261845	ENSP00000265073
ENSP00000261845	ENSP00000265354
ENSP00000261845	ENSP00000265960
ENSP00000261845	ENSP00000272542
ENSP00000261845	ENSP00000280772
ENSP00000261845	ENSP00000296328
ENSP00000261845	ENSP00000299601
ENSP00000261845	ENSP00000299767
ENSP00000261845	ENSP00000303315
ENSP00000261845	ENSP00000310649
ENSP00000261845	ENSP00000310701
ENSP00000261845	ENSP00000314949
ENSP00000261845	ENSP00000324444
ENSP00000261845	ENSP00000332444
ENSP00000261845	ENSP00000340019
ENSP00000261845	ENSP00000340879
ENSP00000261845	ENSP00000348986

ENSP00000261845	ENSP00000354558
ENSP00000261845	ENSP00000354916
ENSP00000261845	ENSP00000360621
ENSP00000261845	ENSP00000360644
ENSP00000261845	ENSP00000369042
ENSP00000261845	ENSP00000378306
ENSP00000261845	ENSP00000379372
ENSP00000261845	ENSP00000379607
ENSP00000261845	ENSP00000380129
ENSP00000261845	ENSP00000381045
ENSP00000261845	ENSP00000385083
ENSP00000261867	ENSP00000266095
ENSP00000261867	ENSP00000266980
ENSP00000261867	ENSP00000352655
ENSP00000261868	ENSP00000264883
ENSP00000261868	ENSP00000272645
ENSP00000261868	ENSP00000278572
ENSP00000261868	ENSP00000284995
ENSP00000261868	ENSP00000289371
ENSP00000261868	ENSP00000290158
ENSP00000261868	ENSP00000291688
ENSP00000261868	ENSP00000296577
ENSP00000261868	ENSP00000296674
ENSP00000261868	ENSP00000297325
ENSP00000261868	ENSP00000300146
ENSP00000261868	ENSP00000301788
ENSP00000261868	ENSP00000302886
ENSP00000261868	ENSP00000307684
ENSP00000261868	ENSP00000310040
ENSP00000261868	ENSP00000310596
ENSP00000261868	ENSP00000316879
ENSP00000261868	ENSP00000318604
ENSP00000261868	ENSP00000326806
ENSP00000261868	ENSP00000338862
ENSP00000261868	ENSP00000339881
ENSP00000261868	ENSP00000340281
ENSP00000261868	ENSP00000341885
ENSP00000261868	ENSP00000348722
ENSP00000261868	ENSP00000348965
ENSP00000261868	ENSP00000354125
ENSP00000261868	ENSP00000354522
ENSP00000261868	ENSP00000356548
ENSP00000261868	ENSP00000358140
ENSP00000261868	ENSP00000359518
ENSP00000261868	ENSP00000362576
ENSP00000261868	ENSP00000362688

ENSP00000261868	ENSP00000363391
ENSP00000261868	ENSP00000363559
ENSP00000261868	ENSP00000364289
ENSP00000261868	ENSP00000364320
ENSP00000261868	ENSP00000364847
ENSP00000261868	ENSP00000368927
ENSP00000261868	ENSP00000369038
ENSP00000261868	ENSP00000369042
ENSP00000261868	ENSP00000373918
ENSP00000261868	ENSP00000376758
ENSP00000261868	ENSP00000379888
ENSP00000261868	ENSP00000384015
ENSP00000261868	ENSP00000386992
ENSP00000261875	ENSP00000267750
ENSP00000261875	ENSP00000274311
ENSP00000261875	ENSP00000341213
ENSP00000261875	ENSP00000374455
ENSP00000261880	ENSP00000351926
ENSP00000261880	ENSP00000370258
ENSP00000261881	ENSP00000262315
ENSP00000261881	ENSP00000262460
ENSP00000261881	ENSP00000263201
ENSP00000261881	ENSP00000264926
ENSP00000261881	ENSP00000265056
ENSP00000261881	ENSP00000265465
ENSP00000261881	ENSP00000265728
ENSP00000261881	ENSP00000267430
ENSP00000261881	ENSP00000268043
ENSP00000261881	ENSP00000276533
ENSP00000261881	ENSP00000281543
ENSP00000261881	ENSP00000282516
ENSP00000261881	ENSP00000285873
ENSP00000261881	ENSP00000286428
ENSP00000261881	ENSP00000287598
ENSP00000261881	ENSP00000296273
ENSP00000261881	ENSP00000296509
ENSP00000261881	ENSP00000297338
ENSP00000261881	ENSP00000297431
ENSP00000261881	ENSP00000298139
ENSP00000261881	ENSP00000300249
ENSP00000261881	ENSP00000302530
ENSP00000261881	ENSP00000302955
ENSP00000261881	ENSP00000307853
ENSP00000261881	ENSP00000308699
ENSP00000261881	ENSP00000308944
ENSP00000261881	ENSP00000309457

ENSP00000261881	ENSP00000310520
ENSP00000261881	ENSP00000310668
ENSP00000261881	ENSP00000312870
ENSP00000261881	ENSP00000313171
ENSP00000261881	ENSP00000317039
ENSP00000261881	ENSP00000317636
ENSP00000261881	ENSP00000318977
ENSP00000261881	ENSP00000321636
ENSP00000261881	ENSP00000322180
ENSP00000261881	ENSP00000322570
ENSP00000261881	ENSP00000323663
ENSP00000261881	ENSP00000325017
ENSP00000261881	ENSP00000325863
ENSP00000261881	ENSP00000331879
ENSP00000261881	ENSP00000334188
ENSP00000261881	ENSP00000337405
ENSP00000261881	ENSP00000339145
ENSP00000261881	ENSP00000340879
ENSP00000261881	ENSP00000343392
ENSP00000261881	ENSP00000343741
ENSP00000261881	ENSP00000347232
ENSP00000261881	ENSP00000351697
ENSP00000261881	ENSP00000352738
ENSP00000261881	ENSP00000353793
ENSP00000261881	ENSP00000354522
ENSP00000261881	ENSP00000354720
ENSP00000261881	ENSP00000354972
ENSP00000261881	ENSP00000355890
ENSP00000261881	ENSP00000358478
ENSP00000261881	ENSP00000360473
ENSP00000261881	ENSP00000360621
ENSP00000261881	ENSP00000361465
ENSP00000261881	ENSP00000361707
ENSP00000261881	ENSP00000363021
ENSP00000261881	ENSP00000365588
ENSP00000261881	ENSP00000368349
ENSP00000261881	ENSP00000368438
ENSP00000261881	ENSP00000368976
ENSP00000261881	ENSP00000369335
ENSP00000261881	ENSP00000369411
ENSP00000261881	ENSP00000370151
ENSP00000261881	ENSP00000371475
ENSP00000261881	ENSP00000372023
ENSP00000261881	ENSP00000373060
ENSP00000261881	ENSP00000375809
ENSP00000261881	ENSP00000378529

ENSP00000261881	ENSP00000380129
ENSP00000261881	ENSP00000384048
ENSP00000261881	ENSP00000387429
ENSP00000261881	ENSP00000404318
ENSP00000261881	ENSP00000409167
ENSP00000261884	ENSP00000262367
ENSP00000261884	ENSP00000263205
ENSP00000261884	ENSP00000287820
ENSP00000261884	ENSP00000290663
ENSP00000261884	ENSP00000292035
ENSP00000261884	ENSP00000293777
ENSP00000261884	ENSP00000300651
ENSP00000261884	ENSP00000305502
ENSP00000261884	ENSP00000314949
ENSP00000261884	ENSP00000326767
ENSP00000261884	ENSP00000348610
ENSP00000261884	ENSP00000357047
ENSP00000261884	ENSP00000363193
ENSP00000261884	ENSP00000365588
ENSP00000261884	ENSP00000373060
ENSP00000261884	ENSP00000384048
ENSP00000261884	ENSP00000387429
ENSP00000261884	ENSP00000396484
ENSP00000261884	ENSP00000404318
ENSP00000261884	ENSP00000409167
ENSP00000261884	ENSP00000415212
ENSP00000261884	ENSP00000416511
ENSP00000261908	ENSP00000339299
ENSP00000261908	ENSP00000341189
ENSP00000261908	ENSP00000355809
ENSP00000261908	ENSP00000359345
ENSP00000261908	ENSP00000379607
ENSP00000261908	ENSP00000379712
ENSP00000261908	ENSP00000380942
ENSP00000261908	ENSP00000388373
ENSP00000261908	ENSP00000417229
ENSP00000261917	ENSP00000318351
ENSP00000261917	ENSP00000339191
ENSP00000261917	ENSP00000346725
ENSP00000261917	ENSP00000361465
ENSP00000261917	ENSP00000403852
ENSP00000261921	ENSP00000368253
ENSP00000261937	ENSP00000266037
ENSP00000261937	ENSP00000267101
ENSP00000261937	ENSP00000280193
ENSP00000261937	ENSP00000311005

ENSP00000261937	ENSP00000329219
ENSP00000261937	ENSP00000340944
ENSP00000261937	ENSP00000341189
ENSP00000261937	ENSP00000343785
ENSP00000261937	ENSP00000352121
ENSP00000261937	ENSP00000354995
ENSP00000261942	ENSP00000262776
ENSP00000261942	ENSP00000262854
ENSP00000261942	ENSP00000263202
ENSP00000261942	ENSP00000263966
ENSP00000261942	ENSP00000273067
ENSP00000261942	ENSP00000274140
ENSP00000261942	ENSP00000283943
ENSP00000261942	ENSP00000284811
ENSP00000261942	ENSP00000285928
ENSP00000261942	ENSP00000287936
ENSP00000261942	ENSP00000288828
ENSP00000261942	ENSP00000290649
ENSP00000261942	ENSP00000292363
ENSP00000261942	ENSP00000297313
ENSP00000261942	ENSP00000299626
ENSP00000261942	ENSP00000301281
ENSP00000261942	ENSP00000301764
ENSP00000261942	ENSP00000302176
ENSP00000261942	ENSP00000304604
ENSP00000261942	ENSP00000309031
ENSP00000261942	ENSP00000309141
ENSP00000261942	ENSP00000311496
ENSP00000261942	ENSP00000317087
ENSP00000261942	ENSP00000321617
ENSP00000261942	ENSP00000326813
ENSP00000261942	ENSP00000331487
ENSP00000261942	ENSP00000337053
ENSP00000261942	ENSP00000343001
ENSP00000261942	ENSP00000344741
ENSP00000261942	ENSP00000348069
ENSP00000261942	ENSP00000354775
ENSP00000261942	ENSP00000356591
ENSP00000261942	ENSP00000359380
ENSP00000261942	ENSP00000361990
ENSP00000261942	ENSP00000364403
ENSP00000261942	ENSP00000366395
ENSP00000261942	ENSP00000366748
ENSP00000261942	ENSP00000369335
ENSP00000261942	ENSP00000375881
ENSP00000261942	ENSP00000379457

ENSP00000261942	ENSP00000380460
ENSP00000261942	ENSP00000381045
ENSP00000261942	ENSP00000382269
ENSP00000261942	ENSP00000383911
ENSP00000261942	ENSP00000401445
ENSP00000261951	ENSP00000262300
ENSP00000261951	ENSP00000262803
ENSP00000261951	ENSP00000263629
ENSP00000261951	ENSP00000263736
ENSP00000261951	ENSP00000264157
ENSP00000261951	ENSP00000273064
ENSP00000261951	ENSP00000276072
ENSP00000261951	ENSP00000278616
ENSP00000261951	ENSP00000283882
ENSP00000261951	ENSP00000285896
ENSP00000261951	ENSP00000296328
ENSP00000261951	ENSP00000296509
ENSP00000261951	ENSP00000300648
ENSP00000261951	ENSP00000300738
ENSP00000261951	ENSP00000302160
ENSP00000261951	ENSP00000302955
ENSP00000261951	ENSP00000310596
ENSP00000261951	ENSP00000312735
ENSP00000261951	ENSP00000312981
ENSP00000261951	ENSP00000313171
ENSP00000261951	ENSP00000314949
ENSP00000261951	ENSP00000315700
ENSP00000261951	ENSP00000319104
ENSP00000261951	ENSP00000320949
ENSP00000261951	ENSP00000321711
ENSP00000261951	ENSP00000330060
ENSP00000261951	ENSP00000331879
ENSP00000261951	ENSP00000338967
ENSP00000261951	ENSP00000340823
ENSP00000261951	ENSP00000341743
ENSP00000261951	ENSP00000343741
ENSP00000261951	ENSP00000350491
ENSP00000261951	ENSP00000350990
ENSP00000261951	ENSP00000351284
ENSP00000261951	ENSP00000352738
ENSP00000261951	ENSP00000354522
ENSP00000261951	ENSP00000354673
ENSP00000261951	ENSP00000355051
ENSP00000261951	ENSP00000356405
ENSP00000261951	ENSP00000359552
ENSP00000261951	ENSP00000360515

ENSP00000261951	ENSP00000361949
ENSP00000261951	ENSP00000366135
ENSP00000261951	ENSP00000366557
ENSP00000261951	ENSP00000368438
ENSP00000261951	ENSP00000369965
ENSP00000261951	ENSP00000370151
ENSP00000261951	ENSP00000372023
ENSP00000261951	ENSP00000387170
ENSP00000261951	ENSP00000391723
ENSP00000261965	ENSP00000262300
ENSP00000261965	ENSP00000263181
ENSP00000261965	ENSP00000267068
ENSP00000261965	ENSP00000270861
ENSP00000261965	ENSP00000274289
ENSP00000261965	ENSP00000288976
ENSP00000261965	ENSP00000296509
ENSP00000261965	ENSP00000300584
ENSP00000261965	ENSP00000305941
ENSP00000261965	ENSP00000331831
ENSP00000261965	ENSP00000335357
ENSP00000261965	ENSP00000346566
ENSP00000261965	ENSP00000348573
ENSP00000261965	ENSP00000352572
ENSP00000261965	ENSP00000354916
ENSP00000261965	ENSP00000356405
ENSP00000261965	ENSP00000356448
ENSP00000261965	ENSP00000359206
ENSP00000261965	ENSP00000360473
ENSP00000261965	ENSP00000360532
ENSP00000261965	ENSP00000363734
ENSP00000261965	ENSP00000367049
ENSP00000261965	ENSP00000370517
ENSP00000261965	ENSP00000372694
ENSP00000261965	ENSP00000377470
ENSP00000261965	ENSP00000380921
ENSP00000261965	ENSP00000382328
ENSP00000261965	ENSP00000387262
ENSP00000261965	ENSP00000402084
ENSP00000261965	ENSP00000412319
ENSP00000261965	ENSP00000415678
ENSP00000261973	ENSP00000262027
ENSP00000261973	ENSP00000263377
ENSP00000261973	ENSP00000265073
ENSP00000261973	ENSP00000266079
ENSP00000261973	ENSP00000266735
ENSP00000261973	ENSP00000296702

ENSP00000261973	ENSP00000300413
ENSP00000261973	ENSP00000301740
ENSP00000261973	ENSP00000303191
ENSP00000261973	ENSP00000304350
ENSP00000261973	ENSP00000305790
ENSP00000261973	ENSP00000306983
ENSP00000261973	ENSP00000307863
ENSP00000261973	ENSP00000309457
ENSP00000261973	ENSP00000310448
ENSP00000261973	ENSP00000315859
ENSP00000261973	ENSP00000316879
ENSP00000261973	ENSP00000319341
ENSP00000261973	ENSP00000322016
ENSP00000261973	ENSP00000323302
ENSP00000261973	ENSP00000324122
ENSP00000261973	ENSP00000326261
ENSP00000261973	ENSP00000326806
ENSP00000261973	ENSP00000329466
ENSP00000261973	ENSP00000332103
ENSP00000261973	ENSP00000337405
ENSP00000261973	ENSP00000342374
ENSP00000261973	ENSP00000344241
ENSP00000261973	ENSP00000346986
ENSP00000261973	ENSP00000348010
ENSP00000261973	ENSP00000349658
ENSP00000261973	ENSP00000359518
ENSP00000261973	ENSP00000360147
ENSP00000261973	ENSP00000360464
ENSP00000261973	ENSP00000361577
ENSP00000261973	ENSP00000362166
ENSP00000261973	ENSP00000362900
ENSP00000261973	ENSP00000362936
ENSP00000261973	ENSP00000363313
ENSP00000261973	ENSP00000364289
ENSP00000261973	ENSP00000366607
ENSP00000261973	ENSP00000368801
ENSP00000261973	ENSP00000369218
ENSP00000261973	ENSP00000370377
ENSP00000261973	ENSP00000372023
ENSP00000261973	ENSP00000376309
ENSP00000261973	ENSP00000379607
ENSP00000261973	ENSP00000383059
ENSP00000261973	ENSP00000389649
ENSP00000261973	ENSP00000391432
ENSP00000261973	ENSP00000392330
ENSP00000261973	ENSP00000394085

ENSP00000261973	ENSP00000397598
ENSP00000261973	ENSP00000412566
ENSP00000261973	ENSP00000416959
ENSP00000261973	ENSP00000420195
ENSP00000262027	ENSP00000264161
ENSP00000262027	ENSP00000266126
ENSP00000262027	ENSP00000274680
ENSP00000262027	ENSP00000276326
ENSP00000262027	ENSP00000281828
ENSP00000262027	ENSP00000284719
ENSP00000262027	ENSP00000294053
ENSP00000262027	ENSP00000294168
ENSP00000262027	ENSP00000300648
ENSP00000262027	ENSP00000303279
ENSP00000262027	ENSP00000306920
ENSP00000262027	ENSP00000307567
ENSP00000262027	ENSP00000307900
ENSP00000262027	ENSP00000317904
ENSP00000262027	ENSP00000318921
ENSP00000262027	ENSP00000327821
ENSP00000262027	ENSP00000338862
ENSP00000262027	ENSP00000339933
ENSP00000262027	ENSP00000340698
ENSP00000262027	ENSP00000344537
ENSP00000262027	ENSP00000353741
ENSP00000262027	ENSP00000355890
ENSP00000262027	ENSP00000358812
ENSP00000262027	ENSP00000360327
ENSP00000262027	ENSP00000362576
ENSP00000262027	ENSP00000364794
ENSP00000262027	ENSP00000367029
ENSP00000262027	ENSP00000367408
ENSP00000262027	ENSP00000367498
ENSP00000262027	ENSP00000369038
ENSP00000262027	ENSP00000373918
ENSP00000262027	ENSP00000375863
ENSP00000262027	ENSP00000379607
ENSP00000262027	ENSP00000417764
ENSP00000262030	ENSP00000262375
ENSP00000262030	ENSP00000262455
ENSP00000262030	ENSP00000263208
ENSP00000262030	ENSP00000263642
ENSP00000262030	ENSP00000263791
ENSP00000262030	ENSP00000263805
ENSP00000262030	ENSP00000264065
ENSP00000262030	ENSP00000268379

ENSP00000262030	ENSP00000269143
ENSP00000262030	ENSP00000270538
ENSP00000262030	ENSP00000272227
ENSP00000262030	ENSP00000273398
ENSP00000262030	ENSP00000276326
ENSP00000262030	ENSP00000282007
ENSP00000262030	ENSP00000282050
ENSP00000262030	ENSP00000282549
ENSP00000262030	ENSP00000284320
ENSP00000262030	ENSP00000284719
ENSP00000262030	ENSP00000284727
ENSP00000262030	ENSP00000290299
ENSP00000262030	ENSP00000292180
ENSP00000262030	ENSP00000293362
ENSP00000262030	ENSP00000295030
ENSP00000262030	ENSP00000296930
ENSP00000262030	ENSP00000298852
ENSP00000262030	ENSP00000299543
ENSP00000262030	ENSP00000299767
ENSP00000262030	ENSP00000300688
ENSP00000262030	ENSP00000301585
ENSP00000262030	ENSP00000301587
ENSP00000262030	ENSP00000303511
ENSP00000262030	ENSP00000304668
ENSP00000262030	ENSP00000305465
ENSP00000262030	ENSP00000305958
ENSP00000262030	ENSP00000307567
ENSP00000262030	ENSP00000309565
ENSP00000262030	ENSP00000312735
ENSP00000262030	ENSP00000313953
ENSP00000262030	ENSP00000314380
ENSP00000262030	ENSP00000323313
ENSP00000262030	ENSP00000327070
ENSP00000262030	ENSP00000329002
ENSP00000262030	ENSP00000332887
ENSP00000262030	ENSP00000335203
ENSP00000262030	ENSP00000341243
ENSP00000262030	ENSP00000344431
ENSP00000262030	ENSP00000346022
ENSP00000262030	ENSP00000346067
ENSP00000262030	ENSP00000349142
ENSP00000262030	ENSP00000351492
ENSP00000262030	ENSP00000354632
ENSP00000262030	ENSP00000354876
ENSP00000262030	ENSP00000354982
ENSP00000262030	ENSP00000355890

ENSP00000262030	ENSP00000358737
ENSP00000262030	ENSP00000360286
ENSP00000262030	ENSP00000360782
ENSP00000262030	ENSP00000361005
ENSP00000262030	ENSP00000361207
ENSP00000262030	ENSP00000361686
ENSP00000262030	ENSP00000362688
ENSP00000262030	ENSP00000364486
ENSP00000262030	ENSP00000367934
ENSP00000262030	ENSP00000368646
ENSP00000262030	ENSP00000370414
ENSP00000262030	ENSP00000370741
ENSP00000262030	ENSP00000373952
ENSP00000262030	ENSP00000379607
ENSP00000262030	ENSP00000387262
ENSP00000262030	ENSP00000389649
ENSP00000262030	ENSP00000392270
ENSP00000262030	ENSP00000403817
ENSP00000262030	ENSP00000417190
ENSP00000262032	ENSP00000353622
ENSP00000262032	ENSP00000357881
ENSP00000262039	ENSP00000262850
ENSP00000262039	ENSP00000263974
ENSP00000262039	ENSP00000264380
ENSP00000262039	ENSP00000264864
ENSP00000262039	ENSP00000264926
ENSP00000262039	ENSP00000273398
ENSP00000262039	ENSP00000277746
ENSP00000262039	ENSP00000278412
ENSP00000262039	ENSP00000279230
ENSP00000262039	ENSP00000282412
ENSP00000262039	ENSP00000284957
ENSP00000262039	ENSP00000290330
ENSP00000262039	ENSP00000294168
ENSP00000262039	ENSP00000296343
ENSP00000262039	ENSP00000298552
ENSP00000262039	ENSP00000306682
ENSP00000262039	ENSP00000307684
ENSP00000262039	ENSP00000309457
ENSP00000262039	ENSP00000312411
ENSP00000262039	ENSP00000313420
ENSP00000262039	ENSP00000314499
ENSP00000262039	ENSP00000320646
ENSP00000262039	ENSP00000325863
ENSP00000262039	ENSP00000342681
ENSP00000262039	ENSP00000344537

ENSP00000262039	ENSP00000344668
ENSP00000262039	ENSP00000346566
ENSP00000262039	ENSP00000348455
ENSP00000262039	ENSP00000348784
ENSP00000262039	ENSP00000349205
ENSP00000262039	ENSP00000349785
ENSP00000262039	ENSP00000354519
ENSP00000262039	ENSP00000354558
ENSP00000262039	ENSP00000355231
ENSP00000262039	ENSP00000358423
ENSP00000262039	ENSP00000364893
ENSP00000262039	ENSP00000366565
ENSP00000262039	ENSP00000367265
ENSP00000262039	ENSP00000369335
ENSP00000262039	ENSP00000369810
ENSP00000262039	ENSP00000373702
ENSP00000262039	ENSP00000402515
ENSP00000262041	ENSP00000344609
ENSP00000262041	ENSP00000345445
ENSP00000262041	ENSP00000356213
ENSP00000262041	ENSP00000364524
ENSP00000262041	ENSP00000381499
ENSP00000262043	ENSP00000269703
ENSP00000262043	ENSP00000329466
ENSP00000262043	ENSP00000356448
ENSP00000262052	ENSP00000268379
ENSP00000262052	ENSP00000280154
ENSP00000262052	ENSP00000300658
ENSP00000262052	ENSP00000308750
ENSP00000262052	ENSP00000311200
ENSP00000262052	ENSP00000317468
ENSP00000262052	ENSP00000338387
ENSP00000262052	ENSP00000339845
ENSP00000262052	ENSP00000345530
ENSP00000262052	ENSP00000349877
ENSP00000262052	ENSP00000355865
ENSP00000262052	ENSP00000360782
ENSP00000262052	ENSP00000371169
ENSP00000262052	ENSP00000374455
ENSP00000262055	ENSP00000358242
ENSP00000262055	ENSP00000368959
ENSP00000262056	ENSP00000264718
ENSP00000262056	ENSP00000266735
ENSP00000262056	ENSP00000267415
ENSP00000262056	ENSP00000268482
ENSP00000262056	ENSP00000273064

ENSP00000262056	ENSP00000276603
ENSP00000262056	ENSP00000303191
ENSP00000262056	ENSP00000304370
ENSP00000262056	ENSP00000305790
ENSP00000262056	ENSP00000310040
ENSP00000262056	ENSP00000317992
ENSP00000262056	ENSP00000320658
ENSP00000262056	ENSP00000327821
ENSP00000262056	ENSP00000328690
ENSP00000262056	ENSP00000328854
ENSP00000262056	ENSP00000335321
ENSP00000262056	ENSP00000336702
ENSP00000262056	ENSP00000337701
ENSP00000262056	ENSP00000338862
ENSP00000262056	ENSP00000343535
ENSP00000262056	ENSP00000347329
ENSP00000262056	ENSP00000348010
ENSP00000262056	ENSP00000350249
ENSP00000262056	ENSP00000354125
ENSP00000262056	ENSP00000354673
ENSP00000262056	ENSP00000358140
ENSP00000262056	ENSP00000358423
ENSP00000262056	ENSP00000362306
ENSP00000262056	ENSP00000362576
ENSP00000262056	ENSP00000362688
ENSP00000262056	ENSP00000366557
ENSP00000262056	ENSP00000368797
ENSP00000262056	ENSP00000370372
ENSP00000262056	ENSP00000371607
ENSP00000262056	ENSP00000373700
ENSP00000262056	ENSP00000376973
ENSP00000262056	ENSP00000377496
ENSP00000262056	ENSP00000391723
ENSP00000262056	ENSP00000400591
ENSP00000262059	ENSP00000262367
ENSP00000262059	ENSP00000344456
ENSP00000262059	ENSP00000355050
ENSP00000262059	ENSP00000400806
ENSP00000262077	ENSP00000262982
ENSP00000262077	ENSP00000264883
ENSP00000262077	ENSP00000265351
ENSP00000262077	ENSP00000265388
ENSP00000262077	ENSP00000270176
ENSP00000262077	ENSP00000272645
ENSP00000262077	ENSP00000283195
ENSP00000262077	ENSP00000285968

ENSP00000262077	ENSP00000286428
ENSP00000262077	ENSP00000288937
ENSP00000262077	ENSP00000290158
ENSP00000262077	ENSP00000290231
ENSP00000262077	ENSP00000291688
ENSP00000262077	ENSP00000294008
ENSP00000262077	ENSP00000296257
ENSP00000262077	ENSP00000296509
ENSP00000262077	ENSP00000302886
ENSP00000262077	ENSP00000305503
ENSP00000262077	ENSP00000308275
ENSP00000262077	ENSP00000308622
ENSP00000262077	ENSP00000310668
ENSP00000262077	ENSP00000314949
ENSP00000262077	ENSP00000315017
ENSP00000262077	ENSP00000316032
ENSP00000262077	ENSP00000326806
ENSP00000262077	ENSP00000327821
ENSP00000262077	ENSP00000329466
ENSP00000262077	ENSP00000331817
ENSP00000262077	ENSP00000333813
ENSP00000262077	ENSP00000336712
ENSP00000262077	ENSP00000343741
ENSP00000262077	ENSP00000344456
ENSP00000262077	ENSP00000345895
ENSP00000262077	ENSP00000348577
ENSP00000262077	ENSP00000350283
ENSP00000262077	ENSP00000352400
ENSP00000262077	ENSP00000354673
ENSP00000262077	ENSP00000354742
ENSP00000262077	ENSP00000356448
ENSP00000262077	ENSP00000356744
ENSP00000262077	ENSP00000356989
ENSP00000262077	ENSP00000357283
ENSP00000262077	ENSP00000360031
ENSP00000262077	ENSP00000360286
ENSP00000262077	ENSP00000361418
ENSP00000262077	ENSP00000363763
ENSP00000262077	ENSP00000364289
ENSP00000262077	ENSP00000364293
ENSP00000262077	ENSP00000365837
ENSP00000262077	ENSP00000368439
ENSP00000262077	ENSP00000368856
ENSP00000262077	ENSP00000369042
ENSP00000262077	ENSP00000371155
ENSP00000262077	ENSP00000372023

ENSP00000262077	ENSP00000372199
ENSP00000262077	ENSP00000382779
ENSP00000262077	ENSP00000390158
ENSP00000262077	ENSP00000402338
ENSP00000262096	ENSP00000344456
ENSP00000262096	ENSP00000362021
ENSP00000262096	ENSP00000377372
ENSP00000262102	ENSP00000264169
ENSP00000262127	ENSP00000333537
ENSP00000262127	ENSP00000342510
ENSP00000262127	ENSP00000349576
ENSP00000262127	ENSP00000354848
ENSP00000262127	ENSP00000368464
ENSP00000262127	ENSP00000374529
ENSP00000262127	ENSP00000382390
ENSP00000262127	ENSP00000382595
ENSP00000262133	ENSP00000262643
ENSP00000262133	ENSP00000263121
ENSP00000262133	ENSP00000263360
ENSP00000262133	ENSP00000267163
ENSP00000262133	ENSP00000274255
ENSP00000262133	ENSP00000276072
ENSP00000262133	ENSP00000302640
ENSP00000262133	ENSP00000305899
ENSP00000262133	ENSP00000310623
ENSP00000262133	ENSP00000317872
ENSP00000262133	ENSP00000320340
ENSP00000262133	ENSP00000323050
ENSP00000262133	ENSP00000329029
ENSP00000262133	ENSP00000329102
ENSP00000262133	ENSP00000332353
ENSP00000262133	ENSP00000335261
ENSP00000262133	ENSP00000336866
ENSP00000262133	ENSP00000340305
ENSP00000262133	ENSP00000341947
ENSP00000262133	ENSP00000350283
ENSP00000262133	ENSP00000352514
ENSP00000262133	ENSP00000355922
ENSP00000262133	ENSP00000362592
ENSP00000262133	ENSP00000365877
ENSP00000262133	ENSP00000368686
ENSP00000262133	ENSP00000386165
ENSP00000262134	ENSP00000302177
ENSP00000262134	ENSP00000311505
ENSP00000262144	ENSP00000263208
ENSP00000262144	ENSP00000264169

ENSP00000262144	ENSP00000290130
ENSP00000262144	ENSP00000293883
ENSP00000262144	ENSP00000297431
ENSP00000262144	ENSP00000339881
ENSP00000262144	ENSP00000343885
ENSP00000262144	ENSP00000344741
ENSP00000262144	ENSP00000360286
ENSP00000262144	ENSP00000360621
ENSP00000262144	ENSP00000368438
ENSP00000262144	ENSP00000369335
ENSP00000262144	ENSP00000371546
ENSP00000262144	ENSP00000382779
ENSP00000262173	ENSP00000263228
ENSP00000262173	ENSP00000264157
ENSP00000262173	ENSP00000286621
ENSP00000262173	ENSP00000296328
ENSP00000262173	ENSP00000307181
ENSP00000262173	ENSP00000309433
ENSP00000262173	ENSP00000312735
ENSP00000262173	ENSP00000314949
ENSP00000262173	ENSP00000315931
ENSP00000262173	ENSP00000319104
ENSP00000262173	ENSP00000348762
ENSP00000262173	ENSP00000358497
ENSP00000262173	ENSP00000361287
ENSP00000262173	ENSP00000364289
ENSP00000262173	ENSP00000373715
ENSP00000262173	ENSP00000374529
ENSP00000262177	ENSP00000264731
ENSP00000262177	ENSP00000294008
ENSP00000262177	ENSP00000294053
ENSP00000262177	ENSP00000302239
ENSP00000262177	ENSP00000302478
ENSP00000262177	ENSP00000305958
ENSP00000262177	ENSP00000329558
ENSP00000262177	ENSP00000334061
ENSP00000262177	ENSP00000346791
ENSP00000262177	ENSP00000360286
ENSP00000262177	ENSP00000361310
ENSP00000262177	ENSP00000362306
ENSP00000262177	ENSP00000365435
ENSP00000262177	ENSP00000367208
ENSP00000262177	ENSP00000381003
ENSP00000262177	ENSP00000397435
ENSP00000262193	ENSP00000262966
ENSP00000262193	ENSP00000264639

ENSP00000262193	ENSP00000266126
ENSP00000262193	ENSP00000268668
ENSP00000262193	ENSP00000277541
ENSP00000262193	ENSP00000282050
ENSP00000262193	ENSP00000290541
ENSP00000262193	ENSP00000290949
ENSP00000262193	ENSP00000293362
ENSP00000262193	ENSP00000295901
ENSP00000262193	ENSP00000296786
ENSP00000262193	ENSP00000298852
ENSP00000262193	ENSP00000309474
ENSP00000262193	ENSP00000310129
ENSP00000262193	ENSP00000311344
ENSP00000262193	ENSP00000311505
ENSP00000262193	ENSP00000314837
ENSP00000262193	ENSP00000320543
ENSP00000262193	ENSP00000325919
ENSP00000262193	ENSP00000326366
ENSP00000262193	ENSP00000328940
ENSP00000262193	ENSP00000346453
ENSP00000262193	ENSP00000348442
ENSP00000262193	ENSP00000350512
ENSP00000262193	ENSP00000350708
ENSP00000262193	ENSP00000352425
ENSP00000262193	ENSP00000356130
ENSP00000262193	ENSP00000360268
ENSP00000262193	ENSP00000360519
ENSP00000262193	ENSP00000362334
ENSP00000262193	ENSP00000365775
ENSP00000262193	ENSP00000367910
ENSP00000262193	ENSP00000370222
ENSP00000262193	ENSP00000370741
ENSP00000262193	ENSP00000373715
ENSP00000262193	ENSP00000374455
ENSP00000262193	ENSP00000375986
ENSP00000262193	ENSP00000379217
ENSP00000262193	ENSP00000379607
ENSP00000262193	ENSP00000381577
ENSP00000262193	ENSP00000384211
ENSP00000262193	ENSP00000396937
ENSP00000262193	ENSP00000398597
ENSP00000262193	ENSP00000403852
ENSP00000262193	ENSP00000418734
ENSP00000262198	ENSP00000396620
ENSP00000262213	ENSP00000346022
ENSP00000262213	ENSP00000376445

ENSP00000262213	ENSP00000392330
ENSP00000262215	ENSP00000262544
ENSP00000262215	ENSP00000262982
ENSP00000262215	ENSP00000263121
ENSP00000262215	ENSP00000265175
ENSP00000262215	ENSP00000267064
ENSP00000262215	ENSP00000295066
ENSP00000262215	ENSP00000305503
ENSP00000262215	ENSP00000308496
ENSP00000262215	ENSP00000311134
ENSP00000262215	ENSP00000318195
ENSP00000262215	ENSP00000323967
ENSP00000262215	ENSP00000325002
ENSP00000262215	ENSP00000328690
ENSP00000262215	ENSP00000334100
ENSP00000262215	ENSP00000337463
ENSP00000262215	ENSP00000345530
ENSP00000262215	ENSP00000346453
ENSP00000262215	ENSP00000349131
ENSP00000262215	ENSP00000349213
ENSP00000262215	ENSP00000350409
ENSP00000262215	ENSP00000351682
ENSP00000262215	ENSP00000351957
ENSP00000262215	ENSP00000352738
ENSP00000262215	ENSP00000354347
ENSP00000262215	ENSP00000354851
ENSP00000262215	ENSP00000355890
ENSP00000262215	ENSP00000359000
ENSP00000262215	ENSP00000359067
ENSP00000262215	ENSP00000359837
ENSP00000262215	ENSP00000365943
ENSP00000262215	ENSP00000368565
ENSP00000262215	ENSP00000379228
ENSP00000262215	ENSP00000380702
ENSP00000262215	ENSP00000382723
ENSP00000262215	ENSP00000388872
ENSP00000262215	ENSP00000389516
ENSP00000262215	ENSP00000392028
ENSP00000262215	ENSP00000395863
ENSP00000262215	ENSP00000396620
ENSP00000262215	ENSP00000401287
ENSP00000262215	ENSP00000405864
ENSP00000262215	ENSP00000412805
ENSP00000262238	ENSP00000262367
ENSP00000262238	ENSP00000262904
ENSP00000262238	ENSP00000263360

ENSP00000262238	ENSP00000264010
ENSP00000262238	ENSP00000267163
ENSP00000262238	ENSP00000271883
ENSP00000262238	ENSP00000274255
ENSP00000262238	ENSP00000276603
ENSP00000262238	ENSP00000276893
ENSP00000262238	ENSP00000277541
ENSP00000262238	ENSP00000296504
ENSP00000262238	ENSP00000296930
ENSP00000262238	ENSP00000297261
ENSP00000262238	ENSP00000303315
ENSP00000262238	ENSP00000306245
ENSP00000262238	ENSP00000309555
ENSP00000262238	ENSP00000313420
ENSP00000262238	ENSP00000314311
ENSP00000262238	ENSP00000329357
ENSP00000262238	ENSP00000336842
ENSP00000262238	ENSP00000338127
ENSP00000262238	ENSP00000341551
ENSP00000262238	ENSP00000343785
ENSP00000262238	ENSP00000348069
ENSP00000262238	ENSP00000349640
ENSP00000262238	ENSP00000350283
ENSP00000262238	ENSP00000351490
ENSP00000262238	ENSP00000351947
ENSP00000262238	ENSP00000352257
ENSP00000262238	ENSP00000353622
ENSP00000262238	ENSP00000354995
ENSP00000262238	ENSP00000355153
ENSP00000262238	ENSP00000355205
ENSP00000262238	ENSP00000359531
ENSP00000262238	ENSP00000359958
ENSP00000262238	ENSP00000376204
ENSP00000262238	ENSP00000384198
ENSP00000262238	ENSP00000391457
ENSP00000262238	ENSP00000391735
ENSP00000262238	ENSP00000393275
ENSP00000262238	ENSP00000395535
ENSP00000262238	ENSP00000400476
ENSP00000262238	ENSP00000404121
ENSP00000262238	ENSP00000408792
ENSP00000262238	ENSP00000417132
ENSP00000262238	ENSP00000417281
ENSP00000262238	ENSP00000419494
ENSP00000262265	ENSP00000264718
ENSP00000262265	ENSP00000266712

ENSP00000262265	ENSP00000268057
ENSP00000262265	ENSP00000272521
ENSP00000262265	ENSP00000300181
ENSP00000262265	ENSP00000323275
ENSP00000262265	ENSP00000323424
ENSP00000262265	ENSP00000324122
ENSP00000262265	ENSP00000333018
ENSP00000262265	ENSP00000350011
ENSP00000262265	ENSP00000353971
ENSP00000262265	ENSP00000355621
ENSP00000262265	ENSP00000357220
ENSP00000262265	ENSP00000360497
ENSP00000262265	ENSP00000360876
ENSP00000262265	ENSP00000361010
ENSP00000262265	ENSP00000362386
ENSP00000262265	ENSP00000362546
ENSP00000262265	ENSP00000364298
ENSP00000262265	ENSP00000366135
ENSP00000262265	ENSP00000370258
ENSP00000262265	ENSP00000371169
ENSP00000262265	ENSP00000397530
ENSP00000262265	ENSP00000398812
ENSP00000262265	ENSP00000412439
ENSP00000262291	ENSP00000281537
ENSP00000262291	ENSP00000331201
ENSP00000262291	ENSP00000345656
ENSP00000262294	ENSP00000264108
ENSP00000262294	ENSP00000289013
ENSP00000262294	ENSP00000299601
ENSP00000262294	ENSP00000323511
ENSP00000262294	ENSP00000326767
ENSP00000262294	ENSP00000343818
ENSP00000262294	ENSP00000347836
ENSP00000262294	ENSP00000349640
ENSP00000262294	ENSP00000354270
ENSP00000262294	ENSP00000360116
ENSP00000262294	ENSP00000364126
ENSP00000262294	ENSP00000365435
ENSP00000262294	ENSP00000377793
ENSP00000262294	ENSP00000381031
ENSP00000262294	ENSP00000381795
ENSP00000262294	ENSP00000386895
ENSP00000262294	ENSP00000400806
ENSP00000262300	ENSP00000263181
ENSP00000262300	ENSP00000263208
ENSP00000262300	ENSP00000263228

ENSP00000262300	ENSP00000263710
ENSP00000262300	ENSP00000264108
ENSP00000262300	ENSP00000264169
ENSP00000262300	ENSP00000264731
ENSP00000262300	ENSP00000265148
ENSP00000262300	ENSP00000265562
ENSP00000262300	ENSP00000268533
ENSP00000262300	ENSP00000270225
ENSP00000262300	ENSP00000281187
ENSP00000262300	ENSP00000281708
ENSP00000262300	ENSP00000282388
ENSP00000262300	ENSP00000283228
ENSP00000262300	ENSP00000285873
ENSP00000262300	ENSP00000285896
ENSP00000262300	ENSP00000287598
ENSP00000262300	ENSP00000295951
ENSP00000262300	ENSP00000296509
ENSP00000262300	ENSP00000296733
ENSP00000262300	ENSP00000297338
ENSP00000262300	ENSP00000298048
ENSP00000262300	ENSP00000299767
ENSP00000262300	ENSP00000301764
ENSP00000262300	ENSP00000302530
ENSP00000262300	ENSP00000304226
ENSP00000262300	ENSP00000305790
ENSP00000262300	ENSP00000306124
ENSP00000262300	ENSP00000310649
ENSP00000262300	ENSP00000311005
ENSP00000262300	ENSP00000311083
ENSP00000262300	ENSP00000312150
ENSP00000262300	ENSP00000315659
ENSP00000262300	ENSP00000316845
ENSP00000262300	ENSP00000318884
ENSP00000262300	ENSP00000319169
ENSP00000262300	ENSP00000320488
ENSP00000262300	ENSP00000322170
ENSP00000262300	ENSP00000322957
ENSP00000262300	ENSP00000323377
ENSP00000262300	ENSP00000327315
ENSP00000262300	ENSP00000329623
ENSP00000262300	ENSP00000332171
ENSP00000262300	ENSP00000333950
ENSP00000262300	ENSP00000340879
ENSP00000262300	ENSP00000341848
ENSP00000262300	ENSP00000343741
ENSP00000262300	ENSP00000345656

ENSP00000262300	ENSP00000348163
ENSP00000262300	ENSP00000348215
ENSP00000262300	ENSP00000348573
ENSP00000262300	ENSP00000351113
ENSP00000262300	ENSP00000351885
ENSP00000262300	ENSP00000352572
ENSP00000262300	ENSP00000354486
ENSP00000262300	ENSP00000354558
ENSP00000262300	ENSP00000355884
ENSP00000262300	ENSP00000355922
ENSP00000262300	ENSP00000359552
ENSP00000262300	ENSP00000360310
ENSP00000262300	ENSP00000360621
ENSP00000262300	ENSP00000361009
ENSP00000262300	ENSP00000362171
ENSP00000262300	ENSP00000362578
ENSP00000262300	ENSP00000365963
ENSP00000262300	ENSP00000368140
ENSP00000262300	ENSP00000371475
ENSP00000262300	ENSP00000372023
ENSP00000262300	ENSP00000373107
ENSP00000262300	ENSP00000375986
ENSP00000262300	ENSP00000376410
ENSP00000262300	ENSP00000377047
ENSP00000262300	ENSP00000378529
ENSP00000262300	ENSP00000380450
ENSP00000262300	ENSP00000382177
ENSP00000262300	ENSP00000386621
ENSP00000262300	ENSP00000391723
ENSP00000262300	ENSP00000394056
ENSP00000262300	ENSP00000397351
ENSP00000262300	ENSP00000403557
ENSP00000262300	ENSP00000407981
ENSP00000262300	ENSP00000411038
ENSP00000262300	ENSP00000412297
ENSP00000262300	ENSP00000414808
ENSP00000262300	ENSP00000417963
ENSP00000262304	ENSP00000313169
ENSP00000262304	ENSP00000341189
ENSP00000262304	ENSP00000344456
ENSP00000262304	ENSP00000346839
ENSP00000262304	ENSP00000368391
ENSP00000262305	ENSP00000309871
ENSP00000262305	ENSP00000416511
ENSP00000262306	ENSP00000262394
ENSP00000262306	ENSP00000264607

ENSP00000262306	ENSP00000283921
ENSP00000262306	ENSP00000284811
ENSP00000262306	ENSP00000290663
ENSP00000262306	ENSP00000291900
ENSP00000262306	ENSP00000296328
ENSP00000262306	ENSP00000298288
ENSP00000262306	ENSP00000307854
ENSP00000262306	ENSP00000334188
ENSP00000262306	ENSP00000343298
ENSP00000262306	ENSP00000351407
ENSP00000262306	ENSP00000370968
ENSP00000262306	ENSP00000379607
ENSP00000262306	ENSP00000395574
ENSP00000262306	ENSP00000397435
ENSP00000262306	ENSP00000409346
ENSP00000262315	ENSP00000262460
ENSP00000262315	ENSP00000263181
ENSP00000262315	ENSP00000263201
ENSP00000262315	ENSP00000263202
ENSP00000262315	ENSP00000263208
ENSP00000262315	ENSP00000263274
ENSP00000262315	ENSP00000263710
ENSP00000262315	ENSP00000264515
ENSP00000262315	ENSP00000264926
ENSP00000262315	ENSP00000265007
ENSP00000262315	ENSP00000265056
ENSP00000262315	ENSP00000265140
ENSP00000262315	ENSP00000265148
ENSP00000262315	ENSP00000265354
ENSP00000262315	ENSP00000265465
ENSP00000262315	ENSP00000265728
ENSP00000262315	ENSP00000268043
ENSP00000262315	ENSP00000269214
ENSP00000262315	ENSP00000278412
ENSP00000262315	ENSP00000278616
ENSP00000262315	ENSP00000282516
ENSP00000262315	ENSP00000282572
ENSP00000262315	ENSP00000286428
ENSP00000262315	ENSP00000287598
ENSP00000262315	ENSP00000291688
ENSP00000262315	ENSP00000295066
ENSP00000262315	ENSP00000296273
ENSP00000262315	ENSP00000296509
ENSP00000262315	ENSP00000297316
ENSP00000262315	ENSP00000297338
ENSP00000262315	ENSP00000297431

ENSP00000262315	ENSP00000298139
ENSP00000262315	ENSP00000300249
ENSP00000262315	ENSP00000300738
ENSP00000262315	ENSP00000302530
ENSP00000262315	ENSP00000304593
ENSP00000262315	ENSP00000306999
ENSP00000262315	ENSP00000307853
ENSP00000262315	ENSP00000309572
ENSP00000262315	ENSP00000310649
ENSP00000262315	ENSP00000311873
ENSP00000262315	ENSP00000312870
ENSP00000262315	ENSP00000315700
ENSP00000262315	ENSP00000317636
ENSP00000262315	ENSP00000318977
ENSP00000262315	ENSP00000321636
ENSP00000262315	ENSP00000322180
ENSP00000262315	ENSP00000322570
ENSP00000262315	ENSP00000322957
ENSP00000262315	ENSP00000323663
ENSP00000262315	ENSP00000325017
ENSP00000262315	ENSP00000325863
ENSP00000262315	ENSP00000328720
ENSP00000262315	ENSP00000332444
ENSP00000262315	ENSP00000334188
ENSP00000262315	ENSP00000335357
ENSP00000262315	ENSP00000336606
ENSP00000262315	ENSP00000336868
ENSP00000262315	ENSP00000340879
ENSP00000262315	ENSP00000343392
ENSP00000262315	ENSP00000343741
ENSP00000262315	ENSP00000343745
ENSP00000262315	ENSP00000347232
ENSP00000262315	ENSP00000347719
ENSP00000262315	ENSP00000350491
ENSP00000262315	ENSP00000350854
ENSP00000262315	ENSP00000350967
ENSP00000262315	ENSP00000351284
ENSP00000262315	ENSP00000351346
ENSP00000262315	ENSP00000351697
ENSP00000262315	ENSP00000352257
ENSP00000262315	ENSP00000353458
ENSP00000262315	ENSP00000353793
ENSP00000262315	ENSP00000354522
ENSP00000262315	ENSP00000354720
ENSP00000262315	ENSP00000354742
ENSP00000262315	ENSP00000354916

ENSP00000262315	ENSP00000355560
ENSP00000262315	ENSP00000355922
ENSP00000262315	ENSP00000356989
ENSP00000262315	ENSP00000358886
ENSP00000262315	ENSP00000359518
ENSP00000262315	ENSP00000359567
ENSP00000262315	ENSP00000360310
ENSP00000262315	ENSP00000360473
ENSP00000262315	ENSP00000360621
ENSP00000262315	ENSP00000360803
ENSP00000262315	ENSP00000363021
ENSP00000262315	ENSP00000366974
ENSP00000262315	ENSP00000368349
ENSP00000262315	ENSP00000368438
ENSP00000262315	ENSP00000368646
ENSP00000262315	ENSP00000368976
ENSP00000262315	ENSP00000369335
ENSP00000262315	ENSP00000369411
ENSP00000262315	ENSP00000370151
ENSP00000262315	ENSP00000371475
ENSP00000262315	ENSP00000372023
ENSP00000262315	ENSP00000375809
ENSP00000262315	ENSP00000377384
ENSP00000262315	ENSP00000377793
ENSP00000262315	ENSP00000380504
ENSP00000262315	ENSP00000381290
ENSP00000262315	ENSP00000381657
ENSP00000262315	ENSP00000382562
ENSP00000262315	ENSP00000384048
ENSP00000262315	ENSP00000396704
ENSP00000262315	ENSP00000402515
ENSP00000262315	ENSP00000413632
ENSP00000262319	ENSP00000278616
ENSP00000262319	ENSP00000295901
ENSP00000262319	ENSP00000313420
ENSP00000262319	ENSP00000316032
ENSP00000262319	ENSP00000337797
ENSP00000262319	ENSP00000343741
ENSP00000262319	ENSP00000347733
ENSP00000262319	ENSP00000352257
ENSP00000262319	ENSP00000353073
ENSP00000262319	ENSP00000353971
ENSP00000262319	ENSP00000354558
ENSP00000262319	ENSP00000357907
ENSP00000262319	ENSP00000359531
ENSP00000262319	ENSP00000362546

ENSP00000262319	ENSP00000364847
ENSP00000262319	ENSP00000367454
ENSP00000262319	ENSP00000371888
ENSP00000262319	ENSP00000397435
ENSP00000262319	ENSP00000402515
ENSP00000262320	ENSP00000262643
ENSP00000262320	ENSP00000264552
ENSP00000262320	ENSP00000266000
ENSP00000262320	ENSP00000268058
ENSP00000262320	ENSP00000283195
ENSP00000262320	ENSP00000288199
ENSP00000262320	ENSP00000288840
ENSP00000262320	ENSP00000295901
ENSP00000262320	ENSP00000296257
ENSP00000262320	ENSP00000298552
ENSP00000262320	ENSP00000299259
ENSP00000262320	ENSP00000302327
ENSP00000262320	ENSP00000310129
ENSP00000262320	ENSP00000311344
ENSP00000262320	ENSP00000311579
ENSP00000262320	ENSP00000313391
ENSP00000262320	ENSP00000314499
ENSP00000262320	ENSP00000325690
ENSP00000262320	ENSP00000329097
ENSP00000262320	ENSP00000329117
ENSP00000262320	ENSP00000337439
ENSP00000262320	ENSP00000343129
ENSP00000262320	ENSP00000344456
ENSP00000262320	ENSP00000345731
ENSP00000262320	ENSP00000347184
ENSP00000262320	ENSP00000351492
ENSP00000262320	ENSP00000352514
ENSP00000262320	ENSP00000354964
ENSP00000262320	ENSP00000357442
ENSP00000262320	ENSP00000359206
ENSP00000262320	ENSP00000367797
ENSP00000262320	ENSP00000375986
ENSP00000262320	ENSP00000382423
ENSP00000262320	ENSP00000392270
ENSP00000262345	ENSP00000264818
ENSP00000262345	ENSP00000303231
ENSP00000262345	ENSP00000314425
ENSP00000262345	ENSP00000314499
ENSP00000262366	ENSP00000264126
ENSP00000262366	ENSP00000307863
ENSP00000262366	ENSP00000311825

ENSP00000262366	ENSP00000313059
ENSP00000262366	ENSP00000325785
ENSP00000262366	ENSP00000339353
ENSP00000262366	ENSP00000344456
ENSP00000262366	ENSP00000351137
ENSP00000262366	ENSP00000359976
ENSP00000262367	ENSP00000262518
ENSP00000262367	ENSP00000262519
ENSP00000262367	ENSP00000262643
ENSP00000262367	ENSP00000262904
ENSP00000262367	ENSP00000263062
ENSP00000262367	ENSP00000263121
ENSP00000262367	ENSP00000263205
ENSP00000262367	ENSP00000264637
ENSP00000262367	ENSP00000264731
ENSP00000262367	ENSP00000264834
ENSP00000262367	ENSP00000265354
ENSP00000262367	ENSP00000265372
ENSP00000262367	ENSP00000266000
ENSP00000262367	ENSP00000267064
ENSP00000262367	ENSP00000268058
ENSP00000262367	ENSP00000272134
ENSP00000262367	ENSP00000272937
ENSP00000262367	ENSP00000278612
ENSP00000262367	ENSP00000282030
ENSP00000262367	ENSP00000282441
ENSP00000262367	ENSP00000282892
ENSP00000262367	ENSP00000283290
ENSP00000262367	ENSP00000283921
ENSP00000262367	ENSP00000287820
ENSP00000262367	ENSP00000290663
ENSP00000262367	ENSP00000291582
ENSP00000262367	ENSP00000292035
ENSP00000262367	ENSP00000292535
ENSP00000262367	ENSP00000294168
ENSP00000262367	ENSP00000294409
ENSP00000262367	ENSP00000295025
ENSP00000262367	ENSP00000296215
ENSP00000262367	ENSP00000298139
ENSP00000262367	ENSP00000302111
ENSP00000262367	ENSP00000302640
ENSP00000262367	ENSP00000303315
ENSP00000262367	ENSP00000304169
ENSP00000262367	ENSP00000305422
ENSP00000262367	ENSP00000306245
ENSP00000262367	ENSP00000308741

ENSP00000262367	ENSP00000313829
ENSP00000262367	ENSP00000314004
ENSP00000262367	ENSP00000314129
ENSP00000262367	ENSP00000314499
ENSP00000262367	ENSP00000314949
ENSP00000262367	ENSP00000315768
ENSP00000262367	ENSP00000316032
ENSP00000262367	ENSP00000316879
ENSP00000262367	ENSP00000319104
ENSP00000262367	ENSP00000319169
ENSP00000262367	ENSP00000321345
ENSP00000262367	ENSP00000325414
ENSP00000262367	ENSP00000325634
ENSP00000262367	ENSP00000325690
ENSP00000262367	ENSP00000325863
ENSP00000262367	ENSP00000326767
ENSP00000262367	ENSP00000326933
ENSP00000262367	ENSP00000327048
ENSP00000262367	ENSP00000328103
ENSP00000262367	ENSP00000329357
ENSP00000262367	ENSP00000329411
ENSP00000262367	ENSP00000331057
ENSP00000262367	ENSP00000334061
ENSP00000262367	ENSP00000334280
ENSP00000262367	ENSP00000336790
ENSP00000262367	ENSP00000338235
ENSP00000262367	ENSP00000339527
ENSP00000262367	ENSP00000340896
ENSP00000262367	ENSP00000341208
ENSP00000262367	ENSP00000341551
ENSP00000262367	ENSP00000342307
ENSP00000262367	ENSP00000343957
ENSP00000262367	ENSP00000344352
ENSP00000262367	ENSP00000344456
ENSP00000262367	ENSP00000344547
ENSP00000262367	ENSP00000344683
ENSP00000262367	ENSP00000345681
ENSP00000262367	ENSP00000346762
ENSP00000262367	ENSP00000346879
ENSP00000262367	ENSP00000346886
ENSP00000262367	ENSP00000347184
ENSP00000262367	ENSP00000348069
ENSP00000262367	ENSP00000348551
ENSP00000262367	ENSP00000348554
ENSP00000262367	ENSP00000348577
ENSP00000262367	ENSP00000348632

ENSP00000262367	ENSP00000350199
ENSP00000262367	ENSP00000350249
ENSP00000262367	ENSP00000350283
ENSP00000262367	ENSP00000350633
ENSP00000262367	ENSP00000350767
ENSP00000262367	ENSP00000351894
ENSP00000262367	ENSP00000351908
ENSP00000262367	ENSP00000352257
ENSP00000262367	ENSP00000352514
ENSP00000262367	ENSP00000352673
ENSP00000262367	ENSP00000353344
ENSP00000262367	ENSP00000354995
ENSP00000262367	ENSP00000355001
ENSP00000262367	ENSP00000355759
ENSP00000262367	ENSP00000356623
ENSP00000262367	ENSP00000357047
ENSP00000262367	ENSP00000357442
ENSP00000262367	ENSP00000358404
ENSP00000262367	ENSP00000359290
ENSP00000262367	ENSP00000359531
ENSP00000262367	ENSP00000361014
ENSP00000262367	ENSP00000362013
ENSP00000262367	ENSP00000362127
ENSP00000262367	ENSP00000362314
ENSP00000262367	ENSP00000362592
ENSP00000262367	ENSP00000362948
ENSP00000262367	ENSP00000363021
ENSP00000262367	ENSP00000363804
ENSP00000262367	ENSP00000364895
ENSP00000262367	ENSP00000364929
ENSP00000262367	ENSP00000365588
ENSP00000262367	ENSP00000365877
ENSP00000262367	ENSP00000366915
ENSP00000262367	ENSP00000370408
ENSP00000262367	ENSP00000371308
ENSP00000262367	ENSP00000372170
ENSP00000262367	ENSP00000373060
ENSP00000262367	ENSP00000376204
ENSP00000262367	ENSP00000376611
ENSP00000262367	ENSP00000379003
ENSP00000262367	ENSP00000380054
ENSP00000262367	ENSP00000380414
ENSP00000262367	ENSP00000381031
ENSP00000262367	ENSP00000381098
ENSP00000262367	ENSP00000381657
ENSP00000262367	ENSP00000384523

ENSP00000262367	ENSP00000386165
ENSP00000262367	ENSP00000386200
ENSP00000262367	ENSP00000391723
ENSP00000262367	ENSP00000391774
ENSP00000262367	ENSP00000394394
ENSP00000262367	ENSP00000395535
ENSP00000262367	ENSP00000396876
ENSP00000262367	ENSP00000399968
ENSP00000262367	ENSP00000417281
ENSP00000262374	ENSP00000296292
ENSP00000262374	ENSP00000315925
ENSP00000262374	ENSP00000380793
ENSP00000262374	ENSP00000387477
ENSP00000262374	ENSP00000417764
ENSP00000262375	ENSP00000264380
ENSP00000262375	ENSP00000265351
ENSP00000262375	ENSP00000266095
ENSP00000262375	ENSP00000274376
ENSP00000262375	ENSP00000276533
ENSP00000262375	ENSP00000282516
ENSP00000262375	ENSP00000290219
ENSP00000262375	ENSP00000294053
ENSP00000262375	ENSP00000297185
ENSP00000262375	ENSP00000299626
ENSP00000262375	ENSP00000301585
ENSP00000262375	ENSP00000329558
ENSP00000262375	ENSP00000331268
ENSP00000262375	ENSP00000332353
ENSP00000262375	ENSP00000340093
ENSP00000262375	ENSP00000343535
ENSP00000262375	ENSP00000350283
ENSP00000262375	ENSP00000360782
ENSP00000262375	ENSP00000364814
ENSP00000262375	ENSP00000365891
ENSP00000262375	ENSP00000366179
ENSP00000262375	ENSP00000368523
ENSP00000262375	ENSP00000368565
ENSP00000262375	ENSP00000387262
ENSP00000262375	ENSP00000394382
ENSP00000262375	ENSP00000396292
ENSP00000262375	ENSP00000404403
ENSP00000262384	ENSP00000310596
ENSP00000262384	ENSP00000419371
ENSP00000262394	ENSP00000282892
ENSP00000262394	ENSP00000284811
ENSP00000262394	ENSP00000298937

ENSP00000262394	ENSP00000301764
ENSP00000262394	ENSP00000304593
ENSP00000262394	ENSP00000356405
ENSP00000262394	ENSP00000357907
ENSP00000262394	ENSP00000360473
ENSP00000262394	ENSP00000386759
ENSP00000262394	ENSP00000396937
ENSP00000262406	ENSP00000334134
ENSP00000262406	ENSP00000344524
ENSP00000262406	ENSP00000360141
ENSP00000262419	ENSP00000264515
ENSP00000262419	ENSP00000264982
ENSP00000262419	ENSP00000278886
ENSP00000262419	ENSP00000293362
ENSP00000262419	ENSP00000326514
ENSP00000262419	ENSP00000360532
ENSP00000262419	ENSP00000362424
ENSP00000262428	ENSP00000269122
ENSP00000262428	ENSP00000276416
ENSP00000262428	ENSP00000300086
ENSP00000262428	ENSP00000314499
ENSP00000262428	ENSP00000314879
ENSP00000262428	ENSP00000326238
ENSP00000262428	ENSP00000334836
ENSP00000262428	ENSP00000348163
ENSP00000262428	ENSP00000348602
ENSP00000262428	ENSP00000349016
ENSP00000262428	ENSP00000359225
ENSP00000262428	ENSP00000386456
ENSP00000262430	ENSP00000282441
ENSP00000262430	ENSP00000360286
ENSP00000262430	ENSP00000361616
ENSP00000262455	ENSP00000265462
ENSP00000262455	ENSP00000278572
ENSP00000262455	ENSP00000292180
ENSP00000262455	ENSP00000299767
ENSP00000262455	ENSP00000300896
ENSP00000262455	ENSP00000318147
ENSP00000262455	ENSP00000364893
ENSP00000262455	ENSP00000366434
ENSP00000262455	ENSP00000379042
ENSP00000262457	ENSP00000299163
ENSP00000262457	ENSP00000311505
ENSP00000262457	ENSP00000313169
ENSP00000262457	ENSP00000314004
ENSP00000262457	ENSP00000331310

ENSP00000262457	ENSP00000338766
ENSP00000262457	ENSP00000356774
ENSP00000262460	ENSP00000263201
ENSP00000262460	ENSP00000265056
ENSP00000262460	ENSP00000265140
ENSP00000262460	ENSP00000265465
ENSP00000262460	ENSP00000276533
ENSP00000262460	ENSP00000294016
ENSP00000262460	ENSP00000296509
ENSP00000262460	ENSP00000298288
ENSP00000262460	ENSP00000312870
ENSP00000262460	ENSP00000322180
ENSP00000262460	ENSP00000322570
ENSP00000262460	ENSP00000340879
ENSP00000262460	ENSP00000344874
ENSP00000262460	ENSP00000351697
ENSP00000262460	ENSP00000353793
ENSP00000262460	ENSP00000354920
ENSP00000262460	ENSP00000356825
ENSP00000262460	ENSP00000368349
ENSP00000262460	ENSP00000368438
ENSP00000262460	ENSP00000370151
ENSP00000262460	ENSP00000380504
ENSP00000262460	ENSP00000384223
ENSP00000262460	ENSP00000386357
ENSP00000262460	ENSP00000401018
ENSP00000262460	ENSP00000413632
ENSP00000262460	ENSP00000419361
ENSP00000262461	ENSP00000348278
ENSP00000262507	ENSP00000265896
ENSP00000262507	ENSP00000288532
ENSP00000262507	ENSP00000300452
ENSP00000262507	ENSP00000310547
ENSP00000262507	ENSP00000322316
ENSP00000262507	ENSP00000333946
ENSP00000262507	ENSP00000342056
ENSP00000262507	ENSP00000355517
ENSP00000262507	ENSP00000355739
ENSP00000262510	ENSP00000336762
ENSP00000262518	ENSP00000262519
ENSP00000262518	ENSP00000263062
ENSP00000262518	ENSP00000263116
ENSP00000262518	ENSP00000263208
ENSP00000262518	ENSP00000263377
ENSP00000262518	ENSP00000263390
ENSP00000262518	ENSP00000263974

ENSP00000262518	ENSP00000264501
ENSP00000262518	ENSP00000264515
ENSP00000262518	ENSP00000265990
ENSP00000262518	ENSP00000266070
ENSP00000262518	ENSP00000267064
ENSP00000262518	ENSP00000267197
ENSP00000262518	ENSP00000269385
ENSP00000262518	ENSP00000269468
ENSP00000262518	ENSP00000276072
ENSP00000262518	ENSP00000280190
ENSP00000262518	ENSP00000283882
ENSP00000262518	ENSP00000286317
ENSP00000262518	ENSP00000286428
ENSP00000262518	ENSP00000287598
ENSP00000262518	ENSP00000291688
ENSP00000262518	ENSP00000295066
ENSP00000262518	ENSP00000296509
ENSP00000262518	ENSP00000296786
ENSP00000262518	ENSP00000299601
ENSP00000262518	ENSP00000300648
ENSP00000262518	ENSP00000301740
ENSP00000262518	ENSP00000302160
ENSP00000262518	ENSP00000302530
ENSP00000262518	ENSP00000304593
ENSP00000262518	ENSP00000306983
ENSP00000262518	ENSP00000307023
ENSP00000262518	ENSP00000307208
ENSP00000262518	ENSP00000310448
ENSP00000262518	ENSP00000310521
ENSP00000262518	ENSP00000310649
ENSP00000262518	ENSP00000312697
ENSP00000262518	ENSP00000312778
ENSP00000262518	ENSP00000313171
ENSP00000262518	ENSP00000314311
ENSP00000262518	ENSP00000315791
ENSP00000262518	ENSP00000320566
ENSP00000262518	ENSP00000322579
ENSP00000262518	ENSP00000323302
ENSP00000262518	ENSP00000323720
ENSP00000262518	ENSP00000325677
ENSP00000262518	ENSP00000325690
ENSP00000262518	ENSP00000325863
ENSP00000262518	ENSP00000329933
ENSP00000262518	ENSP00000331310
ENSP00000262518	ENSP00000334061
ENSP00000262518	ENSP00000334188

ENSP0000262518	ENSP00000336842
ENSP0000262518	ENSP00000337405
ENSP0000262518	ENSP00000339090
ENSP0000262518	ENSP00000340271
ENSP0000262518	ENSP00000340896
ENSP0000262518	ENSP00000341159
ENSP0000262518	ENSP00000347168
ENSP0000262518	ENSP00000347719
ENSP0000262518	ENSP00000349213
ENSP0000262518	ENSP00000350767
ENSP0000262518	ENSP00000352738
ENSP0000262518	ENSP00000353622
ENSP0000262518	ENSP00000355013
ENSP0000262518	ENSP00000356405
ENSP0000262518	ENSP00000356989
ENSP0000262518	ENSP00000357887
ENSP0000262518	ENSP00000359518
ENSP0000262518	ENSP00000360116
ENSP0000262518	ENSP00000360473
ENSP0000262518	ENSP00000360515
ENSP0000262518	ENSP00000362166
ENSP0000262518	ENSP00000362352
ENSP0000262518	ENSP00000362532
ENSP0000262518	ENSP00000362592
ENSP0000262518	ENSP00000362900
ENSP0000262518	ENSP00000363313
ENSP0000262518	ENSP00000363734
ENSP0000262518	ENSP00000364929
ENSP0000262518	ENSP00000366135
ENSP0000262518	ENSP00000366974
ENSP0000262518	ENSP00000367034
ENSP0000262518	ENSP00000369335
ENSP0000262518	ENSP00000369695
ENSP0000262518	ENSP00000370611
ENSP0000262518	ENSP00000372694
ENSP0000262518	ENSP00000374213
ENSP0000262518	ENSP00000374280
ENSP0000262518	ENSP00000374529
ENSP0000262518	ENSP00000375986
ENSP0000262518	ENSP00000376204
ENSP0000262518	ENSP00000379217
ENSP0000262518	ENSP00000380460
ENSP0000262518	ENSP00000382562
ENSP0000262518	ENSP00000386759
ENSP0000262518	ENSP00000390475
ENSP0000262518	ENSP00000399968

ENSP00000262518	ENSP00000402240
ENSP00000262518	ENSP00000406293
ENSP00000262518	ENSP00000412319
ENSP00000262518	ENSP00000415678
ENSP00000262519	ENSP00000263201
ENSP00000262519	ENSP00000264108
ENSP00000262519	ENSP00000264515
ENSP00000262519	ENSP00000264951
ENSP00000262519	ENSP00000278412
ENSP00000262519	ENSP00000278616
ENSP00000262519	ENSP00000280190
ENSP00000262519	ENSP00000283875
ENSP00000262519	ENSP00000286835
ENSP00000262519	ENSP00000290158
ENSP00000262519	ENSP00000295066
ENSP00000262519	ENSP00000296490
ENSP00000262519	ENSP00000296755
ENSP00000262519	ENSP00000297792
ENSP00000262519	ENSP00000300231
ENSP00000262519	ENSP00000300658
ENSP00000262519	ENSP00000304360
ENSP00000262519	ENSP00000304593
ENSP00000262519	ENSP00000307272
ENSP00000262519	ENSP00000309555
ENSP00000262519	ENSP00000311513
ENSP00000262519	ENSP00000312735
ENSP00000262519	ENSP00000312778
ENSP00000262519	ENSP00000314949
ENSP00000262519	ENSP00000317334
ENSP00000262519	ENSP00000319169
ENSP00000262519	ENSP00000320566
ENSP00000262519	ENSP00000325677
ENSP00000262519	ENSP00000329933
ENSP00000262519	ENSP00000340879
ENSP00000262519	ENSP00000340896
ENSP00000262519	ENSP00000341280
ENSP00000262519	ENSP00000343741
ENSP00000262519	ENSP00000348551
ENSP00000262519	ENSP00000349049
ENSP00000262519	ENSP00000351284
ENSP00000262519	ENSP00000352821
ENSP00000262519	ENSP00000353698
ENSP00000262519	ENSP00000353998
ENSP00000262519	ENSP00000354720
ENSP00000262519	ENSP00000354977
ENSP00000262519	ENSP00000355013

ENSP00000262519	ENSP00000356146
ENSP00000262519	ENSP00000357887
ENSP00000262519	ENSP00000360621
ENSP00000262519	ENSP00000362352
ENSP00000262519	ENSP00000366974
ENSP00000262519	ENSP00000369716
ENSP00000262519	ENSP00000370151
ENSP00000262519	ENSP00000370968
ENSP00000262519	ENSP00000371475
ENSP00000262519	ENSP00000374152
ENSP00000262519	ENSP00000374213
ENSP00000262519	ENSP00000374280
ENSP00000262519	ENSP00000376946
ENSP00000262519	ENSP00000377747
ENSP00000262519	ENSP00000381657
ENSP00000262519	ENSP00000382688
ENSP00000262519	ENSP00000386759
ENSP00000262519	ENSP00000390475
ENSP00000262519	ENSP00000414321
ENSP00000262544	ENSP00000276014
ENSP00000262544	ENSP00000294179
ENSP00000262544	ENSP00000296137
ENSP00000262544	ENSP00000299601
ENSP00000262544	ENSP00000299767
ENSP00000262544	ENSP00000311134
ENSP00000262544	ENSP00000316032
ENSP00000262544	ENSP00000329466
ENSP00000262544	ENSP00000342381
ENSP00000262544	ENSP00000342889
ENSP00000262544	ENSP00000344460
ENSP00000262544	ENSP00000345530
ENSP00000262544	ENSP00000354791
ENSP00000262544	ENSP00000356530
ENSP00000262544	ENSP00000357480
ENSP00000262544	ENSP00000362399
ENSP00000262544	ENSP00000363944
ENSP00000262544	ENSP00000364898
ENSP00000262544	ENSP00000371888
ENSP00000262544	ENSP00000377280
ENSP00000262544	ENSP00000379228
ENSP00000262544	ENSP00000390783
ENSP00000262544	ENSP00000401445
ENSP00000262554	ENSP00000264335
ENSP00000262554	ENSP00000282412
ENSP00000262554	ENSP00000297313
ENSP00000262554	ENSP00000304500

ENSP00000262554	ENSP00000326699
ENSP00000262554	ENSP00000337463
ENSP00000262554	ENSP00000341213
ENSP00000262554	ENSP00000344106
ENSP00000262554	ENSP00000348455
ENSP00000262554	ENSP00000354347
ENSP00000262554	ENSP00000354519
ENSP00000262554	ENSP00000355968
ENSP00000262554	ENSP00000361287
ENSP00000262554	ENSP00000364188
ENSP00000262554	ENSP00000364847
ENSP00000262554	ENSP00000366748
ENSP00000262554	ENSP00000370607
ENSP00000262554	ENSP00000382723
ENSP00000262554	ENSP00000389516
ENSP00000262554	ENSP00000395863
ENSP00000262554	ENSP00000401287
ENSP00000262554	ENSP00000405864
ENSP00000262554	ENSP00000412805
ENSP00000262577	ENSP00000291386
ENSP00000262577	ENSP00000315791
ENSP00000262577	ENSP00000317872
ENSP00000262577	ENSP00000339353
ENSP00000262577	ENSP00000348010
ENSP00000262577	ENSP00000355185
ENSP00000262577	ENSP00000358576
ENSP00000262577	ENSP00000367527
ENSP00000262577	ENSP00000398208
ENSP00000262584	ENSP00000262812
ENSP00000262584	ENSP00000263629
ENSP00000262584	ENSP00000263857
ENSP00000262584	ENSP00000264951
ENSP00000262584	ENSP00000264995
ENSP00000262584	ENSP00000265100
ENSP00000262584	ENSP00000265245
ENSP00000262584	ENSP00000268206
ENSP00000262584	ENSP00000268379
ENSP00000262584	ENSP00000268661
ENSP00000262584	ENSP00000270625
ENSP00000262584	ENSP00000272418
ENSP00000262584	ENSP00000274242
ENSP00000262584	ENSP00000274606
ENSP00000262584	ENSP00000277632
ENSP00000262584	ENSP00000278572
ENSP00000262584	ENSP00000287038
ENSP00000262584	ENSP00000289371

ENSP00000262584	ENSP00000294189
ENSP00000262584	ENSP00000296577
ENSP00000262584	ENSP00000296783
ENSP00000262584	ENSP00000296802
ENSP00000262584	ENSP00000296805
ENSP00000262584	ENSP00000297908
ENSP00000262584	ENSP00000298283
ENSP00000262584	ENSP00000298310
ENSP00000262584	ENSP00000302886
ENSP00000262584	ENSP00000305702
ENSP00000262584	ENSP00000306548
ENSP00000262584	ENSP00000307889
ENSP00000262584	ENSP00000309334
ENSP00000262584	ENSP00000309830
ENSP00000262584	ENSP00000311028
ENSP00000262584	ENSP00000311430
ENSP00000262584	ENSP00000314348
ENSP00000262584	ENSP00000314949
ENSP00000262584	ENSP00000317376
ENSP00000262584	ENSP00000318646
ENSP00000262584	ENSP00000319851
ENSP00000262584	ENSP00000322419
ENSP00000262584	ENSP00000329466
ENSP00000262584	ENSP00000333667
ENSP00000262584	ENSP00000333837
ENSP00000262584	ENSP00000336702
ENSP00000262584	ENSP00000337839
ENSP00000262584	ENSP00000338573
ENSP00000262584	ENSP00000339095
ENSP00000262584	ENSP00000339795
ENSP00000262584	ENSP00000341082
ENSP00000262584	ENSP00000341885
ENSP00000262584	ENSP00000343392
ENSP00000262584	ENSP00000343885
ENSP00000262584	ENSP00000345156
ENSP00000262584	ENSP00000346015
ENSP00000262584	ENSP00000346022
ENSP00000262584	ENSP00000346027
ENSP00000262584	ENSP00000346037
ENSP00000262584	ENSP00000346050
ENSP00000262584	ENSP00000346088
ENSP00000262584	ENSP00000346725
ENSP00000262584	ENSP00000347087
ENSP00000262584	ENSP00000354040
ENSP00000262584	ENSP00000354125
ENSP00000262584	ENSP00000354739

ENSP00000262584	ENSP00000356290
ENSP00000262584	ENSP00000358695
ENSP00000262584	ENSP00000359345
ENSP00000262584	ENSP00000361076
ENSP00000262584	ENSP00000362306
ENSP00000262584	ENSP00000362334
ENSP00000262584	ENSP00000362744
ENSP00000262584	ENSP00000363018
ENSP00000262584	ENSP00000363559
ENSP00000262584	ENSP00000363676
ENSP00000262584	ENSP00000364320
ENSP00000262584	ENSP00000365806
ENSP00000262584	ENSP00000366519
ENSP00000262584	ENSP00000369757
ENSP00000262584	ENSP00000370589
ENSP00000262584	ENSP00000373090
ENSP00000262584	ENSP00000375730
ENSP00000262584	ENSP00000377486
ENSP00000262584	ENSP00000377617
ENSP00000262584	ENSP00000377640
ENSP00000262584	ENSP00000377865
ENSP00000262584	ENSP00000378160
ENSP00000262584	ENSP00000379607
ENSP00000262584	ENSP00000379888
ENSP00000262584	ENSP00000380156
ENSP00000262584	ENSP00000380453
ENSP00000262584	ENSP00000380982
ENSP00000262584	ENSP00000382392
ENSP00000262584	ENSP00000386717
ENSP00000262584	ENSP00000388330
ENSP00000262584	ENSP00000389103
ENSP00000262584	ENSP00000391723
ENSP00000262584	ENSP00000393241
ENSP00000262584	ENSP00000393393
ENSP00000262584	ENSP00000394290
ENSP00000262584	ENSP00000401371
ENSP00000262584	ENSP00000402338
ENSP00000262584	ENSP00000403175
ENSP00000262584	ENSP00000403576
ENSP00000262584	ENSP00000403852
ENSP00000262584	ENSP00000409074
ENSP00000262584	ENSP00000409367
ENSP00000262584	ENSP00000411162
ENSP00000262584	ENSP00000412583
ENSP00000262584	ENSP00000414321
ENSP00000262584	ENSP00000416110

ENSP00000262584	ENSP00000417229
ENSP00000262584	ENSP00000419599
ENSP00000262585	ENSP00000282441
ENSP00000262607	ENSP00000264220
ENSP00000262607	ENSP00000286621
ENSP00000262607	ENSP00000301740
ENSP00000262607	ENSP00000307684
ENSP00000262607	ENSP00000367615
ENSP00000262608	ENSP00000263620
ENSP00000262608	ENSP00000263974
ENSP00000262608	ENSP00000264183
ENSP00000262608	ENSP00000264193
ENSP00000262608	ENSP00000265990
ENSP00000262608	ENSP00000269127
ENSP00000262608	ENSP00000276072
ENSP00000262608	ENSP00000279873
ENSP00000262608	ENSP00000297338
ENSP00000262608	ENSP00000303315
ENSP00000262608	ENSP00000306983
ENSP00000262608	ENSP00000307208
ENSP00000262608	ENSP00000316114
ENSP00000262608	ENSP00000322579
ENSP00000262608	ENSP00000323720
ENSP00000262608	ENSP00000334061
ENSP00000262608	ENSP00000335044
ENSP00000262608	ENSP00000337393
ENSP00000262608	ENSP00000347602
ENSP00000262608	ENSP00000349213
ENSP00000262608	ENSP00000350078
ENSP00000262608	ENSP00000350967
ENSP00000262608	ENSP00000353622
ENSP00000262608	ENSP00000354633
ENSP00000262608	ENSP00000354673
ENSP00000262608	ENSP00000355205
ENSP00000262608	ENSP00000356530
ENSP00000262608	ENSP00000359290
ENSP00000262608	ENSP00000360676
ENSP00000262608	ENSP00000361998
ENSP00000262608	ENSP00000362068
ENSP00000262608	ENSP00000363779
ENSP00000262608	ENSP00000369695
ENSP00000262608	ENSP00000373340
ENSP00000262608	ENSP00000377747
ENSP00000262608	ENSP00000379372
ENSP00000262608	ENSP00000381657
ENSP00000262608	ENSP00000392028

ENSP00000262608	ENSP00000401445
ENSP00000262629	ENSP00000311035
ENSP00000262629	ENSP00000362205
ENSP00000262629	ENSP00000364898
ENSP00000262629	ENSP00000371328
ENSP00000262630	ENSP0000289081
ENSP00000262630	ENSP00000338157
ENSP00000262630	ENSP00000345681
ENSP00000262633	ENSP00000309166
ENSP00000262633	ENSP00000315859
ENSP00000262633	ENSP00000352941
ENSP00000262633	ENSP00000363313
ENSP00000262633	ENSP00000365439
ENSP00000262643	ENSP00000262904
ENSP00000262643	ENSP00000263121
ENSP00000262643	ENSP00000264160
ENSP00000262643	ENSP00000265069
ENSP00000262643	ENSP00000265433
ENSP00000262643	ENSP00000265717
ENSP00000262643	ENSP00000267101
ENSP00000262643	ENSP00000267163
ENSP00000262643	ENSP00000268043
ENSP00000262643	ENSP00000269214
ENSP00000262643	ENSP00000269385
ENSP00000262643	ENSP00000269397
ENSP00000262643	ENSP00000274255
ENSP00000262643	ENSP00000276893
ENSP00000262643	ENSP00000277541
ENSP00000262643	ENSP00000278412
ENSP00000262643	ENSP00000278612
ENSP00000262643	ENSP00000278616
ENSP00000262643	ENSP00000281537
ENSP00000262643	ENSP00000281708
ENSP00000262643	ENSP00000286364
ENSP00000262643	ENSP00000287239
ENSP00000262643	ENSP00000287598
ENSP00000262643	ENSP00000295025
ENSP00000262643	ENSP00000296509
ENSP00000262643	ENSP00000297261
ENSP00000262643	ENSP00000298552
ENSP00000262643	ENSP00000301019
ENSP00000262643	ENSP00000302530
ENSP00000262643	ENSP00000306999
ENSP00000262643	ENSP00000309871
ENSP00000262643	ENSP00000312385
ENSP00000262643	ENSP00000313084

ENSP00000262643	ENSP00000313420
ENSP00000262643	ENSP00000319279
ENSP00000262643	ENSP00000320485
ENSP00000262643	ENSP00000321636
ENSP00000262643	ENSP00000322180
ENSP00000262643	ENSP00000325863
ENSP00000262643	ENSP00000330074
ENSP00000262643	ENSP00000332353
ENSP00000262643	ENSP00000335321
ENSP00000262643	ENSP00000339299
ENSP00000262643	ENSP00000340896
ENSP00000262643	ENSP00000341551
ENSP00000262643	ENSP00000342307
ENSP00000262643	ENSP00000343785
ENSP00000262643	ENSP00000344456
ENSP00000262643	ENSP00000344504
ENSP00000262643	ENSP00000345731
ENSP00000262643	ENSP00000347602
ENSP00000262643	ENSP00000348550
ENSP00000262643	ENSP00000348573
ENSP00000262643	ENSP00000350283
ENSP00000262643	ENSP00000350491
ENSP00000262643	ENSP00000351284
ENSP00000262643	ENSP00000351492
ENSP00000262643	ENSP00000352121
ENSP00000262643	ENSP00000353344
ENSP00000262643	ENSP00000353622
ENSP00000262643	ENSP00000354558
ENSP00000262643	ENSP00000355010
ENSP00000262643	ENSP00000355865
ENSP00000262643	ENSP00000356595
ENSP00000262643	ENSP00000356655
ENSP00000262643	ENSP00000357442
ENSP00000262643	ENSP00000358799
ENSP00000262643	ENSP00000359652
ENSP00000262643	ENSP00000363193
ENSP00000262643	ENSP00000363827
ENSP00000262643	ENSP00000364524
ENSP00000262643	ENSP00000364912
ENSP00000262643	ENSP00000366863
ENSP00000262643	ENSP00000367972
ENSP00000262643	ENSP00000368678
ENSP00000262643	ENSP00000368686
ENSP00000262643	ENSP00000368698
ENSP00000262643	ENSP00000369497
ENSP00000262643	ENSP00000369757

ENSP00000262643	ENSP00000376204
ENSP00000262643	ENSP00000377793
ENSP00000262643	ENSP00000377862
ENSP00000262643	ENSP00000378295
ENSP00000262643	ENSP00000380256
ENSP00000262643	ENSP00000383986
ENSP00000262643	ENSP00000414259
ENSP00000262644	ENSP00000263791
ENSP00000262644	ENSP00000285928
ENSP00000262644	ENSP00000286317
ENSP00000262644	ENSP00000292180
ENSP00000262644	ENSP00000304604
ENSP00000262644	ENSP00000304668
ENSP00000262644	ENSP00000314441
ENSP00000262644	ENSP00000343081
ENSP00000262644	ENSP00000344460
ENSP00000262644	ENSP00000345731
ENSP00000262644	ENSP00000346142
ENSP00000262644	ENSP00000347206
ENSP00000262644	ENSP00000348722
ENSP00000262644	ENSP00000367408
ENSP00000262644	ENSP00000370741
ENSP00000262648	ENSP00000307046
ENSP00000262710	ENSP00000315859
ENSP00000262710	ENSP00000326261
ENSP00000262710	ENSP00000326514
ENSP00000262710	ENSP00000329466
ENSP00000262710	ENSP00000337340
ENSP00000262710	ENSP00000377262
ENSP00000262710	ENSP00000379607
ENSP00000262715	ENSP00000264515
ENSP00000262715	ENSP00000264951
ENSP00000262715	ENSP00000266126
ENSP00000262715	ENSP00000282007
ENSP00000262715	ENSP00000290541
ENSP00000262715	ENSP00000304604
ENSP00000262715	ENSP00000309572
ENSP00000262715	ENSP00000310596
ENSP00000262715	ENSP00000318861
ENSP00000262715	ENSP00000331817
ENSP00000262715	ENSP00000339191
ENSP00000262715	ENSP00000340896
ENSP00000262715	ENSP00000356811
ENSP00000262715	ENSP00000356989
ENSP00000262715	ENSP00000360310
ENSP00000262715	ENSP00000361433

ENSP00000262726	ENSP00000263119
ENSP00000262726	ENSP00000311505
ENSP00000262726	ENSP00000340278
ENSP00000262726	ENSP00000352608
ENSP00000262726	ENSP00000353622
ENSP00000262726	ENSP00000377372
ENSP00000262726	ENSP00000378306
ENSP00000262764	ENSP00000265276
ENSP00000262764	ENSP00000265689
ENSP00000262764	ENSP00000272645
ENSP00000262764	ENSP00000273037
ENSP00000262764	ENSP00000284601
ENSP00000262764	ENSP00000307850
ENSP00000262764	ENSP00000355607
ENSP00000262764	ENSP00000358497
ENSP00000262764	ENSP00000369860
ENSP00000262764	ENSP00000375986
ENSP00000262764	ENSP00000376268
ENSP00000262764	ENSP00000387185
ENSP00000262764	ENSP00000396452
ENSP00000262765	ENSP00000265388
ENSP00000262765	ENSP00000290158
ENSP00000262765	ENSP00000291688
ENSP00000262765	ENSP00000307525
ENSP00000262765	ENSP00000308622
ENSP00000262765	ENSP00000312318
ENSP00000262765	ENSP00000316032
ENSP00000262765	ENSP00000323046
ENSP00000262765	ENSP00000336712
ENSP00000262765	ENSP00000346453
ENSP00000262765	ENSP00000348577
ENSP00000262765	ENSP00000361418
ENSP00000262765	ENSP00000361433
ENSP00000262765	ENSP00000364289
ENSP00000262765	ENSP00000366135
ENSP00000262765	ENSP00000369042
ENSP00000262765	ENSP00000371559
ENSP00000262776	ENSP00000263056
ENSP00000262776	ENSP00000304236
ENSP00000262776	ENSP00000329967
ENSP00000262776	ENSP00000346839
ENSP00000262776	ENSP00000355180
ENSP00000262776	ENSP00000379607
ENSP00000262794	ENSP00000262803
ENSP00000262794	ENSP00000264645
ENSP00000262794	ENSP00000268043

ENSP00000262794	ENSP00000280665
ENSP00000262794	ENSP00000286835
ENSP00000262794	ENSP00000292478
ENSP00000262794	ENSP00000307525
ENSP00000262794	ENSP00000311873
ENSP00000262794	ENSP00000312042
ENSP00000262794	ENSP00000325863
ENSP00000262794	ENSP00000343745
ENSP00000262794	ENSP00000349208
ENSP00000262794	ENSP00000356146
ENSP00000262794	ENSP00000358799
ENSP00000262794	ENSP00000361949
ENSP00000262794	ENSP00000363021
ENSP00000262794	ENSP00000363745
ENSP00000262794	ENSP00000366843
ENSP00000262794	ENSP00000369335
ENSP00000262794	ENSP00000370150
ENSP00000262794	ENSP00000373272
ENSP00000262803	ENSP00000263073
ENSP00000262803	ENSP00000263208
ENSP00000262803	ENSP00000263228
ENSP00000262803	ENSP00000264645
ENSP00000262803	ENSP00000264705
ENSP00000262803	ENSP00000264951
ENSP00000262803	ENSP00000268043
ENSP00000262803	ENSP00000268482
ENSP00000262803	ENSP00000270066
ENSP00000262803	ENSP00000276201
ENSP00000262803	ENSP00000278572
ENSP00000262803	ENSP00000286835
ENSP00000262803	ENSP00000294053
ENSP00000262803	ENSP00000296577
ENSP00000262803	ENSP00000300648
ENSP00000262803	ENSP00000300917
ENSP00000262803	ENSP00000302160
ENSP00000262803	ENSP00000304370
ENSP00000262803	ENSP00000307525
ENSP00000262803	ENSP00000309433
ENSP00000262803	ENSP00000309572
ENSP00000262803	ENSP00000310040
ENSP00000262803	ENSP00000310260
ENSP00000262803	ENSP00000310596
ENSP00000262803	ENSP00000314949
ENSP00000262803	ENSP00000315859
ENSP00000262803	ENSP00000316032
ENSP00000262803	ENSP00000319977

ENSP00000262803	ENSP00000320503
ENSP00000262803	ENSP00000325677
ENSP00000262803	ENSP00000325863
ENSP00000262803	ENSP00000326261
ENSP00000262803	ENSP00000326424
ENSP00000262803	ENSP00000326806
ENSP00000262803	ENSP00000327309
ENSP00000262803	ENSP00000329466
ENSP00000262803	ENSP00000329715
ENSP00000262803	ENSP00000331310
ENSP00000262803	ENSP00000331817
ENSP00000262803	ENSP00000331907
ENSP00000262803	ENSP00000334148
ENSP00000262803	ENSP00000336931
ENSP00000262803	ENSP00000340766
ENSP00000262803	ENSP00000343741
ENSP00000262803	ENSP00000343745
ENSP00000262803	ENSP00000346120
ENSP00000262803	ENSP00000347733
ENSP00000262803	ENSP00000348708
ENSP00000262803	ENSP00000350512
ENSP00000262803	ENSP00000351811
ENSP00000262803	ENSP00000353741
ENSP00000262803	ENSP00000353804
ENSP00000262803	ENSP00000354125
ENSP00000262803	ENSP00000354499
ENSP00000262803	ENSP00000354554
ENSP00000262803	ENSP00000355261
ENSP00000262803	ENSP00000356146
ENSP00000262803	ENSP00000356448
ENSP00000262803	ENSP00000356811
ENSP00000262803	ENSP00000357459
ENSP00000262803	ENSP00000358064
ENSP00000262803	ENSP00000358140
ENSP00000262803	ENSP00000358799
ENSP00000262803	ENSP00000359518
ENSP00000262803	ENSP00000360455
ENSP00000262803	ENSP00000360532
ENSP00000262803	ENSP00000360922
ENSP00000262803	ENSP00000361076
ENSP00000262803	ENSP00000361433
ENSP00000262803	ENSP00000362900
ENSP00000262803	ENSP00000362936
ENSP00000262803	ENSP00000363021
ENSP00000262803	ENSP00000363329
ENSP00000262803	ENSP00000363676

ENSP00000262803	ENSP00000363745
ENSP00000262803	ENSP00000364119
ENSP00000262803	ENSP00000364289
ENSP00000262803	ENSP00000364895
ENSP00000262803	ENSP00000364912
ENSP00000262803	ENSP00000366135
ENSP00000262803	ENSP00000366607
ENSP00000262803	ENSP00000366974
ENSP00000262803	ENSP00000367835
ENSP00000262803	ENSP00000369042
ENSP00000262803	ENSP00000369335
ENSP00000262803	ENSP00000369757
ENSP00000262803	ENSP00000370150
ENSP00000262803	ENSP00000373715
ENSP00000262803	ENSP00000374390
ENSP00000262803	ENSP00000377496
ENSP00000262803	ENSP00000379282
ENSP00000262803	ENSP00000379607
ENSP00000262803	ENSP00000380414
ENSP00000262803	ENSP00000387911
ENSP00000262803	ENSP00000401371
ENSP00000262803	ENSP00000402515
ENSP00000262803	ENSP00000417229
ENSP00000262803	ENSP00000417686
ENSP00000262812	ENSP00000264027
ENSP00000262812	ENSP00000264028
ENSP00000262812	ENSP00000265070
ENSP00000262812	ENSP00000265175
ENSP00000262812	ENSP00000268719
ENSP00000262812	ENSP00000270176
ENSP00000262812	ENSP00000283351
ENSP00000262812	ENSP00000297185
ENSP00000262812	ENSP00000305459
ENSP00000262812	ENSP00000306124
ENSP00000262812	ENSP00000310521
ENSP00000262812	ENSP00000314615
ENSP00000262812	ENSP00000321239
ENSP00000262812	ENSP00000325002
ENSP00000262812	ENSP00000326921
ENSP00000262812	ENSP00000329419
ENSP00000262812	ENSP00000331201
ENSP00000262812	ENSP00000340019
ENSP00000262812	ENSP00000345530
ENSP00000262812	ENSP00000345656
ENSP00000262812	ENSP00000347498
ENSP00000262812	ENSP00000349887

ENSP00000262812	ENSP00000349970
ENSP00000262812	ENSP00000352738
ENSP00000262812	ENSP00000356405
ENSP00000262812	ENSP00000357048
ENSP00000262812	ENSP00000359552
ENSP00000262812	ENSP00000360798
ENSP00000262812	ENSP00000361686
ENSP00000262812	ENSP00000364188
ENSP00000262812	ENSP00000366974
ENSP00000262812	ENSP00000379228
ENSP00000262812	ENSP00000396704
ENSP00000262812	ENSP00000402240
ENSP00000262843	ENSP00000343129
ENSP00000262843	ENSP00000348784
ENSP00000262843	ENSP00000360116
ENSP00000262843	ENSP00000361276
ENSP00000262843	ENSP00000363095
ENSP00000262843	ENSP00000374455
ENSP00000262844	ENSP00000340019
ENSP00000262844	ENSP00000363559
ENSP00000262850	ENSP00000264276
ENSP00000262850	ENSP00000265537
ENSP00000262850	ENSP00000270776
ENSP00000262850	ENSP00000278616
ENSP00000262850	ENSP00000278671
ENSP00000262850	ENSP00000285046
ENSP00000262850	ENSP00000290429
ENSP00000262850	ENSP00000296137
ENSP00000262850	ENSP00000296328
ENSP00000262850	ENSP00000302478
ENSP00000262850	ENSP00000311430
ENSP00000262850	ENSP00000314949
ENSP00000262850	ENSP00000317955
ENSP00000262850	ENSP00000326534
ENSP00000262850	ENSP00000327821
ENSP00000262850	ENSP00000334061
ENSP00000262850	ENSP00000343657
ENSP00000262850	ENSP00000343701
ENSP00000262850	ENSP00000344446
ENSP00000262850	ENSP00000344456
ENSP00000262850	ENSP00000345144
ENSP00000262850	ENSP00000347733
ENSP00000262850	ENSP00000348577
ENSP00000262850	ENSP00000352121
ENSP00000262850	ENSP00000353393
ENSP00000262850	ENSP00000354558

ENSP00000262850	ENSP00000355205
ENSP00000262850	ENSP00000356548
ENSP00000262850	ENSP00000357288
ENSP00000262850	ENSP00000358423
ENSP00000262850	ENSP00000363162
ENSP00000262850	ENSP00000365811
ENSP00000262850	ENSP00000366135
ENSP00000262850	ENSP00000366843
ENSP00000262850	ENSP00000367079
ENSP00000262850	ENSP00000367766
ENSP00000262850	ENSP00000370517
ENSP00000262850	ENSP00000377954
ENSP00000262850	ENSP00000390158
ENSP00000262850	ENSP00000415203
ENSP00000262854	ENSP00000264028
ENSP00000262854	ENSP00000265717
ENSP00000262854	ENSP00000265990
ENSP00000262854	ENSP00000278616
ENSP00000262854	ENSP00000294119
ENSP00000262854	ENSP00000294507
ENSP00000262854	ENSP00000299767
ENSP00000262854	ENSP00000300648
ENSP00000262854	ENSP00000307684
ENSP00000262854	ENSP00000309031
ENSP00000262854	ENSP00000326119
ENSP00000262854	ENSP00000328940
ENSP00000262854	ENSP00000329466
ENSP00000262854	ENSP00000331817
ENSP00000262854	ENSP00000337839
ENSP00000262854	ENSP00000343535
ENSP00000262854	ENSP00000344683
ENSP00000262854	ENSP00000346067
ENSP00000262854	ENSP00000355153
ENSP00000262854	ENSP00000358022
ENSP00000262854	ENSP00000358854
ENSP00000262854	ENSP00000359837
ENSP00000262854	ENSP00000360286
ENSP00000262854	ENSP00000360532
ENSP00000262854	ENSP00000365891
ENSP00000262854	ENSP00000366135
ENSP00000262854	ENSP00000366179
ENSP00000262854	ENSP00000366974
ENSP00000262854	ENSP00000367208
ENSP00000262854	ENSP00000368438
ENSP00000262854	ENSP00000368565
ENSP00000262854	ENSP00000387170

ENSP00000262878	ENSP00000264093
ENSP00000262878	ENSP00000301764
ENSP00000262878	ENSP00000322706
ENSP00000262878	ENSP00000376445
ENSP00000262878	ENSP00000386722
ENSP00000262879	ENSP00000263642
ENSP00000262890	ENSP00000263925
ENSP00000262890	ENSP00000276603
ENSP00000262890	ENSP00000300086
ENSP00000262890	ENSP00000304102
ENSP00000262890	ENSP00000323687
ENSP00000262890	ENSP00000324491
ENSP00000262890	ENSP00000370410
ENSP00000262890	ENSP00000378306
ENSP00000262895	ENSP00000300417
ENSP00000262895	ENSP00000347206
ENSP00000262895	ENSP00000349892
ENSP00000262901	ENSP00000266058
ENSP00000262901	ENSP00000281043
ENSP00000262901	ENSP00000351407
ENSP00000262901	ENSP00000381840
ENSP00000262903	ENSP00000265990
ENSP00000262903	ENSP00000300648
ENSP00000262903	ENSP00000307684
ENSP00000262903	ENSP00000329411
ENSP00000262903	ENSP00000331817
ENSP00000262903	ENSP00000348010
ENSP00000262903	ENSP00000359837
ENSP00000262903	ENSP00000360286
ENSP00000262903	ENSP00000366135
ENSP00000262903	ENSP00000366179
ENSP00000262903	ENSP00000367208
ENSP00000262903	ENSP00000368565
ENSP00000262903	ENSP00000387170
ENSP00000262904	ENSP00000263384
ENSP00000262904	ENSP00000264161
ENSP00000262904	ENSP00000267064
ENSP00000262904	ENSP00000267101
ENSP00000262904	ENSP00000267163
ENSP00000262904	ENSP00000282050
ENSP00000262904	ENSP00000304668
ENSP00000262904	ENSP00000309555
ENSP00000262904	ENSP00000311005
ENSP00000262904	ENSP00000313059
ENSP00000262904	ENSP00000314129
ENSP00000262904	ENSP00000320485

ENSP00000262904	ENSP00000329357
ENSP00000262904	ENSP00000332995
ENSP00000262904	ENSP00000345681
ENSP00000262904	ENSP00000346227
ENSP00000262904	ENSP00000353344
ENSP00000262904	ENSP00000362208
ENSP00000262904	ENSP00000362592
ENSP00000262904	ENSP00000363827
ENSP00000262904	ENSP00000364912
ENSP00000262904	ENSP00000367079
ENSP00000262904	ENSP00000368438
ENSP00000262904	ENSP00000368686
ENSP00000262904	ENSP00000378529
ENSP00000262904	ENSP00000419494
ENSP00000262919	ENSP00000350512
ENSP00000262919	ENSP00000364092
ENSP00000262947	ENSP00000363559
ENSP00000262962	ENSP00000264233
ENSP00000262962	ENSP00000268206
ENSP00000262962	ENSP00000268482
ENSP00000262962	ENSP00000291688
ENSP00000262962	ENSP00000295488
ENSP00000262962	ENSP00000296805
ENSP00000262962	ENSP00000298532
ENSP00000262962	ENSP00000301740
ENSP00000262962	ENSP00000303191
ENSP00000262962	ENSP00000304350
ENSP00000262962	ENSP00000304370
ENSP00000262962	ENSP00000326806
ENSP00000262962	ENSP00000331907
ENSP00000262962	ENSP00000332103
ENSP00000262962	ENSP00000334148
ENSP00000262962	ENSP00000351137
ENSP00000262962	ENSP00000359454
ENSP00000262962	ENSP00000360532
ENSP00000262962	ENSP00000361577
ENSP00000262962	ENSP00000362166
ENSP00000262962	ENSP00000364212
ENSP00000262962	ENSP00000364289
ENSP00000262962	ENSP00000366557
ENSP00000262962	ENSP00000371888
ENSP00000262962	ENSP00000376973
ENSP00000262962	ENSP00000387006
ENSP00000262962	ENSP00000412566
ENSP00000262962	ENSP00000416583
ENSP00000262966	ENSP00000290541

ENSP00000262966	ENSP00000298852
ENSP00000262966	ENSP00000299206
ENSP00000262966	ENSP00000309198
ENSP00000262966	ENSP00000310129
ENSP00000262966	ENSP00000310590
ENSP00000262966	ENSP00000337501
ENSP00000262966	ENSP00000338703
ENSP00000262966	ENSP00000339095
ENSP00000262966	ENSP00000347997
ENSP00000262966	ENSP00000354673
ENSP00000262966	ENSP00000354883
ENSP00000262966	ENSP00000360216
ENSP00000262966	ENSP00000361699
ENSP00000262966	ENSP00000362334
ENSP00000262966	ENSP00000369531
ENSP00000262966	ENSP00000384211
ENSP00000262966	ENSP00000386212
ENSP00000262966	ENSP00000401508
ENSP00000262966	ENSP00000417748
ENSP00000262982	ENSP00000265351
ENSP00000262982	ENSP00000276326
ENSP00000262982	ENSP00000280346
ENSP00000262982	ENSP00000283195
ENSP00000262982	ENSP00000290158
ENSP00000262982	ENSP00000290649
ENSP00000262982	ENSP00000291688
ENSP00000262982	ENSP00000296273
ENSP00000262982	ENSP00000298910
ENSP00000262982	ENSP00000299601
ENSP00000262982	ENSP00000300574
ENSP00000262982	ENSP00000307939
ENSP00000262982	ENSP00000310129
ENSP00000262982	ENSP00000311505
ENSP00000262982	ENSP00000325448
ENSP00000262982	ENSP00000327213
ENSP00000262982	ENSP00000328103
ENSP00000262982	ENSP00000329029
ENSP00000262982	ENSP00000329466
ENSP00000262982	ENSP00000337405
ENSP00000262982	ENSP00000339881
ENSP00000262982	ENSP00000340823
ENSP00000262982	ENSP00000343701
ENSP00000262982	ENSP00000345656
ENSP00000262982	ENSP00000346453
ENSP00000262982	ENSP00000351682
ENSP00000262982	ENSP00000357748

ENSP00000262982	ENSP00000359531
ENSP00000262982	ENSP00000359688
ENSP00000262982	ENSP00000360473
ENSP00000262982	ENSP00000360532
ENSP00000262982	ENSP00000364289
ENSP00000262982	ENSP00000364320
ENSP00000262982	ENSP00000366135
ENSP00000262982	ENSP00000369042
ENSP00000262982	ENSP00000369335
ENSP00000262982	ENSP00000369411
ENSP00000262982	ENSP00000369757
ENSP00000262982	ENSP00000370151
ENSP00000262982	ENSP00000376973
ENSP00000262982	ENSP00000377280
ENSP00000262982	ENSP00000380460
ENSP00000262982	ENSP00000380982
ENSP00000262982	ENSP00000381863
ENSP00000262990	ENSP00000309606
ENSP00000262990	ENSP00000361010
ENSP00000262995	ENSP00000274376
ENSP00000262995	ENSP00000275635
ENSP00000262995	ENSP00000281171
ENSP00000262995	ENSP00000296474
ENSP00000262995	ENSP00000300574
ENSP00000262995	ENSP00000303554
ENSP00000262995	ENSP00000308774
ENSP00000262995	ENSP00000310561
ENSP00000262995	ENSP00000327077
ENSP00000262995	ENSP00000330408
ENSP00000262995	ENSP00000339186
ENSP00000262995	ENSP00000340944
ENSP00000262995	ENSP00000347942
ENSP00000262995	ENSP00000348081
ENSP00000262995	ENSP00000348307
ENSP00000262995	ENSP00000352121
ENSP00000262995	ENSP00000354927
ENSP00000262995	ENSP00000356346
ENSP00000262995	ENSP00000357494
ENSP00000262995	ENSP00000360916
ENSP00000262995	ENSP00000361202
ENSP00000262995	ENSP00000364893
ENSP00000262995	ENSP00000365016
ENSP00000262995	ENSP00000378368
ENSP00000262995	ENSP00000384675
ENSP00000263026	ENSP00000359206
ENSP00000263026	ENSP00000380184

ENSP00000263036	ENSP00000306245
ENSP00000263036	ENSP00000315112
ENSP00000263036	ENSP00000317891
ENSP00000263036	ENSP00000324573
ENSP00000263036	ENSP00000329411
ENSP00000263036	ENSP00000329967
ENSP00000263036	ENSP00000347184
ENSP00000263036	ENSP00000363089
ENSP00000263036	ENSP00000371875
ENSP00000263036	ENSP00000379612
ENSP00000263036	ENSP00000380019
ENSP00000263036	ENSP00000380054
ENSP00000263036	ENSP00000381698
ENSP00000263036	ENSP00000382423
ENSP00000263036	ENSP00000384391
ENSP00000263038	ENSP00000264108
ENSP00000263038	ENSP00000264169
ENSP00000263038	ENSP00000268717
ENSP00000263038	ENSP00000270708
ENSP00000263038	ENSP00000353393
ENSP00000263038	ENSP00000362987
ENSP00000263038	ENSP00000364578
ENSP00000263043	ENSP00000267803
ENSP00000263043	ENSP00000276014
ENSP00000263043	ENSP00000283195
ENSP00000263043	ENSP00000302114
ENSP00000263043	ENSP00000304169
ENSP00000263043	ENSP00000304226
ENSP00000263043	ENSP00000307272
ENSP00000263043	ENSP00000310701
ENSP00000263043	ENSP00000311083
ENSP00000263043	ENSP00000316845
ENSP00000263043	ENSP00000346809
ENSP00000263043	ENSP00000354927
ENSP00000263043	ENSP00000355866
ENSP00000263043	ENSP00000362110
ENSP00000263043	ENSP00000363021
ENSP00000263043	ENSP00000365005
ENSP00000263043	ENSP00000372023
ENSP00000263043	ENSP00000381932
ENSP00000263043	ENSP00000382423
ENSP00000263043	ENSP00000384675
ENSP00000263043	ENSP00000400591
ENSP00000263056	ENSP00000269122
ENSP00000263056	ENSP00000276416
ENSP00000263056	ENSP00000288976

ENSP00000263056	ENSP00000295025
ENSP00000263056	ENSP00000297185
ENSP00000263056	ENSP00000305958
ENSP00000263056	ENSP00000321203
ENSP00000263056	ENSP00000335357
ENSP00000263056	ENSP00000339952
ENSP00000263056	ENSP00000348602
ENSP00000263056	ENSP00000351113
ENSP00000263056	ENSP00000354916
ENSP00000263056	ENSP00000363708
ENSP00000263056	ENSP00000364893
ENSP00000263062	ENSP00000263360
ENSP00000263062	ENSP00000265354
ENSP00000263062	ENSP00000265990
ENSP00000263062	ENSP00000283290
ENSP00000263062	ENSP00000303315
ENSP00000263062	ENSP00000306983
ENSP00000263062	ENSP00000312697
ENSP00000263062	ENSP00000320566
ENSP00000263062	ENSP00000331310
ENSP00000263062	ENSP00000347733
ENSP00000263062	ENSP00000355205
ENSP00000263062	ENSP00000357887
ENSP00000263062	ENSP00000359518
ENSP00000263062	ENSP00000362166
ENSP00000263062	ENSP00000366974
ENSP00000263062	ENSP00000369695
ENSP00000263062	ENSP00000374213
ENSP00000263073	ENSP00000264951
ENSP00000263073	ENSP00000278616
ENSP00000263073	ENSP00000280665
ENSP00000263073	ENSP00000298746
ENSP00000263073	ENSP00000309572
ENSP00000263073	ENSP00000325863
ENSP00000263073	ENSP00000326806
ENSP00000263073	ENSP00000332103
ENSP00000263073	ENSP00000336842
ENSP00000263073	ENSP00000348708
ENSP00000263073	ENSP00000351284
ENSP00000263073	ENSP00000352257
ENSP00000263073	ENSP00000354673
ENSP00000263073	ENSP00000355261
ENSP00000263073	ENSP00000358563
ENSP00000263073	ENSP00000361577
ENSP00000263073	ENSP00000380504
ENSP00000263073	ENSP00000412566

ENSP00000263083	ENSP00000264731
ENSP00000263083	ENSP00000268206
ENSP00000263083	ENSP00000296805
ENSP00000263083	ENSP00000302955
ENSP00000263083	ENSP00000321320
ENSP00000263083	ENSP00000334564
ENSP00000263083	ENSP00000343885
ENSP00000263083	ENSP00000348877
ENSP00000263083	ENSP00000361465
ENSP00000263083	ENSP00000419599
ENSP00000263092	ENSP00000308546
ENSP00000263102	ENSP00000311344
ENSP00000263102	ENSP00000340271
ENSP00000263102	ENSP00000343535
ENSP00000263116	ENSP00000264027
ENSP00000263116	ENSP00000264501
ENSP00000263116	ENSP00000265960
ENSP00000263116	ENSP00000272322
ENSP00000263116	ENSP00000273398
ENSP00000263116	ENSP00000294179
ENSP00000263116	ENSP00000299138
ENSP00000263116	ENSP00000299601
ENSP00000263116	ENSP00000300584
ENSP00000263116	ENSP00000304593
ENSP00000263116	ENSP00000305459
ENSP00000263116	ENSP00000308496
ENSP00000263116	ENSP00000314615
ENSP00000263116	ENSP00000318604
ENSP00000263116	ENSP00000321711
ENSP00000263116	ENSP00000326534
ENSP00000263116	ENSP00000328079
ENSP00000263116	ENSP00000328524
ENSP00000263116	ENSP00000329419
ENSP00000263116	ENSP00000336724
ENSP00000263116	ENSP00000341551
ENSP00000263116	ENSP00000341662
ENSP00000263116	ENSP00000349959
ENSP00000263116	ENSP00000354158
ENSP00000263116	ENSP00000354486
ENSP00000263116	ENSP00000354522
ENSP00000263116	ENSP00000356405
ENSP00000263116	ENSP00000357887
ENSP00000263116	ENSP00000359518
ENSP00000263116	ENSP00000368959
ENSP00000263116	ENSP00000371888
ENSP00000263116	ENSP00000372697

ENSP00000263116	ENSP00000374213
ENSP00000263116	ENSP00000376871
ENSP00000263116	ENSP00000377769
ENSP00000263116	ENSP00000381293
ENSP00000263116	ENSP00000386918
ENSP00000263116	ENSP00000389399
ENSP00000263116	ENSP00000390783
ENSP00000263116	ENSP00000390831
ENSP00000263116	ENSP00000391197
ENSP00000263116	ENSP00000394071
ENSP00000263116	ENSP00000397441
ENSP00000263116	ENSP00000401435
ENSP00000263116	ENSP00000402240
ENSP00000263116	ENSP00000402515
ENSP00000263116	ENSP00000402935
ENSP00000263116	ENSP00000404016
ENSP00000263116	ENSP00000406988
ENSP00000263116	ENSP00000409952
ENSP00000263116	ENSP00000411197
ENSP00000263119	ENSP00000263208
ENSP00000263119	ENSP00000271555
ENSP00000263119	ENSP00000314949
ENSP00000263119	ENSP00000348602
ENSP00000263119	ENSP00000350199
ENSP00000263119	ENSP00000353622
ENSP00000263119	ENSP00000365877
ENSP00000263119	ENSP00000378306
ENSP00000263119	ENSP00000417281
ENSP00000263121	ENSP00000263208
ENSP00000263121	ENSP00000263736
ENSP00000263121	ENSP00000264834
ENSP00000263121	ENSP00000265354
ENSP00000263121	ENSP00000265990
ENSP00000263121	ENSP00000266126
ENSP00000263121	ENSP00000267064
ENSP00000263121	ENSP00000267163
ENSP00000263121	ENSP00000271555
ENSP00000263121	ENSP00000278412
ENSP00000263121	ENSP00000278616
ENSP00000263121	ENSP00000280190
ENSP00000263121	ENSP00000282388
ENSP00000263121	ENSP00000282892
ENSP00000263121	ENSP00000283109
ENSP00000263121	ENSP00000284957
ENSP00000263121	ENSP00000285021
ENSP00000263121	ENSP00000286317

ENSP00000263121	ENSP00000287878
ENSP00000263121	ENSP00000290663
ENSP00000263121	ENSP00000295321
ENSP00000263121	ENSP00000297325
ENSP00000263121	ENSP00000299601
ENSP00000263121	ENSP00000303315
ENSP00000263121	ENSP00000304292
ENSP00000263121	ENSP00000305422
ENSP00000263121	ENSP00000306245
ENSP00000263121	ENSP00000309433
ENSP00000263121	ENSP00000310649
ENSP00000263121	ENSP00000312778
ENSP00000263121	ENSP00000314949
ENSP00000263121	ENSP00000319104
ENSP00000263121	ENSP00000319169
ENSP00000263121	ENSP00000320485
ENSP00000263121	ENSP00000320566
ENSP00000263121	ENSP00000323720
ENSP00000263121	ENSP00000323967
ENSP00000263121	ENSP00000325369
ENSP00000263121	ENSP00000325677
ENSP00000263121	ENSP00000325690
ENSP00000263121	ENSP00000326806
ENSP00000263121	ENSP00000329357
ENSP00000263121	ENSP00000334130
ENSP00000263121	ENSP00000334280
ENSP00000263121	ENSP00000335044
ENSP00000263121	ENSP00000340409
ENSP00000263121	ENSP00000340836
ENSP00000263121	ENSP00000341243
ENSP00000263121	ENSP00000341805
ENSP00000263121	ENSP00000342434
ENSP00000263121	ENSP00000344537
ENSP00000263121	ENSP00000345702
ENSP00000263121	ENSP00000346566
ENSP00000263121	ENSP00000347168
ENSP00000263121	ENSP00000348234
ENSP00000263121	ENSP00000349213
ENSP00000263121	ENSP00000350283
ENSP00000263121	ENSP00000350767
ENSP00000263121	ENSP00000351284
ENSP00000263121	ENSP00000351596
ENSP00000263121	ENSP00000351947
ENSP00000263121	ENSP00000353344
ENSP00000263121	ENSP00000353622
ENSP00000263121	ENSP00000354723

ENSP00000263121	ENSP00000356263
ENSP00000263121	ENSP00000356405
ENSP00000263121	ENSP00000356846
ENSP00000263121	ENSP00000360532
ENSP00000263121	ENSP00000360621
ENSP00000263121	ENSP00000362592
ENSP00000263121	ENSP00000362948
ENSP00000263121	ENSP00000364929
ENSP00000263121	ENSP00000366974
ENSP00000263121	ENSP00000367034
ENSP00000263121	ENSP00000370003
ENSP00000263121	ENSP00000370408
ENSP00000263121	ENSP00000377665
ENSP00000263121	ENSP00000379607
ENSP00000263121	ENSP00000380718
ENSP00000263121	ENSP00000384015
ENSP00000263121	ENSP00000391774
ENSP00000263121	ENSP00000392028
ENSP00000263121	ENSP00000394869
ENSP00000263121	ENSP00000395535
ENSP00000263121	ENSP00000402240
ENSP00000263121	ENSP00000417281
ENSP00000263121	ENSP00000420588
ENSP00000263150	ENSP00000291688
ENSP00000263150	ENSP00000295025
ENSP00000263150	ENSP00000316454
ENSP00000263150	ENSP00000341957
ENSP00000263150	ENSP00000381031
ENSP00000263174	ENSP00000380921
ENSP00000263181	ENSP00000263710
ENSP00000263181	ENSP00000265104
ENSP00000263181	ENSP00000271452
ENSP00000263181	ENSP00000286428
ENSP00000263181	ENSP00000287598
ENSP00000263181	ENSP00000296509
ENSP00000263181	ENSP00000300231
ENSP00000263181	ENSP00000300249
ENSP00000263181	ENSP00000301019
ENSP00000263181	ENSP00000301332
ENSP00000263181	ENSP00000302530
ENSP00000263181	ENSP00000322180
ENSP00000263181	ENSP00000327009
ENSP00000263181	ENSP00000332592
ENSP00000263181	ENSP00000346566
ENSP00000263181	ENSP00000347719
ENSP00000263181	ENSP00000348965

ENSP00000263181	ENSP00000351665
ENSP00000263181	ENSP00000353793
ENSP00000263181	ENSP00000354158
ENSP00000263181	ENSP00000355922
ENSP00000263181	ENSP00000356579
ENSP00000263181	ENSP00000357643
ENSP00000263181	ENSP00000360473
ENSP00000263181	ENSP00000363734
ENSP00000263181	ENSP00000368976
ENSP00000263181	ENSP00000372023
ENSP00000263181	ENSP00000372694
ENSP00000263181	ENSP00000378529
ENSP00000263181	ENSP00000382562
ENSP00000263181	ENSP00000402084
ENSP00000263181	ENSP00000412319
ENSP00000263181	ENSP00000414402
ENSP00000263181	ENSP00000415678
ENSP00000263187	ENSP00000263642
ENSP00000263187	ENSP00000263795
ENSP00000263187	ENSP00000264233
ENSP00000263187	ENSP00000265304
ENSP00000263187	ENSP00000267430
ENSP00000263187	ENSP00000285021
ENSP00000263187	ENSP00000286428
ENSP00000263187	ENSP00000306561
ENSP00000263187	ENSP00000307853
ENSP00000263187	ENSP00000310520
ENSP00000263187	ENSP00000311873
ENSP00000263187	ENSP00000321636
ENSP00000263187	ENSP00000322570
ENSP00000263187	ENSP00000348020
ENSP00000263187	ENSP00000351284
ENSP00000263187	ENSP00000351697
ENSP00000263187	ENSP00000353332
ENSP00000263187	ENSP00000354522
ENSP00000263187	ENSP00000360268
ENSP00000263187	ENSP00000360310
ENSP00000263187	ENSP00000361310
ENSP00000263187	ENSP00000363021
ENSP00000263187	ENSP00000364289
ENSP00000263187	ENSP00000368349
ENSP00000263187	ENSP00000368438
ENSP00000263187	ENSP00000369335
ENSP00000263187	ENSP00000389709
ENSP00000263187	ENSP00000397830
ENSP00000263196	ENSP00000300574

ENSP00000263196	ENSP00000340671
ENSP00000263201	ENSP00000263208
ENSP00000263201	ENSP00000263857
ENSP00000263201	ENSP00000264108
ENSP00000263201	ENSP00000264233
ENSP00000263201	ENSP00000265056
ENSP00000263201	ENSP00000265354
ENSP00000263201	ENSP00000265728
ENSP00000263201	ENSP00000267197
ENSP00000263201	ENSP00000268043
ENSP00000263201	ENSP00000276533
ENSP00000263201	ENSP00000278616
ENSP00000263201	ENSP00000281708
ENSP00000263201	ENSP00000295897
ENSP00000263201	ENSP00000296273
ENSP00000263201	ENSP00000296328
ENSP00000263201	ENSP00000296509
ENSP00000263201	ENSP00000297431
ENSP00000263201	ENSP00000300738
ENSP00000263201	ENSP00000310596
ENSP00000263201	ENSP00000313171
ENSP00000263201	ENSP00000313420
ENSP00000263201	ENSP00000314949
ENSP00000263201	ENSP00000317039
ENSP00000263201	ENSP00000321636
ENSP00000263201	ENSP00000322180
ENSP00000263201	ENSP00000323663
ENSP00000263201	ENSP00000334061
ENSP00000263201	ENSP00000336606
ENSP00000263201	ENSP00000340019
ENSP00000263201	ENSP00000340879
ENSP00000263201	ENSP00000343392
ENSP00000263201	ENSP00000343701
ENSP00000263201	ENSP00000343741
ENSP00000263201	ENSP00000351284
ENSP00000263201	ENSP00000352121
ENSP00000263201	ENSP00000353622
ENSP00000263201	ENSP00000353793
ENSP00000263201	ENSP00000354522
ENSP00000263201	ENSP00000354558
ENSP00000263201	ENSP00000355153
ENSP00000263201	ENSP00000360621
ENSP00000263201	ENSP00000365811
ENSP00000263201	ENSP00000365877
ENSP00000263201	ENSP00000366974
ENSP00000263201	ENSP00000368349

ENSP00000263201	ENSP00000369411
ENSP00000263201	ENSP00000370151
ENSP00000263201	ENSP00000371475
ENSP00000263201	ENSP00000372023
ENSP00000263201	ENSP00000380504
ENSP00000263201	ENSP00000382562
ENSP00000263201	ENSP00000384109
ENSP00000263201	ENSP00000397435
ENSP00000263201	ENSP00000401018
ENSP00000263201	ENSP00000402515
ENSP00000263202	ENSP00000263966
ENSP00000263202	ENSP00000264864
ENSP00000263202	ENSP00000270225
ENSP00000263202	ENSP00000273067
ENSP00000263202	ENSP00000274140
ENSP00000263202	ENSP00000274255
ENSP00000263202	ENSP00000276416
ENSP00000263202	ENSP00000287936
ENSP00000263202	ENSP00000290649
ENSP00000263202	ENSP00000290810
ENSP00000263202	ENSP00000294119
ENSP00000263202	ENSP00000296273
ENSP00000263202	ENSP00000296328
ENSP00000263202	ENSP00000296509
ENSP00000263202	ENSP00000299045
ENSP00000263202	ENSP00000300249
ENSP00000263202	ENSP00000302176
ENSP00000263202	ENSP00000305810
ENSP00000263202	ENSP00000309031
ENSP00000263202	ENSP00000309141
ENSP00000263202	ENSP00000310129
ENSP00000263202	ENSP00000311496
ENSP00000263202	ENSP00000313171
ENSP00000263202	ENSP00000314004
ENSP00000263202	ENSP00000317087
ENSP00000263202	ENSP00000321617
ENSP00000263202	ENSP00000325919
ENSP00000263202	ENSP00000326813
ENSP00000263202	ENSP00000330343
ENSP00000263202	ENSP00000331487
ENSP00000263202	ENSP00000338413
ENSP00000263202	ENSP00000343001
ENSP00000263202	ENSP00000346130
ENSP00000263202	ENSP00000348602
ENSP00000263202	ENSP00000350512
ENSP00000263202	ENSP00000360286

ENSP00000263202	ENSP00000364240
ENSP00000263202	ENSP00000366395
ENSP00000263202	ENSP00000366843
ENSP00000263202	ENSP00000370222
ENSP00000263202	ENSP00000379457
ENSP00000263202	ENSP00000380460
ENSP00000263202	ENSP00000381282
ENSP00000263202	ENSP00000390783
ENSP00000263205	ENSP00000263377
ENSP00000263205	ENSP00000263390
ENSP00000263205	ENSP00000264160
ENSP00000263205	ENSP00000265350
ENSP00000263205	ENSP00000267064
ENSP00000263205	ENSP00000268711
ENSP00000263205	ENSP00000282007
ENSP00000263205	ENSP00000286317
ENSP00000263205	ENSP00000290663
ENSP00000263205	ENSP00000292035
ENSP00000263205	ENSP00000293777
ENSP00000263205	ENSP00000297347
ENSP00000263205	ENSP00000299543
ENSP00000263205	ENSP00000300651
ENSP00000263205	ENSP00000323720
ENSP00000263205	ENSP00000325612
ENSP00000263205	ENSP00000326767
ENSP00000263205	ENSP00000337340
ENSP00000263205	ENSP00000341551
ENSP00000263205	ENSP00000347733
ENSP00000263205	ENSP00000348069
ENSP00000263205	ENSP00000354951
ENSP00000263205	ENSP00000357047
ENSP00000263205	ENSP00000357907
ENSP00000263205	ENSP00000360371
ENSP00000263205	ENSP00000362948
ENSP00000263205	ENSP00000363193
ENSP00000263205	ENSP00000365588
ENSP00000263205	ENSP00000373060
ENSP00000263205	ENSP00000384048
ENSP00000263205	ENSP00000387429
ENSP00000263205	ENSP00000391774
ENSP00000263205	ENSP00000392617
ENSP00000263205	ENSP00000396484
ENSP00000263205	ENSP00000404318
ENSP00000263205	ENSP00000409167
ENSP00000263205	ENSP00000415212
ENSP00000263205	ENSP00000416511

ENSP00000263205	ENSP00000418734
ENSP00000263208	ENSP00000263956
ENSP00000263208	ENSP00000264515
ENSP00000263208	ENSP00000264705
ENSP00000263208	ENSP00000265372
ENSP00000263208	ENSP00000265990
ENSP00000263208	ENSP00000267064
ENSP00000263208	ENSP00000269127
ENSP00000263208	ENSP00000271555
ENSP00000263208	ENSP00000278412
ENSP00000263208	ENSP00000279392
ENSP00000263208	ENSP00000282541
ENSP00000263208	ENSP00000283928
ENSP00000263208	ENSP00000287239
ENSP00000263208	ENSP00000291688
ENSP00000263208	ENSP00000296577
ENSP00000263208	ENSP00000296786
ENSP00000263208	ENSP00000298852
ENSP00000263208	ENSP00000299601
ENSP00000263208	ENSP00000299767
ENSP00000263208	ENSP00000302160
ENSP00000263208	ENSP00000309871
ENSP00000263208	ENSP00000310129
ENSP00000263208	ENSP00000310596
ENSP00000263208	ENSP00000310649
ENSP00000263208	ENSP00000311977
ENSP00000263208	ENSP00000314949
ENSP00000263208	ENSP00000315700
ENSP00000263208	ENSP00000317334
ENSP00000263208	ENSP00000319170
ENSP00000263208	ENSP00000321810
ENSP00000263208	ENSP00000322180
ENSP00000263208	ENSP00000322242
ENSP00000263208	ENSP00000328720
ENSP00000263208	ENSP00000329933
ENSP00000263208	ENSP00000331310
ENSP00000263208	ENSP00000336842
ENSP00000263208	ENSP00000338093
ENSP00000263208	ENSP00000341243
ENSP00000263208	ENSP00000342434
ENSP00000263208	ENSP00000344741
ENSP00000263208	ENSP00000346566
ENSP00000263208	ENSP00000348577
ENSP00000263208	ENSP00000353458
ENSP00000263208	ENSP00000353622
ENSP00000263208	ENSP00000353998

ENSP00000263208	ENSP00000354522
ENSP00000263208	ENSP00000355205
ENSP00000263208	ENSP00000355884
ENSP00000263208	ENSP00000355890
ENSP00000263208	ENSP00000356405
ENSP00000263208	ENSP00000356448
ENSP00000263208	ENSP00000357907
ENSP00000263208	ENSP00000360532
ENSP00000263208	ENSP00000361699
ENSP00000263208	ENSP00000362413
ENSP00000263208	ENSP00000362592
ENSP00000263208	ENSP00000362688
ENSP00000263208	ENSP00000364524
ENSP00000263208	ENSP00000365837
ENSP00000263208	ENSP00000366672
ENSP00000263208	ENSP00000366953
ENSP00000263208	ENSP00000366974
ENSP00000263208	ENSP00000368438
ENSP00000263208	ENSP00000369075
ENSP00000263208	ENSP00000369716
ENSP00000263208	ENSP00000370741
ENSP00000263208	ENSP00000371236
ENSP00000263208	ENSP00000374213
ENSP00000263208	ENSP00000376534
ENSP00000263208	ENSP00000376946
ENSP00000263208	ENSP00000377747
ENSP00000263208	ENSP00000379217
ENSP00000263208	ENSP00000380460
ENSP00000263208	ENSP00000380504
ENSP00000263208	ENSP00000386759
ENSP00000263208	ENSP00000387219
ENSP00000263208	ENSP00000387982
ENSP00000263208	ENSP00000388872
ENSP00000263208	ENSP00000392028
ENSP00000263208	ENSP00000393393
ENSP00000263208	ENSP00000402084
ENSP00000263208	ENSP00000418734
ENSP00000263208	ENSP00000420714
ENSP00000263209	ENSP00000314491
ENSP00000263209	ENSP00000318195
ENSP00000263209	ENSP00000339845
ENSP00000263209	ENSP00000356520
ENSP00000263209	ENSP00000363745
ENSP00000263209	ENSP00000404121
ENSP00000263228	ENSP00000263274
ENSP00000263228	ENSP00000263702

ENSP00000263228	ENSP00000263966
ENSP00000263228	ENSP00000264380
ENSP00000263228	ENSP00000265070
ENSP00000263228	ENSP00000265465
ENSP00000263228	ENSP00000274289
ENSP00000263228	ENSP00000276014
ENSP00000263228	ENSP00000281708
ENSP00000263228	ENSP00000290810
ENSP00000263228	ENSP00000295030
ENSP00000263228	ENSP00000296161
ENSP00000263228	ENSP00000300648
ENSP00000263228	ENSP00000303315
ENSP00000263228	ENSP00000306906
ENSP00000263228	ENSP00000310521
ENSP00000263228	ENSP00000313034
ENSP00000263228	ENSP00000325863
ENSP00000263228	ENSP00000333946
ENSP00000263228	ENSP00000334188
ENSP00000263228	ENSP00000336606
ENSP00000263228	ENSP00000337701
ENSP00000263228	ENSP00000338967
ENSP00000263228	ENSP00000345530
ENSP00000263228	ENSP00000350512
ENSP00000263228	ENSP00000354972
ENSP00000263228	ENSP00000355028
ENSP00000263228	ENSP00000356807
ENSP00000263228	ENSP00000359206
ENSP00000263228	ENSP00000360149
ENSP00000263228	ENSP00000361310
ENSP00000263228	ENSP00000367299
ENSP00000263228	ENSP00000368959
ENSP00000263228	ENSP00000376410
ENSP00000263228	ENSP00000379228
ENSP00000263228	ENSP00000388320
ENSP00000263228	ENSP00000402084
ENSP00000263233	ENSP00000278379
ENSP00000263233	ENSP00000284240
ENSP00000263233	ENSP00000293441
ENSP00000263233	ENSP00000322457
ENSP00000263233	ENSP00000346550
ENSP00000263233	ENSP00000377148
ENSP00000263233	ENSP00000380921
ENSP00000263233	ENSP00000382177
ENSP00000263246	ENSP00000276603
ENSP00000263246	ENSP00000290332
ENSP00000263246	ENSP00000295987

ENSP00000263246	ENSP00000300086
ENSP00000263246	ENSP00000306477
ENSP00000263246	ENSP00000307900
ENSP00000263246	ENSP00000312664
ENSP00000263246	ENSP00000313829
ENSP00000263246	ENSP00000330484
ENSP00000263246	ENSP00000340019
ENSP00000263246	ENSP00000343248
ENSP00000263246	ENSP00000347548
ENSP00000263246	ENSP00000350249
ENSP00000263246	ENSP00000350297
ENSP00000263246	ENSP00000352706
ENSP00000263246	ENSP00000352802
ENSP00000263246	ENSP00000356694
ENSP00000263246	ENSP00000359096
ENSP00000263246	ENSP00000359100
ENSP00000263246	ENSP00000359691
ENSP00000263246	ENSP00000365891
ENSP00000263246	ENSP00000368439
ENSP00000263246	ENSP00000369897
ENSP00000263246	ENSP00000390948
ENSP00000263256	ENSP00000386134
ENSP00000263265	ENSP00000286835
ENSP00000263265	ENSP00000315859
ENSP00000263265	ENSP00000325863
ENSP00000263265	ENSP00000341382
ENSP00000263265	ENSP00000348215
ENSP00000263265	ENSP00000352257
ENSP00000263265	ENSP00000356146
ENSP00000263265	ENSP00000358902
ENSP00000263265	ENSP00000380450
ENSP00000263274	ENSP00000267430
ENSP00000263274	ENSP00000268043
ENSP00000263274	ENSP00000282516
ENSP00000263274	ENSP00000285021
ENSP00000263274	ENSP00000296273
ENSP00000263274	ENSP00000298139
ENSP00000263274	ENSP00000299206
ENSP00000263274	ENSP00000299601
ENSP00000263274	ENSP00000307853
ENSP00000263274	ENSP00000308944
ENSP00000263274	ENSP00000310520
ENSP00000263274	ENSP00000311873
ENSP00000263274	ENSP00000313171
ENSP00000263274	ENSP00000315700
ENSP00000263274	ENSP00000317636

ENSP00000263274	ENSP00000318977
ENSP00000263274	ENSP00000322570
ENSP00000263274	ENSP00000325423
ENSP00000263274	ENSP00000325863
ENSP00000263274	ENSP00000340879
ENSP00000263274	ENSP00000343741
ENSP00000263274	ENSP00000347232
ENSP00000263274	ENSP00000347978
ENSP00000263274	ENSP00000351284
ENSP00000263274	ENSP00000351697
ENSP00000263274	ENSP00000360216
ENSP00000263274	ENSP00000364929
ENSP00000263274	ENSP00000368349
ENSP00000263274	ENSP00000368438
ENSP00000263274	ENSP00000369411
ENSP00000263274	ENSP00000370151
ENSP00000263274	ENSP00000371475
ENSP00000263274	ENSP00000372023
ENSP00000263274	ENSP00000402515
ENSP00000263277	ENSP00000269122
ENSP00000263277	ENSP00000323377
ENSP00000263277	ENSP00000340693
ENSP00000263277	ENSP00000342032
ENSP00000263277	ENSP00000347169
ENSP00000263277	ENSP00000349465
ENSP00000263277	ENSP00000376268
ENSP00000263277	ENSP00000414920
ENSP00000263278	ENSP00000340671
ENSP00000263339	ENSP00000275015
ENSP00000263339	ENSP00000329002
ENSP00000263339	ENSP00000330959
ENSP00000263339	ENSP00000381698
ENSP00000263341	ENSP00000329357
ENSP00000263341	ENSP00000330959
ENSP00000263346	ENSP00000361010
ENSP00000263360	ENSP00000264709
ENSP00000263360	ENSP00000264926
ENSP00000263360	ENSP00000267163
ENSP00000263360	ENSP00000269122
ENSP00000263360	ENSP00000269197
ENSP00000263360	ENSP00000294339
ENSP00000263360	ENSP00000295901
ENSP00000263360	ENSP00000300291
ENSP00000263360	ENSP00000301764
ENSP00000263360	ENSP00000310040
ENSP00000263360	ENSP00000310129

ENSP00000263360	ENSP00000311677
ENSP00000263360	ENSP00000314080
ENSP00000263360	ENSP00000316578
ENSP00000263360	ENSP00000317790
ENSP00000263360	ENSP00000319169
ENSP00000263360	ENSP00000332995
ENSP00000263360	ENSP00000338727
ENSP00000263360	ENSP00000340820
ENSP00000263360	ENSP00000341280
ENSP00000263360	ENSP00000343535
ENSP00000263360	ENSP00000352516
ENSP00000263360	ENSP00000353375
ENSP00000263360	ENSP00000353508
ENSP00000263360	ENSP00000354995
ENSP00000263360	ENSP00000358862
ENSP00000263360	ENSP00000359321
ENSP00000263360	ENSP00000361076
ENSP00000263360	ENSP00000361824
ENSP00000263360	ENSP00000362413
ENSP00000263360	ENSP00000362592
ENSP00000263360	ENSP00000364119
ENSP00000263360	ENSP00000364839
ENSP00000263360	ENSP00000366557
ENSP00000263360	ENSP00000369411
ENSP00000263360	ENSP00000370968
ENSP00000263360	ENSP00000375857
ENSP00000263360	ENSP00000376204
ENSP00000263360	ENSP00000381840
ENSP00000263360	ENSP00000382688
ENSP00000263360	ENSP00000383333
ENSP00000263360	ENSP00000387006
ENSP00000263360	ENSP00000404658
ENSP00000263360	ENSP00000417980
ENSP00000263368	ENSP00000386717
ENSP00000263377	ENSP00000264157
ENSP00000263377	ENSP00000264335
ENSP00000263377	ENSP00000276072
ENSP00000263377	ENSP00000283882
ENSP00000263377	ENSP00000295321
ENSP00000263377	ENSP00000296273
ENSP00000263377	ENSP00000296702
ENSP00000263377	ENSP00000297338
ENSP00000263377	ENSP00000300574
ENSP00000263377	ENSP00000300651
ENSP00000263377	ENSP00000305689
ENSP00000263377	ENSP00000312697

ENSP00000263377	ENSP00000313171
ENSP00000263377	ENSP00000319104
ENSP00000263377	ENSP00000323720
ENSP00000263377	ENSP00000328773
ENSP00000263377	ENSP00000329933
ENSP00000263377	ENSP00000340896
ENSP00000263377	ENSP00000348610
ENSP00000263377	ENSP00000350767
ENSP00000263377	ENSP00000351284
ENSP00000263377	ENSP00000354522
ENSP00000263377	ENSP00000354673
ENSP00000263377	ENSP00000355205
ENSP00000263377	ENSP00000360532
ENSP00000263377	ENSP00000361998
ENSP00000263377	ENSP00000363193
ENSP00000263377	ENSP00000366974
ENSP00000263377	ENSP00000369411
ENSP00000263377	ENSP00000374213
ENSP00000263377	ENSP00000379946
ENSP00000263377	ENSP00000394085
ENSP00000263379	ENSP00000349365
ENSP00000263381	ENSP00000311825
ENSP00000263381	ENSP00000351947
ENSP00000263381	ENSP00000380718
ENSP00000263381	ENSP00000417281
ENSP00000263381	ENSP00000417980
ENSP00000263383	ENSP00000322991
ENSP00000263383	ENSP00000339435
ENSP00000263383	ENSP00000352257
ENSP00000263383	ENSP00000353073
ENSP00000263384	ENSP00000276014
ENSP00000263384	ENSP00000387006
ENSP00000263390	ENSP00000264157
ENSP00000263390	ENSP00000264515
ENSP00000263390	ENSP00000265073
ENSP00000263390	ENSP00000265343
ENSP00000263390	ENSP00000265350
ENSP00000263390	ENSP00000266732
ENSP00000263390	ENSP00000268711
ENSP00000263390	ENSP00000272645
ENSP00000263390	ENSP00000276072
ENSP00000263390	ENSP00000281928
ENSP00000263390	ENSP00000282007
ENSP00000263390	ENSP00000282892
ENSP00000263390	ENSP00000283882
ENSP00000263390	ENSP00000286317

ENSP00000263390	ENSP00000290663
ENSP00000263390	ENSP00000292035
ENSP00000263390	ENSP00000293371
ENSP00000263390	ENSP00000293777
ENSP00000263390	ENSP00000295066
ENSP00000263390	ENSP00000295685
ENSP00000263390	ENSP00000296388
ENSP00000263390	ENSP00000296564
ENSP00000263390	ENSP00000297347
ENSP00000263390	ENSP00000299424
ENSP00000263390	ENSP00000299543
ENSP00000263390	ENSP00000300651
ENSP00000263390	ENSP00000301788
ENSP00000263390	ENSP00000302160
ENSP00000263390	ENSP00000304593
ENSP00000263390	ENSP00000305422
ENSP00000263390	ENSP00000305689
ENSP00000263390	ENSP00000312735
ENSP00000263390	ENSP00000314949
ENSP00000263390	ENSP00000317636
ENSP00000263390	ENSP00000320346
ENSP00000263390	ENSP00000323720
ENSP00000263390	ENSP00000324124
ENSP00000263390	ENSP00000325376
ENSP00000263390	ENSP00000325612
ENSP00000263390	ENSP00000325677
ENSP00000263390	ENSP00000326767
ENSP00000263390	ENSP00000334188
ENSP00000263390	ENSP00000337340
ENSP00000263390	ENSP00000340271
ENSP00000263390	ENSP00000342343
ENSP00000263390	ENSP00000342889
ENSP00000263390	ENSP00000343745
ENSP00000263390	ENSP00000344537
ENSP00000263390	ENSP00000347733
ENSP00000263390	ENSP00000348069
ENSP00000263390	ENSP00000348610
ENSP00000263390	ENSP00000354633
ENSP00000263390	ENSP00000354681
ENSP00000263390	ENSP00000354951
ENSP00000263390	ENSP00000354977
ENSP00000263390	ENSP00000355051
ENSP00000263390	ENSP00000357047
ENSP00000263390	ENSP00000357887
ENSP00000263390	ENSP00000357907
ENSP00000263390	ENSP00000358854

ENSP00000263390	ENSP00000360116
ENSP00000263390	ENSP00000360519
ENSP00000263390	ENSP00000362068
ENSP00000263390	ENSP00000362948
ENSP00000263390	ENSP00000363193
ENSP00000263390	ENSP00000366135
ENSP00000263390	ENSP00000367406
ENSP00000263390	ENSP00000369695
ENSP00000263390	ENSP00000370119
ENSP00000263390	ENSP00000370968
ENSP00000263390	ENSP00000374213
ENSP00000263390	ENSP00000380054
ENSP00000263390	ENSP00000386134
ENSP00000263390	ENSP00000386759
ENSP00000263390	ENSP00000396749
ENSP00000263390	ENSP00000403852
ENSP00000263409	ENSP00000279804
ENSP00000263409	ENSP00000338799
ENSP00000263409	ENSP00000340944
ENSP00000263409	ENSP00000355370
ENSP00000263409	ENSP00000415900
ENSP00000263413	ENSP00000328940
ENSP00000263413	ENSP00000354850
ENSP00000263413	ENSP00000396937
ENSP00000263461	ENSP00000296328
ENSP00000263461	ENSP00000342538
ENSP00000263468	ENSP00000265295
ENSP00000263468	ENSP00000278919
ENSP00000263468	ENSP00000334002
ENSP00000263468	ENSP00000347184
ENSP00000263468	ENSP00000355596
ENSP00000263468	ENSP00000358778
ENSP00000263468	ENSP00000362441
ENSP00000263468	ENSP00000368797
ENSP00000263468	ENSP00000378180
ENSP00000263468	ENSP00000378338
ENSP00000263468	ENSP00000387262
ENSP00000263512	ENSP00000355136
ENSP00000263552	ENSP00000331310
ENSP00000263552	ENSP00000360398
ENSP00000263552	ENSP00000371888
ENSP00000263552	ENSP00000386722
ENSP00000263563	ENSP00000329411
ENSP00000263563	ENSP00000360215
ENSP00000263576	ENSP00000264157
ENSP00000263576	ENSP00000264883

ENSP00000263576	ENSP00000265351
ENSP00000263576	ENSP00000265963
ENSP00000263576	ENSP00000272444
ENSP00000263576	ENSP00000280154
ENSP00000263576	ENSP00000290231
ENSP00000263576	ENSP00000291688
ENSP00000263576	ENSP00000298532
ENSP00000263576	ENSP00000300146
ENSP00000263576	ENSP00000302160
ENSP00000263576	ENSP00000302790
ENSP00000263576	ENSP00000307850
ENSP00000263576	ENSP00000308622
ENSP00000263576	ENSP00000310596
ENSP00000263576	ENSP00000316032
ENSP00000263576	ENSP00000320566
ENSP00000263576	ENSP00000331817
ENSP00000263576	ENSP00000337405
ENSP00000263576	ENSP00000348107
ENSP00000263576	ENSP00000348577
ENSP00000263576	ENSP00000353741
ENSP00000263576	ENSP00000354522
ENSP00000263576	ENSP00000356448
ENSP00000263576	ENSP00000358497
ENSP00000263576	ENSP00000358812
ENSP00000263576	ENSP00000361949
ENSP00000263576	ENSP00000366135
ENSP00000263576	ENSP00000369897
ENSP00000263576	ENSP00000370105
ENSP00000263576	ENSP00000371155
ENSP00000263576	ENSP00000374529
ENSP00000263576	ENSP00000375809
ENSP00000263576	ENSP00000391944
ENSP00000263577	ENSP00000344456
ENSP00000263578	ENSP00000366135
ENSP00000263578	ENSP00000374280
ENSP00000263579	ENSP00000263857
ENSP00000263579	ENSP00000264029
ENSP00000263579	ENSP00000264951
ENSP00000263579	ENSP00000296328
ENSP00000263579	ENSP00000314949
ENSP00000263579	ENSP00000361120
ENSP00000263579	ENSP00000363384
ENSP00000263620	ENSP00000268058
ENSP00000263620	ENSP00000303315
ENSP00000263620	ENSP00000307684
ENSP00000263620	ENSP00000310649

ENSP00000263620	ENSP00000313419
ENSP00000263620	ENSP00000323720
ENSP00000263620	ENSP00000343023
ENSP00000263620	ENSP00000368686
ENSP00000263629	ENSP00000263791
ENSP00000263629	ENSP00000264995
ENSP00000263629	ENSP00000265537
ENSP00000263629	ENSP00000265732
ENSP00000263629	ENSP00000268661
ENSP00000263629	ENSP00000274606
ENSP00000263629	ENSP00000274680
ENSP00000263629	ENSP00000278572
ENSP00000263629	ENSP00000282276
ENSP00000263629	ENSP00000292478
ENSP00000263629	ENSP00000294053
ENSP00000263629	ENSP00000296577
ENSP00000263629	ENSP00000296802
ENSP00000263629	ENSP00000298310
ENSP00000263629	ENSP00000298746
ENSP00000263629	ENSP00000299601
ENSP00000263629	ENSP00000301585
ENSP00000263629	ENSP00000302160
ENSP00000263629	ENSP00000302801
ENSP00000263629	ENSP00000304668
ENSP00000263629	ENSP00000307889
ENSP00000263629	ENSP00000308897
ENSP00000263629	ENSP00000309433
ENSP00000263629	ENSP00000311430
ENSP00000263629	ENSP00000318646
ENSP00000263629	ENSP00000320309
ENSP00000263629	ENSP00000325448
ENSP00000263629	ENSP00000329558
ENSP00000263629	ENSP00000339795
ENSP00000263629	ENSP00000340093
ENSP00000263629	ENSP00000343557
ENSP00000263629	ENSP00000346022
ENSP00000263629	ENSP00000346120
ENSP00000263629	ENSP00000346236
ENSP00000263629	ENSP00000346762
ENSP00000263629	ENSP00000348107
ENSP00000263629	ENSP00000352678
ENSP00000263629	ENSP00000354125
ENSP00000263629	ENSP00000354739
ENSP00000263629	ENSP00000356290
ENSP00000263629	ENSP00000358140
ENSP00000263629	ENSP00000358497

ENSP00000263629	ENSP00000358563
ENSP00000263629	ENSP00000358812
ENSP00000263629	ENSP00000359345
ENSP00000263629	ENSP00000361076
ENSP00000263629	ENSP00000361982
ENSP00000263629	ENSP00000362744
ENSP00000263629	ENSP00000363944
ENSP00000263629	ENSP00000364119
ENSP00000263629	ENSP00000364320
ENSP00000263629	ENSP00000367498
ENSP00000263629	ENSP00000367637
ENSP00000263629	ENSP00000369042
ENSP00000263629	ENSP00000369162
ENSP00000263629	ENSP00000370258
ENSP00000263629	ENSP00000373404
ENSP00000263629	ENSP00000373700
ENSP00000263629	ENSP00000377617
ENSP00000263629	ENSP00000379888
ENSP00000263629	ENSP00000380982
ENSP00000263629	ENSP00000393953
ENSP00000263629	ENSP00000417229
ENSP00000263642	ENSP00000264926
ENSP00000263642	ENSP00000265304
ENSP00000263642	ENSP00000267430
ENSP00000263642	ENSP00000272444
ENSP00000263642	ENSP00000287437
ENSP00000263642	ENSP00000300413
ENSP00000263642	ENSP00000306561
ENSP00000263642	ENSP00000307853
ENSP00000263642	ENSP00000308944
ENSP00000263642	ENSP00000310520
ENSP00000263642	ENSP00000314491
ENSP00000263642	ENSP00000317334
ENSP00000263642	ENSP00000317376
ENSP00000263642	ENSP00000322570
ENSP00000263642	ENSP00000323439
ENSP00000263642	ENSP00000340019
ENSP00000263642	ENSP00000340305
ENSP00000263642	ENSP00000343313
ENSP00000263642	ENSP00000343392
ENSP00000263642	ENSP00000343741
ENSP00000263642	ENSP00000351284
ENSP00000263642	ENSP00000351697
ENSP00000263642	ENSP00000354957
ENSP00000263642	ENSP00000355388
ENSP00000263642	ENSP00000356411

ENSP00000263642	ENSP00000358576
ENSP00000263642	ENSP00000362094
ENSP00000263642	ENSP00000364270
ENSP00000263642	ENSP00000365439
ENSP00000263642	ENSP00000368349
ENSP00000263642	ENSP00000368438
ENSP00000263642	ENSP00000369335
ENSP00000263642	ENSP00000370151
ENSP00000263642	ENSP00000371634
ENSP00000263642	ENSP00000377344
ENSP00000263642	ENSP00000377527
ENSP00000263642	ENSP00000394649
ENSP00000263642	ENSP00000396937
ENSP00000263642	ENSP00000401980
ENSP00000263642	ENSP00000406868
ENSP00000263642	ENSP00000410833
ENSP00000263642	ENSP00000420714
ENSP00000263655	ENSP00000378890
ENSP00000263657	ENSP00000268802
ENSP00000263657	ENSP00000270583
ENSP00000263657	ENSP00000270625
ENSP00000263657	ENSP00000274606
ENSP00000263657	ENSP00000274849
ENSP00000263657	ENSP00000278572
ENSP00000263657	ENSP00000283109
ENSP00000263657	ENSP00000291576
ENSP00000263657	ENSP00000296792
ENSP00000263657	ENSP00000296802
ENSP00000263657	ENSP00000297990
ENSP00000263657	ENSP00000298746
ENSP00000263657	ENSP00000301364
ENSP00000263657	ENSP00000302896
ENSP00000263657	ENSP00000308179
ENSP00000263657	ENSP00000311028
ENSP00000263657	ENSP00000311135
ENSP00000263657	ENSP00000311430
ENSP00000263657	ENSP00000314193
ENSP00000263657	ENSP00000315674
ENSP00000263657	ENSP00000317232
ENSP00000263657	ENSP00000317334
ENSP00000263657	ENSP00000318604
ENSP00000263657	ENSP00000318646
ENSP00000263657	ENSP00000320917
ENSP00000263657	ENSP00000324122
ENSP00000263657	ENSP00000325448
ENSP00000263657	ENSP00000326981

ENSP00000263657	ENSP00000327179
ENSP00000263657	ENSP00000328854
ENSP00000263657	ENSP00000339095
ENSP00000263657	ENSP00000339795
ENSP00000263657	ENSP00000340019
ENSP00000263657	ENSP00000342381
ENSP00000263657	ENSP00000346050
ENSP00000263657	ENSP00000346067
ENSP00000263657	ENSP00000349142
ENSP00000263657	ENSP00000355471
ENSP00000263657	ENSP00000355541
ENSP00000263657	ENSP00000356134
ENSP00000263657	ENSP00000358563
ENSP00000263657	ENSP00000358812
ENSP00000263657	ENSP00000359606
ENSP00000263657	ENSP00000360031
ENSP00000263657	ENSP00000361076
ENSP00000263657	ENSP00000361982
ENSP00000263657	ENSP00000362744
ENSP00000263657	ENSP00000363642
ENSP00000263657	ENSP00000363676
ENSP00000263657	ENSP00000363746
ENSP00000263657	ENSP00000364289
ENSP00000263657	ENSP00000364293
ENSP00000263657	ENSP00000364320
ENSP00000263657	ENSP00000365837
ENSP00000263657	ENSP00000366135
ENSP00000263657	ENSP00000366557
ENSP00000263657	ENSP00000368984
ENSP00000263657	ENSP00000370589
ENSP00000263657	ENSP00000371101
ENSP00000263657	ENSP00000372695
ENSP00000263657	ENSP00000376946
ENSP00000263657	ENSP00000377865
ENSP00000263657	ENSP00000379888
ENSP00000263657	ENSP00000380982
ENSP00000263657	ENSP00000384302
ENSP00000263657	ENSP00000384474
ENSP00000263657	ENSP00000386134
ENSP00000263657	ENSP00000389103
ENSP00000263657	ENSP00000399454
ENSP00000263657	ENSP00000402338
ENSP00000263657	ENSP00000402869
ENSP00000263657	ENSP00000405614
ENSP00000263657	ENSP00000410396
ENSP00000263657	ENSP00000413152

ENSP00000263663	ENSP00000302640
ENSP00000263663	ENSP00000314971
ENSP00000263663	ENSP00000325120
ENSP00000263663	ENSP00000327072
ENSP00000263663	ENSP00000366225
ENSP00000263666	ENSP00000268717
ENSP00000263666	ENSP00000286627
ENSP00000263666	ENSP00000295926
ENSP00000263666	ENSP00000307235
ENSP00000263666	ENSP00000356623
ENSP00000263666	ENSP00000363571
ENSP00000263666	ENSP00000363708
ENSP00000263672	ENSP00000306574
ENSP00000263672	ENSP00000329553
ENSP00000263672	ENSP00000347948
ENSP00000263681	ENSP00000313829
ENSP00000263681	ENSP00000330343
ENSP00000263681	ENSP00000333275
ENSP00000263681	ENSP00000333633
ENSP00000263681	ENSP00000352514
ENSP00000263681	ENSP00000352516
ENSP00000263681	ENSP00000354951
ENSP00000263681	ENSP00000362592
ENSP00000263681	ENSP00000368438
ENSP00000263681	ENSP00000379607
ENSP00000263686	ENSP00000265077
ENSP00000263686	ENSP00000278407
ENSP00000263686	ENSP00000329380
ENSP00000263686	ENSP00000351602
ENSP00000263686	ENSP00000373614
ENSP00000263686	ENSP00000401287
ENSP00000263688	ENSP00000305255
ENSP00000263688	ENSP00000309457
ENSP00000263688	ENSP00000326534
ENSP00000263688	ENSP00000327650
ENSP00000263688	ENSP00000331268
ENSP00000263688	ENSP00000356562
ENSP00000263688	ENSP00000360183
ENSP00000263688	ENSP00000369810
ENSP00000263688	ENSP00000385347
ENSP00000263688	ENSP00000390783
ENSP00000263697	ENSP00000301740
ENSP00000263697	ENSP00000326261
ENSP00000263702	ENSP00000272238
ENSP00000263702	ENSP00000311134
ENSP00000263708	ENSP00000280362

ENSP00000263708	ENSP00000300574
ENSP00000263708	ENSP00000302111
ENSP00000263708	ENSP00000362441
ENSP00000263708	ENSP00000371051
ENSP00000263710	ENSP00000263934
ENSP00000263710	ENSP00000264951
ENSP00000263710	ENSP00000265104
ENSP00000263710	ENSP00000271452
ENSP00000263710	ENSP00000286428
ENSP00000263710	ENSP00000287598
ENSP00000263710	ENSP00000291688
ENSP00000263710	ENSP00000296509
ENSP00000263710	ENSP00000302530
ENSP00000263710	ENSP00000323300
ENSP00000263710	ENSP00000334188
ENSP00000263710	ENSP00000337445
ENSP00000263710	ENSP00000347345
ENSP00000263710	ENSP00000347719
ENSP00000263710	ENSP00000348554
ENSP00000263710	ENSP00000348965
ENSP00000263710	ENSP00000351665
ENSP00000263710	ENSP00000355560
ENSP00000263710	ENSP00000356989
ENSP00000263710	ENSP00000360532
ENSP00000263710	ENSP00000363734
ENSP00000263710	ENSP00000366828
ENSP00000263710	ENSP00000372694
ENSP00000263710	ENSP00000377793
ENSP00000263710	ENSP00000378529
ENSP00000263710	ENSP00000380129
ENSP00000263710	ENSP00000382562
ENSP00000263710	ENSP00000412319
ENSP00000263710	ENSP00000414402
ENSP00000263710	ENSP00000415678
ENSP00000263734	ENSP00000264731
ENSP00000263734	ENSP00000268057
ENSP00000263734	ENSP00000272937
ENSP00000263734	ENSP00000282077
ENSP00000263734	ENSP00000298852
ENSP00000263734	ENSP00000300086
ENSP00000263734	ENSP00000329357
ENSP00000263734	ENSP00000333568
ENSP00000263734	ENSP00000341208
ENSP00000263734	ENSP00000351407
ENSP00000263734	ENSP00000351490
ENSP00000263734	ENSP00000353701

ENSP00000263734	ENSP00000355601
ENSP00000263734	ENSP00000367408
ENSP00000263734	ENSP00000381031
ENSP00000263736	ENSP00000272252
ENSP00000263736	ENSP00000278412
ENSP00000263736	ENSP00000280614
ENSP00000263736	ENSP00000285896
ENSP00000263736	ENSP00000292535
ENSP00000263736	ENSP00000309142
ENSP00000263736	ENSP00000333376
ENSP00000263736	ENSP00000353458
ENSP00000263736	ENSP00000354977
ENSP00000263736	ENSP00000356405
ENSP00000263736	ENSP00000365025
ENSP00000263736	ENSP00000373715
ENSP00000263736	ENSP00000374280
ENSP00000263753	ENSP00000311344
ENSP00000263753	ENSP00000313420
ENSP00000263753	ENSP00000316121
ENSP00000263753	ENSP00000339795
ENSP00000263753	ENSP00000346037
ENSP00000263753	ENSP00000352257
ENSP00000263753	ENSP00000355944
ENSP00000263753	ENSP00000361076
ENSP00000263753	ENSP00000363018
ENSP00000263753	ENSP00000378295
ENSP00000263753	ENSP00000417963
ENSP00000263773	ENSP00000272163
ENSP00000263773	ENSP00000313829
ENSP00000263773	ENSP00000318884
ENSP00000263773	ENSP00000333950
ENSP00000263773	ENSP00000370719
ENSP00000263773	ENSP00000397351
ENSP00000263773	ENSP00000412566
ENSP00000263774	ENSP00000264230
ENSP00000263774	ENSP00000266126
ENSP00000263774	ENSP00000266544
ENSP00000263774	ENSP00000295890
ENSP00000263774	ENSP00000301585
ENSP00000263774	ENSP00000315774
ENSP00000263774	ENSP00000322450
ENSP00000263774	ENSP00000323076
ENSP00000263774	ENSP00000327268
ENSP00000263774	ENSP00000337127
ENSP00000263774	ENSP00000347988
ENSP00000263774	ENSP00000354665

ENSP00000263774	ENSP00000354687
ENSP00000263774	ENSP00000354813
ENSP00000263774	ENSP00000354961
ENSP00000263774	ENSP00000355877
ENSP00000263774	ENSP00000356972
ENSP00000263774	ENSP00000362873
ENSP00000263774	ENSP00000369176
ENSP00000263774	ENSP00000394869
ENSP00000263791	ENSP00000266079
ENSP00000263791	ENSP00000266126
ENSP00000263791	ENSP00000273783
ENSP00000263791	ENSP00000284202
ENSP00000263791	ENSP00000289371
ENSP00000263791	ENSP00000296509
ENSP00000263791	ENSP00000298746
ENSP00000263791	ENSP00000299767
ENSP00000263791	ENSP00000300648
ENSP00000263791	ENSP00000302801
ENSP00000263791	ENSP00000303315
ENSP00000263791	ENSP00000304350
ENSP00000263791	ENSP00000310448
ENSP00000263791	ENSP00000311005
ENSP00000263791	ENSP00000313490
ENSP00000263791	ENSP00000313674
ENSP00000263791	ENSP00000315925
ENSP00000263791	ENSP00000323302
ENSP00000263791	ENSP00000324122
ENSP00000263791	ENSP00000325448
ENSP00000263791	ENSP00000336702
ENSP00000263791	ENSP00000341551
ENSP00000263791	ENSP00000342381
ENSP00000263791	ENSP00000353575
ENSP00000263791	ENSP00000356548
ENSP00000263791	ENSP00000358563
ENSP00000263791	ENSP00000360676
ENSP00000263791	ENSP00000361982
ENSP00000263791	ENSP00000363308
ENSP00000263791	ENSP00000363313
ENSP00000263791	ENSP00000363582
ENSP00000263791	ENSP00000364119
ENSP00000263791	ENSP00000370258
ENSP00000263791	ENSP00000370589
ENSP00000263791	ENSP00000374399
ENSP00000263791	ENSP00000381031
ENSP00000263791	ENSP00000394869
ENSP00000263791	ENSP00000417229

ENSP00000263795	ENSP00000264108
ENSP00000263795	ENSP00000264233
ENSP00000263795	ENSP00000265081
ENSP00000263795	ENSP00000265304
ENSP00000263795	ENSP00000278412
ENSP00000263795	ENSP00000285021
ENSP00000263795	ENSP00000293860
ENSP00000263795	ENSP00000296380
ENSP00000263795	ENSP00000296577
ENSP00000263795	ENSP00000299601
ENSP00000263795	ENSP00000303088
ENSP00000263795	ENSP00000307525
ENSP00000263795	ENSP00000310129
ENSP00000263795	ENSP00000313171
ENSP00000263795	ENSP00000313350
ENSP00000263795	ENSP00000317039
ENSP00000263795	ENSP00000328854
ENSP00000263795	ENSP00000336752
ENSP00000263795	ENSP00000336868
ENSP00000263795	ENSP00000341382
ENSP00000263795	ENSP00000343741
ENSP00000263795	ENSP00000343943
ENSP00000263795	ENSP00000346725
ENSP00000263795	ENSP00000347232
ENSP00000263795	ENSP00000348596
ENSP00000263795	ENSP00000351575
ENSP00000263795	ENSP00000352257
ENSP00000263795	ENSP00000353741
ENSP00000263795	ENSP00000358478
ENSP00000263795	ENSP00000359290
ENSP00000263795	ENSP00000360031
ENSP00000263795	ENSP00000363021
ENSP00000263795	ENSP00000365534
ENSP00000263795	ENSP00000366828
ENSP00000263795	ENSP00000366974
ENSP00000263795	ENSP00000379372
ENSP00000263795	ENSP00000380504
ENSP00000263795	ENSP00000384109
ENSP00000263795	ENSP00000388872
ENSP00000263795	ENSP00000394649
ENSP00000263795	ENSP00000396704
ENSP00000263795	ENSP00000397441
ENSP00000263795	ENSP00000402515
ENSP00000263795	ENSP00000403852
ENSP00000263795	ENSP00000406868
ENSP00000263795	ENSP00000407047

ENSP00000263805	ENSP00000264108
ENSP00000263805	ENSP00000265056
ENSP00000263805	ENSP00000313059
ENSP00000263805	ENSP00000325690
ENSP00000263805	ENSP00000330343
ENSP00000263805	ENSP00000355759
ENSP00000263805	ENSP00000362041
ENSP00000263805	ENSP00000362592
ENSP00000263805	ENSP00000381698
ENSP00000263805	ENSP00000392601
ENSP00000263816	ENSP00000273951
ENSP00000263816	ENSP00000277480
ENSP00000263816	ENSP00000278282
ENSP00000263816	ENSP00000295897
ENSP00000263816	ENSP00000296785
ENSP00000263816	ENSP00000313084
ENSP00000263816	ENSP00000313391
ENSP00000263816	ENSP00000321334
ENSP00000263816	ENSP00000330393
ENSP00000263816	ENSP00000332681
ENSP00000263816	ENSP00000340698
ENSP00000263816	ENSP00000345731
ENSP00000263816	ENSP00000347504
ENSP00000263816	ENSP00000348986
ENSP00000263816	ENSP00000355133
ENSP00000263816	ENSP00000355146
ENSP00000263816	ENSP00000367064
ENSP00000263816	ENSP00000368678
ENSP00000263816	ENSP00000381793
ENSP00000263817	ENSP00000265316
ENSP00000263817	ENSP00000301263
ENSP00000263817	ENSP00000310129
ENSP00000263817	ENSP00000329002
ENSP00000263817	ENSP00000362334
ENSP00000263817	ENSP00000410758
ENSP00000263851	ENSP00000306157
ENSP00000263856	ENSP00000265388
ENSP00000263856	ENSP00000273398
ENSP00000263856	ENSP00000290330
ENSP00000263856	ENSP00000297265
ENSP00000263856	ENSP00000302239
ENSP00000263856	ENSP00000302896
ENSP00000263856	ENSP00000310440
ENSP00000263856	ENSP00000317468
ENSP00000263856	ENSP00000318629
ENSP00000263856	ENSP00000330408

ENSP00000263856	ENSP00000331201
ENSP00000263856	ENSP00000331719
ENSP00000263856	ENSP00000339208
ENSP00000263856	ENSP00000345216
ENSP00000263856	ENSP00000356602
ENSP00000263856	ENSP00000360916
ENSP00000263856	ENSP00000366225
ENSP00000263856	ENSP00000366565
ENSP00000263856	ENSP00000366746
ENSP00000263856	ENSP00000367299
ENSP00000263856	ENSP00000378368
ENSP00000263856	ENSP00000379500
ENSP00000263856	ENSP00000391432
ENSP00000263857	ENSP00000263956
ENSP00000263857	ENSP00000264010
ENSP00000263857	ENSP00000264995
ENSP00000263857	ENSP00000268661
ENSP00000263857	ENSP00000272418
ENSP00000263857	ENSP00000276014
ENSP00000263857	ENSP00000278412
ENSP00000263857	ENSP00000278572
ENSP00000263857	ENSP00000286317
ENSP00000263857	ENSP00000286835
ENSP00000263857	ENSP00000288937
ENSP00000263857	ENSP00000288976
ENSP00000263857	ENSP00000291386
ENSP00000263857	ENSP00000293860
ENSP00000263857	ENSP00000296674
ENSP00000263857	ENSP00000296786
ENSP00000263857	ENSP00000298281
ENSP00000263857	ENSP00000298746
ENSP00000263857	ENSP00000299853
ENSP00000263857	ENSP00000302478
ENSP00000263857	ENSP00000302640
ENSP00000263857	ENSP00000303515
ENSP00000263857	ENSP00000309433
ENSP00000263857	ENSP00000310966
ENSP00000263857	ENSP00000311977
ENSP00000263857	ENSP00000314311
ENSP00000263857	ENSP00000314971
ENSP00000263857	ENSP00000315791
ENSP00000263857	ENSP00000317334
ENSP00000263857	ENSP00000318604
ENSP00000263857	ENSP00000321636
ENSP00000263857	ENSP00000322570
ENSP00000263857	ENSP00000323720

ENSP00000263857	ENSP00000324122
ENSP00000263857	ENSP00000324124
ENSP00000263857	ENSP00000327821
ENSP00000263857	ENSP00000329466
ENSP00000263857	ENSP00000331111
ENSP00000263857	ENSP00000332171
ENSP00000263857	ENSP00000333920
ENSP00000263857	ENSP00000335357
ENSP00000263857	ENSP00000336606
ENSP00000263857	ENSP00000337405
ENSP00000263857	ENSP00000341885
ENSP00000263857	ENSP00000342889
ENSP00000263857	ENSP00000345096
ENSP00000263857	ENSP00000346015
ENSP00000263857	ENSP00000348010
ENSP00000263857	ENSP00000348089
ENSP00000263857	ENSP00000351284
ENSP00000263857	ENSP00000351575
ENSP00000263857	ENSP00000351697
ENSP00000263857	ENSP00000351885
ENSP00000263857	ENSP00000354916
ENSP00000263857	ENSP00000355051
ENSP00000263857	ENSP00000356146
ENSP00000263857	ENSP00000356470
ENSP00000263857	ENSP00000356807
ENSP00000263857	ENSP00000357882
ENSP00000263857	ENSP00000358497
ENSP00000263857	ENSP00000358563
ENSP00000263857	ENSP00000358812
ENSP00000263857	ENSP00000359290
ENSP00000263857	ENSP00000359688
ENSP00000263857	ENSP00000359818
ENSP00000263857	ENSP00000360532
ENSP00000263857	ENSP00000360676
ENSP00000263857	ENSP00000361465
ENSP00000263857	ENSP00000361982
ENSP00000263857	ENSP00000363021
ENSP00000263857	ENSP00000363779
ENSP00000263857	ENSP00000363944
ENSP00000263857	ENSP00000364289
ENSP00000263857	ENSP00000364293
ENSP00000263857	ENSP00000366135
ENSP00000263857	ENSP00000366557
ENSP00000263857	ENSP00000366828
ENSP00000263857	ENSP00000366974
ENSP00000263857	ENSP00000367029

ENSP00000263857	ENSP00000368349
ENSP00000263857	ENSP00000368927
ENSP00000263857	ENSP00000370589
ENSP00000263857	ENSP00000370968
ENSP00000263857	ENSP00000373404
ENSP00000263857	ENSP00000374213
ENSP00000263857	ENSP00000375809
ENSP00000263857	ENSP00000376946
ENSP00000263857	ENSP00000379158
ENSP00000263857	ENSP00000379372
ENSP00000263857	ENSP00000380460
ENSP00000263857	ENSP00000380504
ENSP00000263857	ENSP00000383503
ENSP00000263857	ENSP00000389103
ENSP00000263857	ENSP00000392859
ENSP00000263857	ENSP00000397636
ENSP00000263857	ENSP00000403852
ENSP00000263857	ENSP00000405636
ENSP00000263857	ENSP00000410530
ENSP00000263857	ENSP00000410954
ENSP00000263857	ENSP00000414110
ENSP00000263857	ENSP00000416599
ENSP00000263881	ENSP00000284637
ENSP00000263881	ENSP00000350512
ENSP00000263881	ENSP00000355136
ENSP00000263881	ENSP00000361640
ENSP00000263881	ENSP00000366746
ENSP00000263881	ENSP00000382423
ENSP00000263897	ENSP00000264157
ENSP00000263897	ENSP00000286448
ENSP00000263897	ENSP00000294179
ENSP00000263897	ENSP00000298854
ENSP00000263897	ENSP00000304051
ENSP00000263897	ENSP00000304321
ENSP00000263897	ENSP00000305255
ENSP00000263897	ENSP00000305810
ENSP00000263897	ENSP00000308944
ENSP00000263897	ENSP00000310521
ENSP00000263897	ENSP00000317714
ENSP00000263897	ENSP00000324628
ENSP00000263897	ENSP00000327650
ENSP00000263897	ENSP00000328203
ENSP00000263897	ENSP00000334657
ENSP00000263897	ENSP00000342755
ENSP00000263897	ENSP00000354032
ENSP00000263897	ENSP00000355028

ENSP00000263897	ENSP00000355721
ENSP00000263897	ENSP00000361990
ENSP00000263897	ENSP00000362399
ENSP00000263897	ENSP00000366225
ENSP00000263897	ENSP00000366395
ENSP00000263897	ENSP00000374529
ENSP00000263897	ENSP00000381293
ENSP00000263897	ENSP00000390783
ENSP00000263897	ENSP00000417281
ENSP00000263925	ENSP00000264935
ENSP00000263925	ENSP00000283635
ENSP00000263925	ENSP00000283646
ENSP00000263925	ENSP00000300181
ENSP00000263925	ENSP00000301727
ENSP00000263925	ENSP00000311202
ENSP00000263925	ENSP00000336606
ENSP00000263925	ENSP00000341679
ENSP00000263925	ENSP00000347169
ENSP00000263925	ENSP00000349576
ENSP00000263925	ENSP00000354201
ENSP00000263925	ENSP00000354911
ENSP00000263925	ENSP00000359507
ENSP00000263925	ENSP00000369962
ENSP00000263925	ENSP00000382595
ENSP00000263925	ENSP00000384391
ENSP00000263934	ENSP00000284981
ENSP00000263934	ENSP00000290607
ENSP00000263934	ENSP00000299601
ENSP00000263934	ENSP00000340698
ENSP00000263934	ENSP00000340820
ENSP00000263934	ENSP00000345731
ENSP00000263934	ENSP00000350283
ENSP00000263934	ENSP00000353508
ENSP00000263934	ENSP00000357651
ENSP00000263934	ENSP00000358283
ENSP00000263934	ENSP00000388373
ENSP00000263934	ENSP00000414187
ENSP00000263956	ENSP00000264720
ENSP00000263956	ENSP00000265351
ENSP00000263956	ENSP00000278412
ENSP00000263956	ENSP00000296328
ENSP00000263956	ENSP00000302790
ENSP00000263956	ENSP00000303088
ENSP00000263956	ENSP00000304704
ENSP00000263956	ENSP00000314949
ENSP00000263956	ENSP00000315791

ENSP00000263956	ENSP00000329029
ENSP00000263956	ENSP00000329466
ENSP00000263956	ENSP00000332444
ENSP00000263956	ENSP00000334564
ENSP00000263956	ENSP00000336856
ENSP00000263956	ENSP00000339353
ENSP00000263956	ENSP00000341243
ENSP00000263956	ENSP00000342889
ENSP00000263956	ENSP00000348010
ENSP00000263956	ENSP00000349213
ENSP00000263956	ENSP00000351575
ENSP00000263956	ENSP00000351947
ENSP00000263956	ENSP00000353622
ENSP00000263956	ENSP00000354723
ENSP00000263956	ENSP00000355185
ENSP00000263956	ENSP00000355384
ENSP00000263956	ENSP00000358576
ENSP00000263956	ENSP00000361180
ENSP00000263956	ENSP00000361219
ENSP00000263956	ENSP00000366828
ENSP00000263956	ENSP00000367527
ENSP00000263956	ENSP00000369100
ENSP00000263956	ENSP00000374455
ENSP00000263956	ENSP00000398208
ENSP00000263966	ENSP00000267169
ENSP00000263966	ENSP00000274255
ENSP00000263966	ENSP00000286835
ENSP00000263966	ENSP00000297185
ENSP00000263966	ENSP00000303279
ENSP00000263966	ENSP00000308339
ENSP00000263966	ENSP00000322457
ENSP00000263966	ENSP00000323913
ENSP00000263966	ENSP00000331487
ENSP00000263966	ENSP00000335304
ENSP00000263966	ENSP00000343535
ENSP00000263966	ENSP00000353804
ENSP00000263966	ENSP00000354720
ENSP00000263966	ENSP00000356146
ENSP00000263966	ENSP00000356652
ENSP00000263966	ENSP00000358497
ENSP00000263966	ENSP00000364486
ENSP00000263966	ENSP00000364794
ENSP00000263966	ENSP00000370258
ENSP00000263966	ENSP00000380460
ENSP00000263966	ENSP00000406490
ENSP00000263966	ENSP00000419235

ENSP00000263974	ENSP00000264193
ENSP00000263974	ENSP00000265354
ENSP00000263974	ENSP00000265970
ENSP00000263974	ENSP00000265990
ENSP00000263974	ENSP00000269127
ENSP00000263974	ENSP00000276072
ENSP00000263974	ENSP00000278616
ENSP00000263974	ENSP00000298532
ENSP00000263974	ENSP00000299424
ENSP00000263974	ENSP00000303315
ENSP00000263974	ENSP00000306983
ENSP00000263974	ENSP00000307684
ENSP00000263974	ENSP00000308944
ENSP00000263974	ENSP00000313420
ENSP00000263974	ENSP00000314949
ENSP00000263974	ENSP00000316114
ENSP00000263974	ENSP00000320949
ENSP00000263974	ENSP00000327072
ENSP00000263974	ENSP00000329029
ENSP00000263974	ENSP00000331310
ENSP00000263974	ENSP00000332171
ENSP00000263974	ENSP00000333920
ENSP00000263974	ENSP00000334061
ENSP00000263974	ENSP00000337405
ENSP00000263974	ENSP00000339353
ENSP00000263974	ENSP00000340271
ENSP00000263974	ENSP00000341243
ENSP00000263974	ENSP00000343741
ENSP00000263974	ENSP00000344537
ENSP00000263974	ENSP00000345702
ENSP00000263974	ENSP00000347733
ENSP00000263974	ENSP00000348089
ENSP00000263974	ENSP00000350359
ENSP00000263974	ENSP00000352121
ENSP00000263974	ENSP00000354558
ENSP00000263974	ENSP00000354633
ENSP00000263974	ENSP00000355051
ENSP00000263974	ENSP00000355205
ENSP00000263974	ENSP00000358854
ENSP00000263974	ENSP00000359531
ENSP00000263974	ENSP00000360515
ENSP00000263974	ENSP00000362068
ENSP00000263974	ENSP00000366974
ENSP00000263974	ENSP00000367406
ENSP00000263974	ENSP00000369695
ENSP00000263974	ENSP00000369716

ENSP00000263974	ENSP00000374213
ENSP00000263974	ENSP00000377747
ENSP00000263974	ENSP00000386759
ENSP00000263974	ENSP00000392028
ENSP00000263974	ENSP00000396620
ENSP00000263974	ENSP00000397259
ENSP00000263974	ENSP00000402338
ENSP00000263974	ENSP00000402515
ENSP00000263974	ENSP00000414006
ENSP00000263985	ENSP00000264161
ENSP00000263985	ENSP00000348395
ENSP00000263985	ENSP00000358042
ENSP00000263985	ENSP00000364035
ENSP00000263997	ENSP00000272645
ENSP00000263997	ENSP00000283351
ENSP00000263997	ENSP00000296328
ENSP00000263997	ENSP00000301788
ENSP00000263997	ENSP00000303315
ENSP00000263997	ENSP00000305459
ENSP00000263997	ENSP00000307850
ENSP00000263997	ENSP00000312735
ENSP00000263997	ENSP00000314949
ENSP00000263997	ENSP00000324124
ENSP00000263997	ENSP00000327716
ENSP00000263997	ENSP00000340823
ENSP00000263997	ENSP00000342889
ENSP00000263997	ENSP00000353698
ENSP00000263997	ENSP00000359290
ENSP00000263997	ENSP00000362135
ENSP00000263997	ENSP00000377969
ENSP00000263997	ENSP00000403852
ENSP00000264005	ENSP00000265052
ENSP00000264005	ENSP00000265175
ENSP00000264005	ENSP00000322218
ENSP00000264005	ENSP00000332258
ENSP00000264005	ENSP00000356591
ENSP00000264010	ENSP00000264169
ENSP00000264010	ENSP00000276072
ENSP00000264010	ENSP00000296930
ENSP00000264010	ENSP00000297338
ENSP00000264010	ENSP00000302640
ENSP00000264010	ENSP00000310966
ENSP00000264010	ENSP00000314949
ENSP00000264010	ENSP00000316578
ENSP00000264010	ENSP00000329466
ENSP00000264010	ENSP00000330343

ENSP00000264010	ENSP00000340271
ENSP00000264010	ENSP00000341551
ENSP00000264010	ENSP00000353622
ENSP00000264010	ENSP00000354720
ENSP00000264010	ENSP00000355759
ENSP00000264010	ENSP00000357283
ENSP00000264010	ENSP00000362441
ENSP00000264010	ENSP00000367029
ENSP00000264010	ENSP00000392028
ENSP00000264010	ENSP00000417281
ENSP00000264020	ENSP00000302913
ENSP00000264027	ENSP00000264451
ENSP00000264027	ENSP00000265896
ENSP00000264027	ENSP00000266095
ENSP00000264027	ENSP00000269703
ENSP00000264027	ENSP00000272163
ENSP00000264027	ENSP00000272322
ENSP00000264027	ENSP00000277010
ENSP00000264027	ENSP00000285949
ENSP00000264027	ENSP00000287936
ENSP00000264027	ENSP00000310721
ENSP00000264027	ENSP00000320604
ENSP00000264027	ENSP00000325425
ENSP00000264027	ENSP00000330812
ENSP00000264027	ENSP00000332258
ENSP00000264027	ENSP00000333946
ENSP00000264027	ENSP00000348380
ENSP00000264027	ENSP00000348762
ENSP00000264027	ENSP00000354522
ENSP00000264027	ENSP00000355517
ENSP00000264027	ENSP00000356591
ENSP00000264027	ENSP00000356989
ENSP00000264027	ENSP00000357775
ENSP00000264027	ENSP00000357998
ENSP00000264027	ENSP00000358695
ENSP00000264027	ENSP00000359067
ENSP00000264027	ENSP00000359380
ENSP00000264027	ENSP00000360918
ENSP00000264027	ENSP00000371236
ENSP00000264027	ENSP00000372697
ENSP00000264027	ENSP00000376268
ENSP00000264027	ENSP00000379430
ENSP00000264027	ENSP00000386284
ENSP00000264027	ENSP00000389934
ENSP00000264027	ENSP00000390831
ENSP00000264027	ENSP00000391197

ENSP00000264027	ENSP00000401445
ENSP00000264027	ENSP00000404016
ENSP00000264027	ENSP00000406988
ENSP00000264027	ENSP00000409952
ENSP00000264028	ENSP00000265070
ENSP00000264028	ENSP00000269216
ENSP00000264028	ENSP00000270176
ENSP00000264028	ENSP00000283943
ENSP00000264028	ENSP00000286448
ENSP00000264028	ENSP00000299767
ENSP00000264028	ENSP00000310521
ENSP00000264028	ENSP00000312735
ENSP00000264028	ENSP00000325002
ENSP00000264028	ENSP00000325369
ENSP00000264028	ENSP00000329419
ENSP00000264028	ENSP00000330945
ENSP00000264028	ENSP00000341243
ENSP00000264028	ENSP00000345530
ENSP00000264028	ENSP00000345681
ENSP00000264028	ENSP00000346015
ENSP00000264028	ENSP00000349970
ENSP00000264028	ENSP00000352738
ENSP00000264028	ENSP00000355231
ENSP00000264028	ENSP00000356744
ENSP00000264028	ENSP00000357048
ENSP00000264028	ENSP00000360183
ENSP00000264028	ENSP00000360532
ENSP00000264028	ENSP00000360798
ENSP00000264028	ENSP00000361287
ENSP00000264028	ENSP00000364188
ENSP00000264028	ENSP00000365567
ENSP00000264028	ENSP00000379065
ENSP00000264028	ENSP00000379228
ENSP00000264028	ENSP00000384434
ENSP00000264028	ENSP00000397435
ENSP00000264028	ENSP00000397441
ENSP00000264029	ENSP00000264335
ENSP00000264029	ENSP00000272902
ENSP00000264029	ENSP00000301329
ENSP00000264029	ENSP00000303208
ENSP00000264029	ENSP00000306574
ENSP00000264029	ENSP00000311768
ENSP00000264029	ENSP00000313432
ENSP00000264029	ENSP00000322991
ENSP00000264029	ENSP00000326813
ENSP00000264029	ENSP00000331817

ENSP00000264029	ENSP00000338703
ENSP00000264029	ENSP00000344460
ENSP00000264029	ENSP00000348395
ENSP00000264029	ENSP00000349022
ENSP00000264029	ENSP00000353458
ENSP00000264029	ENSP00000356903
ENSP00000264029	ENSP00000360031
ENSP00000264029	ENSP00000360286
ENSP00000264029	ENSP00000362463
ENSP00000264029	ENSP00000364293
ENSP00000264029	ENSP00000369335
ENSP00000264029	ENSP00000387185
ENSP00000264029	ENSP00000396704
ENSP00000264036	ENSP00000323687
ENSP00000264042	ENSP00000306523
ENSP00000264042	ENSP00000339826
ENSP00000264047	ENSP00000328088
ENSP00000264047	ENSP00000345530
ENSP00000264065	ENSP00000265174
ENSP00000264065	ENSP00000265462
ENSP00000264065	ENSP00000270538
ENSP00000264065	ENSP00000278572
ENSP00000264065	ENSP00000292180
ENSP00000264065	ENSP00000294053
ENSP00000264065	ENSP00000296411
ENSP00000264065	ENSP00000296577
ENSP00000264065	ENSP00000296805
ENSP00000264065	ENSP00000297338
ENSP00000264065	ENSP00000299767
ENSP00000264065	ENSP00000304350
ENSP00000264065	ENSP00000307525
ENSP00000264065	ENSP00000312735
ENSP00000264065	ENSP00000318147
ENSP00000264065	ENSP00000321259
ENSP00000264065	ENSP00000325312
ENSP00000264065	ENSP00000330836
ENSP00000264065	ENSP00000335321
ENSP00000264065	ENSP00000351137
ENSP00000264065	ENSP00000357998
ENSP00000264065	ENSP00000362110
ENSP00000264065	ENSP00000368565
ENSP00000264065	ENSP00000379607
ENSP00000264093	ENSP00000270625
ENSP00000264093	ENSP00000271452
ENSP00000264093	ENSP00000322706
ENSP00000264093	ENSP00000382595

ENSP00000264093	ENSP00000394382
ENSP00000264108	ENSP00000264169
ENSP00000264108	ENSP00000264183
ENSP00000264108	ENSP00000264705
ENSP00000264108	ENSP00000265056
ENSP00000264108	ENSP00000265462
ENSP00000264108	ENSP00000267197
ENSP00000264108	ENSP00000267568
ENSP00000264108	ENSP00000267953
ENSP00000264108	ENSP00000274242
ENSP00000264108	ENSP00000278412
ENSP00000264108	ENSP00000282185
ENSP00000264108	ENSP00000282412
ENSP00000264108	ENSP00000286349
ENSP00000264108	ENSP00000297431
ENSP00000264108	ENSP00000299767
ENSP00000264108	ENSP00000306682
ENSP00000264108	ENSP00000312244
ENSP00000264108	ENSP00000312870
ENSP00000264108	ENSP00000323678
ENSP00000264108	ENSP00000329466
ENSP00000264108	ENSP00000331310
ENSP00000264108	ENSP00000331817
ENSP00000264108	ENSP00000346453
ENSP00000264108	ENSP00000347602
ENSP00000264108	ENSP00000350767
ENSP00000264108	ENSP00000354125
ENSP00000264108	ENSP00000355011
ENSP00000264108	ENSP00000357965
ENSP00000264108	ENSP00000358140
ENSP00000264108	ENSP00000359818
ENSP00000264108	ENSP00000360515
ENSP00000264108	ENSP00000360621
ENSP00000264108	ENSP00000365877
ENSP00000264108	ENSP00000366974
ENSP00000264108	ENSP00000367408
ENSP00000264108	ENSP00000370258
ENSP00000264108	ENSP00000377793
ENSP00000264108	ENSP00000379607
ENSP00000264108	ENSP00000402084
ENSP00000264108	ENSP00000404658
ENSP00000264108	ENSP00000417132
ENSP00000264108	ENSP00000417229
ENSP00000264108	ENSP00000420588
ENSP00000264122	ENSP00000269485
ENSP00000264122	ENSP00000278888

ENSP00000264122	ENSP00000281172
ENSP00000264122	ENSP00000295901
ENSP00000264122	ENSP00000296474
ENSP00000264122	ENSP00000300574
ENSP00000264122	ENSP00000302913
ENSP00000264122	ENSP00000304311
ENSP00000264122	ENSP00000305133
ENSP00000264122	ENSP00000308774
ENSP00000264122	ENSP00000309474
ENSP00000264122	ENSP00000313419
ENSP00000264122	ENSP00000316460
ENSP00000264122	ENSP00000340944
ENSP00000264122	ENSP00000343785
ENSP00000264122	ENSP00000345530
ENSP00000264122	ENSP00000347942
ENSP00000264122	ENSP00000349727
ENSP00000264122	ENSP00000350297
ENSP00000264122	ENSP00000355136
ENSP00000264122	ENSP00000360916
ENSP00000264122	ENSP00000363089
ENSP00000264122	ENSP00000364893
ENSP00000264122	ENSP00000364898
ENSP00000264122	ENSP00000366843
ENSP00000264122	ENSP00000370719
ENSP00000264122	ENSP00000380150
ENSP00000264122	ENSP00000380921
ENSP00000264126	ENSP00000264233
ENSP00000264126	ENSP00000265148
ENSP00000264126	ENSP00000324020
ENSP00000264126	ENSP00000341848
ENSP00000264126	ENSP00000345731
ENSP00000264126	ENSP00000353854
ENSP00000264126	ENSP00000354158
ENSP00000264126	ENSP00000354486
ENSP00000264126	ENSP00000355922
ENSP00000264126	ENSP00000360141
ENSP00000264126	ENSP00000361467
ENSP00000264126	ENSP00000363921
ENSP00000264126	ENSP00000368872
ENSP00000264126	ENSP00000380150
ENSP00000264151	ENSP00000272424
ENSP00000264151	ENSP00000311984
ENSP00000264151	ENSP00000356476
ENSP00000264151	ENSP00000361186
ENSP00000264151	ENSP00000365534
ENSP00000264151	ENSP00000365943

ENSP00000264157	ENSP00000264515
ENSP00000264157	ENSP00000264926
ENSP00000264157	ENSP00000265343
ENSP00000264157	ENSP00000267163
ENSP00000264157	ENSP00000273064
ENSP00000264157	ENSP00000283109
ENSP00000264157	ENSP00000285896
ENSP00000264157	ENSP00000286448
ENSP00000264157	ENSP00000287598
ENSP00000264157	ENSP00000294179
ENSP00000264157	ENSP00000295066
ENSP00000264157	ENSP00000299601
ENSP00000264157	ENSP00000302160
ENSP00000264157	ENSP00000302530
ENSP00000264157	ENSP00000304903
ENSP00000264157	ENSP00000305810
ENSP00000264157	ENSP00000307850
ENSP00000264157	ENSP00000308546
ENSP00000264157	ENSP00000320566
ENSP00000264157	ENSP00000320949
ENSP00000264157	ENSP00000323720
ENSP00000264157	ENSP00000325677
ENSP00000264157	ENSP00000328773
ENSP00000264157	ENSP00000331817
ENSP00000264157	ENSP00000340896
ENSP00000264157	ENSP00000348010
ENSP00000264157	ENSP00000353622
ENSP00000264157	ENSP00000354673
ENSP00000264157	ENSP00000356405
ENSP00000264157	ENSP00000357440
ENSP00000264157	ENSP00000359290
ENSP00000264157	ENSP00000360183
ENSP00000264157	ENSP00000362900
ENSP00000264157	ENSP00000366225
ENSP00000264157	ENSP00000368459
ENSP00000264157	ENSP00000369695
ENSP00000264157	ENSP00000374280
ENSP00000264157	ENSP00000381657
ENSP00000264157	ENSP00000390783
ENSP00000264158	ENSP00000351832
ENSP00000264158	ENSP00000368686
ENSP00000264159	ENSP00000325863
ENSP00000264159	ENSP00000369335
ENSP00000264160	ENSP00000265350
ENSP00000264160	ENSP00000267064
ENSP00000264160	ENSP00000268379

ENSP00000264160	ENSP00000286317
ENSP00000264160	ENSP00000292035
ENSP00000264160	ENSP00000293777
ENSP00000264160	ENSP00000323720
ENSP00000264160	ENSP00000357907
ENSP00000264160	ENSP00000362948
ENSP00000264160	ENSP00000384048
ENSP00000264160	ENSP00000391774
ENSP00000264160	ENSP00000392617
ENSP00000264160	ENSP00000409279
ENSP00000264161	ENSP00000276326
ENSP00000264161	ENSP00000282276
ENSP00000264161	ENSP00000296802
ENSP00000264161	ENSP00000302896
ENSP00000264161	ENSP00000303279
ENSP00000264161	ENSP00000307567
ENSP00000264161	ENSP00000317334
ENSP00000264161	ENSP00000318604
ENSP00000264161	ENSP00000319977
ENSP00000264161	ENSP00000326699
ENSP00000264161	ENSP00000329715
ENSP00000264161	ENSP00000333934
ENSP00000264161	ENSP00000340019
ENSP00000264161	ENSP00000354522
ENSP00000264161	ENSP00000355890
ENSP00000264161	ENSP00000358042
ENSP00000264161	ENSP00000358335
ENSP00000264161	ENSP00000360327
ENSP00000264161	ENSP00000361557
ENSP00000264161	ENSP00000362576
ENSP00000264161	ENSP00000364035
ENSP00000264161	ENSP00000364794
ENSP00000264161	ENSP00000364815
ENSP00000264161	ENSP00000367923
ENSP00000264161	ENSP00000368140
ENSP00000264161	ENSP00000369038
ENSP00000264161	ENSP00000376946
ENSP00000264161	ENSP00000379607
ENSP00000264161	ENSP00000380460
ENSP00000264161	ENSP00000381891
ENSP00000264161	ENSP00000391432
ENSP00000264161	ENSP00000401121
ENSP00000264161	ENSP00000403359
ENSP00000264161	ENSP00000415316
ENSP00000264167	ENSP00000341243
ENSP00000264167	ENSP00000349016

ENSP00000264167	ENSP00000355607
ENSP00000264169	ENSP00000265056
ENSP00000264169	ENSP00000265728
ENSP00000264169	ENSP00000276326
ENSP00000264169	ENSP00000297431
ENSP00000264169	ENSP00000320885
ENSP00000264169	ENSP00000330343
ENSP00000264169	ENSP00000331817
ENSP00000264169	ENSP00000341243
ENSP00000264169	ENSP00000343701
ENSP00000264169	ENSP00000344866
ENSP00000264169	ENSP00000352257
ENSP00000264169	ENSP00000355153
ENSP00000264169	ENSP00000360621
ENSP00000264169	ENSP00000362592
ENSP00000264169	ENSP00000363021
ENSP00000264169	ENSP00000366953
ENSP00000264169	ENSP00000387982
ENSP00000264169	ENSP00000402084
ENSP00000264170	ENSP00000314441
ENSP00000264170	ENSP00000328671
ENSP00000264170	ENSP00000401508
ENSP00000264183	ENSP00000264501
ENSP00000264183	ENSP00000265990
ENSP00000264183	ENSP00000282841
ENSP00000264183	ENSP00000283195
ENSP00000264183	ENSP00000283882
ENSP00000264183	ENSP00000286428
ENSP00000264183	ENSP00000300249
ENSP00000264183	ENSP00000303315
ENSP00000264183	ENSP00000304593
ENSP00000264183	ENSP00000306983
ENSP00000264183	ENSP00000307208
ENSP00000264183	ENSP00000307684
ENSP00000264183	ENSP00000310649
ENSP00000264183	ENSP00000321617
ENSP00000264183	ENSP00000322242
ENSP00000264183	ENSP00000323720
ENSP00000264183	ENSP00000334188
ENSP00000264183	ENSP00000340896
ENSP00000264183	ENSP00000347602
ENSP00000264183	ENSP00000348784
ENSP00000264183	ENSP00000349213
ENSP00000264183	ENSP00000353622
ENSP00000264183	ENSP00000356470
ENSP00000264183	ENSP00000356989

ENSP00000264183	ENSP00000360473
ENSP00000264183	ENSP00000362166
ENSP00000264183	ENSP00000369695
ENSP00000264183	ENSP00000371888
ENSP00000264183	ENSP00000373340
ENSP00000264183	ENSP00000377262
ENSP00000264183	ENSP00000386759
ENSP00000264183	ENSP00000392617
ENSP00000264193	ENSP00000267426
ENSP00000264193	ENSP00000276072
ENSP00000264193	ENSP00000283033
ENSP00000264193	ENSP00000286091
ENSP00000264193	ENSP00000294168
ENSP00000264193	ENSP00000297185
ENSP00000264193	ENSP00000299424
ENSP00000264193	ENSP00000309595
ENSP00000264193	ENSP00000318147
ENSP00000264193	ENSP00000342026
ENSP00000264193	ENSP00000344537
ENSP00000264193	ENSP00000354633
ENSP00000264193	ENSP00000358854
ENSP00000264193	ENSP00000361998
ENSP00000264193	ENSP00000362068
ENSP00000264193	ENSP00000362576
ENSP00000264193	ENSP00000364815
ENSP00000264193	ENSP00000366974
ENSP00000264193	ENSP00000367406
ENSP00000264193	ENSP00000368646
ENSP00000264193	ENSP00000401121
ENSP00000264193	ENSP00000403359
ENSP00000264193	ENSP00000415316
ENSP00000264193	ENSP00000417763
ENSP00000264198	ENSP00000296257
ENSP00000264198	ENSP00000299138
ENSP00000264198	ENSP00000360116
ENSP00000264198	ENSP00000402316
ENSP00000264218	ENSP00000305877
ENSP00000264220	ENSP00000291576
ENSP00000264220	ENSP00000341243
ENSP00000264220	ENSP00000348577
ENSP00000264220	ENSP00000357880
ENSP00000264220	ENSP00000359976
ENSP00000264220	ENSP00000361965
ENSP00000264220	ENSP00000363021
ENSP00000264220	ENSP00000378350
ENSP00000264220	ENSP00000382595

ENSP00000264228	ENSP00000286621
ENSP00000264230	ENSP00000264995
ENSP00000264230	ENSP00000266544
ENSP00000264230	ENSP00000267425
ENSP00000264230	ENSP00000287038
ENSP00000264230	ENSP00000287295
ENSP00000264230	ENSP00000296411
ENSP00000264230	ENSP00000296802
ENSP00000264230	ENSP00000298746
ENSP00000264230	ENSP00000301364
ENSP00000264230	ENSP00000301585
ENSP00000264230	ENSP00000307525
ENSP00000264230	ENSP00000309830
ENSP00000264230	ENSP00000310042
ENSP00000264230	ENSP00000314132
ENSP00000264230	ENSP00000317992
ENSP00000264230	ENSP00000322396
ENSP00000264230	ENSP00000333837
ENSP00000264230	ENSP00000338862
ENSP00000264230	ENSP00000339145
ENSP00000264230	ENSP00000341692
ENSP00000264230	ENSP00000342181
ENSP00000264230	ENSP00000346725
ENSP00000264230	ENSP00000348596
ENSP00000264230	ENSP00000354040
ENSP00000264230	ENSP00000358400
ENSP00000264230	ENSP00000358563
ENSP00000264230	ENSP00000359345
ENSP00000264230	ENSP00000359688
ENSP00000264230	ENSP00000360412
ENSP00000264230	ENSP00000361982
ENSP00000264230	ENSP00000363384
ENSP00000264230	ENSP00000363559
ENSP00000264230	ENSP00000364037
ENSP00000264230	ENSP00000364320
ENSP00000264230	ENSP00000377640
ENSP00000264230	ENSP00000379888
ENSP00000264230	ENSP00000380982
ENSP00000264230	ENSP00000381793
ENSP00000264230	ENSP00000383402
ENSP00000264230	ENSP00000384490
ENSP00000264230	ENSP00000402338
ENSP00000264230	ENSP00000419740
ENSP00000264233	ENSP00000265081
ENSP00000264233	ENSP00000267812
ENSP00000264233	ENSP00000268206

ENSP00000264233	ENSP00000274712
ENSP00000264233	ENSP00000275820
ENSP00000264233	ENSP00000296805
ENSP00000264233	ENSP00000298746
ENSP00000264233	ENSP00000300738
ENSP00000264233	ENSP00000302160
ENSP00000264233	ENSP00000310448
ENSP00000264233	ENSP00000315379
ENSP00000264233	ENSP00000322570
ENSP00000264233	ENSP00000324122
ENSP00000264233	ENSP00000331087
ENSP00000264233	ENSP00000335321
ENSP00000264233	ENSP00000338788
ENSP00000264233	ENSP00000343557
ENSP00000264233	ENSP00000346566
ENSP00000264233	ENSP00000347232
ENSP00000264233	ENSP00000351137
ENSP00000264233	ENSP00000351492
ENSP00000264233	ENSP00000351697
ENSP00000264233	ENSP00000357631
ENSP00000264233	ENSP00000358554
ENSP00000264233	ENSP00000358563
ENSP00000264233	ENSP00000358810
ENSP00000264233	ENSP00000361310
ENSP00000264233	ENSP00000361982
ENSP00000264233	ENSP00000362110
ENSP00000264233	ENSP00000364289
ENSP00000264233	ENSP00000366557
ENSP00000264233	ENSP00000367835
ENSP00000264233	ENSP00000368349
ENSP00000264233	ENSP00000368565
ENSP00000264233	ENSP00000370589
ENSP00000264233	ENSP00000376973
ENSP00000264233	ENSP00000387006
ENSP00000264233	ENSP00000393953
ENSP00000264233	ENSP00000394649
ENSP00000264233	ENSP00000401566
ENSP00000264233	ENSP00000406868
ENSP00000264245	ENSP00000370719
ENSP00000264246	ENSP00000303939
ENSP00000264246	ENSP00000319476
ENSP00000264246	ENSP00000332049
ENSP00000264255	ENSP00000265462
ENSP00000264255	ENSP00000286428
ENSP00000264255	ENSP00000292180
ENSP00000264255	ENSP00000296577

ENSP00000264255	ENSP00000311343
ENSP00000264255	ENSP00000312735
ENSP00000264255	ENSP00000317334
ENSP00000264255	ENSP00000350512
ENSP00000264255	ENSP00000357651
ENSP00000264255	ENSP00000362110
ENSP00000264255	ENSP00000363734
ENSP00000264255	ENSP00000372694
ENSP00000264255	ENSP00000375881
ENSP00000264255	ENSP00000412319
ENSP00000264255	ENSP00000415678
ENSP00000264266	ENSP00000284320
ENSP00000264276	ENSP00000317955
ENSP00000264276	ENSP00000348577
ENSP00000264276	ENSP00000354739
ENSP00000264312	ENSP00000337396
ENSP00000264313	ENSP00000346566
ENSP00000264313	ENSP00000380921
ENSP00000264318	ENSP00000332258
ENSP00000264335	ENSP00000264705
ENSP00000264335	ENSP00000264839
ENSP00000264335	ENSP00000265727
ENSP00000264335	ENSP00000265960
ENSP00000264335	ENSP00000269122
ENSP00000264335	ENSP00000269397
ENSP00000264335	ENSP00000281282
ENSP00000264335	ENSP00000281623
ENSP00000264335	ENSP00000283928
ENSP00000264335	ENSP00000284776
ENSP00000264335	ENSP00000286627
ENSP00000264335	ENSP00000287038
ENSP00000264335	ENSP00000287878
ENSP00000264335	ENSP00000288602
ENSP00000264335	ENSP00000290429
ENSP00000264335	ENSP00000292055
ENSP00000264335	ENSP00000293406
ENSP00000264335	ENSP00000295756
ENSP00000264335	ENSP00000296474
ENSP00000264335	ENSP00000296509
ENSP00000264335	ENSP00000298552
ENSP00000264335	ENSP00000298852
ENSP00000264335	ENSP00000300249
ENSP00000264335	ENSP00000300574
ENSP00000264335	ENSP00000300738
ENSP00000264335	ENSP00000301280
ENSP00000264335	ENSP00000301740

ENSP00000264335	ENSP00000302239
ENSP00000264335	ENSP00000303507
ENSP00000264335	ENSP00000303928
ENSP00000264335	ENSP00000304350
ENSP00000264335	ENSP00000306275
ENSP00000264335	ENSP00000307272
ENSP00000264335	ENSP00000309103
ENSP00000264335	ENSP00000310129
ENSP00000264335	ENSP00000311005
ENSP00000264335	ENSP00000311430
ENSP00000264335	ENSP00000311827
ENSP00000264335	ENSP00000311873
ENSP00000264335	ENSP00000313059
ENSP00000264335	ENSP00000322570
ENSP00000264335	ENSP00000323780
ENSP00000264335	ENSP00000329380
ENSP00000264335	ENSP00000332513
ENSP00000264335	ENSP00000332721
ENSP00000264335	ENSP00000332931
ENSP00000264335	ENSP00000337439
ENSP00000264335	ENSP00000338345
ENSP00000264335	ENSP00000339186
ENSP00000264335	ENSP00000339527
ENSP00000264335	ENSP00000340019
ENSP00000264335	ENSP00000340237
ENSP00000264335	ENSP00000343144
ENSP00000264335	ENSP00000345530
ENSP00000264335	ENSP00000346067
ENSP00000264335	ENSP00000347345
ENSP00000264335	ENSP00000347978
ENSP00000264335	ENSP00000348157
ENSP00000264335	ENSP00000348442
ENSP00000264335	ENSP00000348551
ENSP00000264335	ENSP00000349959
ENSP00000264335	ENSP00000350249
ENSP00000264335	ENSP00000351284
ENSP00000264335	ENSP00000351908
ENSP00000264335	ENSP00000352738
ENSP00000264335	ENSP00000353622
ENSP00000264335	ENSP00000354497
ENSP00000264335	ENSP00000354718
ENSP00000264335	ENSP00000354876
ENSP00000264335	ENSP00000354916
ENSP00000264335	ENSP00000354927
ENSP00000264335	ENSP00000355596
ENSP00000264335	ENSP00000355884

ENSP00000264335	ENSP00000359531
ENSP00000264335	ENSP00000359567
ENSP00000264335	ENSP00000360532
ENSP00000264335	ENSP00000360621
ENSP00000264335	ENSP00000362399
ENSP00000264335	ENSP00000363021
ENSP00000264335	ENSP00000363433
ENSP00000264335	ENSP00000364929
ENSP00000264335	ENSP00000365016
ENSP00000264335	ENSP00000366863
ENSP00000264335	ENSP00000367766
ENSP00000264335	ENSP00000368438
ENSP00000264335	ENSP00000368976
ENSP00000264335	ENSP00000371734
ENSP00000264335	ENSP00000372023
ENSP00000264335	ENSP00000372857
ENSP00000264335	ENSP00000377496
ENSP00000264335	ENSP00000378529
ENSP00000264335	ENSP00000379607
ENSP00000264335	ENSP00000381293
ENSP00000264335	ENSP00000381932
ENSP00000264335	ENSP00000382177
ENSP00000264335	ENSP00000382423
ENSP00000264335	ENSP00000384675
ENSP00000264335	ENSP00000388731
ENSP00000264335	ENSP00000395574
ENSP00000264335	ENSP00000397598
ENSP00000264335	ENSP00000399863
ENSP00000264335	ENSP00000405963
ENSP00000264380	ENSP00000264639
ENSP00000264380	ENSP00000264705
ENSP00000264380	ENSP00000265970
ENSP00000264380	ENSP00000273398
ENSP00000264380	ENSP00000274680
ENSP00000264380	ENSP00000278616
ENSP00000264380	ENSP00000286317
ENSP00000264380	ENSP00000286428
ENSP00000264380	ENSP00000288828
ENSP00000264380	ENSP00000300648
ENSP00000264380	ENSP00000305682
ENSP00000264380	ENSP00000317334
ENSP00000264380	ENSP00000320309
ENSP00000264380	ENSP00000322957
ENSP00000264380	ENSP00000323377
ENSP00000264380	ENSP00000325448
ENSP00000264380	ENSP00000326921

ENSP00000264380	ENSP00000329558
ENSP00000264380	ENSP00000331201
ENSP00000264380	ENSP00000334188
ENSP00000264380	ENSP00000336842
ENSP00000264380	ENSP00000338093
ENSP00000264380	ENSP00000343741
ENSP00000264380	ENSP00000346022
ENSP00000264380	ENSP00000347498
ENSP00000264380	ENSP00000352121
ENSP00000264380	ENSP00000353904
ENSP00000264380	ENSP00000354340
ENSP00000264380	ENSP00000354519
ENSP00000264380	ENSP00000354522
ENSP00000264380	ENSP00000354558
ENSP00000264380	ENSP00000356989
ENSP00000264380	ENSP00000359067
ENSP00000264380	ENSP00000360473
ENSP00000264380	ENSP00000360798
ENSP00000264380	ENSP00000360992
ENSP00000264380	ENSP00000361287
ENSP00000264380	ENSP00000376946
ENSP00000264380	ENSP00000379500
ENSP00000264380	ENSP00000382177
ENSP00000264380	ENSP00000386621
ENSP00000264380	ENSP00000403852
ENSP00000264389	ENSP00000267540
ENSP00000264389	ENSP00000268638
ENSP00000264389	ENSP00000268717
ENSP00000264389	ENSP00000270708
ENSP00000264389	ENSP00000298852
ENSP00000264389	ENSP00000299259
ENSP00000264389	ENSP00000301764
ENSP00000264389	ENSP00000304102
ENSP00000264389	ENSP00000304822
ENSP00000264389	ENSP00000306245
ENSP00000264389	ENSP00000320648
ENSP00000264389	ENSP00000324444
ENSP00000264389	ENSP00000345144
ENSP00000264389	ENSP00000346340
ENSP00000264389	ENSP00000350512
ENSP00000264389	ENSP00000355958
ENSP00000264389	ENSP00000357674
ENSP00000264389	ENSP00000359206
ENSP00000264389	ENSP00000362314
ENSP00000264389	ENSP00000362334
ENSP00000264389	ENSP00000407401

ENSP00000264399	ENSP00000279230
ENSP00000264399	ENSP00000280362
ENSP00000264399	ENSP00000318351
ENSP00000264399	ENSP00000346725
ENSP00000264399	ENSP00000353452
ENSP00000264399	ENSP00000361465
ENSP00000264399	ENSP00000361650
ENSP00000264399	ENSP00000365550
ENSP00000264399	ENSP00000403852
ENSP00000264447	ENSP00000326261
ENSP00000264447	ENSP00000329466
ENSP00000264447	ENSP00000342300
ENSP00000264447	ENSP00000354848
ENSP00000264447	ENSP00000360532
ENSP00000264448	ENSP00000274473
ENSP00000264451	ENSP00000330343
ENSP00000264451	ENSP00000350708
ENSP00000264451	ENSP00000399968
ENSP00000264474	ENSP00000274030
ENSP00000264474	ENSP00000301607
ENSP00000264474	ENSP00000357731
ENSP00000264474	ENSP00000357753
ENSP00000264474	ENSP00000379607
ENSP00000264497	ENSP00000338010
ENSP00000264499	ENSP00000268057
ENSP00000264499	ENSP00000295240
ENSP00000264499	ENSP00000296135
ENSP00000264499	ENSP00000317334
ENSP00000264499	ENSP00000319062
ENSP00000264499	ENSP00000334100
ENSP00000264499	ENSP00000353701
ENSP00000264499	ENSP00000370031
ENSP00000264499	ENSP00000376946
ENSP00000264501	ENSP00000264515
ENSP00000264501	ENSP00000265070
ENSP00000264501	ENSP00000273064
ENSP00000264501	ENSP00000276416
ENSP00000264501	ENSP00000283351
ENSP00000264501	ENSP00000291688
ENSP00000264501	ENSP00000299626
ENSP00000264501	ENSP00000304593
ENSP00000264501	ENSP00000306124
ENSP00000264501	ENSP00000312244
ENSP00000264501	ENSP00000319979
ENSP00000264501	ENSP00000320566
ENSP00000264501	ENSP00000321711

ENSP00000264501	ENSP00000325677
ENSP00000264501	ENSP00000326699
ENSP00000264501	ENSP00000331310
ENSP00000264501	ENSP00000331719
ENSP00000264501	ENSP00000334188
ENSP00000264501	ENSP00000340271
ENSP00000264501	ENSP00000346809
ENSP00000264501	ENSP00000347602
ENSP00000264501	ENSP00000348107
ENSP00000264501	ENSP00000348602
ENSP00000264501	ENSP00000349877
ENSP00000264501	ENSP00000351947
ENSP00000264501	ENSP00000353622
ENSP00000264501	ENSP00000354522
ENSP00000264501	ENSP00000356530
ENSP00000264501	ENSP00000359518
ENSP00000264501	ENSP00000359552
ENSP00000264501	ENSP00000360473
ENSP00000264501	ENSP00000364929
ENSP00000264501	ENSP00000374213
ENSP00000264501	ENSP00000380460
ENSP00000264501	ENSP00000380793
ENSP00000264501	ENSP00000401445
ENSP00000264501	ENSP00000402240
ENSP00000264501	ENSP00000415203
ENSP00000264515	ENSP00000265351
ENSP00000264515	ENSP00000267163
ENSP00000264515	ENSP00000267197
ENSP00000264515	ENSP00000271555
ENSP00000264515	ENSP00000273067
ENSP00000264515	ENSP00000274140
ENSP00000264515	ENSP00000283882
ENSP00000264515	ENSP00000285873
ENSP00000264515	ENSP00000286317
ENSP00000264515	ENSP00000287820
ENSP00000264515	ENSP00000295066
ENSP00000264515	ENSP00000296490
ENSP00000264515	ENSP00000296786
ENSP00000264515	ENSP00000298937
ENSP00000264515	ENSP00000299601
ENSP00000264515	ENSP00000301764
ENSP00000264515	ENSP00000302239
ENSP00000264515	ENSP00000302790
ENSP00000264515	ENSP00000303584
ENSP00000264515	ENSP00000304593
ENSP00000264515	ENSP00000306983

ENSP00000264515	ENSP00000309141
ENSP00000264515	ENSP00000309555
ENSP00000264515	ENSP00000311496
ENSP00000264515	ENSP00000317087
ENSP00000264515	ENSP00000317334
ENSP00000264515	ENSP00000321617
ENSP00000264515	ENSP00000322242
ENSP00000264515	ENSP00000322579
ENSP00000264515	ENSP00000323302
ENSP00000264515	ENSP00000325326
ENSP00000264515	ENSP00000325377
ENSP00000264515	ENSP00000325677
ENSP00000264515	ENSP00000326519
ENSP00000264515	ENSP00000327716
ENSP00000264515	ENSP00000329967
ENSP00000264515	ENSP00000334130
ENSP00000264515	ENSP00000337088
ENSP00000264515	ENSP00000337405
ENSP00000264515	ENSP00000340766
ENSP00000264515	ENSP00000340896
ENSP00000264515	ENSP00000344456
ENSP00000264515	ENSP00000350967
ENSP00000264515	ENSP00000351894
ENSP00000264515	ENSP00000352820
ENSP00000264515	ENSP00000352821
ENSP00000264515	ENSP00000353059
ENSP00000264515	ENSP00000353622
ENSP00000264515	ENSP00000353793
ENSP00000264515	ENSP00000355205
ENSP00000264515	ENSP00000356405
ENSP00000264515	ENSP00000356989
ENSP00000264515	ENSP00000357442
ENSP00000264515	ENSP00000357887
ENSP00000264515	ENSP00000358064
ENSP00000264515	ENSP00000360515
ENSP00000264515	ENSP00000360532
ENSP00000264515	ENSP00000360621
ENSP00000264515	ENSP00000362532
ENSP00000264515	ENSP00000363313
ENSP00000264515	ENSP00000363779
ENSP00000264515	ENSP00000363787
ENSP00000264515	ENSP00000365891
ENSP00000264515	ENSP00000366953
ENSP00000264515	ENSP00000366974
ENSP00000264515	ENSP00000367029
ENSP00000264515	ENSP00000372699

ENSP00000264515	ENSP00000374213
ENSP00000264515	ENSP00000374280
ENSP00000264515	ENSP00000374529
ENSP00000264515	ENSP00000379217
ENSP00000264515	ENSP00000380460
ENSP00000264515	ENSP00000382779
ENSP00000264515	ENSP00000384048
ENSP00000264515	ENSP00000384109
ENSP00000264515	ENSP00000386759
ENSP00000264515	ENSP00000390475
ENSP00000264515	ENSP00000396271
ENSP00000264515	ENSP00000396439
ENSP00000264515	ENSP00000400572
ENSP00000264515	ENSP00000401445
ENSP00000264515	ENSP00000402240
ENSP00000264515	ENSP00000406293
ENSP00000264515	ENSP00000412190
ENSP00000264552	ENSP00000265388
ENSP00000264552	ENSP00000271843
ENSP00000264552	ENSP00000281043
ENSP00000264552	ENSP00000288774
ENSP00000264552	ENSP00000298585
ENSP00000264552	ENSP00000300738
ENSP00000264552	ENSP00000308546
ENSP00000264552	ENSP00000311010
ENSP00000264552	ENSP00000311280
ENSP00000264552	ENSP00000331487
ENSP00000264552	ENSP00000338413
ENSP00000264552	ENSP00000339381
ENSP00000264552	ENSP00000340820
ENSP00000264552	ENSP00000342755
ENSP00000264552	ENSP00000343535
ENSP00000264552	ENSP00000345656
ENSP00000264552	ENSP00000348632
ENSP00000264552	ENSP00000352673
ENSP00000264552	ENSP00000352676
ENSP00000264552	ENSP00000353375
ENSP00000264552	ENSP00000353508
ENSP00000264552	ENSP00000360532
ENSP00000264552	ENSP00000363089
ENSP00000264552	ENSP00000378529
ENSP00000264563	ENSP00000338799
ENSP00000264595	ENSP00000295897
ENSP00000264595	ENSP00000349437
ENSP00000264596	ENSP00000302896
ENSP00000264596	ENSP00000326981

ENSP00000264605	ENSP00000382177
ENSP00000264607	ENSP00000273480
ENSP00000264607	ENSP00000284811
ENSP00000264637	ENSP00000267622
ENSP00000264637	ENSP00000268058
ENSP00000264637	ENSP00000271002
ENSP00000264637	ENSP00000282892
ENSP00000264637	ENSP00000290663
ENSP00000264637	ENSP00000299259
ENSP00000264637	ENSP00000300651
ENSP00000264637	ENSP00000313420
ENSP00000264637	ENSP00000322457
ENSP00000264637	ENSP00000323880
ENSP00000264637	ENSP00000325612
ENSP00000264637	ENSP00000326767
ENSP00000264637	ENSP00000327213
ENSP00000264637	ENSP00000341692
ENSP00000264637	ENSP00000348551
ENSP00000264637	ENSP00000348610
ENSP00000264637	ENSP00000351894
ENSP00000264637	ENSP00000352257
ENSP00000264637	ENSP00000354633
ENSP00000264637	ENSP00000354718
ENSP00000264637	ENSP00000355759
ENSP00000264637	ENSP00000359531
ENSP00000264637	ENSP00000363193
ENSP00000264637	ENSP00000363812
ENSP00000264637	ENSP00000365877
ENSP00000264637	ENSP00000367787
ENSP00000264637	ENSP00000370826
ENSP00000264637	ENSP00000376611
ENSP00000264637	ENSP00000378286
ENSP00000264637	ENSP00000381098
ENSP00000264637	ENSP00000399968
ENSP00000264638	ENSP00000300574
ENSP00000264638	ENSP00000337838
ENSP00000264638	ENSP00000353154
ENSP00000264638	ENSP00000367408
ENSP00000264639	ENSP00000265728
ENSP00000264639	ENSP00000276326
ENSP00000264639	ENSP00000288680
ENSP00000264639	ENSP00000290330
ENSP00000264639	ENSP00000290541
ENSP00000264639	ENSP00000290974
ENSP00000264639	ENSP00000295901
ENSP00000264639	ENSP00000296543

ENSP00000264639	ENSP00000296786
ENSP00000264639	ENSP00000298852
ENSP00000264639	ENSP00000299259
ENSP00000264639	ENSP00000309474
ENSP00000264639	ENSP00000310129
ENSP00000264639	ENSP00000311083
ENSP00000264639	ENSP00000311665
ENSP00000264639	ENSP00000319977
ENSP00000264639	ENSP00000321711
ENSP00000264639	ENSP00000323663
ENSP00000264639	ENSP00000325376
ENSP00000264639	ENSP00000341213
ENSP00000264639	ENSP00000346022
ENSP00000264639	ENSP00000346236
ENSP00000264639	ENSP00000347232
ENSP00000264639	ENSP00000348442
ENSP00000264639	ENSP00000350708
ENSP00000264639	ENSP00000356602
ENSP00000264639	ENSP00000357980
ENSP00000264639	ENSP00000360183
ENSP00000264639	ENSP00000360532
ENSP00000264639	ENSP00000362334
ENSP00000264639	ENSP00000362344
ENSP00000264639	ENSP00000364591
ENSP00000264639	ENSP00000369497
ENSP00000264639	ENSP00000376758
ENSP00000264639	ENSP00000381045
ENSP00000264639	ENSP00000381237
ENSP00000264639	ENSP00000382595
ENSP00000264639	ENSP00000384211
ENSP00000264639	ENSP00000386212
ENSP00000264639	ENSP00000396937
ENSP00000264645	ENSP00000276201
ENSP00000264645	ENSP00000294785
ENSP00000264645	ENSP00000345793
ENSP00000264645	ENSP00000351524
ENSP00000264645	ENSP00000358799
ENSP00000264645	ENSP00000360922
ENSP00000264645	ENSP00000361949
ENSP00000264645	ENSP00000363745
ENSP00000264645	ENSP00000366607
ENSP00000264645	ENSP00000379607
ENSP00000264663	ENSP00000329466
ENSP00000264663	ENSP00000349977
ENSP00000264670	ENSP00000264951
ENSP00000264670	ENSP00000264995

ENSP00000264670	ENSP00000265245
ENSP00000264670	ENSP00000265849
ENSP00000264670	ENSP00000274606
ENSP00000264670	ENSP00000296930
ENSP00000264670	ENSP00000297990
ENSP00000264670	ENSP00000298937
ENSP00000264670	ENSP00000300648
ENSP00000264670	ENSP00000302768
ENSP00000264670	ENSP00000302886
ENSP00000264670	ENSP00000302896
ENSP00000264670	ENSP00000309433
ENSP00000264670	ENSP00000314311
ENSP00000264670	ENSP00000314441
ENSP00000264670	ENSP00000316879
ENSP00000264670	ENSP00000317992
ENSP00000264670	ENSP00000318195
ENSP00000264670	ENSP00000322396
ENSP00000264670	ENSP00000322419
ENSP00000264670	ENSP00000324122
ENSP00000264670	ENSP00000328671
ENSP00000264670	ENSP00000329466
ENSP00000264670	ENSP00000333934
ENSP00000264670	ENSP00000334188
ENSP00000264670	ENSP00000334836
ENSP00000264670	ENSP00000338573
ENSP00000264670	ENSP00000338862
ENSP00000264670	ENSP00000339145
ENSP00000264670	ENSP00000339795
ENSP00000264670	ENSP00000342181
ENSP00000264670	ENSP00000345096
ENSP00000264670	ENSP00000346725
ENSP00000264670	ENSP00000354040
ENSP00000264670	ENSP00000355086
ENSP00000264670	ENSP00000356290
ENSP00000264670	ENSP00000356548
ENSP00000264670	ENSP00000356989
ENSP00000264670	ENSP00000358812
ENSP00000264670	ENSP00000359345
ENSP00000264670	ENSP00000359688
ENSP00000264670	ENSP00000360412
ENSP00000264670	ENSP00000362153
ENSP00000264670	ENSP00000363018
ENSP00000264670	ENSP00000363559
ENSP00000264670	ENSP00000363676
ENSP00000264670	ENSP00000364119
ENSP00000264670	ENSP00000364320

ENSP00000264670	ENSP00000365806
ENSP00000264670	ENSP00000366135
ENSP00000264670	ENSP00000366557
ENSP00000264670	ENSP00000370589
ENSP00000264670	ENSP00000370741
ENSP00000264670	ENSP00000373090
ENSP00000264670	ENSP00000377640
ENSP00000264670	ENSP00000380982
ENSP00000264670	ENSP00000386456
ENSP00000264670	ENSP00000394290
ENSP00000264670	ENSP00000401508
ENSP00000264670	ENSP00000402338
ENSP00000264670	ENSP00000403576
ENSP00000264670	ENSP00000409074
ENSP00000264670	ENSP00000409367
ENSP00000264670	ENSP00000411162
ENSP00000264670	ENSP00000420357
ENSP00000264689	ENSP00000287859
ENSP00000264689	ENSP00000380349
ENSP00000264690	ENSP00000265023
ENSP00000264690	ENSP00000372224
ENSP00000264703	ENSP00000301788
ENSP00000264705	ENSP00000264731
ENSP00000264705	ENSP00000265970
ENSP00000264705	ENSP00000266095
ENSP00000264705	ENSP00000266126
ENSP00000264705	ENSP00000267845
ENSP00000264705	ENSP00000269122
ENSP00000264705	ENSP00000275015
ENSP00000264705	ENSP00000286835
ENSP00000264705	ENSP00000296255
ENSP00000264705	ENSP00000305958
ENSP00000264705	ENSP00000307567
ENSP00000264705	ENSP00000309830
ENSP00000264705	ENSP00000311344
ENSP00000264705	ENSP00000313851
ENSP00000264705	ENSP00000315925
ENSP00000264705	ENSP00000317334
ENSP00000264705	ENSP00000321606
ENSP00000264705	ENSP00000323856
ENSP00000264705	ENSP00000325690
ENSP00000264705	ENSP00000326699
ENSP00000264705	ENSP00000328671
ENSP00000264705	ENSP00000330484
ENSP00000264705	ENSP00000339435
ENSP00000264705	ENSP00000339484

ENSP00000264705	ENSP00000340019
ENSP00000264705	ENSP00000343103
ENSP00000264705	ENSP00000344055
ENSP00000264705	ENSP00000346067
ENSP00000264705	ENSP00000347495
ENSP00000264705	ENSP00000347997
ENSP00000264705	ENSP00000348708
ENSP00000264705	ENSP00000350199
ENSP00000264705	ENSP00000350708
ENSP00000264705	ENSP00000350928
ENSP00000264705	ENSP00000354347
ENSP00000264705	ENSP00000354522
ENSP00000264705	ENSP00000355890
ENSP00000264705	ENSP00000356146
ENSP00000264705	ENSP00000358400
ENSP00000264705	ENSP00000358423
ENSP00000264705	ENSP00000359096
ENSP00000264705	ENSP00000359100
ENSP00000264705	ENSP00000360268
ENSP00000264705	ENSP00000360327
ENSP00000264705	ENSP00000361699
ENSP00000264705	ENSP00000362298
ENSP00000264705	ENSP00000363205
ENSP00000264705	ENSP00000363676
ENSP00000264705	ENSP00000364119
ENSP00000264705	ENSP00000364895
ENSP00000264705	ENSP00000365534
ENSP00000264705	ENSP00000366249
ENSP00000264705	ENSP00000366974
ENSP00000264705	ENSP00000368699
ENSP00000264705	ENSP00000369162
ENSP00000264705	ENSP00000369456
ENSP00000264705	ENSP00000370517
ENSP00000264705	ENSP00000370589
ENSP00000264705	ENSP00000370883
ENSP00000264705	ENSP00000373039
ENSP00000264705	ENSP00000376946
ENSP00000264705	ENSP00000377527
ENSP00000264705	ENSP00000394869
ENSP00000264705	ENSP00000396152
ENSP00000264705	ENSP00000408058
ENSP00000264705	ENSP00000410416
ENSP00000264708	ENSP00000306100
ENSP00000264709	ENSP00000267163
ENSP00000264709	ENSP00000269397
ENSP00000264709	ENSP00000276893

ENSP00000264709	ENSP00000281043
ENSP00000264709	ENSP00000300086
ENSP00000264709	ENSP00000308750
ENSP00000264709	ENSP00000319169
ENSP00000264709	ENSP00000327213
ENSP00000264709	ENSP00000343535
ENSP00000264709	ENSP00000352516
ENSP00000264709	ENSP00000355388
ENSP00000264709	ENSP00000357965
ENSP00000264709	ENSP00000364895
ENSP00000264709	ENSP00000365877
ENSP00000264709	ENSP00000379888
ENSP00000264709	ENSP00000404658
ENSP00000264709	ENSP00000417980
ENSP00000264717	ENSP00000350512
ENSP00000264717	ENSP00000357965
ENSP00000264718	ENSP00000265351
ENSP00000264718	ENSP00000297185
ENSP00000264718	ENSP00000299853
ENSP00000264718	ENSP00000301764
ENSP00000264718	ENSP00000302790
ENSP00000264718	ENSP00000306123
ENSP00000264718	ENSP00000307491
ENSP00000264718	ENSP00000309474
ENSP00000264718	ENSP00000310129
ENSP00000264718	ENSP00000312735
ENSP00000264718	ENSP00000314949
ENSP00000264718	ENSP00000317334
ENSP00000264718	ENSP00000322016
ENSP00000264718	ENSP00000340019
ENSP00000264718	ENSP00000353698
ENSP00000264718	ENSP00000363250
ENSP00000264718	ENSP00000364270
ENSP00000264718	ENSP00000370616
ENSP00000264718	ENSP00000372199
ENSP00000264718	ENSP00000376097
ENSP00000264718	ENSP00000406888
ENSP00000264720	ENSP00000267163
ENSP00000264720	ENSP00000307272
ENSP00000264720	ENSP00000334564
ENSP00000264720	ENSP00000348510
ENSP00000264720	ENSP00000351947
ENSP00000264720	ENSP00000354558
ENSP00000264720	ENSP00000360532
ENSP00000264720	ENSP00000361180
ENSP00000264720	ENSP00000361219

ENSP00000264723	ENSP00000357484
ENSP00000264731	ENSP00000265148
ENSP00000264731	ENSP00000265983
ENSP00000264731	ENSP00000268699
ENSP00000264731	ENSP00000281708
ENSP00000264731	ENSP00000282050
ENSP00000264731	ENSP00000282441
ENSP00000264731	ENSP00000285398
ENSP00000264731	ENSP00000286835
ENSP00000264731	ENSP00000300231
ENSP00000264731	ENSP00000301019
ENSP00000264731	ENSP00000301764
ENSP00000264731	ENSP00000304102
ENSP00000264731	ENSP00000304592
ENSP00000264731	ENSP00000307684
ENSP00000264731	ENSP00000307858
ENSP00000264731	ENSP00000310129
ENSP00000264731	ENSP00000310723
ENSP00000264731	ENSP00000314556
ENSP00000264731	ENSP00000317334
ENSP00000264731	ENSP00000317872
ENSP00000264731	ENSP00000325690
ENSP00000264731	ENSP00000326550
ENSP00000264731	ENSP00000327145
ENSP00000264731	ENSP00000333934
ENSP00000264731	ENSP00000335371
ENSP00000264731	ENSP00000341957
ENSP00000264731	ENSP00000346067
ENSP00000264731	ENSP00000347733
ENSP00000264731	ENSP00000348163
ENSP00000264731	ENSP00000348708
ENSP00000264731	ENSP00000350283
ENSP00000264731	ENSP00000353735
ENSP00000264731	ENSP00000354218
ENSP00000264731	ENSP00000354478
ENSP00000264731	ENSP00000354486
ENSP00000264731	ENSP00000354739
ENSP00000264731	ENSP00000355153
ENSP00000264731	ENSP00000355231
ENSP00000264731	ENSP00000356379
ENSP00000264731	ENSP00000356520
ENSP00000264731	ENSP00000359206
ENSP00000264731	ENSP00000363745
ENSP00000264731	ENSP00000363899
ENSP00000264731	ENSP00000364929
ENSP00000264731	ENSP00000365439

ENSP00000264731	ENSP00000367265
ENSP00000264731	ENSP00000371729
ENSP00000264731	ENSP00000372023
ENSP00000264731	ENSP00000375730
ENSP00000264731	ENSP00000376309
ENSP00000264731	ENSP00000377640
ENSP00000264731	ENSP00000382834
ENSP00000264731	ENSP00000391774
ENSP00000264731	ENSP00000392617
ENSP00000264731	ENSP00000396876
ENSP00000264731	ENSP00000417281
ENSP00000264734	ENSP00000281537
ENSP00000264748	ENSP00000343785
ENSP00000264748	ENSP00000363571
ENSP00000264758	ENSP00000270257
ENSP00000264758	ENSP00000353622
ENSP00000264758	ENSP00000361824
ENSP00000264758	ENSP00000387019
ENSP00000264790	ENSP00000355153
ENSP00000264818	ENSP00000270139
ENSP00000264818	ENSP00000303939
ENSP00000264818	ENSP00000305133
ENSP00000264818	ENSP00000315768
ENSP00000264818	ENSP00000319169
ENSP00000264818	ENSP00000338799
ENSP00000264818	ENSP00000339328
ENSP00000264818	ENSP00000340944
ENSP00000264818	ENSP00000343957
ENSP00000264818	ENSP00000350967
ENSP00000264818	ENSP00000356346
ENSP00000264818	ENSP00000360730
ENSP00000264818	ENSP00000365016
ENSP00000264818	ENSP00000416583
ENSP00000264834	ENSP00000265963
ENSP00000264834	ENSP00000267064
ENSP00000264834	ENSP00000320485
ENSP00000264834	ENSP00000323967
ENSP00000264834	ENSP00000334061
ENSP00000264834	ENSP00000353622
ENSP00000264839	ENSP00000296452
ENSP00000264839	ENSP00000353362
ENSP00000264839	ENSP00000354158
ENSP00000264839	ENSP00000373600
ENSP00000264839	ENSP00000380271
ENSP00000264864	ENSP00000265970
ENSP00000264864	ENSP00000296154

ENSP00000264864	ENSP00000354782
ENSP00000264864	ENSP00000365891
ENSP00000264883	ENSP00000265351
ENSP00000264883	ENSP00000265388
ENSP00000264883	ENSP00000269122
ENSP00000264883	ENSP00000272645
ENSP00000264883	ENSP00000283195
ENSP00000264883	ENSP00000285968
ENSP00000264883	ENSP00000290158
ENSP00000264883	ENSP00000290231
ENSP00000264883	ENSP00000291688
ENSP00000264883	ENSP00000296215
ENSP00000264883	ENSP00000298937
ENSP00000264883	ENSP00000301329
ENSP00000264883	ENSP00000302790
ENSP00000264883	ENSP00000302886
ENSP00000264883	ENSP00000305503
ENSP00000264883	ENSP00000307939
ENSP00000264883	ENSP00000308622
ENSP00000264883	ENSP00000310668
ENSP00000264883	ENSP00000311768
ENSP00000264883	ENSP00000316032
ENSP00000264883	ENSP00000331310
ENSP00000264883	ENSP00000334061
ENSP00000264883	ENSP00000336712
ENSP00000264883	ENSP00000343701
ENSP00000264883	ENSP00000346120
ENSP00000264883	ENSP00000346453
ENSP00000264883	ENSP00000350512
ENSP00000264883	ENSP00000352021
ENSP00000264883	ENSP00000356548
ENSP00000264883	ENSP00000360286
ENSP00000264883	ENSP00000362463
ENSP00000264883	ENSP00000364293
ENSP00000264883	ENSP00000371155
ENSP00000264883	ENSP00000386992
ENSP00000264883	ENSP00000403536
ENSP00000264883	ENSP00000404151
ENSP00000264917	ENSP00000283875
ENSP00000264917	ENSP00000286835
ENSP00000264917	ENSP00000294016
ENSP00000264917	ENSP00000295890
ENSP00000264917	ENSP00000297338
ENSP00000264917	ENSP00000344874
ENSP00000264917	ENSP00000351015
ENSP00000264917	ENSP00000356146

ENSP00000264917	ENSP00000356405
ENSP00000264917	ENSP00000356825
ENSP00000264917	ENSP00000379607
ENSP00000264917	ENSP00000419361
ENSP00000264926	ENSP00000265038
ENSP00000264926	ENSP00000265140
ENSP00000264926	ENSP00000265433
ENSP00000264926	ENSP00000265728
ENSP00000264926	ENSP00000265970
ENSP00000264926	ENSP00000267430
ENSP00000264926	ENSP00000268043
ENSP00000264926	ENSP00000268717
ENSP00000264926	ENSP00000269214
ENSP00000264926	ENSP00000278616
ENSP00000264926	ENSP00000282077
ENSP00000264926	ENSP00000284811
ENSP00000264926	ENSP00000285021
ENSP00000264926	ENSP00000287437
ENSP00000264926	ENSP00000287647
ENSP00000264926	ENSP00000295050
ENSP00000264926	ENSP00000295926
ENSP00000264926	ENSP00000296257
ENSP00000264926	ENSP00000296273
ENSP00000264926	ENSP00000296792
ENSP00000264926	ENSP00000298139
ENSP00000264926	ENSP00000301671
ENSP00000264926	ENSP00000301740
ENSP00000264926	ENSP00000301764
ENSP00000264926	ENSP00000302120
ENSP00000264926	ENSP00000302160
ENSP00000264926	ENSP00000302955
ENSP00000264926	ENSP00000306561
ENSP00000264926	ENSP00000306999
ENSP00000264926	ENSP00000307491
ENSP00000264926	ENSP00000307853
ENSP00000264926	ENSP00000308179
ENSP00000264926	ENSP00000308351
ENSP00000264926	ENSP00000308944
ENSP00000264926	ENSP00000310520
ENSP00000264926	ENSP00000310842
ENSP00000264926	ENSP00000311873
ENSP00000264926	ENSP00000313171
ENSP00000264926	ENSP00000313420
ENSP00000264926	ENSP00000314193
ENSP00000264926	ENSP00000315700
ENSP00000264926	ENSP00000317334

ENSP00000264926	ENSP00000317636
ENSP00000264926	ENSP00000318977
ENSP00000264926	ENSP00000322570
ENSP00000264926	ENSP00000323439
ENSP00000264926	ENSP00000323663
ENSP00000264926	ENSP00000325677
ENSP00000264926	ENSP00000325863
ENSP00000264926	ENSP00000327197
ENSP00000264926	ENSP00000334474
ENSP00000264926	ENSP00000336868
ENSP00000264926	ENSP00000340019
ENSP00000264926	ENSP00000340879
ENSP00000264926	ENSP00000341382
ENSP00000264926	ENSP00000342026
ENSP00000264926	ENSP00000343392
ENSP00000264926	ENSP00000343741
ENSP00000264926	ENSP00000343745
ENSP00000264926	ENSP00000347168
ENSP00000264926	ENSP00000347232
ENSP00000264926	ENSP00000347733
ENSP00000264926	ENSP00000347978
ENSP00000264926	ENSP00000350767
ENSP00000264926	ENSP00000351284
ENSP00000264926	ENSP00000351697
ENSP00000264926	ENSP00000352121
ENSP00000264926	ENSP00000353332
ENSP00000264926	ENSP00000353793
ENSP00000264926	ENSP00000354558
ENSP00000264926	ENSP00000354720
ENSP00000264926	ENSP00000354957
ENSP00000264926	ENSP00000355028
ENSP00000264926	ENSP00000355077
ENSP00000264926	ENSP00000355958
ENSP00000264926	ENSP00000358019
ENSP00000264926	ENSP00000358862
ENSP00000264926	ENSP00000360532
ENSP00000264926	ENSP00000361310
ENSP00000264926	ENSP00000361990
ENSP00000264926	ENSP00000363021
ENSP00000264926	ENSP00000364270
ENSP00000264926	ENSP00000364813
ENSP00000264926	ENSP00000365588
ENSP00000264926	ENSP00000366974
ENSP00000264926	ENSP00000367034
ENSP00000264926	ENSP00000368349
ENSP00000264926	ENSP00000368438

ENSP00000264926	ENSP00000368646
ENSP00000264926	ENSP00000369335
ENSP00000264926	ENSP00000369411
ENSP00000264926	ENSP00000370150
ENSP00000264926	ENSP00000370151
ENSP00000264926	ENSP00000371475
ENSP00000264926	ENSP00000372023
ENSP00000264926	ENSP00000372316
ENSP00000264926	ENSP00000372883
ENSP00000264926	ENSP00000373060
ENSP00000264926	ENSP00000374529
ENSP00000264926	ENSP00000375809
ENSP00000264926	ENSP00000377655
ENSP00000264926	ENSP00000380460
ENSP00000264926	ENSP00000380504
ENSP00000264926	ENSP00000381657
ENSP00000264926	ENSP00000382595
ENSP00000264926	ENSP00000382779
ENSP00000264926	ENSP00000384048
ENSP00000264926	ENSP00000386212
ENSP00000264926	ENSP00000387429
ENSP00000264926	ENSP00000394791
ENSP00000264926	ENSP00000396068
ENSP00000264926	ENSP00000396484
ENSP00000264926	ENSP00000402515
ENSP00000264926	ENSP00000402527
ENSP00000264926	ENSP00000403345
ENSP00000264926	ENSP00000404318
ENSP00000264926	ENSP00000406280
ENSP00000264926	ENSP00000407964
ENSP00000264926	ENSP00000408994
ENSP00000264926	ENSP00000409167
ENSP00000264926	ENSP00000410758
ENSP00000264926	ENSP00000414006
ENSP00000264926	ENSP00000414634
ENSP00000264926	ENSP00000415212
ENSP00000264926	ENSP00000416511
ENSP00000264932	ENSP00000297185
ENSP00000264932	ENSP00000301329
ENSP00000264932	ENSP00000301585
ENSP00000264932	ENSP00000301761
ENSP00000264932	ENSP00000329466
ENSP00000264932	ENSP00000333667
ENSP00000264932	ENSP00000340019
ENSP00000264932	ENSP00000355518
ENSP00000264932	ENSP00000356953

ENSP00000264932	ENSP00000359211
ENSP00000264932	ENSP00000364649
ENSP00000264932	ENSP00000364699
ENSP00000264932	ENSP00000366482
ENSP00000264932	ENSP00000379607
ENSP00000264932	ENSP00000387262
ENSP00000264935	ENSP00000289431
ENSP00000264935	ENSP00000327077
ENSP00000264935	ENSP00000338157
ENSP00000264935	ENSP00000370410
ENSP00000264951	ENSP00000265990
ENSP00000264951	ENSP00000267197
ENSP00000264951	ENSP00000268533
ENSP00000264951	ENSP00000268661
ENSP00000264951	ENSP00000272444
ENSP00000264951	ENSP00000274311
ENSP00000264951	ENSP00000276201
ENSP00000264951	ENSP00000280665
ENSP00000264951	ENSP00000282884
ENSP00000264951	ENSP00000285873
ENSP00000264951	ENSP00000286835
ENSP00000264951	ENSP00000296328
ENSP00000264951	ENSP00000296783
ENSP00000264951	ENSP00000296802
ENSP00000264951	ENSP00000298746
ENSP00000264951	ENSP00000298767
ENSP00000264951	ENSP00000299138
ENSP00000264951	ENSP00000300146
ENSP00000264951	ENSP00000300413
ENSP00000264951	ENSP00000301788
ENSP00000264951	ENSP00000302160
ENSP00000264951	ENSP00000302768
ENSP00000264951	ENSP00000302896
ENSP00000264951	ENSP00000304593
ENSP00000264951	ENSP00000305702
ENSP00000264951	ENSP00000307525
ENSP00000264951	ENSP00000307863
ENSP00000264951	ENSP00000310596
ENSP00000264951	ENSP00000311430
ENSP00000264951	ENSP00000313350
ENSP00000264951	ENSP00000314311
ENSP00000264951	ENSP00000314348
ENSP00000264951	ENSP00000314441
ENSP00000264951	ENSP00000314949
ENSP00000264951	ENSP00000316879
ENSP00000264951	ENSP00000318195

ENSP00000264951	ENSP00000318604
ENSP00000264951	ENSP00000319341
ENSP00000264951	ENSP00000320566
ENSP00000264951	ENSP00000321711
ENSP00000264951	ENSP00000323302
ENSP00000264951	ENSP00000324122
ENSP00000264951	ENSP00000325376
ENSP00000264951	ENSP00000326806
ENSP00000264951	ENSP00000326981
ENSP00000264951	ENSP00000328464
ENSP00000264951	ENSP00000328690
ENSP00000264951	ENSP00000329466
ENSP00000264951	ENSP00000331817
ENSP00000264951	ENSP00000334044
ENSP00000264951	ENSP00000334188
ENSP00000264951	ENSP00000336927
ENSP00000264951	ENSP00000337839
ENSP00000264951	ENSP00000339484
ENSP00000264951	ENSP00000339503
ENSP00000264951	ENSP00000340281
ENSP00000264951	ENSP00000340766
ENSP00000264951	ENSP00000341743
ENSP00000264951	ENSP00000344220
ENSP00000264951	ENSP00000345096
ENSP00000264951	ENSP00000345917
ENSP00000264951	ENSP00000347087
ENSP00000264951	ENSP00000348708
ENSP00000264951	ENSP00000351596
ENSP00000264951	ENSP00000351665
ENSP00000264951	ENSP00000352021
ENSP00000264951	ENSP00000352820
ENSP00000264951	ENSP00000353059
ENSP00000264951	ENSP00000354125
ENSP00000264951	ENSP00000354340
ENSP00000264951	ENSP00000354522
ENSP00000264951	ENSP00000354673
ENSP00000264951	ENSP00000354972
ENSP00000264951	ENSP00000355261
ENSP00000264951	ENSP00000355541
ENSP00000264951	ENSP00000356104
ENSP00000264951	ENSP00000356146
ENSP00000264951	ENSP00000356405
ENSP00000264951	ENSP00000356579
ENSP00000264951	ENSP00000356811
ENSP00000264951	ENSP00000358497
ENSP00000264951	ENSP00000358563

ENSP00000264951	ENSP00000359345
ENSP00000264951	ENSP00000359939
ENSP00000264951	ENSP00000360473
ENSP00000264951	ENSP00000361076
ENSP00000264951	ENSP00000361465
ENSP00000264951	ENSP00000361949
ENSP00000264951	ENSP00000361982
ENSP00000264951	ENSP00000362166
ENSP00000264951	ENSP00000362532
ENSP00000264951	ENSP00000362744
ENSP00000264951	ENSP00000362900
ENSP00000264951	ENSP00000362936
ENSP00000264951	ENSP00000363313
ENSP00000264951	ENSP00000363944
ENSP00000264951	ENSP00000364543
ENSP00000264951	ENSP00000364813
ENSP00000264951	ENSP00000365131
ENSP00000264951	ENSP00000366135
ENSP00000264951	ENSP00000366249
ENSP00000264951	ENSP00000366607
ENSP00000264951	ENSP00000366746
ENSP00000264951	ENSP00000366953
ENSP00000264951	ENSP00000368699
ENSP00000264951	ENSP00000368887
ENSP00000264951	ENSP00000370589
ENSP00000264951	ENSP00000370741
ENSP00000264951	ENSP00000372883
ENSP00000264951	ENSP00000373039
ENSP00000264951	ENSP00000373715
ENSP00000264951	ENSP00000374213
ENSP00000264951	ENSP00000377148
ENSP00000264951	ENSP00000378306
ENSP00000264951	ENSP00000379888
ENSP00000264951	ENSP00000380251
ENSP00000264951	ENSP00000380982
ENSP00000264951	ENSP00000382392
ENSP00000264951	ENSP00000387170
ENSP00000264951	ENSP00000387303
ENSP00000264951	ENSP00000392617
ENSP00000264951	ENSP00000392859
ENSP00000264951	ENSP00000396152
ENSP00000264951	ENSP00000397259
ENSP00000264951	ENSP00000398597
ENSP00000264951	ENSP00000402240
ENSP00000264951	ENSP00000402338
ENSP00000264951	ENSP00000403345

ENSP00000264951	ENSP00000403852
ENSP00000264951	ENSP00000406280
ENSP00000264951	ENSP00000410416
ENSP00000264951	ENSP00000410758
ENSP00000264951	ENSP00000414006
ENSP00000264951	ENSP00000414634
ENSP00000264951	ENSP00000417229
ENSP00000264951	ENSP00000419628
ENSP00000264951	ENSP00000419740
ENSP00000264977	ENSP00000268459
ENSP00000264977	ENSP00000294008
ENSP00000264977	ENSP00000298229
ENSP00000264977	ENSP00000311344
ENSP00000264977	ENSP00000335321
ENSP00000264977	ENSP00000348573
ENSP00000264977	ENSP00000351885
ENSP00000264977	ENSP00000355296
ENSP00000264977	ENSP00000358886
ENSP00000264982	ENSP00000272139
ENSP00000264982	ENSP00000309606
ENSP00000264982	ENSP00000327213
ENSP00000264982	ENSP00000338157
ENSP00000264982	ENSP00000343129
ENSP00000264982	ENSP00000355944
ENSP00000264982	ENSP00000360464
ENSP00000264982	ENSP00000377944
ENSP00000264992	ENSP00000267101
ENSP00000264992	ENSP00000368755
ENSP00000264995	ENSP00000265100
ENSP00000264995	ENSP00000265245
ENSP00000264995	ENSP00000268206
ENSP00000264995	ENSP00000268379
ENSP00000264995	ENSP00000270625
ENSP00000264995	ENSP00000272418
ENSP00000264995	ENSP00000272542
ENSP00000264995	ENSP00000274242
ENSP00000264995	ENSP00000274606
ENSP00000264995	ENSP00000274849
ENSP00000264995	ENSP00000278572
ENSP00000264995	ENSP00000279242
ENSP00000264995	ENSP00000287038
ENSP00000264995	ENSP00000288937
ENSP00000264995	ENSP00000289371
ENSP00000264995	ENSP00000294189
ENSP00000264995	ENSP00000296102
ENSP00000264995	ENSP00000296411

ENSP00000264995	ENSP00000296577
ENSP00000264995	ENSP00000296802
ENSP00000264995	ENSP00000296805
ENSP00000264995	ENSP00000297908
ENSP00000264995	ENSP00000297990
ENSP00000264995	ENSP00000298283
ENSP00000264995	ENSP00000298746
ENSP00000264995	ENSP00000300151
ENSP00000264995	ENSP00000301585
ENSP00000264995	ENSP00000302886
ENSP00000264995	ENSP00000302896
ENSP00000264995	ENSP00000306548
ENSP00000264995	ENSP00000307525
ENSP00000264995	ENSP00000307889
ENSP00000264995	ENSP00000308275
ENSP00000264995	ENSP00000308897
ENSP00000264995	ENSP00000309334
ENSP00000264995	ENSP00000309433
ENSP00000264995	ENSP00000309830
ENSP00000264995	ENSP00000311028
ENSP00000264995	ENSP00000311430
ENSP00000264995	ENSP00000311648
ENSP00000264995	ENSP00000312311
ENSP00000264995	ENSP00000314949
ENSP00000264995	ENSP00000315017
ENSP00000264995	ENSP00000317376
ENSP00000264995	ENSP00000317992
ENSP00000264995	ENSP00000318374
ENSP00000264995	ENSP00000318646
ENSP00000264995	ENSP00000322396
ENSP00000264995	ENSP00000322419
ENSP00000264995	ENSP00000323858
ENSP00000264995	ENSP00000325312
ENSP00000264995	ENSP00000326981
ENSP00000264995	ENSP00000332887
ENSP00000264995	ENSP00000333401
ENSP00000264995	ENSP00000336702
ENSP00000264995	ENSP00000338573
ENSP00000264995	ENSP00000338862
ENSP00000264995	ENSP00000339095
ENSP00000264995	ENSP00000339145
ENSP00000264995	ENSP00000339795
ENSP00000264995	ENSP00000341082
ENSP00000264995	ENSP00000341885
ENSP00000264995	ENSP00000342181
ENSP00000264995	ENSP00000343392

ENSP00000264995	ENSP00000345096
ENSP00000264995	ENSP00000345156
ENSP00000264995	ENSP00000346015
ENSP00000264995	ENSP00000346022
ENSP00000264995	ENSP00000346027
ENSP00000264995	ENSP00000346037
ENSP00000264995	ENSP00000346050
ENSP00000264995	ENSP00000346088
ENSP00000264995	ENSP00000346725
ENSP00000264995	ENSP00000347087
ENSP00000264995	ENSP00000348596
ENSP00000264995	ENSP00000348886
ENSP00000264995	ENSP00000348965
ENSP00000264995	ENSP00000354040
ENSP00000264995	ENSP00000354125
ENSP00000264995	ENSP00000354525
ENSP00000264995	ENSP00000354739
ENSP00000264995	ENSP00000356290
ENSP00000264995	ENSP00000358563
ENSP00000264995	ENSP00000358854
ENSP00000264995	ENSP00000359345
ENSP00000264995	ENSP00000359688
ENSP00000264995	ENSP00000359939
ENSP00000264995	ENSP00000360031
ENSP00000264995	ENSP00000360286
ENSP00000264995	ENSP00000360412
ENSP00000264995	ENSP00000361076
ENSP00000264995	ENSP00000361465
ENSP00000264995	ENSP00000361982
ENSP00000264995	ENSP00000362153
ENSP00000264995	ENSP00000362744
ENSP00000264995	ENSP00000363018
ENSP00000264995	ENSP00000363559
ENSP00000264995	ENSP00000363676
ENSP00000264995	ENSP00000364320
ENSP00000264995	ENSP00000368984
ENSP00000264995	ENSP00000369757
ENSP00000264995	ENSP00000369965
ENSP00000264995	ENSP00000370930
ENSP00000264995	ENSP00000372093
ENSP00000264995	ENSP00000373404
ENSP00000264995	ENSP00000375730
ENSP00000264995	ENSP00000377486
ENSP00000264995	ENSP00000377617
ENSP00000264995	ENSP00000377640
ENSP00000264995	ENSP00000377865

ENSP00000264995	ENSP00000378160
ENSP00000264995	ENSP00000379888
ENSP00000264995	ENSP00000380156
ENSP00000264995	ENSP00000380982
ENSP00000264995	ENSP00000386717
ENSP00000264995	ENSP00000389103
ENSP00000264995	ENSP00000392859
ENSP00000264995	ENSP00000393393
ENSP00000264995	ENSP00000402338
ENSP00000264995	ENSP00000403817
ENSP00000264995	ENSP00000414321
ENSP00000264995	ENSP00000417229
ENSP00000264995	ENSP00000417602
ENSP00000265000	ENSP00000301011
ENSP00000265007	ENSP00000342023
ENSP00000265007	ENSP00000362068
ENSP00000265007	ENSP00000364683
ENSP00000265007	ENSP00000367406
ENSP00000265007	ENSP00000368976
ENSP00000265016	ENSP00000339191
ENSP00000265023	ENSP00000284981
ENSP00000265023	ENSP00000329380
ENSP00000265023	ENSP00000339328
ENSP00000265023	ENSP00000355627
ENSP00000265023	ENSP00000358106
ENSP00000265023	ENSP00000379383
ENSP00000265026	ENSP00000362463
ENSP00000265038	ENSP00000290349
ENSP00000265038	ENSP00000290846
ENSP00000265038	ENSP00000301764
ENSP00000265038	ENSP00000304102
ENSP00000265038	ENSP00000314949
ENSP00000265038	ENSP00000348089
ENSP00000265038	ENSP00000350512
ENSP00000265038	ENSP00000351137
ENSP00000265038	ENSP00000367934
ENSP00000265038	ENSP00000370125
ENSP00000265038	ENSP00000384109
ENSP00000265044	ENSP00000317331
ENSP00000265044	ENSP00000341213
ENSP00000265044	ENSP00000343412
ENSP00000265044	ENSP00000361707
ENSP00000265044	ENSP00000364188
ENSP00000265044	ENSP00000365175
ENSP00000265044	ENSP00000385083
ENSP00000265044	ENSP00000387477

ENSP00000265044	ENSP00000415363
ENSP00000265052	ENSP00000300441
ENSP00000265052	ENSP00000301180
ENSP00000265052	ENSP00000320646
ENSP00000265052	ENSP00000348762
ENSP00000265052	ENSP00000357650
ENSP00000265052	ENSP00000361667
ENSP00000265056	ENSP00000265465
ENSP00000265056	ENSP00000265728
ENSP00000265056	ENSP00000265758
ENSP00000265056	ENSP00000265963
ENSP00000265056	ENSP00000269159
ENSP00000265056	ENSP00000269701
ENSP00000265056	ENSP00000273398
ENSP00000265056	ENSP00000274311
ENSP00000265056	ENSP00000275053
ENSP00000265056	ENSP00000276533
ENSP00000265056	ENSP00000276603
ENSP00000265056	ENSP00000278412
ENSP00000265056	ENSP00000278616
ENSP00000265056	ENSP00000285896
ENSP00000265056	ENSP00000286428
ENSP00000265056	ENSP00000290330
ENSP00000265056	ENSP00000295926
ENSP00000265056	ENSP00000296509
ENSP00000265056	ENSP00000297338
ENSP00000265056	ENSP00000297431
ENSP00000265056	ENSP00000297873
ENSP00000265056	ENSP00000300086
ENSP00000265056	ENSP00000300738
ENSP00000265056	ENSP00000301019
ENSP00000265056	ENSP00000302111
ENSP00000265056	ENSP00000307525
ENSP00000265056	ENSP00000307900
ENSP00000265056	ENSP00000310260
ENSP00000265056	ENSP00000313420
ENSP00000265056	ENSP00000315379
ENSP00000265056	ENSP00000315702
ENSP00000265056	ENSP00000322180
ENSP00000265056	ENSP00000322570
ENSP00000265056	ENSP00000323099
ENSP00000265056	ENSP00000324444
ENSP00000265056	ENSP00000339109
ENSP00000265056	ENSP00000339353
ENSP00000265056	ENSP00000340698
ENSP00000265056	ENSP00000340879

ENSP00000265056	ENSP00000343741
ENSP00000265056	ENSP00000346566
ENSP00000265056	ENSP00000347087
ENSP00000265056	ENSP00000347978
ENSP00000265056	ENSP00000348565
ENSP00000265056	ENSP00000349213
ENSP00000265056	ENSP00000350491
ENSP00000265056	ENSP00000351284
ENSP00000265056	ENSP00000351697
ENSP00000265056	ENSP00000352522
ENSP00000265056	ENSP00000353098
ENSP00000265056	ENSP00000353793
ENSP00000265056	ENSP00000355011
ENSP00000265056	ENSP00000355153
ENSP00000265056	ENSP00000359211
ENSP00000265056	ENSP00000359531
ENSP00000265056	ENSP00000359691
ENSP00000265056	ENSP00000360268
ENSP00000265056	ENSP00000360412
ENSP00000265056	ENSP00000360532
ENSP00000265056	ENSP00000360621
ENSP00000265056	ENSP00000361076
ENSP00000265056	ENSP00000362352
ENSP00000265056	ENSP00000362744
ENSP00000265056	ENSP00000363021
ENSP00000265056	ENSP00000364037
ENSP00000265056	ENSP00000366395
ENSP00000265056	ENSP00000366974
ENSP00000265056	ENSP00000368349
ENSP00000265056	ENSP00000369335
ENSP00000265056	ENSP00000369716
ENSP00000265056	ENSP00000370151
ENSP00000265056	ENSP00000371169
ENSP00000265056	ENSP00000371475
ENSP00000265056	ENSP00000372023
ENSP00000265056	ENSP00000377486
ENSP00000265056	ENSP00000377747
ENSP00000265056	ENSP00000386239
ENSP00000265056	ENSP00000401018
ENSP00000265069	ENSP00000269385
ENSP00000265069	ENSP00000306123
ENSP00000265069	ENSP00000308546
ENSP00000265069	ENSP00000329466
ENSP00000265069	ENSP00000333376
ENSP00000265069	ENSP00000350009
ENSP00000265069	ENSP00000355011

ENSP00000265069	ENSP00000366607
ENSP00000265069	ENSP00000379607
ENSP00000265070	ENSP00000273398
ENSP00000265070	ENSP00000281187
ENSP00000265070	ENSP00000325002
ENSP00000265070	ENSP00000327716
ENSP00000265070	ENSP00000328079
ENSP00000265070	ENSP00000329419
ENSP00000265070	ENSP00000357048
ENSP00000265073	ENSP00000265354
ENSP00000265073	ENSP00000276072
ENSP00000265073	ENSP00000278412
ENSP00000265073	ENSP00000283875
ENSP00000265073	ENSP00000286428
ENSP00000265073	ENSP00000296702
ENSP00000265073	ENSP00000299543
ENSP00000265073	ENSP00000299601
ENSP00000265073	ENSP00000301740
ENSP00000265073	ENSP00000304593
ENSP00000265073	ENSP00000310596
ENSP00000265073	ENSP00000311005
ENSP00000265073	ENSP00000311816
ENSP00000265073	ENSP00000312411
ENSP00000265073	ENSP00000312735
ENSP00000265073	ENSP00000314949
ENSP00000265073	ENSP00000320566
ENSP00000265073	ENSP00000322016
ENSP00000265073	ENSP00000328720
ENSP00000265073	ENSP00000329029
ENSP00000265073	ENSP00000329357
ENSP00000265073	ENSP00000329933
ENSP00000265073	ENSP00000331310
ENSP00000265073	ENSP00000332444
ENSP00000265073	ENSP00000336842
ENSP00000265073	ENSP00000336868
ENSP00000265073	ENSP00000340271
ENSP00000265073	ENSP00000348107
ENSP00000265073	ENSP00000348510
ENSP00000265073	ENSP00000351575
ENSP00000265073	ENSP00000352738
ENSP00000265073	ENSP00000353622
ENSP00000265073	ENSP00000354522
ENSP00000265073	ENSP00000357907
ENSP00000265073	ENSP00000359531
ENSP00000265073	ENSP00000360147
ENSP00000265073	ENSP00000360464

ENSP00000265073	ENSP00000361180
ENSP00000265073	ENSP00000361465
ENSP00000265073	ENSP00000363779
ENSP00000265073	ENSP00000364929
ENSP00000265073	ENSP00000366974
ENSP00000265073	ENSP00000368438
ENSP00000265073	ENSP00000368801
ENSP00000265073	ENSP00000368927
ENSP00000265073	ENSP00000369218
ENSP00000265073	ENSP00000370105
ENSP00000265073	ENSP00000370377
ENSP00000265073	ENSP00000380460
ENSP00000265073	ENSP00000380504
ENSP00000265073	ENSP00000383059
ENSP00000265073	ENSP00000384597
ENSP00000265073	ENSP00000406293
ENSP00000265073	ENSP00000420588
ENSP00000265077	ENSP00000363548
ENSP00000265077	ENSP00000373614
ENSP00000265077	ENSP00000384169
ENSP00000265081	ENSP00000265304
ENSP00000265081	ENSP00000265849
ENSP00000265081	ENSP00000267430
ENSP00000265081	ENSP00000268043
ENSP00000265081	ENSP00000268124
ENSP00000265081	ENSP00000285021
ENSP00000265081	ENSP00000285398
ENSP00000265081	ENSP00000294008
ENSP00000265081	ENSP00000300738
ENSP00000265081	ENSP00000301764
ENSP00000265081	ENSP00000306561
ENSP00000265081	ENSP00000307853
ENSP00000265081	ENSP00000308944
ENSP00000265081	ENSP00000310520
ENSP00000265081	ENSP00000311873
ENSP00000265081	ENSP00000318977
ENSP00000265081	ENSP00000321239
ENSP00000265081	ENSP00000321636
ENSP00000265081	ENSP00000322570
ENSP00000265081	ENSP00000337127
ENSP00000265081	ENSP00000341243
ENSP00000265081	ENSP00000343741
ENSP00000265081	ENSP00000343745
ENSP00000265081	ENSP00000347978
ENSP00000265081	ENSP00000348020
ENSP00000265081	ENSP00000350283

ENSP00000265081	ENSP00000351284
ENSP00000265081	ENSP00000351697
ENSP00000265081	ENSP00000351947
ENSP00000265081	ENSP00000358478
ENSP00000265081	ENSP00000358576
ENSP00000265081	ENSP00000360268
ENSP00000265081	ENSP00000360310
ENSP00000265081	ENSP00000361310
ENSP00000265081	ENSP00000363021
ENSP00000265081	ENSP00000364270
ENSP00000265081	ENSP00000368349
ENSP00000265081	ENSP00000368438
ENSP00000265081	ENSP00000369335
ENSP00000265081	ENSP00000370222
ENSP00000265081	ENSP00000375809
ENSP00000265081	ENSP00000380504
ENSP00000265085	ENSP00000268957
ENSP00000265085	ENSP00000297837
ENSP00000265085	ENSP00000312042
ENSP00000265085	ENSP00000362993
ENSP00000265085	ENSP00000391723
ENSP00000265087	ENSP00000287295
ENSP00000265087	ENSP00000353575
ENSP00000265100	ENSP00000268206
ENSP00000265100	ENSP00000268616
ENSP00000265100	ENSP00000268661
ENSP00000265100	ENSP00000269499
ENSP00000265100	ENSP00000270625
ENSP00000265100	ENSP00000274242
ENSP00000265100	ENSP00000274606
ENSP00000265100	ENSP00000281141
ENSP00000265100	ENSP00000287038
ENSP00000265100	ENSP00000294189
ENSP00000265100	ENSP00000296805
ENSP00000265100	ENSP00000297908
ENSP00000265100	ENSP00000298283
ENSP00000265100	ENSP00000306548
ENSP00000265100	ENSP00000307889
ENSP00000265100	ENSP00000309334
ENSP00000265100	ENSP00000309830
ENSP00000265100	ENSP00000311028
ENSP00000265100	ENSP00000311430
ENSP00000265100	ENSP00000317376
ENSP00000265100	ENSP00000318646
ENSP00000265100	ENSP00000322419
ENSP00000265100	ENSP00000333837

ENSP00000265100	ENSP00000337839
ENSP00000265100	ENSP00000339095
ENSP00000265100	ENSP00000341082
ENSP00000265100	ENSP00000341538
ENSP00000265100	ENSP00000343392
ENSP00000265100	ENSP00000345156
ENSP00000265100	ENSP00000346015
ENSP00000265100	ENSP00000346022
ENSP00000265100	ENSP00000346027
ENSP00000265100	ENSP00000346037
ENSP00000265100	ENSP00000346050
ENSP00000265100	ENSP00000346088
ENSP00000265100	ENSP00000354739
ENSP00000265100	ENSP00000359345
ENSP00000265100	ENSP00000359531
ENSP00000265100	ENSP00000361076
ENSP00000265100	ENSP00000362688
ENSP00000265100	ENSP00000362744
ENSP00000265100	ENSP00000363018
ENSP00000265100	ENSP00000363676
ENSP00000265100	ENSP00000364320
ENSP00000265100	ENSP00000369757
ENSP00000265100	ENSP00000375730
ENSP00000265100	ENSP00000377486
ENSP00000265100	ENSP00000377640
ENSP00000265100	ENSP00000377865
ENSP00000265100	ENSP00000378160
ENSP00000265100	ENSP00000379607
ENSP00000265100	ENSP00000380156
ENSP00000265100	ENSP00000386717
ENSP00000265100	ENSP00000389103
ENSP00000265100	ENSP00000391723
ENSP00000265100	ENSP00000393241
ENSP00000265100	ENSP00000393393
ENSP00000265100	ENSP00000402338
ENSP00000265100	ENSP00000403175
ENSP00000265100	ENSP00000412583
ENSP00000265100	ENSP00000414321
ENSP00000265100	ENSP00000416110
ENSP00000265104	ENSP00000265148
ENSP00000265104	ENSP00000276014
ENSP00000265104	ENSP00000295951
ENSP00000265104	ENSP00000296509
ENSP00000265104	ENSP00000300231
ENSP00000265104	ENSP00000300249
ENSP00000265104	ENSP00000301332

ENSP00000265104	ENSP00000333950
ENSP00000265104	ENSP00000347626
ENSP00000265104	ENSP00000351665
ENSP00000265104	ENSP00000354158
ENSP00000265104	ENSP00000355922
ENSP00000265104	ENSP00000357643
ENSP00000265104	ENSP00000360310
ENSP00000265104	ENSP00000366974
ENSP00000265104	ENSP00000368976
ENSP00000265104	ENSP00000374566
ENSP00000265104	ENSP00000377793
ENSP00000265104	ENSP00000382177
ENSP00000265107	ENSP00000278174
ENSP00000265107	ENSP00000304370
ENSP00000265107	ENSP00000307491
ENSP00000265107	ENSP00000334148
ENSP00000265132	ENSP00000295897
ENSP00000265132	ENSP00000321260
ENSP00000265132	ENSP00000344460
ENSP00000265132	ENSP00000346839
ENSP00000265132	ENSP00000381891
ENSP00000265140	ENSP00000265728
ENSP00000265140	ENSP00000276533
ENSP00000265140	ENSP00000287598
ENSP00000265140	ENSP00000292475
ENSP00000265140	ENSP00000296509
ENSP00000265140	ENSP00000300738
ENSP00000265140	ENSP00000302530
ENSP00000265140	ENSP00000305899
ENSP00000265140	ENSP00000307525
ENSP00000265140	ENSP00000308944
ENSP00000265140	ENSP00000313171
ENSP00000265140	ENSP00000323663
ENSP00000265140	ENSP00000340879
ENSP00000265140	ENSP00000349576
ENSP00000265140	ENSP00000370151
ENSP00000265140	ENSP00000371475
ENSP00000265140	ENSP00000372023
ENSP00000265140	ENSP00000377384
ENSP00000265140	ENSP00000381657
ENSP00000265140	ENSP00000401018
ENSP00000265148	ENSP00000267163
ENSP00000265148	ENSP00000271452
ENSP00000265148	ENSP00000271715
ENSP00000265148	ENSP00000275780
ENSP00000265148	ENSP00000285116

ENSP00000265148	ENSP00000286428
ENSP00000265148	ENSP00000287598
ENSP00000265148	ENSP00000295951
ENSP00000265148	ENSP00000296509
ENSP00000265148	ENSP00000297338
ENSP00000265148	ENSP00000300249
ENSP00000265148	ENSP00000301019
ENSP00000265148	ENSP00000301332
ENSP00000265148	ENSP00000308750
ENSP00000265148	ENSP00000317714
ENSP00000265148	ENSP00000322180
ENSP00000265148	ENSP00000322915
ENSP00000265148	ENSP00000327009
ENSP00000265148	ENSP00000332592
ENSP00000265148	ENSP00000333950
ENSP00000265148	ENSP00000336587
ENSP00000265148	ENSP00000345892
ENSP00000265148	ENSP00000346566
ENSP00000265148	ENSP00000347719
ENSP00000265148	ENSP00000348965
ENSP00000265148	ENSP00000351665
ENSP00000265148	ENSP00000353793
ENSP00000265148	ENSP00000355922
ENSP00000265148	ENSP00000356579
ENSP00000265148	ENSP00000360473
ENSP00000265148	ENSP00000363734
ENSP00000265148	ENSP00000368976
ENSP00000265148	ENSP00000372023
ENSP00000265148	ENSP00000372694
ENSP00000265148	ENSP00000373952
ENSP00000265148	ENSP00000377793
ENSP00000265148	ENSP00000378529
ENSP00000265148	ENSP00000379607
ENSP00000265148	ENSP00000382562
ENSP00000265148	ENSP00000402084
ENSP00000265148	ENSP00000412319
ENSP00000265148	ENSP00000414402
ENSP00000265148	ENSP00000415678
ENSP00000265171	ENSP00000267101
ENSP00000265171	ENSP00000290974
ENSP00000265171	ENSP00000312262
ENSP00000265171	ENSP00000322775
ENSP00000265171	ENSP00000325421
ENSP00000265171	ENSP00000334061
ENSP00000265171	ENSP00000357283
ENSP00000265171	ENSP00000413625

ENSP00000265174	ENSP00000354916
ENSP00000265175	ENSP00000265565
ENSP00000265175	ENSP00000280190
ENSP00000265175	ENSP00000285243
ENSP00000265175	ENSP00000294179
ENSP00000265175	ENSP00000296137
ENSP00000265175	ENSP00000299596
ENSP00000265175	ENSP00000304051
ENSP00000265175	ENSP00000308339
ENSP00000265175	ENSP00000312778
ENSP00000265175	ENSP00000316032
ENSP00000265175	ENSP00000319104
ENSP00000265175	ENSP00000325612
ENSP00000265175	ENSP00000327716
ENSP00000265175	ENSP00000330945
ENSP00000265175	ENSP00000331817
ENSP00000265175	ENSP00000344460
ENSP00000265175	ENSP00000347329
ENSP00000265175	ENSP00000350708
ENSP00000265175	ENSP00000352708
ENSP00000265175	ENSP00000353904
ENSP00000265175	ENSP00000362948
ENSP00000265175	ENSP00000371888
ENSP00000265175	ENSP00000377280
ENSP00000265175	ENSP00000387170
ENSP00000265175	ENSP00000391774
ENSP00000265175	ENSP00000401445
ENSP00000265192	ENSP00000363676
ENSP00000265195	ENSP00000265295
ENSP00000265195	ENSP00000307786
ENSP00000265195	ENSP00000322706
ENSP00000265195	ENSP00000337688
ENSP00000265195	ENSP00000346874
ENSP00000265195	ENSP00000349131
ENSP00000265195	ENSP00000356530
ENSP00000265195	ENSP00000357998
ENSP00000265195	ENSP00000365943
ENSP00000265195	ENSP00000386621
ENSP00000265195	ENSP00000401445
ENSP00000265245	ENSP00000267425
ENSP00000265245	ENSP00000268661
ENSP00000265245	ENSP00000278572
ENSP00000265245	ENSP00000287038
ENSP00000265245	ENSP00000296802
ENSP00000265245	ENSP00000298283
ENSP00000265245	ENSP00000298746

ENSP00000265245	ENSP00000301364
ENSP00000265245	ENSP00000302886
ENSP00000265245	ENSP00000305702
ENSP00000265245	ENSP00000307525
ENSP00000265245	ENSP00000309830
ENSP00000265245	ENSP00000310042
ENSP00000265245	ENSP00000314348
ENSP00000265245	ENSP00000317992
ENSP00000265245	ENSP00000322396
ENSP00000265245	ENSP00000338862
ENSP00000265245	ENSP00000339145
ENSP00000265245	ENSP00000339795
ENSP00000265245	ENSP00000341885
ENSP00000265245	ENSP00000346453
ENSP00000265245	ENSP00000346725
ENSP00000265245	ENSP00000348596
ENSP00000265245	ENSP00000354040
ENSP00000265245	ENSP00000354739
ENSP00000265245	ENSP00000356290
ENSP00000265245	ENSP00000356920
ENSP00000265245	ENSP00000358400
ENSP00000265245	ENSP00000358563
ENSP00000265245	ENSP00000359345
ENSP00000265245	ENSP00000359688
ENSP00000265245	ENSP00000360412
ENSP00000265245	ENSP00000361076
ENSP00000265245	ENSP00000361982
ENSP00000265245	ENSP00000363018
ENSP00000265245	ENSP00000363384
ENSP00000265245	ENSP00000363559
ENSP00000265245	ENSP00000363676
ENSP00000265245	ENSP00000364037
ENSP00000265245	ENSP00000364320
ENSP00000265245	ENSP00000366519
ENSP00000265245	ENSP00000377640
ENSP00000265245	ENSP00000379888
ENSP00000265245	ENSP00000380156
ENSP00000265245	ENSP00000380982
ENSP00000265245	ENSP00000382392
ENSP00000265245	ENSP00000388464
ENSP00000265245	ENSP00000389103
ENSP00000265245	ENSP00000402338
ENSP00000265245	ENSP00000419740
ENSP00000265260	ENSP00000276893
ENSP00000265260	ENSP00000300086
ENSP00000265271	ENSP00000315859

ENSP00000265276	ENSP00000317177
ENSP00000265276	ENSP00000342793
ENSP00000265276	ENSP00000349078
ENSP00000265295	ENSP00000274695
ENSP00000265295	ENSP00000311344
ENSP00000265295	ENSP00000357998
ENSP00000265295	ENSP00000363205
ENSP00000265295	ENSP00000370607
ENSP00000265295	ENSP00000370719
ENSP00000265295	ENSP00000374328
ENSP00000265304	ENSP00000265562
ENSP00000265304	ENSP00000265806
ENSP00000265304	ENSP00000265881
ENSP00000265304	ENSP00000267430
ENSP00000265304	ENSP00000267845
ENSP00000265304	ENSP00000268124
ENSP00000265304	ENSP00000294339
ENSP00000265304	ENSP00000296509
ENSP00000265304	ENSP00000298139
ENSP00000265304	ENSP00000298999
ENSP00000265304	ENSP00000301329
ENSP00000265304	ENSP00000301364
ENSP00000265304	ENSP00000301585
ENSP00000265304	ENSP00000303434
ENSP00000265304	ENSP00000306561
ENSP00000265304	ENSP00000307387
ENSP00000265304	ENSP00000311083
ENSP00000265304	ENSP00000313350
ENSP00000265304	ENSP00000317636
ENSP00000265304	ENSP00000321636
ENSP00000265304	ENSP00000329466
ENSP00000265304	ENSP00000331310
ENSP00000265304	ENSP00000332721
ENSP00000265304	ENSP00000341243
ENSP00000265304	ENSP00000343526
ENSP00000265304	ENSP00000343745
ENSP00000265304	ENSP00000347232
ENSP00000265304	ENSP00000349078
ENSP00000265304	ENSP00000350928
ENSP00000265304	ENSP00000351284
ENSP00000265304	ENSP00000358854
ENSP00000265304	ENSP00000361465
ENSP00000265304	ENSP00000362298
ENSP00000265304	ENSP00000362306
ENSP00000265304	ENSP00000362463
ENSP00000265304	ENSP00000363021

ENSP00000265304	ENSP00000364126
ENSP00000265304	ENSP00000379607
ENSP00000265304	ENSP00000392466
ENSP00000265304	ENSP00000394649
ENSP00000265304	ENSP00000396704
ENSP00000265304	ENSP00000406868
ENSP00000265304	ENSP00000407047
ENSP00000265316	ENSP00000269216
ENSP00000265316	ENSP00000314733
ENSP00000265316	ENSP00000346206
ENSP00000265316	ENSP00000359526
ENSP00000265316	ENSP00000372722
ENSP00000265316	ENSP00000381293
ENSP00000265316	ENSP00000401149
ENSP00000265316	ENSP00000402316
ENSP00000265316	ENSP00000405356
ENSP00000265316	ENSP00000413080
ENSP00000265316	ENSP00000415660
ENSP00000265334	ENSP00000358478
ENSP00000265343	ENSP00000267101
ENSP00000265343	ENSP00000288602
ENSP00000265343	ENSP00000305689
ENSP00000265343	ENSP00000311005
ENSP00000265343	ENSP00000337340
ENSP00000265343	ENSP00000344456
ENSP00000265343	ENSP00000352121
ENSP00000265343	ENSP00000369695
ENSP00000265343	ENSP00000380054
ENSP00000265350	ENSP00000267064
ENSP00000265350	ENSP00000268711
ENSP00000265350	ENSP00000282007
ENSP00000265350	ENSP00000286317
ENSP00000265350	ENSP00000290663
ENSP00000265350	ENSP00000292035
ENSP00000265350	ENSP00000293777
ENSP00000265350	ENSP00000297347
ENSP00000265350	ENSP00000300651
ENSP00000265350	ENSP00000323720
ENSP00000265350	ENSP00000325612
ENSP00000265350	ENSP00000326128
ENSP00000265350	ENSP00000326767
ENSP00000265350	ENSP00000337340
ENSP00000265350	ENSP00000342343
ENSP00000265350	ENSP00000357907
ENSP00000265350	ENSP00000359206
ENSP00000265350	ENSP00000362948

ENSP00000265350	ENSP00000363193
ENSP00000265350	ENSP00000369162
ENSP00000265350	ENSP00000381216
ENSP00000265350	ENSP00000384048
ENSP00000265350	ENSP00000391774
ENSP00000265350	ENSP00000392617
ENSP00000265350	ENSP00000418734
ENSP00000265351	ENSP00000267425
ENSP00000265351	ENSP00000270176
ENSP00000265351	ENSP00000275730
ENSP00000265351	ENSP00000283109
ENSP00000265351	ENSP00000283195
ENSP00000265351	ENSP00000285873
ENSP00000265351	ENSP00000290231
ENSP00000265351	ENSP00000291688
ENSP00000265351	ENSP00000295989
ENSP00000265351	ENSP00000296786
ENSP00000265351	ENSP00000301364
ENSP00000265351	ENSP00000301838
ENSP00000265351	ENSP00000302886
ENSP00000265351	ENSP00000310649
ENSP00000265351	ENSP00000310668
ENSP00000265351	ENSP00000311135
ENSP00000265351	ENSP00000312735
ENSP00000265351	ENSP00000314004
ENSP00000265351	ENSP00000315791
ENSP00000265351	ENSP00000316032
ENSP00000265351	ENSP00000318195
ENSP00000265351	ENSP00000318604
ENSP00000265351	ENSP00000329357
ENSP00000265351	ENSP00000329466
ENSP00000265351	ENSP00000336842
ENSP00000265351	ENSP00000337405
ENSP00000265351	ENSP00000341551
ENSP00000265351	ENSP00000346453
ENSP00000265351	ENSP00000348577
ENSP00000265351	ENSP00000350869
ENSP00000265351	ENSP00000352400
ENSP00000265351	ENSP00000354720
ENSP00000265351	ENSP00000354742
ENSP00000265351	ENSP00000354916
ENSP00000265351	ENSP00000355884
ENSP00000265351	ENSP00000355890
ENSP00000265351	ENSP00000356405
ENSP00000265351	ENSP00000356548
ENSP00000265351	ENSP00000356744

ENSP00000265351	ENSP00000357907
ENSP00000265351	ENSP00000359000
ENSP00000265351	ENSP00000360532
ENSP00000265351	ENSP00000361186
ENSP00000265351	ENSP00000361287
ENSP00000265351	ENSP00000364293
ENSP00000265351	ENSP00000371155
ENSP00000265351	ENSP00000373715
ENSP00000265351	ENSP00000378529
ENSP00000265351	ENSP00000381567
ENSP00000265351	ENSP00000384302
ENSP00000265351	ENSP00000386918
ENSP00000265351	ENSP00000386992
ENSP00000265351	ENSP00000404121
ENSP00000265354	ENSP00000265990
ENSP00000265354	ENSP00000267064
ENSP00000265354	ENSP00000267101
ENSP00000265354	ENSP00000268058
ENSP00000265354	ENSP00000270861
ENSP00000265354	ENSP00000277541
ENSP00000265354	ENSP00000278412
ENSP00000265354	ENSP00000281437
ENSP00000265354	ENSP00000294168
ENSP00000265354	ENSP00000295025
ENSP00000265354	ENSP00000296839
ENSP00000265354	ENSP00000302501
ENSP00000265354	ENSP00000305422
ENSP00000265354	ENSP00000305502
ENSP00000265354	ENSP00000307208
ENSP00000265354	ENSP00000311005
ENSP00000265354	ENSP00000313420
ENSP00000265354	ENSP00000320461
ENSP00000265354	ENSP00000323780
ENSP00000265354	ENSP00000326296
ENSP00000265354	ENSP00000326366
ENSP00000265354	ENSP00000327959
ENSP00000265354	ENSP00000328720
ENSP00000265354	ENSP00000329357
ENSP00000265354	ENSP00000331608
ENSP00000265354	ENSP00000339004
ENSP00000265354	ENSP00000339086
ENSP00000265354	ENSP00000339527
ENSP00000265354	ENSP00000340944
ENSP00000265354	ENSP00000341324
ENSP00000265354	ENSP00000342307
ENSP00000265354	ENSP00000342828

ENSP00000265354	ENSP00000344537
ENSP00000265354	ENSP00000344666
ENSP00000265354	ENSP00000345702
ENSP00000265354	ENSP00000347168
ENSP00000265354	ENSP00000347733
ENSP00000265354	ENSP00000348551
ENSP00000265354	ENSP00000349213
ENSP00000265354	ENSP00000350681
ENSP00000265354	ENSP00000350767
ENSP00000265354	ENSP00000351894
ENSP00000265354	ENSP00000354927
ENSP00000265354	ENSP00000361064
ENSP00000265354	ENSP00000361998
ENSP00000265354	ENSP00000362717
ENSP00000265354	ENSP00000362824
ENSP00000265354	ENSP00000363046
ENSP00000265354	ENSP00000364946
ENSP00000265354	ENSP00000366534
ENSP00000265354	ENSP00000366974
ENSP00000265354	ENSP00000367034
ENSP00000265354	ENSP00000367972
ENSP00000265354	ENSP00000370256
ENSP00000265354	ENSP00000377969
ENSP00000265354	ENSP00000378132
ENSP00000265354	ENSP00000379678
ENSP00000265354	ENSP00000382423
ENSP00000265354	ENSP00000384675
ENSP00000265354	ENSP00000386200
ENSP00000265372	ENSP00000281437
ENSP00000265372	ENSP00000306245
ENSP00000265372	ENSP00000310036
ENSP00000265372	ENSP00000329102
ENSP00000265372	ENSP00000329357
ENSP00000265372	ENSP00000343023
ENSP00000265372	ENSP00000345702
ENSP00000265372	ENSP00000371897
ENSP00000265372	ENSP00000377384
ENSP00000265372	ENSP00000394394
ENSP00000265381	ENSP00000277541
ENSP00000265381	ENSP00000284981
ENSP00000265381	ENSP00000293662
ENSP00000265381	ENSP00000326366
ENSP00000265381	ENSP00000345731
ENSP00000265381	ENSP00000362399
ENSP00000265381	ENSP00000363163
ENSP00000265381	ENSP00000367408

ENSP00000265381	ENSP00000370410
ENSP00000265381	ENSP00000380054
ENSP00000265388	ENSP00000283290
ENSP00000265388	ENSP00000290231
ENSP00000265388	ENSP00000312370
ENSP00000265388	ENSP00000316032
ENSP00000265388	ENSP00000336712
ENSP00000265388	ENSP00000341213
ENSP00000265388	ENSP00000352400
ENSP00000265388	ENSP00000353622
ENSP00000265388	ENSP00000362820
ENSP00000265388	ENSP00000362900
ENSP00000265388	ENSP00000369335
ENSP00000265388	ENSP00000377262
ENSP00000265388	ENSP00000381863
ENSP00000265388	ENSP00000416959
ENSP00000265433	ENSP00000269385
ENSP00000265433	ENSP00000274255
ENSP00000265433	ENSP00000276603
ENSP00000265433	ENSP00000278616
ENSP00000265433	ENSP00000286800
ENSP00000265433	ENSP00000287647
ENSP00000265433	ENSP00000298139
ENSP00000265433	ENSP00000310966
ENSP00000265433	ENSP00000313420
ENSP00000265433	ENSP00000317636
ENSP00000265433	ENSP00000323099
ENSP00000265433	ENSP00000325863
ENSP00000265433	ENSP00000342011
ENSP00000265433	ENSP00000343741
ENSP00000265433	ENSP00000347232
ENSP00000265433	ENSP00000350283
ENSP00000265433	ENSP00000350359
ENSP00000265433	ENSP00000355759
ENSP00000265433	ENSP00000360532
ENSP00000265433	ENSP00000362578
ENSP00000265433	ENSP00000365588
ENSP00000265433	ENSP00000367527
ENSP00000265433	ENSP00000367787
ENSP00000265433	ENSP00000368438
ENSP00000265433	ENSP00000371475
ENSP00000265433	ENSP00000372023
ENSP00000265433	ENSP00000373060
ENSP00000265433	ENSP00000384048
ENSP00000265433	ENSP00000409167
ENSP00000265433	ENSP00000417281

ENSP00000265437	ENSP00000357674
ENSP00000265459	ENSP00000363163
ENSP00000265459	ENSP00000367408
ENSP00000265459	ENSP00000383623
ENSP00000265462	ENSP00000268483
ENSP00000265462	ENSP00000272227
ENSP00000265462	ENSP00000282541
ENSP00000265462	ENSP00000283033
ENSP00000265462	ENSP00000286091
ENSP00000265462	ENSP00000299608
ENSP00000265462	ENSP00000301585
ENSP00000265462	ENSP00000304908
ENSP00000265462	ENSP00000305465
ENSP00000265462	ENSP00000311083
ENSP00000265462	ENSP00000312735
ENSP00000265462	ENSP00000323313
ENSP00000265462	ENSP00000330836
ENSP00000265462	ENSP00000334044
ENSP00000265462	ENSP00000337443
ENSP00000265462	ENSP00000339484
ENSP00000265462	ENSP00000341947
ENSP00000265462	ENSP00000342026
ENSP00000265462	ENSP00000352408
ENSP00000265462	ENSP00000356574
ENSP00000265462	ENSP00000358307
ENSP00000265462	ENSP00000364320
ENSP00000265462	ENSP00000366249
ENSP00000265462	ENSP00000368646
ENSP00000265462	ENSP00000368699
ENSP00000265462	ENSP00000371388
ENSP00000265462	ENSP00000373039
ENSP00000265462	ENSP00000373477
ENSP00000265462	ENSP00000379607
ENSP00000265462	ENSP00000396152
ENSP00000265462	ENSP00000410416
ENSP00000265462	ENSP00000412922
ENSP00000265465	ENSP00000268043
ENSP00000265465	ENSP00000270225
ENSP00000265465	ENSP00000276533
ENSP00000265465	ENSP00000278412
ENSP00000265465	ENSP00000285896
ENSP00000265465	ENSP00000296273
ENSP00000265465	ENSP00000298139
ENSP00000265465	ENSP00000300245
ENSP00000265465	ENSP00000310557
ENSP00000265465	ENSP00000312870

ENSP00000265465	ENSP00000313171
ENSP00000265465	ENSP00000318977
ENSP00000265465	ENSP00000328023
ENSP00000265465	ENSP00000330343
ENSP00000265465	ENSP00000331907
ENSP00000265465	ENSP00000334061
ENSP00000265465	ENSP00000347232
ENSP00000265465	ENSP00000350491
ENSP00000265465	ENSP00000353793
ENSP00000265465	ENSP00000355759
ENSP00000265465	ENSP00000356290
ENSP00000265465	ENSP00000360286
ENSP00000265465	ENSP00000360621
ENSP00000265465	ENSP00000368349
ENSP00000265465	ENSP00000369411
ENSP00000265465	ENSP00000369757
ENSP00000265465	ENSP00000370151
ENSP00000265465	ENSP00000373715
ENSP00000265465	ENSP00000377617
ENSP00000265465	ENSP00000380504
ENSP00000265517	ENSP00000299767
ENSP00000265537	ENSP00000277865
ENSP00000265537	ENSP00000296411
ENSP00000265537	ENSP00000300648
ENSP00000265537	ENSP00000302886
ENSP00000265537	ENSP00000307684
ENSP00000265537	ENSP00000313603
ENSP00000265537	ENSP00000325312
ENSP00000265537	ENSP00000354522
ENSP00000265537	ENSP00000354554
ENSP00000265537	ENSP00000355471
ENSP00000265537	ENSP00000355890
ENSP00000265537	ENSP00000356134
ENSP00000265537	ENSP00000356811
ENSP00000265537	ENSP00000360327
ENSP00000265537	ENSP00000360515
ENSP00000265537	ENSP00000364986
ENSP00000265537	ENSP00000370741
ENSP00000265537	ENSP00000373082
ENSP00000265537	ENSP00000380460
ENSP00000265537	ENSP00000389065
ENSP00000265537	ENSP00000411327
ENSP00000265537	ENSP00000412553
ENSP00000265537	ENSP00000413319
ENSP00000265537	ENSP00000414373
ENSP00000265562	ENSP00000280362

ENSP00000265562	ENSP00000286835
ENSP00000265562	ENSP00000299601
ENSP00000265562	ENSP00000300413
ENSP00000265562	ENSP00000302160
ENSP00000265562	ENSP00000302239
ENSP00000265562	ENSP00000307387
ENSP00000265562	ENSP00000310596
ENSP00000265562	ENSP00000311200
ENSP00000265562	ENSP00000319341
ENSP00000265562	ENSP00000331201
ENSP00000265562	ENSP00000339186
ENSP00000265562	ENSP00000341189
ENSP00000265562	ENSP00000345530
ENSP00000265562	ENSP00000353701
ENSP00000265562	ENSP00000354673
ENSP00000265562	ENSP00000354927
ENSP00000265562	ENSP00000356146
ENSP00000265562	ENSP00000365005
ENSP00000265562	ENSP00000366565
ENSP00000265562	ENSP00000375986
ENSP00000265562	ENSP00000379228
ENSP00000265562	ENSP00000402084
ENSP00000265562	ENSP00000417764
ENSP00000265565	ENSP00000304051
ENSP00000265565	ENSP00000344741
ENSP00000265643	ENSP00000308733
ENSP00000265651	ENSP00000300107
ENSP00000265689	ENSP00000274255
ENSP00000265689	ENSP00000303554
ENSP00000265689	ENSP00000314615
ENSP00000265689	ENSP00000317177
ENSP00000265689	ENSP00000320488
ENSP00000265689	ENSP00000321239
ENSP00000265689	ENSP00000325506
ENSP00000265689	ENSP00000325863
ENSP00000265689	ENSP00000342609
ENSP00000265689	ENSP00000342793
ENSP00000265689	ENSP00000361667
ENSP00000265689	ENSP00000362171
ENSP00000265689	ENSP00000369860
ENSP00000265689	ENSP00000376268
ENSP00000265689	ENSP00000386722
ENSP00000265717	ENSP00000273390
ENSP00000265717	ENSP00000277865
ENSP00000265717	ENSP00000286835
ENSP00000265717	ENSP00000287878

ENSP00000265717	ENSP00000295709
ENSP00000265717	ENSP00000297338
ENSP00000265717	ENSP00000300146
ENSP00000265717	ENSP00000301831
ENSP00000265717	ENSP00000310649
ENSP00000265717	ENSP00000310701
ENSP00000265717	ENSP00000315615
ENSP00000265717	ENSP00000318351
ENSP00000265717	ENSP00000318775
ENSP00000265717	ENSP00000329357
ENSP00000265717	ENSP00000337736
ENSP00000265717	ENSP00000339109
ENSP00000265717	ENSP00000343313
ENSP00000265717	ENSP00000344666
ENSP00000265717	ENSP00000346725
ENSP00000265717	ENSP00000348573
ENSP00000265717	ENSP00000351327
ENSP00000265717	ENSP00000352425
ENSP00000265717	ENSP00000353508
ENSP00000265717	ENSP00000354558
ENSP00000265717	ENSP00000356146
ENSP00000265717	ENSP00000360485
ENSP00000265717	ENSP00000361465
ENSP00000265717	ENSP00000363649
ENSP00000265717	ENSP00000383295
ENSP00000265717	ENSP00000384313
ENSP00000265717	ENSP00000396620
ENSP00000265717	ENSP00000403852
ENSP00000265727	ENSP00000281172
ENSP00000265727	ENSP00000360472
ENSP00000265728	ENSP00000270861
ENSP00000265728	ENSP00000274289
ENSP00000265728	ENSP00000286428
ENSP00000265728	ENSP00000297431
ENSP00000265728	ENSP00000301280
ENSP00000265728	ENSP00000317039
ENSP00000265728	ENSP00000321636
ENSP00000265728	ENSP00000322180
ENSP00000265728	ENSP00000335357
ENSP00000265728	ENSP00000337088
ENSP00000265728	ENSP00000339109
ENSP00000265728	ENSP00000340879
ENSP00000265728	ENSP00000343741
ENSP00000265728	ENSP00000351284
ENSP00000265728	ENSP00000353793
ENSP00000265728	ENSP00000354916

ENSP00000265728	ENSP00000360473
ENSP00000265728	ENSP00000360621
ENSP00000265728	ENSP00000368349
ENSP00000265728	ENSP00000368438
ENSP00000265728	ENSP00000369335
ENSP00000265728	ENSP00000370151
ENSP00000265728	ENSP00000372023
ENSP00000265728	ENSP00000380504
ENSP00000265728	ENSP00000413632
ENSP00000265732	ENSP00000298310
ENSP00000265732	ENSP00000299335
ENSP00000265732	ENSP00000320566
ENSP00000265732	ENSP00000325448
ENSP00000265732	ENSP00000356405
ENSP00000265732	ENSP00000360025
ENSP00000265732	ENSP00000380453
ENSP00000265748	ENSP00000299424
ENSP00000265748	ENSP00000360532
ENSP00000265748	ENSP00000380921
ENSP00000265753	ENSP00000266735
ENSP00000265753	ENSP00000268482
ENSP00000265753	ENSP00000293406
ENSP00000265753	ENSP00000303191
ENSP00000265753	ENSP00000305790
ENSP00000265753	ENSP00000312066
ENSP00000265753	ENSP00000327821
ENSP00000265753	ENSP00000328690
ENSP00000265753	ENSP00000335321
ENSP00000265753	ENSP00000336702
ENSP00000265753	ENSP00000338862
ENSP00000265753	ENSP00000341947
ENSP00000265753	ENSP00000346725
ENSP00000265753	ENSP00000348010
ENSP00000265753	ENSP00000359345
ENSP00000265753	ENSP00000362110
ENSP00000265753	ENSP00000366557
ENSP00000265753	ENSP00000376973
ENSP00000265753	ENSP00000398350
ENSP00000265753	ENSP00000400591
ENSP00000265753	ENSP00000412566
ENSP00000265758	ENSP00000277010
ENSP00000265758	ENSP00000309433
ENSP00000265758	ENSP00000311135
ENSP00000265758	ENSP00000312870
ENSP00000265758	ENSP00000325448
ENSP00000265758	ENSP00000332171

ENSP00000265758	ENSP00000333920
ENSP00000265758	ENSP00000364289
ENSP00000265758	ENSP00000376268
ENSP00000265758	ENSP00000380982
ENSP00000265758	ENSP00000396937
ENSP00000265806	ENSP00000388330
ENSP00000265810	ENSP00000350249
ENSP00000265810	ENSP00000358994
ENSP00000265810	ENSP00000377496
ENSP00000265825	ENSP00000283713
ENSP00000265827	ENSP00000341679
ENSP00000265849	ENSP00000265970
ENSP00000265849	ENSP00000267430
ENSP00000265849	ENSP00000278856
ENSP00000265849	ENSP00000285021
ENSP00000265849	ENSP00000286800
ENSP00000265849	ENSP00000287295
ENSP00000265849	ENSP00000290158
ENSP00000265849	ENSP00000298852
ENSP00000265849	ENSP00000301764
ENSP00000265849	ENSP00000307853
ENSP00000265849	ENSP00000310520
ENSP00000265849	ENSP00000311873
ENSP00000265849	ENSP00000312995
ENSP00000265849	ENSP00000313420
ENSP00000265849	ENSP00000318977
ENSP00000265849	ENSP00000322570
ENSP00000265849	ENSP00000324127
ENSP00000265849	ENSP00000325002
ENSP00000265849	ENSP00000343190
ENSP00000265849	ENSP00000347978
ENSP00000265849	ENSP00000348020
ENSP00000265849	ENSP00000351284
ENSP00000265849	ENSP00000351697
ENSP00000265849	ENSP00000354497
ENSP00000265849	ENSP00000359818
ENSP00000265849	ENSP00000361310
ENSP00000265849	ENSP00000364270
ENSP00000265849	ENSP00000368349
ENSP00000265849	ENSP00000368438
ENSP00000265849	ENSP00000369335
ENSP00000265849	ENSP00000369497
ENSP00000265849	ENSP00000384211
ENSP00000265857	ENSP00000270708
ENSP00000265857	ENSP00000272322
ENSP00000265857	ENSP00000286448

ENSP00000265857	ENSP00000290649
ENSP00000265857	ENSP00000294053
ENSP00000265857	ENSP00000297185
ENSP00000265857	ENSP00000303584
ENSP00000265857	ENSP00000305459
ENSP00000265857	ENSP00000309433
ENSP00000265857	ENSP00000310521
ENSP00000265857	ENSP00000314499
ENSP00000265857	ENSP00000327716
ENSP00000265857	ENSP00000328023
ENSP00000265857	ENSP00000333934
ENSP00000265857	ENSP00000338533
ENSP00000265857	ENSP00000347948
ENSP00000265857	ENSP00000349887
ENSP00000265857	ENSP00000350708
ENSP00000265857	ENSP00000357887
ENSP00000265857	ENSP00000362824
ENSP00000265857	ENSP00000364847
ENSP00000265857	ENSP00000377040
ENSP00000265857	ENSP00000383911
ENSP00000265857	ENSP00000390783
ENSP00000265857	ENSP00000397441
ENSP00000265872	ENSP00000286186
ENSP00000265872	ENSP00000296137
ENSP00000265872	ENSP00000307863
ENSP00000265872	ENSP00000310040
ENSP00000265872	ENSP00000323913
ENSP00000265872	ENSP00000326514
ENSP00000265872	ENSP00000329466
ENSP00000265872	ENSP00000352668
ENSP00000265872	ENSP00000360497
ENSP00000265872	ENSP00000365439
ENSP00000265872	ENSP00000366829
ENSP00000265872	ENSP00000382239
ENSP00000265872	ENSP00000417281
ENSP00000265881	ENSP00000296577
ENSP00000265881	ENSP00000321711
ENSP00000265881	ENSP00000340019
ENSP00000265881	ENSP00000343313
ENSP00000265881	ENSP00000352021
ENSP00000265881	ENSP00000361010
ENSP00000265881	ENSP00000366135
ENSP00000265881	ENSP00000372093
ENSP00000265896	ENSP00000269703
ENSP00000265896	ENSP00000272163
ENSP00000265896	ENSP00000274137

ENSP00000265896	ENSP00000277010
ENSP00000265896	ENSP00000282841
ENSP00000265896	ENSP00000285949
ENSP00000265896	ENSP00000307214
ENSP00000265896	ENSP00000310721
ENSP00000265896	ENSP00000320604
ENSP00000265896	ENSP00000325425
ENSP00000265896	ENSP00000330812
ENSP00000265896	ENSP00000331719
ENSP00000265896	ENSP00000334044
ENSP00000265896	ENSP00000339484
ENSP00000265896	ENSP00000341662
ENSP00000265896	ENSP00000342136
ENSP00000265896	ENSP00000348380
ENSP00000265896	ENSP00000349078
ENSP00000265896	ENSP00000355890
ENSP00000265896	ENSP00000356405
ENSP00000265896	ENSP00000357650
ENSP00000265896	ENSP00000360327
ENSP00000265896	ENSP00000360918
ENSP00000265896	ENSP00000366249
ENSP00000265896	ENSP00000368699
ENSP00000265896	ENSP00000369038
ENSP00000265896	ENSP00000373039
ENSP00000265896	ENSP00000396152
ENSP00000265896	ENSP00000401508
ENSP00000265896	ENSP00000410416
ENSP00000265944	ENSP00000268182
ENSP00000265944	ENSP00000295685
ENSP00000265944	ENSP00000303315
ENSP00000265944	ENSP00000352802
ENSP00000265960	ENSP00000272444
ENSP00000265960	ENSP00000272972
ENSP00000265960	ENSP00000298630
ENSP00000265960	ENSP00000301729
ENSP00000265960	ENSP00000306124
ENSP00000265960	ENSP00000307272
ENSP00000265960	ENSP00000311005
ENSP00000265960	ENSP00000322532
ENSP00000265960	ENSP00000346142
ENSP00000265960	ENSP00000347206
ENSP00000265960	ENSP00000349959
ENSP00000265960	ENSP00000354558
ENSP00000265960	ENSP00000358497
ENSP00000265960	ENSP00000359345
ENSP00000265960	ENSP00000359552

ENSP00000265960	ENSP00000362314
ENSP00000265960	ENSP00000369757
ENSP00000265960	ENSP00000371888
ENSP00000265960	ENSP00000373399
ENSP00000265960	ENSP00000377865
ENSP00000265960	ENSP00000389103
ENSP00000265960	ENSP00000399511
ENSP00000265960	ENSP00000401435
ENSP00000265963	ENSP00000273075
ENSP00000265963	ENSP00000278412
ENSP00000265963	ENSP00000282326
ENSP00000265963	ENSP00000282884
ENSP00000265963	ENSP00000283875
ENSP00000265963	ENSP00000285021
ENSP00000265963	ENSP00000285398
ENSP00000265963	ENSP00000307850
ENSP00000265963	ENSP00000310520
ENSP00000265963	ENSP00000317232
ENSP00000265963	ENSP00000317891
ENSP00000265963	ENSP00000341243
ENSP00000265963	ENSP00000347978
ENSP00000265963	ENSP00000348168
ENSP00000265963	ENSP00000350708
ENSP00000265963	ENSP00000359290
ENSP00000265963	ENSP00000359818
ENSP00000265963	ENSP00000362208
ENSP00000265963	ENSP00000363021
ENSP00000265963	ENSP00000363812
ENSP00000265963	ENSP00000364270
ENSP00000265963	ENSP00000368959
ENSP00000265963	ENSP00000373340
ENSP00000265963	ENSP00000375809
ENSP00000265963	ENSP00000384312
ENSP00000265963	ENSP00000413152
ENSP00000265970	ENSP00000272227
ENSP00000265970	ENSP00000278412
ENSP00000265970	ENSP00000279230
ENSP00000265970	ENSP00000283195
ENSP00000265970	ENSP00000294168
ENSP00000265970	ENSP00000298552
ENSP00000265970	ENSP00000302886
ENSP00000265970	ENSP00000306124
ENSP00000265970	ENSP00000307684
ENSP00000265970	ENSP00000317790
ENSP00000265970	ENSP00000325690
ENSP00000265970	ENSP00000337127

ENSP00000265970	ENSP00000343362
ENSP00000265970	ENSP00000344537
ENSP00000265970	ENSP00000346437
ENSP00000265970	ENSP00000357547
ENSP00000265970	ENSP00000358140
ENSP00000265970	ENSP00000361824
ENSP00000265970	ENSP00000362413
ENSP00000265970	ENSP00000362807
ENSP00000265970	ENSP00000365439
ENSP00000265970	ENSP00000369335
ENSP00000265970	ENSP00000406490
ENSP00000265981	ENSP00000302120
ENSP00000265983	ENSP00000277865
ENSP00000265983	ENSP00000295897
ENSP00000265990	ENSP00000267064
ENSP00000265990	ENSP00000270225
ENSP00000265990	ENSP00000272065
ENSP00000265990	ENSP00000278412
ENSP00000265990	ENSP00000283928
ENSP00000265990	ENSP00000285021
ENSP00000265990	ENSP00000290524
ENSP00000265990	ENSP00000294168
ENSP00000265990	ENSP00000296257
ENSP00000265990	ENSP00000296328
ENSP00000265990	ENSP00000300648
ENSP00000265990	ENSP00000301012
ENSP00000265990	ENSP00000302640
ENSP00000265990	ENSP00000306983
ENSP00000265990	ENSP00000307208
ENSP00000265990	ENSP00000307684
ENSP00000265990	ENSP00000307850
ENSP00000265990	ENSP00000308944
ENSP00000265990	ENSP00000312735
ENSP00000265990	ENSP00000314949
ENSP00000265990	ENSP00000320566
ENSP00000265990	ENSP00000322242
ENSP00000265990	ENSP00000323720
ENSP00000265990	ENSP00000325863
ENSP00000265990	ENSP00000326806
ENSP00000265990	ENSP00000327197
ENSP00000265990	ENSP00000329933
ENSP00000265990	ENSP00000331310
ENSP00000265990	ENSP00000332171
ENSP00000265990	ENSP00000332208
ENSP00000265990	ENSP00000333657
ENSP00000265990	ENSP00000333920

ENSP00000265990	ENSP00000334474
ENSP00000265990	ENSP00000336842
ENSP00000265990	ENSP00000336856
ENSP00000265990	ENSP00000341243
ENSP00000265990	ENSP00000342181
ENSP00000265990	ENSP00000342434
ENSP00000265990	ENSP00000342793
ENSP00000265990	ENSP00000344537
ENSP00000265990	ENSP00000347168
ENSP00000265990	ENSP00000347602
ENSP00000265990	ENSP00000347719
ENSP00000265990	ENSP00000347733
ENSP00000265990	ENSP00000348714
ENSP00000265990	ENSP00000349213
ENSP00000265990	ENSP00000350767
ENSP00000265990	ENSP00000351284
ENSP00000265990	ENSP00000351575
ENSP00000265990	ENSP00000353458
ENSP00000265990	ENSP00000353622
ENSP00000265990	ENSP00000354673
ENSP00000265990	ENSP00000354723
ENSP00000265990	ENSP00000355205
ENSP00000265990	ENSP00000358478
ENSP00000265990	ENSP00000358854
ENSP00000265990	ENSP00000358886
ENSP00000265990	ENSP00000359290
ENSP00000265990	ENSP00000359531
ENSP00000265990	ENSP00000360515
ENSP00000265990	ENSP00000360532
ENSP00000265990	ENSP00000361245
ENSP00000265990	ENSP00000361465
ENSP00000265990	ENSP00000362352
ENSP00000265990	ENSP00000362592
ENSP00000265990	ENSP00000364289
ENSP00000265990	ENSP00000366974
ENSP00000265990	ENSP00000367034
ENSP00000265990	ENSP00000369335
ENSP00000265990	ENSP00000369695
ENSP00000265990	ENSP00000369716
ENSP00000265990	ENSP00000374213
ENSP00000265990	ENSP00000374280
ENSP00000265990	ENSP00000376534
ENSP00000265990	ENSP00000377655
ENSP00000265990	ENSP00000379372
ENSP00000265990	ENSP00000384597
ENSP00000265990	ENSP00000394791

ENSP00000265990	ENSP00000397259
ENSP00000265990	ENSP00000397644
ENSP00000265990	ENSP00000402527
ENSP00000265990	ENSP00000417281
ENSP00000265990	ENSP00000418734
ENSP00000265993	ENSP00000304941
ENSP00000266000	ENSP00000268058
ENSP00000266000	ENSP00000291582
ENSP00000266000	ENSP00000296257
ENSP00000266000	ENSP00000301838
ENSP00000266000	ENSP00000306548
ENSP00000266000	ENSP00000307889
ENSP00000266000	ENSP00000312697
ENSP00000266000	ENSP00000326767
ENSP00000266000	ENSP00000329140
ENSP00000266000	ENSP00000340278
ENSP00000266000	ENSP00000341551
ENSP00000266000	ENSP00000343535
ENSP00000266000	ENSP00000344460
ENSP00000266000	ENSP00000347979
ENSP00000266000	ENSP00000349640
ENSP00000266000	ENSP00000351908
ENSP00000266000	ENSP00000352516
ENSP00000266000	ENSP00000355153
ENSP00000266000	ENSP00000355896
ENSP00000266000	ENSP00000358404
ENSP00000266000	ENSP00000362441
ENSP00000266000	ENSP00000364398
ENSP00000266000	ENSP00000366974
ENSP00000266000	ENSP00000373952
ENSP00000266000	ENSP00000380414
ENSP00000266000	ENSP00000396876
ENSP00000266000	ENSP00000404623
ENSP00000266000	ENSP00000417281
ENSP00000266022	ENSP00000305790
ENSP00000266022	ENSP00000329466
ENSP00000266022	ENSP00000375986
ENSP00000266025	ENSP00000288422
ENSP00000266025	ENSP00000352706
ENSP00000266025	ENSP00000356231
ENSP00000266025	ENSP00000358335
ENSP00000266025	ENSP00000361949
ENSP00000266025	ENSP00000394791
ENSP00000266037	ENSP00000300574
ENSP00000266037	ENSP00000305422
ENSP00000266037	ENSP00000313829

ENSP00000266037	ENSP00000326366
ENSP00000266037	ENSP00000369716
ENSP00000266037	ENSP00000387593
ENSP00000266058	ENSP00000355809
ENSP00000266058	ENSP00000356595
ENSP00000266058	ENSP00000381840
ENSP00000266066	ENSP00000268459
ENSP00000266066	ENSP00000310800
ENSP00000266066	ENSP00000352516
ENSP00000266066	ENSP00000363081
ENSP00000266066	ENSP00000404658
ENSP00000266069	ENSP00000268719
ENSP00000266069	ENSP00000294008
ENSP00000266069	ENSP00000300022
ENSP00000266069	ENSP00000302830
ENSP00000266069	ENSP00000311825
ENSP00000266069	ENSP00000315859
ENSP00000266069	ENSP00000320623
ENSP00000266069	ENSP00000351221
ENSP00000266069	ENSP00000358417
ENSP00000266069	ENSP00000374260
ENSP00000266069	ENSP00000379568
ENSP00000266070	ENSP00000269703
ENSP00000266070	ENSP00000295066
ENSP00000266070	ENSP00000299601
ENSP00000266070	ENSP00000304593
ENSP00000266070	ENSP00000308466
ENSP00000266070	ENSP00000329466
ENSP00000266070	ENSP00000353059
ENSP00000266070	ENSP00000356405
ENSP00000266070	ENSP00000356448
ENSP00000266070	ENSP00000357887
ENSP00000266070	ENSP00000360532
ENSP00000266070	ENSP00000365439
ENSP00000266070	ENSP00000374213
ENSP00000266070	ENSP00000374280
ENSP00000266070	ENSP00000386759
ENSP00000266079	ENSP00000266735
ENSP00000266079	ENSP00000267812
ENSP00000266079	ENSP00000268482
ENSP00000266079	ENSP00000268483
ENSP00000266079	ENSP00000270538
ENSP00000266079	ENSP00000271628
ENSP00000266079	ENSP00000274712
ENSP00000266079	ENSP00000278412
ENSP00000266079	ENSP00000300146

ENSP00000266079	ENSP00000300413
ENSP00000266079	ENSP00000301740
ENSP00000266079	ENSP00000301764
ENSP00000266079	ENSP00000302160
ENSP00000266079	ENSP00000304350
ENSP00000266079	ENSP00000304903
ENSP00000266079	ENSP00000305790
ENSP00000266079	ENSP00000308546
ENSP00000266079	ENSP00000310448
ENSP00000266079	ENSP00000310596
ENSP00000266079	ENSP00000312981
ENSP00000266079	ENSP00000313674
ENSP00000266079	ENSP00000315035
ENSP00000266079	ENSP00000315379
ENSP00000266079	ENSP00000315859
ENSP00000266079	ENSP00000318861
ENSP00000266079	ENSP00000319341
ENSP00000266079	ENSP00000324122
ENSP00000266079	ENSP00000329466
ENSP00000266079	ENSP00000331487
ENSP00000266079	ENSP00000331817
ENSP00000266079	ENSP00000332103
ENSP00000266079	ENSP00000335321
ENSP00000266079	ENSP00000337194
ENSP00000266079	ENSP00000337405
ENSP00000266079	ENSP00000342374
ENSP00000266079	ENSP00000347087
ENSP00000266079	ENSP00000348010
ENSP00000266079	ENSP00000348163
ENSP00000266079	ENSP00000358605
ENSP00000266079	ENSP00000360532
ENSP00000266079	ENSP00000361557
ENSP00000266079	ENSP00000361577
ENSP00000266079	ENSP00000362110
ENSP00000266079	ENSP00000363313
ENSP00000266079	ENSP00000364289
ENSP00000266079	ENSP00000364813
ENSP00000266079	ENSP00000372883
ENSP00000266079	ENSP00000378529
ENSP00000266079	ENSP00000387170
ENSP00000266079	ENSP00000391432
ENSP00000266079	ENSP00000396301
ENSP00000266079	ENSP00000400591
ENSP00000266079	ENSP00000403345
ENSP00000266079	ENSP00000406280
ENSP00000266079	ENSP00000410758

ENSP00000266079	ENSP00000412566
ENSP00000266079	ENSP00000414006
ENSP00000266079	ENSP00000414634
ENSP00000266079	ENSP00000416959
ENSP00000266087	ENSP00000304102
ENSP00000266087	ENSP00000311280
ENSP00000266087	ENSP00000317872
ENSP00000266087	ENSP00000327704
ENSP00000266095	ENSP00000267845
ENSP00000266095	ENSP00000272418
ENSP00000266095	ENSP00000274680
ENSP00000266095	ENSP00000280665
ENSP00000266095	ENSP00000282587
ENSP00000266095	ENSP00000287936
ENSP00000266095	ENSP00000295890
ENSP00000266095	ENSP00000298569
ENSP00000266095	ENSP00000302114
ENSP00000266095	ENSP00000302177
ENSP00000266095	ENSP00000302441
ENSP00000266095	ENSP00000313432
ENSP00000266095	ENSP00000314196
ENSP00000266095	ENSP00000317334
ENSP00000266095	ENSP00000319323
ENSP00000266095	ENSP00000320309
ENSP00000266095	ENSP00000322218
ENSP00000266095	ENSP00000331719
ENSP00000266095	ENSP00000340093
ENSP00000266095	ENSP00000341885
ENSP00000266095	ENSP00000343190
ENSP00000266095	ENSP00000348886
ENSP00000266095	ENSP00000350263
ENSP00000266095	ENSP00000350928
ENSP00000266095	ENSP00000353073
ENSP00000266095	ENSP00000354519
ENSP00000266095	ENSP00000356329
ENSP00000266095	ENSP00000356587
ENSP00000266095	ENSP00000361642
ENSP00000266095	ENSP00000362298
ENSP00000266095	ENSP00000365007
ENSP00000266095	ENSP00000368140
ENSP00000266095	ENSP00000368439
ENSP00000266095	ENSP00000369843
ENSP00000266095	ENSP00000379836
ENSP00000266095	ENSP00000382492
ENSP00000266095	ENSP00000389813
ENSP00000266095	ENSP00000415203

ENSP00000266126	ENSP00000266971
ENSP00000266126	ENSP00000269122
ENSP00000266126	ENSP00000273075
ENSP00000266126	ENSP00000273783
ENSP00000266126	ENSP00000284957
ENSP00000266126	ENSP00000285873
ENSP00000266126	ENSP00000287878
ENSP00000266126	ENSP00000302239
ENSP00000266126	ENSP00000302801
ENSP00000266126	ENSP00000304292
ENSP00000266126	ENSP00000304668
ENSP00000266126	ENSP00000317334
ENSP00000266126	ENSP00000325448
ENSP00000266126	ENSP00000330408
ENSP00000266126	ENSP00000339435
ENSP00000266126	ENSP00000340836
ENSP00000266126	ENSP00000342502
ENSP00000266126	ENSP00000343701
ENSP00000266126	ENSP00000343885
ENSP00000266126	ENSP00000344456
ENSP00000266126	ENSP00000345096
ENSP00000266126	ENSP00000349016
ENSP00000266126	ENSP00000350215
ENSP00000266126	ENSP00000352820
ENSP00000266126	ENSP00000353059
ENSP00000266126	ENSP00000353575
ENSP00000266126	ENSP00000364119
ENSP00000266126	ENSP00000364212
ENSP00000266126	ENSP00000365811
ENSP00000266126	ENSP00000366953
ENSP00000266126	ENSP00000369038
ENSP00000266126	ENSP00000374399
ENSP00000266126	ENSP00000377665
ENSP00000266126	ENSP00000381293
ENSP00000266126	ENSP00000382595
ENSP00000266126	ENSP00000392859
ENSP00000266126	ENSP00000394869
ENSP00000266182	ENSP00000280665
ENSP00000266182	ENSP00000313171
ENSP00000266182	ENSP00000320503
ENSP00000266269	ENSP00000286800
ENSP00000266483	ENSP00000295770
ENSP00000266483	ENSP00000296255
ENSP00000266483	ENSP00000299626
ENSP00000266483	ENSP00000333813
ENSP00000266483	ENSP00000340466

ENSP00000266483	ENSP00000360149
ENSP00000266483	ENSP00000360644
ENSP00000266483	ENSP00000364188
ENSP00000266483	ENSP00000380793
ENSP00000266529	ENSP00000297151
ENSP00000266529	ENSP00000303191
ENSP00000266529	ENSP00000338862
ENSP00000266529	ENSP00000366557
ENSP00000266529	ENSP00000376973
ENSP00000266529	ENSP00000400591
ENSP00000266544	ENSP00000268379
ENSP00000266544	ENSP00000301585
ENSP00000266544	ENSP00000325448
ENSP00000266544	ENSP00000327268
ENSP00000266544	ENSP00000330737
ENSP00000266544	ENSP00000343885
ENSP00000266544	ENSP00000356972
ENSP00000266544	ENSP00000360782
ENSP00000266544	ENSP00000364847
ENSP00000266544	ENSP00000380523
ENSP00000266544	ENSP00000387219
ENSP00000266544	ENSP00000397435
ENSP00000266556	ENSP00000278937
ENSP00000266556	ENSP00000307786
ENSP00000266556	ENSP00000341141
ENSP00000266556	ENSP00000348307
ENSP00000266556	ENSP00000356991
ENSP00000266556	ENSP00000382178
ENSP00000266556	ENSP00000382323
ENSP00000266557	ENSP00000276893
ENSP00000266557	ENSP00000342681
ENSP00000266604	ENSP00000345917
ENSP00000266604	ENSP00000379607
ENSP00000266671	ENSP00000345156
ENSP00000266671	ENSP00000361949
ENSP00000266673	ENSP00000354032
ENSP00000266712	ENSP00000280350
ENSP00000266712	ENSP00000298292
ENSP00000266712	ENSP00000377769
ENSP00000266712	ENSP00000397441
ENSP00000266732	ENSP00000267163
ENSP00000266732	ENSP00000268711
ENSP00000266732	ENSP00000296137
ENSP00000266732	ENSP00000297338
ENSP00000266732	ENSP00000320508
ENSP00000266732	ENSP00000337340

ENSP00000266732	ENSP00000343535
ENSP00000266732	ENSP00000344456
ENSP00000266732	ENSP00000351947
ENSP00000266732	ENSP00000353073
ENSP00000266732	ENSP00000357283
ENSP00000266732	ENSP00000360532
ENSP00000266732	ENSP00000370083
ENSP00000266735	ENSP00000268482
ENSP00000266735	ENSP00000268483
ENSP00000266735	ENSP00000272645
ENSP00000266735	ENSP00000281950
ENSP00000266735	ENSP00000285873
ENSP00000266735	ENSP00000293406
ENSP00000266735	ENSP00000300413
ENSP00000266735	ENSP00000301740
ENSP00000266735	ENSP00000301764
ENSP00000266735	ENSP00000302160
ENSP00000266735	ENSP00000303191
ENSP00000266735	ENSP00000304350
ENSP00000266735	ENSP00000304370
ENSP00000266735	ENSP00000304903
ENSP00000266735	ENSP00000305790
ENSP00000266735	ENSP00000306561
ENSP00000266735	ENSP00000308533
ENSP00000266735	ENSP00000308546
ENSP00000266735	ENSP00000310448
ENSP00000266735	ENSP00000310596
ENSP00000266735	ENSP00000313674
ENSP00000266735	ENSP00000314491
ENSP00000266735	ENSP00000315035
ENSP00000266735	ENSP00000315379
ENSP00000266735	ENSP00000318861
ENSP00000266735	ENSP00000322016
ENSP00000266735	ENSP00000323288
ENSP00000266735	ENSP00000323302
ENSP00000266735	ENSP00000324122
ENSP00000266735	ENSP00000325448
ENSP00000266735	ENSP00000326261
ENSP00000266735	ENSP00000326806
ENSP00000266735	ENSP00000334148
ENSP00000266735	ENSP00000335321
ENSP00000266735	ENSP00000337405
ENSP00000266735	ENSP00000342374
ENSP00000266735	ENSP00000346986
ENSP00000266735	ENSP00000348010
ENSP00000266735	ENSP00000358716

ENSP00000266735	ENSP00000359127
ENSP00000266735	ENSP00000359939
ENSP00000266735	ENSP00000360532
ENSP00000266735	ENSP00000360876
ENSP00000266735	ENSP00000362110
ENSP00000266735	ENSP00000362936
ENSP00000266735	ENSP00000363313
ENSP00000266735	ENSP00000364289
ENSP00000266735	ENSP00000366557
ENSP00000266735	ENSP00000370083
ENSP00000266735	ENSP00000370119
ENSP00000266735	ENSP00000376309
ENSP00000266735	ENSP00000376758
ENSP00000266735	ENSP00000376973
ENSP00000266735	ENSP00000378529
ENSP00000266735	ENSP00000379607
ENSP00000266735	ENSP00000400591
ENSP00000266735	ENSP00000410758
ENSP00000266735	ENSP00000412566
ENSP00000266735	ENSP00000416959
ENSP00000266735	ENSP00000420195
ENSP00000266743	ENSP00000308699
ENSP00000266743	ENSP00000341382
ENSP00000266743	ENSP00000354720
ENSP00000266743	ENSP00000369497
ENSP00000266744	ENSP00000272937
ENSP00000266744	ENSP00000277541
ENSP00000266744	ENSP00000302216
ENSP00000266744	ENSP00000316578
ENSP00000266744	ENSP00000317333
ENSP00000266744	ENSP00000332353
ENSP00000266744	ENSP00000350012
ENSP00000266744	ENSP00000358404
ENSP00000266971	ENSP00000325136
ENSP00000266971	ENSP00000334474
ENSP00000266971	ENSP00000337852
ENSP00000266971	ENSP00000378350
ENSP00000266971	ENSP00000394869
ENSP00000266971	ENSP00000407401
ENSP00000266980	ENSP00000355968
ENSP00000266980	ENSP00000379836
ENSP00000266987	ENSP00000282892
ENSP00000266987	ENSP00000311747
ENSP00000266987	ENSP00000334813
ENSP00000266987	ENSP00000343745
ENSP00000266987	ENSP00000344666

ENSP00000266987	ENSP00000360916
ENSP00000267017	ENSP00000307822
ENSP00000267023	ENSP00000278616
ENSP00000267023	ENSP00000310260
ENSP00000267023	ENSP00000318641
ENSP00000267023	ENSP00000327889
ENSP00000267023	ENSP00000385722
ENSP00000267064	ENSP00000269385
ENSP00000267064	ENSP00000269397
ENSP00000267064	ENSP00000271452
ENSP00000267064	ENSP00000271555
ENSP00000267064	ENSP00000276072
ENSP00000267064	ENSP00000276603
ENSP00000267064	ENSP00000278412
ENSP00000267064	ENSP00000283109
ENSP00000267064	ENSP00000286317
ENSP00000267064	ENSP00000287239
ENSP00000267064	ENSP00000290158
ENSP00000267064	ENSP00000290663
ENSP00000267064	ENSP00000292035
ENSP00000267064	ENSP00000293777
ENSP00000267064	ENSP00000297347
ENSP00000267064	ENSP00000298532
ENSP00000267064	ENSP00000299767
ENSP00000267064	ENSP00000300086
ENSP00000267064	ENSP00000300151
ENSP00000267064	ENSP00000306983
ENSP00000267064	ENSP00000308750
ENSP00000267064	ENSP00000311816
ENSP00000267064	ENSP00000314311
ENSP00000267064	ENSP00000314949
ENSP00000267064	ENSP00000315768
ENSP00000267064	ENSP00000317872
ENSP00000267064	ENSP00000320485
ENSP00000267064	ENSP00000323720
ENSP00000267064	ENSP00000323967
ENSP00000267064	ENSP00000324463
ENSP00000267064	ENSP00000325612
ENSP00000267064	ENSP00000325690
ENSP00000267064	ENSP00000325863
ENSP00000267064	ENSP00000326128
ENSP00000267064	ENSP00000326261
ENSP00000267064	ENSP00000326806
ENSP00000267064	ENSP00000329357
ENSP00000267064	ENSP00000329466
ENSP00000267064	ENSP00000334848

ENSP00000267064	ENSP00000335044
ENSP00000267064	ENSP00000336712
ENSP00000267064	ENSP00000341805
ENSP00000267064	ENSP00000342434
ENSP00000267064	ENSP00000342905
ENSP00000267064	ENSP00000344456
ENSP00000267064	ENSP00000344537
ENSP00000267064	ENSP00000347168
ENSP00000267064	ENSP00000348234
ENSP00000267064	ENSP00000349213
ENSP00000267064	ENSP00000350283
ENSP00000267064	ENSP00000350767
ENSP00000267064	ENSP00000351947
ENSP00000267064	ENSP00000353458
ENSP00000267064	ENSP00000353622
ENSP00000267064	ENSP00000353735
ENSP00000267064	ENSP00000354920
ENSP00000267064	ENSP00000354995
ENSP00000267064	ENSP00000355051
ENSP00000267064	ENSP00000357047
ENSP00000267064	ENSP00000357494
ENSP00000267064	ENSP00000358854
ENSP00000267064	ENSP00000359321
ENSP00000267064	ENSP00000360532
ENSP00000267064	ENSP00000362068
ENSP00000267064	ENSP00000362748
ENSP00000267064	ENSP00000362936
ENSP00000267064	ENSP00000362948
ENSP00000267064	ENSP00000363021
ENSP00000267064	ENSP00000364929
ENSP00000267064	ENSP00000365588
ENSP00000267064	ENSP00000366828
ENSP00000267064	ENSP00000366974
ENSP00000267064	ENSP00000367034
ENSP00000267064	ENSP00000368686
ENSP00000267064	ENSP00000369351
ENSP00000267064	ENSP00000369695
ENSP00000267064	ENSP00000370719
ENSP00000267064	ENSP00000371875
ENSP00000267064	ENSP00000373060
ENSP00000267064	ENSP00000373347
ENSP00000267064	ENSP00000374213
ENSP00000267064	ENSP00000378180
ENSP00000267064	ENSP00000378394
ENSP00000267064	ENSP00000379607
ENSP00000267064	ENSP00000381216

ENSP00000267064	ENSP00000384048
ENSP00000267064	ENSP00000384223
ENSP00000267064	ENSP00000384490
ENSP00000267064	ENSP00000387429
ENSP00000267064	ENSP00000391774
ENSP00000267064	ENSP00000392028
ENSP00000267064	ENSP00000392617
ENSP00000267064	ENSP00000404318
ENSP00000267064	ENSP00000408910
ENSP00000267064	ENSP00000409167
ENSP00000267064	ENSP00000417281
ENSP00000267068	ENSP00000363313
ENSP00000267101	ENSP00000274376
ENSP00000267101	ENSP00000277541
ENSP00000267101	ENSP00000278780
ENSP00000267101	ENSP00000281092
ENSP00000267101	ENSP00000286364
ENSP00000267101	ENSP00000288602
ENSP00000267101	ENSP00000289422
ENSP00000267101	ENSP00000296255
ENSP00000267101	ENSP00000300574
ENSP00000267101	ENSP00000302886
ENSP00000267101	ENSP00000303315
ENSP00000267101	ENSP00000306245
ENSP00000267101	ENSP00000334061
ENSP00000267101	ENSP00000338799
ENSP00000267101	ENSP00000338983
ENSP00000267101	ENSP00000339952
ENSP00000267101	ENSP00000340944
ENSP00000267101	ENSP00000341189
ENSP00000267101	ENSP00000342755
ENSP00000267101	ENSP00000343785
ENSP00000267101	ENSP00000344456
ENSP00000267101	ENSP00000347294
ENSP00000267101	ENSP00000350512
ENSP00000267101	ENSP00000353154
ENSP00000267101	ENSP00000355001
ENSP00000267101	ENSP00000356595
ENSP00000267101	ENSP00000358404
ENSP00000267101	ENSP00000359474
ENSP00000267101	ENSP00000360916
ENSP00000267101	ENSP00000363812
ENSP00000267101	ENSP00000364893
ENSP00000267101	ENSP00000364898
ENSP00000267101	ENSP00000372703
ENSP00000267101	ENSP00000378165

ENSP00000267101	ENSP00000383295
ENSP00000267101	ENSP00000384675
ENSP00000267101	ENSP00000393286
ENSP00000267101	ENSP00000393870
ENSP00000267101	ENSP00000399938
ENSP00000267101	ENSP00000400104
ENSP00000267101	ENSP00000402590
ENSP00000267101	ENSP00000415034
ENSP00000267101	ENSP00000417281
ENSP00000267101	ENSP00000418748
ENSP00000267103	ENSP00000270225
ENSP00000267103	ENSP00000293362
ENSP00000267103	ENSP00000301729
ENSP00000267103	ENSP00000322532
ENSP00000267103	ENSP00000331719
ENSP00000267103	ENSP00000336856
ENSP00000267103	ENSP00000338413
ENSP00000267103	ENSP00000350512
ENSP00000267103	ENSP00000352706
ENSP00000267103	ENSP00000366030
ENSP00000267103	ENSP00000368439
ENSP00000267103	ENSP00000368605
ENSP00000267103	ENSP00000420588
ENSP00000267113	ENSP00000300648
ENSP00000267113	ENSP00000346015
ENSP00000267163	ENSP00000267622
ENSP00000267163	ENSP00000268058
ENSP00000267163	ENSP00000269397
ENSP00000267163	ENSP00000274031
ENSP00000267163	ENSP00000274255
ENSP00000267163	ENSP00000276072
ENSP00000267163	ENSP00000276893
ENSP00000267163	ENSP00000284000
ENSP00000267163	ENSP00000286186
ENSP00000267163	ENSP00000287820
ENSP00000267163	ENSP00000292535
ENSP00000267163	ENSP00000294339
ENSP00000267163	ENSP00000296930
ENSP00000267163	ENSP00000301727
ENSP00000267163	ENSP00000302640
ENSP00000267163	ENSP00000302886
ENSP00000267163	ENSP00000303315
ENSP00000267163	ENSP00000303507
ENSP00000267163	ENSP00000305422
ENSP00000267163	ENSP00000305899
ENSP00000267163	ENSP00000306245

ENSP00000267163	ENSP00000312664
ENSP00000267163	ENSP00000314004
ENSP00000267163	ENSP00000317872
ENSP00000267163	ENSP00000318195
ENSP00000267163	ENSP00000318352
ENSP00000267163	ENSP00000320340
ENSP00000267163	ENSP00000323050
ENSP00000267163	ENSP00000324020
ENSP00000267163	ENSP00000325677
ENSP00000267163	ENSP00000329029
ENSP00000267163	ENSP00000329102
ENSP00000267163	ENSP00000329357
ENSP00000267163	ENSP00000331310
ENSP00000267163	ENSP00000332353
ENSP00000267163	ENSP00000336866
ENSP00000267163	ENSP00000338157
ENSP00000267163	ENSP00000340305
ENSP00000267163	ENSP00000341848
ENSP00000267163	ENSP00000341947
ENSP00000267163	ENSP00000342307
ENSP00000267163	ENSP00000347602
ENSP00000267163	ENSP00000348554
ENSP00000267163	ENSP00000348986
ENSP00000267163	ENSP00000349049
ENSP00000267163	ENSP00000350283
ENSP00000267163	ENSP00000350359
ENSP00000267163	ENSP00000351407
ENSP00000267163	ENSP00000351575
ENSP00000267163	ENSP00000351894
ENSP00000267163	ENSP00000352514
ENSP00000267163	ENSP00000352516
ENSP00000267163	ENSP00000354158
ENSP00000267163	ENSP00000354486
ENSP00000267163	ENSP00000355922
ENSP00000267163	ENSP00000357283
ENSP00000267163	ENSP00000362592
ENSP00000267163	ENSP00000363787
ENSP00000267163	ENSP00000364524
ENSP00000267163	ENSP00000365439
ENSP00000267163	ENSP00000365877
ENSP00000267163	ENSP00000365899
ENSP00000267163	ENSP00000367797
ENSP00000267163	ENSP00000367972
ENSP00000267163	ENSP00000368349
ENSP00000267163	ENSP00000368686
ENSP00000267163	ENSP00000369519

ENSP00000267163	ENSP00000370047
ENSP00000267163	ENSP00000372023
ENSP00000267163	ENSP00000378529
ENSP00000267163	ENSP00000382688
ENSP00000267163	ENSP00000384026
ENSP00000267163	ENSP00000386165
ENSP00000267163	ENSP00000392466
ENSP00000267163	ENSP00000396439
ENSP00000267163	ENSP00000413720
ENSP00000267163	ENSP00000414187
ENSP00000267163	ENSP00000417281
ENSP00000267169	ENSP00000314733
ENSP00000267169	ENSP00000340019
ENSP00000267169	ENSP00000351407
ENSP00000267169	ENSP00000393596
ENSP00000267176	ENSP00000277541
ENSP00000267176	ENSP00000328169
ENSP00000267197	ENSP00000278616
ENSP00000267197	ENSP00000280190
ENSP00000267197	ENSP00000283875
ENSP00000267197	ENSP00000286835
ENSP00000267197	ENSP00000290158
ENSP00000267197	ENSP00000295066
ENSP00000267197	ENSP00000296490
ENSP00000267197	ENSP00000296755
ENSP00000267197	ENSP00000300231
ENSP00000267197	ENSP00000300658
ENSP00000267197	ENSP00000304360
ENSP00000267197	ENSP00000304593
ENSP00000267197	ENSP00000307272
ENSP00000267197	ENSP00000311513
ENSP00000267197	ENSP00000312735
ENSP00000267197	ENSP00000312778
ENSP00000267197	ENSP00000314949
ENSP00000267197	ENSP00000317334
ENSP00000267197	ENSP00000319169
ENSP00000267197	ENSP00000320566
ENSP00000267197	ENSP00000325677
ENSP00000267197	ENSP00000329933
ENSP00000267197	ENSP00000340879
ENSP00000267197	ENSP00000340896
ENSP00000267197	ENSP00000341280
ENSP00000267197	ENSP00000343741
ENSP00000267197	ENSP00000348551
ENSP00000267197	ENSP00000349049
ENSP00000267197	ENSP00000351284

ENSP00000267197	ENSP00000352821
ENSP00000267197	ENSP00000353698
ENSP00000267197	ENSP00000353998
ENSP00000267197	ENSP00000354720
ENSP00000267197	ENSP00000354977
ENSP00000267197	ENSP00000355013
ENSP00000267197	ENSP00000356146
ENSP00000267197	ENSP00000360621
ENSP00000267197	ENSP00000366974
ENSP00000267197	ENSP00000369716
ENSP00000267197	ENSP00000370151
ENSP00000267197	ENSP00000370968
ENSP00000267197	ENSP00000371475
ENSP00000267197	ENSP00000374152
ENSP00000267197	ENSP00000374213
ENSP00000267197	ENSP00000376946
ENSP00000267197	ENSP00000377747
ENSP00000267197	ENSP00000381657
ENSP00000267197	ENSP00000382688
ENSP00000267197	ENSP00000386759
ENSP00000267197	ENSP00000390475
ENSP00000267197	ENSP00000414321
ENSP00000267294	ENSP00000358404
ENSP00000267339	ENSP00000348010
ENSP00000267339	ENSP00000349959
ENSP00000267396	ENSP00000267415
ENSP00000267415	ENSP00000268605
ENSP00000267415	ENSP00000276603
ENSP00000267415	ENSP00000280097
ENSP00000267415	ENSP00000290216
ENSP00000267415	ENSP00000292385
ENSP00000267415	ENSP00000299427
ENSP00000267415	ENSP00000300086
ENSP00000267415	ENSP00000301843
ENSP00000267415	ENSP00000303042
ENSP00000267415	ENSP00000303570
ENSP00000267415	ENSP00000304854
ENSP00000267415	ENSP00000319170
ENSP00000267415	ENSP00000321636
ENSP00000267415	ENSP00000322457
ENSP00000267415	ENSP00000323782
ENSP00000267415	ENSP00000324884
ENSP00000267415	ENSP00000338989
ENSP00000267415	ENSP00000346067
ENSP00000267415	ENSP00000348394
ENSP00000267415	ENSP00000350249

ENSP00000267415	ENSP00000353701
ENSP00000267415	ENSP00000354826
ENSP00000267415	ENSP00000358081
ENSP00000267415	ENSP00000361894
ENSP00000267415	ENSP00000361965
ENSP00000267415	ENSP00000367086
ENSP00000267415	ENSP00000368887
ENSP00000267415	ENSP00000370517
ENSP00000267415	ENSP00000370521
ENSP00000267415	ENSP00000372689
ENSP00000267415	ENSP00000377496
ENSP00000267415	ENSP00000384391
ENSP00000267415	ENSP00000391944
ENSP00000267415	ENSP00000407401
ENSP00000267425	ENSP00000272521
ENSP00000267425	ENSP00000274606
ENSP00000267425	ENSP00000278856
ENSP00000267425	ENSP00000291576
ENSP00000267425	ENSP00000296411
ENSP00000267425	ENSP00000296792
ENSP00000267425	ENSP00000297579
ENSP00000267425	ENSP00000297990
ENSP00000267425	ENSP00000298746
ENSP00000267425	ENSP00000301364
ENSP00000267425	ENSP00000302886
ENSP00000267425	ENSP00000305702
ENSP00000267425	ENSP00000311135
ENSP00000267425	ENSP00000314193
ENSP00000267425	ENSP00000314348
ENSP00000267425	ENSP00000318195
ENSP00000267425	ENSP00000321449
ENSP00000267425	ENSP00000324122
ENSP00000267425	ENSP00000325312
ENSP00000267425	ENSP00000327179
ENSP00000267425	ENSP00000331907
ENSP00000267425	ENSP00000338573
ENSP00000267425	ENSP00000343557
ENSP00000267425	ENSP00000346120
ENSP00000267425	ENSP00000354040
ENSP00000267425	ENSP00000355471
ENSP00000267425	ENSP00000355541
ENSP00000267425	ENSP00000356134
ENSP00000267425	ENSP00000358563
ENSP00000267425	ENSP00000358812
ENSP00000267425	ENSP00000360031
ENSP00000267425	ENSP00000361982

ENSP00000267425	ENSP00000362153
ENSP00000267425	ENSP00000363642
ENSP00000267425	ENSP00000363746
ENSP00000267425	ENSP00000365806
ENSP00000267425	ENSP00000366557
ENSP00000267425	ENSP00000369042
ENSP00000267425	ENSP00000370589
ENSP00000267425	ENSP00000371101
ENSP00000267425	ENSP00000372695
ENSP00000267425	ENSP00000373090
ENSP00000267425	ENSP00000374390
ENSP00000267425	ENSP00000384302
ENSP00000267425	ENSP00000386621
ENSP00000267425	ENSP00000394290
ENSP00000267425	ENSP00000399454
ENSP00000267425	ENSP00000402869
ENSP00000267425	ENSP00000403576
ENSP00000267425	ENSP00000405614
ENSP00000267425	ENSP00000409074
ENSP00000267425	ENSP00000409367
ENSP00000267425	ENSP00000411162
ENSP00000267430	ENSP00000268043
ENSP00000267430	ENSP00000268206
ENSP00000267430	ENSP00000287437
ENSP00000267430	ENSP00000289081
ENSP00000267430	ENSP00000290949
ENSP00000267430	ENSP00000296215
ENSP00000267430	ENSP00000296509
ENSP00000267430	ENSP00000296805
ENSP00000267430	ENSP00000302951
ENSP00000267430	ENSP00000302955
ENSP00000267430	ENSP00000306561
ENSP00000267430	ENSP00000307853
ENSP00000267430	ENSP00000308944
ENSP00000267430	ENSP00000310356
ENSP00000267430	ENSP00000310520
ENSP00000267430	ENSP00000311873
ENSP00000267430	ENSP00000313171
ENSP00000267430	ENSP00000314491
ENSP00000267430	ENSP00000315700
ENSP00000267430	ENSP00000317039
ENSP00000267430	ENSP00000318977
ENSP00000267430	ENSP00000321636
ENSP00000267430	ENSP00000322570
ENSP00000267430	ENSP00000323439
ENSP00000267430	ENSP00000323511

ENSP00000267430	ENSP00000325863
ENSP00000267430	ENSP00000326819
ENSP00000267430	ENSP00000330875
ENSP00000267430	ENSP00000333283
ENSP00000267430	ENSP00000334188
ENSP00000267430	ENSP00000336606
ENSP00000267430	ENSP00000337353
ENSP00000267430	ENSP00000340305
ENSP00000267430	ENSP00000340879
ENSP00000267430	ENSP00000341243
ENSP00000267430	ENSP00000341382
ENSP00000267430	ENSP00000343392
ENSP00000267430	ENSP00000343741
ENSP00000267430	ENSP00000343745
ENSP00000267430	ENSP00000344741
ENSP00000267430	ENSP00000345917
ENSP00000267430	ENSP00000347232
ENSP00000267430	ENSP00000347978
ENSP00000267430	ENSP00000349393
ENSP00000267430	ENSP00000351284
ENSP00000267430	ENSP00000351697
ENSP00000267430	ENSP00000352257
ENSP00000267430	ENSP00000353332
ENSP00000267430	ENSP00000354522
ENSP00000267430	ENSP00000354720
ENSP00000267430	ENSP00000354957
ENSP00000267430	ENSP00000355077
ENSP00000267430	ENSP00000355185
ENSP00000267430	ENSP00000355388
ENSP00000267430	ENSP00000358019
ENSP00000267430	ENSP00000358478
ENSP00000267430	ENSP00000358576
ENSP00000267430	ENSP00000359818
ENSP00000267430	ENSP00000359904
ENSP00000267430	ENSP00000360116
ENSP00000267430	ENSP00000360310
ENSP00000267430	ENSP00000360803
ENSP00000267430	ENSP00000361310
ENSP00000267430	ENSP00000363021
ENSP00000267430	ENSP00000364126
ENSP00000267430	ENSP00000364270
ENSP00000267430	ENSP00000364699
ENSP00000267430	ENSP00000367527
ENSP00000267430	ENSP00000367787
ENSP00000267430	ENSP00000367808
ENSP00000267430	ENSP00000367910

ENSP00000267430	ENSP00000368349
ENSP00000267430	ENSP00000368438
ENSP00000267430	ENSP00000369335
ENSP00000267430	ENSP00000370151
ENSP00000267430	ENSP00000370376
ENSP00000267430	ENSP00000371475
ENSP00000267430	ENSP00000373952
ENSP00000267430	ENSP00000381657
ENSP00000267430	ENSP00000385021
ENSP00000267430	ENSP00000394649
ENSP00000267430	ENSP00000396704
ENSP00000267430	ENSP00000400806
ENSP00000267430	ENSP00000402515
ENSP00000267430	ENSP00000406490
ENSP00000267430	ENSP00000406868
ENSP00000267430	ENSP00000407047
ENSP00000267484	ENSP00000269159
ENSP00000267484	ENSP00000273398
ENSP00000267484	ENSP00000280527
ENSP00000267484	ENSP00000290299
ENSP00000267484	ENSP00000292475
ENSP00000267484	ENSP00000294724
ENSP00000267484	ENSP00000320885
ENSP00000267484	ENSP00000321606
ENSP00000267484	ENSP00000323377
ENSP00000267484	ENSP00000332979
ENSP00000267484	ENSP00000337838
ENSP00000267484	ENSP00000344106
ENSP00000267484	ENSP00000345656
ENSP00000267484	ENSP00000347232
ENSP00000267484	ENSP00000355777
ENSP00000267484	ENSP00000363349
ENSP00000267484	ENSP00000363397
ENSP00000267484	ENSP00000368959
ENSP00000267484	ENSP00000369862
ENSP00000267484	ENSP00000385083
ENSP00000267484	ENSP00000389934
ENSP00000267522	ENSP00000269159
ENSP00000267540	ENSP00000276570
ENSP00000267540	ENSP00000360798
ENSP00000267540	ENSP00000370719
ENSP00000267584	ENSP00000298545
ENSP00000267584	ENSP00000346577
ENSP00000267615	ENSP00000346340
ENSP00000267615	ENSP00000350512
ENSP00000267615	ENSP00000360532

ENSP00000267622	ENSP00000351407
ENSP00000267622	ENSP00000379607
ENSP00000267750	ENSP00000327716
ENSP00000267750	ENSP00000366565
ENSP00000267750	ENSP00000420608
ENSP00000267807	ENSP00000295951
ENSP00000267807	ENSP00000298317
ENSP00000267807	ENSP00000322915
ENSP00000267807	ENSP00000323288
ENSP00000267807	ENSP00000336587
ENSP00000267807	ENSP00000350094
ENSP00000267807	ENSP00000373331
ENSP00000267812	ENSP00000268206
ENSP00000267812	ENSP00000268482
ENSP00000267812	ENSP00000271628
ENSP00000267812	ENSP00000274712
ENSP00000267812	ENSP00000295488
ENSP00000267812	ENSP00000296215
ENSP00000267812	ENSP00000296702
ENSP00000267812	ENSP00000296805
ENSP00000267812	ENSP00000298532
ENSP00000267812	ENSP00000300413
ENSP00000267812	ENSP00000301764
ENSP00000267812	ENSP00000303191
ENSP00000267812	ENSP00000304350
ENSP00000267812	ENSP00000305790
ENSP00000267812	ENSP00000307863
ENSP00000267812	ENSP00000308369
ENSP00000267812	ENSP00000310448
ENSP00000267812	ENSP00000310723
ENSP00000267812	ENSP00000315379
ENSP00000267812	ENSP00000318861
ENSP00000267812	ENSP00000319341
ENSP00000267812	ENSP00000323302
ENSP00000267812	ENSP00000324122
ENSP00000267812	ENSP00000332103
ENSP00000267812	ENSP00000335321
ENSP00000267812	ENSP00000339090
ENSP00000267812	ENSP00000342374
ENSP00000267812	ENSP00000346236
ENSP00000267812	ENSP00000351137
ENSP00000267812	ENSP00000357631
ENSP00000267812	ENSP00000358554
ENSP00000267812	ENSP00000358605
ENSP00000267812	ENSP00000359454
ENSP00000267812	ENSP00000360488

ENSP00000267812	ENSP00000360532
ENSP00000267812	ENSP00000360828
ENSP00000267812	ENSP00000361577
ENSP00000267812	ENSP00000362110
ENSP00000267812	ENSP00000363313
ENSP00000267812	ENSP00000366557
ENSP00000267812	ENSP00000370589
ENSP00000267812	ENSP00000378338
ENSP00000267812	ENSP00000380336
ENSP00000267812	ENSP00000394085
ENSP00000267812	ENSP00000396301
ENSP00000267812	ENSP00000412566
ENSP00000267812	ENSP00000419851
ENSP00000267845	ENSP00000268251
ENSP00000267845	ENSP00000370571
ENSP00000267884	ENSP00000282999
ENSP00000267884	ENSP00000283871
ENSP00000267884	ENSP00000294360
ENSP00000267884	ENSP00000305230
ENSP00000267884	ENSP00000312066
ENSP00000267884	ENSP00000342181
ENSP00000267884	ENSP00000346120
ENSP00000267884	ENSP00000370589
ENSP00000267884	ENSP00000379607
ENSP00000267884	ENSP00000403817
ENSP00000267935	ENSP00000287598
ENSP00000267935	ENSP00000308236
ENSP00000267953	ENSP00000293288
ENSP00000267953	ENSP00000309103
ENSP00000267953	ENSP00000314132
ENSP00000267953	ENSP00000318822
ENSP00000267953	ENSP00000326119
ENSP00000267953	ENSP00000346697
ENSP00000267953	ENSP00000363591
ENSP00000267970	ENSP00000381031
ENSP00000267978	ENSP00000273075
ENSP00000267978	ENSP00000288976
ENSP00000267978	ENSP00000299853
ENSP00000267978	ENSP00000335357
ENSP00000267978	ENSP00000354720
ENSP00000267978	ENSP00000354916
ENSP00000267978	ENSP00000357674
ENSP00000267978	ENSP00000361465
ENSP00000267978	ENSP00000365175
ENSP00000267978	ENSP00000387477
ENSP00000267978	ENSP00000415363

ENSP00000268043	ENSP00000278616
ENSP00000268043	ENSP00000287598
ENSP00000268043	ENSP00000288774
ENSP00000268043	ENSP00000296273
ENSP00000268043	ENSP00000296755
ENSP00000268043	ENSP00000297431
ENSP00000268043	ENSP00000298139
ENSP00000268043	ENSP00000300231
ENSP00000268043	ENSP00000300738
ENSP00000268043	ENSP00000302530
ENSP00000268043	ENSP00000306561
ENSP00000268043	ENSP00000307853
ENSP00000268043	ENSP00000308944
ENSP00000268043	ENSP00000309572
ENSP00000268043	ENSP00000310520
ENSP00000268043	ENSP00000311873
ENSP00000268043	ENSP00000313171
ENSP00000268043	ENSP00000317039
ENSP00000268043	ENSP00000317636
ENSP00000268043	ENSP00000318977
ENSP00000268043	ENSP00000321636
ENSP00000268043	ENSP00000322180
ENSP00000268043	ENSP00000322570
ENSP00000268043	ENSP00000323439
ENSP00000268043	ENSP00000324792
ENSP00000268043	ENSP00000325863
ENSP00000268043	ENSP00000326534
ENSP00000268043	ENSP00000330343
ENSP00000268043	ENSP00000336606
ENSP00000268043	ENSP00000341243
ENSP00000268043	ENSP00000341382
ENSP00000268043	ENSP00000343392
ENSP00000268043	ENSP00000343741
ENSP00000268043	ENSP00000347232
ENSP00000268043	ENSP00000347978
ENSP00000268043	ENSP00000349393
ENSP00000268043	ENSP00000350491
ENSP00000268043	ENSP00000351284
ENSP00000268043	ENSP00000351524
ENSP00000268043	ENSP00000351697
ENSP00000268043	ENSP00000351885
ENSP00000268043	ENSP00000352257
ENSP00000268043	ENSP00000353793
ENSP00000268043	ENSP00000354522
ENSP00000268043	ENSP00000354957
ENSP00000268043	ENSP00000355922

ENSP00000268043	ENSP00000358478
ENSP00000268043	ENSP00000360817
ENSP00000268043	ENSP00000362135
ENSP00000268043	ENSP00000363021
ENSP00000268043	ENSP00000365588
ENSP00000268043	ENSP00000366135
ENSP00000268043	ENSP00000367787
ENSP00000268043	ENSP00000368349
ENSP00000268043	ENSP00000368438
ENSP00000268043	ENSP00000369335
ENSP00000268043	ENSP00000369411
ENSP00000268043	ENSP00000370151
ENSP00000268043	ENSP00000371475
ENSP00000268043	ENSP00000372023
ENSP00000268043	ENSP00000373060
ENSP00000268043	ENSP00000375809
ENSP00000268043	ENSP00000384048
ENSP00000268043	ENSP00000386759
ENSP00000268043	ENSP00000387429
ENSP00000268043	ENSP00000396484
ENSP00000268043	ENSP00000402515
ENSP00000268043	ENSP00000404318
ENSP00000268043	ENSP00000409167
ENSP00000268043	ENSP00000415212
ENSP00000268043	ENSP00000416511
ENSP00000268043	ENSP00000417963
ENSP00000268057	ENSP00000274353
ENSP00000268057	ENSP00000280350
ENSP00000268057	ENSP00000295240
ENSP00000268057	ENSP00000298292
ENSP00000268057	ENSP00000319062
ENSP00000268057	ENSP00000327077
ENSP00000268057	ENSP00000331746
ENSP00000268057	ENSP00000334100
ENSP00000268057	ENSP00000353701
ENSP00000268057	ENSP00000354791
ENSP00000268057	ENSP00000358140
ENSP00000268057	ENSP00000370031
ENSP00000268057	ENSP00000397644
ENSP00000268058	ENSP00000282728
ENSP00000268058	ENSP00000286317
ENSP00000268058	ENSP00000287820
ENSP00000268058	ENSP00000288199
ENSP00000268058	ENSP00000293362
ENSP00000268058	ENSP00000296257
ENSP00000268058	ENSP00000296273

ENSP00000268058	ENSP00000296930
ENSP00000268058	ENSP00000300086
ENSP00000268058	ENSP00000306163
ENSP00000268058	ENSP00000306245
ENSP00000268058	ENSP00000309572
ENSP00000268058	ENSP00000311005
ENSP00000268058	ENSP00000311280
ENSP00000268058	ENSP00000316578
ENSP00000268058	ENSP00000321636
ENSP00000268058	ENSP00000327048
ENSP00000268058	ENSP00000327959
ENSP00000268058	ENSP00000328088
ENSP00000268058	ENSP00000329357
ENSP00000268058	ENSP00000329623
ENSP00000268058	ENSP00000330343
ENSP00000268058	ENSP00000333275
ENSP00000268058	ENSP00000338157
ENSP00000268058	ENSP00000341024
ENSP00000268058	ENSP00000343023
ENSP00000268058	ENSP00000345681
ENSP00000268058	ENSP00000347836
ENSP00000268058	ENSP00000347979
ENSP00000268058	ENSP00000348551
ENSP00000268058	ENSP00000351407
ENSP00000268058	ENSP00000353622
ENSP00000268058	ENSP00000354558
ENSP00000268058	ENSP00000355153
ENSP00000268058	ENSP00000357965
ENSP00000268058	ENSP00000360116
ENSP00000268058	ENSP00000363676
ENSP00000268058	ENSP00000364320
ENSP00000268058	ENSP00000365877
ENSP00000268058	ENSP00000366607
ENSP00000268058	ENSP00000367797
ENSP00000268058	ENSP00000376611
ENSP00000268058	ENSP00000376903
ENSP00000268058	ENSP00000381045
ENSP00000268058	ENSP00000384048
ENSP00000268058	ENSP00000396704
ENSP00000268058	ENSP00000396876
ENSP00000268058	ENSP00000402527
ENSP00000268058	ENSP00000404623
ENSP00000268058	ENSP00000417281
ENSP00000268124	ENSP00000290429
ENSP00000268124	ENSP00000299206
ENSP00000268124	ENSP00000300738

ENSP00000268124	ENSP00000322570
ENSP00000268124	ENSP00000351697
ENSP00000268124	ENSP00000360216
ENSP00000268124	ENSP00000361310
ENSP00000268124	ENSP00000368349
ENSP00000268164	ENSP00000399436
ENSP00000268182	ENSP00000276326
ENSP00000268182	ENSP00000282441
ENSP00000268182	ENSP00000285873
ENSP00000268182	ENSP00000286835
ENSP00000268182	ENSP00000296733
ENSP00000268182	ENSP00000298910
ENSP00000268182	ENSP00000300648
ENSP00000268182	ENSP00000305785
ENSP00000268182	ENSP00000307272
ENSP00000268182	ENSP00000315615
ENSP00000268182	ENSP00000322608
ENSP00000268182	ENSP00000326514
ENSP00000268182	ENSP00000329466
ENSP00000268182	ENSP00000337088
ENSP00000268182	ENSP00000344456
ENSP00000268182	ENSP00000348163
ENSP00000268182	ENSP00000349016
ENSP00000268182	ENSP00000351015
ENSP00000268182	ENSP00000351665
ENSP00000268182	ENSP00000354927
ENSP00000268182	ENSP00000356146
ENSP00000268182	ENSP00000356530
ENSP00000268182	ENSP00000358994
ENSP00000268182	ENSP00000360076
ENSP00000268182	ENSP00000360532
ENSP00000268182	ENSP00000360922
ENSP00000268182	ENSP00000364403
ENSP00000268182	ENSP00000368190
ENSP00000268182	ENSP00000368797
ENSP00000268182	ENSP00000378529
ENSP00000268182	ENSP00000382177
ENSP00000268182	ENSP00000384675
ENSP00000268182	ENSP00000391249
ENSP00000268182	ENSP00000397598
ENSP00000268206	ENSP00000268661
ENSP00000268206	ENSP00000270625
ENSP00000268206	ENSP00000272418
ENSP00000268206	ENSP00000274712
ENSP00000268206	ENSP00000278572
ENSP00000268206	ENSP00000284719

ENSP00000268206	ENSP00000288937
ENSP00000268206	ENSP00000295488
ENSP00000268206	ENSP00000296674
ENSP00000268206	ENSP00000297908
ENSP00000268206	ENSP00000298283
ENSP00000268206	ENSP00000300151
ENSP00000268206	ENSP00000302160
ENSP00000268206	ENSP00000302896
ENSP00000268206	ENSP00000306548
ENSP00000268206	ENSP00000307853
ENSP00000268206	ENSP00000308897
ENSP00000268206	ENSP00000309474
ENSP00000268206	ENSP00000310448
ENSP00000268206	ENSP00000310520
ENSP00000268206	ENSP00000311028
ENSP00000268206	ENSP00000311430
ENSP00000268206	ENSP00000312735
ENSP00000268206	ENSP00000315379
ENSP00000268206	ENSP00000317376
ENSP00000268206	ENSP00000318646
ENSP00000268206	ENSP00000318861
ENSP00000268206	ENSP00000321320
ENSP00000268206	ENSP00000325448
ENSP00000268206	ENSP00000326981
ENSP00000268206	ENSP00000335321
ENSP00000268206	ENSP00000336606
ENSP00000268206	ENSP00000336702
ENSP00000268206	ENSP00000339795
ENSP00000268206	ENSP00000340305
ENSP00000268206	ENSP00000341082
ENSP00000268206	ENSP00000341885
ENSP00000268206	ENSP00000343557
ENSP00000268206	ENSP00000346015
ENSP00000268206	ENSP00000346022
ENSP00000268206	ENSP00000346067
ENSP00000268206	ENSP00000351137
ENSP00000268206	ENSP00000354125
ENSP00000268206	ENSP00000354525
ENSP00000268206	ENSP00000354739
ENSP00000268206	ENSP00000356811
ENSP00000268206	ENSP00000357631
ENSP00000268206	ENSP00000358554
ENSP00000268206	ENSP00000359345
ENSP00000268206	ENSP00000359454
ENSP00000268206	ENSP00000362036
ENSP00000268206	ENSP00000362110

ENSP00000268206	ENSP00000363018
ENSP00000268206	ENSP00000363559
ENSP00000268206	ENSP00000363676
ENSP00000268206	ENSP00000364289
ENSP00000268206	ENSP00000364320
ENSP00000268206	ENSP00000366557
ENSP00000268206	ENSP00000373404
ENSP00000268206	ENSP00000375730
ENSP00000268206	ENSP00000376973
ENSP00000268206	ENSP00000377486
ENSP00000268206	ENSP00000377865
ENSP00000268206	ENSP00000379339
ENSP00000268206	ENSP00000387006
ENSP00000268206	ENSP00000388330
ENSP00000268206	ENSP00000389103
ENSP00000268206	ENSP00000393241
ENSP00000268206	ENSP00000393953
ENSP00000268206	ENSP00000403175
ENSP00000268206	ENSP00000412583
ENSP00000268206	ENSP00000416110
ENSP00000268206	ENSP00000417229
ENSP00000268206	ENSP00000419599
ENSP00000268206	ENSP00000420714
ENSP00000268251	ENSP00000300574
ENSP00000268251	ENSP00000310623
ENSP00000268251	ENSP00000350928
ENSP00000268251	ENSP00000356104
ENSP00000268251	ENSP00000359518
ENSP00000268251	ENSP00000362298
ENSP00000268251	ENSP00000370611
ENSP00000268379	ENSP00000270538
ENSP00000268379	ENSP00000271373
ENSP00000268379	ENSP00000274787
ENSP00000268379	ENSP00000282050
ENSP00000268379	ENSP00000283429
ENSP00000268379	ENSP00000285083
ENSP00000268379	ENSP00000287022
ENSP00000268379	ENSP00000290299
ENSP00000268379	ENSP00000295767
ENSP00000268379	ENSP00000296674
ENSP00000268379	ENSP00000297564
ENSP00000268379	ENSP00000298852
ENSP00000268379	ENSP00000299335
ENSP00000268379	ENSP00000299767
ENSP00000268379	ENSP00000301585
ENSP00000268379	ENSP00000307786

ENSP00000268379	ENSP00000309565
ENSP00000268379	ENSP00000314380
ENSP00000268379	ENSP00000317159
ENSP00000268379	ENSP00000317334
ENSP00000268379	ENSP00000317780
ENSP00000268379	ENSP00000320236
ENSP00000268379	ENSP00000323046
ENSP00000268379	ENSP00000325677
ENSP00000268379	ENSP00000328940
ENSP00000268379	ENSP00000329466
ENSP00000268379	ENSP00000332887
ENSP00000268379	ENSP00000337838
ENSP00000268379	ENSP00000338293
ENSP00000268379	ENSP00000338345
ENSP00000268379	ENSP00000339933
ENSP00000268379	ENSP00000340019
ENSP00000268379	ENSP00000346067
ENSP00000268379	ENSP00000349142
ENSP00000268379	ENSP00000353698
ENSP00000268379	ENSP00000354499
ENSP00000268379	ENSP00000354554
ENSP00000268379	ENSP00000354876
ENSP00000268379	ENSP00000354982
ENSP00000268379	ENSP00000355572
ENSP00000268379	ENSP00000358042
ENSP00000268379	ENSP00000358695
ENSP00000268379	ENSP00000359067
ENSP00000268379	ENSP00000359127
ENSP00000268379	ENSP00000361042
ENSP00000268379	ENSP00000361207
ENSP00000268379	ENSP00000361433
ENSP00000268379	ENSP00000363018
ENSP00000268379	ENSP00000365243
ENSP00000268379	ENSP00000365766
ENSP00000268379	ENSP00000367923
ENSP00000268379	ENSP00000367934
ENSP00000268379	ENSP00000368523
ENSP00000268379	ENSP00000377527
ENSP00000268379	ENSP00000387262
ENSP00000268379	ENSP00000392985
ENSP00000268379	ENSP00000404381
ENSP00000268459	ENSP00000322304
ENSP00000268459	ENSP00000344456
ENSP00000268459	ENSP00000346155
ENSP00000268459	ENSP00000351605
ENSP00000268459	ENSP00000410715

ENSP00000268482	ENSP00000268483
ENSP00000268482	ENSP00000268802
ENSP00000268482	ENSP00000296215
ENSP00000268482	ENSP00000297151
ENSP00000268482	ENSP00000297990
ENSP00000268482	ENSP00000300086
ENSP00000268482	ENSP00000301740
ENSP00000268482	ENSP00000301764
ENSP00000268482	ENSP00000303191
ENSP00000268482	ENSP00000304350
ENSP00000268482	ENSP00000304370
ENSP00000268482	ENSP00000305790
ENSP00000268482	ENSP00000310448
ENSP00000268482	ENSP00000312981
ENSP00000268482	ENSP00000315379
ENSP00000268482	ENSP00000315674
ENSP00000268482	ENSP00000315859
ENSP00000268482	ENSP00000324122
ENSP00000268482	ENSP00000326806
ENSP00000268482	ENSP00000328854
ENSP00000268482	ENSP00000329466
ENSP00000268482	ENSP00000331817
ENSP00000268482	ENSP00000331907
ENSP00000268482	ENSP00000335321
ENSP00000268482	ENSP00000339723
ENSP00000268482	ENSP00000343557
ENSP00000268482	ENSP00000346022
ENSP00000268482	ENSP00000351137
ENSP00000268482	ENSP00000351492
ENSP00000268482	ENSP00000352738
ENSP00000268482	ENSP00000355541
ENSP00000268482	ENSP00000358812
ENSP00000268482	ENSP00000360031
ENSP00000268482	ENSP00000360497
ENSP00000268482	ENSP00000360532
ENSP00000268482	ENSP00000361557
ENSP00000268482	ENSP00000361577
ENSP00000268482	ENSP00000362110
ENSP00000268482	ENSP00000362936
ENSP00000268482	ENSP00000363313
ENSP00000268482	ENSP00000364289
ENSP00000268482	ENSP00000366135
ENSP00000268482	ENSP00000366557
ENSP00000268482	ENSP00000366829
ENSP00000268482	ENSP00000367835
ENSP00000268482	ENSP00000370222

ENSP00000268482	ENSP00000370460
ENSP00000268482	ENSP00000371169
ENSP00000268482	ENSP00000376973
ENSP00000268482	ENSP00000383059
ENSP00000268482	ENSP00000387006
ENSP00000268482	ENSP00000391432
ENSP00000268483	ENSP00000274712
ENSP00000268483	ENSP00000278572
ENSP00000268483	ENSP00000292180
ENSP00000268483	ENSP00000300146
ENSP00000268483	ENSP00000300413
ENSP00000268483	ENSP00000302160
ENSP00000268483	ENSP00000304350
ENSP00000268483	ENSP00000305790
ENSP00000268483	ENSP00000310448
ENSP00000268483	ENSP00000310596
ENSP00000268483	ENSP00000315035
ENSP00000268483	ENSP00000315379
ENSP00000268483	ENSP00000318861
ENSP00000268483	ENSP00000319341
ENSP00000268483	ENSP00000324122
ENSP00000268483	ENSP00000332103
ENSP00000268483	ENSP00000335321
ENSP00000268483	ENSP00000337405
ENSP00000268483	ENSP00000342374
ENSP00000268483	ENSP00000348010
ENSP00000268483	ENSP00000348602
ENSP00000268483	ENSP00000357880
ENSP00000268483	ENSP00000361577
ENSP00000268483	ENSP00000362110
ENSP00000268483	ENSP00000364813
ENSP00000268483	ENSP00000372883
ENSP00000268483	ENSP00000375881
ENSP00000268483	ENSP00000403345
ENSP00000268483	ENSP00000406280
ENSP00000268483	ENSP00000412566
ENSP00000268483	ENSP00000414006
ENSP00000268483	ENSP00000414634
ENSP00000268489	ENSP00000278886
ENSP00000268533	ENSP00000302160
ENSP00000268533	ENSP00000311876
ENSP00000268533	ENSP00000360782
ENSP00000268533	ENSP00000361993
ENSP00000268533	ENSP00000364212
ENSP00000268605	ENSP00000276414
ENSP00000268605	ENSP00000276603

ENSP00000268605	ENSP00000293288
ENSP00000268605	ENSP00000300086
ENSP00000268605	ENSP00000301838
ENSP00000268605	ENSP00000312664
ENSP00000268605	ENSP00000323511
ENSP00000268605	ENSP00000341268
ENSP00000268605	ENSP00000347979
ENSP00000268605	ENSP00000348307
ENSP00000268605	ENSP00000349022
ENSP00000268605	ENSP00000350249
ENSP00000268605	ENSP00000386394
ENSP00000268605	ENSP00000416959
ENSP00000268616	ENSP00000268802
ENSP00000268616	ENSP00000278572
ENSP00000268616	ENSP00000286835
ENSP00000268616	ENSP00000300413
ENSP00000268616	ENSP00000307889
ENSP00000268616	ENSP00000309334
ENSP00000268616	ENSP00000317992
ENSP00000268616	ENSP00000319341
ENSP00000268616	ENSP00000331817
ENSP00000268616	ENSP00000338862
ENSP00000268616	ENSP00000339529
ENSP00000268616	ENSP00000346015
ENSP00000268616	ENSP00000346050
ENSP00000268616	ENSP00000346088
ENSP00000268616	ENSP00000356146
ENSP00000268616	ENSP00000362900
ENSP00000268616	ENSP00000363676
ENSP00000268616	ENSP00000366519
ENSP00000268616	ENSP00000377640
ENSP00000268616	ENSP00000377793
ENSP00000268616	ENSP00000378160
ENSP00000268616	ENSP00000379888
ENSP00000268616	ENSP00000380495
ENSP00000268616	ENSP00000382392
ENSP00000268616	ENSP00000386717
ENSP00000268638	ENSP00000299259
ENSP00000268638	ENSP00000304102
ENSP00000268638	ENSP00000350512
ENSP00000268638	ENSP00000364895
ENSP00000268638	ENSP00000374455
ENSP00000268638	ENSP00000377218
ENSP00000268661	ENSP00000270625
ENSP00000268661	ENSP00000272418
ENSP00000268661	ENSP00000272542

ENSP00000268661	ENSP00000274242
ENSP00000268661	ENSP00000274606
ENSP00000268661	ENSP00000274849
ENSP00000268661	ENSP00000278572
ENSP00000268661	ENSP00000278856
ENSP00000268661	ENSP00000287038
ENSP00000268661	ENSP00000288937
ENSP00000268661	ENSP00000289371
ENSP00000268661	ENSP00000294189
ENSP00000268661	ENSP00000296411
ENSP00000268661	ENSP00000296577
ENSP00000268661	ENSP00000296802
ENSP00000268661	ENSP00000296805
ENSP00000268661	ENSP00000297908
ENSP00000268661	ENSP00000297990
ENSP00000268661	ENSP00000298283
ENSP00000268661	ENSP00000298746
ENSP00000268661	ENSP00000300151
ENSP00000268661	ENSP00000302886
ENSP00000268661	ENSP00000302896
ENSP00000268661	ENSP00000305702
ENSP00000268661	ENSP00000306548
ENSP00000268661	ENSP00000307525
ENSP00000268661	ENSP00000307889
ENSP00000268661	ENSP00000308897
ENSP00000268661	ENSP00000309334
ENSP00000268661	ENSP00000309433
ENSP00000268661	ENSP00000309830
ENSP00000268661	ENSP00000310042
ENSP00000268661	ENSP00000311028
ENSP00000268661	ENSP00000311430
ENSP00000268661	ENSP00000312066
ENSP00000268661	ENSP00000312311
ENSP00000268661	ENSP00000314949
ENSP00000268661	ENSP00000317376
ENSP00000268661	ENSP00000317992
ENSP00000268661	ENSP00000318374
ENSP00000268661	ENSP00000318646
ENSP00000268661	ENSP00000322396
ENSP00000268661	ENSP00000322419
ENSP00000268661	ENSP00000323858
ENSP00000268661	ENSP00000325312
ENSP00000268661	ENSP00000326981
ENSP00000268661	ENSP00000328464
ENSP00000268661	ENSP00000328690
ENSP00000268661	ENSP00000336702

ENSP00000268661	ENSP00000338573
ENSP00000268661	ENSP00000338862
ENSP00000268661	ENSP00000339095
ENSP00000268661	ENSP00000339145
ENSP00000268661	ENSP00000339795
ENSP00000268661	ENSP00000341885
ENSP00000268661	ENSP00000342181
ENSP00000268661	ENSP00000343392
ENSP00000268661	ENSP00000345096
ENSP00000268661	ENSP00000345156
ENSP00000268661	ENSP00000346015
ENSP00000268661	ENSP00000346022
ENSP00000268661	ENSP00000346027
ENSP00000268661	ENSP00000346037
ENSP00000268661	ENSP00000346050
ENSP00000268661	ENSP00000346088
ENSP00000268661	ENSP00000346120
ENSP00000268661	ENSP00000346725
ENSP00000268661	ENSP00000348596
ENSP00000268661	ENSP00000351596
ENSP00000268661	ENSP00000352738
ENSP00000268661	ENSP00000354040
ENSP00000268661	ENSP00000354125
ENSP00000268661	ENSP00000354525
ENSP00000268661	ENSP00000354739
ENSP00000268661	ENSP00000356290
ENSP00000268661	ENSP00000358563
ENSP00000268661	ENSP00000359345
ENSP00000268661	ENSP00000359688
ENSP00000268661	ENSP00000359939
ENSP00000268661	ENSP00000360031
ENSP00000268661	ENSP00000360412
ENSP00000268661	ENSP00000360828
ENSP00000268661	ENSP00000361076
ENSP00000268661	ENSP00000361465
ENSP00000268661	ENSP00000361982
ENSP00000268661	ENSP00000362153
ENSP00000268661	ENSP00000362744
ENSP00000268661	ENSP00000363018
ENSP00000268661	ENSP00000363559
ENSP00000268661	ENSP00000363676
ENSP00000268661	ENSP00000364320
ENSP00000268661	ENSP00000365806
ENSP00000268661	ENSP00000366519
ENSP00000268661	ENSP00000367024
ENSP00000268661	ENSP00000369757

ENSP00000268661	ENSP00000369965
ENSP00000268661	ENSP00000373090
ENSP00000268661	ENSP00000373404
ENSP00000268661	ENSP00000374390
ENSP00000268661	ENSP00000375730
ENSP00000268661	ENSP00000377486
ENSP00000268661	ENSP00000377617
ENSP00000268661	ENSP00000377640
ENSP00000268661	ENSP00000377865
ENSP00000268661	ENSP00000378160
ENSP00000268661	ENSP00000379888
ENSP00000268661	ENSP00000380156
ENSP00000268661	ENSP00000380495
ENSP00000268661	ENSP00000380982
ENSP00000268661	ENSP00000382392
ENSP00000268661	ENSP00000385903
ENSP00000268661	ENSP00000386717
ENSP00000268661	ENSP00000389103
ENSP00000268661	ENSP00000392859
ENSP00000268661	ENSP00000393393
ENSP00000268661	ENSP00000394290
ENSP00000268661	ENSP00000402338
ENSP00000268661	ENSP00000403576
ENSP00000268661	ENSP00000403817
ENSP00000268661	ENSP00000409074
ENSP00000268661	ENSP00000409367
ENSP00000268661	ENSP00000411162
ENSP00000268661	ENSP00000414321
ENSP00000268661	ENSP00000417229
ENSP00000268661	ENSP00000417763
ENSP00000268668	ENSP00000277865
ENSP00000268668	ENSP00000282541
ENSP00000268668	ENSP00000298552
ENSP00000268668	ENSP00000312671
ENSP00000268668	ENSP00000317790
ENSP00000268668	ENSP00000341838
ENSP00000268668	ENSP00000356972
ENSP00000268668	ENSP00000357484
ENSP00000268668	ENSP00000361824
ENSP00000268668	ENSP00000364524
ENSP00000268668	ENSP00000367408
ENSP00000268668	ENSP00000379394
ENSP00000268668	ENSP00000382064
ENSP00000268668	ENSP00000388841
ENSP00000268676	ENSP00000345892
ENSP00000268676	ENSP00000374455

ENSP00000268676	ENSP00000389913
ENSP00000268679	ENSP00000294339
ENSP00000268679	ENSP00000302216
ENSP00000268679	ENSP00000331057
ENSP00000268679	ENSP00000334061
ENSP00000268679	ENSP00000348551
ENSP00000268679	ENSP00000351250
ENSP00000268679	ENSP00000353622
ENSP00000268679	ENSP00000367972
ENSP00000268679	ENSP00000392466
ENSP00000268699	ENSP00000311505
ENSP00000268711	ENSP00000269397
ENSP00000268711	ENSP00000272645
ENSP00000268711	ENSP00000281928
ENSP00000268711	ENSP00000282007
ENSP00000268711	ENSP00000282892
ENSP00000268711	ENSP00000286317
ENSP00000268711	ENSP00000290663
ENSP00000268711	ENSP00000292035
ENSP00000268711	ENSP00000293371
ENSP00000268711	ENSP00000293777
ENSP00000268711	ENSP00000295685
ENSP00000268711	ENSP00000296930
ENSP00000268711	ENSP00000297185
ENSP00000268711	ENSP00000297347
ENSP00000268711	ENSP00000300413
ENSP00000268711	ENSP00000300648
ENSP00000268711	ENSP00000300651
ENSP00000268711	ENSP00000301788
ENSP00000268711	ENSP00000312735
ENSP00000268711	ENSP00000314949
ENSP00000268711	ENSP00000323720
ENSP00000268711	ENSP00000325612
ENSP00000268711	ENSP00000326767
ENSP00000268711	ENSP00000334061
ENSP00000268711	ENSP00000335261
ENSP00000268711	ENSP00000337340
ENSP00000268711	ENSP00000339957
ENSP00000268711	ENSP00000340019
ENSP00000268711	ENSP00000342343
ENSP00000268711	ENSP00000343745
ENSP00000268711	ENSP00000348610
ENSP00000268711	ENSP00000354681
ENSP00000268711	ENSP00000357047
ENSP00000268711	ENSP00000357907
ENSP00000268711	ENSP00000359727

ENSP00000268711	ENSP00000360519
ENSP00000268711	ENSP00000362948
ENSP00000268711	ENSP00000363193
ENSP00000268711	ENSP00000370119
ENSP00000268711	ENSP00000403852
ENSP00000268717	ENSP00000270708
ENSP00000268717	ENSP00000288602
ENSP00000268717	ENSP00000299259
ENSP00000268717	ENSP00000301764
ENSP00000268717	ENSP00000304102
ENSP00000268717	ENSP00000304822
ENSP00000268717	ENSP00000318944
ENSP00000268717	ENSP00000325794
ENSP00000268717	ENSP00000332681
ENSP00000268717	ENSP00000341030
ENSP00000268717	ENSP00000342755
ENSP00000268717	ENSP00000344456
ENSP00000268717	ENSP00000346340
ENSP00000268717	ENSP00000348157
ENSP00000268717	ENSP00000350512
ENSP00000268717	ENSP00000352928
ENSP00000268717	ENSP00000356438
ENSP00000268717	ENSP00000356641
ENSP00000268717	ENSP00000358854
ENSP00000268717	ENSP00000362110
ENSP00000268717	ENSP00000364578
ENSP00000268717	ENSP00000384109
ENSP00000268719	ENSP00000282077
ENSP00000268719	ENSP00000294724
ENSP00000268719	ENSP00000301463
ENSP00000268719	ENSP00000302830
ENSP00000268719	ENSP00000307889
ENSP00000268719	ENSP00000315791
ENSP00000268719	ENSP00000348010
ENSP00000268719	ENSP00000351221
ENSP00000268720	ENSP00000274030
ENSP00000268720	ENSP00000348455
ENSP00000268802	ENSP00000269499
ENSP00000268802	ENSP00000283109
ENSP00000268802	ENSP00000285873
ENSP00000268802	ENSP00000296509
ENSP00000268802	ENSP00000296802
ENSP00000268802	ENSP00000298852
ENSP00000268802	ENSP00000301364
ENSP00000268802	ENSP00000302896
ENSP00000268802	ENSP00000305702

ENSP00000268802	ENSP00000310129
ENSP00000268802	ENSP00000311135
ENSP00000268802	ENSP00000314348
ENSP00000268802	ENSP00000318646
ENSP00000268802	ENSP00000326981
ENSP00000268802	ENSP00000327179
ENSP00000268802	ENSP00000337839
ENSP00000268802	ENSP00000339095
ENSP00000268802	ENSP00000342381
ENSP00000268802	ENSP00000346120
ENSP00000268802	ENSP00000347087
ENSP00000268802	ENSP00000348708
ENSP00000268802	ENSP00000355471
ENSP00000268802	ENSP00000356134
ENSP00000268802	ENSP00000356548
ENSP00000268802	ENSP00000358812
ENSP00000268802	ENSP00000360031
ENSP00000268802	ENSP00000362334
ENSP00000268802	ENSP00000363308
ENSP00000268802	ENSP00000366557
ENSP00000268802	ENSP00000370222
ENSP00000268802	ENSP00000374390
ENSP00000268802	ENSP00000378529
ENSP00000268802	ENSP00000379888
ENSP00000268802	ENSP00000386212
ENSP00000268802	ENSP00000392601
ENSP00000268802	ENSP00000393241
ENSP00000268802	ENSP00000403175
ENSP00000268802	ENSP00000416110
ENSP00000268835	ENSP00000329715
ENSP00000268835	ENSP00000348442
ENSP00000268835	ENSP00000350512
ENSP00000268957	ENSP00000273064
ENSP00000268957	ENSP00000274255
ENSP00000268957	ENSP00000288840
ENSP00000268957	ENSP00000313829
ENSP00000268957	ENSP00000320949
ENSP00000268957	ENSP00000330060
ENSP00000268957	ENSP00000334061
ENSP00000268957	ENSP00000341551
ENSP00000268957	ENSP00000344456
ENSP00000268957	ENSP00000350990
ENSP00000268957	ENSP00000365439
ENSP00000268957	ENSP00000401371
ENSP00000268981	ENSP00000301021
ENSP00000269025	ENSP00000365005

ENSP00000269033	ENSP00000309629
ENSP00000269033	ENSP00000364320
ENSP00000269053	ENSP00000378326
ENSP00000269122	ENSP00000272322
ENSP00000269122	ENSP00000273398
ENSP00000269122	ENSP00000275015
ENSP00000269122	ENSP00000282050
ENSP00000269122	ENSP00000283195
ENSP00000269122	ENSP00000290158
ENSP00000269122	ENSP00000292385
ENSP00000269122	ENSP00000295025
ENSP00000269122	ENSP00000299138
ENSP00000269122	ENSP00000299259
ENSP00000269122	ENSP00000299767
ENSP00000269122	ENSP00000301843
ENSP00000269122	ENSP00000303507
ENSP00000269122	ENSP00000304592
ENSP00000269122	ENSP00000307567
ENSP00000269122	ENSP00000309610
ENSP00000269122	ENSP00000312411
ENSP00000269122	ENSP00000313059
ENSP00000269122	ENSP00000314129
ENSP00000269122	ENSP00000314499
ENSP00000269122	ENSP00000317790
ENSP00000269122	ENSP00000319977
ENSP00000269122	ENSP00000320543
ENSP00000269122	ENSP00000323377
ENSP00000269122	ENSP00000325369
ENSP00000269122	ENSP00000327077
ENSP00000269122	ENSP00000329466
ENSP00000269122	ENSP00000329715
ENSP00000269122	ENSP00000331201
ENSP00000269122	ENSP00000333018
ENSP00000269122	ENSP00000337478
ENSP00000269122	ENSP00000338345
ENSP00000269122	ENSP00000347184
ENSP00000269122	ENSP00000347314
ENSP00000269122	ENSP00000348128
ENSP00000269122	ENSP00000348602
ENSP00000269122	ENSP00000349131
ENSP00000269122	ENSP00000350199
ENSP00000269122	ENSP00000352257
ENSP00000269122	ENSP00000352603
ENSP00000269122	ENSP00000354061
ENSP00000269122	ENSP00000354519
ENSP00000269122	ENSP00000354681

ENSP00000269122	ENSP00000355629
ENSP00000269122	ENSP00000359067
ENSP00000269122	ENSP00000359484
ENSP00000269122	ENSP00000360183
ENSP00000269122	ENSP00000360798
ENSP00000269122	ENSP00000360992
ENSP00000269122	ENSP00000361824
ENSP00000269122	ENSP00000362105
ENSP00000269122	ENSP00000362399
ENSP00000269122	ENSP00000362727
ENSP00000269122	ENSP00000363349
ENSP00000269122	ENSP00000365233
ENSP00000269122	ENSP00000365891
ENSP00000269122	ENSP00000365943
ENSP00000269122	ENSP00000366746
ENSP00000269122	ENSP00000367841
ENSP00000269122	ENSP00000368818
ENSP00000269122	ENSP00000370719
ENSP00000269122	ENSP00000371341
ENSP00000269122	ENSP00000377148
ENSP00000269122	ENSP00000377527
ENSP00000269122	ENSP00000379607
ENSP00000269122	ENSP00000380921
ENSP00000269122	ENSP00000381891
ENSP00000269122	ENSP00000381932
ENSP00000269122	ENSP00000386156
ENSP00000269122	ENSP00000388340
ENSP00000269122	ENSP00000390783
ENSP00000269122	ENSP00000392617
ENSP00000269122	ENSP00000394869
ENSP00000269122	ENSP00000402240
ENSP00000269122	ENSP00000404247
ENSP00000269122	ENSP00000407487
ENSP00000269122	ENSP00000419126
ENSP00000269127	ENSP00000299424
ENSP00000269127	ENSP00000299601
ENSP00000269127	ENSP00000307684
ENSP00000269127	ENSP00000322180
ENSP00000269127	ENSP00000329933
ENSP00000269127	ENSP00000336842
ENSP00000269127	ENSP00000343190
ENSP00000269127	ENSP00000343741
ENSP00000269127	ENSP00000347733
ENSP00000269127	ENSP00000360515
ENSP00000269127	ENSP00000364289
ENSP00000269127	ENSP00000372023

ENSP00000269127	ENSP00000374280
ENSP00000269127	ENSP00000377262
ENSP00000269127	ENSP00000386759
ENSP00000269143	ENSP00000274680
ENSP00000269143	ENSP00000282050
ENSP00000269143	ENSP00000287295
ENSP00000269143	ENSP00000287936
ENSP00000269143	ENSP00000295890
ENSP00000269143	ENSP00000298569
ENSP00000269143	ENSP00000298852
ENSP00000269143	ENSP00000300145
ENSP00000269143	ENSP00000301585
ENSP00000269143	ENSP00000317780
ENSP00000269143	ENSP00000320309
ENSP00000269143	ENSP00000325448
ENSP00000269143	ENSP00000337127
ENSP00000269143	ENSP00000339435
ENSP00000269143	ENSP00000348554
ENSP00000269143	ENSP00000348577
ENSP00000269143	ENSP00000348886
ENSP00000269143	ENSP00000349142
ENSP00000269143	ENSP00000353826
ENSP00000269143	ENSP00000354554
ENSP00000269143	ENSP00000355231
ENSP00000269143	ENSP00000356329
ENSP00000269143	ENSP00000360270
ENSP00000269143	ENSP00000361845
ENSP00000269143	ENSP00000362576
ENSP00000269143	ENSP00000365766
ENSP00000269143	ENSP00000367498
ENSP00000269143	ENSP00000381891
ENSP00000269143	ENSP00000387262
ENSP00000269159	ENSP00000312870
ENSP00000269159	ENSP00000322570
ENSP00000269159	ENSP00000332513
ENSP00000269159	ENSP00000337838
ENSP00000269159	ENSP00000347948
ENSP00000269197	ENSP00000269385
ENSP00000269197	ENSP00000269397
ENSP00000269197	ENSP00000274031
ENSP00000269197	ENSP00000308750
ENSP00000269197	ENSP00000314311
ENSP00000269197	ENSP00000323074
ENSP00000269197	ENSP00000329097
ENSP00000269197	ENSP00000352463
ENSP00000269197	ENSP00000354526

ENSP00000269197	ENSP00000367570
ENSP00000269197	ENSP00000376204
ENSP00000269197	ENSP00000384490
ENSP00000269197	ENSP00000404658
ENSP00000269197	ENSP00000417132
ENSP00000269214	ENSP00000276014
ENSP00000269214	ENSP00000282516
ENSP00000269214	ENSP00000282572
ENSP00000269214	ENSP00000296273
ENSP00000269214	ENSP00000296509
ENSP00000269214	ENSP00000297338
ENSP00000269214	ENSP00000308699
ENSP00000269214	ENSP00000308944
ENSP00000269214	ENSP00000313171
ENSP00000269214	ENSP00000313851
ENSP00000269214	ENSP00000322570
ENSP00000269214	ENSP00000323439
ENSP00000269214	ENSP00000325017
ENSP00000269214	ENSP00000341382
ENSP00000269214	ENSP00000349049
ENSP00000269214	ENSP00000351697
ENSP00000269214	ENSP00000353332
ENSP00000269214	ENSP00000354720
ENSP00000269214	ENSP00000354957
ENSP00000269214	ENSP00000357965
ENSP00000269214	ENSP00000361310
ENSP00000269214	ENSP00000365877
ENSP00000269214	ENSP00000368349
ENSP00000269214	ENSP00000368438
ENSP00000269214	ENSP00000369411
ENSP00000269214	ENSP00000370151
ENSP00000269214	ENSP00000372689
ENSP00000269216	ENSP00000269385
ENSP00000269216	ENSP00000281092
ENSP00000269216	ENSP00000282728
ENSP00000269216	ENSP00000295079
ENSP00000269216	ENSP00000300146
ENSP00000269216	ENSP00000300651
ENSP00000269216	ENSP00000304689
ENSP00000269216	ENSP00000305958
ENSP00000269216	ENSP00000307023
ENSP00000269216	ENSP00000307208
ENSP00000269216	ENSP00000307900
ENSP00000269216	ENSP00000315859
ENSP00000269216	ENSP00000329357
ENSP00000269216	ENSP00000330836

ENSP00000269216	ENSP00000332353
ENSP00000269216	ENSP00000341551
ENSP00000269216	ENSP00000344401
ENSP00000269216	ENSP00000345096
ENSP00000269216	ENSP00000346879
ENSP00000269216	ENSP00000349213
ENSP00000269216	ENSP00000349393
ENSP00000269216	ENSP00000351015
ENSP00000269216	ENSP00000357113
ENSP00000269216	ENSP00000357348
ENSP00000269216	ENSP00000359691
ENSP00000269216	ENSP00000366843
ENSP00000269216	ENSP00000373272
ENSP00000269216	ENSP00000380054
ENSP00000269216	ENSP00000380414
ENSP00000269216	ENSP00000391723
ENSP00000269216	ENSP00000392466
ENSP00000269216	ENSP00000396620
ENSP00000269216	ENSP00000399968
ENSP00000269221	ENSP00000296137
ENSP00000269280	ENSP00000300589
ENSP00000269280	ENSP00000312664
ENSP00000269280	ENSP00000329623
ENSP00000269280	ENSP00000353059
ENSP00000269280	ENSP00000354159
ENSP00000269280	ENSP00000376849
ENSP00000269346	ENSP00000345530
ENSP00000269385	ENSP00000269397
ENSP00000269385	ENSP00000272286
ENSP00000269385	ENSP00000278412
ENSP00000269385	ENSP00000281928
ENSP00000269385	ENSP00000288985
ENSP00000269385	ENSP00000296930
ENSP00000269385	ENSP00000297689
ENSP00000269385	ENSP00000301364
ENSP00000269385	ENSP00000306999
ENSP00000269385	ENSP00000314311
ENSP00000269385	ENSP00000318195
ENSP00000269385	ENSP00000320340
ENSP00000269385	ENSP00000320485
ENSP00000269385	ENSP00000320917
ENSP00000269385	ENSP00000323687
ENSP00000269385	ENSP00000331817
ENSP00000269385	ENSP00000334474
ENSP00000269385	ENSP00000336856
ENSP00000269385	ENSP00000336868

ENSP00000269385	ENSP00000343535
ENSP00000269385	ENSP00000350848
ENSP00000269385	ENSP00000351250
ENSP00000269385	ENSP00000354040
ENSP00000269385	ENSP00000354478
ENSP00000269385	ENSP00000354730
ENSP00000269385	ENSP00000354850
ENSP00000269385	ENSP00000354995
ENSP00000269385	ENSP00000355011
ENSP00000269385	ENSP00000355205
ENSP00000269385	ENSP00000356548
ENSP00000269385	ENSP00000357965
ENSP00000269385	ENSP00000358812
ENSP00000269385	ENSP00000358862
ENSP00000269385	ENSP00000359321
ENSP00000269385	ENSP00000360922
ENSP00000269385	ENSP00000361287
ENSP00000269385	ENSP00000363193
ENSP00000269385	ENSP00000363787
ENSP00000269385	ENSP00000364839
ENSP00000269385	ENSP00000364895
ENSP00000269385	ENSP00000366271
ENSP00000269385	ENSP00000367787
ENSP00000269385	ENSP00000369695
ENSP00000269385	ENSP00000371634
ENSP00000269385	ENSP00000374213
ENSP00000269385	ENSP00000376204
ENSP00000269385	ENSP00000376534
ENSP00000269385	ENSP00000380982
ENSP00000269385	ENSP00000382392
ENSP00000269385	ENSP00000386759
ENSP00000269385	ENSP00000396439
ENSP00000269385	ENSP00000404121
ENSP00000269385	ENSP00000418734
ENSP00000269389	ENSP00000312027
ENSP00000269397	ENSP00000269468
ENSP00000269397	ENSP00000281092
ENSP00000269397	ENSP00000302501
ENSP00000269397	ENSP00000308750
ENSP00000269397	ENSP00000311825
ENSP00000269397	ENSP00000338345
ENSP00000269397	ENSP00000341024
ENSP00000269397	ENSP00000343535
ENSP00000269397	ENSP00000348089
ENSP00000269397	ENSP00000350297
ENSP00000269397	ENSP00000353793

ENSP00000269397	ENSP00000354720
ENSP00000269397	ENSP00000355922
ENSP00000269397	ENSP00000357643
ENSP00000269397	ENSP00000358862
ENSP00000269397	ENSP00000359321
ENSP00000269397	ENSP00000360621
ENSP00000269397	ENSP00000360992
ENSP00000269397	ENSP00000361287
ENSP00000269397	ENSP00000363787
ENSP00000269397	ENSP00000365877
ENSP00000269397	ENSP00000368349
ENSP00000269397	ENSP00000368438
ENSP00000269397	ENSP00000373566
ENSP00000269397	ENSP00000384490
ENSP00000269397	ENSP00000392028
ENSP00000269397	ENSP00000396439
ENSP00000269397	ENSP00000402515
ENSP00000269397	ENSP00000417132
ENSP00000269397	ENSP00000418734
ENSP00000269466	ENSP00000280701
ENSP00000269466	ENSP00000287675
ENSP00000269466	ENSP00000290429
ENSP00000269466	ENSP00000310042
ENSP00000269466	ENSP00000320488
ENSP00000269466	ENSP00000362171
ENSP00000269466	ENSP00000364320
ENSP00000269468	ENSP00000295066
ENSP00000269468	ENSP00000299601
ENSP00000269468	ENSP00000301280
ENSP00000269468	ENSP00000304593
ENSP00000269468	ENSP00000308466
ENSP00000269468	ENSP00000348799
ENSP00000269468	ENSP00000354848
ENSP00000269468	ENSP00000356405
ENSP00000269468	ENSP00000357887
ENSP00000269468	ENSP00000357965
ENSP00000269468	ENSP00000365877
ENSP00000269468	ENSP00000368438
ENSP00000269468	ENSP00000374213
ENSP00000269468	ENSP00000374280
ENSP00000269468	ENSP00000386759
ENSP00000269468	ENSP00000396749
ENSP00000269468	ENSP00000396976
ENSP00000269485	ENSP00000303148
ENSP00000269485	ENSP00000341243
ENSP00000269485	ENSP00000358335

ENSP00000269499	ENSP00000278572
ENSP00000269499	ENSP00000286835
ENSP00000269499	ENSP00000300413
ENSP00000269499	ENSP00000302478
ENSP00000269499	ENSP00000307889
ENSP00000269499	ENSP00000309334
ENSP00000269499	ENSP00000317992
ENSP00000269499	ENSP00000319341
ENSP00000269499	ENSP00000331817
ENSP00000269499	ENSP00000338862
ENSP00000269499	ENSP00000339529
ENSP00000269499	ENSP00000346015
ENSP00000269499	ENSP00000346050
ENSP00000269499	ENSP00000346088
ENSP00000269499	ENSP00000356146
ENSP00000269499	ENSP00000362900
ENSP00000269499	ENSP00000363676
ENSP00000269499	ENSP00000366519
ENSP00000269499	ENSP00000377640
ENSP00000269499	ENSP00000377793
ENSP00000269499	ENSP00000378160
ENSP00000269499	ENSP00000379888
ENSP00000269499	ENSP00000380495
ENSP00000269499	ENSP00000382392
ENSP00000269499	ENSP00000386717
ENSP00000269582	ENSP00000276603
ENSP00000269582	ENSP00000300086
ENSP00000269701	ENSP00000359100
ENSP00000269701	ENSP00000360532
ENSP00000269703	ENSP00000277010
ENSP00000269703	ENSP00000341662
ENSP00000269703	ENSP00000348762
ENSP00000269856	ENSP00000281708
ENSP00000269856	ENSP00000312411
ENSP00000269856	ENSP00000361707
ENSP00000269856	ENSP00000367459
ENSP00000269932	ENSP00000270708
ENSP00000269932	ENSP00000295709
ENSP00000269932	ENSP00000295901
ENSP00000269967	ENSP00000281708
ENSP00000269967	ENSP00000301764
ENSP00000269967	ENSP00000305790
ENSP00000269967	ENSP00000335321
ENSP00000269967	ENSP00000356541
ENSP00000269967	ENSP00000365439
ENSP00000269980	ENSP00000302830

ENSP00000269980	ENSP00000315693
ENSP00000269980	ENSP00000318351
ENSP00000269980	ENSP00000342056
ENSP00000269980	ENSP00000346453
ENSP00000269980	ENSP00000356405
ENSP00000269980	ENSP00000367637
ENSP00000269980	ENSP00000370377
ENSP00000270066	ENSP00000300917
ENSP00000270066	ENSP00000402515
ENSP00000270112	ENSP00000307272
ENSP00000270112	ENSP00000309629
ENSP00000270112	ENSP00000336740
ENSP00000270139	ENSP00000295408
ENSP00000270139	ENSP00000315768
ENSP00000270139	ENSP00000340944
ENSP00000270139	ENSP00000343957
ENSP00000270139	ENSP00000356346
ENSP00000270139	ENSP00000359206
ENSP00000270176	ENSP00000290231
ENSP00000270176	ENSP00000290949
ENSP00000270176	ENSP00000302790
ENSP00000270176	ENSP00000316032
ENSP00000270176	ENSP00000320488
ENSP00000270176	ENSP00000327821
ENSP00000270176	ENSP00000328079
ENSP00000270176	ENSP00000329419
ENSP00000270176	ENSP00000332247
ENSP00000270176	ENSP00000348577
ENSP00000270176	ENSP00000356737
ENSP00000270176	ENSP00000357048
ENSP00000270176	ENSP00000362171
ENSP00000270176	ENSP00000362576
ENSP00000270176	ENSP00000371155
ENSP00000270176	ENSP00000373770
ENSP00000270223	ENSP00000294008
ENSP00000270223	ENSP00000295897
ENSP00000270223	ENSP00000296028
ENSP00000270223	ENSP00000307491
ENSP00000270223	ENSP00000311028
ENSP00000270223	ENSP00000347988
ENSP00000270223	ENSP00000349486
ENSP00000270223	ENSP00000355599
ENSP00000270223	ENSP00000356972
ENSP00000270223	ENSP00000366156
ENSP00000270223	ENSP00000370517
ENSP00000270223	ENSP00000381863

ENSP00000270223	ENSP00000401121
ENSP00000270223	ENSP00000403359
ENSP00000270225	ENSP00000283195
ENSP00000270225	ENSP00000286428
ENSP00000270225	ENSP00000288532
ENSP00000270225	ENSP00000300648
ENSP00000270225	ENSP00000300738
ENSP00000270225	ENSP00000301788
ENSP00000270225	ENSP00000308944
ENSP00000270225	ENSP00000322242
ENSP00000270225	ENSP00000330343
ENSP00000270225	ENSP00000334474
ENSP00000270225	ENSP00000341189
ENSP00000270225	ENSP00000342381
ENSP00000270225	ENSP00000343023
ENSP00000270225	ENSP00000346566
ENSP00000270225	ENSP00000347345
ENSP00000270225	ENSP00000347978
ENSP00000270225	ENSP00000353622
ENSP00000270225	ENSP00000354742
ENSP00000270225	ENSP00000358478
ENSP00000270225	ENSP00000359939
ENSP00000270225	ENSP00000365773
ENSP00000270225	ENSP00000368332
ENSP00000270225	ENSP00000369038
ENSP00000270225	ENSP00000402084
ENSP00000270257	ENSP00000281950
ENSP00000270257	ENSP00000302160
ENSP00000270257	ENSP00000342374
ENSP00000270257	ENSP00000346037
ENSP00000270257	ENSP00000370083
ENSP00000270257	ENSP00000370119
ENSP00000270257	ENSP00000373363
ENSP00000270257	ENSP00000392270
ENSP00000270257	ENSP00000400591
ENSP00000270257	ENSP00000412566
ENSP00000270458	ENSP00000310003
ENSP00000270538	ENSP00000282050
ENSP00000270538	ENSP00000288602
ENSP00000270538	ENSP00000294008
ENSP00000270538	ENSP00000297185
ENSP00000270538	ENSP00000311430
ENSP00000270538	ENSP00000315693
ENSP00000270538	ENSP00000316240
ENSP00000270538	ENSP00000317159
ENSP00000270538	ENSP00000318115

ENSP00000270538	ENSP00000329558
ENSP00000270538	ENSP00000331479
ENSP00000270538	ENSP00000340019
ENSP00000270538	ENSP00000348886
ENSP00000270538	ENSP00000358695
ENSP00000270538	ENSP00000360782
ENSP00000270538	ENSP00000362041
ENSP00000270538	ENSP00000366179
ENSP00000270538	ENSP00000368066
ENSP00000270538	ENSP00000368523
ENSP00000270538	ENSP00000371451
ENSP00000270538	ENSP00000377640
ENSP00000270538	ENSP00000389934
ENSP00000270583	ENSP00000276603
ENSP00000270583	ENSP00000318195
ENSP00000270583	ENSP00000361949
ENSP00000270583	ENSP00000362900
ENSP00000270625	ENSP00000272418
ENSP00000270625	ENSP00000274311
ENSP00000270625	ENSP00000274606
ENSP00000270625	ENSP00000274849
ENSP00000270625	ENSP00000278572
ENSP00000270625	ENSP00000280190
ENSP00000270625	ENSP00000280557
ENSP00000270625	ENSP00000283109
ENSP00000270625	ENSP00000287038
ENSP00000270625	ENSP00000295930
ENSP00000270625	ENSP00000296674
ENSP00000270625	ENSP00000296792
ENSP00000270625	ENSP00000296802
ENSP00000270625	ENSP00000296805
ENSP00000270625	ENSP00000297908
ENSP00000270625	ENSP00000297990
ENSP00000270625	ENSP00000298283
ENSP00000270625	ENSP00000300151
ENSP00000270625	ENSP00000301364
ENSP00000270625	ENSP00000301585
ENSP00000270625	ENSP00000302896
ENSP00000270625	ENSP00000305702
ENSP00000270625	ENSP00000306548
ENSP00000270625	ENSP00000307889
ENSP00000270625	ENSP00000308897
ENSP00000270625	ENSP00000309334
ENSP00000270625	ENSP00000309433
ENSP00000270625	ENSP00000311028
ENSP00000270625	ENSP00000311430

ENSP00000270625	ENSP00000314348
ENSP00000270625	ENSP00000315674
ENSP00000270625	ENSP00000317376
ENSP00000270625	ENSP00000318646
ENSP00000270625	ENSP00000320917
ENSP00000270625	ENSP00000323856
ENSP00000270625	ENSP00000325376
ENSP00000270625	ENSP00000326981
ENSP00000270625	ENSP00000331019
ENSP00000270625	ENSP00000336702
ENSP00000270625	ENSP00000337127
ENSP00000270625	ENSP00000337839
ENSP00000270625	ENSP00000339095
ENSP00000270625	ENSP00000339795
ENSP00000270625	ENSP00000341885
ENSP00000270625	ENSP00000342300
ENSP00000270625	ENSP00000345096
ENSP00000270625	ENSP00000345156
ENSP00000270625	ENSP00000346050
ENSP00000270625	ENSP00000346067
ENSP00000270625	ENSP00000347087
ENSP00000270625	ENSP00000354125
ENSP00000270625	ENSP00000354525
ENSP00000270625	ENSP00000354739
ENSP00000270625	ENSP00000355471
ENSP00000270625	ENSP00000355866
ENSP00000270625	ENSP00000356134
ENSP00000270625	ENSP00000358140
ENSP00000270625	ENSP00000358812
ENSP00000270625	ENSP00000359345
ENSP00000270625	ENSP00000360031
ENSP00000270625	ENSP00000361076
ENSP00000270625	ENSP00000361465
ENSP00000270625	ENSP00000362036
ENSP00000270625	ENSP00000362688
ENSP00000270625	ENSP00000362744
ENSP00000270625	ENSP00000363018
ENSP00000270625	ENSP00000363308
ENSP00000270625	ENSP00000363642
ENSP00000270625	ENSP00000363676
ENSP00000270625	ENSP00000364320
ENSP00000270625	ENSP00000366557
ENSP00000270625	ENSP00000368565
ENSP00000270625	ENSP00000368790
ENSP00000270625	ENSP00000369757
ENSP00000270625	ENSP00000370258

ENSP00000270625	ENSP00000371101
ENSP00000270625	ENSP00000372445
ENSP00000270625	ENSP00000373404
ENSP00000270625	ENSP00000375730
ENSP00000270625	ENSP00000379339
ENSP00000270625	ENSP00000379607
ENSP00000270625	ENSP00000379888
ENSP00000270625	ENSP00000389103
ENSP00000270625	ENSP00000392859
ENSP00000270625	ENSP00000393241
ENSP00000270625	ENSP00000402338
ENSP00000270625	ENSP00000403175
ENSP00000270625	ENSP00000412583
ENSP00000270625	ENSP00000414321
ENSP00000270625	ENSP00000416110
ENSP00000270625	ENSP00000417229
ENSP00000270708	ENSP00000282003
ENSP00000270708	ENSP00000299259
ENSP00000270708	ENSP00000304822
ENSP00000270708	ENSP00000305459
ENSP00000270708	ENSP00000305958
ENSP00000270708	ENSP00000341030
ENSP00000270708	ENSP00000356290
ENSP00000270708	ENSP00000359484
ENSP00000270708	ENSP00000362824
ENSP00000270708	ENSP00000363734
ENSP00000270708	ENSP00000364578
ENSP00000270708	ENSP00000365131
ENSP00000270708	ENSP00000407487
ENSP00000270708	ENSP00000412319
ENSP00000270776	ENSP00000276603
ENSP00000270776	ENSP00000280605
ENSP00000270776	ENSP00000290332
ENSP00000270776	ENSP00000311505
ENSP00000270776	ENSP00000324792
ENSP00000270776	ENSP00000327070
ENSP00000270776	ENSP00000330343
ENSP00000270776	ENSP00000336927
ENSP00000270776	ENSP00000341243
ENSP00000270776	ENSP00000361990
ENSP00000270776	ENSP00000362463
ENSP00000270776	ENSP00000368438
ENSP00000270776	ENSP00000396068
ENSP00000270776	ENSP00000397009
ENSP00000270776	ENSP00000410833
ENSP00000270800	ENSP00000329384

ENSP00000270861	ENSP00000271555
ENSP00000270861	ENSP00000285873
ENSP00000270861	ENSP00000288976
ENSP00000270861	ENSP00000290158
ENSP00000270861	ENSP00000295951
ENSP00000270861	ENSP00000296509
ENSP00000270861	ENSP00000296733
ENSP00000270861	ENSP00000297338
ENSP00000270861	ENSP00000298139
ENSP00000270861	ENSP00000300584
ENSP00000270861	ENSP00000307272
ENSP00000270861	ENSP00000307853
ENSP00000270861	ENSP00000308699
ENSP00000270861	ENSP00000310649
ENSP00000270861	ENSP00000313034
ENSP00000270861	ENSP00000322608
ENSP00000270861	ENSP00000323663
ENSP00000270861	ENSP00000335357
ENSP00000270861	ENSP00000336868
ENSP00000270861	ENSP00000341551
ENSP00000270861	ENSP00000341848
ENSP00000270861	ENSP00000342510
ENSP00000270861	ENSP00000346566
ENSP00000270861	ENSP00000347232
ENSP00000270861	ENSP00000348554
ENSP00000270861	ENSP00000348573
ENSP00000270861	ENSP00000351284
ENSP00000270861	ENSP00000352572
ENSP00000270861	ENSP00000354720
ENSP00000270861	ENSP00000354916
ENSP00000270861	ENSP00000354927
ENSP00000270861	ENSP00000355922
ENSP00000270861	ENSP00000359206
ENSP00000270861	ENSP00000360076
ENSP00000270861	ENSP00000362578
ENSP00000270861	ENSP00000367638
ENSP00000270861	ENSP00000368017
ENSP00000270861	ENSP00000368349
ENSP00000270861	ENSP00000369716
ENSP00000270861	ENSP00000371308
ENSP00000270861	ENSP00000371451
ENSP00000270861	ENSP00000372689
ENSP00000270861	ENSP00000377655
ENSP00000270861	ENSP00000378350
ENSP00000270861	ENSP00000378529
ENSP00000270861	ENSP00000381932

ENSP00000270861	ENSP00000382271
ENSP00000270861	ENSP00000383145
ENSP00000270861	ENSP00000402527
ENSP00000271002	ENSP00000276201
ENSP00000271002	ENSP00000315397
ENSP00000271002	ENSP00000333633
ENSP00000271002	ENSP00000348128
ENSP00000271002	ENSP00000364737
ENSP00000271002	ENSP00000369862
ENSP00000271373	ENSP00000298910
ENSP00000271373	ENSP00000308610
ENSP00000271373	ENSP00000337396
ENSP00000271373	ENSP00000341213
ENSP00000271373	ENSP00000364649
ENSP00000271373	ENSP00000373600
ENSP00000271373	ENSP00000387262
ENSP00000271375	ENSP00000294179
ENSP00000271375	ENSP00000305459
ENSP00000271375	ENSP00000308496
ENSP00000271375	ENSP00000327716
ENSP00000271375	ENSP00000360183
ENSP00000271375	ENSP00000397441
ENSP00000271375	ENSP00000402240
ENSP00000271385	ENSP00000364320
ENSP00000271452	ENSP00000278412
ENSP00000271452	ENSP00000281513
ENSP00000271452	ENSP00000282074
ENSP00000271452	ENSP00000283006
ENSP00000271452	ENSP00000296509
ENSP00000271452	ENSP00000296702
ENSP00000271452	ENSP00000298048
ENSP00000271452	ENSP00000301332
ENSP00000271452	ENSP00000302530
ENSP00000271452	ENSP00000302640
ENSP00000271452	ENSP00000305941
ENSP00000271452	ENSP00000323439
ENSP00000271452	ENSP00000325677
ENSP00000271452	ENSP00000326514
ENSP00000271452	ENSP00000331831
ENSP00000271452	ENSP00000336856
ENSP00000271452	ENSP00000336868
ENSP00000271452	ENSP00000341382
ENSP00000271452	ENSP00000341680
ENSP00000271452	ENSP00000341848
ENSP00000271452	ENSP00000344352
ENSP00000271452	ENSP00000348573

ENSP00000271452	ENSP00000352572
ENSP00000271452	ENSP00000353735
ENSP00000271452	ENSP00000354486
ENSP00000271452	ENSP00000354720
ENSP00000271452	ENSP00000354957
ENSP00000271452	ENSP00000355922
ENSP00000271452	ENSP00000360532
ENSP00000271452	ENSP00000363055
ENSP00000271452	ENSP00000368801
ENSP00000271452	ENSP00000368976
ENSP00000271452	ENSP00000369075
ENSP00000271452	ENSP00000370408
ENSP00000271452	ENSP00000370557
ENSP00000271452	ENSP00000374566
ENSP00000271452	ENSP00000377446
ENSP00000271452	ENSP00000420588
ENSP00000271526	ENSP00000360532
ENSP00000271526	ENSP00000361162
ENSP00000271526	ENSP00000366829
ENSP00000271526	ENSP00000414634
ENSP00000271555	ENSP00000274289
ENSP00000271555	ENSP00000287598
ENSP00000271555	ENSP00000288976
ENSP00000271555	ENSP00000290158
ENSP00000271555	ENSP00000294168
ENSP00000271555	ENSP00000299424
ENSP00000271555	ENSP00000302530
ENSP00000271555	ENSP00000311005
ENSP00000271555	ENSP00000319169
ENSP00000271555	ENSP00000325690
ENSP00000271555	ENSP00000328720
ENSP00000271555	ENSP00000329357
ENSP00000271555	ENSP00000331057
ENSP00000271555	ENSP00000335357
ENSP00000271555	ENSP00000337088
ENSP00000271555	ENSP00000338345
ENSP00000271555	ENSP00000340896
ENSP00000271555	ENSP00000344537
ENSP00000271555	ENSP00000349049
ENSP00000271555	ENSP00000349213
ENSP00000271555	ENSP00000350512
ENSP00000271555	ENSP00000354916
ENSP00000271555	ENSP00000359818
ENSP00000271555	ENSP00000361998
ENSP00000271555	ENSP00000362717
ENSP00000271555	ENSP00000365439

ENSP00000271555	ENSP00000366534
ENSP00000271555	ENSP00000373715
ENSP00000271588	ENSP00000308610
ENSP00000271628	ENSP00000273480
ENSP00000271628	ENSP00000284776
ENSP00000271628	ENSP00000296702
ENSP00000271628	ENSP00000301740
ENSP00000271628	ENSP00000303191
ENSP00000271628	ENSP00000304350
ENSP00000271628	ENSP00000304370
ENSP00000271628	ENSP00000304903
ENSP00000271628	ENSP00000305790
ENSP00000271628	ENSP00000309166
ENSP00000271628	ENSP00000310448
ENSP00000271628	ENSP00000313674
ENSP00000271628	ENSP00000318861
ENSP00000271628	ENSP00000319169
ENSP00000271628	ENSP00000326261
ENSP00000271628	ENSP00000326806
ENSP00000271628	ENSP00000332103
ENSP00000271628	ENSP00000334148
ENSP00000271628	ENSP00000335321
ENSP00000271628	ENSP00000339090
ENSP00000271628	ENSP00000349594
ENSP00000271628	ENSP00000352308
ENSP00000271628	ENSP00000355136
ENSP00000271628	ENSP00000355261
ENSP00000271628	ENSP00000356263
ENSP00000271628	ENSP00000358554
ENSP00000271628	ENSP00000358605
ENSP00000271628	ENSP00000360532
ENSP00000271628	ENSP00000361577
ENSP00000271628	ENSP00000362110
ENSP00000271628	ENSP00000364482
ENSP00000271628	ENSP00000366607
ENSP00000271628	ENSP00000366829
ENSP00000271628	ENSP00000368801
ENSP00000271628	ENSP00000370719
ENSP00000271628	ENSP00000375863
ENSP00000271628	ENSP00000379607
ENSP00000271628	ENSP00000381031
ENSP00000271628	ENSP00000391798
ENSP00000271628	ENSP00000404121
ENSP00000271628	ENSP00000412566
ENSP00000271628	ENSP00000419235
ENSP00000271643	ENSP00000273075

ENSP00000271643	ENSP00000280527
ENSP00000271643	ENSP00000309052
ENSP00000271643	ENSP00000330836
ENSP00000271643	ENSP00000333537
ENSP00000271643	ENSP00000348307
ENSP00000271643	ENSP00000356991
ENSP00000271715	ENSP00000295951
ENSP00000271715	ENSP00000298317
ENSP00000271715	ENSP00000322915
ENSP00000271715	ENSP00000323288
ENSP00000271715	ENSP00000324020
ENSP00000271715	ENSP00000329357
ENSP00000271715	ENSP00000329466
ENSP00000271715	ENSP00000336587
ENSP00000271715	ENSP00000341848
ENSP00000271715	ENSP00000350094
ENSP00000271715	ENSP00000354158
ENSP00000271715	ENSP00000354486
ENSP00000271715	ENSP00000355922
ENSP00000271715	ENSP00000360916
ENSP00000271715	ENSP00000373331
ENSP00000271715	ENSP00000378295
ENSP00000271715	ENSP00000396620
ENSP00000271764	ENSP00000274242
ENSP00000271764	ENSP00000278572
ENSP00000271764	ENSP00000280557
ENSP00000271764	ENSP00000283875
ENSP00000271764	ENSP00000296802
ENSP00000271764	ENSP00000301364
ENSP00000271764	ENSP00000347087
ENSP00000271764	ENSP00000360532
ENSP00000271764	ENSP00000362744
ENSP00000271764	ENSP00000367615
ENSP00000271843	ENSP00000284811
ENSP00000271843	ENSP00000350708
ENSP00000271843	ENSP00000367787
ENSP00000271843	ENSP00000374359
ENSP00000271883	ENSP00000353622
ENSP00000272065	ENSP00000272227
ENSP00000272065	ENSP00000344456
ENSP00000272065	ENSP00000349823
ENSP00000272065	ENSP00000358307
ENSP00000272065	ENSP00000361824
ENSP00000272065	ENSP00000363559
ENSP00000272065	ENSP00000363763
ENSP00000272065	ENSP00000377486

ENSP00000272065	ENSP00000379607
ENSP00000272065	ENSP00000399511
ENSP00000272091	ENSP00000294008
ENSP00000272091	ENSP00000302562
ENSP00000272091	ENSP00000304370
ENSP00000272091	ENSP00000308546
ENSP00000272091	ENSP00000334148
ENSP00000272117	ENSP00000293362
ENSP00000272134	ENSP00000296145
ENSP00000272134	ENSP00000297261
ENSP00000272139	ENSP00000276603
ENSP00000272139	ENSP00000304370
ENSP00000272139	ENSP00000315859
ENSP00000272139	ENSP00000334148
ENSP00000272139	ENSP00000379607
ENSP00000272163	ENSP00000277010
ENSP00000272163	ENSP00000285949
ENSP00000272163	ENSP00000310721
ENSP00000272163	ENSP00000320604
ENSP00000272163	ENSP00000322957
ENSP00000272163	ENSP00000324729
ENSP00000272163	ENSP00000325425
ENSP00000272163	ENSP00000330812
ENSP00000272163	ENSP00000333946
ENSP00000272163	ENSP00000341662
ENSP00000272163	ENSP00000348762
ENSP00000272163	ENSP00000355517
ENSP00000272163	ENSP00000358413
ENSP00000272163	ENSP00000360918
ENSP00000272163	ENSP00000379430
ENSP00000272163	ENSP00000395535
ENSP00000272167	ENSP00000299824
ENSP00000272167	ENSP00000348762
ENSP00000272167	ENSP00000349145
ENSP00000272203	ENSP00000286835
ENSP00000272203	ENSP00000315859
ENSP00000272203	ENSP00000325863
ENSP00000272203	ENSP00000341382
ENSP00000272203	ENSP00000348215
ENSP00000272203	ENSP00000352257
ENSP00000272203	ENSP00000356146
ENSP00000272203	ENSP00000358902
ENSP00000272203	ENSP00000380450
ENSP00000272227	ENSP00000278572
ENSP00000272227	ENSP00000280700
ENSP00000272227	ENSP00000282050

ENSP00000272227	ENSP00000286091
ENSP00000272227	ENSP00000286428
ENSP00000272227	ENSP00000292180
ENSP00000272227	ENSP00000296577
ENSP00000272227	ENSP00000299767
ENSP00000272227	ENSP00000310129
ENSP00000272227	ENSP00000312735
ENSP00000272227	ENSP00000318147
ENSP00000272227	ENSP00000321259
ENSP00000272227	ENSP00000326699
ENSP00000272227	ENSP00000330836
ENSP00000272227	ENSP00000331369
ENSP00000272227	ENSP00000341243
ENSP00000272227	ENSP00000347948
ENSP00000272227	ENSP00000357880
ENSP00000272227	ENSP00000360286
ENSP00000272227	ENSP00000368646
ENSP00000272227	ENSP00000379607
ENSP00000272238	ENSP00000304500
ENSP00000272238	ENSP00000314615
ENSP00000272238	ENSP00000316845
ENSP00000272238	ENSP00000332247
ENSP00000272238	ENSP00000333255
ENSP00000272238	ENSP00000333873
ENSP00000272238	ENSP00000343313
ENSP00000272238	ENSP00000352522
ENSP00000272238	ENSP00000363162
ENSP00000272252	ENSP00000280871
ENSP00000272252	ENSP00000294543
ENSP00000272252	ENSP00000315791
ENSP00000272252	ENSP00000319104
ENSP00000272252	ENSP00000321259
ENSP00000272252	ENSP00000348010
ENSP00000272252	ENSP00000351284
ENSP00000272252	ENSP00000362900
ENSP00000272252	ENSP00000364289
ENSP00000272252	ENSP00000368119
ENSP00000272252	ENSP00000379500
ENSP00000272286	ENSP00000278572
ENSP00000272286	ENSP00000315791
ENSP00000272286	ENSP00000336762
ENSP00000272286	ENSP00000355968
ENSP00000272286	ENSP00000359321
ENSP00000272286	ENSP00000362900
ENSP00000272286	ENSP00000364895
ENSP00000272286	ENSP00000376204

ENSP00000272286	ENSP00000379003
ENSP00000272286	ENSP00000417085
ENSP00000272322	ENSP00000272427
ENSP00000272322	ENSP00000273398
ENSP00000272322	ENSP00000286448
ENSP00000272322	ENSP00000305255
ENSP00000272322	ENSP00000308496
ENSP00000272322	ENSP00000326869
ENSP00000272322	ENSP00000331268
ENSP00000272322	ENSP00000341662
ENSP00000272322	ENSP00000343741
ENSP00000272322	ENSP00000353564
ENSP00000272322	ENSP00000354558
ENSP00000272322	ENSP00000360183
ENSP00000272322	ENSP00000369099
ENSP00000272322	ENSP00000369736
ENSP00000272322	ENSP00000372697
ENSP00000272322	ENSP00000376268
ENSP00000272322	ENSP00000389934
ENSP00000272322	ENSP00000390831
ENSP00000272322	ENSP00000391197
ENSP00000272322	ENSP00000401435
ENSP00000272322	ENSP00000404016
ENSP00000272322	ENSP00000406988
ENSP00000272322	ENSP00000409952
ENSP00000272418	ENSP00000274606
ENSP00000272418	ENSP00000274849
ENSP00000272418	ENSP00000278572
ENSP00000272418	ENSP00000280557
ENSP00000272418	ENSP00000295491
ENSP00000272418	ENSP00000296674
ENSP00000272418	ENSP00000296802
ENSP00000272418	ENSP00000296805
ENSP00000272418	ENSP00000297908
ENSP00000272418	ENSP00000297990
ENSP00000272418	ENSP00000301585
ENSP00000272418	ENSP00000301962
ENSP00000272418	ENSP00000302896
ENSP00000272418	ENSP00000305653
ENSP00000272418	ENSP00000308897
ENSP00000272418	ENSP00000310590
ENSP00000272418	ENSP00000311028
ENSP00000272418	ENSP00000311135
ENSP00000272418	ENSP00000312735
ENSP00000272418	ENSP00000314949
ENSP00000272418	ENSP00000317376

ENSP00000272418	ENSP00000318646
ENSP00000272418	ENSP00000321636
ENSP00000272418	ENSP00000323856
ENSP00000272418	ENSP00000324122
ENSP00000272418	ENSP00000326981
ENSP00000272418	ENSP00000331019
ENSP00000272418	ENSP00000336702
ENSP00000272418	ENSP00000337127
ENSP00000272418	ENSP00000337839
ENSP00000272418	ENSP00000339095
ENSP00000272418	ENSP00000341692
ENSP00000272418	ENSP00000343081
ENSP00000272418	ENSP00000345156
ENSP00000272418	ENSP00000346015
ENSP00000272418	ENSP00000346027
ENSP00000272418	ENSP00000346050
ENSP00000272418	ENSP00000346067
ENSP00000272418	ENSP00000346120
ENSP00000272418	ENSP00000346725
ENSP00000272418	ENSP00000348722
ENSP00000272418	ENSP00000350491
ENSP00000272418	ENSP00000352706
ENSP00000272418	ENSP00000354739
ENSP00000272418	ENSP00000355471
ENSP00000272418	ENSP00000356134
ENSP00000272418	ENSP00000358140
ENSP00000272418	ENSP00000358812
ENSP00000272418	ENSP00000361076
ENSP00000272418	ENSP00000361206
ENSP00000272418	ENSP00000361699
ENSP00000272418	ENSP00000362036
ENSP00000272418	ENSP00000362135
ENSP00000272418	ENSP00000362208
ENSP00000272418	ENSP00000362744
ENSP00000272418	ENSP00000363018
ENSP00000272418	ENSP00000363676
ENSP00000272418	ENSP00000364320
ENSP00000272418	ENSP00000364883
ENSP00000272418	ENSP00000368790
ENSP00000272418	ENSP00000369757
ENSP00000272418	ENSP00000370589
ENSP00000272418	ENSP00000372445
ENSP00000272418	ENSP00000373404
ENSP00000272418	ENSP00000379339
ENSP00000272418	ENSP00000379888
ENSP00000272418	ENSP00000382250

ENSP00000272418	ENSP00000393241
ENSP00000272418	ENSP00000396704
ENSP00000272418	ENSP00000403175
ENSP00000272418	ENSP00000412583
ENSP00000272418	ENSP00000414321
ENSP00000272418	ENSP00000416110
ENSP00000272418	ENSP00000420714
ENSP00000272424	ENSP00000330836
ENSP00000272424	ENSP00000349658
ENSP00000272424	ENSP00000351284
ENSP00000272424	ENSP00000352257
ENSP00000272424	ENSP00000361186
ENSP00000272427	ENSP00000272972
ENSP00000272427	ENSP00000273666
ENSP00000272427	ENSP00000316845
ENSP00000272427	ENSP00000317714
ENSP00000272427	ENSP00000323377
ENSP00000272427	ENSP00000326514
ENSP00000272427	ENSP00000332326
ENSP00000272427	ENSP00000334100
ENSP00000272427	ENSP00000341538
ENSP00000272427	ENSP00000348215
ENSP00000272427	ENSP00000353564
ENSP00000272427	ENSP00000355721
ENSP00000272427	ENSP00000356231
ENSP00000272427	ENSP00000362399
ENSP00000272427	ENSP00000365630
ENSP00000272427	ENSP00000368565
ENSP00000272427	ENSP00000369409
ENSP00000272427	ENSP00000373399
ENSP00000272427	ENSP00000380450
ENSP00000272427	ENSP00000389934
ENSP00000272438	ENSP00000327309
ENSP00000272438	ENSP00000375881
ENSP00000272444	ENSP00000289371
ENSP00000272444	ENSP00000293406
ENSP00000272444	ENSP00000296328
ENSP00000272444	ENSP00000301788
ENSP00000272444	ENSP00000307567
ENSP00000272444	ENSP00000314949
ENSP00000272444	ENSP00000332444
ENSP00000272444	ENSP00000342793
ENSP00000272444	ENSP00000355013
ENSP00000272444	ENSP00000358140
ENSP00000272444	ENSP00000362166
ENSP00000272444	ENSP00000370258

ENSP00000272444	ENSP00000388872
ENSP00000272444	ENSP00000396704
ENSP00000272520	ENSP00000278407
ENSP00000272521	ENSP00000275820
ENSP00000272521	ENSP00000280350
ENSP00000272521	ENSP00000291576
ENSP00000272521	ENSP00000296792
ENSP00000272521	ENSP00000297579
ENSP00000272521	ENSP00000297990
ENSP00000272521	ENSP00000298292
ENSP00000272521	ENSP00000308179
ENSP00000272521	ENSP00000311135
ENSP00000272521	ENSP00000314193
ENSP00000272521	ENSP00000315674
ENSP00000272521	ENSP00000321449
ENSP00000272521	ENSP00000323302
ENSP00000272521	ENSP00000326981
ENSP00000272521	ENSP00000327179
ENSP00000272521	ENSP00000328854
ENSP00000272521	ENSP00000355471
ENSP00000272521	ENSP00000355541
ENSP00000272521	ENSP00000358812
ENSP00000272521	ENSP00000363313
ENSP00000272521	ENSP00000363642
ENSP00000272521	ENSP00000363746
ENSP00000272521	ENSP00000370589
ENSP00000272521	ENSP00000371169
ENSP00000272521	ENSP00000372695
ENSP00000272521	ENSP00000399454
ENSP00000272521	ENSP00000402869
ENSP00000272521	ENSP00000405614
ENSP00000272542	ENSP00000273130
ENSP00000272542	ENSP00000297282
ENSP00000272542	ENSP00000344055
ENSP00000272542	ENSP00000356811
ENSP00000272542	ENSP00000361264
ENSP00000272602	ENSP00000331727
ENSP00000272638	ENSP00000290649
ENSP00000272638	ENSP00000299767
ENSP00000272638	ENSP00000337053
ENSP00000272638	ENSP00000343001
ENSP00000272638	ENSP00000380460
ENSP00000272645	ENSP00000273783
ENSP00000272645	ENSP00000278412
ENSP00000272645	ENSP00000282326
ENSP00000272645	ENSP00000286835

ENSP00000272645	ENSP00000288199
ENSP00000272645	ENSP00000292363
ENSP00000272645	ENSP00000293860
ENSP00000272645	ENSP00000295131
ENSP00000272645	ENSP00000296802
ENSP00000272645	ENSP00000299543
ENSP00000272645	ENSP00000300146
ENSP00000272645	ENSP00000300413
ENSP00000272645	ENSP00000301788
ENSP00000272645	ENSP00000304321
ENSP00000272645	ENSP00000305503
ENSP00000272645	ENSP00000306906
ENSP00000272645	ENSP00000306983
ENSP00000272645	ENSP00000310260
ENSP00000272645	ENSP00000312735
ENSP00000272645	ENSP00000314311
ENSP00000272645	ENSP00000314949
ENSP00000272645	ENSP00000315950
ENSP00000272645	ENSP00000317177
ENSP00000272645	ENSP00000317636
ENSP00000272645	ENSP00000317872
ENSP00000272645	ENSP00000318641
ENSP00000272645	ENSP00000323046
ENSP00000272645	ENSP00000323720
ENSP00000272645	ENSP00000324124
ENSP00000272645	ENSP00000324792
ENSP00000272645	ENSP00000327889
ENSP00000272645	ENSP00000329029
ENSP00000272645	ENSP00000330343
ENSP00000272645	ENSP00000331111
ENSP00000272645	ENSP00000335261
ENSP00000272645	ENSP00000337340
ENSP00000272645	ENSP00000340823
ENSP00000272645	ENSP00000342121
ENSP00000272645	ENSP00000342374
ENSP00000272645	ENSP00000342793
ENSP00000272645	ENSP00000342889
ENSP00000272645	ENSP00000343129
ENSP00000272645	ENSP00000350967
ENSP00000272645	ENSP00000354775
ENSP00000272645	ENSP00000355013
ENSP00000272645	ENSP00000356146
ENSP00000272645	ENSP00000356989
ENSP00000272645	ENSP00000358140
ENSP00000272645	ENSP00000359518
ENSP00000272645	ENSP00000359531

ENSP00000272645	ENSP00000360515
ENSP00000272645	ENSP00000361433
ENSP00000272645	ENSP00000362900
ENSP00000272645	ENSP00000363021
ENSP00000272645	ENSP00000363779
ENSP00000272645	ENSP00000364813
ENSP00000272645	ENSP00000366828
ENSP00000272645	ENSP00000368438
ENSP00000272645	ENSP00000369695
ENSP00000272645	ENSP00000370258
ENSP00000272645	ENSP00000371800
ENSP00000272645	ENSP00000372883
ENSP00000272645	ENSP00000377969
ENSP00000272645	ENSP00000381064
ENSP00000272645	ENSP00000383503
ENSP00000272645	ENSP00000386759
ENSP00000272645	ENSP00000397259
ENSP00000272645	ENSP00000397636
ENSP00000272645	ENSP00000403345
ENSP00000272645	ENSP00000403852
ENSP00000272645	ENSP00000405636
ENSP00000272645	ENSP00000406280
ENSP00000272645	ENSP00000406293
ENSP00000272645	ENSP00000410530
ENSP00000272645	ENSP00000410758
ENSP00000272645	ENSP00000410954
ENSP00000272645	ENSP00000412566
ENSP00000272645	ENSP00000414006
ENSP00000272645	ENSP00000414110
ENSP00000272645	ENSP00000414634
ENSP00000272645	ENSP00000416599
ENSP00000272748	ENSP00000368959
ENSP00000272902	ENSP00000296490
ENSP00000272902	ENSP00000301329
ENSP00000272902	ENSP00000304185
ENSP00000272902	ENSP00000307004
ENSP00000272902	ENSP00000311768
ENSP00000272902	ENSP00000333298
ENSP00000272902	ENSP00000349022
ENSP00000272902	ENSP00000353904
ENSP00000272902	ENSP00000358854
ENSP00000272902	ENSP00000360286
ENSP00000272902	ENSP00000365147
ENSP00000272902	ENSP00000373509
ENSP00000272937	ENSP00000302216
ENSP00000272937	ENSP00000351407

ENSP00000272937	ENSP00000365735
ENSP00000272972	ENSP00000286428
ENSP00000272972	ENSP00000290846
ENSP00000272972	ENSP00000307684
ENSP00000272972	ENSP00000345988
ENSP00000272972	ENSP00000356989
ENSP00000272972	ENSP00000357748
ENSP00000272972	ENSP00000360473
ENSP00000272972	ENSP00000378306
ENSP00000272972	ENSP00000389934
ENSP00000273037	ENSP00000317177
ENSP00000273037	ENSP00000342793
ENSP00000273064	ENSP00000273067
ENSP00000273064	ENSP00000274140
ENSP00000273064	ENSP00000283646
ENSP00000273064	ENSP00000285896
ENSP00000273064	ENSP00000295926
ENSP00000273064	ENSP00000296509
ENSP00000273064	ENSP00000296786
ENSP00000273064	ENSP00000307900
ENSP00000273064	ENSP00000308546
ENSP00000273064	ENSP00000309141
ENSP00000273064	ENSP00000311496
ENSP00000273064	ENSP00000314491
ENSP00000273064	ENSP00000315693
ENSP00000273064	ENSP00000317087
ENSP00000273064	ENSP00000320924
ENSP00000273064	ENSP00000320949
ENSP00000273064	ENSP00000352802
ENSP00000273064	ENSP00000354673
ENSP00000273064	ENSP00000356448
ENSP00000273064	ENSP00000359691
ENSP00000273064	ENSP00000361287
ENSP00000273064	ENSP00000368438
ENSP00000273064	ENSP00000369965
ENSP00000273064	ENSP00000373474
ENSP00000273064	ENSP00000374529
ENSP00000273064	ENSP00000379217
ENSP00000273064	ENSP00000386878
ENSP00000273064	ENSP00000402240
ENSP00000273064	ENSP00000403817
ENSP00000273064	ENSP00000412922
ENSP00000273067	ENSP00000290649
ENSP00000273067	ENSP00000295066
ENSP00000273067	ENSP00000304051
ENSP00000273067	ENSP00000315791

ENSP00000273067	ENSP00000331487
ENSP00000273067	ENSP00000357998
ENSP00000273067	ENSP00000366395
ENSP00000273067	ENSP00000379457
ENSP00000273067	ENSP00000383719
ENSP00000273075	ENSP00000286091
ENSP00000273075	ENSP00000287936
ENSP00000273075	ENSP00000308275
ENSP00000273075	ENSP00000333950
ENSP00000273075	ENSP00000340836
ENSP00000273075	ENSP00000342056
ENSP00000273075	ENSP00000342300
ENSP00000273075	ENSP00000349785
ENSP00000273075	ENSP00000353655
ENSP00000273075	ENSP00000355627
ENSP00000273075	ENSP00000366863
ENSP00000273075	ENSP00000391723
ENSP00000273075	ENSP00000394869
ENSP00000273077	ENSP00000299601
ENSP00000273130	ENSP00000296509
ENSP00000273130	ENSP00000314837
ENSP00000273130	ENSP00000348965
ENSP00000273130	ENSP00000352572
ENSP00000273130	ENSP00000360252
ENSP00000273130	ENSP00000360532
ENSP00000273130	ENSP00000379607
ENSP00000273258	ENSP00000290299
ENSP00000273258	ENSP00000300688
ENSP00000273308	ENSP00000286091
ENSP00000273308	ENSP00000312834
ENSP00000273308	ENSP00000349298
ENSP00000273308	ENSP00000399511
ENSP00000273317	ENSP00000295959
ENSP00000273317	ENSP00000304226
ENSP00000273317	ENSP00000355601
ENSP00000273390	ENSP00000337736
ENSP00000273390	ENSP00000380702
ENSP00000273398	ENSP00000278671
ENSP00000273398	ENSP00000281708
ENSP00000273398	ENSP00000282841
ENSP00000273398	ENSP00000284719
ENSP00000273398	ENSP00000284727
ENSP00000273398	ENSP00000285896
ENSP00000273398	ENSP00000286448
ENSP00000273398	ENSP00000289359
ENSP00000273398	ENSP00000290330

ENSP00000273398	ENSP00000290949
ENSP00000273398	ENSP00000290974
ENSP00000273398	ENSP00000294785
ENSP00000273398	ENSP00000301825
ENSP00000273398	ENSP00000304169
ENSP00000273398	ENSP00000304592
ENSP00000273398	ENSP00000305069
ENSP00000273398	ENSP00000305255
ENSP00000273398	ENSP00000309457
ENSP00000273398	ENSP00000310129
ENSP00000273398	ENSP00000313603
ENSP00000273398	ENSP00000313953
ENSP00000273398	ENSP00000314508
ENSP00000273398	ENSP00000317334
ENSP00000273398	ENSP00000318944
ENSP00000273398	ENSP00000320924
ENSP00000273398	ENSP00000326534
ENSP00000273398	ENSP00000327650
ENSP00000273398	ENSP00000330408
ENSP00000273398	ENSP00000333298
ENSP00000273398	ENSP00000333946
ENSP00000273398	ENSP00000337501
ENSP00000273398	ENSP00000337838
ENSP00000273398	ENSP00000340019
ENSP00000273398	ENSP00000343741
ENSP00000273398	ENSP00000344106
ENSP00000273398	ENSP00000345528
ENSP00000273398	ENSP00000346453
ENSP00000273398	ENSP00000348163
ENSP00000273398	ENSP00000349078
ENSP00000273398	ENSP00000350854
ENSP00000273398	ENSP00000351346
ENSP00000273398	ENSP00000351520
ENSP00000273398	ENSP00000352522
ENSP00000273398	ENSP00000352738
ENSP00000273398	ENSP00000352802
ENSP00000273398	ENSP00000353073
ENSP00000273398	ENSP00000353332
ENSP00000273398	ENSP00000354347
ENSP00000273398	ENSP00000356655
ENSP00000273398	ENSP00000357341
ENSP00000273398	ENSP00000358033
ENSP00000273398	ENSP00000359067
ENSP00000273398	ENSP00000360183
ENSP00000273398	ENSP00000360269
ENSP00000273398	ENSP00000360473

ENSP00000273398	ENSP00000361845
ENSP00000273398	ENSP00000362399
ENSP00000273398	ENSP00000363162
ENSP00000273398	ENSP00000365388
ENSP00000273398	ENSP00000365840
ENSP00000273398	ENSP00000367299
ENSP00000273398	ENSP00000367923
ENSP00000273398	ENSP00000368439
ENSP00000273398	ENSP00000369099
ENSP00000273398	ENSP00000369810
ENSP00000273398	ENSP00000372697
ENSP00000273398	ENSP00000373082
ENSP00000273398	ENSP00000375809
ENSP00000273398	ENSP00000376945
ENSP00000273398	ENSP00000376946
ENSP00000273398	ENSP00000378368
ENSP00000273398	ENSP00000386878
ENSP00000273398	ENSP00000386992
ENSP00000273398	ENSP00000389065
ENSP00000273398	ENSP00000389934
ENSP00000273398	ENSP00000390831
ENSP00000273398	ENSP00000391197
ENSP00000273398	ENSP00000391723
ENSP00000273398	ENSP00000397598
ENSP00000273398	ENSP00000402240
ENSP00000273398	ENSP00000403397
ENSP00000273398	ENSP00000404016
ENSP00000273398	ENSP00000406988
ENSP00000273398	ENSP00000407487
ENSP00000273398	ENSP00000409952
ENSP00000273398	ENSP00000411327
ENSP00000273398	ENSP00000412553
ENSP00000273398	ENSP00000413319
ENSP00000273398	ENSP00000413632
ENSP00000273398	ENSP00000414373
ENSP00000273398	ENSP00000417748
ENSP00000273480	ENSP00000276533
ENSP00000273480	ENSP00000277458
ENSP00000273480	ENSP00000292211
ENSP00000273480	ENSP00000295066
ENSP00000273480	ENSP00000297290
ENSP00000273480	ENSP00000302120
ENSP00000273480	ENSP00000304350
ENSP00000273480	ENSP00000305721
ENSP00000273480	ENSP00000307786
ENSP00000273480	ENSP00000313691

ENSP00000273480	ENSP00000313881
ENSP00000273480	ENSP00000314004
ENSP00000273480	ENSP00000318775
ENSP00000273480	ENSP00000321927
ENSP00000273480	ENSP00000322730
ENSP00000273480	ENSP00000334657
ENSP00000273480	ENSP00000339109
ENSP00000273480	ENSP00000359206
ENSP00000273480	ENSP00000362110
ENSP00000273480	ENSP00000365025
ENSP00000273480	ENSP00000389934
ENSP00000273588	ENSP00000275605
ENSP00000273588	ENSP00000319531
ENSP00000273588	ENSP00000333667
ENSP00000273588	ENSP00000358417
ENSP00000273588	ENSP00000365773
ENSP00000273590	ENSP00000341551
ENSP00000273598	ENSP00000352265
ENSP00000273666	ENSP00000334100
ENSP00000273666	ENSP00000343144
ENSP00000273666	ENSP00000347169
ENSP00000273666	ENSP00000349486
ENSP00000273666	ENSP00000361465
ENSP00000273666	ENSP00000386621
ENSP00000273666	ENSP00000389934
ENSP00000273783	ENSP00000295066
ENSP00000273783	ENSP00000304668
ENSP00000273783	ENSP00000310440
ENSP00000273783	ENSP00000312042
ENSP00000273783	ENSP00000312735
ENSP00000273783	ENSP00000319979
ENSP00000273783	ENSP00000325448
ENSP00000273783	ENSP00000327821
ENSP00000273783	ENSP00000330343
ENSP00000273783	ENSP00000330408
ENSP00000273783	ENSP00000353575
ENSP00000273783	ENSP00000361467
ENSP00000273783	ENSP00000362993
ENSP00000273783	ENSP00000364119
ENSP00000273783	ENSP00000373509
ENSP00000273783	ENSP00000382595
ENSP00000273783	ENSP00000394869
ENSP00000273920	ENSP00000286428
ENSP00000273920	ENSP00000296273
ENSP00000273920	ENSP00000342755
ENSP00000273920	ENSP00000353660

ENSP00000273920	ENSP00000369411
ENSP00000273920	ENSP00000370151
ENSP00000273951	ENSP00000367064
ENSP00000273962	ENSP00000314311
ENSP00000273962	ENSP00000314441
ENSP00000273962	ENSP00000327821
ENSP00000273962	ENSP00000328671
ENSP00000273962	ENSP00000333934
ENSP00000273962	ENSP00000346453
ENSP00000273962	ENSP00000356476
ENSP00000273962	ENSP00000356548
ENSP00000273962	ENSP00000364320
ENSP00000273962	ENSP00000365837
ENSP00000273968	ENSP00000367346
ENSP00000273968	ENSP00000369820
ENSP00000273986	ENSP00000329623
ENSP00000273986	ENSP00000361686
ENSP00000273986	ENSP00000370607
ENSP00000273986	ENSP00000392330
ENSP00000274000	ENSP00000377148
ENSP00000274030	ENSP00000309555
ENSP00000274030	ENSP00000311344
ENSP00000274030	ENSP00000327145
ENSP00000274031	ENSP00000299424
ENSP00000274031	ENSP00000309555
ENSP00000274031	ENSP00000344537
ENSP00000274031	ENSP00000350633
ENSP00000274031	ENSP00000352516
ENSP00000274140	ENSP00000287936
ENSP00000274140	ENSP00000288774
ENSP00000274140	ENSP00000290649
ENSP00000274140	ENSP00000295066
ENSP00000274140	ENSP00000303511
ENSP00000274140	ENSP00000304051
ENSP00000274140	ENSP00000315791
ENSP00000274140	ENSP00000320885
ENSP00000274140	ENSP00000331487
ENSP00000274140	ENSP00000333836
ENSP00000274140	ENSP00000348010
ENSP00000274140	ENSP00000357998
ENSP00000274140	ENSP00000366395
ENSP00000274140	ENSP00000379457
ENSP00000274140	ENSP00000383719
ENSP00000274140	ENSP00000387347
ENSP00000274242	ENSP00000274606
ENSP00000274242	ENSP00000283875

ENSP00000274242	ENSP00000287038
ENSP00000274242	ENSP00000294189
ENSP00000274242	ENSP00000298283
ENSP00000274242	ENSP00000300151
ENSP00000274242	ENSP00000306548
ENSP00000274242	ENSP00000307889
ENSP00000274242	ENSP00000308897
ENSP00000274242	ENSP00000309334
ENSP00000274242	ENSP00000309830
ENSP00000274242	ENSP00000311430
ENSP00000274242	ENSP00000317334
ENSP00000274242	ENSP00000322419
ENSP00000274242	ENSP00000327309
ENSP00000274242	ENSP00000330945
ENSP00000274242	ENSP00000339484
ENSP00000274242	ENSP00000339795
ENSP00000274242	ENSP00000345156
ENSP00000274242	ENSP00000346015
ENSP00000274242	ENSP00000346022
ENSP00000274242	ENSP00000346027
ENSP00000274242	ENSP00000346037
ENSP00000274242	ENSP00000346088
ENSP00000274242	ENSP00000346120
ENSP00000274242	ENSP00000347329
ENSP00000274242	ENSP00000347997
ENSP00000274242	ENSP00000350708
ENSP00000274242	ENSP00000353998
ENSP00000274242	ENSP00000354525
ENSP00000274242	ENSP00000354739
ENSP00000274242	ENSP00000359345
ENSP00000274242	ENSP00000361076
ENSP00000274242	ENSP00000363018
ENSP00000274242	ENSP00000363559
ENSP00000274242	ENSP00000363676
ENSP00000274242	ENSP00000364320
ENSP00000274242	ENSP00000366249
ENSP00000274242	ENSP00000368699
ENSP00000274242	ENSP00000373039
ENSP00000274242	ENSP00000373404
ENSP00000274242	ENSP00000374455
ENSP00000274242	ENSP00000375730
ENSP00000274242	ENSP00000376946
ENSP00000274242	ENSP00000377640
ENSP00000274242	ENSP00000377865
ENSP00000274242	ENSP00000378160
ENSP00000274242	ENSP00000380156

ENSP00000274242	ENSP00000381654
ENSP00000274242	ENSP00000386717
ENSP00000274242	ENSP00000389103
ENSP00000274242	ENSP00000393393
ENSP00000274242	ENSP00000396152
ENSP00000274242	ENSP00000402338
ENSP00000274242	ENSP00000410416
ENSP00000274255	ENSP00000278616
ENSP00000274255	ENSP00000284811
ENSP00000274255	ENSP00000294339
ENSP00000274255	ENSP00000301019
ENSP00000274255	ENSP00000301764
ENSP00000274255	ENSP00000304102
ENSP00000274255	ENSP00000308620
ENSP00000274255	ENSP00000311083
ENSP00000274255	ENSP00000311280
ENSP00000274255	ENSP00000320566
ENSP00000274255	ENSP00000325863
ENSP00000274255	ENSP00000329357
ENSP00000274255	ENSP00000334188
ENSP00000274255	ENSP00000335628
ENSP00000274255	ENSP00000337393
ENSP00000274255	ENSP00000339527
ENSP00000274255	ENSP00000341551
ENSP00000274255	ENSP00000343701
ENSP00000274255	ENSP00000350512
ENSP00000274255	ENSP00000360621
ENSP00000274255	ENSP00000362608
ENSP00000274255	ENSP00000369497
ENSP00000274255	ENSP00000378529
ENSP00000274255	ENSP00000391723
ENSP00000274255	ENSP00000402084
ENSP00000274255	ENSP00000413720
ENSP00000274289	ENSP00000277541
ENSP00000274289	ENSP00000285873
ENSP00000274289	ENSP00000288976
ENSP00000274289	ENSP00000290158
ENSP00000274289	ENSP00000295951
ENSP00000274289	ENSP00000296509
ENSP00000274289	ENSP00000296733
ENSP00000274289	ENSP00000297338
ENSP00000274289	ENSP00000298139
ENSP00000274289	ENSP00000300584
ENSP00000274289	ENSP00000304226
ENSP00000274289	ENSP00000307272
ENSP00000274289	ENSP00000307853

ENSP00000274289	ENSP00000308699
ENSP00000274289	ENSP00000310649
ENSP00000274289	ENSP00000317636
ENSP00000274289	ENSP00000321386
ENSP00000274289	ENSP00000322218
ENSP00000274289	ENSP00000322608
ENSP00000274289	ENSP00000323663
ENSP00000274289	ENSP00000333873
ENSP00000274289	ENSP00000335357
ENSP00000274289	ENSP00000336712
ENSP00000274289	ENSP00000336868
ENSP00000274289	ENSP00000342510
ENSP00000274289	ENSP00000346566
ENSP00000274289	ENSP00000347232
ENSP00000274289	ENSP00000348554
ENSP00000274289	ENSP00000351284
ENSP00000274289	ENSP00000352572
ENSP00000274289	ENSP00000354720
ENSP00000274289	ENSP00000354916
ENSP00000274289	ENSP00000355922
ENSP00000274289	ENSP00000359206
ENSP00000274289	ENSP00000360076
ENSP00000274289	ENSP00000362578
ENSP00000274289	ENSP00000363779
ENSP00000274289	ENSP00000367408
ENSP00000274289	ENSP00000367638
ENSP00000274289	ENSP00000368017
ENSP00000274289	ENSP00000368349
ENSP00000274289	ENSP00000371308
ENSP00000274289	ENSP00000371451
ENSP00000274289	ENSP00000372689
ENSP00000274289	ENSP00000377655
ENSP00000274289	ENSP00000378350
ENSP00000274289	ENSP00000378529
ENSP00000274289	ENSP00000381932
ENSP00000274289	ENSP00000382423
ENSP00000274289	ENSP00000383145
ENSP00000274289	ENSP00000402527
ENSP00000274311	ENSP00000287295
ENSP00000274311	ENSP00000296577
ENSP00000274311	ENSP00000296674
ENSP00000274311	ENSP00000302896
ENSP00000274311	ENSP00000308897
ENSP00000274311	ENSP00000309561
ENSP00000274311	ENSP00000310842
ENSP00000274311	ENSP00000311028

ENSP00000274311	ENSP00000318115
ENSP00000274311	ENSP00000339095
ENSP00000274311	ENSP00000346022
ENSP00000274311	ENSP00000354739
ENSP00000274311	ENSP00000355541
ENSP00000274311	ENSP00000356811
ENSP00000274311	ENSP00000360268
ENSP00000274311	ENSP00000360532
ENSP00000274311	ENSP00000364320
ENSP00000274311	ENSP00000368253
ENSP00000274311	ENSP00000369162
ENSP00000274311	ENSP00000369757
ENSP00000274311	ENSP00000377865
ENSP00000274311	ENSP00000379339
ENSP00000274311	ENSP00000388658
ENSP00000274311	ENSP00000414321
ENSP00000274353	ENSP00000353701
ENSP00000274368	ENSP00000296099
ENSP00000274376	ENSP00000276297
ENSP00000274376	ENSP00000287600
ENSP00000274376	ENSP00000295987
ENSP00000274376	ENSP00000298552
ENSP00000274376	ENSP00000303042
ENSP00000274376	ENSP00000303507
ENSP00000274376	ENSP00000307046
ENSP00000274376	ENSP00000313829
ENSP00000274376	ENSP00000316460
ENSP00000274376	ENSP00000339191
ENSP00000274376	ENSP00000340019
ENSP00000274376	ENSP00000342681
ENSP00000274376	ENSP00000346550
ENSP00000274376	ENSP00000347184
ENSP00000274376	ENSP00000356346
ENSP00000274376	ENSP00000359206
ENSP00000274376	ENSP00000360922
ENSP00000274376	ENSP00000363763
ENSP00000274376	ENSP00000364898
ENSP00000274376	ENSP00000371897
ENSP00000274376	ENSP00000376946
ENSP00000274458	ENSP00000308236
ENSP00000274458	ENSP00000365401
ENSP00000274458	ENSP00000419475
ENSP00000274473	ENSP00000290541
ENSP00000274473	ENSP00000310094
ENSP00000274473	ENSP00000310561
ENSP00000274473	ENSP00000317902

ENSP00000274473	ENSP00000326924
ENSP00000274473	ENSP00000347532
ENSP00000274473	ENSP00000352047
ENSP00000274473	ENSP00000360817
ENSP00000274473	ENSP00000368612
ENSP00000274473	ENSP00000381793
ENSP00000274473	ENSP00000383402
ENSP00000274473	ENSP00000396976
ENSP00000274532	ENSP00000344844
ENSP00000274606	ENSP00000278572
ENSP00000274606	ENSP00000280557
ENSP00000274606	ENSP00000283109
ENSP00000274606	ENSP00000287038
ENSP00000274606	ENSP00000289371
ENSP00000274606	ENSP00000294189
ENSP00000274606	ENSP00000296411
ENSP00000274606	ENSP00000296577
ENSP00000274606	ENSP00000296674
ENSP00000274606	ENSP00000296802
ENSP00000274606	ENSP00000298283
ENSP00000274606	ENSP00000298746
ENSP00000274606	ENSP00000300151
ENSP00000274606	ENSP00000301585
ENSP00000274606	ENSP00000302886
ENSP00000274606	ENSP00000302896
ENSP00000274606	ENSP00000305702
ENSP00000274606	ENSP00000306548
ENSP00000274606	ENSP00000307491
ENSP00000274606	ENSP00000307525
ENSP00000274606	ENSP00000307889
ENSP00000274606	ENSP00000308897
ENSP00000274606	ENSP00000309334
ENSP00000274606	ENSP00000309830
ENSP00000274606	ENSP00000310448
ENSP00000274606	ENSP00000311028
ENSP00000274606	ENSP00000311430
ENSP00000274606	ENSP00000312587
ENSP00000274606	ENSP00000313674
ENSP00000274606	ENSP00000314311
ENSP00000274606	ENSP00000314348
ENSP00000274606	ENSP00000315182
ENSP00000274606	ENSP00000317376
ENSP00000274606	ENSP00000317992
ENSP00000274606	ENSP00000318646
ENSP00000274606	ENSP00000320917
ENSP00000274606	ENSP00000321326

ENSP00000274606	ENSP00000322396
ENSP00000274606	ENSP00000322419
ENSP00000274606	ENSP00000323302
ENSP00000274606	ENSP00000323856
ENSP00000274606	ENSP00000325312
ENSP00000274606	ENSP00000326806
ENSP00000274606	ENSP00000326981
ENSP00000274606	ENSP00000328854
ENSP00000274606	ENSP00000331019
ENSP00000274606	ENSP00000332103
ENSP00000274606	ENSP00000332198
ENSP00000274606	ENSP00000336702
ENSP00000274606	ENSP00000338862
ENSP00000274606	ENSP00000339095
ENSP00000274606	ENSP00000339795
ENSP00000274606	ENSP00000341885
ENSP00000274606	ENSP00000345096
ENSP00000274606	ENSP00000345156
ENSP00000274606	ENSP00000345917
ENSP00000274606	ENSP00000346015
ENSP00000274606	ENSP00000346022
ENSP00000274606	ENSP00000346027
ENSP00000274606	ENSP00000346037
ENSP00000274606	ENSP00000346050
ENSP00000274606	ENSP00000346067
ENSP00000274606	ENSP00000346088
ENSP00000274606	ENSP00000346120
ENSP00000274606	ENSP00000346453
ENSP00000274606	ENSP00000346725
ENSP00000274606	ENSP00000350011
ENSP00000274606	ENSP00000352021
ENSP00000274606	ENSP00000352738
ENSP00000274606	ENSP00000354040
ENSP00000274606	ENSP00000354525
ENSP00000274606	ENSP00000354739
ENSP00000274606	ENSP00000354812
ENSP00000274606	ENSP00000355541
ENSP00000274606	ENSP00000356410
ENSP00000274606	ENSP00000358563
ENSP00000274606	ENSP00000358812
ENSP00000274606	ENSP00000359345
ENSP00000274606	ENSP00000359688
ENSP00000274606	ENSP00000359939
ENSP00000274606	ENSP00000360031
ENSP00000274606	ENSP00000360412
ENSP00000274606	ENSP00000361232

ENSP00000274606	ENSP00000361982
ENSP00000274606	ENSP00000362744
ENSP00000274606	ENSP00000363018
ENSP00000274606	ENSP00000363313
ENSP00000274606	ENSP00000363384
ENSP00000274606	ENSP00000363559
ENSP00000274606	ENSP00000363676
ENSP00000274606	ENSP00000363944
ENSP00000274606	ENSP00000364320
ENSP00000274606	ENSP00000364543
ENSP00000274606	ENSP00000366519
ENSP00000274606	ENSP00000369757
ENSP00000274606	ENSP00000372199
ENSP00000274606	ENSP00000372827
ENSP00000274606	ENSP00000373404
ENSP00000274606	ENSP00000374390
ENSP00000274606	ENSP00000375730
ENSP00000274606	ENSP00000377640
ENSP00000274606	ENSP00000377865
ENSP00000274606	ENSP00000377969
ENSP00000274606	ENSP00000378160
ENSP00000274606	ENSP00000379607
ENSP00000274606	ENSP00000379888
ENSP00000274606	ENSP00000380156
ENSP00000274606	ENSP00000380495
ENSP00000274606	ENSP00000380982
ENSP00000274606	ENSP00000382392
ENSP00000274606	ENSP00000386717
ENSP00000274606	ENSP00000388464
ENSP00000274606	ENSP00000389103
ENSP00000274606	ENSP00000393241
ENSP00000274606	ENSP00000393393
ENSP00000274606	ENSP00000394400
ENSP00000274606	ENSP00000399530
ENSP00000274606	ENSP00000400626
ENSP00000274606	ENSP00000402338
ENSP00000274606	ENSP00000403175
ENSP00000274606	ENSP00000412310
ENSP00000274606	ENSP00000412566
ENSP00000274606	ENSP00000412583
ENSP00000274606	ENSP00000414321
ENSP00000274606	ENSP00000416110
ENSP00000274606	ENSP00000417763
ENSP00000274606	ENSP00000419740
ENSP00000274606	ENSP00000420588
ENSP00000274680	ENSP00000281828

ENSP00000274680	ENSP00000282276
ENSP00000274680	ENSP00000289371
ENSP00000274680	ENSP00000298569
ENSP00000274680	ENSP00000301585
ENSP00000274680	ENSP00000302114
ENSP00000274680	ENSP00000302955
ENSP00000274680	ENSP00000303279
ENSP00000274680	ENSP00000311766
ENSP00000274680	ENSP00000317334
ENSP00000274680	ENSP00000319323
ENSP00000274680	ENSP00000333376
ENSP00000274680	ENSP00000335675
ENSP00000274680	ENSP00000340019
ENSP00000274680	ENSP00000340093
ENSP00000274680	ENSP00000341543
ENSP00000274680	ENSP00000344241
ENSP00000274680	ENSP00000348886
ENSP00000274680	ENSP00000351380
ENSP00000274680	ENSP00000352219
ENSP00000274680	ENSP00000352738
ENSP00000274680	ENSP00000354204
ENSP00000274680	ENSP00000356329
ENSP00000274680	ENSP00000356405
ENSP00000274680	ENSP00000356587
ENSP00000274680	ENSP00000358140
ENSP00000274680	ENSP00000362576
ENSP00000274680	ENSP00000364794
ENSP00000274680	ENSP00000367498
ENSP00000274680	ENSP00000368140
ENSP00000274680	ENSP00000370083
ENSP00000274680	ENSP00000370119
ENSP00000274680	ENSP00000370377
ENSP00000274680	ENSP00000370607
ENSP00000274680	ENSP00000374399
ENSP00000274680	ENSP00000376946
ENSP00000274680	ENSP00000379888
ENSP00000274680	ENSP00000394085
ENSP00000274712	ENSP00000295488
ENSP00000274712	ENSP00000296702
ENSP00000274712	ENSP00000296805
ENSP00000274712	ENSP00000300413
ENSP00000274712	ENSP00000301764
ENSP00000274712	ENSP00000302160
ENSP00000274712	ENSP00000304350
ENSP00000274712	ENSP00000305790
ENSP00000274712	ENSP00000310448

ENSP00000274712	ENSP00000313674
ENSP00000274712	ENSP00000315035
ENSP00000274712	ENSP00000315379
ENSP00000274712	ENSP00000318861
ENSP00000274712	ENSP00000319341
ENSP00000274712	ENSP00000323302
ENSP00000274712	ENSP00000324122
ENSP00000274712	ENSP00000332103
ENSP00000274712	ENSP00000335321
ENSP00000274712	ENSP00000342374
ENSP00000274712	ENSP00000357631
ENSP00000274712	ENSP00000359454
ENSP00000274712	ENSP00000361577
ENSP00000274712	ENSP00000362110
ENSP00000274712	ENSP00000363313
ENSP00000274712	ENSP00000364813
ENSP00000274712	ENSP00000370589
ENSP00000274712	ENSP00000372883
ENSP00000274712	ENSP00000403345
ENSP00000274712	ENSP00000406280
ENSP00000274712	ENSP00000410758
ENSP00000274712	ENSP00000412566
ENSP00000274712	ENSP00000414006
ENSP00000274712	ENSP00000414634
ENSP00000274787	ENSP00000317159
ENSP00000274787	ENSP00000325562
ENSP00000274787	ENSP00000336752
ENSP00000274787	ENSP00000354499
ENSP00000274787	ENSP00000354876
ENSP00000274787	ENSP00000354982
ENSP00000274787	ENSP00000368140
ENSP00000274813	ENSP00000281317
ENSP00000274813	ENSP00000301585
ENSP00000274813	ENSP00000381970
ENSP00000274849	ENSP00000278572
ENSP00000274849	ENSP00000291576
ENSP00000274849	ENSP00000297990
ENSP00000274849	ENSP00000301364
ENSP00000274849	ENSP00000305702
ENSP00000274849	ENSP00000308179
ENSP00000274849	ENSP00000311135
ENSP00000274849	ENSP00000314348
ENSP00000274849	ENSP00000324122
ENSP00000274849	ENSP00000327179
ENSP00000274849	ENSP00000332340
ENSP00000274849	ENSP00000339095

ENSP00000274849	ENSP00000339795
ENSP00000274849	ENSP00000341885
ENSP00000274849	ENSP00000354125
ENSP00000274849	ENSP00000355541
ENSP00000274849	ENSP00000358812
ENSP00000274849	ENSP00000363642
ENSP00000274849	ENSP00000366557
ENSP00000274849	ENSP00000370589
ENSP00000274849	ENSP00000371101
ENSP00000274849	ENSP00000386134
ENSP00000274849	ENSP00000414321
ENSP00000274849	ENSP00000417229
ENSP00000274897	ENSP00000357283
ENSP00000275015	ENSP00000293362
ENSP00000275015	ENSP00000295025
ENSP00000275015	ENSP00000297185
ENSP00000275015	ENSP00000313420
ENSP00000275015	ENSP00000314556
ENSP00000275015	ENSP00000318115
ENSP00000275015	ENSP00000348551
ENSP00000275015	ENSP00000349748
ENSP00000275015	ENSP00000351416
ENSP00000275015	ENSP00000355890
ENSP00000275015	ENSP00000359727
ENSP00000275015	ENSP00000364794
ENSP00000275015	ENSP00000368797
ENSP00000275015	ENSP00000379093
ENSP00000275053	ENSP00000278412
ENSP00000275053	ENSP00000281708
ENSP00000275053	ENSP00000386239
ENSP00000275428	ENSP00000319531
ENSP00000275517	ENSP00000296257
ENSP00000275517	ENSP00000297338
ENSP00000275517	ENSP00000313851
ENSP00000275517	ENSP00000341382
ENSP00000275517	ENSP00000352257
ENSP00000275517	ENSP00000354720
ENSP00000275517	ENSP00000358497
ENSP00000275605	ENSP00000302620
ENSP00000275605	ENSP00000302896
ENSP00000275605	ENSP00000331817
ENSP00000275605	ENSP00000333667
ENSP00000275605	ENSP00000363021
ENSP00000275605	ENSP00000365534
ENSP00000275605	ENSP00000365837
ENSP00000275605	ENSP00000374213

ENSP00000275605	ENSP00000382595
ENSP00000275635	ENSP00000356505
ENSP00000275635	ENSP00000384675
ENSP00000275699	ENSP00000300101
ENSP00000275730	ENSP00000292476
ENSP00000275730	ENSP00000302790
ENSP00000275730	ENSP00000372857
ENSP00000275780	ENSP00000278919
ENSP00000275780	ENSP00000293925
ENSP00000275780	ENSP00000295951
ENSP00000275780	ENSP00000301019
ENSP00000275780	ENSP00000324020
ENSP00000275780	ENSP00000329411
ENSP00000275780	ENSP00000336587
ENSP00000275780	ENSP00000348273
ENSP00000275780	ENSP00000362578
ENSP00000275820	ENSP00000279230
ENSP00000275820	ENSP00000295488
ENSP00000275820	ENSP00000323511
ENSP00000275820	ENSP00000327179
ENSP00000275820	ENSP00000345988
ENSP00000275820	ENSP00000359454
ENSP00000275820	ENSP00000359837
ENSP00000275820	ENSP00000366179
ENSP00000275820	ENSP00000368565
ENSP00000275820	ENSP00000384302
ENSP00000276014	ENSP00000281708
ENSP00000276014	ENSP00000287598
ENSP00000276014	ENSP00000296509
ENSP00000276014	ENSP00000298048
ENSP00000276014	ENSP00000302530
ENSP00000276014	ENSP00000306999
ENSP00000276014	ENSP00000307023
ENSP00000276014	ENSP00000307853
ENSP00000276014	ENSP00000309871
ENSP00000276014	ENSP00000310649
ENSP00000276014	ENSP00000311083
ENSP00000276014	ENSP00000317156
ENSP00000276014	ENSP00000320130
ENSP00000276014	ENSP00000322180
ENSP00000276014	ENSP00000334188
ENSP00000276014	ENSP00000343741
ENSP00000276014	ENSP00000344460
ENSP00000276014	ENSP00000346566
ENSP00000276014	ENSP00000348965
ENSP00000276014	ENSP00000351665

ENSP00000276014	ENSP00000353622
ENSP00000276014	ENSP00000353698
ENSP00000276014	ENSP00000353793
ENSP00000276014	ENSP00000354916
ENSP00000276014	ENSP00000356405
ENSP00000276014	ENSP00000356989
ENSP00000276014	ENSP00000360147
ENSP00000276014	ENSP00000360621
ENSP00000276014	ENSP00000361465
ENSP00000276014	ENSP00000362171
ENSP00000276014	ENSP00000363827
ENSP00000276014	ENSP00000368976
ENSP00000276014	ENSP00000372023
ENSP00000276014	ENSP00000377793
ENSP00000276014	ENSP00000378529
ENSP00000276014	ENSP00000384675
ENSP00000276062	ENSP00000296028
ENSP00000276062	ENSP00000300651
ENSP00000276062	ENSP00000357484
ENSP00000276072	ENSP00000283195
ENSP00000276072	ENSP00000283875
ENSP00000276072	ENSP00000285896
ENSP00000276072	ENSP00000290524
ENSP00000276072	ENSP00000293406
ENSP00000276072	ENSP00000296328
ENSP00000276072	ENSP00000299424
ENSP00000276072	ENSP00000302640
ENSP00000276072	ENSP00000306245
ENSP00000276072	ENSP00000306983
ENSP00000276072	ENSP00000307208
ENSP00000276072	ENSP00000307684
ENSP00000276072	ENSP00000314949
ENSP00000276072	ENSP00000320949
ENSP00000276072	ENSP00000322570
ENSP00000276072	ENSP00000329029
ENSP00000276072	ENSP00000332171
ENSP00000276072	ENSP00000333920
ENSP00000276072	ENSP00000340896
ENSP00000276072	ENSP00000341805
ENSP00000276072	ENSP00000344537
ENSP00000276072	ENSP00000345702
ENSP00000276072	ENSP00000345793
ENSP00000276072	ENSP00000348168
ENSP00000276072	ENSP00000349213
ENSP00000276072	ENSP00000351490
ENSP00000276072	ENSP00000351697

ENSP00000276072	ENSP00000353622
ENSP00000276072	ENSP00000354633
ENSP00000276072	ENSP00000354673
ENSP00000276072	ENSP00000355051
ENSP00000276072	ENSP00000355205
ENSP00000276072	ENSP00000355759
ENSP00000276072	ENSP00000357047
ENSP00000276072	ENSP00000357494
ENSP00000276072	ENSP00000358854
ENSP00000276072	ENSP00000358886
ENSP00000276072	ENSP00000359290
ENSP00000276072	ENSP00000359531
ENSP00000276072	ENSP00000360329
ENSP00000276072	ENSP00000360515
ENSP00000276072	ENSP00000361998
ENSP00000276072	ENSP00000362068
ENSP00000276072	ENSP00000366974
ENSP00000276072	ENSP00000367406
ENSP00000276072	ENSP00000368349
ENSP00000276072	ENSP00000369695
ENSP00000276072	ENSP00000372170
ENSP00000276072	ENSP00000374213
ENSP00000276072	ENSP00000377969
ENSP00000276072	ENSP00000378160
ENSP00000276072	ENSP00000378394
ENSP00000276072	ENSP00000379372
ENSP00000276072	ENSP00000380982
ENSP00000276072	ENSP00000381031
ENSP00000276072	ENSP00000384597
ENSP00000276072	ENSP00000386621
ENSP00000276072	ENSP00000386717
ENSP00000276072	ENSP00000388872
ENSP00000276072	ENSP00000417281
ENSP00000276201	ENSP00000280665
ENSP00000276201	ENSP00000296577
ENSP00000276201	ENSP00000310596
ENSP00000276201	ENSP00000315791
ENSP00000276201	ENSP00000315859
ENSP00000276201	ENSP00000316879
ENSP00000276201	ENSP00000317872
ENSP00000276201	ENSP00000326767
ENSP00000276201	ENSP00000326806
ENSP00000276201	ENSP00000331817
ENSP00000276201	ENSP00000334808
ENSP00000276201	ENSP00000338862
ENSP00000276201	ENSP00000340823

ENSP00000276201	ENSP00000347733
ENSP00000276201	ENSP00000348708
ENSP00000276201	ENSP00000349640
ENSP00000276201	ENSP00000352400
ENSP00000276201	ENSP00000353741
ENSP00000276201	ENSP00000355261
ENSP00000276201	ENSP00000356989
ENSP00000276201	ENSP00000361557
ENSP00000276201	ENSP00000362135
ENSP00000276201	ENSP00000364289
ENSP00000276201	ENSP00000379607
ENSP00000276201	ENSP00000391432
ENSP00000276201	ENSP00000402240
ENSP00000276201	ENSP00000402515
ENSP00000276297	ENSP00000292894
ENSP00000276297	ENSP00000315702
ENSP00000276297	ENSP00000325423
ENSP00000276297	ENSP00000347948
ENSP00000276297	ENSP00000361027
ENSP00000276297	ENSP00000379290
ENSP00000276326	ENSP00000282050
ENSP00000276326	ENSP00000292385
ENSP00000276326	ENSP00000295897
ENSP00000276326	ENSP00000295901
ENSP00000276326	ENSP00000296255
ENSP00000276326	ENSP00000296930
ENSP00000276326	ENSP00000297185
ENSP00000276326	ENSP00000298852
ENSP00000276326	ENSP00000309629
ENSP00000276326	ENSP00000310129
ENSP00000276326	ENSP00000311766
ENSP00000276326	ENSP00000314556
ENSP00000276326	ENSP00000317334
ENSP00000276326	ENSP00000318195
ENSP00000276326	ENSP00000319169
ENSP00000276326	ENSP00000321546
ENSP00000276326	ENSP00000324573
ENSP00000276326	ENSP00000325785
ENSP00000276326	ENSP00000327821
ENSP00000276326	ENSP00000328088
ENSP00000276326	ENSP00000338814
ENSP00000276326	ENSP00000348784
ENSP00000276326	ENSP00000354720
ENSP00000276326	ENSP00000354739
ENSP00000276326	ENSP00000357048
ENSP00000276326	ENSP00000358417

ENSP00000276326	ENSP00000358994
ENSP00000276326	ENSP00000369042
ENSP00000276326	ENSP00000370517
ENSP00000276326	ENSP00000379888
ENSP00000276326	ENSP00000380921
ENSP00000276326	ENSP00000382177
ENSP00000276326	ENSP00000407487
ENSP00000276326	ENSP00000407879
ENSP00000276414	ENSP00000416959
ENSP00000276416	ENSP00000277865
ENSP00000276416	ENSP00000286428
ENSP00000276416	ENSP00000292385
ENSP00000276416	ENSP00000301843
ENSP00000276416	ENSP00000310621
ENSP00000276416	ENSP00000311005
ENSP00000276416	ENSP00000315659
ENSP00000276416	ENSP00000316845
ENSP00000276416	ENSP00000317468
ENSP00000276416	ENSP00000320566
ENSP00000276416	ENSP00000320924
ENSP00000276416	ENSP00000326238
ENSP00000276416	ENSP00000334188
ENSP00000276416	ENSP00000338967
ENSP00000276416	ENSP00000345728
ENSP00000276416	ENSP00000347919
ENSP00000276416	ENSP00000348163
ENSP00000276416	ENSP00000348215
ENSP00000276416	ENSP00000349877
ENSP00000276416	ENSP00000350708
ENSP00000276416	ENSP00000352802
ENSP00000276416	ENSP00000353622
ENSP00000276416	ENSP00000354558
ENSP00000276416	ENSP00000354927
ENSP00000276416	ENSP00000356989
ENSP00000276416	ENSP00000358812
ENSP00000276416	ENSP00000358831
ENSP00000276416	ENSP00000360798
ENSP00000276416	ENSP00000362115
ENSP00000276416	ENSP00000363734
ENSP00000276416	ENSP00000365005
ENSP00000276416	ENSP00000365891
ENSP00000276416	ENSP00000366863
ENSP00000276416	ENSP00000369042
ENSP00000276416	ENSP00000370377
ENSP00000276416	ENSP00000372694
ENSP00000276416	ENSP00000380460

ENSP00000276416	ENSP00000386878
ENSP00000276416	ENSP00000391249
ENSP00000276416	ENSP00000397598
ENSP00000276416	ENSP00000412319
ENSP00000276416	ENSP00000413625
ENSP00000276416	ENSP00000415678
ENSP00000276416	ENSP00000419599
ENSP00000276533	ENSP00000278412
ENSP00000276533	ENSP00000312870
ENSP00000276533	ENSP00000322570
ENSP00000276533	ENSP00000350491
ENSP00000276533	ENSP00000353793
ENSP00000276533	ENSP00000354522
ENSP00000276533	ENSP00000368349
ENSP00000276533	ENSP00000380504
ENSP00000276533	ENSP00000401018
ENSP00000276533	ENSP00000413632
ENSP00000276569	ENSP00000281172
ENSP00000276570	ENSP00000294053
ENSP00000276570	ENSP00000303191
ENSP00000276570	ENSP00000304350
ENSP00000276570	ENSP00000305790
ENSP00000276570	ENSP00000329558
ENSP00000276570	ENSP00000332103
ENSP00000276570	ENSP00000335321
ENSP00000276570	ENSP00000342374
ENSP00000276570	ENSP00000351137
ENSP00000276570	ENSP00000360532
ENSP00000276570	ENSP00000361577
ENSP00000276570	ENSP00000362110
ENSP00000276570	ENSP00000366557
ENSP00000276570	ENSP00000412566
ENSP00000276585	ENSP00000301585
ENSP00000276585	ENSP00000301962
ENSP00000276590	ENSP00000299601
ENSP00000276603	ENSP00000278616
ENSP00000276603	ENSP00000280097
ENSP00000276603	ENSP00000281623
ENSP00000276603	ENSP00000283871
ENSP00000276603	ENSP00000287647
ENSP00000276603	ENSP00000288602
ENSP00000276603	ENSP00000288757
ENSP00000276603	ENSP00000290158
ENSP00000276603	ENSP00000290291
ENSP00000276603	ENSP00000292385
ENSP00000276603	ENSP00000294740

ENSP00000276603	ENSP00000299767
ENSP00000276603	ENSP00000300086
ENSP00000276603	ENSP00000300417
ENSP00000276603	ENSP00000300961
ENSP00000276603	ENSP00000301011
ENSP00000276603	ENSP00000301764
ENSP00000276603	ENSP00000302647
ENSP00000276603	ENSP00000302886
ENSP00000276603	ENSP00000303042
ENSP00000276603	ENSP00000303712
ENSP00000276603	ENSP00000304854
ENSP00000276603	ENSP00000305631
ENSP00000276603	ENSP00000309572
ENSP00000276603	ENSP00000309629
ENSP00000276603	ENSP00000310723
ENSP00000276603	ENSP00000311579
ENSP00000276603	ENSP00000313420
ENSP00000276603	ENSP00000314311
ENSP00000276603	ENSP00000319169
ENSP00000276603	ENSP00000321259
ENSP00000276603	ENSP00000322242
ENSP00000276603	ENSP00000324884
ENSP00000276603	ENSP00000325677
ENSP00000276603	ENSP00000325941
ENSP00000276603	ENSP00000328187
ENSP00000276603	ENSP00000328671
ENSP00000276603	ENSP00000328690
ENSP00000276603	ENSP00000328773
ENSP00000276603	ENSP00000334040
ENSP00000276603	ENSP00000334836
ENSP00000276603	ENSP00000336927
ENSP00000276603	ENSP00000337838
ENSP00000276603	ENSP00000338573
ENSP00000276603	ENSP00000338989
ENSP00000276603	ENSP00000339095
ENSP00000276603	ENSP00000340466
ENSP00000276603	ENSP00000341885
ENSP00000276603	ENSP00000342026
ENSP00000276603	ENSP00000343023
ENSP00000276603	ENSP00000343657
ENSP00000276603	ENSP00000344106
ENSP00000276603	ENSP00000344547
ENSP00000276603	ENSP00000345917
ENSP00000276603	ENSP00000346065
ENSP00000276603	ENSP00000346120
ENSP00000276603	ENSP00000347232

ENSP00000276603	ENSP00000347495
ENSP00000276603	ENSP00000347592
ENSP00000276603	ENSP00000347719
ENSP00000276603	ENSP00000348602
ENSP00000276603	ENSP00000349393
ENSP00000276603	ENSP00000350249
ENSP00000276603	ENSP00000350283
ENSP00000276603	ENSP00000352021
ENSP00000276603	ENSP00000353375
ENSP00000276603	ENSP00000353701
ENSP00000276603	ENSP00000354558
ENSP00000276603	ENSP00000354620
ENSP00000276603	ENSP00000354826
ENSP00000276603	ENSP00000354848
ENSP00000276603	ENSP00000355922
ENSP00000276603	ENSP00000356591
ENSP00000276603	ENSP00000357711
ENSP00000276603	ENSP00000358081
ENSP00000276603	ENSP00000358576
ENSP00000276603	ENSP00000358718
ENSP00000276603	ENSP00000361202
ENSP00000276603	ENSP00000361467
ENSP00000276603	ENSP00000361824
ENSP00000276603	ENSP00000361894
ENSP00000276603	ENSP00000361949
ENSP00000276603	ENSP00000364898
ENSP00000276603	ENSP00000366156
ENSP00000276603	ENSP00000367086
ENSP00000276603	ENSP00000367406
ENSP00000276603	ENSP00000367787
ENSP00000276603	ENSP00000368646
ENSP00000276603	ENSP00000369843
ENSP00000276603	ENSP00000370517
ENSP00000276603	ENSP00000371475
ENSP00000276603	ENSP00000372689
ENSP00000276603	ENSP00000377496
ENSP00000276603	ENSP00000379372
ENSP00000276603	ENSP00000379888
ENSP00000276603	ENSP00000379933
ENSP00000276603	ENSP00000382595
ENSP00000276603	ENSP00000384160
ENSP00000276603	ENSP00000384312
ENSP00000276603	ENSP00000391944
ENSP00000276603	ENSP00000394390
ENSP00000276603	ENSP00000396439
ENSP00000276603	ENSP00000403536

ENSP00000276646	ENSP00000294189
ENSP00000276646	ENSP00000326514
ENSP00000276646	ENSP00000341680
ENSP00000276654	ENSP00000357674
ENSP00000276689	ENSP00000317872
ENSP00000276689	ENSP00000326699
ENSP00000276692	ENSP00000284719
ENSP00000276692	ENSP00000348107
ENSP00000276692	ENSP00000348554
ENSP00000276692	ENSP00000368984
ENSP00000276692	ENSP00000396704
ENSP00000276893	ENSP00000293288
ENSP00000276893	ENSP00000297273
ENSP00000276893	ENSP00000302120
ENSP00000276893	ENSP00000307853
ENSP00000276893	ENSP00000339897
ENSP00000276893	ENSP00000344456
ENSP00000276893	ENSP00000348632
ENSP00000276893	ENSP00000352516
ENSP00000276893	ENSP00000356346
ENSP00000276893	ENSP00000358022
ENSP00000276893	ENSP00000358770
ENSP00000276893	ENSP00000368438
ENSP00000276893	ENSP00000417281
ENSP00000277010	ENSP00000285949
ENSP00000277010	ENSP00000299626
ENSP00000277010	ENSP00000301825
ENSP00000277010	ENSP00000310721
ENSP00000277010	ENSP00000317334
ENSP00000277010	ENSP00000320604
ENSP00000277010	ENSP00000325425
ENSP00000277010	ENSP00000330812
ENSP00000277010	ENSP00000331268
ENSP00000277010	ENSP00000333946
ENSP00000277010	ENSP00000341662
ENSP00000277010	ENSP00000346809
ENSP00000277010	ENSP00000348380
ENSP00000277010	ENSP00000349887
ENSP00000277010	ENSP00000350263
ENSP00000277010	ENSP00000351520
ENSP00000277010	ENSP00000353073
ENSP00000277010	ENSP00000354238
ENSP00000277010	ENSP00000355517
ENSP00000277010	ENSP00000355601
ENSP00000277010	ENSP00000358831
ENSP00000277010	ENSP00000360149

ENSP00000277010	ENSP00000360918
ENSP00000277010	ENSP00000365567
ENSP00000277010	ENSP00000365840
ENSP00000277010	ENSP00000380793
ENSP00000277010	ENSP00000387019
ENSP00000277010	ENSP00000401445
ENSP00000277010	ENSP00000415203
ENSP00000277165	ENSP00000329466
ENSP00000277165	ENSP00000355231
ENSP00000277165	ENSP00000379607
ENSP00000277225	ENSP00000417281
ENSP00000277458	ENSP00000284811
ENSP00000277458	ENSP00000337163
ENSP00000277480	ENSP00000361405
ENSP00000277480	ENSP00000369816
ENSP00000277480	ENSP00000380349
ENSP00000277508	ENSP00000356346
ENSP00000277541	ENSP00000280700
ENSP00000277541	ENSP00000281537
ENSP00000277541	ENSP00000281708
ENSP00000277541	ENSP00000282516
ENSP00000277541	ENSP00000284981
ENSP00000277541	ENSP00000292476
ENSP00000277541	ENSP00000294785
ENSP00000277541	ENSP00000295025
ENSP00000277541	ENSP00000295902
ENSP00000277541	ENSP00000302216
ENSP00000277541	ENSP00000307491
ENSP00000277541	ENSP00000309968
ENSP00000277541	ENSP00000310040
ENSP00000277541	ENSP00000311825
ENSP00000277541	ENSP00000312150
ENSP00000277541	ENSP00000322885
ENSP00000277541	ENSP00000326366
ENSP00000277541	ENSP00000328169
ENSP00000277541	ENSP00000331057
ENSP00000277541	ENSP00000331201
ENSP00000277541	ENSP00000331608
ENSP00000277541	ENSP00000332353
ENSP00000277541	ENSP00000339299
ENSP00000277541	ENSP00000344456
ENSP00000277541	ENSP00000345731
ENSP00000277541	ENSP00000347169
ENSP00000277541	ENSP00000347733
ENSP00000277541	ENSP00000348550
ENSP00000277541	ENSP00000349486

ENSP00000277541	ENSP00000352121
ENSP00000277541	ENSP00000352257
ENSP00000277541	ENSP00000352516
ENSP00000277541	ENSP00000353344
ENSP00000277541	ENSP00000355580
ENSP00000277541	ENSP00000355809
ENSP00000277541	ENSP00000357348
ENSP00000277541	ENSP00000357484
ENSP00000277541	ENSP00000358997
ENSP00000277541	ENSP00000359474
ENSP00000277541	ENSP00000363921
ENSP00000277541	ENSP00000364293
ENSP00000277541	ENSP00000364902
ENSP00000277541	ENSP00000364912
ENSP00000277541	ENSP00000365439
ENSP00000277541	ENSP00000365735
ENSP00000277541	ENSP00000365891
ENSP00000277541	ENSP00000377265
ENSP00000277541	ENSP00000378132
ENSP00000277541	ENSP00000381293
ENSP00000277541	ENSP00000383623
ENSP00000277541	ENSP00000392466
ENSP00000277541	ENSP00000402084
ENSP00000277541	ENSP00000417281
ENSP00000277554	ENSP00000281172
ENSP00000277554	ENSP00000308944
ENSP00000277554	ENSP00000328940
ENSP00000277554	ENSP00000330343
ENSP00000277554	ENSP00000358478
ENSP00000277554	ENSP00000364895
ENSP00000277570	ENSP00000281172
ENSP00000277570	ENSP00000339245
ENSP00000277570	ENSP00000350249
ENSP00000277575	ENSP00000281172
ENSP00000277575	ENSP00000360916
ENSP00000277632	ENSP00000350512
ENSP00000277657	ENSP00000381031
ENSP00000277746	ENSP00000302886
ENSP00000277746	ENSP00000324560
ENSP00000277746	ENSP00000352928
ENSP00000277746	ENSP00000355231
ENSP00000277746	ENSP00000362413
ENSP00000277865	ENSP00000282541
ENSP00000277865	ENSP00000290597
ENSP00000277865	ENSP00000290649
ENSP00000277865	ENSP00000292475

ENSP00000277865	ENSP00000293195
ENSP00000277865	ENSP00000301585
ENSP00000277865	ENSP00000301587
ENSP00000277865	ENSP00000304592
ENSP00000277865	ENSP00000308610
ENSP00000277865	ENSP00000310596
ENSP00000277865	ENSP00000312042
ENSP00000277865	ENSP00000337688
ENSP00000277865	ENSP00000339933
ENSP00000277865	ENSP00000340019
ENSP00000277865	ENSP00000341208
ENSP00000277865	ENSP00000341838
ENSP00000277865	ENSP00000342564
ENSP00000277865	ENSP00000347140
ENSP00000277865	ENSP00000348602
ENSP00000277865	ENSP00000348877
ENSP00000277865	ENSP00000354957
ENSP00000277865	ENSP00000358737
ENSP00000277865	ENSP00000359211
ENSP00000277865	ENSP00000361064
ENSP00000277865	ENSP00000366395
ENSP00000277865	ENSP00000368226
ENSP00000277865	ENSP00000371888
ENSP00000277865	ENSP00000377954
ENSP00000277865	ENSP00000379065
ENSP00000278060	ENSP00000366156
ENSP00000278060	ENSP00000408058
ENSP00000278070	ENSP00000284811
ENSP00000278070	ENSP00000339527
ENSP00000278175	ENSP00000368683
ENSP00000278243	ENSP00000306124
ENSP00000278243	ENSP00000326699
ENSP00000278243	ENSP00000359552
ENSP00000278282	ENSP00000346839
ENSP00000278282	ENSP00000367064
ENSP00000278379	ENSP00000340820
ENSP00000278407	ENSP00000295897
ENSP00000278412	ENSP00000278616
ENSP00000278412	ENSP00000283351
ENSP00000278412	ENSP00000283875
ENSP00000278412	ENSP00000283882
ENSP00000278412	ENSP00000286428
ENSP00000278412	ENSP00000287598
ENSP00000278412	ENSP00000290649
ENSP00000278412	ENSP00000295321
ENSP00000278412	ENSP00000295926

ENSP00000278412	ENSP00000296273
ENSP00000278412	ENSP00000297338
ENSP00000278412	ENSP00000299601
ENSP00000278412	ENSP00000299853
ENSP00000278412	ENSP00000300648
ENSP00000278412	ENSP00000300738
ENSP00000278412	ENSP00000301364
ENSP00000278412	ENSP00000302111
ENSP00000278412	ENSP00000302530
ENSP00000278412	ENSP00000302640
ENSP00000278412	ENSP00000302955
ENSP00000278412	ENSP00000304668
ENSP00000278412	ENSP00000305958
ENSP00000278412	ENSP00000307684
ENSP00000278412	ENSP00000310966
ENSP00000278412	ENSP00000311873
ENSP00000278412	ENSP00000313171
ENSP00000278412	ENSP00000313420
ENSP00000278412	ENSP00000314949
ENSP00000278412	ENSP00000319104
ENSP00000278412	ENSP00000320488
ENSP00000278412	ENSP00000320885
ENSP00000278412	ENSP00000321636
ENSP00000278412	ENSP00000322570
ENSP00000278412	ENSP00000325448
ENSP00000278412	ENSP00000332995
ENSP00000278412	ENSP00000333667
ENSP00000278412	ENSP00000334061
ENSP00000278412	ENSP00000334538
ENSP00000278412	ENSP00000335357
ENSP00000278412	ENSP00000336856
ENSP00000278412	ENSP00000338990
ENSP00000278412	ENSP00000340823
ENSP00000278412	ENSP00000341243
ENSP00000278412	ENSP00000343190
ENSP00000278412	ENSP00000343741
ENSP00000278412	ENSP00000344504
ENSP00000278412	ENSP00000344741
ENSP00000278412	ENSP00000346120
ENSP00000278412	ENSP00000347733
ENSP00000278412	ENSP00000351250
ENSP00000278412	ENSP00000351697
ENSP00000278412	ENSP00000351947
ENSP00000278412	ENSP00000352121
ENSP00000278412	ENSP00000353793
ENSP00000278412	ENSP00000354522

ENSP00000278412	ENSP00000354558
ENSP00000278412	ENSP00000355013
ENSP00000278412	ENSP00000355388
ENSP00000278412	ENSP00000356405
ENSP00000278412	ENSP00000356520
ENSP00000278412	ENSP00000358812
ENSP00000278412	ENSP00000359305
ENSP00000278412	ENSP00000359531
ENSP00000278412	ENSP00000360515
ENSP00000278412	ENSP00000360621
ENSP00000278412	ENSP00000360817
ENSP00000278412	ENSP00000361642
ENSP00000278412	ENSP00000362171
ENSP00000278412	ENSP00000362592
ENSP00000278412	ENSP00000362744
ENSP00000278412	ENSP00000364289
ENSP00000278412	ENSP00000365025
ENSP00000278412	ENSP00000365877
ENSP00000278412	ENSP00000366135
ENSP00000278412	ENSP00000366395
ENSP00000278412	ENSP00000366974
ENSP00000278412	ENSP00000367029
ENSP00000278412	ENSP00000368349
ENSP00000278412	ENSP00000369411
ENSP00000278412	ENSP00000369757
ENSP00000278412	ENSP00000370151
ENSP00000278412	ENSP00000374280
ENSP00000278412	ENSP00000377793
ENSP00000278412	ENSP00000379607
ENSP00000278412	ENSP00000380129
ENSP00000278412	ENSP00000380414
ENSP00000278412	ENSP00000380702
ENSP00000278412	ENSP00000386227
ENSP00000278412	ENSP00000386239
ENSP00000278412	ENSP00000386759
ENSP00000278412	ENSP00000392617
ENSP00000278412	ENSP00000396704
ENSP00000278412	ENSP00000402515
ENSP00000278412	ENSP00000403852
ENSP00000278412	ENSP00000404658
ENSP00000278412	ENSP00000412553
ENSP00000278412	ENSP00000420588
ENSP00000278422	ENSP00000356231
ENSP00000278422	ENSP00000361206
ENSP00000278422	ENSP00000370984
ENSP00000278483	ENSP00000305503

ENSP00000278483	ENSP00000350512
ENSP00000278572	ENSP00000280557
ENSP00000278572	ENSP00000283109
ENSP00000278572	ENSP00000286091
ENSP00000278572	ENSP00000287239
ENSP00000278572	ENSP00000287878
ENSP00000278572	ENSP00000288937
ENSP00000278572	ENSP00000289371
ENSP00000278572	ENSP00000295930
ENSP00000278572	ENSP00000296577
ENSP00000278572	ENSP00000296674
ENSP00000278572	ENSP00000296802
ENSP00000278572	ENSP00000296805
ENSP00000278572	ENSP00000297338
ENSP00000278572	ENSP00000297908
ENSP00000278572	ENSP00000298283
ENSP00000278572	ENSP00000300151
ENSP00000278572	ENSP00000301280
ENSP00000278572	ENSP00000301585
ENSP00000278572	ENSP00000302896
ENSP00000278572	ENSP00000305785
ENSP00000278572	ENSP00000306548
ENSP00000278572	ENSP00000306561
ENSP00000278572	ENSP00000307889
ENSP00000278572	ENSP00000308546
ENSP00000278572	ENSP00000308897
ENSP00000278572	ENSP00000309334
ENSP00000278572	ENSP00000311028
ENSP00000278572	ENSP00000311430
ENSP00000278572	ENSP00000312735
ENSP00000278572	ENSP00000314949
ENSP00000278572	ENSP00000315674
ENSP00000278572	ENSP00000317331
ENSP00000278572	ENSP00000317376
ENSP00000278572	ENSP00000318195
ENSP00000278572	ENSP00000318646
ENSP00000278572	ENSP00000319977
ENSP00000278572	ENSP00000321636
ENSP00000278572	ENSP00000323856
ENSP00000278572	ENSP00000326981
ENSP00000278572	ENSP00000328464
ENSP00000278572	ENSP00000331019
ENSP00000278572	ENSP00000336702
ENSP00000278572	ENSP00000337839
ENSP00000278572	ENSP00000339095
ENSP00000278572	ENSP00000339145

ENSP00000278572	ENSP00000339795
ENSP00000278572	ENSP00000340879
ENSP00000278572	ENSP00000341268
ENSP00000278572	ENSP00000341885
ENSP00000278572	ENSP00000342381
ENSP00000278572	ENSP00000344683
ENSP00000278572	ENSP00000345096
ENSP00000278572	ENSP00000345156
ENSP00000278572	ENSP00000346015
ENSP00000278572	ENSP00000346022
ENSP00000278572	ENSP00000346027
ENSP00000278572	ENSP00000346050
ENSP00000278572	ENSP00000346067
ENSP00000278572	ENSP00000346725
ENSP00000278572	ENSP00000347495
ENSP00000278572	ENSP00000354739
ENSP00000278572	ENSP00000354995
ENSP00000278572	ENSP00000355471
ENSP00000278572	ENSP00000356134
ENSP00000278572	ENSP00000356548
ENSP00000278572	ENSP00000356811
ENSP00000278572	ENSP00000358140
ENSP00000278572	ENSP00000359345
ENSP00000278572	ENSP00000361076
ENSP00000278572	ENSP00000361465
ENSP00000278572	ENSP00000362036
ENSP00000278572	ENSP00000362576
ENSP00000278572	ENSP00000362744
ENSP00000278572	ENSP00000363018
ENSP00000278572	ENSP00000363624
ENSP00000278572	ENSP00000363676
ENSP00000278572	ENSP00000363921
ENSP00000278572	ENSP00000364320
ENSP00000278572	ENSP00000364893
ENSP00000278572	ENSP00000365830
ENSP00000278572	ENSP00000365837
ENSP00000278572	ENSP00000367024
ENSP00000278572	ENSP00000368646
ENSP00000278572	ENSP00000368790
ENSP00000278572	ENSP00000368927
ENSP00000278572	ENSP00000369757
ENSP00000278572	ENSP00000371236
ENSP00000278572	ENSP00000372199
ENSP00000278572	ENSP00000373404
ENSP00000278572	ENSP00000375730
ENSP00000278572	ENSP00000376611

ENSP00000278572	ENSP00000379339
ENSP00000278572	ENSP00000379607
ENSP00000278572	ENSP00000379888
ENSP00000278572	ENSP00000385903
ENSP00000278572	ENSP00000389103
ENSP00000278572	ENSP00000392859
ENSP00000278572	ENSP00000393241
ENSP00000278572	ENSP00000401371
ENSP00000278572	ENSP00000403175
ENSP00000278572	ENSP00000412583
ENSP00000278572	ENSP00000414321
ENSP00000278572	ENSP00000416110
ENSP00000278572	ENSP00000416892
ENSP00000278572	ENSP00000417281
ENSP00000278572	ENSP00000417763
ENSP00000278572	ENSP00000420714
ENSP00000278612	ENSP00000305958
ENSP00000278612	ENSP00000318085
ENSP00000278612	ENSP00000347733
ENSP00000278616	ENSP00000279230
ENSP00000278616	ENSP00000280362
ENSP00000278616	ENSP00000280614
ENSP00000278616	ENSP00000283943
ENSP00000278616	ENSP00000285021
ENSP00000278616	ENSP00000287598
ENSP00000278616	ENSP00000287647
ENSP00000278616	ENSP00000290330
ENSP00000278616	ENSP00000294168
ENSP00000278616	ENSP00000295897
ENSP00000278616	ENSP00000295924
ENSP00000278616	ENSP00000296509
ENSP00000278616	ENSP00000296550
ENSP00000278616	ENSP00000297338
ENSP00000278616	ENSP00000298139
ENSP00000278616	ENSP00000298552
ENSP00000278616	ENSP00000299575
ENSP00000278616	ENSP00000300651
ENSP00000278616	ENSP00000300738
ENSP00000278616	ENSP00000301019
ENSP00000278616	ENSP00000301727
ENSP00000278616	ENSP00000301764
ENSP00000278616	ENSP00000302239
ENSP00000278616	ENSP00000302530
ENSP00000278616	ENSP00000302543
ENSP00000278616	ENSP00000307208
ENSP00000278616	ENSP00000307684

ENSP00000278616	ENSP00000309572
ENSP00000278616	ENSP00000310842
ENSP00000278616	ENSP00000311344
ENSP00000278616	ENSP00000311873
ENSP00000278616	ENSP00000313420
ENSP00000278616	ENSP00000323050
ENSP00000278616	ENSP00000323099
ENSP00000278616	ENSP00000323439
ENSP00000278616	ENSP00000325677
ENSP00000278616	ENSP00000325863
ENSP00000278616	ENSP00000329466
ENSP00000278616	ENSP00000330408
ENSP00000278616	ENSP00000334474
ENSP00000278616	ENSP00000336842
ENSP00000278616	ENSP00000336868
ENSP00000278616	ENSP00000337127
ENSP00000278616	ENSP00000337353
ENSP00000278616	ENSP00000339299
ENSP00000278616	ENSP00000339527
ENSP00000278616	ENSP00000340766
ENSP00000278616	ENSP00000340879
ENSP00000278616	ENSP00000342889
ENSP00000278616	ENSP00000343741
ENSP00000278616	ENSP00000344537
ENSP00000278616	ENSP00000344741
ENSP00000278616	ENSP00000345988
ENSP00000278616	ENSP00000346566
ENSP00000278616	ENSP00000347168
ENSP00000278616	ENSP00000347232
ENSP00000278616	ENSP00000348069
ENSP00000278616	ENSP00000348573
ENSP00000278616	ENSP00000348784
ENSP00000278616	ENSP00000349205
ENSP00000278616	ENSP00000349213
ENSP00000278616	ENSP00000349393
ENSP00000278616	ENSP00000350199
ENSP00000278616	ENSP00000350283
ENSP00000278616	ENSP00000350491
ENSP00000278616	ENSP00000350767
ENSP00000278616	ENSP00000350785
ENSP00000278616	ENSP00000351284
ENSP00000278616	ENSP00000351947
ENSP00000278616	ENSP00000352257
ENSP00000278616	ENSP00000352572
ENSP00000278616	ENSP00000353622
ENSP00000278616	ENSP00000353793

ENSP00000278616	ENSP00000353971
ENSP00000278616	ENSP00000354522
ENSP00000278616	ENSP00000354720
ENSP00000278616	ENSP00000354927
ENSP00000278616	ENSP00000355185
ENSP00000278616	ENSP00000355261
ENSP00000278616	ENSP00000355759
ENSP00000278616	ENSP00000355922
ENSP00000278616	ENSP00000356562
ENSP00000278616	ENSP00000358335
ENSP00000278616	ENSP00000358423
ENSP00000278616	ENSP00000360310
ENSP00000278616	ENSP00000362135
ENSP00000278616	ENSP00000362166
ENSP00000278616	ENSP00000362546
ENSP00000278616	ENSP00000362578
ENSP00000278616	ENSP00000363021
ENSP00000278616	ENSP00000364270
ENSP00000278616	ENSP00000364893
ENSP00000278616	ENSP00000365588
ENSP00000278616	ENSP00000365891
ENSP00000278616	ENSP00000366565
ENSP00000278616	ENSP00000366974
ENSP00000278616	ENSP00000367034
ENSP00000278616	ENSP00000367527
ENSP00000278616	ENSP00000368305
ENSP00000278616	ENSP00000368438
ENSP00000278616	ENSP00000369038
ENSP00000278616	ENSP00000369335
ENSP00000278616	ENSP00000369411
ENSP00000278616	ENSP00000369497
ENSP00000278616	ENSP00000370151
ENSP00000278616	ENSP00000371475
ENSP00000278616	ENSP00000372023
ENSP00000278616	ENSP00000373060
ENSP00000278616	ENSP00000373474
ENSP00000278616	ENSP00000375863
ENSP00000278616	ENSP00000377384
ENSP00000278616	ENSP00000380504
ENSP00000278616	ENSP00000381577
ENSP00000278616	ENSP00000382423
ENSP00000278616	ENSP00000384048
ENSP00000278616	ENSP00000407401
ENSP00000278616	ENSP00000409167
ENSP00000278616	ENSP00000417281
ENSP00000278618	ENSP00000294008

ENSP00000278618	ENSP00000296490
ENSP00000278618	ENSP00000304592
ENSP00000278618	ENSP00000346874
ENSP00000278618	ENSP00000352408
ENSP00000278618	ENSP00000355384
ENSP00000278618	ENSP00000360286
ENSP00000278671	ENSP00000357288
ENSP00000278671	ENSP00000358423
ENSP00000278715	ENSP00000314949
ENSP00000278715	ENSP00000357775
ENSP00000278715	ENSP00000386284
ENSP00000278742	ENSP00000296129
ENSP00000278742	ENSP00000342098
ENSP00000278780	ENSP00000295373
ENSP00000278780	ENSP00000344456
ENSP00000278780	ENSP00000356520
ENSP00000278780	ENSP00000358404
ENSP00000278780	ENSP00000392601
ENSP00000278856	ENSP00000296802
ENSP00000278856	ENSP00000302886
ENSP00000278856	ENSP00000305702
ENSP00000278856	ENSP00000311430
ENSP00000278856	ENSP00000314348
ENSP00000278856	ENSP00000317992
ENSP00000278856	ENSP00000323858
ENSP00000278856	ENSP00000328690
ENSP00000278856	ENSP00000338573
ENSP00000278856	ENSP00000338862
ENSP00000278856	ENSP00000339145
ENSP00000278856	ENSP00000339795
ENSP00000278856	ENSP00000346725
ENSP00000278856	ENSP00000349393
ENSP00000278856	ENSP00000352021
ENSP00000278856	ENSP00000353246
ENSP00000278856	ENSP00000354040
ENSP00000278856	ENSP00000355899
ENSP00000278856	ENSP00000356476
ENSP00000278856	ENSP00000359345
ENSP00000278856	ENSP00000359688
ENSP00000278856	ENSP00000360412
ENSP00000278856	ENSP00000360828
ENSP00000278856	ENSP00000361076
ENSP00000278856	ENSP00000361092
ENSP00000278856	ENSP00000362344
ENSP00000278856	ENSP00000363559
ENSP00000278856	ENSP00000364320

ENSP00000278856	ENSP00000366519
ENSP00000278856	ENSP00000368887
ENSP00000278856	ENSP00000377640
ENSP00000278856	ENSP00000377865
ENSP00000278856	ENSP00000380982
ENSP00000278856	ENSP00000382392
ENSP00000278856	ENSP00000389103
ENSP00000278856	ENSP00000402338
ENSP00000278856	ENSP00000406490
ENSP00000278865	ENSP00000335357
ENSP00000278886	ENSP00000300087
ENSP00000278886	ENSP00000300417
ENSP00000278886	ENSP00000300850
ENSP00000278886	ENSP00000303315
ENSP00000278886	ENSP00000309189
ENSP00000278886	ENSP00000314004
ENSP00000278886	ENSP00000318821
ENSP00000278886	ENSP00000324444
ENSP00000278886	ENSP00000324827
ENSP00000278886	ENSP00000347538
ENSP00000278886	ENSP00000354270
ENSP00000278886	ENSP00000355944
ENSP00000278886	ENSP00000358605
ENSP00000278886	ENSP00000364126
ENSP00000278886	ENSP00000368017
ENSP00000278886	ENSP00000377944
ENSP00000278886	ENSP00000378529
ENSP00000278886	ENSP00000381031
ENSP00000278886	ENSP00000381795
ENSP00000278886	ENSP00000382562
ENSP00000278886	ENSP00000395574
ENSP00000278888	ENSP00000340944
ENSP00000278888	ENSP00000364898
ENSP00000278919	ENSP00000297440
ENSP00000278919	ENSP00000307850
ENSP00000278919	ENSP00000315693
ENSP00000278919	ENSP00000333873
ENSP00000278919	ENSP00000341170
ENSP00000278919	ENSP00000343479
ENSP00000278919	ENSP00000345262
ENSP00000278919	ENSP00000347184
ENSP00000278919	ENSP00000351832
ENSP00000278919	ENSP00000354720
ENSP00000278919	ENSP00000355022
ENSP00000278919	ENSP00000355596
ENSP00000278919	ENSP00000358778

ENSP00000278919	ENSP00000365962
ENSP00000278919	ENSP00000375082
ENSP00000278919	ENSP00000377969
ENSP00000278919	ENSP00000387262
ENSP00000278919	ENSP00000408020
ENSP00000278935	ENSP00000323099
ENSP00000278935	ENSP00000343741
ENSP00000278937	ENSP00000336790
ENSP00000278937	ENSP00000341637
ENSP00000278980	ENSP00000308236
ENSP00000279036	ENSP00000309430
ENSP00000279036	ENSP00000337478
ENSP00000279036	ENSP00000347206
ENSP00000279036	ENSP00000355601
ENSP00000279036	ENSP00000359848
ENSP00000279036	ENSP00000404247
ENSP00000279147	ENSP00000296577
ENSP00000279147	ENSP00000395777
ENSP00000279230	ENSP00000282884
ENSP00000279230	ENSP00000295065
ENSP00000279230	ENSP00000320340
ENSP00000279230	ENSP00000322218
ENSP00000279230	ENSP00000339381
ENSP00000279230	ENSP00000344668
ENSP00000279230	ENSP00000347978
ENSP00000279230	ENSP00000352121
ENSP00000279230	ENSP00000354558
ENSP00000279230	ENSP00000360141
ENSP00000279230	ENSP00000361141
ENSP00000279230	ENSP00000363921
ENSP00000279230	ENSP00000368349
ENSP00000279230	ENSP00000370410
ENSP00000279230	ENSP00000373399
ENSP00000279242	ENSP00000288937
ENSP00000279242	ENSP00000300151
ENSP00000279242	ENSP00000301585
ENSP00000279242	ENSP00000306548
ENSP00000279242	ENSP00000308275
ENSP00000279242	ENSP00000312311
ENSP00000279242	ENSP00000315017
ENSP00000279242	ENSP00000333401
ENSP00000279242	ENSP00000354125
ENSP00000279242	ENSP00000357048
ENSP00000279242	ENSP00000370930
ENSP00000279242	ENSP00000377486
ENSP00000279242	ENSP00000379607

ENSP00000279242	ENSP00000417602
ENSP00000279281	ENSP00000339245
ENSP00000279281	ENSP00000353332
ENSP00000279281	ENSP00000360532
ENSP00000279392	ENSP00000311505
ENSP00000279804	ENSP00000338799
ENSP00000279873	ENSP00000303315
ENSP00000279873	ENSP00000307684
ENSP00000279873	ENSP00000310649
ENSP00000279873	ENSP00000323720
ENSP00000280057	ENSP00000360464
ENSP00000280057	ENSP00000366829
ENSP00000280082	ENSP00000313829
ENSP00000280083	ENSP00000295897
ENSP00000280083	ENSP00000303315
ENSP00000280097	ENSP00000350249
ENSP00000280098	ENSP00000296783
ENSP00000280098	ENSP00000309175
ENSP00000280098	ENSP00000313691
ENSP00000280098	ENSP00000336856
ENSP00000280098	ENSP00000354586
ENSP00000280098	ENSP00000370408
ENSP00000280098	ENSP00000370421
ENSP00000280098	ENSP00000384109
ENSP00000280154	ENSP00000316879
ENSP00000280154	ENSP00000340281
ENSP00000280154	ENSP00000346037
ENSP00000280154	ENSP00000359206
ENSP00000280154	ENSP00000359345
ENSP00000280154	ENSP00000379607
ENSP00000280187	ENSP00000315693
ENSP00000280190	ENSP00000283195
ENSP00000280190	ENSP00000290158
ENSP00000280190	ENSP00000293777
ENSP00000280190	ENSP00000296755
ENSP00000280190	ENSP00000297338
ENSP00000280190	ENSP00000300231
ENSP00000280190	ENSP00000304593
ENSP00000280190	ENSP00000308699
ENSP00000280190	ENSP00000315791
ENSP00000280190	ENSP00000323302
ENSP00000280190	ENSP00000323720
ENSP00000280190	ENSP00000326128
ENSP00000280190	ENSP00000334188
ENSP00000280190	ENSP00000336842
ENSP00000280190	ENSP00000356405

ENSP00000280190	ENSP00000356989
ENSP00000280190	ENSP00000357887
ENSP00000280190	ENSP00000360076
ENSP00000280190	ENSP00000362948
ENSP00000280190	ENSP00000363313
ENSP00000280190	ENSP00000363734
ENSP00000280190	ENSP00000372694
ENSP00000280190	ENSP00000374213
ENSP00000280190	ENSP00000386759
ENSP00000280190	ENSP00000391774
ENSP00000280190	ENSP00000412319
ENSP00000280190	ENSP00000415678
ENSP00000280191	ENSP00000352708
ENSP00000280236	ENSP00000351327
ENSP00000280346	ENSP00000282077
ENSP00000280346	ENSP00000284320
ENSP00000280346	ENSP00000295119
ENSP00000280346	ENSP00000299601
ENSP00000280346	ENSP00000300107
ENSP00000280346	ENSP00000304370
ENSP00000280346	ENSP00000308610
ENSP00000280346	ENSP00000315693
ENSP00000280346	ENSP00000327070
ENSP00000280346	ENSP00000329466
ENSP00000280346	ENSP00000334148
ENSP00000280346	ENSP00000335304
ENSP00000280346	ENSP00000342056
ENSP00000280346	ENSP00000345282
ENSP00000280346	ENSP00000367637
ENSP00000280346	ENSP00000367923
ENSP00000280346	ENSP00000370377
ENSP00000280346	ENSP00000379500
ENSP00000280346	ENSP00000379607
ENSP00000280346	ENSP00000387262
ENSP00000280346	ENSP00000388872
ENSP00000280346	ENSP00000389934
ENSP00000280346	ENSP00000394382
ENSP00000280346	ENSP00000417190
ENSP00000280350	ENSP00000323275
ENSP00000280350	ENSP00000323424
ENSP00000280350	ENSP00000324122
ENSP00000280350	ENSP00000333018
ENSP00000280350	ENSP00000350011
ENSP00000280350	ENSP00000355621
ENSP00000280350	ENSP00000357220
ENSP00000280350	ENSP00000360497

ENSP00000280350	ENSP00000360876
ENSP00000280350	ENSP00000361010
ENSP00000280350	ENSP00000362386
ENSP00000280350	ENSP00000364298
ENSP00000280350	ENSP00000366135
ENSP00000280350	ENSP00000397530
ENSP00000280350	ENSP00000398812
ENSP00000280350	ENSP00000412439
ENSP00000280357	ENSP00000367408
ENSP00000280362	ENSP00000281171
ENSP00000280362	ENSP00000283228
ENSP00000280362	ENSP00000292476
ENSP00000280362	ENSP00000303554
ENSP00000280362	ENSP00000313420
ENSP00000280362	ENSP00000315931
ENSP00000280362	ENSP00000340944
ENSP00000280362	ENSP00000343741
ENSP00000280362	ENSP00000348395
ENSP00000280362	ENSP00000349213
ENSP00000280362	ENSP00000349932
ENSP00000280362	ENSP00000355923
ENSP00000280362	ENSP00000356346
ENSP00000280362	ENSP00000362306
ENSP00000280362	ENSP00000368646
ENSP00000280362	ENSP00000374069
ENSP00000280362	ENSP00000377047
ENSP00000280362	ENSP00000379933
ENSP00000280362	ENSP00000380336
ENSP00000280362	ENSP00000394794
ENSP00000280527	ENSP00000283147
ENSP00000280527	ENSP00000340896
ENSP00000280527	ENSP00000345395
ENSP00000280527	ENSP00000350215
ENSP00000280527	ENSP00000355580
ENSP00000280527	ENSP00000357484
ENSP00000280527	ENSP00000362778
ENSP00000280527	ENSP00000382323
ENSP00000280557	ENSP00000283875
ENSP00000280557	ENSP00000288937
ENSP00000280557	ENSP00000296577
ENSP00000280557	ENSP00000296674
ENSP00000280557	ENSP00000296802
ENSP00000280557	ENSP00000301364
ENSP00000280557	ENSP00000302896
ENSP00000280557	ENSP00000310129
ENSP00000280557	ENSP00000311028

ENSP00000280557	ENSP00000316879
ENSP00000280557	ENSP00000317376
ENSP00000280557	ENSP00000318646
ENSP00000280557	ENSP00000323856
ENSP00000280557	ENSP00000326981
ENSP00000280557	ENSP00000331019
ENSP00000280557	ENSP00000336702
ENSP00000280557	ENSP00000339095
ENSP00000280557	ENSP00000340281
ENSP00000280557	ENSP00000341885
ENSP00000280557	ENSP00000346050
ENSP00000280557	ENSP00000346067
ENSP00000280557	ENSP00000351813
ENSP00000280557	ENSP00000354125
ENSP00000280557	ENSP00000358140
ENSP00000280557	ENSP00000361686
ENSP00000280557	ENSP00000362744
ENSP00000280557	ENSP00000363308
ENSP00000280557	ENSP00000364119
ENSP00000280557	ENSP00000364320
ENSP00000280557	ENSP00000368927
ENSP00000280557	ENSP00000369757
ENSP00000280557	ENSP00000370258
ENSP00000280557	ENSP00000379339
ENSP00000280557	ENSP00000379888
ENSP00000280557	ENSP00000393241
ENSP00000280557	ENSP00000396937
ENSP00000280557	ENSP00000403175
ENSP00000280557	ENSP00000412583
ENSP00000280557	ENSP00000414321
ENSP00000280557	ENSP00000416110
ENSP00000280557	ENSP00000417229
ENSP00000280557	ENSP00000420714
ENSP00000280605	ENSP00000301729
ENSP00000280605	ENSP00000305958
ENSP00000280605	ENSP00000321259
ENSP00000280605	ENSP00000336752
ENSP00000280605	ENSP00000348395
ENSP00000280605	ENSP00000366620
ENSP00000280605	ENSP00000368438
ENSP00000280614	ENSP00000296328
ENSP00000280614	ENSP00000296509
ENSP00000280614	ENSP00000310596
ENSP00000280614	ENSP00000313171
ENSP00000280614	ENSP00000314949
ENSP00000280614	ENSP00000319104

ENSP00000280614	ENSP00000341743
ENSP00000280614	ENSP00000343741
ENSP00000280614	ENSP00000351284
ENSP00000280614	ENSP00000354673
ENSP00000280614	ENSP00000354850
ENSP00000280614	ENSP00000356405
ENSP00000280614	ENSP00000360515
ENSP00000280614	ENSP00000368438
ENSP00000280614	ENSP00000369965
ENSP00000280614	ENSP00000372023
ENSP00000280665	ENSP00000285896
ENSP00000280665	ENSP00000298832
ENSP00000280665	ENSP00000300146
ENSP00000280665	ENSP00000300413
ENSP00000280665	ENSP00000302160
ENSP00000280665	ENSP00000310596
ENSP00000280665	ENSP00000318195
ENSP00000280665	ENSP00000319341
ENSP00000280665	ENSP00000320503
ENSP00000280665	ENSP00000322419
ENSP00000280665	ENSP00000340766
ENSP00000280665	ENSP00000341551
ENSP00000280665	ENSP00000341848
ENSP00000280665	ENSP00000344989
ENSP00000280665	ENSP00000346015
ENSP00000280665	ENSP00000348573
ENSP00000280665	ENSP00000348708
ENSP00000280665	ENSP00000351596
ENSP00000280665	ENSP00000351811
ENSP00000280665	ENSP00000352572
ENSP00000280665	ENSP00000354486
ENSP00000280665	ENSP00000355261
ENSP00000280665	ENSP00000355922
ENSP00000280665	ENSP00000356811
ENSP00000280665	ENSP00000359939
ENSP00000280665	ENSP00000361949
ENSP00000280665	ENSP00000364813
ENSP00000280665	ENSP00000366843
ENSP00000280665	ENSP00000368591
ENSP00000280665	ENSP00000372883
ENSP00000280665	ENSP00000373272
ENSP00000280665	ENSP00000373715
ENSP00000280665	ENSP00000379144
ENSP00000280665	ENSP00000382595
ENSP00000280665	ENSP00000394400
ENSP00000280665	ENSP00000398597

ENSP00000280665	ENSP00000403345
ENSP00000280665	ENSP00000406280
ENSP00000280665	ENSP00000410758
ENSP00000280665	ENSP00000414006
ENSP00000280665	ENSP00000414634
ENSP00000280700	ENSP00000283290
ENSP00000280700	ENSP00000290649
ENSP00000280700	ENSP00000290663
ENSP00000280700	ENSP00000290810
ENSP00000280700	ENSP00000294119
ENSP00000280700	ENSP00000298852
ENSP00000280700	ENSP00000305465
ENSP00000280700	ENSP00000324560
ENSP00000280700	ENSP00000333950
ENSP00000280700	ENSP00000334044
ENSP00000280700	ENSP00000339484
ENSP00000280700	ENSP00000340836
ENSP00000280700	ENSP00000346437
ENSP00000280700	ENSP00000348565
ENSP00000280700	ENSP00000349785
ENSP00000280700	ENSP00000350708
ENSP00000280700	ENSP00000354340
ENSP00000280700	ENSP00000359067
ENSP00000280700	ENSP00000366249
ENSP00000280700	ENSP00000366395
ENSP00000280700	ENSP00000368699
ENSP00000280700	ENSP00000373039
ENSP00000280700	ENSP00000379457
ENSP00000280700	ENSP00000396152
ENSP00000280700	ENSP00000400312
ENSP00000280700	ENSP00000410416
ENSP00000280701	ENSP00000287675
ENSP00000280701	ENSP00000290429
ENSP00000280701	ENSP00000292475
ENSP00000280701	ENSP00000300107
ENSP00000280701	ENSP00000301587
ENSP00000280701	ENSP00000304102
ENSP00000280701	ENSP00000305682
ENSP00000280701	ENSP00000310040
ENSP00000280701	ENSP00000337445
ENSP00000280701	ENSP00000350512
ENSP00000280701	ENSP00000358737
ENSP00000280701	ENSP00000360992
ENSP00000280701	ENSP00000365773
ENSP00000280701	ENSP00000370414
ENSP00000280701	ENSP00000389649

ENSP00000280701	ENSP00000404923
ENSP00000280772	ENSP00000301838
ENSP00000280772	ENSP00000317790
ENSP00000280772	ENSP00000328968
ENSP00000280772	ENSP00000347979
ENSP00000280772	ENSP00000354560
ENSP00000280772	ENSP00000360252
ENSP00000280772	ENSP00000361824
ENSP00000280772	ENSP00000396915
ENSP00000280800	ENSP00000333298
ENSP00000280800	ENSP00000379607
ENSP00000280871	ENSP00000368119
ENSP00000280979	ENSP00000300574
ENSP00000280979	ENSP00000341680
ENSP00000280979	ENSP00000360532
ENSP00000280979	ENSP00000378306
ENSP00000281043	ENSP00000281708
ENSP00000281043	ENSP00000298552
ENSP00000281043	ENSP00000309555
ENSP00000281043	ENSP00000329357
ENSP00000281043	ENSP00000351490
ENSP00000281043	ENSP00000354558
ENSP00000281043	ENSP00000355153
ENSP00000281043	ENSP00000364524
ENSP00000281043	ENSP00000365735
ENSP00000281043	ENSP00000381499
ENSP00000281043	ENSP00000392028
ENSP00000281092	ENSP00000297689
ENSP00000281092	ENSP00000301843
ENSP00000281092	ENSP00000316460
ENSP00000281092	ENSP00000341208
ENSP00000281092	ENSP00000344456
ENSP00000281092	ENSP00000381293
ENSP00000281092	ENSP00000381567
ENSP00000281141	ENSP00000295951
ENSP00000281141	ENSP00000326981
ENSP00000281141	ENSP00000362578
ENSP00000281141	ENSP00000362688
ENSP00000281141	ENSP00000364119
ENSP00000281141	ENSP00000379933
ENSP00000281142	ENSP00000361027
ENSP00000281171	ENSP00000335306
ENSP00000281171	ENSP00000344220
ENSP00000281171	ENSP00000344456
ENSP00000281171	ENSP00000362608
ENSP00000281171	ENSP00000365891

ENSP00000281171	ENSP00000381293
ENSP00000281172	ENSP00000296218
ENSP00000281172	ENSP00000305790
ENSP00000281172	ENSP00000327077
ENSP00000281172	ENSP00000341082
ENSP00000281172	ENSP00000352047
ENSP00000281172	ENSP00000352257
ENSP00000281172	ENSP00000354566
ENSP00000281172	ENSP00000355296
ENSP00000281172	ENSP00000358770
ENSP00000281172	ENSP00000359837
ENSP00000281172	ENSP00000366936
ENSP00000281172	ENSP00000367408
ENSP00000281172	ENSP00000367440
ENSP00000281172	ENSP00000384675
ENSP00000281187	ENSP00000284957
ENSP00000281187	ENSP00000286835
ENSP00000281187	ENSP00000295767
ENSP00000281187	ENSP00000299138
ENSP00000281187	ENSP00000304292
ENSP00000281187	ENSP00000308496
ENSP00000281187	ENSP00000317332
ENSP00000281187	ENSP00000340836
ENSP00000281187	ENSP00000352175
ENSP00000281187	ENSP00000354522
ENSP00000281187	ENSP00000356146
ENSP00000281187	ENSP00000358715
ENSP00000281187	ENSP00000360183
ENSP00000281187	ENSP00000363349
ENSP00000281187	ENSP00000377665
ENSP00000281187	ENSP00000397441
ENSP00000281187	ENSP00000402935
ENSP00000281243	ENSP00000311505
ENSP00000281273	ENSP00000295897
ENSP00000281273	ENSP00000339532
ENSP00000281273	ENSP00000369871
ENSP00000281282	ENSP00000292535
ENSP00000281282	ENSP00000294543
ENSP00000281282	ENSP00000313059
ENSP00000281282	ENSP00000318884
ENSP00000281282	ENSP00000322608
ENSP00000281282	ENSP00000333950
ENSP00000281282	ENSP00000360076
ENSP00000281282	ENSP00000369871
ENSP00000281282	ENSP00000376410
ENSP00000281282	ENSP00000391249

ENSP00000281282	ENSP00000397351
ENSP00000281282	ENSP00000397598
ENSP00000281282	ENSP00000407487
ENSP00000281382	ENSP00000326869
ENSP00000281382	ENSP00000415203
ENSP00000281437	ENSP00000303315
ENSP00000281437	ENSP00000306245
ENSP00000281437	ENSP00000350359
ENSP00000281537	ENSP00000299106
ENSP00000281537	ENSP00000301843
ENSP00000281537	ENSP00000304604
ENSP00000281537	ENSP00000313829
ENSP00000281537	ENSP00000323856
ENSP00000281537	ENSP00000329466
ENSP00000281537	ENSP00000346550
ENSP00000281537	ENSP00000347379
ENSP00000281537	ENSP00000355923
ENSP00000281537	ENSP00000360286
ENSP00000281537	ENSP00000361824
ENSP00000281537	ENSP00000367972
ENSP00000281537	ENSP00000368797
ENSP00000281537	ENSP00000368824
ENSP00000281537	ENSP00000370410
ENSP00000281537	ENSP00000383623
ENSP00000281543	ENSP00000295890
ENSP00000281543	ENSP00000296674
ENSP00000281543	ENSP00000308897
ENSP00000281543	ENSP00000310520
ENSP00000281543	ENSP00000318977
ENSP00000281543	ENSP00000354673
ENSP00000281543	ENSP00000354739
ENSP00000281623	ENSP00000311280
ENSP00000281708	ENSP00000290810
ENSP00000281708	ENSP00000296930
ENSP00000281708	ENSP00000303315
ENSP00000281708	ENSP00000306788
ENSP00000281708	ENSP00000310129
ENSP00000281708	ENSP00000310701
ENSP00000281708	ENSP00000312411
ENSP00000281708	ENSP00000320648
ENSP00000281708	ENSP00000320768
ENSP00000281708	ENSP00000326366
ENSP00000281708	ENSP00000337501
ENSP00000281708	ENSP00000339529
ENSP00000281708	ENSP00000347451
ENSP00000281708	ENSP00000348069

ENSP00000281708	ENSP00000348554
ENSP00000281708	ENSP00000350512
ENSP00000281708	ENSP00000353793
ENSP00000281708	ENSP00000354558
ENSP00000281708	ENSP00000355865
ENSP00000281708	ENSP00000358022
ENSP00000281708	ENSP00000358140
ENSP00000281708	ENSP00000362413
ENSP00000281708	ENSP00000366915
ENSP00000281708	ENSP00000367208
ENSP00000281708	ENSP00000367527
ENSP00000281708	ENSP00000368805
ENSP00000281708	ENSP00000369757
ENSP00000281708	ENSP00000370151
ENSP00000281708	ENSP00000378350
ENSP00000281708	ENSP00000378529
ENSP00000281708	ENSP00000384109
ENSP00000281708	ENSP00000417281
ENSP00000281708	ENSP00000417748
ENSP00000281828	ENSP00000282276
ENSP00000281828	ENSP00000289371
ENSP00000281828	ENSP00000294008
ENSP00000281828	ENSP00000302114
ENSP00000281828	ENSP00000303279
ENSP00000281828	ENSP00000311766
ENSP00000281828	ENSP00000317334
ENSP00000281828	ENSP00000320309
ENSP00000281828	ENSP00000340019
ENSP00000281828	ENSP00000340093
ENSP00000281828	ENSP00000348565
ENSP00000281828	ENSP00000348886
ENSP00000281828	ENSP00000351380
ENSP00000281828	ENSP00000352928
ENSP00000281828	ENSP00000364794
ENSP00000281828	ENSP00000366395
ENSP00000281828	ENSP00000368140
ENSP00000281828	ENSP00000370083
ENSP00000281828	ENSP00000370119
ENSP00000281828	ENSP00000370377
ENSP00000281828	ENSP00000370607
ENSP00000281828	ENSP00000376946
ENSP00000281834	ENSP00000368538
ENSP00000281928	ENSP00000286317
ENSP00000281928	ENSP00000290663
ENSP00000281928	ENSP00000293777
ENSP00000281928	ENSP00000302327

ENSP00000281928	ENSP00000323720
ENSP00000281928	ENSP00000325612
ENSP00000281928	ENSP00000326767
ENSP00000281928	ENSP00000334474
ENSP00000281928	ENSP00000337340
ENSP00000281928	ENSP00000357047
ENSP00000281928	ENSP00000357442
ENSP00000281928	ENSP00000357494
ENSP00000281928	ENSP00000357907
ENSP00000281928	ENSP00000365588
ENSP00000281928	ENSP00000373060
ENSP00000281928	ENSP00000384048
ENSP00000281928	ENSP00000387429
ENSP00000281928	ENSP00000391774
ENSP00000281928	ENSP00000396484
ENSP00000281928	ENSP00000404318
ENSP00000281928	ENSP00000409167
ENSP00000281928	ENSP00000415212
ENSP00000281928	ENSP00000416511
ENSP00000281950	ENSP00000300413
ENSP00000281950	ENSP00000308533
ENSP00000281950	ENSP00000321706
ENSP00000281950	ENSP00000328940
ENSP00000281950	ENSP00000342374
ENSP00000281950	ENSP00000358716
ENSP00000281950	ENSP00000360532
ENSP00000281950	ENSP00000370083
ENSP00000281950	ENSP00000370119
ENSP00000281950	ENSP00000392270
ENSP00000281950	ENSP00000400591
ENSP00000281950	ENSP00000412566
ENSP00000282003	ENSP00000360329
ENSP00000282007	ENSP00000282892
ENSP00000282007	ENSP00000285398
ENSP00000282007	ENSP00000285873
ENSP00000282007	ENSP00000290158
ENSP00000282007	ENSP00000290663
ENSP00000282007	ENSP00000291688
ENSP00000282007	ENSP00000292035
ENSP00000282007	ENSP00000297347
ENSP00000282007	ENSP00000298281
ENSP00000282007	ENSP00000300651
ENSP00000282007	ENSP00000308944
ENSP00000282007	ENSP00000312735
ENSP00000282007	ENSP00000314949
ENSP00000282007	ENSP00000315791

ENSP00000282007	ENSP00000323720
ENSP00000282007	ENSP00000323858
ENSP00000282007	ENSP00000325612
ENSP00000282007	ENSP00000326767
ENSP00000282007	ENSP00000329466
ENSP00000282007	ENSP00000331817
ENSP00000282007	ENSP00000336752
ENSP00000282007	ENSP00000336868
ENSP00000282007	ENSP00000337340
ENSP00000282007	ENSP00000343701
ENSP00000282007	ENSP00000343741
ENSP00000282007	ENSP00000348610
ENSP00000282007	ENSP00000352738
ENSP00000282007	ENSP00000352820
ENSP00000282007	ENSP00000353059
ENSP00000282007	ENSP00000356470
ENSP00000282007	ENSP00000357047
ENSP00000282007	ENSP00000357907
ENSP00000282007	ENSP00000362900
ENSP00000282007	ENSP00000363193
ENSP00000282007	ENSP00000364289
ENSP00000282007	ENSP00000365811
ENSP00000282007	ENSP00000366953
ENSP00000282007	ENSP00000372023
ENSP00000282007	ENSP00000417132
ENSP00000282030	ENSP00000307208
ENSP00000282030	ENSP00000323074
ENSP00000282030	ENSP00000329097
ENSP00000282030	ENSP00000352463
ENSP00000282030	ENSP00000367570
ENSP00000282030	ENSP00000368438
ENSP00000282032	ENSP00000326477
ENSP00000282032	ENSP00000357965
ENSP00000282050	ENSP00000284727
ENSP00000282050	ENSP00000287038
ENSP00000282050	ENSP00000290299
ENSP00000282050	ENSP00000290541
ENSP00000282050	ENSP00000292475
ENSP00000282050	ENSP00000296255
ENSP00000282050	ENSP00000299767
ENSP00000282050	ENSP00000300688
ENSP00000282050	ENSP00000301585
ENSP00000282050	ENSP00000301587
ENSP00000282050	ENSP00000306003
ENSP00000282050	ENSP00000309365
ENSP00000282050	ENSP00000311245

ENSP00000282050	ENSP00000311505
ENSP00000282050	ENSP00000311766
ENSP00000282050	ENSP00000317331
ENSP00000282050	ENSP00000319992
ENSP00000282050	ENSP00000325562
ENSP00000282050	ENSP00000327070
ENSP00000282050	ENSP00000331897
ENSP00000282050	ENSP00000332247
ENSP00000282050	ENSP00000332887
ENSP00000282050	ENSP00000335203
ENSP00000282050	ENSP00000341243
ENSP00000282050	ENSP00000343943
ENSP00000282050	ENSP00000346050
ENSP00000282050	ENSP00000349142
ENSP00000282050	ENSP00000350512
ENSP00000282050	ENSP00000352219
ENSP00000282050	ENSP00000354632
ENSP00000282050	ENSP00000355265
ENSP00000282050	ENSP00000355518
ENSP00000282050	ENSP00000356953
ENSP00000282050	ENSP00000358737
ENSP00000282050	ENSP00000360782
ENSP00000282050	ENSP00000361005
ENSP00000282050	ENSP00000361686
ENSP00000282050	ENSP00000363018
ENSP00000282050	ENSP00000363162
ENSP00000282050	ENSP00000363533
ENSP00000282050	ENSP00000364486
ENSP00000282050	ENSP00000365243
ENSP00000282050	ENSP00000367923
ENSP00000282050	ENSP00000367934
ENSP00000282050	ENSP00000369810
ENSP00000282050	ENSP00000369816
ENSP00000282050	ENSP00000370414
ENSP00000282050	ENSP00000374328
ENSP00000282050	ENSP00000379500
ENSP00000282050	ENSP00000379607
ENSP00000282050	ENSP00000380921
ENSP00000282050	ENSP00000387262
ENSP00000282050	ENSP00000389649
ENSP00000282050	ENSP00000393241
ENSP00000282050	ENSP00000393393
ENSP00000282050	ENSP00000401770
ENSP00000282050	ENSP00000403175
ENSP00000282050	ENSP00000412583
ENSP00000282050	ENSP00000416110

ENSP00000282050	ENSP00000417190
ENSP00000282058	ENSP00000301019
ENSP00000282058	ENSP00000360621
ENSP00000282058	ENSP00000369871
ENSP00000282074	ENSP00000302530
ENSP00000282074	ENSP00000306574
ENSP00000282074	ENSP00000363055
ENSP00000282074	ENSP00000370557
ENSP00000282077	ENSP00000288774
ENSP00000282077	ENSP00000302886
ENSP00000282077	ENSP00000307260
ENSP00000282077	ENSP00000307272
ENSP00000282077	ENSP00000307305
ENSP00000282077	ENSP00000313243
ENSP00000282077	ENSP00000322242
ENSP00000282077	ENSP00000344220
ENSP00000282077	ENSP00000348069
ENSP00000282077	ENSP00000348564
ENSP00000282077	ENSP00000354558
ENSP00000282077	ENSP00000357440
ENSP00000282077	ENSP00000359552
ENSP00000282077	ENSP00000361120
ENSP00000282077	ENSP00000361965
ENSP00000282077	ENSP00000380150
ENSP00000282077	ENSP00000394382
ENSP00000282091	ENSP00000321999
ENSP00000282185	ENSP00000283290
ENSP00000282185	ENSP00000296755
ENSP00000282185	ENSP00000343313
ENSP00000282185	ENSP00000343535
ENSP00000282185	ENSP00000346437
ENSP00000282185	ENSP00000349785
ENSP00000282251	ENSP00000296509
ENSP00000282251	ENSP00000298532
ENSP00000282251	ENSP00000304370
ENSP00000282251	ENSP00000334148
ENSP00000282251	ENSP00000345702
ENSP00000282251	ENSP00000360532
ENSP00000282276	ENSP00000294168
ENSP00000282276	ENSP00000303279
ENSP00000282276	ENSP00000306920
ENSP00000282276	ENSP00000307567
ENSP00000282276	ENSP00000318921
ENSP00000282276	ENSP00000327821
ENSP00000282276	ENSP00000337445
ENSP00000282276	ENSP00000339933

ENSP00000282276	ENSP00000344537
ENSP00000282276	ENSP00000355890
ENSP00000282276	ENSP00000358812
ENSP00000282276	ENSP00000360327
ENSP00000282276	ENSP00000362576
ENSP00000282276	ENSP00000364794
ENSP00000282276	ENSP00000367029
ENSP00000282276	ENSP00000369038
ENSP00000282276	ENSP00000373918
ENSP00000282276	ENSP00000374399
ENSP00000282326	ENSP00000282892
ENSP00000282326	ENSP00000283875
ENSP00000282326	ENSP00000285398
ENSP00000282326	ENSP00000286317
ENSP00000282326	ENSP00000287820
ENSP00000282326	ENSP00000299687
ENSP00000282326	ENSP00000303191
ENSP00000282326	ENSP00000306523
ENSP00000282326	ENSP00000312735
ENSP00000282326	ENSP00000340896
ENSP00000282326	ENSP00000342295
ENSP00000282326	ENSP00000358478
ENSP00000282326	ENSP00000360532
ENSP00000282326	ENSP00000398677
ENSP00000282388	ENSP00000283882
ENSP00000282388	ENSP00000322419
ENSP00000282388	ENSP00000335357
ENSP00000282388	ENSP00000343001
ENSP00000282388	ENSP00000350078
ENSP00000282388	ENSP00000351596
ENSP00000282388	ENSP00000354739
ENSP00000282388	ENSP00000354916
ENSP00000282388	ENSP00000355296
ENSP00000282388	ENSP00000359345
ENSP00000282388	ENSP00000364699
ENSP00000282388	ENSP00000365756
ENSP00000282412	ENSP00000301364
ENSP00000282412	ENSP00000311005
ENSP00000282412	ENSP00000315182
ENSP00000282412	ENSP00000349205
ENSP00000282412	ENSP00000350012
ENSP00000282412	ENSP00000353904
ENSP00000282412	ENSP00000355866
ENSP00000282412	ENSP00000356263
ENSP00000282412	ENSP00000358335
ENSP00000282412	ENSP00000360916

ENSP00000282412	ENSP00000361949
ENSP00000282412	ENSP00000365735
ENSP00000282412	ENSP00000368699
ENSP00000282412	ENSP00000370517
ENSP00000282412	ENSP00000378306
ENSP00000282412	ENSP00000378624
ENSP00000282412	ENSP00000381045
ENSP00000282412	ENSP00000401445
ENSP00000282441	ENSP00000288840
ENSP00000282441	ENSP00000295206
ENSP00000282441	ENSP00000301788
ENSP00000282441	ENSP00000307275
ENSP00000282441	ENSP00000310701
ENSP00000282441	ENSP00000318646
ENSP00000282441	ENSP00000324729
ENSP00000282441	ENSP00000339933
ENSP00000282441	ENSP00000341957
ENSP00000282441	ENSP00000344456
ENSP00000282441	ENSP00000352514
ENSP00000282441	ENSP00000352738
ENSP00000282441	ENSP00000355296
ENSP00000282441	ENSP00000355398
ENSP00000282441	ENSP00000356104
ENSP00000282441	ENSP00000359206
ENSP00000282441	ENSP00000361027
ENSP00000282441	ENSP00000363559
ENSP00000282441	ENSP00000366915
ENSP00000282441	ENSP00000383719
ENSP00000282441	ENSP00000408020
ENSP00000282470	ENSP00000355596
ENSP00000282486	ENSP00000284322
ENSP00000282486	ENSP00000303315
ENSP00000282486	ENSP00000356520
ENSP00000282486	ENSP00000365439
ENSP00000282486	ENSP00000396620
ENSP00000282516	ENSP00000296273
ENSP00000282516	ENSP00000296509
ENSP00000282516	ENSP00000297338
ENSP00000282516	ENSP00000299259
ENSP00000282516	ENSP00000306999
ENSP00000282516	ENSP00000308699
ENSP00000282516	ENSP00000313171
ENSP00000282516	ENSP00000313851
ENSP00000282516	ENSP00000318977
ENSP00000282516	ENSP00000322180
ENSP00000282516	ENSP00000329466

ENSP00000282516	ENSP00000334061
ENSP00000282516	ENSP00000343023
ENSP00000282516	ENSP00000347978
ENSP00000282516	ENSP00000349393
ENSP00000282516	ENSP00000353793
ENSP00000282516	ENSP00000354720
ENSP00000282516	ENSP00000355388
ENSP00000282516	ENSP00000357047
ENSP00000282516	ENSP00000360310
ENSP00000282516	ENSP00000360532
ENSP00000282516	ENSP00000361310
ENSP00000282516	ENSP00000361990
ENSP00000282516	ENSP00000364895
ENSP00000282516	ENSP00000365007
ENSP00000282516	ENSP00000367787
ENSP00000282516	ENSP00000369411
ENSP00000282516	ENSP00000369695
ENSP00000282516	ENSP00000370151
ENSP00000282516	ENSP00000372689
ENSP00000282516	ENSP00000376127
ENSP00000282516	ENSP00000382562
ENSP00000282516	ENSP00000391723
ENSP00000282516	ENSP00000404381
ENSP00000282541	ENSP00000290299
ENSP00000282541	ENSP00000292475
ENSP00000282541	ENSP00000296684
ENSP00000282541	ENSP00000308610
ENSP00000282541	ENSP00000317159
ENSP00000282541	ENSP00000318131
ENSP00000282541	ENSP00000320488
ENSP00000282541	ENSP00000324792
ENSP00000282541	ENSP00000330343
ENSP00000282541	ENSP00000336702
ENSP00000282541	ENSP00000341838
ENSP00000282541	ENSP00000353452
ENSP00000282541	ENSP00000355587
ENSP00000282541	ENSP00000358737
ENSP00000282541	ENSP00000362171
ENSP00000282541	ENSP00000363873
ENSP00000282541	ENSP00000364188
ENSP00000282541	ENSP00000368565
ENSP00000282541	ENSP00000375986
ENSP00000282541	ENSP00000397009
ENSP00000282541	ENSP00000397598
ENSP00000282541	ENSP00000417963
ENSP00000282549	ENSP00000310561

ENSP00000282549	ENSP00000361418
ENSP00000282549	ENSP00000365735
ENSP00000282549	ENSP00000377640
ENSP00000282572	ENSP00000287598
ENSP00000282572	ENSP00000296509
ENSP00000282572	ENSP00000302530
ENSP00000282572	ENSP00000306999
ENSP00000282572	ENSP00000307853
ENSP00000282572	ENSP00000310649
ENSP00000282572	ENSP00000343741
ENSP00000282572	ENSP00000351284
ENSP00000282572	ENSP00000352257
ENSP00000282572	ENSP00000353698
ENSP00000282572	ENSP00000360621
ENSP00000282572	ENSP00000360916
ENSP00000282572	ENSP00000363021
ENSP00000282572	ENSP00000363827
ENSP00000282572	ENSP00000368438
ENSP00000282572	ENSP00000370719
ENSP00000282572	ENSP00000372023
ENSP00000282572	ENSP00000377793
ENSP00000282587	ENSP00000350263
ENSP00000282587	ENSP00000353622
ENSP00000282587	ENSP00000372700
ENSP00000282611	ENSP00000311200
ENSP00000282633	ENSP00000402935
ENSP00000282728	ENSP00000306245
ENSP00000282728	ENSP00000345681
ENSP00000282728	ENSP00000350512
ENSP00000282841	ENSP00000283351
ENSP00000282841	ENSP00000287936
ENSP00000282841	ENSP00000295888
ENSP00000282841	ENSP00000322706
ENSP00000282841	ENSP00000333813
ENSP00000282841	ENSP00000333946
ENSP00000282841	ENSP00000341679
ENSP00000282841	ENSP00000349078
ENSP00000282841	ENSP00000349877
ENSP00000282841	ENSP00000355607
ENSP00000282841	ENSP00000356405
ENSP00000282841	ENSP00000360149
ENSP00000282841	ENSP00000365775
ENSP00000282841	ENSP00000366557
ENSP00000282841	ENSP00000374443
ENSP00000282841	ENSP00000376268
ENSP00000282841	ENSP00000380793

ENSP00000282841	ENSP00000381302
ENSP00000282841	ENSP00000383295
ENSP00000282841	ENSP00000386609
ENSP00000282841	ENSP00000415034
ENSP00000282884	ENSP00000283875
ENSP00000282884	ENSP00000286317
ENSP00000282884	ENSP00000298937
ENSP00000282884	ENSP00000301788
ENSP00000282884	ENSP00000323720
ENSP00000282884	ENSP00000343701
ENSP00000282884	ENSP00000345988
ENSP00000282884	ENSP00000348550
ENSP00000282884	ENSP00000350967
ENSP00000282884	ENSP00000356591
ENSP00000282884	ENSP00000357907
ENSP00000282884	ENSP00000358140
ENSP00000282884	ENSP00000359290
ENSP00000282884	ENSP00000363779
ENSP00000282884	ENSP00000363921
ENSP00000282884	ENSP00000364092
ENSP00000282892	ENSP00000283875
ENSP00000282892	ENSP00000286317
ENSP00000282892	ENSP00000293879
ENSP00000282892	ENSP00000296702
ENSP00000282892	ENSP00000296792
ENSP00000282892	ENSP00000297338
ENSP00000282892	ENSP00000297347
ENSP00000282892	ENSP00000299424
ENSP00000282892	ENSP00000299767
ENSP00000282892	ENSP00000300651
ENSP00000282892	ENSP00000303315
ENSP00000282892	ENSP00000306983
ENSP00000282892	ENSP00000307684
ENSP00000282892	ENSP00000311665
ENSP00000282892	ENSP00000314949
ENSP00000282892	ENSP00000318313
ENSP00000282892	ENSP00000322570
ENSP00000282892	ENSP00000323720
ENSP00000282892	ENSP00000326767
ENSP00000282892	ENSP00000337340
ENSP00000282892	ENSP00000339449
ENSP00000282892	ENSP00000348069
ENSP00000282892	ENSP00000348129
ENSP00000282892	ENSP00000350283
ENSP00000282892	ENSP00000351697
ENSP00000282892	ENSP00000356641

ENSP00000282892	ENSP00000356774
ENSP00000282892	ENSP00000357907
ENSP00000282892	ENSP00000359531
ENSP00000282892	ENSP00000360916
ENSP00000282892	ENSP00000362948
ENSP00000282892	ENSP00000363193
ENSP00000282892	ENSP00000368349
ENSP00000282892	ENSP00000368589
ENSP00000282892	ENSP00000369695
ENSP00000282892	ENSP00000369965
ENSP00000282892	ENSP00000377969
ENSP00000282892	ENSP00000395574
ENSP00000282999	ENSP00000294360
ENSP00000282999	ENSP00000300648
ENSP00000282999	ENSP00000305230
ENSP00000282999	ENSP00000312066
ENSP00000282999	ENSP00000314311
ENSP00000282999	ENSP00000327070
ENSP00000282999	ENSP00000336712
ENSP00000282999	ENSP00000342181
ENSP00000282999	ENSP00000343526
ENSP00000282999	ENSP00000343701
ENSP00000282999	ENSP00000362592
ENSP00000282999	ENSP00000369042
ENSP00000282999	ENSP00000369411
ENSP00000283006	ENSP00000296509
ENSP00000283006	ENSP00000336868
ENSP00000283006	ENSP00000357452
ENSP00000283006	ENSP00000364737
ENSP00000283006	ENSP00000377007
ENSP00000283006	ENSP00000420608
ENSP00000283027	ENSP00000309595
ENSP00000283027	ENSP00000351113
ENSP00000283033	ENSP00000292180
ENSP00000283033	ENSP00000297431
ENSP00000283033	ENSP00000299767
ENSP00000283033	ENSP00000309871
ENSP00000283033	ENSP00000317997
ENSP00000283033	ENSP00000321259
ENSP00000283033	ENSP00000331268
ENSP00000283033	ENSP00000340879
ENSP00000283033	ENSP00000341208
ENSP00000283033	ENSP00000382064
ENSP00000283109	ENSP00000285873
ENSP00000283109	ENSP00000293406
ENSP00000283109	ENSP00000296674

ENSP00000283109	ENSP00000296733
ENSP00000283109	ENSP00000296802
ENSP00000283109	ENSP00000297990
ENSP00000283109	ENSP00000301364
ENSP00000283109	ENSP00000302896
ENSP00000283109	ENSP00000308944
ENSP00000283109	ENSP00000311028
ENSP00000283109	ENSP00000314311
ENSP00000283109	ENSP00000314348
ENSP00000283109	ENSP00000317334
ENSP00000283109	ENSP00000317376
ENSP00000283109	ENSP00000318646
ENSP00000283109	ENSP00000323858
ENSP00000283109	ENSP00000326981
ENSP00000283109	ENSP00000331019
ENSP00000283109	ENSP00000337839
ENSP00000283109	ENSP00000339095
ENSP00000283109	ENSP00000341885
ENSP00000283109	ENSP00000342381
ENSP00000283109	ENSP00000346050
ENSP00000283109	ENSP00000351947
ENSP00000283109	ENSP00000352738
ENSP00000283109	ENSP00000354125
ENSP00000283109	ENSP00000355471
ENSP00000283109	ENSP00000355899
ENSP00000283109	ENSP00000356134
ENSP00000283109	ENSP00000356548
ENSP00000283109	ENSP00000358140
ENSP00000283109	ENSP00000360031
ENSP00000283109	ENSP00000364293
ENSP00000283109	ENSP00000365025
ENSP00000283109	ENSP00000366121
ENSP00000283109	ENSP00000368927
ENSP00000283109	ENSP00000369162
ENSP00000283109	ENSP00000369757
ENSP00000283109	ENSP00000372199
ENSP00000283109	ENSP00000376946
ENSP00000283109	ENSP00000378529
ENSP00000283109	ENSP00000379339
ENSP00000283109	ENSP00000379888
ENSP00000283109	ENSP00000393241
ENSP00000283109	ENSP00000402240
ENSP00000283109	ENSP00000403175
ENSP00000283109	ENSP00000414321
ENSP00000283109	ENSP00000416110
ENSP00000283109	ENSP00000417229

ENSP00000283109	ENSP00000418734
ENSP00000283109	ENSP00000420714
ENSP00000283147	ENSP00000297261
ENSP00000283147	ENSP00000328181
ENSP00000283147	ENSP00000332353
ENSP00000283147	ENSP00000363708
ENSP00000283195	ENSP00000285968
ENSP00000283195	ENSP00000290158
ENSP00000283195	ENSP00000294724
ENSP00000283195	ENSP00000295897
ENSP00000283195	ENSP00000297338
ENSP00000283195	ENSP00000300128
ENSP00000283195	ENSP00000302886
ENSP00000283195	ENSP00000305503
ENSP00000283195	ENSP00000306697
ENSP00000283195	ENSP00000309474
ENSP00000283195	ENSP00000310668
ENSP00000283195	ENSP00000312778
ENSP00000283195	ENSP00000316032
ENSP00000283195	ENSP00000316121
ENSP00000283195	ENSP00000322218
ENSP00000283195	ENSP00000325312
ENSP00000283195	ENSP00000325690
ENSP00000283195	ENSP00000327821
ENSP00000283195	ENSP00000329466
ENSP00000283195	ENSP00000330343
ENSP00000283195	ENSP00000336712
ENSP00000283195	ENSP00000343023
ENSP00000283195	ENSP00000343701
ENSP00000283195	ENSP00000344456
ENSP00000283195	ENSP00000346453
ENSP00000283195	ENSP00000348551
ENSP00000283195	ENSP00000348577
ENSP00000283195	ENSP00000352400
ENSP00000283195	ENSP00000353741
ENSP00000283195	ENSP00000354560
ENSP00000283195	ENSP00000354718
ENSP00000283195	ENSP00000354742
ENSP00000283195	ENSP00000354895
ENSP00000283195	ENSP00000355865
ENSP00000283195	ENSP00000356405
ENSP00000283195	ENSP00000356548
ENSP00000283195	ENSP00000360286
ENSP00000283195	ENSP00000360532
ENSP00000283195	ENSP00000361418
ENSP00000283195	ENSP00000366557

ENSP00000283195	ENSP00000369042
ENSP00000283195	ENSP00000369162
ENSP00000283195	ENSP00000371155
ENSP00000283195	ENSP00000386759
ENSP00000283195	ENSP00000386992
ENSP00000283195	ENSP00000391723
ENSP00000283195	ENSP00000394791
ENSP00000283195	ENSP00000396704
ENSP00000283195	ENSP00000414187
ENSP00000283195	ENSP00000417281
ENSP00000283228	ENSP00000288602
ENSP00000283228	ENSP00000311005
ENSP00000283228	ENSP00000339952
ENSP00000283228	ENSP00000375986
ENSP00000283228	ENSP00000402084
ENSP00000283290	ENSP00000287239
ENSP00000283290	ENSP00000296755
ENSP00000283290	ENSP00000311318
ENSP00000283290	ENSP00000318313
ENSP00000283290	ENSP00000328940
ENSP00000283290	ENSP00000334061
ENSP00000283290	ENSP00000335557
ENSP00000283290	ENSP00000343313
ENSP00000283290	ENSP00000346437
ENSP00000283290	ENSP00000349785
ENSP00000283290	ENSP00000356379
ENSP00000283290	ENSP00000361418
ENSP00000283290	ENSP00000404923
ENSP00000283351	ENSP00000291574
ENSP00000283351	ENSP00000296786
ENSP00000283351	ENSP00000299206
ENSP00000283351	ENSP00000301021
ENSP00000283351	ENSP00000302783
ENSP00000283351	ENSP00000303554
ENSP00000283351	ENSP00000305459
ENSP00000283351	ENSP00000308496
ENSP00000283351	ENSP00000311134
ENSP00000283351	ENSP00000316990
ENSP00000283351	ENSP00000325506
ENSP00000283351	ENSP00000326869
ENSP00000283351	ENSP00000327716
ENSP00000283351	ENSP00000348442
ENSP00000283351	ENSP00000349887
ENSP00000283351	ENSP00000352708
ENSP00000283351	ENSP00000352738
ENSP00000283351	ENSP00000355231

ENSP00000283351	ENSP00000360183
ENSP00000283351	ENSP00000360216
ENSP00000283351	ENSP00000360644
ENSP00000283351	ENSP00000360798
ENSP00000283351	ENSP00000369860
ENSP00000283351	ENSP00000373979
ENSP00000283351	ENSP00000376268
ENSP00000283351	ENSP00000377769
ENSP00000283351	ENSP00000397441
ENSP00000283351	ENSP00000402240
ENSP00000283351	ENSP00000404923
ENSP00000283351	ENSP00000409231
ENSP00000283426	ENSP00000313172
ENSP00000283429	ENSP00000323880
ENSP00000283429	ENSP00000360782
ENSP00000283628	ENSP00000351250
ENSP00000283628	ENSP00000362013
ENSP00000283628	ENSP00000389175
ENSP00000283628	ENSP00000417281
ENSP00000283635	ENSP00000325589
ENSP00000283635	ENSP00000334538
ENSP00000283635	ENSP00000340210
ENSP00000283635	ENSP00000354119
ENSP00000283635	ENSP00000356346
ENSP00000283635	ENSP00000356652
ENSP00000283646	ENSP00000329715
ENSP00000283646	ENSP00000337443
ENSP00000283646	ENSP00000349142
ENSP00000283646	ENSP00000356602
ENSP00000283646	ENSP00000359976
ENSP00000283646	ENSP00000369736
ENSP00000283646	ENSP00000370410
ENSP00000283646	ENSP00000372697
ENSP00000283646	ENSP00000386394
ENSP00000283646	ENSP00000390831
ENSP00000283646	ENSP00000391197
ENSP00000283646	ENSP00000401435
ENSP00000283646	ENSP00000404016
ENSP00000283646	ENSP00000406988
ENSP00000283646	ENSP00000409952
ENSP00000283871	ENSP00000311825
ENSP00000283875	ENSP00000285398
ENSP00000283875	ENSP00000312735
ENSP00000283875	ENSP00000314949
ENSP00000283875	ENSP00000329933
ENSP00000283875	ENSP00000336856

ENSP00000283875	ENSP00000340409
ENSP00000283875	ENSP00000340823
ENSP00000283875	ENSP00000342889
ENSP00000283875	ENSP00000343129
ENSP00000283875	ENSP00000344537
ENSP00000283875	ENSP00000346453
ENSP00000283875	ENSP00000346762
ENSP00000283875	ENSP00000348168
ENSP00000283875	ENSP00000348915
ENSP00000283875	ENSP00000350283
ENSP00000283875	ENSP00000353332
ENSP00000283875	ENSP00000354633
ENSP00000283875	ENSP00000357965
ENSP00000283875	ENSP00000359531
ENSP00000283875	ENSP00000361465
ENSP00000283875	ENSP00000362352
ENSP00000283875	ENSP00000370105
ENSP00000283875	ENSP00000375809
ENSP00000283875	ENSP00000377969
ENSP00000283875	ENSP00000378503
ENSP00000283875	ENSP00000379372
ENSP00000283875	ENSP00000417281
ENSP00000283882	ENSP00000286428
ENSP00000283882	ENSP00000287598
ENSP00000283882	ENSP00000291688
ENSP00000283882	ENSP00000293860
ENSP00000283882	ENSP00000295066
ENSP00000283882	ENSP00000296509
ENSP00000283882	ENSP00000299601
ENSP00000283882	ENSP00000300249
ENSP00000283882	ENSP00000302530
ENSP00000283882	ENSP00000304593
ENSP00000283882	ENSP00000306561
ENSP00000283882	ENSP00000306983
ENSP00000283882	ENSP00000307208
ENSP00000283882	ENSP00000307684
ENSP00000283882	ENSP00000307853
ENSP00000283882	ENSP00000312244
ENSP00000283882	ENSP00000312697
ENSP00000283882	ENSP00000314129
ENSP00000283882	ENSP00000320566
ENSP00000283882	ENSP00000322180
ENSP00000283882	ENSP00000322579
ENSP00000283882	ENSP00000325677
ENSP00000283882	ENSP00000329933
ENSP00000283882	ENSP00000331111

ENSP00000283882	ENSP00000331310
ENSP00000283882	ENSP00000334188
ENSP00000283882	ENSP00000337405
ENSP00000283882	ENSP00000339090
ENSP00000283882	ENSP00000340093
ENSP00000283882	ENSP00000340271
ENSP00000283882	ENSP00000340896
ENSP00000283882	ENSP00000347602
ENSP00000283882	ENSP00000347719
ENSP00000283882	ENSP00000348551
ENSP00000283882	ENSP00000351284
ENSP00000283882	ENSP00000352738
ENSP00000283882	ENSP00000353622
ENSP00000283882	ENSP00000355205
ENSP00000283882	ENSP00000356405
ENSP00000283882	ENSP00000356989
ENSP00000283882	ENSP00000357887
ENSP00000283882	ENSP00000359518
ENSP00000283882	ENSP00000360116
ENSP00000283882	ENSP00000360473
ENSP00000283882	ENSP00000360515
ENSP00000283882	ENSP00000362166
ENSP00000283882	ENSP00000362352
ENSP00000283882	ENSP00000362592
ENSP00000283882	ENSP00000364929
ENSP00000283882	ENSP00000369695
ENSP00000283882	ENSP00000374213
ENSP00000283882	ENSP00000374280
ENSP00000283882	ENSP00000376204
ENSP00000283882	ENSP00000378180
ENSP00000283882	ENSP00000381031
ENSP00000283882	ENSP00000382562
ENSP00000283882	ENSP00000383503
ENSP00000283882	ENSP00000386759
ENSP00000283882	ENSP00000397636
ENSP00000283882	ENSP00000402240
ENSP00000283882	ENSP00000405636
ENSP00000283882	ENSP00000406293
ENSP00000283882	ENSP00000410530
ENSP00000283882	ENSP00000410954
ENSP00000283882	ENSP00000414110
ENSP00000283882	ENSP00000416599
ENSP00000283921	ENSP00000303088
ENSP00000283921	ENSP00000318423
ENSP00000283921	ENSP00000340944
ENSP00000283921	ENSP00000362588

ENSP00000283928	ENSP00000291688
ENSP00000283928	ENSP00000298630
ENSP00000283928	ENSP00000307272
ENSP00000283928	ENSP00000313420
ENSP00000283928	ENSP00000334564
ENSP00000283928	ENSP00000341743
ENSP00000283928	ENSP00000347733
ENSP00000283928	ENSP00000354558
ENSP00000283928	ENSP00000355511
ENSP00000283928	ENSP00000363944
ENSP00000283928	ENSP00000365025
ENSP00000283928	ENSP00000371888
ENSP00000283928	ENSP00000394071
ENSP00000283928	ENSP00000402515
ENSP00000283943	ENSP00000295951
ENSP00000283943	ENSP00000302111
ENSP00000283943	ENSP00000308268
ENSP00000283943	ENSP00000308546
ENSP00000283943	ENSP00000309610
ENSP00000283943	ENSP00000310129
ENSP00000283943	ENSP00000323967
ENSP00000283943	ENSP00000327716
ENSP00000283943	ENSP00000329466
ENSP00000283943	ENSP00000334044
ENSP00000283943	ENSP00000335304
ENSP00000283943	ENSP00000339484
ENSP00000283943	ENSP00000340823
ENSP00000283943	ENSP00000343001
ENSP00000283943	ENSP00000343535
ENSP00000283943	ENSP00000355153
ENSP00000283943	ENSP00000361990
ENSP00000283943	ENSP00000362578
ENSP00000283943	ENSP00000366249
ENSP00000283943	ENSP00000366513
ENSP00000283943	ENSP00000368699
ENSP00000283943	ENSP00000373039
ENSP00000283943	ENSP00000379457
ENSP00000283943	ENSP00000386918
ENSP00000283943	ENSP00000396152
ENSP00000283943	ENSP00000410416
ENSP00000284000	ENSP00000305422
ENSP00000284000	ENSP00000306245
ENSP00000284000	ENSP00000336790
ENSP00000284000	ENSP00000340671
ENSP00000284000	ENSP00000340823
ENSP00000284000	ENSP00000368686

ENSP00000284000	ENSP00000386165
ENSP00000284000	ENSP00000392617
ENSP00000284031	ENSP00000311505
ENSP00000284202	ENSP00000300648
ENSP00000284202	ENSP00000304102
ENSP00000284202	ENSP00000304668
ENSP00000284202	ENSP00000310040
ENSP00000284202	ENSP00000318646
ENSP00000284202	ENSP00000348442
ENSP00000284202	ENSP00000350512
ENSP00000284202	ENSP00000354125
ENSP00000284202	ENSP00000356811
ENSP00000284202	ENSP00000358140
ENSP00000284202	ENSP00000360992
ENSP00000284202	ENSP00000388920
ENSP00000284202	ENSP00000417229
ENSP00000284202	ENSP00000420357
ENSP00000284268	ENSP00000346067
ENSP00000284320	ENSP00000308610
ENSP00000284320	ENSP00000315693
ENSP00000284320	ENSP00000318115
ENSP00000284320	ENSP00000320508
ENSP00000284320	ENSP00000354204
ENSP00000284320	ENSP00000357360
ENSP00000284320	ENSP00000358997
ENSP00000284320	ENSP00000361686
ENSP00000284320	ENSP00000361993
ENSP00000284320	ENSP00000364289
ENSP00000284320	ENSP00000364699
ENSP00000284320	ENSP00000368140
ENSP00000284320	ENSP00000387262
ENSP00000284601	ENSP00000285896
ENSP00000284601	ENSP00000294724
ENSP00000284601	ENSP00000317904
ENSP00000284601	ENSP00000350132
ENSP00000284601	ENSP00000350283
ENSP00000284601	ENSP00000384490
ENSP00000284637	ENSP00000302239
ENSP00000284637	ENSP00000307387
ENSP00000284637	ENSP00000309871
ENSP00000284637	ENSP00000322457
ENSP00000284637	ENSP00000324857
ENSP00000284637	ENSP00000345530
ENSP00000284637	ENSP00000361699
ENSP00000284637	ENSP00000362807
ENSP00000284637	ENSP00000363250

ENSP00000284637	ENSP00000365891
ENSP00000284637	ENSP00000370003
ENSP00000284637	ENSP00000379228
ENSP00000284719	ENSP00000287652
ENSP00000284719	ENSP00000290330
ENSP00000284719	ENSP00000290846
ENSP00000284719	ENSP00000291576
ENSP00000284719	ENSP00000296805
ENSP00000284719	ENSP00000300648
ENSP00000284719	ENSP00000304185
ENSP00000284719	ENSP00000310129
ENSP00000284719	ENSP00000310649
ENSP00000284719	ENSP00000312042
ENSP00000284719	ENSP00000319169
ENSP00000284719	ENSP00000322016
ENSP00000284719	ENSP00000323046
ENSP00000284719	ENSP00000345023
ENSP00000284719	ENSP00000355941
ENSP00000284719	ENSP00000358695
ENSP00000284719	ENSP00000358812
ENSP00000284719	ENSP00000361287
ENSP00000284719	ENSP00000361433
ENSP00000284719	ENSP00000364815
ENSP00000284719	ENSP00000367299
ENSP00000284719	ENSP00000369038
ENSP00000284719	ENSP00000384302
ENSP00000284719	ENSP00000396704
ENSP00000284719	ENSP00000401121
ENSP00000284719	ENSP00000403359
ENSP00000284719	ENSP00000410758
ENSP00000284719	ENSP00000415316
ENSP00000284727	ENSP00000290299
ENSP00000284727	ENSP00000295890
ENSP00000284727	ENSP00000297185
ENSP00000284727	ENSP00000301587
ENSP00000284727	ENSP00000332247
ENSP00000284727	ENSP00000343885
ENSP00000284727	ENSP00000349142
ENSP00000284727	ENSP00000352522
ENSP00000284727	ENSP00000354632
ENSP00000284727	ENSP00000354673
ENSP00000284727	ENSP00000358737
ENSP00000284727	ENSP00000361982
ENSP00000284727	ENSP00000363162
ENSP00000284727	ENSP00000370414
ENSP00000284727	ENSP00000379282

ENSP00000284727	ENSP00000390746
ENSP00000284770	ENSP00000309606
ENSP00000284776	ENSP00000324857
ENSP00000284776	ENSP00000346022
ENSP00000284776	ENSP00000346453
ENSP00000284776	ENSP00000352425
ENSP00000284776	ENSP00000356595
ENSP00000284776	ENSP00000361824
ENSP00000284776	ENSP00000383623
ENSP00000284776	ENSP00000400258
ENSP00000284776	ENSP00000417281
ENSP00000284811	ENSP00000287878
ENSP00000284811	ENSP00000288985
ENSP00000284811	ENSP00000290663
ENSP00000284811	ENSP00000291900
ENSP00000284811	ENSP00000296328
ENSP00000284811	ENSP00000298288
ENSP00000284811	ENSP00000301717
ENSP00000284811	ENSP00000304102
ENSP00000284811	ENSP00000305133
ENSP00000284811	ENSP00000307854
ENSP00000284811	ENSP00000308236
ENSP00000284811	ENSP00000308944
ENSP00000284811	ENSP00000314949
ENSP00000284811	ENSP00000318674
ENSP00000284811	ENSP00000321386
ENSP00000284811	ENSP00000321927
ENSP00000284811	ENSP00000325448
ENSP00000284811	ENSP00000330098
ENSP00000284811	ENSP00000334675
ENSP00000284811	ENSP00000343298
ENSP00000284811	ENSP00000348089
ENSP00000284811	ENSP00000350009
ENSP00000284811	ENSP00000350633
ENSP00000284811	ENSP00000351284
ENSP00000284811	ENSP00000353698
ENSP00000284811	ENSP00000358478
ENSP00000284811	ENSP00000361707
ENSP00000284811	ENSP00000366271
ENSP00000284811	ENSP00000370968
ENSP00000284811	ENSP00000381567
ENSP00000284811	ENSP00000395574
ENSP00000284811	ENSP00000403852
ENSP00000284811	ENSP00000409346
ENSP00000284811	ENSP00000418111
ENSP00000284818	ENSP00000363089

ENSP00000284957	ENSP00000295888
ENSP00000284957	ENSP00000299138
ENSP00000284957	ENSP00000317955
ENSP00000284957	ENSP00000320898
ENSP00000284957	ENSP00000334100
ENSP00000284957	ENSP00000353564
ENSP00000284957	ENSP00000354522
ENSP00000284957	ENSP00000354558
ENSP00000284957	ENSP00000354957
ENSP00000284957	ENSP00000364212
ENSP00000284957	ENSP00000366746
ENSP00000284957	ENSP00000374443
ENSP00000284957	ENSP00000377148
ENSP00000284957	ENSP00000381293
ENSP00000284957	ENSP00000383295
ENSP00000284957	ENSP00000397441
ENSP00000284957	ENSP00000415034
ENSP00000284957	ENSP00000419628
ENSP00000284981	ENSP00000287878
ENSP00000284981	ENSP00000289749
ENSP00000284981	ENSP00000290810
ENSP00000284981	ENSP00000294785
ENSP00000284981	ENSP00000295974
ENSP00000284981	ENSP00000296861
ENSP00000284981	ENSP00000306245
ENSP00000284981	ENSP00000309968
ENSP00000284981	ENSP00000313391
ENSP00000284981	ENSP00000325136
ENSP00000284981	ENSP00000326366
ENSP00000284981	ENSP00000329668
ENSP00000284981	ENSP00000331201
ENSP00000284981	ENSP00000332979
ENSP00000284981	ENSP00000338345
ENSP00000284981	ENSP00000339191
ENSP00000284981	ENSP00000340019
ENSP00000284981	ENSP00000340820
ENSP00000284981	ENSP00000341957
ENSP00000284981	ENSP00000345096
ENSP00000284981	ENSP00000347169
ENSP00000284981	ENSP00000348273
ENSP00000284981	ENSP00000349022
ENSP00000284981	ENSP00000349679
ENSP00000284981	ENSP00000350718
ENSP00000284981	ENSP00000351015
ENSP00000284981	ENSP00000351908
ENSP00000284981	ENSP00000355865

ENSP00000284981	ENSP00000355896
ENSP00000284981	ENSP00000360141
ENSP00000284981	ENSP00000361824
ENSP00000284981	ENSP00000362399
ENSP00000284981	ENSP00000363193
ENSP00000284981	ENSP00000363827
ENSP00000284981	ENSP00000366395
ENSP00000284981	ENSP00000366513
ENSP00000284981	ENSP00000376445
ENSP00000284981	ENSP00000378368
ENSP00000284981	ENSP00000381293
ENSP00000284981	ENSP00000384223
ENSP00000284981	ENSP00000390948
ENSP00000284981	ENSP00000392330
ENSP00000284981	ENSP00000417281
ENSP00000284995	ENSP00000304704
ENSP00000284995	ENSP00000305524
ENSP00000284995	ENSP00000323046
ENSP00000284995	ENSP00000327487
ENSP00000284995	ENSP00000353998
ENSP00000284995	ENSP00000355299
ENSP00000284995	ENSP00000361433
ENSP00000284995	ENSP00000402240
ENSP00000285021	ENSP00000285398
ENSP00000285021	ENSP00000288828
ENSP00000285021	ENSP00000288985
ENSP00000285021	ENSP00000296273
ENSP00000285021	ENSP00000297185
ENSP00000285021	ENSP00000298852
ENSP00000285021	ENSP00000301280
ENSP00000285021	ENSP00000301764
ENSP00000285021	ENSP00000302160
ENSP00000285021	ENSP00000303554
ENSP00000285021	ENSP00000304350
ENSP00000285021	ENSP00000306561
ENSP00000285021	ENSP00000308466
ENSP00000285021	ENSP00000308944
ENSP00000285021	ENSP00000310520
ENSP00000285021	ENSP00000313171
ENSP00000285021	ENSP00000317334
ENSP00000285021	ENSP00000318674
ENSP00000285021	ENSP00000321386
ENSP00000285021	ENSP00000321927
ENSP00000285021	ENSP00000322570
ENSP00000285021	ENSP00000323439
ENSP00000285021	ENSP00000324122

ENSP00000285021	ENSP00000325506
ENSP00000285021	ENSP00000330098
ENSP00000285021	ENSP00000334675
ENSP00000285021	ENSP00000336606
ENSP00000285021	ENSP00000343001
ENSP00000285021	ENSP00000343392
ENSP00000285021	ENSP00000343741
ENSP00000285021	ENSP00000347836
ENSP00000285021	ENSP00000348020
ENSP00000285021	ENSP00000348089
ENSP00000285021	ENSP00000349213
ENSP00000285021	ENSP00000349393
ENSP00000285021	ENSP00000350708
ENSP00000285021	ENSP00000351697
ENSP00000285021	ENSP00000354720
ENSP00000285021	ENSP00000355205
ENSP00000285021	ENSP00000358478
ENSP00000285021	ENSP00000360116
ENSP00000285021	ENSP00000362441
ENSP00000285021	ENSP00000364270
ENSP00000285021	ENSP00000366271
ENSP00000285021	ENSP00000366974
ENSP00000285021	ENSP00000367787
ENSP00000285021	ENSP00000368349
ENSP00000285021	ENSP00000368438
ENSP00000285021	ENSP00000369411
ENSP00000285021	ENSP00000369716
ENSP00000285021	ENSP00000369860
ENSP00000285021	ENSP00000369897
ENSP00000285021	ENSP00000370151
ENSP00000285021	ENSP00000370589
ENSP00000285021	ENSP00000372023
ENSP00000285021	ENSP00000376268
ENSP00000285021	ENSP00000376611
ENSP00000285021	ENSP00000376946
ENSP00000285021	ENSP00000391723
ENSP00000285021	ENSP00000391944
ENSP00000285021	ENSP00000406490
ENSP00000285021	ENSP00000418111
ENSP00000285046	ENSP00000306523
ENSP00000285046	ENSP00000358423
ENSP00000285071	ENSP00000354558
ENSP00000285083	ENSP00000317159
ENSP00000285083	ENSP00000326579
ENSP00000285083	ENSP00000361686
ENSP00000285083	ENSP00000378350

ENSP00000285116	ENSP00000319417
ENSP00000285116	ENSP00000333433
ENSP00000285116	ENSP00000370557
ENSP00000285243	ENSP00000304704
ENSP00000285243	ENSP00000308339
ENSP00000285243	ENSP00000310521
ENSP00000285243	ENSP00000322419
ENSP00000285243	ENSP00000356530
ENSP00000285243	ENSP00000362208
ENSP00000285243	ENSP00000362807
ENSP00000285243	ENSP00000401445
ENSP00000285273	ENSP00000292180
ENSP00000285273	ENSP00000322706
ENSP00000285273	ENSP00000355968
ENSP00000285279	ENSP00000359321
ENSP00000285298	ENSP00000301585
ENSP00000285298	ENSP00000348965
ENSP00000285298	ENSP00000379607
ENSP00000285397	ENSP00000312042
ENSP00000285398	ENSP00000290158
ENSP00000285398	ENSP00000300648
ENSP00000285398	ENSP00000303507
ENSP00000285398	ENSP00000305958
ENSP00000285398	ENSP00000306124
ENSP00000285398	ENSP00000307567
ENSP00000285398	ENSP00000314949
ENSP00000285398	ENSP00000324792
ENSP00000285398	ENSP00000329029
ENSP00000285398	ENSP00000330343
ENSP00000285398	ENSP00000339826
ENSP00000285398	ENSP00000341243
ENSP00000285398	ENSP00000346791
ENSP00000285398	ENSP00000347978
ENSP00000285398	ENSP00000348089
ENSP00000285398	ENSP00000348168
ENSP00000285398	ENSP00000350708
ENSP00000285398	ENSP00000351284
ENSP00000285398	ENSP00000355890
ENSP00000285398	ENSP00000357651
ENSP00000285398	ENSP00000359531
ENSP00000285398	ENSP00000359552
ENSP00000285398	ENSP00000362298
ENSP00000285398	ENSP00000362344
ENSP00000285398	ENSP00000367208
ENSP00000285398	ENSP00000375809
ENSP00000285398	ENSP00000381003

ENSP00000285398	ENSP00000391723
ENSP00000285402	ENSP00000323300
ENSP00000285402	ENSP00000363391
ENSP00000285518	ENSP00000294543
ENSP00000285518	ENSP00000302177
ENSP00000285518	ENSP00000354876
ENSP00000285518	ENSP00000355890
ENSP00000285518	ENSP00000357998
ENSP00000285873	ENSP00000290541
ENSP00000285873	ENSP00000297990
ENSP00000285873	ENSP00000300413
ENSP00000285873	ENSP00000301364
ENSP00000285873	ENSP00000308533
ENSP00000285873	ENSP00000310596
ENSP00000285873	ENSP00000310649
ENSP00000285873	ENSP00000315859
ENSP00000285873	ENSP00000316879
ENSP00000285873	ENSP00000320949
ENSP00000285873	ENSP00000321706
ENSP00000285873	ENSP00000322396
ENSP00000285873	ENSP00000324122
ENSP00000285873	ENSP00000329466
ENSP00000285873	ENSP00000331817
ENSP00000285873	ENSP00000339145
ENSP00000285873	ENSP00000340896
ENSP00000285873	ENSP00000342374
ENSP00000285873	ENSP00000343741
ENSP00000285873	ENSP00000356331
ENSP00000285873	ENSP00000356811
ENSP00000285873	ENSP00000358716
ENSP00000285873	ENSP00000360310
ENSP00000285873	ENSP00000361433
ENSP00000285873	ENSP00000363812
ENSP00000285873	ENSP00000366557
ENSP00000285873	ENSP00000370083
ENSP00000285873	ENSP00000370119
ENSP00000285873	ENSP00000370258
ENSP00000285873	ENSP00000372703
ENSP00000285873	ENSP00000382562
ENSP00000285873	ENSP00000393286
ENSP00000285873	ENSP00000393870
ENSP00000285873	ENSP00000400104
ENSP00000285873	ENSP00000400591
ENSP00000285873	ENSP00000402084
ENSP00000285873	ENSP00000402338
ENSP00000285873	ENSP00000402590

ENSP00000285873	ENSP00000412566
ENSP00000285879	ENSP00000300688
ENSP00000285879	ENSP00000317985
ENSP00000285879	ENSP00000355177
ENSP00000285879	ENSP00000377486
ENSP00000285879	ENSP00000377969
ENSP00000285896	ENSP00000295926
ENSP00000285896	ENSP00000296328
ENSP00000285896	ENSP00000296509
ENSP00000285896	ENSP00000302955
ENSP00000285896	ENSP00000304226
ENSP00000285896	ENSP00000307272
ENSP00000285896	ENSP00000314949
ENSP00000285896	ENSP00000317334
ENSP00000285896	ENSP00000319104
ENSP00000285896	ENSP00000320949
ENSP00000285896	ENSP00000322570
ENSP00000285896	ENSP00000330060
ENSP00000285896	ENSP00000331879
ENSP00000285896	ENSP00000339245
ENSP00000285896	ENSP00000339529
ENSP00000285896	ENSP00000350491
ENSP00000285896	ENSP00000351284
ENSP00000285896	ENSP00000351697
ENSP00000285896	ENSP00000354673
ENSP00000285896	ENSP00000356652
ENSP00000285896	ENSP00000359552
ENSP00000285896	ENSP00000360515
ENSP00000285896	ENSP00000361990
ENSP00000285896	ENSP00000366135
ENSP00000285896	ENSP00000366746
ENSP00000285896	ENSP00000368349
ENSP00000285896	ENSP00000368438
ENSP00000285896	ENSP00000369965
ENSP00000285896	ENSP00000372023
ENSP00000285896	ENSP00000374529
ENSP00000285896	ENSP00000381932
ENSP00000285896	ENSP00000383901
ENSP00000285896	ENSP00000391723
ENSP00000285896	ENSP00000396068
ENSP00000285896	ENSP00000402084
ENSP00000285928	ENSP00000304350
ENSP00000285928	ENSP00000343190
ENSP00000285928	ENSP00000347206
ENSP00000285928	ENSP00000369400
ENSP00000285928	ENSP00000379457

ENSP00000285928	ENSP00000401435
ENSP00000285949	ENSP00000310621
ENSP00000285949	ENSP00000341662
ENSP00000285949	ENSP00000348762
ENSP00000285968	ENSP00000290231
ENSP00000285968	ENSP00000291688
ENSP00000285968	ENSP00000294724
ENSP00000285968	ENSP00000300648
ENSP00000285968	ENSP00000305503
ENSP00000285968	ENSP00000308622
ENSP00000285968	ENSP00000310668
ENSP00000285968	ENSP00000316032
ENSP00000285968	ENSP00000329466
ENSP00000285968	ENSP00000352021
ENSP00000285968	ENSP00000353073
ENSP00000285968	ENSP00000355890
ENSP00000285968	ENSP00000356448
ENSP00000285968	ENSP00000360286
ENSP00000285968	ENSP00000364847
ENSP00000285968	ENSP00000371155
ENSP00000285968	ENSP00000382779
ENSP00000285968	ENSP00000402240
ENSP00000286067	ENSP00000381031
ENSP00000286070	ENSP00000300069
ENSP00000286070	ENSP00000315017
ENSP00000286070	ENSP00000334808
ENSP00000286070	ENSP00000336712
ENSP00000286070	ENSP00000349594
ENSP00000286070	ENSP00000355231
ENSP00000286070	ENSP00000360112
ENSP00000286070	ENSP00000360532
ENSP00000286070	ENSP00000386520
ENSP00000286091	ENSP00000292180
ENSP00000286091	ENSP00000299626
ENSP00000286091	ENSP00000299767
ENSP00000286091	ENSP00000318147
ENSP00000286091	ENSP00000321259
ENSP00000286091	ENSP00000327070
ENSP00000286091	ENSP00000329466
ENSP00000286091	ENSP00000331268
ENSP00000286091	ENSP00000332513
ENSP00000286091	ENSP00000340466
ENSP00000286091	ENSP00000340879
ENSP00000286091	ENSP00000349970
ENSP00000286091	ENSP00000362110
ENSP00000286091	ENSP00000366395

ENSP00000286091	ENSP00000379042
ENSP00000286091	ENSP00000380793
ENSP00000286091	ENSP00000382064
ENSP00000286091	ENSP00000401445
ENSP00000286186	ENSP00000301838
ENSP00000286186	ENSP00000312664
ENSP00000286186	ENSP00000314949
ENSP00000286186	ENSP00000318822
ENSP00000286186	ENSP00000329623
ENSP00000286186	ENSP00000343313
ENSP00000286186	ENSP00000347979
ENSP00000286186	ENSP00000356694
ENSP00000286186	ENSP00000365318
ENSP00000286186	ENSP00000384223
ENSP00000286186	ENSP00000404623
ENSP00000286186	ENSP00000419494
ENSP00000286195	ENSP00000312318
ENSP00000286195	ENSP00000340995
ENSP00000286234	ENSP00000307272
ENSP00000286234	ENSP00000349959
ENSP00000286234	ENSP00000354558
ENSP00000286234	ENSP00000359206
ENSP00000286234	ENSP00000371888
ENSP00000286307	ENSP00000319169
ENSP00000286307	ENSP00000319341
ENSP00000286307	ENSP00000370083
ENSP00000286307	ENSP00000370119
ENSP00000286307	ENSP00000412566
ENSP00000286317	ENSP00000290663
ENSP00000286317	ENSP00000290974
ENSP00000286317	ENSP00000291688
ENSP00000286317	ENSP00000292035
ENSP00000286317	ENSP00000293777
ENSP00000286317	ENSP00000294168
ENSP00000286317	ENSP00000295066
ENSP00000286317	ENSP00000296328
ENSP00000286317	ENSP00000297347
ENSP00000286317	ENSP00000299424
ENSP00000286317	ENSP00000299543
ENSP00000286317	ENSP00000299767
ENSP00000286317	ENSP00000300651
ENSP00000286317	ENSP00000301788
ENSP00000286317	ENSP00000302478
ENSP00000286317	ENSP00000303423
ENSP00000286317	ENSP00000304593
ENSP00000286317	ENSP00000306983

ENSP00000286317	ENSP00000307850
ENSP00000286317	ENSP00000312735
ENSP00000286317	ENSP00000314949
ENSP00000286317	ENSP00000320566
ENSP00000286317	ENSP00000323720
ENSP00000286317	ENSP00000323858
ENSP00000286317	ENSP00000324124
ENSP00000286317	ENSP00000325612
ENSP00000286317	ENSP00000325677
ENSP00000286317	ENSP00000326128
ENSP00000286317	ENSP00000326767
ENSP00000286317	ENSP00000326806
ENSP00000286317	ENSP00000331201
ENSP00000286317	ENSP00000331310
ENSP00000286317	ENSP00000336842
ENSP00000286317	ENSP00000337340
ENSP00000286317	ENSP00000340896
ENSP00000286317	ENSP00000342343
ENSP00000286317	ENSP00000343129
ENSP00000286317	ENSP00000343701
ENSP00000286317	ENSP00000344537
ENSP00000286317	ENSP00000347345
ENSP00000286317	ENSP00000348069
ENSP00000286317	ENSP00000352219
ENSP00000286317	ENSP00000353622
ENSP00000286317	ENSP00000355541
ENSP00000286317	ENSP00000355629
ENSP00000286317	ENSP00000355759
ENSP00000286317	ENSP00000356405
ENSP00000286317	ENSP00000357047
ENSP00000286317	ENSP00000357887
ENSP00000286317	ENSP00000357907
ENSP00000286317	ENSP00000359290
ENSP00000286317	ENSP00000359818
ENSP00000286317	ENSP00000360821
ENSP00000286317	ENSP00000361465
ENSP00000286317	ENSP00000361990
ENSP00000286317	ENSP00000362344
ENSP00000286317	ENSP00000362413
ENSP00000286317	ENSP00000362948
ENSP00000286317	ENSP00000363779
ENSP00000286317	ENSP00000365811
ENSP00000286317	ENSP00000366225
ENSP00000286317	ENSP00000366974
ENSP00000286317	ENSP00000369695
ENSP00000286317	ENSP00000373399

ENSP00000286317	ENSP00000374280
ENSP00000286317	ENSP00000374529
ENSP00000286317	ENSP00000376946
ENSP00000286317	ENSP00000381216
ENSP00000286317	ENSP00000384048
ENSP00000286317	ENSP00000386759
ENSP00000286317	ENSP00000391774
ENSP00000286317	ENSP00000392617
ENSP00000286317	ENSP00000403852
ENSP00000286317	ENSP00000418734
ENSP00000286364	ENSP00000314420
ENSP00000286364	ENSP00000340944
ENSP00000286364	ENSP00000343785
ENSP00000286364	ENSP00000357494
ENSP00000286428	ENSP00000290810
ENSP00000286428	ENSP00000295066
ENSP00000286428	ENSP00000296273
ENSP00000286428	ENSP00000296509
ENSP00000286428	ENSP00000300231
ENSP00000286428	ENSP00000300249
ENSP00000286428	ENSP00000302530
ENSP00000286428	ENSP00000303554
ENSP00000286428	ENSP00000304593
ENSP00000286428	ENSP00000305503
ENSP00000286428	ENSP00000308944
ENSP00000286428	ENSP00000312244
ENSP00000286428	ENSP00000312870
ENSP00000286428	ENSP00000314311
ENSP00000286428	ENSP00000317334
ENSP00000286428	ENSP00000318029
ENSP00000286428	ENSP00000320130
ENSP00000286428	ENSP00000321927
ENSP00000286428	ENSP00000322180
ENSP00000286428	ENSP00000325017
ENSP00000286428	ENSP00000325506
ENSP00000286428	ENSP00000328938
ENSP00000286428	ENSP00000330836
ENSP00000286428	ENSP00000331310
ENSP00000286428	ENSP00000334188
ENSP00000286428	ENSP00000334675
ENSP00000286428	ENSP00000340019
ENSP00000286428	ENSP00000347602
ENSP00000286428	ENSP00000347719
ENSP00000286428	ENSP00000348551
ENSP00000286428	ENSP00000348565
ENSP00000286428	ENSP00000348602

ENSP00000286428	ENSP00000351284
ENSP00000286428	ENSP00000351665
ENSP00000286428	ENSP00000353622
ENSP00000286428	ENSP00000353793
ENSP00000286428	ENSP00000354158
ENSP00000286428	ENSP00000354340
ENSP00000286428	ENSP00000355205
ENSP00000286428	ENSP00000355560
ENSP00000286428	ENSP00000355922
ENSP00000286428	ENSP00000356989
ENSP00000286428	ENSP00000357643
ENSP00000286428	ENSP00000357887
ENSP00000286428	ENSP00000360270
ENSP00000286428	ENSP00000360473
ENSP00000286428	ENSP00000360515
ENSP00000286428	ENSP00000361845
ENSP00000286428	ENSP00000362441
ENSP00000286428	ENSP00000363313
ENSP00000286428	ENSP00000363734
ENSP00000286428	ENSP00000363779
ENSP00000286428	ENSP00000369411
ENSP00000286428	ENSP00000369736
ENSP00000286428	ENSP00000369860
ENSP00000286428	ENSP00000370151
ENSP00000286428	ENSP00000372694
ENSP00000286428	ENSP00000373399
ENSP00000286428	ENSP00000374213
ENSP00000286428	ENSP00000374566
ENSP00000286428	ENSP00000376268
ENSP00000286428	ENSP00000376946
ENSP00000286428	ENSP00000377793
ENSP00000286428	ENSP00000381891
ENSP00000286428	ENSP00000382562
ENSP00000286428	ENSP00000412319
ENSP00000286428	ENSP00000412922
ENSP00000286428	ENSP00000415678
ENSP00000286428	ENSP00000417229
ENSP00000286428	ENSP00000417763
ENSP00000286448	ENSP00000294179
ENSP00000286448	ENSP00000304292
ENSP00000286448	ENSP00000305255
ENSP00000286448	ENSP00000305810
ENSP00000286448	ENSP00000309457
ENSP00000286448	ENSP00000310521
ENSP00000286448	ENSP00000312735
ENSP00000286448	ENSP00000317714

ENSP00000286448	ENSP00000320885
ENSP00000286448	ENSP00000324628
ENSP00000286448	ENSP00000325002
ENSP00000286448	ENSP00000327650
ENSP00000286448	ENSP00000330320
ENSP00000286448	ENSP00000344055
ENSP00000286448	ENSP00000345656
ENSP00000286448	ENSP00000347408
ENSP00000286448	ENSP00000349887
ENSP00000286448	ENSP00000350512
ENSP00000286448	ENSP00000353073
ENSP00000286448	ENSP00000356405
ENSP00000286448	ENSP00000357887
ENSP00000286448	ENSP00000360183
ENSP00000286448	ENSP00000366225
ENSP00000286448	ENSP00000369810
ENSP00000286448	ENSP00000374529
ENSP00000286448	ENSP00000381293
ENSP00000286448	ENSP00000390783
ENSP00000286448	ENSP00000397441
ENSP00000286448	ENSP00000417748
ENSP00000286448	ENSP00000418661
ENSP00000286523	ENSP00000361705
ENSP00000286523	ENSP00000365439
ENSP00000286523	ENSP00000380921
ENSP00000286523	ENSP00000417281
ENSP00000286621	ENSP00000305976
ENSP00000286621	ENSP00000315931
ENSP00000286621	ENSP00000327650
ENSP00000286621	ENSP00000333019
ENSP00000286621	ENSP00000340088
ENSP00000286621	ENSP00000354558
ENSP00000286621	ENSP00000356290
ENSP00000286621	ENSP00000359976
ENSP00000286621	ENSP00000361965
ENSP00000286621	ENSP00000362041
ENSP00000286621	ENSP00000364320
ENSP00000286621	ENSP00000367615
ENSP00000286621	ENSP00000369519
ENSP00000286621	ENSP00000377617
ENSP00000286621	ENSP00000394394
ENSP00000286621	ENSP00000419851
ENSP00000286627	ENSP00000295897
ENSP00000286627	ENSP00000295987
ENSP00000286627	ENSP00000300231
ENSP00000286627	ENSP00000301843

ENSP00000286627	ENSP00000341189
ENSP00000286719	ENSP00000296802
ENSP00000286719	ENSP00000312224
ENSP00000286719	ENSP00000317872
ENSP00000286719	ENSP00000336752
ENSP00000286719	ENSP00000339353
ENSP00000286719	ENSP00000343392
ENSP00000286719	ENSP00000345096
ENSP00000286719	ENSP00000348784
ENSP00000286719	ENSP00000359049
ENSP00000286719	ENSP00000361141
ENSP00000286719	ENSP00000366672
ENSP00000286719	ENSP00000367197
ENSP00000286719	ENSP00000417963
ENSP00000286758	ENSP00000300098
ENSP00000286800	ENSP00000325863
ENSP00000286800	ENSP00000347232
ENSP00000286800	ENSP00000348632
ENSP00000286800	ENSP00000350283
ENSP00000286800	ENSP00000355865
ENSP00000286800	ENSP00000369497
ENSP00000286800	ENSP00000377492
ENSP00000286835	ENSP00000294119
ENSP00000286835	ENSP00000296328
ENSP00000286835	ENSP00000296783
ENSP00000286835	ENSP00000298281
ENSP00000286835	ENSP00000299138
ENSP00000286835	ENSP00000299543
ENSP00000286835	ENSP00000301788
ENSP00000286835	ENSP00000302768
ENSP00000286835	ENSP00000305255
ENSP00000286835	ENSP00000305459
ENSP00000286835	ENSP00000307387
ENSP00000286835	ENSP00000308496
ENSP00000286835	ENSP00000310621
ENSP00000286835	ENSP00000312735
ENSP00000286835	ENSP00000314499
ENSP00000286835	ENSP00000314615
ENSP00000286835	ENSP00000314949
ENSP00000286835	ENSP00000316879
ENSP00000286835	ENSP00000320566
ENSP00000286835	ENSP00000323046
ENSP00000286835	ENSP00000326806
ENSP00000286835	ENSP00000333019
ENSP00000286835	ENSP00000333376
ENSP00000286835	ENSP00000337475

ENSP00000286835	ENSP00000337839
ENSP00000286835	ENSP00000339845
ENSP00000286835	ENSP00000340271
ENSP00000286835	ENSP00000340281
ENSP00000286835	ENSP00000340954
ENSP00000286835	ENSP00000343745
ENSP00000286835	ENSP00000345656
ENSP00000286835	ENSP00000347345
ENSP00000286835	ENSP00000347408
ENSP00000286835	ENSP00000349877
ENSP00000286835	ENSP00000351015
ENSP00000286835	ENSP00000351524
ENSP00000286835	ENSP00000355231
ENSP00000286835	ENSP00000356405
ENSP00000286835	ENSP00000359939
ENSP00000286835	ENSP00000360270
ENSP00000286835	ENSP00000360327
ENSP00000286835	ENSP00000360817
ENSP00000286835	ENSP00000361433
ENSP00000286835	ENSP00000361465
ENSP00000286835	ENSP00000361845
ENSP00000286835	ENSP00000362900
ENSP00000286835	ENSP00000364289
ENSP00000286835	ENSP00000364929
ENSP00000286835	ENSP00000366135
ENSP00000286835	ENSP00000368801
ENSP00000286835	ENSP00000368824
ENSP00000286835	ENSP00000368984
ENSP00000286835	ENSP00000371236
ENSP00000286835	ENSP00000372697
ENSP00000286835	ENSP00000374280
ENSP00000286835	ENSP00000389934
ENSP00000286835	ENSP00000390831
ENSP00000286835	ENSP00000391197
ENSP00000286835	ENSP00000397441
ENSP00000286835	ENSP00000402240
ENSP00000286835	ENSP00000403852
ENSP00000286835	ENSP00000404016
ENSP00000286835	ENSP00000406988
ENSP00000286835	ENSP00000409952
ENSP00000286835	ENSP00000419851
ENSP00000287022	ENSP00000297564
ENSP00000287022	ENSP00000307786
ENSP00000287022	ENSP00000309565
ENSP00000287022	ENSP00000317159
ENSP00000287022	ENSP00000317780

ENSP00000287022	ENSP00000332887
ENSP00000287022	ENSP00000336927
ENSP00000287022	ENSP00000354499
ENSP00000287022	ENSP00000354554
ENSP00000287022	ENSP00000354876
ENSP00000287022	ENSP00000354982
ENSP00000287022	ENSP00000355572
ENSP00000287022	ENSP00000367934
ENSP00000287022	ENSP00000387262
ENSP00000287025	ENSP00000292478
ENSP00000287025	ENSP00000299259
ENSP00000287025	ENSP00000301585
ENSP00000287038	ENSP00000294189
ENSP00000287038	ENSP00000296411
ENSP00000287038	ENSP00000296802
ENSP00000287038	ENSP00000297990
ENSP00000287038	ENSP00000298283
ENSP00000287038	ENSP00000300151
ENSP00000287038	ENSP00000302886
ENSP00000287038	ENSP00000302896
ENSP00000287038	ENSP00000306548
ENSP00000287038	ENSP00000307889
ENSP00000287038	ENSP00000308897
ENSP00000287038	ENSP00000309334
ENSP00000287038	ENSP00000309830
ENSP00000287038	ENSP00000311028
ENSP00000287038	ENSP00000311430
ENSP00000287038	ENSP00000312664
ENSP00000287038	ENSP00000317376
ENSP00000287038	ENSP00000318646
ENSP00000287038	ENSP00000322419
ENSP00000287038	ENSP00000325312
ENSP00000287038	ENSP00000326981
ENSP00000287038	ENSP00000329466
ENSP00000287038	ENSP00000338573
ENSP00000287038	ENSP00000338862
ENSP00000287038	ENSP00000339095
ENSP00000287038	ENSP00000339145
ENSP00000287038	ENSP00000339795
ENSP00000287038	ENSP00000345156
ENSP00000287038	ENSP00000346015
ENSP00000287038	ENSP00000346022
ENSP00000287038	ENSP00000346027
ENSP00000287038	ENSP00000346037
ENSP00000287038	ENSP00000346050
ENSP00000287038	ENSP00000346088

ENSP00000287038	ENSP00000346725
ENSP00000287038	ENSP00000347087
ENSP00000287038	ENSP00000354040
ENSP00000287038	ENSP00000354525
ENSP00000287038	ENSP00000354739
ENSP00000287038	ENSP00000355759
ENSP00000287038	ENSP00000355996
ENSP00000287038	ENSP00000359345
ENSP00000287038	ENSP00000361076
ENSP00000287038	ENSP00000362153
ENSP00000287038	ENSP00000362744
ENSP00000287038	ENSP00000363018
ENSP00000287038	ENSP00000363559
ENSP00000287038	ENSP00000363676
ENSP00000287038	ENSP00000364289
ENSP00000287038	ENSP00000364320
ENSP00000287038	ENSP00000365806
ENSP00000287038	ENSP00000369757
ENSP00000287038	ENSP00000370589
ENSP00000287038	ENSP00000372199
ENSP00000287038	ENSP00000373090
ENSP00000287038	ENSP00000373404
ENSP00000287038	ENSP00000375730
ENSP00000287038	ENSP00000377640
ENSP00000287038	ENSP00000377865
ENSP00000287038	ENSP00000378160
ENSP00000287038	ENSP00000379607
ENSP00000287038	ENSP00000379888
ENSP00000287038	ENSP00000380156
ENSP00000287038	ENSP00000380982
ENSP00000287038	ENSP00000386717
ENSP00000287038	ENSP00000389103
ENSP00000287038	ENSP00000393393
ENSP00000287038	ENSP00000394290
ENSP00000287038	ENSP00000401371
ENSP00000287038	ENSP00000402338
ENSP00000287038	ENSP00000403576
ENSP00000287038	ENSP00000409074
ENSP00000287038	ENSP00000409367
ENSP00000287038	ENSP00000411162
ENSP00000287038	ENSP00000414321
ENSP00000287038	ENSP00000417970
ENSP00000287078	ENSP00000360569
ENSP00000287218	ENSP00000373363
ENSP00000287239	ENSP00000296755
ENSP00000287239	ENSP00000297338

ENSP00000287239	ENSP00000297431
ENSP00000287239	ENSP00000307208
ENSP00000287239	ENSP00000311513
ENSP00000287239	ENSP00000319169
ENSP00000287239	ENSP00000320485
ENSP00000287239	ENSP00000338093
ENSP00000287239	ENSP00000343392
ENSP00000287239	ENSP00000349213
ENSP00000287239	ENSP00000351284
ENSP00000287239	ENSP00000352514
ENSP00000287239	ENSP00000355388
ENSP00000287239	ENSP00000356595
ENSP00000287239	ENSP00000365877
ENSP00000287239	ENSP00000366974
ENSP00000287239	ENSP00000381657
ENSP00000287239	ENSP00000386759
ENSP00000287295	ENSP00000290649
ENSP00000287295	ENSP00000296084
ENSP00000287295	ENSP00000300181
ENSP00000287295	ENSP00000301585
ENSP00000287295	ENSP00000305906
ENSP00000287295	ENSP00000311766
ENSP00000287295	ENSP00000317327
ENSP00000287295	ENSP00000322218
ENSP00000287295	ENSP00000337797
ENSP00000287295	ENSP00000341159
ENSP00000287295	ENSP00000341213
ENSP00000287295	ENSP00000341268
ENSP00000287295	ENSP00000344456
ENSP00000287295	ENSP00000346155
ENSP00000287295	ENSP00000347314
ENSP00000287295	ENSP00000347948
ENSP00000287295	ENSP00000348577
ENSP00000287295	ENSP00000351908
ENSP00000287295	ENSP00000352219
ENSP00000287295	ENSP00000362751
ENSP00000287295	ENSP00000365435
ENSP00000287295	ENSP00000369860
ENSP00000287295	ENSP00000369862
ENSP00000287295	ENSP00000379607
ENSP00000287295	ENSP00000381698
ENSP00000287295	ENSP00000386239
ENSP00000287295	ENSP00000411584
ENSP00000287322	ENSP00000329623
ENSP00000287322	ENSP00000379607
ENSP00000287437	ENSP00000296257

ENSP00000287437	ENSP00000298139
ENSP00000287437	ENSP00000322242
ENSP00000287437	ENSP00000323439
ENSP00000287437	ENSP00000327197
ENSP00000287437	ENSP00000334474
ENSP00000287437	ENSP00000343741
ENSP00000287437	ENSP00000347232
ENSP00000287437	ENSP00000347836
ENSP00000287437	ENSP00000352257
ENSP00000287437	ENSP00000354522
ENSP00000287437	ENSP00000354742
ENSP00000287437	ENSP00000354957
ENSP00000287437	ENSP00000355077
ENSP00000287437	ENSP00000356437
ENSP00000287437	ENSP00000356448
ENSP00000287437	ENSP00000358019
ENSP00000287437	ENSP00000364270
ENSP00000287437	ENSP00000365588
ENSP00000287437	ENSP00000367406
ENSP00000287437	ENSP00000368438
ENSP00000287437	ENSP00000369335
ENSP00000287437	ENSP00000370151
ENSP00000287437	ENSP00000371475
ENSP00000287437	ENSP00000373060
ENSP00000287437	ENSP00000384048
ENSP00000287437	ENSP00000387429
ENSP00000287437	ENSP00000394791
ENSP00000287437	ENSP00000396484
ENSP00000287437	ENSP00000396704
ENSP00000287437	ENSP00000404318
ENSP00000287437	ENSP00000409167
ENSP00000287437	ENSP00000415212
ENSP00000287437	ENSP00000416511
ENSP00000287482	ENSP00000313034
ENSP00000287482	ENSP00000362021
ENSP00000287482	ENSP00000371308
ENSP00000287598	ENSP00000296215
ENSP00000287598	ENSP00000296273
ENSP00000287598	ENSP00000296509
ENSP00000287598	ENSP00000297338
ENSP00000287598	ENSP00000298532
ENSP00000287598	ENSP00000299381
ENSP00000287598	ENSP00000300249
ENSP00000287598	ENSP00000300584
ENSP00000287598	ENSP00000301019
ENSP00000287598	ENSP00000301332

ENSP00000287598	ENSP00000302530
ENSP00000287598	ENSP00000304593
ENSP00000287598	ENSP00000307208
ENSP00000287598	ENSP00000311873
ENSP00000287598	ENSP00000313171
ENSP00000287598	ENSP00000313243
ENSP00000287598	ENSP00000313420
ENSP00000287598	ENSP00000314004
ENSP00000287598	ENSP00000315659
ENSP00000287598	ENSP00000315791
ENSP00000287598	ENSP00000318775
ENSP00000287598	ENSP00000318977
ENSP00000287598	ENSP00000322180
ENSP00000287598	ENSP00000322579
ENSP00000287598	ENSP00000323439
ENSP00000287598	ENSP00000324729
ENSP00000287598	ENSP00000325377
ENSP00000287598	ENSP00000325863
ENSP00000287598	ENSP00000329933
ENSP00000287598	ENSP00000330408
ENSP00000287598	ENSP00000331152
ENSP00000287598	ENSP00000331310
ENSP00000287598	ENSP00000334188
ENSP00000287598	ENSP00000335463
ENSP00000287598	ENSP00000336842
ENSP00000287598	ENSP00000339109
ENSP00000287598	ENSP00000341382
ENSP00000287598	ENSP00000343526
ENSP00000287598	ENSP00000343535
ENSP00000287598	ENSP00000343741
ENSP00000287598	ENSP00000346566
ENSP00000287598	ENSP00000347076
ENSP00000287598	ENSP00000347719
ENSP00000287598	ENSP00000347978
ENSP00000287598	ENSP00000348010
ENSP00000287598	ENSP00000348554
ENSP00000287598	ENSP00000350199
ENSP00000287598	ENSP00000351284
ENSP00000287598	ENSP00000351665
ENSP00000287598	ENSP00000352572
ENSP00000287598	ENSP00000353332
ENSP00000287598	ENSP00000353793
ENSP00000287598	ENSP00000354522
ENSP00000287598	ENSP00000354558
ENSP00000287598	ENSP00000354720
ENSP00000287598	ENSP00000354957

ENSP00000287598	ENSP00000355205
ENSP00000287598	ENSP00000355388
ENSP00000287598	ENSP00000355560
ENSP00000287598	ENSP00000355759
ENSP00000287598	ENSP00000355922
ENSP00000287598	ENSP00000355944
ENSP00000287598	ENSP00000356319
ENSP00000287598	ENSP00000356989
ENSP00000287598	ENSP00000357440
ENSP00000287598	ENSP00000357651
ENSP00000287598	ENSP00000357887
ENSP00000287598	ENSP00000357907
ENSP00000287598	ENSP00000359518
ENSP00000287598	ENSP00000360532
ENSP00000287598	ENSP00000360621
ENSP00000287598	ENSP00000363055
ENSP00000287598	ENSP00000363322
ENSP00000287598	ENSP00000364119
ENSP00000287598	ENSP00000365025
ENSP00000287598	ENSP00000367034
ENSP00000287598	ENSP00000368438
ENSP00000287598	ENSP00000368976
ENSP00000287598	ENSP00000369411
ENSP00000287598	ENSP00000369497
ENSP00000287598	ENSP00000370151
ENSP00000287598	ENSP00000370557
ENSP00000287598	ENSP00000370589
ENSP00000287598	ENSP00000374213
ENSP00000287598	ENSP00000374566
ENSP00000287598	ENSP00000375809
ENSP00000287598	ENSP00000375986
ENSP00000287598	ENSP00000378165
ENSP00000287598	ENSP00000378350
ENSP00000287598	ENSP00000382177
ENSP00000287598	ENSP00000382271
ENSP00000287598	ENSP00000382562
ENSP00000287598	ENSP00000386759
ENSP00000287598	ENSP00000394394
ENSP00000287598	ENSP00000402084
ENSP00000287598	ENSP00000408910
ENSP00000287600	ENSP00000367766
ENSP00000287600	ENSP00000377769
ENSP00000287647	ENSP00000289081
ENSP00000287647	ENSP00000304133
ENSP00000287647	ENSP00000307491
ENSP00000287647	ENSP00000310842

ENSP00000287647	ENSP00000311579
ENSP00000287647	ENSP00000325863
ENSP00000287647	ENSP00000326819
ENSP00000287647	ENSP00000337088
ENSP00000287647	ENSP00000339527
ENSP00000287647	ENSP00000343526
ENSP00000287647	ENSP00000343741
ENSP00000287647	ENSP00000346453
ENSP00000287647	ENSP00000347232
ENSP00000287647	ENSP00000350283
ENSP00000287647	ENSP00000354497
ENSP00000287647	ENSP00000355185
ENSP00000287647	ENSP00000358576
ENSP00000287647	ENSP00000360532
ENSP00000287647	ENSP00000367910
ENSP00000287647	ENSP00000368438
ENSP00000287647	ENSP00000369497
ENSP00000287647	ENSP00000372316
ENSP00000287647	ENSP00000373060
ENSP00000287647	ENSP00000381891
ENSP00000287647	ENSP00000385021
ENSP00000287647	ENSP00000386165
ENSP00000287647	ENSP00000409167
ENSP00000287652	ENSP00000340305
ENSP00000287652	ENSP00000348107
ENSP00000287652	ENSP00000348554
ENSP00000287652	ENSP00000368984
ENSP00000287652	ENSP00000396704
ENSP00000287675	ENSP00000290429
ENSP00000287675	ENSP00000309595
ENSP00000287675	ENSP00000312370
ENSP00000287675	ENSP00000337445
ENSP00000287675	ENSP00000345341
ENSP00000287675	ENSP00000346453
ENSP00000287675	ENSP00000361686
ENSP00000287706	ENSP00000382177
ENSP00000287713	ENSP00000340820
ENSP00000287713	ENSP00000353375
ENSP00000287713	ENSP00000353508
ENSP00000287713	ENSP00000370150
ENSP00000287820	ENSP00000294753
ENSP00000287820	ENSP00000300651
ENSP00000287820	ENSP00000304945
ENSP00000287820	ENSP00000305422
ENSP00000287820	ENSP00000307604
ENSP00000287820	ENSP00000320949

ENSP00000287820	ENSP00000322457
ENSP00000287820	ENSP00000324422
ENSP00000287820	ENSP00000327213
ENSP00000287820	ENSP00000329357
ENSP00000287820	ENSP00000337088
ENSP00000287820	ENSP00000340896
ENSP00000287820	ENSP00000348128
ENSP00000287820	ENSP00000348551
ENSP00000287820	ENSP00000348610
ENSP00000287820	ENSP00000349803
ENSP00000287820	ENSP00000350894
ENSP00000287820	ENSP00000351894
ENSP00000287820	ENSP00000357965
ENSP00000287820	ENSP00000358778
ENSP00000287820	ENSP00000363804
ENSP00000287820	ENSP00000378286
ENSP00000287820	ENSP00000378503
ENSP00000287820	ENSP00000381098
ENSP00000287820	ENSP00000382204
ENSP00000287820	ENSP00000392028
ENSP00000287820	ENSP00000395535
ENSP00000287820	ENSP00000399968
ENSP00000287820	ENSP00000417281
ENSP00000287859	ENSP00000308179
ENSP00000287878	ENSP00000287936
ENSP00000287878	ENSP00000288532
ENSP00000287878	ENSP00000294189
ENSP00000287878	ENSP00000296755
ENSP00000287878	ENSP00000298281
ENSP00000287878	ENSP00000300231
ENSP00000287878	ENSP00000304704
ENSP00000287878	ENSP00000307525
ENSP00000287878	ENSP00000307889
ENSP00000287878	ENSP00000311028
ENSP00000287878	ENSP00000313490
ENSP00000287878	ENSP00000340466
ENSP00000287878	ENSP00000340954
ENSP00000287878	ENSP00000345096
ENSP00000287878	ENSP00000348554
ENSP00000287878	ENSP00000358140
ENSP00000287878	ENSP00000359345
ENSP00000287878	ENSP00000361310
ENSP00000287878	ENSP00000362413
ENSP00000287878	ENSP00000363308
ENSP00000287878	ENSP00000363676
ENSP00000287878	ENSP00000367637

ENSP00000287878	ENSP00000369456
ENSP00000287878	ENSP00000377446
ENSP00000287878	ENSP00000379093
ENSP00000287878	ENSP00000394869
ENSP00000287878	ENSP00000419851
ENSP00000287936	ENSP00000290649
ENSP00000287936	ENSP00000304051
ENSP00000287936	ENSP00000309141
ENSP00000287936	ENSP00000311766
ENSP00000287936	ENSP00000312458
ENSP00000287936	ENSP00000317087
ENSP00000287936	ENSP00000320604
ENSP00000287936	ENSP00000325690
ENSP00000287936	ENSP00000333950
ENSP00000287936	ENSP00000337053
ENSP00000287936	ENSP00000344741
ENSP00000287936	ENSP00000345096
ENSP00000287936	ENSP00000346142
ENSP00000287936	ENSP00000347836
ENSP00000287936	ENSP00000349078
ENSP00000287936	ENSP00000350012
ENSP00000287936	ENSP00000352219
ENSP00000287936	ENSP00000352738
ENSP00000287936	ENSP00000354522
ENSP00000287936	ENSP00000358033
ENSP00000287936	ENSP00000360918
ENSP00000287936	ENSP00000365388
ENSP00000287936	ENSP00000366395
ENSP00000287936	ENSP00000376268
ENSP00000287936	ENSP00000381302
ENSP00000288065	ENSP00000381891
ENSP00000288087	ENSP00000346437
ENSP00000288087	ENSP00000347538
ENSP00000288167	ENSP00000295981
ENSP00000288167	ENSP00000328111
ENSP00000288167	ENSP00000332995
ENSP00000288167	ENSP00000345984
ENSP00000288167	ENSP00000368438
ENSP00000288199	ENSP00000288840
ENSP00000288199	ENSP00000296161
ENSP00000288199	ENSP00000300417
ENSP00000288199	ENSP00000302120
ENSP00000288199	ENSP00000312735
ENSP00000288199	ENSP00000324124
ENSP00000288199	ENSP00000340305
ENSP00000288199	ENSP00000367797

ENSP00000288199	ENSP00000403852
ENSP00000288266	ENSP00000314615
ENSP00000288266	ENSP00000331310
ENSP00000288266	ENSP00000333833
ENSP00000288266	ENSP00000342793
ENSP00000288266	ENSP00000344456
ENSP00000288266	ENSP00000360183
ENSP00000288266	ENSP00000362592
ENSP00000288422	ENSP00000317332
ENSP00000288422	ENSP00000342056
ENSP00000288422	ENSP00000348632
ENSP00000288422	ENSP00000355231
ENSP00000288422	ENSP00000358335
ENSP00000288422	ENSP00000363680
ENSP00000288502	ENSP00000295025
ENSP00000288532	ENSP00000290810
ENSP00000288532	ENSP00000310547
ENSP00000288532	ENSP00000338413
ENSP00000288532	ENSP00000345096
ENSP00000288532	ENSP00000346437
ENSP00000288532	ENSP00000348565
ENSP00000288532	ENSP00000354340
ENSP00000288532	ENSP00000355739
ENSP00000288532	ENSP00000359385
ENSP00000288532	ENSP00000360286
ENSP00000288532	ENSP00000367408
ENSP00000288602	ENSP00000297185
ENSP00000288602	ENSP00000303315
ENSP00000288602	ENSP00000306124
ENSP00000288602	ENSP00000309871
ENSP00000288602	ENSP00000312326
ENSP00000288602	ENSP00000318115
ENSP00000288602	ENSP00000336740
ENSP00000288602	ENSP00000339952
ENSP00000288602	ENSP00000340944
ENSP00000288602	ENSP00000342300
ENSP00000288602	ENSP00000343785
ENSP00000288602	ENSP00000343847
ENSP00000288602	ENSP00000347294
ENSP00000288602	ENSP00000353344
ENSP00000288602	ENSP00000356595
ENSP00000288602	ENSP00000358770
ENSP00000288602	ENSP00000359474
ENSP00000288602	ENSP00000363812
ENSP00000288602	ENSP00000364912
ENSP00000288602	ENSP00000368824

ENSP00000288602	ENSP00000372703
ENSP00000288602	ENSP00000380349
ENSP00000288602	ENSP00000382423
ENSP00000288602	ENSP00000393286
ENSP00000288602	ENSP00000393870
ENSP00000288602	ENSP00000400104
ENSP00000288602	ENSP00000402590
ENSP00000288774	ENSP00000295030
ENSP00000288774	ENSP00000296257
ENSP00000288774	ENSP00000298139
ENSP00000288774	ENSP00000299138
ENSP00000288774	ENSP00000302160
ENSP00000288774	ENSP00000303511
ENSP00000288774	ENSP00000307853
ENSP00000288774	ENSP00000309141
ENSP00000288774	ENSP00000313171
ENSP00000288774	ENSP00000313881
ENSP00000288774	ENSP00000317087
ENSP00000288774	ENSP00000317636
ENSP00000288774	ENSP00000321636
ENSP00000288774	ENSP00000325677
ENSP00000288774	ENSP00000325863
ENSP00000288774	ENSP00000327197
ENSP00000288774	ENSP00000329933
ENSP00000288774	ENSP00000330343
ENSP00000288774	ENSP00000331310
ENSP00000288774	ENSP00000334188
ENSP00000288774	ENSP00000334474
ENSP00000288774	ENSP00000337353
ENSP00000288774	ENSP00000347232
ENSP00000288774	ENSP00000347836
ENSP00000288774	ENSP00000349016
ENSP00000288774	ENSP00000349543
ENSP00000288774	ENSP00000351284
ENSP00000288774	ENSP00000356405
ENSP00000288774	ENSP00000356989
ENSP00000288774	ENSP00000357051
ENSP00000288774	ENSP00000358312
ENSP00000288774	ENSP00000358862
ENSP00000288774	ENSP00000366135
ENSP00000288774	ENSP00000369335
ENSP00000288774	ENSP00000377655
ENSP00000288774	ENSP00000377793
ENSP00000288774	ENSP00000380460
ENSP00000288774	ENSP00000394791
ENSP00000288774	ENSP00000396704

ENSP00000288774	ENSP00000402527
ENSP00000288774	ENSP00000407401
ENSP00000288828	ENSP00000290974
ENSP00000288828	ENSP00000294973
ENSP00000288828	ENSP00000300086
ENSP00000288828	ENSP00000300648
ENSP00000288828	ENSP00000305958
ENSP00000288828	ENSP00000343686
ENSP00000288828	ENSP00000348848
ENSP00000288828	ENSP00000350249
ENSP00000288828	ENSP00000353010
ENSP00000288828	ENSP00000353094
ENSP00000288828	ENSP00000358081
ENSP00000288828	ENSP00000362688
ENSP00000288828	ENSP00000363349
ENSP00000288828	ENSP00000364486
ENSP00000288828	ENSP00000364847
ENSP00000288828	ENSP00000368755
ENSP00000288828	ENSP00000370517
ENSP00000288828	ENSP00000379093
ENSP00000288828	ENSP00000419235
ENSP00000288840	ENSP00000328103
ENSP00000288840	ENSP00000341551
ENSP00000288840	ENSP00000345530
ENSP00000288840	ENSP00000352514
ENSP00000288840	ENSP00000358335
ENSP00000288840	ENSP00000392270
ENSP00000288840	ENSP00000397435
ENSP00000288937	ENSP00000296102
ENSP00000288937	ENSP00000296805
ENSP00000288937	ENSP00000297908
ENSP00000288937	ENSP00000300151
ENSP00000288937	ENSP00000301585
ENSP00000288937	ENSP00000302896
ENSP00000288937	ENSP00000305503
ENSP00000288937	ENSP00000306080
ENSP00000288937	ENSP00000306548
ENSP00000288937	ENSP00000308275
ENSP00000288937	ENSP00000308897
ENSP00000288937	ENSP00000311028
ENSP00000288937	ENSP00000312311
ENSP00000288937	ENSP00000314949
ENSP00000288937	ENSP00000315017
ENSP00000288937	ENSP00000317376
ENSP00000288937	ENSP00000323288
ENSP00000288937	ENSP00000324122

ENSP00000288937	ENSP00000326981
ENSP00000288937	ENSP00000333401
ENSP00000288937	ENSP00000333837
ENSP00000288937	ENSP00000339503
ENSP00000288937	ENSP00000339532
ENSP00000288937	ENSP00000339795
ENSP00000288937	ENSP00000339845
ENSP00000288937	ENSP00000341082
ENSP00000288937	ENSP00000343745
ENSP00000288937	ENSP00000346015
ENSP00000288937	ENSP00000346022
ENSP00000288937	ENSP00000346067
ENSP00000288937	ENSP00000354525
ENSP00000288937	ENSP00000358812
ENSP00000288937	ENSP00000359345
ENSP00000288937	ENSP00000360286
ENSP00000288937	ENSP00000362036
ENSP00000288937	ENSP00000362208
ENSP00000288937	ENSP00000363018
ENSP00000288937	ENSP00000363676
ENSP00000288937	ENSP00000364320
ENSP00000288937	ENSP00000368140
ENSP00000288937	ENSP00000370589
ENSP00000288937	ENSP00000373404
ENSP00000288937	ENSP00000374443
ENSP00000288937	ENSP00000375730
ENSP00000288937	ENSP00000377486
ENSP00000288937	ENSP00000377865
ENSP00000288937	ENSP00000389103
ENSP00000288937	ENSP00000393953
ENSP00000288937	ENSP00000417602
ENSP00000288976	ENSP00000293860
ENSP00000288976	ENSP00000295951
ENSP00000288976	ENSP00000296509
ENSP00000288976	ENSP00000297338
ENSP00000288976	ENSP00000297431
ENSP00000288976	ENSP00000300584
ENSP00000288976	ENSP00000308699
ENSP00000288976	ENSP00000310649
ENSP00000288976	ENSP00000310668
ENSP00000288976	ENSP00000322570
ENSP00000288976	ENSP00000324124
ENSP00000288976	ENSP00000325863
ENSP00000288976	ENSP00000331111
ENSP00000288976	ENSP00000343741
ENSP00000288976	ENSP00000346566

ENSP00000288976	ENSP00000351596
ENSP00000288976	ENSP00000354927
ENSP00000288976	ENSP00000361465
ENSP00000288976	ENSP00000362578
ENSP00000288976	ENSP00000365005
ENSP00000288976	ENSP00000367029
ENSP00000288976	ENSP00000371475
ENSP00000288976	ENSP00000372023
ENSP00000288976	ENSP00000377793
ENSP00000288976	ENSP00000382423
ENSP00000288976	ENSP00000382562
ENSP00000288976	ENSP00000383503
ENSP00000288976	ENSP00000397636
ENSP00000288976	ENSP00000405636
ENSP00000288976	ENSP00000410530
ENSP00000288976	ENSP00000410954
ENSP00000288976	ENSP00000414110
ENSP00000288976	ENSP00000416599
ENSP00000288985	ENSP00000296755
ENSP00000288985	ENSP00000300231
ENSP00000288985	ENSP00000302111
ENSP00000288985	ENSP00000318674
ENSP00000288985	ENSP00000321386
ENSP00000288985	ENSP00000321927
ENSP00000288985	ENSP00000325863
ENSP00000288985	ENSP00000330098
ENSP00000288985	ENSP00000343741
ENSP00000288985	ENSP00000360621
ENSP00000288985	ENSP00000364126
ENSP00000288985	ENSP00000366135
ENSP00000288985	ENSP00000366271
ENSP00000288985	ENSP00000369335
ENSP00000288985	ENSP00000370150
ENSP00000288985	ENSP00000384490
ENSP00000288985	ENSP00000418111
ENSP00000289013	ENSP00000299601
ENSP00000289013	ENSP00000300413
ENSP00000289013	ENSP00000302160
ENSP00000289013	ENSP00000310596
ENSP00000289013	ENSP00000319341
ENSP00000289013	ENSP00000337675
ENSP00000289013	ENSP00000353701
ENSP00000289013	ENSP00000354718
ENSP00000289081	ENSP00000296702
ENSP00000289081	ENSP00000296930
ENSP00000289081	ENSP00000299767

ENSP00000289081	ENSP00000326819
ENSP00000289081	ENSP00000330875
ENSP00000289081	ENSP00000344456
ENSP00000289081	ENSP00000346050
ENSP00000289081	ENSP00000354632
ENSP00000289081	ENSP00000361824
ENSP00000289081	ENSP00000367808
ENSP00000289081	ENSP00000367910
ENSP00000289081	ENSP00000373952
ENSP00000289081	ENSP00000385021
ENSP00000289081	ENSP00000396301
ENSP00000289359	ENSP00000310440
ENSP00000289371	ENSP00000294053
ENSP00000289371	ENSP00000296577
ENSP00000289371	ENSP00000296802
ENSP00000289371	ENSP00000298310
ENSP00000289371	ENSP00000298746
ENSP00000289371	ENSP00000299601
ENSP00000289371	ENSP00000302801
ENSP00000289371	ENSP00000304668
ENSP00000289371	ENSP00000307889
ENSP00000289371	ENSP00000308897
ENSP00000289371	ENSP00000309433
ENSP00000289371	ENSP00000311430
ENSP00000289371	ENSP00000314348
ENSP00000289371	ENSP00000318646
ENSP00000289371	ENSP00000325448
ENSP00000289371	ENSP00000329466
ENSP00000289371	ENSP00000329558
ENSP00000289371	ENSP00000339795
ENSP00000289371	ENSP00000340093
ENSP00000289371	ENSP00000343557
ENSP00000289371	ENSP00000346022
ENSP00000289371	ENSP00000346120
ENSP00000289371	ENSP00000346236
ENSP00000289371	ENSP00000346762
ENSP00000289371	ENSP00000347087
ENSP00000289371	ENSP00000354125
ENSP00000289371	ENSP00000354739
ENSP00000289371	ENSP00000356290
ENSP00000289371	ENSP00000358140
ENSP00000289371	ENSP00000358497
ENSP00000289371	ENSP00000358563
ENSP00000289371	ENSP00000358812
ENSP00000289371	ENSP00000359345
ENSP00000289371	ENSP00000360532

ENSP00000289371	ENSP00000360828
ENSP00000289371	ENSP00000361076
ENSP00000289371	ENSP00000361232
ENSP00000289371	ENSP00000361982
ENSP00000289371	ENSP00000362688
ENSP00000289371	ENSP00000362744
ENSP00000289371	ENSP00000363944
ENSP00000289371	ENSP00000364119
ENSP00000289371	ENSP00000366974
ENSP00000289371	ENSP00000367498
ENSP00000289371	ENSP00000367637
ENSP00000289371	ENSP00000368927
ENSP00000289371	ENSP00000369162
ENSP00000289371	ENSP00000370258
ENSP00000289371	ENSP00000372093
ENSP00000289371	ENSP00000373404
ENSP00000289371	ENSP00000374399
ENSP00000289371	ENSP00000377617
ENSP00000289371	ENSP00000378286
ENSP00000289371	ENSP00000379888
ENSP00000289371	ENSP00000380982
ENSP00000289371	ENSP00000393953
ENSP00000289371	ENSP00000417229
ENSP00000289373	ENSP00000322170
ENSP00000289382	ENSP00000290231
ENSP00000289422	ENSP00000323967
ENSP00000289422	ENSP00000348307
ENSP00000289431	ENSP00000295025
ENSP00000289431	ENSP00000312244
ENSP00000289431	ENSP00000313752
ENSP00000289431	ENSP00000352408
ENSP00000289431	ENSP00000360473
ENSP00000289431	ENSP00000363734
ENSP00000289431	ENSP00000366434
ENSP00000289431	ENSP00000367408
ENSP00000289431	ENSP00000370376
ENSP00000289431	ENSP00000412319
ENSP00000289528	ENSP00000309474
ENSP00000289528	ENSP00000310129
ENSP00000289528	ENSP00000331487
ENSP00000289528	ENSP00000363338
ENSP00000289619	ENSP00000350249
ENSP00000289749	ENSP00000365131
ENSP00000289968	ENSP00000300574
ENSP00000289968	ENSP00000301843
ENSP00000289968	ENSP00000315659

ENSP00000289968	ENSP00000344666
ENSP00000289968	ENSP00000361027
ENSP00000289968	ENSP00000380921
ENSP00000289968	ENSP00000413625
ENSP00000290130	ENSP00000336868
ENSP00000290130	ENSP00000362592
ENSP00000290130	ENSP00000366953
ENSP00000290130	ENSP00000367034
ENSP00000290130	ENSP00000387982
ENSP00000290155	ENSP00000294952
ENSP00000290158	ENSP00000290649
ENSP00000290158	ENSP00000291688
ENSP00000290158	ENSP00000292432
ENSP00000290158	ENSP00000296257
ENSP00000290158	ENSP00000297338
ENSP00000290158	ENSP00000298746
ENSP00000290158	ENSP00000300128
ENSP00000290158	ENSP00000300403
ENSP00000290158	ENSP00000300413
ENSP00000290158	ENSP00000301019
ENSP00000290158	ENSP00000302111
ENSP00000290158	ENSP00000302955
ENSP00000290158	ENSP00000304370
ENSP00000290158	ENSP00000305503
ENSP00000290158	ENSP00000306561
ENSP00000290158	ENSP00000308546
ENSP00000290158	ENSP00000308622
ENSP00000290158	ENSP00000309334
ENSP00000290158	ENSP00000309831
ENSP00000290158	ENSP00000310082
ENSP00000290158	ENSP00000310668
ENSP00000290158	ENSP00000311430
ENSP00000290158	ENSP00000311873
ENSP00000290158	ENSP00000312778
ENSP00000290158	ENSP00000313674
ENSP00000290158	ENSP00000316032
ENSP00000290158	ENSP00000316879
ENSP00000290158	ENSP00000317955
ENSP00000290158	ENSP00000320130
ENSP00000290158	ENSP00000322579
ENSP00000290158	ENSP00000324122
ENSP00000290158	ENSP00000324203
ENSP00000290158	ENSP00000326806
ENSP00000290158	ENSP00000327197
ENSP00000290158	ENSP00000327213
ENSP00000290158	ENSP00000328103

ENSP00000290158	ENSP00000329466
ENSP00000290158	ENSP00000331310
ENSP00000290158	ENSP00000332103
ENSP00000290158	ENSP00000334148
ENSP00000290158	ENSP00000337405
ENSP00000290158	ENSP00000340281
ENSP00000290158	ENSP00000341170
ENSP00000290158	ENSP00000341243
ENSP00000290158	ENSP00000341551
ENSP00000290158	ENSP00000342374
ENSP00000290158	ENSP00000343701
ENSP00000290158	ENSP00000343741
ENSP00000290158	ENSP00000344241
ENSP00000290158	ENSP00000344504
ENSP00000290158	ENSP00000344547
ENSP00000290158	ENSP00000344741
ENSP00000290158	ENSP00000345702
ENSP00000290158	ENSP00000345895
ENSP00000290158	ENSP00000346453
ENSP00000290158	ENSP00000346725
ENSP00000290158	ENSP00000347184
ENSP00000290158	ENSP00000347314
ENSP00000290158	ENSP00000348577
ENSP00000290158	ENSP00000352400
ENSP00000290158	ENSP00000354125
ENSP00000290158	ENSP00000354497
ENSP00000290158	ENSP00000356448
ENSP00000290158	ENSP00000358563
ENSP00000290158	ENSP00000358605
ENSP00000290158	ENSP00000359345
ENSP00000290158	ENSP00000359403
ENSP00000290158	ENSP00000359518
ENSP00000290158	ENSP00000359688
ENSP00000290158	ENSP00000360532
ENSP00000290158	ENSP00000360798
ENSP00000290158	ENSP00000361982
ENSP00000290158	ENSP00000362352
ENSP00000290158	ENSP00000362532
ENSP00000290158	ENSP00000362936
ENSP00000290158	ENSP00000363779
ENSP00000290158	ENSP00000364289
ENSP00000290158	ENSP00000364929
ENSP00000290158	ENSP00000366135
ENSP00000290158	ENSP00000367208
ENSP00000290158	ENSP00000369042
ENSP00000290158	ENSP00000369716

ENSP00000290158	ENSP00000370083
ENSP00000290158	ENSP00000370105
ENSP00000290158	ENSP00000370119
ENSP00000290158	ENSP00000370588
ENSP00000290158	ENSP00000370589
ENSP00000290158	ENSP00000370741
ENSP00000290158	ENSP00000372023
ENSP00000290158	ENSP00000374443
ENSP00000290158	ENSP00000375730
ENSP00000290158	ENSP00000376309
ENSP00000290158	ENSP00000377655
ENSP00000290158	ENSP00000379213
ENSP00000290158	ENSP00000379607
ENSP00000290158	ENSP00000380504
ENSP00000290158	ENSP00000381282
ENSP00000290158	ENSP00000382779
ENSP00000290158	ENSP00000384109
ENSP00000290158	ENSP00000394791
ENSP00000290158	ENSP00000402527
ENSP00000290158	ENSP00000412566
ENSP00000290158	ENSP00000414321
ENSP00000290158	ENSP00000416959
ENSP00000290158	ENSP00000417229
ENSP00000290158	ENSP00000418734
ENSP00000290216	ENSP00000300086
ENSP00000290219	ENSP00000356713
ENSP00000290231	ENSP00000291688
ENSP00000290231	ENSP00000301740
ENSP00000290231	ENSP00000302790
ENSP00000290231	ENSP00000302886
ENSP00000290231	ENSP00000308622
ENSP00000290231	ENSP00000310668
ENSP00000290231	ENSP00000312753
ENSP00000290231	ENSP00000313829
ENSP00000290231	ENSP00000326261
ENSP00000290231	ENSP00000343701
ENSP00000290231	ENSP00000352400
ENSP00000290231	ENSP00000354673
ENSP00000290231	ENSP00000354957
ENSP00000290231	ENSP00000356744
ENSP00000290231	ENSP00000361180
ENSP00000290231	ENSP00000366135
ENSP00000290231	ENSP00000379607
ENSP00000290231	ENSP00000382779
ENSP00000290231	ENSP00000392985
ENSP00000290231	ENSP00000397441

ENSP00000290231	ENSP00000403536
ENSP00000290291	ENSP00000300086
ENSP00000290291	ENSP00000326261
ENSP00000290299	ENSP00000292475
ENSP00000290299	ENSP00000294008
ENSP00000290299	ENSP00000295890
ENSP00000290299	ENSP00000300688
ENSP00000290299	ENSP00000301587
ENSP00000290299	ENSP00000306003
ENSP00000290299	ENSP00000306106
ENSP00000290299	ENSP00000317159
ENSP00000290299	ENSP00000317780
ENSP00000290299	ENSP00000324956
ENSP00000290299	ENSP00000332887
ENSP00000290299	ENSP00000333255
ENSP00000290299	ENSP00000337838
ENSP00000290299	ENSP00000341838
ENSP00000290299	ENSP00000349142
ENSP00000290299	ENSP00000354554
ENSP00000290299	ENSP00000354632
ENSP00000290299	ENSP00000354791
ENSP00000290299	ENSP00000358737
ENSP00000290299	ENSP00000358831
ENSP00000290299	ENSP00000359321
ENSP00000290299	ENSP00000360782
ENSP00000290299	ENSP00000361686
ENSP00000290299	ENSP00000364188
ENSP00000290299	ENSP00000365243
ENSP00000290299	ENSP00000367934
ENSP00000290299	ENSP00000370414
ENSP00000290299	ENSP00000387262
ENSP00000290299	ENSP00000388275
ENSP00000290299	ENSP00000389649
ENSP00000290299	ENSP00000392330
ENSP00000290330	ENSP00000297265
ENSP00000290330	ENSP00000310440
ENSP00000290330	ENSP00000314949
ENSP00000290330	ENSP00000317468
ENSP00000290330	ENSP00000354522
ENSP00000290330	ENSP00000366565
ENSP00000290330	ENSP00000367299
ENSP00000290330	ENSP00000386394
ENSP00000290332	ENSP00000327070
ENSP00000290332	ENSP00000336702
ENSP00000290332	ENSP00000352706
ENSP00000290349	ENSP00000338990

ENSP00000290349	ENSP00000348565
ENSP00000290349	ENSP00000352928
ENSP00000290401	ENSP00000361032
ENSP00000290429	ENSP00000301585
ENSP00000290429	ENSP00000304102
ENSP00000290429	ENSP00000306080
ENSP00000290429	ENSP00000312735
ENSP00000290429	ENSP00000315693
ENSP00000290429	ENSP00000322570
ENSP00000290429	ENSP00000324122
ENSP00000290429	ENSP00000330343
ENSP00000290429	ENSP00000337445
ENSP00000290429	ENSP00000340019
ENSP00000290429	ENSP00000351697
ENSP00000290429	ENSP00000364320
ENSP00000290429	ENSP00000367407
ENSP00000290429	ENSP00000368349
ENSP00000290429	ENSP00000394382
ENSP00000290524	ENSP00000296785
ENSP00000290524	ENSP00000300738
ENSP00000290524	ENSP00000307208
ENSP00000290524	ENSP00000316328
ENSP00000290524	ENSP00000332171
ENSP00000290524	ENSP00000333920
ENSP00000290524	ENSP00000343741
ENSP00000290524	ENSP00000344537
ENSP00000290524	ENSP00000349213
ENSP00000290524	ENSP00000360532
ENSP00000290524	ENSP00000368349
ENSP00000290524	ENSP00000368438
ENSP00000290524	ENSP00000372023
ENSP00000290524	ENSP00000375986
ENSP00000290541	ENSP00000293362
ENSP00000290541	ENSP00000294008
ENSP00000290541	ENSP00000295901
ENSP00000290541	ENSP00000298852
ENSP00000290541	ENSP00000300249
ENSP00000290541	ENSP00000307746
ENSP00000290541	ENSP00000309474
ENSP00000290541	ENSP00000310129
ENSP00000290541	ENSP00000311083
ENSP00000290541	ENSP00000311344
ENSP00000290541	ENSP00000314837
ENSP00000290541	ENSP00000318195
ENSP00000290541	ENSP00000325919
ENSP00000290541	ENSP00000343535

ENSP00000290541	ENSP00000348442
ENSP00000290541	ENSP00000352820
ENSP00000290541	ENSP00000353059
ENSP00000290541	ENSP00000358147
ENSP00000290541	ENSP00000362334
ENSP00000290541	ENSP00000366249
ENSP00000290541	ENSP00000366953
ENSP00000290541	ENSP00000370222
ENSP00000290541	ENSP00000384211
ENSP00000290541	ENSP00000396937
ENSP00000290541	ENSP00000403852
ENSP00000290541	ENSP00000404232
ENSP00000290541	ENSP00000416583
ENSP00000290541	ENSP00000418734
ENSP00000290597	ENSP00000349577
ENSP00000290607	ENSP00000301457
ENSP00000290607	ENSP00000343818
ENSP00000290607	ENSP00000348128
ENSP00000290649	ENSP00000294053
ENSP00000290649	ENSP00000294119
ENSP00000290649	ENSP00000294785
ENSP00000290649	ENSP00000296328
ENSP00000290649	ENSP00000296755
ENSP00000290649	ENSP00000299767
ENSP00000290649	ENSP00000300231
ENSP00000290649	ENSP00000309141
ENSP00000290649	ENSP00000312042
ENSP00000290649	ENSP00000317087
ENSP00000290649	ENSP00000317334
ENSP00000290649	ENSP00000318115
ENSP00000290649	ENSP00000321606
ENSP00000290649	ENSP00000329002
ENSP00000290649	ENSP00000331487
ENSP00000290649	ENSP00000340019
ENSP00000290649	ENSP00000344741
ENSP00000290649	ENSP00000346130
ENSP00000290649	ENSP00000347184
ENSP00000290649	ENSP00000348877
ENSP00000290649	ENSP00000350708
ENSP00000290649	ENSP00000357998
ENSP00000290649	ENSP00000359305
ENSP00000290649	ENSP00000359345
ENSP00000290649	ENSP00000359976
ENSP00000290649	ENSP00000360621
ENSP00000290649	ENSP00000361057
ENSP00000290649	ENSP00000365131

ENSP00000290649	ENSP00000365534
ENSP00000290649	ENSP00000366156
ENSP00000290649	ENSP00000366395
ENSP00000290649	ENSP00000379457
ENSP00000290649	ENSP00000381282
ENSP00000290649	ENSP00000384900
ENSP00000290649	ENSP00000389182
ENSP00000290663	ENSP00000292035
ENSP00000290663	ENSP00000293777
ENSP00000290663	ENSP00000295685
ENSP00000290663	ENSP00000295899
ENSP00000290663	ENSP00000297347
ENSP00000290663	ENSP00000299543
ENSP00000290663	ENSP00000300651
ENSP00000290663	ENSP00000323720
ENSP00000290663	ENSP00000325612
ENSP00000290663	ENSP00000326128
ENSP00000290663	ENSP00000326767
ENSP00000290663	ENSP00000337340
ENSP00000290663	ENSP00000342343
ENSP00000290663	ENSP00000342434
ENSP00000290663	ENSP00000349016
ENSP00000290663	ENSP00000357047
ENSP00000290663	ENSP00000357907
ENSP00000290663	ENSP00000362948
ENSP00000290663	ENSP00000363193
ENSP00000290663	ENSP00000381216
ENSP00000290663	ENSP00000384048
ENSP00000290663	ENSP00000391774
ENSP00000290663	ENSP00000392617
ENSP00000290663	ENSP00000418734
ENSP00000290795	ENSP00000381031
ENSP00000290810	ENSP00000299626
ENSP00000290810	ENSP00000300738
ENSP00000290810	ENSP00000301788
ENSP00000290810	ENSP00000323822
ENSP00000290810	ENSP00000340879
ENSP00000290810	ENSP00000341957
ENSP00000290810	ENSP00000346566
ENSP00000290810	ENSP00000354340
ENSP00000290810	ENSP00000358147
ENSP00000290810	ENSP00000359939
ENSP00000290810	ENSP00000363779
ENSP00000290810	ENSP00000365773
ENSP00000290810	ENSP00000366434
ENSP00000290810	ENSP00000368887

ENSP00000290846	ENSP00000294724
ENSP00000290846	ENSP00000314615
ENSP00000290846	ENSP00000340019
ENSP00000290846	ENSP00000351644
ENSP00000290846	ENSP00000356405
ENSP00000290846	ENSP00000361005
ENSP00000290846	ENSP00000362592
ENSP00000290846	ENSP00000373399
ENSP00000290846	ENSP00000386759
ENSP00000290846	ENSP00000402038
ENSP00000290866	ENSP00000302176
ENSP00000290866	ENSP00000340409
ENSP00000290913	ENSP00000345445
ENSP00000290913	ENSP00000387262
ENSP00000290949	ENSP00000292114
ENSP00000290949	ENSP00000307853
ENSP00000290949	ENSP00000310520
ENSP00000290949	ENSP00000314615
ENSP00000290949	ENSP00000326534
ENSP00000290949	ENSP00000332247
ENSP00000290949	ENSP00000333255
ENSP00000290949	ENSP00000351284
ENSP00000290949	ENSP00000351697
ENSP00000290949	ENSP00000352522
ENSP00000290949	ENSP00000356744
ENSP00000290949	ENSP00000358777
ENSP00000290949	ENSP00000360183
ENSP00000290949	ENSP00000363162
ENSP00000290949	ENSP00000365756
ENSP00000290949	ENSP00000367697
ENSP00000290949	ENSP00000369513
ENSP00000290953	ENSP00000335074
ENSP00000290974	ENSP00000334188
ENSP00000290974	ENSP00000346022
ENSP00000290974	ENSP00000354519
ENSP00000291232	ENSP00000365048
ENSP00000291386	ENSP00000292476
ENSP00000291386	ENSP00000296328
ENSP00000291386	ENSP00000296490
ENSP00000291386	ENSP00000297338
ENSP00000291386	ENSP00000298281
ENSP00000291386	ENSP00000299543
ENSP00000291386	ENSP00000304704
ENSP00000291386	ENSP00000312735
ENSP00000291386	ENSP00000314949
ENSP00000291386	ENSP00000317872

ENSP00000291386	ENSP00000325377
ENSP00000291386	ENSP00000329029
ENSP00000291386	ENSP00000332444
ENSP00000291386	ENSP00000336752
ENSP00000291386	ENSP00000339353
ENSP00000291386	ENSP00000340896
ENSP00000291386	ENSP00000354720
ENSP00000291386	ENSP00000355205
ENSP00000291386	ENSP00000358497
ENSP00000291386	ENSP00000359531
ENSP00000291386	ENSP00000366030
ENSP00000291386	ENSP00000366434
ENSP00000291386	ENSP00000382779
ENSP00000291386	ENSP00000387019
ENSP00000291526	ENSP00000321106
ENSP00000291539	ENSP00000348554
ENSP00000291539	ENSP00000351015
ENSP00000291539	ENSP00000362987
ENSP00000291539	ENSP00000363095
ENSP00000291560	ENSP00000356405
ENSP00000291560	ENSP00000357440
ENSP00000291565	ENSP00000325863
ENSP00000291565	ENSP00000351284
ENSP00000291565	ENSP00000360116
ENSP00000291565	ENSP00000379607
ENSP00000291574	ENSP00000302783
ENSP00000291574	ENSP00000311134
ENSP00000291574	ENSP00000316990
ENSP00000291574	ENSP00000352708
ENSP00000291574	ENSP00000359000
ENSP00000291574	ENSP00000373979
ENSP00000291574	ENSP00000376871
ENSP00000291574	ENSP00000402935
ENSP00000291576	ENSP00000296792
ENSP00000291576	ENSP00000297579
ENSP00000291576	ENSP00000297990
ENSP00000291576	ENSP00000298746
ENSP00000291576	ENSP00000301012
ENSP00000291576	ENSP00000302896
ENSP00000291576	ENSP00000305702
ENSP00000291576	ENSP00000308179
ENSP00000291576	ENSP00000310590
ENSP00000291576	ENSP00000311135
ENSP00000291576	ENSP00000311430
ENSP00000291576	ENSP00000314193
ENSP00000291576	ENSP00000314348

ENSP00000291576	ENSP00000315674
ENSP00000291576	ENSP00000321449
ENSP00000291576	ENSP00000322419
ENSP00000291576	ENSP00000326981
ENSP00000291576	ENSP00000327179
ENSP00000291576	ENSP00000328854
ENSP00000291576	ENSP00000329466
ENSP00000291576	ENSP00000338862
ENSP00000291576	ENSP00000339145
ENSP00000291576	ENSP00000339795
ENSP00000291576	ENSP00000353246
ENSP00000291576	ENSP00000354040
ENSP00000291576	ENSP00000354125
ENSP00000291576	ENSP00000354742
ENSP00000291576	ENSP00000355471
ENSP00000291576	ENSP00000355541
ENSP00000291576	ENSP00000358563
ENSP00000291576	ENSP00000358812
ENSP00000291576	ENSP00000359345
ENSP00000291576	ENSP00000360031
ENSP00000291576	ENSP00000362105
ENSP00000291576	ENSP00000362352
ENSP00000291576	ENSP00000362744
ENSP00000291576	ENSP00000363642
ENSP00000291576	ENSP00000363746
ENSP00000291576	ENSP00000366974
ENSP00000291576	ENSP00000370258
ENSP00000291576	ENSP00000370589
ENSP00000291576	ENSP00000371101
ENSP00000291576	ENSP00000371169
ENSP00000291576	ENSP00000372695
ENSP00000291576	ENSP00000373404
ENSP00000291576	ENSP00000379042
ENSP00000291576	ENSP00000384302
ENSP00000291576	ENSP00000386134
ENSP00000291576	ENSP00000399454
ENSP00000291576	ENSP00000402869
ENSP00000291576	ENSP00000405614
ENSP00000291576	ENSP00000410396
ENSP00000291577	ENSP00000379607
ENSP00000291582	ENSP00000300574
ENSP00000291582	ENSP00000331310
ENSP00000291582	ENSP00000342181
ENSP00000291582	ENSP00000353622
ENSP00000291582	ENSP00000360031
ENSP00000291582	ENSP00000362592

ENSP00000291672	ENSP00000387911
ENSP00000291688	ENSP00000294168
ENSP00000291688	ENSP00000295066
ENSP00000291688	ENSP00000300896
ENSP00000291688	ENSP00000301788
ENSP00000291688	ENSP00000303434
ENSP00000291688	ENSP00000304593
ENSP00000291688	ENSP00000304704
ENSP00000291688	ENSP00000305503
ENSP00000291688	ENSP00000308622
ENSP00000291688	ENSP00000310042
ENSP00000291688	ENSP00000310596
ENSP00000291688	ENSP00000310668
ENSP00000291688	ENSP00000315791
ENSP00000291688	ENSP00000316032
ENSP00000291688	ENSP00000318374
ENSP00000291688	ENSP00000320566
ENSP00000291688	ENSP00000321711
ENSP00000291688	ENSP00000323858
ENSP00000291688	ENSP00000325677
ENSP00000291688	ENSP00000326806
ENSP00000291688	ENSP00000329466
ENSP00000291688	ENSP00000331817
ENSP00000291688	ENSP00000332340
ENSP00000291688	ENSP00000333376
ENSP00000291688	ENSP00000334808
ENSP00000291688	ENSP00000334836
ENSP00000291688	ENSP00000336712
ENSP00000291688	ENSP00000336842
ENSP00000291688	ENSP00000337405
ENSP00000291688	ENSP00000343535
ENSP00000291688	ENSP00000343701
ENSP00000291688	ENSP00000343741
ENSP00000291688	ENSP00000344537
ENSP00000291688	ENSP00000346986
ENSP00000291688	ENSP00000347733
ENSP00000291688	ENSP00000348010
ENSP00000291688	ENSP00000348107
ENSP00000291688	ENSP00000350009
ENSP00000291688	ENSP00000352400
ENSP00000291688	ENSP00000353622
ENSP00000291688	ENSP00000354125
ENSP00000291688	ENSP00000354742
ENSP00000291688	ENSP00000355185
ENSP00000291688	ENSP00000356405
ENSP00000291688	ENSP00000356448

ENSP00000291688	ENSP00000356470
ENSP00000291688	ENSP00000357887
ENSP00000291688	ENSP00000358576
ENSP00000291688	ENSP00000358886
ENSP00000291688	ENSP00000360286
ENSP00000291688	ENSP00000360515
ENSP00000291688	ENSP00000362532
ENSP00000291688	ENSP00000362900
ENSP00000291688	ENSP00000363313
ENSP00000291688	ENSP00000364289
ENSP00000291688	ENSP00000365811
ENSP00000291688	ENSP00000366135
ENSP00000291688	ENSP00000366513
ENSP00000291688	ENSP00000366974
ENSP00000291688	ENSP00000367527
ENSP00000291688	ENSP00000369042
ENSP00000291688	ENSP00000369897
ENSP00000291688	ENSP00000370589
ENSP00000291688	ENSP00000371155
ENSP00000291688	ENSP00000372023
ENSP00000291688	ENSP00000374213
ENSP00000291688	ENSP00000378350
ENSP00000291688	ENSP00000381654
ENSP00000291688	ENSP00000381863
ENSP00000291688	ENSP00000382562
ENSP00000291688	ENSP00000382779
ENSP00000291688	ENSP00000386456
ENSP00000291688	ENSP00000386992
ENSP00000291688	ENSP00000387006
ENSP00000291688	ENSP00000391944
ENSP00000291688	ENSP00000396620
ENSP00000291688	ENSP00000396937
ENSP00000291688	ENSP00000397259
ENSP00000291688	ENSP00000398208
ENSP00000291688	ENSP00000403397
ENSP00000291688	ENSP00000417229
ENSP00000291688	ENSP00000419628
ENSP00000292035	ENSP00000293777
ENSP00000292035	ENSP00000297347
ENSP00000292035	ENSP00000300651
ENSP00000292035	ENSP00000314615
ENSP00000292035	ENSP00000323720
ENSP00000292035	ENSP00000326128
ENSP00000292035	ENSP00000326767
ENSP00000292035	ENSP00000337340
ENSP00000292035	ENSP00000340019

ENSP00000292035	ENSP00000342343
ENSP00000292035	ENSP00000354951
ENSP00000292035	ENSP00000357047
ENSP00000292035	ENSP00000357907
ENSP00000292035	ENSP00000362948
ENSP00000292035	ENSP00000363193
ENSP00000292035	ENSP00000369162
ENSP00000292035	ENSP00000381216
ENSP00000292035	ENSP00000384048
ENSP00000292035	ENSP00000385021
ENSP00000292035	ENSP00000391774
ENSP00000292035	ENSP00000392617
ENSP00000292035	ENSP00000418734
ENSP00000292055	ENSP00000334061
ENSP00000292055	ENSP00000343392
ENSP00000292055	ENSP00000377793
ENSP00000292055	ENSP00000378306
ENSP00000292114	ENSP00000333255
ENSP00000292114	ENSP00000358777
ENSP00000292114	ENSP00000367697
ENSP00000292114	ENSP00000369513
ENSP00000292180	ENSP00000299608
ENSP00000292180	ENSP00000301585
ENSP00000292180	ENSP00000301788
ENSP00000292180	ENSP00000304592
ENSP00000292180	ENSP00000304908
ENSP00000292180	ENSP00000305465
ENSP00000292180	ENSP00000310129
ENSP00000292180	ENSP00000318602
ENSP00000292180	ENSP00000321706
ENSP00000292180	ENSP00000322706
ENSP00000292180	ENSP00000323313
ENSP00000292180	ENSP00000330836
ENSP00000292180	ENSP00000340019
ENSP00000292180	ENSP00000344460
ENSP00000292180	ENSP00000347345
ENSP00000292180	ENSP00000355890
ENSP00000292180	ENSP00000356574
ENSP00000292180	ENSP00000357904
ENSP00000292180	ENSP00000358307
ENSP00000292180	ENSP00000359818
ENSP00000292180	ENSP00000360327
ENSP00000292180	ENSP00000365775
ENSP00000292180	ENSP00000371888
ENSP00000292180	ENSP00000386722
ENSP00000292180	ENSP00000391685

ENSP00000292180	ENSP00000394085
ENSP00000292180	ENSP00000401508
ENSP00000292199	ENSP00000369618
ENSP00000292199	ENSP00000369871
ENSP00000292211	ENSP00000312439
ENSP00000292363	ENSP00000309831
ENSP00000292363	ENSP00000312735
ENSP00000292363	ENSP00000324124
ENSP00000292363	ENSP00000327704
ENSP00000292363	ENSP00000365131
ENSP00000292363	ENSP00000377969
ENSP00000292363	ENSP00000403852
ENSP00000292363	ENSP00000417281
ENSP00000292385	ENSP00000298910
ENSP00000292385	ENSP00000314499
ENSP00000292385	ENSP00000315659
ENSP00000292385	ENSP00000326238
ENSP00000292385	ENSP00000334836
ENSP00000292385	ENSP00000348163
ENSP00000292385	ENSP00000348602
ENSP00000292385	ENSP00000349016
ENSP00000292385	ENSP00000350249
ENSP00000292385	ENSP00000359225
ENSP00000292385	ENSP00000373600
ENSP00000292385	ENSP00000377496
ENSP00000292385	ENSP00000386456
ENSP00000292385	ENSP00000411409
ENSP00000292432	ENSP00000310649
ENSP00000292432	ENSP00000312652
ENSP00000292432	ENSP00000336790
ENSP00000292432	ENSP00000339933
ENSP00000292432	ENSP00000364895
ENSP00000292432	ENSP00000377040
ENSP00000292433	ENSP00000344572
ENSP00000292475	ENSP00000300688
ENSP00000292475	ENSP00000301587
ENSP00000292475	ENSP00000306003
ENSP00000292475	ENSP00000317159
ENSP00000292475	ENSP00000317780
ENSP00000292475	ENSP00000318089
ENSP00000292475	ENSP00000329360
ENSP00000292475	ENSP00000337838
ENSP00000292475	ENSP00000341838
ENSP00000292475	ENSP00000343313
ENSP00000292475	ENSP00000346037
ENSP00000292475	ENSP00000349142

ENSP00000292475	ENSP00000358737
ENSP00000292475	ENSP00000361686
ENSP00000292475	ENSP00000364188
ENSP00000292475	ENSP00000365243
ENSP00000292475	ENSP00000370414
ENSP00000292475	ENSP00000385083
ENSP00000292475	ENSP00000389649
ENSP00000292476	ENSP00000296490
ENSP00000292476	ENSP00000296930
ENSP00000292476	ENSP00000298281
ENSP00000292476	ENSP00000300291
ENSP00000292476	ENSP00000304704
ENSP00000292476	ENSP00000315791
ENSP00000292476	ENSP00000317872
ENSP00000292476	ENSP00000325377
ENSP00000292476	ENSP00000331817
ENSP00000292476	ENSP00000332444
ENSP00000292476	ENSP00000336752
ENSP00000292476	ENSP00000339353
ENSP00000292476	ENSP00000348010
ENSP00000292476	ENSP00000355185
ENSP00000292476	ENSP00000356405
ENSP00000292476	ENSP00000358576
ENSP00000292476	ENSP00000367527
ENSP00000292476	ENSP00000398208
ENSP00000292478	ENSP00000298310
ENSP00000292478	ENSP00000299335
ENSP00000292478	ENSP00000301585
ENSP00000292478	ENSP00000325448
ENSP00000292478	ENSP00000354554
ENSP00000292478	ENSP00000356405
ENSP00000292478	ENSP00000361949
ENSP00000292478	ENSP00000363745
ENSP00000292478	ENSP00000380453
ENSP00000292494	ENSP00000354782
ENSP00000292513	ENSP00000360141
ENSP00000292535	ENSP00000293860
ENSP00000292535	ENSP00000303088
ENSP00000292535	ENSP00000308546
ENSP00000292535	ENSP00000315379
ENSP00000292535	ENSP00000315859
ENSP00000292535	ENSP00000317636
ENSP00000292535	ENSP00000320251
ENSP00000292535	ENSP00000324122
ENSP00000292535	ENSP00000341024
ENSP00000292535	ENSP00000344468

ENSP00000292535	ENSP00000347379
ENSP00000292535	ENSP00000351490
ENSP00000292535	ENSP00000355899
ENSP00000292535	ENSP00000363313
ENSP00000292535	ENSP00000365735
ENSP00000292535	ENSP00000381698
ENSP00000292535	ENSP00000402338
ENSP00000292535	ENSP00000407879
ENSP00000292535	ENSP00000408236
ENSP00000292535	ENSP00000410396
ENSP00000292566	ENSP00000356231
ENSP00000292566	ENSP00000364476
ENSP00000292616	ENSP00000360532
ENSP00000292616	ENSP00000374455
ENSP00000292894	ENSP00000330945
ENSP00000293195	ENSP00000320949
ENSP00000293195	ENSP00000329029
ENSP00000293195	ENSP00000340019
ENSP00000293195	ENSP00000342087
ENSP00000293195	ENSP00000357748
ENSP00000293195	ENSP00000359211
ENSP00000293195	ENSP00000359531
ENSP00000293195	ENSP00000372654
ENSP00000293195	ENSP00000377865
ENSP00000293230	ENSP00000380921
ENSP00000293288	ENSP00000298852
ENSP00000293288	ENSP00000306124
ENSP00000293288	ENSP00000308546
ENSP00000293288	ENSP00000309103
ENSP00000293288	ENSP00000318822
ENSP00000293288	ENSP00000326119
ENSP00000293288	ENSP00000329623
ENSP00000293288	ENSP00000334061
ENSP00000293288	ENSP00000338345
ENSP00000293288	ENSP00000340019
ENSP00000293288	ENSP00000340278
ENSP00000293288	ENSP00000348455
ENSP00000293288	ENSP00000352257
ENSP00000293288	ENSP00000358022
ENSP00000293288	ENSP00000361005
ENSP00000293288	ENSP00000363591
ENSP00000293288	ENSP00000378180
ENSP00000293288	ENSP00000401445
ENSP00000293330	ENSP00000307822
ENSP00000293350	ENSP00000311665
ENSP00000293350	ENSP00000343526

ENSP00000293350	ENSP00000366620
ENSP00000293362	ENSP00000298852
ENSP00000293362	ENSP00000307491
ENSP00000293362	ENSP00000308750
ENSP00000293362	ENSP00000314971
ENSP00000293362	ENSP00000324274
ENSP00000293362	ENSP00000325919
ENSP00000293362	ENSP00000328940
ENSP00000293362	ENSP00000334148
ENSP00000293362	ENSP00000335808
ENSP00000293362	ENSP00000336856
ENSP00000293362	ENSP00000341680
ENSP00000293362	ENSP00000343313
ENSP00000293362	ENSP00000352706
ENSP00000293362	ENSP00000354201
ENSP00000293362	ENSP00000355153
ENSP00000293362	ENSP00000355944
ENSP00000293362	ENSP00000362334
ENSP00000293362	ENSP00000363921
ENSP00000293362	ENSP00000368439
ENSP00000293362	ENSP00000370222
ENSP00000293362	ENSP00000372023
ENSP00000293362	ENSP00000384474
ENSP00000293362	ENSP00000386212
ENSP00000293362	ENSP00000417132
ENSP00000293362	ENSP00000417281
ENSP00000293371	ENSP00000294984
ENSP00000293371	ENSP00000295897
ENSP00000293371	ENSP00000304903
ENSP00000293371	ENSP00000311505
ENSP00000293371	ENSP00000312318
ENSP00000293371	ENSP00000347948
ENSP00000293371	ENSP00000354270
ENSP00000293371	ENSP00000379607
ENSP00000293371	ENSP00000417281
ENSP00000293404	ENSP00000293406
ENSP00000293404	ENSP00000338165
ENSP00000293404	ENSP00000387262
ENSP00000293406	ENSP00000295491
ENSP00000293406	ENSP00000296792
ENSP00000293406	ENSP00000298746
ENSP00000293406	ENSP00000300413
ENSP00000293406	ENSP00000307863
ENSP00000293406	ENSP00000308944
ENSP00000293406	ENSP00000309830
ENSP00000293406	ENSP00000310596

ENSP00000293406	ENSP00000312042
ENSP00000293406	ENSP00000320503
ENSP00000293406	ENSP00000324122
ENSP00000293406	ENSP00000325290
ENSP00000293406	ENSP00000326411
ENSP00000293406	ENSP00000326806
ENSP00000293406	ENSP00000327070
ENSP00000293406	ENSP00000328690
ENSP00000293406	ENSP00000332103
ENSP00000293406	ENSP00000341743
ENSP00000293406	ENSP00000342374
ENSP00000293406	ENSP00000351284
ENSP00000293406	ENSP00000352738
ENSP00000293406	ENSP00000356130
ENSP00000293406	ENSP00000358478
ENSP00000293406	ENSP00000358497
ENSP00000293406	ENSP00000358563
ENSP00000293406	ENSP00000359518
ENSP00000293406	ENSP00000361206
ENSP00000293406	ENSP00000361310
ENSP00000293406	ENSP00000361577
ENSP00000293406	ENSP00000361982
ENSP00000293406	ENSP00000362936
ENSP00000293406	ENSP00000362993
ENSP00000293406	ENSP00000364813
ENSP00000293406	ENSP00000366607
ENSP00000293406	ENSP00000366843
ENSP00000293406	ENSP00000370589
ENSP00000293406	ENSP00000372883
ENSP00000293406	ENSP00000373363
ENSP00000293406	ENSP00000377793
ENSP00000293406	ENSP00000380460
ENSP00000293406	ENSP00000391723
ENSP00000293406	ENSP00000397259
ENSP00000293406	ENSP00000400591
ENSP00000293406	ENSP00000402935
ENSP00000293406	ENSP00000403345
ENSP00000293406	ENSP00000406280
ENSP00000293406	ENSP00000410758
ENSP00000293406	ENSP00000412566
ENSP00000293406	ENSP00000414006
ENSP00000293406	ENSP00000414634
ENSP00000293441	ENSP00000296452
ENSP00000293441	ENSP00000306138
ENSP00000293441	ENSP00000340688
ENSP00000293441	ENSP00000345731

ENSP00000293441	ENSP00000354927
ENSP00000293441	ENSP00000361824
ENSP00000293441	ENSP00000362762
ENSP00000293441	ENSP00000363163
ENSP00000293441	ENSP00000364893
ENSP00000293441	ENSP00000365005
ENSP00000293441	ENSP00000365550
ENSP00000293441	ENSP00000381098
ENSP00000293441	ENSP00000381698
ENSP00000293441	ENSP00000382423
ENSP00000293441	ENSP00000400258
ENSP00000293662	ENSP00000304604
ENSP00000293662	ENSP00000306138
ENSP00000293662	ENSP00000367408
ENSP00000293662	ENSP00000408236
ENSP00000293695	ENSP00000385122
ENSP00000293709	ENSP00000314380
ENSP00000293760	ENSP00000357283
ENSP00000293760	ENSP00000380921
ENSP00000293777	ENSP00000297347
ENSP00000293777	ENSP00000300651
ENSP00000293777	ENSP00000308759
ENSP00000293777	ENSP00000312778
ENSP00000293777	ENSP00000323720
ENSP00000293777	ENSP00000325612
ENSP00000293777	ENSP00000326128
ENSP00000293777	ENSP00000326767
ENSP00000293777	ENSP00000337340
ENSP00000293777	ENSP00000342343
ENSP00000293777	ENSP00000357047
ENSP00000293777	ENSP00000357907
ENSP00000293777	ENSP00000362948
ENSP00000293777	ENSP00000363193
ENSP00000293777	ENSP00000381216
ENSP00000293777	ENSP00000384048
ENSP00000293777	ENSP00000391774
ENSP00000293777	ENSP00000392617
ENSP00000293777	ENSP00000418734
ENSP00000293845	ENSP00000300181
ENSP00000293845	ENSP00000380467
ENSP00000293860	ENSP00000296509
ENSP00000293860	ENSP00000299853
ENSP00000293860	ENSP00000301788
ENSP00000293860	ENSP00000302160
ENSP00000293860	ENSP00000302478
ENSP00000293860	ENSP00000303088

ENSP00000293860	ENSP00000314949
ENSP00000293860	ENSP00000318604
ENSP00000293860	ENSP00000324124
ENSP00000293860	ENSP00000331111
ENSP00000293860	ENSP00000334188
ENSP00000293860	ENSP00000334564
ENSP00000293860	ENSP00000335357
ENSP00000293860	ENSP00000342889
ENSP00000293860	ENSP00000347345
ENSP00000293860	ENSP00000350512
ENSP00000293860	ENSP00000351575
ENSP00000293860	ENSP00000354916
ENSP00000293860	ENSP00000357887
ENSP00000293860	ENSP00000359976
ENSP00000293860	ENSP00000360515
ENSP00000293860	ENSP00000361465
ENSP00000293860	ENSP00000366828
ENSP00000293860	ENSP00000367029
ENSP00000293860	ENSP00000370968
ENSP00000293860	ENSP00000378736
ENSP00000293860	ENSP00000383503
ENSP00000293860	ENSP00000386134
ENSP00000293860	ENSP00000397636
ENSP00000293860	ENSP00000405636
ENSP00000293860	ENSP00000410530
ENSP00000293860	ENSP00000410954
ENSP00000293860	ENSP00000414110
ENSP00000293860	ENSP00000416599
ENSP00000293879	ENSP00000298937
ENSP00000293879	ENSP00000304593
ENSP00000293879	ENSP00000357907
ENSP00000293879	ENSP00000360473
ENSP00000293879	ENSP00000386759
ENSP00000293879	ENSP00000396937
ENSP00000293883	ENSP00000318861
ENSP00000293883	ENSP00000339881
ENSP00000293883	ENSP00000360286
ENSP00000293883	ENSP00000371169
ENSP00000293883	ENSP00000371546
ENSP00000293883	ENSP00000382779
ENSP00000294008	ENSP00000295897
ENSP00000294008	ENSP00000296805
ENSP00000294008	ENSP00000300086
ENSP00000294008	ENSP00000301585
ENSP00000294008	ENSP00000301634
ENSP00000294008	ENSP00000301788

ENSP00000294008	ENSP00000301843
ENSP00000294008	ENSP00000307525
ENSP00000294008	ENSP00000307853
ENSP00000294008	ENSP00000308546
ENSP00000294008	ENSP00000309555
ENSP00000294008	ENSP00000310448
ENSP00000294008	ENSP00000310520
ENSP00000294008	ENSP00000310668
ENSP00000294008	ENSP00000311245
ENSP00000294008	ENSP00000314311
ENSP00000294008	ENSP00000315383
ENSP00000294008	ENSP00000315397
ENSP00000294008	ENSP00000315931
ENSP00000294008	ENSP00000317376
ENSP00000294008	ENSP00000317904
ENSP00000294008	ENSP00000321503
ENSP00000294008	ENSP00000323527
ENSP00000294008	ENSP00000323687
ENSP00000294008	ENSP00000324122
ENSP00000294008	ENSP00000324422
ENSP00000294008	ENSP00000325002
ENSP00000294008	ENSP00000328690
ENSP00000294008	ENSP00000328940
ENSP00000294008	ENSP00000335557
ENSP00000294008	ENSP00000335808
ENSP00000294008	ENSP00000338814
ENSP00000294008	ENSP00000339897
ENSP00000294008	ENSP00000340278
ENSP00000294008	ENSP00000340879
ENSP00000294008	ENSP00000341138
ENSP00000294008	ENSP00000343701
ENSP00000294008	ENSP00000345895
ENSP00000294008	ENSP00000346437
ENSP00000294008	ENSP00000347408
ENSP00000294008	ENSP00000348163
ENSP00000294008	ENSP00000348565
ENSP00000294008	ENSP00000348577
ENSP00000294008	ENSP00000349486
ENSP00000294008	ENSP00000351416
ENSP00000294008	ENSP00000354045
ENSP00000294008	ENSP00000355607
ENSP00000294008	ENSP00000355759
ENSP00000294008	ENSP00000356807
ENSP00000294008	ENSP00000357597
ENSP00000294008	ENSP00000357789
ENSP00000294008	ENSP00000357823

ENSP00000294008	ENSP00000359225
ENSP00000294008	ENSP00000360498
ENSP00000294008	ENSP00000361658
ENSP00000294008	ENSP00000362036
ENSP00000294008	ENSP00000362041
ENSP00000294008	ENSP00000364037
ENSP00000294008	ENSP00000364847
ENSP00000294008	ENSP00000366863
ENSP00000294008	ENSP00000366953
ENSP00000294008	ENSP00000369042
ENSP00000294008	ENSP00000370543
ENSP00000294008	ENSP00000374455
ENSP00000294008	ENSP00000382779
ENSP00000294008	ENSP00000386520
ENSP00000294008	ENSP00000403359
ENSP00000294008	ENSP00000406888
ENSP00000294008	ENSP00000407401
ENSP00000294008	ENSP00000418823
ENSP00000294016	ENSP00000351015
ENSP00000294016	ENSP00000367208
ENSP00000294053	ENSP00000297185
ENSP00000294053	ENSP00000299767
ENSP00000294053	ENSP00000300107
ENSP00000294053	ENSP00000300648
ENSP00000294053	ENSP00000302896
ENSP00000294053	ENSP00000304102
ENSP00000294053	ENSP00000304370
ENSP00000294053	ENSP00000305958
ENSP00000294053	ENSP00000312356
ENSP00000294053	ENSP00000313490
ENSP00000294053	ENSP00000313603
ENSP00000294053	ENSP00000316240
ENSP00000294053	ENSP00000320650
ENSP00000294053	ENSP00000322570
ENSP00000294053	ENSP00000324792
ENSP00000294053	ENSP00000325448
ENSP00000294053	ENSP00000326981
ENSP00000294053	ENSP00000329558
ENSP00000294053	ENSP00000330343
ENSP00000294053	ENSP00000334044
ENSP00000294053	ENSP00000334148
ENSP00000294053	ENSP00000338533
ENSP00000294053	ENSP00000339484
ENSP00000294053	ENSP00000342026
ENSP00000294053	ENSP00000344431
ENSP00000294053	ENSP00000345793

ENSP00000294053	ENSP00000346791
ENSP00000294053	ENSP00000350708
ENSP00000294053	ENSP00000351697
ENSP00000294053	ENSP00000353826
ENSP00000294053	ENSP00000354977
ENSP00000294053	ENSP00000356811
ENSP00000294053	ENSP00000358478
ENSP00000294053	ENSP00000358812
ENSP00000294053	ENSP00000359345
ENSP00000294053	ENSP00000360329
ENSP00000294053	ENSP00000362041
ENSP00000294053	ENSP00000362413
ENSP00000294053	ENSP00000364815
ENSP00000294053	ENSP00000365007
ENSP00000294053	ENSP00000366179
ENSP00000294053	ENSP00000366249
ENSP00000294053	ENSP00000366395
ENSP00000294053	ENSP00000366620
ENSP00000294053	ENSP00000366800
ENSP00000294053	ENSP00000367208
ENSP00000294053	ENSP00000368349
ENSP00000294053	ENSP00000368523
ENSP00000294053	ENSP00000368646
ENSP00000294053	ENSP00000368699
ENSP00000294053	ENSP00000369038
ENSP00000294053	ENSP00000371451
ENSP00000294053	ENSP00000373039
ENSP00000294053	ENSP00000373082
ENSP00000294053	ENSP00000373918
ENSP00000294053	ENSP00000374280
ENSP00000294053	ENSP00000374455
ENSP00000294053	ENSP00000378605
ENSP00000294053	ENSP00000381003
ENSP00000294053	ENSP00000384211
ENSP00000294053	ENSP00000389065
ENSP00000294053	ENSP00000396152
ENSP00000294053	ENSP00000397441
ENSP00000294053	ENSP00000401121
ENSP00000294053	ENSP00000403359
ENSP00000294053	ENSP00000404381
ENSP00000294053	ENSP00000410416
ENSP00000294053	ENSP00000411327
ENSP00000294053	ENSP00000412553
ENSP00000294053	ENSP00000413319
ENSP00000294053	ENSP00000414373
ENSP00000294053	ENSP00000415316

ENSP00000294119	ENSP00000300896
ENSP00000294119	ENSP00000301764
ENSP00000294119	ENSP00000303279
ENSP00000294119	ENSP00000309031
ENSP00000294119	ENSP00000312981
ENSP00000294119	ENSP00000331487
ENSP00000294119	ENSP00000341551
ENSP00000294119	ENSP00000343001
ENSP00000294119	ENSP00000350283
ENSP00000294119	ENSP00000350708
ENSP00000294119	ENSP00000352408
ENSP00000294119	ENSP00000354775
ENSP00000294119	ENSP00000356146
ENSP00000294119	ENSP00000361990
ENSP00000294119	ENSP00000364403
ENSP00000294119	ENSP00000364486
ENSP00000294119	ENSP00000364794
ENSP00000294119	ENSP00000368797
ENSP00000294119	ENSP00000370258
ENSP00000294119	ENSP00000375881
ENSP00000294119	ENSP00000380460
ENSP00000294119	ENSP00000381045
ENSP00000294129	ENSP00000300574
ENSP00000294129	ENSP00000313829
ENSP00000294129	ENSP00000350512
ENSP00000294129	ENSP00000356694
ENSP00000294129	ENSP00000357650
ENSP00000294129	ENSP00000360500
ENSP00000294129	ENSP00000360798
ENSP00000294129	ENSP00000364893
ENSP00000294129	ENSP00000365891
ENSP00000294129	ENSP00000373347
ENSP00000294129	ENSP00000386722
ENSP00000294129	ENSP00000413625
ENSP00000294168	ENSP00000299424
ENSP00000294168	ENSP00000303315
ENSP00000294168	ENSP00000306983
ENSP00000294168	ENSP00000307684
ENSP00000294168	ENSP00000313420
ENSP00000294168	ENSP00000315791
ENSP00000294168	ENSP00000316114
ENSP00000294168	ENSP00000320949
ENSP00000294168	ENSP00000329933
ENSP00000294168	ENSP00000331310
ENSP00000294168	ENSP00000334061
ENSP00000294168	ENSP00000337405

ENSP00000294168	ENSP00000343741
ENSP00000294168	ENSP00000347733
ENSP00000294168	ENSP00000348010
ENSP00000294168	ENSP00000351575
ENSP00000294168	ENSP00000352121
ENSP00000294168	ENSP00000354558
ENSP00000294168	ENSP00000358886
ENSP00000294168	ENSP00000360515
ENSP00000294168	ENSP00000369695
ENSP00000294168	ENSP00000397259
ENSP00000294168	ENSP00000402515
ENSP00000294179	ENSP00000301281
ENSP00000294179	ENSP00000302176
ENSP00000294179	ENSP00000305255
ENSP00000294179	ENSP00000305647
ENSP00000294179	ENSP00000305810
ENSP00000294179	ENSP00000309031
ENSP00000294179	ENSP00000310521
ENSP00000294179	ENSP00000311134
ENSP00000294179	ENSP00000317714
ENSP00000294179	ENSP00000324628
ENSP00000294179	ENSP00000327309
ENSP00000294179	ENSP00000327716
ENSP00000294179	ENSP00000330945
ENSP00000294179	ENSP00000349131
ENSP00000294179	ENSP00000349887
ENSP00000294179	ENSP00000349970
ENSP00000294179	ENSP00000353073
ENSP00000294179	ENSP00000360183
ENSP00000294179	ENSP00000362399
ENSP00000294179	ENSP00000365943
ENSP00000294179	ENSP00000374529
ENSP00000294179	ENSP00000381293
ENSP00000294179	ENSP00000386918
ENSP00000294179	ENSP00000390783
ENSP00000294179	ENSP00000397441
ENSP00000294189	ENSP00000296674
ENSP00000294189	ENSP00000298283
ENSP00000294189	ENSP00000298310
ENSP00000294189	ENSP00000300151
ENSP00000294189	ENSP00000303315
ENSP00000294189	ENSP00000306548
ENSP00000294189	ENSP00000307889
ENSP00000294189	ENSP00000308897
ENSP00000294189	ENSP00000309334
ENSP00000294189	ENSP00000309830

ENSP00000294189	ENSP00000311430
ENSP00000294189	ENSP00000322419
ENSP00000294189	ENSP00000323687
ENSP00000294189	ENSP00000324122
ENSP00000294189	ENSP00000339095
ENSP00000294189	ENSP00000339795
ENSP00000294189	ENSP00000345156
ENSP00000294189	ENSP00000346015
ENSP00000294189	ENSP00000346022
ENSP00000294189	ENSP00000346027
ENSP00000294189	ENSP00000346037
ENSP00000294189	ENSP00000346088
ENSP00000294189	ENSP00000346120
ENSP00000294189	ENSP00000354525
ENSP00000294189	ENSP00000354739
ENSP00000294189	ENSP00000359345
ENSP00000294189	ENSP00000361076
ENSP00000294189	ENSP00000363018
ENSP00000294189	ENSP00000363676
ENSP00000294189	ENSP00000364320
ENSP00000294189	ENSP00000370589
ENSP00000294189	ENSP00000373404
ENSP00000294189	ENSP00000375730
ENSP00000294189	ENSP00000377640
ENSP00000294189	ENSP00000377865
ENSP00000294189	ENSP00000378160
ENSP00000294189	ENSP00000380156
ENSP00000294189	ENSP00000386717
ENSP00000294189	ENSP00000389103
ENSP00000294189	ENSP00000391723
ENSP00000294189	ENSP00000393393
ENSP00000294189	ENSP00000414321
ENSP00000294309	ENSP00000311200
ENSP00000294339	ENSP00000300574
ENSP00000294339	ENSP00000301459
ENSP00000294339	ENSP00000329357
ENSP00000294339	ENSP00000329715
ENSP00000294339	ENSP00000331057
ENSP00000294339	ENSP00000341024
ENSP00000294339	ENSP00000345681
ENSP00000294339	ENSP00000347184
ENSP00000294339	ENSP00000347733
ENSP00000294339	ENSP00000348657
ENSP00000294339	ENSP00000349049
ENSP00000294339	ENSP00000349723
ENSP00000294339	ENSP00000351250

ENSP00000294339	ENSP00000352257
ENSP00000294339	ENSP00000353622
ENSP00000294339	ENSP00000354522
ENSP00000294339	ENSP00000355759
ENSP00000294339	ENSP00000360371
ENSP00000294339	ENSP00000365877
ENSP00000294339	ENSP00000369716
ENSP00000294339	ENSP00000392466
ENSP00000294409	ENSP00000367408
ENSP00000294409	ENSP00000399968
ENSP00000294484	ENSP00000359100
ENSP00000294484	ENSP00000380702
ENSP00000294507	ENSP00000333298
ENSP00000294507	ENSP00000345530
ENSP00000294507	ENSP00000354782
ENSP00000294507	ENSP00000360798
ENSP00000294543	ENSP00000310551
ENSP00000294543	ENSP00000314036
ENSP00000294543	ENSP00000325369
ENSP00000294543	ENSP00000344055
ENSP00000294543	ENSP00000350199
ENSP00000294543	ENSP00000351926
ENSP00000294543	ENSP00000352603
ENSP00000294543	ENSP00000355963
ENSP00000294543	ENSP00000377148
ENSP00000294543	ENSP00000407879
ENSP00000294664	ENSP00000300249
ENSP00000294664	ENSP00000327009
ENSP00000294664	ENSP00000334188
ENSP00000294664	ENSP00000351665
ENSP00000294664	ENSP00000356579
ENSP00000294664	ENSP00000377793
ENSP00000294702	ENSP00000301280
ENSP00000294702	ENSP00000345681
ENSP00000294702	ENSP00000349049
ENSP00000294702	ENSP00000349465
ENSP00000294702	ENSP00000364895
ENSP00000294724	ENSP00000300413
ENSP00000294724	ENSP00000304226
ENSP00000294724	ENSP00000307900
ENSP00000294724	ENSP00000312735
ENSP00000294724	ENSP00000317904
ENSP00000294724	ENSP00000319341
ENSP00000294724	ENSP00000337838
ENSP00000294724	ENSP00000340019
ENSP00000294724	ENSP00000345464

ENSP00000294724	ENSP00000348455
ENSP00000294724	ENSP00000353622
ENSP00000294724	ENSP00000359100
ENSP00000294724	ENSP00000359691
ENSP00000294724	ENSP00000361418
ENSP00000294724	ENSP00000364813
ENSP00000294724	ENSP00000372883
ENSP00000294724	ENSP00000379607
ENSP00000294724	ENSP00000381932
ENSP00000294724	ENSP00000403345
ENSP00000294724	ENSP00000406280
ENSP00000294724	ENSP00000410758
ENSP00000294724	ENSP00000414006
ENSP00000294724	ENSP00000414634
ENSP00000294728	ENSP00000304915
ENSP00000294740	ENSP00000332931
ENSP00000294740	ENSP00000337340
ENSP00000294740	ENSP00000338157
ENSP00000294740	ENSP00000346986
ENSP00000294740	ENSP00000354782
ENSP00000294740	ENSP00000355601
ENSP00000294740	ENSP00000368253
ENSP00000294740	ENSP00000381031
ENSP00000294753	ENSP00000303088
ENSP00000294753	ENSP00000323074
ENSP00000294753	ENSP00000341280
ENSP00000294753	ENSP00000359837
ENSP00000294785	ENSP00000306864
ENSP00000294785	ENSP00000310729
ENSP00000294785	ENSP00000312435
ENSP00000294785	ENSP00000326366
ENSP00000294785	ENSP00000333298
ENSP00000294785	ENSP00000345229
ENSP00000294785	ENSP00000349022
ENSP00000294785	ENSP00000358105
ENSP00000294785	ENSP00000366395
ENSP00000294785	ENSP00000366460
ENSP00000294785	ENSP00000366603
ENSP00000294785	ENSP00000416097
ENSP00000294811	ENSP00000379607
ENSP00000294848	ENSP00000345681
ENSP00000294848	ENSP00000346065
ENSP00000294848	ENSP00000349208
ENSP00000294952	ENSP00000300738
ENSP00000294973	ENSP00000300086
ENSP00000294973	ENSP00000340879

ENSP00000294973	ENSP00000350249
ENSP00000294973	ENSP00000350928
ENSP00000294973	ENSP00000391723
ENSP00000294973	ENSP00000401508
ENSP00000294984	ENSP00000314976
ENSP00000295025	ENSP00000297185
ENSP00000295025	ENSP00000304311
ENSP00000295025	ENSP00000304803
ENSP00000295025	ENSP00000308236
ENSP00000295025	ENSP00000314556
ENSP00000295025	ENSP00000320758
ENSP00000295025	ENSP00000321203
ENSP00000295025	ENSP00000322532
ENSP00000295025	ENSP00000325612
ENSP00000295025	ENSP00000329357
ENSP00000295025	ENSP00000329967
ENSP00000295025	ENSP00000333122
ENSP00000295025	ENSP00000333633
ENSP00000295025	ENSP00000334061
ENSP00000295025	ENSP00000337065
ENSP00000295025	ENSP00000344456
ENSP00000295025	ENSP00000352516
ENSP00000295025	ENSP00000353622
ENSP00000295025	ENSP00000356623
ENSP00000295025	ENSP00000358997
ENSP00000295025	ENSP00000359531
ENSP00000295025	ENSP00000364893
ENSP00000295025	ENSP00000370736
ENSP00000295025	ENSP00000379607
ENSP00000295025	ENSP00000415836
ENSP00000295030	ENSP00000303511
ENSP00000295030	ENSP00000320885
ENSP00000295030	ENSP00000321449
ENSP00000295030	ENSP00000339435
ENSP00000295030	ENSP00000345702
ENSP00000295030	ENSP00000348762
ENSP00000295030	ENSP00000349016
ENSP00000295030	ENSP00000349543
ENSP00000295030	ENSP00000356563
ENSP00000295030	ENSP00000357051
ENSP00000295030	ENSP00000358312
ENSP00000295030	ENSP00000407401
ENSP00000295050	ENSP00000368438
ENSP00000295065	ENSP00000345988
ENSP00000295065	ENSP00000355596
ENSP00000295065	ENSP00000358799

ENSP00000295065	ENSP00000361076
ENSP00000295066	ENSP00000296490
ENSP00000295066	ENSP00000296786
ENSP00000295066	ENSP00000299601
ENSP00000295066	ENSP00000304593
ENSP00000295066	ENSP00000306983
ENSP00000295066	ENSP00000307208
ENSP00000295066	ENSP00000309141
ENSP00000295066	ENSP00000311496
ENSP00000295066	ENSP00000315379
ENSP00000295066	ENSP00000316109
ENSP00000295066	ENSP00000317087
ENSP00000295066	ENSP00000317334
ENSP00000295066	ENSP00000318222
ENSP00000295066	ENSP00000322242
ENSP00000295066	ENSP00000324792
ENSP00000295066	ENSP00000325677
ENSP00000295066	ENSP00000326261
ENSP00000295066	ENSP00000327716
ENSP00000295066	ENSP00000330343
ENSP00000295066	ENSP00000334188
ENSP00000295066	ENSP00000336868
ENSP00000295066	ENSP00000340896
ENSP00000295066	ENSP00000351596
ENSP00000295066	ENSP00000351894
ENSP00000295066	ENSP00000353622
ENSP00000295066	ENSP00000357887
ENSP00000295066	ENSP00000358147
ENSP00000295066	ENSP00000360515
ENSP00000295066	ENSP00000361276
ENSP00000295066	ENSP00000361748
ENSP00000295066	ENSP00000362127
ENSP00000295066	ENSP00000362166
ENSP00000295066	ENSP00000366135
ENSP00000295066	ENSP00000366434
ENSP00000295066	ENSP00000367029
ENSP00000295066	ENSP00000373340
ENSP00000295066	ENSP00000374213
ENSP00000295066	ENSP00000374529
ENSP00000295066	ENSP00000376946
ENSP00000295066	ENSP00000379217
ENSP00000295066	ENSP00000379607
ENSP00000295066	ENSP00000380460
ENSP00000295066	ENSP00000384048
ENSP00000295066	ENSP00000386759
ENSP00000295066	ENSP00000390475

ENSP00000295066	ENSP00000396749
ENSP00000295066	ENSP00000402240
ENSP00000295066	ENSP00000406293
ENSP00000295079	ENSP00000347948
ENSP00000295079	ENSP00000397435
ENSP00000295117	ENSP00000386722
ENSP00000295119	ENSP00000305255
ENSP00000295119	ENSP00000310668
ENSP00000295119	ENSP00000350512
ENSP00000295121	ENSP00000304350
ENSP00000295121	ENSP00000331087
ENSP00000295121	ENSP00000362546
ENSP00000295121	ENSP00000401566
ENSP00000295131	ENSP00000312735
ENSP00000295131	ENSP00000324124
ENSP00000295131	ENSP00000360116
ENSP00000295131	ENSP00000387006
ENSP00000295131	ENSP00000403852
ENSP00000295148	ENSP00000381045
ENSP00000295206	ENSP00000326296
ENSP00000295206	ENSP00000354586
ENSP00000295206	ENSP00000362588
ENSP00000295206	ENSP00000374240
ENSP00000295240	ENSP00000353701
ENSP00000295240	ENSP00000370031
ENSP00000295321	ENSP00000299601
ENSP00000295321	ENSP00000302478
ENSP00000295321	ENSP00000307491
ENSP00000295321	ENSP00000312735
ENSP00000295321	ENSP00000314949
ENSP00000295321	ENSP00000315768
ENSP00000295321	ENSP00000319104
ENSP00000295321	ENSP00000349213
ENSP00000295321	ENSP00000351250
ENSP00000295321	ENSP00000361465
ENSP00000295321	ENSP00000362900
ENSP00000295321	ENSP00000374280
ENSP00000295321	ENSP00000386759
ENSP00000295367	ENSP00000301607
ENSP00000295367	ENSP00000357731
ENSP00000295367	ENSP00000357753
ENSP00000295373	ENSP00000297591
ENSP00000295373	ENSP00000297990
ENSP00000295373	ENSP00000315674
ENSP00000295373	ENSP00000328854
ENSP00000295373	ENSP00000329466

ENSP00000295373	ENSP00000345917
ENSP00000295373	ENSP00000346120
ENSP00000295373	ENSP00000355541
ENSP00000295373	ENSP00000358812
ENSP00000295373	ENSP00000360031
ENSP00000295373	ENSP00000360532
ENSP00000295408	ENSP00000329668
ENSP00000295408	ENSP00000331831
ENSP00000295408	ENSP00000343785
ENSP00000295408	ENSP00000363708
ENSP00000295408	ENSP00000371341
ENSP00000295408	ENSP00000379372
ENSP00000295440	ENSP00000398028
ENSP00000295488	ENSP00000296805
ENSP00000295488	ENSP00000298139
ENSP00000295488	ENSP00000298746
ENSP00000295488	ENSP00000302160
ENSP00000295488	ENSP00000310448
ENSP00000295488	ENSP00000315379
ENSP00000295488	ENSP00000317636
ENSP00000295488	ENSP00000324122
ENSP00000295488	ENSP00000331087
ENSP00000295488	ENSP00000335321
ENSP00000295488	ENSP00000338788
ENSP00000295488	ENSP00000343557
ENSP00000295488	ENSP00000351137
ENSP00000295488	ENSP00000351492
ENSP00000295488	ENSP00000357631
ENSP00000295488	ENSP00000358554
ENSP00000295488	ENSP00000358563
ENSP00000295488	ENSP00000358810
ENSP00000295488	ENSP00000361982
ENSP00000295488	ENSP00000362110
ENSP00000295488	ENSP00000364289
ENSP00000295488	ENSP00000364543
ENSP00000295488	ENSP00000366557
ENSP00000295488	ENSP00000367835
ENSP00000295488	ENSP00000368565
ENSP00000295488	ENSP00000370589
ENSP00000295488	ENSP00000372316
ENSP00000295488	ENSP00000372827
ENSP00000295488	ENSP00000376973
ENSP00000295488	ENSP00000387006
ENSP00000295488	ENSP00000393953
ENSP00000295488	ENSP00000394400
ENSP00000295488	ENSP00000399530

ENSP00000295488	ENSP00000400626
ENSP00000295488	ENSP00000401566
ENSP00000295488	ENSP00000412310
ENSP00000295491	ENSP00000337127
ENSP00000295491	ENSP00000341692
ENSP00000295491	ENSP00000359000
ENSP00000295491	ENSP00000362036
ENSP00000295491	ENSP00000362208
ENSP00000295491	ENSP00000372445
ENSP00000295550	ENSP00000355180
ENSP00000295633	ENSP00000304236
ENSP00000295640	ENSP00000329466
ENSP00000295640	ENSP00000360147
ENSP00000295640	ENSP00000370517
ENSP00000295641	ENSP00000341551
ENSP00000295641	ENSP00000363089
ENSP00000295645	ENSP00000311343
ENSP00000295645	ENSP00000321320
ENSP00000295645	ENSP00000343190
ENSP00000295645	ENSP00000353998
ENSP00000295645	ENSP00000369335
ENSP00000295682	ENSP00000341213
ENSP00000295682	ENSP00000344106
ENSP00000295682	ENSP00000345656
ENSP00000295682	ENSP00000346874
ENSP00000295682	ENSP00000397435
ENSP00000295685	ENSP00000298910
ENSP00000295685	ENSP00000301843
ENSP00000295685	ENSP00000326238
ENSP00000295685	ENSP00000330343
ENSP00000295685	ENSP00000331201
ENSP00000295685	ENSP00000337340
ENSP00000295685	ENSP00000341243
ENSP00000295685	ENSP00000349465
ENSP00000295685	ENSP00000350757
ENSP00000295685	ENSP00000360821
ENSP00000295685	ENSP00000365025
ENSP00000295685	ENSP00000365147
ENSP00000295685	ENSP00000365891
ENSP00000295685	ENSP00000373600
ENSP00000295685	ENSP00000380431
ENSP00000295685	ENSP00000382177
ENSP00000295702	ENSP00000296255
ENSP00000295702	ENSP00000299767
ENSP00000295702	ENSP00000317331
ENSP00000295702	ENSP00000327070

ENSP00000295702	ENSP00000328023
ENSP00000295702	ENSP00000349142
ENSP00000295702	ENSP00000361686
ENSP00000295702	ENSP00000364188
ENSP00000295702	ENSP00000370607
ENSP00000295702	ENSP00000386918
ENSP00000295704	ENSP00000360116
ENSP00000295709	ENSP00000297261
ENSP00000295709	ENSP00000332353
ENSP00000295709	ENSP00000340954
ENSP00000295709	ENSP00000343818
ENSP00000295709	ENSP00000345530
ENSP00000295709	ENSP00000354586
ENSP00000295709	ENSP00000355866
ENSP00000295709	ENSP00000358918
ENSP00000295709	ENSP00000379228
ENSP00000295743	ENSP00000297261
ENSP00000295743	ENSP00000305459
ENSP00000295746	ENSP00000378350
ENSP00000295755	ENSP00000334061
ENSP00000295756	ENSP00000334061
ENSP00000295756	ENSP00000354566
ENSP00000295756	ENSP00000354782
ENSP00000295767	ENSP00000299138
ENSP00000295767	ENSP00000307786
ENSP00000295767	ENSP00000320236
ENSP00000295767	ENSP00000321239
ENSP00000295767	ENSP00000325136
ENSP00000295767	ENSP00000342015
ENSP00000295767	ENSP00000355518
ENSP00000295767	ENSP00000360782
ENSP00000295767	ENSP00000361993
ENSP00000295767	ENSP00000365766
ENSP00000295767	ENSP00000368066
ENSP00000295767	ENSP00000377914
ENSP00000295767	ENSP00000387262
ENSP00000295770	ENSP00000296255
ENSP00000295770	ENSP00000299626
ENSP00000295770	ENSP00000319979
ENSP00000295770	ENSP00000326366
ENSP00000295770	ENSP00000331288
ENSP00000295770	ENSP00000333813
ENSP00000295770	ENSP00000340466
ENSP00000295770	ENSP00000344460
ENSP00000295770	ENSP00000360149
ENSP00000295770	ENSP00000360644

ENSP00000295770	ENSP00000362688
ENSP00000295770	ENSP00000364188
ENSP00000295770	ENSP00000366748
ENSP00000295770	ENSP00000380793
ENSP00000295770	ENSP00000385347
ENSP00000295824	ENSP00000340879
ENSP00000295824	ENSP00000355167
ENSP00000295886	ENSP00000403817
ENSP00000295887	ENSP00000304500
ENSP00000295887	ENSP00000345528
ENSP00000295887	ENSP00000361057
ENSP00000295887	ENSP00000364188
ENSP00000295887	ENSP00000370607
ENSP00000295888	ENSP00000338345
ENSP00000295888	ENSP00000343313
ENSP00000295888	ENSP00000347184
ENSP00000295888	ENSP00000374455
ENSP00000295890	ENSP00000298569
ENSP00000295890	ENSP00000317159
ENSP00000295890	ENSP00000318115
ENSP00000295890	ENSP00000319323
ENSP00000295890	ENSP00000320236
ENSP00000295890	ENSP00000328023
ENSP00000295890	ENSP00000332887
ENSP00000295890	ENSP00000345096
ENSP00000295890	ENSP00000346142
ENSP00000295890	ENSP00000348886
ENSP00000295890	ENSP00000352219
ENSP00000295890	ENSP00000354499
ENSP00000295890	ENSP00000354554
ENSP00000295890	ENSP00000354632
ENSP00000295890	ENSP00000354876
ENSP00000295890	ENSP00000354982
ENSP00000295890	ENSP00000356329
ENSP00000295890	ENSP00000356587
ENSP00000295890	ENSP00000356972
ENSP00000295890	ENSP00000358737
ENSP00000295890	ENSP00000365766
ENSP00000295890	ENSP00000366800
ENSP00000295890	ENSP00000370930
ENSP00000295890	ENSP00000371607
ENSP00000295897	ENSP00000296028
ENSP00000295897	ENSP00000296129
ENSP00000295897	ENSP00000300648
ENSP00000295897	ENSP00000301764
ENSP00000295897	ENSP00000303476

ENSP00000295897	ENSP00000303928
ENSP00000295897	ENSP00000306361
ENSP00000295897	ENSP00000306522
ENSP00000295897	ENSP00000310338
ENSP00000295897	ENSP00000311505
ENSP00000295897	ENSP00000319623
ENSP00000295897	ENSP00000320885
ENSP00000295897	ENSP00000320949
ENSP00000295897	ENSP00000325546
ENSP00000295897	ENSP00000326581
ENSP00000295897	ENSP00000328968
ENSP00000295897	ENSP00000335371
ENSP00000295897	ENSP00000339186
ENSP00000295897	ENSP00000341280
ENSP00000295897	ENSP00000343745
ENSP00000295897	ENSP00000346634
ENSP00000295897	ENSP00000346839
ENSP00000295897	ENSP00000350425
ENSP00000295897	ENSP00000350447
ENSP00000295897	ENSP00000353741
ENSP00000295897	ENSP00000353769
ENSP00000295897	ENSP00000354119
ENSP00000295897	ENSP00000354159
ENSP00000295897	ENSP00000355296
ENSP00000295897	ENSP00000355675
ENSP00000295897	ENSP00000356969
ENSP00000295897	ENSP00000358590
ENSP00000295897	ENSP00000359596
ENSP00000295897	ENSP00000360519
ENSP00000295897	ENSP00000366434
ENSP00000295897	ENSP00000367064
ENSP00000295897	ENSP00000368190
ENSP00000295897	ENSP00000371729
ENSP00000295897	ENSP00000373301
ENSP00000295897	ENSP00000378288
ENSP00000295897	ENSP00000379607
ENSP00000295897	ENSP00000387262
ENSP00000295897	ENSP00000389427
ENSP00000295897	ENSP00000393887
ENSP00000295897	ENSP00000396688
ENSP00000295897	ENSP00000397435
ENSP00000295897	ENSP00000398617
ENSP00000295897	ENSP00000411286
ENSP00000295897	ENSP00000416561
ENSP00000295898	ENSP00000379607
ENSP00000295899	ENSP00000342343

ENSP00000295899	ENSP00000362948
ENSP00000295899	ENSP00000367265
ENSP00000295899	ENSP00000380969
ENSP00000295899	ENSP00000386394
ENSP00000295901	ENSP00000298852
ENSP00000295901	ENSP00000304668
ENSP00000295901	ENSP00000307491
ENSP00000295901	ENSP00000309198
ENSP00000295901	ENSP00000309474
ENSP00000295901	ENSP00000310129
ENSP00000295901	ENSP00000311665
ENSP00000295901	ENSP00000321711
ENSP00000295901	ENSP00000328940
ENSP00000295901	ENSP00000348442
ENSP00000295901	ENSP00000350708
ENSP00000295901	ENSP00000356290
ENSP00000295901	ENSP00000359727
ENSP00000295901	ENSP00000360821
ENSP00000295901	ENSP00000362334
ENSP00000295901	ENSP00000369497
ENSP00000295901	ENSP00000370258
ENSP00000295901	ENSP00000370419
ENSP00000295901	ENSP00000371341
ENSP00000295901	ENSP00000375809
ENSP00000295901	ENSP00000378350
ENSP00000295901	ENSP00000378529
ENSP00000295901	ENSP00000381891
ENSP00000295901	ENSP00000384211
ENSP00000295901	ENSP00000386212
ENSP00000295901	ENSP00000396937
ENSP00000295902	ENSP00000310800
ENSP00000295902	ENSP00000313309
ENSP00000295902	ENSP00000333950
ENSP00000295902	ENSP00000334003
ENSP00000295902	ENSP00000366121
ENSP00000295902	ENSP00000396771
ENSP00000295926	ENSP00000299601
ENSP00000295926	ENSP00000302160
ENSP00000295926	ENSP00000318944
ENSP00000295926	ENSP00000320949
ENSP00000295926	ENSP00000323720
ENSP00000295926	ENSP00000325794
ENSP00000295926	ENSP00000342755
ENSP00000295926	ENSP00000348565
ENSP00000295926	ENSP00000353622
ENSP00000295926	ENSP00000354673

ENSP00000295926	ENSP00000356641
ENSP00000295926	ENSP00000358854
ENSP00000295926	ENSP00000381657
ENSP00000295926	ENSP00000386239
ENSP00000295930	ENSP00000296674
ENSP00000295930	ENSP00000302896
ENSP00000295930	ENSP00000311028
ENSP00000295930	ENSP00000318646
ENSP00000295930	ENSP00000323856
ENSP00000295930	ENSP00000331019
ENSP00000295930	ENSP00000339095
ENSP00000295930	ENSP00000341885
ENSP00000295930	ENSP00000346050
ENSP00000295930	ENSP00000346067
ENSP00000295930	ENSP00000362744
ENSP00000295930	ENSP00000363308
ENSP00000295930	ENSP00000364813
ENSP00000295930	ENSP00000369757
ENSP00000295930	ENSP00000379888
ENSP00000295930	ENSP00000393241
ENSP00000295930	ENSP00000394085
ENSP00000295930	ENSP00000403175
ENSP00000295930	ENSP00000412583
ENSP00000295930	ENSP00000414006
ENSP00000295930	ENSP00000414321
ENSP00000295930	ENSP00000416110
ENSP00000295951	ENSP00000296509
ENSP00000295951	ENSP00000296783
ENSP00000295951	ENSP00000297325
ENSP00000295951	ENSP00000298317
ENSP00000295951	ENSP00000299853
ENSP00000295951	ENSP00000300249
ENSP00000295951	ENSP00000300584
ENSP00000295951	ENSP00000301019
ENSP00000295951	ENSP00000310649
ENSP00000295951	ENSP00000314499
ENSP00000295951	ENSP00000315702
ENSP00000295951	ENSP00000318262
ENSP00000295951	ENSP00000322915
ENSP00000295951	ENSP00000323288
ENSP00000295951	ENSP00000324020
ENSP00000295951	ENSP00000324956
ENSP00000295951	ENSP00000326296
ENSP00000295951	ENSP00000329933
ENSP00000295951	ENSP00000335357
ENSP00000295951	ENSP00000336587

ENSP00000295951	ENSP00000340305
ENSP00000295951	ENSP00000341848
ENSP00000295951	ENSP00000343362
ENSP00000295951	ENSP00000346255
ENSP00000295951	ENSP00000348965
ENSP00000295951	ENSP00000350071
ENSP00000295951	ENSP00000350094
ENSP00000295951	ENSP00000350708
ENSP00000295951	ENSP00000352676
ENSP00000295951	ENSP00000354158
ENSP00000295951	ENSP00000354486
ENSP00000295951	ENSP00000354558
ENSP00000295951	ENSP00000354673
ENSP00000295951	ENSP00000354916
ENSP00000295951	ENSP00000355388
ENSP00000295951	ENSP00000355922
ENSP00000295951	ENSP00000358810
ENSP00000295951	ENSP00000361465
ENSP00000295951	ENSP00000371388
ENSP00000295951	ENSP00000371888
ENSP00000295951	ENSP00000373331
ENSP00000295951	ENSP00000384015
ENSP00000295951	ENSP00000402084
ENSP00000295951	ENSP00000403397
ENSP00000295951	ENSP00000414402
ENSP00000295951	ENSP00000417963
ENSP00000295959	ENSP00000317691
ENSP00000295959	ENSP00000350098
ENSP00000295959	ENSP00000367439
ENSP00000295959	ENSP00000369391
ENSP00000295959	ENSP00000389182
ENSP00000295974	ENSP00000341551
ENSP00000295981	ENSP00000320936
ENSP00000295981	ENSP00000337432
ENSP00000295981	ENSP00000345984
ENSP00000295987	ENSP00000300574
ENSP00000295987	ENSP00000320340
ENSP00000295987	ENSP00000320758
ENSP00000295987	ENSP00000344055
ENSP00000295987	ENSP00000347232
ENSP00000295987	ENSP00000348602
ENSP00000295987	ENSP00000354158
ENSP00000295987	ENSP00000355133
ENSP00000295987	ENSP00000355865
ENSP00000295987	ENSP00000361824
ENSP00000295987	ENSP00000362399

ENSP00000295987	ENSP00000367408
ENSP00000295989	ENSP00000300648
ENSP00000295989	ENSP00000302790
ENSP00000295989	ENSP00000304500
ENSP00000295989	ENSP00000309198
ENSP00000295989	ENSP00000360621
ENSP00000295989	ENSP00000369042
ENSP00000296015	ENSP00000296215
ENSP00000296015	ENSP00000301740
ENSP00000296015	ENSP00000318861
ENSP00000296015	ENSP00000360464
ENSP00000296015	ENSP00000360488
ENSP00000296015	ENSP00000396301
ENSP00000296028	ENSP00000300651
ENSP00000296028	ENSP00000325002
ENSP00000296028	ENSP00000358042
ENSP00000296043	ENSP00000360532
ENSP00000296084	ENSP00000296930
ENSP00000296084	ENSP00000299138
ENSP00000296084	ENSP00000310668
ENSP00000296084	ENSP00000313420
ENSP00000296084	ENSP00000318115
ENSP00000296084	ENSP00000320084
ENSP00000296084	ENSP00000322730
ENSP00000296084	ENSP00000337353
ENSP00000296084	ENSP00000341637
ENSP00000296084	ENSP00000344456
ENSP00000296084	ENSP00000347581
ENSP00000296084	ENSP00000348965
ENSP00000296084	ENSP00000349932
ENSP00000296084	ENSP00000352425
ENSP00000296084	ENSP00000353582
ENSP00000296084	ENSP00000354251
ENSP00000296084	ENSP00000355759
ENSP00000296084	ENSP00000358293
ENSP00000296084	ENSP00000362656
ENSP00000296084	ENSP00000363763
ENSP00000296084	ENSP00000364860
ENSP00000296084	ENSP00000366221
ENSP00000296084	ENSP00000368438
ENSP00000296084	ENSP00000377862
ENSP00000296084	ENSP00000406367
ENSP00000296096	ENSP00000331057
ENSP00000296099	ENSP00000312652
ENSP00000296102	ENSP00000300151
ENSP00000296102	ENSP00000305653

ENSP00000296102	ENSP00000308897
ENSP00000296102	ENSP00000312311
ENSP00000296102	ENSP00000354525
ENSP00000296102	ENSP00000357048
ENSP00000296102	ENSP00000372093
ENSP00000296102	ENSP00000373404
ENSP00000296137	ENSP00000311505
ENSP00000296137	ENSP00000314544
ENSP00000296137	ENSP00000328720
ENSP00000296137	ENSP00000349785
ENSP00000296137	ENSP00000353375
ENSP00000296137	ENSP00000357283
ENSP00000296137	ENSP00000358423
ENSP00000296137	ENSP00000358857
ENSP00000296145	ENSP00000296875
ENSP00000296145	ENSP00000320309
ENSP00000296145	ENSP00000336762
ENSP00000296149	ENSP00000353622
ENSP00000296154	ENSP00000306124
ENSP00000296154	ENSP00000342667
ENSP00000296154	ENSP00000388035
ENSP00000296154	ENSP00000406066
ENSP00000296154	ENSP00000411286
ENSP00000296161	ENSP00000347836
ENSP00000296215	ENSP00000301740
ENSP00000296215	ENSP00000302530
ENSP00000296215	ENSP00000310448
ENSP00000296215	ENSP00000315379
ENSP00000296215	ENSP00000315859
ENSP00000296215	ENSP00000324274
ENSP00000296215	ENSP00000325863
ENSP00000296215	ENSP00000335321
ENSP00000296215	ENSP00000339090
ENSP00000296215	ENSP00000339845
ENSP00000296215	ENSP00000341551
ENSP00000296215	ENSP00000343741
ENSP00000296215	ENSP00000343745
ENSP00000296215	ENSP00000351490
ENSP00000296215	ENSP00000351492
ENSP00000296215	ENSP00000353735
ENSP00000296215	ENSP00000356920
ENSP00000296215	ENSP00000359518
ENSP00000296215	ENSP00000370460
ENSP00000296215	ENSP00000373657
ENSP00000296215	ENSP00000377262
ENSP00000296215	ENSP00000382177

ENSP00000296215	ENSP00000396301
ENSP00000296218	ENSP00000337340
ENSP00000296218	ENSP00000347184
ENSP00000296233	ENSP00000398789
ENSP00000296238	ENSP00000310561
ENSP00000296255	ENSP00000299626
ENSP00000296255	ENSP00000299767
ENSP00000296255	ENSP00000304592
ENSP00000296255	ENSP00000306124
ENSP00000296255	ENSP00000317331
ENSP00000296255	ENSP00000317334
ENSP00000296255	ENSP00000319713
ENSP00000296255	ENSP00000319979
ENSP00000296255	ENSP00000320508
ENSP00000296255	ENSP00000332887
ENSP00000296255	ENSP00000333656
ENSP00000296255	ENSP00000333813
ENSP00000296255	ENSP00000335557
ENSP00000296255	ENSP00000337053
ENSP00000296255	ENSP00000339495
ENSP00000296255	ENSP00000339933
ENSP00000296255	ENSP00000340466
ENSP00000296255	ENSP00000341213
ENSP00000296255	ENSP00000341538
ENSP00000296255	ENSP00000347495
ENSP00000296255	ENSP00000356530
ENSP00000296255	ENSP00000359552
ENSP00000296255	ENSP00000360149
ENSP00000296255	ENSP00000360270
ENSP00000296255	ENSP00000360644
ENSP00000296255	ENSP00000361057
ENSP00000296255	ENSP00000361845
ENSP00000296255	ENSP00000362592
ENSP00000296255	ENSP00000362688
ENSP00000296255	ENSP00000364188
ENSP00000296255	ENSP00000370883
ENSP00000296255	ENSP00000377527
ENSP00000296255	ENSP00000379042
ENSP00000296255	ENSP00000380793
ENSP00000296255	ENSP00000382779
ENSP00000296255	ENSP00000401445
ENSP00000296257	ENSP00000296509
ENSP00000296257	ENSP00000298139
ENSP00000296257	ENSP00000308622
ENSP00000296257	ENSP00000313851
ENSP00000296257	ENSP00000316032

ENSP00000296257	ENSP00000317636
ENSP00000296257	ENSP00000318977
ENSP00000296257	ENSP00000322242
ENSP00000296257	ENSP00000325863
ENSP00000296257	ENSP00000330343
ENSP00000296257	ENSP00000331817
ENSP00000296257	ENSP00000336606
ENSP00000296257	ENSP00000336712
ENSP00000296257	ENSP00000343023
ENSP00000296257	ENSP00000343701
ENSP00000296257	ENSP00000343818
ENSP00000296257	ENSP00000346453
ENSP00000296257	ENSP00000347232
ENSP00000296257	ENSP00000347979
ENSP00000296257	ENSP00000348577
ENSP00000296257	ENSP00000350283
ENSP00000296257	ENSP00000351284
ENSP00000296257	ENSP00000352257
ENSP00000296257	ENSP00000354522
ENSP00000296257	ENSP00000356448
ENSP00000296257	ENSP00000359221
ENSP00000296257	ENSP00000361162
ENSP00000296257	ENSP00000365811
ENSP00000296257	ENSP00000368391
ENSP00000296257	ENSP00000369335
ENSP00000296257	ENSP00000369716
ENSP00000296257	ENSP00000376611
ENSP00000296257	ENSP00000376799
ENSP00000296257	ENSP00000377655
ENSP00000296257	ENSP00000396704
ENSP00000296257	ENSP00000402527
ENSP00000296257	ENSP00000417281
ENSP00000296266	ENSP00000311505
ENSP00000296266	ENSP00000362413
ENSP00000296273	ENSP00000296509
ENSP00000296273	ENSP00000300738
ENSP00000296273	ENSP00000302530
ENSP00000296273	ENSP00000306999
ENSP00000296273	ENSP00000307853
ENSP00000296273	ENSP00000308546
ENSP00000296273	ENSP00000309572
ENSP00000296273	ENSP00000310520
ENSP00000296273	ENSP00000311873
ENSP00000296273	ENSP00000313171
ENSP00000296273	ENSP00000317334
ENSP00000296273	ENSP00000318977

ENSP00000296273	ENSP00000321636
ENSP00000296273	ENSP00000322570
ENSP00000296273	ENSP00000330343
ENSP00000296273	ENSP00000337797
ENSP00000296273	ENSP00000339145
ENSP00000296273	ENSP00000343392
ENSP00000296273	ENSP00000343745
ENSP00000296273	ENSP00000344456
ENSP00000296273	ENSP00000347719
ENSP00000296273	ENSP00000347978
ENSP00000296273	ENSP00000349393
ENSP00000296273	ENSP00000349465
ENSP00000296273	ENSP00000350491
ENSP00000296273	ENSP00000351284
ENSP00000296273	ENSP00000351697
ENSP00000296273	ENSP00000351947
ENSP00000296273	ENSP00000352257
ENSP00000296273	ENSP00000354497
ENSP00000296273	ENSP00000354522
ENSP00000296273	ENSP00000355560
ENSP00000296273	ENSP00000355759
ENSP00000296273	ENSP00000356989
ENSP00000296273	ENSP00000360310
ENSP00000296273	ENSP00000360532
ENSP00000296273	ENSP00000360621
ENSP00000296273	ENSP00000363021
ENSP00000296273	ENSP00000364270
ENSP00000296273	ENSP00000366434
ENSP00000296273	ENSP00000366974
ENSP00000296273	ENSP00000367787
ENSP00000296273	ENSP00000368438
ENSP00000296273	ENSP00000368646
ENSP00000296273	ENSP00000369335
ENSP00000296273	ENSP00000369411
ENSP00000296273	ENSP00000370150
ENSP00000296273	ENSP00000370151
ENSP00000296273	ENSP00000371419
ENSP00000296273	ENSP00000374213
ENSP00000296273	ENSP00000377384
ENSP00000296273	ENSP00000377793
ENSP00000296273	ENSP00000382562
ENSP00000296273	ENSP00000384048
ENSP00000296273	ENSP00000396704
ENSP00000296273	ENSP00000406490
ENSP00000296273	ENSP00000420588
ENSP00000296292	ENSP00000296328

ENSP00000296292	ENSP00000314949
ENSP00000296292	ENSP00000354977
ENSP00000296292	ENSP00000355013
ENSP00000296292	ENSP00000360621
ENSP00000296292	ENSP00000371475
ENSP00000296292	ENSP00000417764
ENSP00000296318	ENSP00000358335
ENSP00000296328	ENSP00000298281
ENSP00000296328	ENSP00000298852
ENSP00000296328	ENSP00000299853
ENSP00000296328	ENSP00000301764
ENSP00000296328	ENSP00000302478
ENSP00000296328	ENSP00000303088
ENSP00000296328	ENSP00000303315
ENSP00000296328	ENSP00000309142
ENSP00000296328	ENSP00000310129
ENSP00000296328	ENSP00000311005
ENSP00000296328	ENSP00000315791
ENSP00000296328	ENSP00000318604
ENSP00000296328	ENSP00000323720
ENSP00000296328	ENSP00000325677
ENSP00000296328	ENSP00000329933
ENSP00000296328	ENSP00000330753
ENSP00000296328	ENSP00000331487
ENSP00000296328	ENSP00000334564
ENSP00000296328	ENSP00000337405
ENSP00000296328	ENSP00000338413
ENSP00000296328	ENSP00000340896
ENSP00000296328	ENSP00000342793
ENSP00000296328	ENSP00000347345
ENSP00000296328	ENSP00000347733
ENSP00000296328	ENSP00000347997
ENSP00000296328	ENSP00000350708
ENSP00000296328	ENSP00000350967
ENSP00000296328	ENSP00000351885
ENSP00000296328	ENSP00000353735
ENSP00000296328	ENSP00000354775
ENSP00000296328	ENSP00000354883
ENSP00000296328	ENSP00000356146
ENSP00000296328	ENSP00000356470
ENSP00000296328	ENSP00000357602
ENSP00000296328	ENSP00000358497
ENSP00000296328	ENSP00000359290
ENSP00000296328	ENSP00000359688
ENSP00000296328	ENSP00000359818
ENSP00000296328	ENSP00000360116

ENSP00000296328	ENSP00000360183
ENSP00000296328	ENSP00000360676
ENSP00000296328	ENSP00000361465
ENSP00000296328	ENSP00000361990
ENSP00000296328	ENSP00000362334
ENSP00000296328	ENSP00000362900
ENSP00000296328	ENSP00000363779
ENSP00000296328	ENSP00000364289
ENSP00000296328	ENSP00000364403
ENSP00000296328	ENSP00000366135
ENSP00000296328	ENSP00000366828
ENSP00000296328	ENSP00000368797
ENSP00000296328	ENSP00000369531
ENSP00000296328	ENSP00000370105
ENSP00000296328	ENSP00000375809
ENSP00000296328	ENSP00000375881
ENSP00000296328	ENSP00000378736
ENSP00000296328	ENSP00000379372
ENSP00000296328	ENSP00000380460
ENSP00000296328	ENSP00000384109
ENSP00000296328	ENSP00000395574
ENSP00000296343	ENSP00000348455
ENSP00000296343	ENSP00000355231
ENSP00000296343	ENSP00000367265
ENSP00000296350	ENSP00000363873
ENSP00000296350	ENSP00000367064
ENSP00000296388	ENSP00000307491
ENSP00000296411	ENSP00000296802
ENSP00000296411	ENSP00000307525
ENSP00000296411	ENSP00000310042
ENSP00000296411	ENSP00000310668
ENSP00000296411	ENSP00000325312
ENSP00000296411	ENSP00000327436
ENSP00000296411	ENSP00000329189
ENSP00000296411	ENSP00000339145
ENSP00000296411	ENSP00000339795
ENSP00000296411	ENSP00000346015
ENSP00000296411	ENSP00000346022
ENSP00000296411	ENSP00000346453
ENSP00000296411	ENSP00000346725
ENSP00000296411	ENSP00000348596
ENSP00000296411	ENSP00000348915
ENSP00000296411	ENSP00000351380
ENSP00000296411	ENSP00000354040
ENSP00000296411	ENSP00000358400
ENSP00000296411	ENSP00000359345

ENSP00000296411	ENSP00000360412
ENSP00000296411	ENSP00000361076
ENSP00000296411	ENSP00000362153
ENSP00000296411	ENSP00000363384
ENSP00000296411	ENSP00000363559
ENSP00000296411	ENSP00000364037
ENSP00000296411	ENSP00000370968
ENSP00000296411	ENSP00000377954
ENSP00000296411	ENSP00000379888
ENSP00000296411	ENSP00000386717
ENSP00000296411	ENSP00000389103
ENSP00000296411	ENSP00000402338
ENSP00000296440	ENSP00000296474
ENSP00000296440	ENSP00000353582
ENSP00000296440	ENSP00000380942
ENSP00000296444	ENSP00000350512
ENSP00000296444	ENSP00000417764
ENSP00000296452	ENSP00000357643
ENSP00000296456	ENSP00000310440
ENSP00000296456	ENSP00000330408
ENSP00000296456	ENSP00000348565
ENSP00000296456	ENSP00000369816
ENSP00000296456	ENSP00000386227
ENSP00000296474	ENSP00000298229
ENSP00000296474	ENSP00000343785
ENSP00000296474	ENSP00000379372
ENSP00000296490	ENSP00000301329
ENSP00000296490	ENSP00000308466
ENSP00000296490	ENSP00000309555
ENSP00000296490	ENSP00000311768
ENSP00000296490	ENSP00000312735
ENSP00000296490	ENSP00000314949
ENSP00000296490	ENSP00000315791
ENSP00000296490	ENSP00000317334
ENSP00000296490	ENSP00000317872
ENSP00000296490	ENSP00000325377
ENSP00000296490	ENSP00000325677
ENSP00000296490	ENSP00000331817
ENSP00000296490	ENSP00000332444
ENSP00000296490	ENSP00000336752
ENSP00000296490	ENSP00000336868
ENSP00000296490	ENSP00000339353
ENSP00000296490	ENSP00000340019
ENSP00000296490	ENSP00000340896
ENSP00000296490	ENSP00000344504
ENSP00000296490	ENSP00000349022

ENSP00000296490	ENSP00000360116
ENSP00000296490	ENSP00000360286
ENSP00000296490	ENSP00000362900
ENSP00000296490	ENSP00000366829
ENSP00000296490	ENSP00000371475
ENSP00000296490	ENSP00000373509
ENSP00000296490	ENSP00000381657
ENSP00000296490	ENSP00000384048
ENSP00000296490	ENSP00000384109
ENSP00000296490	ENSP00000390475
ENSP00000296490	ENSP00000391723
ENSP00000296490	ENSP00000407181
ENSP00000296490	ENSP00000411851
ENSP00000296504	ENSP00000329933
ENSP00000296504	ENSP00000339723
ENSP00000296504	ENSP00000340494
ENSP00000296504	ENSP00000344537
ENSP00000296504	ENSP00000347184
ENSP00000296504	ENSP00000347592
ENSP00000296504	ENSP00000347602
ENSP00000296504	ENSP00000348551
ENSP00000296504	ENSP00000351250
ENSP00000296504	ENSP00000353622
ENSP00000296504	ENSP00000362592
ENSP00000296504	ENSP00000364929
ENSP00000296504	ENSP00000395535
ENSP00000296509	ENSP00000296577
ENSP00000296509	ENSP00000297338
ENSP00000296509	ENSP00000297814
ENSP00000296509	ENSP00000298139
ENSP00000296509	ENSP00000300181
ENSP00000296509	ENSP00000300249
ENSP00000296509	ENSP00000300584
ENSP00000296509	ENSP00000301332
ENSP00000296509	ENSP00000304102
ENSP00000296509	ENSP00000304593
ENSP00000296509	ENSP00000304668
ENSP00000296509	ENSP00000306123
ENSP00000296509	ENSP00000306999
ENSP00000296509	ENSP00000307853
ENSP00000296509	ENSP00000308699
ENSP00000296509	ENSP00000308944
ENSP00000296509	ENSP00000309142
ENSP00000296509	ENSP00000309968
ENSP00000296509	ENSP00000310520
ENSP00000296509	ENSP00000311505

ENSP00000296509	ENSP00000312870
ENSP00000296509	ENSP00000313171
ENSP00000296509	ENSP00000313420
ENSP00000296509	ENSP00000314004
ENSP00000296509	ENSP00000316032
ENSP00000296509	ENSP00000317334
ENSP00000296509	ENSP00000317636
ENSP00000296509	ENSP00000318775
ENSP00000296509	ENSP00000320885
ENSP00000296509	ENSP00000321711
ENSP00000296509	ENSP00000322180
ENSP00000296509	ENSP00000323439
ENSP00000296509	ENSP00000324020
ENSP00000296509	ENSP00000325863
ENSP00000296509	ENSP00000327009
ENSP00000296509	ENSP00000327197
ENSP00000296509	ENSP00000330343
ENSP00000296509	ENSP00000333667
ENSP00000296509	ENSP00000333836
ENSP00000296509	ENSP00000334188
ENSP00000296509	ENSP00000334280
ENSP00000296509	ENSP00000334675
ENSP00000296509	ENSP00000335357
ENSP00000296509	ENSP00000335493
ENSP00000296509	ENSP00000336606
ENSP00000296509	ENSP00000336702
ENSP00000296509	ENSP00000336868
ENSP00000296509	ENSP00000337405
ENSP00000296509	ENSP00000339109
ENSP00000296509	ENSP00000340019
ENSP00000296509	ENSP00000340505
ENSP00000296509	ENSP00000340879
ENSP00000296509	ENSP00000341382
ENSP00000296509	ENSP00000341848
ENSP00000296509	ENSP00000342307
ENSP00000296509	ENSP00000343001
ENSP00000296509	ENSP00000343392
ENSP00000296509	ENSP00000343741
ENSP00000296509	ENSP00000346566
ENSP00000296509	ENSP00000347232
ENSP00000296509	ENSP00000347719
ENSP00000296509	ENSP00000347733
ENSP00000296509	ENSP00000348089
ENSP00000296509	ENSP00000348554
ENSP00000296509	ENSP00000348573
ENSP00000296509	ENSP00000348965

ENSP00000296509	ENSP00000349213
ENSP00000296509	ENSP00000350512
ENSP00000296509	ENSP00000351665
ENSP00000296509	ENSP00000351697
ENSP00000296509	ENSP00000352257
ENSP00000296509	ENSP00000352572
ENSP00000296509	ENSP00000353332
ENSP00000296509	ENSP00000353793
ENSP00000296509	ENSP00000354486
ENSP00000296509	ENSP00000354720
ENSP00000296509	ENSP00000354851
ENSP00000296509	ENSP00000354916
ENSP00000296509	ENSP00000355205
ENSP00000296509	ENSP00000355388
ENSP00000296509	ENSP00000355511
ENSP00000296509	ENSP00000355560
ENSP00000296509	ENSP00000355922
ENSP00000296509	ENSP00000355944
ENSP00000296509	ENSP00000356130
ENSP00000296509	ENSP00000356319
ENSP00000296509	ENSP00000356548
ENSP00000296509	ENSP00000356579
ENSP00000296509	ENSP00000356920
ENSP00000296509	ENSP00000356989
ENSP00000296509	ENSP00000357643
ENSP00000296509	ENSP00000357651
ENSP00000296509	ENSP00000357887
ENSP00000296509	ENSP00000358478
ENSP00000296509	ENSP00000358605
ENSP00000296509	ENSP00000358953
ENSP00000296509	ENSP00000360310
ENSP00000296509	ENSP00000360473
ENSP00000296509	ENSP00000360621
ENSP00000296509	ENSP00000361310
ENSP00000296509	ENSP00000361949
ENSP00000296509	ENSP00000362352
ENSP00000296509	ENSP00000363055
ENSP00000296509	ENSP00000363559
ENSP00000296509	ENSP00000363734
ENSP00000296509	ENSP00000365435
ENSP00000296509	ENSP00000366249
ENSP00000296509	ENSP00000366974
ENSP00000296509	ENSP00000367034
ENSP00000296509	ENSP00000368349
ENSP00000296509	ENSP00000368976
ENSP00000296509	ENSP00000369411

ENSP00000296509	ENSP00000369965
ENSP00000296509	ENSP00000370150
ENSP00000296509	ENSP00000370151
ENSP00000296509	ENSP00000370741
ENSP00000296509	ENSP00000371475
ENSP00000296509	ENSP00000372023
ENSP00000296509	ENSP00000372694
ENSP00000296509	ENSP00000374213
ENSP00000296509	ENSP00000374566
ENSP00000296509	ENSP00000375809
ENSP00000296509	ENSP00000377655
ENSP00000296509	ENSP00000377793
ENSP00000296509	ENSP00000378180
ENSP00000296509	ENSP00000378350
ENSP00000296509	ENSP00000382562
ENSP00000296509	ENSP00000383145
ENSP00000296509	ENSP00000384053
ENSP00000296509	ENSP00000394071
ENSP00000296509	ENSP00000394791
ENSP00000296509	ENSP00000396152
ENSP00000296509	ENSP00000402084
ENSP00000296509	ENSP00000402515
ENSP00000296509	ENSP00000402527
ENSP00000296509	ENSP00000410818
ENSP00000296509	ENSP00000412319
ENSP00000296509	ENSP00000414402
ENSP00000296509	ENSP00000415678
ENSP00000296543	ENSP00000297338
ENSP00000296543	ENSP00000298852
ENSP00000296543	ENSP00000302896
ENSP00000296543	ENSP00000309474
ENSP00000296543	ENSP00000311430
ENSP00000296543	ENSP00000314348
ENSP00000296543	ENSP00000323858
ENSP00000296543	ENSP00000323967
ENSP00000296543	ENSP00000327070
ENSP00000296543	ENSP00000329466
ENSP00000296543	ENSP00000331817
ENSP00000296543	ENSP00000335321
ENSP00000296543	ENSP00000339795
ENSP00000296543	ENSP00000348442
ENSP00000296543	ENSP00000348565
ENSP00000296543	ENSP00000352257
ENSP00000296543	ENSP00000352738
ENSP00000296543	ENSP00000358563
ENSP00000296543	ENSP00000358812

ENSP00000296543	ENSP00000360621
ENSP00000296543	ENSP00000362110
ENSP00000296543	ENSP00000363308
ENSP00000296543	ENSP00000370589
ENSP00000296543	ENSP00000370718
ENSP00000296543	ENSP00000381031
ENSP00000296543	ENSP00000384474
ENSP00000296543	ENSP00000385903
ENSP00000296543	ENSP00000392617
ENSP00000296543	ENSP00000399511
ENSP00000296543	ENSP00000408058
ENSP00000296543	ENSP00000417763
ENSP00000296545	ENSP00000369312
ENSP00000296545	ENSP00000416599
ENSP00000296550	ENSP00000297338
ENSP00000296550	ENSP00000309457
ENSP00000296550	ENSP00000340879
ENSP00000296550	ENSP00000342755
ENSP00000296550	ENSP00000355167
ENSP00000296550	ENSP00000357283
ENSP00000296550	ENSP00000382323
ENSP00000296550	ENSP00000402240
ENSP00000296557	ENSP00000360532
ENSP00000296557	ENSP00000379607
ENSP00000296564	ENSP00000359206
ENSP00000296564	ENSP00000359321
ENSP00000296564	ENSP00000380054
ENSP00000296577	ENSP00000296802
ENSP00000296577	ENSP00000298283
ENSP00000296577	ENSP00000299608
ENSP00000296577	ENSP00000300151
ENSP00000296577	ENSP00000303276
ENSP00000296577	ENSP00000304668
ENSP00000296577	ENSP00000306548
ENSP00000296577	ENSP00000307889
ENSP00000296577	ENSP00000308897
ENSP00000296577	ENSP00000309334
ENSP00000296577	ENSP00000310040
ENSP00000296577	ENSP00000311430
ENSP00000296577	ENSP00000317327
ENSP00000296577	ENSP00000322419
ENSP00000296577	ENSP00000323246
ENSP00000296577	ENSP00000323424
ENSP00000296577	ENSP00000323858
ENSP00000296577	ENSP00000325448
ENSP00000296577	ENSP00000329466

ENSP00000296577	ENSP00000333551
ENSP00000296577	ENSP00000339090
ENSP00000296577	ENSP00000339095
ENSP00000296577	ENSP00000339795
ENSP00000296577	ENSP00000346022
ENSP00000296577	ENSP00000346037
ENSP00000296577	ENSP00000346050
ENSP00000296577	ENSP00000346120
ENSP00000296577	ENSP00000348107
ENSP00000296577	ENSP00000352738
ENSP00000296577	ENSP00000353741
ENSP00000296577	ENSP00000354125
ENSP00000296577	ENSP00000354739
ENSP00000296577	ENSP00000355086
ENSP00000296577	ENSP00000356476
ENSP00000296577	ENSP00000356530
ENSP00000296577	ENSP00000356811
ENSP00000296577	ENSP00000358140
ENSP00000296577	ENSP00000361010
ENSP00000296577	ENSP00000361076
ENSP00000296577	ENSP00000362688
ENSP00000296577	ENSP00000362744
ENSP00000296577	ENSP00000363779
ENSP00000296577	ENSP00000364119
ENSP00000296577	ENSP00000364320
ENSP00000296577	ENSP00000365837
ENSP00000296577	ENSP00000369042
ENSP00000296577	ENSP00000369757
ENSP00000296577	ENSP00000370258
ENSP00000296577	ENSP00000372326
ENSP00000296577	ENSP00000373404
ENSP00000296577	ENSP00000374390
ENSP00000296577	ENSP00000375730
ENSP00000296577	ENSP00000377865
ENSP00000296577	ENSP00000379888
ENSP00000296577	ENSP00000395777
ENSP00000296577	ENSP00000414321
ENSP00000296577	ENSP00000417229
ENSP00000296582	ENSP00000297290
ENSP00000296582	ENSP00000343785
ENSP00000296658	ENSP00000304903
ENSP00000296658	ENSP00000322016
ENSP00000296658	ENSP00000379607
ENSP00000296674	ENSP00000296802
ENSP00000296674	ENSP00000296805
ENSP00000296674	ENSP00000297908

ENSP00000296674	ENSP00000298283
ENSP00000296674	ENSP00000300151
ENSP00000296674	ENSP00000302896
ENSP00000296674	ENSP00000308897
ENSP00000296674	ENSP00000311028
ENSP00000296674	ENSP00000312735
ENSP00000296674	ENSP00000314949
ENSP00000296674	ENSP00000317376
ENSP00000296674	ENSP00000318646
ENSP00000296674	ENSP00000323856
ENSP00000296674	ENSP00000326981
ENSP00000296674	ENSP00000329466
ENSP00000296674	ENSP00000331019
ENSP00000296674	ENSP00000336702
ENSP00000296674	ENSP00000337127
ENSP00000296674	ENSP00000339095
ENSP00000296674	ENSP00000341885
ENSP00000296674	ENSP00000345156
ENSP00000296674	ENSP00000346015
ENSP00000296674	ENSP00000346050
ENSP00000296674	ENSP00000346067
ENSP00000296674	ENSP00000354739
ENSP00000296674	ENSP00000355471
ENSP00000296674	ENSP00000356134
ENSP00000296674	ENSP00000356811
ENSP00000296674	ENSP00000358140
ENSP00000296674	ENSP00000359345
ENSP00000296674	ENSP00000361076
ENSP00000296674	ENSP00000361465
ENSP00000296674	ENSP00000362036
ENSP00000296674	ENSP00000362688
ENSP00000296674	ENSP00000362744
ENSP00000296674	ENSP00000363018
ENSP00000296674	ENSP00000363308
ENSP00000296674	ENSP00000364320
ENSP00000296674	ENSP00000368790
ENSP00000296674	ENSP00000368927
ENSP00000296674	ENSP00000369757
ENSP00000296674	ENSP00000372445
ENSP00000296674	ENSP00000377640
ENSP00000296674	ENSP00000379339
ENSP00000296674	ENSP00000379607
ENSP00000296674	ENSP00000379888
ENSP00000296674	ENSP00000380156
ENSP00000296674	ENSP00000388330
ENSP00000296674	ENSP00000389103

ENSP00000296674	ENSP00000393241
ENSP00000296674	ENSP00000403175
ENSP00000296674	ENSP00000408058
ENSP00000296674	ENSP00000412583
ENSP00000296674	ENSP00000414321
ENSP00000296674	ENSP00000416110
ENSP00000296674	ENSP00000420714
ENSP00000296684	ENSP00000308610
ENSP00000296684	ENSP00000315774
ENSP00000296684	ENSP00000356972
ENSP00000296702	ENSP00000296930
ENSP00000296702	ENSP00000299424
ENSP00000296702	ENSP00000300291
ENSP00000296702	ENSP00000300413
ENSP00000296702	ENSP00000301280
ENSP00000296702	ENSP00000303191
ENSP00000296702	ENSP00000304350
ENSP00000296702	ENSP00000304370
ENSP00000296702	ENSP00000305790
ENSP00000296702	ENSP00000307863
ENSP00000296702	ENSP00000308369
ENSP00000296702	ENSP00000314949
ENSP00000296702	ENSP00000316786
ENSP00000296702	ENSP00000318861
ENSP00000296702	ENSP00000318884
ENSP00000296702	ENSP00000319169
ENSP00000296702	ENSP00000319341
ENSP00000296702	ENSP00000320924
ENSP00000296702	ENSP00000322016
ENSP00000296702	ENSP00000325376
ENSP00000296702	ENSP00000329219
ENSP00000296702	ENSP00000332103
ENSP00000296702	ENSP00000334148
ENSP00000296702	ENSP00000335321
ENSP00000296702	ENSP00000337405
ENSP00000296702	ENSP00000339090
ENSP00000296702	ENSP00000342374
ENSP00000296702	ENSP00000346236
ENSP00000296702	ENSP00000347184
ENSP00000296702	ENSP00000349748
ENSP00000296702	ENSP00000351137
ENSP00000296702	ENSP00000357965
ENSP00000296702	ENSP00000358554
ENSP00000296702	ENSP00000360532
ENSP00000296702	ENSP00000361577
ENSP00000296702	ENSP00000362110

ENSP00000296702	ENSP00000364289
ENSP00000296702	ENSP00000365439
ENSP00000296702	ENSP00000365891
ENSP00000296702	ENSP00000366557
ENSP00000296702	ENSP00000366607
ENSP00000296702	ENSP00000368801
ENSP00000296702	ENSP00000369218
ENSP00000296702	ENSP00000369716
ENSP00000296702	ENSP00000370083
ENSP00000296702	ENSP00000373952
ENSP00000296702	ENSP00000381565
ENSP00000296702	ENSP00000383059
ENSP00000296702	ENSP00000386161
ENSP00000296702	ENSP00000390948
ENSP00000296702	ENSP00000391774
ENSP00000296702	ENSP00000412566
ENSP00000296733	ENSP00000301364
ENSP00000296733	ENSP00000310649
ENSP00000296733	ENSP00000343741
ENSP00000296733	ENSP00000370258
ENSP00000296733	ENSP00000382562
ENSP00000296733	ENSP00000402084
ENSP00000296755	ENSP00000300231
ENSP00000296755	ENSP00000304051
ENSP00000296755	ENSP00000306245
ENSP00000296755	ENSP00000307863
ENSP00000296755	ENSP00000308944
ENSP00000296755	ENSP00000309474
ENSP00000296755	ENSP00000311505
ENSP00000296755	ENSP00000312778
ENSP00000296755	ENSP00000318313
ENSP00000296755	ENSP00000322396
ENSP00000296755	ENSP00000324792
ENSP00000296755	ENSP00000329466
ENSP00000296755	ENSP00000330343
ENSP00000296755	ENSP00000334675
ENSP00000296755	ENSP00000336868
ENSP00000296755	ENSP00000338345
ENSP00000296755	ENSP00000343392
ENSP00000296755	ENSP00000343479
ENSP00000296755	ENSP00000347168
ENSP00000296755	ENSP00000348089
ENSP00000296755	ENSP00000350767
ENSP00000296755	ENSP00000351284
ENSP00000296755	ENSP00000351813
ENSP00000296755	ENSP00000352257

ENSP00000296755	ENSP00000352738
ENSP00000296755	ENSP00000357651
ENSP00000296755	ENSP00000360286
ENSP00000296755	ENSP00000360621
ENSP00000296755	ENSP00000360922
ENSP00000296755	ENSP00000364204
ENSP00000296755	ENSP00000366395
ENSP00000296755	ENSP00000366974
ENSP00000296755	ENSP00000367034
ENSP00000296755	ENSP00000368349
ENSP00000296755	ENSP00000372023
ENSP00000296755	ENSP00000378517
ENSP00000296755	ENSP00000379607
ENSP00000296755	ENSP00000390948
ENSP00000296755	ENSP00000392270
ENSP00000296783	ENSP00000298310
ENSP00000296783	ENSP00000309474
ENSP00000296783	ENSP00000313350
ENSP00000296783	ENSP00000314311
ENSP00000296783	ENSP00000314441
ENSP00000296783	ENSP00000316879
ENSP00000296783	ENSP00000322457
ENSP00000296783	ENSP00000322570
ENSP00000296783	ENSP00000339845
ENSP00000296783	ENSP00000341885
ENSP00000296783	ENSP00000343741
ENSP00000296783	ENSP00000343745
ENSP00000296783	ENSP00000351697
ENSP00000296783	ENSP00000354040
ENSP00000296783	ENSP00000354522
ENSP00000296783	ENSP00000356146
ENSP00000296783	ENSP00000357484
ENSP00000296783	ENSP00000358812
ENSP00000296783	ENSP00000366135
ENSP00000296783	ENSP00000366519
ENSP00000296783	ENSP00000368349
ENSP00000296783	ENSP00000369887
ENSP00000296783	ENSP00000370151
ENSP00000296783	ENSP00000370408
ENSP00000296783	ENSP00000370741
ENSP00000296783	ENSP00000372023
ENSP00000296783	ENSP00000373404
ENSP00000296783	ENSP00000380982
ENSP00000296785	ENSP00000348010
ENSP00000296785	ENSP00000361548
ENSP00000296786	ENSP00000296792

ENSP00000296786	ENSP00000299259
ENSP00000296786	ENSP00000300181
ENSP00000296786	ENSP00000300648
ENSP00000296786	ENSP00000302790
ENSP00000296786	ENSP00000304593
ENSP00000296786	ENSP00000309198
ENSP00000296786	ENSP00000310129
ENSP00000296786	ENSP00000322242
ENSP00000296786	ENSP00000325677
ENSP00000296786	ENSP00000334061
ENSP00000296786	ENSP00000347836
ENSP00000296786	ENSP00000348442
ENSP00000296786	ENSP00000350967
ENSP00000296786	ENSP00000351596
ENSP00000296786	ENSP00000353622
ENSP00000296786	ENSP00000354673
ENSP00000296786	ENSP00000356405
ENSP00000296786	ENSP00000356641
ENSP00000296786	ENSP00000360116
ENSP00000296786	ENSP00000362334
ENSP00000296786	ENSP00000368959
ENSP00000296786	ENSP00000374213
ENSP00000296786	ENSP00000374280
ENSP00000296786	ENSP00000380460
ENSP00000296786	ENSP00000382779
ENSP00000296786	ENSP00000384211
ENSP00000296792	ENSP00000297579
ENSP00000296792	ENSP00000297990
ENSP00000296792	ENSP00000298937
ENSP00000296792	ENSP00000301364
ENSP00000296792	ENSP00000304593
ENSP00000296792	ENSP00000308179
ENSP00000296792	ENSP00000311135
ENSP00000296792	ENSP00000314193
ENSP00000296792	ENSP00000315674
ENSP00000296792	ENSP00000318604
ENSP00000296792	ENSP00000326981
ENSP00000296792	ENSP00000327179
ENSP00000296792	ENSP00000328690
ENSP00000296792	ENSP00000328854
ENSP00000296792	ENSP00000329466
ENSP00000296792	ENSP00000339095
ENSP00000296792	ENSP00000339145
ENSP00000296792	ENSP00000346725
ENSP00000296792	ENSP00000350967
ENSP00000296792	ENSP00000353246

ENSP00000296792	ENSP00000354040
ENSP00000296792	ENSP00000355471
ENSP00000296792	ENSP00000355541
ENSP00000296792	ENSP00000357907
ENSP00000296792	ENSP00000360031
ENSP00000296792	ENSP00000360473
ENSP00000296792	ENSP00000362744
ENSP00000296792	ENSP00000363642
ENSP00000296792	ENSP00000363746
ENSP00000296792	ENSP00000363779
ENSP00000296792	ENSP00000366557
ENSP00000296792	ENSP00000370258
ENSP00000296792	ENSP00000370589
ENSP00000296792	ENSP00000370968
ENSP00000296792	ENSP00000371101
ENSP00000296792	ENSP00000372695
ENSP00000296792	ENSP00000379888
ENSP00000296792	ENSP00000380460
ENSP00000296792	ENSP00000384302
ENSP00000296792	ENSP00000386759
ENSP00000296792	ENSP00000387006
ENSP00000296792	ENSP00000396937
ENSP00000296792	ENSP00000398597
ENSP00000296792	ENSP00000399454
ENSP00000296792	ENSP00000402869
ENSP00000296792	ENSP00000405614
ENSP00000296802	ENSP00000297990
ENSP00000296802	ENSP00000298283
ENSP00000296802	ENSP00000298746
ENSP00000296802	ENSP00000300151
ENSP00000296802	ENSP00000301364
ENSP00000296802	ENSP00000302886
ENSP00000296802	ENSP00000302896
ENSP00000296802	ENSP00000305682
ENSP00000296802	ENSP00000305702
ENSP00000296802	ENSP00000306548
ENSP00000296802	ENSP00000307889
ENSP00000296802	ENSP00000308897
ENSP00000296802	ENSP00000309334
ENSP00000296802	ENSP00000309830
ENSP00000296802	ENSP00000311028
ENSP00000296802	ENSP00000311430
ENSP00000296802	ENSP00000314311
ENSP00000296802	ENSP00000314348
ENSP00000296802	ENSP00000315674
ENSP00000296802	ENSP00000317376

ENSP00000296802	ENSP00000317992
ENSP00000296802	ENSP00000318646
ENSP00000296802	ENSP00000320917
ENSP00000296802	ENSP00000323856
ENSP00000296802	ENSP00000323858
ENSP00000296802	ENSP00000325312
ENSP00000296802	ENSP00000326981
ENSP00000296802	ENSP00000328690
ENSP00000296802	ENSP00000328854
ENSP00000296802	ENSP00000329466
ENSP00000296802	ENSP00000331019
ENSP00000296802	ENSP00000332340
ENSP00000296802	ENSP00000334188
ENSP00000296802	ENSP00000338573
ENSP00000296802	ENSP00000338862
ENSP00000296802	ENSP00000339095
ENSP00000296802	ENSP00000339145
ENSP00000296802	ENSP00000339795
ENSP00000296802	ENSP00000341885
ENSP00000296802	ENSP00000343081
ENSP00000296802	ENSP00000345096
ENSP00000296802	ENSP00000345156
ENSP00000296802	ENSP00000346015
ENSP00000296802	ENSP00000346022
ENSP00000296802	ENSP00000346050
ENSP00000296802	ENSP00000346067
ENSP00000296802	ENSP00000346120
ENSP00000296802	ENSP00000346236
ENSP00000296802	ENSP00000346453
ENSP00000296802	ENSP00000346725
ENSP00000296802	ENSP00000348596
ENSP00000296802	ENSP00000348722
ENSP00000296802	ENSP00000352021
ENSP00000296802	ENSP00000354040
ENSP00000296802	ENSP00000354125
ENSP00000296802	ENSP00000354739
ENSP00000296802	ENSP00000358140
ENSP00000296802	ENSP00000358563
ENSP00000296802	ENSP00000358812
ENSP00000296802	ENSP00000359345
ENSP00000296802	ENSP00000360031
ENSP00000296802	ENSP00000360828
ENSP00000296802	ENSP00000361076
ENSP00000296802	ENSP00000361092
ENSP00000296802	ENSP00000361232
ENSP00000296802	ENSP00000361982

ENSP00000296802	ENSP00000362153
ENSP00000296802	ENSP00000362744
ENSP00000296802	ENSP00000363018
ENSP00000296802	ENSP00000363384
ENSP00000296802	ENSP00000363559
ENSP00000296802	ENSP00000363676
ENSP00000296802	ENSP00000364293
ENSP00000296802	ENSP00000364320
ENSP00000296802	ENSP00000365806
ENSP00000296802	ENSP00000366519
ENSP00000296802	ENSP00000366557
ENSP00000296802	ENSP00000369757
ENSP00000296802	ENSP00000369965
ENSP00000296802	ENSP00000370258
ENSP00000296802	ENSP00000371101
ENSP00000296802	ENSP00000371169
ENSP00000296802	ENSP00000373090
ENSP00000296802	ENSP00000373404
ENSP00000296802	ENSP00000375730
ENSP00000296802	ENSP00000377640
ENSP00000296802	ENSP00000379339
ENSP00000296802	ENSP00000382392
ENSP00000296802	ENSP00000389103
ENSP00000296802	ENSP00000393241
ENSP00000296802	ENSP00000394290
ENSP00000296802	ENSP00000402338
ENSP00000296802	ENSP00000403175
ENSP00000296802	ENSP00000403576
ENSP00000296802	ENSP00000409074
ENSP00000296802	ENSP00000409367
ENSP00000296802	ENSP00000411162
ENSP00000296802	ENSP00000412583
ENSP00000296802	ENSP00000414321
ENSP00000296802	ENSP00000416110
ENSP00000296802	ENSP00000417229
ENSP00000296802	ENSP00000420357
ENSP00000296802	ENSP00000420714
ENSP00000296805	ENSP00000297908
ENSP00000296805	ENSP00000298283
ENSP00000296805	ENSP00000300151
ENSP00000296805	ENSP00000302160
ENSP00000296805	ENSP00000302896
ENSP00000296805	ENSP00000306548
ENSP00000296805	ENSP00000307853
ENSP00000296805	ENSP00000308897
ENSP00000296805	ENSP00000310448

ENSP00000296805	ENSP00000310520
ENSP00000296805	ENSP00000311028
ENSP00000296805	ENSP00000311430
ENSP00000296805	ENSP00000312735
ENSP00000296805	ENSP00000315379
ENSP00000296805	ENSP00000317376
ENSP00000296805	ENSP00000318646
ENSP00000296805	ENSP00000318861
ENSP00000296805	ENSP00000321320
ENSP00000296805	ENSP00000321810
ENSP00000296805	ENSP00000325448
ENSP00000296805	ENSP00000326981
ENSP00000296805	ENSP00000333837
ENSP00000296805	ENSP00000335321
ENSP00000296805	ENSP00000336702
ENSP00000296805	ENSP00000339795
ENSP00000296805	ENSP00000341082
ENSP00000296805	ENSP00000341885
ENSP00000296805	ENSP00000343557
ENSP00000296805	ENSP00000346015
ENSP00000296805	ENSP00000346022
ENSP00000296805	ENSP00000346067
ENSP00000296805	ENSP00000351137
ENSP00000296805	ENSP00000354125
ENSP00000296805	ENSP00000354525
ENSP00000296805	ENSP00000354739
ENSP00000296805	ENSP00000356811
ENSP00000296805	ENSP00000357631
ENSP00000296805	ENSP00000358554
ENSP00000296805	ENSP00000359345
ENSP00000296805	ENSP00000359454
ENSP00000296805	ENSP00000362036
ENSP00000296805	ENSP00000362110
ENSP00000296805	ENSP00000363018
ENSP00000296805	ENSP00000363313
ENSP00000296805	ENSP00000363559
ENSP00000296805	ENSP00000363676
ENSP00000296805	ENSP00000364289
ENSP00000296805	ENSP00000364320
ENSP00000296805	ENSP00000366557
ENSP00000296805	ENSP00000373404
ENSP00000296805	ENSP00000375730
ENSP00000296805	ENSP00000376973
ENSP00000296805	ENSP00000377486
ENSP00000296805	ENSP00000377865
ENSP00000296805	ENSP00000379339

ENSP00000296805	ENSP00000379888
ENSP00000296805	ENSP00000382779
ENSP00000296805	ENSP00000386992
ENSP00000296805	ENSP00000387006
ENSP00000296805	ENSP00000388330
ENSP00000296805	ENSP00000389103
ENSP00000296805	ENSP00000393241
ENSP00000296805	ENSP00000393953
ENSP00000296805	ENSP00000401566
ENSP00000296805	ENSP00000403175
ENSP00000296805	ENSP00000412583
ENSP00000296805	ENSP00000416110
ENSP00000296805	ENSP00000417229
ENSP00000296805	ENSP00000419599
ENSP00000296805	ENSP00000420714
ENSP00000296859	ENSP00000345530
ENSP00000296859	ENSP00000394794
ENSP00000296861	ENSP00000341268
ENSP00000296861	ENSP00000354346
ENSP00000296870	ENSP00000303507
ENSP00000296870	ENSP00000384053
ENSP00000296871	ENSP00000307046
ENSP00000296871	ENSP00000370935
ENSP00000296871	ENSP00000384053
ENSP00000296875	ENSP00000297261
ENSP00000296875	ENSP00000331719
ENSP00000296875	ENSP00000347184
ENSP00000296875	ENSP00000361949
ENSP00000296875	ENSP00000363708
ENSP00000296875	ENSP00000404121
ENSP00000296930	ENSP00000299767
ENSP00000296930	ENSP00000300151
ENSP00000296930	ENSP00000301019
ENSP00000296930	ENSP00000301280
ENSP00000296930	ENSP00000302886
ENSP00000296930	ENSP00000305689
ENSP00000296930	ENSP00000308750
ENSP00000296930	ENSP00000309565
ENSP00000296930	ENSP00000310590
ENSP00000296930	ENSP00000315791
ENSP00000296930	ENSP00000316032
ENSP00000296930	ENSP00000318195
ENSP00000296930	ENSP00000319831
ENSP00000296930	ENSP00000325376
ENSP00000296930	ENSP00000329466
ENSP00000296930	ENSP00000329662

ENSP00000296930	ENSP00000331019
ENSP00000296930	ENSP00000332721
ENSP00000296930	ENSP00000334538
ENSP00000296930	ENSP00000336868
ENSP00000296930	ENSP00000337340
ENSP00000296930	ENSP00000339145
ENSP00000296930	ENSP00000339353
ENSP00000296930	ENSP00000341947
ENSP00000296930	ENSP00000344683
ENSP00000296930	ENSP00000345436
ENSP00000296930	ENSP00000345917
ENSP00000296930	ENSP00000346120
ENSP00000296930	ENSP00000350283
ENSP00000296930	ENSP00000351894
ENSP00000296930	ENSP00000352565
ENSP00000296930	ENSP00000354522
ENSP00000296930	ENSP00000354916
ENSP00000296930	ENSP00000355050
ENSP00000296930	ENSP00000355153
ENSP00000296930	ENSP00000355759
ENSP00000296930	ENSP00000355922
ENSP00000296930	ENSP00000360025
ENSP00000296930	ENSP00000360532
ENSP00000296930	ENSP00000364398
ENSP00000296930	ENSP00000365057
ENSP00000296930	ENSP00000367454
ENSP00000296930	ENSP00000367766
ENSP00000296930	ENSP00000368438
ENSP00000296930	ENSP00000370083
ENSP00000296930	ENSP00000370376
ENSP00000296930	ENSP00000370589
ENSP00000296930	ENSP00000370968
ENSP00000296930	ENSP00000373700
ENSP00000296930	ENSP00000373952
ENSP00000296930	ENSP00000377527
ENSP00000296930	ENSP00000379607
ENSP00000296930	ENSP00000381657
ENSP00000296930	ENSP00000382392
ENSP00000296930	ENSP00000382999
ENSP00000296930	ENSP00000384026
ENSP00000296930	ENSP00000390746
ENSP00000296930	ENSP00000401272
ENSP00000296930	ENSP00000406895
ENSP00000296930	ENSP00000412884
ENSP00000296930	ENSP00000414109
ENSP00000296930	ENSP00000417281

ENSP00000296953	ENSP00000304903
ENSP00000296953	ENSP00000342136
ENSP00000296955	ENSP00000334061
ENSP00000297135	ENSP00000305442
ENSP00000297135	ENSP00000326514
ENSP00000297151	ENSP00000298532
ENSP00000297151	ENSP00000300413
ENSP00000297151	ENSP00000301740
ENSP00000297151	ENSP00000304350
ENSP00000297151	ENSP00000304370
ENSP00000297151	ENSP00000307004
ENSP00000297151	ENSP00000307863
ENSP00000297151	ENSP00000314311
ENSP00000297151	ENSP00000315379
ENSP00000297151	ENSP00000318861
ENSP00000297151	ENSP00000324274
ENSP00000297151	ENSP00000326806
ENSP00000297151	ENSP00000331907
ENSP00000297151	ENSP00000334148
ENSP00000297151	ENSP00000339090
ENSP00000297151	ENSP00000343557
ENSP00000297151	ENSP00000359727
ENSP00000297151	ENSP00000360532
ENSP00000297151	ENSP00000366557
ENSP00000297151	ENSP00000367835
ENSP00000297151	ENSP00000376973
ENSP00000297151	ENSP00000386759
ENSP00000297151	ENSP00000416959
ENSP00000297151	ENSP00000420195
ENSP00000297157	ENSP00000339723
ENSP00000297185	ENSP00000298097
ENSP00000297185	ENSP00000299767
ENSP00000297185	ENSP00000301012
ENSP00000297185	ENSP00000301585
ENSP00000297185	ENSP00000302896
ENSP00000297185	ENSP00000304704
ENSP00000297185	ENSP00000309365
ENSP00000297185	ENSP00000309474
ENSP00000297185	ENSP00000310623
ENSP00000297185	ENSP00000311505
ENSP00000297185	ENSP00000318115
ENSP00000297185	ENSP00000325562
ENSP00000297185	ENSP00000327070
ENSP00000297185	ENSP00000329002
ENSP00000297185	ENSP00000329466
ENSP00000297185	ENSP00000329558

ENSP00000297185	ENSP00000329967
ENSP00000297185	ENSP00000330836
ENSP00000297185	ENSP00000331817
ENSP00000297185	ENSP00000337340
ENSP00000297185	ENSP00000338788
ENSP00000297185	ENSP00000338862
ENSP00000297185	ENSP00000339933
ENSP00000297185	ENSP00000340019
ENSP00000297185	ENSP00000341243
ENSP00000297185	ENSP00000341743
ENSP00000297185	ENSP00000344460
ENSP00000297185	ENSP00000345282
ENSP00000297185	ENSP00000347314
ENSP00000297185	ENSP00000348877
ENSP00000297185	ENSP00000350928
ENSP00000297185	ENSP00000354927
ENSP00000297185	ENSP00000355153
ENSP00000297185	ENSP00000355865
ENSP00000297185	ENSP00000357748
ENSP00000297185	ENSP00000357998
ENSP00000297185	ENSP00000358022
ENSP00000297185	ENSP00000358140
ENSP00000297185	ENSP00000358497
ENSP00000297185	ENSP00000360286
ENSP00000297185	ENSP00000360803
ENSP00000297185	ENSP00000361465
ENSP00000297185	ENSP00000361686
ENSP00000297185	ENSP00000362298
ENSP00000297185	ENSP00000362424
ENSP00000297185	ENSP00000363559
ENSP00000297185	ENSP00000365007
ENSP00000297185	ENSP00000365388
ENSP00000297185	ENSP00000365766
ENSP00000297185	ENSP00000366482
ENSP00000297185	ENSP00000367208
ENSP00000297185	ENSP00000370377
ENSP00000297185	ENSP00000379607
ENSP00000297185	ENSP00000380504
ENSP00000297185	ENSP00000381891
ENSP00000297185	ENSP00000382423
ENSP00000297185	ENSP00000382595
ENSP00000297185	ENSP00000386621
ENSP00000297185	ENSP00000386717
ENSP00000297185	ENSP00000387219
ENSP00000297185	ENSP00000387262
ENSP00000297185	ENSP00000388872

ENSP00000297185	ENSP00000403817
ENSP00000297185	ENSP00000417132
ENSP00000297195	ENSP00000391798
ENSP00000297261	ENSP00000298743
ENSP00000297261	ENSP00000301831
ENSP00000297261	ENSP00000302216
ENSP00000297261	ENSP00000314311
ENSP00000297261	ENSP00000316842
ENSP00000297261	ENSP00000319501
ENSP00000297261	ENSP00000332353
ENSP00000297261	ENSP00000349208
ENSP00000297261	ENSP00000353622
ENSP00000297261	ENSP00000353904
ENSP00000297261	ENSP00000354586
ENSP00000297261	ENSP00000354995
ENSP00000297261	ENSP00000355896
ENSP00000297261	ENSP00000356795
ENSP00000297261	ENSP00000358918
ENSP00000297261	ENSP00000359854
ENSP00000297261	ENSP00000363489
ENSP00000297261	ENSP00000364524
ENSP00000297261	ENSP00000364839
ENSP00000297261	ENSP00000366395
ENSP00000297261	ENSP00000377401
ENSP00000297261	ENSP00000397435
ENSP00000297261	ENSP00000409950
ENSP00000297265	ENSP00000302239
ENSP00000297265	ENSP00000307387
ENSP00000297265	ENSP00000310440
ENSP00000297265	ENSP00000311200
ENSP00000297265	ENSP00000313601
ENSP00000297265	ENSP00000317468
ENSP00000297265	ENSP00000330408
ENSP00000297265	ENSP00000336702
ENSP00000297265	ENSP00000345216
ENSP00000297265	ENSP00000354519
ENSP00000297265	ENSP00000356602
ENSP00000297265	ENSP00000365943
ENSP00000297265	ENSP00000378368
ENSP00000297265	ENSP00000380349
ENSP00000297265	ENSP00000381036
ENSP00000297265	ENSP00000391249
ENSP00000297273	ENSP00000298229
ENSP00000297273	ENSP00000300574
ENSP00000297273	ENSP00000324422
ENSP00000297273	ENSP00000371341

ENSP00000297290	ENSP00000343785
ENSP00000297290	ENSP00000354995
ENSP00000297290	ENSP00000359634
ENSP00000297313	ENSP00000300574
ENSP00000297313	ENSP00000309433
ENSP00000297313	ENSP00000330836
ENSP00000297313	ENSP00000357452
ENSP00000297313	ENSP00000358146
ENSP00000297313	ENSP00000363807
ENSP00000297313	ENSP00000381293
ENSP00000297313	ENSP00000391735
ENSP00000297316	ENSP00000307272
ENSP00000297316	ENSP00000311005
ENSP00000297316	ENSP00000344456
ENSP00000297316	ENSP00000352516
ENSP00000297316	ENSP00000362068
ENSP00000297316	ENSP00000362936
ENSP00000297316	ENSP00000367406
ENSP00000297316	ENSP00000404658
ENSP00000297325	ENSP00000308268
ENSP00000297325	ENSP00000322396
ENSP00000297325	ENSP00000343657
ENSP00000297325	ENSP00000354497
ENSP00000297325	ENSP00000356272
ENSP00000297325	ENSP00000367079
ENSP00000297325	ENSP00000371475
ENSP00000297338	ENSP00000298767
ENSP00000297338	ENSP00000302530
ENSP00000297338	ENSP00000306682
ENSP00000297338	ENSP00000306999
ENSP00000297338	ENSP00000307786
ENSP00000297338	ENSP00000308944
ENSP00000297338	ENSP00000312778
ENSP00000297338	ENSP00000313171
ENSP00000297338	ENSP00000313420
ENSP00000297338	ENSP00000313851
ENSP00000297338	ENSP00000314499
ENSP00000297338	ENSP00000314508
ENSP00000297338	ENSP00000316879
ENSP00000297338	ENSP00000317985
ENSP00000297338	ENSP00000318775
ENSP00000297338	ENSP00000321636
ENSP00000297338	ENSP00000322180
ENSP00000297338	ENSP00000322570
ENSP00000297338	ENSP00000325017
ENSP00000297338	ENSP00000326261

ENSP00000297338	ENSP00000329466
ENSP00000297338	ENSP00000329558
ENSP00000297338	ENSP00000335357
ENSP00000297338	ENSP00000336606
ENSP00000297338	ENSP00000337194
ENSP00000297338	ENSP00000341382
ENSP00000297338	ENSP00000342381
ENSP00000297338	ENSP00000343081
ENSP00000297338	ENSP00000343741
ENSP00000297338	ENSP00000347168
ENSP00000297338	ENSP00000347733
ENSP00000297338	ENSP00000348551
ENSP00000297338	ENSP00000348554
ENSP00000297338	ENSP00000348722
ENSP00000297338	ENSP00000349213
ENSP00000297338	ENSP00000349748
ENSP00000297338	ENSP00000350767
ENSP00000297338	ENSP00000351284
ENSP00000297338	ENSP00000351697
ENSP00000297338	ENSP00000353332
ENSP00000297338	ENSP00000353793
ENSP00000297338	ENSP00000353904
ENSP00000297338	ENSP00000354720
ENSP00000297338	ENSP00000354916
ENSP00000297338	ENSP00000355388
ENSP00000297338	ENSP00000355596
ENSP00000297338	ENSP00000355731
ENSP00000297338	ENSP00000356448
ENSP00000297338	ENSP00000356520
ENSP00000297338	ENSP00000358140
ENSP00000297338	ENSP00000358423
ENSP00000297338	ENSP00000358478
ENSP00000297338	ENSP00000358497
ENSP00000297338	ENSP00000361310
ENSP00000297338	ENSP00000361875
ENSP00000297338	ENSP00000361990
ENSP00000297338	ENSP00000362352
ENSP00000297338	ENSP00000362413
ENSP00000297338	ENSP00000363763
ENSP00000297338	ENSP00000365113
ENSP00000297338	ENSP00000365877
ENSP00000297338	ENSP00000366974
ENSP00000297338	ENSP00000367034
ENSP00000297338	ENSP00000368017
ENSP00000297338	ENSP00000368349
ENSP00000297338	ENSP00000368438

ENSP00000297338	ENSP00000368976
ENSP00000297338	ENSP00000369411
ENSP00000297338	ENSP00000369716
ENSP00000297338	ENSP00000372689
ENSP00000297338	ENSP00000375809
ENSP00000297338	ENSP00000376309
ENSP00000297338	ENSP00000378180
ENSP00000297338	ENSP00000378529
ENSP00000297338	ENSP00000380414
ENSP00000297338	ENSP00000396068
ENSP00000297338	ENSP00000402515
ENSP00000297347	ENSP00000300651
ENSP00000297347	ENSP00000314311
ENSP00000297347	ENSP00000314949
ENSP00000297347	ENSP00000323720
ENSP00000297347	ENSP00000325612
ENSP00000297347	ENSP00000326128
ENSP00000297347	ENSP00000326767
ENSP00000297347	ENSP00000337340
ENSP00000297347	ENSP00000342343
ENSP00000297347	ENSP00000348610
ENSP00000297347	ENSP00000357047
ENSP00000297347	ENSP00000357448
ENSP00000297347	ENSP00000357907
ENSP00000297347	ENSP00000360371
ENSP00000297347	ENSP00000362948
ENSP00000297347	ENSP00000363193
ENSP00000297347	ENSP00000365588
ENSP00000297347	ENSP00000373060
ENSP00000297347	ENSP00000381216
ENSP00000297347	ENSP00000384048
ENSP00000297347	ENSP00000387429
ENSP00000297347	ENSP00000391774
ENSP00000297347	ENSP00000392617
ENSP00000297347	ENSP00000396484
ENSP00000297347	ENSP00000403852
ENSP00000297347	ENSP00000404318
ENSP00000297347	ENSP00000409167
ENSP00000297347	ENSP00000415212
ENSP00000297347	ENSP00000416511
ENSP00000297347	ENSP00000418734
ENSP00000297350	ENSP00000346839
ENSP00000297431	ENSP00000300738
ENSP00000297431	ENSP00000311768
ENSP00000297431	ENSP00000312946
ENSP00000297431	ENSP00000313171

ENSP00000297431	ENSP00000319651
ENSP00000297431	ENSP00000322180
ENSP00000297431	ENSP00000323439
ENSP00000297431	ENSP00000335357
ENSP00000297431	ENSP00000336868
ENSP00000297431	ENSP00000341382
ENSP00000297431	ENSP00000344741
ENSP00000297431	ENSP00000351947
ENSP00000297431	ENSP00000353793
ENSP00000297431	ENSP00000354720
ENSP00000297431	ENSP00000354916
ENSP00000297431	ENSP00000354957
ENSP00000297431	ENSP00000360412
ENSP00000297431	ENSP00000360532
ENSP00000297431	ENSP00000360621
ENSP00000297431	ENSP00000362532
ENSP00000297431	ENSP00000362592
ENSP00000297431	ENSP00000363021
ENSP00000297431	ENSP00000366953
ENSP00000297431	ENSP00000370741
ENSP00000297431	ENSP00000371475
ENSP00000297431	ENSP00000379612
ENSP00000297431	ENSP00000387982
ENSP00000297439	ENSP00000334002
ENSP00000297440	ENSP00000323511
ENSP00000297508	ENSP00000315644
ENSP00000297532	ENSP00000300574
ENSP00000297532	ENSP00000401371
ENSP00000297540	ENSP00000302176
ENSP00000297540	ENSP00000361014
ENSP00000297540	ENSP00000363055
ENSP00000297540	ENSP00000370557
ENSP00000297540	ENSP00000379607
ENSP00000297562	ENSP00000313829
ENSP00000297562	ENSP00000354951
ENSP00000297562	ENSP00000366607
ENSP00000297562	ENSP00000377218
ENSP00000297562	ENSP00000378350
ENSP00000297564	ENSP00000307786
ENSP00000297564	ENSP00000309565
ENSP00000297564	ENSP00000317159
ENSP00000297564	ENSP00000317780
ENSP00000297564	ENSP00000321239
ENSP00000297564	ENSP00000332887
ENSP00000297564	ENSP00000342300
ENSP00000297564	ENSP00000354499

ENSP00000297564	ENSP00000354554
ENSP00000297564	ENSP00000354876
ENSP00000297564	ENSP00000354982
ENSP00000297564	ENSP00000355572
ENSP00000297564	ENSP00000360782
ENSP00000297564	ENSP00000367934
ENSP00000297579	ENSP00000301764
ENSP00000297579	ENSP00000302111
ENSP00000297579	ENSP00000308179
ENSP00000297579	ENSP00000308332
ENSP00000297579	ENSP00000311028
ENSP00000297579	ENSP00000314193
ENSP00000297579	ENSP00000315674
ENSP00000297579	ENSP00000326981
ENSP00000297579	ENSP00000327179
ENSP00000297579	ENSP00000328854
ENSP00000297579	ENSP00000329466
ENSP00000297579	ENSP00000339145
ENSP00000297579	ENSP00000346453
ENSP00000297579	ENSP00000347495
ENSP00000297579	ENSP00000353246
ENSP00000297579	ENSP00000354040
ENSP00000297579	ENSP00000355541
ENSP00000297579	ENSP00000363642
ENSP00000297579	ENSP00000363746
ENSP00000297579	ENSP00000370258
ENSP00000297579	ENSP00000370589
ENSP00000297579	ENSP00000371101
ENSP00000297579	ENSP00000372695
ENSP00000297579	ENSP00000377944
ENSP00000297579	ENSP00000379888
ENSP00000297579	ENSP00000384302
ENSP00000297579	ENSP00000387006
ENSP00000297579	ENSP00000399454
ENSP00000297579	ENSP00000402869
ENSP00000297579	ENSP00000405614
ENSP00000297581	ENSP00000342136
ENSP00000297591	ENSP00000311827
ENSP00000297591	ENSP00000329466
ENSP00000297591	ENSP00000336868
ENSP00000297591	ENSP00000351141
ENSP00000297591	ENSP00000356520
ENSP00000297591	ENSP00000361557
ENSP00000297591	ENSP00000379607
ENSP00000297591	ENSP00000391432
ENSP00000297591	ENSP00000392601

ENSP00000297591	ENSP00000401277
ENSP00000297598	ENSP00000327070
ENSP00000297598	ENSP00000337127
ENSP00000297598	ENSP00000351885
ENSP00000297598	ENSP00000360268
ENSP00000297598	ENSP00000369100
ENSP00000297598	ENSP00000371236
ENSP00000297598	ENSP00000376268
ENSP00000297598	ENSP00000394382
ENSP00000297623	ENSP00000369816
ENSP00000297625	ENSP00000300658
ENSP00000297625	ENSP00000326699
ENSP00000297625	ENSP00000337688
ENSP00000297625	ENSP00000349970
ENSP00000297625	ENSP00000357998
ENSP00000297625	ENSP00000379042
ENSP00000297661	ENSP00000301765
ENSP00000297661	ENSP00000318629
ENSP00000297661	ENSP00000366565
ENSP00000297689	ENSP00000326272
ENSP00000297689	ENSP00000335493
ENSP00000297689	ENSP00000342136
ENSP00000297689	ENSP00000342307
ENSP00000297689	ENSP00000347294
ENSP00000297689	ENSP00000359206
ENSP00000297689	ENSP00000359290
ENSP00000297689	ENSP00000359531
ENSP00000297689	ENSP00000365025
ENSP00000297788	ENSP00000309606
ENSP00000297788	ENSP00000355596
ENSP00000297788	ENSP00000359552
ENSP00000297788	ENSP00000360532
ENSP00000297792	ENSP00000359321
ENSP00000297792	ENSP00000366156
ENSP00000297792	ENSP00000384048
ENSP00000297792	ENSP00000385746
ENSP00000297792	ENSP00000386759
ENSP00000297792	ENSP00000417132
ENSP00000297814	ENSP00000346566
ENSP00000297837	ENSP00000299601
ENSP00000297837	ENSP00000414187
ENSP00000297873	ENSP00000309433
ENSP00000297873	ENSP00000311135
ENSP00000297873	ENSP00000312870
ENSP00000297873	ENSP00000332171
ENSP00000297873	ENSP00000333920

ENSP00000297908	ENSP00000298283
ENSP00000297908	ENSP00000300151
ENSP00000297908	ENSP00000301585
ENSP00000297908	ENSP00000302896
ENSP00000297908	ENSP00000306548
ENSP00000297908	ENSP00000308897
ENSP00000297908	ENSP00000311028
ENSP00000297908	ENSP00000311430
ENSP00000297908	ENSP00000317376
ENSP00000297908	ENSP00000318646
ENSP00000297908	ENSP00000326981
ENSP00000297908	ENSP00000337396
ENSP00000297908	ENSP00000339795
ENSP00000297908	ENSP00000341082
ENSP00000297908	ENSP00000341885
ENSP00000297908	ENSP00000346015
ENSP00000297908	ENSP00000346022
ENSP00000297908	ENSP00000346067
ENSP00000297908	ENSP00000346725
ENSP00000297908	ENSP00000354525
ENSP00000297908	ENSP00000354739
ENSP00000297908	ENSP00000359345
ENSP00000297908	ENSP00000362036
ENSP00000297908	ENSP00000363018
ENSP00000297908	ENSP00000363676
ENSP00000297908	ENSP00000373404
ENSP00000297908	ENSP00000375730
ENSP00000297908	ENSP00000377486
ENSP00000297908	ENSP00000377865
ENSP00000297908	ENSP00000379339
ENSP00000297908	ENSP00000389103
ENSP00000297908	ENSP00000393241
ENSP00000297908	ENSP00000403175
ENSP00000297908	ENSP00000412583
ENSP00000297908	ENSP00000416110
ENSP00000297908	ENSP00000420714
ENSP00000297954	ENSP00000313059
ENSP00000297977	ENSP00000338157
ENSP00000297977	ENSP00000358918
ENSP00000297977	ENSP00000367029
ENSP00000297979	ENSP00000370517
ENSP00000297990	ENSP00000298746
ENSP00000297990	ENSP00000301364
ENSP00000297990	ENSP00000301962
ENSP00000297990	ENSP00000302896
ENSP00000297990	ENSP00000305702

ENSP00000297990	ENSP00000308179
ENSP00000297990	ENSP00000308332
ENSP00000297990	ENSP00000310590
ENSP00000297990	ENSP00000311135
ENSP00000297990	ENSP00000314193
ENSP00000297990	ENSP00000314348
ENSP00000297990	ENSP00000315674
ENSP00000297990	ENSP00000317992
ENSP00000297990	ENSP00000318195
ENSP00000297990	ENSP00000318604
ENSP00000297990	ENSP00000321449
ENSP00000297990	ENSP00000321711
ENSP00000297990	ENSP00000322396
ENSP00000297990	ENSP00000324122
ENSP00000297990	ENSP00000326981
ENSP00000297990	ENSP00000327179
ENSP00000297990	ENSP00000327436
ENSP00000297990	ENSP00000328720
ENSP00000297990	ENSP00000328854
ENSP00000297990	ENSP00000329466
ENSP00000297990	ENSP00000331907
ENSP00000297990	ENSP00000338862
ENSP00000297990	ENSP00000339095
ENSP00000297990	ENSP00000343190
ENSP00000297990	ENSP00000343557
ENSP00000297990	ENSP00000344393
ENSP00000297990	ENSP00000346050
ENSP00000297990	ENSP00000355471
ENSP00000297990	ENSP00000355541
ENSP00000297990	ENSP00000356134
ENSP00000297990	ENSP00000356520
ENSP00000297990	ENSP00000358563
ENSP00000297990	ENSP00000358812
ENSP00000297990	ENSP00000359688
ENSP00000297990	ENSP00000360031
ENSP00000297990	ENSP00000361642
ENSP00000297990	ENSP00000361982
ENSP00000297990	ENSP00000362744
ENSP00000297990	ENSP00000363308
ENSP00000297990	ENSP00000363642
ENSP00000297990	ENSP00000363746
ENSP00000297990	ENSP00000365025
ENSP00000297990	ENSP00000365837
ENSP00000297990	ENSP00000366519
ENSP00000297990	ENSP00000366557
ENSP00000297990	ENSP00000368887

ENSP00000297990	ENSP00000368984
ENSP00000297990	ENSP00000370589
ENSP00000297990	ENSP00000371101
ENSP00000297990	ENSP00000371201
ENSP00000297990	ENSP00000372695
ENSP00000297990	ENSP00000378529
ENSP00000297990	ENSP00000379093
ENSP00000297990	ENSP00000379888
ENSP00000297990	ENSP00000382392
ENSP00000297990	ENSP00000384302
ENSP00000297990	ENSP00000386759
ENSP00000297990	ENSP00000387303
ENSP00000297990	ENSP00000388464
ENSP00000297990	ENSP00000392601
ENSP00000297990	ENSP00000399454
ENSP00000297990	ENSP00000402869
ENSP00000297990	ENSP00000405614
ENSP00000297990	ENSP00000410396
ENSP00000297990	ENSP00000417763
ENSP00000297990	ENSP00000419740
ENSP00000297994	ENSP00000314441
ENSP00000297994	ENSP00000327821
ENSP00000297994	ENSP00000328671
ENSP00000297994	ENSP00000356476
ENSP00000297994	ENSP00000364320
ENSP00000297994	ENSP00000365837
ENSP00000298048	ENSP00000307272
ENSP00000298048	ENSP00000309132
ENSP00000298048	ENSP00000310042
ENSP00000298048	ENSP00000311677
ENSP00000298048	ENSP00000330074
ENSP00000298048	ENSP00000335321
ENSP00000298048	ENSP00000340879
ENSP00000298048	ENSP00000348273
ENSP00000298048	ENSP00000352408
ENSP00000298048	ENSP00000360532
ENSP00000298048	ENSP00000377793
ENSP00000298048	ENSP00000402084
ENSP00000298048	ENSP00000402338
ENSP00000298139	ENSP00000301280
ENSP00000298139	ENSP00000306561
ENSP00000298139	ENSP00000307491
ENSP00000298139	ENSP00000307853
ENSP00000298139	ENSP00000308944
ENSP00000298139	ENSP00000309572
ENSP00000298139	ENSP00000313350

ENSP00000298139	ENSP00000313420
ENSP00000298139	ENSP00000322242
ENSP00000298139	ENSP00000323439
ENSP00000298139	ENSP00000327197
ENSP00000298139	ENSP00000328940
ENSP00000298139	ENSP00000332171
ENSP00000298139	ENSP00000334474
ENSP00000298139	ENSP00000336606
ENSP00000298139	ENSP00000337353
ENSP00000298139	ENSP00000340305
ENSP00000298139	ENSP00000340879
ENSP00000298139	ENSP00000343392
ENSP00000298139	ENSP00000343741
ENSP00000298139	ENSP00000344741
ENSP00000298139	ENSP00000346566
ENSP00000298139	ENSP00000347232
ENSP00000298139	ENSP00000347978
ENSP00000298139	ENSP00000348020
ENSP00000298139	ENSP00000349393
ENSP00000298139	ENSP00000350249
ENSP00000298139	ENSP00000351284
ENSP00000298139	ENSP00000351885
ENSP00000298139	ENSP00000352257
ENSP00000298139	ENSP00000353793
ENSP00000298139	ENSP00000354522
ENSP00000298139	ENSP00000355153
ENSP00000298139	ENSP00000355759
ENSP00000298139	ENSP00000356520
ENSP00000298139	ENSP00000358019
ENSP00000298139	ENSP00000359454
ENSP00000298139	ENSP00000360310
ENSP00000298139	ENSP00000360532
ENSP00000298139	ENSP00000361465
ENSP00000298139	ENSP00000362441
ENSP00000298139	ENSP00000363021
ENSP00000298139	ENSP00000367029
ENSP00000298139	ENSP00000367787
ENSP00000298139	ENSP00000368438
ENSP00000298139	ENSP00000368646
ENSP00000298139	ENSP00000369335
ENSP00000298139	ENSP00000369411
ENSP00000298139	ENSP00000370150
ENSP00000298139	ENSP00000370151
ENSP00000298139	ENSP00000372023
ENSP00000298139	ENSP00000373060
ENSP00000298139	ENSP00000375809

ENSP00000298139	ENSP00000377655
ENSP00000298139	ENSP00000386259
ENSP00000298139	ENSP00000389709
ENSP00000298139	ENSP00000394649
ENSP00000298139	ENSP00000394791
ENSP00000298139	ENSP00000396704
ENSP00000298139	ENSP00000397830
ENSP00000298139	ENSP00000402527
ENSP00000298139	ENSP00000406868
ENSP00000298139	ENSP00000407047
ENSP00000298198	ENSP00000340879
ENSP00000298198	ENSP00000370150
ENSP00000298229	ENSP00000300574
ENSP00000298229	ENSP00000311344
ENSP00000298229	ENSP00000319170
ENSP00000298229	ENSP00000327145
ENSP00000298229	ENSP00000331201
ENSP00000298229	ENSP00000343657
ENSP00000298229	ENSP00000351926
ENSP00000298229	ENSP00000355136
ENSP00000298229	ENSP00000360916
ENSP00000298229	ENSP00000363985
ENSP00000298229	ENSP00000370719
ENSP00000298229	ENSP00000377148
ENSP00000298229	ENSP00000380921
ENSP00000298229	ENSP00000413520
ENSP00000298281	ENSP00000301788
ENSP00000298281	ENSP00000304704
ENSP00000298281	ENSP00000312735
ENSP00000298281	ENSP00000314949
ENSP00000298281	ENSP00000315791
ENSP00000298281	ENSP00000317872
ENSP00000298281	ENSP00000325377
ENSP00000298281	ENSP00000326261
ENSP00000298281	ENSP00000329029
ENSP00000298281	ENSP00000331817
ENSP00000298281	ENSP00000332444
ENSP00000298281	ENSP00000336752
ENSP00000298281	ENSP00000339245
ENSP00000298281	ENSP00000339353
ENSP00000298281	ENSP00000345096
ENSP00000298281	ENSP00000348010
ENSP00000298281	ENSP00000355185
ENSP00000298281	ENSP00000356146
ENSP00000298281	ENSP00000358576
ENSP00000298281	ENSP00000359531

ENSP00000298281	ENSP00000367527
ENSP00000298281	ENSP00000368017
ENSP00000298281	ENSP00000392859
ENSP00000298281	ENSP00000398208
ENSP00000298283	ENSP00000302896
ENSP00000298283	ENSP00000306548
ENSP00000298283	ENSP00000307525
ENSP00000298283	ENSP00000307889
ENSP00000298283	ENSP00000309334
ENSP00000298283	ENSP00000309565
ENSP00000298283	ENSP00000309830
ENSP00000298283	ENSP00000311028
ENSP00000298283	ENSP00000311430
ENSP00000298283	ENSP00000312311
ENSP00000298283	ENSP00000317376
ENSP00000298283	ENSP00000318646
ENSP00000298283	ENSP00000322419
ENSP00000298283	ENSP00000326981
ENSP00000298283	ENSP00000339095
ENSP00000298283	ENSP00000339795
ENSP00000298283	ENSP00000339844
ENSP00000298283	ENSP00000339845
ENSP00000298283	ENSP00000341082
ENSP00000298283	ENSP00000343745
ENSP00000298283	ENSP00000345156
ENSP00000298283	ENSP00000346015
ENSP00000298283	ENSP00000346022
ENSP00000298283	ENSP00000346027
ENSP00000298283	ENSP00000346037
ENSP00000298283	ENSP00000346050
ENSP00000298283	ENSP00000346088
ENSP00000298283	ENSP00000346725
ENSP00000298283	ENSP00000349142
ENSP00000298283	ENSP00000354739
ENSP00000298283	ENSP00000356290
ENSP00000298283	ENSP00000359345
ENSP00000298283	ENSP00000361076
ENSP00000298283	ENSP00000361287
ENSP00000298283	ENSP00000362153
ENSP00000298283	ENSP00000362744
ENSP00000298283	ENSP00000363018
ENSP00000298283	ENSP00000363676
ENSP00000298283	ENSP00000364320
ENSP00000298283	ENSP00000369757
ENSP00000298283	ENSP00000371546
ENSP00000298283	ENSP00000375730

ENSP00000298283	ENSP00000377486
ENSP00000298283	ENSP00000377640
ENSP00000298283	ENSP00000377865
ENSP00000298283	ENSP00000378160
ENSP00000298283	ENSP00000379607
ENSP00000298283	ENSP00000379888
ENSP00000298283	ENSP00000380156
ENSP00000298283	ENSP00000386717
ENSP00000298283	ENSP00000389103
ENSP00000298283	ENSP00000393241
ENSP00000298283	ENSP00000393393
ENSP00000298283	ENSP00000403175
ENSP00000298283	ENSP00000412583
ENSP00000298283	ENSP00000414321
ENSP00000298283	ENSP00000416110
ENSP00000298288	ENSP00000304102
ENSP00000298288	ENSP00000350512
ENSP00000298288	ENSP00000366729
ENSP00000298288	ENSP00000368438
ENSP00000298292	ENSP00000323275
ENSP00000298292	ENSP00000323424
ENSP00000298292	ENSP00000324122
ENSP00000298292	ENSP00000333018
ENSP00000298292	ENSP00000350011
ENSP00000298292	ENSP00000355621
ENSP00000298292	ENSP00000355899
ENSP00000298292	ENSP00000357220
ENSP00000298292	ENSP00000360497
ENSP00000298292	ENSP00000360876
ENSP00000298292	ENSP00000361010
ENSP00000298292	ENSP00000362386
ENSP00000298292	ENSP00000364298
ENSP00000298292	ENSP00000366135
ENSP00000298292	ENSP00000397530
ENSP00000298292	ENSP00000398812
ENSP00000298292	ENSP00000412439
ENSP00000298295	ENSP00000354951
ENSP00000298295	ENSP00000380702
ENSP00000298310	ENSP00000298746
ENSP00000298310	ENSP00000300648
ENSP00000298310	ENSP00000305702
ENSP00000298310	ENSP00000314311
ENSP00000298310	ENSP00000314348
ENSP00000298310	ENSP00000325312
ENSP00000298310	ENSP00000333401
ENSP00000298310	ENSP00000344460

ENSP00000298310	ENSP00000348708
ENSP00000298310	ENSP00000356290
ENSP00000298310	ENSP00000358563
ENSP00000298310	ENSP00000359345
ENSP00000298310	ENSP00000359837
ENSP00000298310	ENSP00000361982
ENSP00000298310	ENSP00000363313
ENSP00000298310	ENSP00000365773
ENSP00000298310	ENSP00000366519
ENSP00000298310	ENSP00000366672
ENSP00000298310	ENSP00000368565
ENSP00000298310	ENSP00000370258
ENSP00000298310	ENSP00000373404
ENSP00000298310	ENSP00000377486
ENSP00000298310	ENSP00000377865
ENSP00000298310	ENSP00000382392
ENSP00000298310	ENSP00000388872
ENSP00000298310	ENSP00000397598
ENSP00000298317	ENSP00000301585
ENSP00000298317	ENSP00000307567
ENSP00000298317	ENSP00000336587
ENSP00000298317	ENSP00000355890
ENSP00000298317	ENSP00000362578
ENSP00000298352	ENSP00000345917
ENSP00000298352	ENSP00000363559
ENSP00000298492	ENSP00000343412
ENSP00000298492	ENSP00000343535
ENSP00000298492	ENSP00000352408
ENSP00000298492	ENSP00000366434
ENSP00000298492	ENSP00000400500
ENSP00000298532	ENSP00000299424
ENSP00000298532	ENSP00000302478
ENSP00000298532	ENSP00000302530
ENSP00000298532	ENSP00000311135
ENSP00000298532	ENSP00000315568
ENSP00000298532	ENSP00000316240
ENSP00000298532	ENSP00000319597
ENSP00000298532	ENSP00000333667
ENSP00000298532	ENSP00000334061
ENSP00000298532	ENSP00000343557
ENSP00000298532	ENSP00000348069
ENSP00000298532	ENSP00000351137
ENSP00000298532	ENSP00000353622
ENSP00000298532	ENSP00000354251
ENSP00000298532	ENSP00000354522
ENSP00000298532	ENSP00000357631

ENSP00000298532	ENSP00000360497
ENSP00000298532	ENSP00000360532
ENSP00000298532	ENSP00000362717
ENSP00000298532	ENSP00000371236
ENSP00000298532	ENSP00000376973
ENSP00000298532	ENSP00000381657
ENSP00000298532	ENSP00000382392
ENSP00000298532	ENSP00000386759
ENSP00000298532	ENSP00000387006
ENSP00000298532	ENSP00000393953
ENSP00000298545	ENSP00000304802
ENSP00000298545	ENSP00000312042
ENSP00000298545	ENSP00000346577
ENSP00000298545	ENSP00000362993
ENSP00000298546	ENSP00000385746
ENSP00000298546	ENSP00000397435
ENSP00000298552	ENSP00000298630
ENSP00000298552	ENSP00000306100
ENSP00000298552	ENSP00000310040
ENSP00000298552	ENSP00000313034
ENSP00000298552	ENSP00000313420
ENSP00000298552	ENSP00000314499
ENSP00000298552	ENSP00000316879
ENSP00000298552	ENSP00000341189
ENSP00000298552	ENSP00000344666
ENSP00000298552	ENSP00000347733
ENSP00000298552	ENSP00000348813
ENSP00000298552	ENSP00000349748
ENSP00000298552	ENSP00000350785
ENSP00000298552	ENSP00000352121
ENSP00000298552	ENSP00000354558
ENSP00000298552	ENSP00000355231
ENSP00000298552	ENSP00000355499
ENSP00000298552	ENSP00000402515
ENSP00000298569	ENSP00000300145
ENSP00000298569	ENSP00000301587
ENSP00000298569	ENSP00000302114
ENSP00000298569	ENSP00000317780
ENSP00000298569	ENSP00000325863
ENSP00000298569	ENSP00000338345
ENSP00000298569	ENSP00000349016
ENSP00000298569	ENSP00000350249
ENSP00000298569	ENSP00000353826
ENSP00000298569	ENSP00000354876
ENSP00000298569	ENSP00000361686
ENSP00000298569	ENSP00000368140

ENSP00000298585	ENSP00000311202
ENSP00000298585	ENSP00000361990
ENSP00000298585	ENSP00000396068
ENSP00000298596	ENSP00000359258
ENSP00000298630	ENSP00000318604
ENSP00000298630	ENSP00000329933
ENSP00000298630	ENSP00000361186
ENSP00000298630	ENSP00000372857
ENSP00000298694	ENSP00000313172
ENSP00000298694	ENSP00000321106
ENSP00000298694	ENSP00000340820
ENSP00000298694	ENSP00000359100
ENSP00000298694	ENSP00000380702
ENSP00000298694	ENSP00000394863
ENSP00000298717	ENSP00000322316
ENSP00000298717	ENSP00000351141
ENSP00000298717	ENSP00000369335
ENSP00000298717	ENSP00000373474
ENSP00000298746	ENSP00000302768
ENSP00000298746	ENSP00000304668
ENSP00000298746	ENSP00000305702
ENSP00000298746	ENSP00000308332
ENSP00000298746	ENSP00000309433
ENSP00000298746	ENSP00000311135
ENSP00000298746	ENSP00000312735
ENSP00000298746	ENSP00000314311
ENSP00000298746	ENSP00000314348
ENSP00000298746	ENSP00000314949
ENSP00000298746	ENSP00000316879
ENSP00000298746	ENSP00000317992
ENSP00000298746	ENSP00000318195
ENSP00000298746	ENSP00000318646
ENSP00000298746	ENSP00000320917
ENSP00000298746	ENSP00000321449
ENSP00000298746	ENSP00000322396
ENSP00000298746	ENSP00000323858
ENSP00000298746	ENSP00000324122
ENSP00000298746	ENSP00000326806
ENSP00000298746	ENSP00000328690
ENSP00000298746	ENSP00000329715
ENSP00000298746	ENSP00000331817
ENSP00000298746	ENSP00000332103
ENSP00000298746	ENSP00000332198
ENSP00000298746	ENSP00000336712
ENSP00000298746	ENSP00000337839
ENSP00000298746	ENSP00000338573

ENSP00000298746	ENSP00000340281
ENSP00000298746	ENSP00000340766
ENSP00000298746	ENSP00000343081
ENSP00000298746	ENSP00000343557
ENSP00000298746	ENSP00000344393
ENSP00000298746	ENSP00000346120
ENSP00000298746	ENSP00000346725
ENSP00000298746	ENSP00000348722
ENSP00000298746	ENSP00000350011
ENSP00000298746	ENSP00000350249
ENSP00000298746	ENSP00000352738
ENSP00000298746	ENSP00000354040
ENSP00000298746	ENSP00000354125
ENSP00000298746	ENSP00000354812
ENSP00000298746	ENSP00000355261
ENSP00000298746	ENSP00000356476
ENSP00000298746	ENSP00000358140
ENSP00000298746	ENSP00000358812
ENSP00000298746	ENSP00000359454
ENSP00000298746	ENSP00000360031
ENSP00000298746	ENSP00000360412
ENSP00000298746	ENSP00000360532
ENSP00000298746	ENSP00000360828
ENSP00000298746	ENSP00000361076
ENSP00000298746	ENSP00000361232
ENSP00000298746	ENSP00000361465
ENSP00000298746	ENSP00000361949
ENSP00000298746	ENSP00000362153
ENSP00000298746	ENSP00000362744
ENSP00000298746	ENSP00000363559
ENSP00000298746	ENSP00000363944
ENSP00000298746	ENSP00000364289
ENSP00000298746	ENSP00000365806
ENSP00000298746	ENSP00000365837
ENSP00000298746	ENSP00000366135
ENSP00000298746	ENSP00000366557
ENSP00000298746	ENSP00000366843
ENSP00000298746	ENSP00000367029
ENSP00000298746	ENSP00000368589
ENSP00000298746	ENSP00000368887
ENSP00000298746	ENSP00000370258
ENSP00000298746	ENSP00000370589
ENSP00000298746	ENSP00000370741
ENSP00000298746	ENSP00000371451
ENSP00000298746	ENSP00000372199
ENSP00000298746	ENSP00000373090

ENSP00000298746	ENSP00000374280
ENSP00000298746	ENSP00000374390
ENSP00000298746	ENSP00000379888
ENSP00000298746	ENSP00000380129
ENSP00000298746	ENSP00000380453
ENSP00000298746	ENSP00000380982
ENSP00000298746	ENSP00000392028
ENSP00000298746	ENSP00000394290
ENSP00000298746	ENSP00000396704
ENSP00000298746	ENSP00000403576
ENSP00000298746	ENSP00000409074
ENSP00000298746	ENSP00000409367
ENSP00000298746	ENSP00000411162
ENSP00000298746	ENSP00000412566
ENSP00000298746	ENSP00000417229
ENSP00000298767	ENSP00000313851
ENSP00000298767	ENSP00000326261
ENSP00000298767	ENSP00000354720
ENSP00000298767	ENSP00000358497
ENSP00000298767	ENSP00000372689
ENSP00000298832	ENSP00000313171
ENSP00000298832	ENSP00000320503
ENSP00000298832	ENSP00000373715
ENSP00000298852	ENSP00000299259
ENSP00000298852	ENSP00000302830
ENSP00000298852	ENSP00000309474
ENSP00000298852	ENSP00000310129
ENSP00000298852	ENSP00000311083
ENSP00000298852	ENSP00000311665
ENSP00000298852	ENSP00000312326
ENSP00000298852	ENSP00000313953
ENSP00000298852	ENSP00000314004
ENSP00000298852	ENSP00000314949
ENSP00000298852	ENSP00000316328
ENSP00000298852	ENSP00000318861
ENSP00000298852	ENSP00000319977
ENSP00000298852	ENSP00000320508
ENSP00000298852	ENSP00000323246
ENSP00000298852	ENSP00000325919
ENSP00000298852	ENSP00000329466
ENSP00000298852	ENSP00000329715
ENSP00000298852	ENSP00000333122
ENSP00000298852	ENSP00000337501
ENSP00000298852	ENSP00000341243
ENSP00000298852	ENSP00000344741
ENSP00000298852	ENSP00000346453

ENSP00000298852	ENSP00000347997
ENSP00000298852	ENSP00000348442
ENSP00000298852	ENSP00000349970
ENSP00000298852	ENSP00000350708
ENSP00000298852	ENSP00000351015
ENSP00000298852	ENSP00000351908
ENSP00000298852	ENSP00000352219
ENSP00000298852	ENSP00000353622
ENSP00000298852	ENSP00000354883
ENSP00000298852	ENSP00000355153
ENSP00000298852	ENSP00000355865
ENSP00000298852	ENSP00000360025
ENSP00000298852	ENSP00000360532
ENSP00000298852	ENSP00000361699
ENSP00000298852	ENSP00000362334
ENSP00000298852	ENSP00000362399
ENSP00000298852	ENSP00000363944
ENSP00000298852	ENSP00000366249
ENSP00000298852	ENSP00000369531
ENSP00000298852	ENSP00000370222
ENSP00000298852	ENSP00000377384
ENSP00000298852	ENSP00000384211
ENSP00000298852	ENSP00000396068
ENSP00000298852	ENSP00000396937
ENSP00000298852	ENSP00000417281
ENSP00000298852	ENSP00000417748
ENSP00000298852	ENSP00000417763
ENSP00000298852	ENSP00000418734
ENSP00000298854	ENSP00000312435
ENSP00000298854	ENSP00000313829
ENSP00000298854	ENSP00000325376
ENSP00000298854	ENSP00000354201
ENSP00000298854	ENSP00000363571
ENSP00000298854	ENSP00000364212
ENSP00000298854	ENSP00000382064
ENSP00000298910	ENSP00000300688
ENSP00000298910	ENSP00000323856
ENSP00000298910	ENSP00000336712
ENSP00000298910	ENSP00000338345
ENSP00000298910	ENSP00000346550
ENSP00000298910	ENSP00000355865
ENSP00000298910	ENSP00000365147
ENSP00000298910	ENSP00000380431
ENSP00000298910	ENSP00000381293
ENSP00000298937	ENSP00000299601
ENSP00000298937	ENSP00000302801

ENSP00000298937	ENSP00000307684
ENSP00000298937	ENSP00000311665
ENSP00000298937	ENSP00000318313
ENSP00000298937	ENSP00000320566
ENSP00000298937	ENSP00000325677
ENSP00000298937	ENSP00000327716
ENSP00000298937	ENSP00000334061
ENSP00000298937	ENSP00000334188
ENSP00000298937	ENSP00000336842
ENSP00000298937	ENSP00000336868
ENSP00000298937	ENSP00000339449
ENSP00000298937	ENSP00000344504
ENSP00000298937	ENSP00000345702
ENSP00000298937	ENSP00000346453
ENSP00000298937	ENSP00000348129
ENSP00000298937	ENSP00000348722
ENSP00000298937	ENSP00000350967
ENSP00000298937	ENSP00000353622
ENSP00000298937	ENSP00000355013
ENSP00000298937	ENSP00000355051
ENSP00000298937	ENSP00000356405
ENSP00000298937	ENSP00000356641
ENSP00000298937	ENSP00000356774
ENSP00000298937	ENSP00000360116
ENSP00000298937	ENSP00000360473
ENSP00000298937	ENSP00000360515
ENSP00000298937	ENSP00000360676
ENSP00000298937	ENSP00000362576
ENSP00000298937	ENSP00000363308
ENSP00000298937	ENSP00000363779
ENSP00000298937	ENSP00000374280
ENSP00000298937	ENSP00000374399
ENSP00000298937	ENSP00000384490
ENSP00000298937	ENSP00000388320
ENSP00000298937	ENSP00000397259
ENSP00000298937	ENSP00000409275
ENSP00000298937	ENSP00000412922
ENSP00000298999	ENSP00000319240
ENSP00000298999	ENSP00000354125
ENSP00000298999	ENSP00000388330
ENSP00000299045	ENSP00000359454
ENSP00000299045	ENSP00000368565
ENSP00000299084	ENSP00000313391
ENSP00000299084	ENSP00000338127
ENSP00000299106	ENSP00000349465
ENSP00000299106	ENSP00000363921

ENSP00000299138	ENSP00000300896
ENSP00000299138	ENSP00000304292
ENSP00000299138	ENSP00000305459
ENSP00000299138	ENSP00000308496
ENSP00000299138	ENSP00000309457
ENSP00000299138	ENSP00000326534
ENSP00000299138	ENSP00000329858
ENSP00000299138	ENSP00000331201
ENSP00000299138	ENSP00000340836
ENSP00000299138	ENSP00000349437
ENSP00000299138	ENSP00000350854
ENSP00000299138	ENSP00000351346
ENSP00000299138	ENSP00000352175
ENSP00000299138	ENSP00000354522
ENSP00000299138	ENSP00000356146
ENSP00000299138	ENSP00000358715
ENSP00000299138	ENSP00000360183
ENSP00000299138	ENSP00000360215
ENSP00000299138	ENSP00000363349
ENSP00000299138	ENSP00000377665
ENSP00000299138	ENSP00000397441
ENSP00000299138	ENSP00000402240
ENSP00000299138	ENSP00000402935
ENSP00000299163	ENSP00000311579
ENSP00000299163	ENSP00000324292
ENSP00000299163	ENSP00000357914
ENSP00000299163	ENSP00000381045
ENSP00000299163	ENSP00000410926
ENSP00000299166	ENSP00000353575
ENSP00000299206	ENSP00000310129
ENSP00000299206	ENSP00000311873
ENSP00000299206	ENSP00000318977
ENSP00000299206	ENSP00000322570
ENSP00000299206	ENSP00000347836
ENSP00000299206	ENSP00000347978
ENSP00000299206	ENSP00000348442
ENSP00000299206	ENSP00000349393
ENSP00000299206	ENSP00000351284
ENSP00000299206	ENSP00000351697
ENSP00000299206	ENSP00000367787
ENSP00000299206	ENSP00000368349
ENSP00000299206	ENSP00000368438
ENSP00000299206	ENSP00000396937
ENSP00000299206	ENSP00000418734
ENSP00000299238	ENSP00000380982
ENSP00000299259	ENSP00000301764

ENSP00000299259	ENSP00000304102
ENSP00000299259	ENSP00000309474
ENSP00000299259	ENSP00000310129
ENSP00000299259	ENSP00000318775
ENSP00000299259	ENSP00000320648
ENSP00000299259	ENSP00000321711
ENSP00000299259	ENSP00000326477
ENSP00000299259	ENSP00000333568
ENSP00000299259	ENSP00000334188
ENSP00000299259	ENSP00000339109
ENSP00000299259	ENSP00000339353
ENSP00000299259	ENSP00000344456
ENSP00000299259	ENSP00000346340
ENSP00000299259	ENSP00000348442
ENSP00000299259	ENSP00000348554
ENSP00000299259	ENSP00000349970
ENSP00000299259	ENSP00000350512
ENSP00000299259	ENSP00000353622
ENSP00000299259	ENSP00000355958
ENSP00000299259	ENSP00000356331
ENSP00000299259	ENSP00000356641
ENSP00000299259	ENSP00000357965
ENSP00000299259	ENSP00000357980
ENSP00000299259	ENSP00000359206
ENSP00000299259	ENSP00000362413
ENSP00000299259	ENSP00000364929
ENSP00000299259	ENSP00000366434
ENSP00000299259	ENSP00000368253
ENSP00000299259	ENSP00000384211
ENSP00000299259	ENSP00000386394
ENSP00000299259	ENSP00000390783
ENSP00000299314	ENSP00000355596
ENSP00000299335	ENSP00000355185
ENSP00000299335	ENSP00000357973
ENSP00000299335	ENSP00000358576
ENSP00000299335	ENSP00000359976
ENSP00000299335	ENSP00000360782
ENSP00000299335	ENSP00000361042
ENSP00000299335	ENSP00000367527
ENSP00000299381	ENSP00000314004
ENSP00000299381	ENSP00000317872
ENSP00000299381	ENSP00000318775
ENSP00000299381	ENSP00000339109
ENSP00000299381	ENSP00000344936
ENSP00000299381	ENSP00000346987
ENSP00000299381	ENSP00000348554

ENSP00000299381	ENSP00000363322
ENSP00000299381	ENSP00000378350
ENSP00000299381	ENSP00000394394
ENSP00000299424	ENSP00000304704
ENSP00000299424	ENSP00000305790
ENSP00000299424	ENSP00000306682
ENSP00000299424	ENSP00000306983
ENSP00000299424	ENSP00000307208
ENSP00000299424	ENSP00000307684
ENSP00000299424	ENSP00000312411
ENSP00000299424	ENSP00000312735
ENSP00000299424	ENSP00000314949
ENSP00000299424	ENSP00000316114
ENSP00000299424	ENSP00000320949
ENSP00000299424	ENSP00000324122
ENSP00000299424	ENSP00000325677
ENSP00000299424	ENSP00000329029
ENSP00000299424	ENSP00000336750
ENSP00000299424	ENSP00000340271
ENSP00000299424	ENSP00000341805
ENSP00000299424	ENSP00000344537
ENSP00000299424	ENSP00000345702
ENSP00000299424	ENSP00000347733
ENSP00000299424	ENSP00000347978
ENSP00000299424	ENSP00000350283
ENSP00000299424	ENSP00000351113
ENSP00000299424	ENSP00000354633
ENSP00000299424	ENSP00000355013
ENSP00000299424	ENSP00000355051
ENSP00000299424	ENSP00000356405
ENSP00000299424	ENSP00000356848
ENSP00000299424	ENSP00000358854
ENSP00000299424	ENSP00000359531
ENSP00000299424	ENSP00000360515
ENSP00000299424	ENSP00000361180
ENSP00000299424	ENSP00000361824
ENSP00000299424	ENSP00000362068
ENSP00000299424	ENSP00000362424
ENSP00000299424	ENSP00000366974
ENSP00000299424	ENSP00000367406
ENSP00000299424	ENSP00000369695
ENSP00000299424	ENSP00000369716
ENSP00000299424	ENSP00000373702
ENSP00000299424	ENSP00000373952
ENSP00000299424	ENSP00000377747
ENSP00000299424	ENSP00000392028

ENSP00000299424	ENSP00000397259
ENSP00000299424	ENSP00000398824
ENSP00000299424	ENSP00000403852
ENSP00000299427	ENSP00000309572
ENSP00000299427	ENSP00000343535
ENSP00000299427	ENSP00000350249
ENSP00000299427	ENSP00000378089
ENSP00000299440	ENSP00000300574
ENSP00000299440	ENSP00000301764
ENSP00000299440	ENSP00000308620
ENSP00000299440	ENSP00000343701
ENSP00000299440	ENSP00000350767
ENSP00000299543	ENSP00000299601
ENSP00000299543	ENSP00000300651
ENSP00000299543	ENSP00000301788
ENSP00000299543	ENSP00000310260
ENSP00000299543	ENSP00000312735
ENSP00000299543	ENSP00000313420
ENSP00000299543	ENSP00000314949
ENSP00000299543	ENSP00000315693
ENSP00000299543	ENSP00000318641
ENSP00000299543	ENSP00000319169
ENSP00000299543	ENSP00000323720
ENSP00000299543	ENSP00000324124
ENSP00000299543	ENSP00000325612
ENSP00000299543	ENSP00000326767
ENSP00000299543	ENSP00000340823
ENSP00000299543	ENSP00000342889
ENSP00000299543	ENSP00000345530
ENSP00000299543	ENSP00000348610
ENSP00000299543	ENSP00000350967
ENSP00000299543	ENSP00000355013
ENSP00000299543	ENSP00000355961
ENSP00000299543	ENSP00000356146
ENSP00000299543	ENSP00000356405
ENSP00000299543	ENSP00000357047
ENSP00000299543	ENSP00000359531
ENSP00000299543	ENSP00000363779
ENSP00000299543	ENSP00000365025
ENSP00000299543	ENSP00000368066
ENSP00000299543	ENSP00000374280
ENSP00000299543	ENSP00000377969
ENSP00000299543	ENSP00000379228
ENSP00000299543	ENSP00000385722
ENSP00000299543	ENSP00000403852
ENSP00000299543	ENSP00000412566

ENSP00000299596	ENSP00000355777
ENSP00000299601	ENSP00000300648
ENSP00000299601	ENSP00000302160
ENSP00000299601	ENSP00000302801
ENSP00000299601	ENSP00000303434
ENSP00000299601	ENSP00000304593
ENSP00000299601	ENSP00000306983
ENSP00000299601	ENSP00000307208
ENSP00000299601	ENSP00000307387
ENSP00000299601	ENSP00000308944
ENSP00000299601	ENSP00000310596
ENSP00000299601	ENSP00000311005
ENSP00000299601	ENSP00000313350
ENSP00000299601	ENSP00000314776
ENSP00000299601	ENSP00000314949
ENSP00000299601	ENSP00000320566
ENSP00000299601	ENSP00000323967
ENSP00000299601	ENSP00000325136
ENSP00000299601	ENSP00000327487
ENSP00000299601	ENSP00000327716
ENSP00000299601	ENSP00000329933
ENSP00000299601	ENSP00000334188
ENSP00000299601	ENSP00000335304
ENSP00000299601	ENSP00000336856
ENSP00000299601	ENSP00000339933
ENSP00000299601	ENSP00000340271
ENSP00000299601	ENSP00000340944
ENSP00000299601	ENSP00000343190
ENSP00000299601	ENSP00000343742
ENSP00000299601	ENSP00000344456
ENSP00000299601	ENSP00000347168
ENSP00000299601	ENSP00000348089
ENSP00000299601	ENSP00000349298
ENSP00000299601	ENSP00000350199
ENSP00000299601	ENSP00000350767
ENSP00000299601	ENSP00000350967
ENSP00000299601	ENSP00000351596
ENSP00000299601	ENSP00000351665
ENSP00000299601	ENSP00000351947
ENSP00000299601	ENSP00000353622
ENSP00000299601	ENSP00000354742
ENSP00000299601	ENSP00000355013
ENSP00000299601	ENSP00000355205
ENSP00000299601	ENSP00000356405
ENSP00000299601	ENSP00000356989
ENSP00000299601	ENSP00000357360

ENSP00000299601	ENSP00000357887
ENSP00000299601	ENSP00000358283
ENSP00000299601	ENSP00000358478
ENSP00000299601	ENSP00000359518
ENSP00000299601	ENSP00000360473
ENSP00000299601	ENSP00000361642
ENSP00000299601	ENSP00000362166
ENSP00000299601	ENSP00000362334
ENSP00000299601	ENSP00000362352
ENSP00000299601	ENSP00000362592
ENSP00000299601	ENSP00000362993
ENSP00000299601	ENSP00000363734
ENSP00000299601	ENSP00000364929
ENSP00000299601	ENSP00000365025
ENSP00000299601	ENSP00000366974
ENSP00000299601	ENSP00000367034
ENSP00000299601	ENSP00000369695
ENSP00000299601	ENSP00000369716
ENSP00000299601	ENSP00000370023
ENSP00000299601	ENSP00000370151
ENSP00000299601	ENSP00000372694
ENSP00000299601	ENSP00000374213
ENSP00000299601	ENSP00000374280
ENSP00000299601	ENSP00000374399
ENSP00000299601	ENSP00000374529
ENSP00000299601	ENSP00000376097
ENSP00000299601	ENSP00000377262
ENSP00000299601	ENSP00000377747
ENSP00000299601	ENSP00000379394
ENSP00000299601	ENSP00000386134
ENSP00000299601	ENSP00000386759
ENSP00000299601	ENSP00000386992
ENSP00000299601	ENSP00000388872
ENSP00000299601	ENSP00000390475
ENSP00000299601	ENSP00000392028
ENSP00000299601	ENSP00000393596
ENSP00000299601	ENSP00000395574
ENSP00000299601	ENSP00000397259
ENSP00000299601	ENSP00000402240
ENSP00000299601	ENSP00000412319
ENSP00000299601	ENSP00000415678
ENSP00000299608	ENSP00000343412
ENSP00000299626	ENSP00000300658
ENSP00000299626	ENSP00000319979
ENSP00000299626	ENSP00000326699
ENSP00000299626	ENSP00000333813

ENSP00000299626	ENSP00000346236
ENSP00000299626	ENSP00000350263
ENSP00000299626	ENSP00000356530
ENSP00000299626	ENSP00000360149
ENSP00000299626	ENSP00000360644
ENSP00000299626	ENSP00000361667
ENSP00000299626	ENSP00000364188
ENSP00000299626	ENSP00000380793
ENSP00000299626	ENSP00000401445
ENSP00000299626	ENSP00000415203
ENSP00000299687	ENSP00000378503
ENSP00000299767	ENSP00000304592
ENSP00000299767	ENSP00000305465
ENSP00000299767	ENSP00000307235
ENSP00000299767	ENSP00000309474
ENSP00000299767	ENSP00000310129
ENSP00000299767	ENSP00000310668
ENSP00000299767	ENSP00000317331
ENSP00000299767	ENSP00000320508
ENSP00000299767	ENSP00000323313
ENSP00000299767	ENSP00000323720
ENSP00000299767	ENSP00000331268
ENSP00000299767	ENSP00000334061
ENSP00000299767	ENSP00000337127
ENSP00000299767	ENSP00000337838
ENSP00000299767	ENSP00000338533
ENSP00000299767	ENSP00000341551
ENSP00000299767	ENSP00000342181
ENSP00000299767	ENSP00000345702
ENSP00000299767	ENSP00000348565
ENSP00000299767	ENSP00000348877
ENSP00000299767	ENSP00000352456
ENSP00000299767	ENSP00000354347
ENSP00000299767	ENSP00000355296
ENSP00000299767	ENSP00000355890
ENSP00000299767	ENSP00000358140
ENSP00000299767	ENSP00000359067
ENSP00000299767	ENSP00000359526
ENSP00000299767	ENSP00000360286
ENSP00000299767	ENSP00000360532
ENSP00000299767	ENSP00000361207
ENSP00000299767	ENSP00000361686
ENSP00000299767	ENSP00000363089
ENSP00000299767	ENSP00000364188
ENSP00000299767	ENSP00000365534
ENSP00000299767	ENSP00000366179

ENSP00000299767	ENSP00000366395
ENSP00000299767	ENSP00000367440
ENSP00000299767	ENSP00000370151
ENSP00000299767	ENSP00000370589
ENSP00000299767	ENSP00000370883
ENSP00000299767	ENSP00000373952
ENSP00000299767	ENSP00000379607
ENSP00000299767	ENSP00000382595
ENSP00000299767	ENSP00000388872
ENSP00000299767	ENSP00000391944
ENSP00000299767	ENSP00000397441
ENSP00000299767	ENSP00000407487
ENSP00000299798	ENSP00000370410
ENSP00000299798	ENSP00000389399
ENSP00000299853	ENSP00000300101
ENSP00000299853	ENSP00000302478
ENSP00000299853	ENSP00000303088
ENSP00000299853	ENSP00000314949
ENSP00000299853	ENSP00000318604
ENSP00000299853	ENSP00000320917
ENSP00000299853	ENSP00000321711
ENSP00000299853	ENSP00000324124
ENSP00000299853	ENSP00000327070
ENSP00000299853	ENSP00000331111
ENSP00000299853	ENSP00000332171
ENSP00000299853	ENSP00000333920
ENSP00000299853	ENSP00000334564
ENSP00000299853	ENSP00000337478
ENSP00000299853	ENSP00000341243
ENSP00000299853	ENSP00000347345
ENSP00000299853	ENSP00000351575
ENSP00000299853	ENSP00000360286
ENSP00000299853	ENSP00000360532
ENSP00000299853	ENSP00000361465
ENSP00000299853	ENSP00000366828
ENSP00000299853	ENSP00000366974
ENSP00000299853	ENSP00000367029
ENSP00000299853	ENSP00000378286
ENSP00000299853	ENSP00000378736
ENSP00000299853	ENSP00000383503
ENSP00000299853	ENSP00000397636
ENSP00000299853	ENSP00000403852
ENSP00000299853	ENSP00000404247
ENSP00000299853	ENSP00000405636
ENSP00000299853	ENSP00000410530
ENSP00000299853	ENSP00000410954

ENSP00000299853	ENSP00000414110
ENSP00000299853	ENSP00000416599
ENSP00000300022	ENSP00000302830
ENSP00000300022	ENSP00000320623
ENSP00000300022	ENSP00000328773
ENSP00000300022	ENSP00000351221
ENSP00000300022	ENSP00000374455
ENSP00000300055	ENSP00000390849
ENSP00000300069	ENSP00000349594
ENSP00000300069	ENSP00000360112
ENSP00000300086	ENSP00000300574
ENSP00000300086	ENSP00000305631
ENSP00000300086	ENSP00000306123
ENSP00000300086	ENSP00000310723
ENSP00000300086	ENSP00000317691
ENSP00000300086	ENSP00000319240
ENSP00000300086	ENSP00000321259
ENSP00000300086	ENSP00000325863
ENSP00000300086	ENSP00000328690
ENSP00000300086	ENSP00000330005
ENSP00000300086	ENSP00000331087
ENSP00000300086	ENSP00000336866
ENSP00000300086	ENSP00000342026
ENSP00000300086	ENSP00000343023
ENSP00000300086	ENSP00000344547
ENSP00000300086	ENSP00000350249
ENSP00000300086	ENSP00000352257
ENSP00000300086	ENSP00000352408
ENSP00000300086	ENSP00000353660
ENSP00000300086	ENSP00000353701
ENSP00000300086	ENSP00000355306
ENSP00000300086	ENSP00000355759
ENSP00000300086	ENSP00000355809
ENSP00000300086	ENSP00000356231
ENSP00000300086	ENSP00000361524
ENSP00000300086	ENSP00000361894
ENSP00000300086	ENSP00000361965
ENSP00000300086	ENSP00000362170
ENSP00000300086	ENSP00000364320
ENSP00000300086	ENSP00000365439
ENSP00000300086	ENSP00000369736
ENSP00000300086	ENSP00000376903
ENSP00000300086	ENSP00000377496
ENSP00000300086	ENSP00000403536
ENSP00000300086	ENSP00000409167
ENSP00000300086	ENSP00000417281

ENSP00000300087	ENSP00000312753
ENSP00000300087	ENSP00000408910
ENSP00000300087	ENSP00000414906
ENSP00000300107	ENSP00000301585
ENSP00000300107	ENSP00000313921
ENSP00000300107	ENSP00000317334
ENSP00000300107	ENSP00000329558
ENSP00000300107	ENSP00000340019
ENSP00000300107	ENSP00000341213
ENSP00000300107	ENSP00000352408
ENSP00000300107	ENSP00000359206
ENSP00000300107	ENSP00000361287
ENSP00000300107	ENSP00000376946
ENSP00000300107	ENSP00000386284
ENSP00000300108	ENSP00000303325
ENSP00000300128	ENSP00000302955
ENSP00000300128	ENSP00000336712
ENSP00000300128	ENSP00000345656
ENSP00000300128	ENSP00000348577
ENSP00000300131	ENSP00000369716
ENSP00000300131	ENSP00000380019
ENSP00000300145	ENSP00000319323
ENSP00000300145	ENSP00000348886
ENSP00000300145	ENSP00000352257
ENSP00000300145	ENSP00000356329
ENSP00000300145	ENSP00000356587
ENSP00000300145	ENSP00000359976
ENSP00000300146	ENSP00000300413
ENSP00000300146	ENSP00000301788
ENSP00000300146	ENSP00000302160
ENSP00000300146	ENSP00000304350
ENSP00000300146	ENSP00000304593
ENSP00000300146	ENSP00000307863
ENSP00000300146	ENSP00000310448
ENSP00000300146	ENSP00000310596
ENSP00000300146	ENSP00000315035
ENSP00000300146	ENSP00000315379
ENSP00000300146	ENSP00000315693
ENSP00000300146	ENSP00000316879
ENSP00000300146	ENSP00000318195
ENSP00000300146	ENSP00000319341
ENSP00000300146	ENSP00000320503
ENSP00000300146	ENSP00000323302
ENSP00000300146	ENSP00000323377
ENSP00000300146	ENSP00000324122
ENSP00000300146	ENSP00000326806

ENSP00000300146	ENSP00000332326
ENSP00000300146	ENSP00000334188
ENSP00000300146	ENSP00000340954
ENSP00000300146	ENSP00000342374
ENSP00000300146	ENSP00000354125
ENSP00000300146	ENSP00000354522
ENSP00000300146	ENSP00000359939
ENSP00000300146	ENSP00000361949
ENSP00000300146	ENSP00000362153
ENSP00000300146	ENSP00000362413
ENSP00000300146	ENSP00000363313
ENSP00000300146	ENSP00000364813
ENSP00000300146	ENSP00000366607
ENSP00000300146	ENSP00000369409
ENSP00000300146	ENSP00000369897
ENSP00000300146	ENSP00000372883
ENSP00000300146	ENSP00000373715
ENSP00000300146	ENSP00000378350
ENSP00000300146	ENSP00000391944
ENSP00000300146	ENSP00000396704
ENSP00000300146	ENSP00000402240
ENSP00000300146	ENSP00000403345
ENSP00000300146	ENSP00000406280
ENSP00000300146	ENSP00000410758
ENSP00000300146	ENSP00000414006
ENSP00000300146	ENSP00000414634
ENSP00000300151	ENSP00000301585
ENSP00000300151	ENSP00000302896
ENSP00000300151	ENSP00000306548
ENSP00000300151	ENSP00000307525
ENSP00000300151	ENSP00000307889
ENSP00000300151	ENSP00000308275
ENSP00000300151	ENSP00000308897
ENSP00000300151	ENSP00000309334
ENSP00000300151	ENSP00000309830
ENSP00000300151	ENSP00000311028
ENSP00000300151	ENSP00000311505
ENSP00000300151	ENSP00000311648
ENSP00000300151	ENSP00000312311
ENSP00000300151	ENSP00000315017
ENSP00000300151	ENSP00000317376
ENSP00000300151	ENSP00000318646
ENSP00000300151	ENSP00000322419
ENSP00000300151	ENSP00000326411
ENSP00000300151	ENSP00000326981
ENSP00000300151	ENSP00000333401

ENSP00000300151	ENSP00000339095
ENSP00000300151	ENSP00000339795
ENSP00000300151	ENSP00000339844
ENSP00000300151	ENSP00000339845
ENSP00000300151	ENSP00000341082
ENSP00000300151	ENSP00000343745
ENSP00000300151	ENSP00000345156
ENSP00000300151	ENSP00000346015
ENSP00000300151	ENSP00000346022
ENSP00000300151	ENSP00000346027
ENSP00000300151	ENSP00000346037
ENSP00000300151	ENSP00000346050
ENSP00000300151	ENSP00000346088
ENSP00000300151	ENSP00000349142
ENSP00000300151	ENSP00000354525
ENSP00000300151	ENSP00000356290
ENSP00000300151	ENSP00000359345
ENSP00000300151	ENSP00000361076
ENSP00000300151	ENSP00000361287
ENSP00000300151	ENSP00000362399
ENSP00000300151	ENSP00000362744
ENSP00000300151	ENSP00000363018
ENSP00000300151	ENSP00000363676
ENSP00000300151	ENSP00000364320
ENSP00000300151	ENSP00000369757
ENSP00000300151	ENSP00000371546
ENSP00000300151	ENSP00000373404
ENSP00000300151	ENSP00000375730
ENSP00000300151	ENSP00000377486
ENSP00000300151	ENSP00000377640
ENSP00000300151	ENSP00000377865
ENSP00000300151	ENSP00000378160
ENSP00000300151	ENSP00000379888
ENSP00000300151	ENSP00000380156
ENSP00000300151	ENSP00000386717
ENSP00000300151	ENSP00000389103
ENSP00000300151	ENSP00000393241
ENSP00000300151	ENSP00000393393
ENSP00000300151	ENSP00000402240
ENSP00000300151	ENSP00000403175
ENSP00000300151	ENSP00000412583
ENSP00000300151	ENSP00000414321
ENSP00000300151	ENSP00000416110
ENSP00000300151	ENSP00000417602
ENSP00000300151	ENSP00000418734
ENSP00000300175	ENSP00000313829

ENSP00000300176	ENSP00000360798
ENSP00000300176	ENSP00000370719
ENSP00000300181	ENSP00000328854
ENSP00000300181	ENSP00000332681
ENSP00000300181	ENSP00000340995
ENSP00000300181	ENSP00000347538
ENSP00000300181	ENSP00000348550
ENSP00000300181	ENSP00000351492
ENSP00000300181	ENSP00000370410
ENSP00000300181	ENSP00000371729
ENSP00000300181	ENSP00000374529
ENSP00000300181	ENSP00000378350
ENSP00000300181	ENSP00000381795
ENSP00000300181	ENSP00000386394
ENSP00000300181	ENSP00000397435
ENSP00000300213	ENSP00000326981
ENSP00000300213	ENSP00000339186
ENSP00000300213	ENSP00000350512
ENSP00000300213	ENSP00000350785
ENSP00000300231	ENSP00000304051
ENSP00000300231	ENSP00000308944
ENSP00000300231	ENSP00000312778
ENSP00000300231	ENSP00000322396
ENSP00000300231	ENSP00000324792
ENSP00000300231	ENSP00000330343
ENSP00000300231	ENSP00000334675
ENSP00000300231	ENSP00000336868
ENSP00000300231	ENSP00000343392
ENSP00000300231	ENSP00000343479
ENSP00000300231	ENSP00000345731
ENSP00000300231	ENSP00000347168
ENSP00000300231	ENSP00000347719
ENSP00000300231	ENSP00000348089
ENSP00000300231	ENSP00000348708
ENSP00000300231	ENSP00000348965
ENSP00000300231	ENSP00000350767
ENSP00000300231	ENSP00000351284
ENSP00000300231	ENSP00000352257
ENSP00000300231	ENSP00000352738
ENSP00000300231	ENSP00000355596
ENSP00000300231	ENSP00000356989
ENSP00000300231	ENSP00000360621
ENSP00000300231	ENSP00000362424
ENSP00000300231	ENSP00000366395
ENSP00000300231	ENSP00000366974
ENSP00000300231	ENSP00000367034

ENSP00000300231	ENSP00000368349
ENSP00000300231	ENSP00000368976
ENSP00000300231	ENSP00000372023
ENSP00000300231	ENSP00000374566
ENSP00000300231	ENSP00000377148
ENSP00000300231	ENSP00000377793
ENSP00000300231	ENSP00000378517
ENSP00000300231	ENSP00000380271
ENSP00000300245	ENSP00000309457
ENSP00000300245	ENSP00000309595
ENSP00000300245	ENSP00000311825
ENSP00000300245	ENSP00000312042
ENSP00000300245	ENSP00000318115
ENSP00000300245	ENSP00000320869
ENSP00000300245	ENSP00000329614
ENSP00000300245	ENSP00000335481
ENSP00000300245	ENSP00000344220
ENSP00000300245	ENSP00000349465
ENSP00000300245	ENSP00000351813
ENSP00000300245	ENSP00000354791
ENSP00000300245	ENSP00000355028
ENSP00000300245	ENSP00000360252
ENSP00000300245	ENSP00000364794
ENSP00000300245	ENSP00000369810
ENSP00000300245	ENSP00000377007
ENSP00000300249	ENSP00000300584
ENSP00000300249	ENSP00000301332
ENSP00000300249	ENSP00000301729
ENSP00000300249	ENSP00000302530
ENSP00000300249	ENSP00000305664
ENSP00000300249	ENSP00000305958
ENSP00000300249	ENSP00000308944
ENSP00000300249	ENSP00000312244
ENSP00000300249	ENSP00000313171
ENSP00000300249	ENSP00000313432
ENSP00000300249	ENSP00000320130
ENSP00000300249	ENSP00000320508
ENSP00000300249	ENSP00000322180
ENSP00000300249	ENSP00000322396
ENSP00000300249	ENSP00000324792
ENSP00000300249	ENSP00000330343
ENSP00000300249	ENSP00000331310
ENSP00000300249	ENSP00000332592
ENSP00000300249	ENSP00000334188
ENSP00000300249	ENSP00000346566
ENSP00000300249	ENSP00000347602

ENSP00000300249	ENSP00000347719
ENSP00000300249	ENSP00000348163
ENSP00000300249	ENSP00000348965
ENSP00000300249	ENSP00000349238
ENSP00000300249	ENSP00000350967
ENSP00000300249	ENSP00000351665
ENSP00000300249	ENSP00000351885
ENSP00000300249	ENSP00000353793
ENSP00000300249	ENSP00000357651
ENSP00000300249	ENSP00000360065
ENSP00000300249	ENSP00000360473
ENSP00000300249	ENSP00000361800
ENSP00000300249	ENSP00000362424
ENSP00000300249	ENSP00000368976
ENSP00000300249	ENSP00000370741
ENSP00000300249	ENSP00000372023
ENSP00000300249	ENSP00000374566
ENSP00000300249	ENSP00000377793
ENSP00000300249	ENSP00000380129
ENSP00000300249	ENSP00000382562
ENSP00000300249	ENSP00000384290
ENSP00000300249	ENSP00000386394
ENSP00000300249	ENSP00000391944
ENSP00000300249	ENSP00000414402
ENSP00000300255	ENSP00000302913
ENSP00000300255	ENSP00000317872
ENSP00000300291	ENSP00000304704
ENSP00000300291	ENSP00000304903
ENSP00000300291	ENSP00000316476
ENSP00000300291	ENSP00000327704
ENSP00000300291	ENSP00000335321
ENSP00000300291	ENSP00000336752
ENSP00000300291	ENSP00000339245
ENSP00000300291	ENSP00000345530
ENSP00000300291	ENSP00000348965
ENSP00000300291	ENSP00000358335
ENSP00000300291	ENSP00000358510
ENSP00000300291	ENSP00000360532
ENSP00000300291	ENSP00000362306
ENSP00000300291	ENSP00000362948
ENSP00000300291	ENSP00000379607
ENSP00000300291	ENSP00000386161
ENSP00000300291	ENSP00000386394
ENSP00000300291	ENSP00000391774
ENSP00000300291	ENSP00000398597
ENSP00000300291	ENSP00000401566

ENSP00000300303	ENSP00000300589
ENSP00000300403	ENSP00000300574
ENSP00000300403	ENSP00000304689
ENSP00000300403	ENSP00000343701
ENSP00000300403	ENSP00000350283
ENSP00000300403	ENSP00000360532
ENSP00000300403	ENSP00000377492
ENSP00000300403	ENSP00000378529
ENSP00000300413	ENSP00000300738
ENSP00000300413	ENSP00000301740
ENSP00000300413	ENSP00000301764
ENSP00000300413	ENSP00000302160
ENSP00000300413	ENSP00000304350
ENSP00000300413	ENSP00000304370
ENSP00000300413	ENSP00000304500
ENSP00000300413	ENSP00000305790
ENSP00000300413	ENSP00000307387
ENSP00000300413	ENSP00000307863
ENSP00000300413	ENSP00000308533
ENSP00000300413	ENSP00000308944
ENSP00000300413	ENSP00000310448
ENSP00000300413	ENSP00000310596
ENSP00000300413	ENSP00000311768
ENSP00000300413	ENSP00000313674
ENSP00000300413	ENSP00000314311
ENSP00000300413	ENSP00000315035
ENSP00000300413	ENSP00000315379
ENSP00000300413	ENSP00000318861
ENSP00000300413	ENSP00000319169
ENSP00000300413	ENSP00000319341
ENSP00000300413	ENSP00000320503
ENSP00000300413	ENSP00000321706
ENSP00000300413	ENSP00000323302
ENSP00000300413	ENSP00000324122
ENSP00000300413	ENSP00000325448
ENSP00000300413	ENSP00000326806
ENSP00000300413	ENSP00000327821
ENSP00000300413	ENSP00000334148
ENSP00000300413	ENSP00000334538
ENSP00000300413	ENSP00000335321
ENSP00000300413	ENSP00000337340
ENSP00000300413	ENSP00000337405
ENSP00000300413	ENSP00000337839
ENSP00000300413	ENSP00000339095
ENSP00000300413	ENSP00000342374
ENSP00000300413	ENSP00000343742

ENSP00000300413	ENSP00000346986
ENSP00000300413	ENSP00000348010
ENSP00000300413	ENSP00000348442
ENSP00000300413	ENSP00000351137
ENSP00000300413	ENSP00000354972
ENSP00000300413	ENSP00000357631
ENSP00000300413	ENSP00000358716
ENSP00000300413	ENSP00000359127
ENSP00000300413	ENSP00000359939
ENSP00000300413	ENSP00000360532
ENSP00000300413	ENSP00000361577
ENSP00000300413	ENSP00000362110
ENSP00000300413	ENSP00000362936
ENSP00000300413	ENSP00000363313
ENSP00000300413	ENSP00000363384
ENSP00000300413	ENSP00000364289
ENSP00000300413	ENSP00000364813
ENSP00000300413	ENSP00000366030
ENSP00000300413	ENSP00000366557
ENSP00000300413	ENSP00000366607
ENSP00000300413	ENSP00000370083
ENSP00000300413	ENSP00000370119
ENSP00000300413	ENSP00000371236
ENSP00000300413	ENSP00000371546
ENSP00000300413	ENSP00000372883
ENSP00000300413	ENSP00000373715
ENSP00000300413	ENSP00000376758
ENSP00000300413	ENSP00000376973
ENSP00000300413	ENSP00000400591
ENSP00000300413	ENSP00000403345
ENSP00000300413	ENSP00000406280
ENSP00000300413	ENSP00000410758
ENSP00000300413	ENSP00000412566
ENSP00000300413	ENSP00000414006
ENSP00000300413	ENSP00000414634
ENSP00000300417	ENSP00000313881
ENSP00000300417	ENSP00000322579
ENSP00000300417	ENSP00000347836
ENSP00000300417	ENSP00000379500
ENSP00000300441	ENSP00000348762
ENSP00000300452	ENSP00000322316
ENSP00000300452	ENSP00000355739
ENSP00000300452	ENSP00000359385
ENSP00000300482	ENSP00000399511
ENSP00000300574	ENSP00000302530
ENSP00000300574	ENSP00000303507

ENSP00000300574	ENSP00000308774
ENSP00000300574	ENSP00000309230
ENSP00000300574	ENSP00000310561
ENSP00000300574	ENSP00000313829
ENSP00000300574	ENSP00000314036
ENSP00000300574	ENSP00000316460
ENSP00000300574	ENSP00000317327
ENSP00000300574	ENSP00000323424
ENSP00000300574	ENSP00000323856
ENSP00000300574	ENSP00000327145
ENSP00000300574	ENSP00000329357
ENSP00000300574	ENSP00000335306
ENSP00000300574	ENSP00000341138
ENSP00000300574	ENSP00000341189
ENSP00000300574	ENSP00000341208
ENSP00000300574	ENSP00000341658
ENSP00000300574	ENSP00000341957
ENSP00000300574	ENSP00000346879
ENSP00000300574	ENSP00000347942
ENSP00000300574	ENSP00000350249
ENSP00000300574	ENSP00000350297
ENSP00000300574	ENSP00000351811
ENSP00000300574	ENSP00000351926
ENSP00000300574	ENSP00000352121
ENSP00000300574	ENSP00000352425
ENSP00000300574	ENSP00000353154
ENSP00000300574	ENSP00000353462
ENSP00000300574	ENSP00000354341
ENSP00000300574	ENSP00000356191
ENSP00000300574	ENSP00000356595
ENSP00000300574	ENSP00000356694
ENSP00000300574	ENSP00000359489
ENSP00000300574	ENSP00000360798
ENSP00000300574	ENSP00000361202
ENSP00000300574	ENSP00000361264
ENSP00000300574	ENSP00000363649
ENSP00000300574	ENSP00000363745
ENSP00000300574	ENSP00000363763
ENSP00000300574	ENSP00000364524
ENSP00000300574	ENSP00000364912
ENSP00000300574	ENSP00000365016
ENSP00000300574	ENSP00000366603
ENSP00000300574	ENSP00000366936
ENSP00000300574	ENSP00000367439
ENSP00000300574	ENSP00000367787
ENSP00000300574	ENSP00000367972

ENSP00000300574	ENSP00000371341
ENSP00000300574	ENSP00000373952
ENSP00000300574	ENSP00000379158
ENSP00000300574	ENSP00000379946
ENSP00000300574	ENSP00000380921
ENSP00000300574	ENSP00000384675
ENSP00000300574	ENSP00000394085
ENSP00000300574	ENSP00000400258
ENSP00000300574	ENSP00000402084
ENSP00000300574	ENSP00000411286
ENSP00000300574	ENSP00000416097
ENSP00000300584	ENSP00000302530
ENSP00000300584	ENSP00000305503
ENSP00000300584	ENSP00000310668
ENSP00000300584	ENSP00000325863
ENSP00000300584	ENSP00000327009
ENSP00000300584	ENSP00000335357
ENSP00000300584	ENSP00000349785
ENSP00000300584	ENSP00000351665
ENSP00000300584	ENSP00000354916
ENSP00000300584	ENSP00000354927
ENSP00000300584	ENSP00000355721
ENSP00000300584	ENSP00000356579
ENSP00000300584	ENSP00000362578
ENSP00000300584	ENSP00000365005
ENSP00000300584	ENSP00000367030
ENSP00000300584	ENSP00000382423
ENSP00000300584	ENSP00000382562
ENSP00000300589	ENSP00000315112
ENSP00000300589	ENSP00000335657
ENSP00000300589	ENSP00000348632
ENSP00000300589	ENSP00000354159
ENSP00000300589	ENSP00000381698
ENSP00000300648	ENSP00000303315
ENSP00000300648	ENSP00000304668
ENSP00000300648	ENSP00000306080
ENSP00000300648	ENSP00000307272
ENSP00000300648	ENSP00000307525
ENSP00000300648	ENSP00000310448
ENSP00000300648	ENSP00000310668
ENSP00000300648	ENSP00000312370
ENSP00000300648	ENSP00000313603
ENSP00000300648	ENSP00000316879
ENSP00000300648	ENSP00000317334
ENSP00000300648	ENSP00000320309
ENSP00000300648	ENSP00000325448

ENSP00000300648	ENSP00000327179
ENSP00000300648	ENSP00000327821
ENSP00000300648	ENSP00000328690
ENSP00000300648	ENSP00000329466
ENSP00000300648	ENSP00000329715
ENSP00000300648	ENSP00000331817
ENSP00000300648	ENSP00000336712
ENSP00000300648	ENSP00000337340
ENSP00000300648	ENSP00000338788
ENSP00000300648	ENSP00000339145
ENSP00000300648	ENSP00000340019
ENSP00000300648	ENSP00000341213
ENSP00000300648	ENSP00000341243
ENSP00000300648	ENSP00000341382
ENSP00000300648	ENSP00000343081
ENSP00000300648	ENSP00000346453
ENSP00000300648	ENSP00000348069
ENSP00000300648	ENSP00000348577
ENSP00000300648	ENSP00000348714
ENSP00000300648	ENSP00000348722
ENSP00000300648	ENSP00000348877
ENSP00000300648	ENSP00000351284
ENSP00000300648	ENSP00000352400
ENSP00000300648	ENSP00000353622
ENSP00000300648	ENSP00000353741
ENSP00000300648	ENSP00000354347
ENSP00000300648	ENSP00000354720
ENSP00000300648	ENSP00000355013
ENSP00000300648	ENSP00000355205
ENSP00000300648	ENSP00000356405
ENSP00000300648	ENSP00000359345
ENSP00000300648	ENSP00000359454
ENSP00000300648	ENSP00000360286
ENSP00000300648	ENSP00000360532
ENSP00000300648	ENSP00000361245
ENSP00000300648	ENSP00000361287
ENSP00000300648	ENSP00000361699
ENSP00000300648	ENSP00000362105
ENSP00000300648	ENSP00000363250
ENSP00000300648	ENSP00000363944
ENSP00000300648	ENSP00000364320
ENSP00000300648	ENSP00000364543
ENSP00000300648	ENSP00000365837
ENSP00000300648	ENSP00000366135
ENSP00000300648	ENSP00000369042
ENSP00000300648	ENSP00000369897

ENSP00000300648	ENSP00000372093
ENSP00000300648	ENSP00000372199
ENSP00000300648	ENSP00000372827
ENSP00000300648	ENSP00000373082
ENSP00000300648	ENSP00000374213
ENSP00000300648	ENSP00000374280
ENSP00000300648	ENSP00000375809
ENSP00000300648	ENSP00000376946
ENSP00000300648	ENSP00000377344
ENSP00000300648	ENSP00000377944
ENSP00000300648	ENSP00000377954
ENSP00000300648	ENSP00000379217
ENSP00000300648	ENSP00000380982
ENSP00000300648	ENSP00000381863
ENSP00000300648	ENSP00000386134
ENSP00000300648	ENSP00000388920
ENSP00000300648	ENSP00000389065
ENSP00000300648	ENSP00000391944
ENSP00000300648	ENSP00000394400
ENSP00000300648	ENSP00000397441
ENSP00000300648	ENSP00000399530
ENSP00000300648	ENSP00000400626
ENSP00000300648	ENSP00000402240
ENSP00000300648	ENSP00000402338
ENSP00000300648	ENSP00000411327
ENSP00000300648	ENSP00000412310
ENSP00000300648	ENSP00000412553
ENSP00000300648	ENSP00000413319
ENSP00000300648	ENSP00000414373
ENSP00000300648	ENSP00000420357
ENSP00000300651	ENSP00000301838
ENSP00000300651	ENSP00000305422
ENSP00000300651	ENSP00000314949
ENSP00000300651	ENSP00000323720
ENSP00000300651	ENSP00000325612
ENSP00000300651	ENSP00000326767
ENSP00000300651	ENSP00000328228
ENSP00000300651	ENSP00000336750
ENSP00000300651	ENSP00000337340
ENSP00000300651	ENSP00000342343
ENSP00000300651	ENSP00000345681
ENSP00000300651	ENSP00000346634
ENSP00000300651	ENSP00000346886
ENSP00000300651	ENSP00000347733
ENSP00000300651	ENSP00000348069
ENSP00000300651	ENSP00000348610

ENSP00000300651	ENSP00000350283
ENSP00000300651	ENSP00000351894
ENSP00000300651	ENSP00000354681
ENSP00000300651	ENSP00000354951
ENSP00000300651	ENSP00000355759
ENSP00000300651	ENSP00000357047
ENSP00000300651	ENSP00000357907
ENSP00000300651	ENSP00000360515
ENSP00000300651	ENSP00000362948
ENSP00000300651	ENSP00000363193
ENSP00000300651	ENSP00000403852
ENSP00000300651	ENSP00000417281
ENSP00000300658	ENSP00000306124
ENSP00000300658	ENSP00000308496
ENSP00000300658	ENSP00000311200
ENSP00000300658	ENSP00000319979
ENSP00000300658	ENSP00000326699
ENSP00000300658	ENSP00000340466
ENSP00000300658	ENSP00000346809
ENSP00000300658	ENSP00000349970
ENSP00000300658	ENSP00000359552
ENSP00000300658	ENSP00000372697
ENSP00000300658	ENSP00000376268
ENSP00000300658	ENSP00000390831
ENSP00000300658	ENSP00000391197
ENSP00000300658	ENSP00000402240
ENSP00000300658	ENSP00000404016
ENSP00000300658	ENSP00000406988
ENSP00000300658	ENSP00000409952
ENSP00000300659	ENSP00000306245
ENSP00000300659	ENSP00000333920
ENSP00000300659	ENSP00000384523
ENSP00000300688	ENSP00000301585
ENSP00000300688	ENSP00000301587
ENSP00000300688	ENSP00000306003
ENSP00000300688	ENSP00000307275
ENSP00000300688	ENSP00000320236
ENSP00000300688	ENSP00000358737
ENSP00000300688	ENSP00000370414
ENSP00000300688	ENSP00000387262
ENSP00000300688	ENSP00000389649
ENSP00000300688	ENSP00000397435
ENSP00000300738	ENSP00000302111
ENSP00000300738	ENSP00000302955
ENSP00000300738	ENSP00000311873
ENSP00000300738	ENSP00000312870

ENSP00000300738	ENSP00000313171
ENSP00000300738	ENSP00000315644
ENSP00000300738	ENSP00000318977
ENSP00000300738	ENSP00000323439
ENSP00000300738	ENSP00000332208
ENSP00000300738	ENSP00000341243
ENSP00000300738	ENSP00000341382
ENSP00000300738	ENSP00000343741
ENSP00000300738	ENSP00000344460
ENSP00000300738	ENSP00000344648
ENSP00000300738	ENSP00000347232
ENSP00000300738	ENSP00000347836
ENSP00000300738	ENSP00000347978
ENSP00000300738	ENSP00000348565
ENSP00000300738	ENSP00000351015
ENSP00000300738	ENSP00000352738
ENSP00000300738	ENSP00000354558
ENSP00000300738	ENSP00000354720
ENSP00000300738	ENSP00000354957
ENSP00000300738	ENSP00000355629
ENSP00000300738	ENSP00000357283
ENSP00000300738	ENSP00000360031
ENSP00000300738	ENSP00000360268
ENSP00000300738	ENSP00000360782
ENSP00000300738	ENSP00000361699
ENSP00000300738	ENSP00000362344
ENSP00000300738	ENSP00000369411
ENSP00000300738	ENSP00000370151
ENSP00000300738	ENSP00000372023
ENSP00000300738	ENSP00000372316
ENSP00000300738	ENSP00000379607
ENSP00000300738	ENSP00000396937
ENSP00000300738	ENSP00000397598
ENSP00000300738	ENSP00000397644
ENSP00000300738	ENSP00000402515
ENSP00000300738	ENSP00000420588
ENSP00000300784	ENSP00000359211
ENSP00000300835	ENSP00000340237
ENSP00000300896	ENSP00000305682
ENSP00000300896	ENSP00000320309
ENSP00000300896	ENSP00000321449
ENSP00000300896	ENSP00000367498
ENSP00000300896	ENSP00000368438
ENSP00000300896	ENSP00000380460
ENSP00000300917	ENSP00000402515
ENSP00000301011	ENSP00000315859

ENSP00000301011	ENSP00000329466
ENSP00000301011	ENSP00000351947
ENSP00000301012	ENSP00000320658
ENSP00000301012	ENSP00000362576
ENSP00000301012	ENSP00000367208
ENSP00000301012	ENSP00000386621
ENSP00000301019	ENSP00000301764
ENSP00000301019	ENSP00000302530
ENSP00000301019	ENSP00000312870
ENSP00000301019	ENSP00000313420
ENSP00000301019	ENSP00000322915
ENSP00000301019	ENSP00000324020
ENSP00000301019	ENSP00000336587
ENSP00000301019	ENSP00000342434
ENSP00000301019	ENSP00000343741
ENSP00000301019	ENSP00000350094
ENSP00000301019	ENSP00000350512
ENSP00000301019	ENSP00000354522
ENSP00000301019	ENSP00000355922
ENSP00000301019	ENSP00000357283
ENSP00000301019	ENSP00000360532
ENSP00000301019	ENSP00000360621
ENSP00000301019	ENSP00000362578
ENSP00000301019	ENSP00000366030
ENSP00000301019	ENSP00000368438
ENSP00000301019	ENSP00000378350
ENSP00000301019	ENSP00000378529
ENSP00000301019	ENSP00000384109
ENSP00000301019	ENSP00000394394
ENSP00000301019	ENSP00000402515
ENSP00000301021	ENSP00000349824
ENSP00000301021	ENSP00000369071
ENSP00000301021	ENSP00000409231
ENSP00000301030	ENSP00000399968
ENSP00000301180	ENSP00000348762
ENSP00000301263	ENSP00000346227
ENSP00000301280	ENSP00000302896
ENSP00000301280	ENSP00000309606
ENSP00000301280	ENSP00000312697
ENSP00000301280	ENSP00000313059
ENSP00000301280	ENSP00000313420
ENSP00000301280	ENSP00000315700
ENSP00000301280	ENSP00000324122
ENSP00000301280	ENSP00000327077
ENSP00000301280	ENSP00000330343
ENSP00000301280	ENSP00000336868

ENSP00000301280	ENSP00000347232
ENSP00000301280	ENSP00000350767
ENSP00000301280	ENSP00000352257
ENSP00000301280	ENSP00000357965
ENSP00000301280	ENSP00000362592
ENSP00000301280	ENSP00000366974
ENSP00000301280	ENSP00000368438
ENSP00000301280	ENSP00000379607
ENSP00000301280	ENSP00000384026
ENSP00000301280	ENSP00000384490
ENSP00000301281	ENSP00000309031
ENSP00000301281	ENSP00000321617
ENSP00000301281	ENSP00000366395
ENSP00000301281	ENSP00000375881
ENSP00000301281	ENSP00000379457
ENSP00000301281	ENSP00000380460
ENSP00000301327	ENSP00000346142
ENSP00000301329	ENSP00000304185
ENSP00000301329	ENSP00000307004
ENSP00000301329	ENSP00000311768
ENSP00000301329	ENSP00000318821
ENSP00000301329	ENSP00000326813
ENSP00000301329	ENSP00000340019
ENSP00000301329	ENSP00000349022
ENSP00000301329	ENSP00000356331
ENSP00000301329	ENSP00000357440
ENSP00000301329	ENSP00000358854
ENSP00000301329	ENSP00000359444
ENSP00000301329	ENSP00000360286
ENSP00000301329	ENSP00000362094
ENSP00000301329	ENSP00000373509
ENSP00000301329	ENSP00000386227
ENSP00000301332	ENSP00000302530
ENSP00000301332	ENSP00000307684
ENSP00000301332	ENSP00000341848
ENSP00000301332	ENSP00000346566
ENSP00000301332	ENSP00000347719
ENSP00000301332	ENSP00000348573
ENSP00000301332	ENSP00000348965
ENSP00000301332	ENSP00000352572
ENSP00000301332	ENSP00000354486
ENSP00000301332	ENSP00000355922
ENSP00000301332	ENSP00000374566
ENSP00000301332	ENSP00000377793
ENSP00000301332	ENSP00000382562
ENSP00000301332	ENSP00000383145

ENSP00000301332	ENSP00000414402
ENSP00000301335	ENSP00000352522
ENSP00000301336	ENSP00000321239
ENSP00000301364	ENSP00000302768
ENSP00000301364	ENSP00000302896
ENSP00000301364	ENSP00000306682
ENSP00000301364	ENSP00000308897
ENSP00000301364	ENSP00000309433
ENSP00000301364	ENSP00000312411
ENSP00000301364	ENSP00000315674
ENSP00000301364	ENSP00000316879
ENSP00000301364	ENSP00000320917
ENSP00000301364	ENSP00000326981
ENSP00000301364	ENSP00000329466
ENSP00000301364	ENSP00000330343
ENSP00000301364	ENSP00000331817
ENSP00000301364	ENSP00000334836
ENSP00000301364	ENSP00000338573
ENSP00000301364	ENSP00000338862
ENSP00000301364	ENSP00000339095
ENSP00000301364	ENSP00000340281
ENSP00000301364	ENSP00000342381
ENSP00000301364	ENSP00000345917
ENSP00000301364	ENSP00000347087
ENSP00000301364	ENSP00000354125
ENSP00000301364	ENSP00000354739
ENSP00000301364	ENSP00000355471
ENSP00000301364	ENSP00000356134
ENSP00000301364	ENSP00000356548
ENSP00000301364	ENSP00000356591
ENSP00000301364	ENSP00000358605
ENSP00000301364	ENSP00000360031
ENSP00000301364	ENSP00000361949
ENSP00000301364	ENSP00000362153
ENSP00000301364	ENSP00000362744
ENSP00000301364	ENSP00000363642
ENSP00000301364	ENSP00000363708
ENSP00000301364	ENSP00000364126
ENSP00000301364	ENSP00000364293
ENSP00000301364	ENSP00000365806
ENSP00000301364	ENSP00000368589
ENSP00000301364	ENSP00000369757
ENSP00000301364	ENSP00000373090
ENSP00000301364	ENSP00000373702
ENSP00000301364	ENSP00000378529
ENSP00000301364	ENSP00000379888

ENSP00000301364	ENSP00000386456
ENSP00000301364	ENSP00000394290
ENSP00000301364	ENSP00000403576
ENSP00000301364	ENSP00000409074
ENSP00000301364	ENSP00000409367
ENSP00000301364	ENSP00000411162
ENSP00000301364	ENSP00000414321
ENSP00000301364	ENSP00000417229
ENSP00000301364	ENSP00000420588
ENSP00000301455	ENSP00000341189
ENSP00000301459	ENSP00000339826
ENSP00000301459	ENSP00000355013
ENSP00000301463	ENSP00000302830
ENSP00000301463	ENSP00000320623
ENSP00000301463	ENSP00000351221
ENSP00000301488	ENSP00000347495
ENSP00000301488	ENSP00000384179
ENSP00000301585	ENSP00000301587
ENSP00000301585	ENSP00000301729
ENSP00000301585	ENSP00000302160
ENSP00000301585	ENSP00000302896
ENSP00000301585	ENSP00000304311
ENSP00000301585	ENSP00000305653
ENSP00000301585	ENSP00000305682
ENSP00000301585	ENSP00000305696
ENSP00000301585	ENSP00000306080
ENSP00000301585	ENSP00000306548
ENSP00000301585	ENSP00000306574
ENSP00000301585	ENSP00000307889
ENSP00000301585	ENSP00000308275
ENSP00000301585	ENSP00000308717
ENSP00000301585	ENSP00000308897
ENSP00000301585	ENSP00000309595
ENSP00000301585	ENSP00000310785
ENSP00000301585	ENSP00000310933
ENSP00000301585	ENSP00000310951
ENSP00000301585	ENSP00000311368
ENSP00000301585	ENSP00000311766
ENSP00000301585	ENSP00000312311
ENSP00000301585	ENSP00000312356
ENSP00000301585	ENSP00000315017
ENSP00000301585	ENSP00000315397
ENSP00000301585	ENSP00000315774
ENSP00000301585	ENSP00000317376
ENSP00000301585	ENSP00000318158
ENSP00000301585	ENSP00000318646

ENSP00000301585	ENSP00000319501
ENSP00000301585	ENSP00000320184
ENSP00000301585	ENSP00000320658
ENSP00000301585	ENSP00000321503
ENSP00000301585	ENSP00000322450
ENSP00000301585	ENSP00000323065
ENSP00000301585	ENSP00000323076
ENSP00000301585	ENSP00000324100
ENSP00000301585	ENSP00000325448
ENSP00000301585	ENSP00000326981
ENSP00000301585	ENSP00000331849
ENSP00000301585	ENSP00000332340
ENSP00000301585	ENSP00000333401
ENSP00000301585	ENSP00000333667
ENSP00000301585	ENSP00000333837
ENSP00000301585	ENSP00000337358
ENSP00000301585	ENSP00000338799
ENSP00000301585	ENSP00000339844
ENSP00000301585	ENSP00000340019
ENSP00000301585	ENSP00000341082
ENSP00000301585	ENSP00000341692
ENSP00000301585	ENSP00000342502
ENSP00000301585	ENSP00000343657
ENSP00000301585	ENSP00000343741
ENSP00000301585	ENSP00000347581
ENSP00000301585	ENSP00000348886
ENSP00000301585	ENSP00000349142
ENSP00000301585	ENSP00000352678
ENSP00000301585	ENSP00000353826
ENSP00000301585	ENSP00000354086
ENSP00000301585	ENSP00000354522
ENSP00000301585	ENSP00000354525
ENSP00000301585	ENSP00000354580
ENSP00000301585	ENSP00000354876
ENSP00000301585	ENSP00000355692
ENSP00000301585	ENSP00000355739
ENSP00000301585	ENSP00000356001
ENSP00000301585	ENSP00000356134
ENSP00000301585	ENSP00000356972
ENSP00000301585	ENSP00000357823
ENSP00000301585	ENSP00000358737
ENSP00000301585	ENSP00000360268
ENSP00000301585	ENSP00000360327
ENSP00000301585	ENSP00000360498
ENSP00000301585	ENSP00000360777
ENSP00000301585	ENSP00000360782

ENSP00000301585	ENSP00000361084
ENSP00000301585	ENSP00000361206
ENSP00000301585	ENSP00000361923
ENSP00000301585	ENSP00000361982
ENSP00000301585	ENSP00000362036
ENSP00000301585	ENSP00000362144
ENSP00000301585	ENSP00000362208
ENSP00000301585	ENSP00000362608
ENSP00000301585	ENSP00000363999
ENSP00000301585	ENSP00000365534
ENSP00000301585	ENSP00000365837
ENSP00000301585	ENSP00000367923
ENSP00000301585	ENSP00000368389
ENSP00000301585	ENSP00000368605
ENSP00000301585	ENSP00000368646
ENSP00000301585	ENSP00000369682
ENSP00000301585	ENSP00000370023
ENSP00000301585	ENSP00000370737
ENSP00000301585	ENSP00000370930
ENSP00000301585	ENSP00000371634
ENSP00000301585	ENSP00000372445
ENSP00000301585	ENSP00000373331
ENSP00000301585	ENSP00000373404
ENSP00000301585	ENSP00000377040
ENSP00000301585	ENSP00000377486
ENSP00000301585	ENSP00000380531
ENSP00000301585	ENSP00000382250
ENSP00000301585	ENSP00000386227
ENSP00000301585	ENSP00000388658
ENSP00000301585	ENSP00000392601
ENSP00000301585	ENSP00000393241
ENSP00000301585	ENSP00000393953
ENSP00000301585	ENSP00000397790
ENSP00000301585	ENSP00000398494
ENSP00000301585	ENSP00000400513
ENSP00000301585	ENSP00000403175
ENSP00000301585	ENSP00000412583
ENSP00000301585	ENSP00000416110
ENSP00000301585	ENSP00000417602
ENSP00000301585	ENSP00000419370
ENSP00000301585	ENSP00000419740
ENSP00000301585	ENSP00000420588
ENSP00000301585	ENSP00000420714
ENSP00000301587	ENSP00000306003
ENSP00000301587	ENSP00000348886
ENSP00000301587	ENSP00000349142

ENSP00000301587	ENSP00000353826
ENSP00000301587	ENSP00000354632
ENSP00000301587	ENSP00000356329
ENSP00000301587	ENSP00000358737
ENSP00000301587	ENSP00000367934
ENSP00000301587	ENSP00000370414
ENSP00000301587	ENSP00000387262
ENSP00000301587	ENSP00000389649
ENSP00000301587	ENSP00000389934
ENSP00000301607	ENSP00000357731
ENSP00000301607	ENSP00000357753
ENSP00000301634	ENSP00000304102
ENSP00000301634	ENSP00000378529
ENSP00000301727	ENSP00000313420
ENSP00000301727	ENSP00000343741
ENSP00000301727	ENSP00000355153
ENSP00000301727	ENSP00000358404
ENSP00000301729	ENSP00000317376
ENSP00000301729	ENSP00000325136
ENSP00000301729	ENSP00000326981
ENSP00000301729	ENSP00000327070
ENSP00000301729	ENSP00000337127
ENSP00000301729	ENSP00000339479
ENSP00000301729	ENSP00000349016
ENSP00000301729	ENSP00000350249
ENSP00000301729	ENSP00000362036
ENSP00000301729	ENSP00000362413
ENSP00000301729	ENSP00000368438
ENSP00000301729	ENSP00000368605
ENSP00000301729	ENSP00000368755
ENSP00000301729	ENSP00000372445
ENSP00000301729	ENSP00000392330
ENSP00000301740	ENSP00000301962
ENSP00000301740	ENSP00000302160
ENSP00000301740	ENSP00000303191
ENSP00000301740	ENSP00000304350
ENSP00000301740	ENSP00000304370
ENSP00000301740	ENSP00000305790
ENSP00000301740	ENSP00000307863
ENSP00000301740	ENSP00000310040
ENSP00000301740	ENSP00000310448
ENSP00000301740	ENSP00000315859
ENSP00000301740	ENSP00000318861
ENSP00000301740	ENSP00000320184
ENSP00000301740	ENSP00000322016
ENSP00000301740	ENSP00000323856

ENSP00000301740	ENSP00000325376
ENSP00000301740	ENSP00000326261
ENSP00000301740	ENSP00000326806
ENSP00000301740	ENSP00000329466
ENSP00000301740	ENSP00000331907
ENSP00000301740	ENSP00000332103
ENSP00000301740	ENSP00000333837
ENSP00000301740	ENSP00000334148
ENSP00000301740	ENSP00000336752
ENSP00000301740	ENSP00000337839
ENSP00000301740	ENSP00000339090
ENSP00000301740	ENSP00000339095
ENSP00000301740	ENSP00000340019
ENSP00000301740	ENSP00000341885
ENSP00000301740	ENSP00000342374
ENSP00000301740	ENSP00000343557
ENSP00000301740	ENSP00000346050
ENSP00000301740	ENSP00000346120
ENSP00000301740	ENSP00000349124
ENSP00000301740	ENSP00000349748
ENSP00000301740	ENSP00000352257
ENSP00000301740	ENSP00000354125
ENSP00000301740	ENSP00000356541
ENSP00000301740	ENSP00000357283
ENSP00000301740	ENSP00000357863
ENSP00000301740	ENSP00000358242
ENSP00000301740	ENSP00000358554
ENSP00000301740	ENSP00000359727
ENSP00000301740	ENSP00000360147
ENSP00000301740	ENSP00000360532
ENSP00000301740	ENSP00000361076
ENSP00000301740	ENSP00000362110
ENSP00000301740	ENSP00000362546
ENSP00000301740	ENSP00000362592
ENSP00000301740	ENSP00000362688
ENSP00000301740	ENSP00000362900
ENSP00000301740	ENSP00000363591
ENSP00000301740	ENSP00000364289
ENSP00000301740	ENSP00000366557
ENSP00000301740	ENSP00000374213
ENSP00000301740	ENSP00000374455
ENSP00000301740	ENSP00000376973
ENSP00000301740	ENSP00000379607
ENSP00000301740	ENSP00000380921
ENSP00000301740	ENSP00000382250
ENSP00000301740	ENSP00000391249

ENSP00000301740	ENSP00000391944
ENSP00000301740	ENSP00000410758
ENSP00000301740	ENSP00000412566
ENSP00000301761	ENSP00000387262
ENSP00000301764	ENSP00000302160
ENSP00000301764	ENSP00000303191
ENSP00000301764	ENSP00000304102
ENSP00000301764	ENSP00000304350
ENSP00000301764	ENSP00000304370
ENSP00000301764	ENSP00000307305
ENSP00000301764	ENSP00000308620
ENSP00000301764	ENSP00000310448
ENSP00000301764	ENSP00000311513
ENSP00000301764	ENSP00000311825
ENSP00000301764	ENSP00000311873
ENSP00000301764	ENSP00000313034
ENSP00000301764	ENSP00000313420
ENSP00000301764	ENSP00000314949
ENSP00000301764	ENSP00000315379
ENSP00000301764	ENSP00000318313
ENSP00000301764	ENSP00000324122
ENSP00000301764	ENSP00000326531
ENSP00000301764	ENSP00000326806
ENSP00000301764	ENSP00000329466
ENSP00000301764	ENSP00000330753
ENSP00000301764	ENSP00000331817
ENSP00000301764	ENSP00000332995
ENSP00000301764	ENSP00000333537
ENSP00000301764	ENSP00000333667
ENSP00000301764	ENSP00000335321
ENSP00000301764	ENSP00000336868
ENSP00000301764	ENSP00000337340
ENSP00000301764	ENSP00000337405
ENSP00000301764	ENSP00000337797
ENSP00000301764	ENSP00000338413
ENSP00000301764	ENSP00000339090
ENSP00000301764	ENSP00000341351
ENSP00000301764	ENSP00000342262
ENSP00000301764	ENSP00000342374
ENSP00000301764	ENSP00000343741
ENSP00000301764	ENSP00000343745
ENSP00000301764	ENSP00000344456
ENSP00000301764	ENSP00000344666
ENSP00000301764	ENSP00000345096
ENSP00000301764	ENSP00000346340
ENSP00000301764	ENSP00000347168

ENSP00000301764	ENSP00000347978
ENSP00000301764	ENSP00000348010
ENSP00000301764	ENSP00000348089
ENSP00000301764	ENSP00000350512
ENSP00000301764	ENSP00000350767
ENSP00000301764	ENSP00000351100
ENSP00000301764	ENSP00000351137
ENSP00000301764	ENSP00000351697
ENSP00000301764	ENSP00000352121
ENSP00000301764	ENSP00000352257
ENSP00000301764	ENSP00000352516
ENSP00000301764	ENSP00000352928
ENSP00000301764	ENSP00000354501
ENSP00000301764	ENSP00000354558
ENSP00000301764	ENSP00000354723
ENSP00000301764	ENSP00000355205
ENSP00000301764	ENSP00000355317
ENSP00000301764	ENSP00000355388
ENSP00000301764	ENSP00000355958
ENSP00000301764	ENSP00000356541
ENSP00000301764	ENSP00000356641
ENSP00000301764	ENSP00000356817
ENSP00000301764	ENSP00000357048
ENSP00000301764	ENSP00000357631
ENSP00000301764	ENSP00000357651
ENSP00000301764	ENSP00000358417
ENSP00000301764	ENSP00000359206
ENSP00000301764	ENSP00000360515
ENSP00000301764	ENSP00000360532
ENSP00000301764	ENSP00000360817
ENSP00000301764	ENSP00000362110
ENSP00000301764	ENSP00000362592
ENSP00000301764	ENSP00000362688
ENSP00000301764	ENSP00000362936
ENSP00000301764	ENSP00000363313
ENSP00000301764	ENSP00000364289
ENSP00000301764	ENSP00000364404
ENSP00000301764	ENSP00000365877
ENSP00000301764	ENSP00000366271
ENSP00000301764	ENSP00000366557
ENSP00000301764	ENSP00000366953
ENSP00000301764	ENSP00000366974
ENSP00000301764	ENSP00000367034
ENSP00000301764	ENSP00000368438
ENSP00000301764	ENSP00000368887
ENSP00000301764	ENSP00000371682

ENSP00000301764	ENSP00000372023
ENSP00000301764	ENSP00000373354
ENSP00000301764	ENSP00000379607
ENSP00000301764	ENSP00000380718
ENSP00000301764	ENSP00000384109
ENSP00000301764	ENSP00000387006
ENSP00000301764	ENSP00000397435
ENSP00000301764	ENSP00000400591
ENSP00000301764	ENSP00000408994
ENSP00000301764	ENSP00000415434
ENSP00000301764	ENSP00000417980
ENSP00000301765	ENSP00000307387
ENSP00000301765	ENSP00000310440
ENSP00000301765	ENSP00000318629
ENSP00000301765	ENSP00000331201
ENSP00000301765	ENSP00000356903
ENSP00000301765	ENSP00000360540
ENSP00000301765	ENSP00000366565
ENSP00000301788	ENSP00000306906
ENSP00000301788	ENSP00000306983
ENSP00000301788	ENSP00000307491
ENSP00000301788	ENSP00000307850
ENSP00000301788	ENSP00000310260
ENSP00000301788	ENSP00000312735
ENSP00000301788	ENSP00000314949
ENSP00000301788	ENSP00000317636
ENSP00000301788	ENSP00000318641
ENSP00000301788	ENSP00000322608
ENSP00000301788	ENSP00000323720
ENSP00000301788	ENSP00000323858
ENSP00000301788	ENSP00000324124
ENSP00000301788	ENSP00000326767
ENSP00000301788	ENSP00000327889
ENSP00000301788	ENSP00000329029
ENSP00000301788	ENSP00000331111
ENSP00000301788	ENSP00000333376
ENSP00000301788	ENSP00000337340
ENSP00000301788	ENSP00000340823
ENSP00000301788	ENSP00000342889
ENSP00000301788	ENSP00000345530
ENSP00000301788	ENSP00000347883
ENSP00000301788	ENSP00000354720
ENSP00000301788	ENSP00000356146
ENSP00000301788	ENSP00000358081
ENSP00000301788	ENSP00000358497
ENSP00000301788	ENSP00000359290

ENSP00000301788	ENSP00000359531
ENSP00000301788	ENSP00000364320
ENSP00000301788	ENSP00000364482
ENSP00000301788	ENSP00000364813
ENSP00000301788	ENSP00000366828
ENSP00000301788	ENSP00000369695
ENSP00000301788	ENSP00000370258
ENSP00000301788	ENSP00000372817
ENSP00000301788	ENSP00000372883
ENSP00000301788	ENSP00000377969
ENSP00000301788	ENSP00000379228
ENSP00000301788	ENSP00000381031
ENSP00000301788	ENSP00000383503
ENSP00000301788	ENSP00000386759
ENSP00000301788	ENSP00000391798
ENSP00000301788	ENSP00000397636
ENSP00000301788	ENSP00000403345
ENSP00000301788	ENSP00000403852
ENSP00000301788	ENSP00000405636
ENSP00000301788	ENSP00000406280
ENSP00000301788	ENSP00000406293
ENSP00000301788	ENSP00000410530
ENSP00000301788	ENSP00000410954
ENSP00000301788	ENSP00000414006
ENSP00000301788	ENSP00000414110
ENSP00000301788	ENSP00000414634
ENSP00000301788	ENSP00000416599
ENSP00000301825	ENSP00000352522
ENSP00000301825	ENSP00000365840
ENSP00000301831	ENSP00000332353
ENSP00000301831	ENSP00000334061
ENSP00000301831	ENSP00000340954
ENSP00000301838	ENSP00000304226
ENSP00000301838	ENSP00000313829
ENSP00000301838	ENSP00000318822
ENSP00000301838	ENSP00000326340
ENSP00000301838	ENSP00000329411
ENSP00000301838	ENSP00000337797
ENSP00000301838	ENSP00000340278
ENSP00000301838	ENSP00000341268
ENSP00000301838	ENSP00000341692
ENSP00000301838	ENSP00000343313
ENSP00000301838	ENSP00000347979
ENSP00000301838	ENSP00000350785
ENSP00000301838	ENSP00000353538
ENSP00000301838	ENSP00000353660

ENSP00000301838	ENSP00000356694
ENSP00000301838	ENSP00000358997
ENSP00000301838	ENSP00000361120
ENSP00000301838	ENSP00000379457
ENSP00000301838	ENSP00000394794
ENSP00000301838	ENSP00000401980
ENSP00000301838	ENSP00000403817
ENSP00000301838	ENSP00000419494
ENSP00000301843	ENSP00000307134
ENSP00000301843	ENSP00000314499
ENSP00000301843	ENSP00000316460
ENSP00000301843	ENSP00000322804
ENSP00000301843	ENSP00000326238
ENSP00000301843	ENSP00000329002
ENSP00000301843	ENSP00000334061
ENSP00000301843	ENSP00000334836
ENSP00000301843	ENSP00000341189
ENSP00000301843	ENSP00000344468
ENSP00000301843	ENSP00000348128
ENSP00000301843	ENSP00000348163
ENSP00000301843	ENSP00000348215
ENSP00000301843	ENSP00000348602
ENSP00000301843	ENSP00000349016
ENSP00000301843	ENSP00000350297
ENSP00000301843	ENSP00000352802
ENSP00000301843	ENSP00000353452
ENSP00000301843	ENSP00000359206
ENSP00000301843	ENSP00000359225
ENSP00000301843	ENSP00000360798
ENSP00000301843	ENSP00000364277
ENSP00000301843	ENSP00000364898
ENSP00000301843	ENSP00000366603
ENSP00000301843	ENSP00000374424
ENSP00000301843	ENSP00000377496
ENSP00000301843	ENSP00000379607
ENSP00000301843	ENSP00000380431
ENSP00000301843	ENSP00000386456
ENSP00000301843	ENSP00000401277
ENSP00000301887	ENSP00000303315
ENSP00000301887	ENSP00000345107
ENSP00000301887	ENSP00000358854
ENSP00000301920	ENSP00000355536
ENSP00000301945	ENSP00000350283
ENSP00000301962	ENSP00000303191
ENSP00000301962	ENSP00000315397
ENSP00000301962	ENSP00000321449

ENSP00000301962	ENSP00000339795
ENSP00000301962	ENSP00000358563
ENSP00000301962	ENSP00000359000
ENSP00000301962	ENSP00000361027
ENSP00000301962	ENSP00000363642
ENSP00000301962	ENSP00000363746
ENSP00000301962	ENSP00000364037
ENSP00000301962	ENSP00000367029
ENSP00000301962	ENSP00000367498
ENSP00000301962	ENSP00000368438
ENSP00000301962	ENSP00000369682
ENSP00000301962	ENSP00000372326
ENSP00000301962	ENSP00000372695
ENSP00000301962	ENSP00000380460
ENSP00000301962	ENSP00000380531
ENSP00000301962	ENSP00000384211
ENSP00000301962	ENSP00000399454
ENSP00000301962	ENSP00000402869
ENSP00000301962	ENSP00000405614
ENSP00000301962	ENSP00000417281
ENSP00000301962	ENSP00000420714
ENSP00000301972	ENSP00000339191
ENSP00000302021	ENSP00000359100
ENSP00000302079	ENSP00000360916
ENSP00000302079	ENSP00000396688
ENSP00000302108	ENSP00000340879
ENSP00000302111	ENSP00000305230
ENSP00000302111	ENSP00000313420
ENSP00000302111	ENSP00000318195
ENSP00000302111	ENSP00000318646
ENSP00000302111	ENSP00000318977
ENSP00000302111	ENSP00000328111
ENSP00000302111	ENSP00000328690
ENSP00000302111	ENSP00000334675
ENSP00000302111	ENSP00000338413
ENSP00000302111	ENSP00000346037
ENSP00000302111	ENSP00000346240
ENSP00000302111	ENSP00000347978
ENSP00000302111	ENSP00000348089
ENSP00000302111	ENSP00000350708
ENSP00000302111	ENSP00000352257
ENSP00000302111	ENSP00000354522
ENSP00000302111	ENSP00000360621
ENSP00000302111	ENSP00000361990
ENSP00000302111	ENSP00000362578
ENSP00000302111	ENSP00000363313

ENSP00000302111	ENSP00000364270
ENSP00000302111	ENSP00000368438
ENSP00000302111	ENSP00000369325
ENSP00000302111	ENSP00000369497
ENSP00000302111	ENSP00000371475
ENSP00000302111	ENSP00000372023
ENSP00000302111	ENSP00000381045
ENSP00000302111	ENSP00000381863
ENSP00000302111	ENSP00000386161
ENSP00000302111	ENSP00000396068
ENSP00000302114	ENSP00000319323
ENSP00000302114	ENSP00000320309
ENSP00000302114	ENSP00000325448
ENSP00000302114	ENSP00000335675
ENSP00000302114	ENSP00000348886
ENSP00000302114	ENSP00000356329
ENSP00000302114	ENSP00000356587
ENSP00000302114	ENSP00000362298
ENSP00000302114	ENSP00000367498
ENSP00000302114	ENSP00000368140
ENSP00000302114	ENSP00000369860
ENSP00000302120	ENSP00000306906
ENSP00000302120	ENSP00000311827
ENSP00000302120	ENSP00000313881
ENSP00000302120	ENSP00000321239
ENSP00000302120	ENSP00000340305
ENSP00000302120	ENSP00000342755
ENSP00000302120	ENSP00000343535
ENSP00000302120	ENSP00000349783
ENSP00000302120	ENSP00000360497
ENSP00000302120	ENSP00000370719
ENSP00000302139	ENSP00000313059
ENSP00000302139	ENSP00000380921
ENSP00000302160	ENSP00000304350
ENSP00000302160	ENSP00000304593
ENSP00000302160	ENSP00000304903
ENSP00000302160	ENSP00000305790
ENSP00000302160	ENSP00000307387
ENSP00000302160	ENSP00000308546
ENSP00000302160	ENSP00000310448
ENSP00000302160	ENSP00000310596
ENSP00000302160	ENSP00000311430
ENSP00000302160	ENSP00000314311
ENSP00000302160	ENSP00000315035
ENSP00000302160	ENSP00000315379
ENSP00000302160	ENSP00000318861

ENSP00000302160	ENSP00000319341
ENSP00000302160	ENSP00000320503
ENSP00000302160	ENSP00000320949
ENSP00000302160	ENSP00000323302
ENSP00000302160	ENSP00000324122
ENSP00000302160	ENSP00000325448
ENSP00000302160	ENSP00000330836
ENSP00000302160	ENSP00000332103
ENSP00000302160	ENSP00000334188
ENSP00000302160	ENSP00000335321
ENSP00000302160	ENSP00000337405
ENSP00000302160	ENSP00000339090
ENSP00000302160	ENSP00000339095
ENSP00000302160	ENSP00000339503
ENSP00000302160	ENSP00000342374
ENSP00000302160	ENSP00000343081
ENSP00000302160	ENSP00000343742
ENSP00000302160	ENSP00000348010
ENSP00000302160	ENSP00000348107
ENSP00000302160	ENSP00000348722
ENSP00000302160	ENSP00000354673
ENSP00000302160	ENSP00000355205
ENSP00000302160	ENSP00000359454
ENSP00000302160	ENSP00000361577
ENSP00000302160	ENSP00000362110
ENSP00000302160	ENSP00000362413
ENSP00000302160	ENSP00000363313
ENSP00000302160	ENSP00000364320
ENSP00000302160	ENSP00000364649
ENSP00000302160	ENSP00000364813
ENSP00000302160	ENSP00000366135
ENSP00000302160	ENSP00000366557
ENSP00000302160	ENSP00000370119
ENSP00000302160	ENSP00000370589
ENSP00000302160	ENSP00000371546
ENSP00000302160	ENSP00000373715
ENSP00000302160	ENSP00000374213
ENSP00000302160	ENSP00000374529
ENSP00000302160	ENSP00000376973
ENSP00000302160	ENSP00000379607
ENSP00000302160	ENSP00000380251
ENSP00000302160	ENSP00000380969
ENSP00000302160	ENSP00000382779
ENSP00000302160	ENSP00000391723
ENSP00000302160	ENSP00000400591
ENSP00000302160	ENSP00000406293

ENSP00000302160	ENSP00000410758
ENSP00000302160	ENSP00000412566
ENSP00000302160	ENSP00000414006
ENSP00000302160	ENSP00000414634
ENSP00000302160	ENSP00000419628
ENSP00000302176	ENSP00000309031
ENSP00000302176	ENSP00000321617
ENSP00000302176	ENSP00000331487
ENSP00000302176	ENSP00000337688
ENSP00000302176	ENSP00000375881
ENSP00000302176	ENSP00000379457
ENSP00000302176	ENSP00000380460
ENSP00000302177	ENSP00000310551
ENSP00000302177	ENSP00000314036
ENSP00000302177	ENSP00000337463
ENSP00000302177	ENSP00000346228
ENSP00000302177	ENSP00000355963
ENSP00000302177	ENSP00000380184
ENSP00000302177	ENSP00000382723
ENSP00000302177	ENSP00000389516
ENSP00000302177	ENSP00000395863
ENSP00000302177	ENSP00000401287
ENSP00000302177	ENSP00000405864
ENSP00000302177	ENSP00000412805
ENSP00000302216	ENSP00000326391
ENSP00000302216	ENSP00000331057
ENSP00000302216	ENSP00000332353
ENSP00000302216	ENSP00000352514
ENSP00000302216	ENSP00000367972
ENSP00000302216	ENSP00000372170
ENSP00000302239	ENSP00000307387
ENSP00000302239	ENSP00000309595
ENSP00000302239	ENSP00000310440
ENSP00000302239	ENSP00000317468
ENSP00000302239	ENSP00000324491
ENSP00000302239	ENSP00000326921
ENSP00000302239	ENSP00000331201
ENSP00000302239	ENSP00000331487
ENSP00000302239	ENSP00000334061
ENSP00000302239	ENSP00000337353
ENSP00000302239	ENSP00000339186
ENSP00000302239	ENSP00000341679
ENSP00000302239	ENSP00000342026
ENSP00000302239	ENSP00000342755
ENSP00000302239	ENSP00000343479
ENSP00000302239	ENSP00000343535

ENSP00000302239	ENSP00000345530
ENSP00000302239	ENSP00000347498
ENSP00000302239	ENSP00000348442
ENSP00000302239	ENSP00000354522
ENSP00000302239	ENSP00000354673
ENSP00000302239	ENSP00000357047
ENSP00000302239	ENSP00000360798
ENSP00000302239	ENSP00000360916
ENSP00000302239	ENSP00000366746
ENSP00000302239	ENSP00000393596
ENSP00000302239	ENSP00000405963
ENSP00000302239	ENSP00000417686
ENSP00000302327	ENSP00000318374
ENSP00000302327	ENSP00000344456
ENSP00000302327	ENSP00000363193
ENSP00000302478	ENSP00000303088
ENSP00000302478	ENSP00000306983
ENSP00000302478	ENSP00000310966
ENSP00000302478	ENSP00000314949
ENSP00000302478	ENSP00000324124
ENSP00000302478	ENSP00000327070
ENSP00000302478	ENSP00000329002
ENSP00000302478	ENSP00000331111
ENSP00000302478	ENSP00000332171
ENSP00000302478	ENSP00000333837
ENSP00000302478	ENSP00000333920
ENSP00000302478	ENSP00000334564
ENSP00000302478	ENSP00000340671
ENSP00000302478	ENSP00000341243
ENSP00000302478	ENSP00000347345
ENSP00000302478	ENSP00000361465
ENSP00000302478	ENSP00000366828
ENSP00000302478	ENSP00000367029
ENSP00000302478	ENSP00000368686
ENSP00000302478	ENSP00000369695
ENSP00000302478	ENSP00000378736
ENSP00000302478	ENSP00000383503
ENSP00000302478	ENSP00000397636
ENSP00000302478	ENSP00000403852
ENSP00000302478	ENSP00000405636
ENSP00000302478	ENSP00000410530
ENSP00000302478	ENSP00000410954
ENSP00000302478	ENSP00000414110
ENSP00000302478	ENSP00000416599
ENSP00000302501	ENSP00000311825
ENSP00000302501	ENSP00000312385

ENSP00000302501	ENSP00000333633
ENSP00000302501	ENSP00000341551
ENSP00000302501	ENSP00000342828
ENSP00000302501	ENSP00000362592
ENSP00000302501	ENSP00000414187
ENSP00000302530	ENSP00000304593
ENSP00000302530	ENSP00000311873
ENSP00000302530	ENSP00000313171
ENSP00000302530	ENSP00000313243
ENSP00000302530	ENSP00000313420
ENSP00000302530	ENSP00000315791
ENSP00000302530	ENSP00000318977
ENSP00000302530	ENSP00000322180
ENSP00000302530	ENSP00000323439
ENSP00000302530	ENSP00000324729
ENSP00000302530	ENSP00000325377
ENSP00000302530	ENSP00000325863
ENSP00000302530	ENSP00000329933
ENSP00000302530	ENSP00000330408
ENSP00000302530	ENSP00000331310
ENSP00000302530	ENSP00000334188
ENSP00000302530	ENSP00000335463
ENSP00000302530	ENSP00000336842
ENSP00000302530	ENSP00000341382
ENSP00000302530	ENSP00000343526
ENSP00000302530	ENSP00000343535
ENSP00000302530	ENSP00000343741
ENSP00000302530	ENSP00000346566
ENSP00000302530	ENSP00000347076
ENSP00000302530	ENSP00000347168
ENSP00000302530	ENSP00000347719
ENSP00000302530	ENSP00000347978
ENSP00000302530	ENSP00000348010
ENSP00000302530	ENSP00000348128
ENSP00000302530	ENSP00000350199
ENSP00000302530	ENSP00000350767
ENSP00000302530	ENSP00000351284
ENSP00000302530	ENSP00000351665
ENSP00000302530	ENSP00000352121
ENSP00000302530	ENSP00000352572
ENSP00000302530	ENSP00000353332
ENSP00000302530	ENSP00000353793
ENSP00000302530	ENSP00000354522
ENSP00000302530	ENSP00000354558
ENSP00000302530	ENSP00000354720
ENSP00000302530	ENSP00000354957

ENSP00000302530	ENSP00000355205
ENSP00000302530	ENSP00000355388
ENSP00000302530	ENSP00000355560
ENSP00000302530	ENSP00000355922
ENSP00000302530	ENSP00000355944
ENSP00000302530	ENSP00000356319
ENSP00000302530	ENSP00000356989
ENSP00000302530	ENSP00000357260
ENSP00000302530	ENSP00000357440
ENSP00000302530	ENSP00000357651
ENSP00000302530	ENSP00000357887
ENSP00000302530	ENSP00000357907
ENSP00000302530	ENSP00000359518
ENSP00000302530	ENSP00000360286
ENSP00000302530	ENSP00000360473
ENSP00000302530	ENSP00000360532
ENSP00000302530	ENSP00000360621
ENSP00000302530	ENSP00000363055
ENSP00000302530	ENSP00000363779
ENSP00000302530	ENSP00000364119
ENSP00000302530	ENSP00000365025
ENSP00000302530	ENSP00000366974
ENSP00000302530	ENSP00000367034
ENSP00000302530	ENSP00000368438
ENSP00000302530	ENSP00000368976
ENSP00000302530	ENSP00000369411
ENSP00000302530	ENSP00000370151
ENSP00000302530	ENSP00000370557
ENSP00000302530	ENSP00000370589
ENSP00000302530	ENSP00000374213
ENSP00000302530	ENSP00000374566
ENSP00000302530	ENSP00000375809
ENSP00000302530	ENSP00000378165
ENSP00000302530	ENSP00000381932
ENSP00000302530	ENSP00000382177
ENSP00000302530	ENSP00000382271
ENSP00000302530	ENSP00000382562
ENSP00000302530	ENSP00000386759
ENSP00000302530	ENSP00000402084
ENSP00000302530	ENSP00000408910
ENSP00000302579	ENSP00000334003
ENSP00000302579	ENSP00000366121
ENSP00000302620	ENSP00000339435
ENSP00000302620	ENSP00000345282
ENSP00000302620	ENSP00000348234
ENSP00000302620	ENSP00000365534

ENSP00000302620	ENSP00000365773
ENSP00000302620	ENSP00000368066
ENSP00000302620	ENSP00000368109
ENSP00000302620	ENSP00000407401
ENSP00000302640	ENSP00000310966
ENSP00000302640	ENSP00000314971
ENSP00000302640	ENSP00000319104
ENSP00000302640	ENSP00000321636
ENSP00000302640	ENSP00000327072
ENSP00000302640	ENSP00000329029
ENSP00000302640	ENSP00000329466
ENSP00000302640	ENSP00000336842
ENSP00000302640	ENSP00000337340
ENSP00000302640	ENSP00000349640
ENSP00000302640	ENSP00000350894
ENSP00000302640	ENSP00000352257
ENSP00000302640	ENSP00000352514
ENSP00000302640	ENSP00000353622
ENSP00000302640	ENSP00000359531
ENSP00000302640	ENSP00000365016
ENSP00000302640	ENSP00000367029
ENSP00000302640	ENSP00000369757
ENSP00000302640	ENSP00000370968
ENSP00000302640	ENSP00000382562
ENSP00000302640	ENSP00000398930
ENSP00000302640	ENSP00000401371
ENSP00000302640	ENSP00000406888
ENSP00000302648	ENSP00000347942
ENSP00000302648	ENSP00000393725
ENSP00000302665	ENSP00000355010
ENSP00000302665	ENSP00000387170
ENSP00000302768	ENSP00000307863
ENSP00000302768	ENSP00000312753
ENSP00000302768	ENSP00000315859
ENSP00000302768	ENSP00000317872
ENSP00000302768	ENSP00000318646
ENSP00000302768	ENSP00000322570
ENSP00000302768	ENSP00000323858
ENSP00000302768	ENSP00000329715
ENSP00000302768	ENSP00000331817
ENSP00000302768	ENSP00000338862
ENSP00000302768	ENSP00000343081
ENSP00000302768	ENSP00000346453
ENSP00000302768	ENSP00000348722
ENSP00000302768	ENSP00000354522
ENSP00000302768	ENSP00000355890

ENSP00000302768	ENSP00000356146
ENSP00000302768	ENSP00000358563
ENSP00000302768	ENSP00000359688
ENSP00000302768	ENSP00000359837
ENSP00000302768	ENSP00000362900
ENSP00000302768	ENSP00000371546
ENSP00000302768	ENSP00000382779
ENSP00000302768	ENSP00000402338
ENSP00000302783	ENSP00000310521
ENSP00000302783	ENSP00000311134
ENSP00000302783	ENSP00000316990
ENSP00000302783	ENSP00000326869
ENSP00000302783	ENSP00000336724
ENSP00000302783	ENSP00000352708
ENSP00000302783	ENSP00000356231
ENSP00000302783	ENSP00000373979
ENSP00000302783	ENSP00000397441
ENSP00000302783	ENSP00000402935
ENSP00000302790	ENSP00000302886
ENSP00000302790	ENSP00000305503
ENSP00000302790	ENSP00000310668
ENSP00000302790	ENSP00000314004
ENSP00000302790	ENSP00000315791
ENSP00000302790	ENSP00000316032
ENSP00000302790	ENSP00000318604
ENSP00000302790	ENSP00000346453
ENSP00000302790	ENSP00000348577
ENSP00000302790	ENSP00000354720
ENSP00000302790	ENSP00000354742
ENSP00000302790	ENSP00000355809
ENSP00000302790	ENSP00000355890
ENSP00000302790	ENSP00000356405
ENSP00000302790	ENSP00000356548
ENSP00000302790	ENSP00000356744
ENSP00000302790	ENSP00000357907
ENSP00000302790	ENSP00000359000
ENSP00000302790	ENSP00000361186
ENSP00000302790	ENSP00000361287
ENSP00000302790	ENSP00000371155
ENSP00000302790	ENSP00000373715
ENSP00000302790	ENSP00000381565
ENSP00000302790	ENSP00000381567
ENSP00000302801	ENSP00000304668
ENSP00000302801	ENSP00000307077
ENSP00000302801	ENSP00000314311
ENSP00000302801	ENSP00000321711

ENSP00000302801	ENSP00000325448
ENSP00000302801	ENSP00000327821
ENSP00000302801	ENSP00000329411
ENSP00000302801	ENSP00000332171
ENSP00000302801	ENSP00000363779
ENSP00000302801	ENSP00000364320
ENSP00000302801	ENSP00000367498
ENSP00000302801	ENSP00000380460
ENSP00000302801	ENSP00000394869
ENSP00000302830	ENSP00000320623
ENSP00000302830	ENSP00000344566
ENSP00000302830	ENSP00000351221
ENSP00000302886	ENSP00000305503
ENSP00000302886	ENSP00000307525
ENSP00000302886	ENSP00000307889
ENSP00000302886	ENSP00000307939
ENSP00000302886	ENSP00000309334
ENSP00000302886	ENSP00000310042
ENSP00000302886	ENSP00000310668
ENSP00000302886	ENSP00000311430
ENSP00000302886	ENSP00000312981
ENSP00000302886	ENSP00000316032
ENSP00000302886	ENSP00000317992
ENSP00000302886	ENSP00000318195
ENSP00000302886	ENSP00000322396
ENSP00000302886	ENSP00000322419
ENSP00000302886	ENSP00000323858
ENSP00000302886	ENSP00000328690
ENSP00000302886	ENSP00000333667
ENSP00000302886	ENSP00000338573
ENSP00000302886	ENSP00000338862
ENSP00000302886	ENSP00000338990
ENSP00000302886	ENSP00000339145
ENSP00000302886	ENSP00000339795
ENSP00000302886	ENSP00000341551
ENSP00000302886	ENSP00000343313
ENSP00000302886	ENSP00000343741
ENSP00000302886	ENSP00000344220
ENSP00000302886	ENSP00000345156
ENSP00000302886	ENSP00000346015
ENSP00000302886	ENSP00000346022
ENSP00000302886	ENSP00000346027
ENSP00000302886	ENSP00000346453
ENSP00000302886	ENSP00000346725
ENSP00000302886	ENSP00000347719
ENSP00000302886	ENSP00000348565

ENSP00000302886	ENSP00000348596
ENSP00000302886	ENSP00000350967
ENSP00000302886	ENSP00000351380
ENSP00000302886	ENSP00000352021
ENSP00000302886	ENSP00000352400
ENSP00000302886	ENSP00000352645
ENSP00000302886	ENSP00000352928
ENSP00000302886	ENSP00000353622
ENSP00000302886	ENSP00000354040
ENSP00000302886	ENSP00000354522
ENSP00000302886	ENSP00000354558
ENSP00000302886	ENSP00000354739
ENSP00000302886	ENSP00000358400
ENSP00000302886	ENSP00000359345
ENSP00000302886	ENSP00000359688
ENSP00000302886	ENSP00000360286
ENSP00000302886	ENSP00000360412
ENSP00000302886	ENSP00000360828
ENSP00000302886	ENSP00000361076
ENSP00000302886	ENSP00000361640
ENSP00000302886	ENSP00000362153
ENSP00000302886	ENSP00000362900
ENSP00000302886	ENSP00000363018
ENSP00000302886	ENSP00000363373
ENSP00000302886	ENSP00000363384
ENSP00000302886	ENSP00000363559
ENSP00000302886	ENSP00000363676
ENSP00000302886	ENSP00000364037
ENSP00000302886	ENSP00000364320
ENSP00000302886	ENSP00000365806
ENSP00000302886	ENSP00000366519
ENSP00000302886	ENSP00000368612
ENSP00000302886	ENSP00000369042
ENSP00000302886	ENSP00000370376
ENSP00000302886	ENSP00000370968
ENSP00000302886	ENSP00000371155
ENSP00000302886	ENSP00000371451
ENSP00000302886	ENSP00000372199
ENSP00000302886	ENSP00000373090
ENSP00000302886	ENSP00000377640
ENSP00000302886	ENSP00000377954
ENSP00000302886	ENSP00000378160
ENSP00000302886	ENSP00000379888
ENSP00000302886	ENSP00000380982
ENSP00000302886	ENSP00000382392
ENSP00000302886	ENSP00000386717

ENSP00000302886	ENSP00000389103
ENSP00000302886	ENSP00000393393
ENSP00000302886	ENSP00000394290
ENSP00000302886	ENSP00000402338
ENSP00000302886	ENSP00000403576
ENSP00000302886	ENSP00000409074
ENSP00000302886	ENSP00000409367
ENSP00000302886	ENSP00000411162
ENSP00000302886	ENSP00000417281
ENSP00000302886	ENSP00000419740
ENSP00000302896	ENSP00000306548
ENSP00000302896	ENSP00000308546
ENSP00000302896	ENSP00000308897
ENSP00000302896	ENSP00000309334
ENSP00000302896	ENSP00000311028
ENSP00000302896	ENSP00000311430
ENSP00000302896	ENSP00000314311
ENSP00000302896	ENSP00000315674
ENSP00000302896	ENSP00000317376
ENSP00000302896	ENSP00000318646
ENSP00000302896	ENSP00000319977
ENSP00000302896	ENSP00000320917
ENSP00000302896	ENSP00000322419
ENSP00000302896	ENSP00000323856
ENSP00000302896	ENSP00000325376
ENSP00000302896	ENSP00000328854
ENSP00000302896	ENSP00000331019
ENSP00000302896	ENSP00000336702
ENSP00000302896	ENSP00000336712
ENSP00000302896	ENSP00000337127
ENSP00000302896	ENSP00000339095
ENSP00000302896	ENSP00000341885
ENSP00000302896	ENSP00000345096
ENSP00000302896	ENSP00000345156
ENSP00000302896	ENSP00000346015
ENSP00000302896	ENSP00000346022
ENSP00000302896	ENSP00000346037
ENSP00000302896	ENSP00000346050
ENSP00000302896	ENSP00000346067
ENSP00000302896	ENSP00000347087
ENSP00000302896	ENSP00000352738
ENSP00000302896	ENSP00000354739
ENSP00000302896	ENSP00000355471
ENSP00000302896	ENSP00000355541
ENSP00000302896	ENSP00000356130
ENSP00000302896	ENSP00000356134

ENSP00000302896	ENSP00000358140
ENSP00000302896	ENSP00000359345
ENSP00000302896	ENSP00000359688
ENSP00000302896	ENSP00000360031
ENSP00000302896	ENSP00000361076
ENSP00000302896	ENSP00000362036
ENSP00000302896	ENSP00000362306
ENSP00000302896	ENSP00000362688
ENSP00000302896	ENSP00000362744
ENSP00000302896	ENSP00000363018
ENSP00000302896	ENSP00000363308
ENSP00000302896	ENSP00000363642
ENSP00000302896	ENSP00000363676
ENSP00000302896	ENSP00000363746
ENSP00000302896	ENSP00000364320
ENSP00000302896	ENSP00000365837
ENSP00000302896	ENSP00000368565
ENSP00000302896	ENSP00000368790
ENSP00000302896	ENSP00000369757
ENSP00000302896	ENSP00000370589
ENSP00000302896	ENSP00000371101
ENSP00000302896	ENSP00000372445
ENSP00000302896	ENSP00000372695
ENSP00000302896	ENSP00000375730
ENSP00000302896	ENSP00000377486
ENSP00000302896	ENSP00000377640
ENSP00000302896	ENSP00000379339
ENSP00000302896	ENSP00000379607
ENSP00000302896	ENSP00000379888
ENSP00000302896	ENSP00000384302
ENSP00000302896	ENSP00000389103
ENSP00000302896	ENSP00000392859
ENSP00000302896	ENSP00000393241
ENSP00000302896	ENSP00000399454
ENSP00000302896	ENSP00000402338
ENSP00000302896	ENSP00000402869
ENSP00000302896	ENSP00000403175
ENSP00000302896	ENSP00000405614
ENSP00000302896	ENSP00000412583
ENSP00000302896	ENSP00000414321
ENSP00000302896	ENSP00000416110
ENSP00000302896	ENSP00000420714
ENSP00000302913	ENSP00000309968
ENSP00000302913	ENSP00000313691
ENSP00000302913	ENSP00000360798
ENSP00000302913	ENSP00000362727

ENSP00000302913	ENSP00000365401
ENSP00000302913	ENSP00000380921
ENSP00000302935	ENSP00000312318
ENSP00000302935	ENSP00000360500
ENSP00000302951	ENSP00000314733
ENSP00000302951	ENSP00000353622
ENSP00000302951	ENSP00000367910
ENSP00000302951	ENSP00000373952
ENSP00000302951	ENSP00000385021
ENSP00000302955	ENSP00000304370
ENSP00000302955	ENSP00000307853
ENSP00000302955	ENSP00000310520
ENSP00000302955	ENSP00000315700
ENSP00000302955	ENSP00000320648
ENSP00000302955	ENSP00000322180
ENSP00000302955	ENSP00000322570
ENSP00000302955	ENSP00000330836
ENSP00000302955	ENSP00000340879
ENSP00000302955	ENSP00000341382
ENSP00000302955	ENSP00000343701
ENSP00000302955	ENSP00000343741
ENSP00000302955	ENSP00000351697
ENSP00000302955	ENSP00000354522
ENSP00000302955	ENSP00000354720
ENSP00000302955	ENSP00000355958
ENSP00000302955	ENSP00000360031
ENSP00000302955	ENSP00000361418
ENSP00000302955	ENSP00000365534
ENSP00000302955	ENSP00000368349
ENSP00000302955	ENSP00000370151
ENSP00000302955	ENSP00000372023
ENSP00000302955	ENSP00000377914
ENSP00000302955	ENSP00000378529
ENSP00000302955	ENSP00000380256
ENSP00000303015	ENSP00000307863
ENSP00000303015	ENSP00000312066
ENSP00000303015	ENSP00000362820
ENSP00000303028	ENSP00000353701
ENSP00000303042	ENSP00000350249
ENSP00000303042	ENSP00000377496
ENSP00000303088	ENSP00000314949
ENSP00000303088	ENSP00000321711
ENSP00000303088	ENSP00000324124
ENSP00000303088	ENSP00000334564
ENSP00000303088	ENSP00000341243
ENSP00000303088	ENSP00000344352

ENSP00000303088	ENSP00000347345
ENSP00000303088	ENSP00000360532
ENSP00000303088	ENSP00000361465
ENSP00000303088	ENSP00000366828
ENSP00000303088	ENSP00000378736
ENSP00000303088	ENSP00000382058
ENSP00000303088	ENSP00000403852
ENSP00000303148	ENSP00000347948
ENSP00000303191	ENSP00000303712
ENSP00000303191	ENSP00000304350
ENSP00000303191	ENSP00000304370
ENSP00000303191	ENSP00000305790
ENSP00000303191	ENSP00000308369
ENSP00000303191	ENSP00000314311
ENSP00000303191	ENSP00000314792
ENSP00000303191	ENSP00000318861
ENSP00000303191	ENSP00000325376
ENSP00000303191	ENSP00000326261
ENSP00000303191	ENSP00000326806
ENSP00000303191	ENSP00000331817
ENSP00000303191	ENSP00000331907
ENSP00000303191	ENSP00000332103
ENSP00000303191	ENSP00000334148
ENSP00000303191	ENSP00000335321
ENSP00000303191	ENSP00000337194
ENSP00000303191	ENSP00000342374
ENSP00000303191	ENSP00000343557
ENSP00000303191	ENSP00000343701
ENSP00000303191	ENSP00000346986
ENSP00000303191	ENSP00000351137
ENSP00000303191	ENSP00000353246
ENSP00000303191	ENSP00000354125
ENSP00000303191	ENSP00000355050
ENSP00000303191	ENSP00000357631
ENSP00000303191	ENSP00000358554
ENSP00000303191	ENSP00000360532
ENSP00000303191	ENSP00000361557
ENSP00000303191	ENSP00000361577
ENSP00000303191	ENSP00000362110
ENSP00000303191	ENSP00000362936
ENSP00000303191	ENSP00000364289
ENSP00000303191	ENSP00000366557
ENSP00000303191	ENSP00000370377
ENSP00000303191	ENSP00000376309
ENSP00000303191	ENSP00000376973
ENSP00000303191	ENSP00000379607

ENSP00000303191	ENSP00000384573
ENSP00000303191	ENSP00000387006
ENSP00000303191	ENSP00000391432
ENSP00000303191	ENSP00000412566
ENSP00000303191	ENSP00000416959
ENSP00000303191	ENSP00000417451
ENSP00000303191	ENSP00000420195
ENSP00000303208	ENSP00000307567
ENSP00000303208	ENSP00000312150
ENSP00000303208	ENSP00000336712
ENSP00000303208	ENSP00000337127
ENSP00000303208	ENSP00000365943
ENSP00000303231	ENSP00000314425
ENSP00000303231	ENSP00000352425
ENSP00000303231	ENSP00000357470
ENSP00000303248	ENSP00000315615
ENSP00000303279	ENSP00000311505
ENSP00000303279	ENSP00000320309
ENSP00000303279	ENSP00000329466
ENSP00000303279	ENSP00000333376
ENSP00000303279	ENSP00000344055
ENSP00000303279	ENSP00000348965
ENSP00000303279	ENSP00000349887
ENSP00000303279	ENSP00000350199
ENSP00000303279	ENSP00000355922
ENSP00000303279	ENSP00000361180
ENSP00000303279	ENSP00000362576
ENSP00000303279	ENSP00000367498
ENSP00000303279	ENSP00000382177
ENSP00000303279	ENSP00000382492
ENSP00000303315	ENSP00000306245
ENSP00000303315	ENSP00000306983
ENSP00000303315	ENSP00000307684
ENSP00000303315	ENSP00000308774
ENSP00000303315	ENSP00000314949
ENSP00000303315	ENSP00000322419
ENSP00000303315	ENSP00000327048
ENSP00000303315	ENSP00000335044
ENSP00000303315	ENSP00000336790
ENSP00000303315	ENSP00000340900
ENSP00000303315	ENSP00000341208
ENSP00000303315	ENSP00000341551
ENSP00000303315	ENSP00000344352
ENSP00000303315	ENSP00000344537
ENSP00000303315	ENSP00000345530
ENSP00000303315	ENSP00000347602

ENSP00000303315	ENSP00000347733
ENSP00000303315	ENSP00000347802
ENSP00000303315	ENSP00000350078
ENSP00000303315	ENSP00000350283
ENSP00000303315	ENSP00000353073
ENSP00000303315	ENSP00000353538
ENSP00000303315	ENSP00000353575
ENSP00000303315	ENSP00000353698
ENSP00000303315	ENSP00000355051
ENSP00000303315	ENSP00000355866
ENSP00000303315	ENSP00000356641
ENSP00000303315	ENSP00000357494
ENSP00000303315	ENSP00000357907
ENSP00000303315	ENSP00000358335
ENSP00000303315	ENSP00000358854
ENSP00000303315	ENSP00000359290
ENSP00000303315	ENSP00000359356
ENSP00000303315	ENSP00000360116
ENSP00000303315	ENSP00000360515
ENSP00000303315	ENSP00000364898
ENSP00000303315	ENSP00000369270
ENSP00000303315	ENSP00000369695
ENSP00000303315	ENSP00000373700
ENSP00000303315	ENSP00000376611
ENSP00000303315	ENSP00000382423
ENSP00000303315	ENSP00000393596
ENSP00000303315	ENSP00000399309
ENSP00000303315	ENSP00000399511
ENSP00000303315	ENSP00000413632
ENSP00000303325	ENSP00000321106
ENSP00000303325	ENSP00000334042
ENSP00000303325	ENSP00000355146
ENSP00000303366	ENSP00000360060
ENSP00000303366	ENSP00000379383
ENSP00000303394	ENSP00000337194
ENSP00000303394	ENSP00000360530
ENSP00000303398	ENSP00000417281
ENSP00000303423	ENSP00000315768
ENSP00000303423	ENSP00000334061
ENSP00000303423	ENSP00000339353
ENSP00000303423	ENSP00000355511
ENSP00000303423	ENSP00000355922
ENSP00000303423	ENSP00000360916
ENSP00000303423	ENSP00000366557
ENSP00000303423	ENSP00000375730
ENSP00000303423	ENSP00000394071

ENSP00000303434	ENSP00000310585
ENSP00000303434	ENSP00000321449
ENSP00000303434	ENSP00000332721
ENSP00000303434	ENSP00000339795
ENSP00000303434	ENSP00000350549
ENSP00000303434	ENSP00000360916
ENSP00000303434	ENSP00000365891
ENSP00000303434	ENSP00000380460
ENSP00000303452	ENSP00000368766
ENSP00000303476	ENSP00000360532
ENSP00000303482	ENSP00000343435
ENSP00000303507	ENSP00000307491
ENSP00000303507	ENSP00000317431
ENSP00000303507	ENSP00000318884
ENSP00000303507	ENSP00000320543
ENSP00000303507	ENSP00000322957
ENSP00000303507	ENSP00000333950
ENSP00000303507	ENSP00000339381
ENSP00000303507	ENSP00000340698
ENSP00000303507	ENSP00000340944
ENSP00000303507	ENSP00000345731
ENSP00000303507	ENSP00000349959
ENSP00000303507	ENSP00000352121
ENSP00000303507	ENSP00000354927
ENSP00000303507	ENSP00000356595
ENSP00000303507	ENSP00000360916
ENSP00000303507	ENSP00000365005
ENSP00000303507	ENSP00000366121
ENSP00000303507	ENSP00000366565
ENSP00000303507	ENSP00000373600
ENSP00000303507	ENSP00000376410
ENSP00000303507	ENSP00000381793
ENSP00000303507	ENSP00000382423
ENSP00000303507	ENSP00000383623
ENSP00000303507	ENSP00000384675
ENSP00000303507	ENSP00000391249
ENSP00000303507	ENSP00000397351
ENSP00000303511	ENSP00000309141
ENSP00000303511	ENSP00000311028
ENSP00000303511	ENSP00000317087
ENSP00000303511	ENSP00000317468
ENSP00000303511	ENSP00000331106
ENSP00000303511	ENSP00000346015
ENSP00000303511	ENSP00000349016
ENSP00000303511	ENSP00000360412
ENSP00000303511	ENSP00000366565

ENSP00000303511	ENSP00000407401
ENSP00000303515	ENSP00000314441
ENSP00000303515	ENSP00000328671
ENSP00000303515	ENSP00000356476
ENSP00000303515	ENSP00000361465
ENSP00000303515	ENSP00000364289
ENSP00000303515	ENSP00000365837
ENSP00000303515	ENSP00000366135
ENSP00000303515	ENSP00000370741
ENSP00000303515	ENSP00000403852
ENSP00000303552	ENSP00000325562
ENSP00000303552	ENSP00000340019
ENSP00000303552	ENSP00000343081
ENSP00000303552	ENSP00000348722
ENSP00000303552	ENSP00000387262
ENSP00000303554	ENSP00000334188
ENSP00000303554	ENSP00000342793
ENSP00000303554	ENSP00000344106
ENSP00000303554	ENSP00000346255
ENSP00000303554	ENSP00000359518
ENSP00000303554	ENSP00000360183
ENSP00000303554	ENSP00000360473
ENSP00000303554	ENSP00000368439
ENSP00000303554	ENSP00000381293
ENSP00000303554	ENSP00000387019
ENSP00000303570	ENSP00000350249
ENSP00000303570	ENSP00000377496
ENSP00000303584	ENSP00000309433
ENSP00000303584	ENSP00000353698
ENSP00000303584	ENSP00000353741
ENSP00000303584	ENSP00000359904
ENSP00000303584	ENSP00000367637
ENSP00000303584	ENSP00000368790
ENSP00000303584	ENSP00000374219
ENSP00000303712	ENSP00000335321
ENSP00000303712	ENSP00000362110
ENSP00000303712	ENSP00000362820
ENSP00000303712	ENSP00000366557
ENSP00000303712	ENSP00000376973
ENSP00000303712	ENSP00000416959
ENSP00000303769	ENSP00000304903
ENSP00000303769	ENSP00000387170
ENSP00000303920	ENSP00000312664
ENSP00000303928	ENSP00000313059
ENSP00000303939	ENSP00000319476
ENSP00000303939	ENSP00000332049

ENSP00000303939	ENSP00000340944
ENSP00000303939	ENSP00000341208
ENSP00000304051	ENSP00000304102
ENSP00000304051	ENSP00000310040
ENSP00000304051	ENSP00000344741
ENSP00000304051	ENSP00000347836
ENSP00000304051	ENSP00000350512
ENSP00000304051	ENSP00000354201
ENSP00000304051	ENSP00000358140
ENSP00000304051	ENSP00000360116
ENSP00000304051	ENSP00000360621
ENSP00000304051	ENSP00000384900
ENSP00000304102	ENSP00000304147
ENSP00000304102	ENSP00000304822
ENSP00000304102	ENSP00000307833
ENSP00000304102	ENSP00000309334
ENSP00000304102	ENSP00000311083
ENSP00000304102	ENSP00000317780
ENSP00000304102	ENSP00000322396
ENSP00000304102	ENSP00000323822
ENSP00000304102	ENSP00000325448
ENSP00000304102	ENSP00000337405
ENSP00000304102	ENSP00000341030
ENSP00000304102	ENSP00000341243
ENSP00000304102	ENSP00000343701
ENSP00000304102	ENSP00000346340
ENSP00000304102	ENSP00000350512
ENSP00000304102	ENSP00000352928
ENSP00000304102	ENSP00000354673
ENSP00000304102	ENSP00000355958
ENSP00000304102	ENSP00000356641
ENSP00000304102	ENSP00000357260
ENSP00000304102	ENSP00000360286
ENSP00000304102	ENSP00000361699
ENSP00000304102	ENSP00000363021
ENSP00000304102	ENSP00000365811
ENSP00000304102	ENSP00000370671
ENSP00000304102	ENSP00000376945
ENSP00000304102	ENSP00000380702
ENSP00000304102	ENSP00000384109
ENSP00000304102	ENSP00000384211
ENSP00000304102	ENSP00000386121
ENSP00000304102	ENSP00000386227
ENSP00000304102	ENSP00000401508
ENSP00000304102	ENSP00000417748
ENSP00000304102	ENSP00000417763

ENSP00000304133	ENSP00000363021
ENSP00000304133	ENSP00000379607
ENSP00000304169	ENSP00000311290
ENSP00000304169	ENSP00000318195
ENSP00000304169	ENSP00000344456
ENSP00000304169	ENSP00000348168
ENSP00000304169	ENSP00000353698
ENSP00000304169	ENSP00000355296
ENSP00000304169	ENSP00000359531
ENSP00000304169	ENSP00000361064
ENSP00000304169	ENSP00000363779
ENSP00000304169	ENSP00000365439
ENSP00000304169	ENSP00000370256
ENSP00000304169	ENSP00000381657
ENSP00000304169	ENSP00000392466
ENSP00000304185	ENSP00000354518
ENSP00000304226	ENSP00000307900
ENSP00000304226	ENSP00000317904
ENSP00000304226	ENSP00000322218
ENSP00000304226	ENSP00000344215
ENSP00000304226	ENSP00000347979
ENSP00000304226	ENSP00000350283
ENSP00000304226	ENSP00000353698
ENSP00000304226	ENSP00000355153
ENSP00000304226	ENSP00000357440
ENSP00000304226	ENSP00000372023
ENSP00000304226	ENSP00000396876
ENSP00000304226	ENSP00000402084
ENSP00000304236	ENSP00000316333
ENSP00000304236	ENSP00000363089
ENSP00000304236	ENSP00000365435
ENSP00000304250	ENSP00000348602
ENSP00000304292	ENSP00000334100
ENSP00000304292	ENSP00000353564
ENSP00000304292	ENSP00000354486
ENSP00000304292	ENSP00000354957
ENSP00000304292	ENSP00000356465
ENSP00000304292	ENSP00000364212
ENSP00000304292	ENSP00000381293
ENSP00000304292	ENSP00000419628
ENSP00000304308	ENSP00000311825
ENSP00000304308	ENSP00000349049
ENSP00000304311	ENSP00000307046
ENSP00000304311	ENSP00000346839
ENSP00000304311	ENSP00000350447
ENSP00000304311	ENSP00000370719

ENSP00000304311	ENSP00000380921
ENSP00000304311	ENSP00000406482
ENSP00000304321	ENSP00000312735
ENSP00000304321	ENSP00000324124
ENSP00000304321	ENSP00000354201
ENSP00000304321	ENSP00000360116
ENSP00000304321	ENSP00000403852
ENSP00000304327	ENSP00000312981
ENSP00000304327	ENSP00000319169
ENSP00000304327	ENSP00000350767
ENSP00000304327	ENSP00000415151
ENSP00000304350	ENSP00000304370
ENSP00000304350	ENSP00000304668
ENSP00000304350	ENSP00000304903
ENSP00000304350	ENSP00000305790
ENSP00000304350	ENSP00000308546
ENSP00000304350	ENSP00000310440
ENSP00000304350	ENSP00000310448
ENSP00000304350	ENSP00000310596
ENSP00000304350	ENSP00000310723
ENSP00000304350	ENSP00000312981
ENSP00000304350	ENSP00000313674
ENSP00000304350	ENSP00000313829
ENSP00000304350	ENSP00000315379
ENSP00000304350	ENSP00000315859
ENSP00000304350	ENSP00000316240
ENSP00000304350	ENSP00000318861
ENSP00000304350	ENSP00000319341
ENSP00000304350	ENSP00000324122
ENSP00000304350	ENSP00000326261
ENSP00000304350	ENSP00000326806
ENSP00000304350	ENSP00000328992
ENSP00000304350	ENSP00000329466
ENSP00000304350	ENSP00000331087
ENSP00000304350	ENSP00000331907
ENSP00000304350	ENSP00000334148
ENSP00000304350	ENSP00000335321
ENSP00000304350	ENSP00000337194
ENSP00000304350	ENSP00000341243
ENSP00000304350	ENSP00000342032
ENSP00000304350	ENSP00000342374
ENSP00000304350	ENSP00000343557
ENSP00000304350	ENSP00000345530
ENSP00000304350	ENSP00000346120
ENSP00000304350	ENSP00000346762
ENSP00000304350	ENSP00000346892

ENSP00000304350	ENSP00000346986
ENSP00000304350	ENSP00000348010
ENSP00000304350	ENSP00000351137
ENSP00000304350	ENSP00000351492
ENSP00000304350	ENSP00000353564
ENSP00000304350	ENSP00000354518
ENSP00000304350	ENSP00000354522
ENSP00000304350	ENSP00000354951
ENSP00000304350	ENSP00000356520
ENSP00000304350	ENSP00000357631
ENSP00000304350	ENSP00000358554
ENSP00000304350	ENSP00000360532
ENSP00000304350	ENSP00000361577
ENSP00000304350	ENSP00000362041
ENSP00000304350	ENSP00000362110
ENSP00000304350	ENSP00000362936
ENSP00000304350	ENSP00000363313
ENSP00000304350	ENSP00000364289
ENSP00000304350	ENSP00000364813
ENSP00000304350	ENSP00000366179
ENSP00000304350	ENSP00000366557
ENSP00000304350	ENSP00000366607
ENSP00000304350	ENSP00000367835
ENSP00000304350	ENSP00000368523
ENSP00000304350	ENSP00000368589
ENSP00000304350	ENSP00000368801
ENSP00000304350	ENSP00000370126
ENSP00000304350	ENSP00000370589
ENSP00000304350	ENSP00000371451
ENSP00000304350	ENSP00000372883
ENSP00000304350	ENSP00000376973
ENSP00000304350	ENSP00000377262
ENSP00000304350	ENSP00000378605
ENSP00000304350	ENSP00000379607
ENSP00000304350	ENSP00000387006
ENSP00000304350	ENSP00000387170
ENSP00000304350	ENSP00000393953
ENSP00000304350	ENSP00000400591
ENSP00000304350	ENSP00000401566
ENSP00000304350	ENSP00000403345
ENSP00000304350	ENSP00000406280
ENSP00000304350	ENSP00000410758
ENSP00000304350	ENSP00000412566
ENSP00000304350	ENSP00000414006
ENSP00000304350	ENSP00000414634
ENSP00000304353	ENSP00000317691

ENSP00000304353	ENSP00000362314
ENSP00000304353	ENSP00000367439
ENSP00000304353	ENSP00000369391
ENSP00000304353	ENSP00000370119
ENSP00000304353	ENSP00000389182
ENSP00000304360	ENSP00000349594
ENSP00000304370	ENSP00000305790
ENSP00000304370	ENSP00000306983
ENSP00000304370	ENSP00000307863
ENSP00000304370	ENSP00000308369
ENSP00000304370	ENSP00000311083
ENSP00000304370	ENSP00000318861
ENSP00000304370	ENSP00000319341
ENSP00000304370	ENSP00000326411
ENSP00000304370	ENSP00000326806
ENSP00000304370	ENSP00000331907
ENSP00000304370	ENSP00000332103
ENSP00000304370	ENSP00000335321
ENSP00000304370	ENSP00000340019
ENSP00000304370	ENSP00000341885
ENSP00000304370	ENSP00000342374
ENSP00000304370	ENSP00000343557
ENSP00000304370	ENSP00000348010
ENSP00000304370	ENSP00000349959
ENSP00000304370	ENSP00000351137
ENSP00000304370	ENSP00000351492
ENSP00000304370	ENSP00000356541
ENSP00000304370	ENSP00000356652
ENSP00000304370	ENSP00000357440
ENSP00000304370	ENSP00000357631
ENSP00000304370	ENSP00000358554
ENSP00000304370	ENSP00000359837
ENSP00000304370	ENSP00000360116
ENSP00000304370	ENSP00000360497
ENSP00000304370	ENSP00000360532
ENSP00000304370	ENSP00000361047
ENSP00000304370	ENSP00000361557
ENSP00000304370	ENSP00000361577
ENSP00000304370	ENSP00000362110
ENSP00000304370	ENSP00000362413
ENSP00000304370	ENSP00000363021
ENSP00000304370	ENSP00000364289
ENSP00000304370	ENSP00000366557
ENSP00000304370	ENSP00000366607
ENSP00000304370	ENSP00000367835
ENSP00000304370	ENSP00000368565

ENSP00000304370	ENSP00000368881
ENSP00000304370	ENSP00000369695
ENSP00000304370	ENSP00000369887
ENSP00000304370	ENSP00000376973
ENSP00000304370	ENSP00000377262
ENSP00000304370	ENSP00000379607
ENSP00000304370	ENSP00000385903
ENSP00000304370	ENSP00000387006
ENSP00000304370	ENSP00000391432
ENSP00000304370	ENSP00000400591
ENSP00000304370	ENSP00000412566
ENSP00000304370	ENSP00000416959
ENSP00000304437	ENSP00000340820
ENSP00000304437	ENSP00000353375
ENSP00000304437	ENSP00000353508
ENSP00000304447	ENSP00000381863
ENSP00000304500	ENSP00000306106
ENSP00000304500	ENSP00000308496
ENSP00000304500	ENSP00000310596
ENSP00000304500	ENSP00000314036
ENSP00000304500	ENSP00000319341
ENSP00000304500	ENSP00000325002
ENSP00000304500	ENSP00000345528
ENSP00000304500	ENSP00000355968
ENSP00000304500	ENSP00000357048
ENSP00000304500	ENSP00000357998
ENSP00000304500	ENSP00000358831
ENSP00000304500	ENSP00000359000
ENSP00000304500	ENSP00000361057
ENSP00000304500	ENSP00000364188
ENSP00000304500	ENSP00000364813
ENSP00000304500	ENSP00000372857
ENSP00000304500	ENSP00000372883
ENSP00000304500	ENSP00000403345
ENSP00000304500	ENSP00000406280
ENSP00000304500	ENSP00000410758
ENSP00000304500	ENSP00000414006
ENSP00000304500	ENSP00000414634
ENSP00000304544	ENSP00000308236
ENSP00000304592	ENSP00000310129
ENSP00000304592	ENSP00000311344
ENSP00000304592	ENSP00000313420
ENSP00000304592	ENSP00000319977
ENSP00000304592	ENSP00000329466
ENSP00000304592	ENSP00000340019
ENSP00000304592	ENSP00000345681

ENSP00000304592	ENSP00000347979
ENSP00000304592	ENSP00000354347
ENSP00000304592	ENSP00000354742
ENSP00000304592	ENSP00000355759
ENSP00000304592	ENSP00000360532
ENSP00000304592	ENSP00000367923
ENSP00000304592	ENSP00000374467
ENSP00000304592	ENSP00000377446
ENSP00000304592	ENSP00000377527
ENSP00000304592	ENSP00000378180
ENSP00000304592	ENSP00000379607
ENSP00000304592	ENSP00000381031
ENSP00000304592	ENSP00000396704
ENSP00000304593	ENSP00000304704
ENSP00000304593	ENSP00000307208
ENSP00000304593	ENSP00000308944
ENSP00000304593	ENSP00000310448
ENSP00000304593	ENSP00000310521
ENSP00000304593	ENSP00000310596
ENSP00000304593	ENSP00000311665
ENSP00000304593	ENSP00000312244
ENSP00000304593	ENSP00000312697
ENSP00000304593	ENSP00000312778
ENSP00000304593	ENSP00000317156
ENSP00000304593	ENSP00000318313
ENSP00000304593	ENSP00000320566
ENSP00000304593	ENSP00000322242
ENSP00000304593	ENSP00000322579
ENSP00000304593	ENSP00000323302
ENSP00000304593	ENSP00000325677
ENSP00000304593	ENSP00000326806
ENSP00000304593	ENSP00000327716
ENSP00000304593	ENSP00000329933
ENSP00000304593	ENSP00000331310
ENSP00000304593	ENSP00000334188
ENSP00000304593	ENSP00000336842
ENSP00000304593	ENSP00000337405
ENSP00000304593	ENSP00000339090
ENSP00000304593	ENSP00000339449
ENSP00000304593	ENSP00000340271
ENSP00000304593	ENSP00000340896
ENSP00000304593	ENSP00000347602
ENSP00000304593	ENSP00000347719
ENSP00000304593	ENSP00000348069
ENSP00000304593	ENSP00000348129
ENSP00000304593	ENSP00000348551

ENSP00000304593	ENSP00000349213
ENSP00000304593	ENSP00000350512
ENSP00000304593	ENSP00000352738
ENSP00000304593	ENSP00000353622
ENSP00000304593	ENSP00000355013
ENSP00000304593	ENSP00000356405
ENSP00000304593	ENSP00000356641
ENSP00000304593	ENSP00000356774
ENSP00000304593	ENSP00000356989
ENSP00000304593	ENSP00000357887
ENSP00000304593	ENSP00000358064
ENSP00000304593	ENSP00000359518
ENSP00000304593	ENSP00000359818
ENSP00000304593	ENSP00000360116
ENSP00000304593	ENSP00000360473
ENSP00000304593	ENSP00000360515
ENSP00000304593	ENSP00000362166
ENSP00000304593	ENSP00000362532
ENSP00000304593	ENSP00000362900
ENSP00000304593	ENSP00000363313
ENSP00000304593	ENSP00000363734
ENSP00000304593	ENSP00000364929
ENSP00000304593	ENSP00000365025
ENSP00000304593	ENSP00000365439
ENSP00000304593	ENSP00000371475
ENSP00000304593	ENSP00000372694
ENSP00000304593	ENSP00000374280
ENSP00000304593	ENSP00000374529
ENSP00000304593	ENSP00000378165
ENSP00000304593	ENSP00000379217
ENSP00000304593	ENSP00000380460
ENSP00000304593	ENSP00000382562
ENSP00000304593	ENSP00000386759
ENSP00000304593	ENSP00000390475
ENSP00000304593	ENSP00000402240
ENSP00000304593	ENSP00000406293
ENSP00000304593	ENSP00000412319
ENSP00000304593	ENSP00000413405
ENSP00000304593	ENSP00000415678
ENSP00000304604	ENSP00000315465
ENSP00000304604	ENSP00000347206
ENSP00000304604	ENSP00000348877
ENSP00000304604	ENSP00000358301
ENSP00000304604	ENSP00000363163
ENSP00000304604	ENSP00000368824
ENSP00000304604	ENSP00000369400

ENSP00000304604	ENSP00000369816
ENSP00000304604	ENSP00000371341
ENSP00000304604	ENSP00000379457
ENSP00000304604	ENSP00000400258
ENSP00000304604	ENSP00000401435
ENSP00000304668	ENSP00000310448
ENSP00000304668	ENSP00000313490
ENSP00000304668	ENSP00000315925
ENSP00000304668	ENSP00000324122
ENSP00000304668	ENSP00000325448
ENSP00000304668	ENSP00000327070
ENSP00000304668	ENSP00000336702
ENSP00000304668	ENSP00000353098
ENSP00000304668	ENSP00000353575
ENSP00000304668	ENSP00000354125
ENSP00000304668	ENSP00000358563
ENSP00000304668	ENSP00000359345
ENSP00000304668	ENSP00000361982
ENSP00000304668	ENSP00000363397
ENSP00000304668	ENSP00000364119
ENSP00000304668	ENSP00000366746
ENSP00000304668	ENSP00000368686
ENSP00000304668	ENSP00000370258
ENSP00000304668	ENSP00000370589
ENSP00000304668	ENSP00000374399
ENSP00000304668	ENSP00000394869
ENSP00000304668	ENSP00000399511
ENSP00000304668	ENSP00000412553
ENSP00000304668	ENSP00000415316
ENSP00000304668	ENSP00000417229
ENSP00000304676	ENSP00000337396
ENSP00000304676	ENSP00000344666
ENSP00000304676	ENSP00000367972
ENSP00000304689	ENSP00000309555
ENSP00000304689	ENSP00000328940
ENSP00000304689	ENSP00000353622
ENSP00000304689	ENSP00000359988
ENSP00000304704	ENSP00000305524
ENSP00000304704	ENSP00000315791
ENSP00000304704	ENSP00000325377
ENSP00000304704	ENSP00000326514
ENSP00000304704	ENSP00000327487
ENSP00000304704	ENSP00000329029
ENSP00000304704	ENSP00000331817
ENSP00000304704	ENSP00000332444
ENSP00000304704	ENSP00000339245

ENSP00000304704	ENSP00000339353
ENSP00000304704	ENSP00000345096
ENSP00000304704	ENSP00000347329
ENSP00000304704	ENSP00000348010
ENSP00000304704	ENSP00000355299
ENSP00000304704	ENSP00000357887
ENSP00000304704	ENSP00000359531
ENSP00000304704	ENSP00000364289
ENSP00000304704	ENSP00000368165
ENSP00000304704	ENSP00000391774
ENSP00000304704	ENSP00000392859
ENSP00000304707	ENSP00000350132
ENSP00000304802	ENSP00000308546
ENSP00000304802	ENSP00000321636
ENSP00000304802	ENSP00000346577
ENSP00000304802	ENSP00000360532
ENSP00000304802	ENSP00000364929
ENSP00000304802	ENSP00000368927
ENSP00000304802	ENSP00000370150
ENSP00000304802	ENSP00000370151
ENSP00000304802	ENSP00000388330
ENSP00000304802	ENSP00000419370
ENSP00000304803	ENSP00000329466
ENSP00000304803	ENSP00000334813
ENSP00000304803	ENSP00000379607
ENSP00000304822	ENSP00000341030
ENSP00000304822	ENSP00000346340
ENSP00000304822	ENSP00000350512
ENSP00000304854	ENSP00000350249
ENSP00000304854	ENSP00000350512
ENSP00000304854	ENSP00000377496
ENSP00000304903	ENSP00000305721
ENSP00000304903	ENSP00000310448
ENSP00000304903	ENSP00000310596
ENSP00000304903	ENSP00000310723
ENSP00000304903	ENSP00000312981
ENSP00000304903	ENSP00000313171
ENSP00000304903	ENSP00000313674
ENSP00000304903	ENSP00000313829
ENSP00000304903	ENSP00000327704
ENSP00000304903	ENSP00000331087
ENSP00000304903	ENSP00000336606
ENSP00000304903	ENSP00000339090
ENSP00000304903	ENSP00000345530
ENSP00000304903	ENSP00000349748
ENSP00000304903	ENSP00000351492

ENSP00000304903	ENSP00000352668
ENSP00000304903	ENSP00000358490
ENSP00000304903	ENSP00000358497
ENSP00000304903	ENSP00000363313
ENSP00000304903	ENSP00000364847
ENSP00000304903	ENSP00000365891
ENSP00000304903	ENSP00000366607
ENSP00000304903	ENSP00000372689
ENSP00000304903	ENSP00000374529
ENSP00000304903	ENSP00000380336
ENSP00000304903	ENSP00000381031
ENSP00000304903	ENSP00000401566
ENSP00000304903	ENSP00000412566
ENSP00000304903	ENSP00000420405
ENSP00000304908	ENSP00000313921
ENSP00000304908	ENSP00000321259
ENSP00000304908	ENSP00000356785
ENSP00000304908	ENSP00000381293
ENSP00000304915	ENSP00000360730
ENSP00000304941	ENSP00000376827
ENSP00000304945	ENSP00000355809
ENSP00000304987	ENSP00000367104
ENSP00000305133	ENSP00000341208
ENSP00000305133	ENSP00000350757
ENSP00000305133	ENSP00000373700
ENSP00000305161	ENSP00000331288
ENSP00000305161	ENSP00000347836
ENSP00000305200	ENSP00000349932
ENSP00000305230	ENSP00000341947
ENSP00000305230	ENSP00000345917
ENSP00000305255	ENSP00000305810
ENSP00000305255	ENSP00000309457
ENSP00000305255	ENSP00000310521
ENSP00000305255	ENSP00000322775
ENSP00000305255	ENSP00000324628
ENSP00000305255	ENSP00000325421
ENSP00000305255	ENSP00000326534
ENSP00000305255	ENSP00000327650
ENSP00000305255	ENSP00000342136
ENSP00000305255	ENSP00000343435
ENSP00000305255	ENSP00000344055
ENSP00000305255	ENSP00000346466
ENSP00000305255	ENSP00000348215
ENSP00000305255	ENSP00000352021
ENSP00000305255	ENSP00000356146
ENSP00000305255	ENSP00000357048

ENSP00000305255	ENSP00000360183
ENSP00000305255	ENSP00000361245
ENSP00000305255	ENSP00000362399
ENSP00000305255	ENSP00000369810
ENSP00000305255	ENSP00000379933
ENSP00000305255	ENSP00000381293
ENSP00000305255	ENSP00000389934
ENSP00000305255	ENSP00000390783
ENSP00000305255	ENSP00000401435
ENSP00000305255	ENSP00000418661
ENSP00000305422	ENSP00000312150
ENSP00000305422	ENSP00000325690
ENSP00000305422	ENSP00000329357
ENSP00000305422	ENSP00000336790
ENSP00000305422	ENSP00000339957
ENSP00000305422	ENSP00000340671
ENSP00000305422	ENSP00000341208
ENSP00000305422	ENSP00000341551
ENSP00000305422	ENSP00000344352
ENSP00000305422	ENSP00000345341
ENSP00000305422	ENSP00000348551
ENSP00000305422	ENSP00000351926
ENSP00000305422	ENSP00000352514
ENSP00000305422	ENSP00000353622
ENSP00000305422	ENSP00000356595
ENSP00000305422	ENSP00000357047
ENSP00000305422	ENSP00000358994
ENSP00000305422	ENSP00000359531
ENSP00000305422	ENSP00000365439
ENSP00000305422	ENSP00000381840
ENSP00000305422	ENSP00000386165
ENSP00000305422	ENSP00000414187
ENSP00000305442	ENSP00000305459
ENSP00000305442	ENSP00000390783
ENSP00000305459	ENSP00000308496
ENSP00000305459	ENSP00000309163
ENSP00000305459	ENSP00000310003
ENSP00000305459	ENSP00000314499
ENSP00000305459	ENSP00000316990
ENSP00000305459	ENSP00000325677
ENSP00000305459	ENSP00000327716
ENSP00000305459	ENSP00000328079
ENSP00000305459	ENSP00000329419
ENSP00000305459	ENSP00000330408
ENSP00000305459	ENSP00000336724
ENSP00000305459	ENSP00000344055

ENSP00000305459	ENSP00000347408
ENSP00000305459	ENSP00000349877
ENSP00000305459	ENSP00000349887
ENSP00000305459	ENSP00000352708
ENSP00000305459	ENSP00000354519
ENSP00000305459	ENSP00000356146
ENSP00000305459	ENSP00000359067
ENSP00000305459	ENSP00000359518
ENSP00000305459	ENSP00000360183
ENSP00000305459	ENSP00000363313
ENSP00000305459	ENSP00000363349
ENSP00000305459	ENSP00000371546
ENSP00000305459	ENSP00000372720
ENSP00000305459	ENSP00000377769
ENSP00000305459	ENSP00000381293
ENSP00000305459	ENSP00000390783
ENSP00000305459	ENSP00000396452
ENSP00000305459	ENSP00000397441
ENSP00000305459	ENSP00000402240
ENSP00000305465	ENSP00000318147
ENSP00000305465	ENSP00000321259
ENSP00000305465	ENSP00000379042
ENSP00000305502	ENSP00000360025
ENSP00000305503	ENSP00000308275
ENSP00000305503	ENSP00000308622
ENSP00000305503	ENSP00000310668
ENSP00000305503	ENSP00000315017
ENSP00000305503	ENSP00000316032
ENSP00000305503	ENSP00000318195
ENSP00000305503	ENSP00000327821
ENSP00000305503	ENSP00000341243
ENSP00000305503	ENSP00000343701
ENSP00000305503	ENSP00000344456
ENSP00000305503	ENSP00000344547
ENSP00000305503	ENSP00000346453
ENSP00000305503	ENSP00000348168
ENSP00000305503	ENSP00000352400
ENSP00000305503	ENSP00000352738
ENSP00000305503	ENSP00000356989
ENSP00000305503	ENSP00000357440
ENSP00000305503	ENSP00000357484
ENSP00000305503	ENSP00000360031
ENSP00000305503	ENSP00000360286
ENSP00000305503	ENSP00000362824
ENSP00000305503	ENSP00000365837
ENSP00000305503	ENSP00000368856

ENSP00000305503	ENSP00000369042
ENSP00000305503	ENSP00000371155
ENSP00000305503	ENSP00000372199
ENSP00000305503	ENSP00000379607
ENSP00000305503	ENSP00000382779
ENSP00000305503	ENSP00000396301
ENSP00000305503	ENSP00000402338
ENSP00000305524	ENSP00000323046
ENSP00000305524	ENSP00000327487
ENSP00000305524	ENSP00000332444
ENSP00000305524	ENSP00000355299
ENSP00000305524	ENSP00000361433
ENSP00000305613	ENSP00000311505
ENSP00000305631	ENSP00000313921
ENSP00000305631	ENSP00000350249
ENSP00000305647	ENSP00000309457
ENSP00000305647	ENSP00000310521
ENSP00000305647	ENSP00000314615
ENSP00000305647	ENSP00000326534
ENSP00000305647	ENSP00000327650
ENSP00000305647	ENSP00000329219
ENSP00000305647	ENSP00000337501
ENSP00000305647	ENSP00000344055
ENSP00000305647	ENSP00000369810
ENSP00000305647	ENSP00000381293
ENSP00000305647	ENSP00000389934
ENSP00000305647	ENSP00000390783
ENSP00000305647	ENSP00000417748
ENSP00000305647	ENSP00000418661
ENSP00000305653	ENSP00000306548
ENSP00000305653	ENSP00000312311
ENSP00000305653	ENSP00000325562
ENSP00000305653	ENSP00000329967
ENSP00000305653	ENSP00000338293
ENSP00000305653	ENSP00000339844
ENSP00000305653	ENSP00000341692
ENSP00000305653	ENSP00000354554
ENSP00000305653	ENSP00000356405
ENSP00000305653	ENSP00000362036
ENSP00000305653	ENSP00000362208
ENSP00000305653	ENSP00000367637
ENSP00000305653	ENSP00000368468
ENSP00000305653	ENSP00000377486
ENSP00000305653	ENSP00000386227
ENSP00000305653	ENSP00000420714
ENSP00000305664	ENSP00000355231

ENSP00000305664	ENSP00000383623
ENSP00000305675	ENSP00000339191
ENSP00000305682	ENSP00000317334
ENSP00000305682	ENSP00000340019
ENSP00000305682	ENSP00000359939
ENSP00000305682	ENSP00000376946
ENSP00000305682	ENSP00000420714
ENSP00000305689	ENSP00000314949
ENSP00000305689	ENSP00000322457
ENSP00000305689	ENSP00000325690
ENSP00000305689	ENSP00000328773
ENSP00000305689	ENSP00000369695
ENSP00000305689	ENSP00000380054
ENSP00000305689	ENSP00000381657
ENSP00000305696	ENSP00000368797
ENSP00000305702	ENSP00000307889
ENSP00000305702	ENSP00000309334
ENSP00000305702	ENSP00000309433
ENSP00000305702	ENSP00000311430
ENSP00000305702	ENSP00000314348
ENSP00000305702	ENSP00000315674
ENSP00000305702	ENSP00000317992
ENSP00000305702	ENSP00000320917
ENSP00000305702	ENSP00000322396
ENSP00000305702	ENSP00000323858
ENSP00000305702	ENSP00000328690
ENSP00000305702	ENSP00000332340
ENSP00000305702	ENSP00000338573
ENSP00000305702	ENSP00000338862
ENSP00000305702	ENSP00000339095
ENSP00000305702	ENSP00000339145
ENSP00000305702	ENSP00000339795
ENSP00000305702	ENSP00000342181
ENSP00000305702	ENSP00000345096
ENSP00000305702	ENSP00000346022
ENSP00000305702	ENSP00000346050
ENSP00000305702	ENSP00000346120
ENSP00000305702	ENSP00000346725
ENSP00000305702	ENSP00000347087
ENSP00000305702	ENSP00000352021
ENSP00000305702	ENSP00000352678
ENSP00000305702	ENSP00000353246
ENSP00000305702	ENSP00000354040
ENSP00000305702	ENSP00000355541
ENSP00000305702	ENSP00000355899
ENSP00000305702	ENSP00000358563

ENSP00000305702	ENSP00000358812
ENSP00000305702	ENSP00000359345
ENSP00000305702	ENSP00000359688
ENSP00000305702	ENSP00000360031
ENSP00000305702	ENSP00000360412
ENSP00000305702	ENSP00000360828
ENSP00000305702	ENSP00000361076
ENSP00000305702	ENSP00000362153
ENSP00000305702	ENSP00000362744
ENSP00000305702	ENSP00000363018
ENSP00000305702	ENSP00000363559
ENSP00000305702	ENSP00000363676
ENSP00000305702	ENSP00000363746
ENSP00000305702	ENSP00000363944
ENSP00000305702	ENSP00000364320
ENSP00000305702	ENSP00000365806
ENSP00000305702	ENSP00000366519
ENSP00000305702	ENSP00000368887
ENSP00000305702	ENSP00000370589
ENSP00000305702	ENSP00000372199
ENSP00000305702	ENSP00000372695
ENSP00000305702	ENSP00000373090
ENSP00000305702	ENSP00000374390
ENSP00000305702	ENSP00000375730
ENSP00000305702	ENSP00000377865
ENSP00000305702	ENSP00000379888
ENSP00000305702	ENSP00000380453
ENSP00000305702	ENSP00000380982
ENSP00000305702	ENSP00000382392
ENSP00000305702	ENSP00000389103
ENSP00000305702	ENSP00000392859
ENSP00000305702	ENSP00000394290
ENSP00000305702	ENSP00000399454
ENSP00000305702	ENSP00000402338
ENSP00000305702	ENSP00000402869
ENSP00000305702	ENSP00000403576
ENSP00000305702	ENSP00000405614
ENSP00000305702	ENSP00000409074
ENSP00000305702	ENSP00000409367
ENSP00000305702	ENSP00000411162
ENSP00000305702	ENSP00000417229
ENSP00000305721	ENSP00000348577
ENSP00000305721	ENSP00000358147
ENSP00000305721	ENSP00000362314
ENSP00000305721	ENSP00000384109
ENSP00000305785	ENSP00000325376

ENSP00000305785	ENSP00000346120
ENSP00000305785	ENSP00000346634
ENSP00000305785	ENSP00000354039
ENSP00000305785	ENSP00000355296
ENSP00000305785	ENSP00000370968
ENSP00000305790	ENSP00000310448
ENSP00000305790	ENSP00000311825
ENSP00000305790	ENSP00000311873
ENSP00000305790	ENSP00000315035
ENSP00000305790	ENSP00000315379
ENSP00000305790	ENSP00000315859
ENSP00000305790	ENSP00000316476
ENSP00000305790	ENSP00000318861
ENSP00000305790	ENSP00000319341
ENSP00000305790	ENSP00000323302
ENSP00000305790	ENSP00000324122
ENSP00000305790	ENSP00000326261
ENSP00000305790	ENSP00000326806
ENSP00000305790	ENSP00000331201
ENSP00000305790	ENSP00000332103
ENSP00000305790	ENSP00000333667
ENSP00000305790	ENSP00000334148
ENSP00000305790	ENSP00000334538
ENSP00000305790	ENSP00000335321
ENSP00000305790	ENSP00000337405
ENSP00000305790	ENSP00000339090
ENSP00000305790	ENSP00000341351
ENSP00000305790	ENSP00000342374
ENSP00000305790	ENSP00000343745
ENSP00000305790	ENSP00000345096
ENSP00000305790	ENSP00000347733
ENSP00000305790	ENSP00000348010
ENSP00000305790	ENSP00000351137
ENSP00000305790	ENSP00000351947
ENSP00000305790	ENSP00000352021
ENSP00000305790	ENSP00000352257
ENSP00000305790	ENSP00000354522
ENSP00000305790	ENSP00000356541
ENSP00000305790	ENSP00000357631
ENSP00000305790	ENSP00000358417
ENSP00000305790	ENSP00000358605
ENSP00000305790	ENSP00000360515
ENSP00000305790	ENSP00000360532
ENSP00000305790	ENSP00000360817
ENSP00000305790	ENSP00000360876
ENSP00000305790	ENSP00000361162

ENSP00000305790	ENSP00000361557
ENSP00000305790	ENSP00000361577
ENSP00000305790	ENSP00000362110
ENSP00000305790	ENSP00000362936
ENSP00000305790	ENSP00000363313
ENSP00000305790	ENSP00000364289
ENSP00000305790	ENSP00000365877
ENSP00000305790	ENSP00000366557
ENSP00000305790	ENSP00000366974
ENSP00000305790	ENSP00000368801
ENSP00000305790	ENSP00000372023
ENSP00000305790	ENSP00000379607
ENSP00000305790	ENSP00000382688
ENSP00000305790	ENSP00000386161
ENSP00000305790	ENSP00000387006
ENSP00000305790	ENSP00000391432
ENSP00000305790	ENSP00000400591
ENSP00000305790	ENSP00000408994
ENSP00000305790	ENSP00000412566
ENSP00000305790	ENSP00000416959
ENSP00000305804	ENSP00000331310
ENSP00000305804	ENSP00000417132
ENSP00000305810	ENSP00000311134
ENSP00000305810	ENSP00000317714
ENSP00000305810	ENSP00000324628
ENSP00000305810	ENSP00000355596
ENSP00000305810	ENSP00000356530
ENSP00000305810	ENSP00000362334
ENSP00000305810	ENSP00000366225
ENSP00000305810	ENSP00000374529
ENSP00000305810	ENSP00000381293
ENSP00000305810	ENSP00000383719
ENSP00000305810	ENSP00000390783
ENSP00000305810	ENSP00000401445
ENSP00000305877	ENSP00000370410
ENSP00000305892	ENSP00000327716
ENSP00000305892	ENSP00000377401
ENSP00000305899	ENSP00000349049
ENSP00000305899	ENSP00000366974
ENSP00000305899	ENSP00000371475
ENSP00000305899	ENSP00000372023
ENSP00000305906	ENSP00000352219
ENSP00000305906	ENSP00000369862
ENSP00000305924	ENSP00000307214
ENSP00000305924	ENSP00000318604
ENSP00000305924	ENSP00000347719

ENSP00000305924	ENSP00000348784
ENSP00000305924	ENSP00000371888
ENSP00000305941	ENSP00000346566
ENSP00000305941	ENSP00000351596
ENSP00000305941	ENSP00000356448
ENSP00000305941	ENSP00000382562
ENSP00000305958	ENSP00000310448
ENSP00000305958	ENSP00000318861
ENSP00000305958	ENSP00000321320
ENSP00000305958	ENSP00000327070
ENSP00000305958	ENSP00000336702
ENSP00000305958	ENSP00000339109
ENSP00000305958	ENSP00000344431
ENSP00000305958	ENSP00000346762
ENSP00000305958	ENSP00000350249
ENSP00000305958	ENSP00000357440
ENSP00000305958	ENSP00000358019
ENSP00000305958	ENSP00000362110
ENSP00000305958	ENSP00000362413
ENSP00000305958	ENSP00000366395
ENSP00000305958	ENSP00000367208
ENSP00000305958	ENSP00000371451
ENSP00000305958	ENSP00000371888
ENSP00000305958	ENSP00000377496
ENSP00000305958	ENSP00000391944
ENSP00000305958	ENSP00000394071
ENSP00000305958	ENSP00000397009
ENSP00000305958	ENSP00000404381
ENSP00000305976	ENSP00000311344
ENSP00000305976	ENSP00000334061
ENSP00000305976	ENSP00000343392
ENSP00000305976	ENSP00000377793
ENSP00000305976	ENSP00000378306
ENSP00000306003	ENSP00000358737
ENSP00000306003	ENSP00000362306
ENSP00000306003	ENSP00000370414
ENSP00000306003	ENSP00000389649
ENSP00000306015	ENSP00000313059
ENSP00000306015	ENSP00000361418
ENSP00000306015	ENSP00000367440
ENSP00000306080	ENSP00000311984
ENSP00000306080	ENSP00000312981
ENSP00000306080	ENSP00000314441
ENSP00000306080	ENSP00000321711
ENSP00000306080	ENSP00000322016
ENSP00000306080	ENSP00000324956

ENSP00000306080	ENSP00000326981
ENSP00000306080	ENSP00000339503
ENSP00000306080	ENSP00000339532
ENSP00000306080	ENSP00000341082
ENSP00000306080	ENSP00000346015
ENSP00000306080	ENSP00000358605
ENSP00000306080	ENSP00000358812
ENSP00000306080	ENSP00000362036
ENSP00000306080	ENSP00000367029
ENSP00000306099	ENSP00000306361
ENSP00000306099	ENSP00000321334
ENSP00000306099	ENSP00000370557
ENSP00000306100	ENSP00000307491
ENSP00000306100	ENSP00000363591
ENSP00000306100	ENSP00000420270
ENSP00000306106	ENSP00000307275
ENSP00000306106	ENSP00000346453
ENSP00000306106	ENSP00000354676
ENSP00000306106	ENSP00000354742
ENSP00000306106	ENSP00000355968
ENSP00000306106	ENSP00000358737
ENSP00000306106	ENSP00000361057
ENSP00000306106	ENSP00000364188
ENSP00000306106	ENSP00000370414
ENSP00000306106	ENSP00000385083
ENSP00000306106	ENSP00000389649
ENSP00000306123	ENSP00000308546
ENSP00000306123	ENSP00000308944
ENSP00000306123	ENSP00000312735
ENSP00000306123	ENSP00000324124
ENSP00000306123	ENSP00000339109
ENSP00000306123	ENSP00000340019
ENSP00000306123	ENSP00000342181
ENSP00000306123	ENSP00000350249
ENSP00000306123	ENSP00000354742
ENSP00000306123	ENSP00000365131
ENSP00000306123	ENSP00000404121
ENSP00000306124	ENSP00000306138
ENSP00000306124	ENSP00000309871
ENSP00000306124	ENSP00000321636
ENSP00000306124	ENSP00000321711
ENSP00000306124	ENSP00000323087
ENSP00000306124	ENSP00000323377
ENSP00000306124	ENSP00000329419
ENSP00000306124	ENSP00000330836
ENSP00000306124	ENSP00000331242

ENSP00000306124	ENSP00000333568
ENSP00000306124	ENSP00000336702
ENSP00000306124	ENSP00000338345
ENSP00000306124	ENSP00000341189
ENSP00000306124	ENSP00000344220
ENSP00000306124	ENSP00000347134
ENSP00000306124	ENSP00000347169
ENSP00000306124	ENSP00000348573
ENSP00000306124	ENSP00000349959
ENSP00000306124	ENSP00000350928
ENSP00000306124	ENSP00000354346
ENSP00000306124	ENSP00000355296
ENSP00000306124	ENSP00000356438
ENSP00000306124	ENSP00000360916
ENSP00000306124	ENSP00000361699
ENSP00000306124	ENSP00000364188
ENSP00000306124	ENSP00000370222
ENSP00000306124	ENSP00000371475
ENSP00000306124	ENSP00000374455
ENSP00000306124	ENSP00000376445
ENSP00000306124	ENSP00000378306
ENSP00000306124	ENSP00000382562
ENSP00000306124	ENSP00000391723
ENSP00000306124	ENSP00000396704
ENSP00000306124	ENSP00000397351
ENSP00000306124	ENSP00000415203
ENSP00000306138	ENSP00000339381
ENSP00000306138	ENSP00000349465
ENSP00000306138	ENSP00000355146
ENSP00000306138	ENSP00000357484
ENSP00000306138	ENSP00000388035
ENSP00000306138	ENSP00000406066
ENSP00000306138	ENSP00000411286
ENSP00000306157	ENSP00000339804
ENSP00000306157	ENSP00000341208
ENSP00000306163	ENSP00000312624
ENSP00000306163	ENSP00000356058
ENSP00000306163	ENSP00000378286
ENSP00000306190	ENSP00000340944
ENSP00000306190	ENSP00000356346
ENSP00000306245	ENSP00000306920
ENSP00000306245	ENSP00000307235
ENSP00000306245	ENSP00000311005
ENSP00000306245	ENSP00000313432
ENSP00000306245	ENSP00000318195
ENSP00000306245	ENSP00000319104

ENSP00000306245	ENSP00000321853
ENSP00000306245	ENSP00000325690
ENSP00000306245	ENSP00000327048
ENSP00000306245	ENSP00000330343
ENSP00000306245	ENSP00000336790
ENSP00000306245	ENSP00000339495
ENSP00000306245	ENSP00000339801
ENSP00000306245	ENSP00000340671
ENSP00000306245	ENSP00000340823
ENSP00000306245	ENSP00000341243
ENSP00000306245	ENSP00000343745
ENSP00000306245	ENSP00000348168
ENSP00000306245	ENSP00000348551
ENSP00000306245	ENSP00000350681
ENSP00000306245	ENSP00000350894
ENSP00000306245	ENSP00000351894
ENSP00000306245	ENSP00000352514
ENSP00000306245	ENSP00000353344
ENSP00000306245	ENSP00000355866
ENSP00000306245	ENSP00000357283
ENSP00000306245	ENSP00000357440
ENSP00000306245	ENSP00000357494
ENSP00000306245	ENSP00000358335
ENSP00000306245	ENSP00000358854
ENSP00000306245	ENSP00000363763
ENSP00000306245	ENSP00000363812
ENSP00000306245	ENSP00000376611
ENSP00000306245	ENSP00000377969
ENSP00000306245	ENSP00000391944
ENSP00000306245	ENSP00000393286
ENSP00000306245	ENSP00000393870
ENSP00000306245	ENSP00000399968
ENSP00000306245	ENSP00000402590
ENSP00000306245	ENSP00000403536
ENSP00000306245	ENSP00000403817
ENSP00000306245	ENSP00000408994
ENSP00000306344	ENSP00000378350
ENSP00000306361	ENSP00000321853
ENSP00000306361	ENSP00000349208
ENSP00000306361	ENSP00000365175
ENSP00000306361	ENSP00000370557
ENSP00000306361	ENSP00000387477
ENSP00000306361	ENSP00000415363
ENSP00000306425	ENSP00000350512
ENSP00000306425	ENSP00000386759
ENSP00000306449	ENSP00000355865

ENSP00000306449	ENSP00000366395
ENSP00000306473	ENSP00000336868
ENSP00000306473	ENSP00000357565
ENSP00000306473	ENSP00000373272
ENSP00000306473	ENSP00000380921
ENSP00000306477	ENSP00000352706
ENSP00000306477	ENSP00000370023
ENSP00000306523	ENSP00000359206
ENSP00000306523	ENSP00000379144
ENSP00000306528	ENSP00000386134
ENSP00000306548	ENSP00000307889
ENSP00000306548	ENSP00000308275
ENSP00000306548	ENSP00000308897
ENSP00000306548	ENSP00000309334
ENSP00000306548	ENSP00000309830
ENSP00000306548	ENSP00000311028
ENSP00000306548	ENSP00000311430
ENSP00000306548	ENSP00000312311
ENSP00000306548	ENSP00000315017
ENSP00000306548	ENSP00000317376
ENSP00000306548	ENSP00000317992
ENSP00000306548	ENSP00000318646
ENSP00000306548	ENSP00000322419
ENSP00000306548	ENSP00000326981
ENSP00000306548	ENSP00000333401
ENSP00000306548	ENSP00000333837
ENSP00000306548	ENSP00000338862
ENSP00000306548	ENSP00000339095
ENSP00000306548	ENSP00000339795
ENSP00000306548	ENSP00000339845
ENSP00000306548	ENSP00000341082
ENSP00000306548	ENSP00000343741
ENSP00000306548	ENSP00000343745
ENSP00000306548	ENSP00000345156
ENSP00000306548	ENSP00000346015
ENSP00000306548	ENSP00000346022
ENSP00000306548	ENSP00000346027
ENSP00000306548	ENSP00000346037
ENSP00000306548	ENSP00000346050
ENSP00000306548	ENSP00000346088
ENSP00000306548	ENSP00000352821
ENSP00000306548	ENSP00000354040
ENSP00000306548	ENSP00000354525
ENSP00000306548	ENSP00000354739
ENSP00000306548	ENSP00000359345
ENSP00000306548	ENSP00000361076

ENSP00000306548	ENSP00000362744
ENSP00000306548	ENSP00000363018
ENSP00000306548	ENSP00000363676
ENSP00000306548	ENSP00000364320
ENSP00000306548	ENSP00000368565
ENSP00000306548	ENSP00000369042
ENSP00000306548	ENSP00000369757
ENSP00000306548	ENSP00000373363
ENSP00000306548	ENSP00000373404
ENSP00000306548	ENSP00000377486
ENSP00000306548	ENSP00000377640
ENSP00000306548	ENSP00000377865
ENSP00000306548	ENSP00000378160
ENSP00000306548	ENSP00000379888
ENSP00000306548	ENSP00000380156
ENSP00000306548	ENSP00000380982
ENSP00000306548	ENSP00000386717
ENSP00000306548	ENSP00000389103
ENSP00000306548	ENSP00000393241
ENSP00000306548	ENSP00000393393
ENSP00000306548	ENSP00000402338
ENSP00000306548	ENSP00000403175
ENSP00000306548	ENSP00000412583
ENSP00000306548	ENSP00000414321
ENSP00000306548	ENSP00000416110
ENSP00000306548	ENSP00000417602
ENSP00000306561	ENSP00000307853
ENSP00000306561	ENSP00000309572
ENSP00000306561	ENSP00000310520
ENSP00000306561	ENSP00000311873
ENSP00000306561	ENSP00000318977
ENSP00000306561	ENSP00000322570
ENSP00000306561	ENSP00000330343
ENSP00000306561	ENSP00000336606
ENSP00000306561	ENSP00000336712
ENSP00000306561	ENSP00000340305
ENSP00000306561	ENSP00000342026
ENSP00000306561	ENSP00000343392
ENSP00000306561	ENSP00000343701
ENSP00000306561	ENSP00000343745
ENSP00000306561	ENSP00000347232
ENSP00000306561	ENSP00000347978
ENSP00000306561	ENSP00000348089
ENSP00000306561	ENSP00000351284
ENSP00000306561	ENSP00000351697
ENSP00000306561	ENSP00000360116

ENSP00000306561	ENSP00000361310
ENSP00000306561	ENSP00000362105
ENSP00000306561	ENSP00000362441
ENSP00000306561	ENSP00000364126
ENSP00000306561	ENSP00000364270
ENSP00000306561	ENSP00000365811
ENSP00000306561	ENSP00000368349
ENSP00000306561	ENSP00000368438
ENSP00000306561	ENSP00000368646
ENSP00000306561	ENSP00000384048
ENSP00000306561	ENSP00000394649
ENSP00000306561	ENSP00000400806
ENSP00000306561	ENSP00000406868
ENSP00000306561	ENSP00000407047
ENSP00000306574	ENSP00000368797
ENSP00000306627	ENSP00000308496
ENSP00000306627	ENSP00000317468
ENSP00000306627	ENSP00000367299
ENSP00000306627	ENSP00000368140
ENSP00000306627	ENSP00000389399
ENSP00000306627	ENSP00000402935
ENSP00000306682	ENSP00000310596
ENSP00000306682	ENSP00000318195
ENSP00000306682	ENSP00000325863
ENSP00000306682	ENSP00000339845
ENSP00000306682	ENSP00000343190
ENSP00000306682	ENSP00000343741
ENSP00000306682	ENSP00000349205
ENSP00000306682	ENSP00000351015
ENSP00000306682	ENSP00000354522
ENSP00000306682	ENSP00000356263
ENSP00000306682	ENSP00000356530
ENSP00000306682	ENSP00000359345
ENSP00000306682	ENSP00000360268
ENSP00000306682	ENSP00000360532
ENSP00000306682	ENSP00000361064
ENSP00000306682	ENSP00000372023
ENSP00000306682	ENSP00000375986
ENSP00000306682	ENSP00000378306
ENSP00000306682	ENSP00000401445
ENSP00000306682	ENSP00000417281
ENSP00000306697	ENSP00000337838
ENSP00000306697	ENSP00000358301
ENSP00000306734	ENSP00000342026
ENSP00000306754	ENSP00000367972
ENSP00000306788	ENSP00000331201

ENSP00000306817	ENSP00000315931
ENSP00000306817	ENSP00000333019
ENSP00000306817	ENSP00000342056
ENSP00000306817	ENSP00000350249
ENSP00000306817	ENSP00000356290
ENSP00000306817	ENSP00000359976
ENSP00000306817	ENSP00000361965
ENSP00000306817	ENSP00000367615
ENSP00000306817	ENSP00000377496
ENSP00000306822	ENSP00000329623
ENSP00000306822	ENSP00000353059
ENSP00000306864	ENSP00000326366
ENSP00000306864	ENSP00000355896
ENSP00000306894	ENSP00000350283
ENSP00000306894	ENSP00000356641
ENSP00000306894	ENSP00000409346
ENSP00000306900	ENSP00000346067
ENSP00000306906	ENSP00000312735
ENSP00000306906	ENSP00000324124
ENSP00000306906	ENSP00000339377
ENSP00000306906	ENSP00000348632
ENSP00000306906	ENSP00000360116
ENSP00000306906	ENSP00000382250
ENSP00000306906	ENSP00000403852
ENSP00000306906	ENSP00000417587
ENSP00000306920	ENSP00000318921
ENSP00000306920	ENSP00000364782
ENSP00000306920	ENSP00000399309
ENSP00000306920	ENSP00000408957
ENSP00000306983	ENSP00000307684
ENSP00000306983	ENSP00000309555
ENSP00000306983	ENSP00000312244
ENSP00000306983	ENSP00000312735
ENSP00000306983	ENSP00000316114
ENSP00000306983	ENSP00000322579
ENSP00000306983	ENSP00000323720
ENSP00000306983	ENSP00000323858
ENSP00000306983	ENSP00000324444
ENSP00000306983	ENSP00000325677
ENSP00000306983	ENSP00000331310
ENSP00000306983	ENSP00000334148
ENSP00000306983	ENSP00000335044
ENSP00000306983	ENSP00000336842
ENSP00000306983	ENSP00000340271
ENSP00000306983	ENSP00000340823
ENSP00000306983	ENSP00000340896

ENSP00000306983	ENSP00000344537
ENSP00000306983	ENSP00000346453
ENSP00000306983	ENSP00000347345
ENSP00000306983	ENSP00000347602
ENSP00000306983	ENSP00000347733
ENSP00000306983	ENSP00000351697
ENSP00000306983	ENSP00000352121
ENSP00000306983	ENSP00000354633
ENSP00000306983	ENSP00000355051
ENSP00000306983	ENSP00000355205
ENSP00000306983	ENSP00000355720
ENSP00000306983	ENSP00000358854
ENSP00000306983	ENSP00000359290
ENSP00000306983	ENSP00000359531
ENSP00000306983	ENSP00000359837
ENSP00000306983	ENSP00000360515
ENSP00000306983	ENSP00000360532
ENSP00000306983	ENSP00000360621
ENSP00000306983	ENSP00000361465
ENSP00000306983	ENSP00000361998
ENSP00000306983	ENSP00000362068
ENSP00000306983	ENSP00000362166
ENSP00000306983	ENSP00000363779
ENSP00000306983	ENSP00000367406
ENSP00000306983	ENSP00000374213
ENSP00000306983	ENSP00000377969
ENSP00000306983	ENSP00000392617
ENSP00000306983	ENSP00000402338
ENSP00000306983	ENSP00000403852
ENSP00000306983	ENSP00000417281
ENSP00000306983	ENSP00000418734
ENSP00000306999	ENSP00000308699
ENSP00000306999	ENSP00000308944
ENSP00000306999	ENSP00000313171
ENSP00000306999	ENSP00000313851
ENSP00000306999	ENSP00000322570
ENSP00000306999	ENSP00000323439
ENSP00000306999	ENSP00000325017
ENSP00000306999	ENSP00000341382
ENSP00000306999	ENSP00000349049
ENSP00000306999	ENSP00000351697
ENSP00000306999	ENSP00000353332
ENSP00000306999	ENSP00000354720
ENSP00000306999	ENSP00000354957
ENSP00000306999	ENSP00000357965
ENSP00000306999	ENSP00000361310

ENSP00000306999	ENSP00000365877
ENSP00000306999	ENSP00000368349
ENSP00000306999	ENSP00000368438
ENSP00000306999	ENSP00000369411
ENSP00000306999	ENSP00000370151
ENSP00000306999	ENSP00000372689
ENSP00000306999	ENSP00000398824
ENSP00000307004	ENSP00000313420
ENSP00000307004	ENSP00000342011
ENSP00000307004	ENSP00000346050
ENSP00000307004	ENSP00000349393
ENSP00000307004	ENSP00000352257
ENSP00000307004	ENSP00000354522
ENSP00000307004	ENSP00000355759
ENSP00000307004	ENSP00000367787
ENSP00000307004	ENSP00000377769
ENSP00000307004	ENSP00000378132
ENSP00000307023	ENSP00000320566
ENSP00000307023	ENSP00000328720
ENSP00000307023	ENSP00000343535
ENSP00000307023	ENSP00000351113
ENSP00000307023	ENSP00000353622
ENSP00000307023	ENSP00000355205
ENSP00000307023	ENSP00000357887
ENSP00000307023	ENSP00000358105
ENSP00000307023	ENSP00000359531
ENSP00000307023	ENSP00000374213
ENSP00000307023	ENSP00000384313
ENSP00000307046	ENSP00000325136
ENSP00000307046	ENSP00000344468
ENSP00000307046	ENSP00000346839
ENSP00000307046	ENSP00000351015
ENSP00000307046	ENSP00000363763
ENSP00000307046	ENSP00000367408
ENSP00000307046	ENSP00000370935
ENSP00000307046	ENSP00000381098
ENSP00000307077	ENSP00000314441
ENSP00000307077	ENSP00000326579
ENSP00000307077	ENSP00000354673
ENSP00000307077	ENSP00000376268
ENSP00000307134	ENSP00000314153
ENSP00000307134	ENSP00000326366
ENSP00000307134	ENSP00000340944
ENSP00000307134	ENSP00000344456
ENSP00000307134	ENSP00000368190
ENSP00000307143	ENSP00000369757

ENSP00000307188	ENSP00000307567
ENSP00000307188	ENSP00000352928
ENSP00000307188	ENSP00000358747
ENSP00000307199	ENSP00000370736
ENSP00000307208	ENSP00000308466
ENSP00000307208	ENSP00000312244
ENSP00000307208	ENSP00000319104
ENSP00000307208	ENSP00000325863
ENSP00000307208	ENSP00000329466
ENSP00000307208	ENSP00000329933
ENSP00000307208	ENSP00000331310
ENSP00000307208	ENSP00000332171
ENSP00000307208	ENSP00000332208
ENSP00000307208	ENSP00000333920
ENSP00000307208	ENSP00000334061
ENSP00000307208	ENSP00000341805
ENSP00000307208	ENSP00000343741
ENSP00000307208	ENSP00000343885
ENSP00000307208	ENSP00000345681
ENSP00000307208	ENSP00000347602
ENSP00000307208	ENSP00000347733
ENSP00000307208	ENSP00000351284
ENSP00000307208	ENSP00000351575
ENSP00000307208	ENSP00000353622
ENSP00000307208	ENSP00000356405
ENSP00000307208	ENSP00000357047
ENSP00000307208	ENSP00000357887
ENSP00000307208	ENSP00000360515
ENSP00000307208	ENSP00000362592
ENSP00000307208	ENSP00000374213
ENSP00000307208	ENSP00000374280
ENSP00000307208	ENSP00000374455
ENSP00000307208	ENSP00000376204
ENSP00000307208	ENSP00000379065
ENSP00000307208	ENSP00000381657
ENSP00000307208	ENSP00000384434
ENSP00000307208	ENSP00000384490
ENSP00000307208	ENSP00000386759
ENSP00000307208	ENSP00000397644
ENSP00000307214	ENSP00000311344
ENSP00000307214	ENSP00000333946
ENSP00000307214	ENSP00000351885
ENSP00000307214	ENSP00000355517
ENSP00000307214	ENSP00000356257
ENSP00000307214	ENSP00000379908
ENSP00000307214	ENSP00000381198

ENSP00000307214	ENSP00000383402
ENSP00000307214	ENSP00000394085
ENSP00000307214	ENSP00000414670
ENSP00000307214	ENSP00000417963
ENSP00000307218	ENSP00000345530
ENSP00000307218	ENSP00000361014
ENSP00000307218	ENSP00000417763
ENSP00000307235	ENSP00000318944
ENSP00000307235	ENSP00000325794
ENSP00000307235	ENSP00000342755
ENSP00000307235	ENSP00000344352
ENSP00000307235	ENSP00000356641
ENSP00000307235	ENSP00000358854
ENSP00000307260	ENSP00000344220
ENSP00000307272	ENSP00000312150
ENSP00000307272	ENSP00000318313
ENSP00000307272	ENSP00000318604
ENSP00000307272	ENSP00000325677
ENSP00000307272	ENSP00000327070
ENSP00000307272	ENSP00000341243
ENSP00000307272	ENSP00000341698
ENSP00000307272	ENSP00000346437
ENSP00000307272	ENSP00000346453
ENSP00000307272	ENSP00000348784
ENSP00000307272	ENSP00000349959
ENSP00000307272	ENSP00000354125
ENSP00000307272	ENSP00000354558
ENSP00000307272	ENSP00000356807
ENSP00000307272	ENSP00000359567
ENSP00000307272	ENSP00000361009
ENSP00000307272	ENSP00000362221
ENSP00000307272	ENSP00000362314
ENSP00000307272	ENSP00000362608
ENSP00000307272	ENSP00000362688
ENSP00000307272	ENSP00000362900
ENSP00000307272	ENSP00000364486
ENSP00000307272	ENSP00000365891
ENSP00000307272	ENSP00000366843
ENSP00000307272	ENSP00000369757
ENSP00000307272	ENSP00000371888
ENSP00000307272	ENSP00000376445
ENSP00000307272	ENSP00000379093
ENSP00000307272	ENSP00000384313
ENSP00000307275	ENSP00000331201
ENSP00000307275	ENSP00000345530
ENSP00000307275	ENSP00000346453

ENSP00000307275	ENSP00000354995
ENSP00000307275	ENSP00000355968
ENSP00000307275	ENSP00000364188
ENSP00000307275	ENSP00000370607
ENSP00000307275	ENSP00000374328
ENSP00000307305	ENSP00000344220
ENSP00000307305	ENSP00000359206
ENSP00000307387	ENSP00000310596
ENSP00000307387	ENSP00000311200
ENSP00000307387	ENSP00000314129
ENSP00000307387	ENSP00000317955
ENSP00000307387	ENSP00000319341
ENSP00000307387	ENSP00000321203
ENSP00000307387	ENSP00000321326
ENSP00000307387	ENSP00000329466
ENSP00000307387	ENSP00000331201
ENSP00000307387	ENSP00000341189
ENSP00000307387	ENSP00000343535
ENSP00000307387	ENSP00000345530
ENSP00000307387	ENSP00000348565
ENSP00000307387	ENSP00000349785
ENSP00000307387	ENSP00000353701
ENSP00000307387	ENSP00000354673
ENSP00000307387	ENSP00000354927
ENSP00000307387	ENSP00000356146
ENSP00000307387	ENSP00000358478
ENSP00000307387	ENSP00000360475
ENSP00000307387	ENSP00000360540
ENSP00000307387	ENSP00000362807
ENSP00000307387	ENSP00000365005
ENSP00000307387	ENSP00000366565
ENSP00000307387	ENSP00000367197
ENSP00000307387	ENSP00000370719
ENSP00000307387	ENSP00000380921
ENSP00000307387	ENSP00000417764
ENSP00000307481	ENSP00000311876
ENSP00000307481	ENSP00000339933
ENSP00000307481	ENSP00000366620
ENSP00000307491	ENSP00000309572
ENSP00000307491	ENSP00000310040
ENSP00000307491	ENSP00000310842
ENSP00000307491	ENSP00000313171
ENSP00000307491	ENSP00000314153
ENSP00000307491	ENSP00000315182
ENSP00000307491	ENSP00000315931
ENSP00000307491	ENSP00000319104

ENSP00000307491	ENSP00000324463
ENSP00000307491	ENSP00000325634
ENSP00000307491	ENSP00000332198
ENSP00000307491	ENSP00000333934
ENSP00000307491	ENSP00000340297
ENSP00000307491	ENSP00000343526
ENSP00000307491	ENSP00000344524
ENSP00000307491	ENSP00000347408
ENSP00000307491	ENSP00000349892
ENSP00000307491	ENSP00000350283
ENSP00000307491	ENSP00000358140
ENSP00000307491	ENSP00000358563
ENSP00000307491	ENSP00000362688
ENSP00000307491	ENSP00000364578
ENSP00000307491	ENSP00000366434
ENSP00000307491	ENSP00000368438
ENSP00000307491	ENSP00000374280
ENSP00000307491	ENSP00000384109
ENSP00000307491	ENSP00000384211
ENSP00000307491	ENSP00000406888
ENSP00000307525	ENSP00000308897
ENSP00000307525	ENSP00000310042
ENSP00000307525	ENSP00000310668
ENSP00000307525	ENSP00000312870
ENSP00000307525	ENSP00000313603
ENSP00000307525	ENSP00000316240
ENSP00000307525	ENSP00000325312
ENSP00000307525	ENSP00000338573
ENSP00000307525	ENSP00000344460
ENSP00000307525	ENSP00000345096
ENSP00000307525	ENSP00000348442
ENSP00000307525	ENSP00000351524
ENSP00000307525	ENSP00000352021
ENSP00000307525	ENSP00000352400
ENSP00000307525	ENSP00000354040
ENSP00000307525	ENSP00000354739
ENSP00000307525	ENSP00000356920
ENSP00000307525	ENSP00000359837
ENSP00000307525	ENSP00000360817
ENSP00000307525	ENSP00000361076
ENSP00000307525	ENSP00000362041
ENSP00000307525	ENSP00000362153
ENSP00000307525	ENSP00000362900
ENSP00000307525	ENSP00000363559
ENSP00000307525	ENSP00000364320
ENSP00000307525	ENSP00000365806

ENSP00000307525	ENSP00000366179
ENSP00000307525	ENSP00000368523
ENSP00000307525	ENSP00000368565
ENSP00000307525	ENSP00000371451
ENSP00000307525	ENSP00000373082
ENSP00000307525	ENSP00000373090
ENSP00000307525	ENSP00000377640
ENSP00000307525	ENSP00000380982
ENSP00000307525	ENSP00000389065
ENSP00000307525	ENSP00000389103
ENSP00000307525	ENSP00000394290
ENSP00000307525	ENSP00000403576
ENSP00000307525	ENSP00000409074
ENSP00000307525	ENSP00000409279
ENSP00000307525	ENSP00000409367
ENSP00000307525	ENSP00000411162
ENSP00000307525	ENSP00000411327
ENSP00000307525	ENSP00000412553
ENSP00000307525	ENSP00000413319
ENSP00000307525	ENSP00000413632
ENSP00000307525	ENSP00000414373
ENSP00000307567	ENSP00000307900
ENSP00000307567	ENSP00000310129
ENSP00000307567	ENSP00000312735
ENSP00000307567	ENSP00000314129
ENSP00000307567	ENSP00000323288
ENSP00000307567	ENSP00000339435
ENSP00000307567	ENSP00000342300
ENSP00000307567	ENSP00000346173
ENSP00000307567	ENSP00000349577
ENSP00000307567	ENSP00000351908
ENSP00000307567	ENSP00000355890
ENSP00000307567	ENSP00000360327
ENSP00000307567	ENSP00000362576
ENSP00000307567	ENSP00000364794
ENSP00000307567	ENSP00000364815
ENSP00000307567	ENSP00000365175
ENSP00000307567	ENSP00000369038
ENSP00000307567	ENSP00000369042
ENSP00000307567	ENSP00000373331
ENSP00000307567	ENSP00000377914
ENSP00000307567	ENSP00000379607
ENSP00000307567	ENSP00000387477
ENSP00000307567	ENSP00000401121
ENSP00000307567	ENSP00000402338
ENSP00000307567	ENSP00000403359

ENSP00000307567	ENSP00000415316
ENSP00000307567	ENSP00000415363
ENSP00000307604	ENSP00000345477
ENSP00000307684	ENSP00000309555
ENSP00000307684	ENSP00000310448
ENSP00000307684	ENSP00000313420
ENSP00000307684	ENSP00000316114
ENSP00000307684	ENSP00000322579
ENSP00000307684	ENSP00000324444
ENSP00000307684	ENSP00000335044
ENSP00000307684	ENSP00000336528
ENSP00000307684	ENSP00000336868
ENSP00000307684	ENSP00000341243
ENSP00000307684	ENSP00000343741
ENSP00000307684	ENSP00000344456
ENSP00000307684	ENSP00000344537
ENSP00000307684	ENSP00000347184
ENSP00000307684	ENSP00000347602
ENSP00000307684	ENSP00000347733
ENSP00000307684	ENSP00000348069
ENSP00000307684	ENSP00000348714
ENSP00000307684	ENSP00000350078
ENSP00000307684	ENSP00000350283
ENSP00000307684	ENSP00000350967
ENSP00000307684	ENSP00000351665
ENSP00000307684	ENSP00000352121
ENSP00000307684	ENSP00000352257
ENSP00000307684	ENSP00000353622
ENSP00000307684	ENSP00000354558
ENSP00000307684	ENSP00000358854
ENSP00000307684	ENSP00000359518
ENSP00000307684	ENSP00000359837
ENSP00000307684	ENSP00000360515
ENSP00000307684	ENSP00000361245
ENSP00000307684	ENSP00000361577
ENSP00000307684	ENSP00000361965
ENSP00000307684	ENSP00000361998
ENSP00000307684	ENSP00000365943
ENSP00000307684	ENSP00000366179
ENSP00000307684	ENSP00000368565
ENSP00000307684	ENSP00000369695
ENSP00000307684	ENSP00000373399
ENSP00000307684	ENSP00000377747
ENSP00000307684	ENSP00000385903
ENSP00000307684	ENSP00000386394
ENSP00000307684	ENSP00000392028

ENSP00000307684	ENSP00000396620
ENSP00000307684	ENSP00000396749
ENSP00000307684	ENSP00000397259
ENSP00000307684	ENSP00000402515
ENSP00000307746	ENSP00000338629
ENSP00000307746	ENSP00000344666
ENSP00000307746	ENSP00000365735
ENSP00000307786	ENSP00000309565
ENSP00000307786	ENSP00000317159
ENSP00000307786	ENSP00000317780
ENSP00000307786	ENSP00000326579
ENSP00000307786	ENSP00000326806
ENSP00000307786	ENSP00000329623
ENSP00000307786	ENSP00000331201
ENSP00000307786	ENSP00000332887
ENSP00000307786	ENSP00000338345
ENSP00000307786	ENSP00000341637
ENSP00000307786	ENSP00000341838
ENSP00000307786	ENSP00000347919
ENSP00000307786	ENSP00000350512
ENSP00000307786	ENSP00000353059
ENSP00000307786	ENSP00000354499
ENSP00000307786	ENSP00000354554
ENSP00000307786	ENSP00000354876
ENSP00000307786	ENSP00000354982
ENSP00000307786	ENSP00000355572
ENSP00000307786	ENSP00000357980
ENSP00000307786	ENSP00000359531
ENSP00000307786	ENSP00000361686
ENSP00000307786	ENSP00000364289
ENSP00000307786	ENSP00000365243
ENSP00000307786	ENSP00000367934
ENSP00000307786	ENSP00000377969
ENSP00000307786	ENSP00000387262
ENSP00000307821	ENSP00000311665
ENSP00000307821	ENSP00000321239
ENSP00000307833	ENSP00000350512
ENSP00000307850	ENSP00000317177
ENSP00000307850	ENSP00000341243
ENSP00000307850	ENSP00000342793
ENSP00000307850	ENSP00000347345
ENSP00000307850	ENSP00000347948
ENSP00000307850	ENSP00000357907
ENSP00000307850	ENSP00000358478
ENSP00000307850	ENSP00000359290
ENSP00000307850	ENSP00000366534

ENSP00000307850	ENSP00000381891
ENSP00000307853	ENSP00000308944
ENSP00000307853	ENSP00000309572
ENSP00000307853	ENSP00000310520
ENSP00000307853	ENSP00000310596
ENSP00000307853	ENSP00000313171
ENSP00000307853	ENSP00000317039
ENSP00000307853	ENSP00000317636
ENSP00000307853	ENSP00000321636
ENSP00000307853	ENSP00000322570
ENSP00000307853	ENSP00000323439
ENSP00000307853	ENSP00000323511
ENSP00000307853	ENSP00000325863
ENSP00000307853	ENSP00000328940
ENSP00000307853	ENSP00000336606
ENSP00000307853	ENSP00000337353
ENSP00000307853	ENSP00000339897
ENSP00000307853	ENSP00000340305
ENSP00000307853	ENSP00000340879
ENSP00000307853	ENSP00000341382
ENSP00000307853	ENSP00000341543
ENSP00000307853	ENSP00000343392
ENSP00000307853	ENSP00000343741
ENSP00000307853	ENSP00000343745
ENSP00000307853	ENSP00000345917
ENSP00000307853	ENSP00000347232
ENSP00000307853	ENSP00000347978
ENSP00000307853	ENSP00000348020
ENSP00000307853	ENSP00000349393
ENSP00000307853	ENSP00000351284
ENSP00000307853	ENSP00000351697
ENSP00000307853	ENSP00000352257
ENSP00000307853	ENSP00000353332
ENSP00000307853	ENSP00000353793
ENSP00000307853	ENSP00000354522
ENSP00000307853	ENSP00000354720
ENSP00000307853	ENSP00000354957
ENSP00000307853	ENSP00000358019
ENSP00000307853	ENSP00000358478
ENSP00000307853	ENSP00000360310
ENSP00000307853	ENSP00000360621
ENSP00000307853	ENSP00000360803
ENSP00000307853	ENSP00000361310
ENSP00000307853	ENSP00000364126
ENSP00000307853	ENSP00000364270
ENSP00000307853	ENSP00000365588

ENSP00000307853	ENSP00000367787
ENSP00000307853	ENSP00000368349
ENSP00000307853	ENSP00000368438
ENSP00000307853	ENSP00000369335
ENSP00000307853	ENSP00000369411
ENSP00000307853	ENSP00000370151
ENSP00000307853	ENSP00000370376
ENSP00000307853	ENSP00000371475
ENSP00000307853	ENSP00000372023
ENSP00000307853	ENSP00000373060
ENSP00000307853	ENSP00000375809
ENSP00000307853	ENSP00000381657
ENSP00000307853	ENSP00000384048
ENSP00000307853	ENSP00000387429
ENSP00000307853	ENSP00000394649
ENSP00000307853	ENSP00000396484
ENSP00000307853	ENSP00000396704
ENSP00000307853	ENSP00000402084
ENSP00000307853	ENSP00000404318
ENSP00000307853	ENSP00000406490
ENSP00000307853	ENSP00000406868
ENSP00000307853	ENSP00000409167
ENSP00000307853	ENSP00000415212
ENSP00000307853	ENSP00000416511
ENSP00000307858	ENSP00000353622
ENSP00000307863	ENSP00000308533
ENSP00000307863	ENSP00000309166
ENSP00000307863	ENSP00000310448
ENSP00000307863	ENSP00000310596
ENSP00000307863	ENSP00000310723
ENSP00000307863	ENSP00000311135
ENSP00000307863	ENSP00000313829
ENSP00000307863	ENSP00000315379
ENSP00000307863	ENSP00000315674
ENSP00000307863	ENSP00000315859
ENSP00000307863	ENSP00000316879
ENSP00000307863	ENSP00000318195
ENSP00000307863	ENSP00000318861
ENSP00000307863	ENSP00000319341
ENSP00000307863	ENSP00000321711
ENSP00000307863	ENSP00000322016
ENSP00000307863	ENSP00000325677
ENSP00000307863	ENSP00000326806
ENSP00000307863	ENSP00000329466
ENSP00000307863	ENSP00000331817
ENSP00000307863	ENSP00000331879

ENSP00000307863	ENSP00000334148
ENSP00000307863	ENSP00000335321
ENSP00000307863	ENSP00000337405
ENSP00000307863	ENSP00000339090
ENSP00000307863	ENSP00000340281
ENSP00000307863	ENSP00000342374
ENSP00000307863	ENSP00000344241
ENSP00000307863	ENSP00000345773
ENSP00000307863	ENSP00000347592
ENSP00000307863	ENSP00000348010
ENSP00000307863	ENSP00000349748
ENSP00000307863	ENSP00000351947
ENSP00000307863	ENSP00000352308
ENSP00000307863	ENSP00000352678
ENSP00000307863	ENSP00000352738
ENSP00000307863	ENSP00000354951
ENSP00000307863	ENSP00000357304
ENSP00000307863	ENSP00000357631
ENSP00000307863	ENSP00000358374
ENSP00000307863	ENSP00000359518
ENSP00000307863	ENSP00000359988
ENSP00000307863	ENSP00000360532
ENSP00000307863	ENSP00000361048
ENSP00000307863	ENSP00000361577
ENSP00000307863	ENSP00000362900
ENSP00000307863	ENSP00000362936
ENSP00000307863	ENSP00000363976
ENSP00000307863	ENSP00000364289
ENSP00000307863	ENSP00000365439
ENSP00000307863	ENSP00000366557
ENSP00000307863	ENSP00000366607
ENSP00000307863	ENSP00000366829
ENSP00000307863	ENSP00000366843
ENSP00000307863	ENSP00000369218
ENSP00000307863	ENSP00000370589
ENSP00000307863	ENSP00000371888
ENSP00000307863	ENSP00000376973
ENSP00000307863	ENSP00000377262
ENSP00000307863	ENSP00000379607
ENSP00000307863	ENSP00000379888
ENSP00000307863	ENSP00000386759
ENSP00000307863	ENSP00000387170
ENSP00000307887	ENSP00000356191
ENSP00000307889	ENSP00000308897
ENSP00000307889	ENSP00000309334
ENSP00000307889	ENSP00000309830

ENSP00000307889	ENSP00000311028
ENSP00000307889	ENSP00000311430
ENSP00000307889	ENSP00000314311
ENSP00000307889	ENSP00000314348
ENSP00000307889	ENSP00000317376
ENSP00000307889	ENSP00000318195
ENSP00000307889	ENSP00000318646
ENSP00000307889	ENSP00000322419
ENSP00000307889	ENSP00000327821
ENSP00000307889	ENSP00000329967
ENSP00000307889	ENSP00000337839
ENSP00000307889	ENSP00000338862
ENSP00000307889	ENSP00000339095
ENSP00000307889	ENSP00000339795
ENSP00000307889	ENSP00000345096
ENSP00000307889	ENSP00000345156
ENSP00000307889	ENSP00000346015
ENSP00000307889	ENSP00000346022
ENSP00000307889	ENSP00000346027
ENSP00000307889	ENSP00000346037
ENSP00000307889	ENSP00000346050
ENSP00000307889	ENSP00000346088
ENSP00000307889	ENSP00000346453
ENSP00000307889	ENSP00000346725
ENSP00000307889	ENSP00000347087
ENSP00000307889	ENSP00000352021
ENSP00000307889	ENSP00000352738
ENSP00000307889	ENSP00000354040
ENSP00000307889	ENSP00000354525
ENSP00000307889	ENSP00000354673
ENSP00000307889	ENSP00000354739
ENSP00000307889	ENSP00000359345
ENSP00000307889	ENSP00000360532
ENSP00000307889	ENSP00000361076
ENSP00000307889	ENSP00000362334
ENSP00000307889	ENSP00000362744
ENSP00000307889	ENSP00000362900
ENSP00000307889	ENSP00000363018
ENSP00000307889	ENSP00000363384
ENSP00000307889	ENSP00000363559
ENSP00000307889	ENSP00000363676
ENSP00000307889	ENSP00000364119
ENSP00000307889	ENSP00000364320
ENSP00000307889	ENSP00000369757
ENSP00000307889	ENSP00000370083
ENSP00000307889	ENSP00000370258

ENSP00000307889	ENSP00000370589
ENSP00000307889	ENSP00000373404
ENSP00000307889	ENSP00000375730
ENSP00000307889	ENSP00000377640
ENSP00000307889	ENSP00000377865
ENSP00000307889	ENSP00000378160
ENSP00000307889	ENSP00000379607
ENSP00000307889	ENSP00000379888
ENSP00000307889	ENSP00000380156
ENSP00000307889	ENSP00000386717
ENSP00000307889	ENSP00000389103
ENSP00000307889	ENSP00000391723
ENSP00000307889	ENSP00000392859
ENSP00000307889	ENSP00000393241
ENSP00000307889	ENSP00000393393
ENSP00000307889	ENSP00000402338
ENSP00000307889	ENSP00000403175
ENSP00000307889	ENSP00000412583
ENSP00000307889	ENSP00000414321
ENSP00000307889	ENSP00000416110
ENSP00000307900	ENSP00000312664
ENSP00000307900	ENSP00000312870
ENSP00000307900	ENSP00000317872
ENSP00000307900	ENSP00000340019
ENSP00000307900	ENSP00000345681
ENSP00000307900	ENSP00000352706
ENSP00000307900	ENSP00000354313
ENSP00000307900	ENSP00000354813
ENSP00000307900	ENSP00000355890
ENSP00000307900	ENSP00000361699
ENSP00000307900	ENSP00000361990
ENSP00000307900	ENSP00000364815
ENSP00000307900	ENSP00000371546
ENSP00000307900	ENSP00000373477
ENSP00000307900	ENSP00000379065
ENSP00000307900	ENSP00000384434
ENSP00000307900	ENSP00000385083
ENSP00000307900	ENSP00000387426
ENSP00000307900	ENSP00000396068
ENSP00000307900	ENSP00000401121
ENSP00000307900	ENSP00000403359
ENSP00000307900	ENSP00000415316
ENSP00000307939	ENSP00000310668
ENSP00000307939	ENSP00000352676
ENSP00000308137	ENSP00000410088
ENSP00000308179	ENSP00000310590

ENSP00000308179	ENSP00000311135
ENSP00000308179	ENSP00000314193
ENSP00000308179	ENSP00000315674
ENSP00000308179	ENSP00000321449
ENSP00000308179	ENSP00000326981
ENSP00000308179	ENSP00000327179
ENSP00000308179	ENSP00000328854
ENSP00000308179	ENSP00000329466
ENSP00000308179	ENSP00000339145
ENSP00000308179	ENSP00000348848
ENSP00000308179	ENSP00000353246
ENSP00000308179	ENSP00000354040
ENSP00000308179	ENSP00000355471
ENSP00000308179	ENSP00000355541
ENSP00000308179	ENSP00000360031
ENSP00000308179	ENSP00000363642
ENSP00000308179	ENSP00000363746
ENSP00000308179	ENSP00000366557
ENSP00000308179	ENSP00000370258
ENSP00000308179	ENSP00000370589
ENSP00000308179	ENSP00000371101
ENSP00000308179	ENSP00000372695
ENSP00000308179	ENSP00000379888
ENSP00000308179	ENSP00000384302
ENSP00000308179	ENSP00000396937
ENSP00000308179	ENSP00000399454
ENSP00000308179	ENSP00000402869
ENSP00000308179	ENSP00000405614
ENSP00000308179	ENSP00000410396
ENSP00000308193	ENSP00000337623
ENSP00000308236	ENSP00000348054
ENSP00000308236	ENSP00000366032
ENSP00000308236	ENSP00000370984
ENSP00000308236	ENSP00000384109
ENSP00000308236	ENSP00000419475
ENSP00000308268	ENSP00000315693
ENSP00000308268	ENSP00000318351
ENSP00000308268	ENSP00000343657
ENSP00000308268	ENSP00000360268
ENSP00000308268	ENSP00000367079
ENSP00000308268	ENSP00000370377
ENSP00000308268	ENSP00000384015
ENSP00000308275	ENSP00000308897
ENSP00000308275	ENSP00000312311
ENSP00000308275	ENSP00000315017
ENSP00000308275	ENSP00000324122

ENSP00000308275	ENSP00000333401
ENSP00000308275	ENSP00000347836
ENSP00000308275	ENSP00000350491
ENSP00000308275	ENSP00000351015
ENSP00000308275	ENSP00000360498
ENSP00000308275	ENSP00000370930
ENSP00000308275	ENSP00000377486
ENSP00000308275	ENSP00000379607
ENSP00000308275	ENSP00000417602
ENSP00000308332	ENSP00000324122
ENSP00000308332	ENSP00000327179
ENSP00000308332	ENSP00000331817
ENSP00000308332	ENSP00000355890
ENSP00000308332	ENSP00000356146
ENSP00000308332	ENSP00000358563
ENSP00000308332	ENSP00000358812
ENSP00000308332	ENSP00000361982
ENSP00000308332	ENSP00000366030
ENSP00000308332	ENSP00000370589
ENSP00000308332	ENSP00000402338
ENSP00000308334	ENSP00000358737
ENSP00000308334	ENSP00000367408
ENSP00000308339	ENSP00000346015
ENSP00000308339	ENSP00000347329
ENSP00000308339	ENSP00000355296
ENSP00000308339	ENSP00000356530
ENSP00000308339	ENSP00000401445
ENSP00000308369	ENSP00000334148
ENSP00000308369	ENSP00000341551
ENSP00000308369	ENSP00000357631
ENSP00000308369	ENSP00000360286
ENSP00000308369	ENSP00000362820
ENSP00000308383	ENSP00000363708
ENSP00000308466	ENSP00000325377
ENSP00000308466	ENSP00000352821
ENSP00000308466	ENSP00000367024
ENSP00000308466	ENSP00000380156
ENSP00000308466	ENSP00000390475
ENSP00000308466	ENSP00000391723
ENSP00000308466	ENSP00000392270
ENSP00000308496	ENSP00000314615
ENSP00000308496	ENSP00000327309
ENSP00000308496	ENSP00000330320
ENSP00000308496	ENSP00000336724
ENSP00000308496	ENSP00000344055
ENSP00000308496	ENSP00000355231

ENSP00000308496	ENSP00000356146
ENSP00000308496	ENSP00000360183
ENSP00000308496	ENSP00000377280
ENSP00000308496	ENSP00000377769
ENSP00000308496	ENSP00000379093
ENSP00000308496	ENSP00000389399
ENSP00000308496	ENSP00000397441
ENSP00000308496	ENSP00000401435
ENSP00000308496	ENSP00000402240
ENSP00000308533	ENSP00000311505
ENSP00000308533	ENSP00000311825
ENSP00000308533	ENSP00000315859
ENSP00000308533	ENSP00000342374
ENSP00000308533	ENSP00000356520
ENSP00000308533	ENSP00000358716
ENSP00000308533	ENSP00000361577
ENSP00000308533	ENSP00000362820
ENSP00000308533	ENSP00000362900
ENSP00000308533	ENSP00000370083
ENSP00000308533	ENSP00000370119
ENSP00000308533	ENSP00000400591
ENSP00000308546	ENSP00000309830
ENSP00000308546	ENSP00000310448
ENSP00000308546	ENSP00000310723
ENSP00000308546	ENSP00000311028
ENSP00000308546	ENSP00000312066
ENSP00000308546	ENSP00000312981
ENSP00000308546	ENSP00000314311
ENSP00000308546	ENSP00000315379
ENSP00000308546	ENSP00000318262
ENSP00000308546	ENSP00000318861
ENSP00000308546	ENSP00000324122
ENSP00000308546	ENSP00000328487
ENSP00000308546	ENSP00000328773
ENSP00000308546	ENSP00000331817
ENSP00000308546	ENSP00000341885
ENSP00000308546	ENSP00000342181
ENSP00000308546	ENSP00000342374
ENSP00000308546	ENSP00000343701
ENSP00000308546	ENSP00000346566
ENSP00000308546	ENSP00000347592
ENSP00000308546	ENSP00000348577
ENSP00000308546	ENSP00000352308
ENSP00000308546	ENSP00000354346
ENSP00000308546	ENSP00000354742
ENSP00000308546	ENSP00000355011

ENSP00000308546	ENSP00000355587
ENSP00000308546	ENSP00000355759
ENSP00000308546	ENSP00000356405
ENSP00000308546	ENSP00000356520
ENSP00000308546	ENSP00000358417
ENSP00000308546	ENSP00000361010
ENSP00000308546	ENSP00000361433
ENSP00000308546	ENSP00000362135
ENSP00000308546	ENSP00000362744
ENSP00000308546	ENSP00000362820
ENSP00000308546	ENSP00000363313
ENSP00000308546	ENSP00000363745
ENSP00000308546	ENSP00000365439
ENSP00000308546	ENSP00000369411
ENSP00000308546	ENSP00000370589
ENSP00000308546	ENSP00000371634
ENSP00000308546	ENSP00000375863
ENSP00000308546	ENSP00000376309
ENSP00000308546	ENSP00000377969
ENSP00000308546	ENSP00000404121
ENSP00000308546	ENSP00000412566
ENSP00000308546	ENSP00000414006
ENSP00000308610	ENSP00000310621
ENSP00000308610	ENSP00000311684
ENSP00000308610	ENSP00000312370
ENSP00000308610	ENSP00000317159
ENSP00000308610	ENSP00000327070
ENSP00000308610	ENSP00000335304
ENSP00000308610	ENSP00000336927
ENSP00000308610	ENSP00000353452
ENSP00000308610	ENSP00000379933
ENSP00000308610	ENSP00000387262
ENSP00000308610	ENSP00000409493
ENSP00000308620	ENSP00000341551
ENSP00000308620	ENSP00000343701
ENSP00000308622	ENSP00000310040
ENSP00000308622	ENSP00000310668
ENSP00000308622	ENSP00000316032
ENSP00000308622	ENSP00000323780
ENSP00000308622	ENSP00000327197
ENSP00000308622	ENSP00000336712
ENSP00000308622	ENSP00000337393
ENSP00000308622	ENSP00000346566
ENSP00000308622	ENSP00000352400
ENSP00000308622	ENSP00000353741
ENSP00000308622	ENSP00000354125

ENSP00000308622	ENSP00000360286
ENSP00000308622	ENSP00000363046
ENSP00000308622	ENSP00000370258
ENSP00000308622	ENSP00000371155
ENSP00000308622	ENSP00000377655
ENSP00000308622	ENSP00000382779
ENSP00000308622	ENSP00000394791
ENSP00000308622	ENSP00000402527
ENSP00000308622	ENSP00000417229
ENSP00000308699	ENSP00000312778
ENSP00000308699	ENSP00000313171
ENSP00000308699	ENSP00000322180
ENSP00000308699	ENSP00000322570
ENSP00000308699	ENSP00000326514
ENSP00000308699	ENSP00000329558
ENSP00000308699	ENSP00000335357
ENSP00000308699	ENSP00000341382
ENSP00000308699	ENSP00000341680
ENSP00000308699	ENSP00000342381
ENSP00000308699	ENSP00000343081
ENSP00000308699	ENSP00000343741
ENSP00000308699	ENSP00000348551
ENSP00000308699	ENSP00000348554
ENSP00000308699	ENSP00000348722
ENSP00000308699	ENSP00000353793
ENSP00000308699	ENSP00000354720
ENSP00000308699	ENSP00000354916
ENSP00000308699	ENSP00000358423
ENSP00000308699	ENSP00000361990
ENSP00000308699	ENSP00000368017
ENSP00000308699	ENSP00000369411
ENSP00000308699	ENSP00000417963
ENSP00000308741	ENSP00000314420
ENSP00000308741	ENSP00000347168
ENSP00000308741	ENSP00000350767
ENSP00000308741	ENSP00000355759
ENSP00000308741	ENSP00000366974
ENSP00000308741	ENSP00000367034
ENSP00000308741	ENSP00000404658
ENSP00000308741	ENSP00000417281
ENSP00000308750	ENSP00000343535
ENSP00000308750	ENSP00000349892
ENSP00000308750	ENSP00000355759
ENSP00000308750	ENSP00000356817
ENSP00000308750	ENSP00000361287
ENSP00000308750	ENSP00000369380

ENSP00000308750	ENSP00000373566
ENSP00000308750	ENSP00000396439
ENSP00000308750	ENSP00000418734
ENSP00000308750	ENSP00000419494
ENSP00000308759	ENSP00000323720
ENSP00000308759	ENSP00000391774
ENSP00000308759	ENSP00000420588
ENSP00000308774	ENSP00000333568
ENSP00000308774	ENSP00000339191
ENSP00000308774	ENSP00000341189
ENSP00000308774	ENSP00000341208
ENSP00000308774	ENSP00000341551
ENSP00000308774	ENSP00000354119
ENSP00000308774	ENSP00000362608
ENSP00000308774	ENSP00000365435
ENSP00000308774	ENSP00000365439
ENSP00000308897	ENSP00000309334
ENSP00000308897	ENSP00000309830
ENSP00000308897	ENSP00000311028
ENSP00000308897	ENSP00000312735
ENSP00000308897	ENSP00000315017
ENSP00000308897	ENSP00000317376
ENSP00000308897	ENSP00000318646
ENSP00000308897	ENSP00000322419
ENSP00000308897	ENSP00000323856
ENSP00000308897	ENSP00000326981
ENSP00000308897	ENSP00000327821
ENSP00000308897	ENSP00000331849
ENSP00000308897	ENSP00000333837
ENSP00000308897	ENSP00000339095
ENSP00000308897	ENSP00000339795
ENSP00000308897	ENSP00000339845
ENSP00000308897	ENSP00000341082
ENSP00000308897	ENSP00000341885
ENSP00000308897	ENSP00000343392
ENSP00000308897	ENSP00000343745
ENSP00000308897	ENSP00000345156
ENSP00000308897	ENSP00000346015
ENSP00000308897	ENSP00000346022
ENSP00000308897	ENSP00000346027
ENSP00000308897	ENSP00000346037
ENSP00000308897	ENSP00000346050
ENSP00000308897	ENSP00000346088
ENSP00000308897	ENSP00000354525
ENSP00000308897	ENSP00000356811
ENSP00000308897	ENSP00000359345

ENSP00000308897	ENSP00000361076
ENSP00000308897	ENSP00000362135
ENSP00000308897	ENSP00000362744
ENSP00000308897	ENSP00000363018
ENSP00000308897	ENSP00000363676
ENSP00000308897	ENSP00000364320
ENSP00000308897	ENSP00000368565
ENSP00000308897	ENSP00000369757
ENSP00000308897	ENSP00000370930
ENSP00000308897	ENSP00000373404
ENSP00000308897	ENSP00000375730
ENSP00000308897	ENSP00000377486
ENSP00000308897	ENSP00000377640
ENSP00000308897	ENSP00000377865
ENSP00000308897	ENSP00000378160
ENSP00000308897	ENSP00000379607
ENSP00000308897	ENSP00000379888
ENSP00000308897	ENSP00000380156
ENSP00000308897	ENSP00000386717
ENSP00000308897	ENSP00000388330
ENSP00000308897	ENSP00000389103
ENSP00000308897	ENSP00000393393
ENSP00000308897	ENSP00000402338
ENSP00000308897	ENSP00000403536
ENSP00000308897	ENSP00000414321
ENSP00000308897	ENSP00000417602
ENSP00000308925	ENSP00000370737
ENSP00000308944	ENSP00000309365
ENSP00000308944	ENSP00000309572
ENSP00000308944	ENSP00000310520
ENSP00000308944	ENSP00000310596
ENSP00000308944	ENSP00000311873
ENSP00000308944	ENSP00000313171
ENSP00000308944	ENSP00000314949
ENSP00000308944	ENSP00000315700
ENSP00000308944	ENSP00000317636
ENSP00000308944	ENSP00000321636
ENSP00000308944	ENSP00000323439
ENSP00000308944	ENSP00000325677
ENSP00000308944	ENSP00000325863
ENSP00000308944	ENSP00000327716
ENSP00000308944	ENSP00000334474
ENSP00000308944	ENSP00000334675
ENSP00000308944	ENSP00000336606
ENSP00000308944	ENSP00000339145
ENSP00000308944	ENSP00000339933

ENSP00000308944	ENSP00000340879
ENSP00000308944	ENSP00000342181
ENSP00000308944	ENSP00000343392
ENSP00000308944	ENSP00000343535
ENSP00000308944	ENSP00000343741
ENSP00000308944	ENSP00000346437
ENSP00000308944	ENSP00000347232
ENSP00000308944	ENSP00000347495
ENSP00000308944	ENSP00000347978
ENSP00000308944	ENSP00000348089
ENSP00000308944	ENSP00000350708
ENSP00000308944	ENSP00000351284
ENSP00000308944	ENSP00000351697
ENSP00000308944	ENSP00000353793
ENSP00000308944	ENSP00000354201
ENSP00000308944	ENSP00000355077
ENSP00000308944	ENSP00000355185
ENSP00000308944	ENSP00000357887
ENSP00000308944	ENSP00000358576
ENSP00000308944	ENSP00000360532
ENSP00000308944	ENSP00000361310
ENSP00000308944	ENSP00000364270
ENSP00000308944	ENSP00000367527
ENSP00000308944	ENSP00000368438
ENSP00000308944	ENSP00000368646
ENSP00000308944	ENSP00000368801
ENSP00000308944	ENSP00000369335
ENSP00000308944	ENSP00000370150
ENSP00000308944	ENSP00000370151
ENSP00000308944	ENSP00000370408
ENSP00000308944	ENSP00000371475
ENSP00000308944	ENSP00000372023
ENSP00000308944	ENSP00000374280
ENSP00000308944	ENSP00000380460
ENSP00000308944	ENSP00000381031
ENSP00000308944	ENSP00000381657
ENSP00000308944	ENSP00000402515
ENSP00000308957	ENSP00000350874
ENSP00000309031	ENSP00000331487
ENSP00000309031	ENSP00000346130
ENSP00000309031	ENSP00000346986
ENSP00000309031	ENSP00000360916
ENSP00000309031	ENSP00000363338
ENSP00000309031	ENSP00000379457
ENSP00000309031	ENSP00000386722
ENSP00000309052	ENSP00000347301

ENSP00000309103	ENSP00000313059
ENSP00000309103	ENSP00000322957
ENSP00000309103	ENSP00000329623
ENSP00000309103	ENSP00000338345
ENSP00000309103	ENSP00000347979
ENSP00000309103	ENSP00000352425
ENSP00000309103	ENSP00000358022
ENSP00000309103	ENSP00000362608
ENSP00000309103	ENSP00000363591
ENSP00000309103	ENSP00000370003
ENSP00000309103	ENSP00000381031
ENSP00000309103	ENSP00000389277
ENSP00000309117	ENSP00000317891
ENSP00000309117	ENSP00000317992
ENSP00000309117	ENSP00000327704
ENSP00000309117	ENSP00000354560
ENSP00000309117	ENSP00000354951
ENSP00000309117	ENSP00000359444
ENSP00000309117	ENSP00000360500
ENSP00000309117	ENSP00000364847
ENSP00000309117	ENSP00000365175
ENSP00000309117	ENSP00000366843
ENSP00000309117	ENSP00000387477
ENSP00000309117	ENSP00000397435
ENSP00000309132	ENSP00000329623
ENSP00000309141	ENSP00000315791
ENSP00000309141	ENSP00000320885
ENSP00000309141	ENSP00000331487
ENSP00000309141	ENSP00000333836
ENSP00000309141	ENSP00000348010
ENSP00000309141	ENSP00000357998
ENSP00000309141	ENSP00000366395
ENSP00000309141	ENSP00000379457
ENSP00000309141	ENSP00000383719
ENSP00000309141	ENSP00000387347
ENSP00000309142	ENSP00000310596
ENSP00000309142	ENSP00000313171
ENSP00000309142	ENSP00000314949
ENSP00000309142	ENSP00000319104
ENSP00000309142	ENSP00000341743
ENSP00000309142	ENSP00000351284
ENSP00000309142	ENSP00000354673
ENSP00000309142	ENSP00000356405
ENSP00000309142	ENSP00000360515
ENSP00000309142	ENSP00000368438
ENSP00000309142	ENSP00000369965

ENSP00000309142	ENSP00000372023
ENSP00000309163	ENSP00000310003
ENSP00000309163	ENSP00000310040
ENSP00000309163	ENSP00000314499
ENSP00000309163	ENSP00000328079
ENSP00000309163	ENSP00000329419
ENSP00000309163	ENSP00000343552
ENSP00000309163	ENSP00000356405
ENSP00000309163	ENSP00000360183
ENSP00000309163	ENSP00000360270
ENSP00000309163	ENSP00000361845
ENSP00000309163	ENSP00000390783
ENSP00000309163	ENSP00000397441
ENSP00000309166	ENSP00000324122
ENSP00000309166	ENSP00000325376
ENSP00000309166	ENSP00000332681
ENSP00000309166	ENSP00000356520
ENSP00000309166	ENSP00000360532
ENSP00000309166	ENSP00000365439
ENSP00000309166	ENSP00000366607
ENSP00000309166	ENSP00000366829
ENSP00000309166	ENSP00000367440
ENSP00000309166	ENSP00000375863
ENSP00000309166	ENSP00000379607
ENSP00000309175	ENSP00000313691
ENSP00000309189	ENSP00000389427
ENSP00000309198	ENSP00000309474
ENSP00000309198	ENSP00000310129
ENSP00000309198	ENSP00000348442
ENSP00000309198	ENSP00000362334
ENSP00000309198	ENSP00000366135
ENSP00000309198	ENSP00000379217
ENSP00000309198	ENSP00000418734
ENSP00000309259	ENSP00000356530
ENSP00000309259	ENSP00000361287
ENSP00000309259	ENSP00000383901
ENSP00000309259	ENSP00000401445
ENSP00000309262	ENSP00000313490
ENSP00000309262	ENSP00000337194
ENSP00000309262	ENSP00000386165
ENSP00000309262	ENSP00000413520
ENSP00000309334	ENSP00000309830
ENSP00000309334	ENSP00000311028
ENSP00000309334	ENSP00000311430
ENSP00000309334	ENSP00000314348
ENSP00000309334	ENSP00000317376

ENSP00000309334	ENSP00000317992
ENSP00000309334	ENSP00000318646
ENSP00000309334	ENSP00000322396
ENSP00000309334	ENSP00000322419
ENSP00000309334	ENSP00000323858
ENSP00000309334	ENSP00000326981
ENSP00000309334	ENSP00000328690
ENSP00000309334	ENSP00000329466
ENSP00000309334	ENSP00000337839
ENSP00000309334	ENSP00000338862
ENSP00000309334	ENSP00000339095
ENSP00000309334	ENSP00000339795
ENSP00000309334	ENSP00000345096
ENSP00000309334	ENSP00000345156
ENSP00000309334	ENSP00000346015
ENSP00000309334	ENSP00000346022
ENSP00000309334	ENSP00000346027
ENSP00000309334	ENSP00000346037
ENSP00000309334	ENSP00000346050
ENSP00000309334	ENSP00000346088
ENSP00000309334	ENSP00000346725
ENSP00000309334	ENSP00000347087
ENSP00000309334	ENSP00000354040
ENSP00000309334	ENSP00000354125
ENSP00000309334	ENSP00000354525
ENSP00000309334	ENSP00000354739
ENSP00000309334	ENSP00000355899
ENSP00000309334	ENSP00000358812
ENSP00000309334	ENSP00000359345
ENSP00000309334	ENSP00000360412
ENSP00000309334	ENSP00000360922
ENSP00000309334	ENSP00000361076
ENSP00000309334	ENSP00000362744
ENSP00000309334	ENSP00000363018
ENSP00000309334	ENSP00000363676
ENSP00000309334	ENSP00000364320
ENSP00000309334	ENSP00000366557
ENSP00000309334	ENSP00000368565
ENSP00000309334	ENSP00000369757
ENSP00000309334	ENSP00000370589
ENSP00000309334	ENSP00000373404
ENSP00000309334	ENSP00000375730
ENSP00000309334	ENSP00000377640
ENSP00000309334	ENSP00000377865
ENSP00000309334	ENSP00000378160
ENSP00000309334	ENSP00000379607

ENSP00000309334	ENSP00000379888
ENSP00000309334	ENSP00000380156
ENSP00000309334	ENSP00000380982
ENSP00000309334	ENSP00000386717
ENSP00000309334	ENSP00000389103
ENSP00000309334	ENSP00000392859
ENSP00000309334	ENSP00000393241
ENSP00000309334	ENSP00000393393
ENSP00000309334	ENSP00000402338
ENSP00000309334	ENSP00000403175
ENSP00000309334	ENSP00000412583
ENSP00000309334	ENSP00000414321
ENSP00000309334	ENSP00000416110
ENSP00000309334	ENSP00000417229
ENSP00000309338	ENSP00000340278
ENSP00000309338	ENSP00000376188
ENSP00000309365	ENSP00000318195
ENSP00000309365	ENSP00000347836
ENSP00000309365	ENSP00000352257
ENSP00000309365	ENSP00000355759
ENSP00000309365	ENSP00000369042
ENSP00000309430	ENSP00000334052
ENSP00000309430	ENSP00000347206
ENSP00000309430	ENSP00000351284
ENSP00000309430	ENSP00000359848
ENSP00000309430	ENSP00000374484
ENSP00000309430	ENSP00000397441
ENSP00000309433	ENSP00000311135
ENSP00000309433	ENSP00000312735
ENSP00000309433	ENSP00000314348
ENSP00000309433	ENSP00000314949
ENSP00000309433	ENSP00000322419
ENSP00000309433	ENSP00000329858
ENSP00000309433	ENSP00000331879
ENSP00000309433	ENSP00000333934
ENSP00000309433	ENSP00000339095
ENSP00000309433	ENSP00000346037
ENSP00000309433	ENSP00000346453
ENSP00000309433	ENSP00000346725
ENSP00000309433	ENSP00000353564
ENSP00000309433	ENSP00000353741
ENSP00000309433	ENSP00000354040
ENSP00000309433	ENSP00000354125
ENSP00000309433	ENSP00000356476
ENSP00000309433	ENSP00000358400
ENSP00000309433	ENSP00000358563

ENSP00000309433	ENSP00000358812
ENSP00000309433	ENSP00000359345
ENSP00000309433	ENSP00000360031
ENSP00000309433	ENSP00000360412
ENSP00000309433	ENSP00000361982
ENSP00000309433	ENSP00000362688
ENSP00000309433	ENSP00000366519
ENSP00000309433	ENSP00000366557
ENSP00000309433	ENSP00000368612
ENSP00000309433	ENSP00000369965
ENSP00000309433	ENSP00000374219
ENSP00000309433	ENSP00000377040
ENSP00000309433	ENSP00000377865
ENSP00000309433	ENSP00000377944
ENSP00000309433	ENSP00000382392
ENSP00000309433	ENSP00000389103
ENSP00000309433	ENSP00000403817
ENSP00000309433	ENSP00000417229
ENSP00000309433	ENSP00000419740
ENSP00000309457	ENSP00000310521
ENSP00000309457	ENSP00000313420
ENSP00000309457	ENSP00000326534
ENSP00000309457	ENSP00000327650
ENSP00000309457	ENSP00000331201
ENSP00000309457	ENSP00000339122
ENSP00000309457	ENSP00000344055
ENSP00000309457	ENSP00000347733
ENSP00000309457	ENSP00000353904
ENSP00000309457	ENSP00000354558
ENSP00000309457	ENSP00000359000
ENSP00000309457	ENSP00000360116
ENSP00000309457	ENSP00000360183
ENSP00000309457	ENSP00000360252
ENSP00000309457	ENSP00000362399
ENSP00000309457	ENSP00000365943
ENSP00000309457	ENSP00000369810
ENSP00000309457	ENSP00000390783
ENSP00000309457	ENSP00000402515
ENSP00000309463	ENSP00000315693
ENSP00000309463	ENSP00000319531
ENSP00000309463	ENSP00000356405
ENSP00000309463	ENSP00000366974
ENSP00000309474	ENSP00000309871
ENSP00000309474	ENSP00000309968
ENSP00000309474	ENSP00000310129
ENSP00000309474	ENSP00000310561

ENSP00000309474	ENSP00000311665
ENSP00000309474	ENSP00000314004
ENSP00000309474	ENSP00000314129
ENSP00000309474	ENSP00000320949
ENSP00000309474	ENSP00000321711
ENSP00000309474	ENSP00000325919
ENSP00000309474	ENSP00000329466
ENSP00000309474	ENSP00000335357
ENSP00000309474	ENSP00000340596
ENSP00000309474	ENSP00000341243
ENSP00000309474	ENSP00000347997
ENSP00000309474	ENSP00000348442
ENSP00000309474	ENSP00000350708
ENSP00000309474	ENSP00000351947
ENSP00000309474	ENSP00000354883
ENSP00000309474	ENSP00000356438
ENSP00000309474	ENSP00000357980
ENSP00000309474	ENSP00000360532
ENSP00000309474	ENSP00000361287
ENSP00000309474	ENSP00000361990
ENSP00000309474	ENSP00000362334
ENSP00000309474	ENSP00000364204
ENSP00000309474	ENSP00000369531
ENSP00000309474	ENSP00000370222
ENSP00000309474	ENSP00000370408
ENSP00000309474	ENSP00000371341
ENSP00000309474	ENSP00000381045
ENSP00000309474	ENSP00000381698
ENSP00000309474	ENSP00000384211
ENSP00000309474	ENSP00000386212
ENSP00000309474	ENSP00000396068
ENSP00000309474	ENSP00000396937
ENSP00000309487	ENSP00000374234
ENSP00000309515	ENSP00000350249
ENSP00000309555	ENSP00000312649
ENSP00000309555	ENSP00000316114
ENSP00000309555	ENSP00000322957
ENSP00000309555	ENSP00000325326
ENSP00000309555	ENSP00000326767
ENSP00000309555	ENSP00000329357
ENSP00000309555	ENSP00000329466
ENSP00000309555	ENSP00000331152
ENSP00000309555	ENSP00000333275
ENSP00000309555	ENSP00000337088
ENSP00000309555	ENSP00000337340
ENSP00000309555	ENSP00000339527

ENSP00000309555	ENSP00000340896
ENSP00000309555	ENSP00000342136
ENSP00000309555	ENSP00000346886
ENSP00000309555	ENSP00000351490
ENSP00000309555	ENSP00000353622
ENSP00000309555	ENSP00000359290
ENSP00000309555	ENSP00000359837
ENSP00000309555	ENSP00000361009
ENSP00000309555	ENSP00000362592
ENSP00000309555	ENSP00000362824
ENSP00000309555	ENSP00000364895
ENSP00000309555	ENSP00000366854
ENSP00000309555	ENSP00000368686
ENSP00000309555	ENSP00000368887
ENSP00000309555	ENSP00000369695
ENSP00000309555	ENSP00000377218
ENSP00000309555	ENSP00000379607
ENSP00000309555	ENSP00000380129
ENSP00000309555	ENSP00000384675
ENSP00000309555	ENSP00000385903
ENSP00000309555	ENSP00000395535
ENSP00000309555	ENSP00000412566
ENSP00000309555	ENSP00000417132
ENSP00000309561	ENSP00000347948
ENSP00000309561	ENSP00000397435
ENSP00000309565	ENSP00000317159
ENSP00000309565	ENSP00000317780
ENSP00000309565	ENSP00000331019
ENSP00000309565	ENSP00000332887
ENSP00000309565	ENSP00000352219
ENSP00000309565	ENSP00000354499
ENSP00000309565	ENSP00000354554
ENSP00000309565	ENSP00000354876
ENSP00000309565	ENSP00000354982
ENSP00000309565	ENSP00000355572
ENSP00000309565	ENSP00000356953
ENSP00000309565	ENSP00000360782
ENSP00000309565	ENSP00000367934
ENSP00000309565	ENSP00000387262
ENSP00000309572	ENSP00000311873
ENSP00000309572	ENSP00000313171
ENSP00000309572	ENSP00000317636
ENSP00000309572	ENSP00000318195
ENSP00000309572	ENSP00000318977
ENSP00000309572	ENSP00000323439
ENSP00000309572	ENSP00000332444

ENSP00000309572	ENSP00000333873
ENSP00000309572	ENSP00000338573
ENSP00000309572	ENSP00000340766
ENSP00000309572	ENSP00000340879
ENSP00000309572	ENSP00000341382
ENSP00000309572	ENSP00000343741
ENSP00000309572	ENSP00000347232
ENSP00000309572	ENSP00000347978
ENSP00000309572	ENSP00000348708
ENSP00000309572	ENSP00000349640
ENSP00000309572	ENSP00000350249
ENSP00000309572	ENSP00000351284
ENSP00000309572	ENSP00000352257
ENSP00000309572	ENSP00000354522
ENSP00000309572	ENSP00000354558
ENSP00000309572	ENSP00000355261
ENSP00000309572	ENSP00000358478
ENSP00000309572	ENSP00000358563
ENSP00000309572	ENSP00000363021
ENSP00000309572	ENSP00000369411
ENSP00000309572	ENSP00000370151
ENSP00000309572	ENSP00000374213
ENSP00000309572	ENSP00000380504
ENSP00000309572	ENSP00000392028
ENSP00000309572	ENSP00000402515
ENSP00000309572	ENSP00000417281
ENSP00000309595	ENSP00000320885
ENSP00000309595	ENSP00000330408
ENSP00000309595	ENSP00000343479
ENSP00000309595	ENSP00000345216
ENSP00000309595	ENSP00000345341
ENSP00000309595	ENSP00000348081
ENSP00000309595	ENSP00000349298
ENSP00000309595	ENSP00000368646
ENSP00000309595	ENSP00000374455
ENSP00000309595	ENSP00000393596
ENSP00000309606	ENSP00000340698
ENSP00000309606	ENSP00000347592
ENSP00000309606	ENSP00000348708
ENSP00000309606	ENSP00000361232
ENSP00000309606	ENSP00000361433
ENSP00000309606	ENSP00000362592
ENSP00000309606	ENSP00000364813
ENSP00000309606	ENSP00000378503
ENSP00000309606	ENSP00000414006
ENSP00000309606	ENSP00000414634

ENSP00000309610	ENSP00000340019
ENSP00000309610	ENSP00000356476
ENSP00000309610	ENSP00000365891
ENSP00000309610	ENSP00000382269
ENSP00000309629	ENSP00000318884
ENSP00000309629	ENSP00000322170
ENSP00000309629	ENSP00000326238
ENSP00000309629	ENSP00000333950
ENSP00000309629	ENSP00000336740
ENSP00000309629	ENSP00000338127
ENSP00000309629	ENSP00000342793
ENSP00000309629	ENSP00000348163
ENSP00000309629	ENSP00000348215
ENSP00000309629	ENSP00000350249
ENSP00000309629	ENSP00000350757
ENSP00000309629	ENSP00000354742
ENSP00000309629	ENSP00000359100
ENSP00000309629	ENSP00000365891
ENSP00000309629	ENSP00000369816
ENSP00000309629	ENSP00000379607
ENSP00000309629	ENSP00000379933
ENSP00000309629	ENSP00000380450
ENSP00000309629	ENSP00000381031
ENSP00000309681	ENSP00000336790
ENSP00000309710	ENSP00000349393
ENSP00000309710	ENSP00000364956
ENSP00000309710	ENSP00000366936
ENSP00000309710	ENSP00000419952
ENSP00000309790	ENSP00000329357
ENSP00000309830	ENSP00000311430
ENSP00000309830	ENSP00000312042
ENSP00000309830	ENSP00000314129
ENSP00000309830	ENSP00000322419
ENSP00000309830	ENSP00000329466
ENSP00000309830	ENSP00000335657
ENSP00000309830	ENSP00000338573
ENSP00000309830	ENSP00000339795
ENSP00000309830	ENSP00000342374
ENSP00000309830	ENSP00000345156
ENSP00000309830	ENSP00000346015
ENSP00000309830	ENSP00000346022
ENSP00000309830	ENSP00000346027
ENSP00000309830	ENSP00000346037
ENSP00000309830	ENSP00000346088
ENSP00000309830	ENSP00000352021
ENSP00000309830	ENSP00000352514

ENSP00000309830	ENSP00000354040
ENSP00000309830	ENSP00000354525
ENSP00000309830	ENSP00000354739
ENSP00000309830	ENSP00000359345
ENSP00000309830	ENSP00000361076
ENSP00000309830	ENSP00000362153
ENSP00000309830	ENSP00000362306
ENSP00000309830	ENSP00000362688
ENSP00000309830	ENSP00000362993
ENSP00000309830	ENSP00000363018
ENSP00000309830	ENSP00000363384
ENSP00000309830	ENSP00000363676
ENSP00000309830	ENSP00000364320
ENSP00000309830	ENSP00000364398
ENSP00000309830	ENSP00000365806
ENSP00000309830	ENSP00000373090
ENSP00000309830	ENSP00000373404
ENSP00000309830	ENSP00000375730
ENSP00000309830	ENSP00000377640
ENSP00000309830	ENSP00000377865
ENSP00000309830	ENSP00000378160
ENSP00000309830	ENSP00000379607
ENSP00000309830	ENSP00000379888
ENSP00000309830	ENSP00000380156
ENSP00000309830	ENSP00000386717
ENSP00000309830	ENSP00000389103
ENSP00000309830	ENSP00000392466
ENSP00000309830	ENSP00000393393
ENSP00000309830	ENSP00000394290
ENSP00000309830	ENSP00000400591
ENSP00000309830	ENSP00000402338
ENSP00000309830	ENSP00000403576
ENSP00000309830	ENSP00000409074
ENSP00000309830	ENSP00000409367
ENSP00000309830	ENSP00000411162
ENSP00000309831	ENSP00000358716
ENSP00000309831	ENSP00000370083
ENSP00000309831	ENSP00000407401
ENSP00000309831	ENSP00000412566
ENSP00000309836	ENSP00000368755
ENSP00000309871	ENSP00000322957
ENSP00000309871	ENSP00000323858
ENSP00000309871	ENSP00000331310
ENSP00000309871	ENSP00000339826
ENSP00000309871	ENSP00000339952
ENSP00000309871	ENSP00000347778

ENSP00000309871	ENSP00000360532
ENSP00000309871	ENSP00000362592
ENSP00000309871	ENSP00000375986
ENSP00000309871	ENSP00000377793
ENSP00000309871	ENSP00000379607
ENSP00000309871	ENSP00000380921
ENSP00000309968	ENSP00000345731
ENSP00000310003	ENSP00000361057
ENSP00000310003	ENSP00000397441
ENSP00000310008	ENSP00000343392
ENSP00000310008	ENSP00000382218
ENSP00000310040	ENSP00000316879
ENSP00000310040	ENSP00000325448
ENSP00000310040	ENSP00000329002
ENSP00000310040	ENSP00000336702
ENSP00000310040	ENSP00000337405
ENSP00000310040	ENSP00000339095
ENSP00000310040	ENSP00000340281
ENSP00000310040	ENSP00000341243
ENSP00000310040	ENSP00000354125
ENSP00000310040	ENSP00000354558
ENSP00000310040	ENSP00000354673
ENSP00000310040	ENSP00000357907
ENSP00000310040	ENSP00000358140
ENSP00000310040	ENSP00000358563
ENSP00000310040	ENSP00000362688
ENSP00000310040	ENSP00000364119
ENSP00000310040	ENSP00000365175
ENSP00000310040	ENSP00000369816
ENSP00000310040	ENSP00000379607
ENSP00000310040	ENSP00000384211
ENSP00000310040	ENSP00000401508
ENSP00000310040	ENSP00000416892
ENSP00000310040	ENSP00000417748
ENSP00000310042	ENSP00000310649
ENSP00000310042	ENSP00000320488
ENSP00000310042	ENSP00000325312
ENSP00000310042	ENSP00000337445
ENSP00000310042	ENSP00000338573
ENSP00000310042	ENSP00000343190
ENSP00000310042	ENSP00000346453
ENSP00000310042	ENSP00000351908
ENSP00000310042	ENSP00000356920
ENSP00000310042	ENSP00000361642
ENSP00000310042	ENSP00000362153
ENSP00000310042	ENSP00000362171

ENSP00000310042	ENSP00000363559
ENSP00000310042	ENSP00000365806
ENSP00000310042	ENSP00000369757
ENSP00000310042	ENSP00000371451
ENSP00000310042	ENSP00000373090
ENSP00000310042	ENSP00000374213
ENSP00000310042	ENSP00000377640
ENSP00000310042	ENSP00000381932
ENSP00000310042	ENSP00000384048
ENSP00000310042	ENSP00000394290
ENSP00000310042	ENSP00000403576
ENSP00000310042	ENSP00000409074
ENSP00000310042	ENSP00000409367
ENSP00000310042	ENSP00000411162
ENSP00000310082	ENSP00000327436
ENSP00000310082	ENSP00000329189
ENSP00000310082	ENSP00000336712
ENSP00000310082	ENSP00000346050
ENSP00000310082	ENSP00000351284
ENSP00000310082	ENSP00000369335
ENSP00000310094	ENSP00000360916
ENSP00000310129	ENSP00000310440
ENSP00000310129	ENSP00000311344
ENSP00000310129	ENSP00000311665
ENSP00000310129	ENSP00000311873
ENSP00000310129	ENSP00000314004
ENSP00000310129	ENSP00000314499
ENSP00000310129	ENSP00000318944
ENSP00000310129	ENSP00000319977
ENSP00000310129	ENSP00000321711
ENSP00000310129	ENSP00000325919
ENSP00000310129	ENSP00000329466
ENSP00000310129	ENSP00000329715
ENSP00000310129	ENSP00000333122
ENSP00000310129	ENSP00000334061
ENSP00000310129	ENSP00000337501
ENSP00000310129	ENSP00000341243
ENSP00000310129	ENSP00000344055
ENSP00000310129	ENSP00000347978
ENSP00000310129	ENSP00000348442
ENSP00000310129	ENSP00000348565
ENSP00000310129	ENSP00000350199
ENSP00000310129	ENSP00000350708
ENSP00000310129	ENSP00000353622
ENSP00000310129	ENSP00000353826
ENSP00000310129	ENSP00000355560

ENSP00000310129	ENSP00000355865
ENSP00000310129	ENSP00000355890
ENSP00000310129	ENSP00000356655
ENSP00000310129	ENSP00000360216
ENSP00000310129	ENSP00000360532
ENSP00000310129	ENSP00000360922
ENSP00000310129	ENSP00000362334
ENSP00000310129	ENSP00000362688
ENSP00000310129	ENSP00000364204
ENSP00000310129	ENSP00000365837
ENSP00000310129	ENSP00000366395
ENSP00000310129	ENSP00000370222
ENSP00000310129	ENSP00000370408
ENSP00000310129	ENSP00000370414
ENSP00000310129	ENSP00000372199
ENSP00000310129	ENSP00000375986
ENSP00000310129	ENSP00000376945
ENSP00000310129	ENSP00000382269
ENSP00000310129	ENSP00000384109
ENSP00000310129	ENSP00000384211
ENSP00000310129	ENSP00000396937
ENSP00000310129	ENSP00000403817
ENSP00000310129	ENSP00000417281
ENSP00000310129	ENSP00000417748
ENSP00000310129	ENSP00000418734
ENSP00000310260	ENSP00000311344
ENSP00000310260	ENSP00000312735
ENSP00000310260	ENSP00000314949
ENSP00000310260	ENSP00000316879
ENSP00000310260	ENSP00000318641
ENSP00000310260	ENSP00000324124
ENSP00000310260	ENSP00000326379
ENSP00000310260	ENSP00000327889
ENSP00000310260	ENSP00000340220
ENSP00000310260	ENSP00000348708
ENSP00000310260	ENSP00000355961
ENSP00000310260	ENSP00000358140
ENSP00000310260	ENSP00000360532
ENSP00000310260	ENSP00000362170
ENSP00000310260	ENSP00000364289
ENSP00000310260	ENSP00000381064
ENSP00000310260	ENSP00000385722
ENSP00000310260	ENSP00000398208
ENSP00000310260	ENSP00000414237
ENSP00000310260	ENSP00000418210
ENSP00000310303	ENSP00000384490

ENSP00000310332	ENSP00000349892
ENSP00000310338	ENSP00000326514
ENSP00000310338	ENSP00000366863
ENSP00000310356	ENSP00000317039
ENSP00000310356	ENSP00000321636
ENSP00000310356	ENSP00000347232
ENSP00000310375	ENSP00000353452
ENSP00000310440	ENSP00000317468
ENSP00000310440	ENSP00000318629
ENSP00000310440	ENSP00000330408
ENSP00000310440	ENSP00000331201
ENSP00000310440	ENSP00000343479
ENSP00000310440	ENSP00000345216
ENSP00000310440	ENSP00000348081
ENSP00000310440	ENSP00000356602
ENSP00000310440	ENSP00000366565
ENSP00000310440	ENSP00000367001
ENSP00000310440	ENSP00000367299
ENSP00000310440	ENSP00000368686
ENSP00000310440	ENSP00000374455
ENSP00000310440	ENSP00000378368
ENSP00000310440	ENSP00000391432
ENSP00000310448	ENSP00000310596
ENSP00000310448	ENSP00000310723
ENSP00000310448	ENSP00000312981
ENSP00000310448	ENSP00000313674
ENSP00000310448	ENSP00000315035
ENSP00000310448	ENSP00000315379
ENSP00000310448	ENSP00000315859
ENSP00000310448	ENSP00000318861
ENSP00000310448	ENSP00000319341
ENSP00000310448	ENSP00000320566
ENSP00000310448	ENSP00000324122
ENSP00000310448	ENSP00000324274
ENSP00000310448	ENSP00000324573
ENSP00000310448	ENSP00000326477
ENSP00000310448	ENSP00000326806
ENSP00000310448	ENSP00000328690
ENSP00000310448	ENSP00000329466
ENSP00000310448	ENSP00000330343
ENSP00000310448	ENSP00000332103
ENSP00000310448	ENSP00000335321
ENSP00000310448	ENSP00000336842
ENSP00000310448	ENSP00000337405
ENSP00000310448	ENSP00000339090
ENSP00000310448	ENSP00000340271

ENSP00000310448	ENSP00000342374
ENSP00000310448	ENSP00000344241
ENSP00000310448	ENSP00000346986
ENSP00000310448	ENSP00000348010
ENSP00000310448	ENSP00000351492
ENSP00000310448	ENSP00000355013
ENSP00000310448	ENSP00000355205
ENSP00000310448	ENSP00000356405
ENSP00000310448	ENSP00000357887
ENSP00000310448	ENSP00000359454
ENSP00000310448	ENSP00000360286
ENSP00000310448	ENSP00000360532
ENSP00000310448	ENSP00000361076
ENSP00000310448	ENSP00000361557
ENSP00000310448	ENSP00000361577
ENSP00000310448	ENSP00000362110
ENSP00000310448	ENSP00000362900
ENSP00000310448	ENSP00000362936
ENSP00000310448	ENSP00000363313
ENSP00000310448	ENSP00000363559
ENSP00000310448	ENSP00000364289
ENSP00000310448	ENSP00000364813
ENSP00000310448	ENSP00000366557
ENSP00000310448	ENSP00000367439
ENSP00000310448	ENSP00000368801
ENSP00000310448	ENSP00000370589
ENSP00000310448	ENSP00000372883
ENSP00000310448	ENSP00000374213
ENSP00000310448	ENSP00000377747
ENSP00000310448	ENSP00000379607
ENSP00000310448	ENSP00000383059
ENSP00000310448	ENSP00000388872
ENSP00000310448	ENSP00000391432
ENSP00000310448	ENSP00000392601
ENSP00000310448	ENSP00000400591
ENSP00000310448	ENSP00000403345
ENSP00000310448	ENSP00000404151
ENSP00000310448	ENSP00000406280
ENSP00000310448	ENSP00000410758
ENSP00000310448	ENSP00000412566
ENSP00000310448	ENSP00000414006
ENSP00000310448	ENSP00000414634
ENSP00000310459	ENSP00000340505
ENSP00000310520	ENSP00000311873
ENSP00000310520	ENSP00000313171
ENSP00000310520	ENSP00000314949

ENSP00000310520	ENSP00000315700
ENSP00000310520	ENSP00000317636
ENSP00000310520	ENSP00000318977
ENSP00000310520	ENSP00000321636
ENSP00000310520	ENSP00000322570
ENSP00000310520	ENSP00000323439
ENSP00000310520	ENSP00000323511
ENSP00000310520	ENSP00000325863
ENSP00000310520	ENSP00000328940
ENSP00000310520	ENSP00000335557
ENSP00000310520	ENSP00000336606
ENSP00000310520	ENSP00000337353
ENSP00000310520	ENSP00000340305
ENSP00000310520	ENSP00000340879
ENSP00000310520	ENSP00000343392
ENSP00000310520	ENSP00000343741
ENSP00000310520	ENSP00000343745
ENSP00000310520	ENSP00000344741
ENSP00000310520	ENSP00000345917
ENSP00000310520	ENSP00000347232
ENSP00000310520	ENSP00000347978
ENSP00000310520	ENSP00000348020
ENSP00000310520	ENSP00000348089
ENSP00000310520	ENSP00000349393
ENSP00000310520	ENSP00000351284
ENSP00000310520	ENSP00000351697
ENSP00000310520	ENSP00000352257
ENSP00000310520	ENSP00000353332
ENSP00000310520	ENSP00000354522
ENSP00000310520	ENSP00000354957
ENSP00000310520	ENSP00000355077
ENSP00000310520	ENSP00000358019
ENSP00000310520	ENSP00000358478
ENSP00000310520	ENSP00000359904
ENSP00000310520	ENSP00000360116
ENSP00000310520	ENSP00000360310
ENSP00000310520	ENSP00000360803
ENSP00000310520	ENSP00000361310
ENSP00000310520	ENSP00000361824
ENSP00000310520	ENSP00000364126
ENSP00000310520	ENSP00000364270
ENSP00000310520	ENSP00000367787
ENSP00000310520	ENSP00000367910
ENSP00000310520	ENSP00000368349
ENSP00000310520	ENSP00000368438
ENSP00000310520	ENSP00000369335

ENSP00000310520	ENSP00000369411
ENSP00000310520	ENSP00000370151
ENSP00000310520	ENSP00000370376
ENSP00000310520	ENSP00000371475
ENSP00000310520	ENSP00000373952
ENSP00000310520	ENSP00000375809
ENSP00000310520	ENSP00000381657
ENSP00000310520	ENSP00000394649
ENSP00000310520	ENSP00000396704
ENSP00000310520	ENSP00000400806
ENSP00000310520	ENSP00000406490
ENSP00000310520	ENSP00000406868
ENSP00000310521	ENSP00000311134
ENSP00000310521	ENSP00000324628
ENSP00000310521	ENSP00000325002
ENSP00000310521	ENSP00000327309
ENSP00000310521	ENSP00000328079
ENSP00000310521	ENSP00000329419
ENSP00000310521	ENSP00000337478
ENSP00000310521	ENSP00000338533
ENSP00000310521	ENSP00000347329
ENSP00000310521	ENSP00000349887
ENSP00000310521	ENSP00000350708
ENSP00000310521	ENSP00000353073
ENSP00000310521	ENSP00000355721
ENSP00000310521	ENSP00000356405
ENSP00000310521	ENSP00000356530
ENSP00000310521	ENSP00000357048
ENSP00000310521	ENSP00000357887
ENSP00000310521	ENSP00000360183
ENSP00000310521	ENSP00000366225
ENSP00000310521	ENSP00000369810
ENSP00000310521	ENSP00000374213
ENSP00000310521	ENSP00000381293
ENSP00000310521	ENSP00000386759
ENSP00000310521	ENSP00000390783
ENSP00000310521	ENSP00000397441
ENSP00000310521	ENSP00000401445
ENSP00000310521	ENSP00000404247
ENSP00000310521	ENSP00000417748
ENSP00000310521	ENSP00000418661
ENSP00000310547	ENSP00000312587
ENSP00000310547	ENSP00000322316
ENSP00000310547	ENSP00000387262
ENSP00000310551	ENSP00000339245
ENSP00000310551	ENSP00000354876

ENSP00000310551	ENSP00000357998
ENSP00000310557	ENSP00000373363
ENSP00000310557	ENSP00000398350
ENSP00000310561	ENSP00000344456
ENSP00000310561	ENSP00000344666
ENSP00000310561	ENSP00000358770
ENSP00000310561	ENSP00000360916
ENSP00000310585	ENSP00000344456
ENSP00000310585	ENSP00000345731
ENSP00000310585	ENSP00000355299
ENSP00000310585	ENSP00000361467
ENSP00000310585	ENSP00000380150
ENSP00000310585	ENSP00000381031
ENSP00000310590	ENSP00000310842
ENSP00000310590	ENSP00000321449
ENSP00000310590	ENSP00000334849
ENSP00000310590	ENSP00000337127
ENSP00000310590	ENSP00000339795
ENSP00000310590	ENSP00000344401
ENSP00000310590	ENSP00000355759
ENSP00000310590	ENSP00000358563
ENSP00000310590	ENSP00000359000
ENSP00000310590	ENSP00000362153
ENSP00000310590	ENSP00000363746
ENSP00000310590	ENSP00000367029
ENSP00000310590	ENSP00000368438
ENSP00000310590	ENSP00000372695
ENSP00000310590	ENSP00000380460
ENSP00000310590	ENSP00000399454
ENSP00000310590	ENSP00000402869
ENSP00000310590	ENSP00000405614
ENSP00000310593	ENSP00000380921
ENSP00000310596	ENSP00000311430
ENSP00000310596	ENSP00000311505
ENSP00000310596	ENSP00000313432
ENSP00000310596	ENSP00000313674
ENSP00000310596	ENSP00000314311
ENSP00000310596	ENSP00000315035
ENSP00000310596	ENSP00000315379
ENSP00000310596	ENSP00000316879
ENSP00000310596	ENSP00000318195
ENSP00000310596	ENSP00000318604
ENSP00000310596	ENSP00000319341
ENSP00000310596	ENSP00000320503
ENSP00000310596	ENSP00000320566
ENSP00000310596	ENSP00000322242

ENSP00000310596	ENSP00000322419
ENSP00000310596	ENSP00000323076
ENSP00000310596	ENSP00000323302
ENSP00000310596	ENSP00000324122
ENSP00000310596	ENSP00000325448
ENSP00000310596	ENSP00000337393
ENSP00000310596	ENSP00000337839
ENSP00000310596	ENSP00000339090
ENSP00000310596	ENSP00000339145
ENSP00000310596	ENSP00000341743
ENSP00000310596	ENSP00000342374
ENSP00000310596	ENSP00000343742
ENSP00000310596	ENSP00000346015
ENSP00000310596	ENSP00000347087
ENSP00000310596	ENSP00000348708
ENSP00000310596	ENSP00000349616
ENSP00000310596	ENSP00000351284
ENSP00000310596	ENSP00000351596
ENSP00000310596	ENSP00000351885
ENSP00000310596	ENSP00000352738
ENSP00000310596	ENSP00000352820
ENSP00000310596	ENSP00000353059
ENSP00000310596	ENSP00000353622
ENSP00000310596	ENSP00000354522
ENSP00000310596	ENSP00000356346
ENSP00000310596	ENSP00000356405
ENSP00000310596	ENSP00000356811
ENSP00000310596	ENSP00000356989
ENSP00000310596	ENSP00000357602
ENSP00000310596	ENSP00000357887
ENSP00000310596	ENSP00000357907
ENSP00000310596	ENSP00000358478
ENSP00000310596	ENSP00000359939
ENSP00000310596	ENSP00000361949
ENSP00000310596	ENSP00000362413
ENSP00000310596	ENSP00000362576
ENSP00000310596	ENSP00000362900
ENSP00000310596	ENSP00000363313
ENSP00000310596	ENSP00000364293
ENSP00000310596	ENSP00000364543
ENSP00000310596	ENSP00000364813
ENSP00000310596	ENSP00000366843
ENSP00000310596	ENSP00000366953
ENSP00000310596	ENSP00000366974
ENSP00000310596	ENSP00000369757
ENSP00000310596	ENSP00000370119

ENSP00000310596	ENSP00000370555
ENSP00000310596	ENSP00000370589
ENSP00000310596	ENSP00000372023
ENSP00000310596	ENSP00000372093
ENSP00000310596	ENSP00000372827
ENSP00000310596	ENSP00000373715
ENSP00000310596	ENSP00000376268
ENSP00000310596	ENSP00000376973
ENSP00000310596	ENSP00000380460
ENSP00000310596	ENSP00000382177
ENSP00000310596	ENSP00000382492
ENSP00000310596	ENSP00000386759
ENSP00000310596	ENSP00000391723
ENSP00000310596	ENSP00000394400
ENSP00000310596	ENSP00000398597
ENSP00000310596	ENSP00000399530
ENSP00000310596	ENSP00000400626
ENSP00000310596	ENSP00000410758
ENSP00000310596	ENSP00000412310
ENSP00000310596	ENSP00000414006
ENSP00000310596	ENSP00000414634
ENSP00000310621	ENSP00000311200
ENSP00000310621	ENSP00000348602
ENSP00000310621	ENSP00000356146
ENSP00000310621	ENSP00000356530
ENSP00000310621	ENSP00000368226
ENSP00000310621	ENSP00000401445
ENSP00000310622	ENSP00000334061
ENSP00000310623	ENSP00000337127
ENSP00000310623	ENSP00000342026
ENSP00000310623	ENSP00000342056
ENSP00000310623	ENSP00000363205
ENSP00000310623	ENSP00000366482
ENSP00000310623	ENSP00000387219
ENSP00000310649	ENSP00000311005
ENSP00000310649	ENSP00000315859
ENSP00000310649	ENSP00000317904
ENSP00000310649	ENSP00000318775
ENSP00000310649	ENSP00000319169
ENSP00000310649	ENSP00000320488
ENSP00000310649	ENSP00000322180
ENSP00000310649	ENSP00000322957
ENSP00000310649	ENSP00000335044
ENSP00000310649	ENSP00000335357
ENSP00000310649	ENSP00000340954
ENSP00000310649	ENSP00000343392

ENSP00000310649	ENSP00000347602
ENSP00000310649	ENSP00000347733
ENSP00000310649	ENSP00000348877
ENSP00000310649	ENSP00000350078
ENSP00000310649	ENSP00000350491
ENSP00000310649	ENSP00000351596
ENSP00000310649	ENSP00000353793
ENSP00000310649	ENSP00000354916
ENSP00000310649	ENSP00000357907
ENSP00000310649	ENSP00000358886
ENSP00000310649	ENSP00000360076
ENSP00000310649	ENSP00000362171
ENSP00000310649	ENSP00000362578
ENSP00000310649	ENSP00000369897
ENSP00000310649	ENSP00000372023
ENSP00000310649	ENSP00000374213
ENSP00000310649	ENSP00000378350
ENSP00000310649	ENSP00000378529
ENSP00000310649	ENSP00000391249
ENSP00000310649	ENSP00000391944
ENSP00000310649	ENSP00000402084
ENSP00000310649	ENSP00000403817
ENSP00000310668	ENSP00000311430
ENSP00000310668	ENSP00000316032
ENSP00000310668	ENSP00000322016
ENSP00000310668	ENSP00000323511
ENSP00000310668	ENSP00000325312
ENSP00000310668	ENSP00000325376
ENSP00000310668	ENSP00000326806
ENSP00000310668	ENSP00000329466
ENSP00000310668	ENSP00000331817
ENSP00000310668	ENSP00000335357
ENSP00000310668	ENSP00000336712
ENSP00000310668	ENSP00000337797
ENSP00000310668	ENSP00000340698
ENSP00000310668	ENSP00000346453
ENSP00000310668	ENSP00000346566
ENSP00000310668	ENSP00000348577
ENSP00000310668	ENSP00000353073
ENSP00000310668	ENSP00000354916
ENSP00000310668	ENSP00000355922
ENSP00000310668	ENSP00000356448
ENSP00000310668	ENSP00000357547
ENSP00000310668	ENSP00000360286
ENSP00000310668	ENSP00000360532
ENSP00000310668	ENSP00000361949

ENSP00000310668	ENSP00000362413
ENSP00000310668	ENSP00000362900
ENSP00000310668	ENSP00000366557
ENSP00000310668	ENSP00000371155
ENSP00000310668	ENSP00000371451
ENSP00000310668	ENSP00000382779
ENSP00000310668	ENSP00000391723
ENSP00000310701	ENSP00000311005
ENSP00000310701	ENSP00000311343
ENSP00000310701	ENSP00000313243
ENSP00000310701	ENSP00000354927
ENSP00000310701	ENSP00000356448
ENSP00000310701	ENSP00000357440
ENSP00000310701	ENSP00000359444
ENSP00000310701	ENSP00000359668
ENSP00000310701	ENSP00000364895
ENSP00000310701	ENSP00000365005
ENSP00000310701	ENSP00000370410
ENSP00000310721	ENSP00000312458
ENSP00000310721	ENSP00000320604
ENSP00000310721	ENSP00000325425
ENSP00000310721	ENSP00000327716
ENSP00000310721	ENSP00000330812
ENSP00000310721	ENSP00000340896
ENSP00000310721	ENSP00000341662
ENSP00000310721	ENSP00000344460
ENSP00000310721	ENSP00000348762
ENSP00000310721	ENSP00000349877
ENSP00000310721	ENSP00000355955
ENSP00000310721	ENSP00000360918
ENSP00000310723	ENSP00000311430
ENSP00000310723	ENSP00000312981
ENSP00000310723	ENSP00000315859
ENSP00000310723	ENSP00000324203
ENSP00000310723	ENSP00000329466
ENSP00000310723	ENSP00000331817
ENSP00000310723	ENSP00000339090
ENSP00000310723	ENSP00000354522
ENSP00000310723	ENSP00000356448
ENSP00000310723	ENSP00000357887
ENSP00000310723	ENSP00000358812
ENSP00000310723	ENSP00000362936
ENSP00000310723	ENSP00000363313
ENSP00000310723	ENSP00000364289
ENSP00000310723	ENSP00000366557
ENSP00000310723	ENSP00000368801

ENSP00000310723	ENSP00000377640
ENSP00000310723	ENSP00000393953
ENSP00000310785	ENSP00000365318
ENSP00000310785	ENSP00000379607
ENSP00000310785	ENSP00000383142
ENSP00000310800	ENSP00000325954
ENSP00000310800	ENSP00000334003
ENSP00000310800	ENSP00000337439
ENSP00000310800	ENSP00000345731
ENSP00000310800	ENSP00000349486
ENSP00000310800	ENSP00000351605
ENSP00000310800	ENSP00000355760
ENSP00000310800	ENSP00000361467
ENSP00000310800	ENSP00000368797
ENSP00000310800	ENSP00000375783
ENSP00000310800	ENSP00000380150
ENSP00000310832	ENSP00000357484
ENSP00000310842	ENSP00000329466
ENSP00000310842	ENSP00000337797
ENSP00000310842	ENSP00000354497
ENSP00000310842	ENSP00000360532
ENSP00000310842	ENSP00000364847
ENSP00000310842	ENSP00000372316
ENSP00000310842	ENSP00000379607
ENSP00000310842	ENSP00000381891
ENSP00000310842	ENSP00000385021
ENSP00000310933	ENSP00000313059
ENSP00000310933	ENSP00000337797
ENSP00000310933	ENSP00000338990
ENSP00000310933	ENSP00000348565
ENSP00000310933	ENSP00000350249
ENSP00000310933	ENSP00000350854
ENSP00000310933	ENSP00000360660
ENSP00000310951	ENSP00000350512
ENSP00000310951	ENSP00000392270
ENSP00000310966	ENSP00000327072
ENSP00000310966	ENSP00000354566
ENSP00000310966	ENSP00000361465
ENSP00000310966	ENSP00000364270
ENSP00000310966	ENSP00000367029
ENSP00000310966	ENSP00000379607
ENSP00000310998	ENSP00000316476
ENSP00000310998	ENSP00000351926
ENSP00000310998	ENSP00000360798
ENSP00000311005	ENSP00000314949
ENSP00000311005	ENSP00000322457

ENSP00000311005	ENSP00000326110
ENSP00000311005	ENSP00000332444
ENSP00000311005	ENSP00000336528
ENSP00000311005	ENSP00000340019
ENSP00000311005	ENSP00000340879
ENSP00000311005	ENSP00000341848
ENSP00000311005	ENSP00000347294
ENSP00000311005	ENSP00000349959
ENSP00000311005	ENSP00000350681
ENSP00000311005	ENSP00000351015
ENSP00000311005	ENSP00000351416
ENSP00000311005	ENSP00000354519
ENSP00000311005	ENSP00000354558
ENSP00000311005	ENSP00000354927
ENSP00000311005	ENSP00000356379
ENSP00000311005	ENSP00000357182
ENSP00000311005	ENSP00000357283
ENSP00000311005	ENSP00000357494
ENSP00000311005	ENSP00000360621
ENSP00000311005	ENSP00000361264
ENSP00000311005	ENSP00000362413
ENSP00000311005	ENSP00000365005
ENSP00000311005	ENSP00000369042
ENSP00000311005	ENSP00000373474
ENSP00000311005	ENSP00000375986
ENSP00000311005	ENSP00000378306
ENSP00000311005	ENSP00000379372
ENSP00000311005	ENSP00000379709
ENSP00000311005	ENSP00000380129
ENSP00000311005	ENSP00000380349
ENSP00000311005	ENSP00000382177
ENSP00000311005	ENSP00000383295
ENSP00000311010	ENSP00000318646
ENSP00000311010	ENSP00000325663
ENSP00000311010	ENSP00000337501
ENSP00000311028	ENSP00000311430
ENSP00000311028	ENSP00000315674
ENSP00000311028	ENSP00000317872
ENSP00000311028	ENSP00000318646
ENSP00000311028	ENSP00000320658
ENSP00000311028	ENSP00000320917
ENSP00000311028	ENSP00000322419
ENSP00000311028	ENSP00000323856
ENSP00000311028	ENSP00000326981
ENSP00000311028	ENSP00000327179
ENSP00000311028	ENSP00000329466

ENSP00000311028	ENSP00000331019
ENSP00000311028	ENSP00000335657
ENSP00000311028	ENSP00000336702
ENSP00000311028	ENSP00000337127
ENSP00000311028	ENSP00000339095
ENSP00000311028	ENSP00000339795
ENSP00000311028	ENSP00000341885
ENSP00000311028	ENSP00000342300
ENSP00000311028	ENSP00000345156
ENSP00000311028	ENSP00000346015
ENSP00000311028	ENSP00000346022
ENSP00000311028	ENSP00000346027
ENSP00000311028	ENSP00000346037
ENSP00000311028	ENSP00000346050
ENSP00000311028	ENSP00000346067
ENSP00000311028	ENSP00000347087
ENSP00000311028	ENSP00000354525
ENSP00000311028	ENSP00000354739
ENSP00000311028	ENSP00000355471
ENSP00000311028	ENSP00000356134
ENSP00000311028	ENSP00000356548
ENSP00000311028	ENSP00000356811
ENSP00000311028	ENSP00000361076
ENSP00000311028	ENSP00000362036
ENSP00000311028	ENSP00000362576
ENSP00000311028	ENSP00000362688
ENSP00000311028	ENSP00000362744
ENSP00000311028	ENSP00000363018
ENSP00000311028	ENSP00000363308
ENSP00000311028	ENSP00000363676
ENSP00000311028	ENSP00000364320
ENSP00000311028	ENSP00000368790
ENSP00000311028	ENSP00000369757
ENSP00000311028	ENSP00000372445
ENSP00000311028	ENSP00000373404
ENSP00000311028	ENSP00000375730
ENSP00000311028	ENSP00000377640
ENSP00000311028	ENSP00000379339
ENSP00000311028	ENSP00000379372
ENSP00000311028	ENSP00000379607
ENSP00000311028	ENSP00000379888
ENSP00000311028	ENSP00000384302
ENSP00000311028	ENSP00000386717
ENSP00000311028	ENSP00000389103
ENSP00000311028	ENSP00000393241
ENSP00000311028	ENSP00000393393

ENSP00000311028	ENSP00000402338
ENSP00000311028	ENSP00000403175
ENSP00000311028	ENSP00000412583
ENSP00000311028	ENSP00000414321
ENSP00000311028	ENSP00000416110
ENSP00000311028	ENSP00000417281
ENSP00000311028	ENSP00000417763
ENSP00000311035	ENSP00000420298
ENSP00000311083	ENSP00000312981
ENSP00000311083	ENSP00000317376
ENSP00000311083	ENSP00000323246
ENSP00000311083	ENSP00000334148
ENSP00000311083	ENSP00000344460
ENSP00000311083	ENSP00000345728
ENSP00000311083	ENSP00000348554
ENSP00000311083	ENSP00000358563
ENSP00000311083	ENSP00000360268
ENSP00000311083	ENSP00000360621
ENSP00000311083	ENSP00000367034
ENSP00000311083	ENSP00000378350
ENSP00000311083	ENSP00000378529
ENSP00000311134	ENSP00000316990
ENSP00000311134	ENSP00000325002
ENSP00000311134	ENSP00000327650
ENSP00000311134	ENSP00000336724
ENSP00000311134	ENSP00000343435
ENSP00000311134	ENSP00000346139
ENSP00000311134	ENSP00000352708
ENSP00000311134	ENSP00000373979
ENSP00000311134	ENSP00000377280
ENSP00000311134	ENSP00000381089
ENSP00000311134	ENSP00000390783
ENSP00000311135	ENSP00000314193
ENSP00000311135	ENSP00000314311
ENSP00000311135	ENSP00000315674
ENSP00000311135	ENSP00000317992
ENSP00000311135	ENSP00000320566
ENSP00000311135	ENSP00000320917
ENSP00000311135	ENSP00000322396
ENSP00000311135	ENSP00000326806
ENSP00000311135	ENSP00000326981
ENSP00000311135	ENSP00000327179
ENSP00000311135	ENSP00000328854
ENSP00000311135	ENSP00000329466
ENSP00000311135	ENSP00000331817
ENSP00000311135	ENSP00000335321

ENSP00000311135	ENSP00000339095
ENSP00000311135	ENSP00000340271
ENSP00000311135	ENSP00000343557
ENSP00000311135	ENSP00000346120
ENSP00000311135	ENSP00000355541
ENSP00000311135	ENSP00000358563
ENSP00000311135	ENSP00000358812
ENSP00000311135	ENSP00000359688
ENSP00000311135	ENSP00000360031
ENSP00000311135	ENSP00000361577
ENSP00000311135	ENSP00000362592
ENSP00000311135	ENSP00000362744
ENSP00000311135	ENSP00000362936
ENSP00000311135	ENSP00000363642
ENSP00000311135	ENSP00000363746
ENSP00000311135	ENSP00000364929
ENSP00000311135	ENSP00000366135
ENSP00000311135	ENSP00000366557
ENSP00000311135	ENSP00000366607
ENSP00000311135	ENSP00000367024
ENSP00000311135	ENSP00000368984
ENSP00000311135	ENSP00000370589
ENSP00000311135	ENSP00000371169
ENSP00000311135	ENSP00000372023
ENSP00000311135	ENSP00000372695
ENSP00000311135	ENSP00000377944
ENSP00000311135	ENSP00000379888
ENSP00000311135	ENSP00000398597
ENSP00000311135	ENSP00000399454
ENSP00000311135	ENSP00000402338
ENSP00000311135	ENSP00000402869
ENSP00000311135	ENSP00000405614
ENSP00000311184	ENSP00000348307
ENSP00000311184	ENSP00000356991
ENSP00000311196	ENSP00000351407
ENSP00000311200	ENSP00000317334
ENSP00000311200	ENSP00000317468
ENSP00000311200	ENSP00000328968
ENSP00000311200	ENSP00000331268
ENSP00000311200	ENSP00000338607
ENSP00000311200	ENSP00000345530
ENSP00000311200	ENSP00000346809
ENSP00000311200	ENSP00000350854
ENSP00000311200	ENSP00000351346
ENSP00000311200	ENSP00000353362
ENSP00000311200	ENSP00000361057

ENSP00000311200	ENSP00000376946
ENSP00000311200	ENSP00000379228
ENSP00000311200	ENSP00000380793
ENSP00000311202	ENSP00000323856
ENSP00000311202	ENSP00000329715
ENSP00000311202	ENSP00000356602
ENSP00000311202	ENSP00000368438
ENSP00000311202	ENSP00000370410
ENSP00000311221	ENSP00000356520
ENSP00000311245	ENSP00000341213
ENSP00000311245	ENSP00000397435
ENSP00000311290	ENSP00000344456
ENSP00000311290	ENSP00000372023
ENSP00000311318	ENSP00000324560
ENSP00000311318	ENSP00000331310
ENSP00000311318	ENSP00000343313
ENSP00000311318	ENSP00000348784
ENSP00000311318	ENSP00000371888
ENSP00000311343	ENSP00000315659
ENSP00000311343	ENSP00000316845
ENSP00000311343	ENSP00000320949
ENSP00000311343	ENSP00000327315
ENSP00000311343	ENSP00000348215
ENSP00000311343	ENSP00000354673
ENSP00000311343	ENSP00000354927
ENSP00000311343	ENSP00000360644
ENSP00000311343	ENSP00000361009
ENSP00000311343	ENSP00000365005
ENSP00000311343	ENSP00000380450
ENSP00000311343	ENSP00000382423
ENSP00000311343	ENSP00000403397
ENSP00000311344	ENSP00000311766
ENSP00000311344	ENSP00000315702
ENSP00000311344	ENSP00000318195
ENSP00000311344	ENSP00000318604
ENSP00000311344	ENSP00000318641
ENSP00000311344	ENSP00000321711
ENSP00000311344	ENSP00000327889
ENSP00000311344	ENSP00000334767
ENSP00000311344	ENSP00000339881
ENSP00000311344	ENSP00000340019
ENSP00000311344	ENSP00000340278
ENSP00000311344	ENSP00000347719
ENSP00000311344	ENSP00000348573
ENSP00000311344	ENSP00000348784
ENSP00000311344	ENSP00000350071

ENSP00000311344	ENSP00000350447
ENSP00000311344	ENSP00000350785
ENSP00000311344	ENSP00000352676
ENSP00000311344	ENSP00000354558
ENSP00000311344	ENSP00000355812
ENSP00000311344	ENSP00000358140
ENSP00000311344	ENSP00000358810
ENSP00000311344	ENSP00000371888
ENSP00000311344	ENSP00000379093
ENSP00000311344	ENSP00000385722
ENSP00000311344	ENSP00000417963
ENSP00000311368	ENSP00000322570
ENSP00000311368	ENSP00000357861
ENSP00000311368	ENSP00000358071
ENSP00000311368	ENSP00000368438
ENSP00000311368	ENSP00000370150
ENSP00000311368	ENSP00000372316
ENSP00000311368	ENSP00000379607
ENSP00000311429	ENSP00000328236
ENSP00000311430	ENSP00000312311
ENSP00000311430	ENSP00000314348
ENSP00000311430	ENSP00000317376
ENSP00000311430	ENSP00000318646
ENSP00000311430	ENSP00000319977
ENSP00000311430	ENSP00000321449
ENSP00000311430	ENSP00000322419
ENSP00000311430	ENSP00000326981
ENSP00000311430	ENSP00000328690
ENSP00000311430	ENSP00000329466
ENSP00000311430	ENSP00000336702
ENSP00000311430	ENSP00000337839
ENSP00000311430	ENSP00000338862
ENSP00000311430	ENSP00000339095
ENSP00000311430	ENSP00000339145
ENSP00000311430	ENSP00000339795
ENSP00000311430	ENSP00000339845
ENSP00000311430	ENSP00000341082
ENSP00000311430	ENSP00000341538
ENSP00000311430	ENSP00000343392
ENSP00000311430	ENSP00000343745
ENSP00000311430	ENSP00000345156
ENSP00000311430	ENSP00000346015
ENSP00000311430	ENSP00000346022
ENSP00000311430	ENSP00000346027
ENSP00000311430	ENSP00000346037
ENSP00000311430	ENSP00000346050

ENSP00000311430	ENSP00000346088
ENSP00000311430	ENSP00000346120
ENSP00000311430	ENSP00000346725
ENSP00000311430	ENSP00000352738
ENSP00000311430	ENSP00000354040
ENSP00000311430	ENSP00000354125
ENSP00000311430	ENSP00000354739
ENSP00000311430	ENSP00000355136
ENSP00000311430	ENSP00000355899
ENSP00000311430	ENSP00000359345
ENSP00000311430	ENSP00000359518
ENSP00000311430	ENSP00000360031
ENSP00000311430	ENSP00000360286
ENSP00000311430	ENSP00000361076
ENSP00000311430	ENSP00000361616
ENSP00000311430	ENSP00000362334
ENSP00000311430	ENSP00000362744
ENSP00000311430	ENSP00000363018
ENSP00000311430	ENSP00000363676
ENSP00000311430	ENSP00000364320
ENSP00000311430	ENSP00000366519
ENSP00000311430	ENSP00000368887
ENSP00000311430	ENSP00000369757
ENSP00000311430	ENSP00000369965
ENSP00000311430	ENSP00000370589
ENSP00000311430	ENSP00000374390
ENSP00000311430	ENSP00000375730
ENSP00000311430	ENSP00000377486
ENSP00000311430	ENSP00000377640
ENSP00000311430	ENSP00000377865
ENSP00000311430	ENSP00000378160
ENSP00000311430	ENSP00000379607
ENSP00000311430	ENSP00000379888
ENSP00000311430	ENSP00000380156
ENSP00000311430	ENSP00000380982
ENSP00000311430	ENSP00000382392
ENSP00000311430	ENSP00000386621
ENSP00000311430	ENSP00000386717
ENSP00000311430	ENSP00000389103
ENSP00000311430	ENSP00000393393
ENSP00000311430	ENSP00000402338
ENSP00000311430	ENSP00000403817
ENSP00000311430	ENSP00000414321
ENSP00000311430	ENSP00000417229
ENSP00000311436	ENSP00000330343
ENSP00000311436	ENSP00000332721

ENSP00000311496	ENSP00000315791
ENSP00000311496	ENSP00000331487
ENSP00000311496	ENSP00000345892
ENSP00000311496	ENSP00000347836
ENSP00000311496	ENSP00000357998
ENSP00000311496	ENSP00000379457
ENSP00000311496	ENSP00000383719
ENSP00000311502	ENSP00000344668
ENSP00000311505	ENSP00000313169
ENSP00000311505	ENSP00000313420
ENSP00000311505	ENSP00000313513
ENSP00000311505	ENSP00000321706
ENSP00000311505	ENSP00000325136
ENSP00000311505	ENSP00000326238
ENSP00000311505	ENSP00000326514
ENSP00000311505	ENSP00000327145
ENSP00000311505	ENSP00000327821
ENSP00000311505	ENSP00000328236
ENSP00000311505	ENSP00000334100
ENSP00000311505	ENSP00000336702
ENSP00000311505	ENSP00000336927
ENSP00000311505	ENSP00000340596
ENSP00000311505	ENSP00000340944
ENSP00000311505	ENSP00000346240
ENSP00000311505	ENSP00000346874
ENSP00000311505	ENSP00000350491
ENSP00000311505	ENSP00000350894
ENSP00000311505	ENSP00000353660
ENSP00000311505	ENSP00000354045
ENSP00000311505	ENSP00000354977
ENSP00000311505	ENSP00000355013
ENSP00000311505	ENSP00000355518
ENSP00000311505	ENSP00000356989
ENSP00000311505	ENSP00000357791
ENSP00000311505	ENSP00000358857
ENSP00000311505	ENSP00000359837
ENSP00000311505	ENSP00000361658
ENSP00000311505	ENSP00000362298
ENSP00000311505	ENSP00000362334
ENSP00000311505	ENSP00000362399
ENSP00000311505	ENSP00000362413
ENSP00000311505	ENSP00000366135
ENSP00000311505	ENSP00000366672
ENSP00000311505	ENSP00000367398
ENSP00000311505	ENSP00000367766
ENSP00000311505	ENSP00000368767

ENSP00000311505	ENSP00000369257
ENSP00000311505	ENSP00000370023
ENSP00000311505	ENSP00000370119
ENSP00000311505	ENSP00000370543
ENSP00000311505	ENSP00000373370
ENSP00000311505	ENSP00000379933
ENSP00000311505	ENSP00000381567
ENSP00000311505	ENSP00000381793
ENSP00000311505	ENSP00000389934
ENSP00000311505	ENSP00000394071
ENSP00000311505	ENSP00000407487
ENSP00000311505	ENSP00000414920
ENSP00000311513	ENSP00000312697
ENSP00000311513	ENSP00000329466
ENSP00000311513	ENSP00000336868
ENSP00000311513	ENSP00000351250
ENSP00000311513	ENSP00000357887
ENSP00000311513	ENSP00000371546
ENSP00000311579	ENSP00000350249
ENSP00000311579	ENSP00000350990
ENSP00000311579	ENSP00000352408
ENSP00000311579	ENSP00000355596
ENSP00000311579	ENSP00000358022
ENSP00000311579	ENSP00000370194
ENSP00000311579	ENSP00000373301
ENSP00000311579	ENSP00000399511
ENSP00000311579	ENSP00000413625
ENSP00000311665	ENSP00000319104
ENSP00000311665	ENSP00000348442
ENSP00000311665	ENSP00000357907
ENSP00000311665	ENSP00000360473
ENSP00000311665	ENSP00000363779
ENSP00000311665	ENSP00000381891
ENSP00000311665	ENSP00000386759
ENSP00000311665	ENSP00000396937
ENSP00000311665	ENSP00000416583
ENSP00000311677	ENSP00000316578
ENSP00000311677	ENSP00000325863
ENSP00000311677	ENSP00000335321
ENSP00000311677	ENSP00000355050
ENSP00000311677	ENSP00000360532
ENSP00000311677	ENSP00000362592
ENSP00000311697	ENSP00000339826
ENSP00000311747	ENSP00000346725
ENSP00000311747	ENSP00000350249
ENSP00000311747	ENSP00000351894

ENSP00000311747	ENSP00000352514
ENSP00000311747	ENSP00000354951
ENSP00000311747	ENSP00000355759
ENSP00000311747	ENSP00000359345
ENSP00000311747	ENSP00000379607
ENSP00000311766	ENSP00000317790
ENSP00000311766	ENSP00000319323
ENSP00000311766	ENSP00000323511
ENSP00000311766	ENSP00000341213
ENSP00000311766	ENSP00000348577
ENSP00000311766	ENSP00000348886
ENSP00000311766	ENSP00000349142
ENSP00000311766	ENSP00000353826
ENSP00000311766	ENSP00000355890
ENSP00000311766	ENSP00000357547
ENSP00000311766	ENSP00000361824
ENSP00000311766	ENSP00000362399
ENSP00000311766	ENSP00000362576
ENSP00000311766	ENSP00000363205
ENSP00000311768	ENSP00000362463
ENSP00000311768	ENSP00000400591
ENSP00000311816	ENSP00000323967
ENSP00000311816	ENSP00000329357
ENSP00000311816	ENSP00000347184
ENSP00000311816	ENSP00000353622
ENSP00000311816	ENSP00000359531
ENSP00000311816	ENSP00000368253
ENSP00000311816	ENSP00000380718
ENSP00000311816	ENSP00000381031
ENSP00000311825	ENSP00000313084
ENSP00000311825	ENSP00000314080
ENSP00000311825	ENSP00000323050
ENSP00000311825	ENSP00000327213
ENSP00000311825	ENSP00000327704
ENSP00000311825	ENSP00000327959
ENSP00000311825	ENSP00000333456
ENSP00000311825	ENSP00000337445
ENSP00000311825	ENSP00000348128
ENSP00000311825	ENSP00000352257
ENSP00000311825	ENSP00000352514
ENSP00000311825	ENSP00000353741
ENSP00000311825	ENSP00000353854
ENSP00000311825	ENSP00000360215
ENSP00000311825	ENSP00000360938
ENSP00000311825	ENSP00000361005
ENSP00000311825	ENSP00000361076

ENSP00000311825	ENSP00000362744
ENSP00000311825	ENSP00000365735
ENSP00000311825	ENSP00000376534
ENSP00000311825	ENSP00000379339
ENSP00000311825	ENSP00000384179
ENSP00000311825	ENSP00000391944
ENSP00000311825	ENSP00000414750
ENSP00000311825	ENSP00000417281
ENSP00000311825	ENSP00000417980
ENSP00000311827	ENSP00000312244
ENSP00000311827	ENSP00000313059
ENSP00000311837	ENSP00000328968
ENSP00000311837	ENSP00000353154
ENSP00000311837	ENSP00000382064
ENSP00000311873	ENSP00000313171
ENSP00000311873	ENSP00000317039
ENSP00000311873	ENSP00000318977
ENSP00000311873	ENSP00000321636
ENSP00000311873	ENSP00000322180
ENSP00000311873	ENSP00000322570
ENSP00000311873	ENSP00000323050
ENSP00000311873	ENSP00000323439
ENSP00000311873	ENSP00000325863
ENSP00000311873	ENSP00000329466
ENSP00000311873	ENSP00000337353
ENSP00000311873	ENSP00000340879
ENSP00000311873	ENSP00000341382
ENSP00000311873	ENSP00000343392
ENSP00000311873	ENSP00000343741
ENSP00000311873	ENSP00000347232
ENSP00000311873	ENSP00000349393
ENSP00000311873	ENSP00000351284
ENSP00000311873	ENSP00000351697
ENSP00000311873	ENSP00000352257
ENSP00000311873	ENSP00000353793
ENSP00000311873	ENSP00000354522
ENSP00000311873	ENSP00000354720
ENSP00000311873	ENSP00000354957
ENSP00000311873	ENSP00000355185
ENSP00000311873	ENSP00000358478
ENSP00000311873	ENSP00000358576
ENSP00000311873	ENSP00000358812
ENSP00000311873	ENSP00000360216
ENSP00000311873	ENSP00000360310
ENSP00000311873	ENSP00000361310
ENSP00000311873	ENSP00000363021

ENSP00000311873	ENSP00000364270
ENSP00000311873	ENSP00000367527
ENSP00000311873	ENSP00000367787
ENSP00000311873	ENSP00000368349
ENSP00000311873	ENSP00000368438
ENSP00000311873	ENSP00000369335
ENSP00000311873	ENSP00000369411
ENSP00000311873	ENSP00000370151
ENSP00000311873	ENSP00000371475
ENSP00000311873	ENSP00000372023
ENSP00000311873	ENSP00000384048
ENSP00000311873	ENSP00000402515
ENSP00000311873	ENSP00000406490
ENSP00000311876	ENSP00000327821
ENSP00000311876	ENSP00000355011
ENSP00000311876	ENSP00000361993
ENSP00000311876	ENSP00000381031
ENSP00000311876	ENSP00000386227
ENSP00000311876	ENSP00000386722
ENSP00000311876	ENSP00000387170
ENSP00000311876	ENSP00000394085
ENSP00000311977	ENSP00000314441
ENSP00000311977	ENSP00000318775
ENSP00000311977	ENSP00000324956
ENSP00000311977	ENSP00000328671
ENSP00000311977	ENSP00000356476
ENSP00000311977	ENSP00000361465
ENSP00000311977	ENSP00000365837
ENSP00000311977	ENSP00000366135
ENSP00000311977	ENSP00000369335
ENSP00000311977	ENSP00000370741
ENSP00000311977	ENSP00000388275
ENSP00000311977	ENSP00000403852
ENSP00000311984	ENSP00000312735
ENSP00000311984	ENSP00000372857
ENSP00000311997	ENSP00000362399
ENSP00000311997	ENSP00000408910
ENSP00000312021	ENSP00000414187
ENSP00000312027	ENSP00000364893
ENSP00000312042	ENSP00000313420
ENSP00000312042	ENSP00000313829
ENSP00000312042	ENSP00000315925
ENSP00000312042	ENSP00000318195
ENSP00000312042	ENSP00000327821
ENSP00000312042	ENSP00000329466
ENSP00000312042	ENSP00000346577

ENSP00000312042	ENSP00000348442
ENSP00000312042	ENSP00000351682
ENSP00000312042	ENSP00000353575
ENSP00000312042	ENSP00000354951
ENSP00000312042	ENSP00000356104
ENSP00000312042	ENSP00000356520
ENSP00000312042	ENSP00000356591
ENSP00000312042	ENSP00000357459
ENSP00000312042	ENSP00000359837
ENSP00000312042	ENSP00000360532
ENSP00000312042	ENSP00000360621
ENSP00000312042	ENSP00000361949
ENSP00000312042	ENSP00000363205
ENSP00000312042	ENSP00000363308
ENSP00000312042	ENSP00000363944
ENSP00000312042	ENSP00000364119
ENSP00000312042	ENSP00000364270
ENSP00000312042	ENSP00000364289
ENSP00000312042	ENSP00000365534
ENSP00000312042	ENSP00000365877
ENSP00000312042	ENSP00000366156
ENSP00000312042	ENSP00000366179
ENSP00000312042	ENSP00000366395
ENSP00000312042	ENSP00000368565
ENSP00000312042	ENSP00000384675
ENSP00000312042	ENSP00000387170
ENSP00000312042	ENSP00000414187
ENSP00000312066	ENSP00000314311
ENSP00000312066	ENSP00000320508
ENSP00000312066	ENSP00000329466
ENSP00000312066	ENSP00000342181
ENSP00000312066	ENSP00000343535
ENSP00000312066	ENSP00000346015
ENSP00000312066	ENSP00000356405
ENSP00000312066	ENSP00000360532
ENSP00000312066	ENSP00000403817
ENSP00000312150	ENSP00000313419
ENSP00000312150	ENSP00000313875
ENSP00000312150	ENSP00000344456
ENSP00000312150	ENSP00000350894
ENSP00000312150	ENSP00000356641
ENSP00000312150	ENSP00000357907
ENSP00000312150	ENSP00000376899
ENSP00000312150	ENSP00000377793
ENSP00000312150	ENSP00000386165
ENSP00000312185	ENSP00000312664

ENSP00000312185	ENSP00000364277
ENSP00000312222	ENSP00000345702
ENSP00000312222	ENSP00000417281
ENSP00000312224	ENSP00000378306
ENSP00000312224	ENSP00000382779
ENSP00000312244	ENSP00000313420
ENSP00000312244	ENSP00000321617
ENSP00000312244	ENSP00000322242
ENSP00000312244	ENSP00000334188
ENSP00000312244	ENSP00000340896
ENSP00000312244	ENSP00000348784
ENSP00000312244	ENSP00000349213
ENSP00000312244	ENSP00000350767
ENSP00000312244	ENSP00000351490
ENSP00000312244	ENSP00000356989
ENSP00000312244	ENSP00000360473
ENSP00000312244	ENSP00000362166
ENSP00000312244	ENSP00000368439
ENSP00000312244	ENSP00000369695
ENSP00000312244	ENSP00000371888
ENSP00000312244	ENSP00000377262
ENSP00000312244	ENSP00000386759
ENSP00000312262	ENSP00000322419
ENSP00000312262	ENSP00000332353
ENSP00000312262	ENSP00000334061
ENSP00000312262	ENSP00000335311
ENSP00000312262	ENSP00000338345
ENSP00000312262	ENSP00000339191
ENSP00000312262	ENSP00000344524
ENSP00000312262	ENSP00000352121
ENSP00000312262	ENSP00000378338
ENSP00000312262	ENSP00000405963
ENSP00000312262	ENSP00000417281
ENSP00000312311	ENSP00000315017
ENSP00000312311	ENSP00000331817
ENSP00000312311	ENSP00000333401
ENSP00000312311	ENSP00000339845
ENSP00000312311	ENSP00000343745
ENSP00000312311	ENSP00000346015
ENSP00000312311	ENSP00000346022
ENSP00000312311	ENSP00000346067
ENSP00000312311	ENSP00000357048
ENSP00000312311	ENSP00000360498
ENSP00000312311	ENSP00000363018
ENSP00000312311	ENSP00000363676
ENSP00000312311	ENSP00000375730

ENSP00000312311	ENSP00000377486
ENSP00000312311	ENSP00000417602
ENSP00000312318	ENSP00000328854
ENSP00000312318	ENSP00000329715
ENSP00000312318	ENSP00000356602
ENSP00000312318	ENSP00000359115
ENSP00000312318	ENSP00000381795
ENSP00000312318	ENSP00000416892
ENSP00000312326	ENSP00000316854
ENSP00000312326	ENSP00000354927
ENSP00000312356	ENSP00000312981
ENSP00000312356	ENSP00000314441
ENSP00000312356	ENSP00000323313
ENSP00000312356	ENSP00000327821
ENSP00000312356	ENSP00000328671
ENSP00000312356	ENSP00000333667
ENSP00000312356	ENSP00000342564
ENSP00000312356	ENSP00000353741
ENSP00000312356	ENSP00000356476
ENSP00000312356	ENSP00000356548
ENSP00000312356	ENSP00000364320
ENSP00000312356	ENSP00000365837
ENSP00000312370	ENSP00000325562
ENSP00000312370	ENSP00000361418
ENSP00000312370	ENSP00000379933
ENSP00000312385	ENSP00000313084
ENSP00000312385	ENSP00000319279
ENSP00000312385	ENSP00000338629
ENSP00000312385	ENSP00000355010
ENSP00000312385	ENSP00000359652
ENSP00000312385	ENSP00000363827
ENSP00000312385	ENSP00000365735
ENSP00000312385	ENSP00000368678
ENSP00000312411	ENSP00000343144
ENSP00000312411	ENSP00000343190
ENSP00000312411	ENSP00000349205
ENSP00000312411	ENSP00000355596
ENSP00000312411	ENSP00000356530
ENSP00000312411	ENSP00000360268
ENSP00000312411	ENSP00000364893
ENSP00000312411	ENSP00000372023
ENSP00000312411	ENSP00000375986
ENSP00000312411	ENSP00000378306
ENSP00000312411	ENSP00000399511
ENSP00000312411	ENSP00000401445
ENSP00000312435	ENSP00000313084

ENSP00000312435	ENSP00000326366
ENSP00000312435	ENSP00000339191
ENSP00000312435	ENSP00000341189
ENSP00000312435	ENSP00000353508
ENSP00000312435	ENSP00000363827
ENSP00000312435	ENSP00000368678
ENSP00000312435	ENSP00000371512
ENSP00000312435	ENSP00000380921
ENSP00000312435	ENSP00000382064
ENSP00000312435	ENSP00000391490
ENSP00000312439	ENSP00000360116
ENSP00000312458	ENSP00000318147
ENSP00000312458	ENSP00000332258
ENSP00000312458	ENSP00000346809
ENSP00000312458	ENSP00000347206
ENSP00000312458	ENSP00000350263
ENSP00000312458	ENSP00000356530
ENSP00000312458	ENSP00000356591
ENSP00000312458	ENSP00000369820
ENSP00000312458	ENSP00000401445
ENSP00000312458	ENSP00000409275
ENSP00000312458	ENSP00000415203
ENSP00000312506	ENSP00000341189
ENSP00000312506	ENSP00000354201
ENSP00000312506	ENSP00000360116
ENSP00000312506	ENSP00000370410
ENSP00000312506	ENSP00000371341
ENSP00000312506	ENSP00000381098
ENSP00000312506	ENSP00000392330
ENSP00000312584	ENSP00000355627
ENSP00000312584	ENSP00000368698
ENSP00000312587	ENSP00000322316
ENSP00000312587	ENSP00000334061
ENSP00000312587	ENSP00000355739
ENSP00000312587	ENSP00000409275
ENSP00000312624	ENSP00000347979
ENSP00000312624	ENSP00000352272
ENSP00000312624	ENSP00000417281
ENSP00000312625	ENSP00000329466
ENSP00000312625	ENSP00000355205
ENSP00000312625	ENSP00000372023
ENSP00000312625	ENSP00000379607
ENSP00000312649	ENSP00000318373
ENSP00000312649	ENSP00000348069
ENSP00000312649	ENSP00000378503
ENSP00000312652	ENSP00000330393

ENSP00000312652	ENSP00000335074
ENSP00000312664	ENSP00000313420
ENSP00000312664	ENSP00000318822
ENSP00000312664	ENSP00000327647
ENSP00000312664	ENSP00000329623
ENSP00000312664	ENSP00000330484
ENSP00000312664	ENSP00000337797
ENSP00000312664	ENSP00000343701
ENSP00000312664	ENSP00000353059
ENSP00000312664	ENSP00000354791
ENSP00000312664	ENSP00000359096
ENSP00000312664	ENSP00000359100
ENSP00000312664	ENSP00000359691
ENSP00000312664	ENSP00000363680
ENSP00000312664	ENSP00000417281
ENSP00000312671	ENSP00000359345
ENSP00000312671	ENSP00000382064
ENSP00000312671	ENSP00000384792
ENSP00000312671	ENSP00000386520
ENSP00000312697	ENSP00000320566
ENSP00000312697	ENSP00000331310
ENSP00000312697	ENSP00000341280
ENSP00000312697	ENSP00000347168
ENSP00000312697	ENSP00000347733
ENSP00000312697	ENSP00000350767
ENSP00000312697	ENSP00000351490
ENSP00000312697	ENSP00000351947
ENSP00000312697	ENSP00000352516
ENSP00000312697	ENSP00000357887
ENSP00000312697	ENSP00000359518
ENSP00000312697	ENSP00000360532
ENSP00000312697	ENSP00000362166
ENSP00000312697	ENSP00000364929
ENSP00000312697	ENSP00000366974
ENSP00000312697	ENSP00000367030
ENSP00000312697	ENSP00000367034
ENSP00000312697	ENSP00000368438
ENSP00000312697	ENSP00000370611
ENSP00000312697	ENSP00000374213
ENSP00000312697	ENSP00000376097
ENSP00000312697	ENSP00000382688
ENSP00000312697	ENSP00000396439
ENSP00000312697	ENSP00000396876
ENSP00000312706	ENSP00000405708
ENSP00000312735	ENSP00000314949
ENSP00000312735	ENSP00000315925

ENSP00000312735	ENSP00000315950
ENSP00000312735	ENSP00000317334
ENSP00000312735	ENSP00000317636
ENSP00000312735	ENSP00000318195
ENSP00000312735	ENSP00000318604
ENSP00000312735	ENSP00000318641
ENSP00000312735	ENSP00000319104
ENSP00000312735	ENSP00000320566
ENSP00000312735	ENSP00000320949
ENSP00000312735	ENSP00000323720
ENSP00000312735	ENSP00000324122
ENSP00000312735	ENSP00000324124
ENSP00000312735	ENSP00000325002
ENSP00000312735	ENSP00000326767
ENSP00000312735	ENSP00000327889
ENSP00000312735	ENSP00000329029
ENSP00000312735	ENSP00000331201
ENSP00000312735	ENSP00000337088
ENSP00000312735	ENSP00000337340
ENSP00000312735	ENSP00000337396
ENSP00000312735	ENSP00000337797
ENSP00000312735	ENSP00000340019
ENSP00000312735	ENSP00000340823
ENSP00000312735	ENSP00000341243
ENSP00000312735	ENSP00000341885
ENSP00000312735	ENSP00000342121
ENSP00000312735	ENSP00000342889
ENSP00000312735	ENSP00000344460
ENSP00000312735	ENSP00000345530
ENSP00000312735	ENSP00000346015
ENSP00000312735	ENSP00000346142
ENSP00000312735	ENSP00000347345
ENSP00000312735	ENSP00000348168
ENSP00000312735	ENSP00000349049
ENSP00000312735	ENSP00000350967
ENSP00000312735	ENSP00000352738
ENSP00000312735	ENSP00000353575
ENSP00000312735	ENSP00000353741
ENSP00000312735	ENSP00000354347
ENSP00000312735	ENSP00000354739
ENSP00000312735	ENSP00000354775
ENSP00000312735	ENSP00000355890
ENSP00000312735	ENSP00000356146
ENSP00000312735	ENSP00000356405
ENSP00000312735	ENSP00000356785
ENSP00000312735	ENSP00000357494

ENSP00000312735	ENSP00000358064
ENSP00000312735	ENSP00000358400
ENSP00000312735	ENSP00000358563
ENSP00000312735	ENSP00000359290
ENSP00000312735	ENSP00000359531
ENSP00000312735	ENSP00000361287
ENSP00000312735	ENSP00000361465
ENSP00000312735	ENSP00000361699
ENSP00000312735	ENSP00000361949
ENSP00000312735	ENSP00000361982
ENSP00000312735	ENSP00000362413
ENSP00000312735	ENSP00000362456
ENSP00000312735	ENSP00000362532
ENSP00000312735	ENSP00000362900
ENSP00000312735	ENSP00000363559
ENSP00000312735	ENSP00000363779
ENSP00000312735	ENSP00000363944
ENSP00000312735	ENSP00000365233
ENSP00000312735	ENSP00000365943
ENSP00000312735	ENSP00000366828
ENSP00000312735	ENSP00000368612
ENSP00000312735	ENSP00000368646
ENSP00000312735	ENSP00000368984
ENSP00000312735	ENSP00000369335
ENSP00000312735	ENSP00000369695
ENSP00000312735	ENSP00000369965
ENSP00000312735	ENSP00000370589
ENSP00000312735	ENSP00000371800
ENSP00000312735	ENSP00000374280
ENSP00000312735	ENSP00000376946
ENSP00000312735	ENSP00000377969
ENSP00000312735	ENSP00000379933
ENSP00000312735	ENSP00000380460
ENSP00000312735	ENSP00000380793
ENSP00000312735	ENSP00000381064
ENSP00000312735	ENSP00000385722
ENSP00000312735	ENSP00000386161
ENSP00000312735	ENSP00000386759
ENSP00000312735	ENSP00000388872
ENSP00000312735	ENSP00000401018
ENSP00000312735	ENSP00000403852
ENSP00000312753	ENSP00000313172
ENSP00000312753	ENSP00000314792
ENSP00000312753	ENSP00000317271
ENSP00000312753	ENSP00000331487
ENSP00000312753	ENSP00000340494

ENSP00000312753	ENSP00000349892
ENSP00000312753	ENSP00000359215
ENSP00000312753	ENSP00000397644
ENSP00000312770	ENSP00000368438
ENSP00000312778	ENSP00000315791
ENSP00000312778	ENSP00000323302
ENSP00000312778	ENSP00000323720
ENSP00000312778	ENSP00000326128
ENSP00000312778	ENSP00000334188
ENSP00000312778	ENSP00000336842
ENSP00000312778	ENSP00000356405
ENSP00000312778	ENSP00000356989
ENSP00000312778	ENSP00000357887
ENSP00000312778	ENSP00000362948
ENSP00000312778	ENSP00000363313
ENSP00000312778	ENSP00000363734
ENSP00000312778	ENSP00000372694
ENSP00000312778	ENSP00000374213
ENSP00000312778	ENSP00000376946
ENSP00000312778	ENSP00000386759
ENSP00000312778	ENSP00000391774
ENSP00000312778	ENSP00000412319
ENSP00000312778	ENSP00000415678
ENSP00000312870	ENSP00000322180
ENSP00000312870	ENSP00000322570
ENSP00000312870	ENSP00000340879
ENSP00000312870	ENSP00000347087
ENSP00000312870	ENSP00000347978
ENSP00000312870	ENSP00000350491
ENSP00000312870	ENSP00000351697
ENSP00000312870	ENSP00000352522
ENSP00000312870	ENSP00000353098
ENSP00000312870	ENSP00000353793
ENSP00000312870	ENSP00000359691
ENSP00000312870	ENSP00000360412
ENSP00000312870	ENSP00000364037
ENSP00000312870	ENSP00000368349
ENSP00000312870	ENSP00000369335
ENSP00000312870	ENSP00000370151
ENSP00000312870	ENSP00000371475
ENSP00000312870	ENSP00000372023
ENSP00000312870	ENSP00000377486
ENSP00000312946	ENSP00000356972
ENSP00000312946	ENSP00000360532
ENSP00000312981	ENSP00000315379
ENSP00000312981	ENSP00000324122

ENSP00000312981	ENSP00000328690
ENSP00000312981	ENSP00000332444
ENSP00000312981	ENSP00000337194
ENSP00000312981	ENSP00000359958
ENSP00000312981	ENSP00000360412
ENSP00000312981	ENSP00000360532
ENSP00000312981	ENSP00000363313
ENSP00000312981	ENSP00000364289
ENSP00000312981	ENSP00000364293
ENSP00000312981	ENSP00000370376
ENSP00000312981	ENSP00000372023
ENSP00000312995	ENSP00000343535
ENSP00000312995	ENSP00000343741
ENSP00000312995	ENSP00000350283
ENSP00000312995	ENSP00000359206
ENSP00000312995	ENSP00000368438
ENSP00000312995	ENSP00000370151
ENSP00000312995	ENSP00000378529
ENSP00000313050	ENSP00000313420
ENSP00000313050	ENSP00000313490
ENSP00000313050	ENSP00000324560
ENSP00000313050	ENSP00000339527
ENSP00000313050	ENSP00000340466
ENSP00000313050	ENSP00000343362
ENSP00000313050	ENSP00000347495
ENSP00000313050	ENSP00000349785
ENSP00000313050	ENSP00000350708
ENSP00000313050	ENSP00000353826
ENSP00000313050	ENSP00000355759
ENSP00000313050	ENSP00000362413
ENSP00000313050	ENSP00000370023
ENSP00000313050	ENSP00000370517
ENSP00000313050	ENSP00000374455
ENSP00000313050	ENSP00000377954
ENSP00000313059	ENSP00000324422
ENSP00000313059	ENSP00000327145
ENSP00000313059	ENSP00000342075
ENSP00000313059	ENSP00000350512
ENSP00000313059	ENSP00000354927
ENSP00000313059	ENSP00000355265
ENSP00000313059	ENSP00000358081
ENSP00000313059	ENSP00000358140
ENSP00000313059	ENSP00000370517
ENSP00000313059	ENSP00000371308
ENSP00000313059	ENSP00000379144
ENSP00000313059	ENSP00000388731

ENSP00000313059	ENSP00000397435
ENSP00000313059	ENSP00000399863
ENSP00000313084	ENSP00000363571
ENSP00000313088	ENSP00000345731
ENSP00000313158	ENSP00000327213
ENSP00000313159	ENSP00000341551
ENSP00000313169	ENSP00000313829
ENSP00000313169	ENSP00000327145
ENSP00000313169	ENSP00000338766
ENSP00000313169	ENSP00000350297
ENSP00000313169	ENSP00000367398
ENSP00000313169	ENSP00000369257
ENSP00000313171	ENSP00000313851
ENSP00000313171	ENSP00000318977
ENSP00000313171	ENSP00000321636
ENSP00000313171	ENSP00000322180
ENSP00000313171	ENSP00000322242
ENSP00000313171	ENSP00000323439
ENSP00000313171	ENSP00000325677
ENSP00000313171	ENSP00000336606
ENSP00000313171	ENSP00000340879
ENSP00000313171	ENSP00000342434
ENSP00000313171	ENSP00000343392
ENSP00000313171	ENSP00000343526
ENSP00000313171	ENSP00000343741
ENSP00000313171	ENSP00000343745
ENSP00000313171	ENSP00000347719
ENSP00000313171	ENSP00000350011
ENSP00000313171	ENSP00000350491
ENSP00000313171	ENSP00000351284
ENSP00000313171	ENSP00000352257
ENSP00000313171	ENSP00000352738
ENSP00000313171	ENSP00000353793
ENSP00000313171	ENSP00000354522
ENSP00000313171	ENSP00000354720
ENSP00000313171	ENSP00000355759
ENSP00000313171	ENSP00000357602
ENSP00000313171	ENSP00000360621
ENSP00000313171	ENSP00000363021
ENSP00000313171	ENSP00000368438
ENSP00000313171	ENSP00000368591
ENSP00000313171	ENSP00000368976
ENSP00000313171	ENSP00000369335
ENSP00000313171	ENSP00000369411
ENSP00000313171	ENSP00000370150
ENSP00000313171	ENSP00000371419

ENSP00000313171	ENSP00000371475
ENSP00000313171	ENSP00000372023
ENSP00000313171	ENSP00000374213
ENSP00000313171	ENSP00000375809
ENSP00000313171	ENSP00000377384
ENSP00000313171	ENSP00000377793
ENSP00000313171	ENSP00000380504
ENSP00000313171	ENSP00000381657
ENSP00000313171	ENSP00000382562
ENSP00000313171	ENSP00000396704
ENSP00000313172	ENSP00000339299
ENSP00000313243	ENSP00000351284
ENSP00000313243	ENSP00000358812
ENSP00000313243	ENSP00000380460
ENSP00000313309	ENSP00000364749
ENSP00000313350	ENSP00000317636
ENSP00000313350	ENSP00000321636
ENSP00000313350	ENSP00000322570
ENSP00000313350	ENSP00000335557
ENSP00000313350	ENSP00000337839
ENSP00000313350	ENSP00000340305
ENSP00000313350	ENSP00000347232
ENSP00000313350	ENSP00000349142
ENSP00000313350	ENSP00000350491
ENSP00000313350	ENSP00000351284
ENSP00000313350	ENSP00000351697
ENSP00000313350	ENSP00000353332
ENSP00000313350	ENSP00000353622
ENSP00000313350	ENSP00000354522
ENSP00000313350	ENSP00000356405
ENSP00000313350	ENSP00000357907
ENSP00000313350	ENSP00000361076
ENSP00000313350	ENSP00000361310
ENSP00000313350	ENSP00000362900
ENSP00000313350	ENSP00000364126
ENSP00000313350	ENSP00000364815
ENSP00000313350	ENSP00000366135
ENSP00000313350	ENSP00000368349
ENSP00000313350	ENSP00000374280
ENSP00000313350	ENSP00000375809
ENSP00000313350	ENSP00000380504
ENSP00000313350	ENSP00000401121
ENSP00000313350	ENSP00000403359
ENSP00000313350	ENSP00000415316
ENSP00000313391	ENSP00000318921
ENSP00000313391	ENSP00000337163

ENSP00000313391	ENSP00000341737
ENSP00000313391	ENSP00000348554
ENSP00000313391	ENSP00000355809
ENSP00000313391	ENSP00000356595
ENSP00000313391	ENSP00000357494
ENSP00000313391	ENSP00000358994
ENSP00000313391	ENSP00000360798
ENSP00000313391	ENSP00000363163
ENSP00000313391	ENSP00000365891
ENSP00000313391	ENSP00000366936
ENSP00000313391	ENSP00000370719
ENSP00000313391	ENSP00000380921
ENSP00000313419	ENSP00000360916
ENSP00000313419	ENSP00000364898
ENSP00000313419	ENSP00000376544
ENSP00000313419	ENSP00000384675
ENSP00000313420	ENSP00000317636
ENSP00000313420	ENSP00000323050
ENSP00000313420	ENSP00000323099
ENSP00000313420	ENSP00000325863
ENSP00000313420	ENSP00000329357
ENSP00000313420	ENSP00000329466
ENSP00000313420	ENSP00000331310
ENSP00000313420	ENSP00000333873
ENSP00000313420	ENSP00000336868
ENSP00000313420	ENSP00000337127
ENSP00000313420	ENSP00000337797
ENSP00000313420	ENSP00000341268
ENSP00000313420	ENSP00000342011
ENSP00000313420	ENSP00000344537
ENSP00000313420	ENSP00000346566
ENSP00000313420	ENSP00000348107
ENSP00000313420	ENSP00000348784
ENSP00000313420	ENSP00000349205
ENSP00000313420	ENSP00000349393
ENSP00000313420	ENSP00000350199
ENSP00000313420	ENSP00000350283
ENSP00000313420	ENSP00000350491
ENSP00000313420	ENSP00000351284
ENSP00000313420	ENSP00000351894
ENSP00000313420	ENSP00000351947
ENSP00000313420	ENSP00000352121
ENSP00000313420	ENSP00000352257
ENSP00000313420	ENSP00000353073
ENSP00000313420	ENSP00000353793
ENSP00000313420	ENSP00000353971

ENSP00000313420	ENSP00000354522
ENSP00000313420	ENSP00000355011
ENSP00000313420	ENSP00000355090
ENSP00000313420	ENSP00000355759
ENSP00000313420	ENSP00000356505
ENSP00000313420	ENSP00000356520
ENSP00000313420	ENSP00000357283
ENSP00000313420	ENSP00000360310
ENSP00000313420	ENSP00000360532
ENSP00000313420	ENSP00000361014
ENSP00000313420	ENSP00000362314
ENSP00000313420	ENSP00000362546
ENSP00000313420	ENSP00000363021
ENSP00000313420	ENSP00000363745
ENSP00000313420	ENSP00000364119
ENSP00000313420	ENSP00000364204
ENSP00000313420	ENSP00000364270
ENSP00000313420	ENSP00000367527
ENSP00000313420	ENSP00000368438
ENSP00000313420	ENSP00000369335
ENSP00000313420	ENSP00000370151
ENSP00000313420	ENSP00000370421
ENSP00000313420	ENSP00000372023
ENSP00000313420	ENSP00000373060
ENSP00000313420	ENSP00000379093
ENSP00000313420	ENSP00000404121
ENSP00000313422	ENSP00000323339
ENSP00000313422	ENSP00000342755
ENSP00000313422	ENSP00000362546
ENSP00000313432	ENSP00000321259
ENSP00000313432	ENSP00000337353
ENSP00000313432	ENSP00000360286
ENSP00000313432	ENSP00000361699
ENSP00000313432	ENSP00000379678
ENSP00000313490	ENSP00000314129
ENSP00000313490	ENSP00000324560
ENSP00000313490	ENSP00000329466
ENSP00000313490	ENSP00000333019
ENSP00000313490	ENSP00000337839
ENSP00000313490	ENSP00000341692
ENSP00000313490	ENSP00000343535
ENSP00000313490	ENSP00000366434
ENSP00000313490	ENSP00000378782
ENSP00000313490	ENSP00000382595
ENSP00000313490	ENSP00000400312
ENSP00000313490	ENSP00000415434

ENSP00000313601	ENSP00000349658
ENSP00000313601	ENSP00000382177
ENSP00000313601	ENSP00000399968
ENSP00000313603	ENSP00000348877
ENSP00000313603	ENSP00000352522
ENSP00000313603	ENSP00000353741
ENSP00000313603	ENSP00000354125
ENSP00000313603	ENSP00000354347
ENSP00000313603	ENSP00000358140
ENSP00000313603	ENSP00000359345
ENSP00000313603	ENSP00000361287
ENSP00000313603	ENSP00000363559
ENSP00000313603	ENSP00000365025
ENSP00000313603	ENSP00000365837
ENSP00000313603	ENSP00000375809
ENSP00000313603	ENSP00000377944
ENSP00000313603	ENSP00000391944
ENSP00000313674	ENSP00000318861
ENSP00000313674	ENSP00000319341
ENSP00000313674	ENSP00000323302
ENSP00000313674	ENSP00000324122
ENSP00000313674	ENSP00000325448
ENSP00000313674	ENSP00000326806
ENSP00000313674	ENSP00000331087
ENSP00000313674	ENSP00000332103
ENSP00000313674	ENSP00000339881
ENSP00000313674	ENSP00000342374
ENSP00000313674	ENSP00000348010
ENSP00000313674	ENSP00000362110
ENSP00000313674	ENSP00000363313
ENSP00000313674	ENSP00000376973
ENSP00000313674	ENSP00000387006
ENSP00000313674	ENSP00000401566
ENSP00000313674	ENSP00000410758
ENSP00000313674	ENSP00000412566
ENSP00000313691	ENSP00000345702
ENSP00000313691	ENSP00000356811
ENSP00000313691	ENSP00000363999
ENSP00000313752	ENSP00000320885
ENSP00000313759	ENSP00000350249
ENSP00000313759	ENSP00000363021
ENSP00000313822	ENSP00000361048
ENSP00000313829	ENSP00000315659
ENSP00000313829	ENSP00000316032
ENSP00000313829	ENSP00000316845
ENSP00000313829	ENSP00000316879

ENSP00000313829	ENSP00000322608
ENSP00000313829	ENSP00000326261
ENSP00000313829	ENSP00000339186
ENSP00000313829	ENSP00000339245
ENSP00000313829	ENSP00000340836
ENSP00000313829	ENSP00000345731
ENSP00000313829	ENSP00000345824
ENSP00000313829	ENSP00000348215
ENSP00000313829	ENSP00000348602
ENSP00000313829	ENSP00000349594
ENSP00000313829	ENSP00000350249
ENSP00000313829	ENSP00000351947
ENSP00000313829	ENSP00000353735
ENSP00000313829	ENSP00000355177
ENSP00000313829	ENSP00000356437
ENSP00000313829	ENSP00000356520
ENSP00000313829	ENSP00000356774
ENSP00000313829	ENSP00000358413
ENSP00000313829	ENSP00000358770
ENSP00000313829	ENSP00000360532
ENSP00000313829	ENSP00000360916
ENSP00000313829	ENSP00000362993
ENSP00000313829	ENSP00000363349
ENSP00000313829	ENSP00000365439
ENSP00000313829	ENSP00000366135
ENSP00000313829	ENSP00000366843
ENSP00000313829	ENSP00000368801
ENSP00000313829	ENSP00000370719
ENSP00000313829	ENSP00000378350
ENSP00000313829	ENSP00000379607
ENSP00000313829	ENSP00000380921
ENSP00000313829	ENSP00000384490
ENSP00000313829	ENSP00000387170
ENSP00000313829	ENSP00000388731
ENSP00000313851	ENSP00000327197
ENSP00000313851	ENSP00000329466
ENSP00000313851	ENSP00000346725
ENSP00000313851	ENSP00000354720
ENSP00000313851	ENSP00000358497
ENSP00000313851	ENSP00000360310
ENSP00000313851	ENSP00000361310
ENSP00000313851	ENSP00000361875
ENSP00000313851	ENSP00000362334
ENSP00000313851	ENSP00000369411
ENSP00000313851	ENSP00000372689
ENSP00000313851	ENSP00000377655

ENSP00000313851	ENSP00000394791
ENSP00000313851	ENSP00000396704
ENSP00000313851	ENSP00000402527
ENSP00000313875	ENSP00000348278
ENSP00000313881	ENSP00000315950
ENSP00000313881	ENSP00000340305
ENSP00000313881	ENSP00000347836
ENSP00000313881	ENSP00000360116
ENSP00000313881	ENSP00000391685
ENSP00000313881	ENSP00000417281
ENSP00000313921	ENSP00000318604
ENSP00000313921	ENSP00000330836
ENSP00000313921	ENSP00000337443
ENSP00000313921	ENSP00000347324
ENSP00000313921	ENSP00000359976
ENSP00000313921	ENSP00000361287
ENSP00000313921	ENSP00000373477
ENSP00000313933	ENSP00000341680
ENSP00000313933	ENSP00000360532
ENSP00000313933	ENSP00000396620
ENSP00000313953	ENSP00000343535
ENSP00000313953	ENSP00000346453
ENSP00000313953	ENSP00000354347
ENSP00000313953	ENSP00000362110
ENSP00000313953	ENSP00000397435
ENSP00000314004	ENSP00000318775
ENSP00000314004	ENSP00000330408
ENSP00000314004	ENSP00000337340
ENSP00000314004	ENSP00000339109
ENSP00000314004	ENSP00000344936
ENSP00000314004	ENSP00000345917
ENSP00000314004	ENSP00000346240
ENSP00000314004	ENSP00000348554
ENSP00000314004	ENSP00000351947
ENSP00000314004	ENSP00000355560
ENSP00000314004	ENSP00000356972
ENSP00000314004	ENSP00000360532
ENSP00000314004	ENSP00000363322
ENSP00000314004	ENSP00000367208
ENSP00000314004	ENSP00000378350
ENSP00000314004	ENSP00000378529
ENSP00000314004	ENSP00000386156
ENSP00000314004	ENSP00000394394
ENSP00000314036	ENSP00000354876
ENSP00000314036	ENSP00000355890
ENSP00000314036	ENSP00000355968

ENSP00000314036	ENSP00000357998
ENSP00000314036	ENSP00000359067
ENSP00000314036	ENSP00000364188
ENSP00000314073	ENSP00000387262
ENSP00000314080	ENSP00000316578
ENSP00000314080	ENSP00000320485
ENSP00000314080	ENSP00000333633
ENSP00000314080	ENSP00000344456
ENSP00000314080	ENSP00000358404
ENSP00000314080	ENSP00000362592
ENSP00000314080	ENSP00000369716
ENSP00000314129	ENSP00000323967
ENSP00000314129	ENSP00000336724
ENSP00000314129	ENSP00000340466
ENSP00000314129	ENSP00000341551
ENSP00000314129	ENSP00000342056
ENSP00000314129	ENSP00000355890
ENSP00000314129	ENSP00000358140
ENSP00000314129	ENSP00000362688
ENSP00000314129	ENSP00000364591
ENSP00000314129	ENSP00000374280
ENSP00000314129	ENSP00000385746
ENSP00000314129	ENSP00000397441
ENSP00000314129	ENSP00000401121
ENSP00000314129	ENSP00000403359
ENSP00000314132	ENSP00000353059
ENSP00000314132	ENSP00000358022
ENSP00000314193	ENSP00000315674
ENSP00000314193	ENSP00000321449
ENSP00000314193	ENSP00000326981
ENSP00000314193	ENSP00000327179
ENSP00000314193	ENSP00000328854
ENSP00000314193	ENSP00000329466
ENSP00000314193	ENSP00000339095
ENSP00000314193	ENSP00000339145
ENSP00000314193	ENSP00000353246
ENSP00000314193	ENSP00000354040
ENSP00000314193	ENSP00000354125
ENSP00000314193	ENSP00000355471
ENSP00000314193	ENSP00000355541
ENSP00000314193	ENSP00000358812
ENSP00000314193	ENSP00000360031
ENSP00000314193	ENSP00000360286
ENSP00000314193	ENSP00000363313
ENSP00000314193	ENSP00000363642
ENSP00000314193	ENSP00000363746

ENSP00000314193	ENSP00000370258
ENSP00000314193	ENSP00000370589
ENSP00000314193	ENSP00000370968
ENSP00000314193	ENSP00000371101
ENSP00000314193	ENSP00000372695
ENSP00000314193	ENSP00000379888
ENSP00000314193	ENSP00000384302
ENSP00000314193	ENSP00000387006
ENSP00000314193	ENSP00000399454
ENSP00000314193	ENSP00000402869
ENSP00000314193	ENSP00000405614
ENSP00000314193	ENSP00000414321
ENSP00000314196	ENSP00000337463
ENSP00000314196	ENSP00000382723
ENSP00000314196	ENSP00000389516
ENSP00000314196	ENSP00000395863
ENSP00000314196	ENSP00000401287
ENSP00000314196	ENSP00000405864
ENSP00000314196	ENSP00000412805
ENSP00000314311	ENSP00000314348
ENSP00000314311	ENSP00000314441
ENSP00000314311	ENSP00000314949
ENSP00000314311	ENSP00000316879
ENSP00000314311	ENSP00000317992
ENSP00000314311	ENSP00000318646
ENSP00000314311	ENSP00000322396
ENSP00000314311	ENSP00000322570
ENSP00000314311	ENSP00000323856
ENSP00000314311	ENSP00000324122
ENSP00000314311	ENSP00000325677
ENSP00000314311	ENSP00000326411
ENSP00000314311	ENSP00000326514
ENSP00000314311	ENSP00000326981
ENSP00000314311	ENSP00000327821
ENSP00000314311	ENSP00000328690
ENSP00000314311	ENSP00000328773
ENSP00000314311	ENSP00000329466
ENSP00000314311	ENSP00000335321
ENSP00000314311	ENSP00000337405
ENSP00000314311	ENSP00000337839
ENSP00000314311	ENSP00000338862
ENSP00000314311	ENSP00000339095
ENSP00000314311	ENSP00000340281
ENSP00000314311	ENSP00000342181
ENSP00000314311	ENSP00000343081
ENSP00000314311	ENSP00000348010

ENSP00000314311	ENSP00000348722
ENSP00000314311	ENSP00000351697
ENSP00000314311	ENSP00000351885
ENSP00000314311	ENSP00000353246
ENSP00000314311	ENSP00000354125
ENSP00000314311	ENSP00000354673
ENSP00000314311	ENSP00000355205
ENSP00000314311	ENSP00000356405
ENSP00000314311	ENSP00000356476
ENSP00000314311	ENSP00000356989
ENSP00000314311	ENSP00000357748
ENSP00000314311	ENSP00000358563
ENSP00000314311	ENSP00000359321
ENSP00000314311	ENSP00000359345
ENSP00000314311	ENSP00000361465
ENSP00000314311	ENSP00000361982
ENSP00000314311	ENSP00000362105
ENSP00000314311	ENSP00000362110
ENSP00000314311	ENSP00000362576
ENSP00000314311	ENSP00000362744
ENSP00000314311	ENSP00000363313
ENSP00000314311	ENSP00000363384
ENSP00000314311	ENSP00000363559
ENSP00000314311	ENSP00000363779
ENSP00000314311	ENSP00000363944
ENSP00000314311	ENSP00000364289
ENSP00000314311	ENSP00000364320
ENSP00000314311	ENSP00000364839
ENSP00000314311	ENSP00000365837
ENSP00000314311	ENSP00000366135
ENSP00000314311	ENSP00000366557
ENSP00000314311	ENSP00000367029
ENSP00000314311	ENSP00000368438
ENSP00000314311	ENSP00000368565
ENSP00000314311	ENSP00000368927
ENSP00000314311	ENSP00000369042
ENSP00000314311	ENSP00000370589
ENSP00000314311	ENSP00000374213
ENSP00000314311	ENSP00000374399
ENSP00000314311	ENSP00000376204
ENSP00000314311	ENSP00000376973
ENSP00000314311	ENSP00000379888
ENSP00000314311	ENSP00000380460
ENSP00000314311	ENSP00000382392
ENSP00000314311	ENSP00000387006
ENSP00000314311	ENSP00000393275

ENSP00000314311	ENSP00000401371
ENSP00000314311	ENSP00000402338
ENSP00000314311	ENSP00000403852
ENSP00000314311	ENSP00000410758
ENSP00000314311	ENSP00000417229
ENSP00000314348	ENSP00000315674
ENSP00000314348	ENSP00000315791
ENSP00000314348	ENSP00000316032
ENSP00000314348	ENSP00000317992
ENSP00000314348	ENSP00000318195
ENSP00000314348	ENSP00000320917
ENSP00000314348	ENSP00000322396
ENSP00000314348	ENSP00000323858
ENSP00000314348	ENSP00000325377
ENSP00000314348	ENSP00000328690
ENSP00000314348	ENSP00000329466
ENSP00000314348	ENSP00000331817
ENSP00000314348	ENSP00000332444
ENSP00000314348	ENSP00000336752
ENSP00000314348	ENSP00000338573
ENSP00000314348	ENSP00000338862
ENSP00000314348	ENSP00000339095
ENSP00000314348	ENSP00000339145
ENSP00000314348	ENSP00000339795
ENSP00000314348	ENSP00000342181
ENSP00000314348	ENSP00000343557
ENSP00000314348	ENSP00000343885
ENSP00000314348	ENSP00000345096
ENSP00000314348	ENSP00000346022
ENSP00000314348	ENSP00000346050
ENSP00000314348	ENSP00000346725
ENSP00000314348	ENSP00000352021
ENSP00000314348	ENSP00000352738
ENSP00000314348	ENSP00000353246
ENSP00000314348	ENSP00000354040
ENSP00000314348	ENSP00000355541
ENSP00000314348	ENSP00000355899
ENSP00000314348	ENSP00000358400
ENSP00000314348	ENSP00000358563
ENSP00000314348	ENSP00000358812
ENSP00000314348	ENSP00000359345
ENSP00000314348	ENSP00000359688
ENSP00000314348	ENSP00000360031
ENSP00000314348	ENSP00000360412
ENSP00000314348	ENSP00000360828
ENSP00000314348	ENSP00000361076

ENSP00000314348	ENSP00000361232
ENSP00000314348	ENSP00000361465
ENSP00000314348	ENSP00000361949
ENSP00000314348	ENSP00000362153
ENSP00000314348	ENSP00000362344
ENSP00000314348	ENSP00000362744
ENSP00000314348	ENSP00000363018
ENSP00000314348	ENSP00000363559
ENSP00000314348	ENSP00000363676
ENSP00000314348	ENSP00000363746
ENSP00000314348	ENSP00000363944
ENSP00000314348	ENSP00000364320
ENSP00000314348	ENSP00000364543
ENSP00000314348	ENSP00000366135
ENSP00000314348	ENSP00000368887
ENSP00000314348	ENSP00000370589
ENSP00000314348	ENSP00000371101
ENSP00000314348	ENSP00000372695
ENSP00000314348	ENSP00000372827
ENSP00000314348	ENSP00000375730
ENSP00000314348	ENSP00000377865
ENSP00000314348	ENSP00000379888
ENSP00000314348	ENSP00000380982
ENSP00000314348	ENSP00000382392
ENSP00000314348	ENSP00000384302
ENSP00000314348	ENSP00000388340
ENSP00000314348	ENSP00000389103
ENSP00000314348	ENSP00000393953
ENSP00000314348	ENSP00000394400
ENSP00000314348	ENSP00000399454
ENSP00000314348	ENSP00000399530
ENSP00000314348	ENSP00000400626
ENSP00000314348	ENSP00000401371
ENSP00000314348	ENSP00000402338
ENSP00000314348	ENSP00000402869
ENSP00000314348	ENSP00000405614
ENSP00000314348	ENSP00000412310
ENSP00000314348	ENSP00000417229
ENSP00000314380	ENSP00000348069
ENSP00000314380	ENSP00000354876
ENSP00000314380	ENSP00000364349
ENSP00000314380	ENSP00000404725
ENSP00000314380	ENSP00000404814
ENSP00000314396	ENSP00000364289
ENSP00000314420	ENSP00000349748
ENSP00000314420	ENSP00000351407

ENSP00000314420	ENSP00000353622
ENSP00000314420	ENSP00000355146
ENSP00000314420	ENSP00000359206
ENSP00000314420	ENSP00000381031
ENSP00000314425	ENSP00000321345
ENSP00000314440	ENSP00000368140
ENSP00000314441	ENSP00000318195
ENSP00000314441	ENSP00000321810
ENSP00000314441	ENSP00000327821
ENSP00000314441	ENSP00000328671
ENSP00000314441	ENSP00000331817
ENSP00000314441	ENSP00000343081
ENSP00000314441	ENSP00000348722
ENSP00000314441	ENSP00000355890
ENSP00000314441	ENSP00000364320
ENSP00000314441	ENSP00000365837
ENSP00000314441	ENSP00000366135
ENSP00000314441	ENSP00000366519
ENSP00000314441	ENSP00000371201
ENSP00000314441	ENSP00000382392
ENSP00000314441	ENSP00000384302
ENSP00000314441	ENSP00000388464
ENSP00000314441	ENSP00000419740
ENSP00000314480	ENSP00000365735
ENSP00000314480	ENSP00000372023
ENSP00000314491	ENSP00000315859
ENSP00000314491	ENSP00000321706
ENSP00000314491	ENSP00000336868
ENSP00000314491	ENSP00000339845
ENSP00000314491	ENSP00000343745
ENSP00000314491	ENSP00000360916
ENSP00000314491	ENSP00000365439
ENSP00000314491	ENSP00000369716
ENSP00000314491	ENSP00000379607
ENSP00000314499	ENSP00000314615
ENSP00000314499	ENSP00000318374
ENSP00000314499	ENSP00000320543
ENSP00000314499	ENSP00000325002
ENSP00000314499	ENSP00000344055
ENSP00000314499	ENSP00000347169
ENSP00000314499	ENSP00000348020
ENSP00000314499	ENSP00000348442
ENSP00000314499	ENSP00000349824
ENSP00000314499	ENSP00000350199
ENSP00000314499	ENSP00000351926
ENSP00000314499	ENSP00000356146

ENSP00000314499	ENSP00000360183
ENSP00000314499	ENSP00000365891
ENSP00000314499	ENSP00000366179
ENSP00000314499	ENSP00000369099
ENSP00000314499	ENSP00000369335
ENSP00000314499	ENSP00000371451
ENSP00000314499	ENSP00000373700
ENSP00000314499	ENSP00000375986
ENSP00000314499	ENSP00000377769
ENSP00000314499	ENSP00000378605
ENSP00000314499	ENSP00000388340
ENSP00000314499	ENSP00000397441
ENSP00000314499	ENSP00000402240
ENSP00000314508	ENSP00000317334
ENSP00000314508	ENSP00000333298
ENSP00000314508	ENSP00000355865
ENSP00000314508	ENSP00000378394
ENSP00000314518	ENSP00000357484
ENSP00000314544	ENSP00000360532
ENSP00000314615	ENSP00000325002
ENSP00000314615	ENSP00000325863
ENSP00000314615	ENSP00000327309
ENSP00000314615	ENSP00000328464
ENSP00000314615	ENSP00000329468
ENSP00000314615	ENSP00000336724
ENSP00000314615	ENSP00000339826
ENSP00000314615	ENSP00000341189
ENSP00000314615	ENSP00000342793
ENSP00000314615	ENSP00000346733
ENSP00000314615	ENSP00000348163
ENSP00000314615	ENSP00000350297
ENSP00000314615	ENSP00000350512
ENSP00000314615	ENSP00000354972
ENSP00000314615	ENSP00000355261
ENSP00000314615	ENSP00000356146
ENSP00000314615	ENSP00000357048
ENSP00000314615	ENSP00000359067
ENSP00000314615	ENSP00000359484
ENSP00000314615	ENSP00000359518
ENSP00000314615	ENSP00000360183
ENSP00000314615	ENSP00000363349
ENSP00000314615	ENSP00000363676
ENSP00000314615	ENSP00000368959
ENSP00000314615	ENSP00000369411
ENSP00000314615	ENSP00000369860
ENSP00000314615	ENSP00000376268

ENSP00000314615	ENSP00000376871
ENSP00000314615	ENSP00000377769
ENSP00000314615	ENSP00000402240
ENSP00000314615	ENSP00000403397
ENSP00000314615	ENSP00000408236
ENSP00000314615	ENSP00000419628
ENSP00000314733	ENSP00000320768
ENSP00000314733	ENSP00000339191
[truncated: 400,334 more chars]
